# Supplementary figures and images for: Unique Features of Satellite DNA Transcription in Different Tissues of Caenorhabditis elegans (part 2 of 2)
Source: Int J Mol Sci. 2023 Feb 3;24(3):2970. doi: 10.3390/ijms24032970 (PMC9918286; doi:10.3390/ijms24032970)

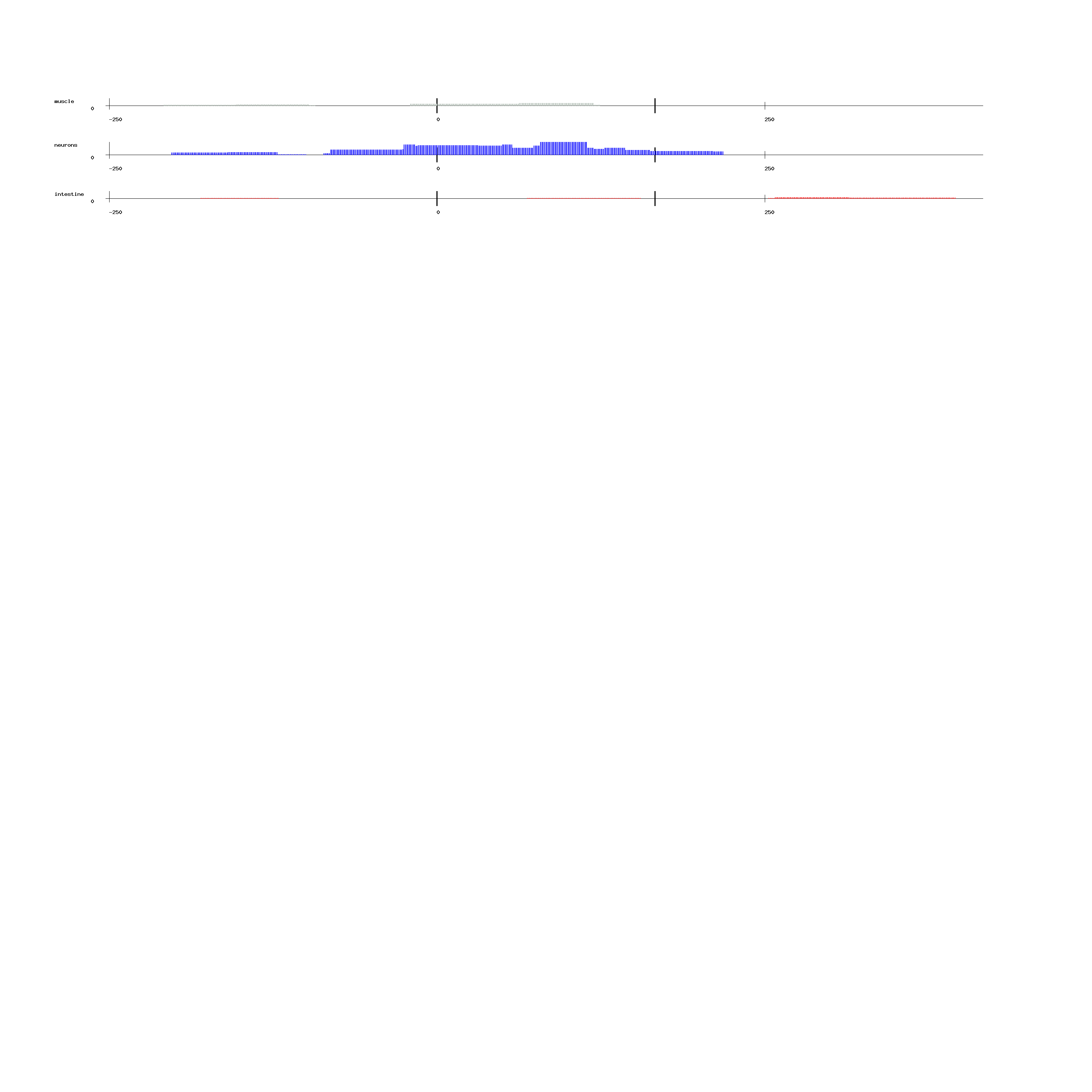

Supplement: Supplementary file 1 [file ijms-24-02970-s001.zip › Supplementary Data S2/2.13906504-13906669.png]

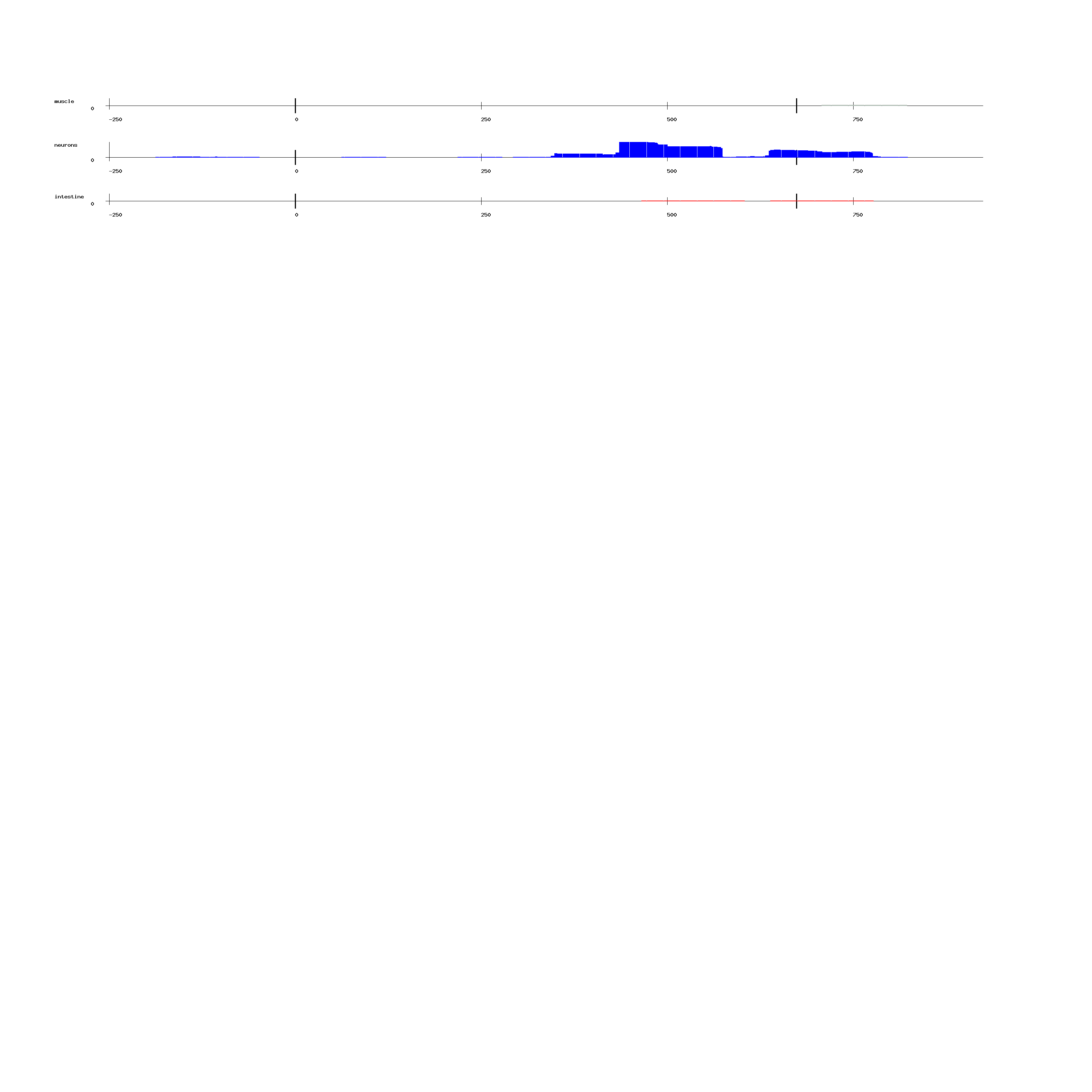

Supplement: Supplementary file 1 [file ijms-24-02970-s001.zip › Supplementary Data S2/2.14192423-14193096.png]

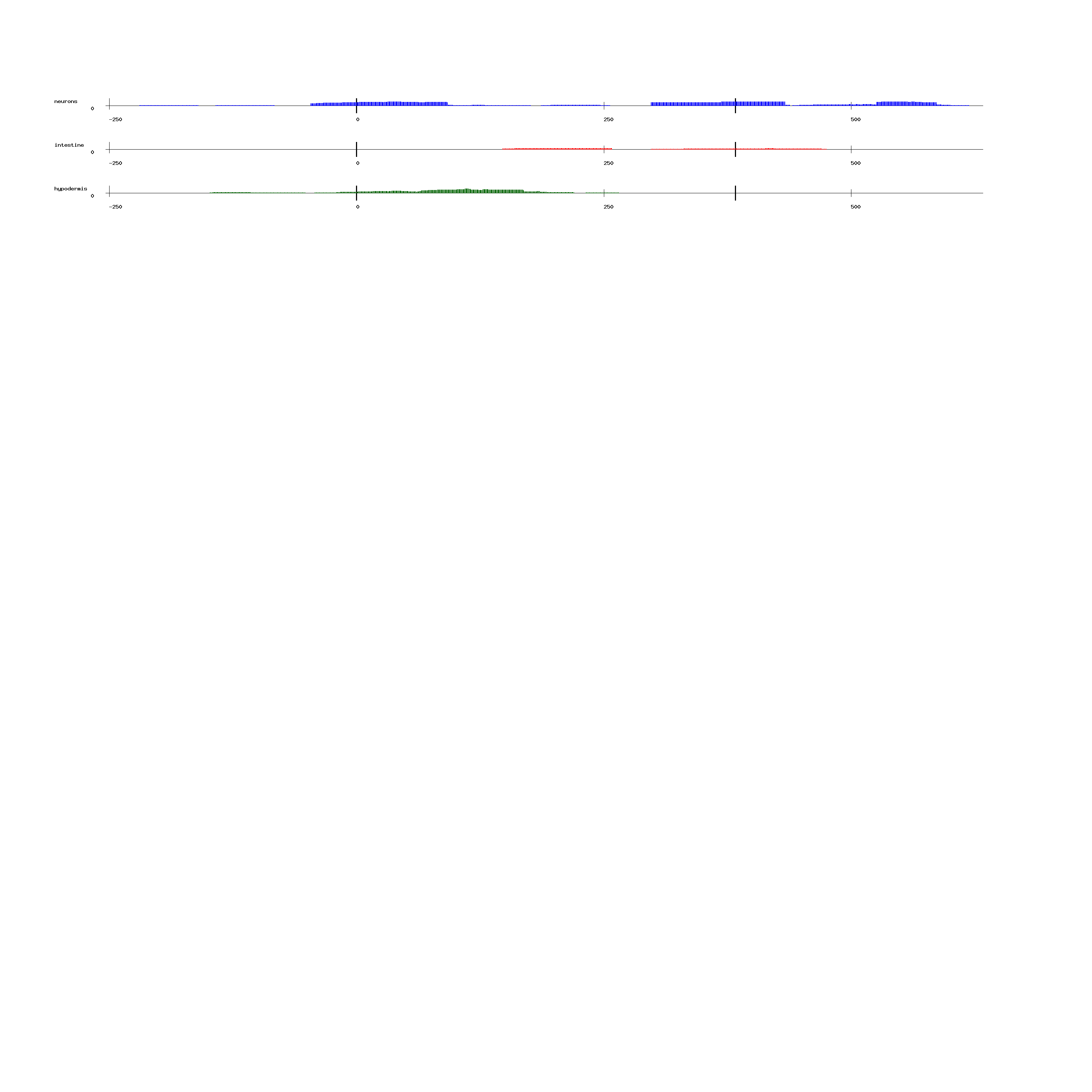

Supplement: Supplementary file 1 [file ijms-24-02970-s001.zip › Supplementary Data S2/2.14269513-14269895.png]

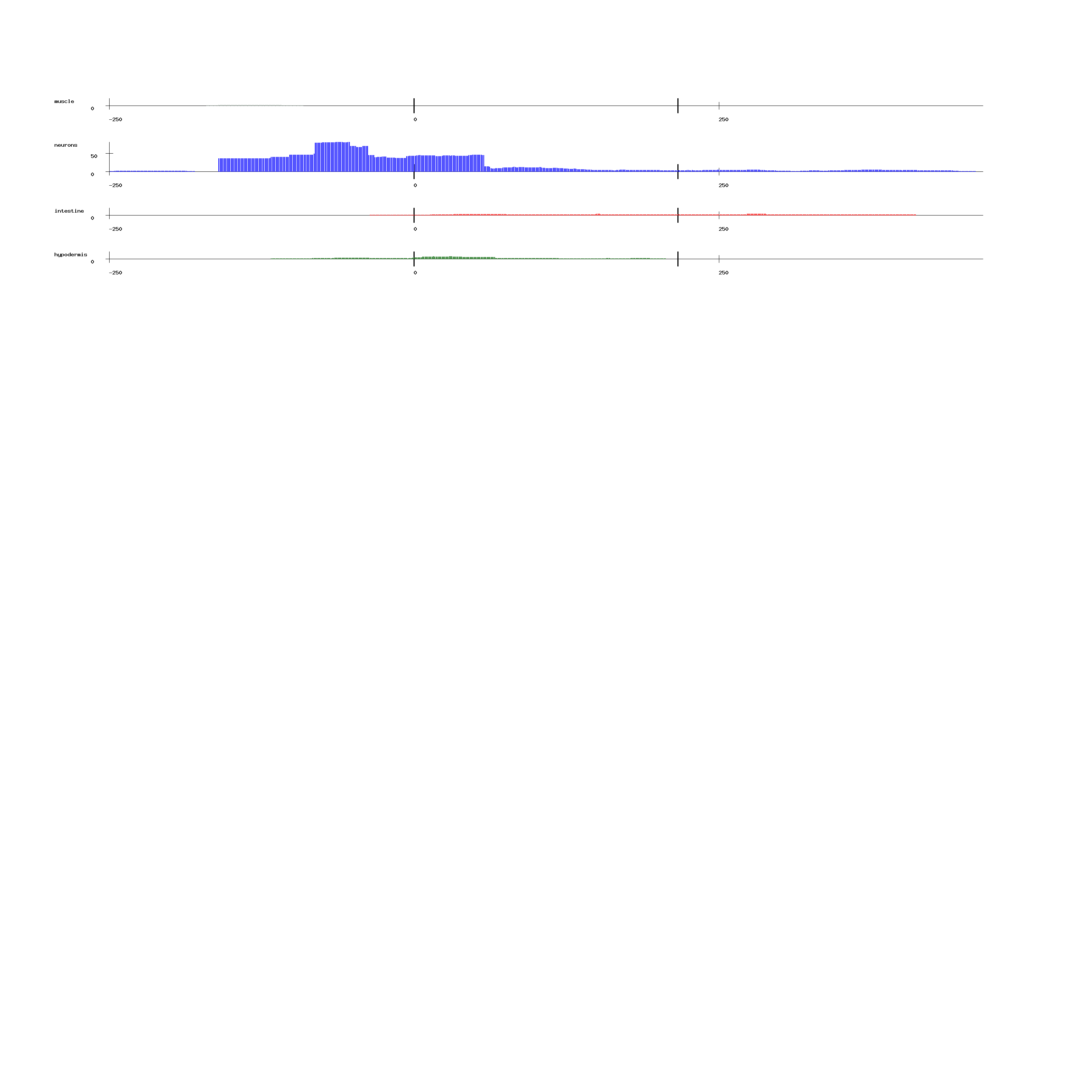

Supplement: Supplementary file 1 [file ijms-24-02970-s001.zip › Supplementary Data S2/2.14273310-14273525.png]

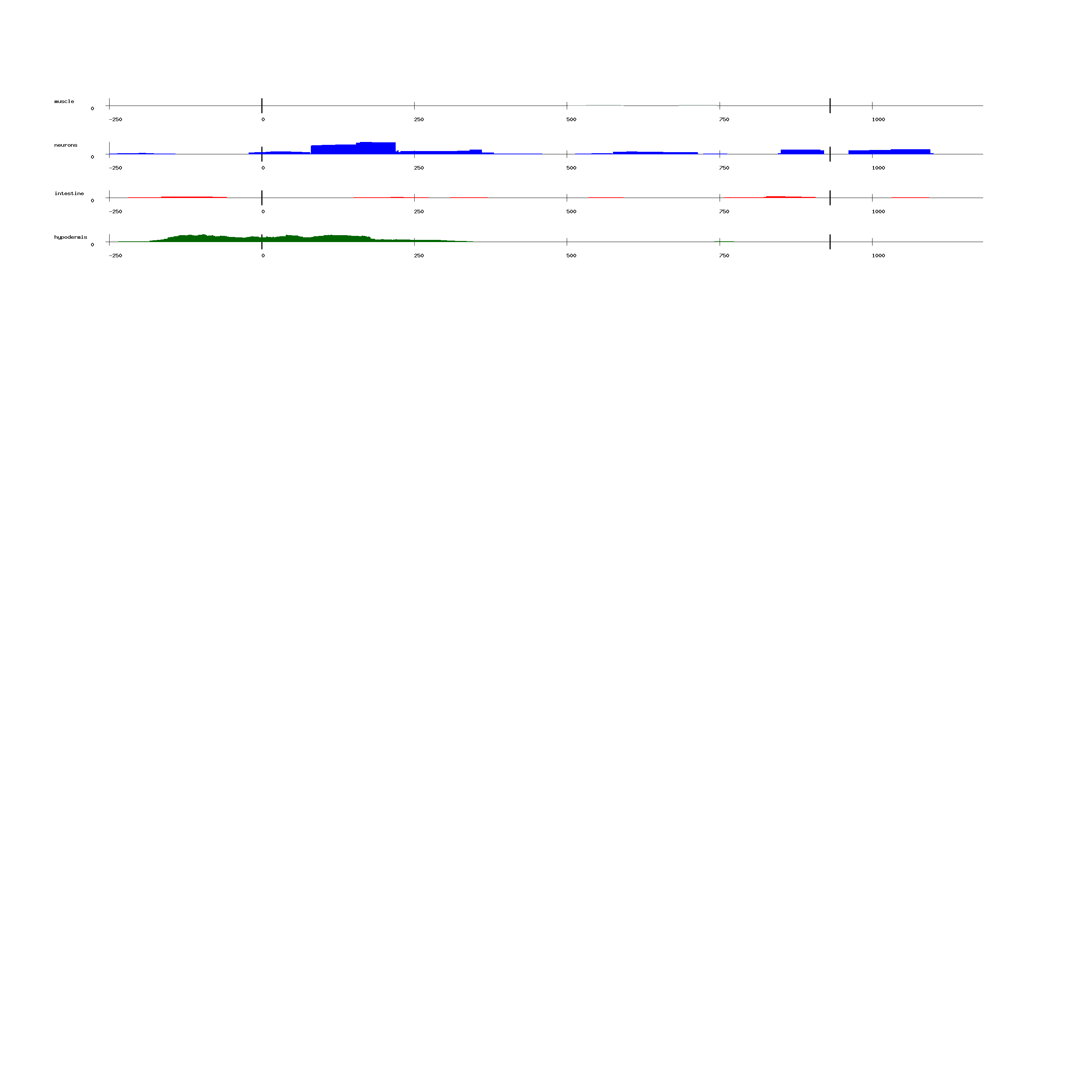

Supplement: Supplementary file 1 [file ijms-24-02970-s001.zip › Supplementary Data S2/2.14274983-14275913.png]

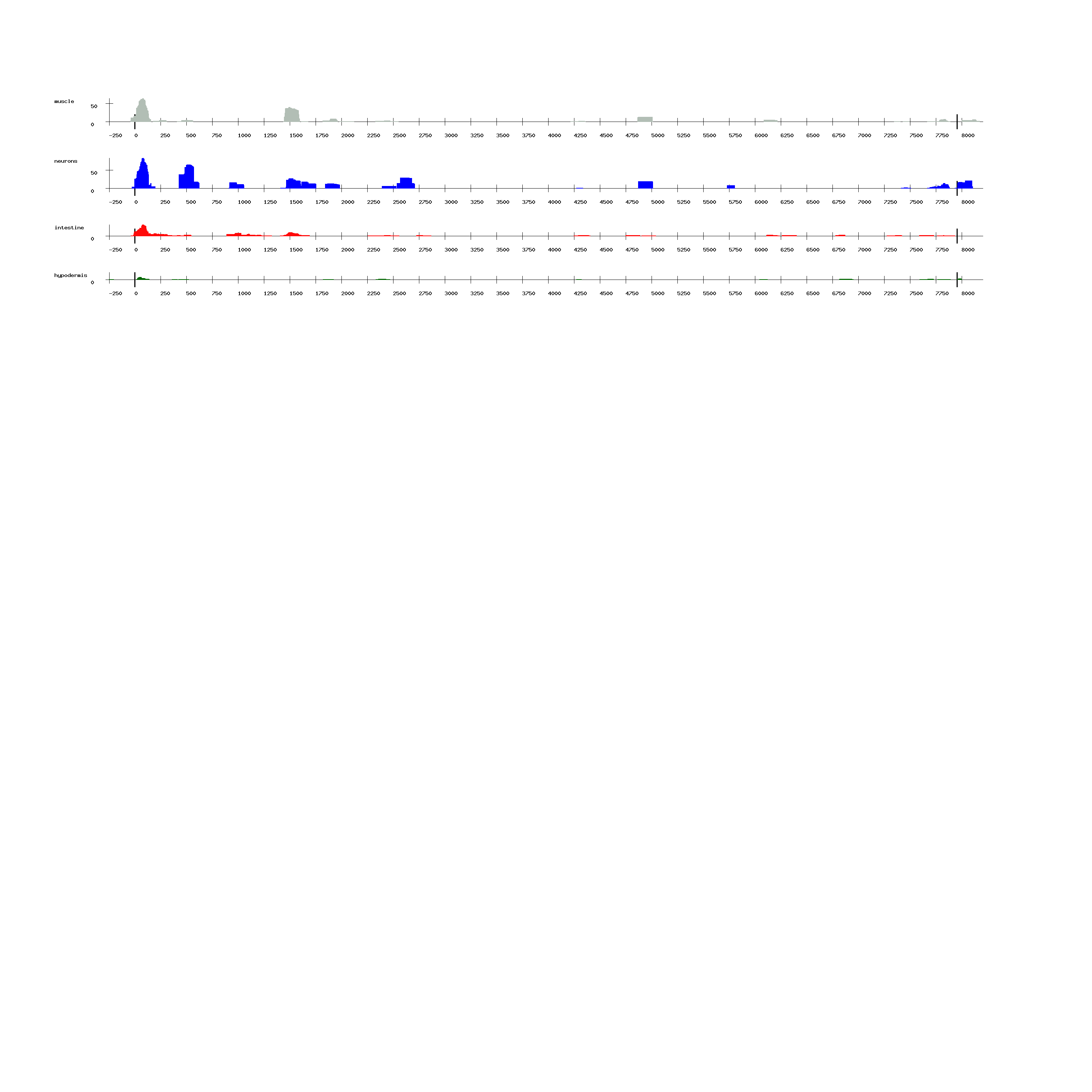

Supplement: Supplementary file 1 [file ijms-24-02970-s001.zip › Supplementary Data S2/2.14323963-14331913.png]

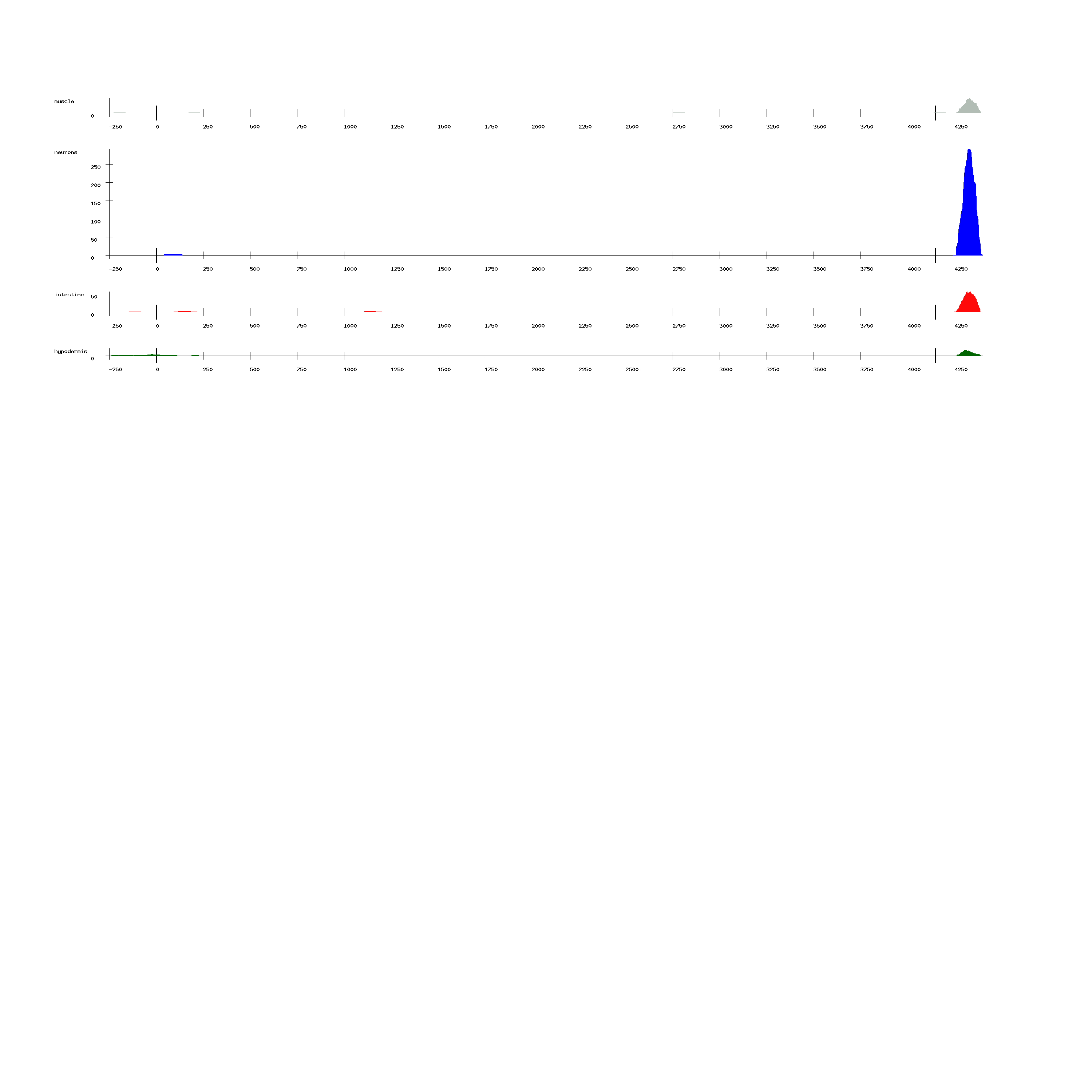

Supplement: Supplementary file 1 [file ijms-24-02970-s001.zip › Supplementary Data S2/2.14333431-14337579.png]

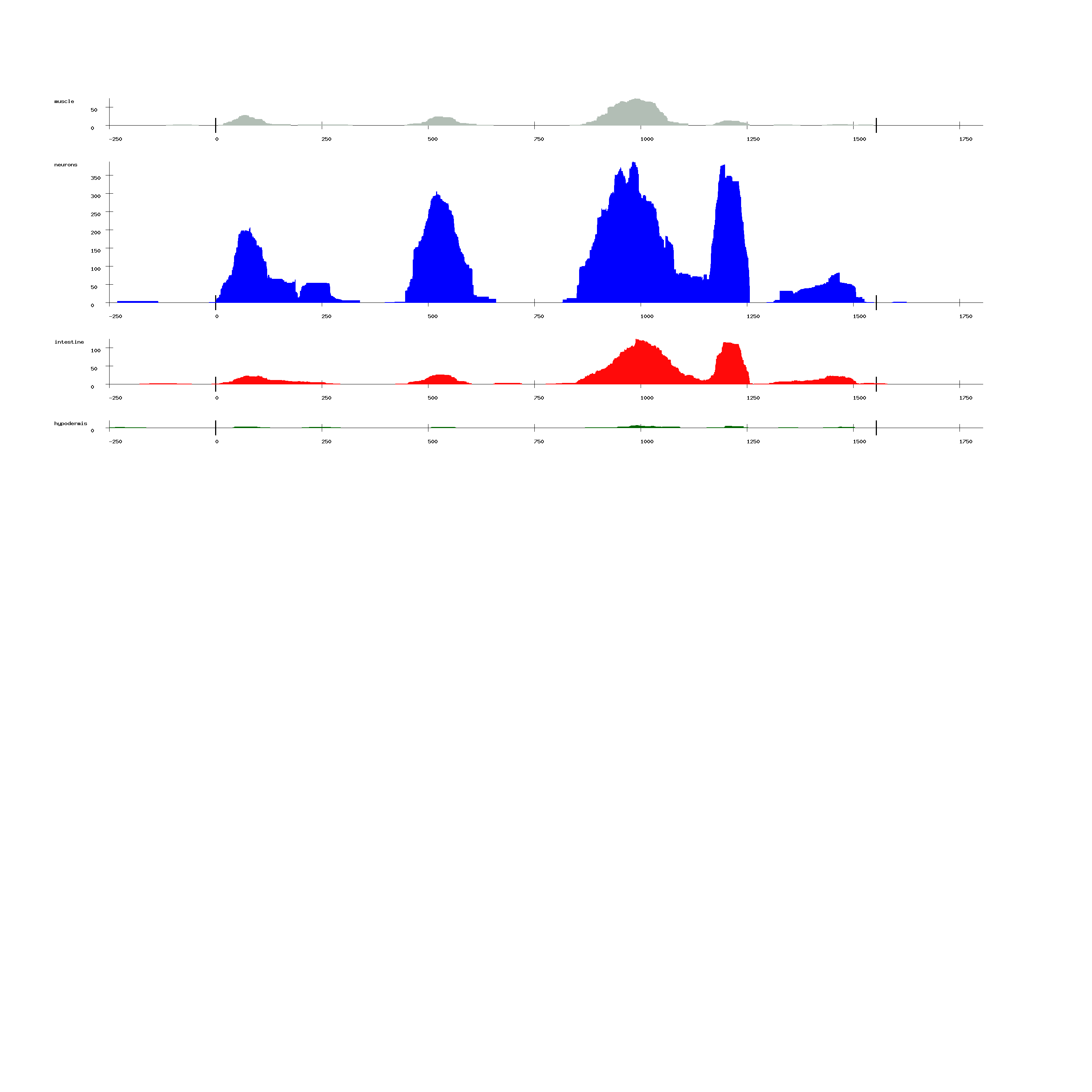

Supplement: Supplementary file 1 [file ijms-24-02970-s001.zip › Supplementary Data S2/2.14337687-14339240.png]

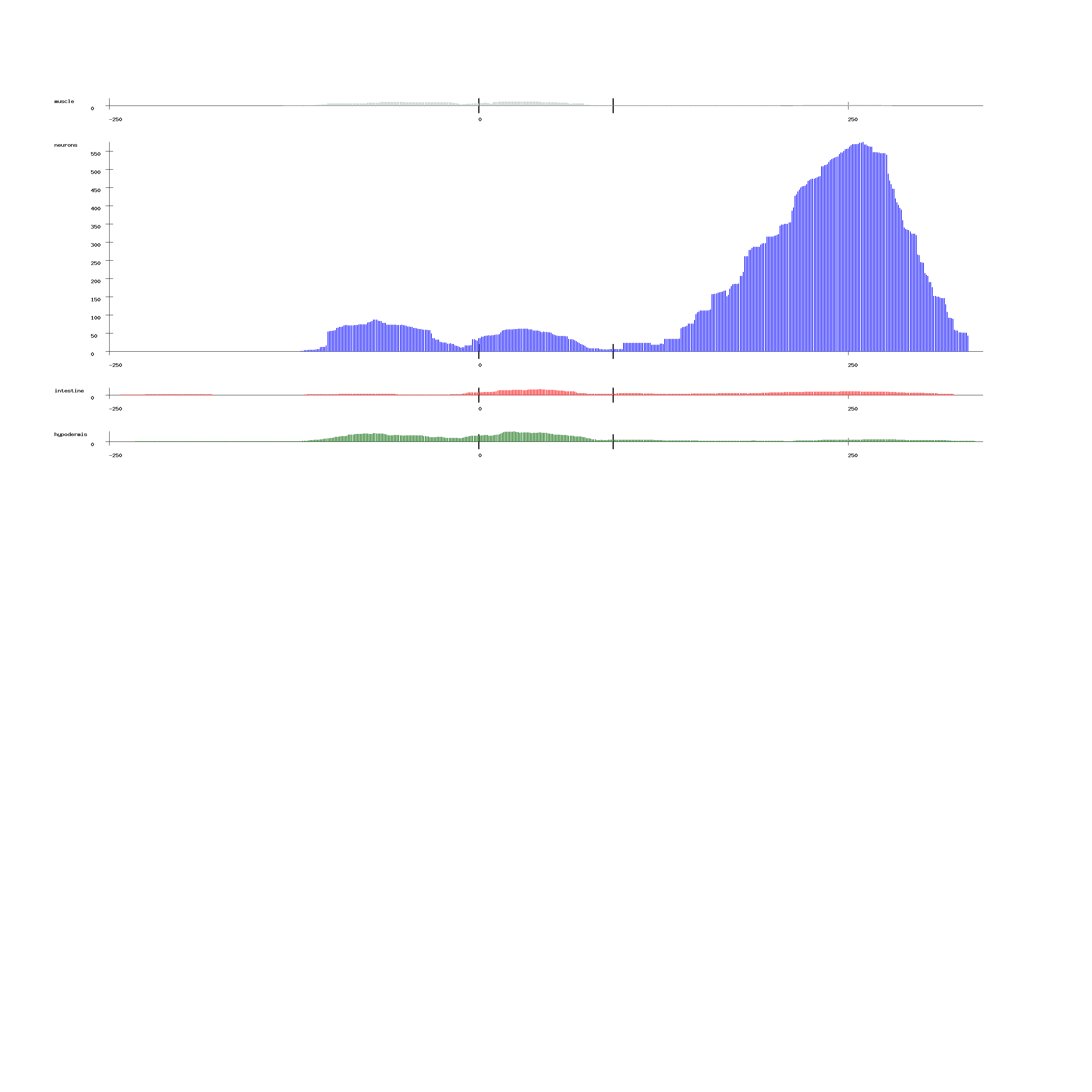

Supplement: Supplementary file 1 [file ijms-24-02970-s001.zip › Supplementary Data S2/2.14993591-14993681.png]

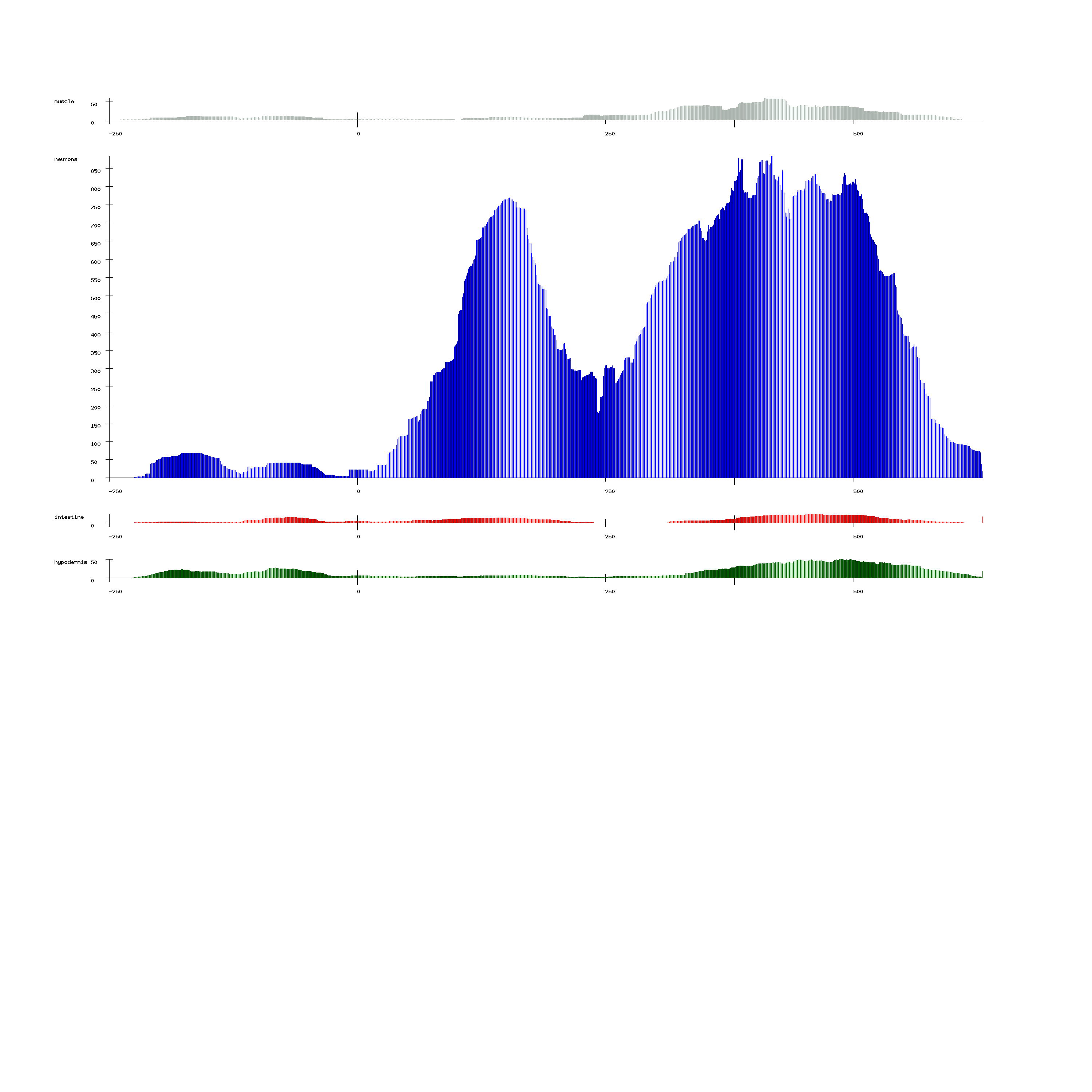

Supplement: Supplementary file 1 [file ijms-24-02970-s001.zip › Supplementary Data S2/2.14993697-14994076.png]

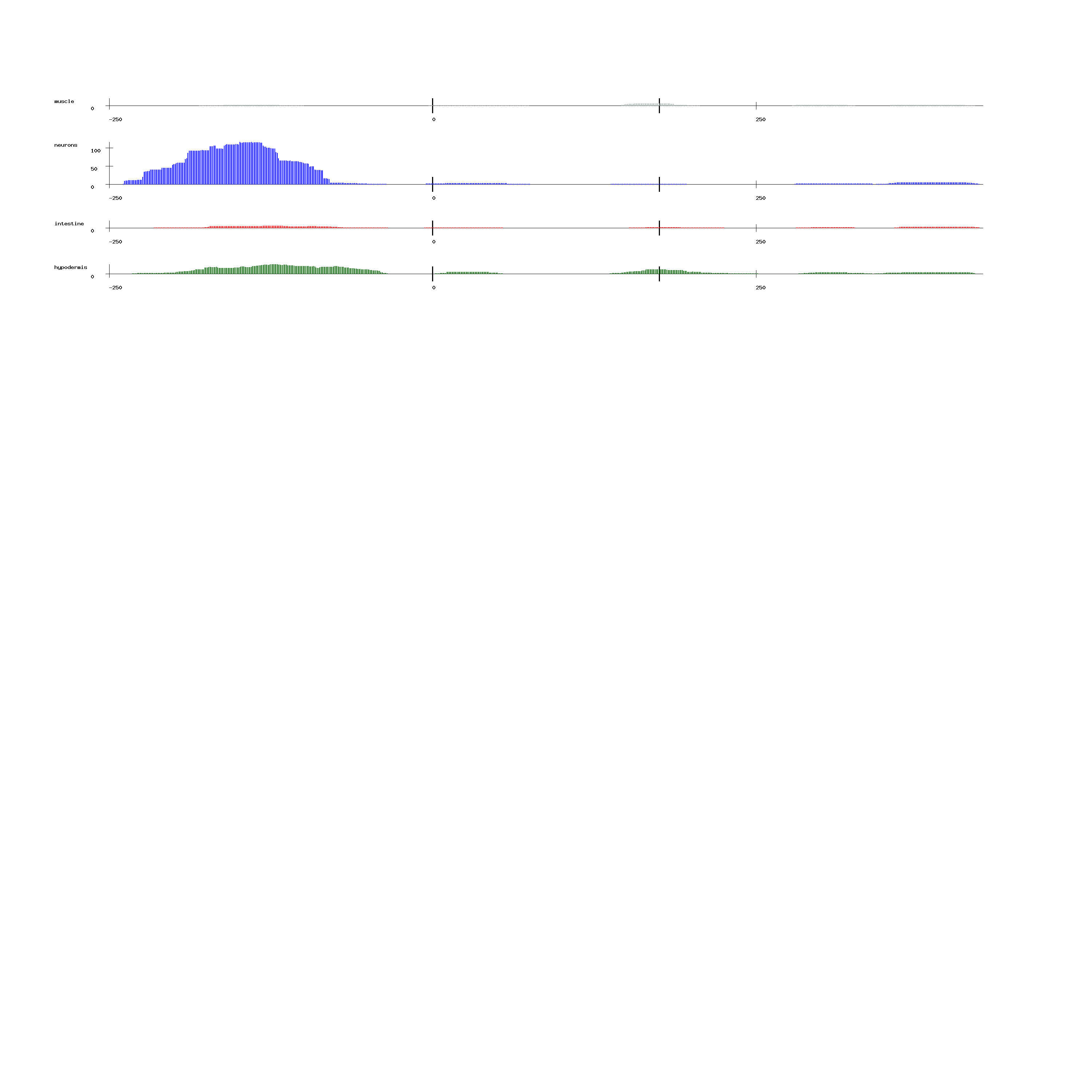

Supplement: Supplementary file 1 [file ijms-24-02970-s001.zip › Supplementary Data S2/2.15000612-15000786.png]

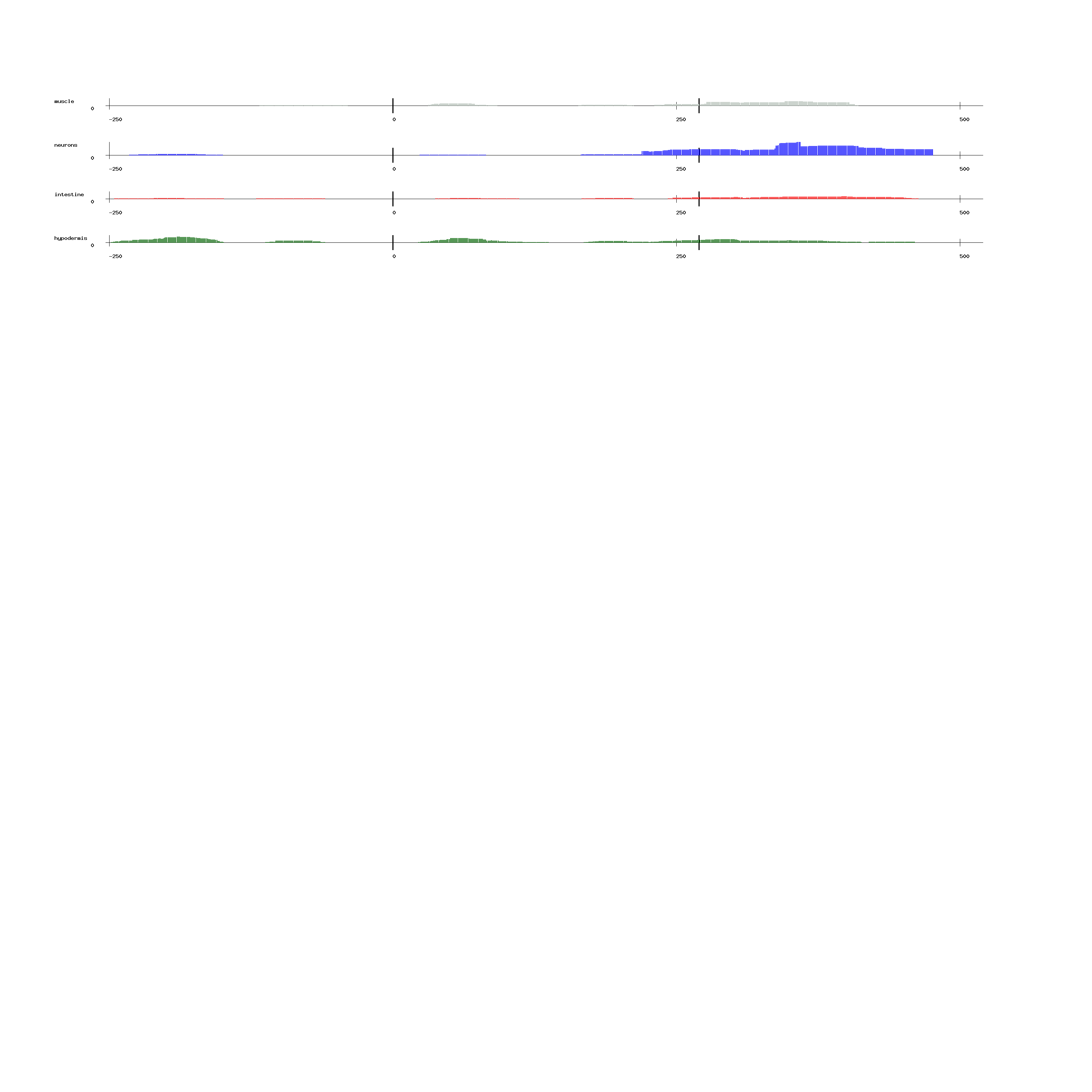

Supplement: Supplementary file 1 [file ijms-24-02970-s001.zip › Supplementary Data S2/2.15000726-15000995.png]

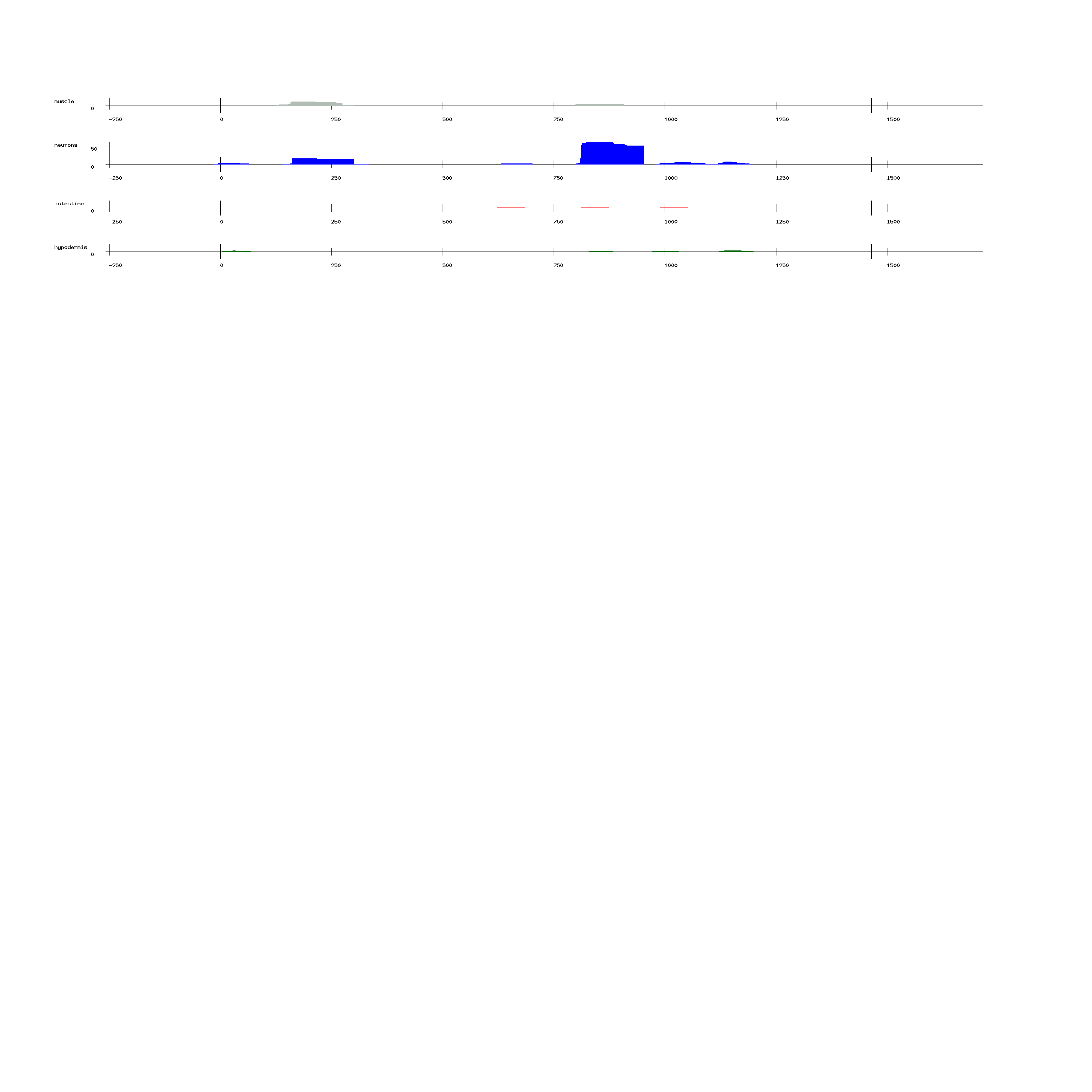

Supplement: Supplementary file 1 [file ijms-24-02970-s001.zip › Supplementary Data S2/2.15186190-15187654.png]

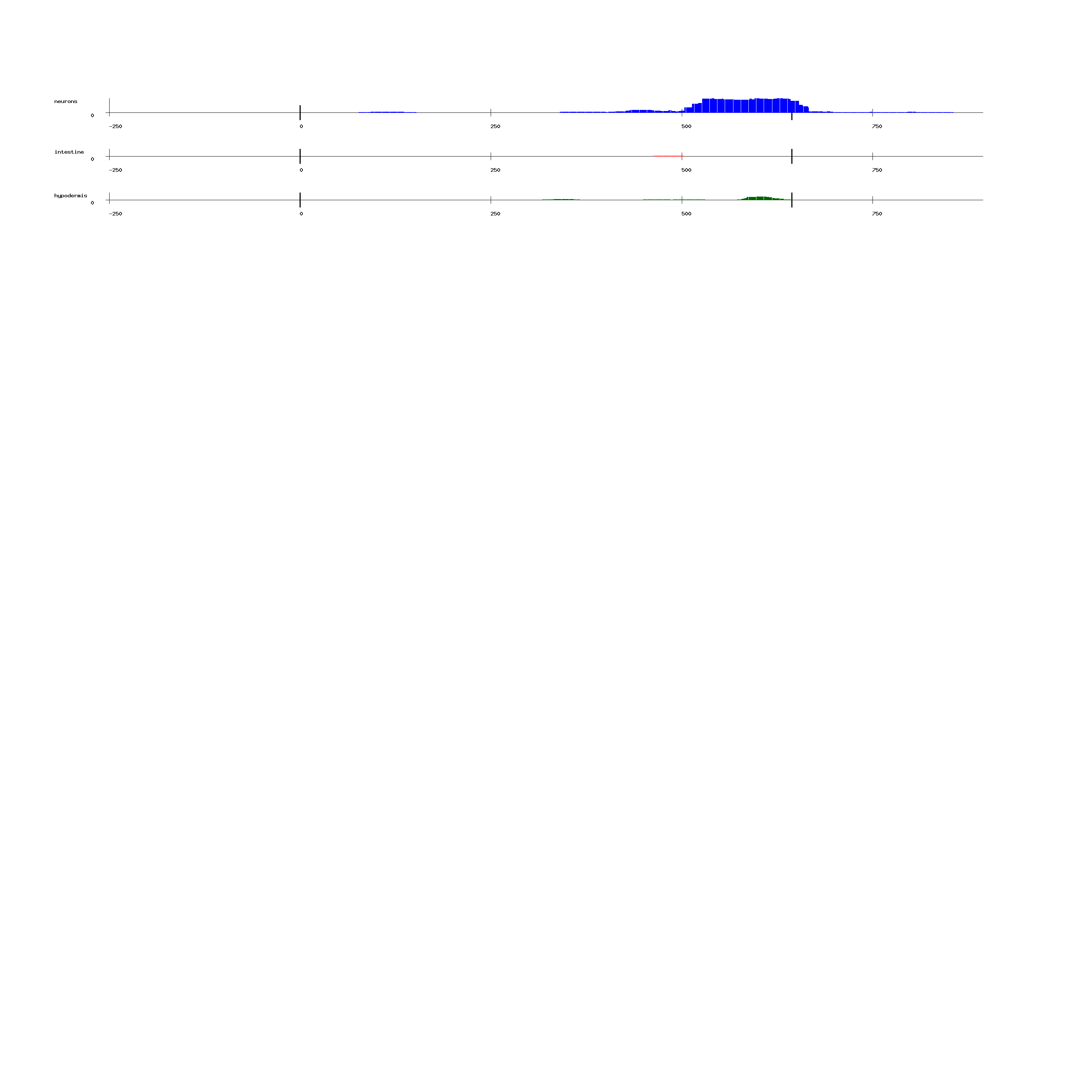

Supplement: Supplementary file 1 [file ijms-24-02970-s001.zip › Supplementary Data S2/2.15188581-15189224.png]

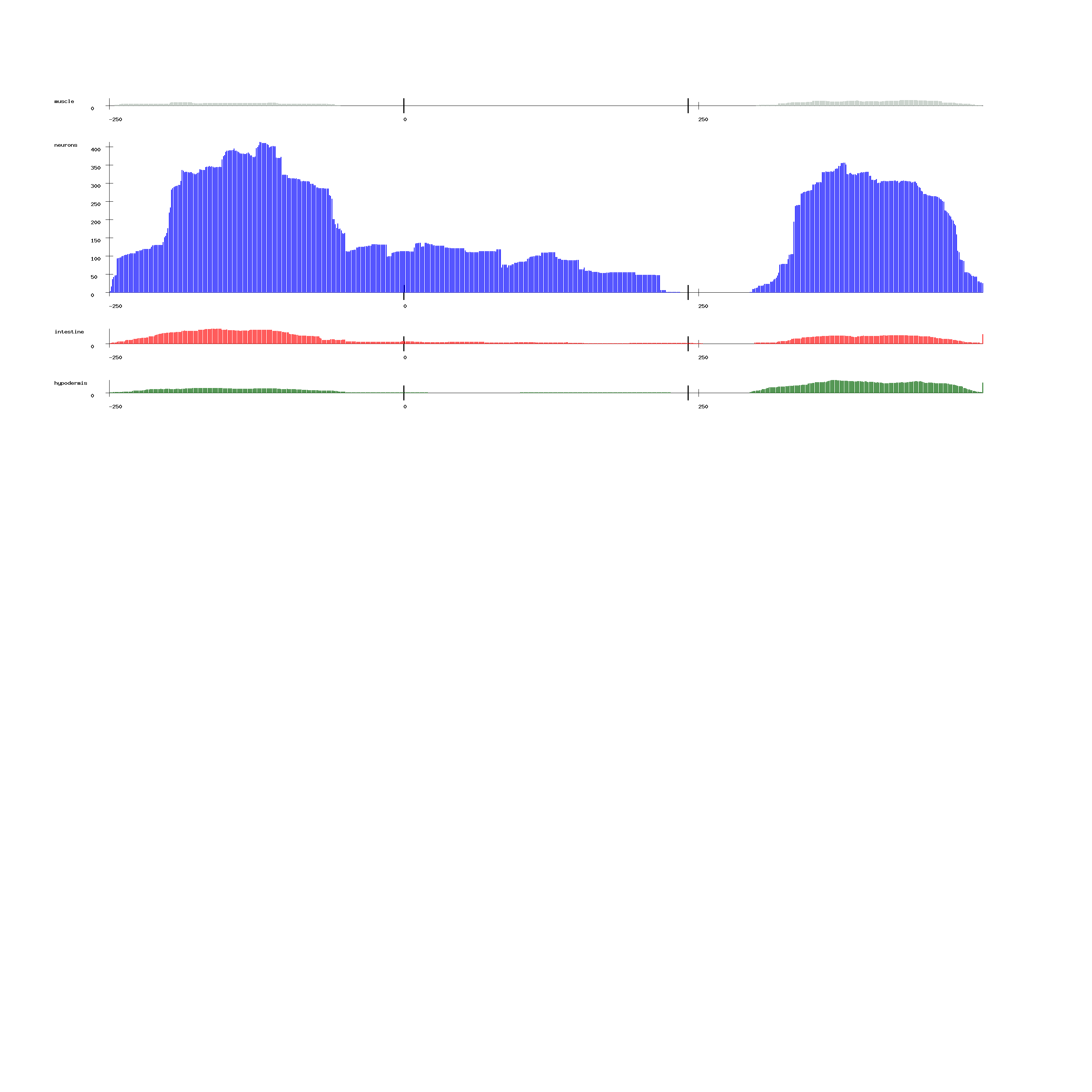

Supplement: Supplementary file 1 [file ijms-24-02970-s001.zip › Supplementary Data S2/2.15278603-15278843.png]

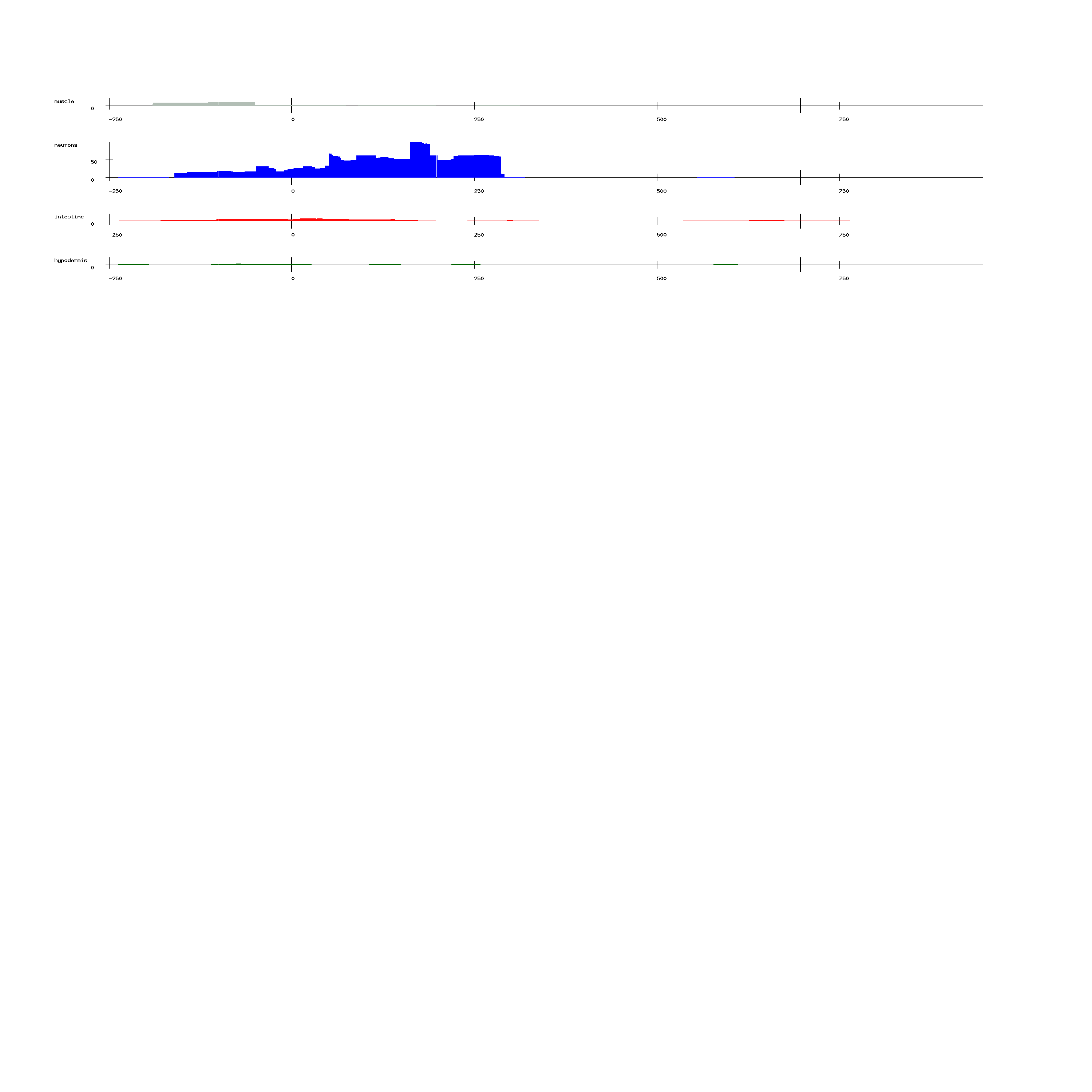

Supplement: Supplementary file 1 [file ijms-24-02970-s001.zip › Supplementary Data S2/2.1564655-1565350.png]

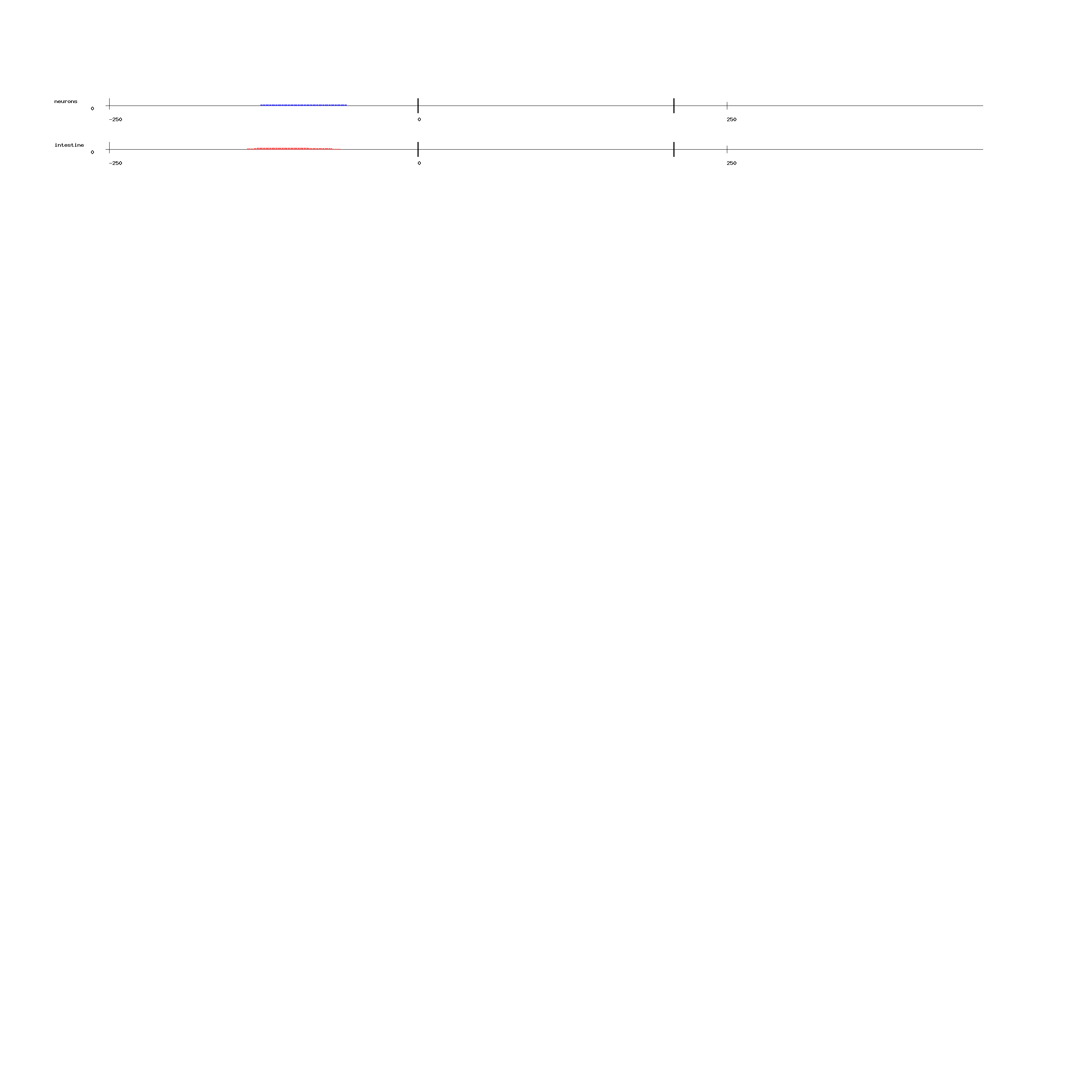

Supplement: Supplementary file 1 [file ijms-24-02970-s001.zip › Supplementary Data S2/2.1793298-1793504.png]

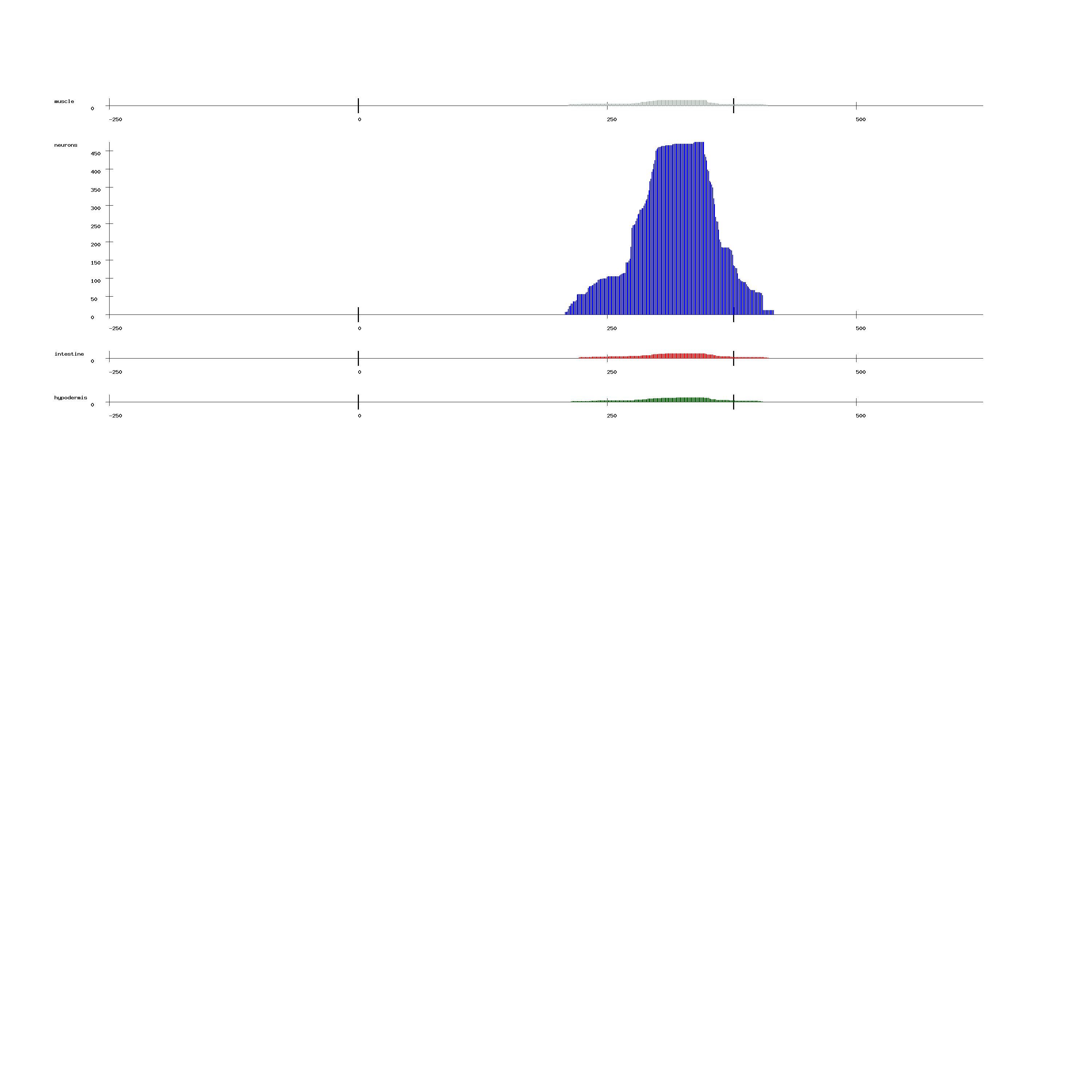

Supplement: Supplementary file 1 [file ijms-24-02970-s001.zip › Supplementary Data S2/2.1796656-1797032.png]

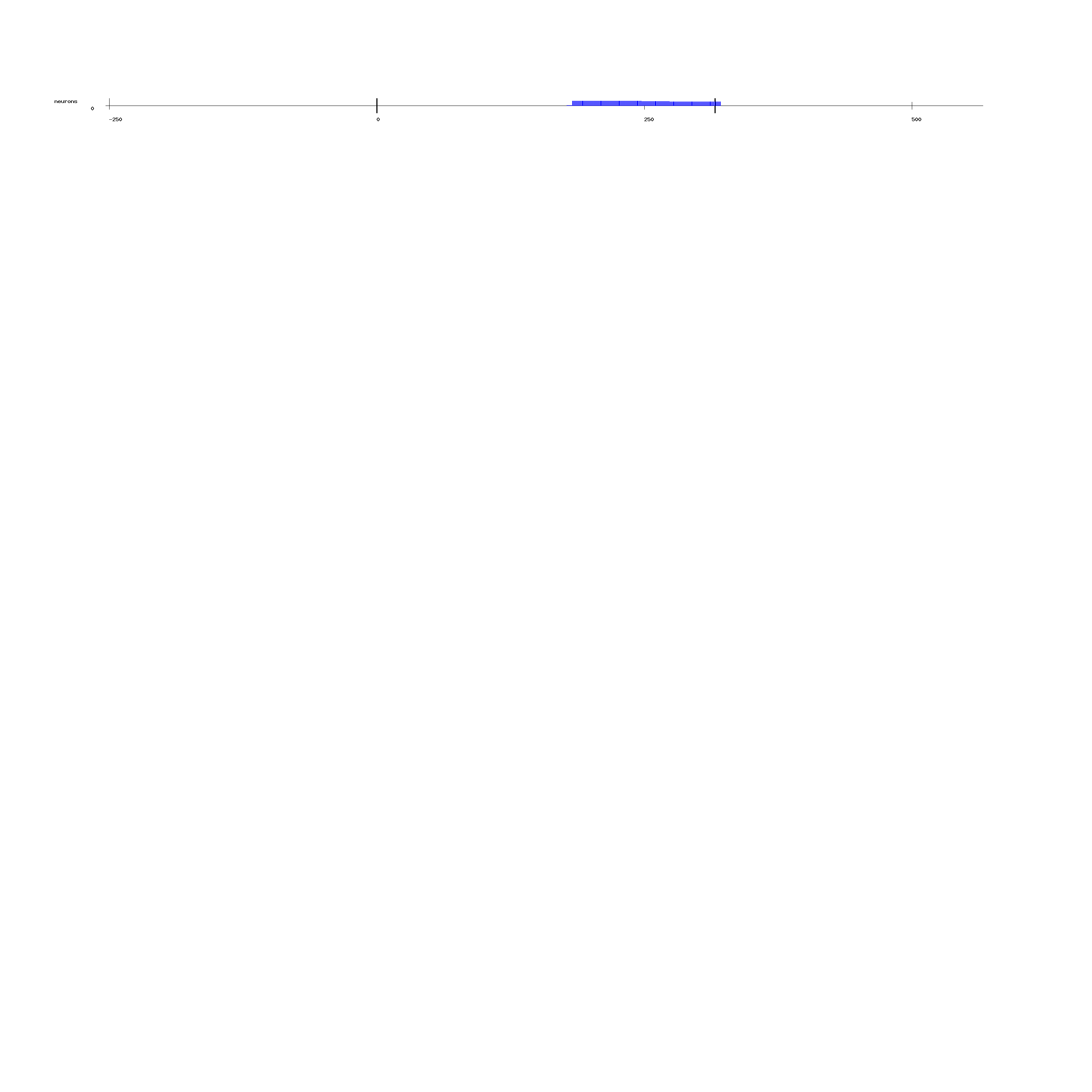

Supplement: Supplementary file 1 [file ijms-24-02970-s001.zip › Supplementary Data S2/2.1858665-1858980.png]

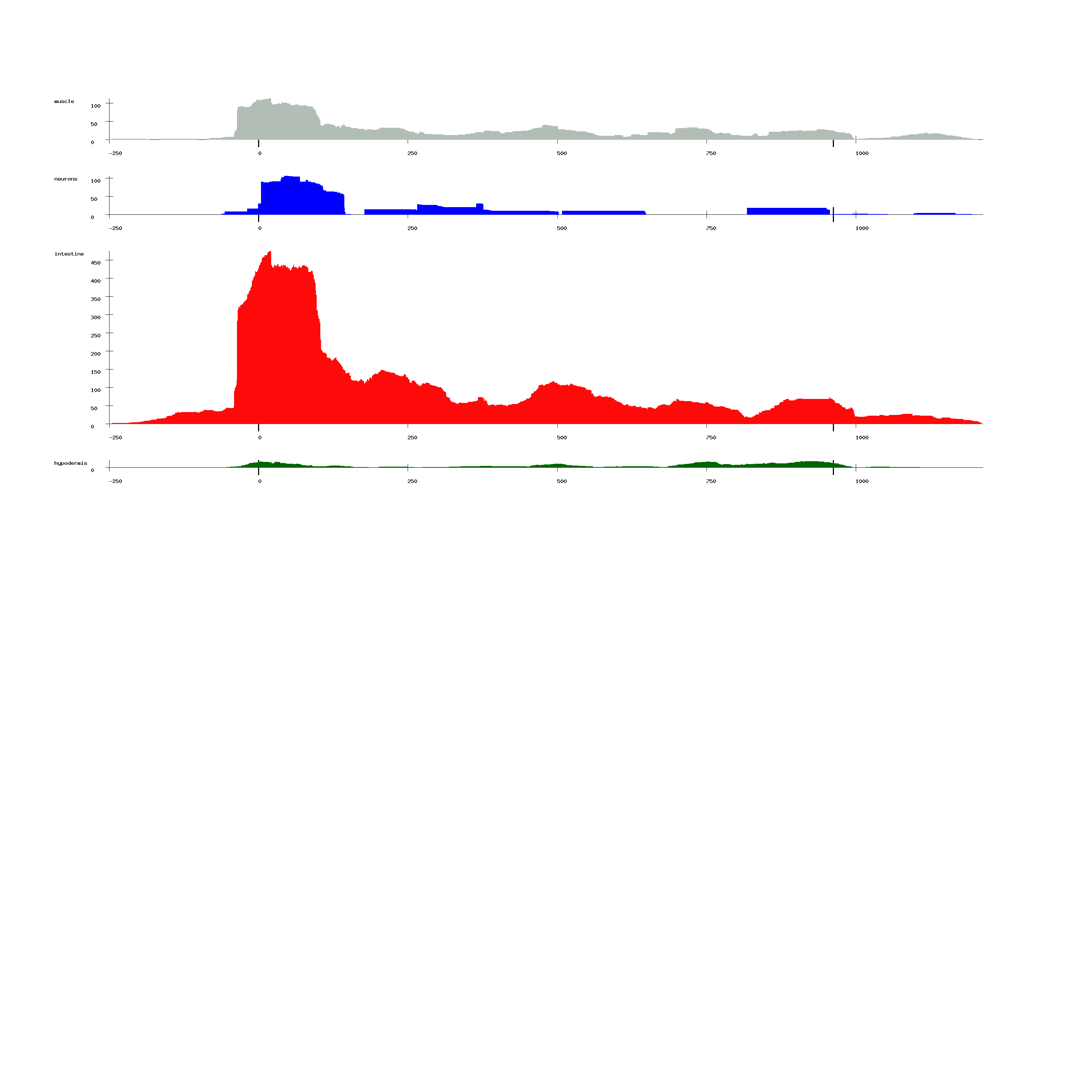

Supplement: Supplementary file 1 [file ijms-24-02970-s001.zip › Supplementary Data S2/2.1962052-1963013.png]

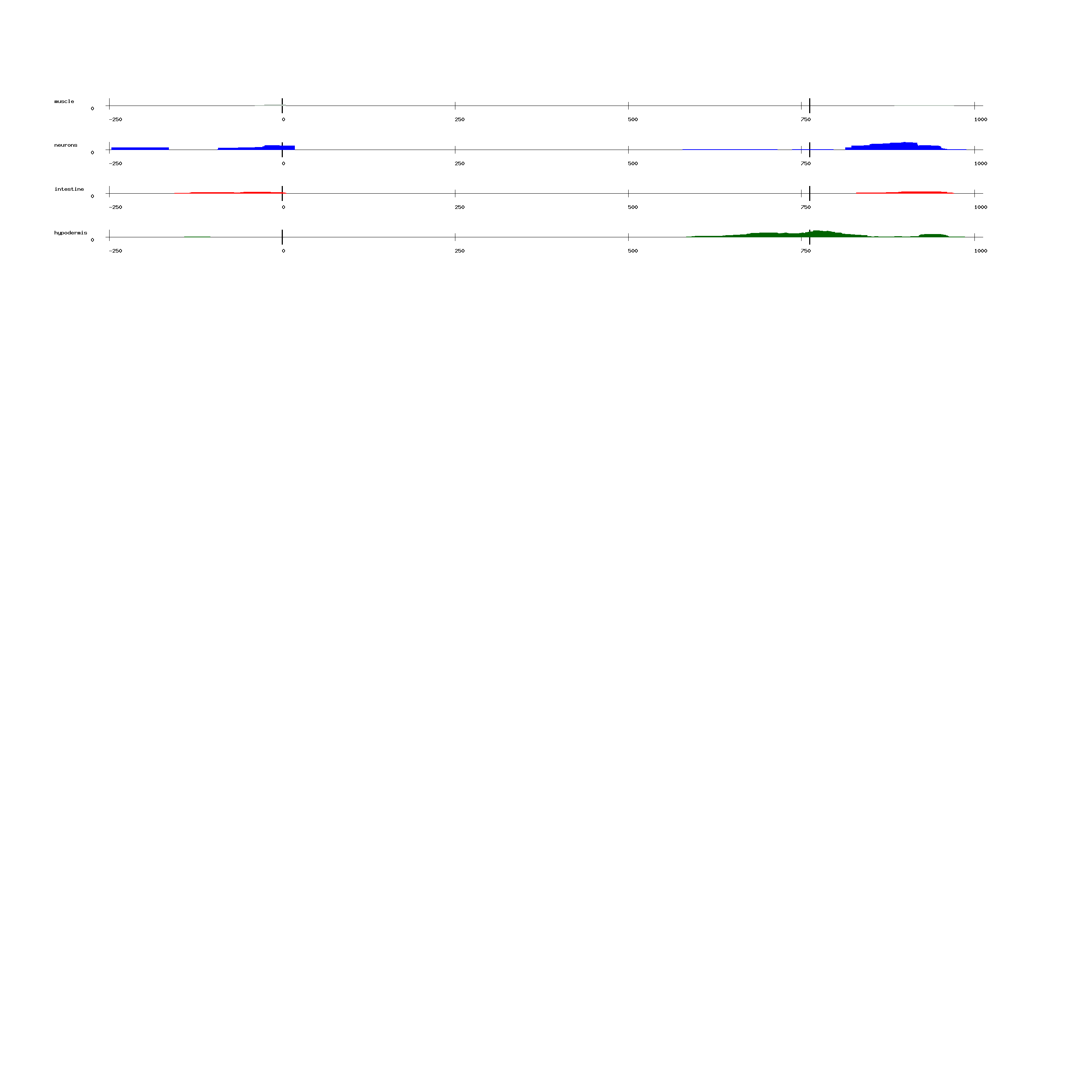

Supplement: Supplementary file 1 [file ijms-24-02970-s001.zip › Supplementary Data S2/2.1968841-1969602.png]

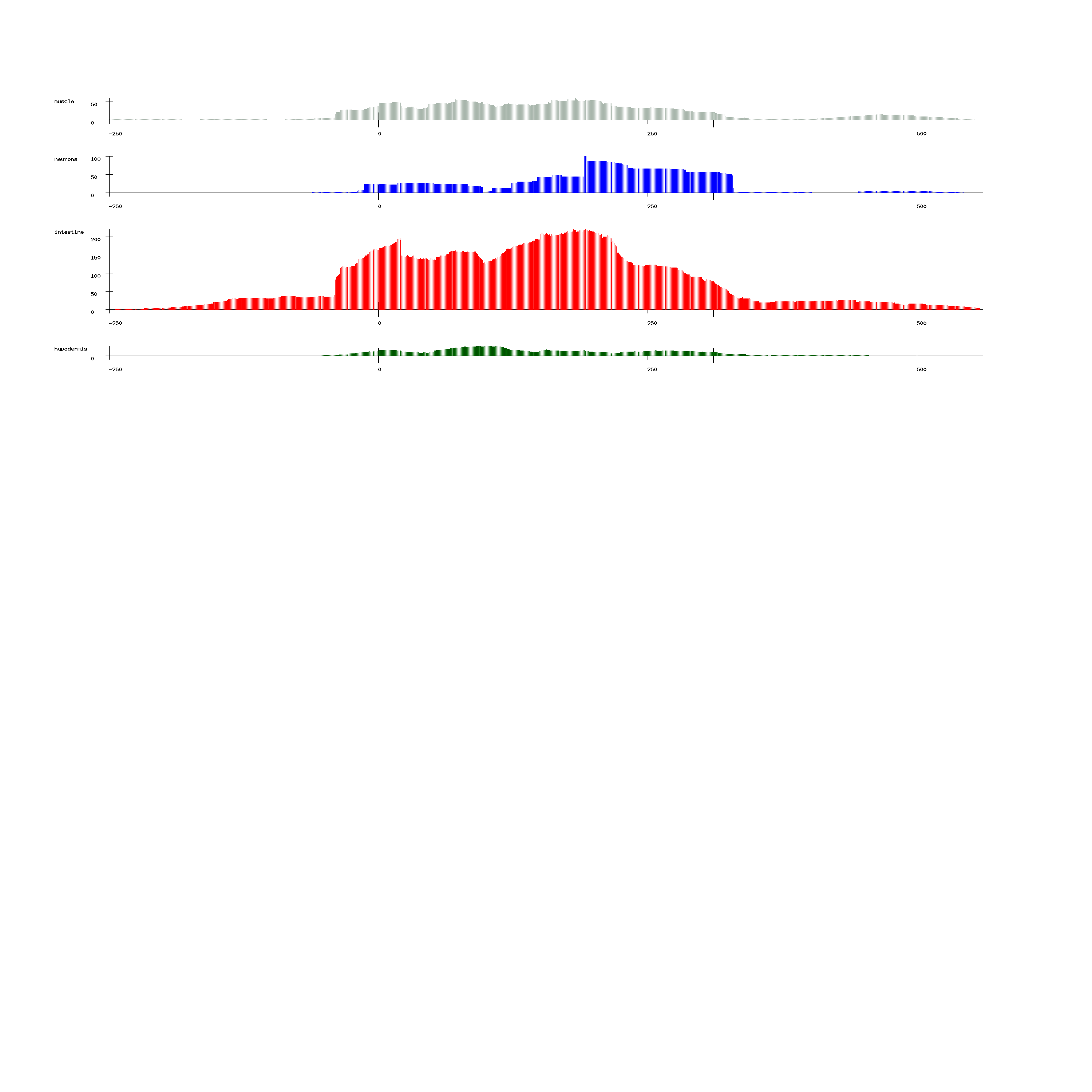

Supplement: Supplementary file 1 [file ijms-24-02970-s001.zip › Supplementary Data S2/2.1970474-1970784.png]

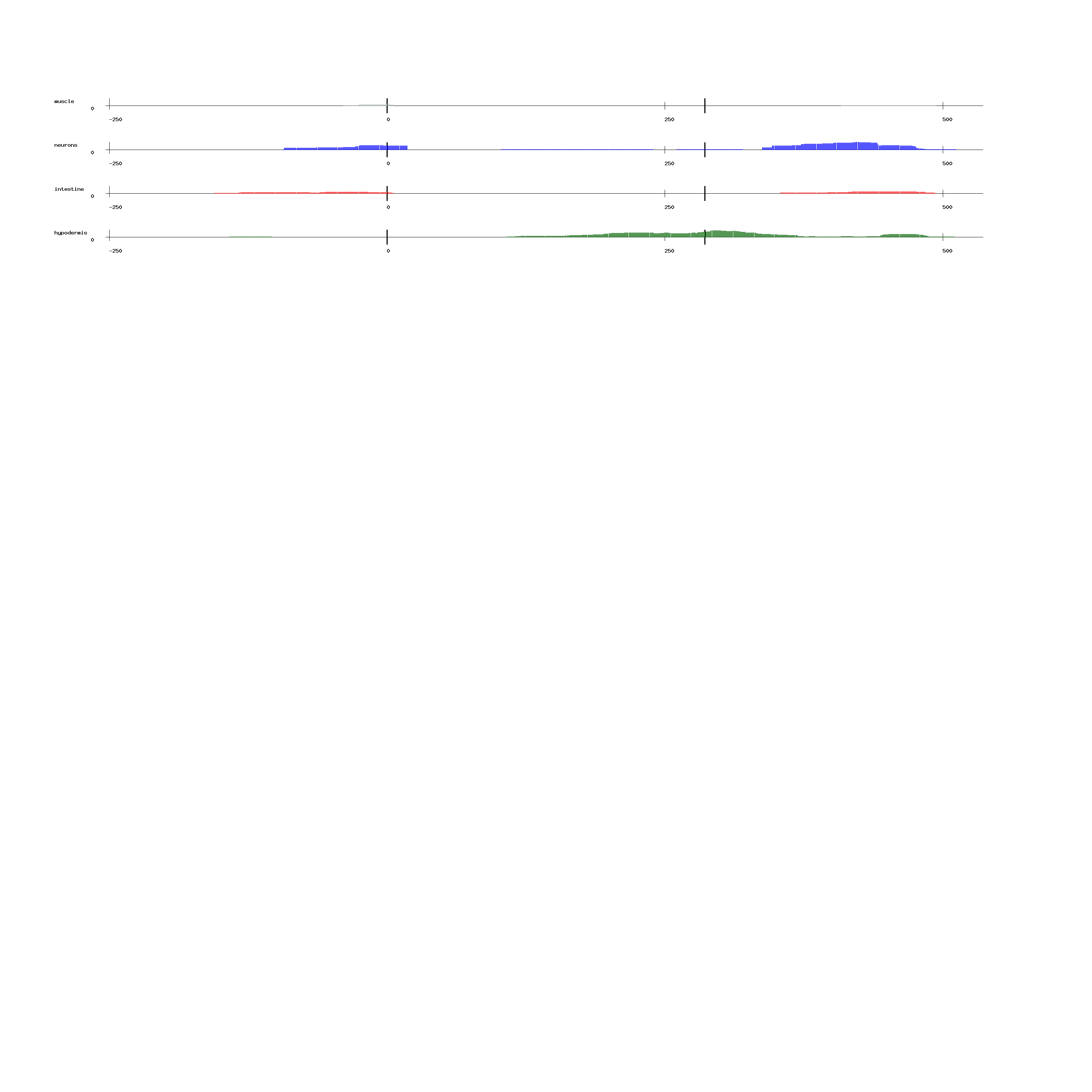

Supplement: Supplementary file 1 [file ijms-24-02970-s001.zip › Supplementary Data S2/2.1976612-1976897.png]

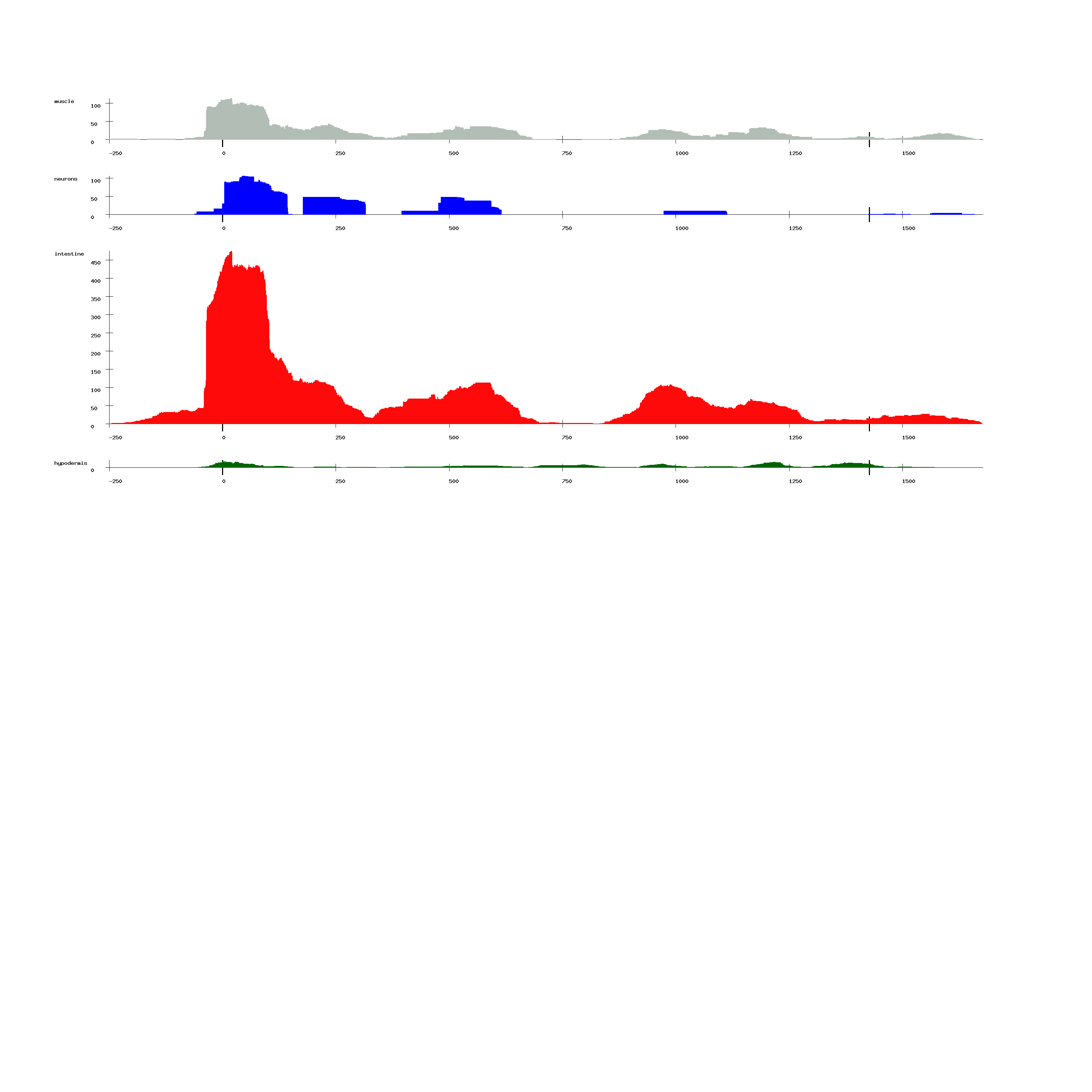

Supplement: Supplementary file 1 [file ijms-24-02970-s001.zip › Supplementary Data S2/2.1977769-1979195.png]

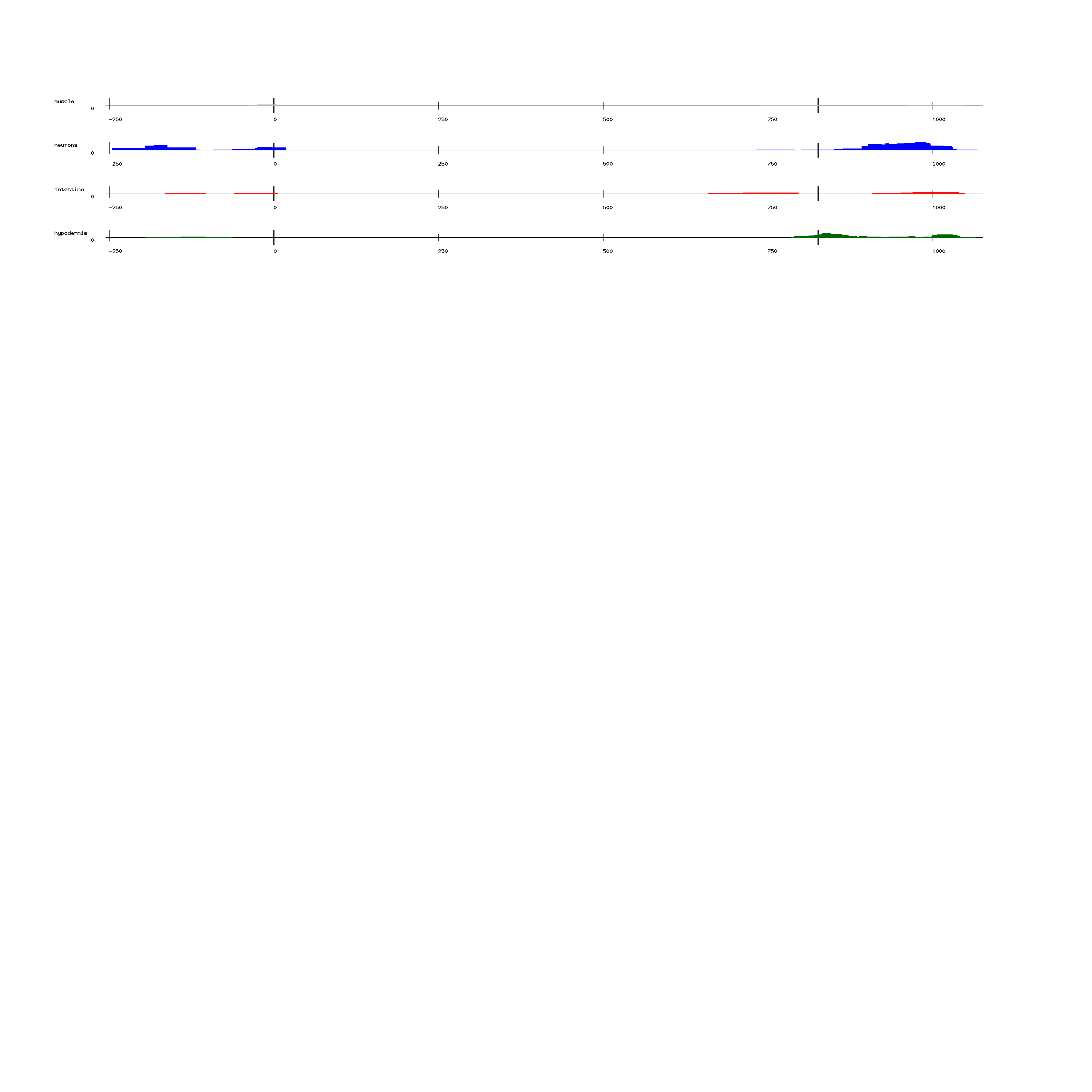

Supplement: Supplementary file 1 [file ijms-24-02970-s001.zip › Supplementary Data S2/2.1985022-1985847.png]

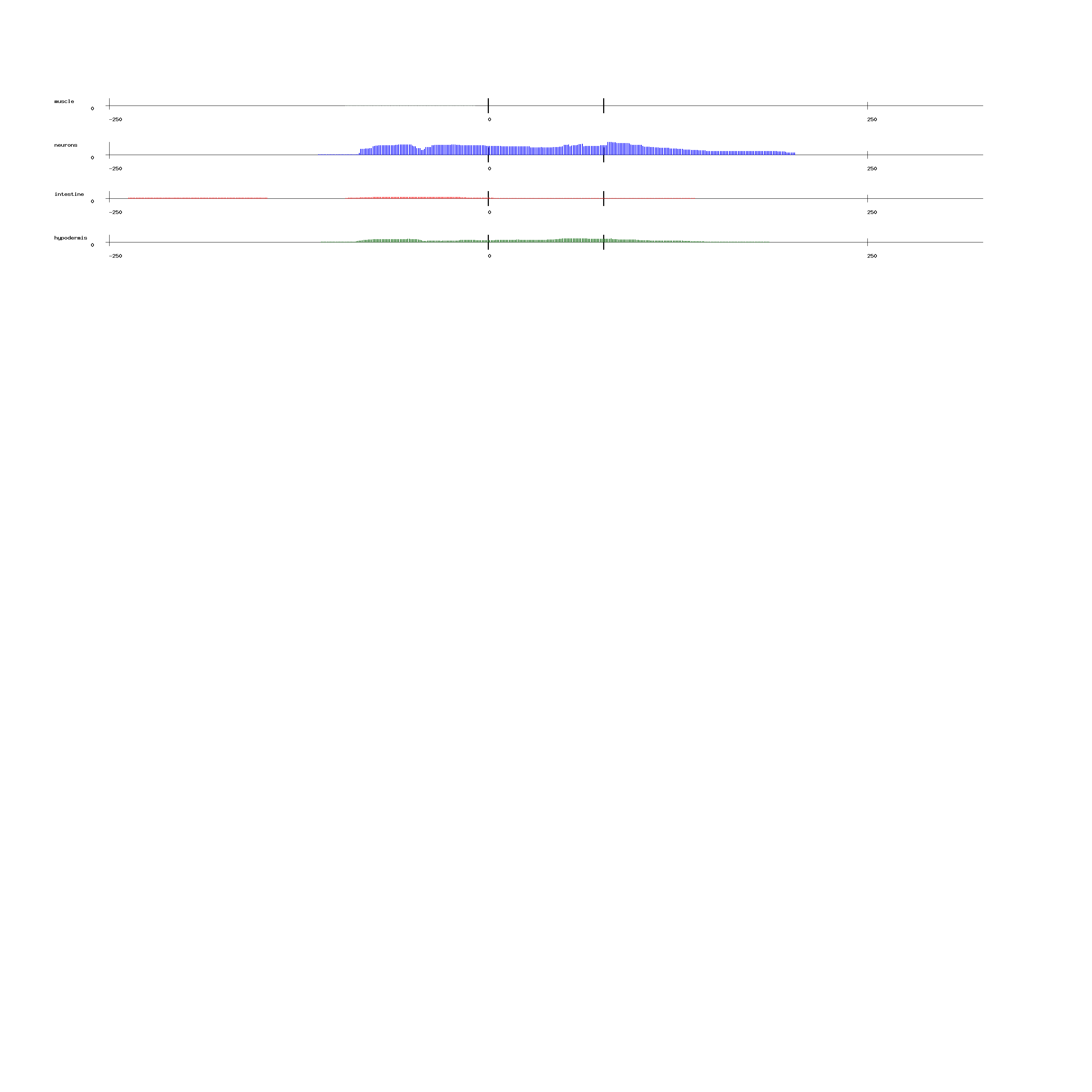

Supplement: Supplementary file 1 [file ijms-24-02970-s001.zip › Supplementary Data S2/2.2002012-2002087.png]

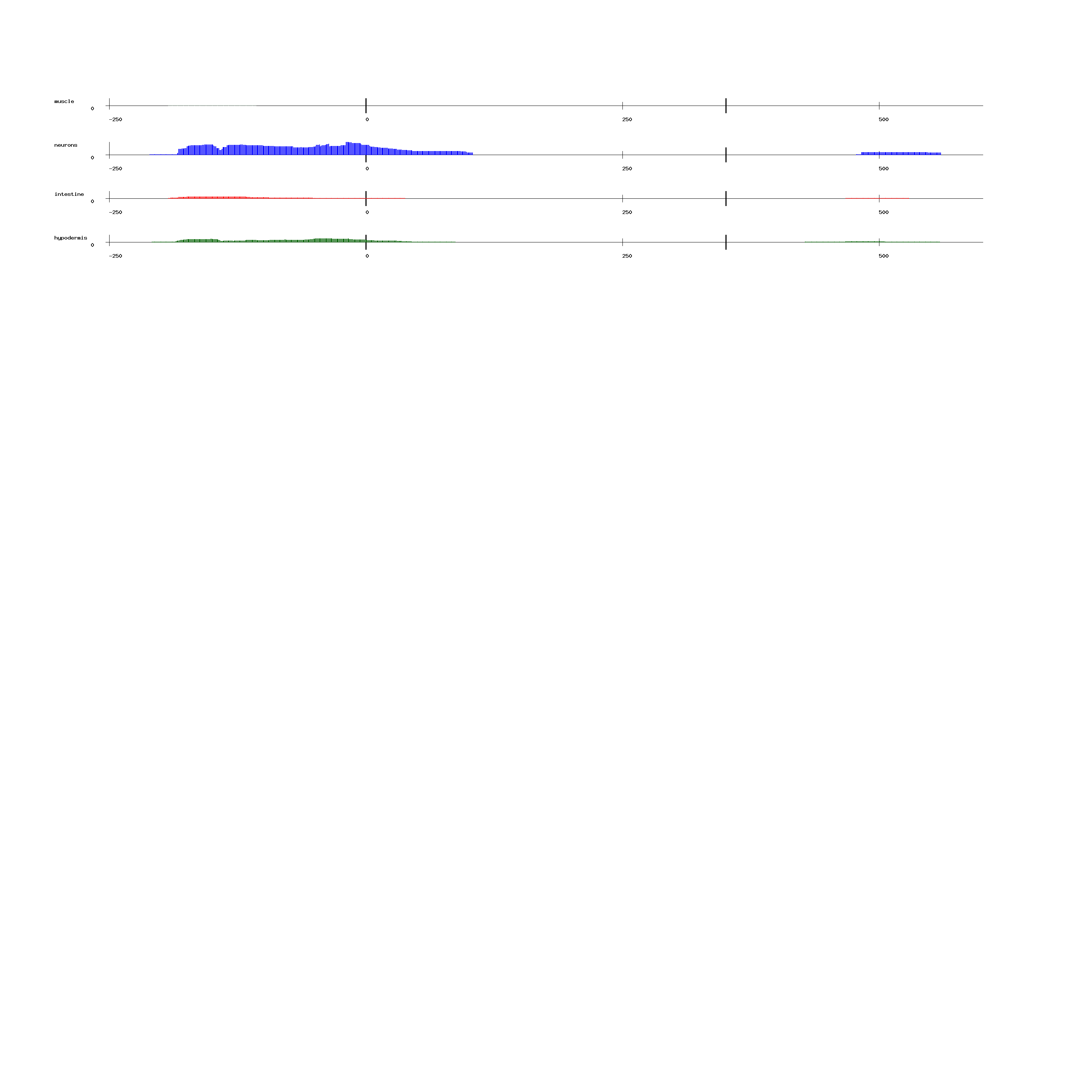

Supplement: Supplementary file 1 [file ijms-24-02970-s001.zip › Supplementary Data S2/2.2002110-2002460.png]

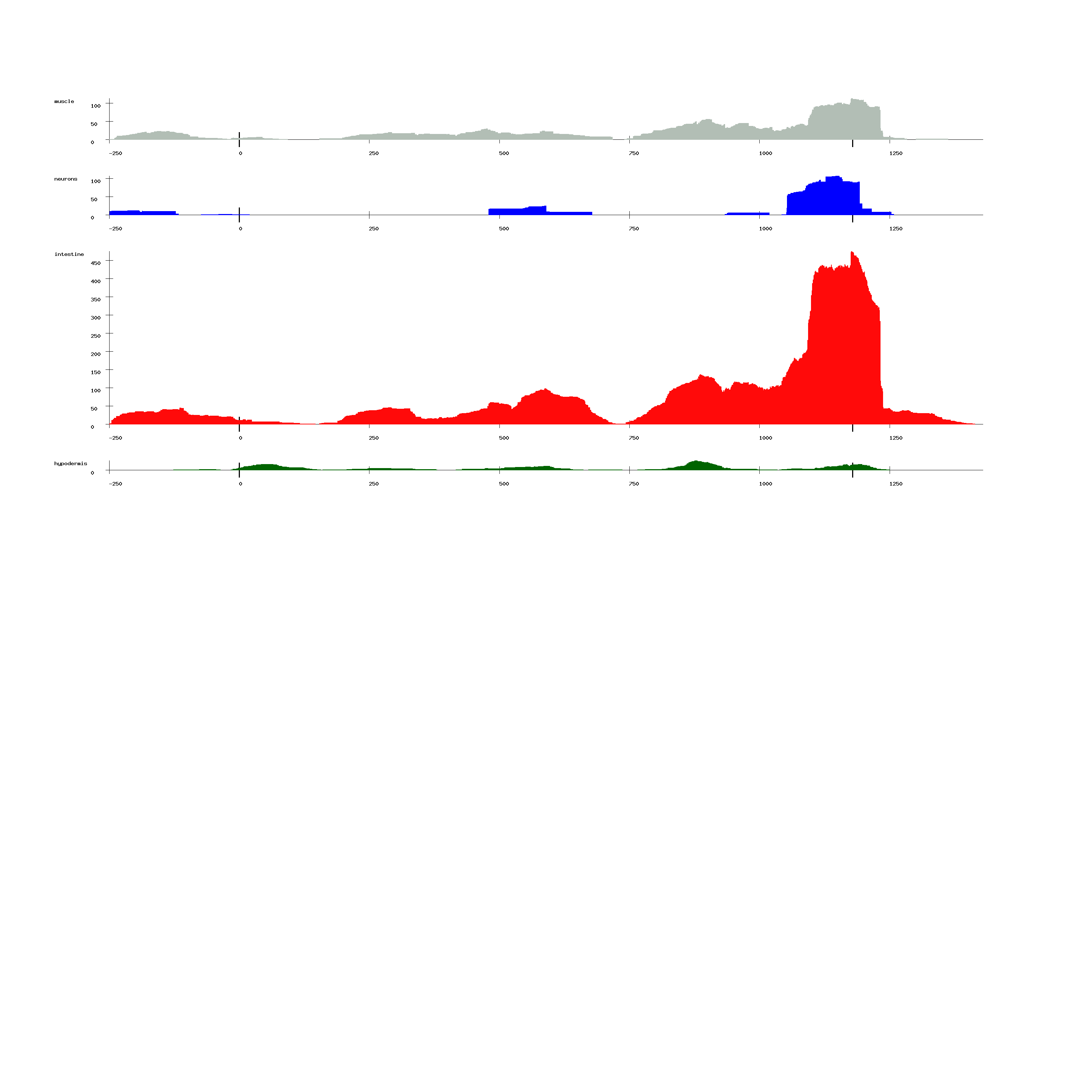

Supplement: Supplementary file 1 [file ijms-24-02970-s001.zip › Supplementary Data S2/2.2008283-2009461.png]

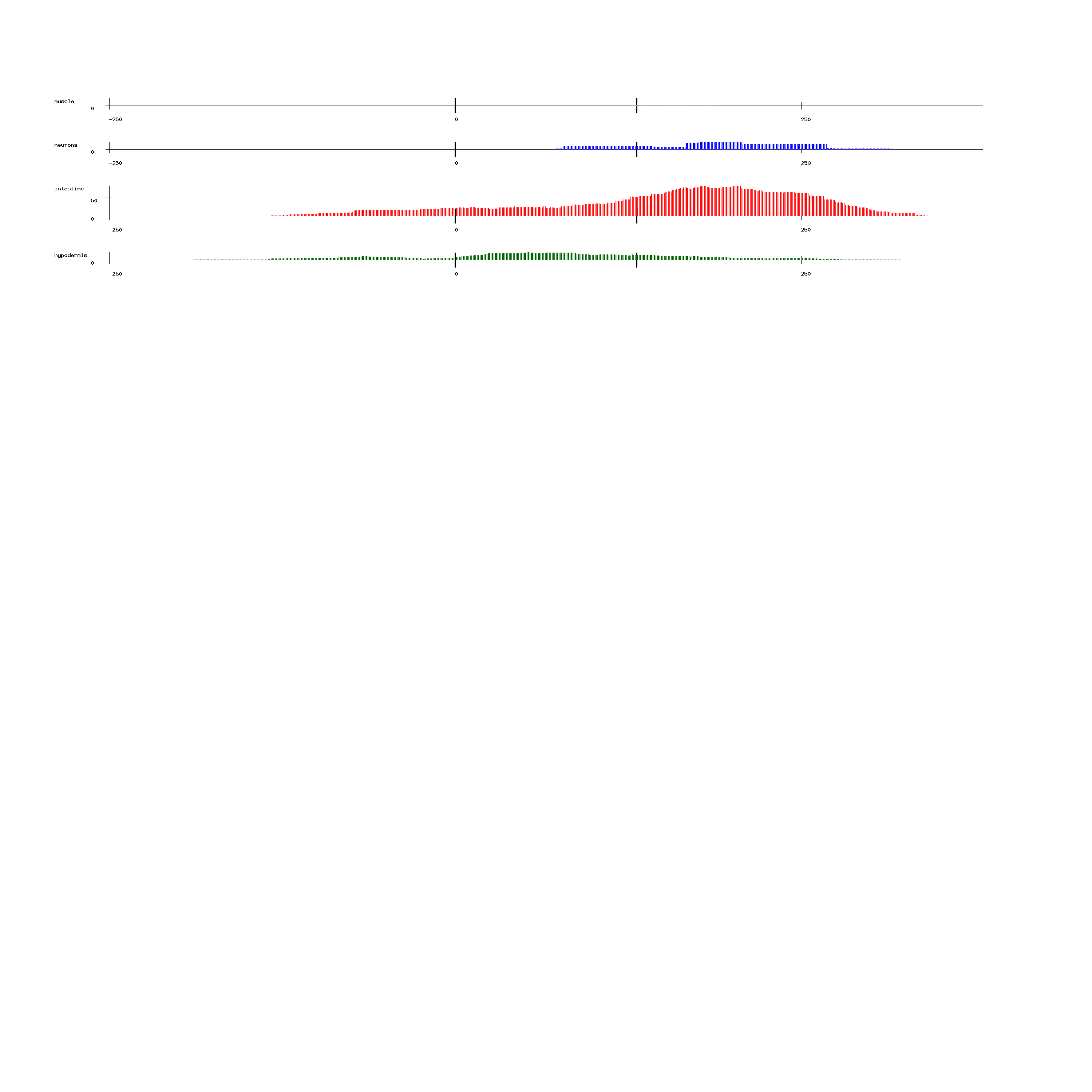

Supplement: Supplementary file 1 [file ijms-24-02970-s001.zip › Supplementary Data S2/2.2157814-2157944.png]

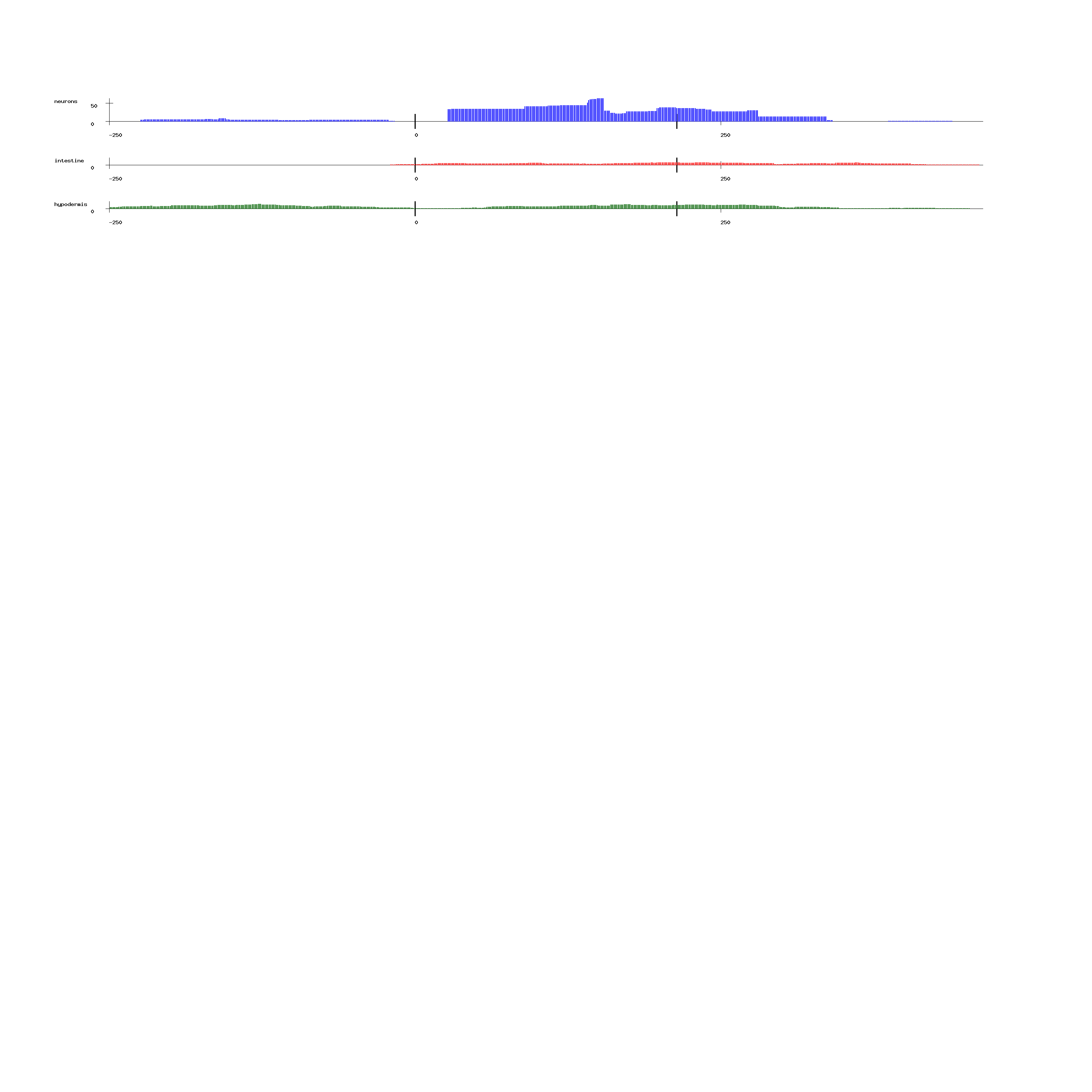

Supplement: Supplementary file 1 [file ijms-24-02970-s001.zip › Supplementary Data S2/2.2273842-2274055.png]

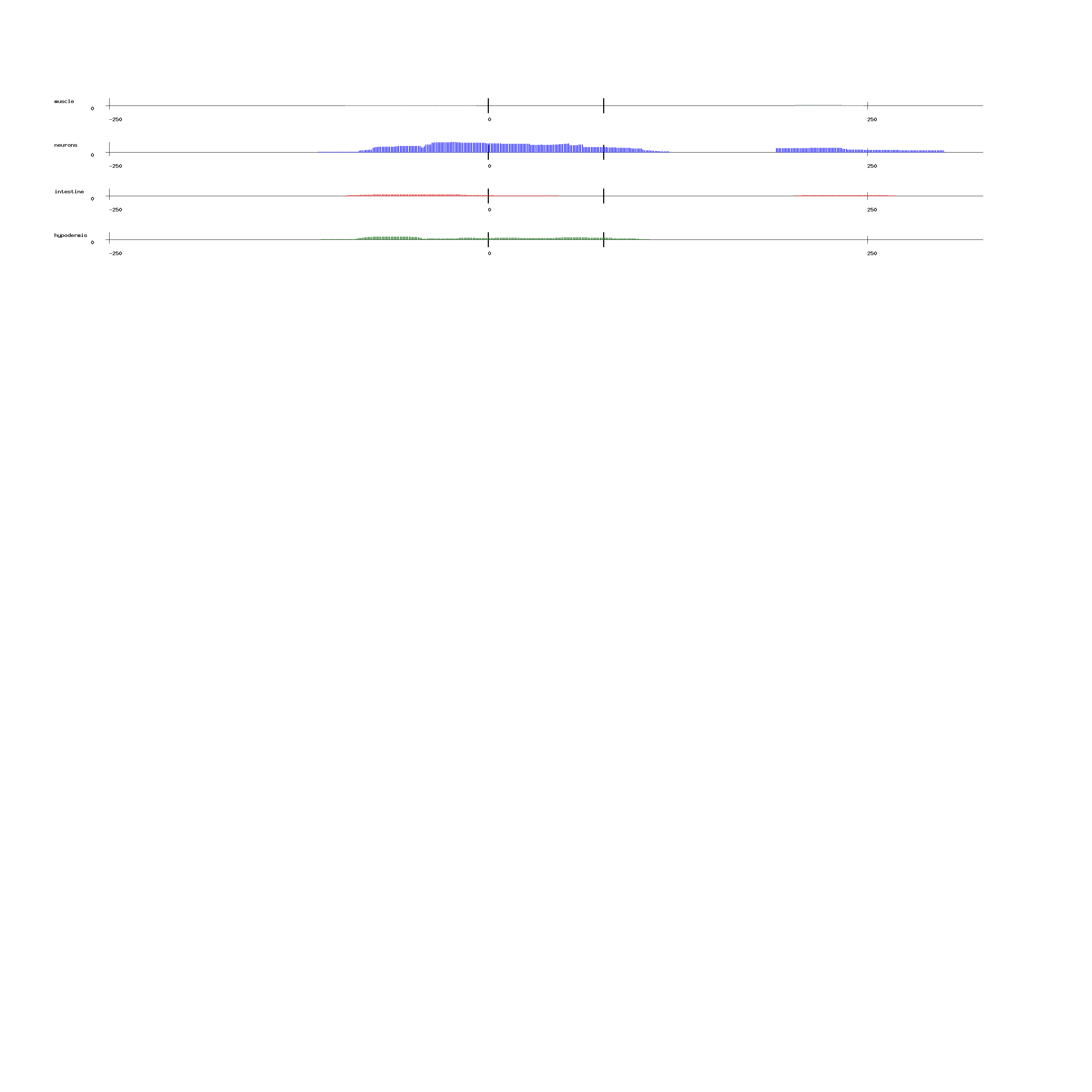

Supplement: Supplementary file 1 [file ijms-24-02970-s001.zip › Supplementary Data S2/2.2386288-2386363.png]

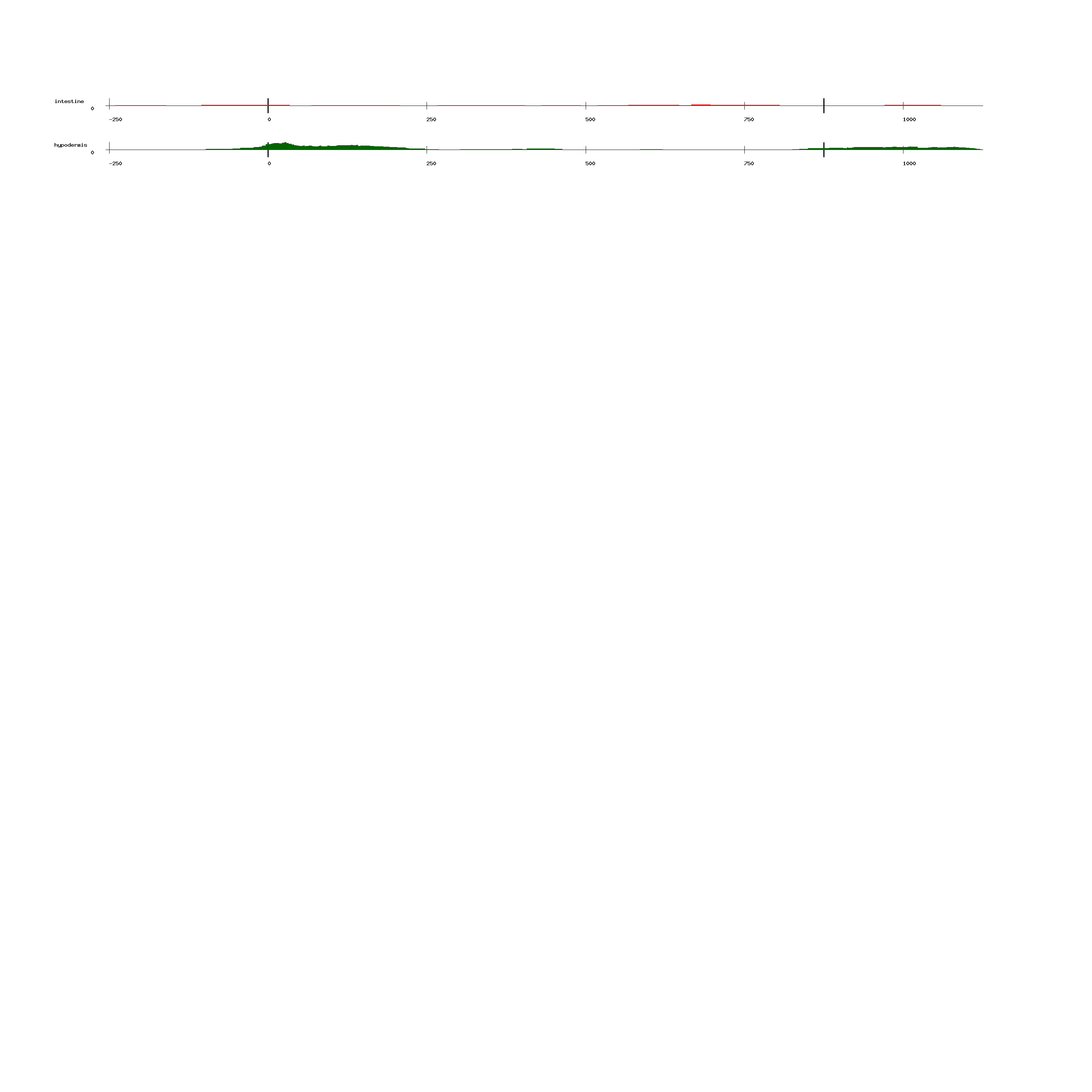

Supplement: Supplementary file 1 [file ijms-24-02970-s001.zip › Supplementary Data S2/2.2529567-2530441.png]

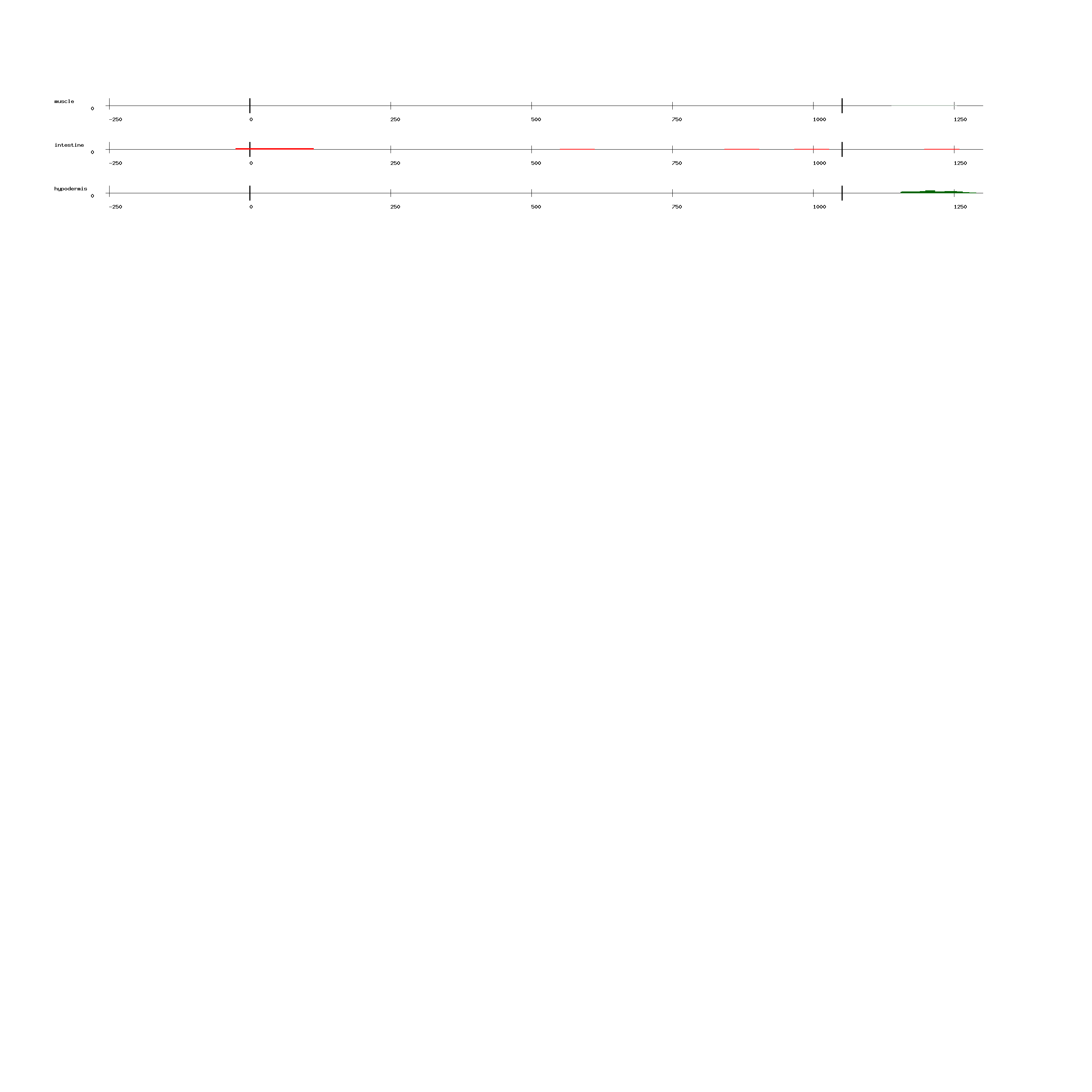

Supplement: Supplementary file 1 [file ijms-24-02970-s001.zip › Supplementary Data S2/2.2577037-2578087.png]

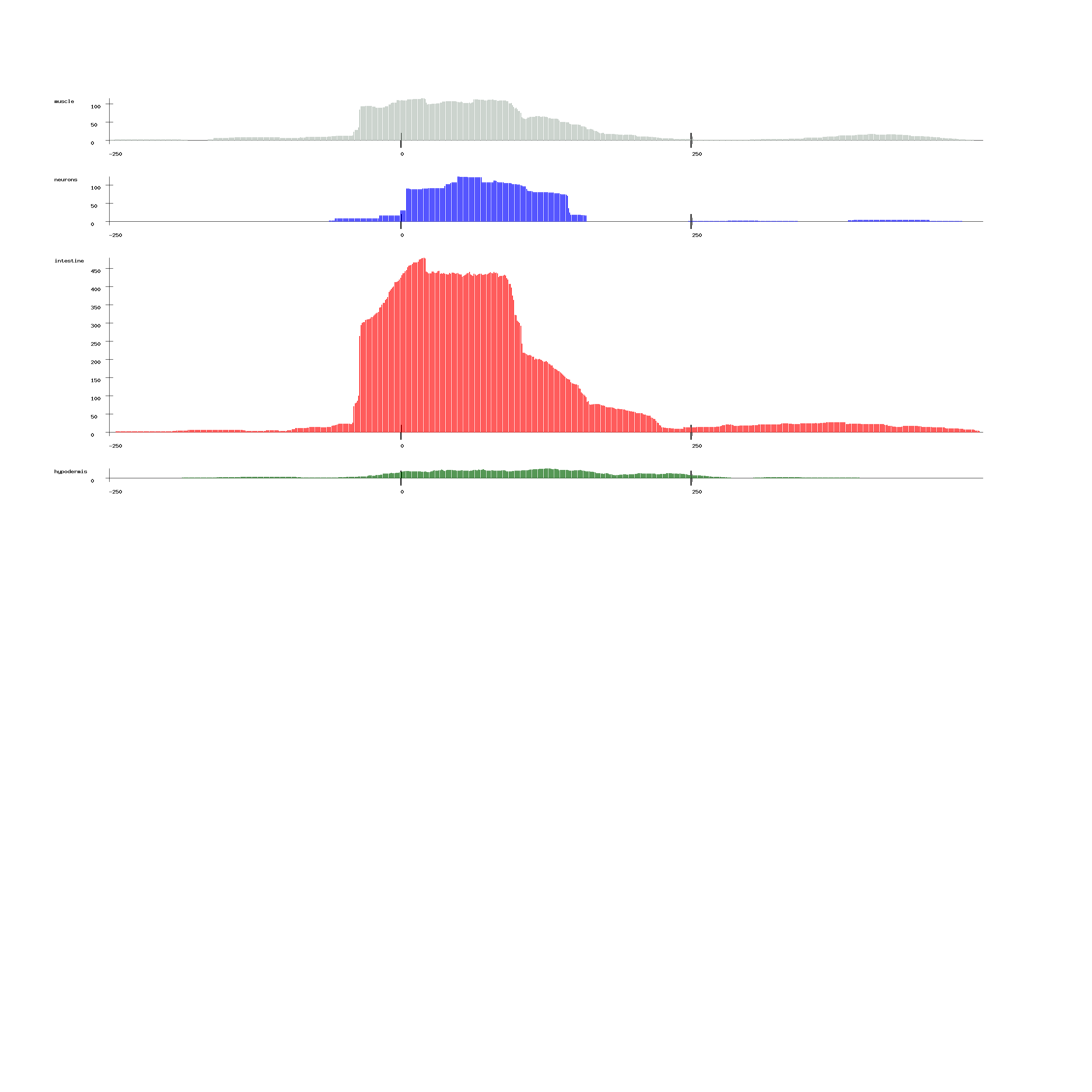

Supplement: Supplementary file 1 [file ijms-24-02970-s001.zip › Supplementary Data S2/2.2593754-2594002.png]

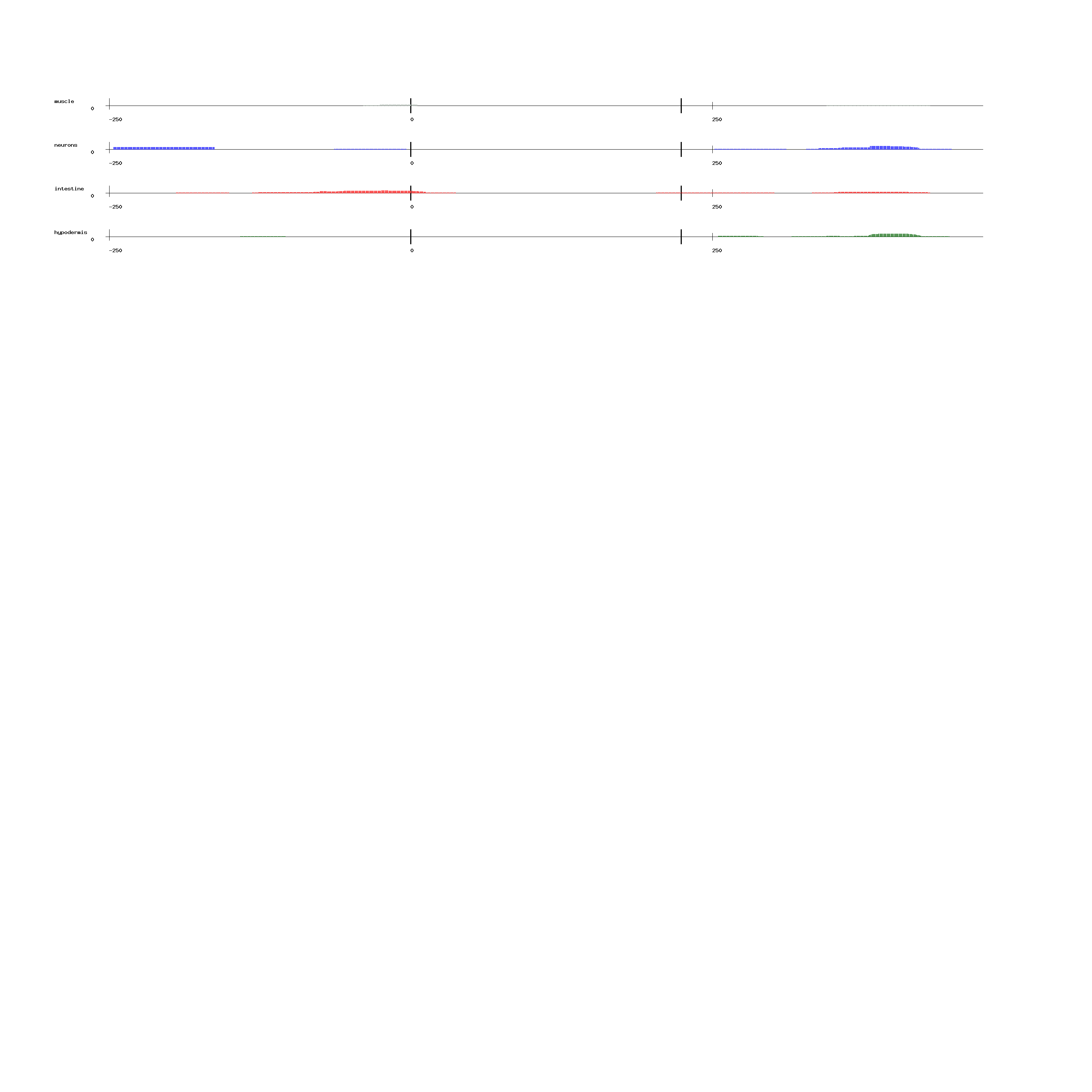

Supplement: Supplementary file 1 [file ijms-24-02970-s001.zip › Supplementary Data S2/2.2599830-2600053.png]

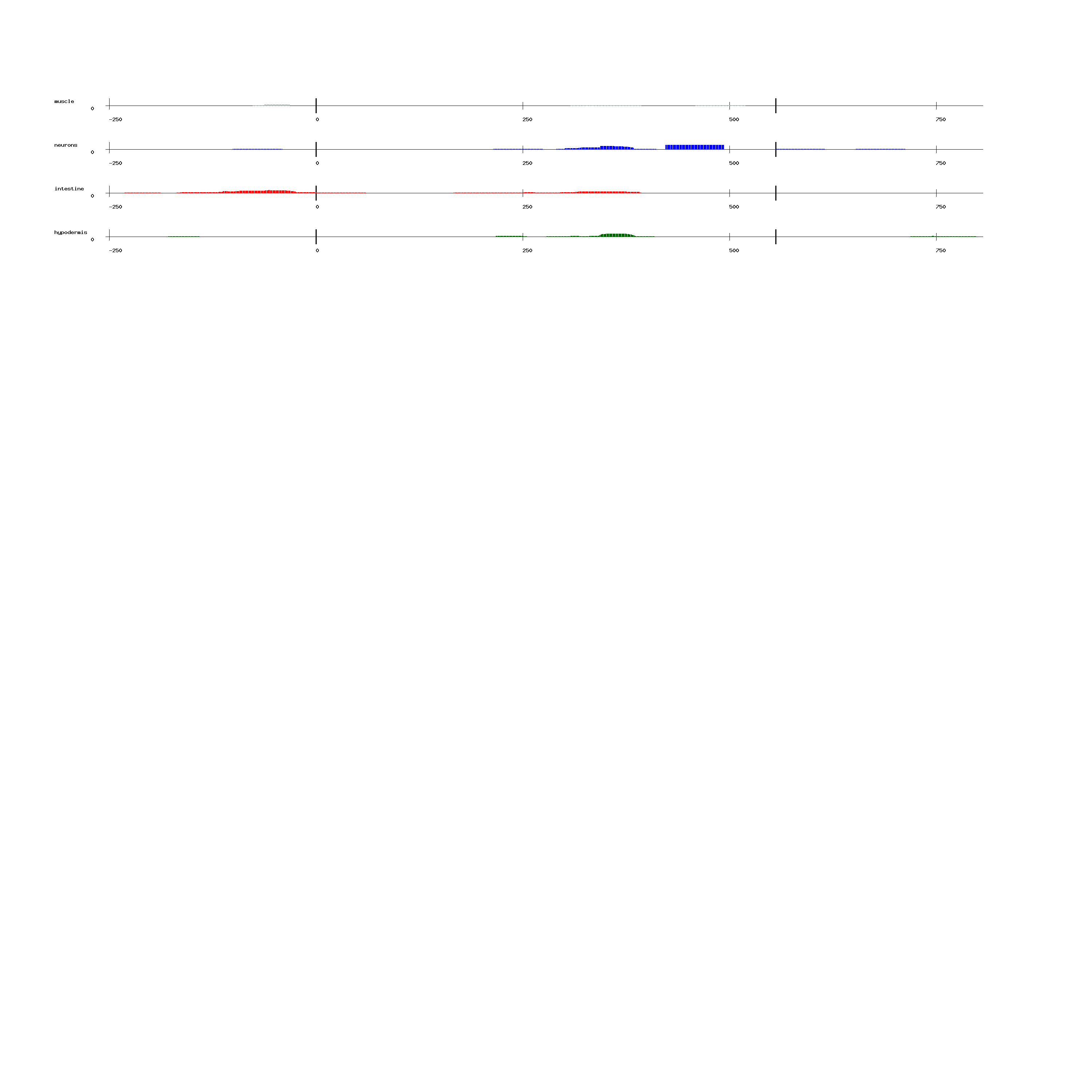

Supplement: Supplementary file 1 [file ijms-24-02970-s001.zip › Supplementary Data S2/2.2599867-2600422.png]

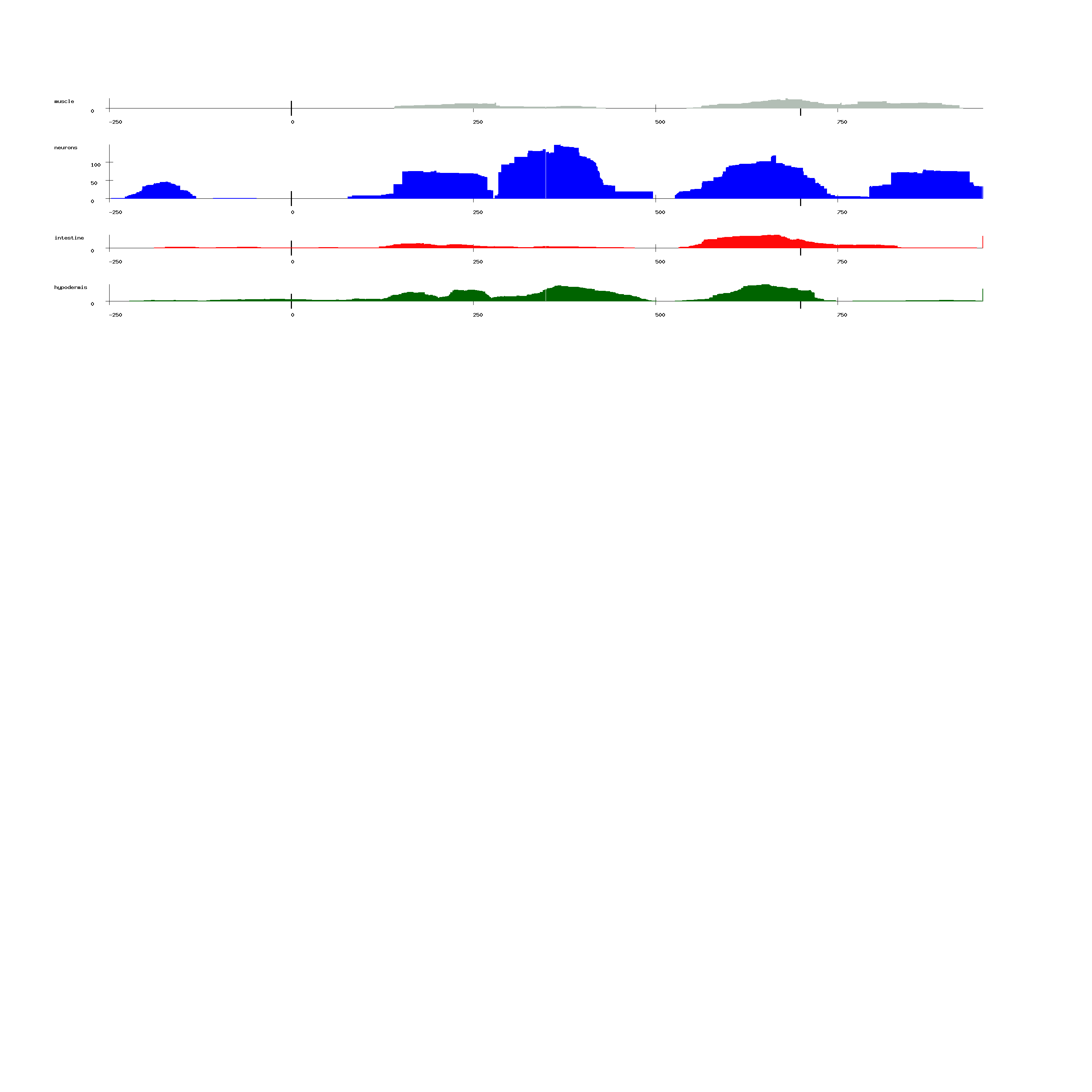

Supplement: Supplementary file 1 [file ijms-24-02970-s001.zip › Supplementary Data S2/2.2712830-2713528.png]

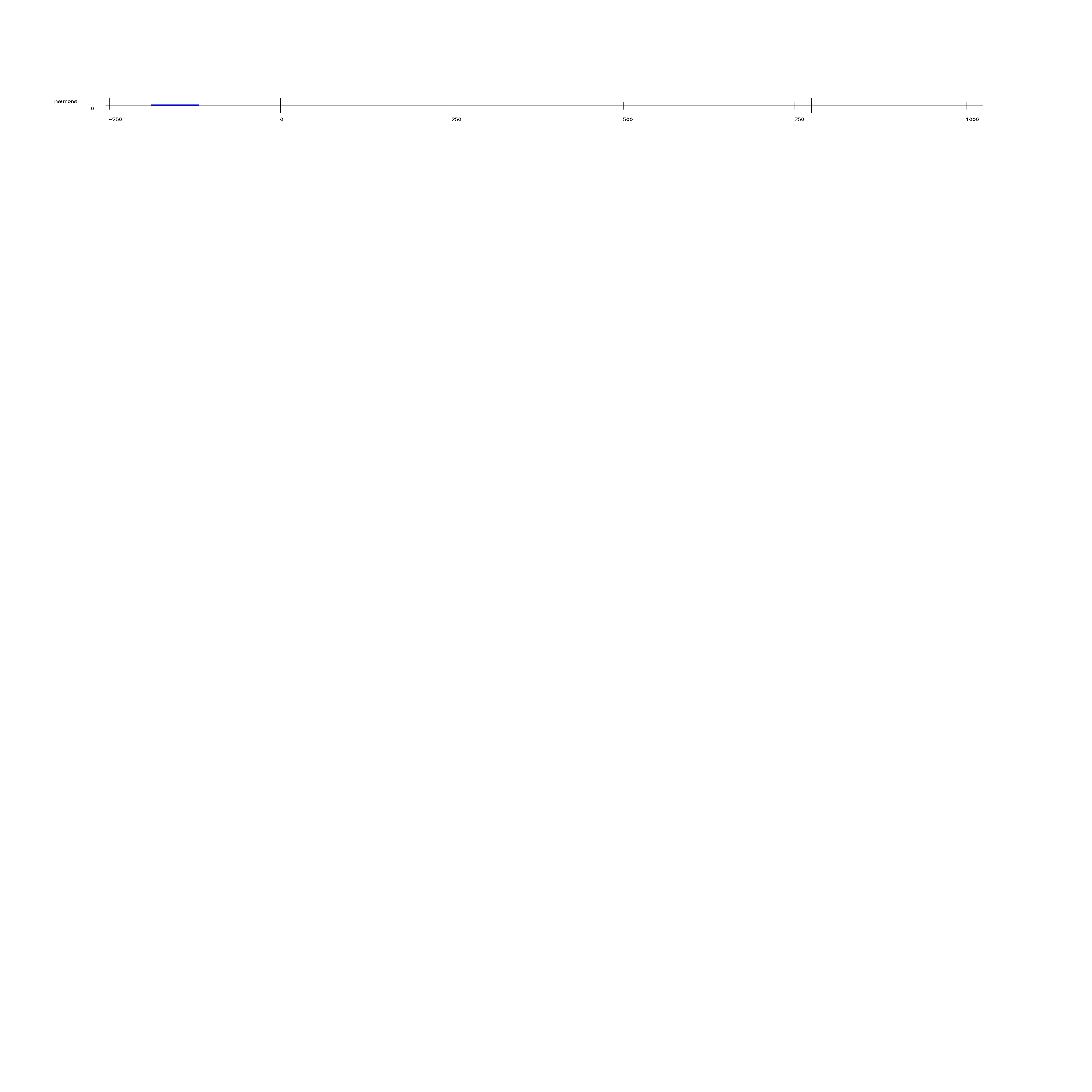

Supplement: Supplementary file 1 [file ijms-24-02970-s001.zip › Supplementary Data S2/2.3111494-3112267.png]

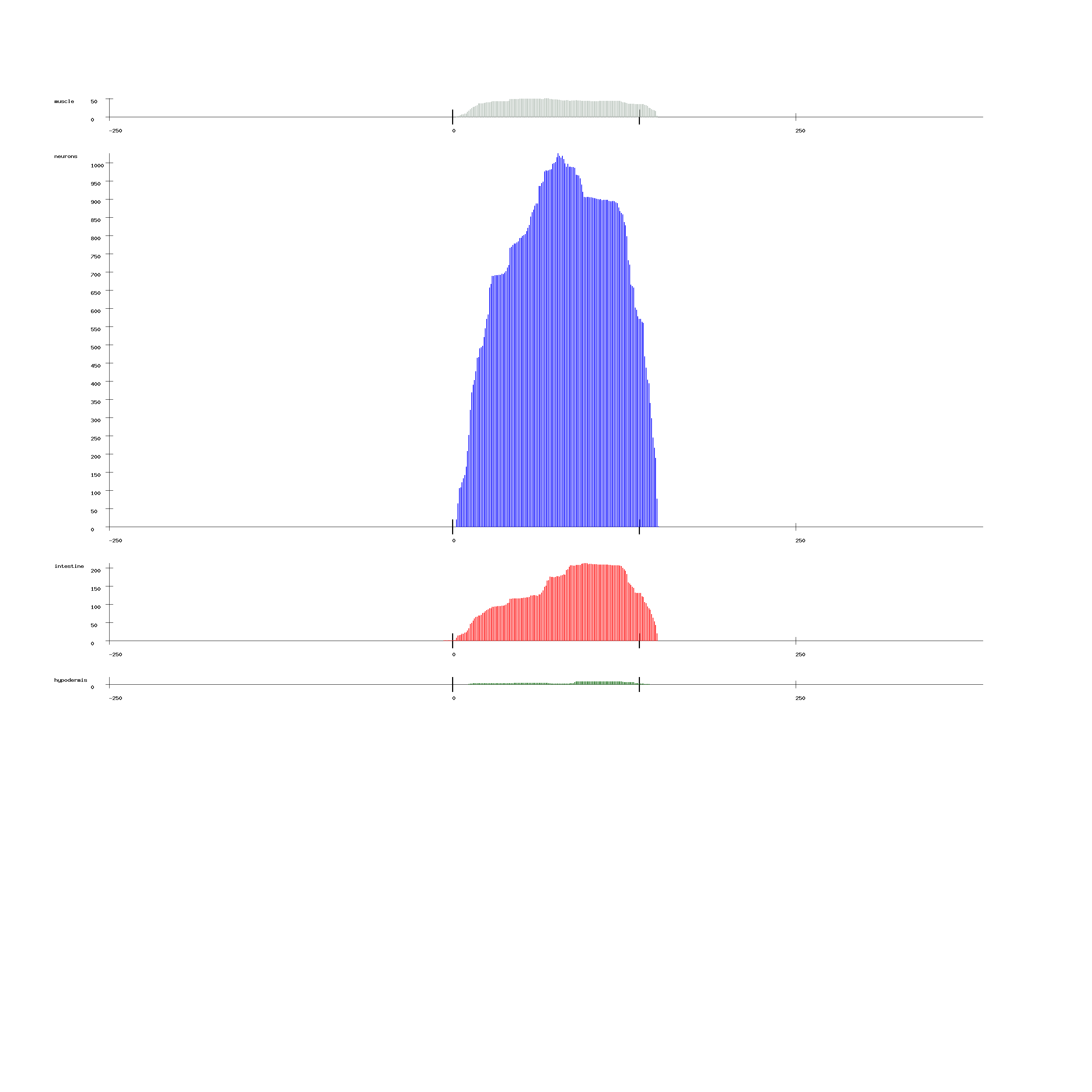

Supplement: Supplementary file 1 [file ijms-24-02970-s001.zip › Supplementary Data S2/2.3290648-3290783.png]

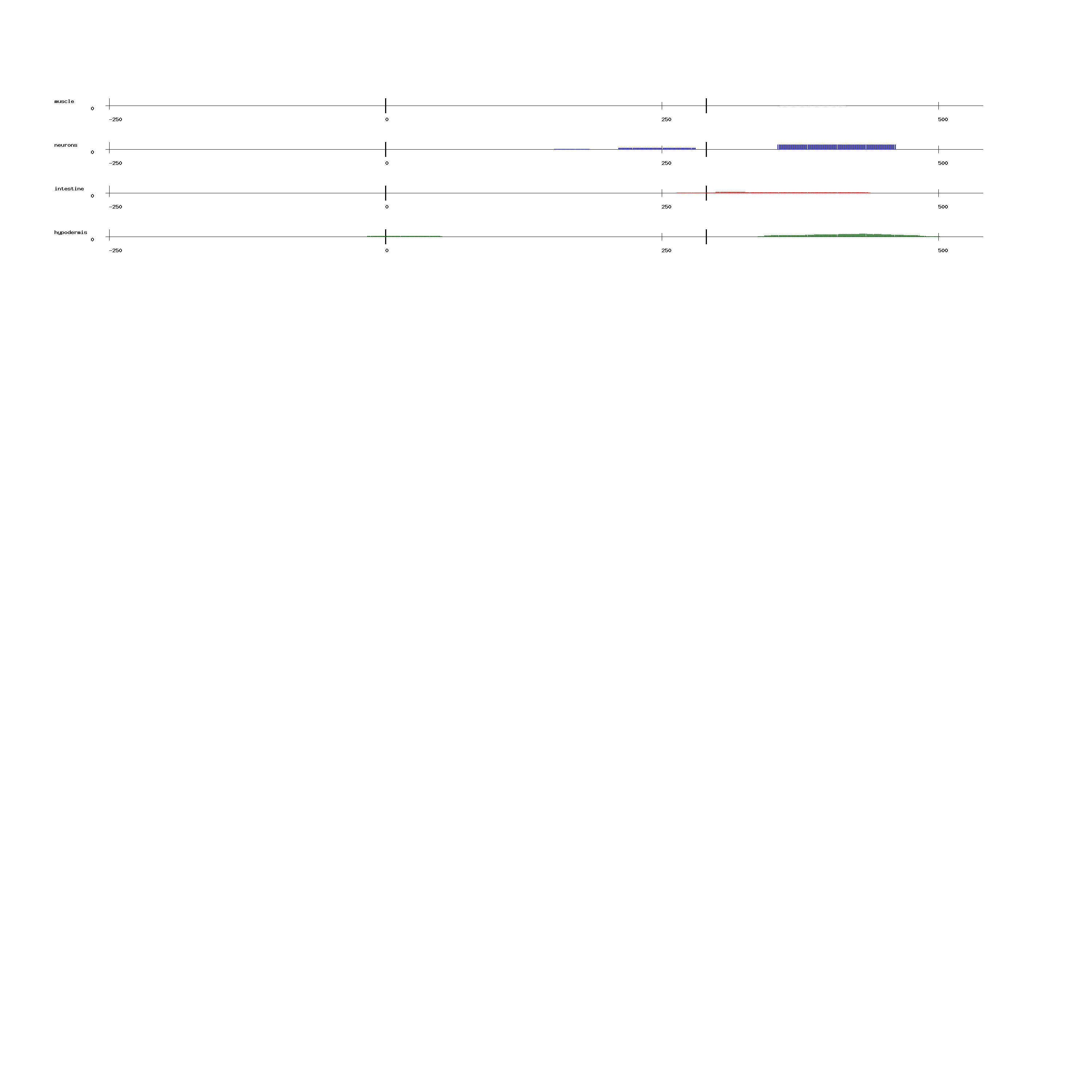

Supplement: Supplementary file 1 [file ijms-24-02970-s001.zip › Supplementary Data S2/2.3937888-3938177.png]

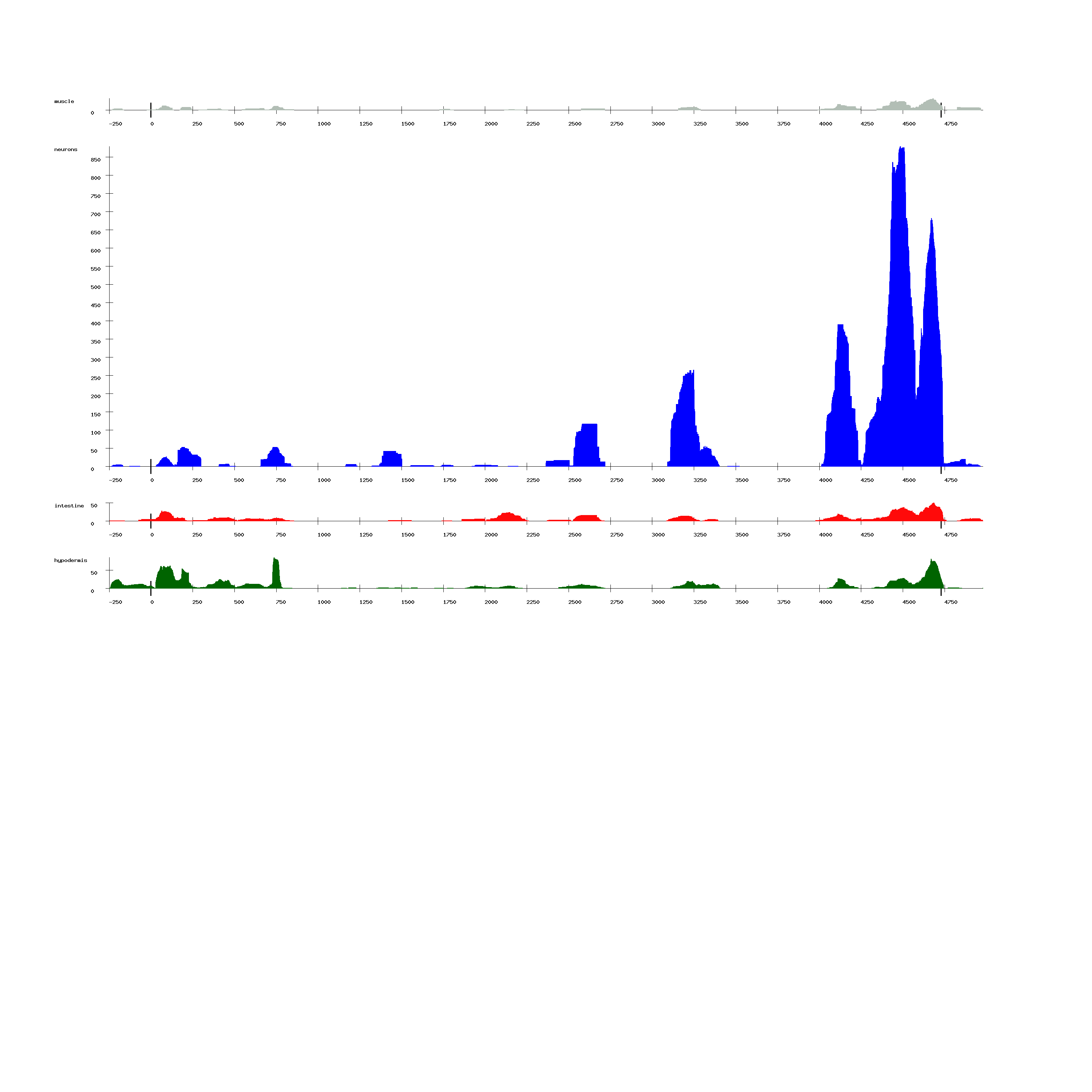

Supplement: Supplementary file 1 [file ijms-24-02970-s001.zip › Supplementary Data S2/2.3941820-3946547.png]

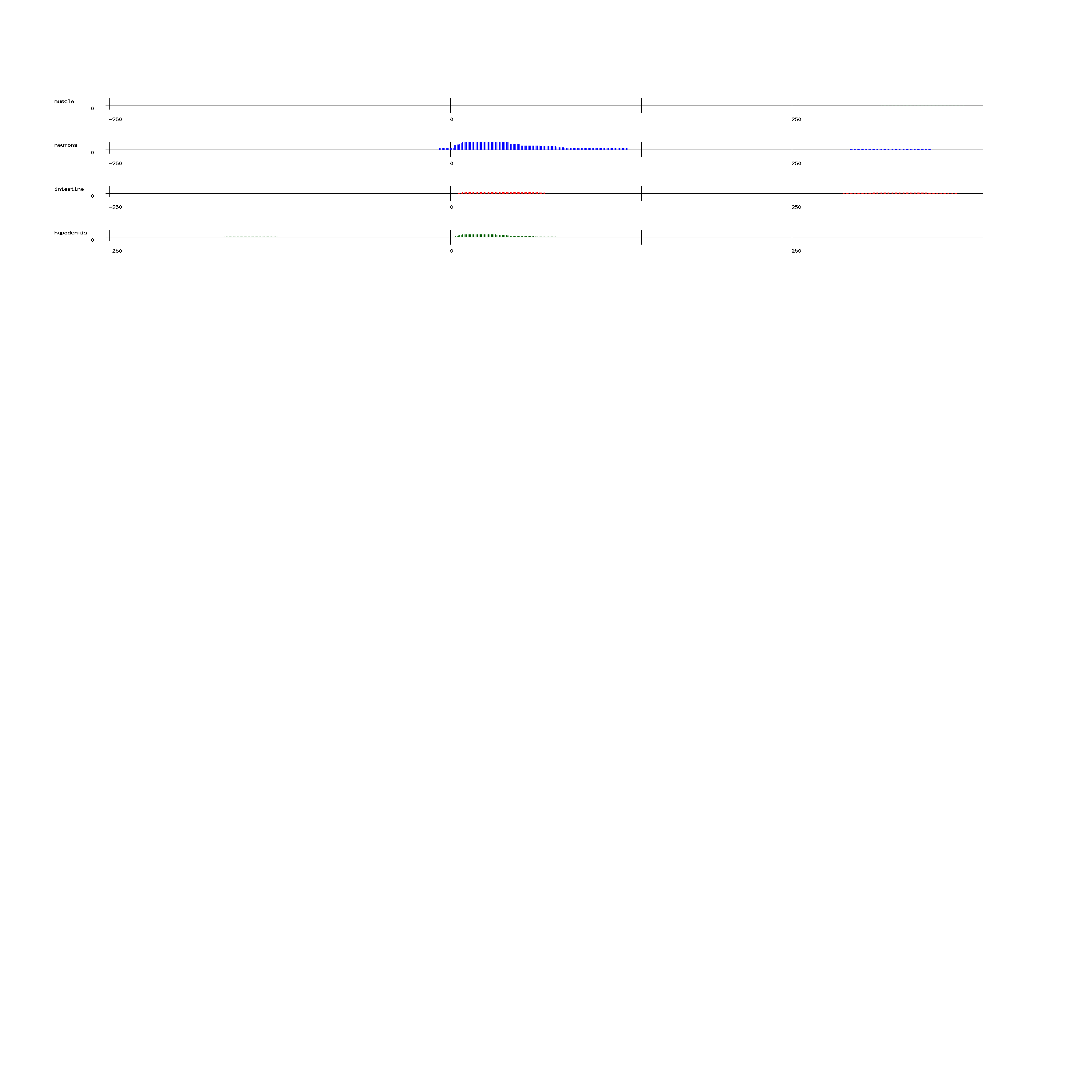

Supplement: Supplementary file 1 [file ijms-24-02970-s001.zip › Supplementary Data S2/2.3994649-3994788.png]

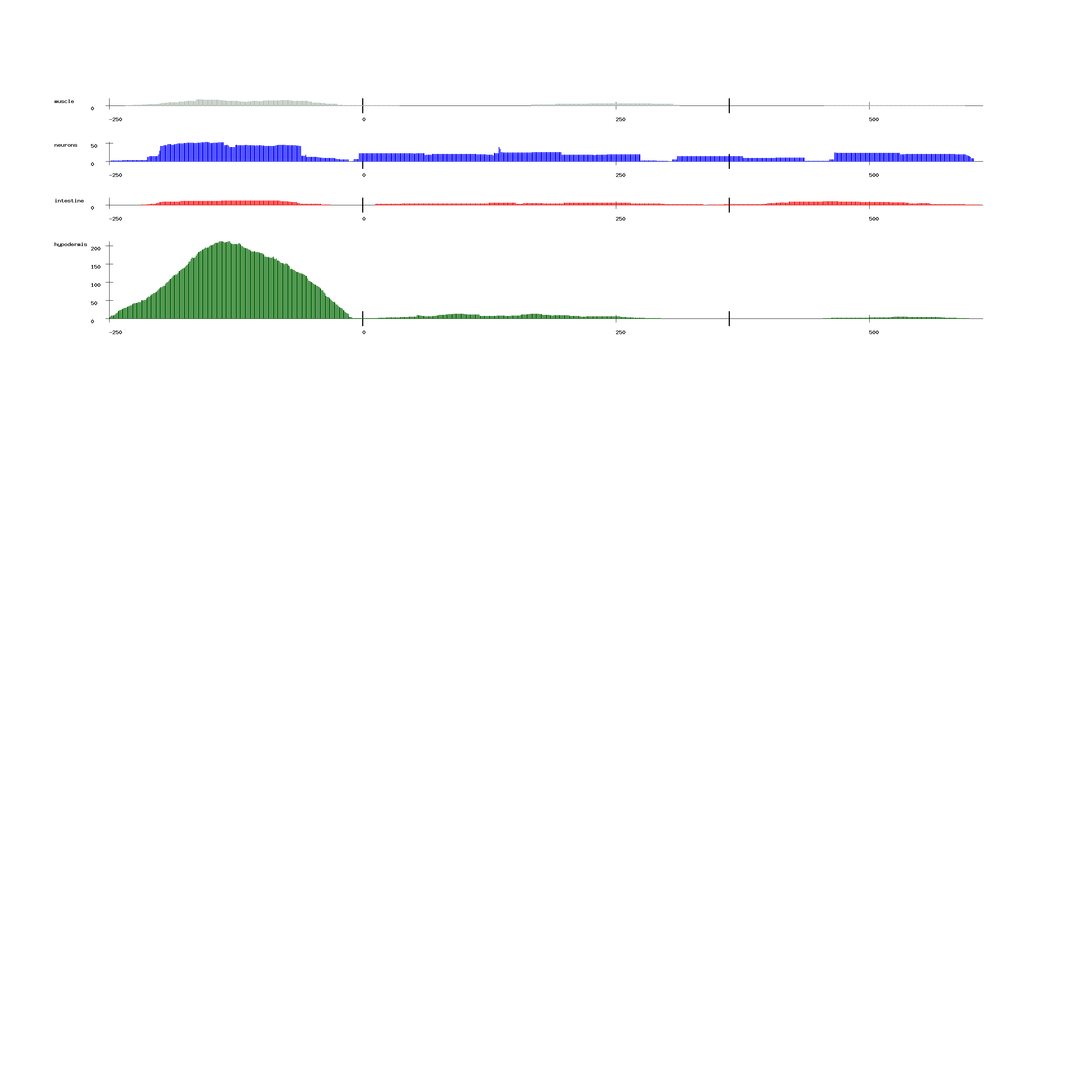

Supplement: Supplementary file 1 [file ijms-24-02970-s001.zip › Supplementary Data S2/2.4054458-4054819.png]

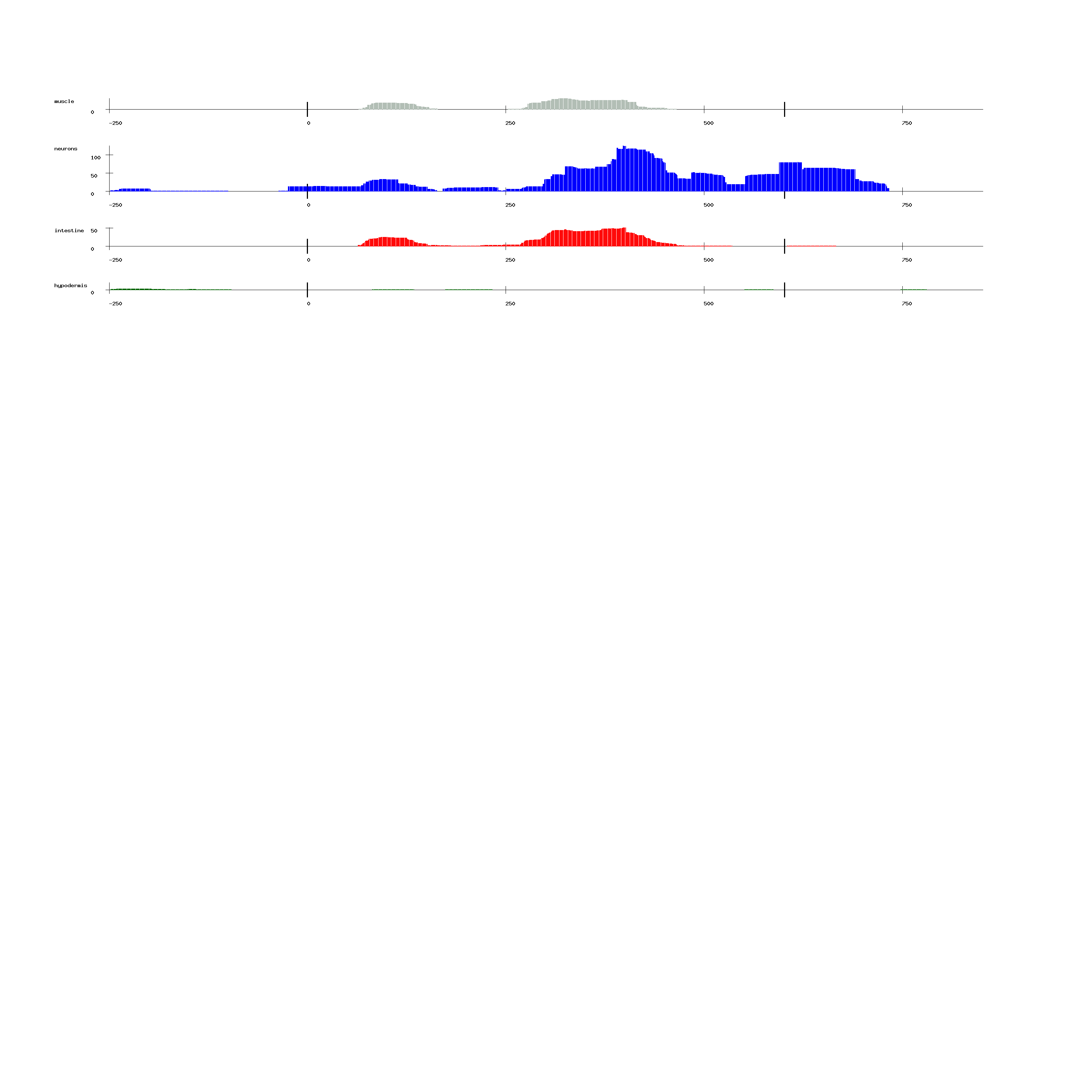

Supplement: Supplementary file 1 [file ijms-24-02970-s001.zip › Supplementary Data S2/2.4259952-4260552.png]

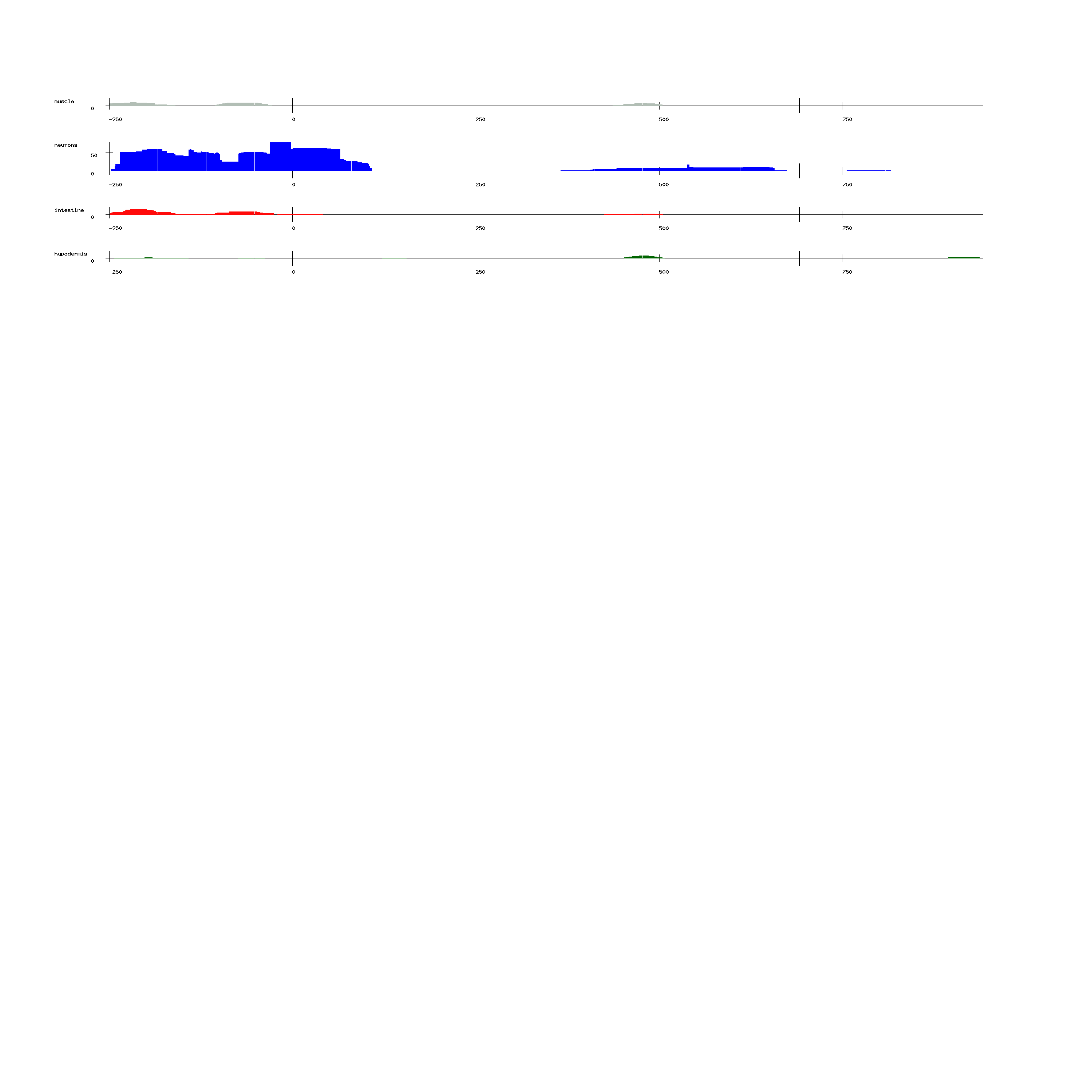

Supplement: Supplementary file 1 [file ijms-24-02970-s001.zip › Supplementary Data S2/2.4260577-4261267.png]

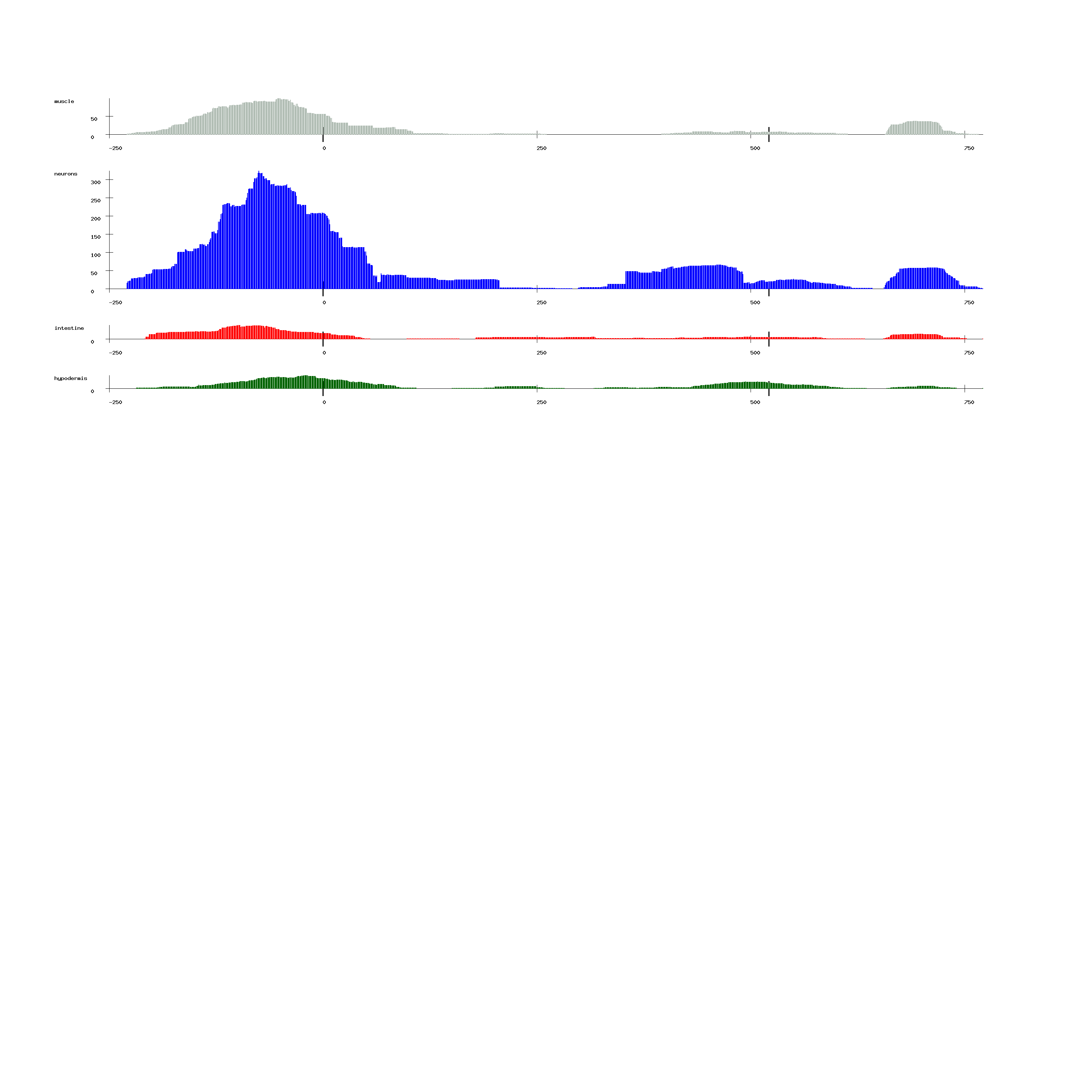

Supplement: Supplementary file 1 [file ijms-24-02970-s001.zip › Supplementary Data S2/2.4554394-4554914.png]

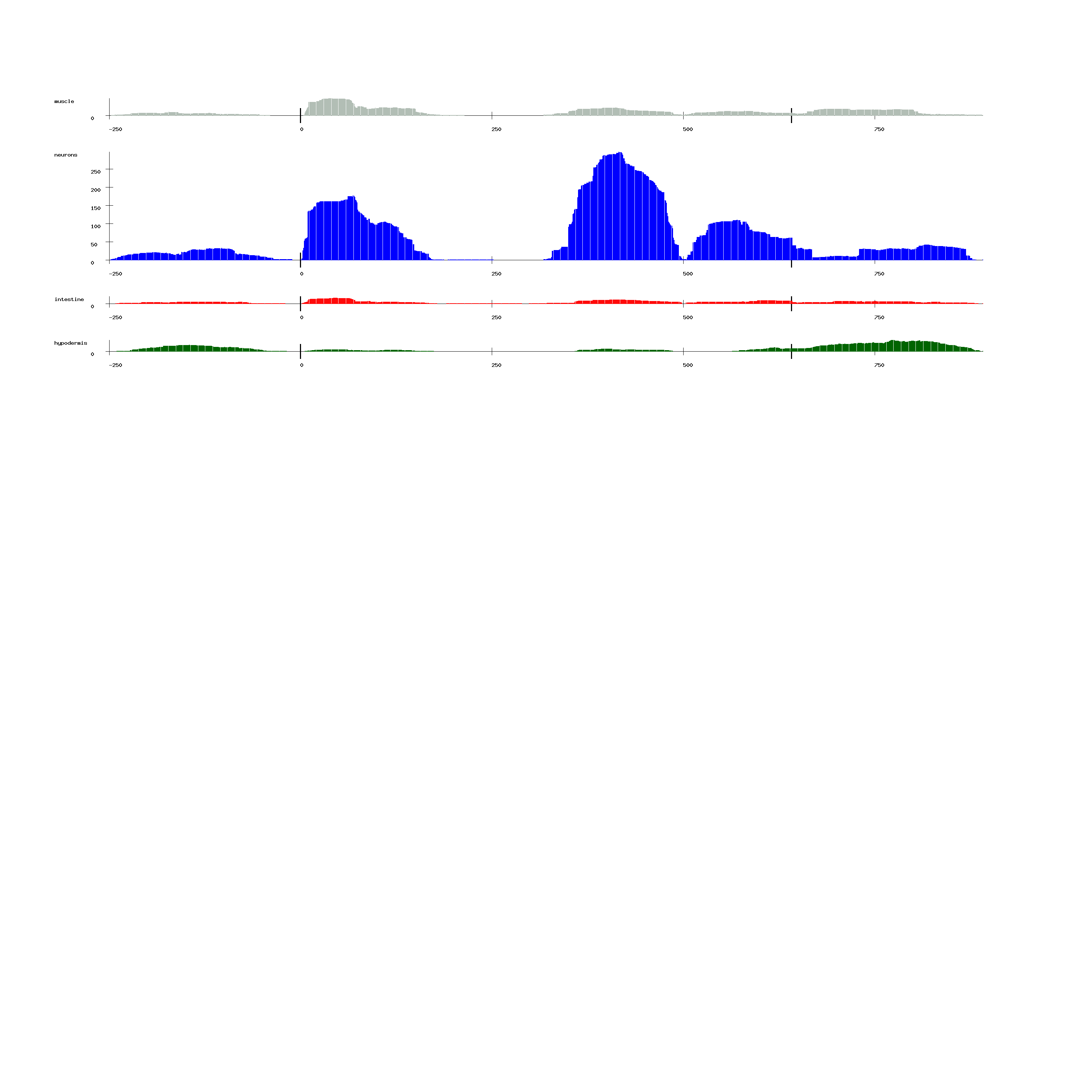

Supplement: Supplementary file 1 [file ijms-24-02970-s001.zip › Supplementary Data S2/2.4555047-4555687.png]

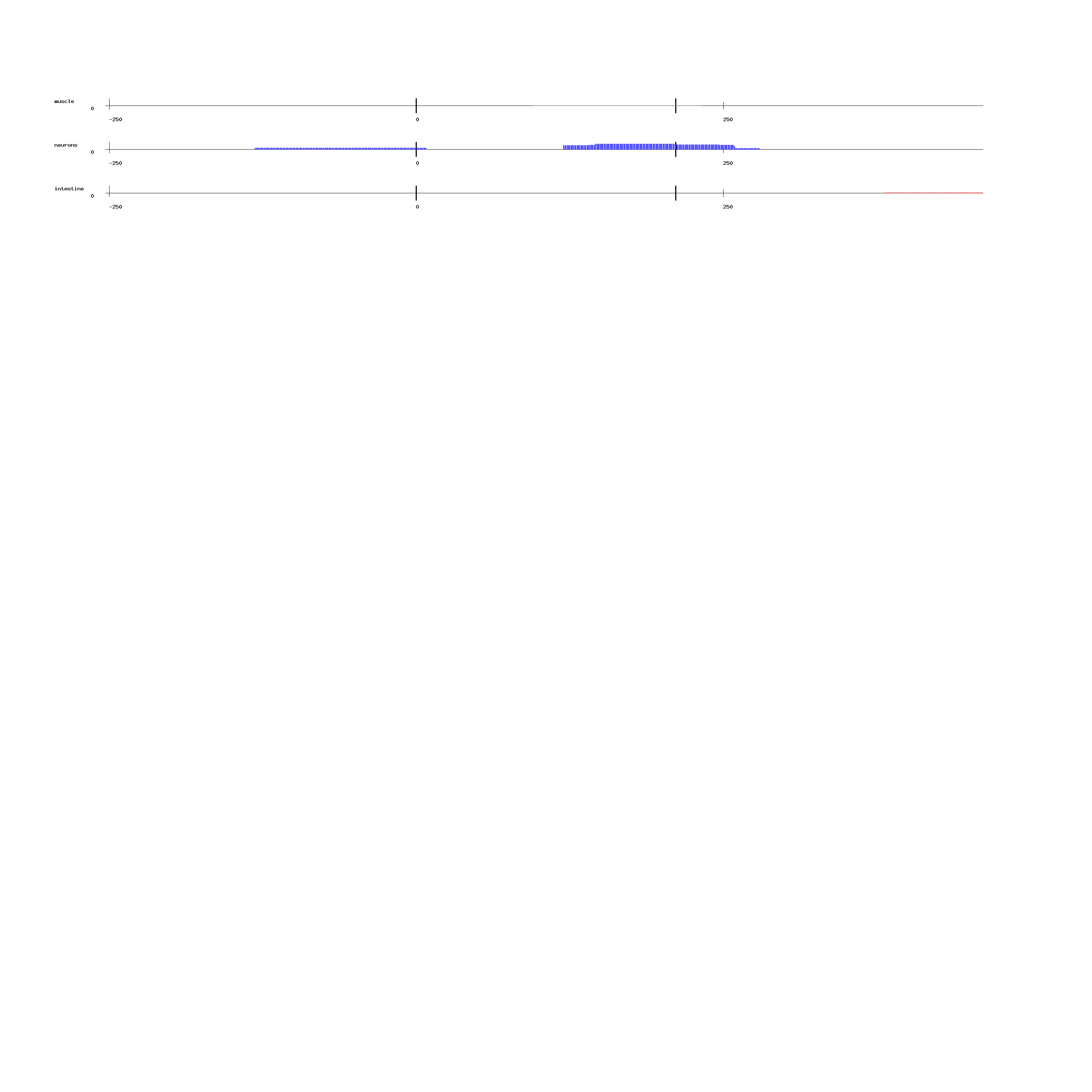

Supplement: Supplementary file 1 [file ijms-24-02970-s001.zip › Supplementary Data S2/2.4569011-4569221.png]

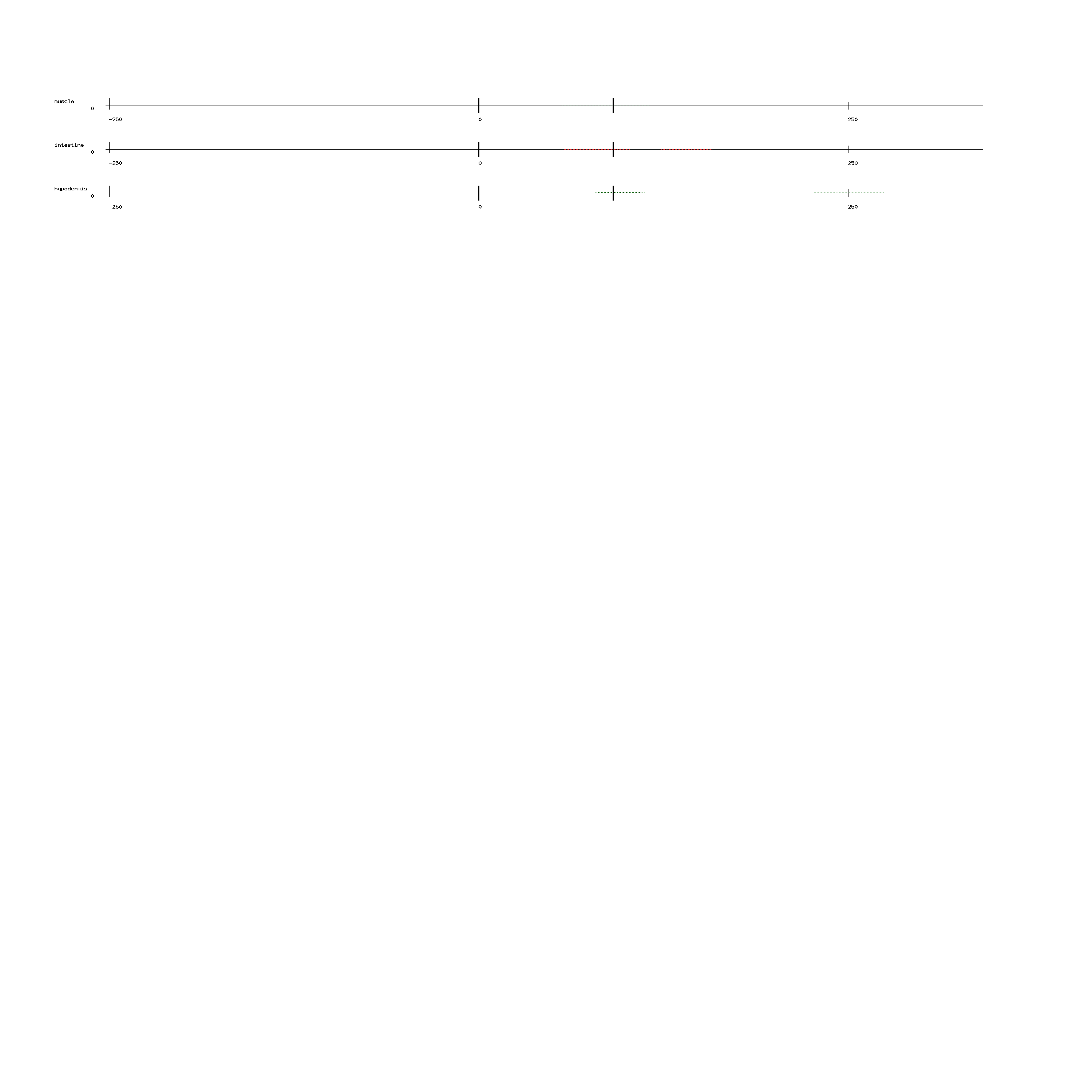

Supplement: Supplementary file 1 [file ijms-24-02970-s001.zip › Supplementary Data S2/2.4684299-4684389.png]

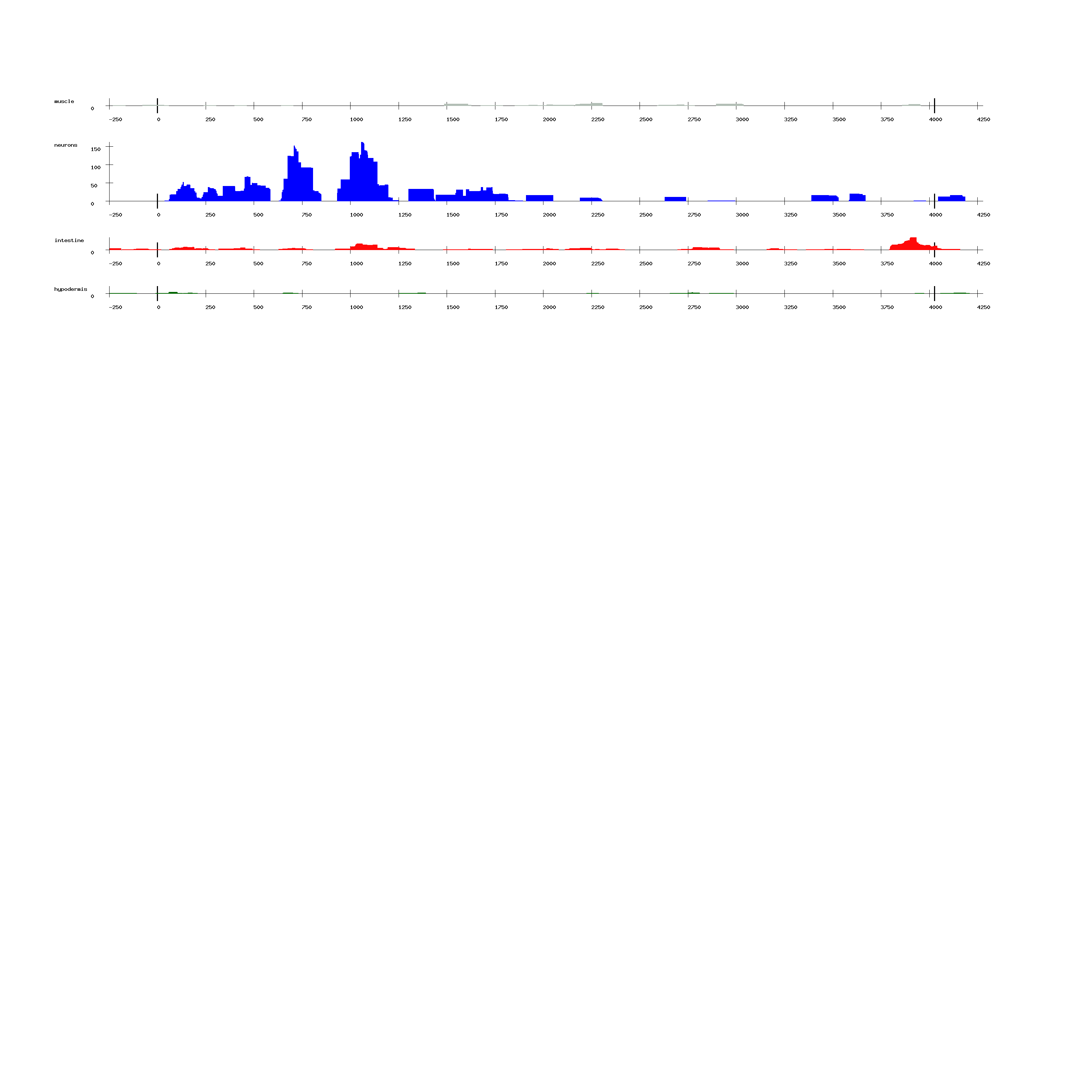

Supplement: Supplementary file 1 [file ijms-24-02970-s001.zip › Supplementary Data S2/2.5073308-5077334.png]

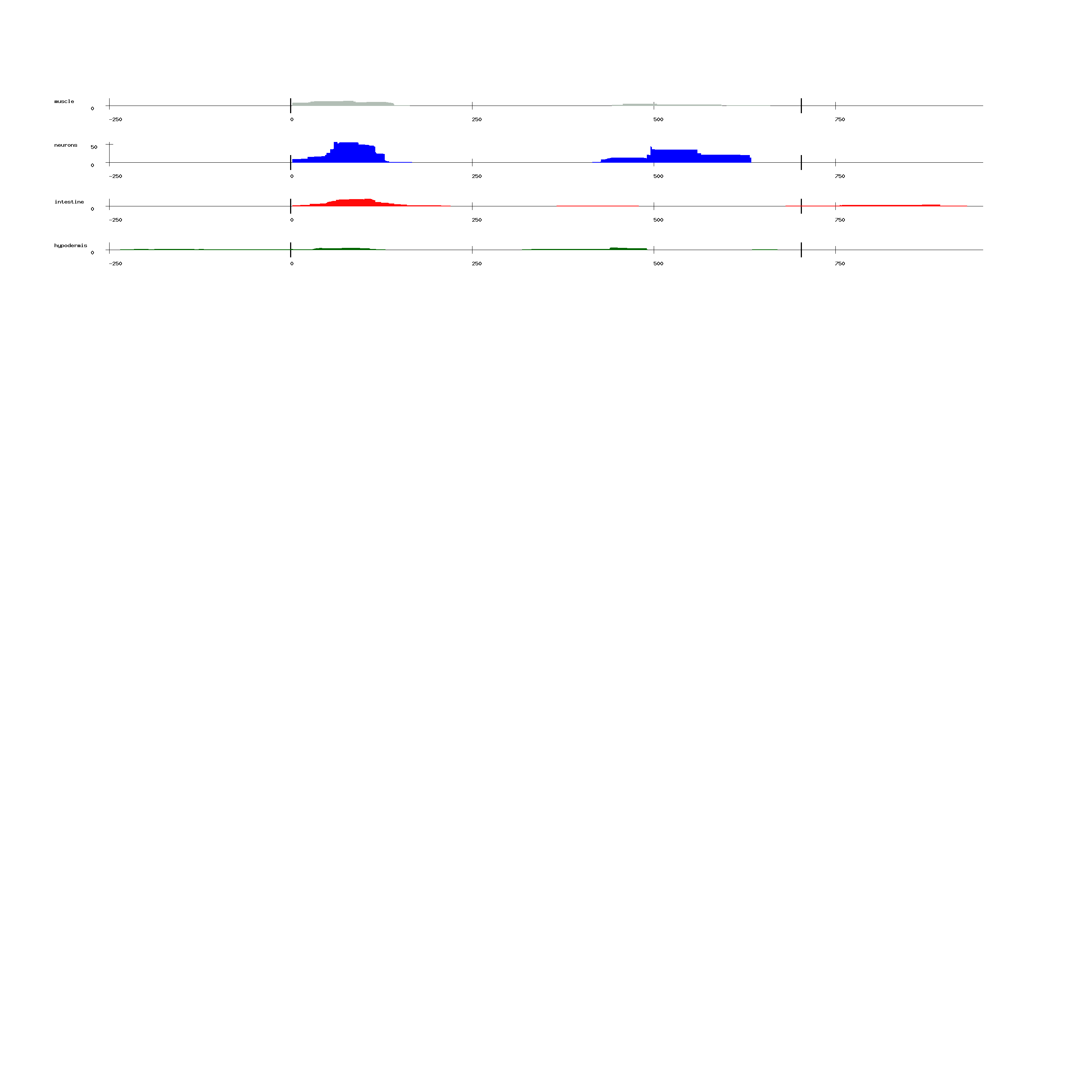

Supplement: Supplementary file 1 [file ijms-24-02970-s001.zip › Supplementary Data S2/2.5097197-5097899.png]

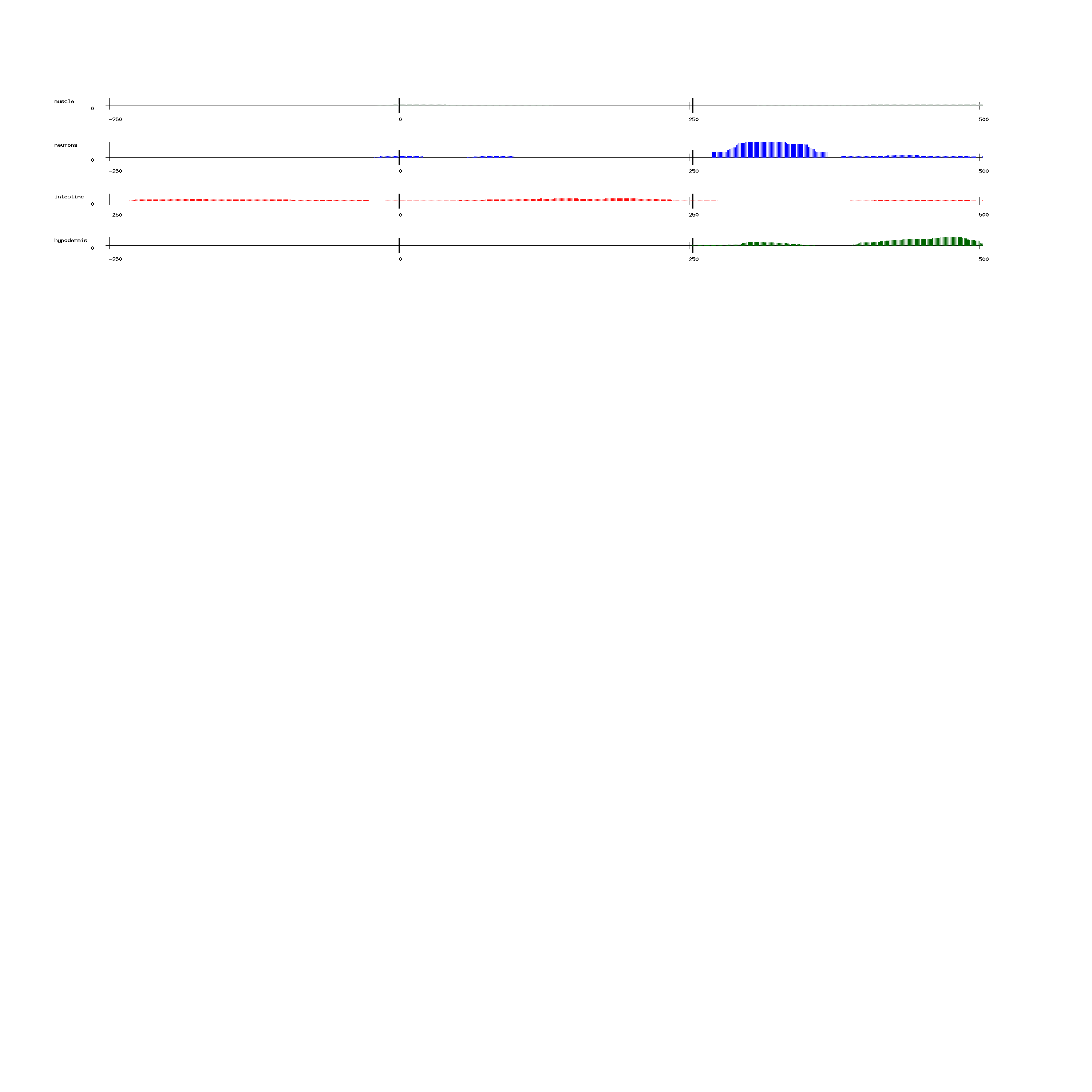

Supplement: Supplementary file 1 [file ijms-24-02970-s001.zip › Supplementary Data S2/2.5144313-5144565.png]

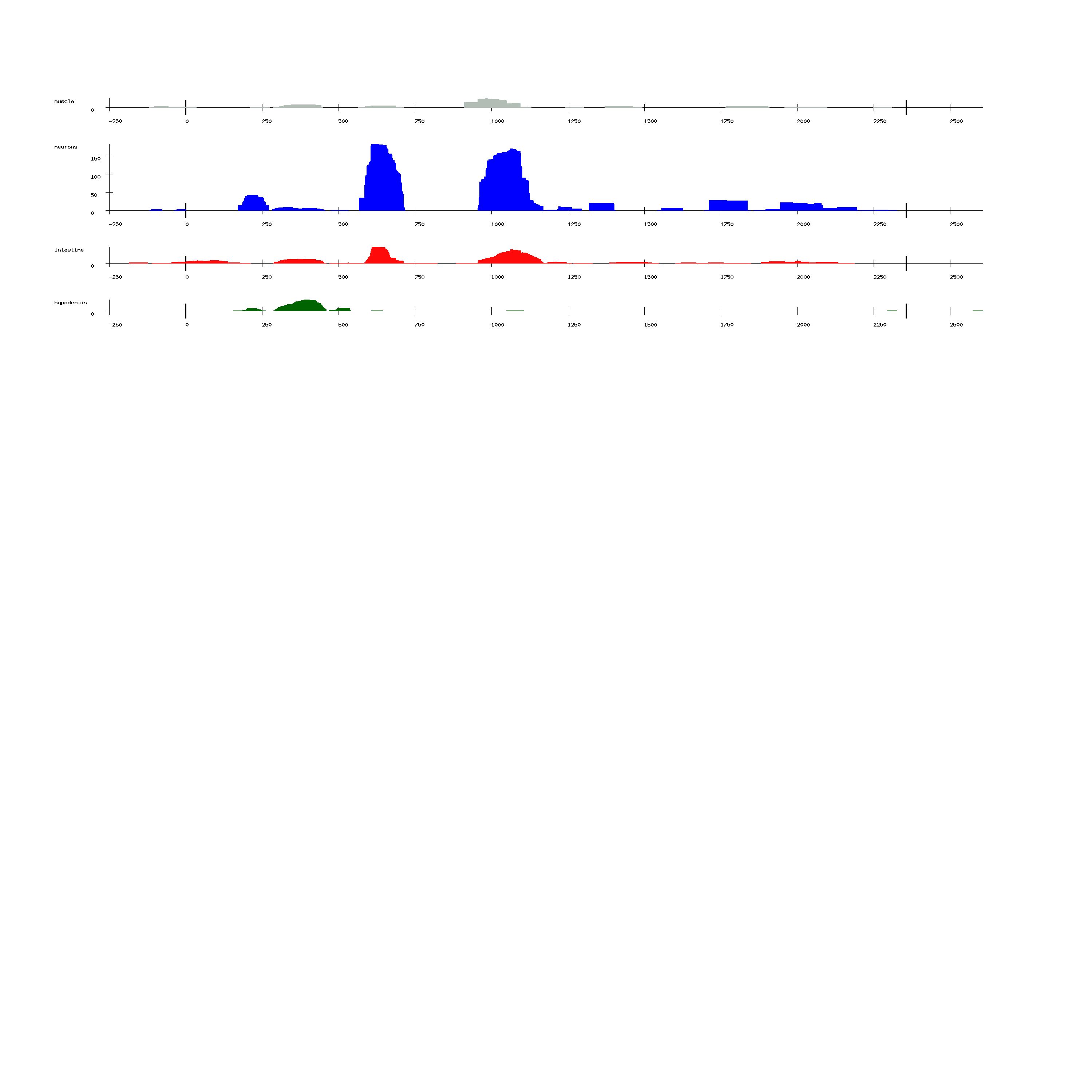

Supplement: Supplementary file 1 [file ijms-24-02970-s001.zip › Supplementary Data S2/2.5144410-5146766.png]

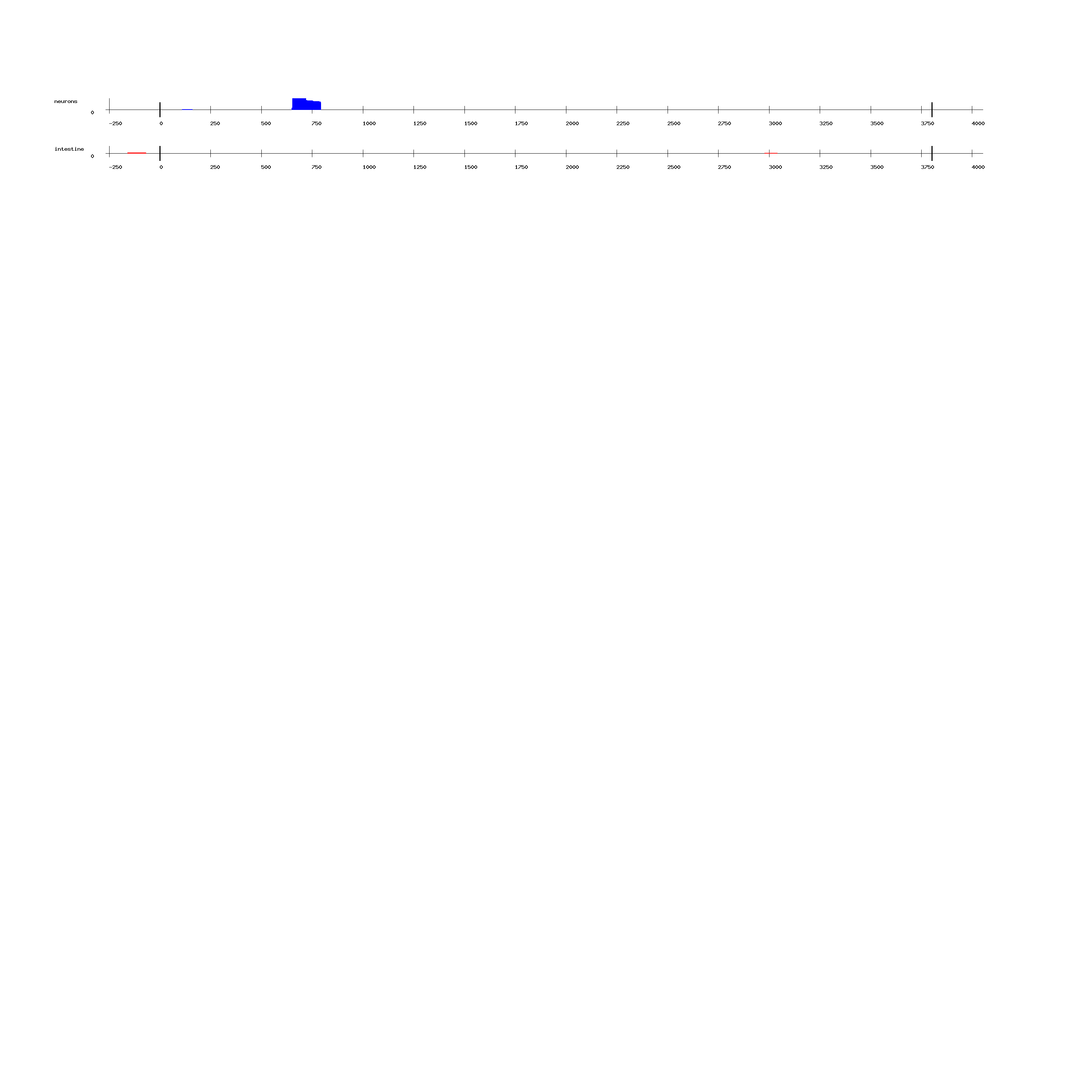

Supplement: Supplementary file 1 [file ijms-24-02970-s001.zip › Supplementary Data S2/2.6073626-6077427.png]

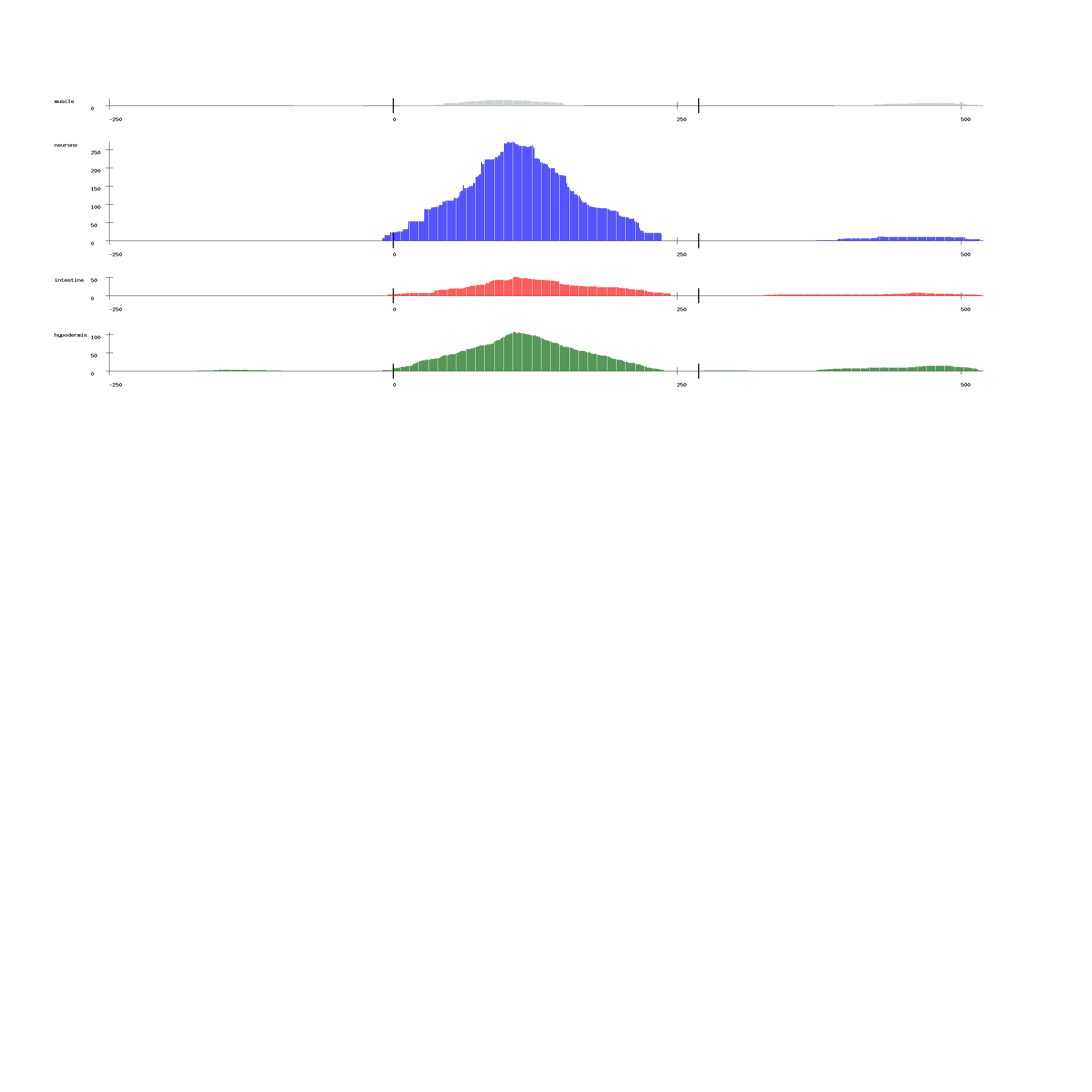

Supplement: Supplementary file 1 [file ijms-24-02970-s001.zip › Supplementary Data S2/2.6082023-6082291.png]

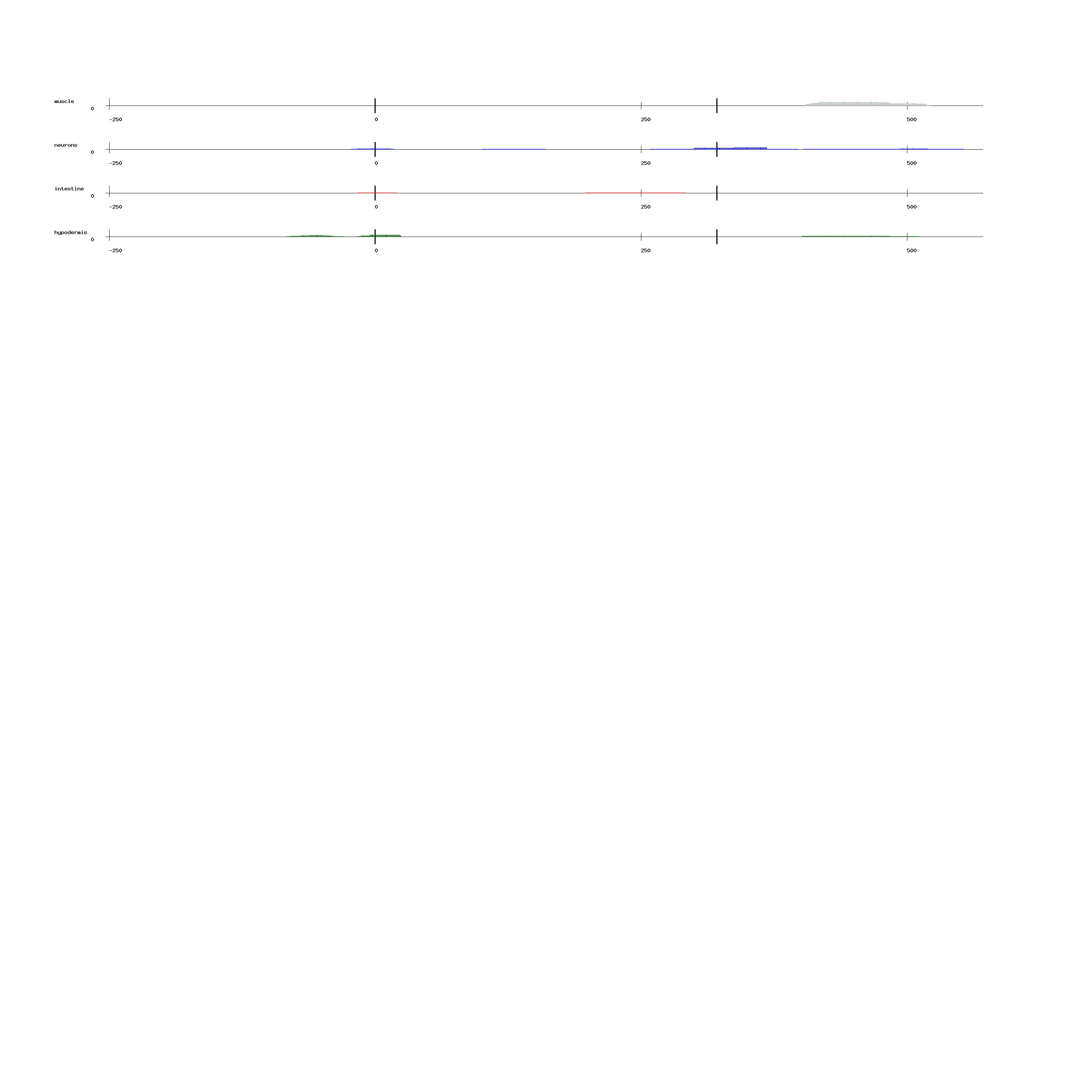

Supplement: Supplementary file 1 [file ijms-24-02970-s001.zip › Supplementary Data S2/2.632493-632813.png]

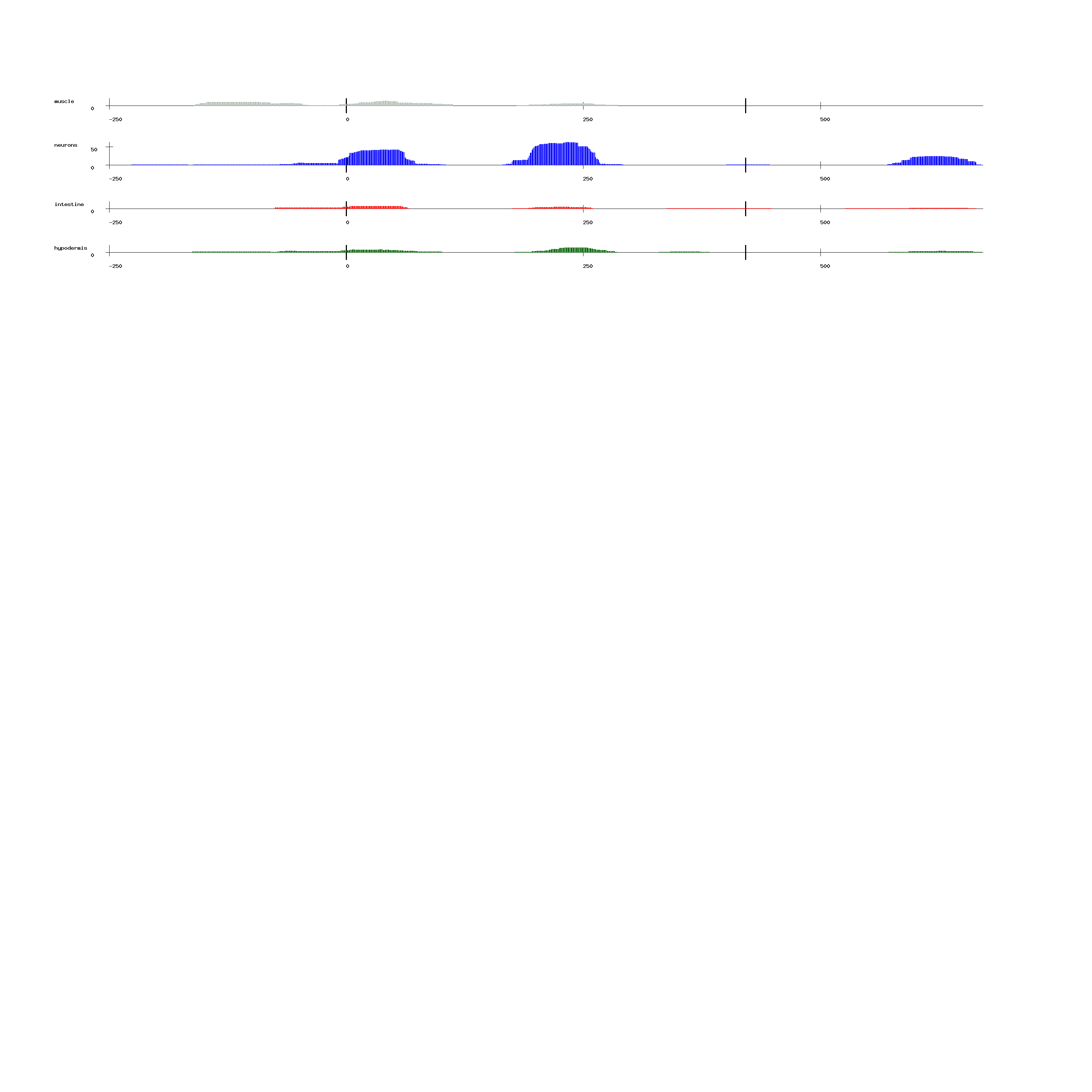

Supplement: Supplementary file 1 [file ijms-24-02970-s001.zip › Supplementary Data S2/2.633057-633477.png]

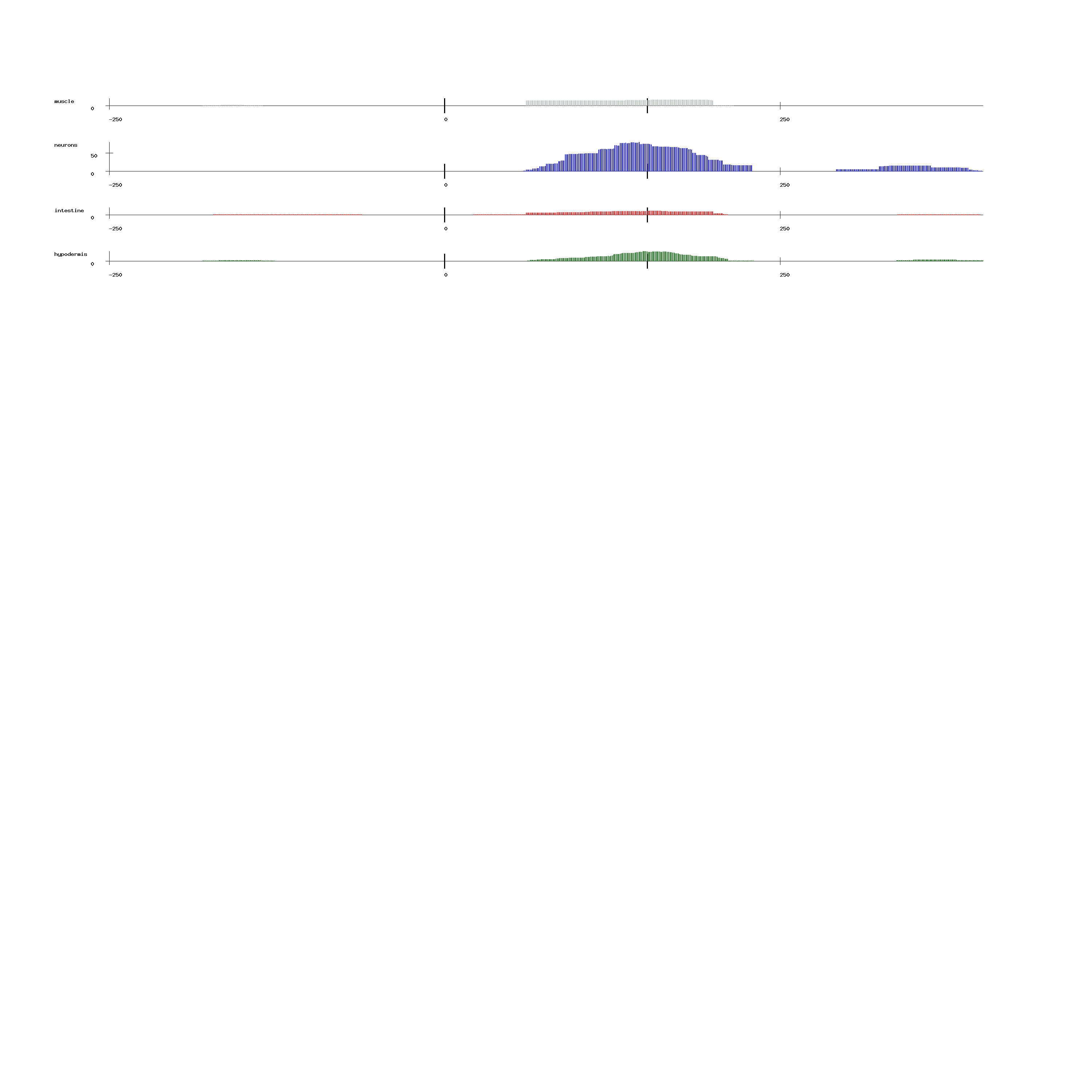

Supplement: Supplementary file 1 [file ijms-24-02970-s001.zip › Supplementary Data S2/2.633567-633717.png]

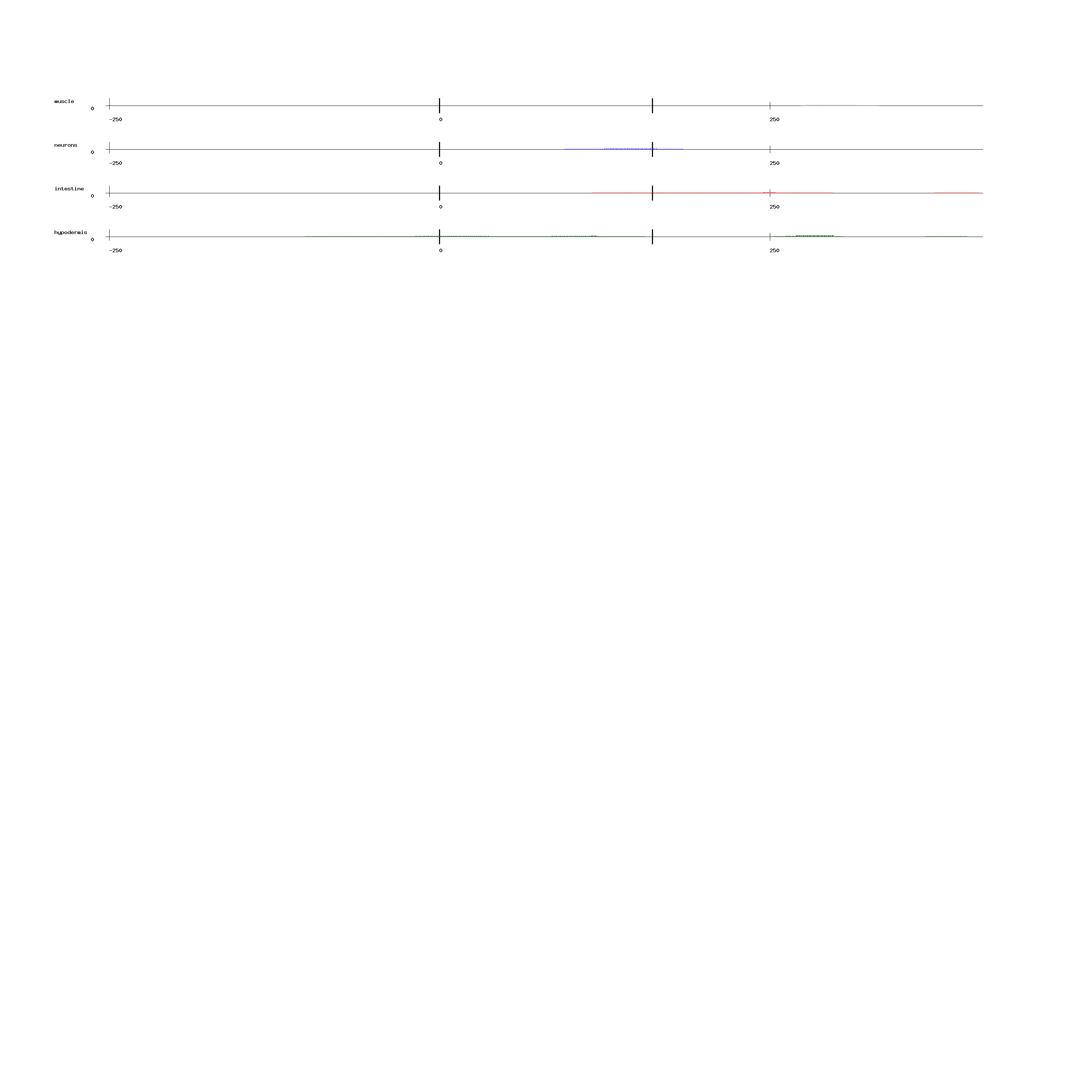

Supplement: Supplementary file 1 [file ijms-24-02970-s001.zip › Supplementary Data S2/2.6434384-6434544.png]

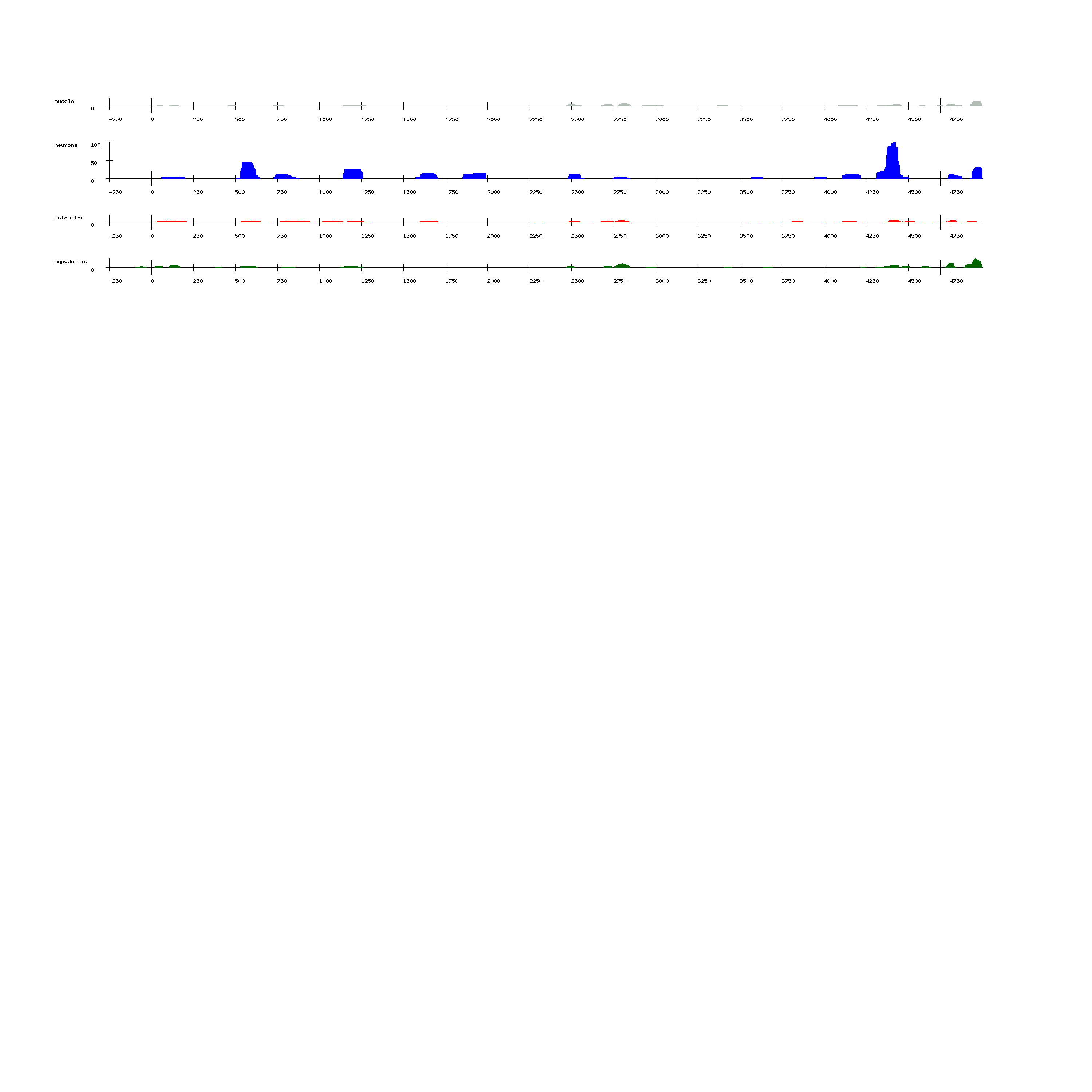

Supplement: Supplementary file 1 [file ijms-24-02970-s001.zip › Supplementary Data S2/2.694364-699056.png]

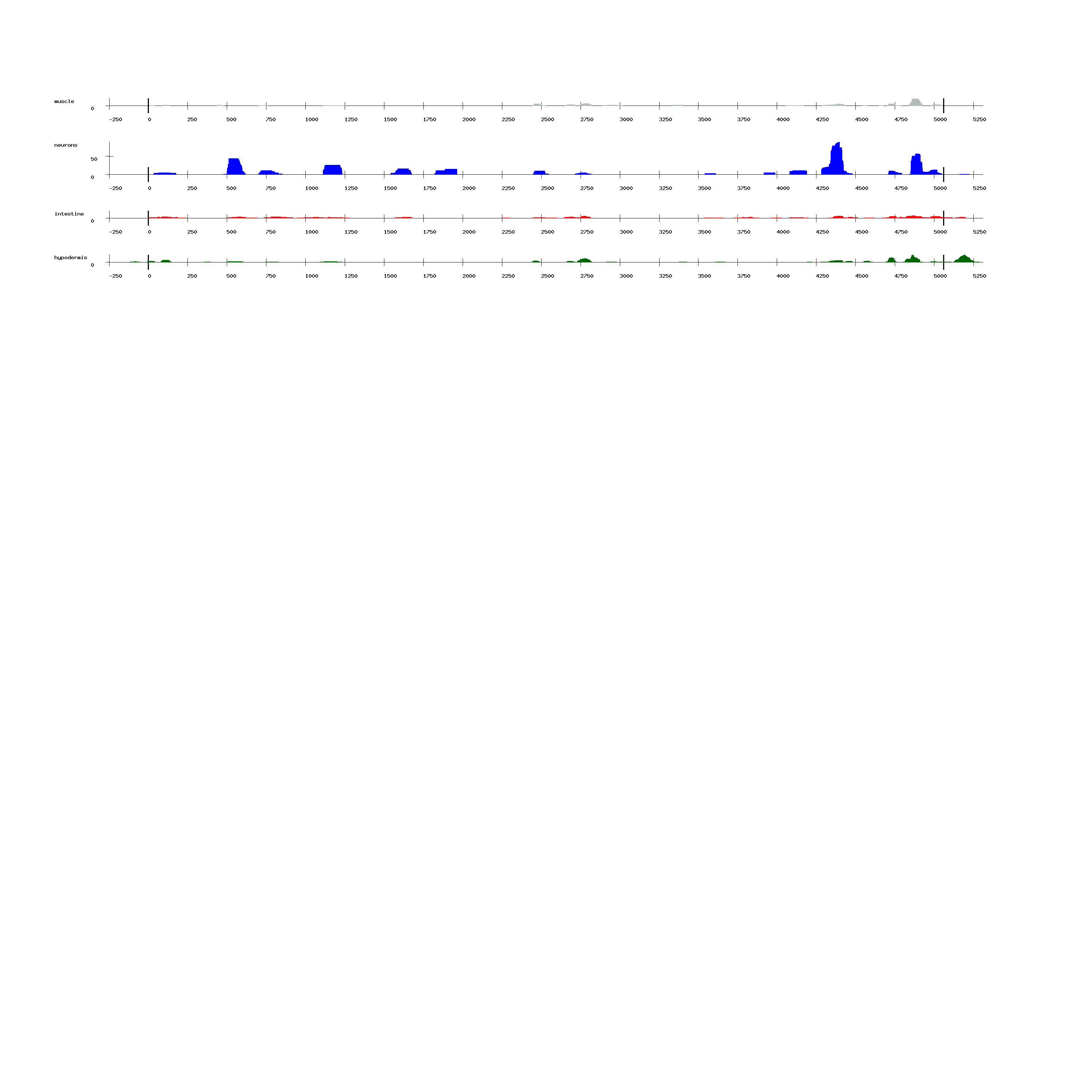

Supplement: Supplementary file 1 [file ijms-24-02970-s001.zip › Supplementary Data S2/2.694391-699449.png]

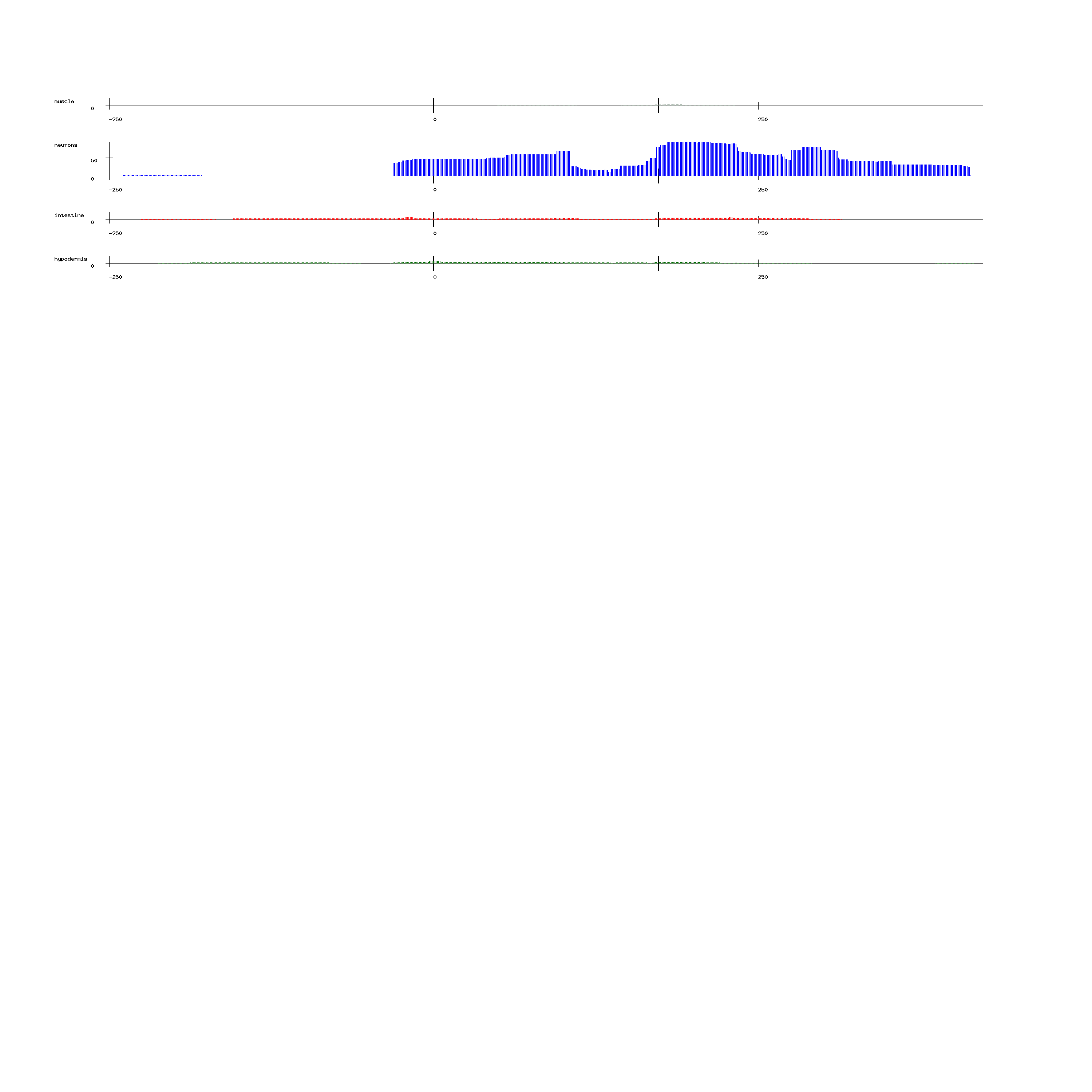

Supplement: Supplementary file 1 [file ijms-24-02970-s001.zip › Supplementary Data S2/2.7379726-7379898.png]

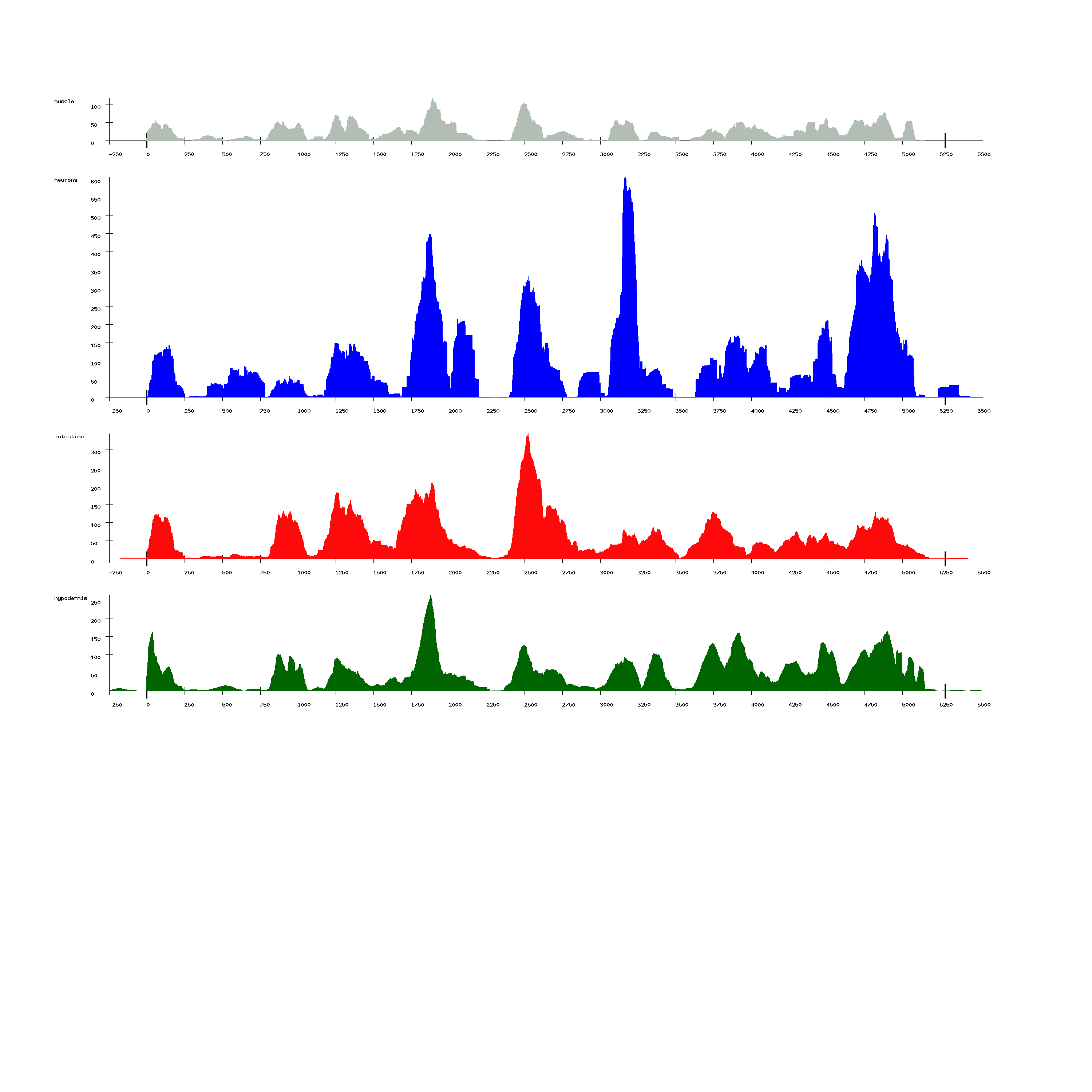

Supplement: Supplementary file 1 [file ijms-24-02970-s001.zip › Supplementary Data S2/2.8287652-8292933.png]

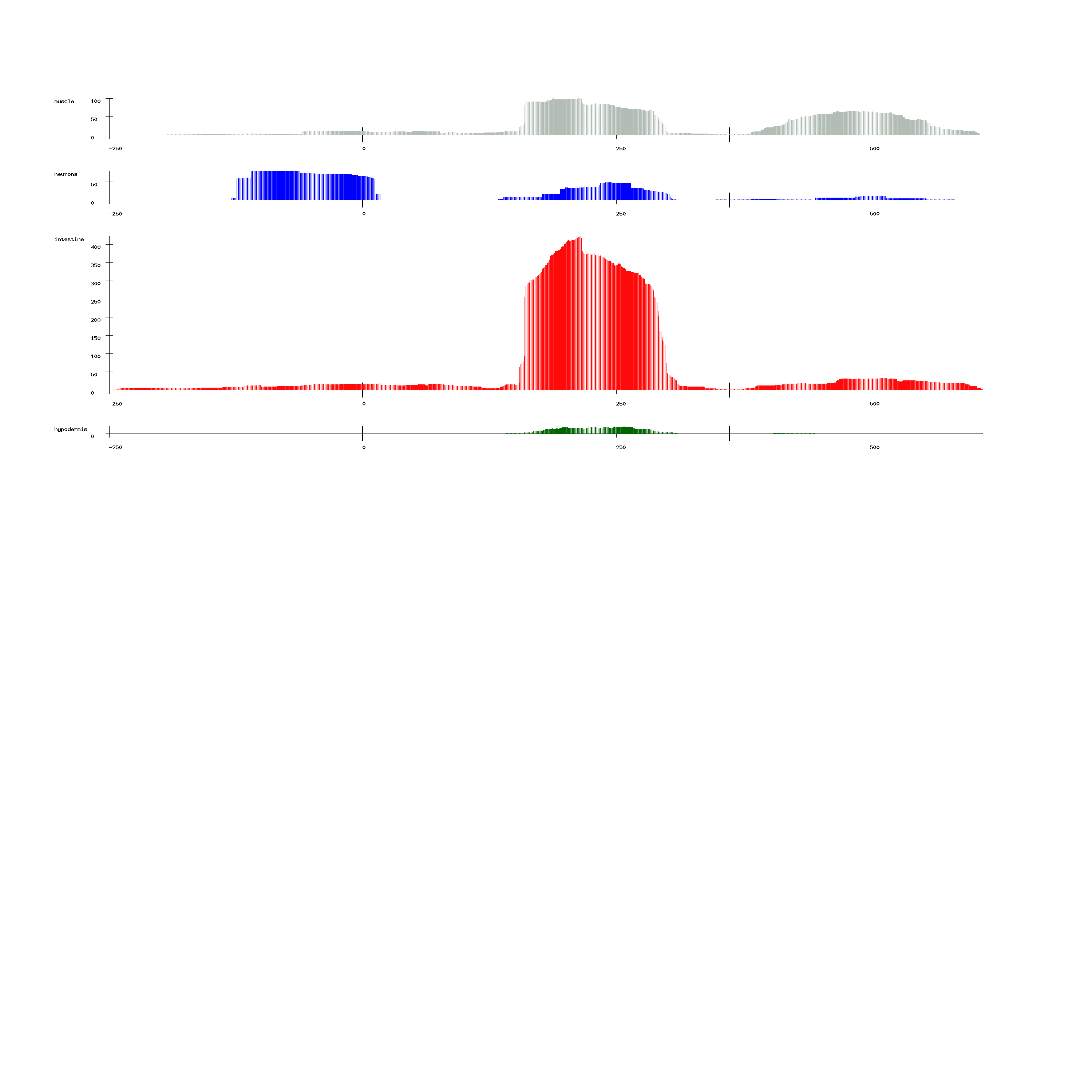

Supplement: Supplementary file 1 [file ijms-24-02970-s001.zip › Supplementary Data S2/2.851256-851616.png]

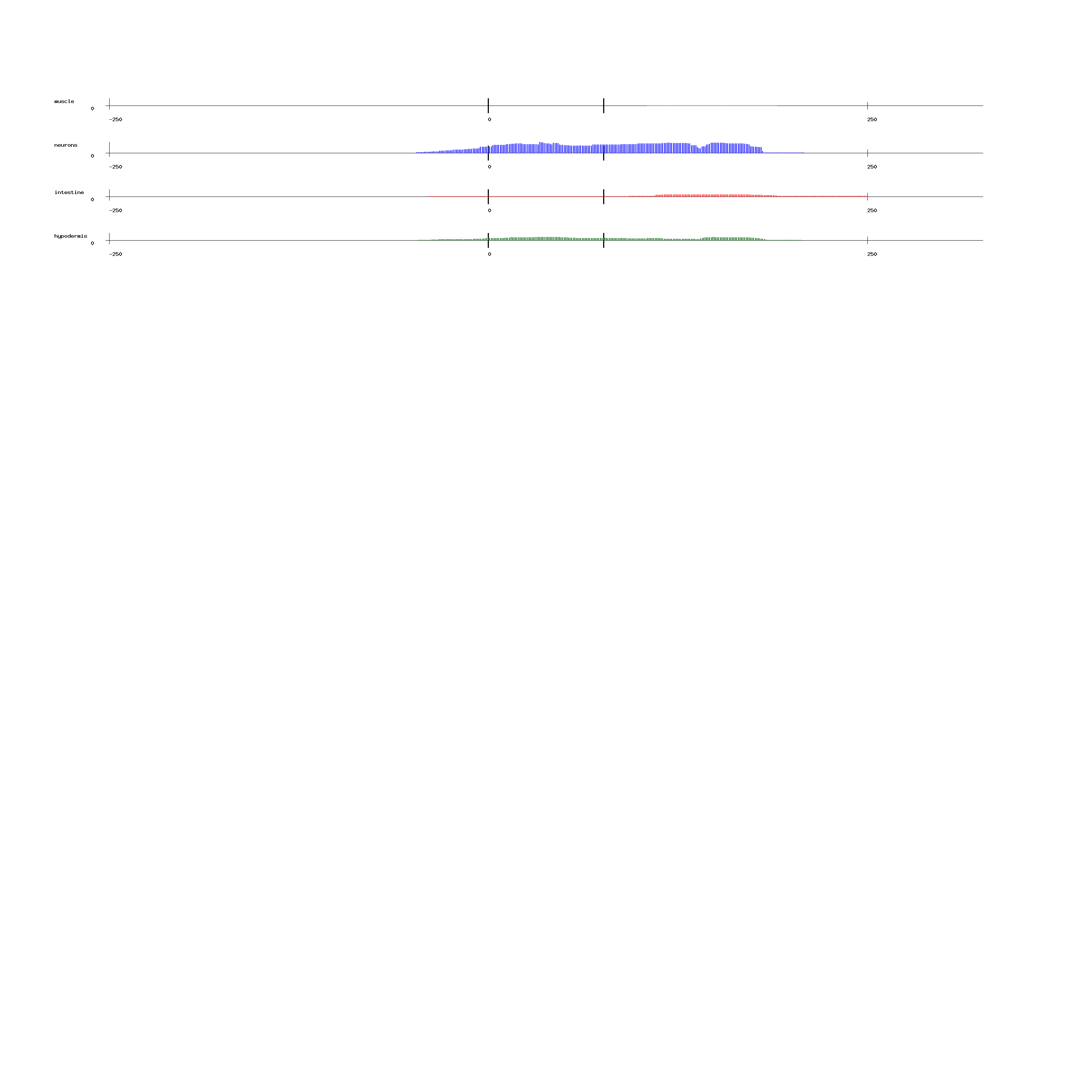

Supplement: Supplementary file 1 [file ijms-24-02970-s001.zip › Supplementary Data S2/2.857683-857758.png]

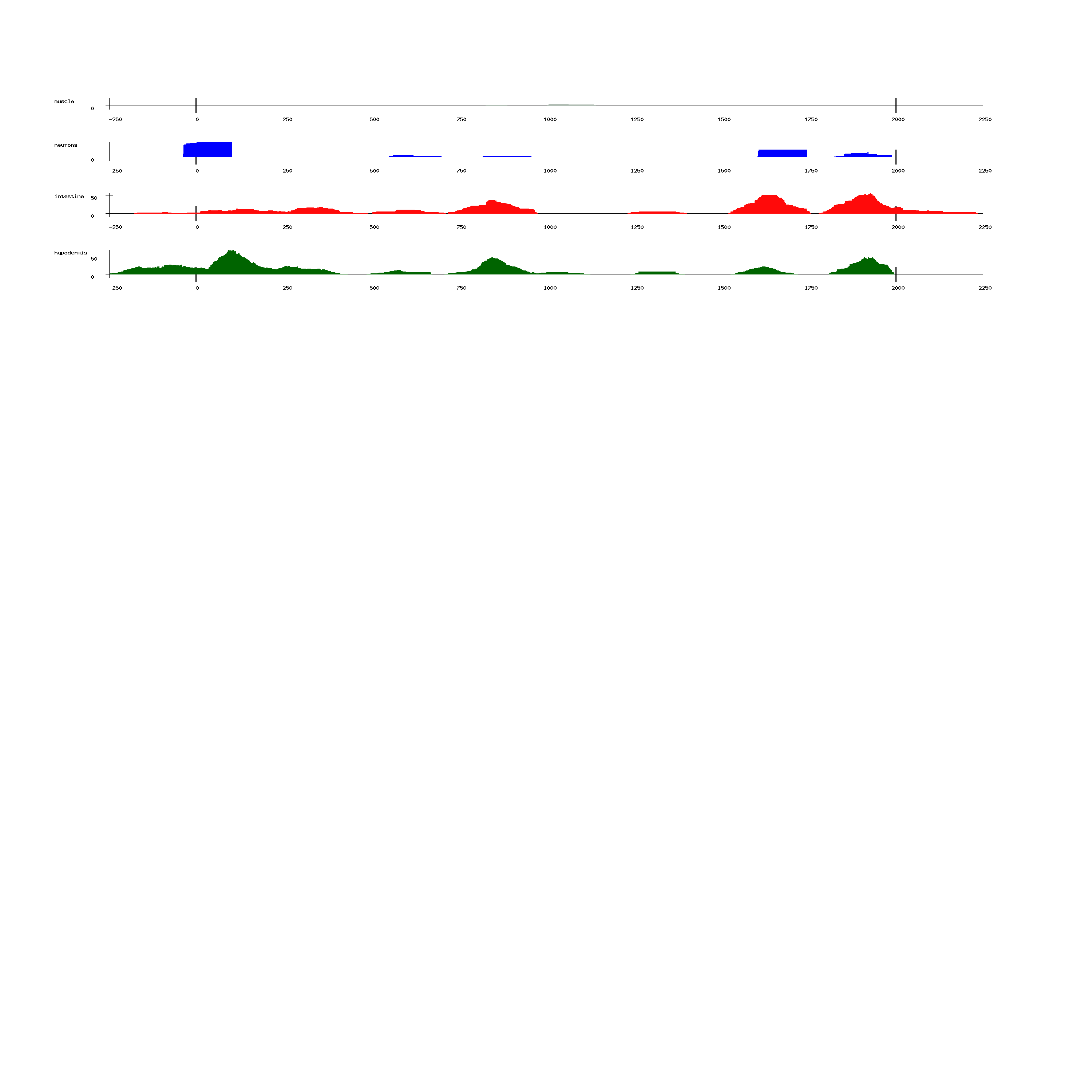

Supplement: Supplementary file 1 [file ijms-24-02970-s001.zip › Supplementary Data S2/2.8975477-8977487.png]

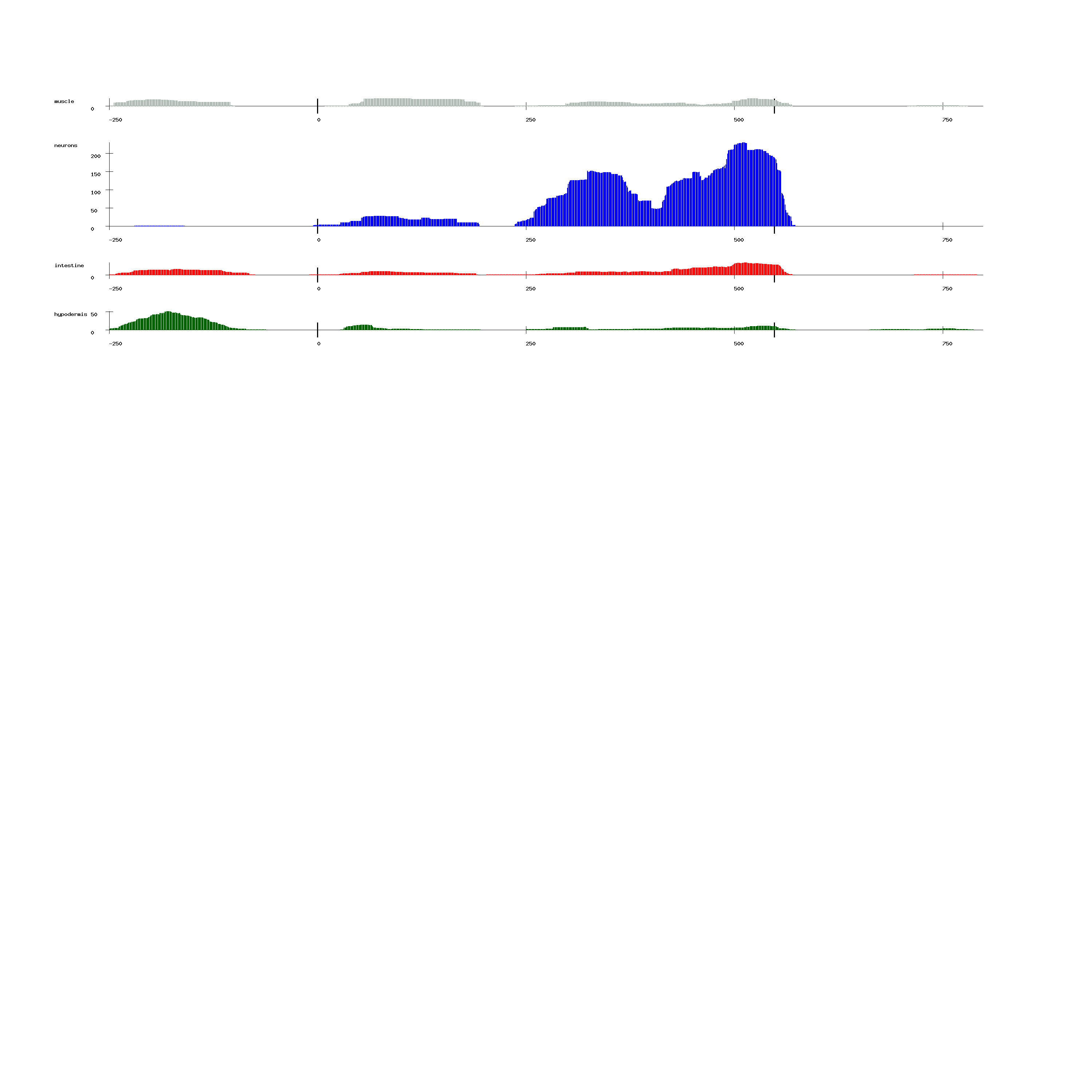

Supplement: Supplementary file 1 [file ijms-24-02970-s001.zip › Supplementary Data S2/2.9404845-9405392.png]

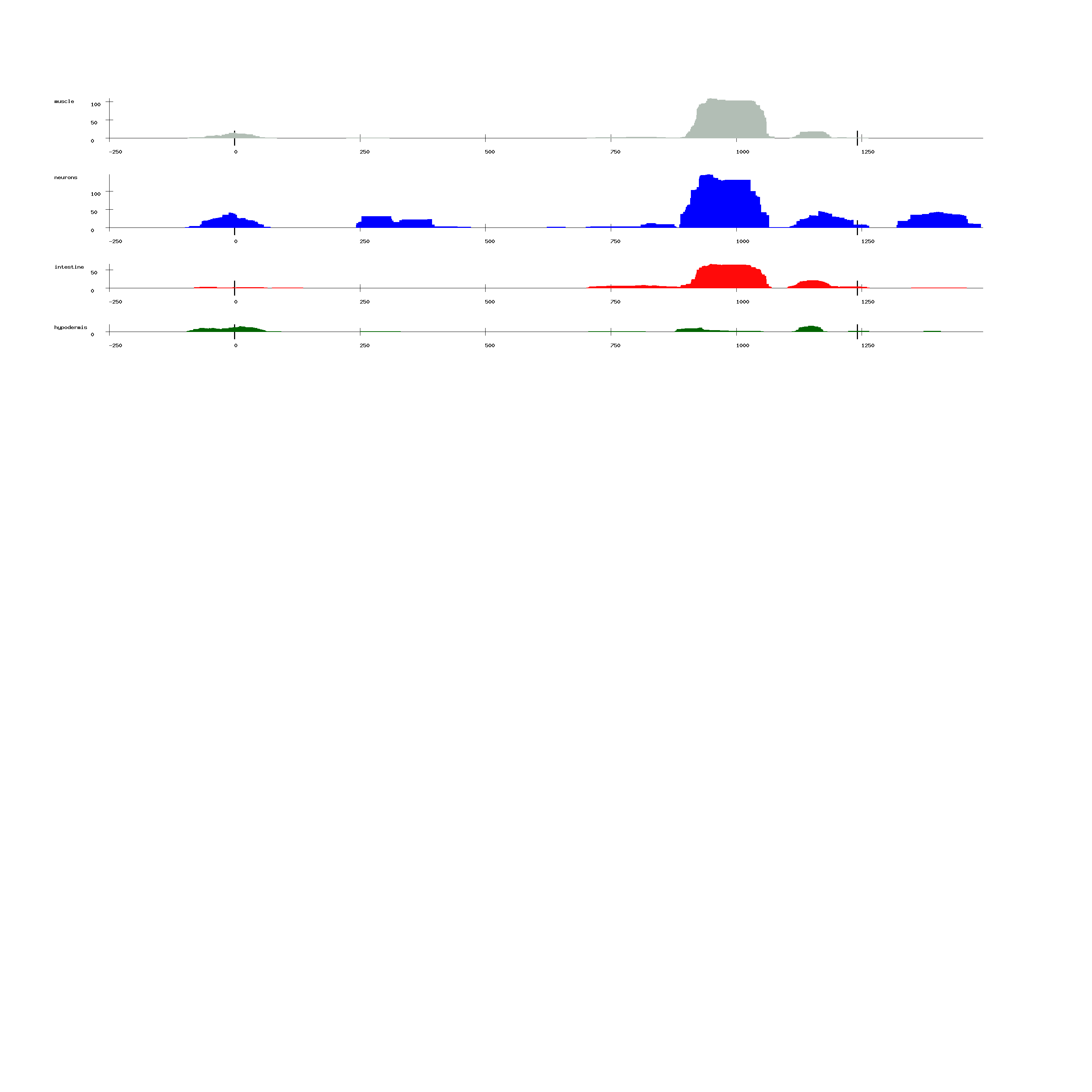

Supplement: Supplementary file 1 [file ijms-24-02970-s001.zip › Supplementary Data S2/2.9631891-9633131.png]

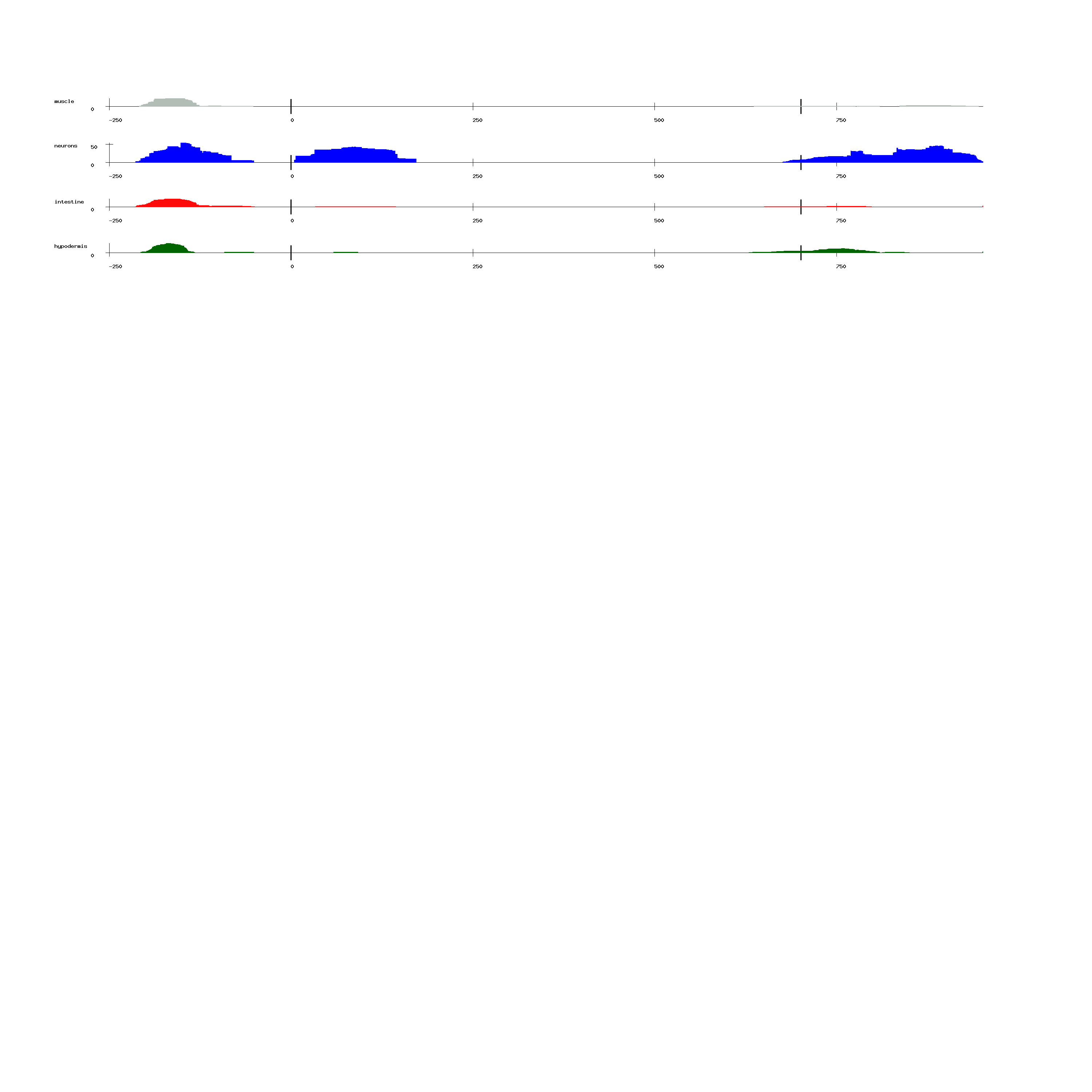

Supplement: Supplementary file 1 [file ijms-24-02970-s001.zip › Supplementary Data S2/2.9633206-9633906.png]

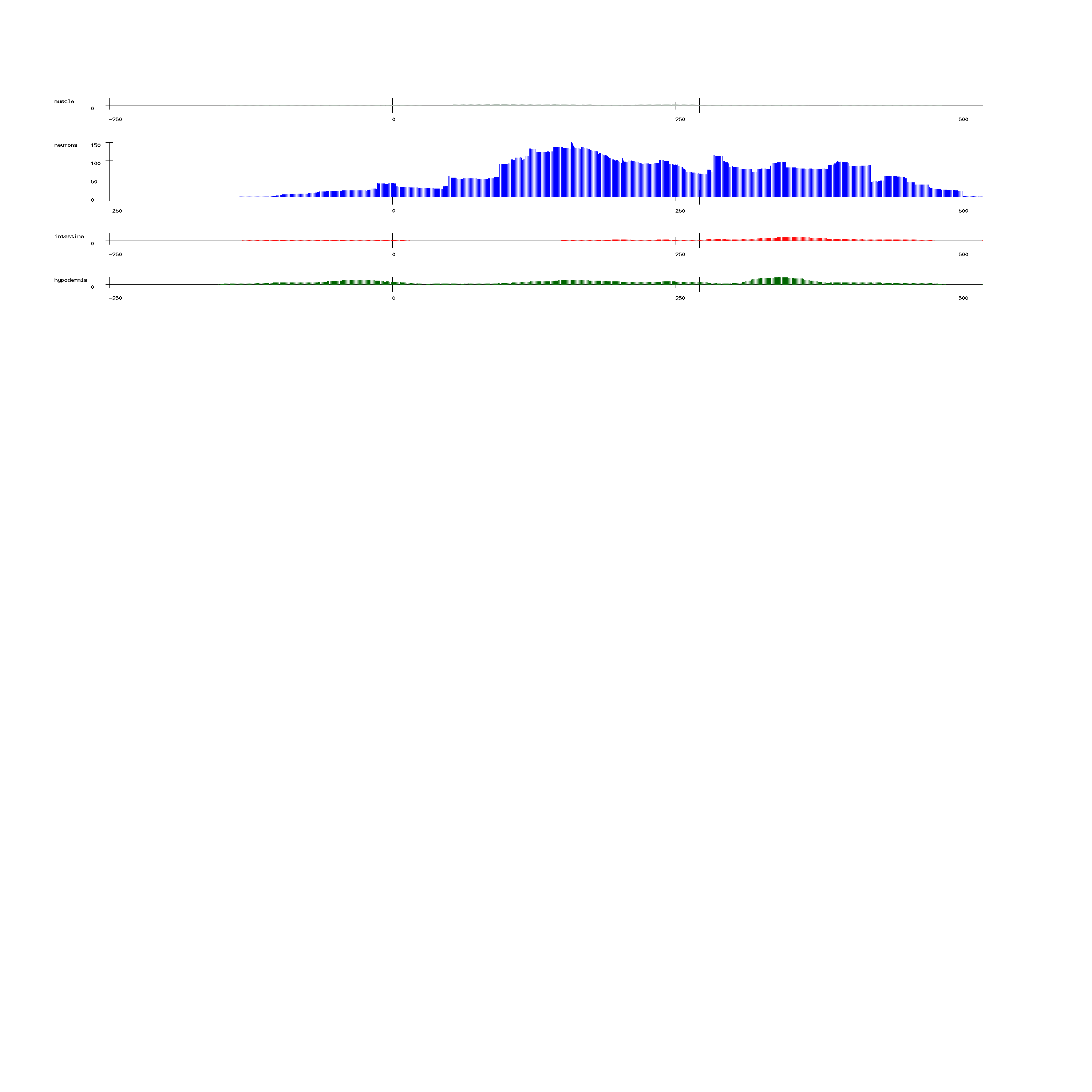

Supplement: Supplementary file 1 [file ijms-24-02970-s001.zip › Supplementary Data S2/2.9633989-9634259.png]

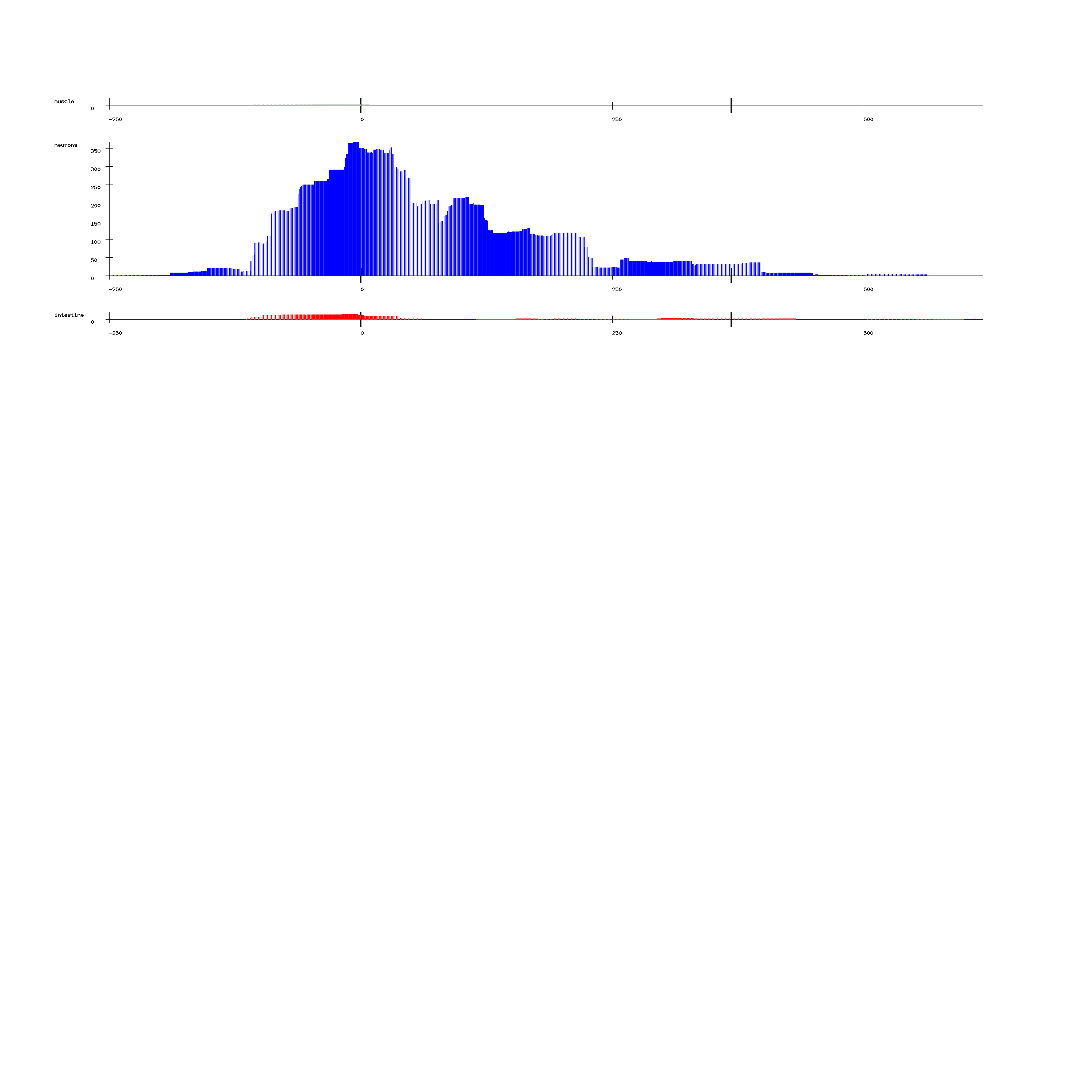

Supplement: Supplementary file 1 [file ijms-24-02970-s001.zip › Supplementary Data S2/2.9693772-9694139.png]

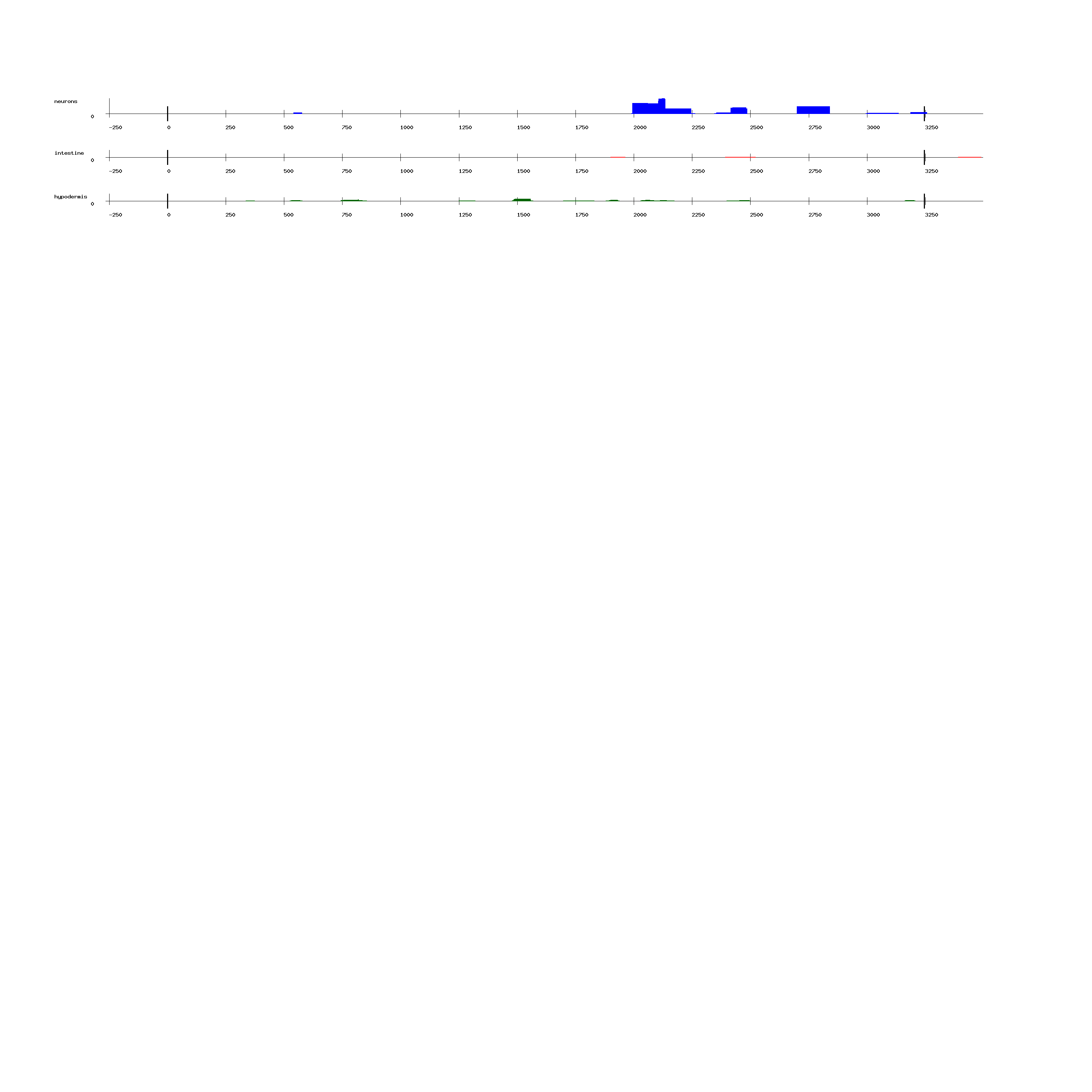

Supplement: Supplementary file 1 [file ijms-24-02970-s001.zip › Supplementary Data S2/3.1017334-1020579.png]

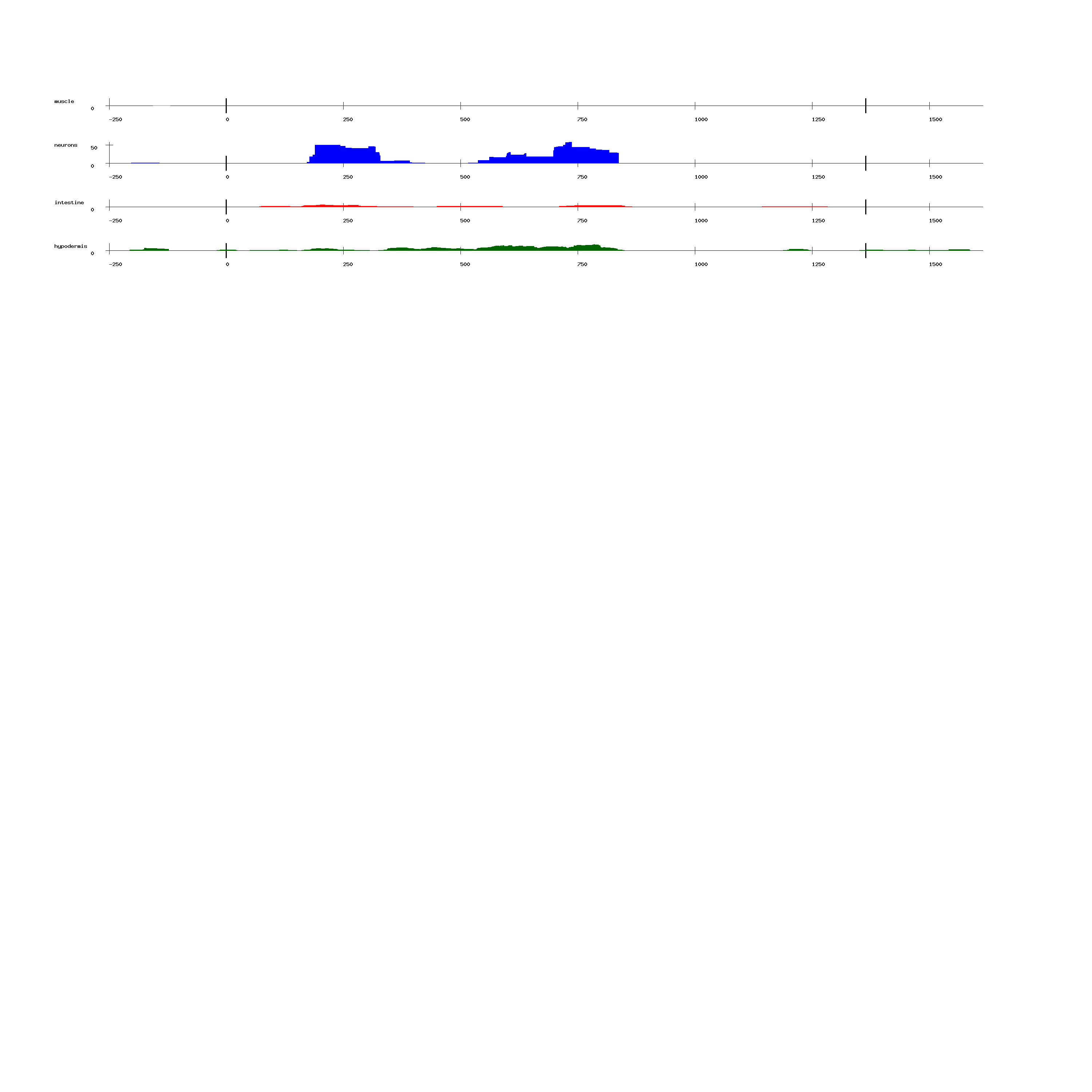

Supplement: Supplementary file 1 [file ijms-24-02970-s001.zip › Supplementary Data S2/3.10224644-10226007.png]

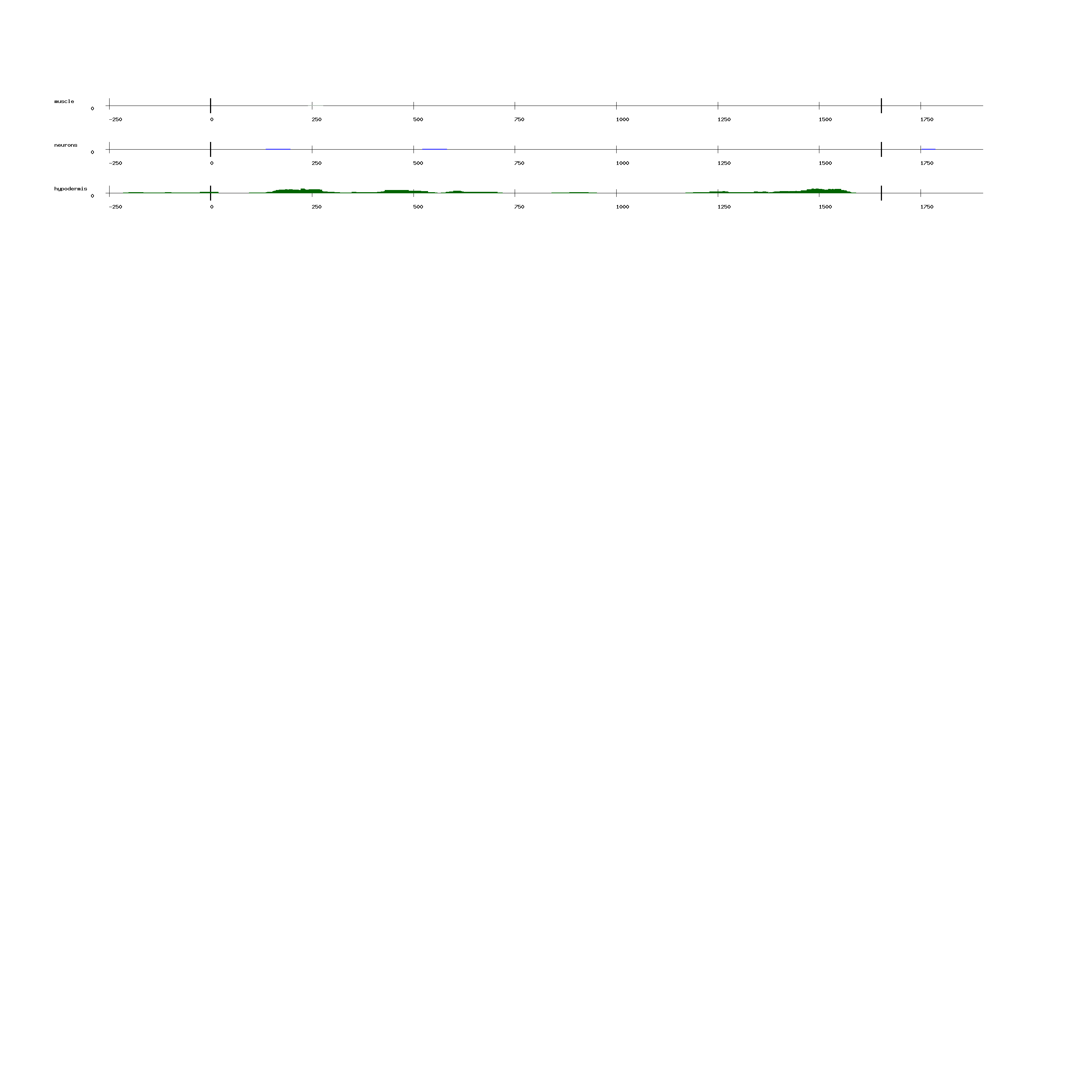

Supplement: Supplementary file 1 [file ijms-24-02970-s001.zip › Supplementary Data S2/3.10226211-10227863.png]

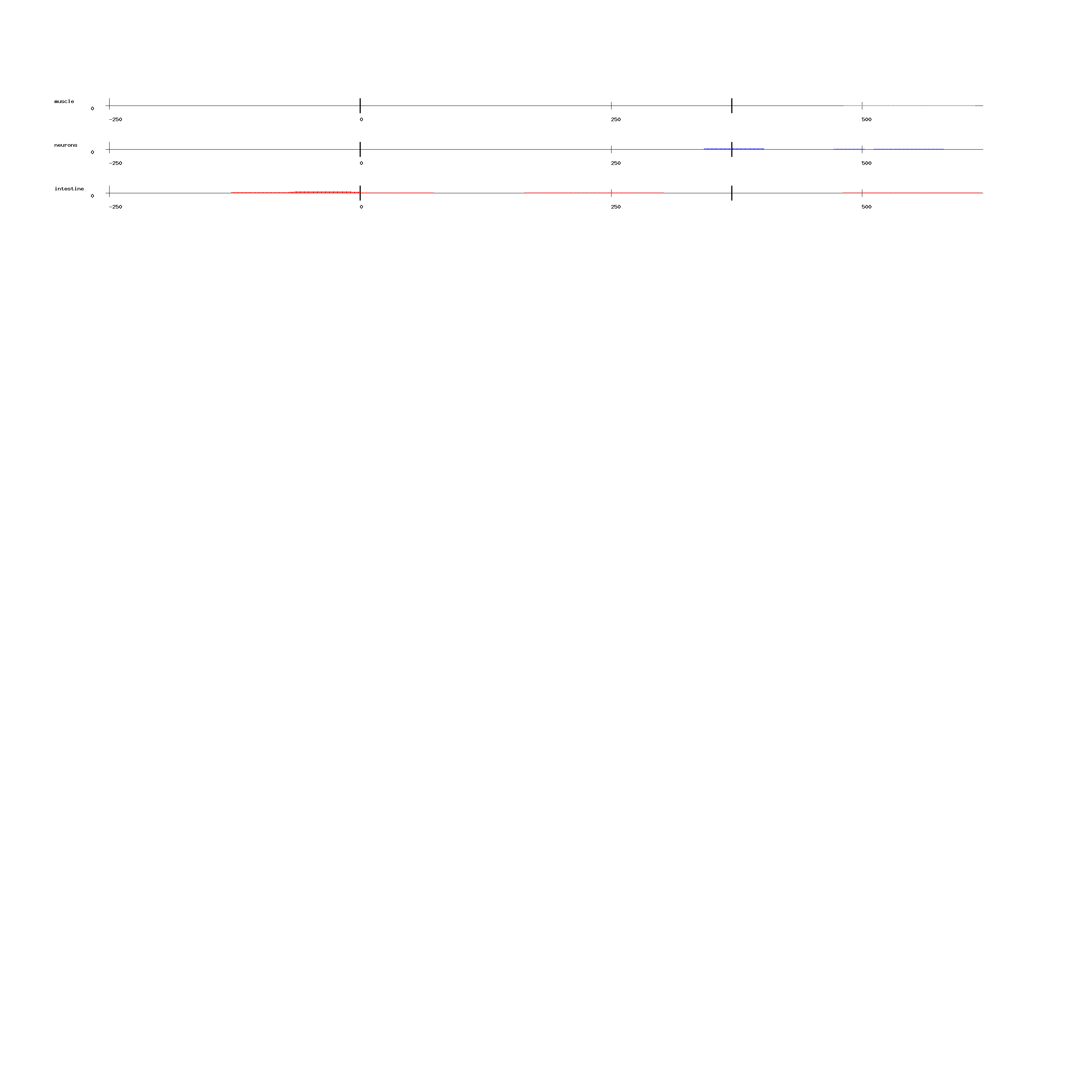

Supplement: Supplementary file 1 [file ijms-24-02970-s001.zip › Supplementary Data S2/3.10636973-10637342.png]

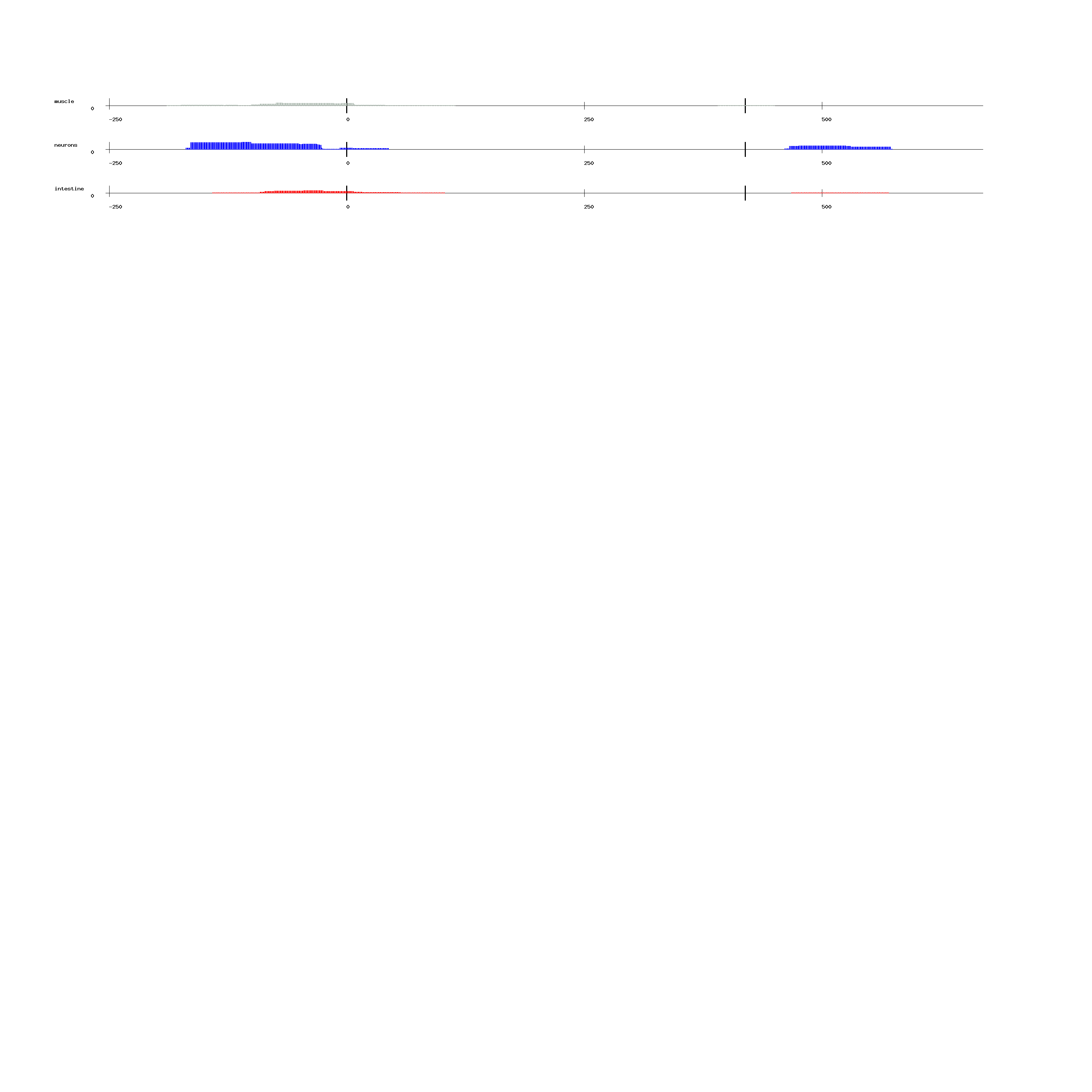

Supplement: Supplementary file 1 [file ijms-24-02970-s001.zip › Supplementary Data S2/3.10678429-10678847.png]

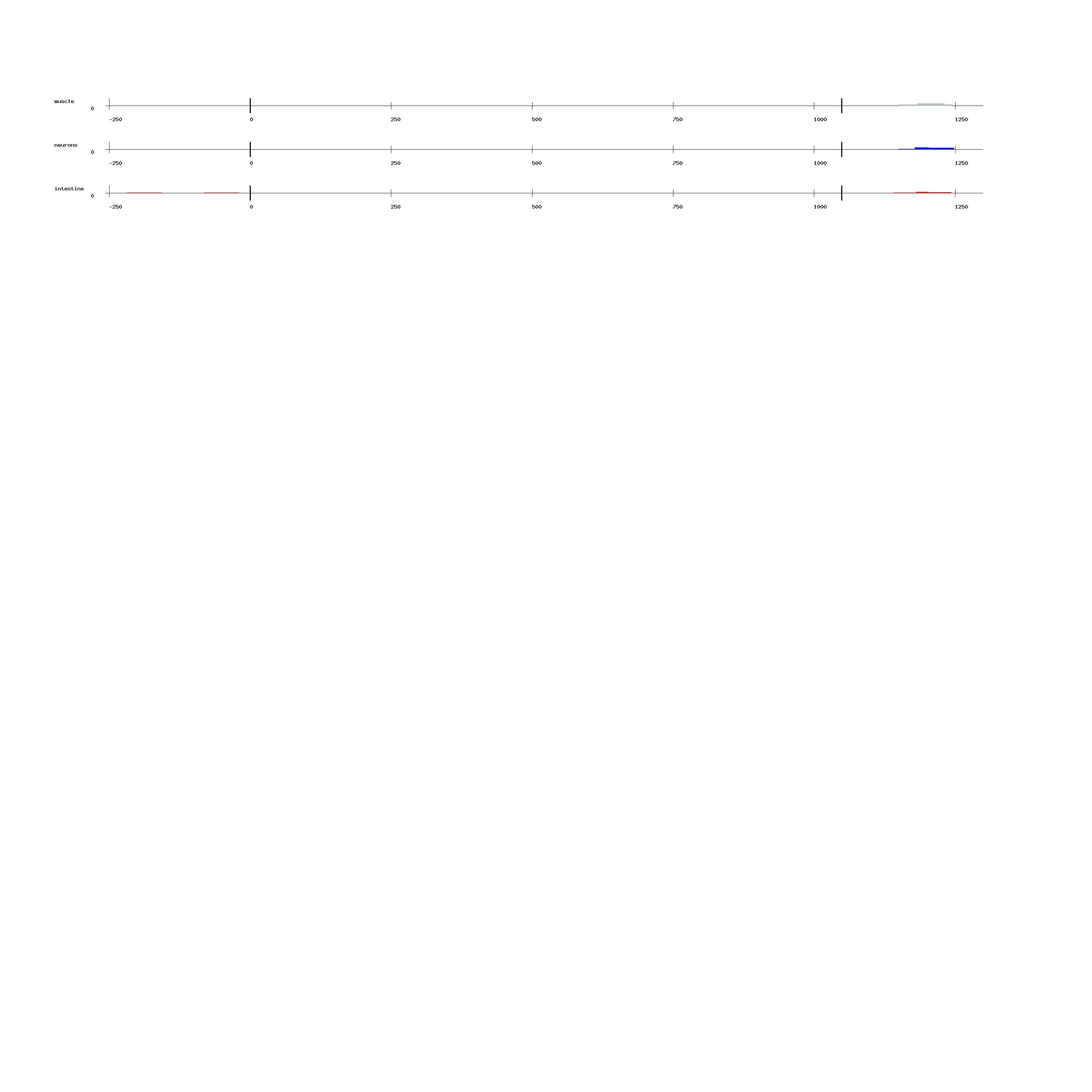

Supplement: Supplementary file 1 [file ijms-24-02970-s001.zip › Supplementary Data S2/3.10683224-10684272.png]

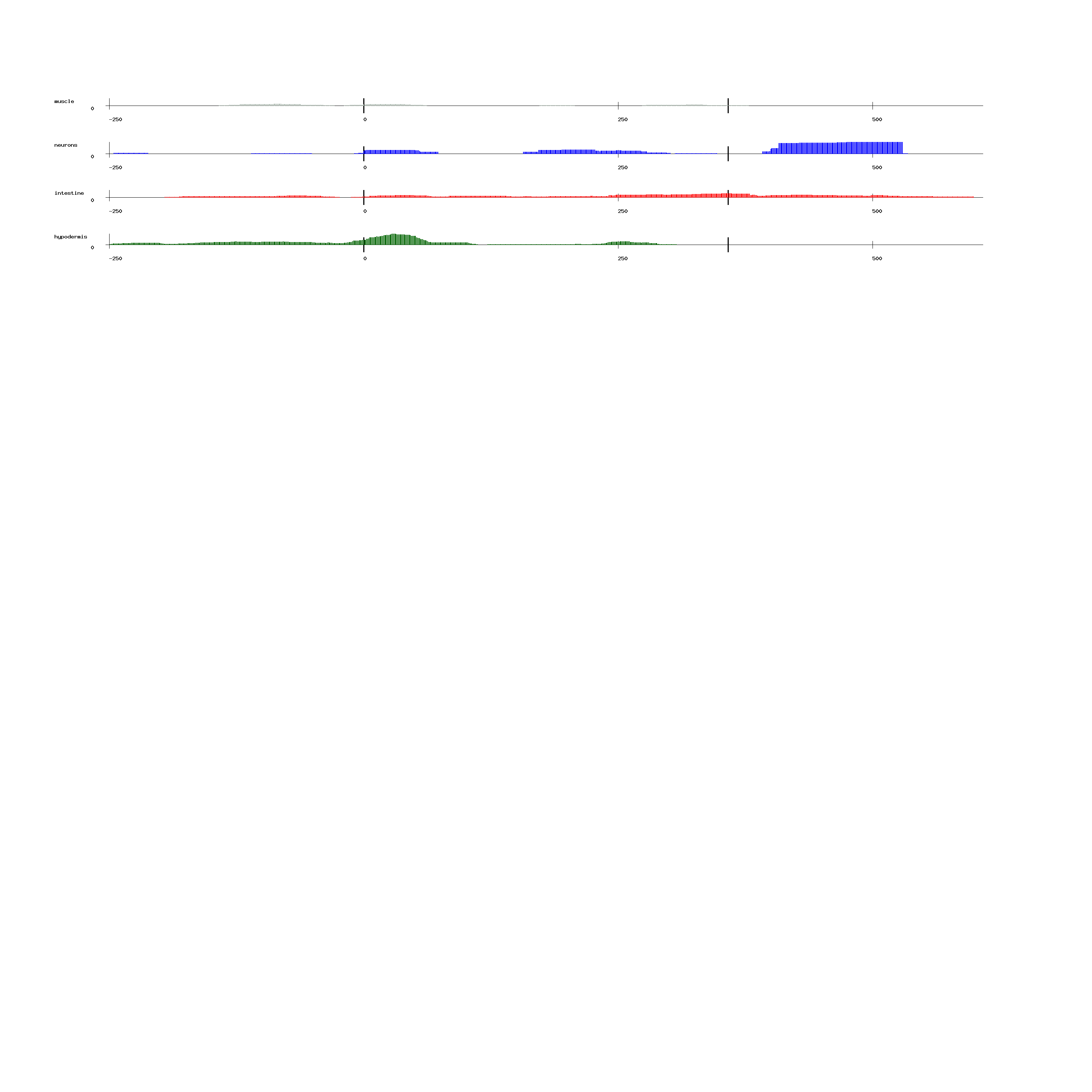

Supplement: Supplementary file 1 [file ijms-24-02970-s001.zip › Supplementary Data S2/3.10792337-10792694.png]

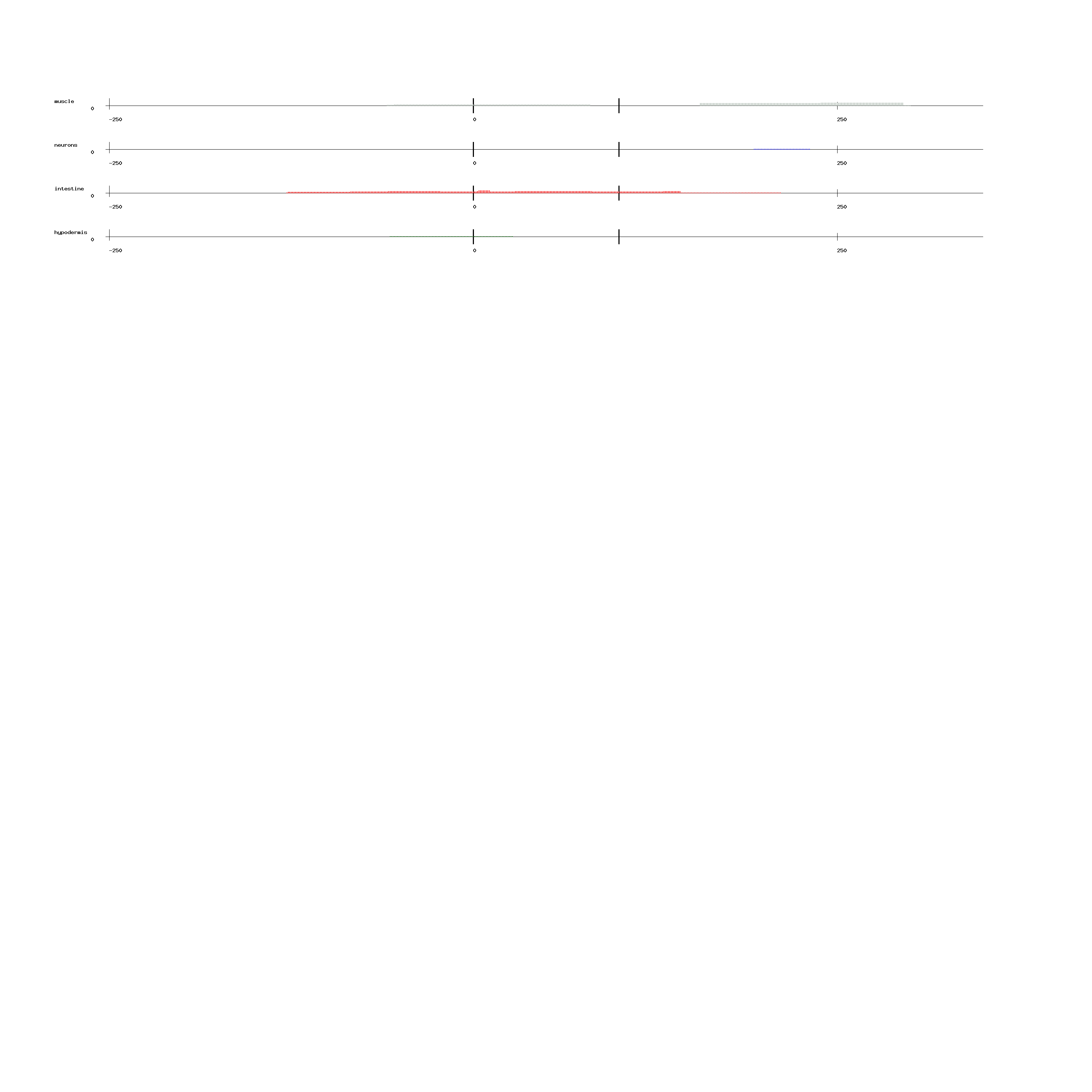

Supplement: Supplementary file 1 [file ijms-24-02970-s001.zip › Supplementary Data S2/3.10796467-10796566.png]

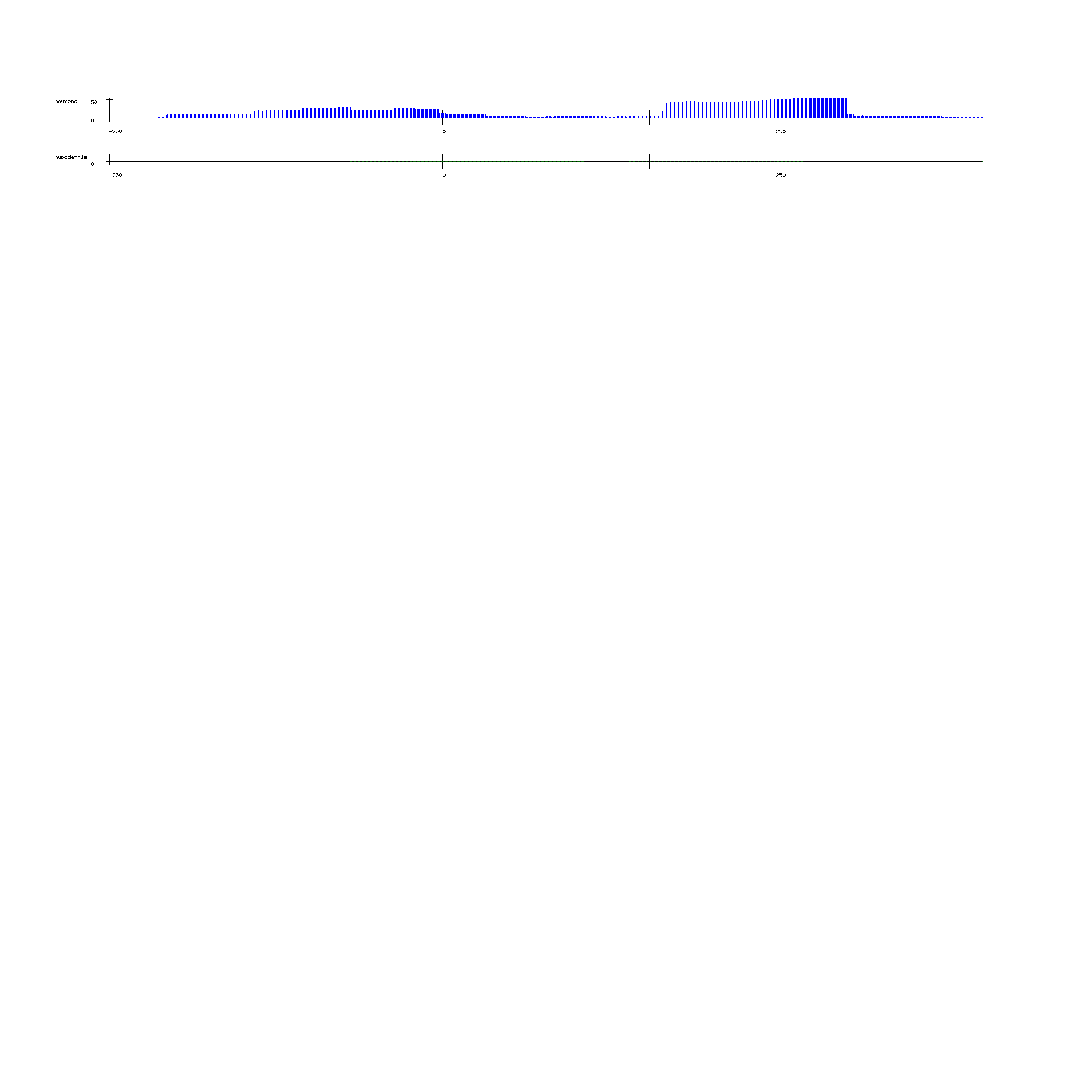

Supplement: Supplementary file 1 [file ijms-24-02970-s001.zip › Supplementary Data S2/3.11002899-11003053.png]

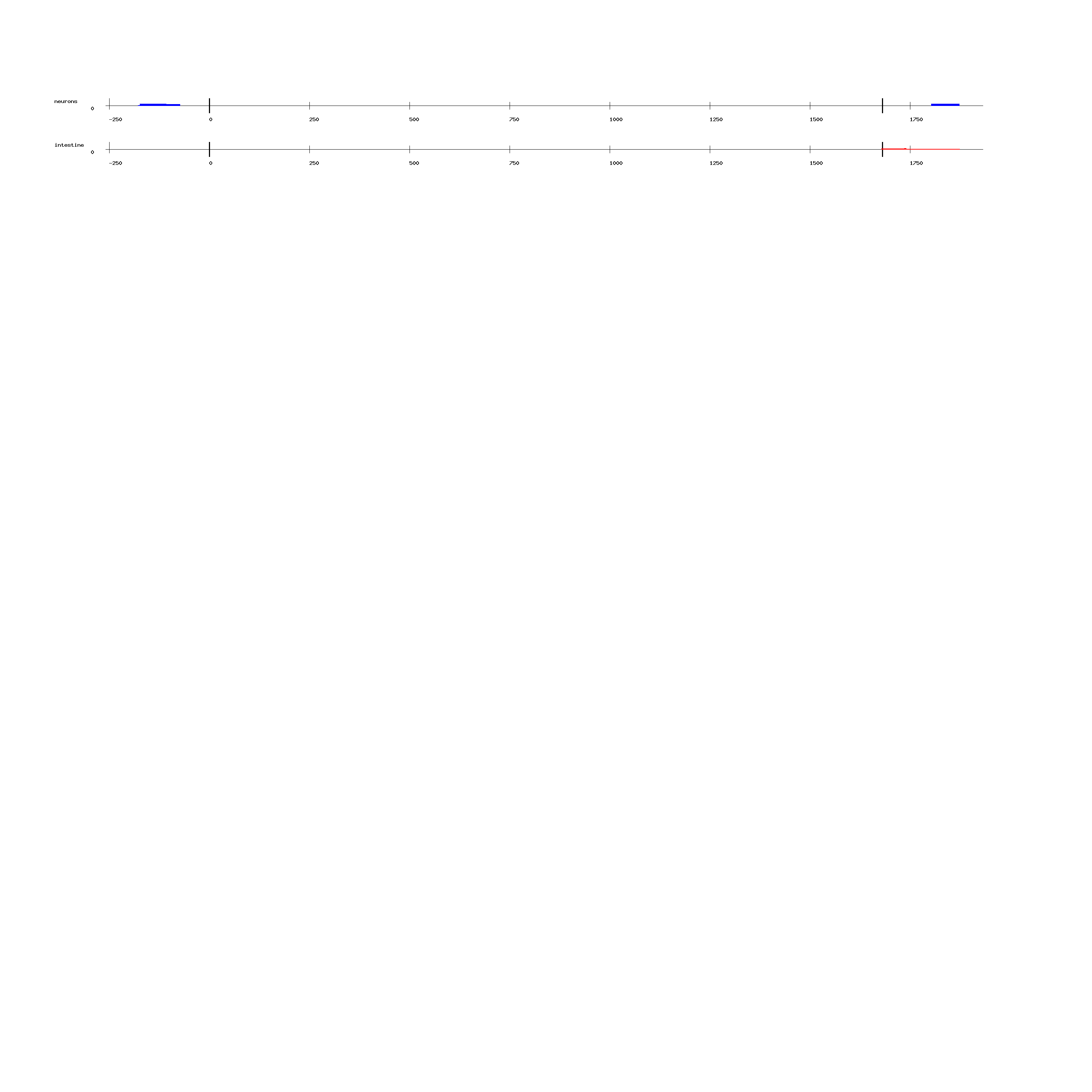

Supplement: Supplementary file 1 [file ijms-24-02970-s001.zip › Supplementary Data S2/3.11004687-11006367.png]

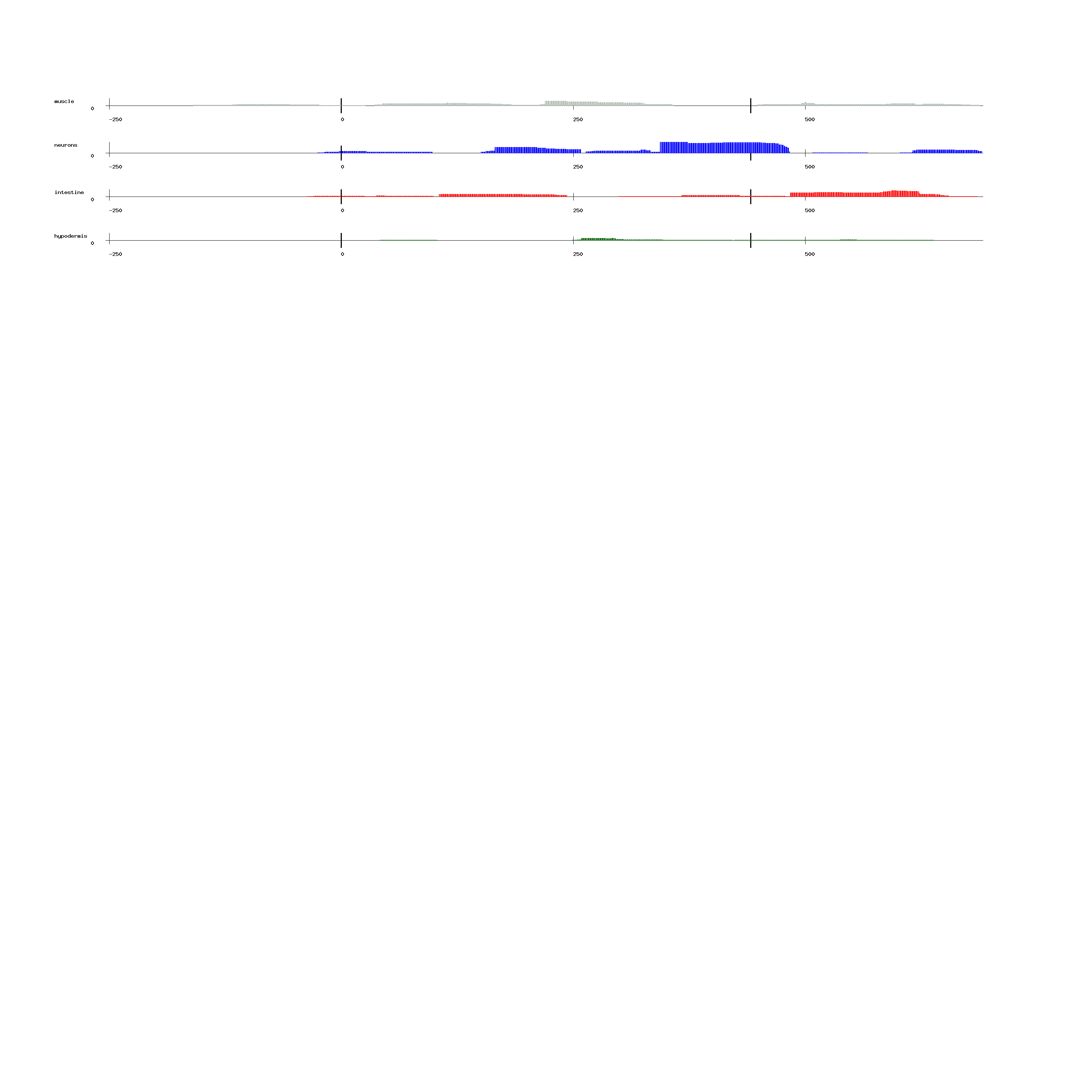

Supplement: Supplementary file 1 [file ijms-24-02970-s001.zip › Supplementary Data S2/3.11242813-11243253.png]

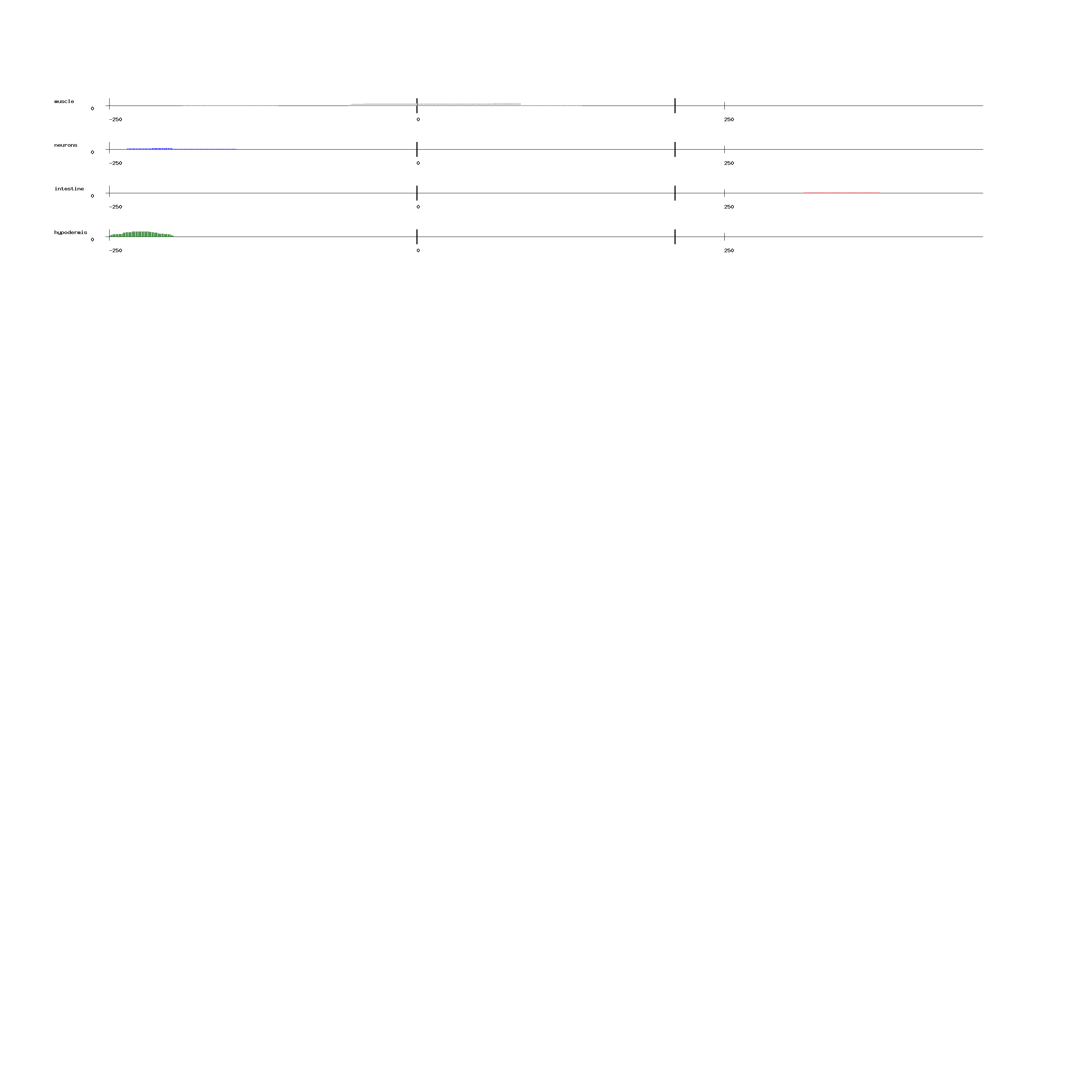

Supplement: Supplementary file 1 [file ijms-24-02970-s001.zip › Supplementary Data S2/3.11311767-11311976.png]

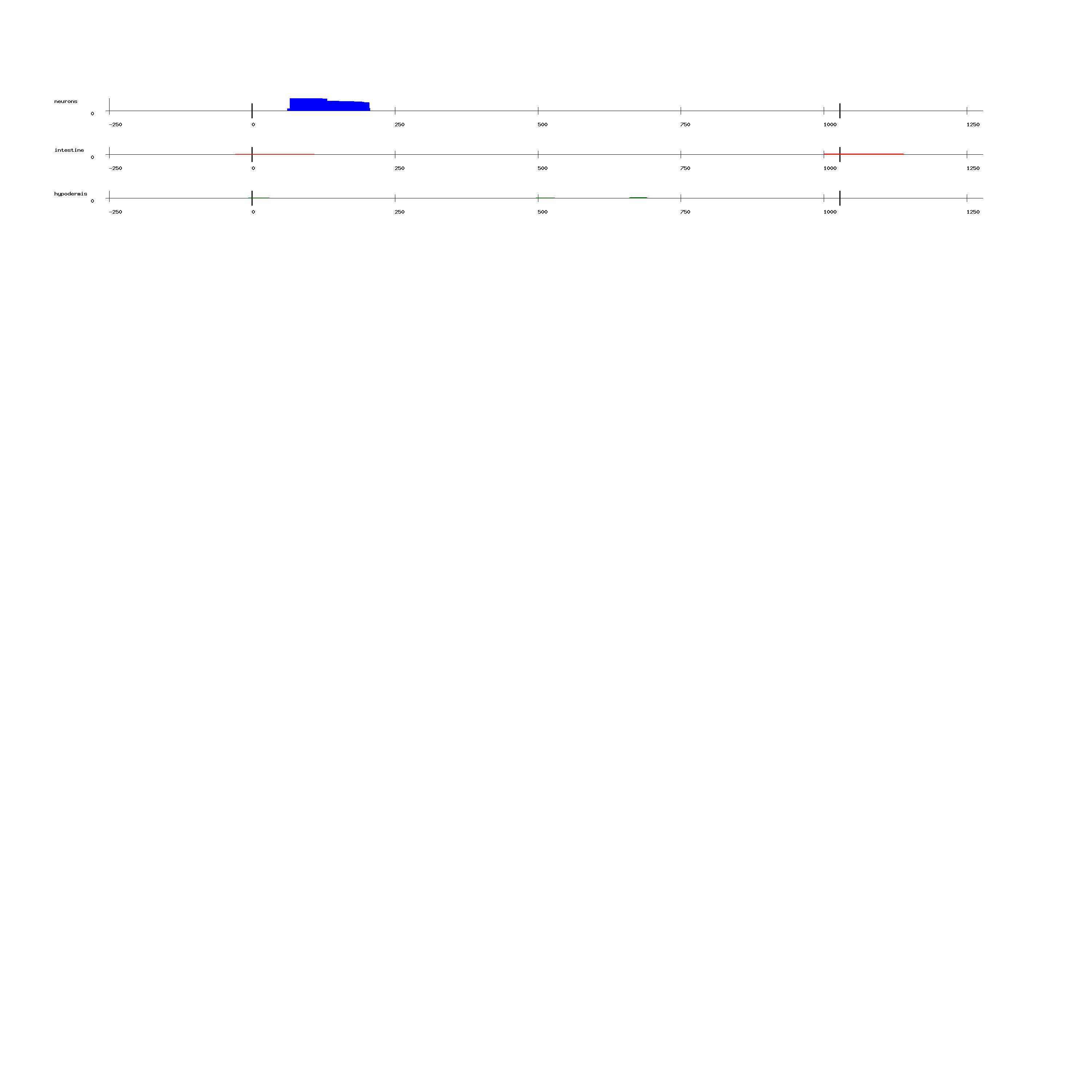

Supplement: Supplementary file 1 [file ijms-24-02970-s001.zip › Supplementary Data S2/3.1148439-1149466.png]

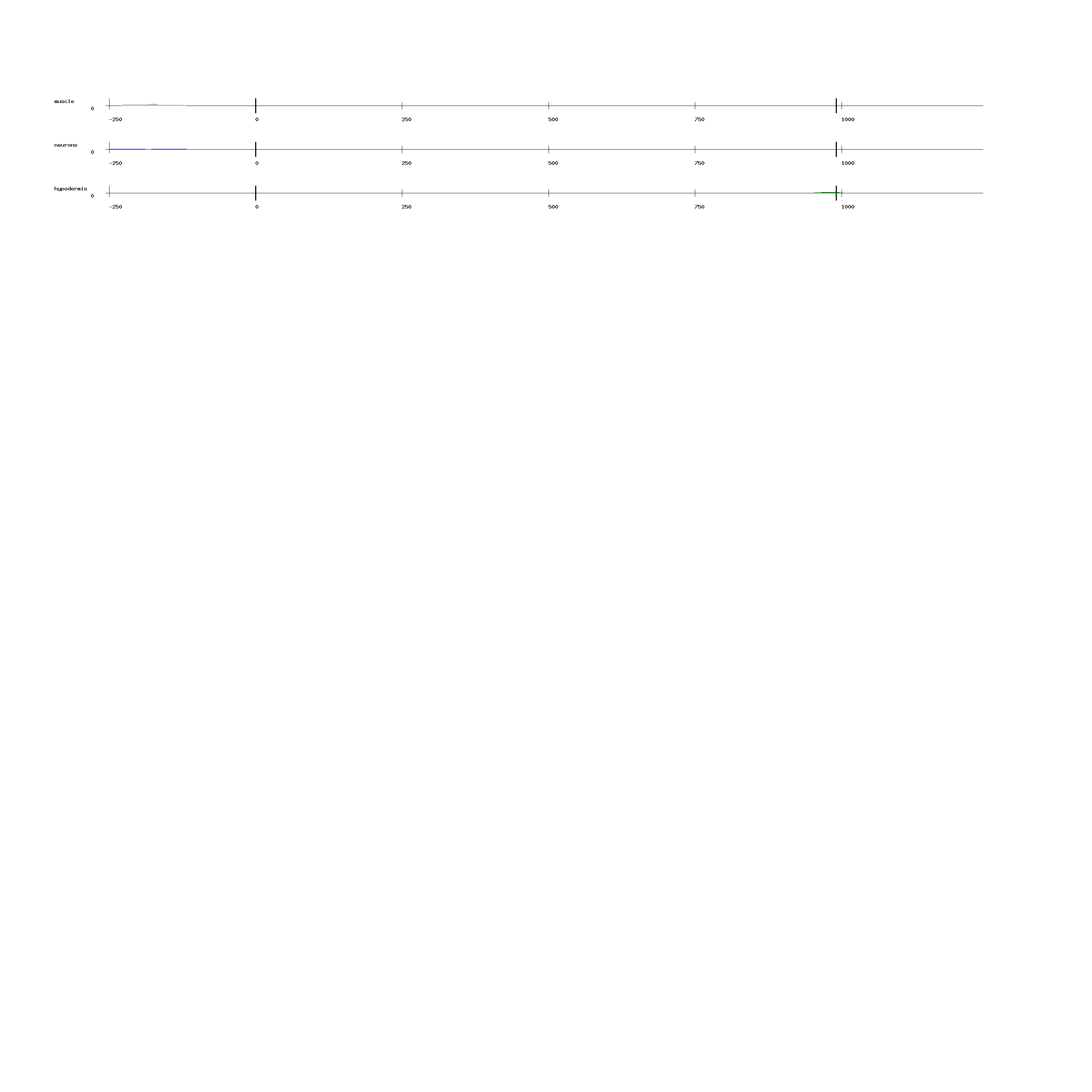

Supplement: Supplementary file 1 [file ijms-24-02970-s001.zip › Supplementary Data S2/3.11546762-11547752.png]

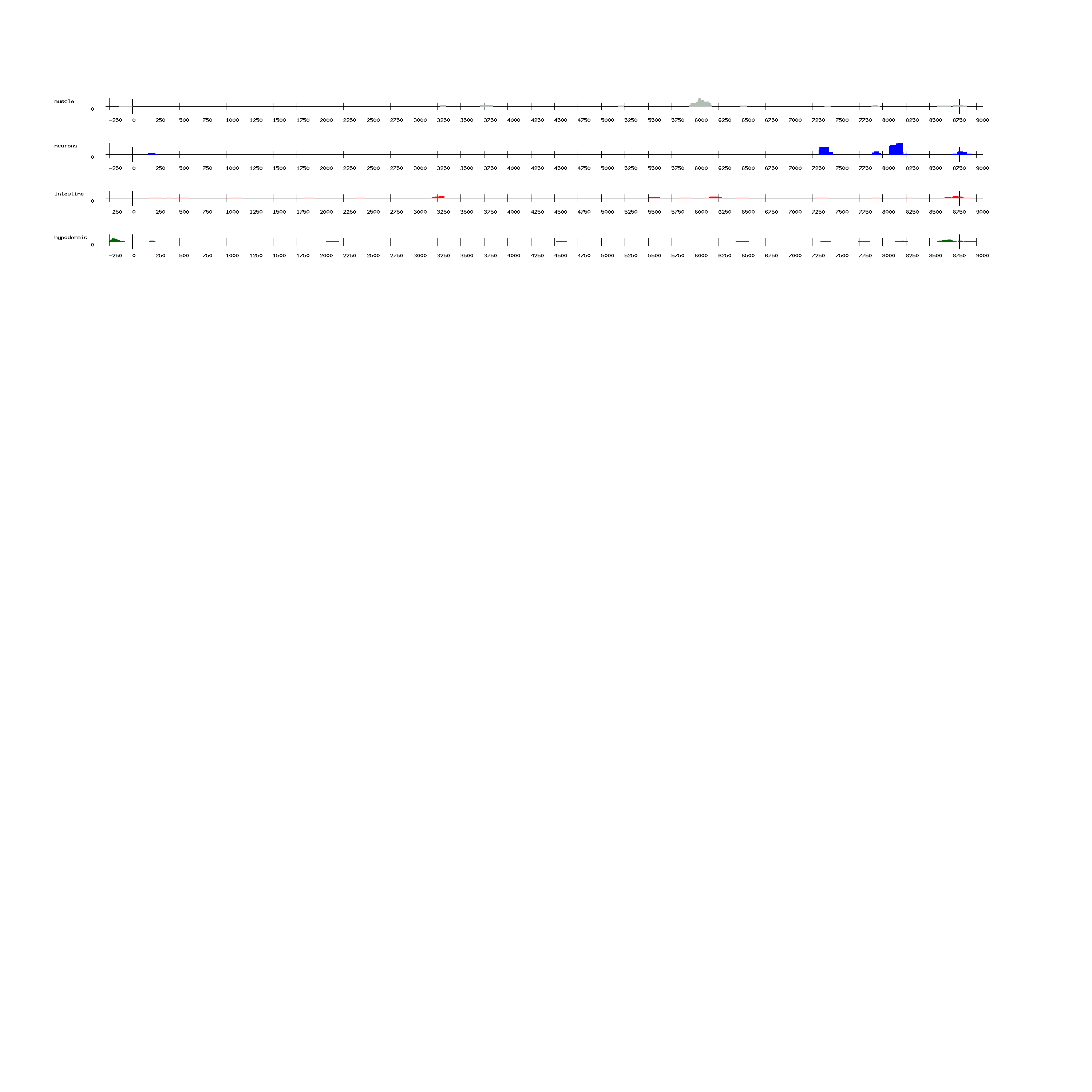

Supplement: Supplementary file 1 [file ijms-24-02970-s001.zip › Supplementary Data S2/3.11593439-11602256.png]

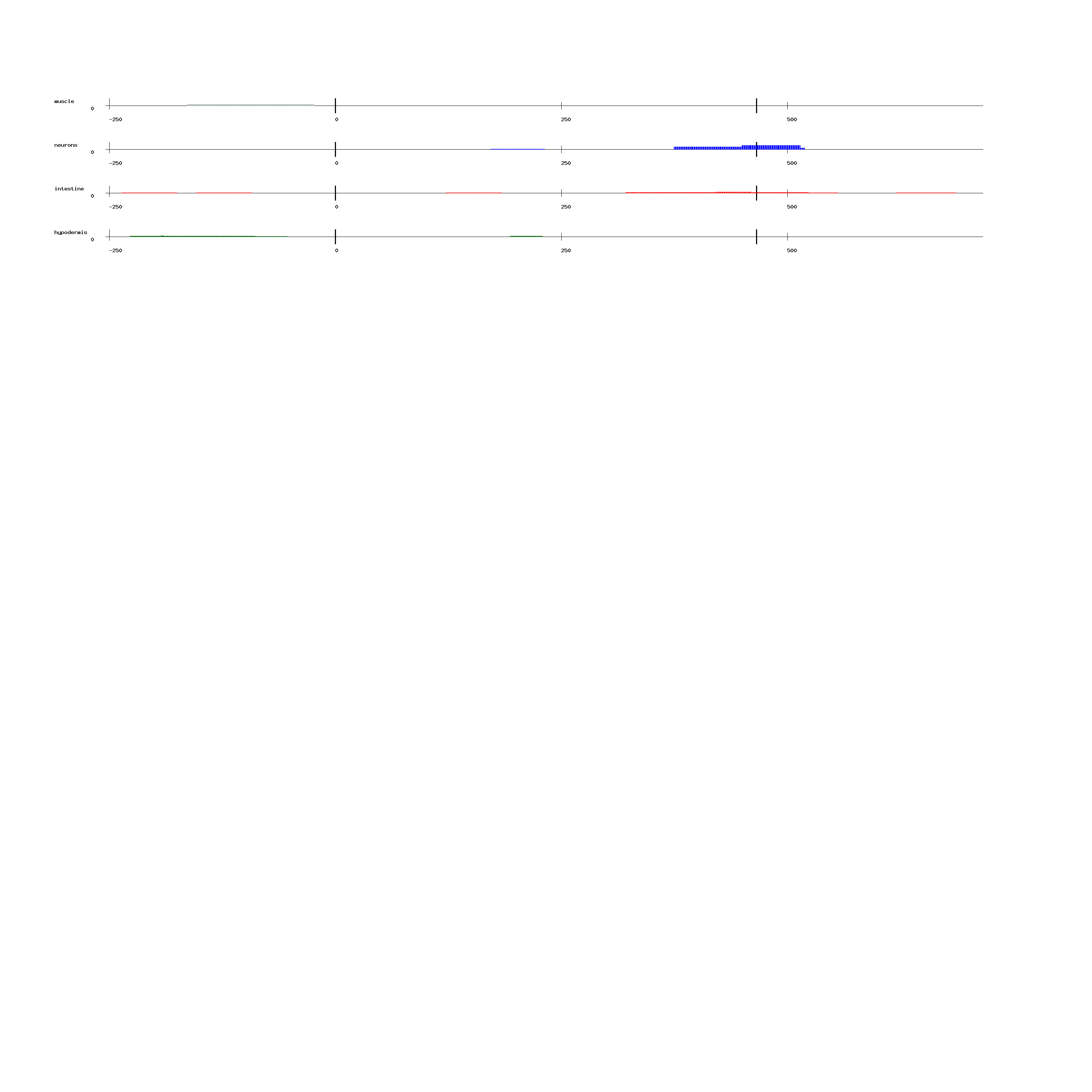

Supplement: Supplementary file 1 [file ijms-24-02970-s001.zip › Supplementary Data S2/3.12004285-12004750.png]

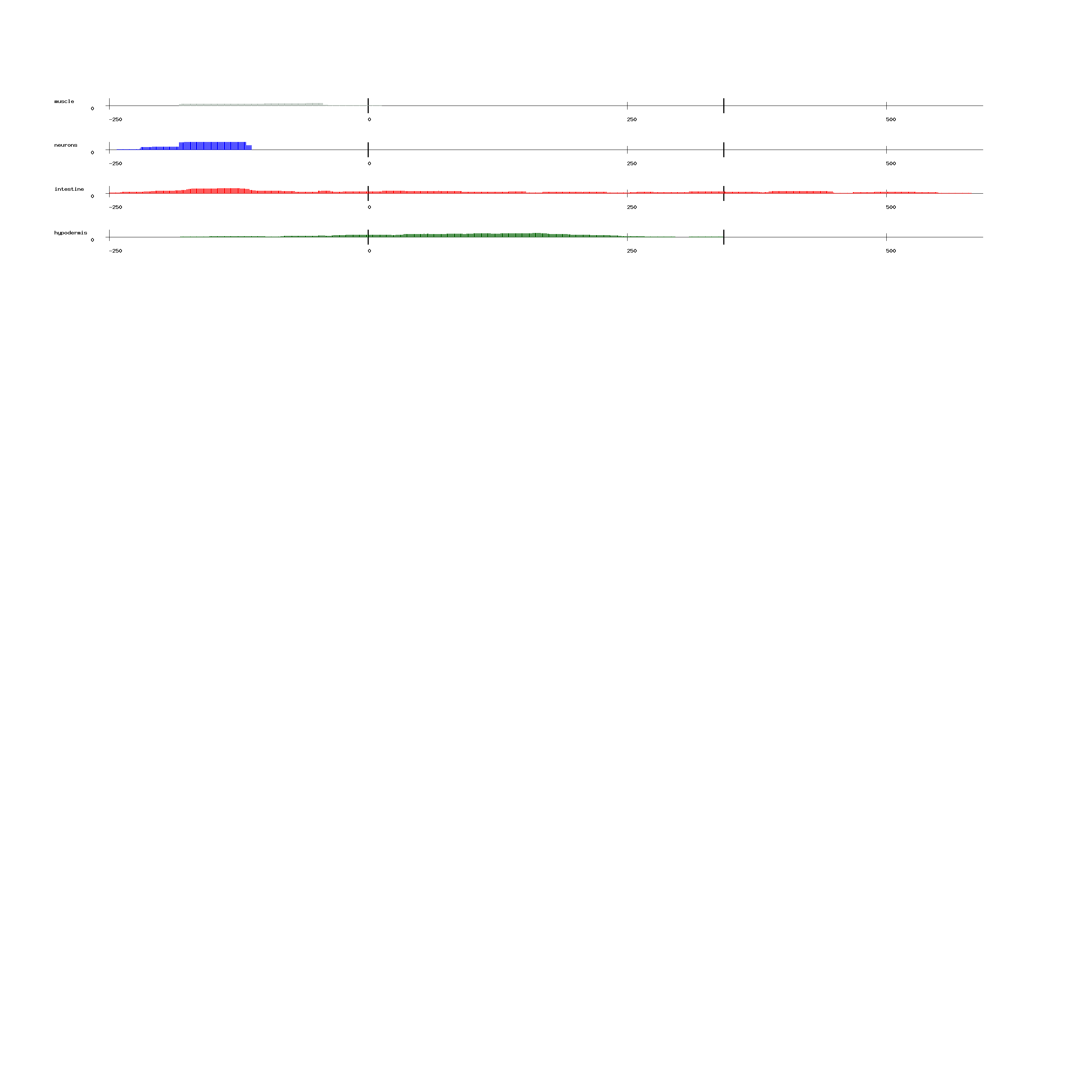

Supplement: Supplementary file 1 [file ijms-24-02970-s001.zip › Supplementary Data S2/3.12005666-12006008.png]

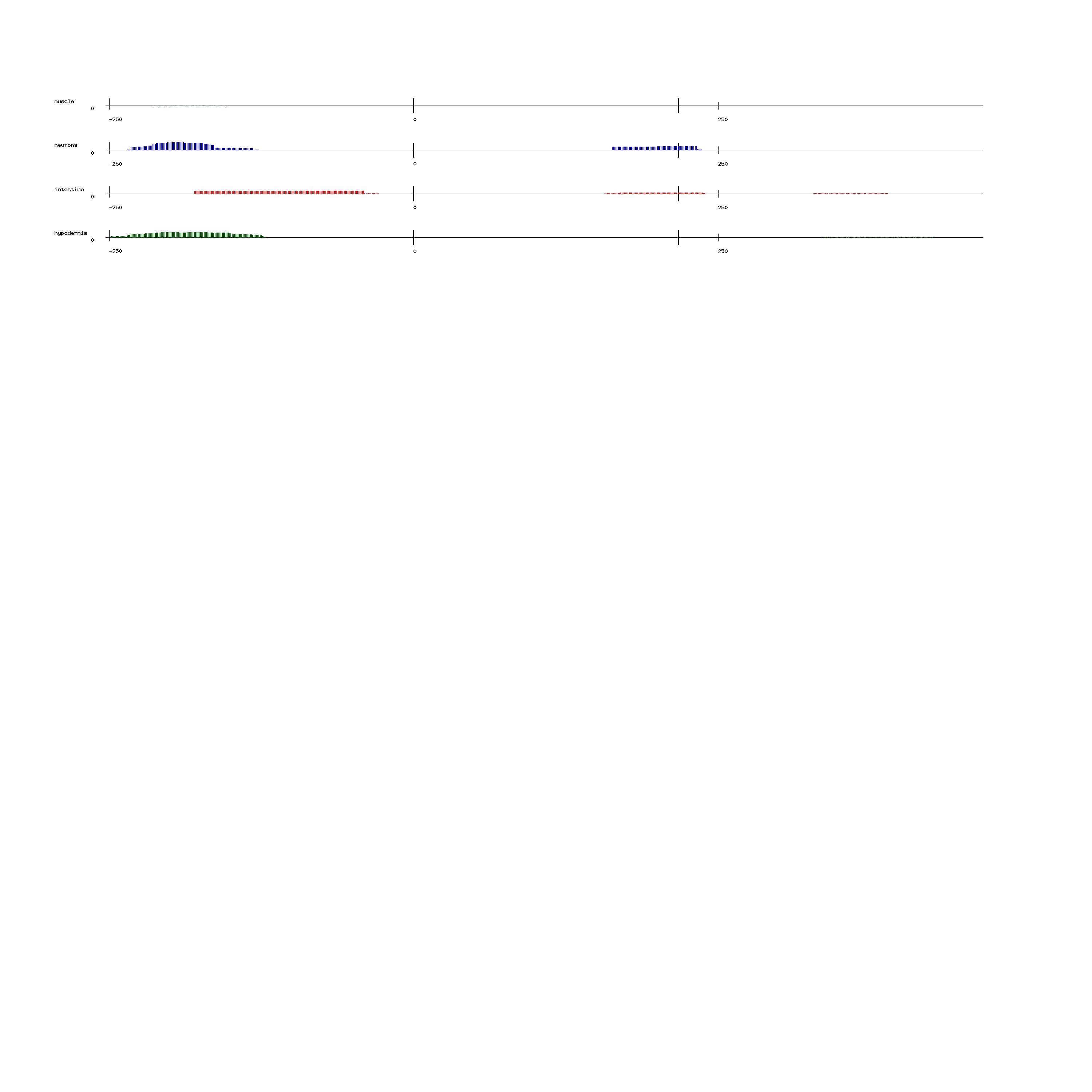

Supplement: Supplementary file 1 [file ijms-24-02970-s001.zip › Supplementary Data S2/3.12009165-12009381.png]

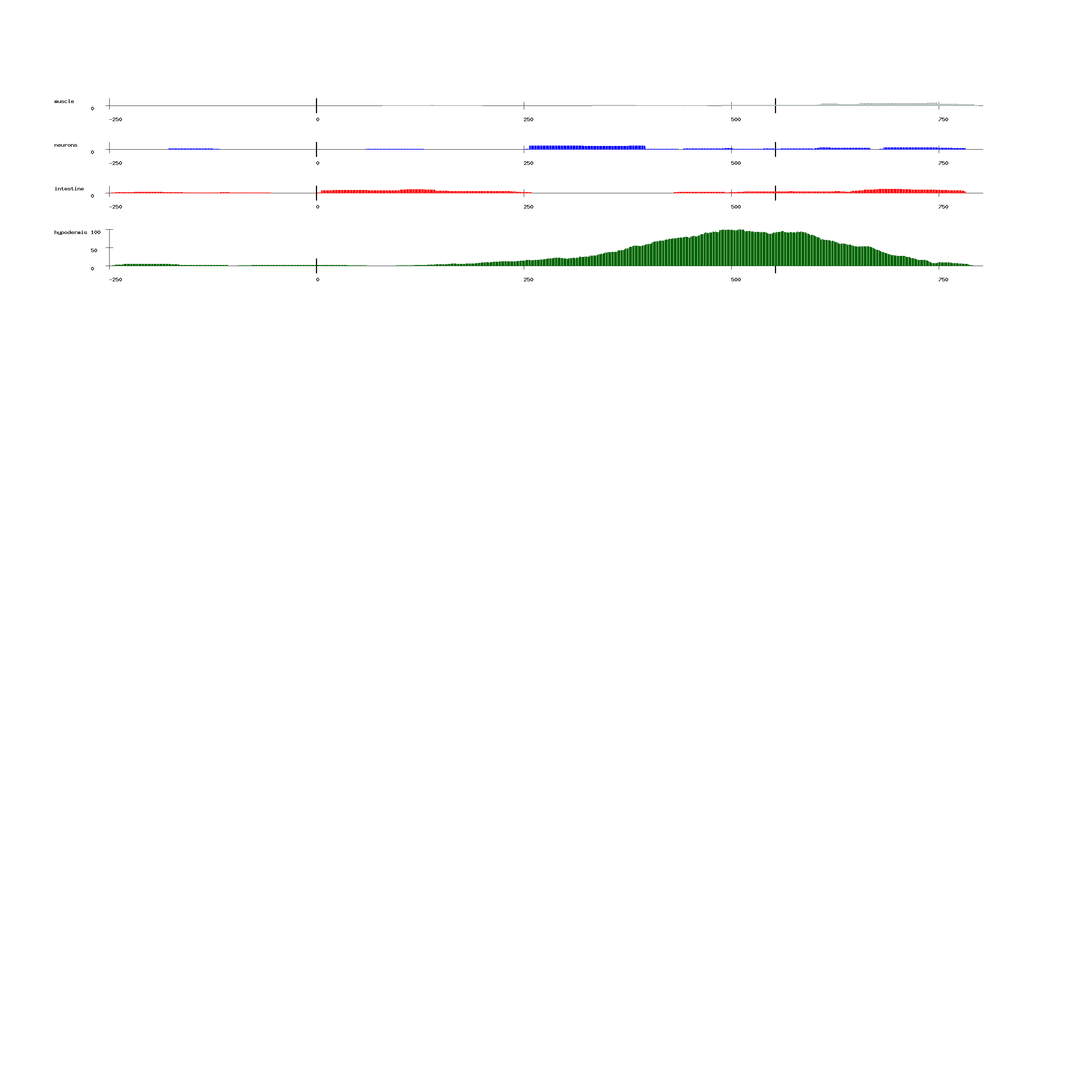

Supplement: Supplementary file 1 [file ijms-24-02970-s001.zip › Supplementary Data S2/3.12017198-12017750.png]

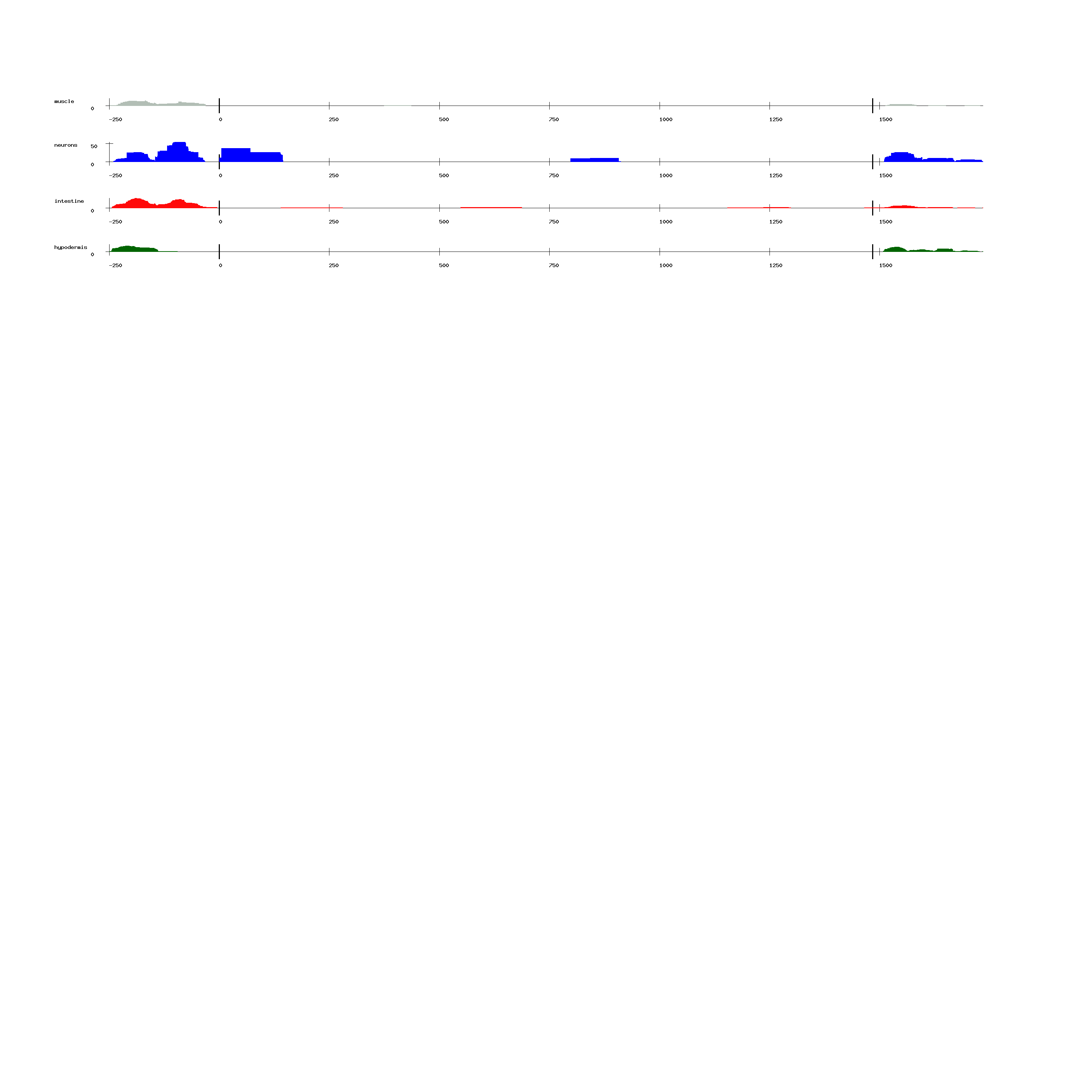

Supplement: Supplementary file 1 [file ijms-24-02970-s001.zip › Supplementary Data S2/3.12072321-12073804.png]

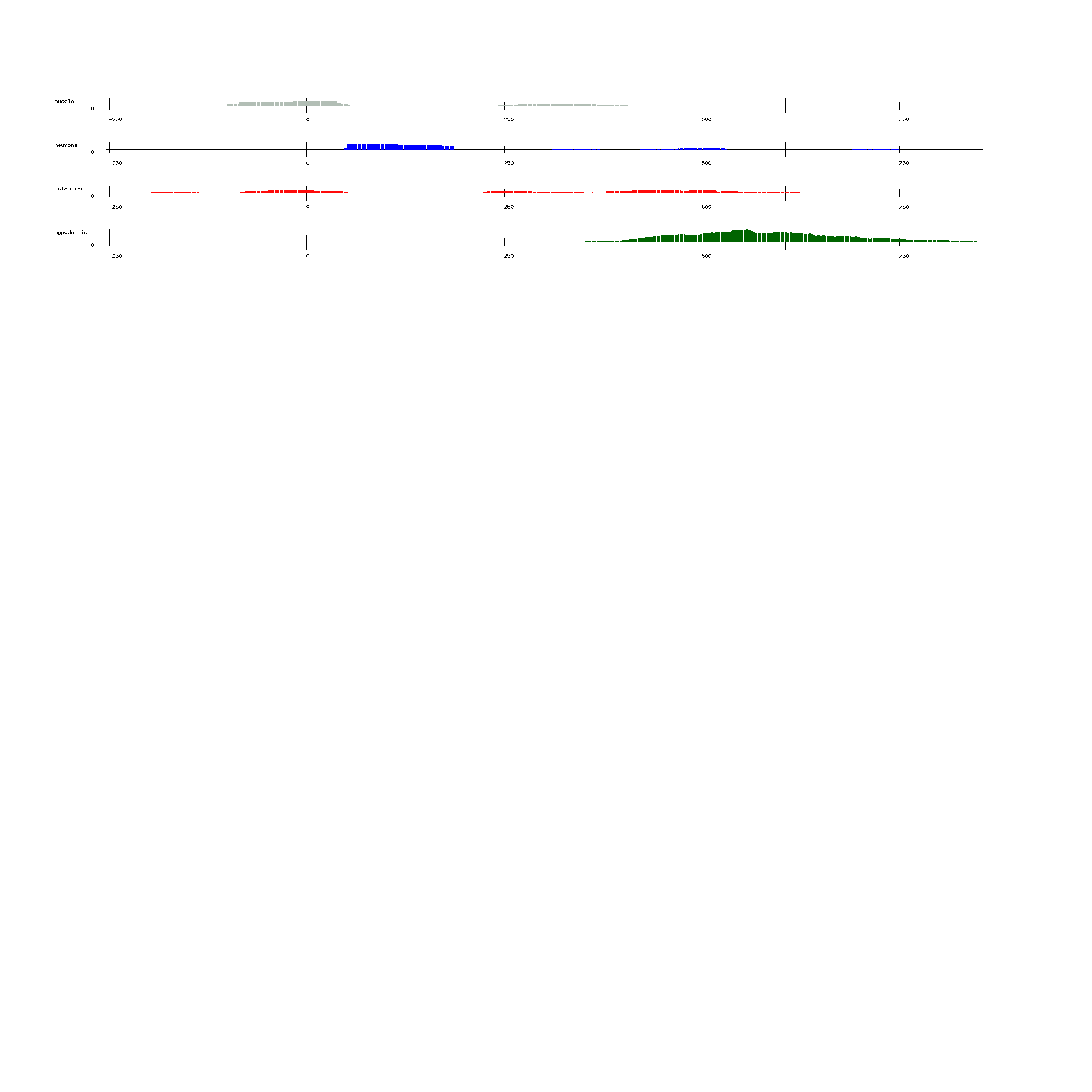

Supplement: Supplementary file 1 [file ijms-24-02970-s001.zip › Supplementary Data S2/3.12305325-12305929.png]

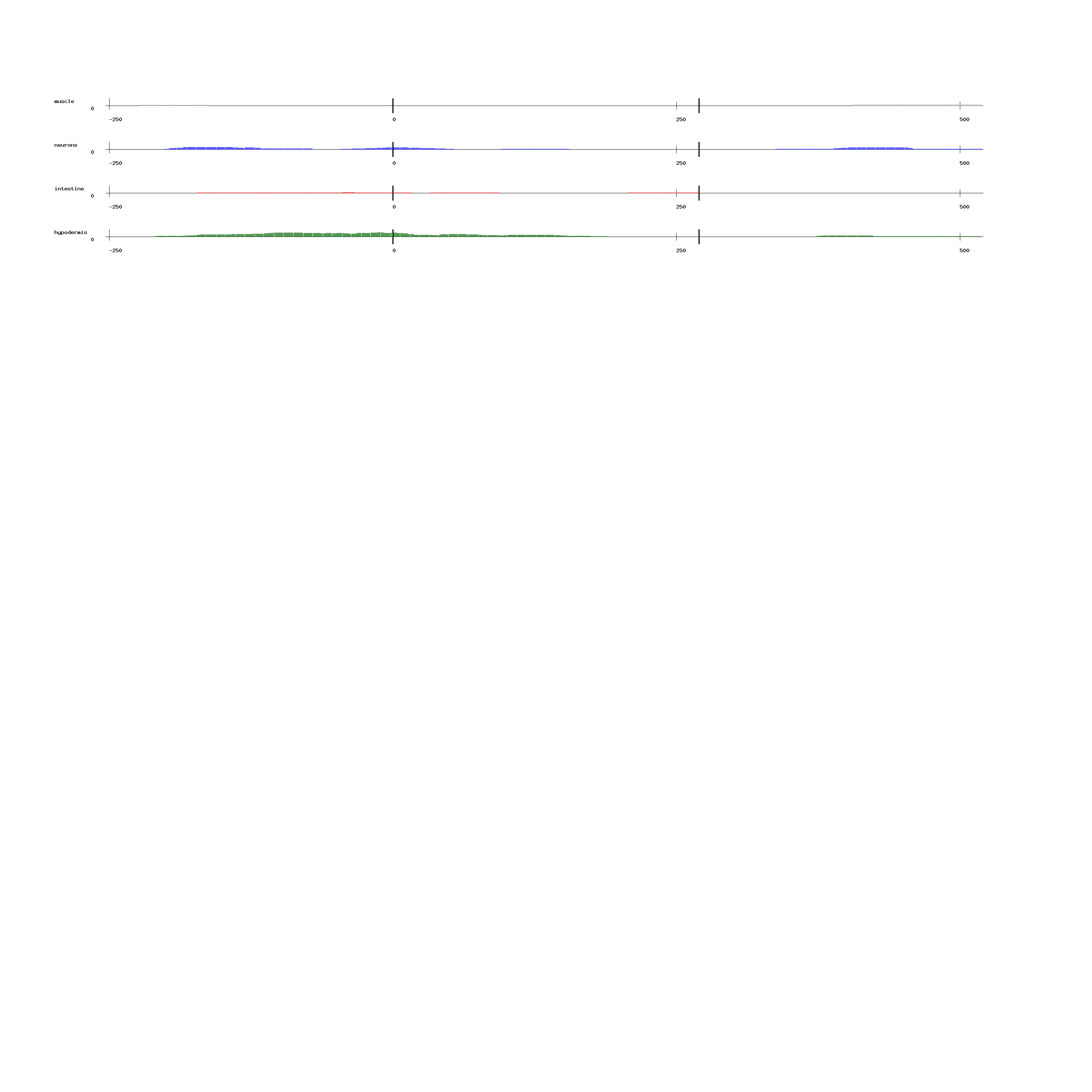

Supplement: Supplementary file 1 [file ijms-24-02970-s001.zip › Supplementary Data S2/3.12581020-12581289.png]

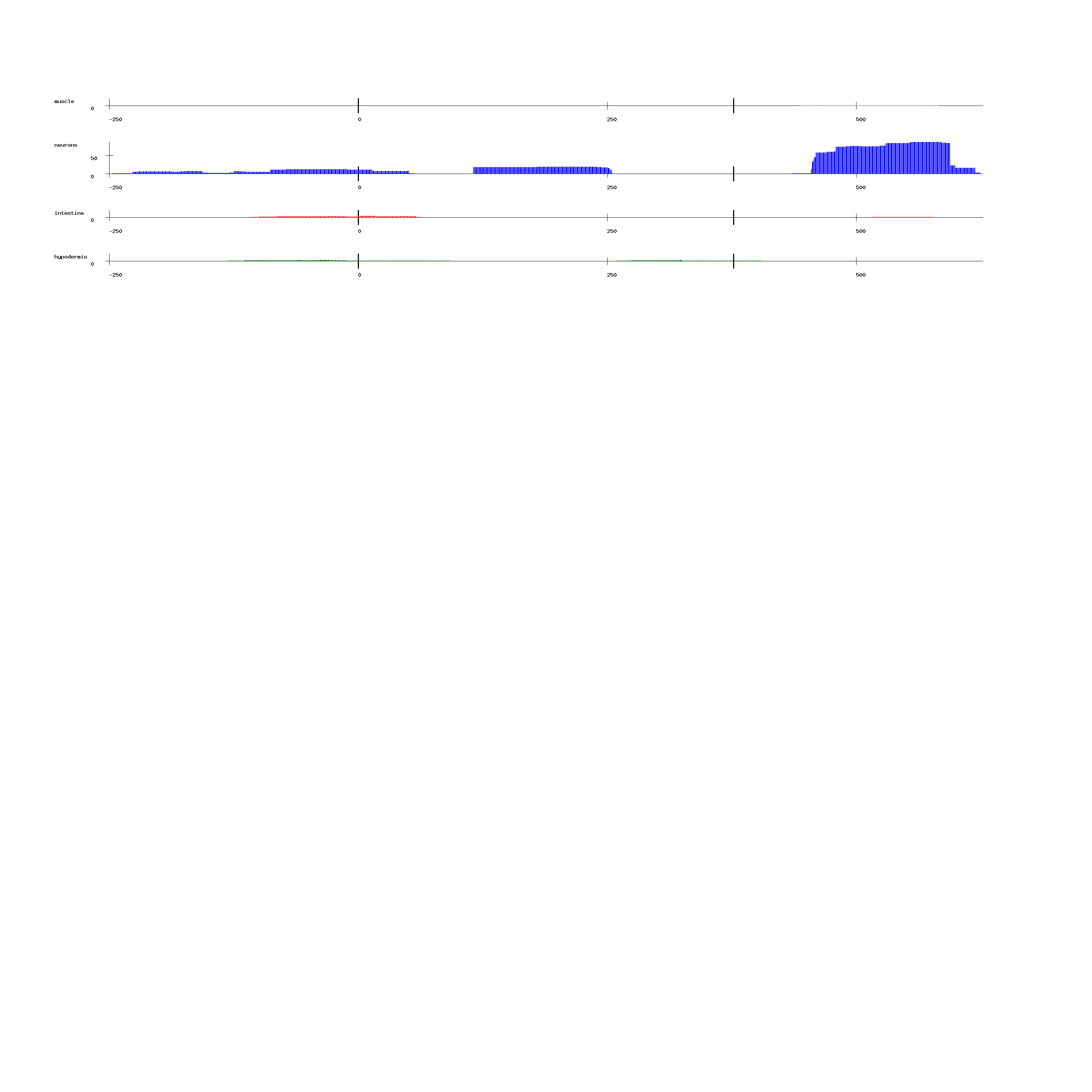

Supplement: Supplementary file 1 [file ijms-24-02970-s001.zip › Supplementary Data S2/3.12584489-12584865.png]

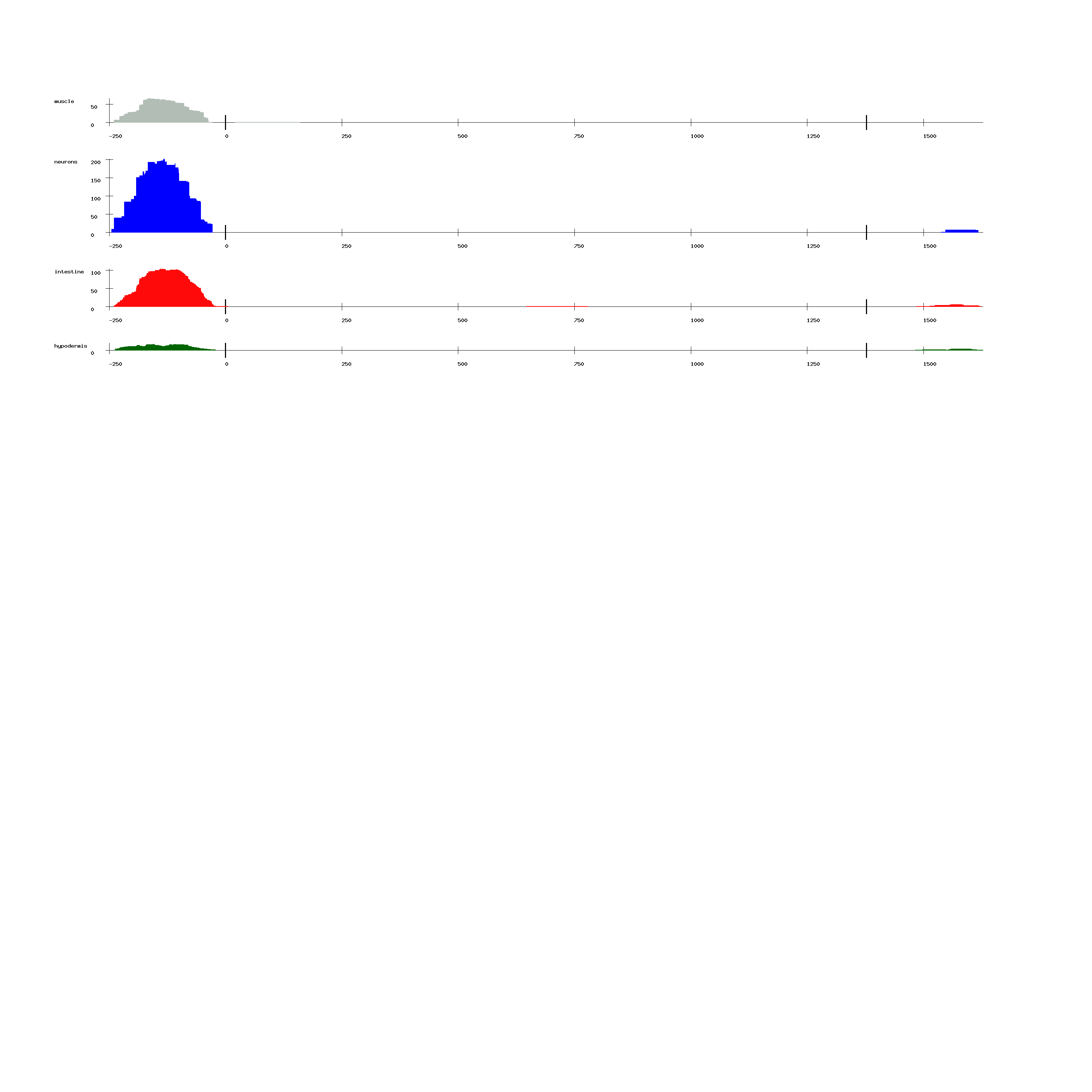

Supplement: Supplementary file 1 [file ijms-24-02970-s001.zip › Supplementary Data S2/3.1260242-1261618.png]

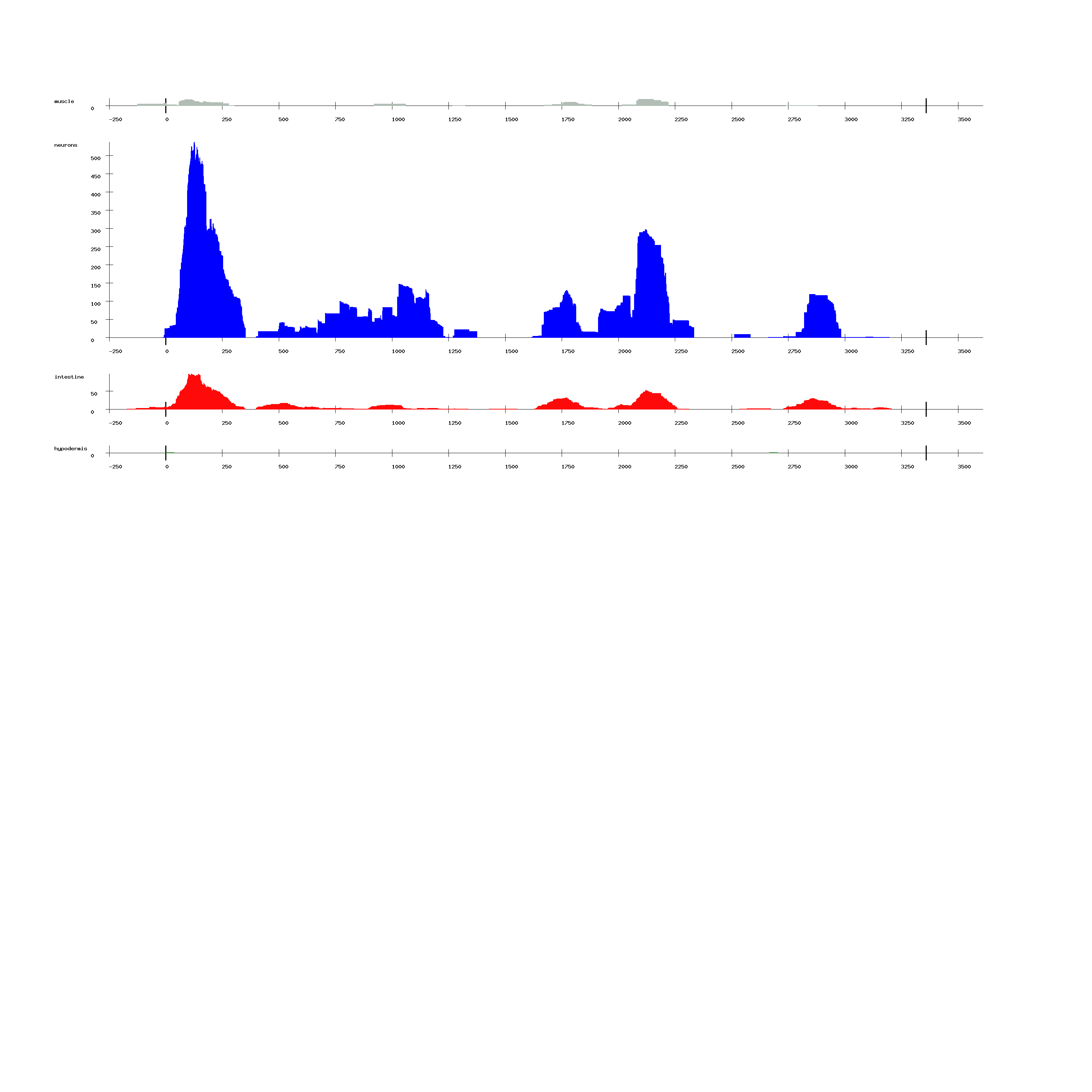

Supplement: Supplementary file 1 [file ijms-24-02970-s001.zip › Supplementary Data S2/3.1299759-1303117.png]

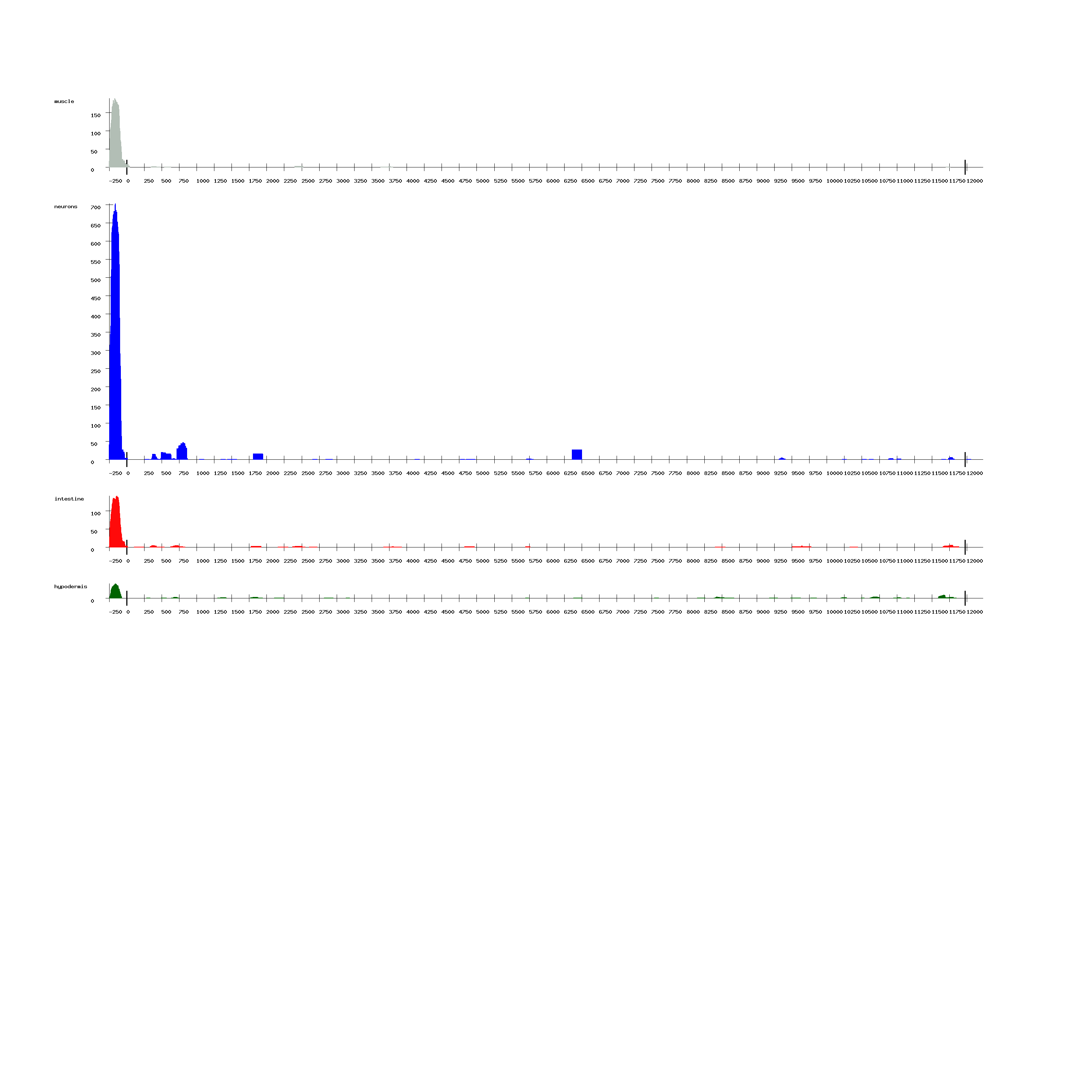

Supplement: Supplementary file 1 [file ijms-24-02970-s001.zip › Supplementary Data S2/3.13074772-13086747.png]

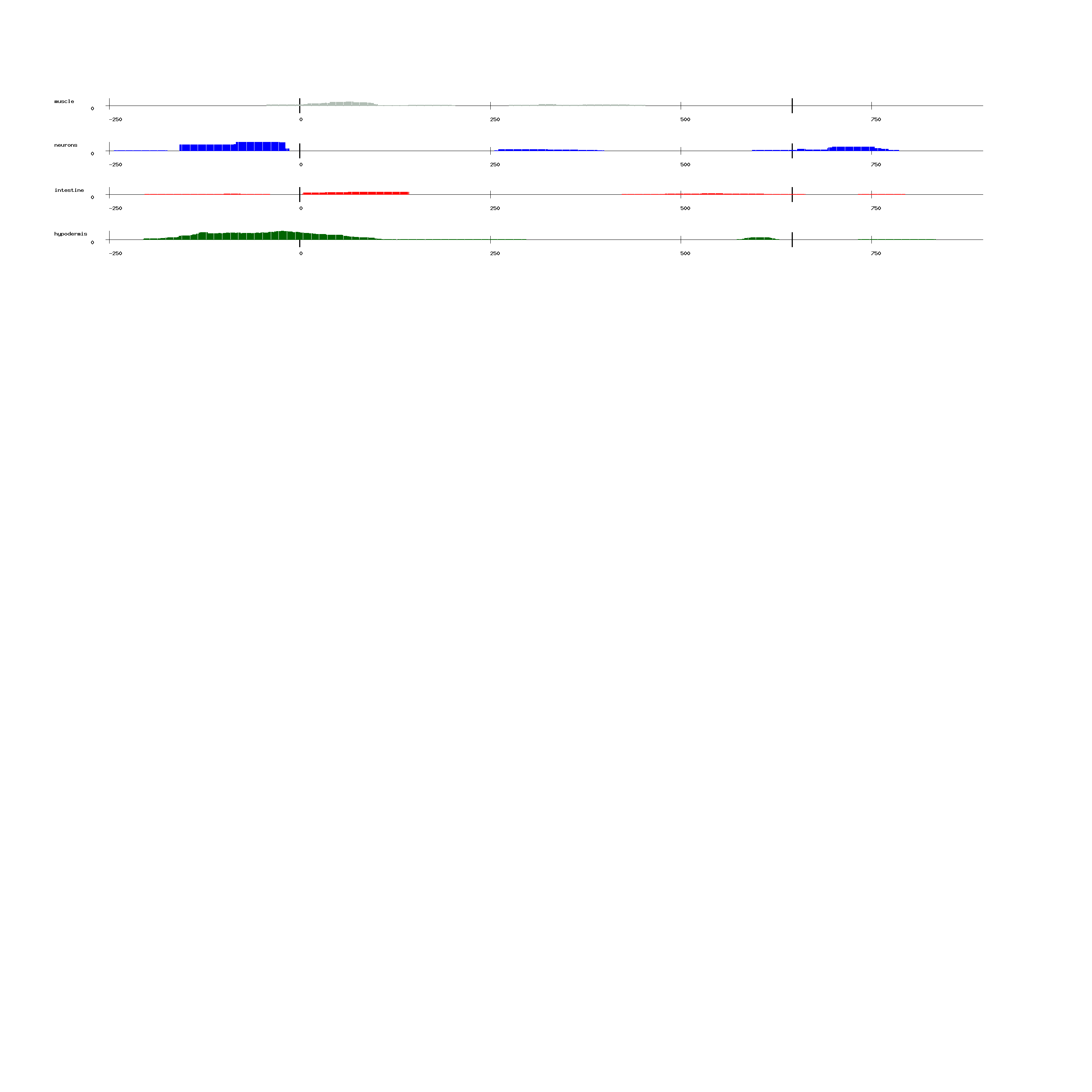

Supplement: Supplementary file 1 [file ijms-24-02970-s001.zip › Supplementary Data S2/3.13099929-13100574.png]

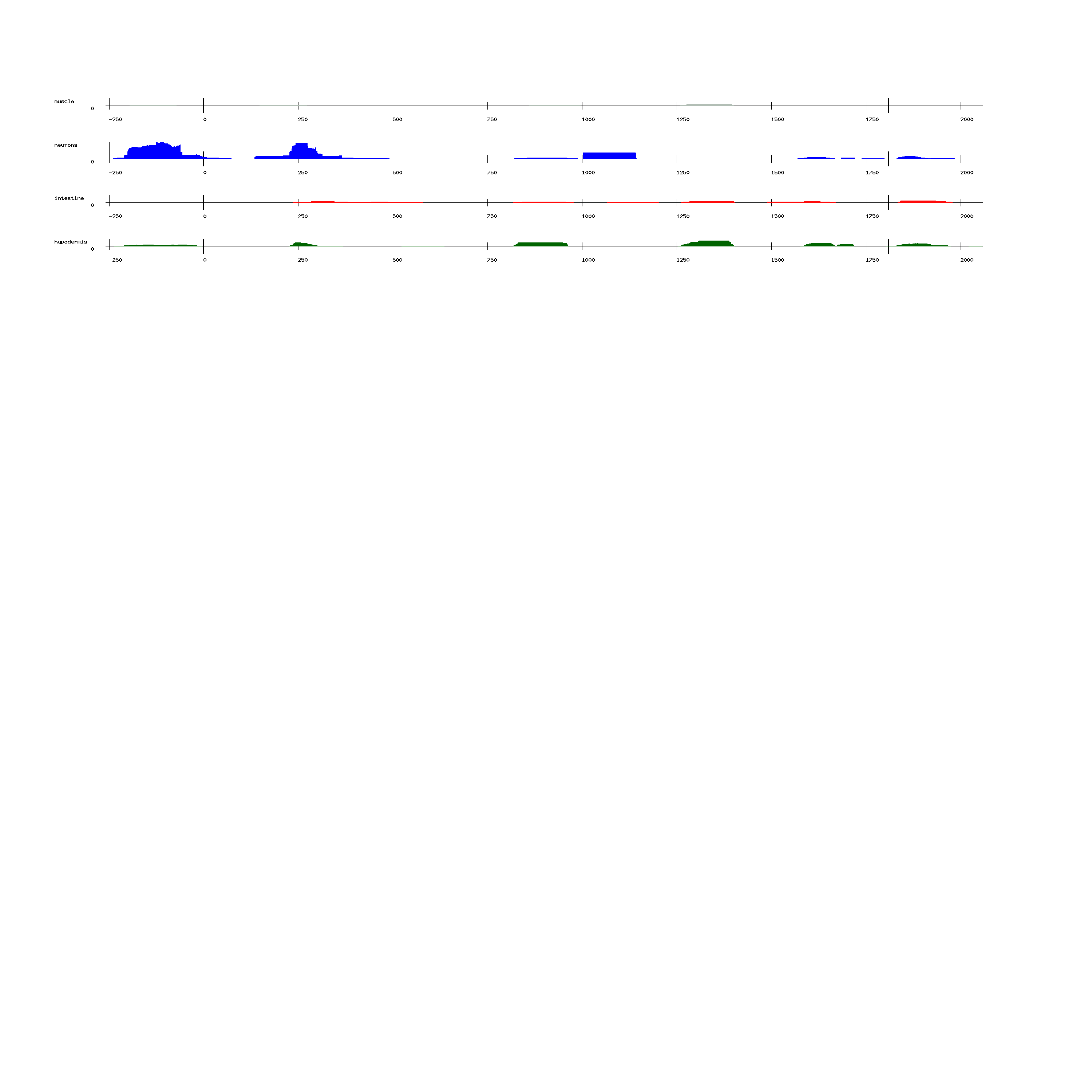

Supplement: Supplementary file 1 [file ijms-24-02970-s001.zip › Supplementary Data S2/3.13243432-13245239.png]
