# Supplementary figures and images for: Unique Features of Satellite DNA Transcription in Different Tissues of Caenorhabditis elegans (part 1 of 2)
Source: Int J Mol Sci. 2023 Feb 3;24(3):2970. doi: 10.3390/ijms24032970 (PMC9918286; doi:10.3390/ijms24032970)

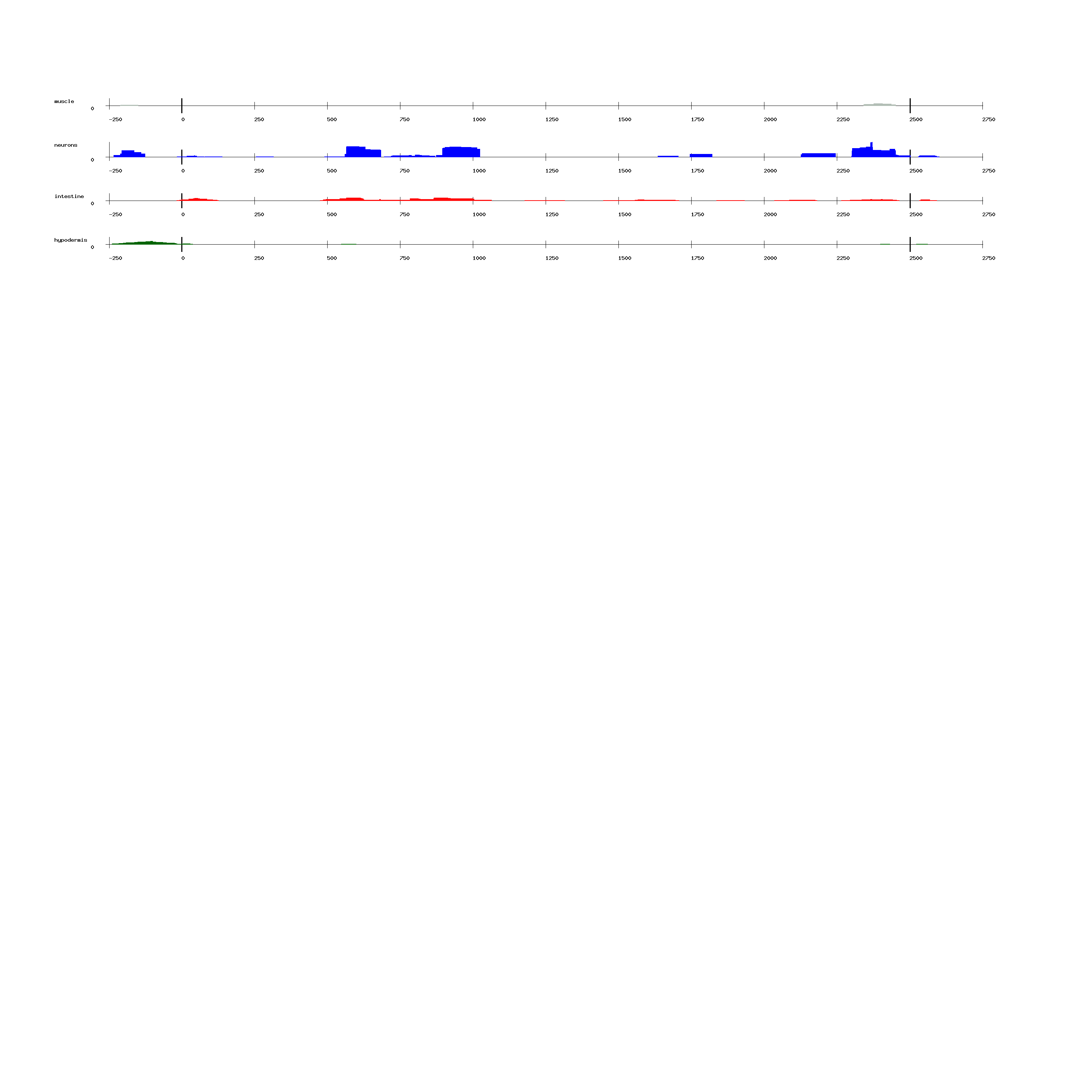

Supplement: Supplementary file 1 [file ijms-24-02970-s001.zip › Supplementary Data S2/1.10130602-10133102.png]

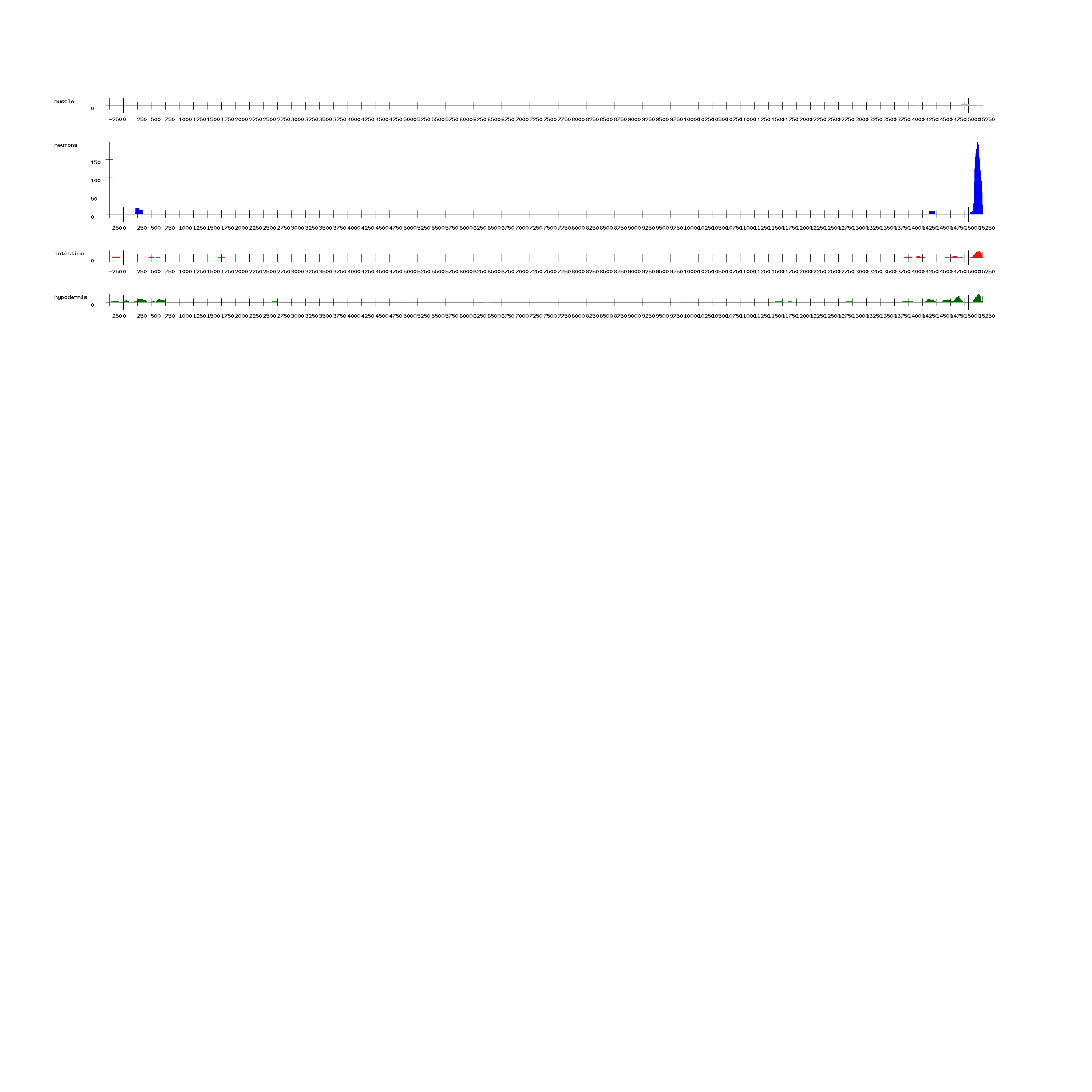

Supplement: Supplementary file 1 [file ijms-24-02970-s001.zip › Supplementary Data S2/1.10204129-10219199.png]

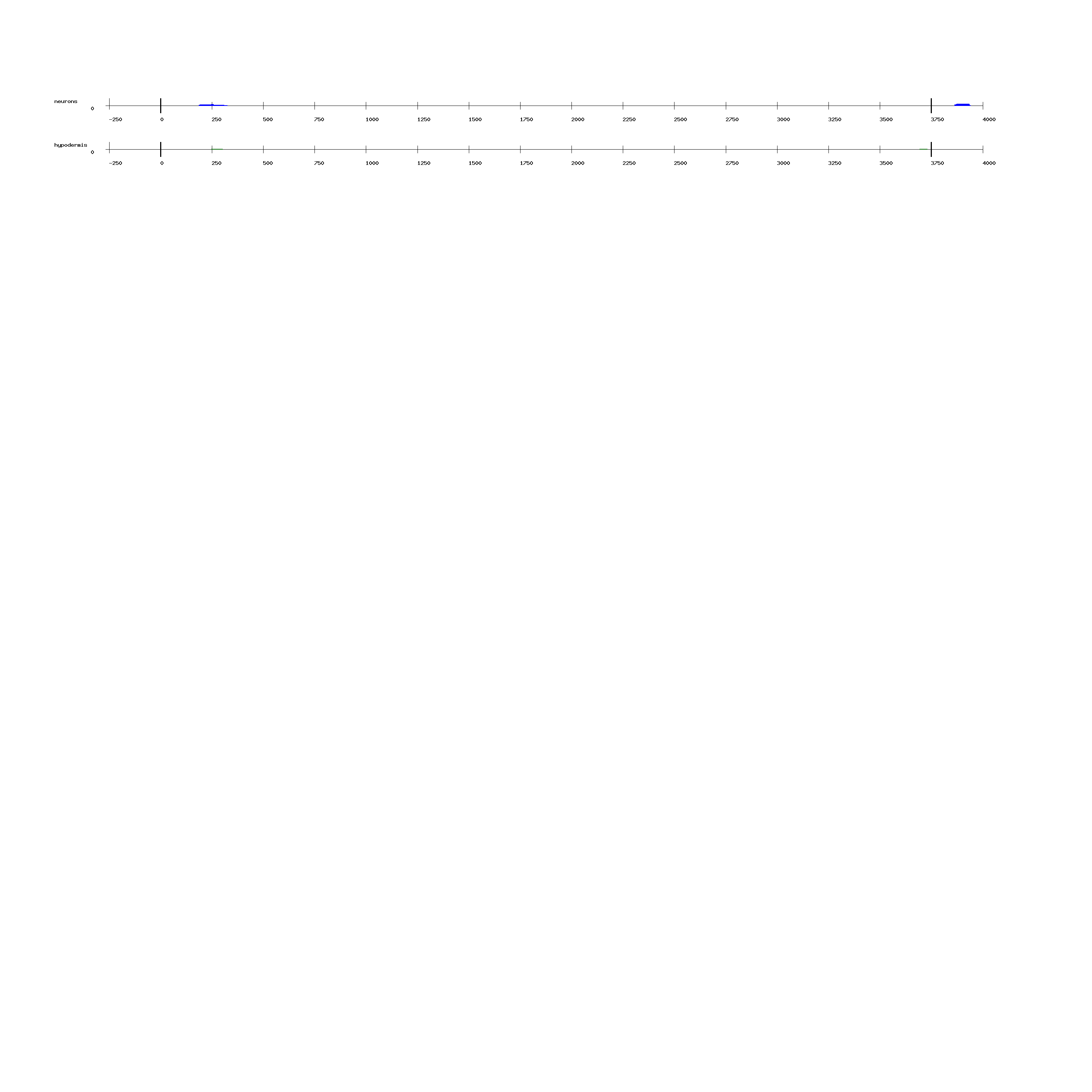

Supplement: Supplementary file 1 [file ijms-24-02970-s001.zip › Supplementary Data S2/1.10851626-10855375.png]

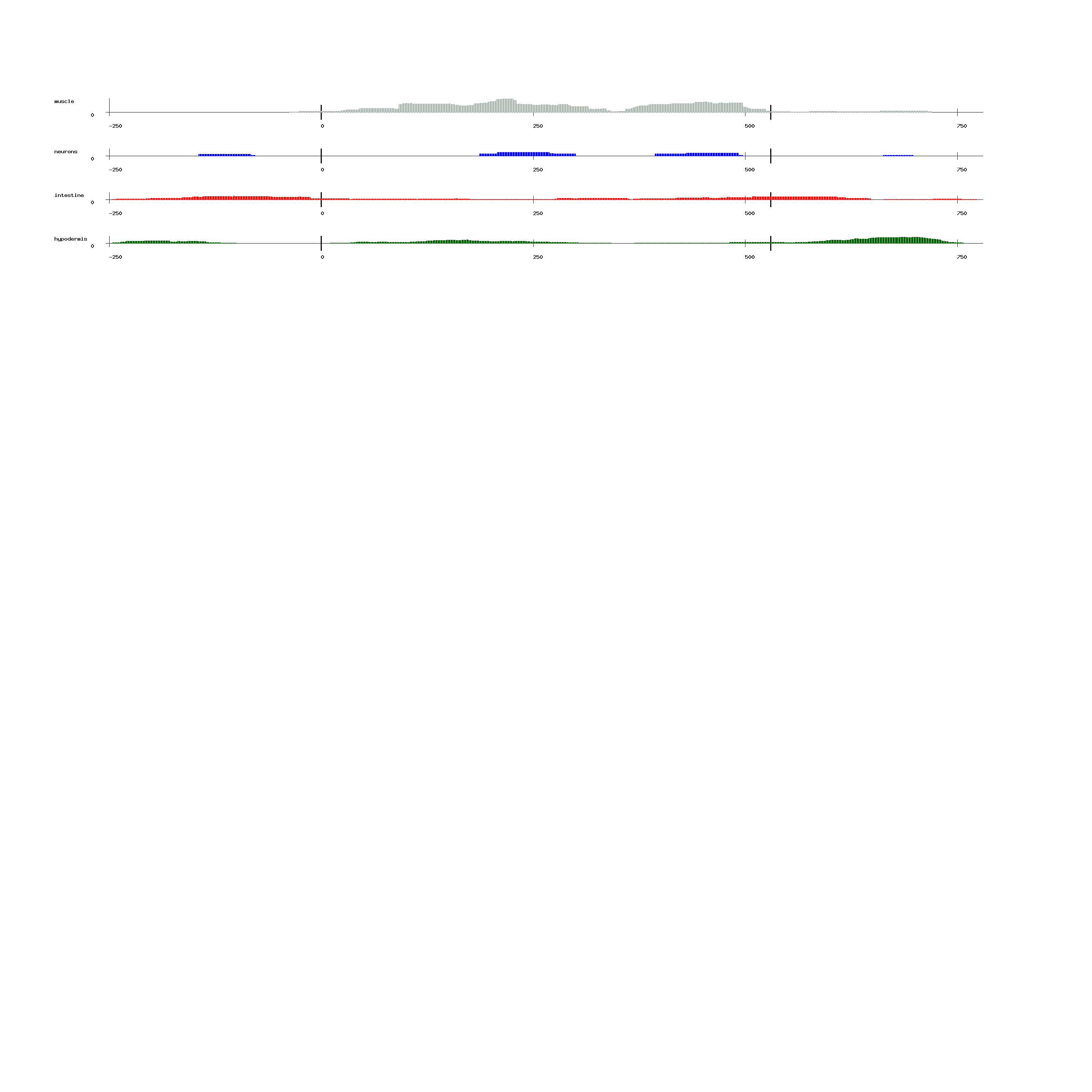

Supplement: Supplementary file 1 [file ijms-24-02970-s001.zip › Supplementary Data S2/1.1097742-1098271.png]

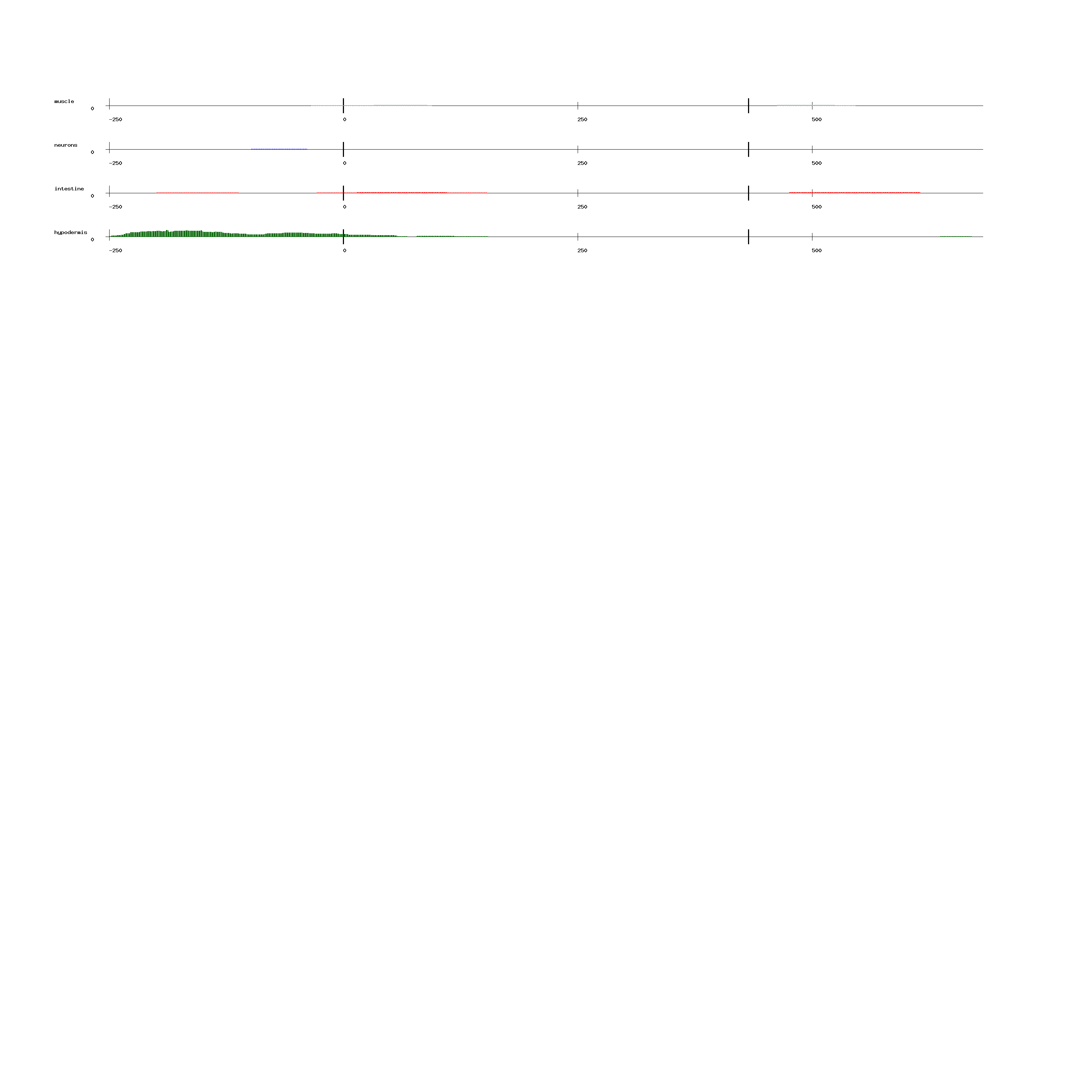

Supplement: Supplementary file 1 [file ijms-24-02970-s001.zip › Supplementary Data S2/1.11061949-11062380.png]

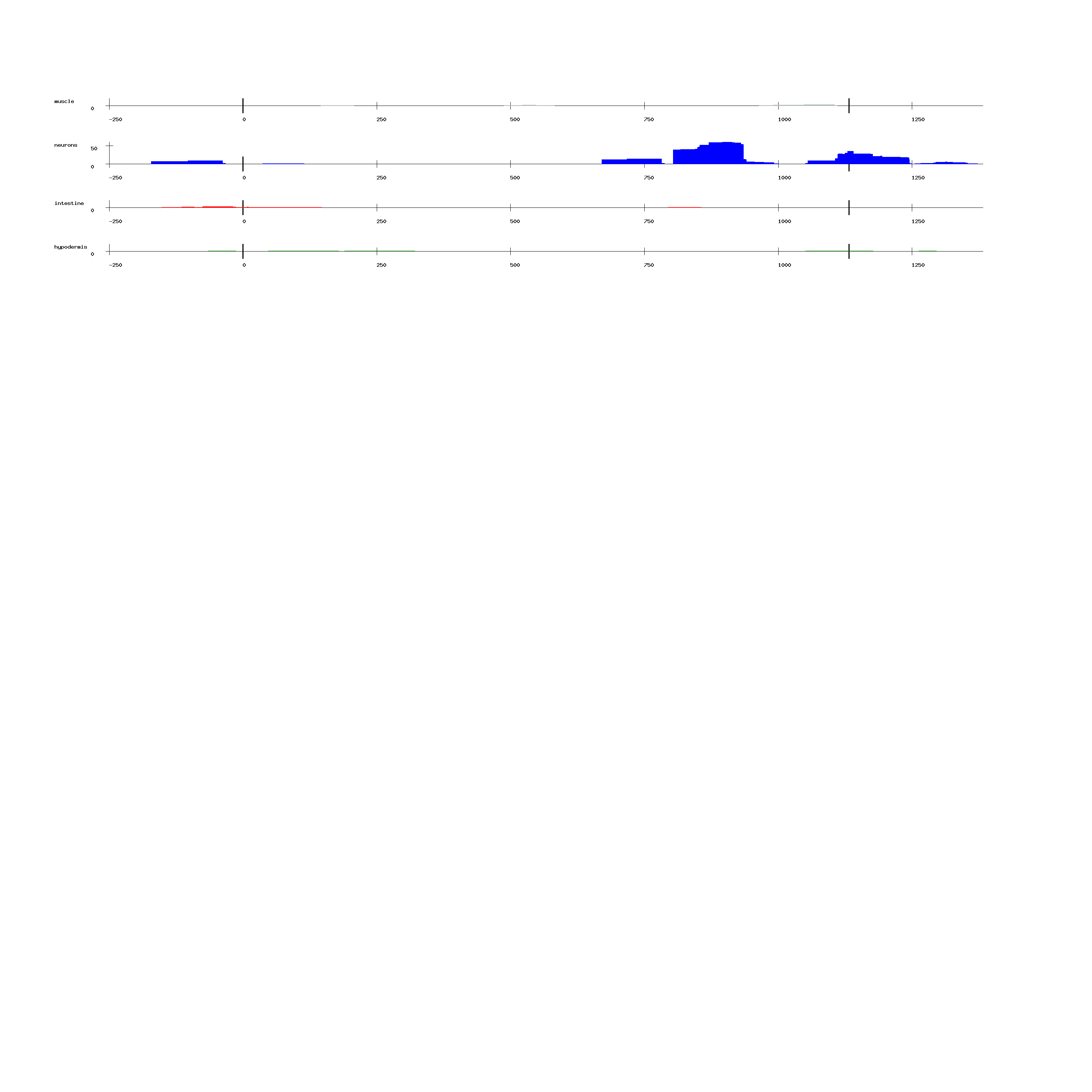

Supplement: Supplementary file 1 [file ijms-24-02970-s001.zip › Supplementary Data S2/1.11186372-11187503.png]

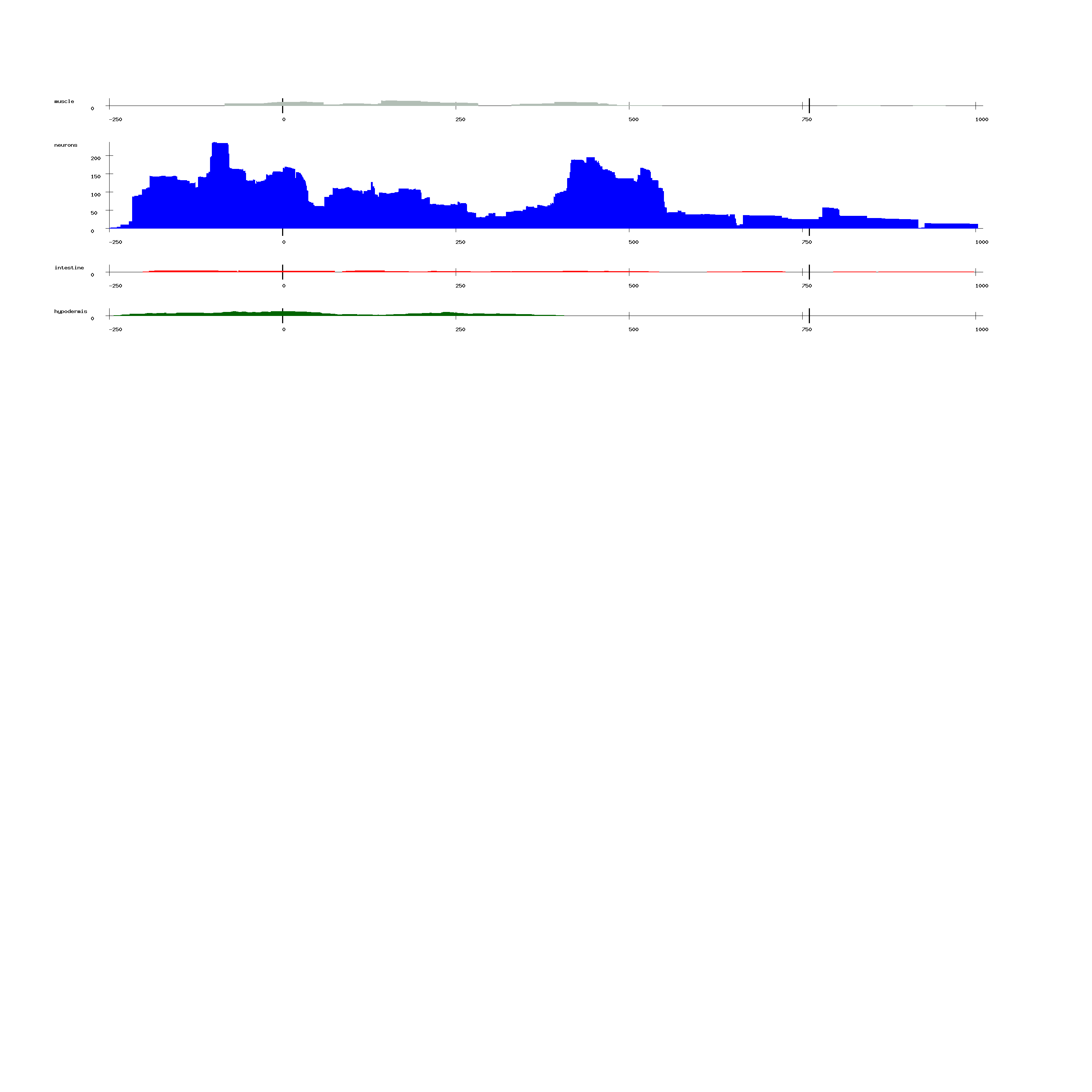

Supplement: Supplementary file 1 [file ijms-24-02970-s001.zip › Supplementary Data S2/1.11190690-11191449.png]

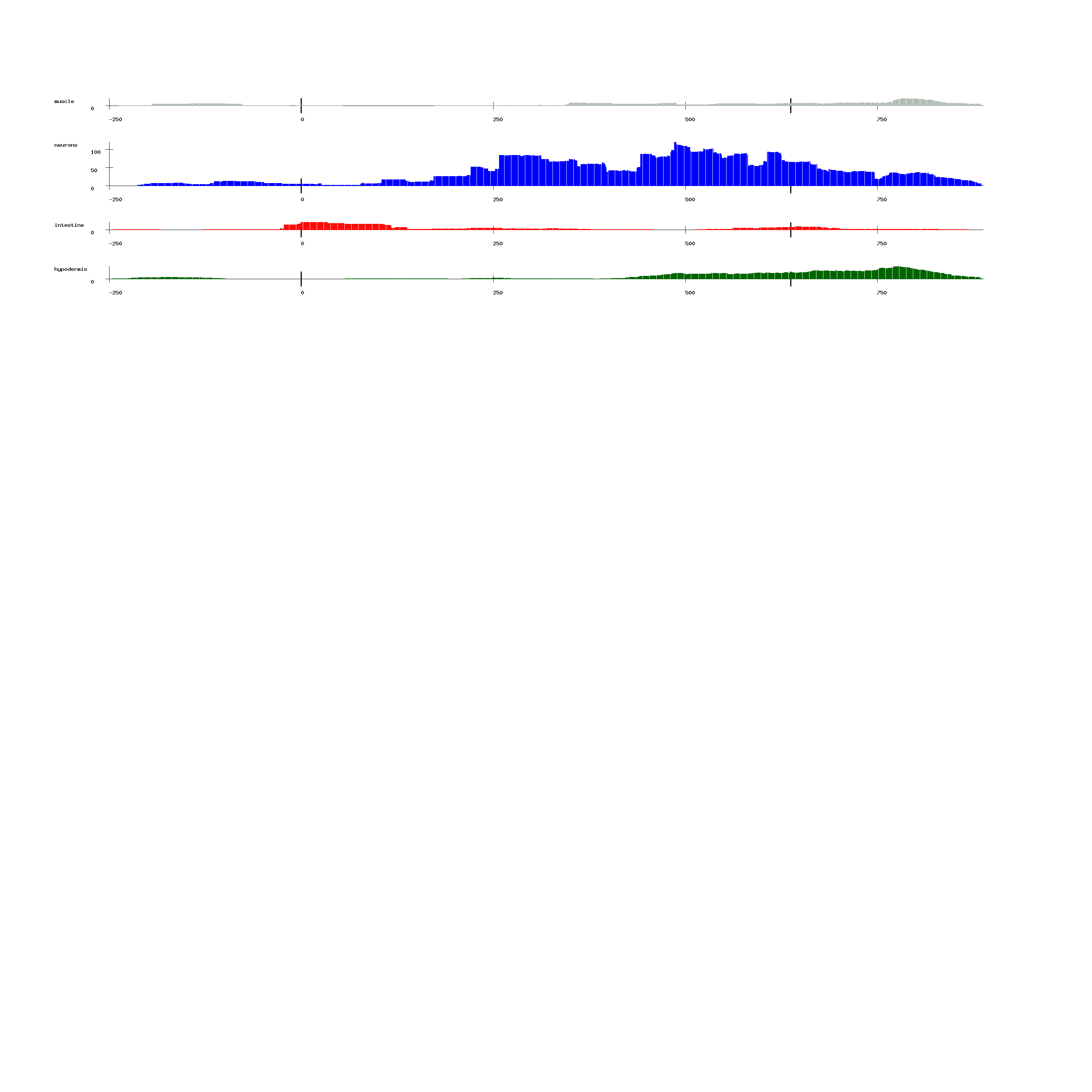

Supplement: Supplementary file 1 [file ijms-24-02970-s001.zip › Supplementary Data S2/1.11201002-11201638.png]

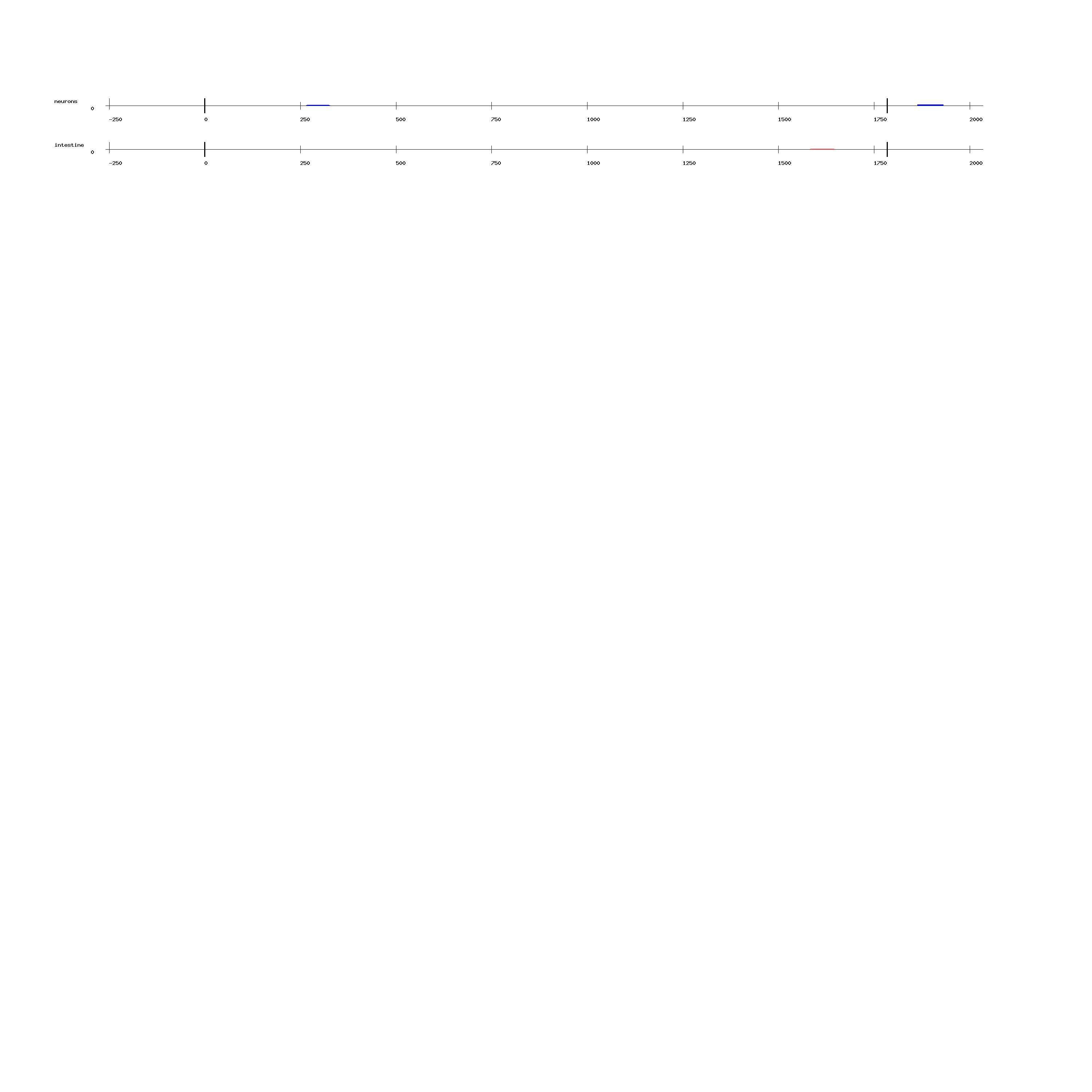

Supplement: Supplementary file 1 [file ijms-24-02970-s001.zip › Supplementary Data S2/1.11349013-11350796.png]

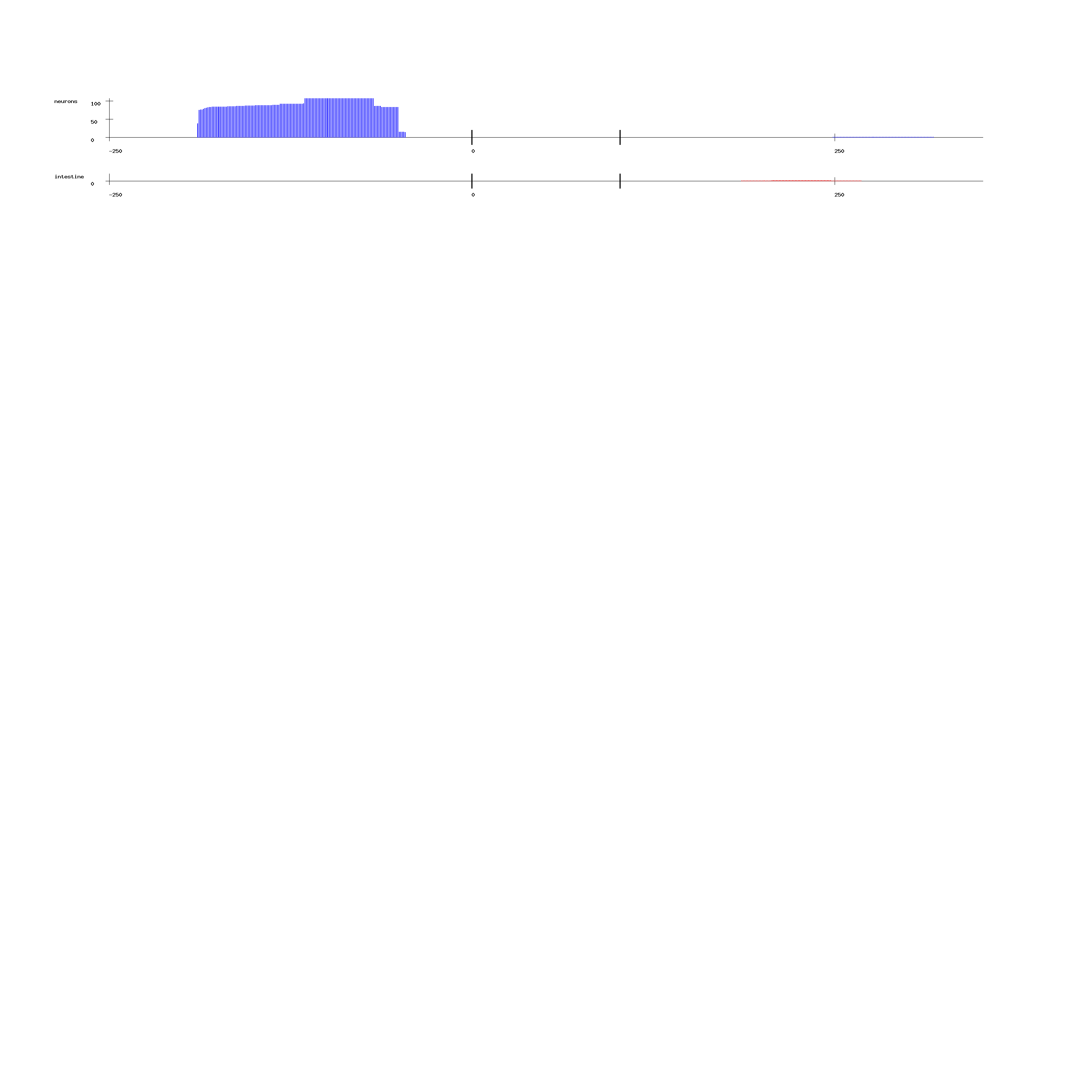

Supplement: Supplementary file 1 [file ijms-24-02970-s001.zip › Supplementary Data S2/1.11357272-11357373.png]

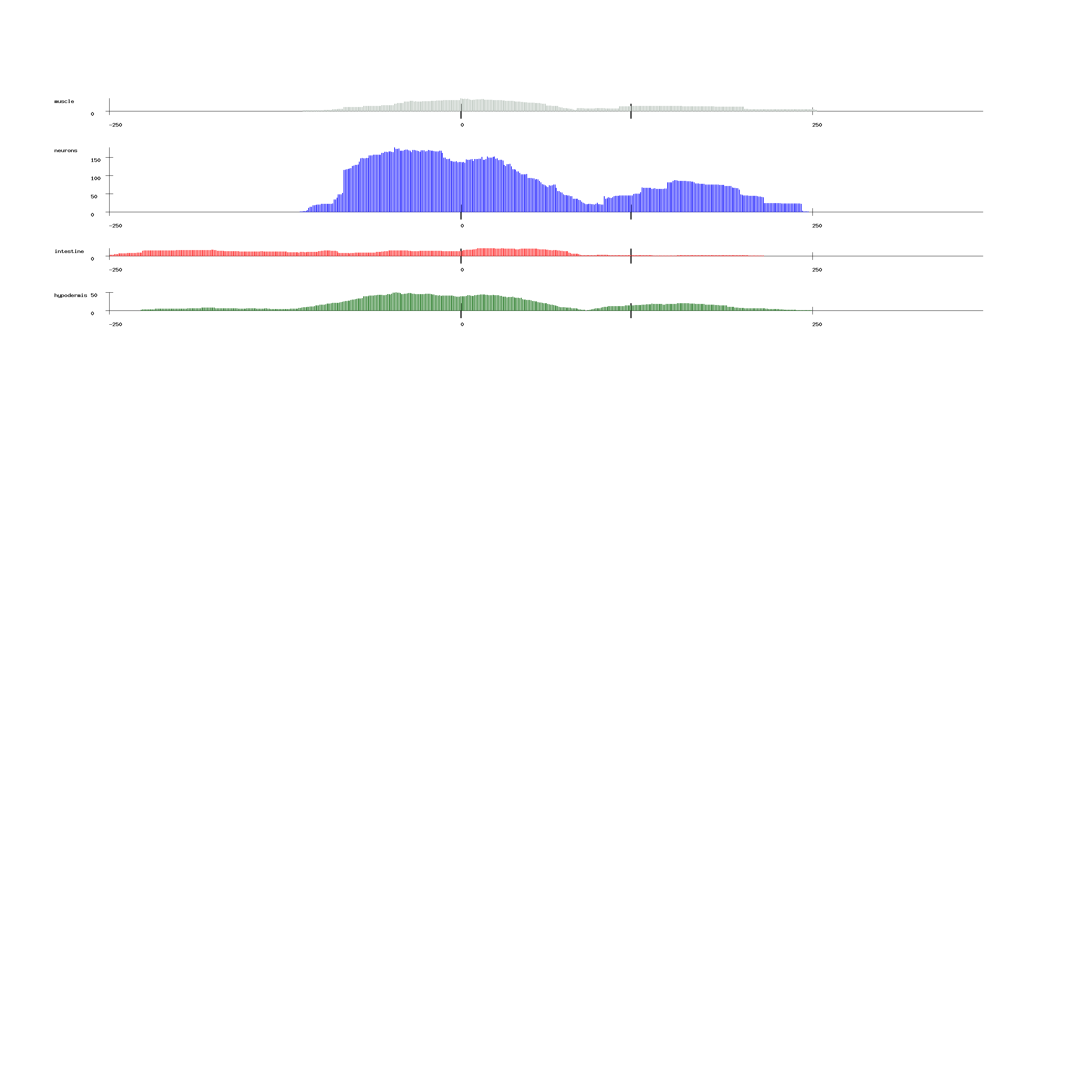

Supplement: Supplementary file 1 [file ijms-24-02970-s001.zip › Supplementary Data S2/1.11480157-11480277.png]

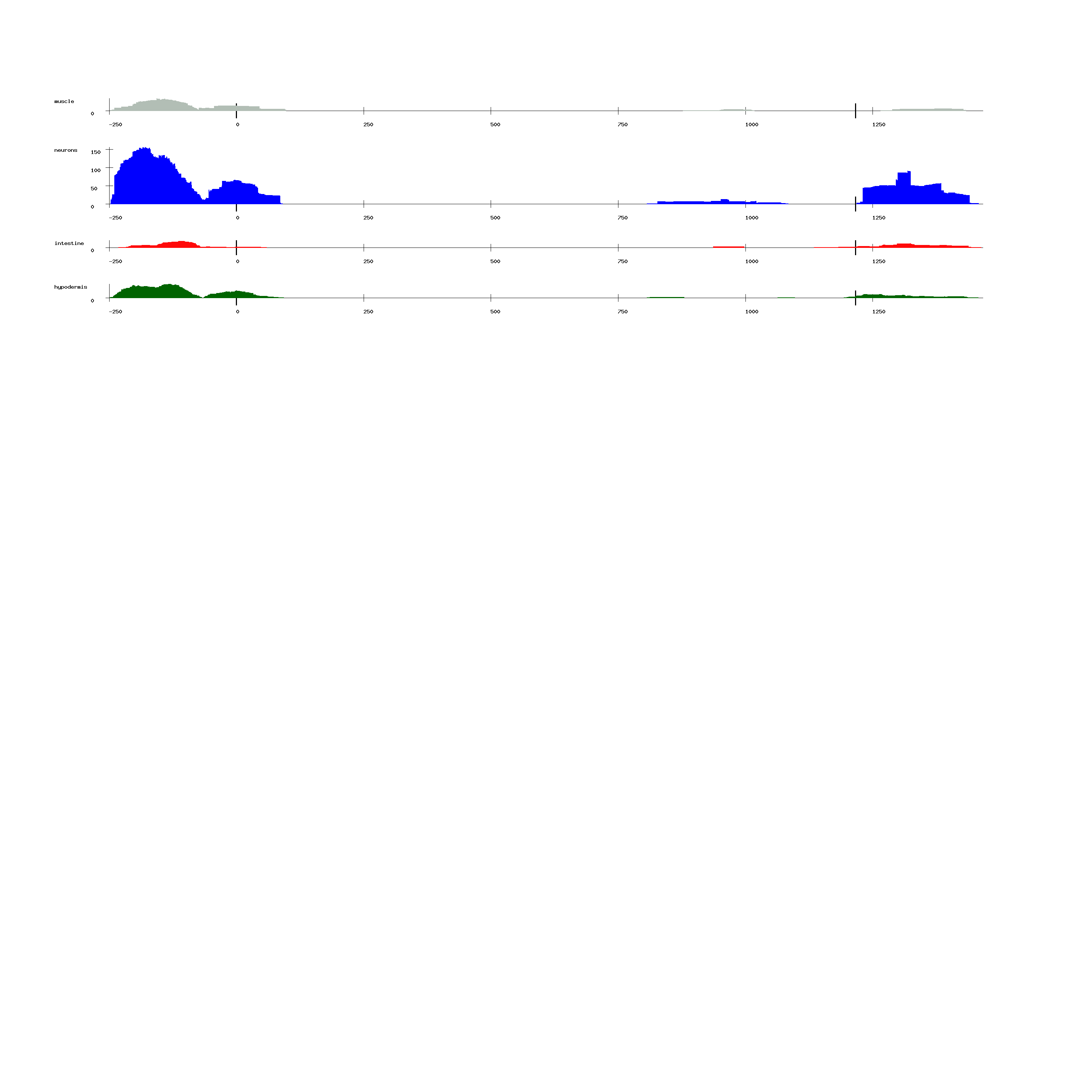

Supplement: Supplementary file 1 [file ijms-24-02970-s001.zip › Supplementary Data S2/1.11480313-11481528.png]

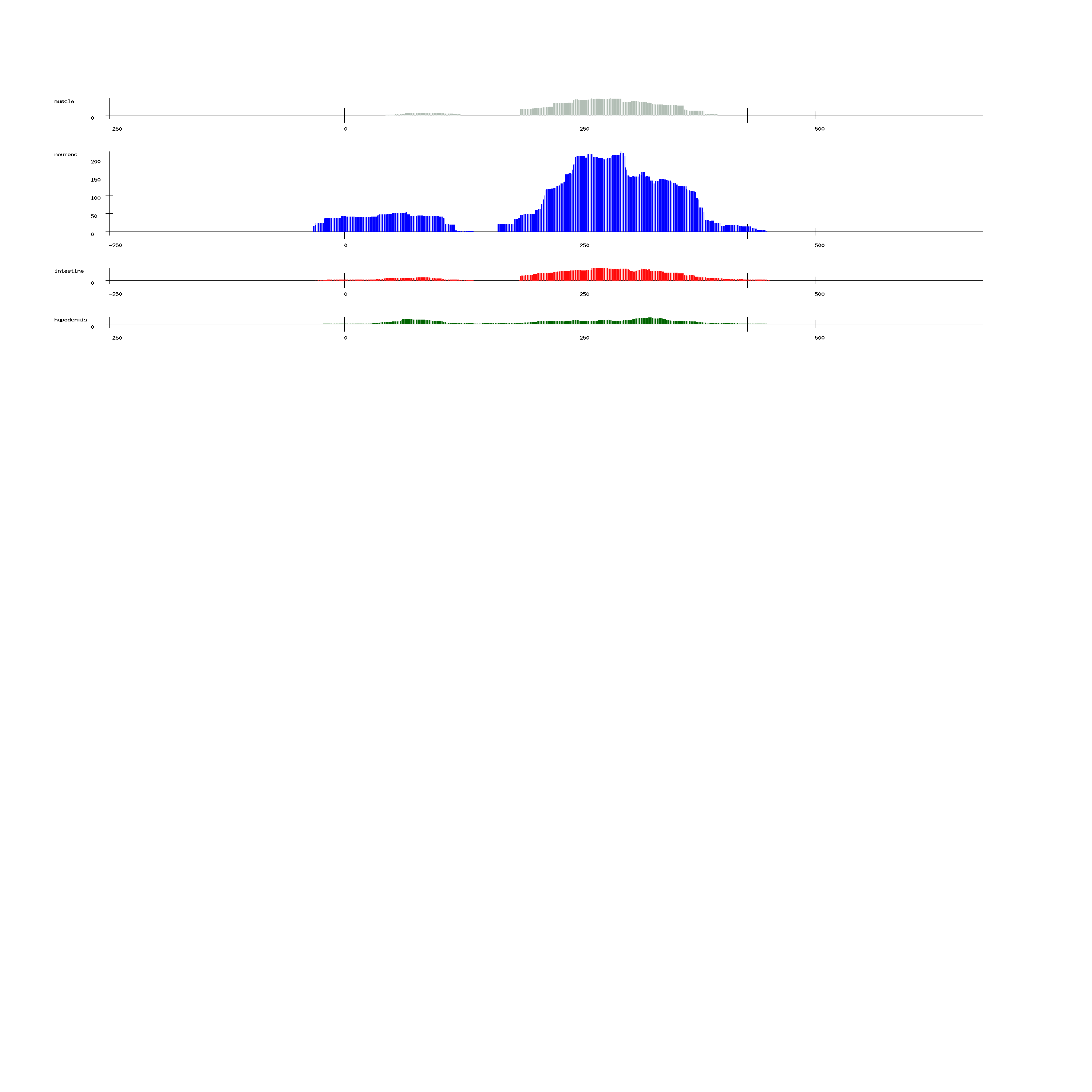

Supplement: Supplementary file 1 [file ijms-24-02970-s001.zip › Supplementary Data S2/1.11829562-11829989.png]

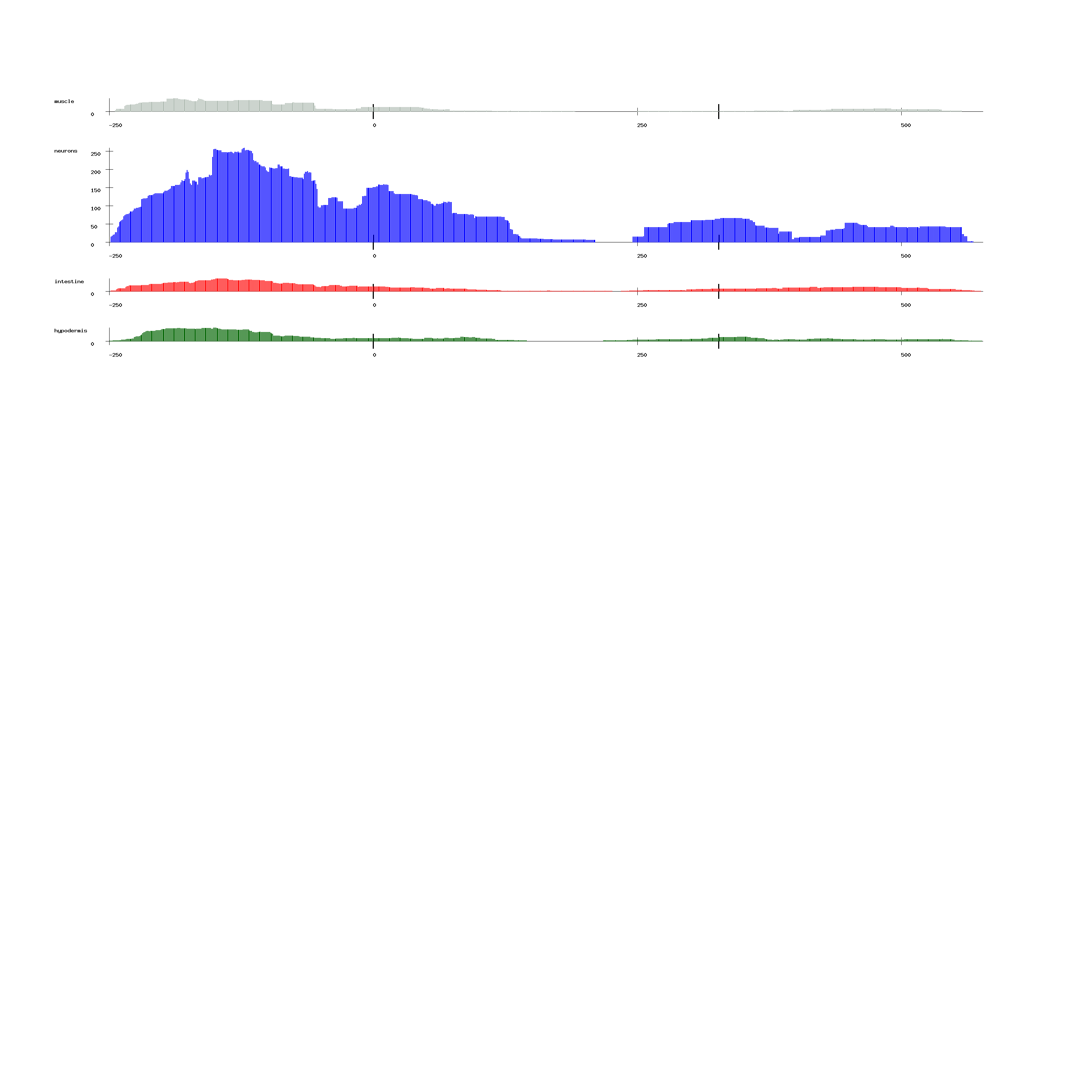

Supplement: Supplementary file 1 [file ijms-24-02970-s001.zip › Supplementary Data S2/1.12192426-12192752.png]

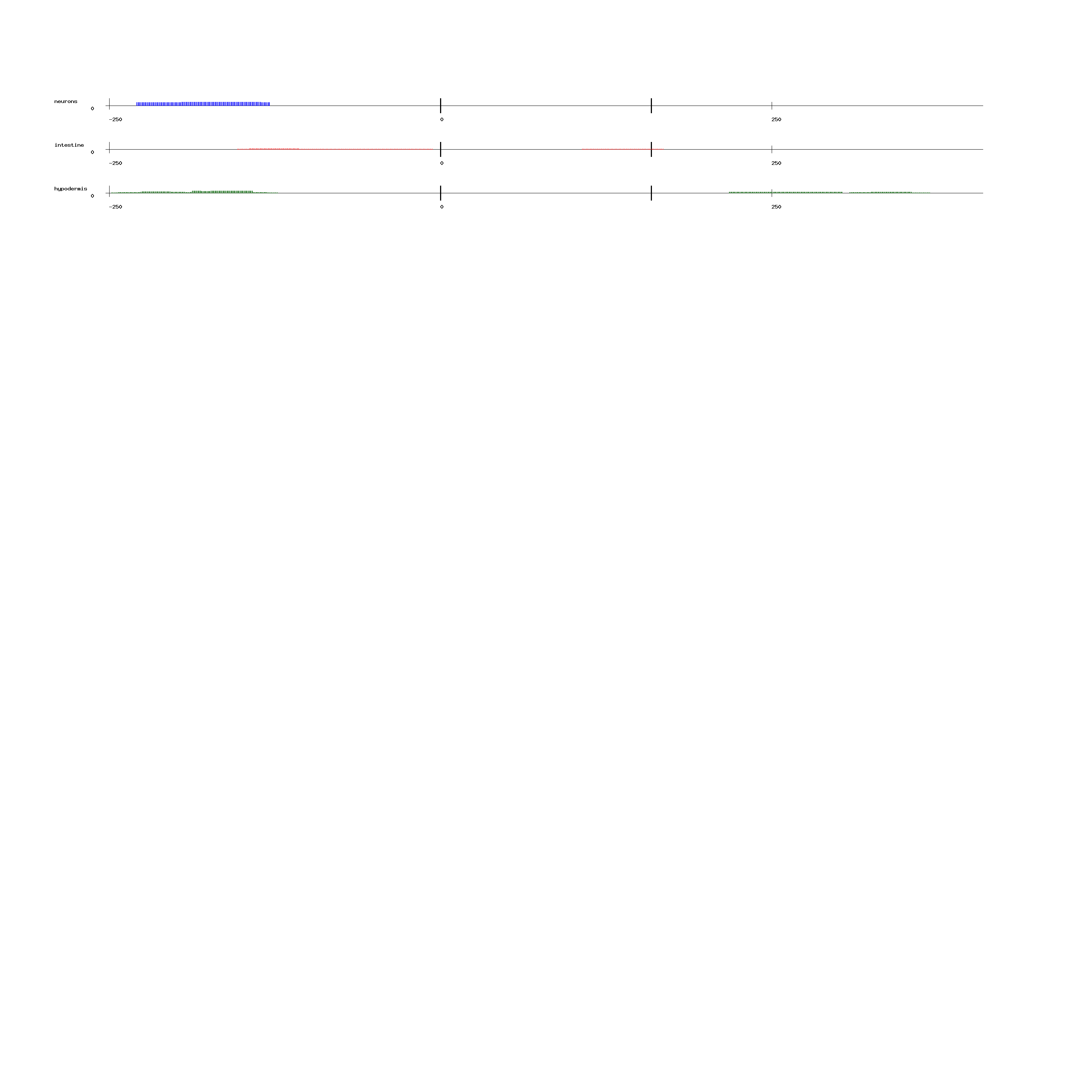

Supplement: Supplementary file 1 [file ijms-24-02970-s001.zip › Supplementary Data S2/1.12258128-12258286.png]

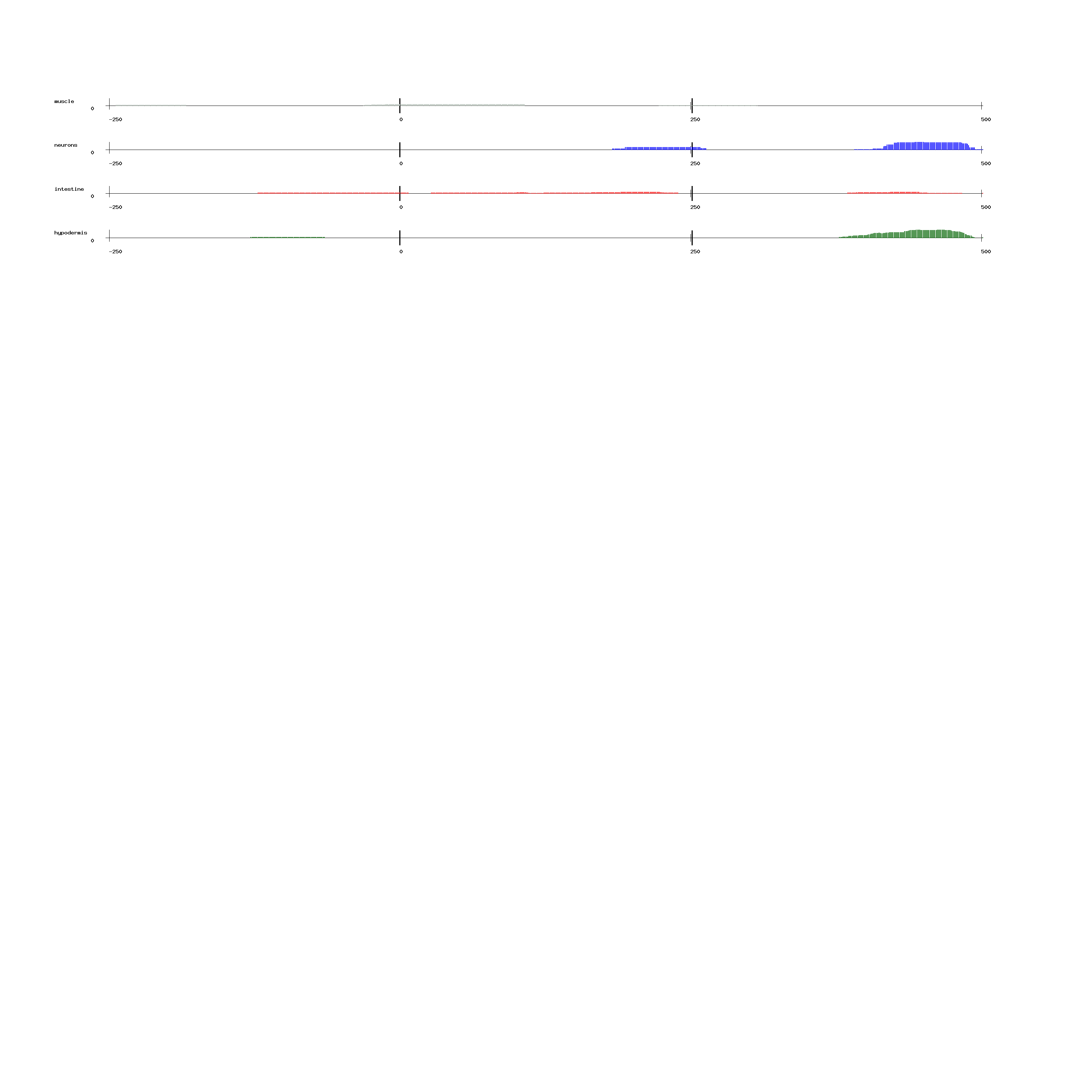

Supplement: Supplementary file 1 [file ijms-24-02970-s001.zip › Supplementary Data S2/1.12480947-12481197.png]

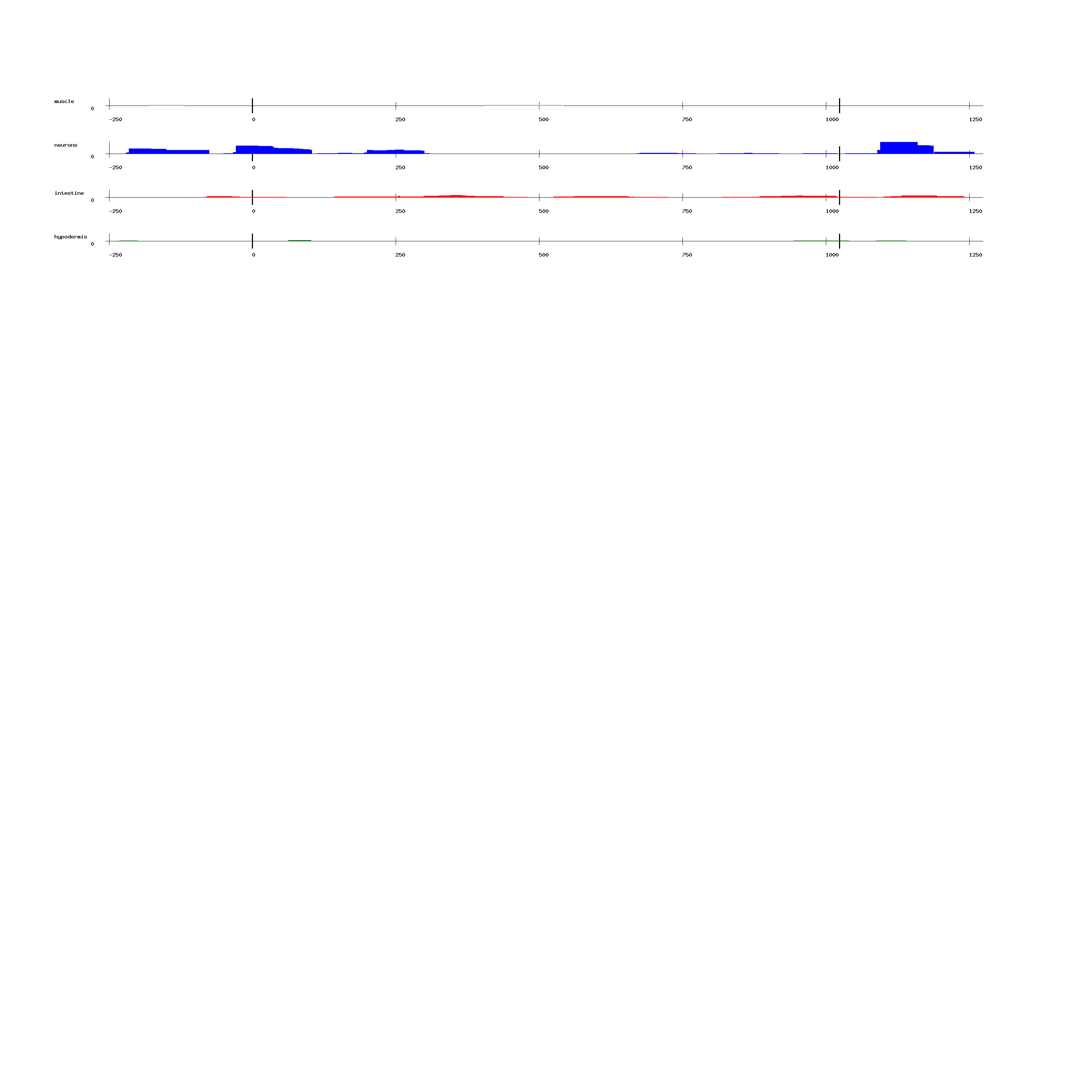

Supplement: Supplementary file 1 [file ijms-24-02970-s001.zip › Supplementary Data S2/1.1278237-1279259.png]

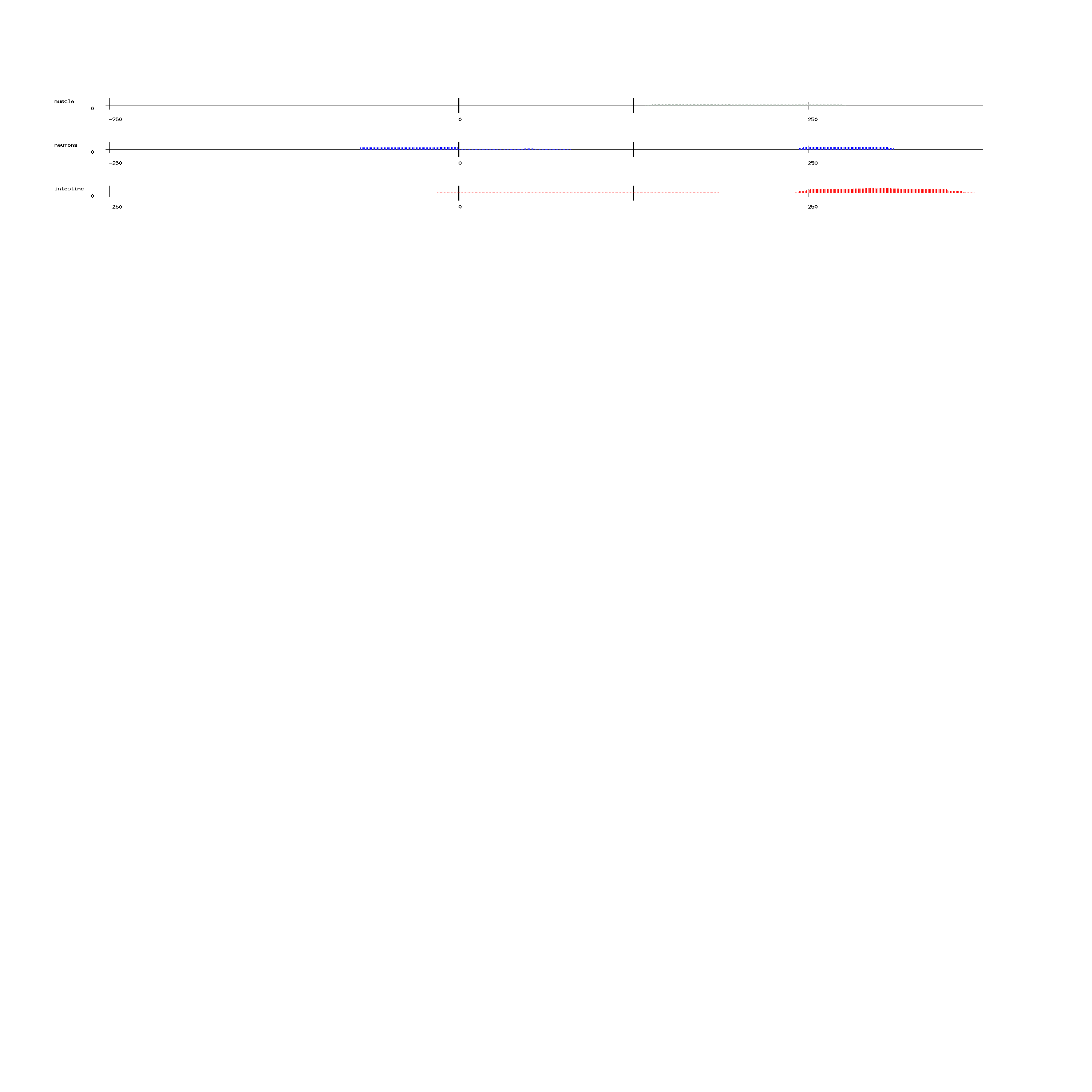

Supplement: Supplementary file 1 [file ijms-24-02970-s001.zip › Supplementary Data S2/1.12910137-12910261.png]

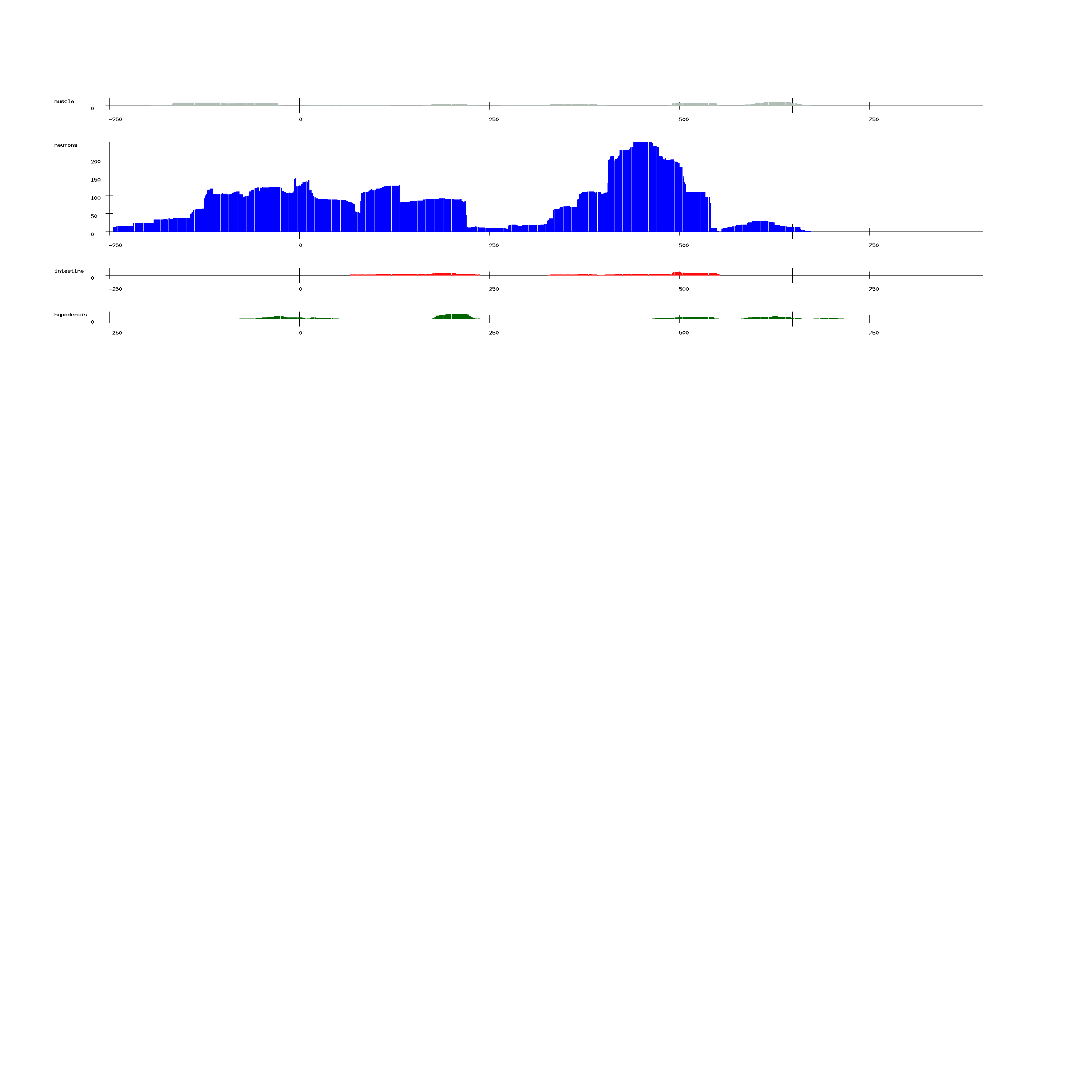

Supplement: Supplementary file 1 [file ijms-24-02970-s001.zip › Supplementary Data S2/1.13231021-13231669.png]

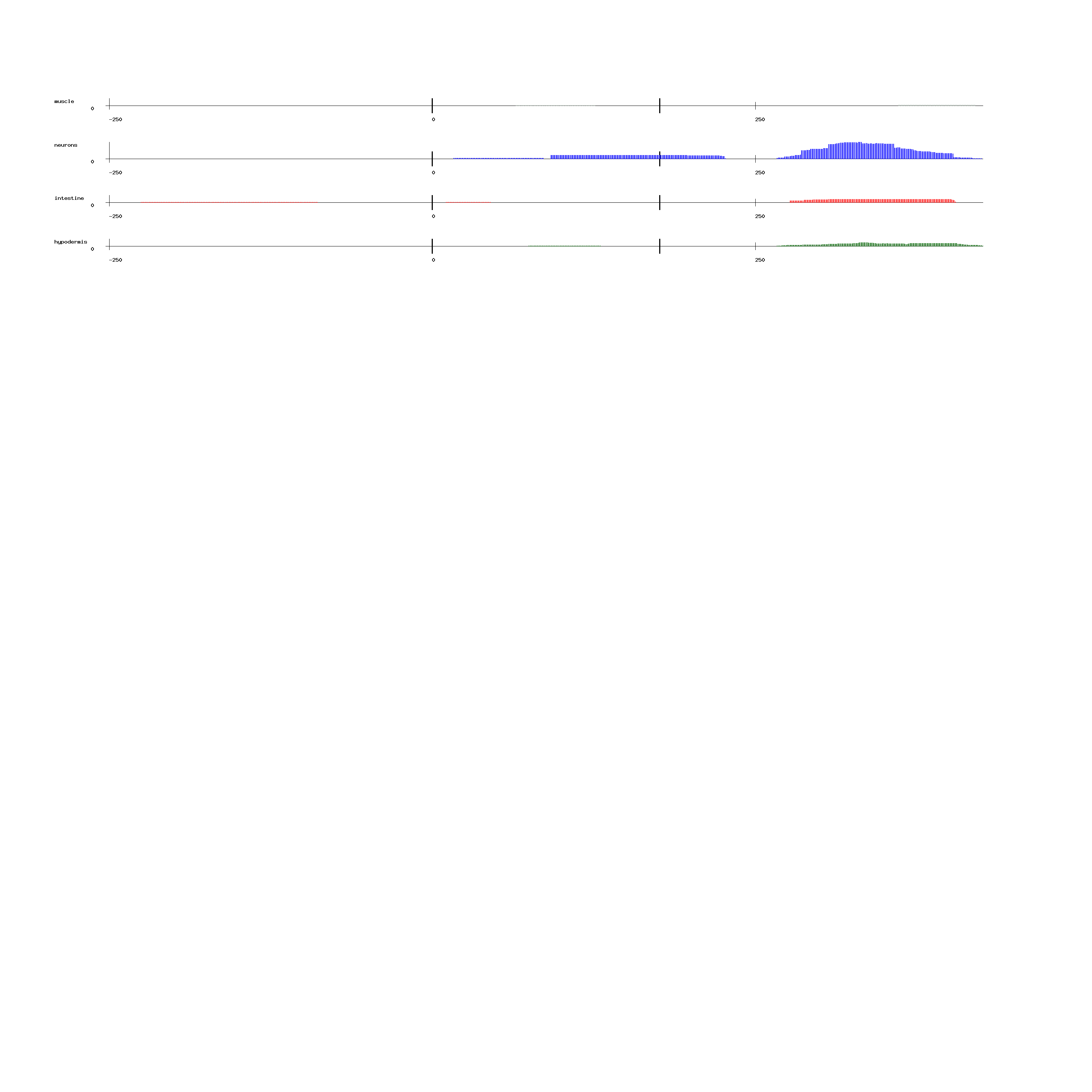

Supplement: Supplementary file 1 [file ijms-24-02970-s001.zip › Supplementary Data S2/1.13453071-13453246.png]

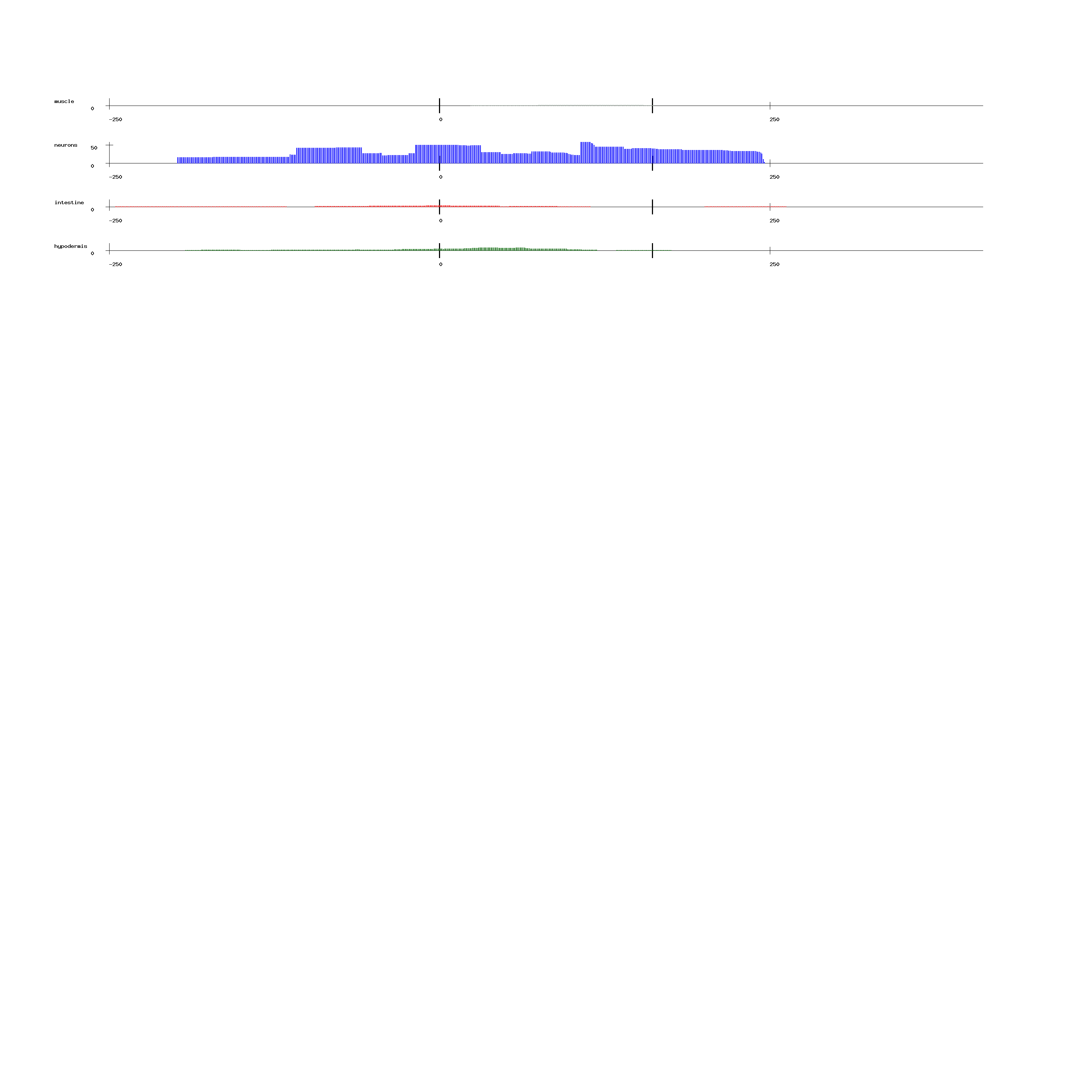

Supplement: Supplementary file 1 [file ijms-24-02970-s001.zip › Supplementary Data S2/1.1362690-1362850.png]

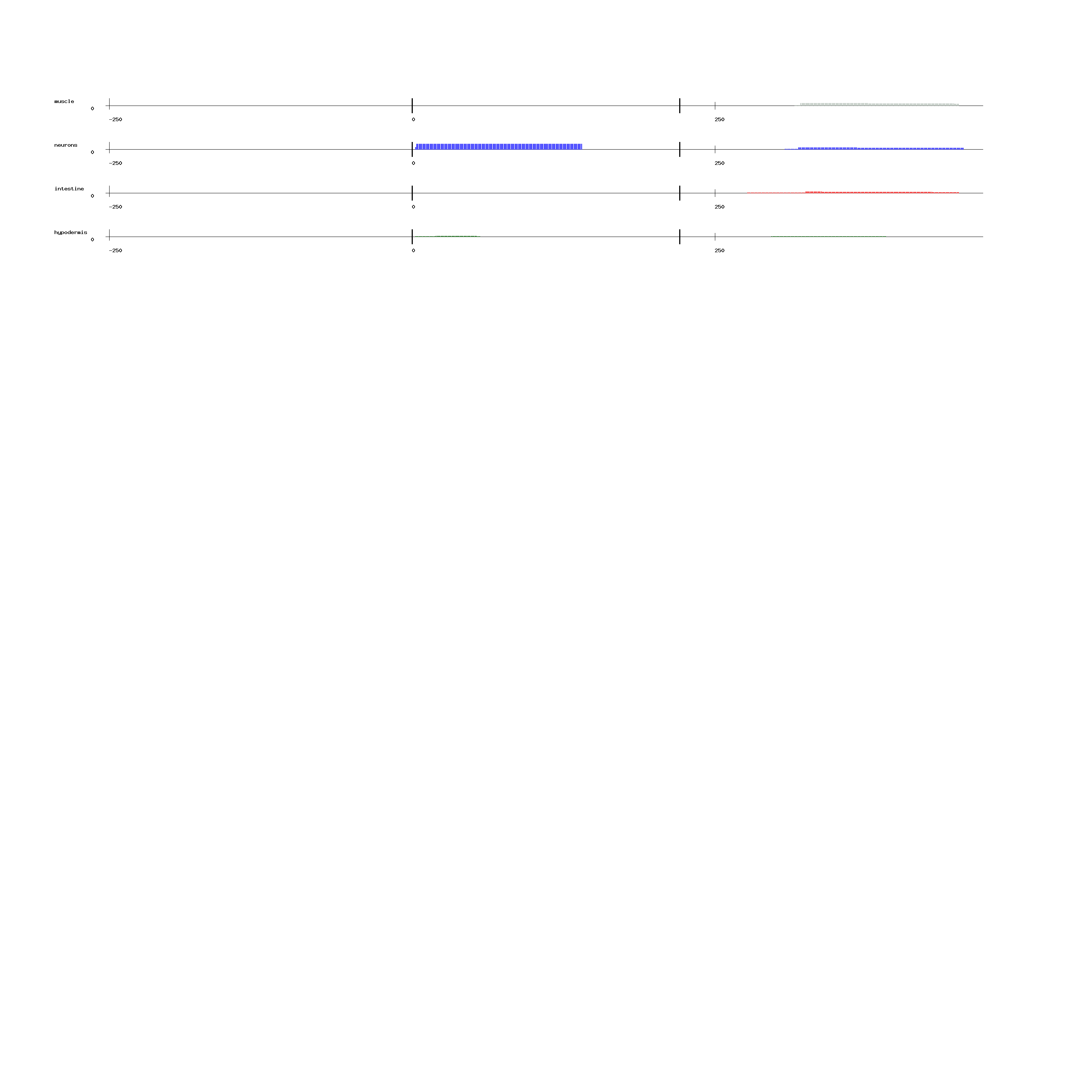

Supplement: Supplementary file 1 [file ijms-24-02970-s001.zip › Supplementary Data S2/1.13929375-13929595.png]

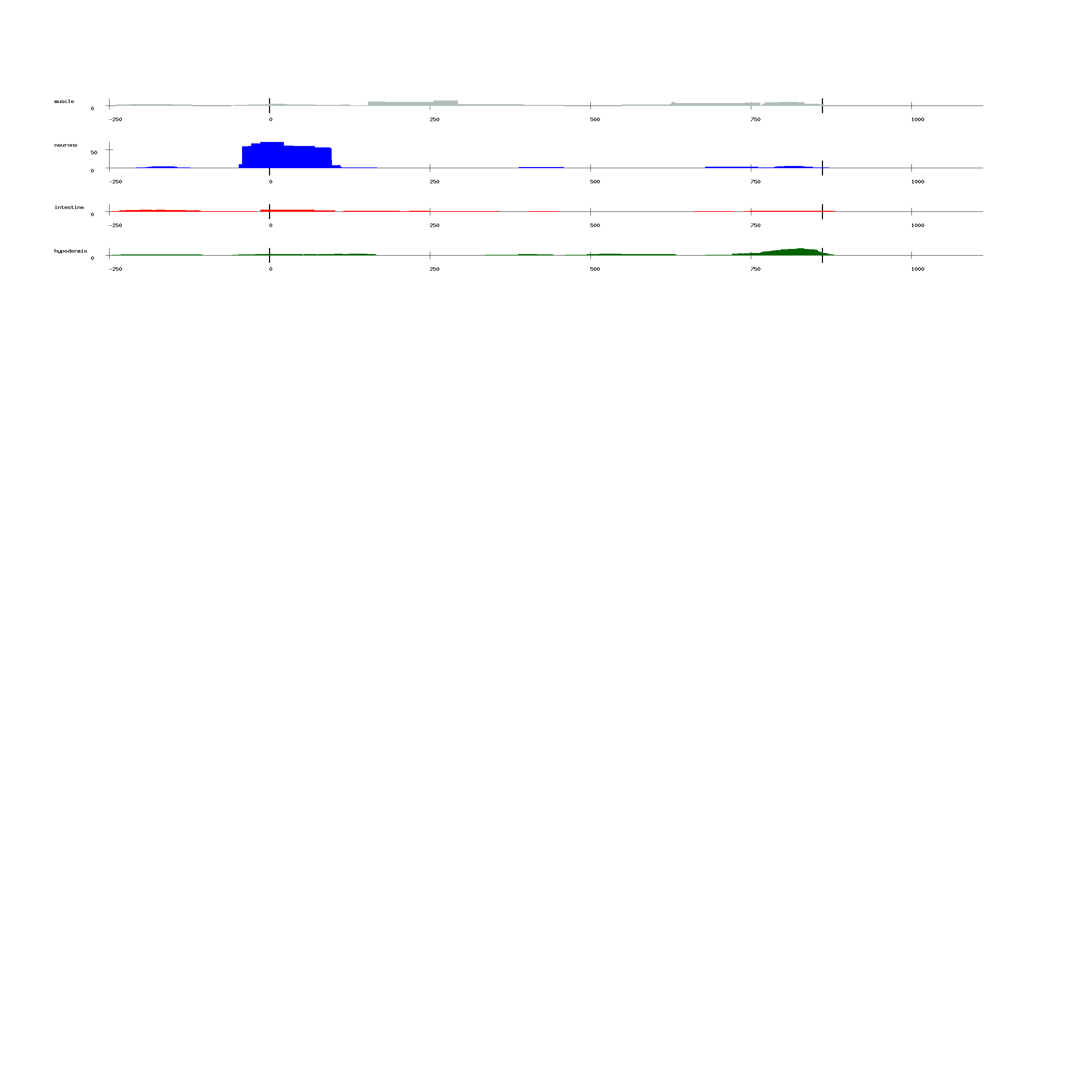

Supplement: Supplementary file 1 [file ijms-24-02970-s001.zip › Supplementary Data S2/1.13935628-13936488.png]

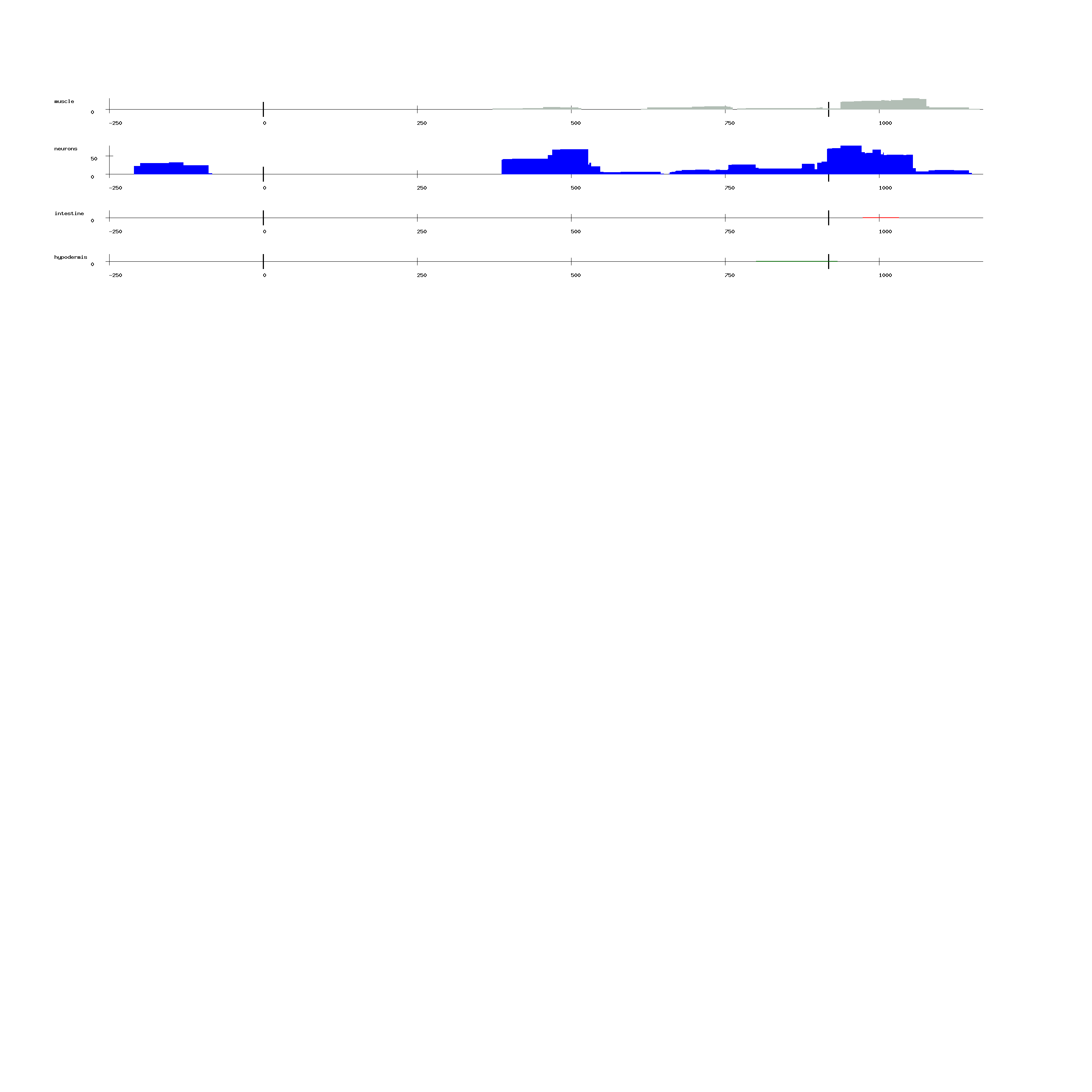

Supplement: Supplementary file 1 [file ijms-24-02970-s001.zip › Supplementary Data S2/1.14024312-14025229.png]

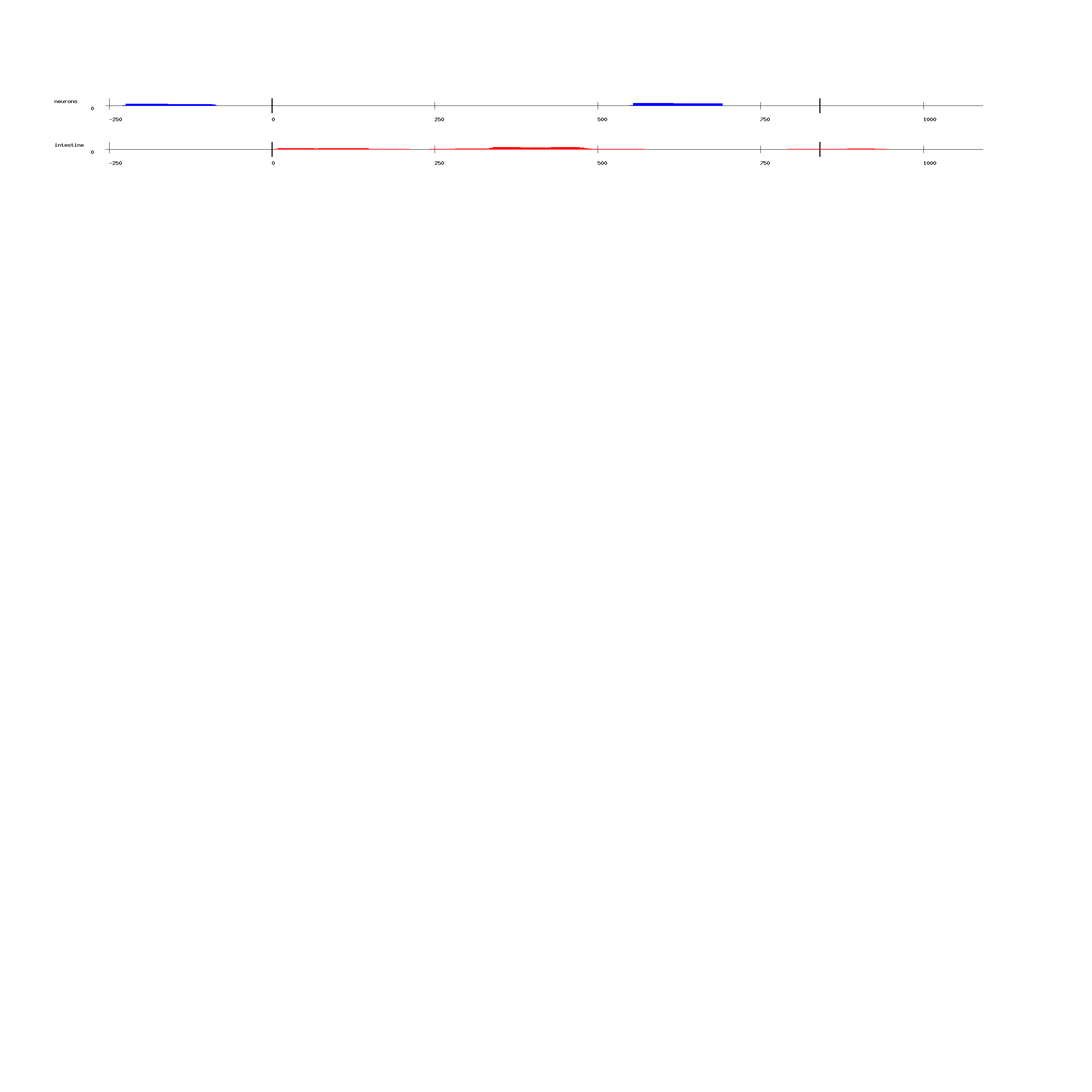

Supplement: Supplementary file 1 [file ijms-24-02970-s001.zip › Supplementary Data S2/1.14033367-14034207.png]

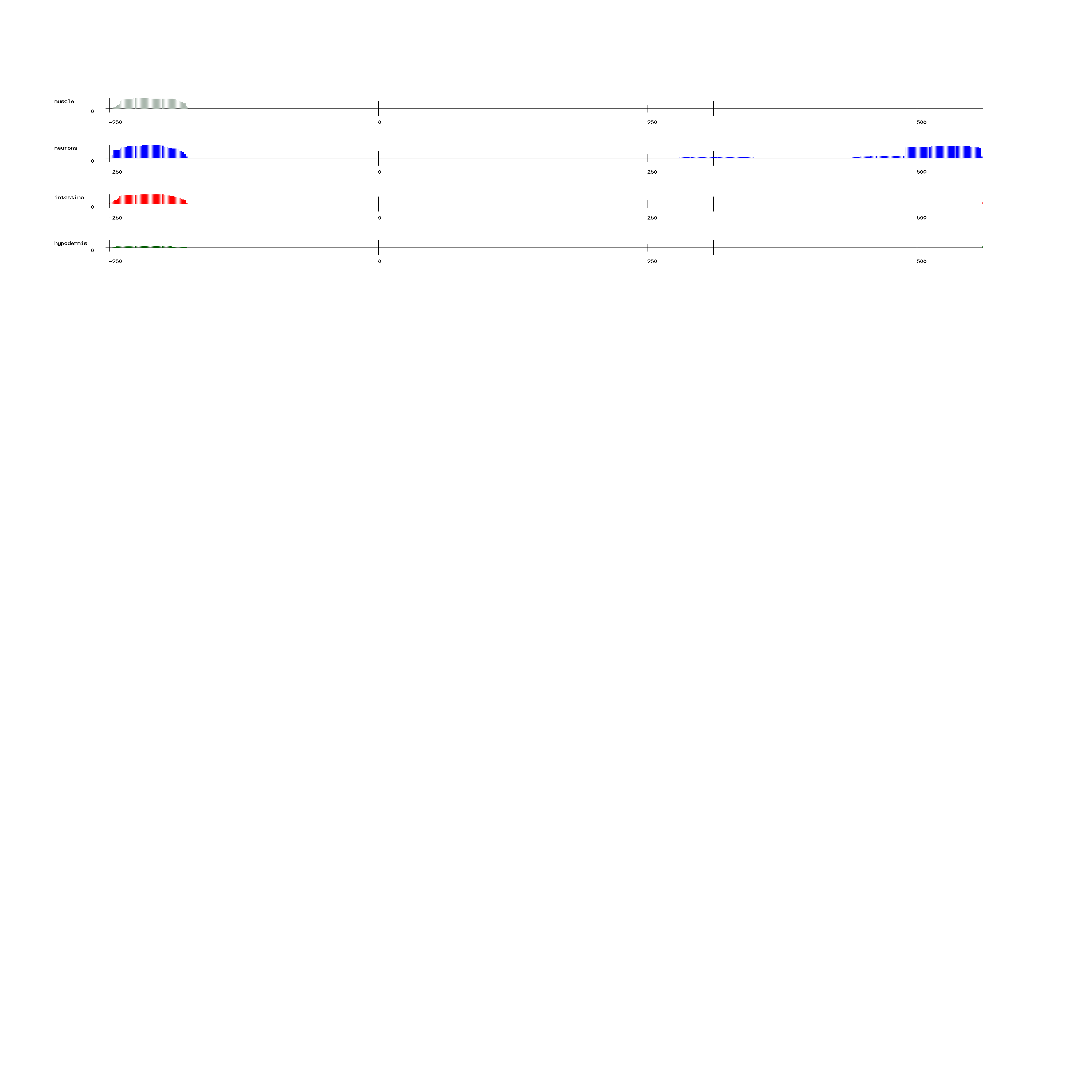

Supplement: Supplementary file 1 [file ijms-24-02970-s001.zip › Supplementary Data S2/1.14128159-14128469.png]

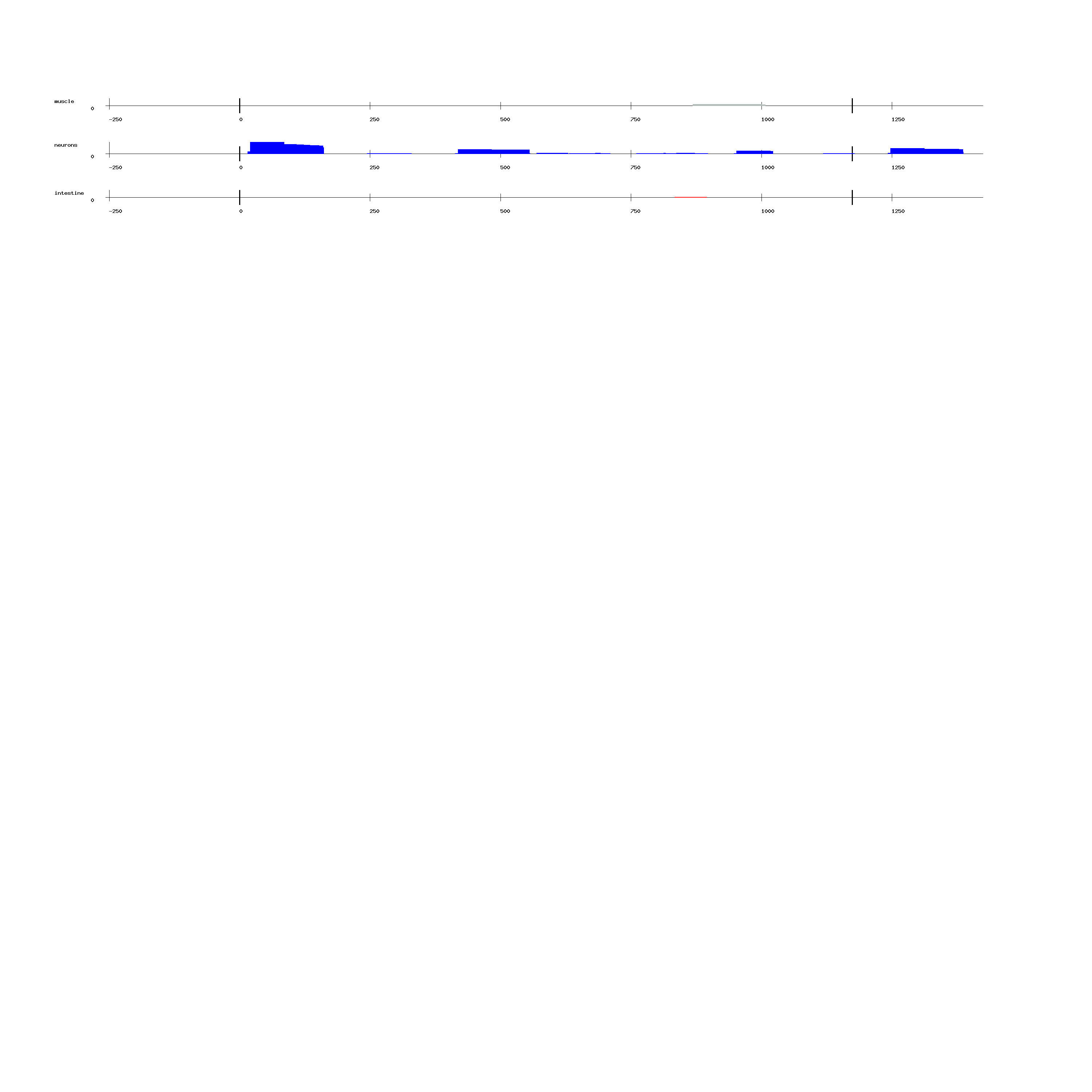

Supplement: Supplementary file 1 [file ijms-24-02970-s001.zip › Supplementary Data S2/1.14257569-14258742.png]

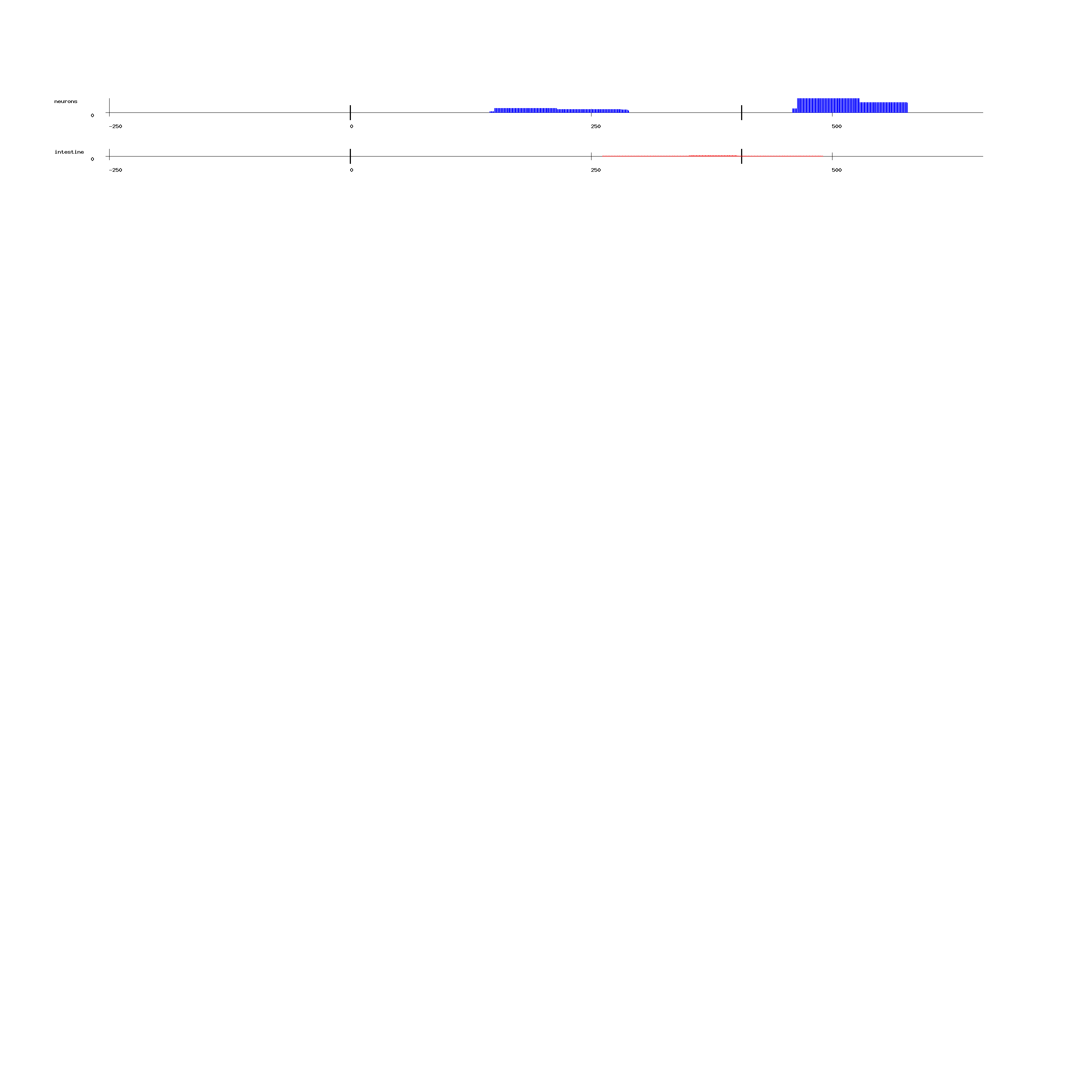

Supplement: Supplementary file 1 [file ijms-24-02970-s001.zip › Supplementary Data S2/1.14329713-14330118.png]

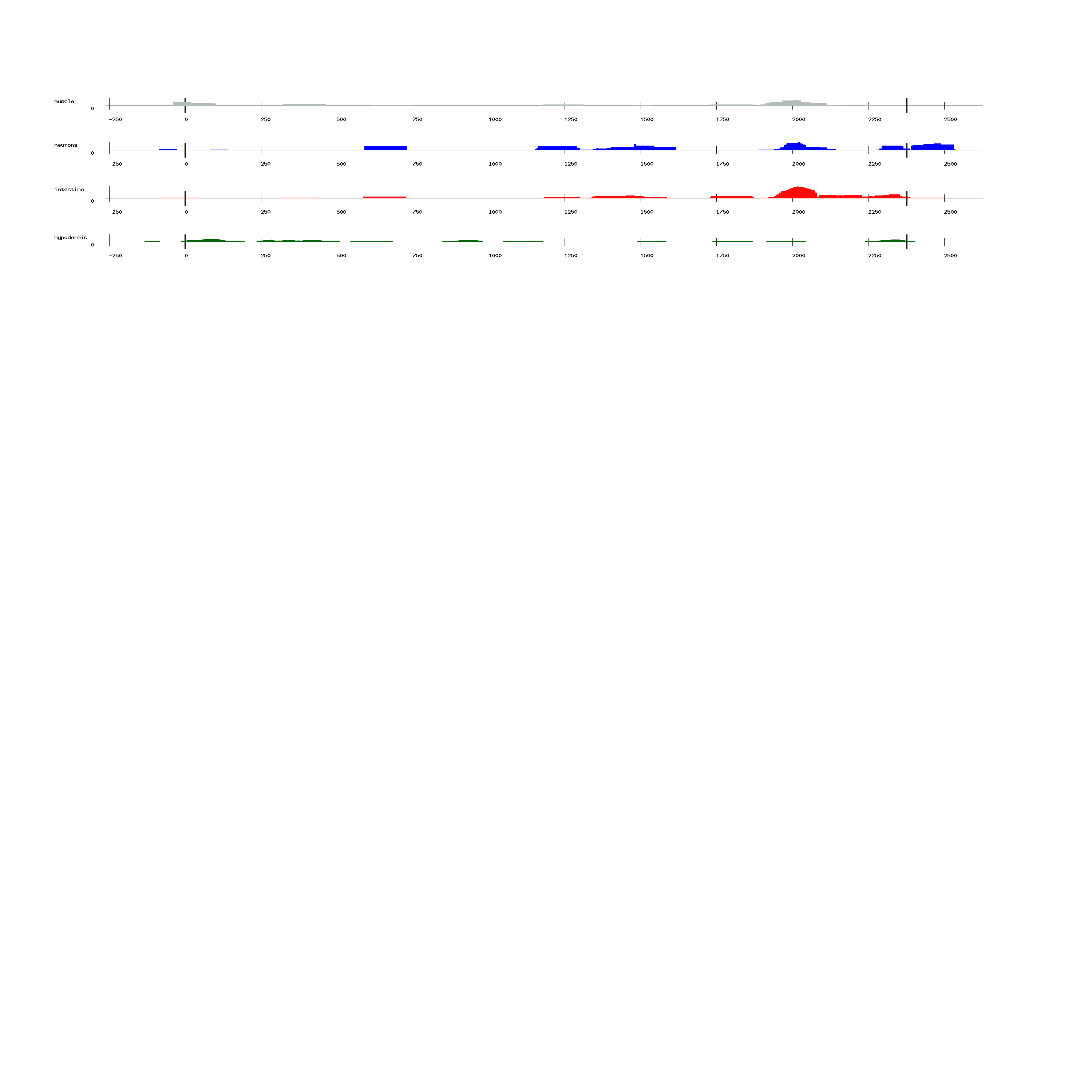

Supplement: Supplementary file 1 [file ijms-24-02970-s001.zip › Supplementary Data S2/1.14452267-14454642.png]

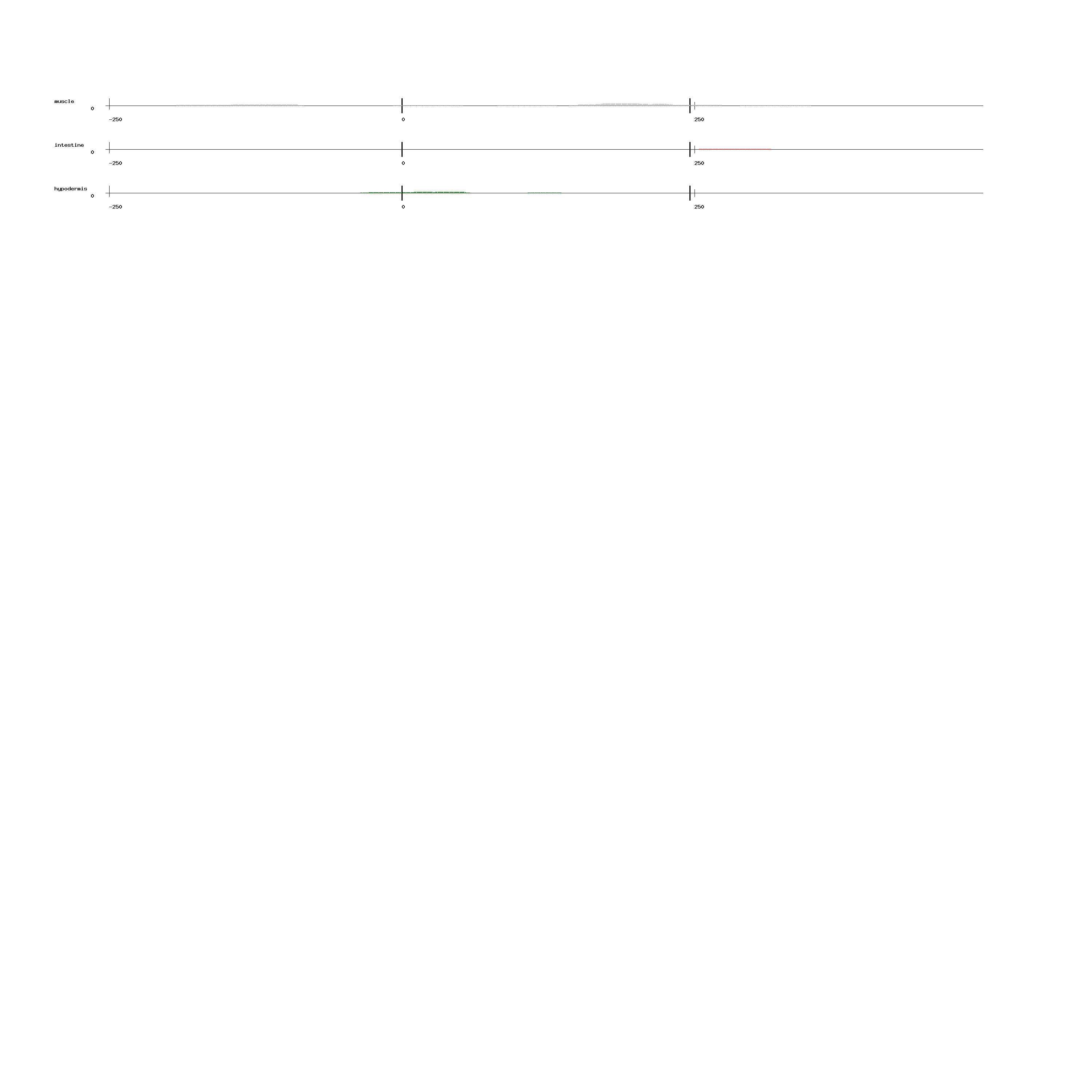

Supplement: Supplementary file 1 [file ijms-24-02970-s001.zip › Supplementary Data S2/1.14619236-14619481.png]

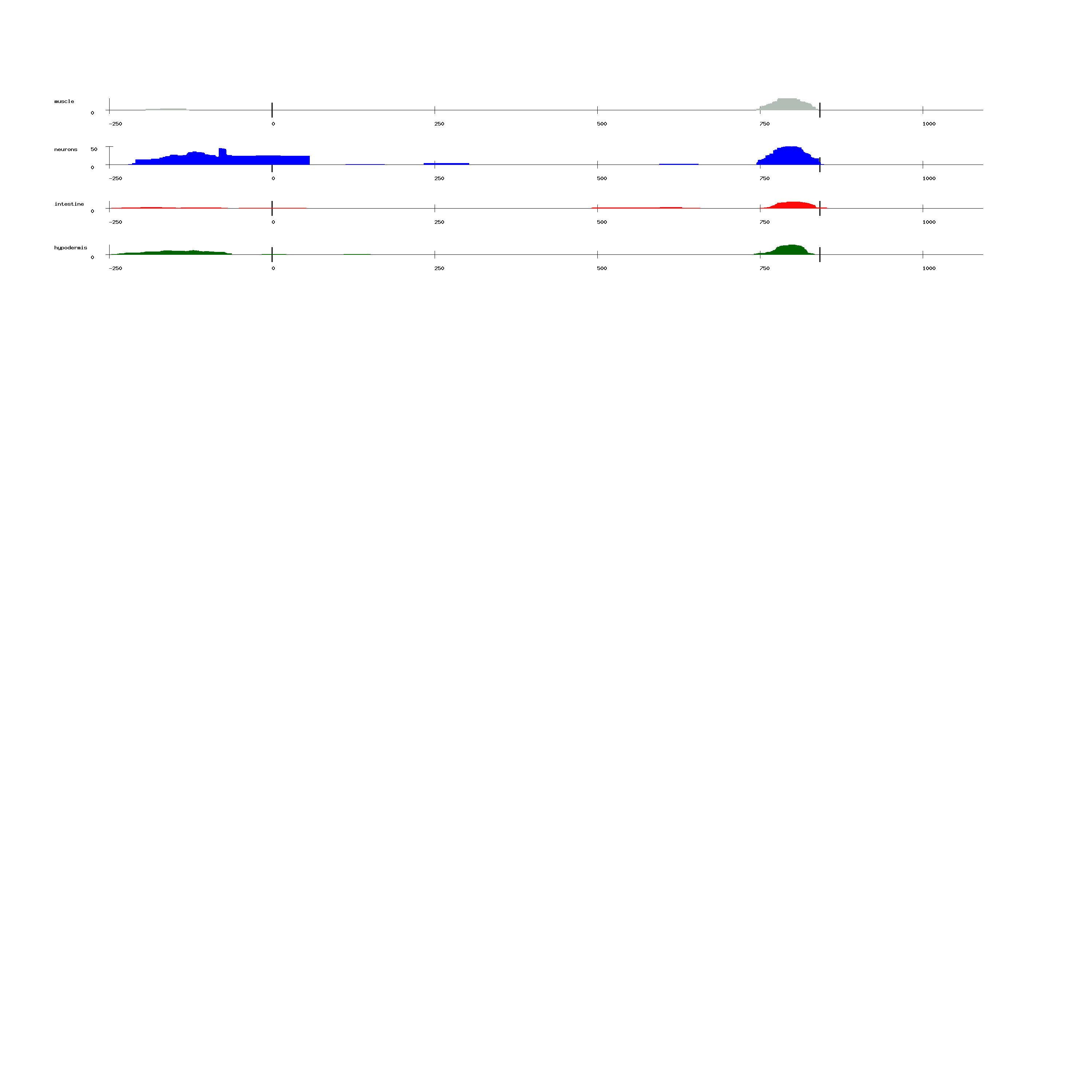

Supplement: Supplementary file 1 [file ijms-24-02970-s001.zip › Supplementary Data S2/1.14686490-14687331.png]

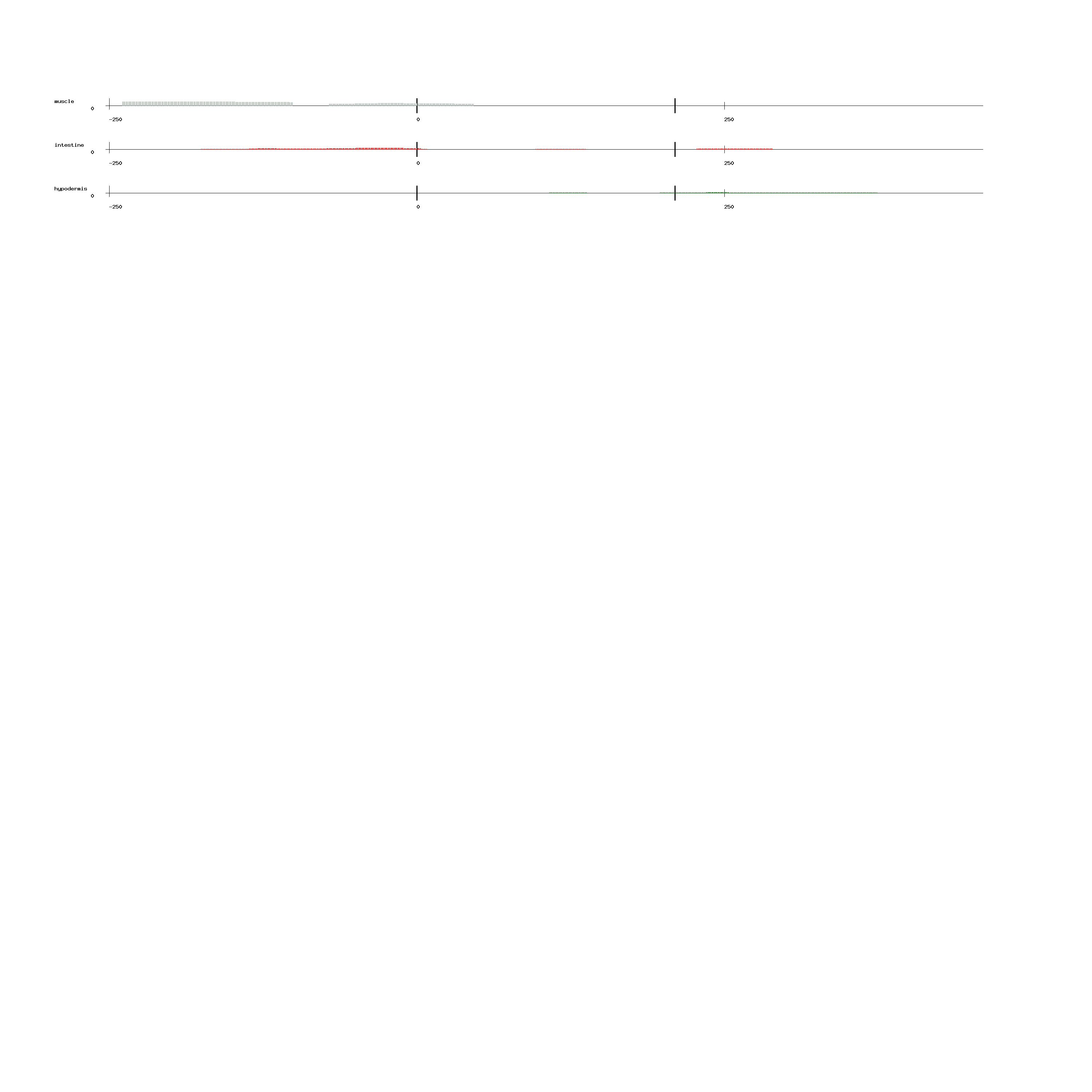

Supplement: Supplementary file 1 [file ijms-24-02970-s001.zip › Supplementary Data S2/1.14843491-14843700.png]

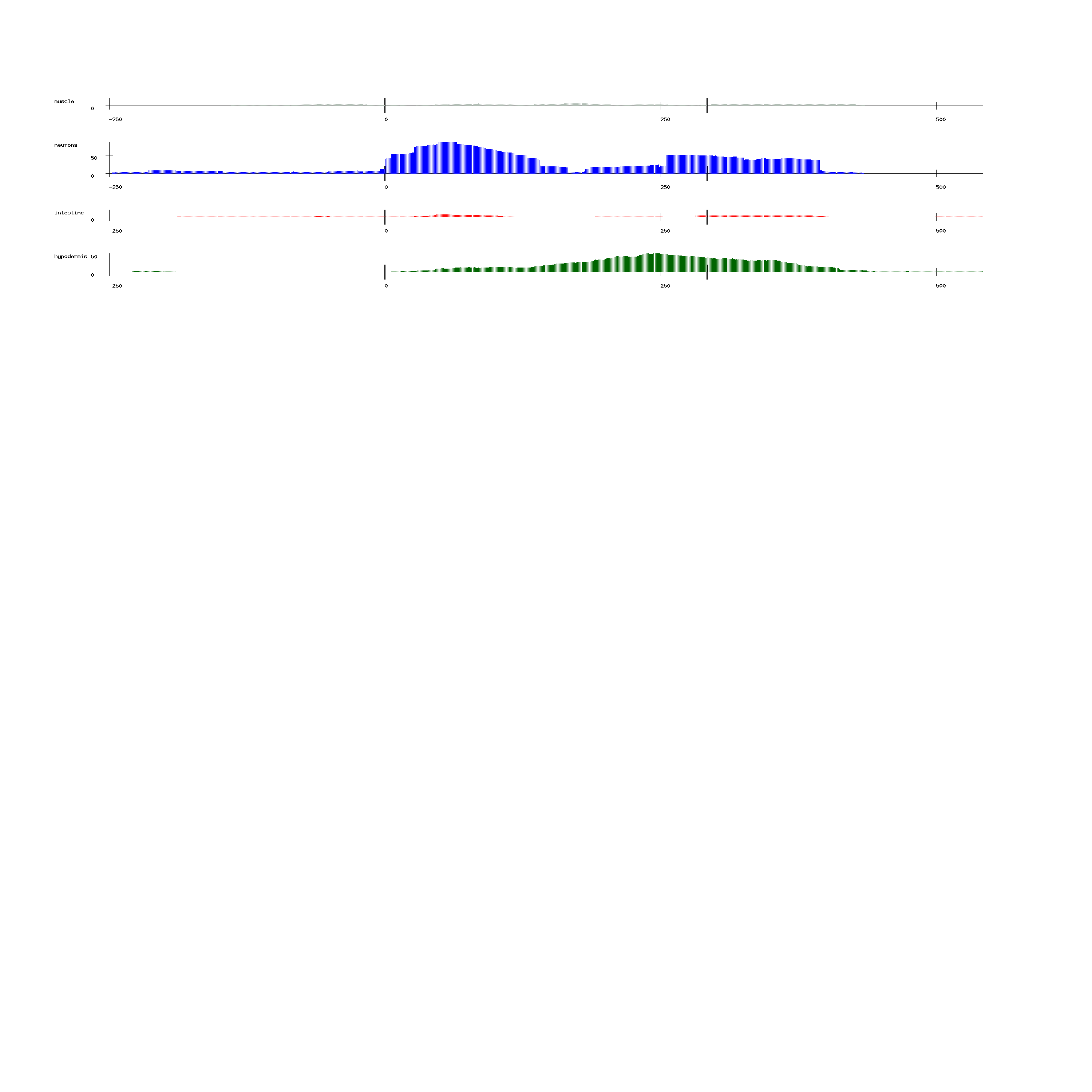

Supplement: Supplementary file 1 [file ijms-24-02970-s001.zip › Supplementary Data S2/1.1564845-1565136.png]

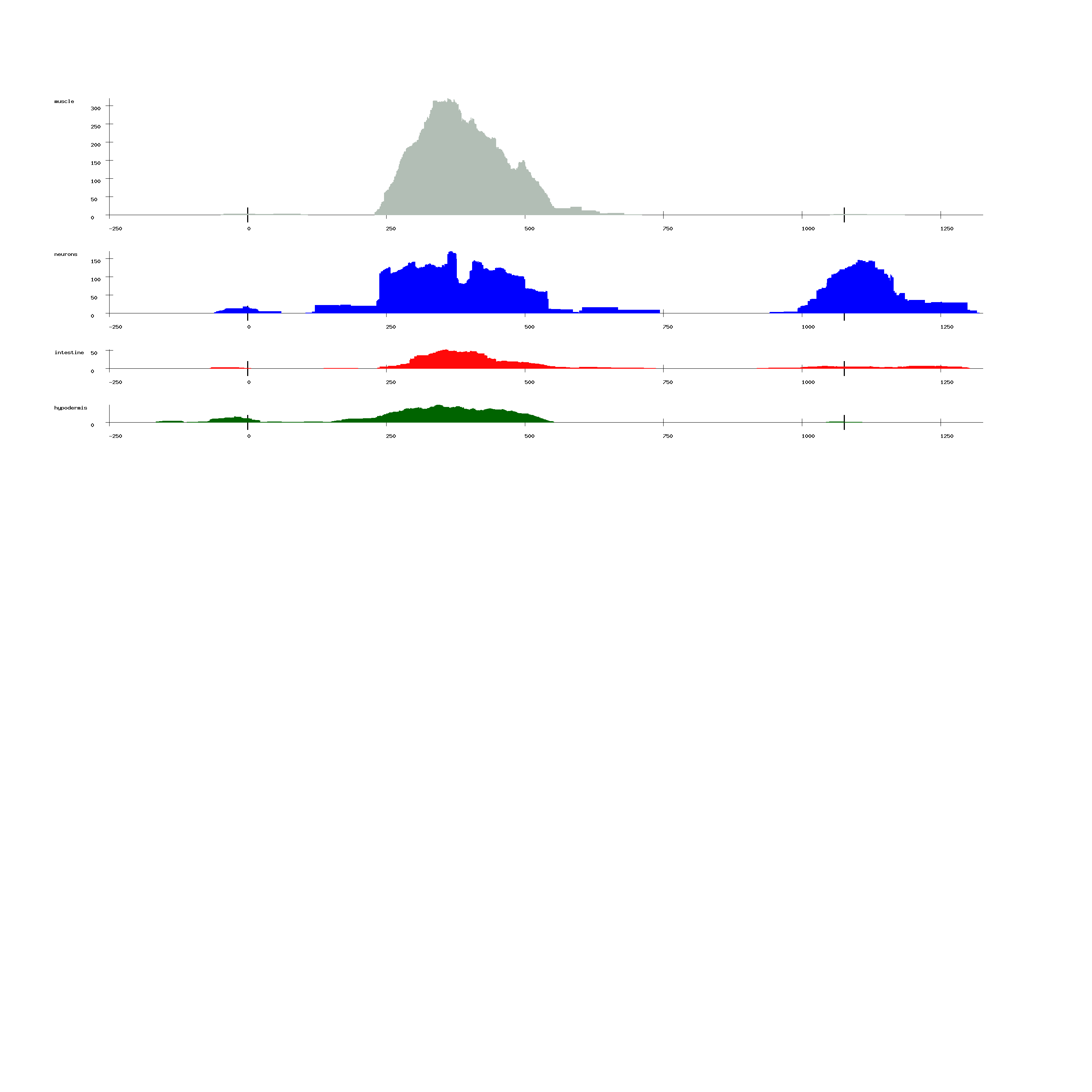

Supplement: Supplementary file 1 [file ijms-24-02970-s001.zip › Supplementary Data S2/1.1593120-1594195.png]

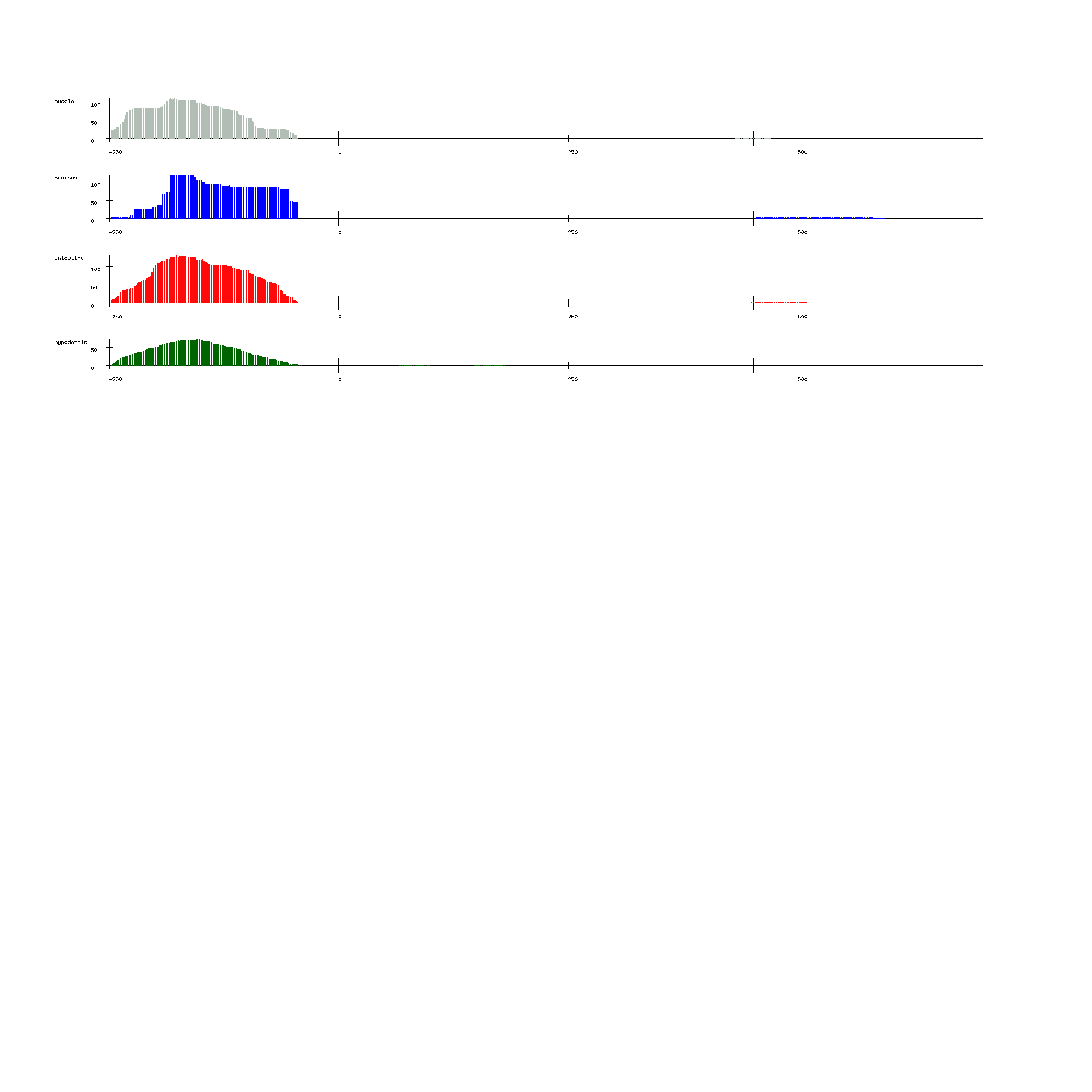

Supplement: Supplementary file 1 [file ijms-24-02970-s001.zip › Supplementary Data S2/1.1796469-1796919.png]

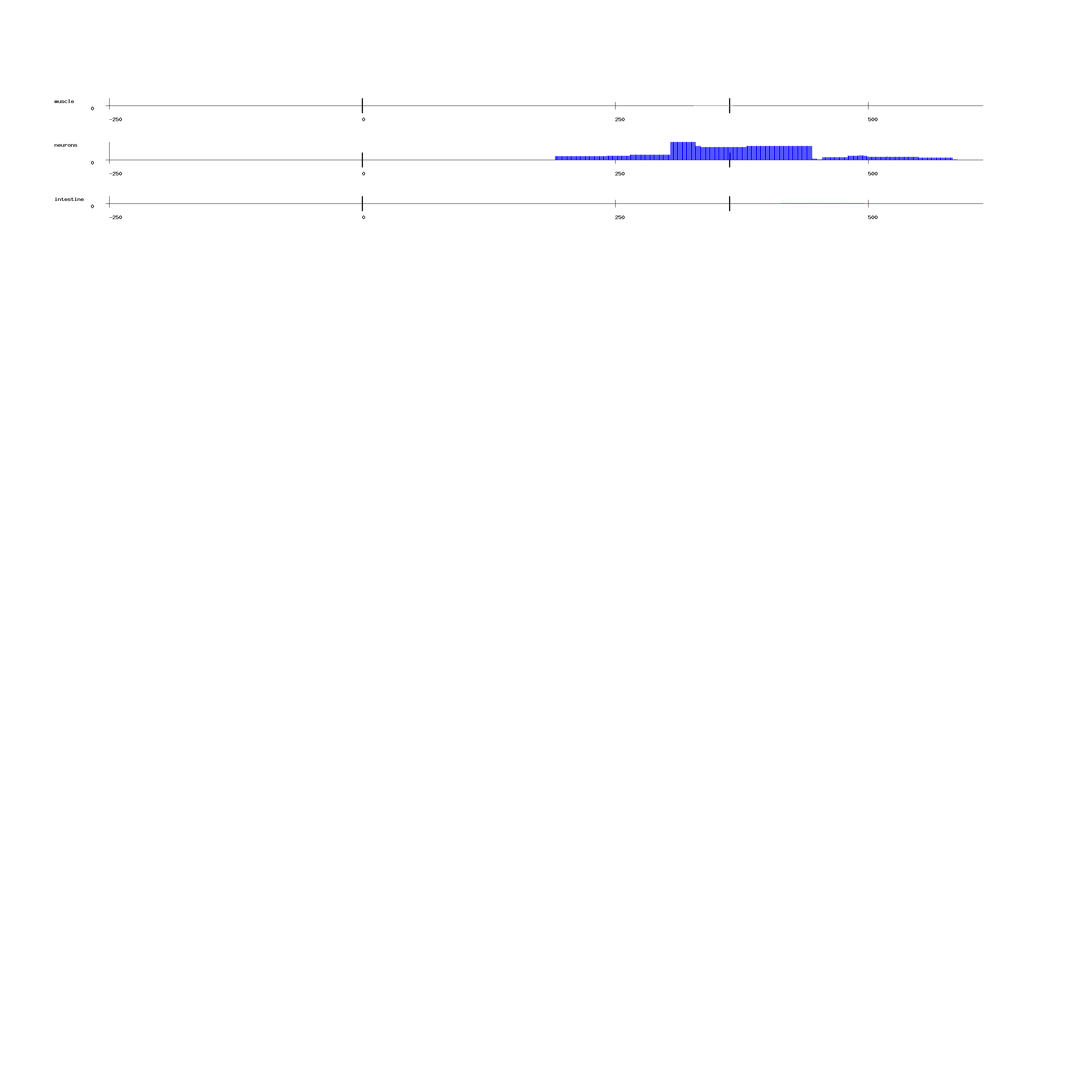

Supplement: Supplementary file 1 [file ijms-24-02970-s001.zip › Supplementary Data S2/1.1807418-1807780.png]

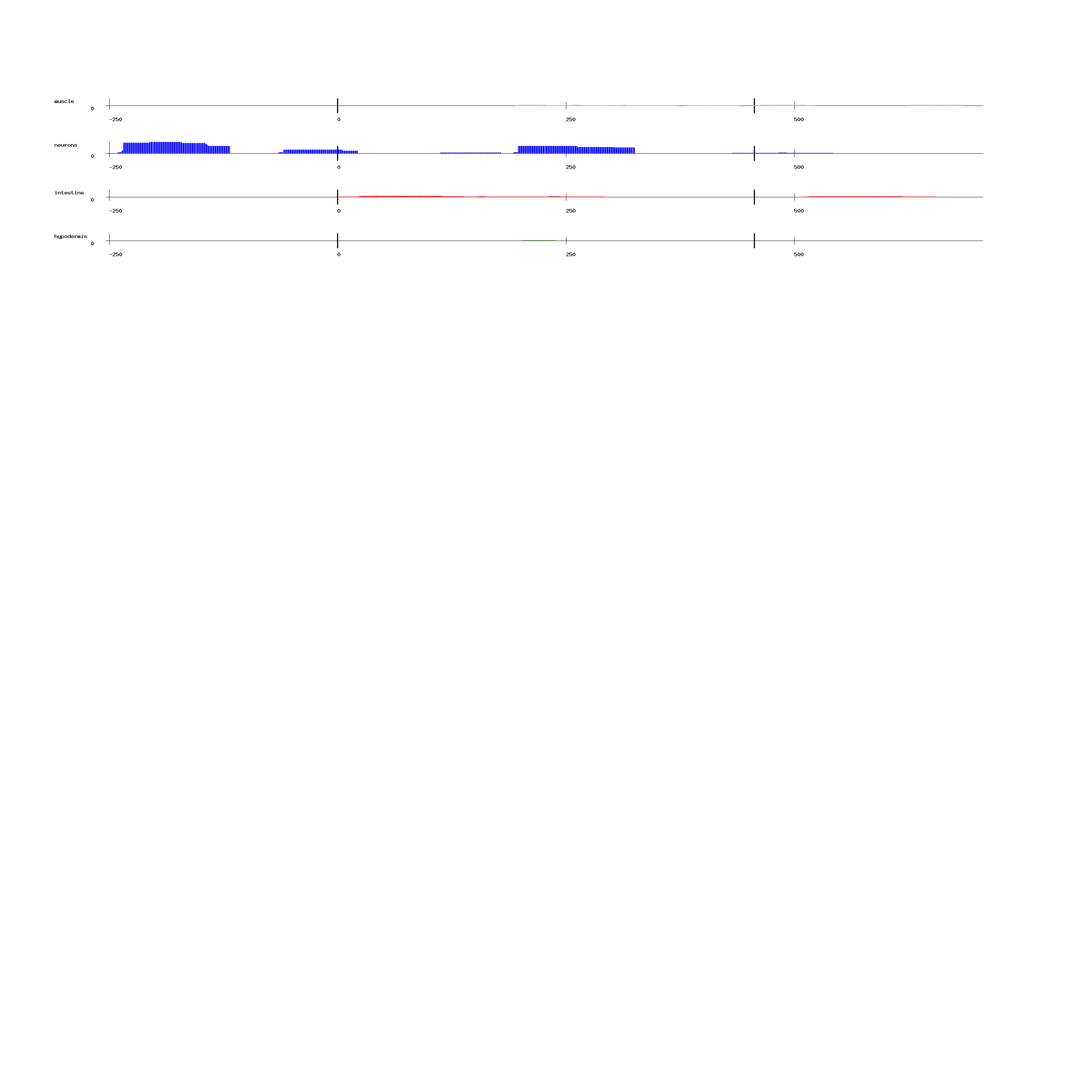

Supplement: Supplementary file 1 [file ijms-24-02970-s001.zip › Supplementary Data S2/1.1884484-1884939.png]

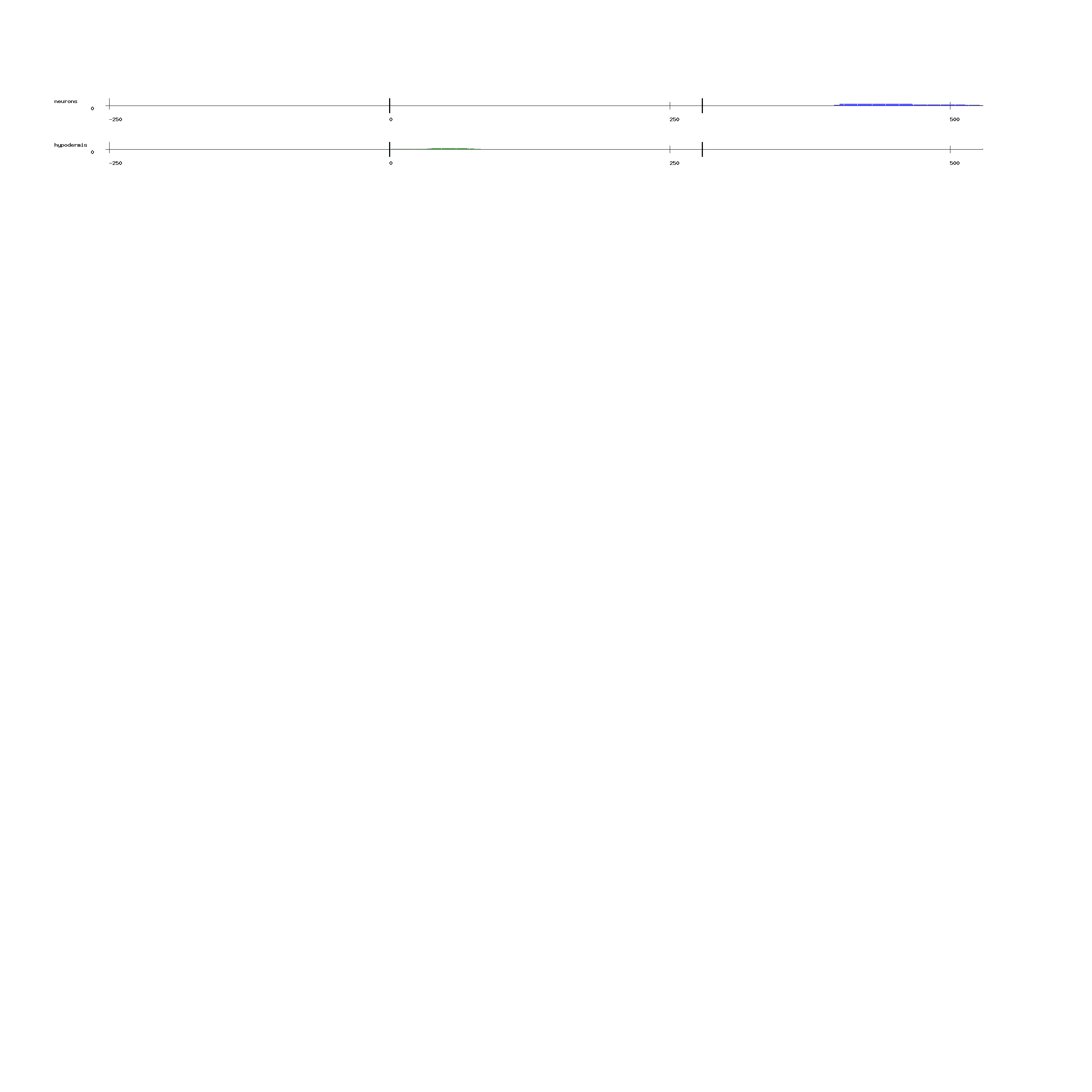

Supplement: Supplementary file 1 [file ijms-24-02970-s001.zip › Supplementary Data S2/1.1949999-1950277.png]

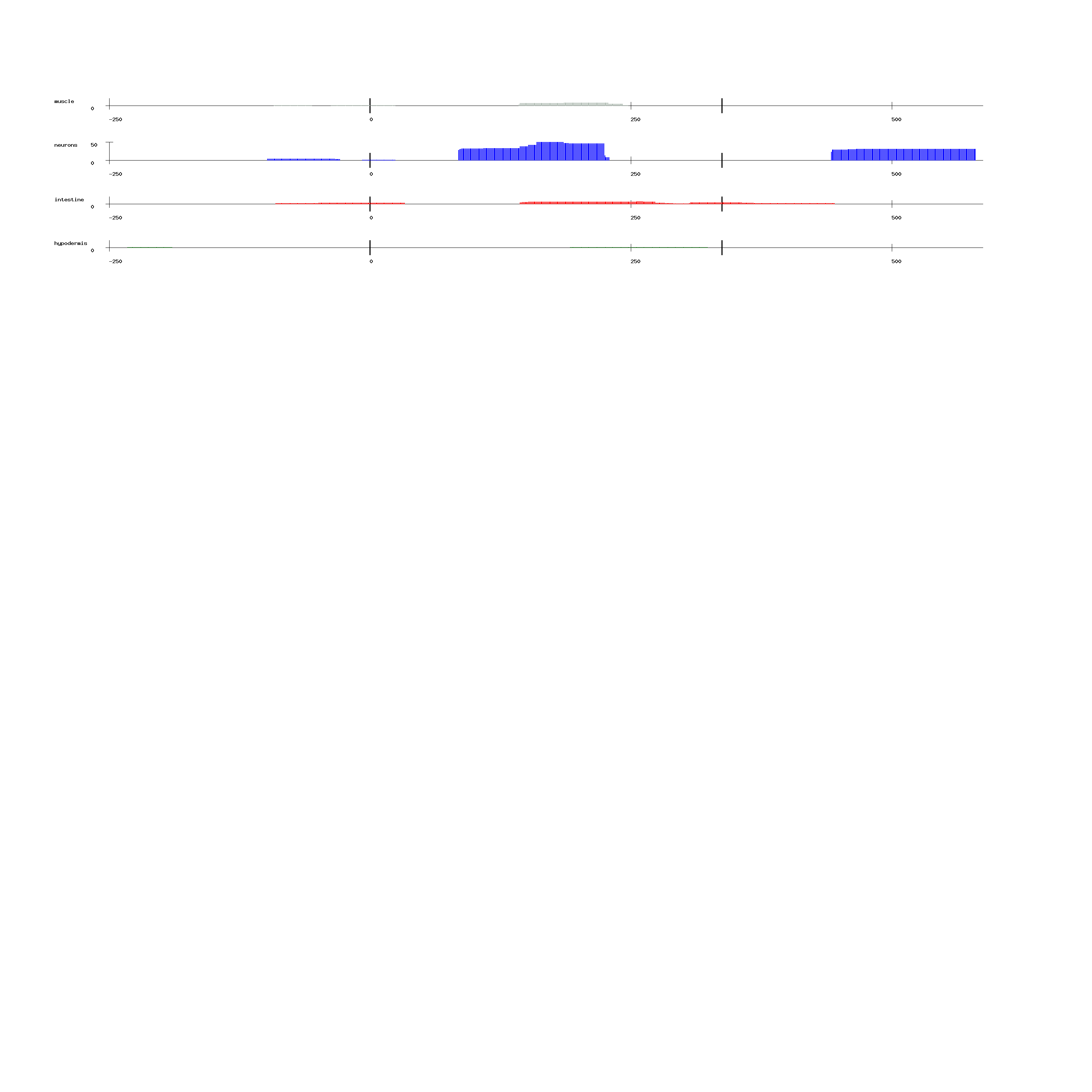

Supplement: Supplementary file 1 [file ijms-24-02970-s001.zip › Supplementary Data S2/1.1987299-1987635.png]

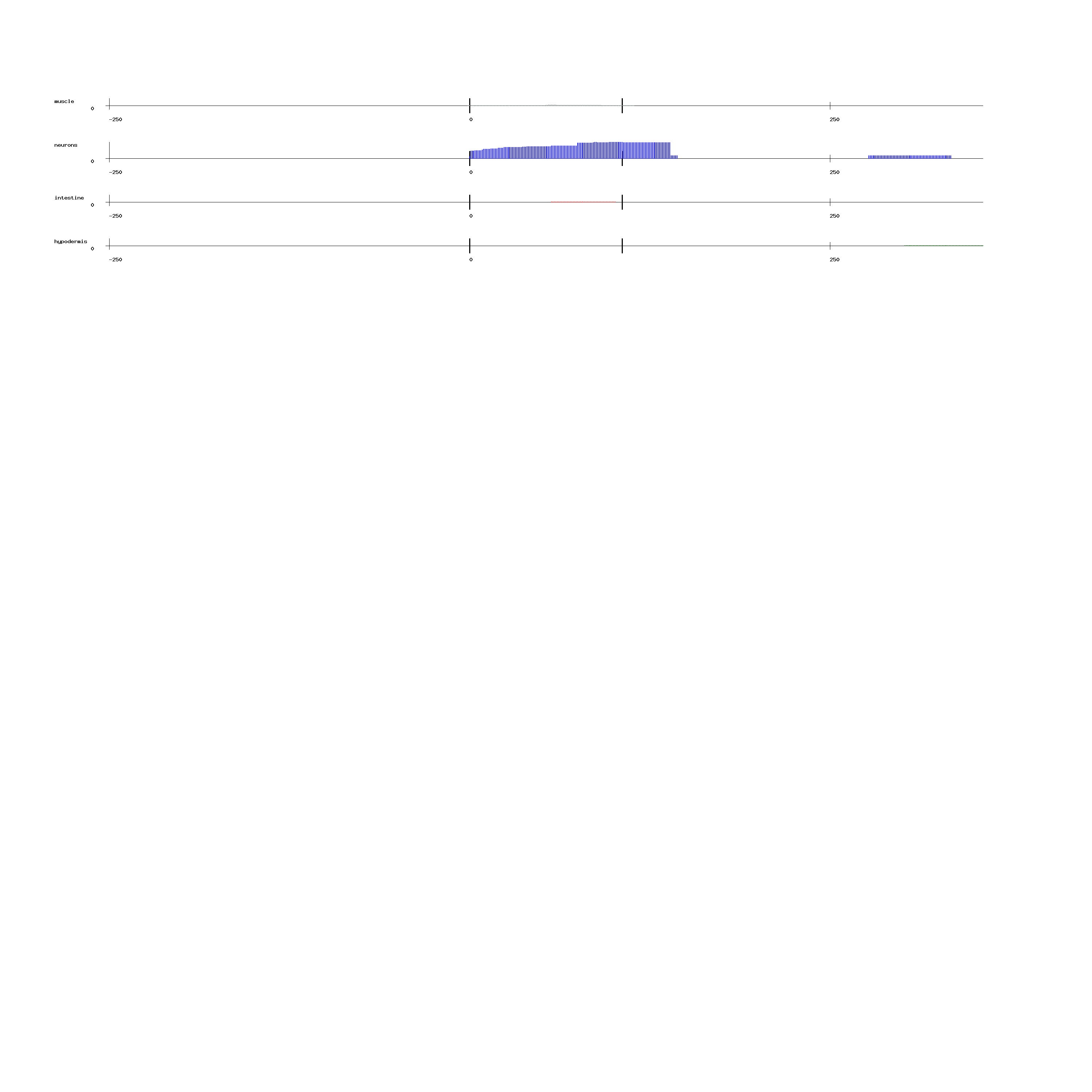

Supplement: Supplementary file 1 [file ijms-24-02970-s001.zip › Supplementary Data S2/1.222749-222854.png]

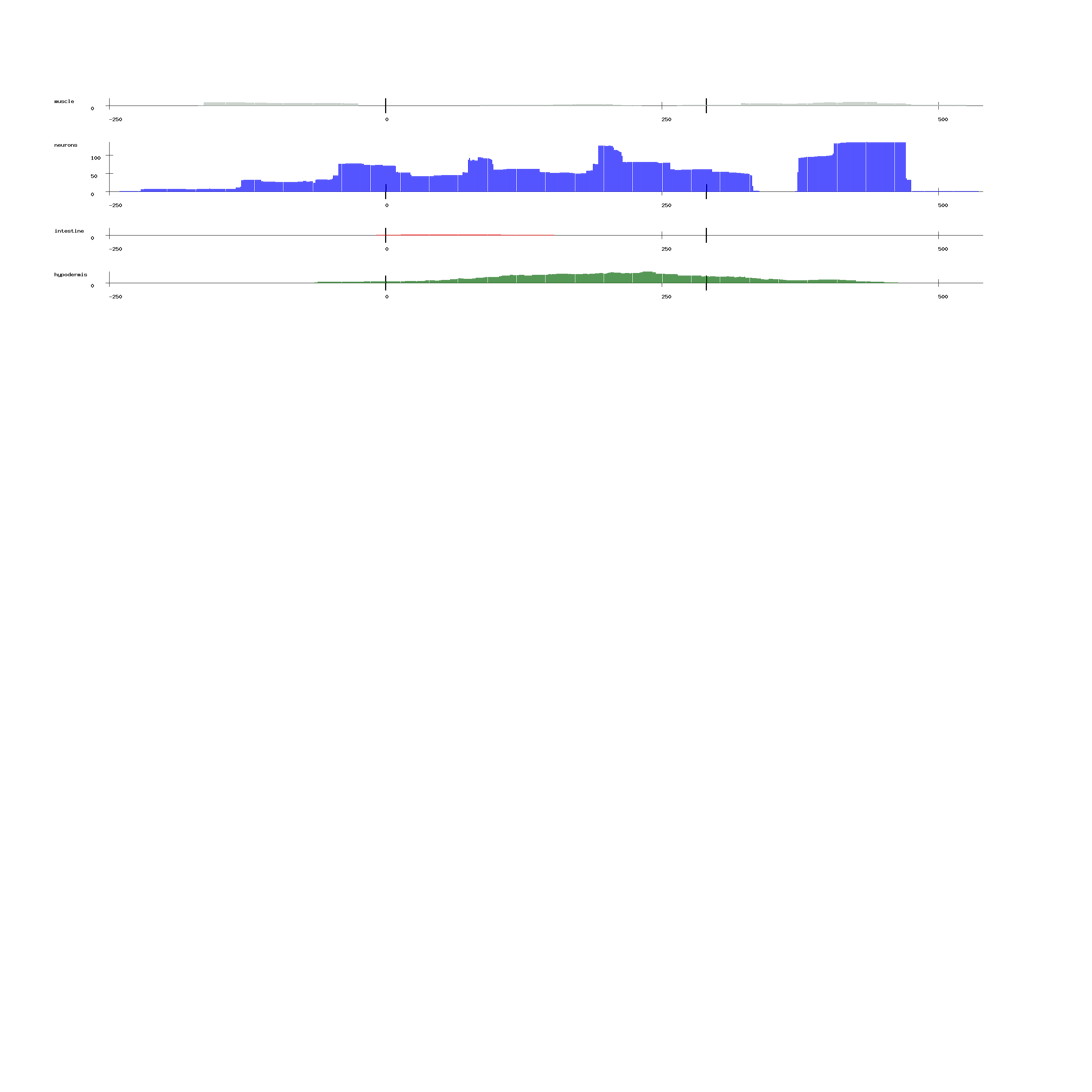

Supplement: Supplementary file 1 [file ijms-24-02970-s001.zip › Supplementary Data S2/1.2524693-2524982.png]

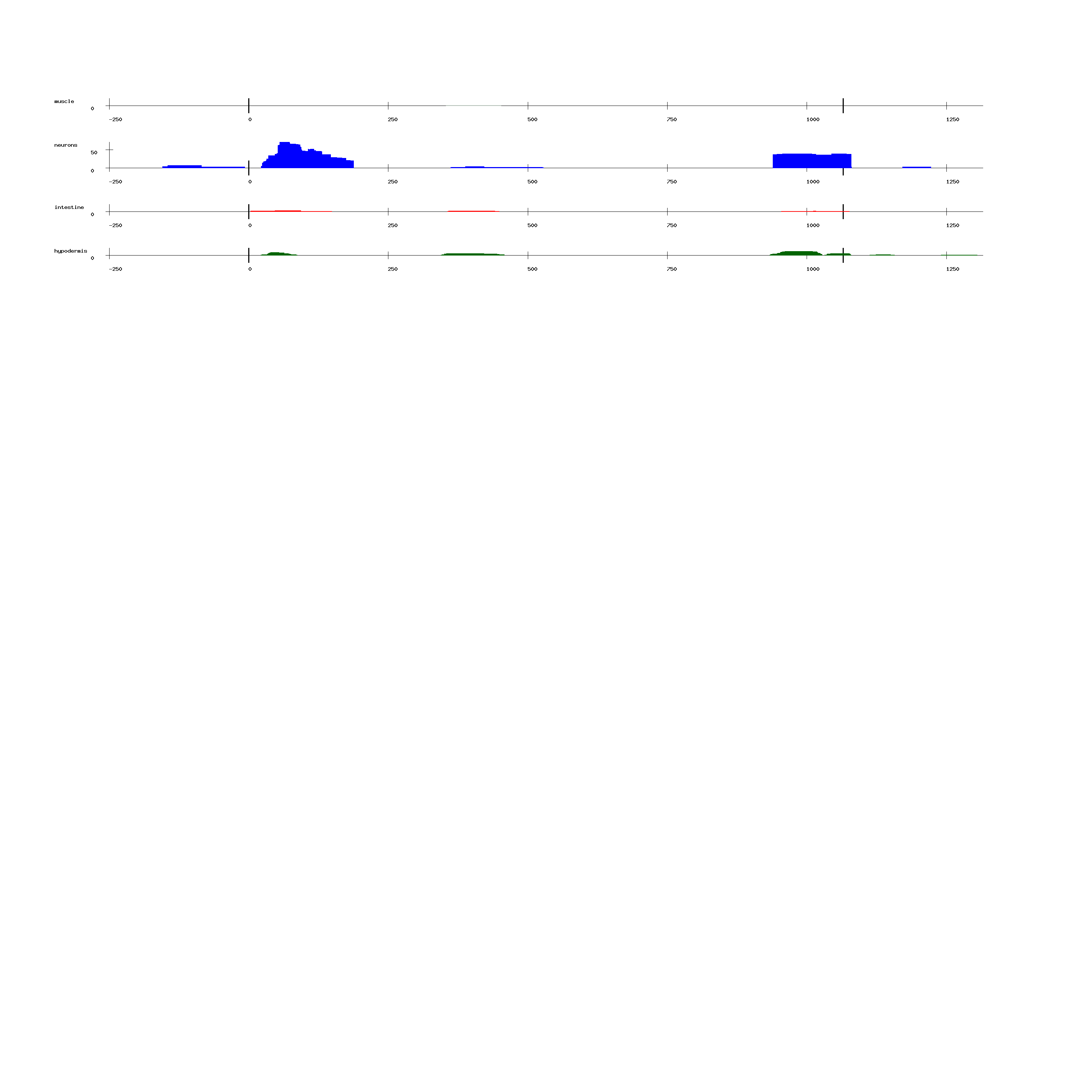

Supplement: Supplementary file 1 [file ijms-24-02970-s001.zip › Supplementary Data S2/1.2542918-2543982.png]

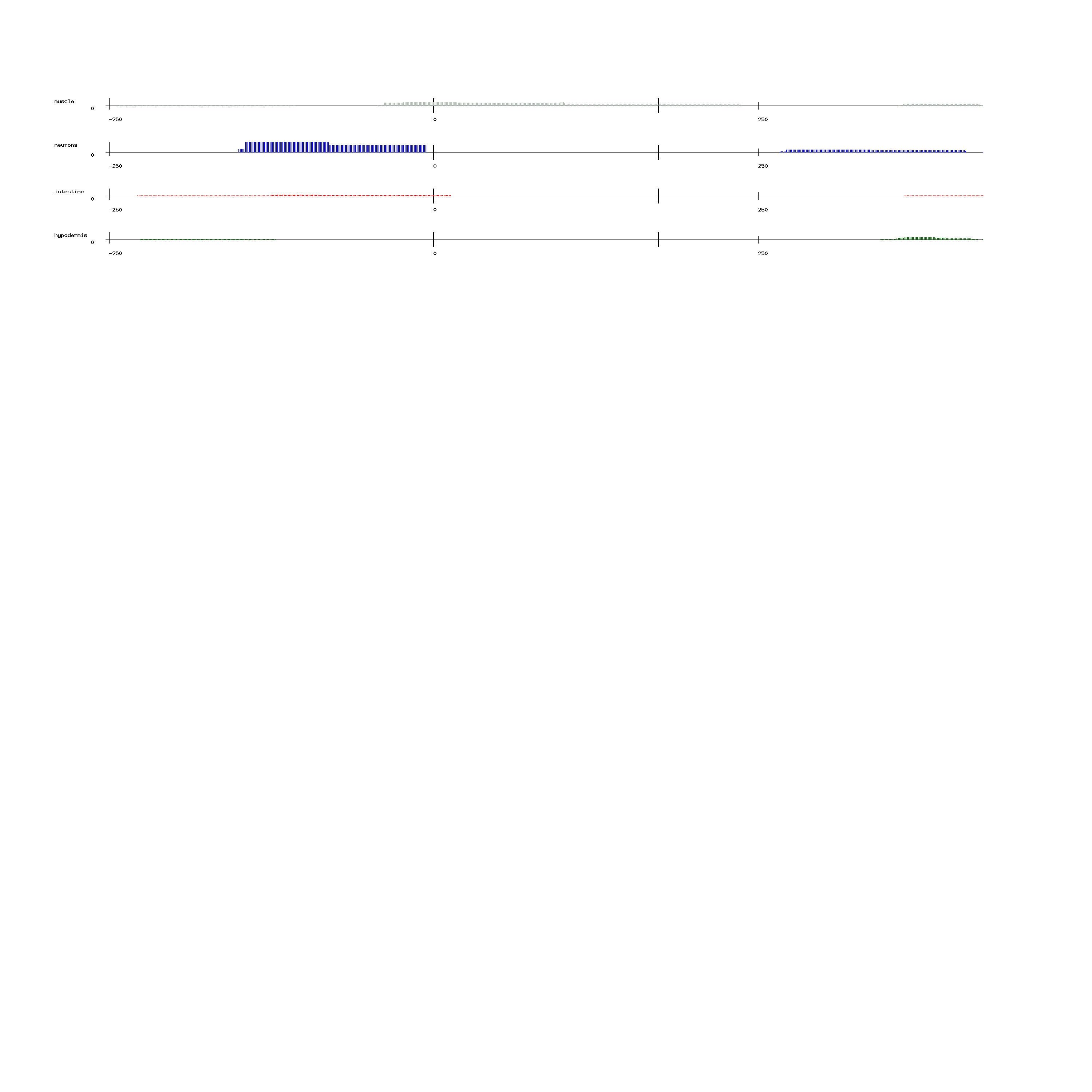

Supplement: Supplementary file 1 [file ijms-24-02970-s001.zip › Supplementary Data S2/1.2858185-2858357.png]

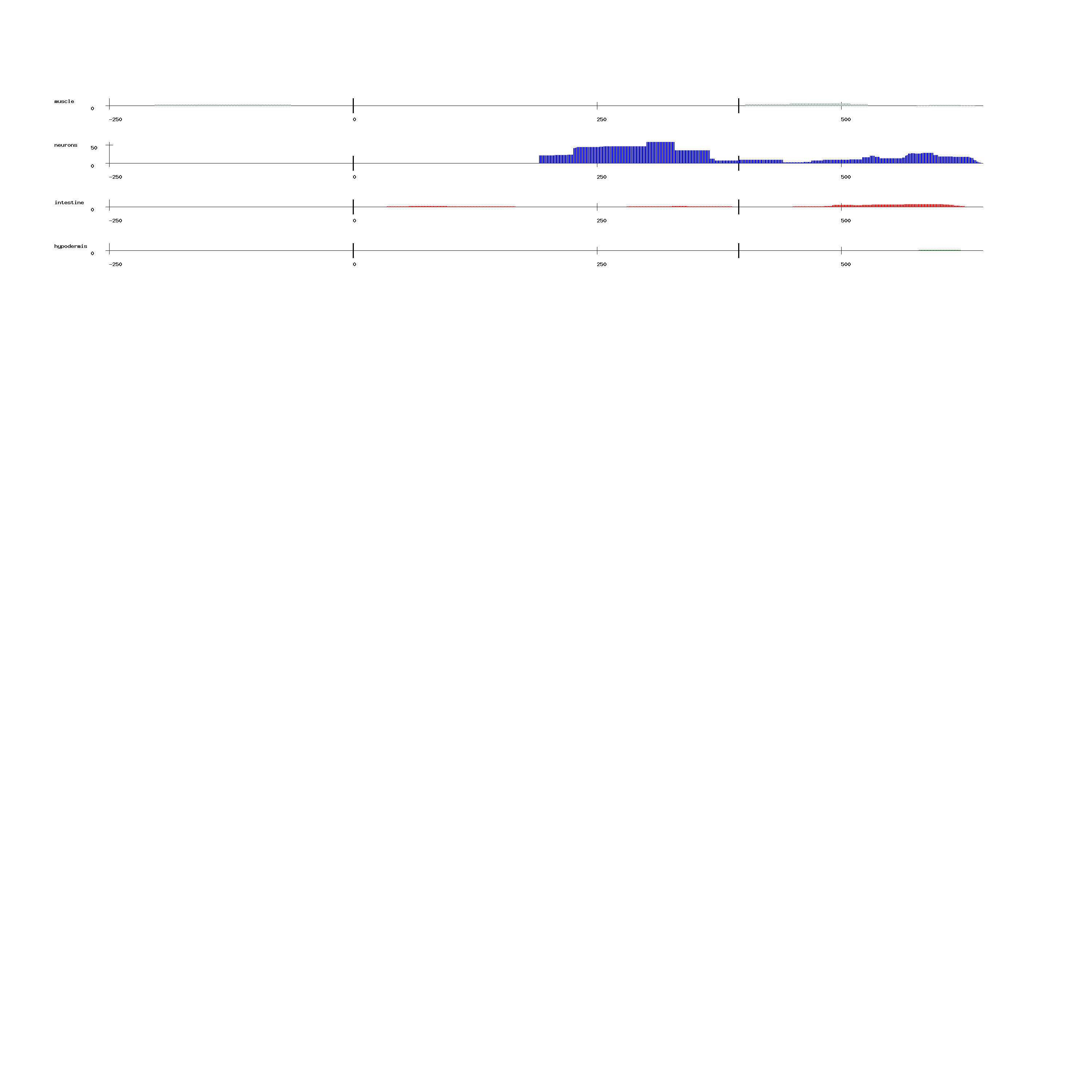

Supplement: Supplementary file 1 [file ijms-24-02970-s001.zip › Supplementary Data S2/1.3114471-3114865.png]

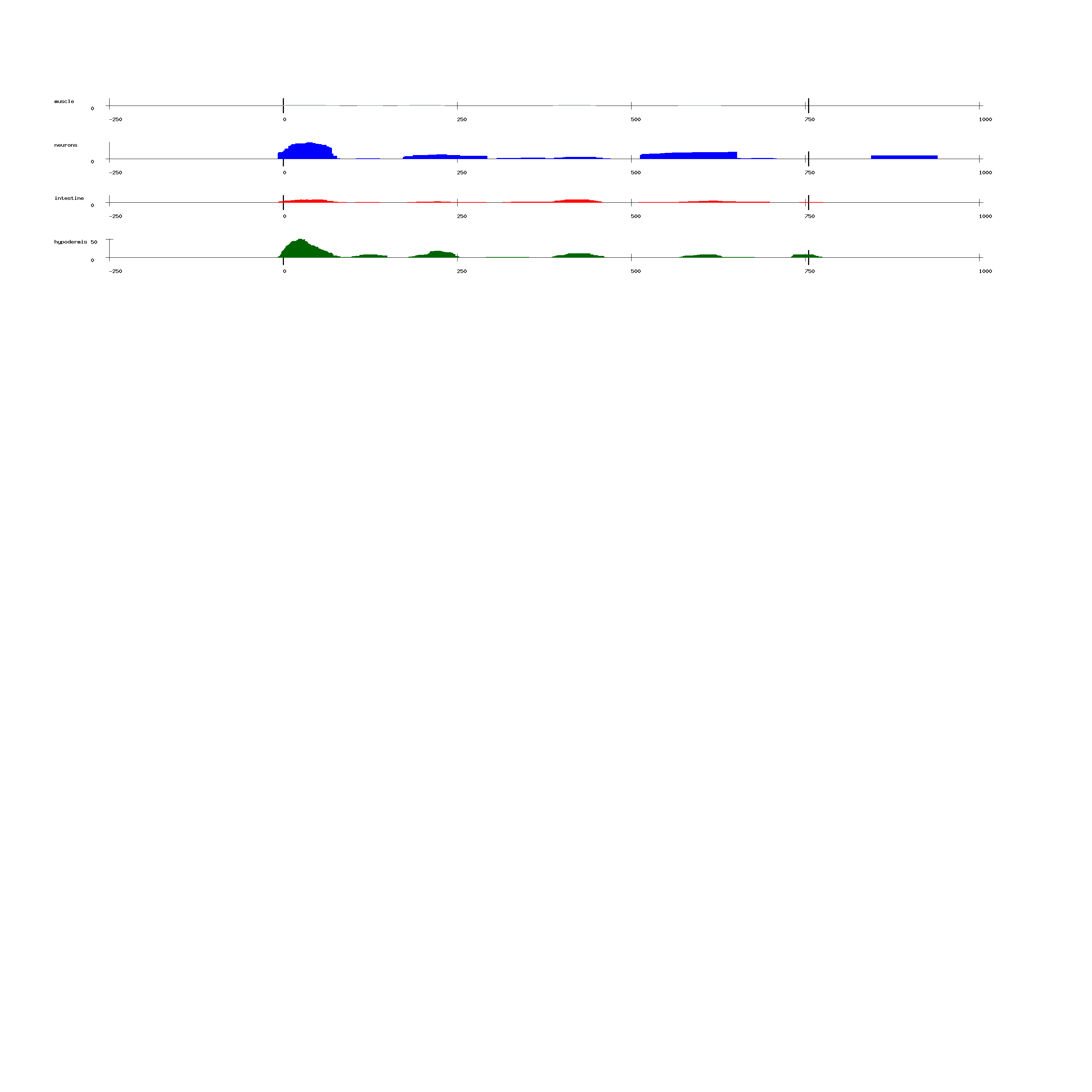

Supplement: Supplementary file 1 [file ijms-24-02970-s001.zip › Supplementary Data S2/1.3171655-3172409.png]

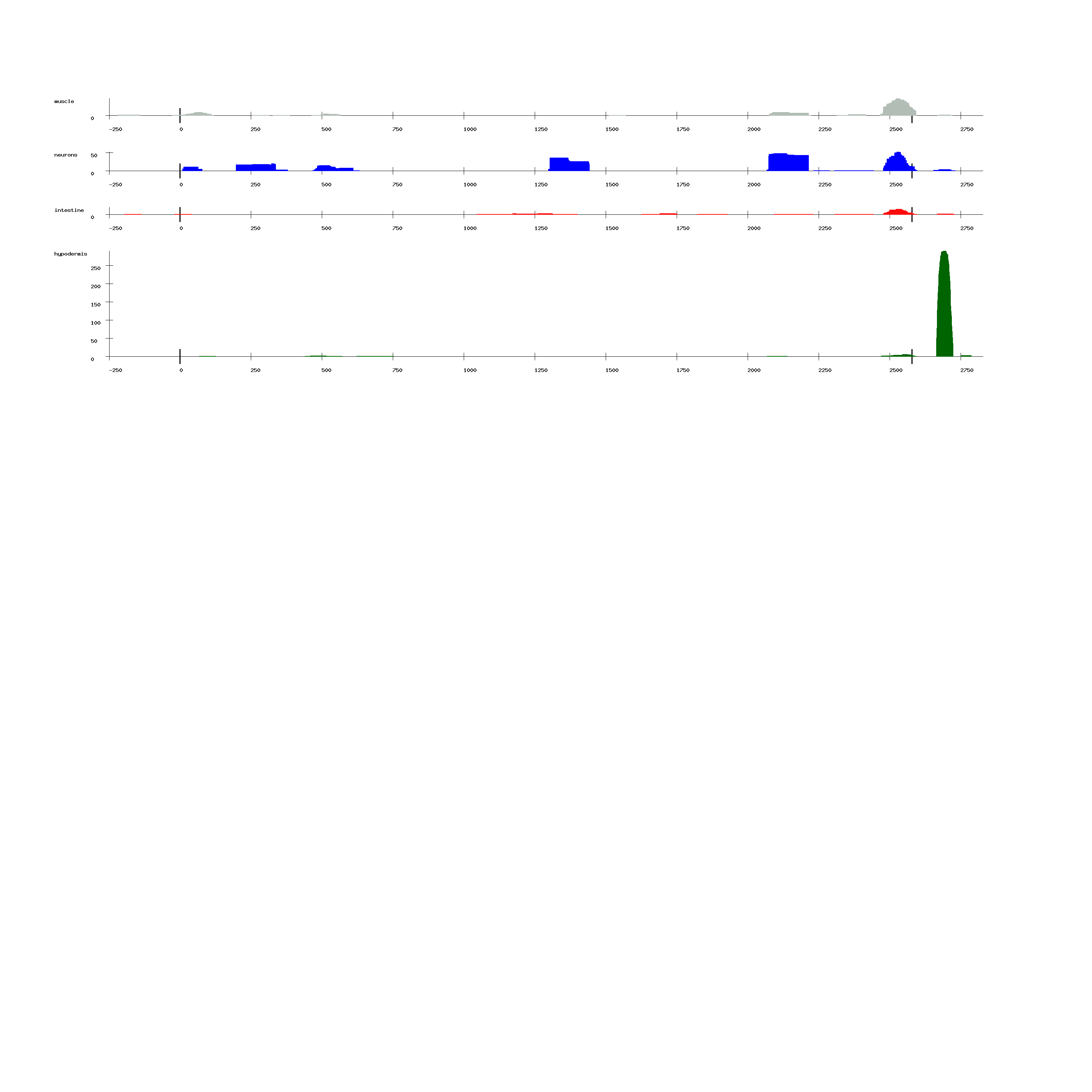

Supplement: Supplementary file 1 [file ijms-24-02970-s001.zip › Supplementary Data S2/1.338561-341137.png]

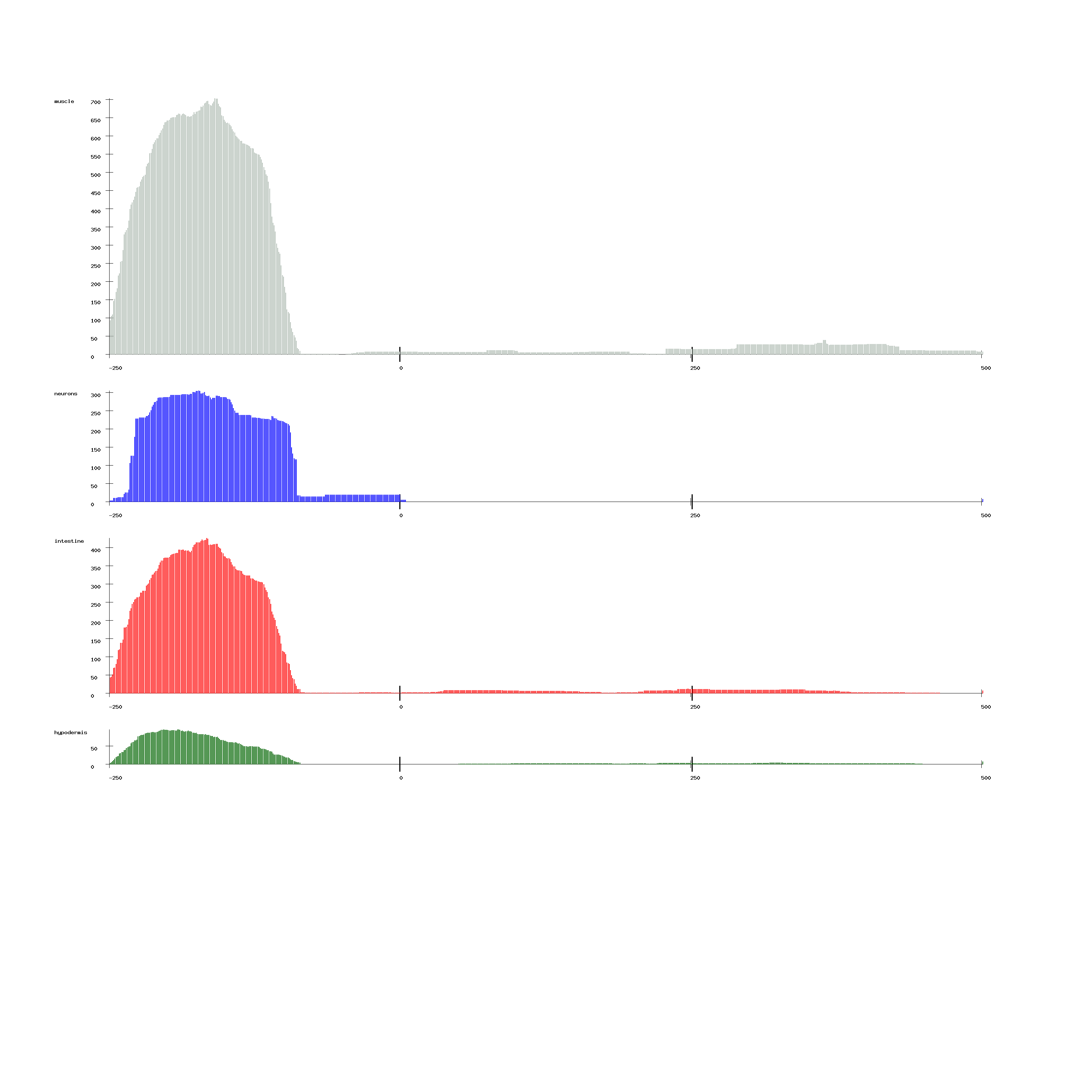

Supplement: Supplementary file 1 [file ijms-24-02970-s001.zip › Supplementary Data S2/1.3424298-3424548.png]

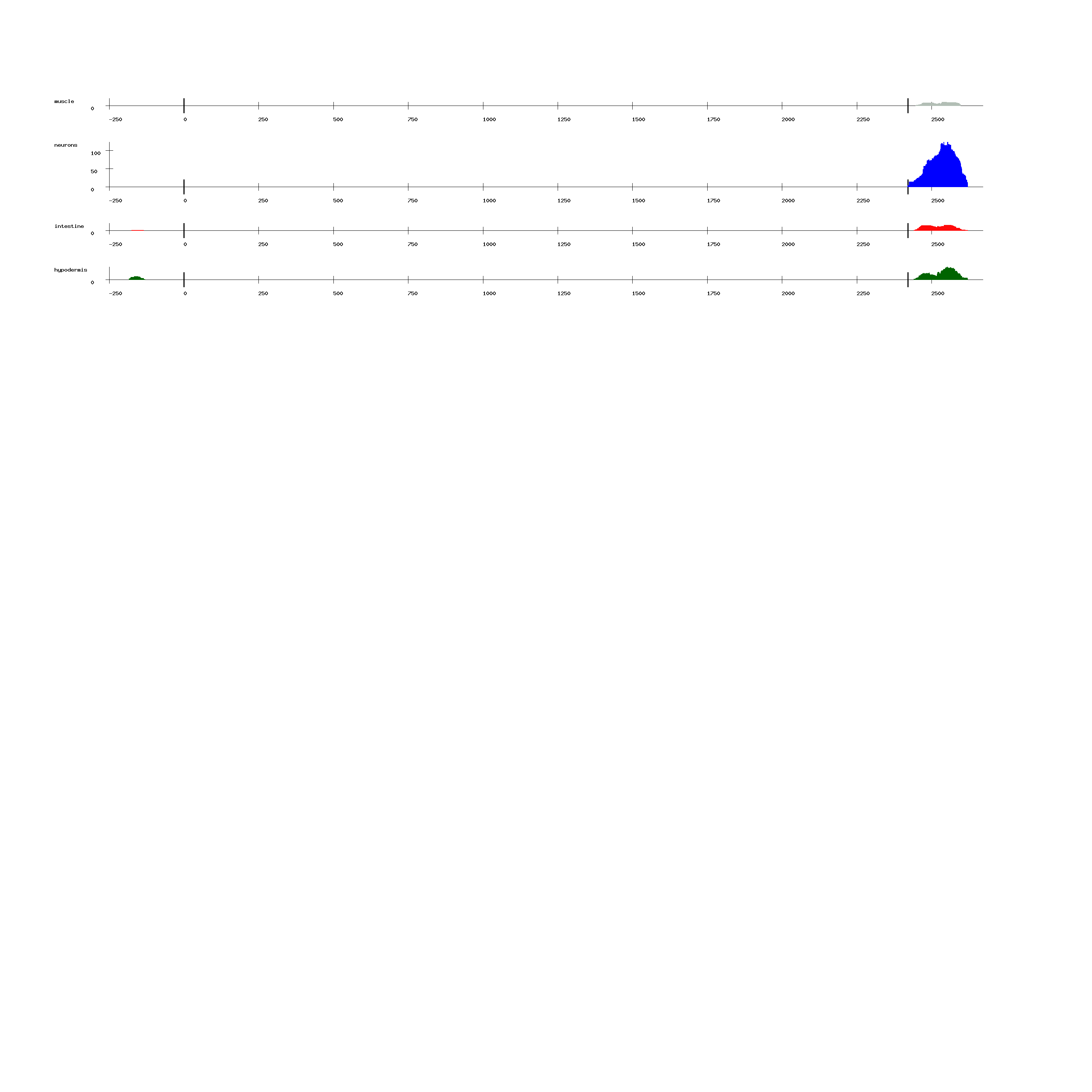

Supplement: Supplementary file 1 [file ijms-24-02970-s001.zip › Supplementary Data S2/1.3485156-3487576.png]

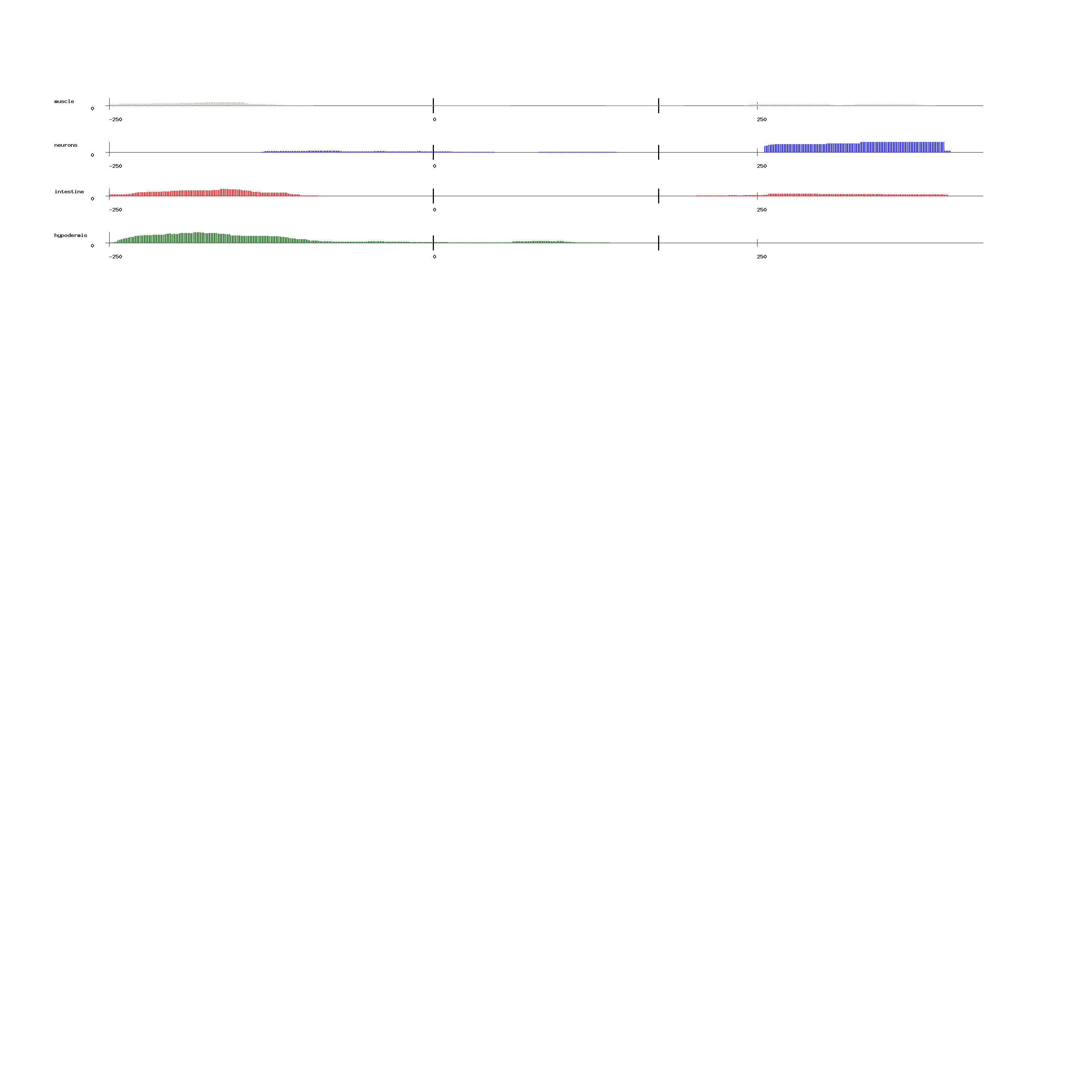

Supplement: Supplementary file 1 [file ijms-24-02970-s001.zip › Supplementary Data S2/1.3602994-3603167.png]

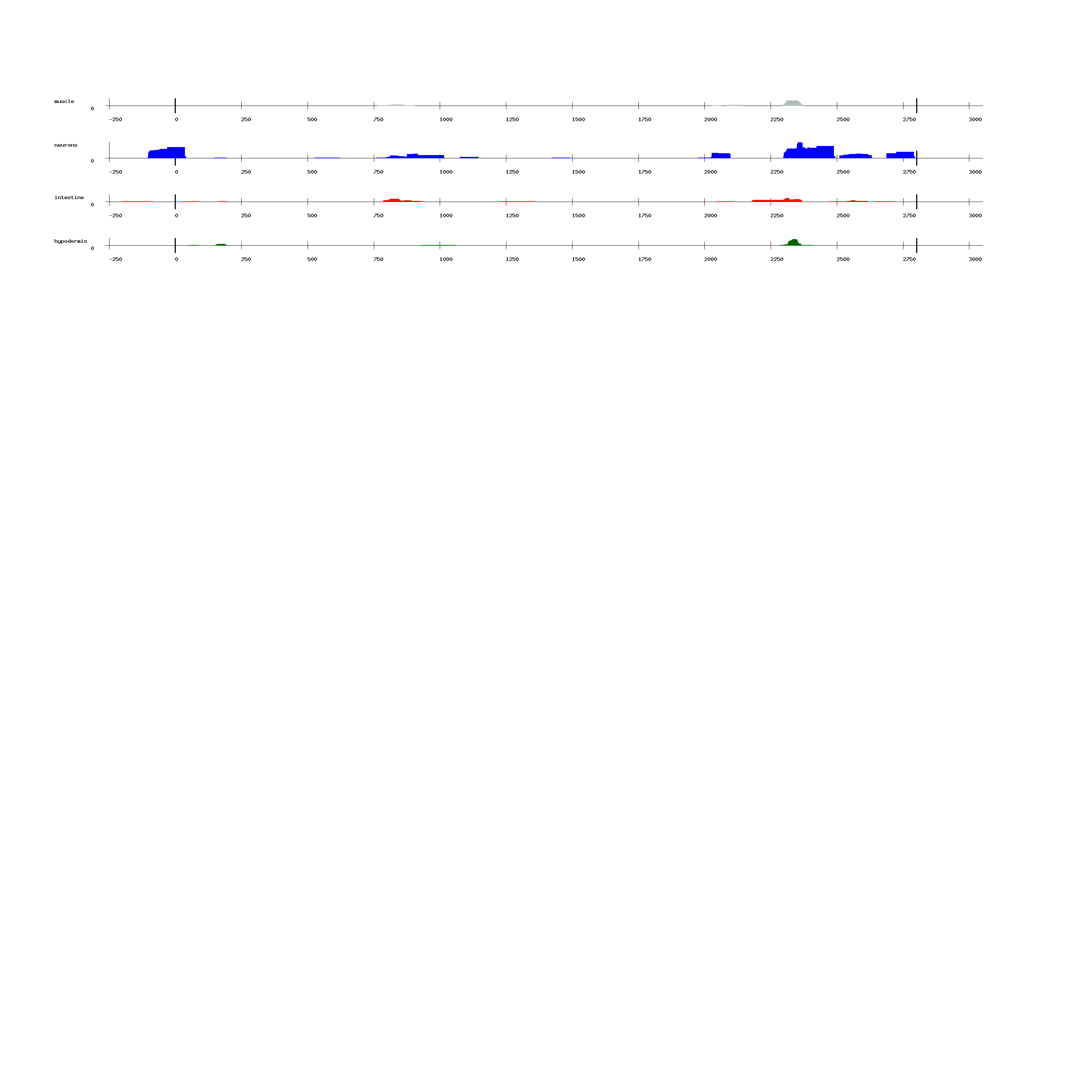

Supplement: Supplementary file 1 [file ijms-24-02970-s001.zip › Supplementary Data S2/1.3763157-3765957.png]

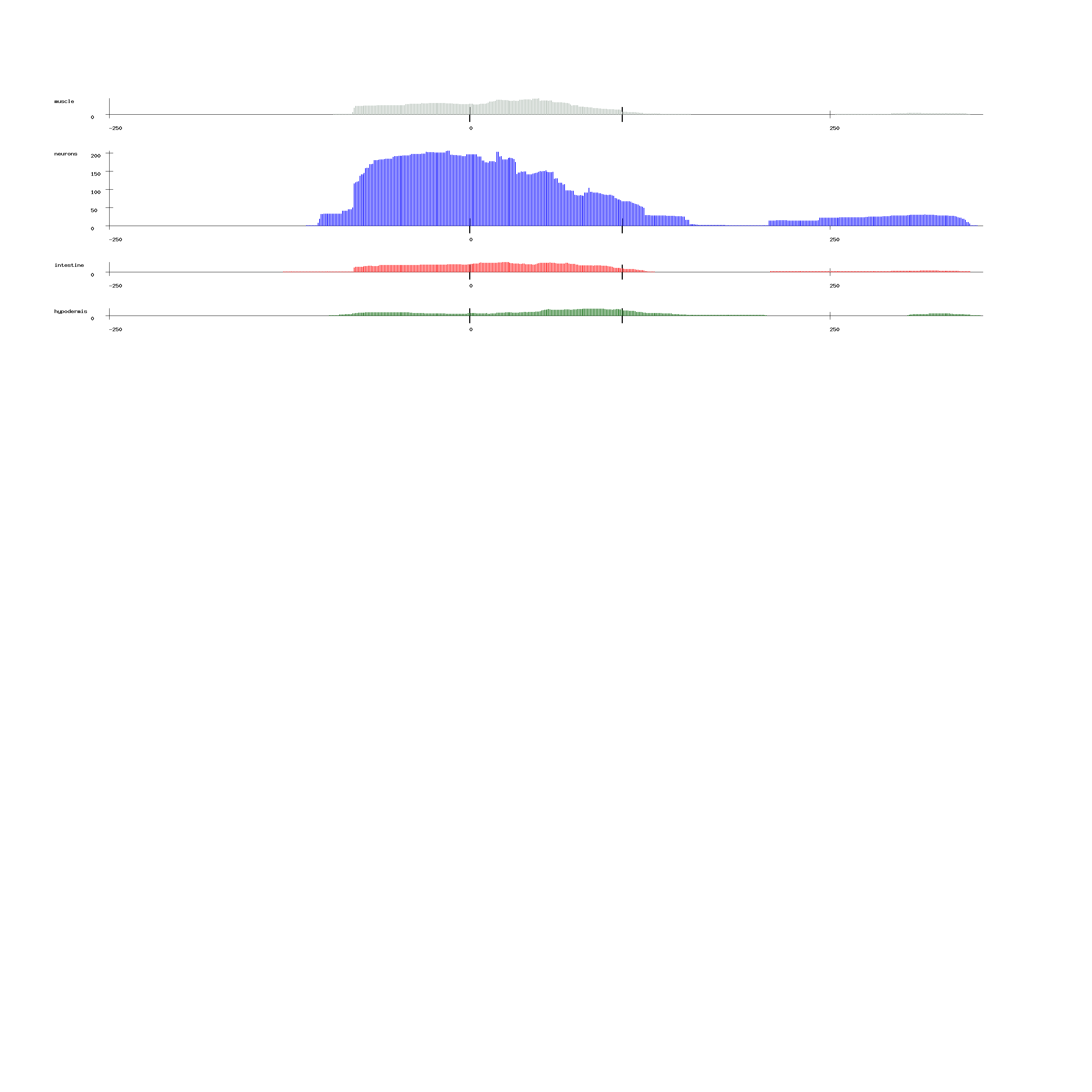

Supplement: Supplementary file 1 [file ijms-24-02970-s001.zip › Supplementary Data S2/1.3980324-3980429.png]

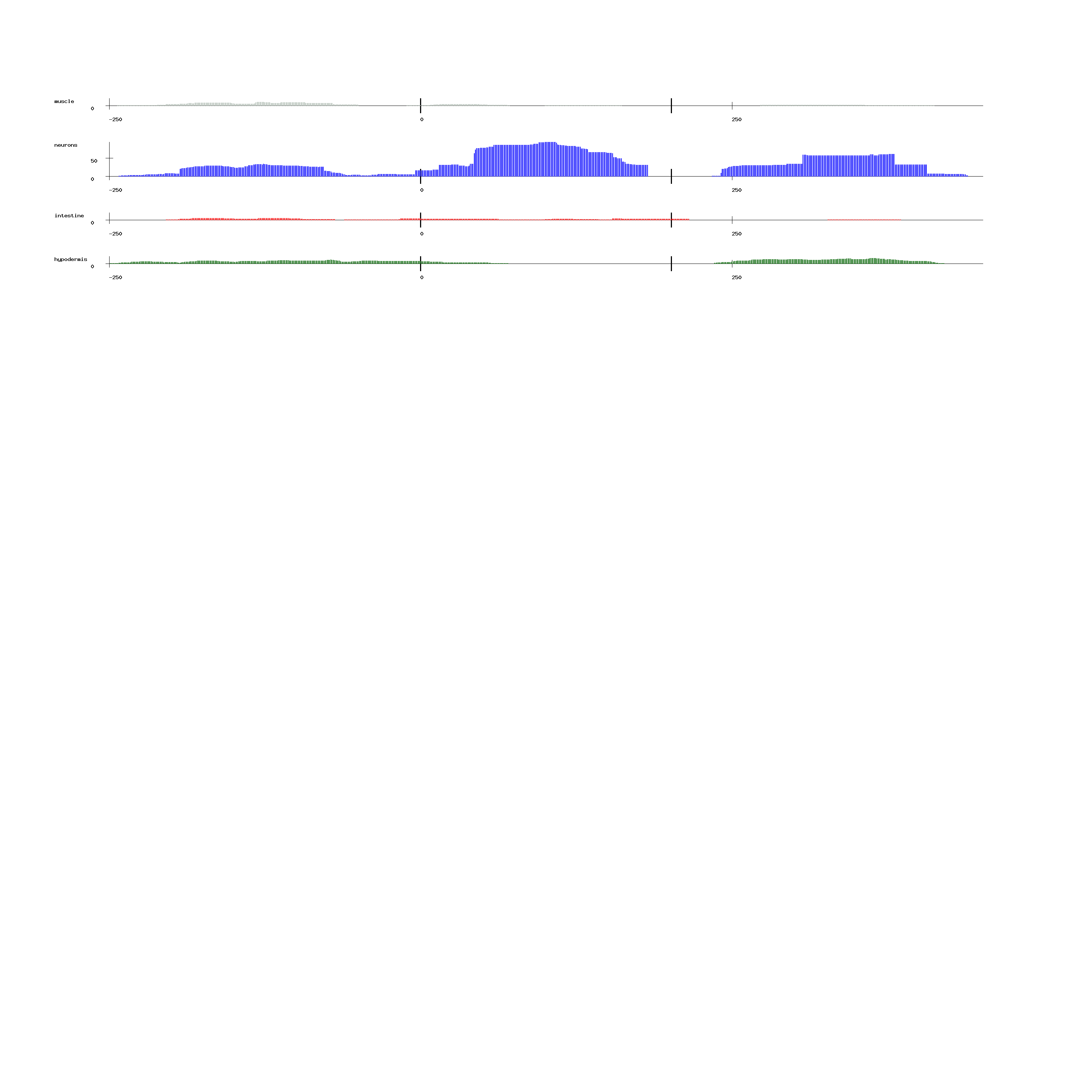

Supplement: Supplementary file 1 [file ijms-24-02970-s001.zip › Supplementary Data S2/1.3980821-3981021.png]

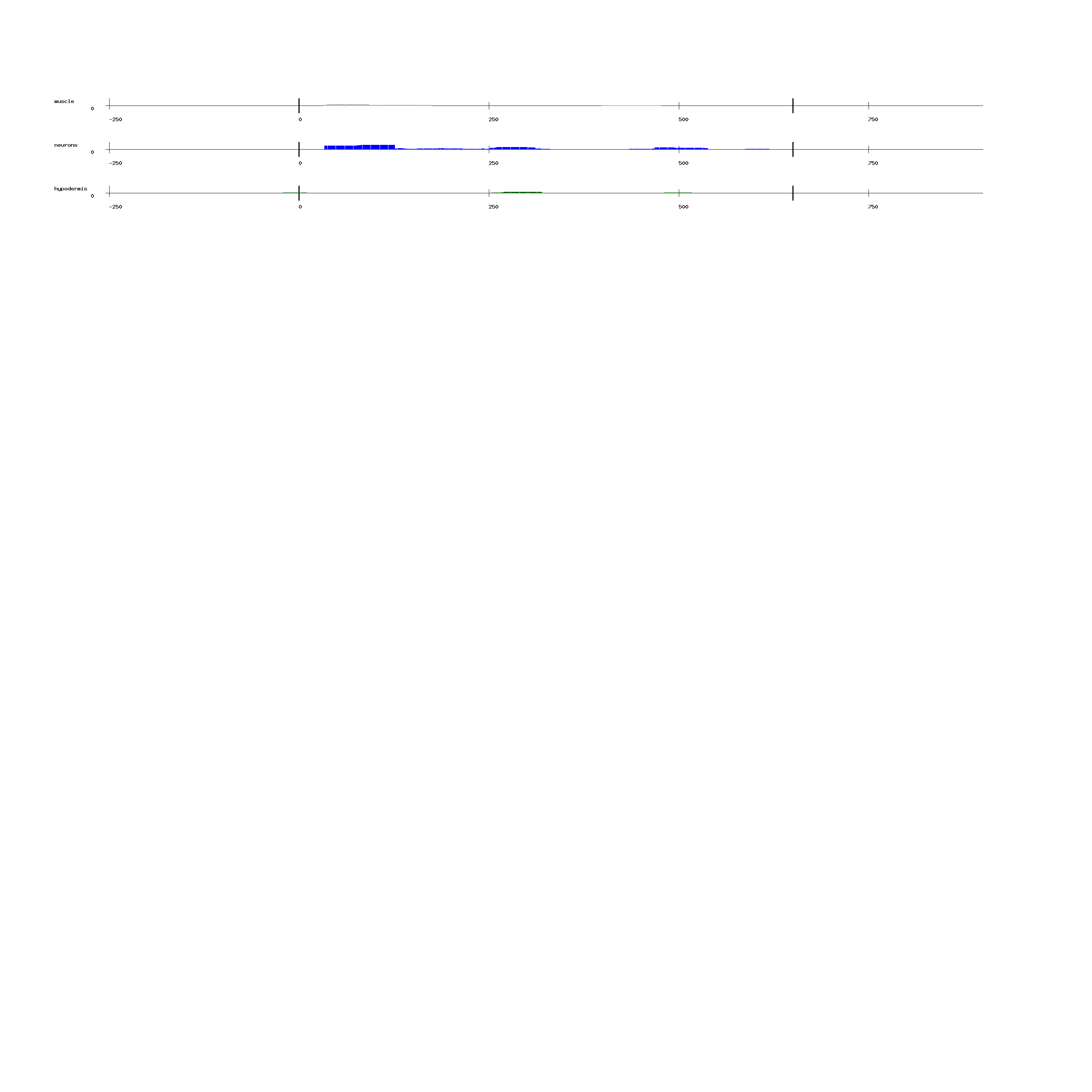

Supplement: Supplementary file 1 [file ijms-24-02970-s001.zip › Supplementary Data S2/1.3990050-3990699.png]

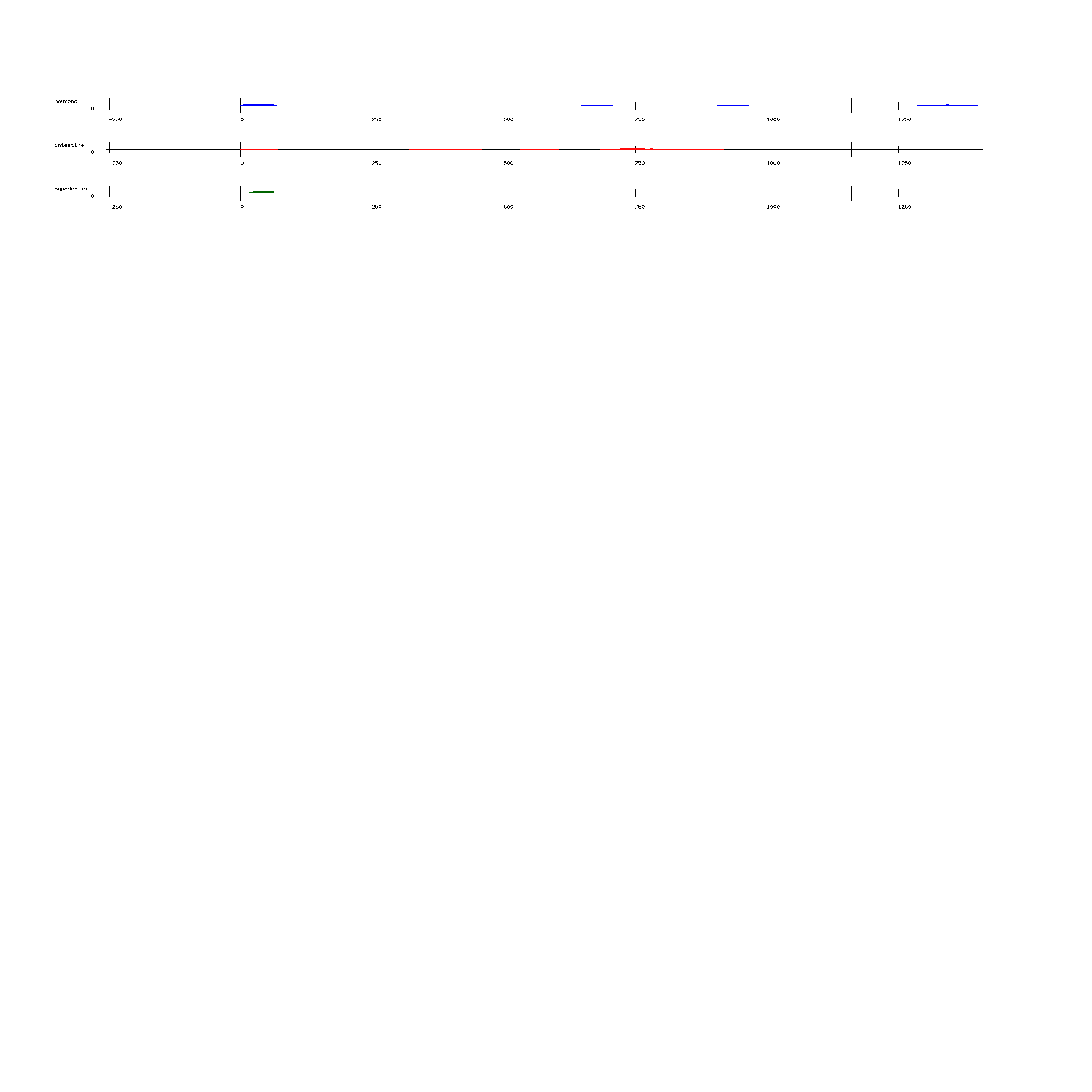

Supplement: Supplementary file 1 [file ijms-24-02970-s001.zip › Supplementary Data S2/1.4118875-4120034.png]

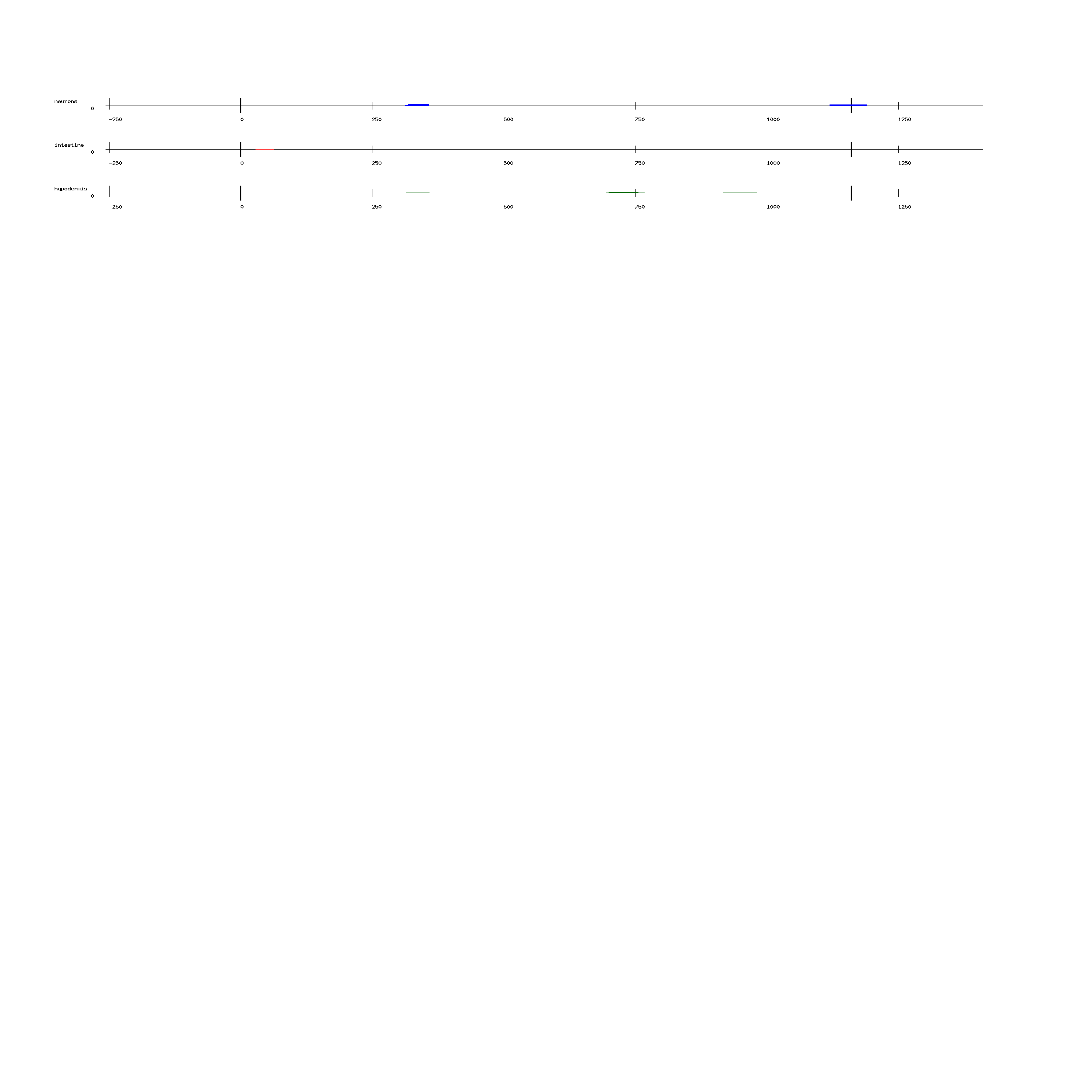

Supplement: Supplementary file 1 [file ijms-24-02970-s001.zip › Supplementary Data S2/1.4280022-4281181.png]

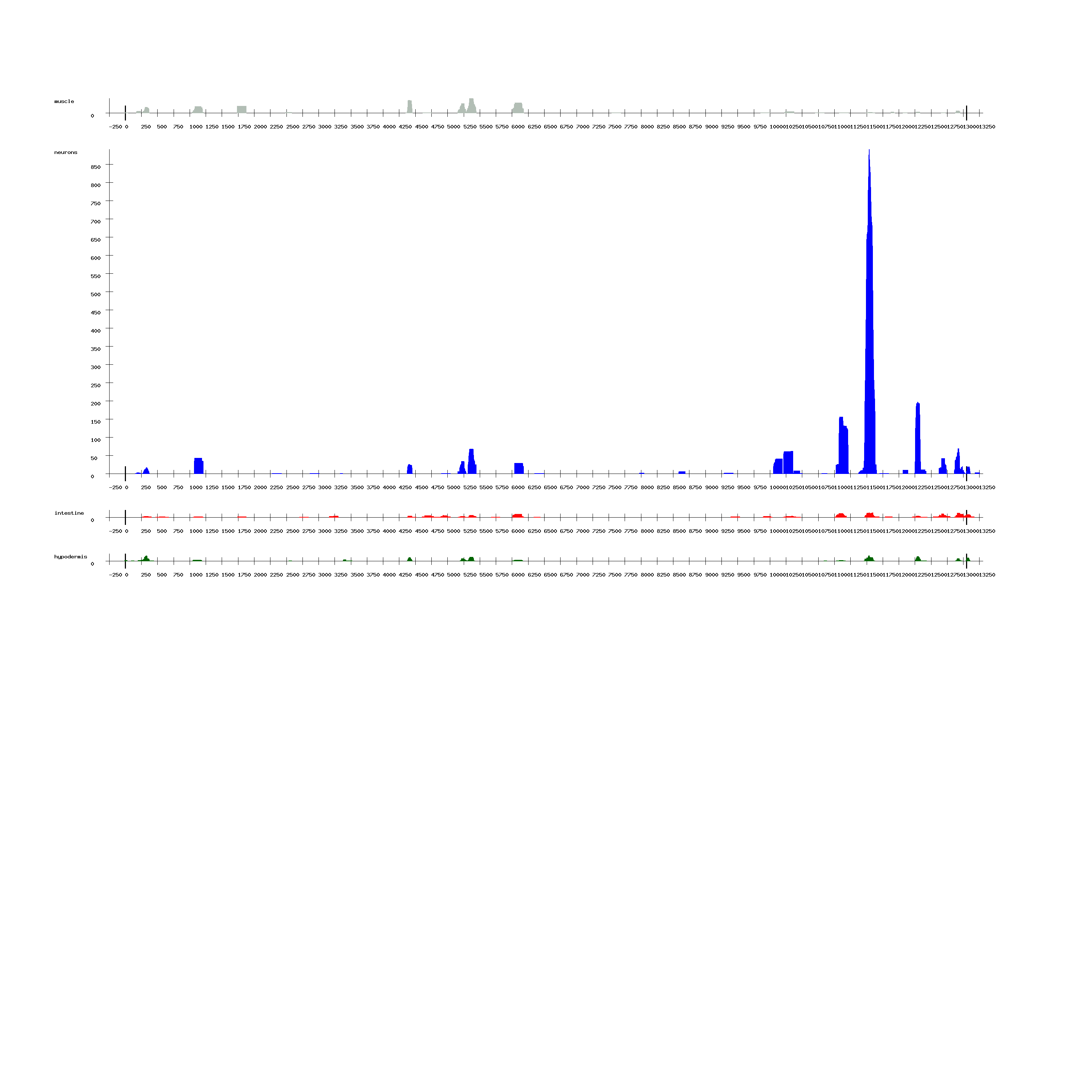

Supplement: Supplementary file 1 [file ijms-24-02970-s001.zip › Supplementary Data S2/1.4281487-4294537.png]

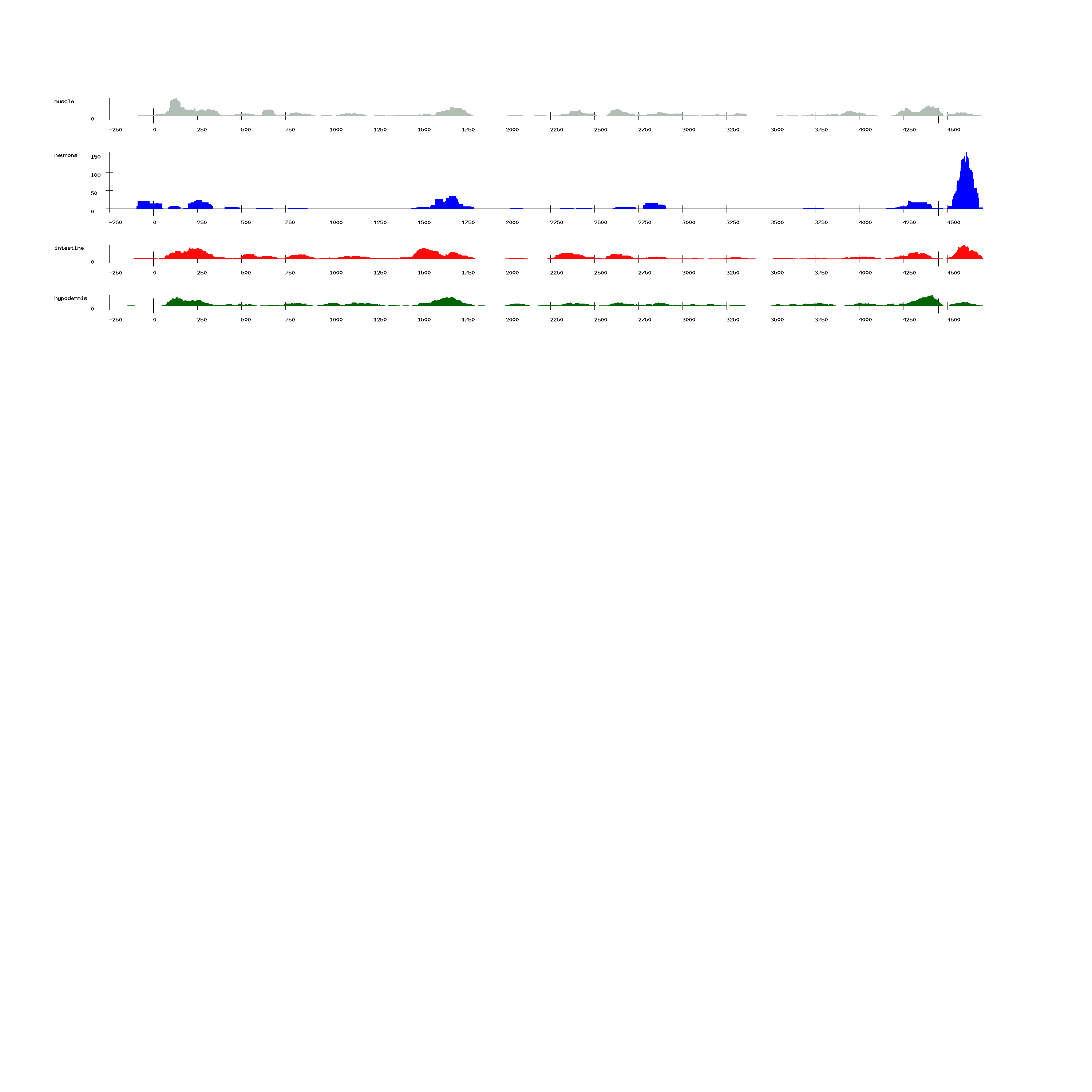

Supplement: Supplementary file 1 [file ijms-24-02970-s001.zip › Supplementary Data S2/1.4540371-4544820.png]

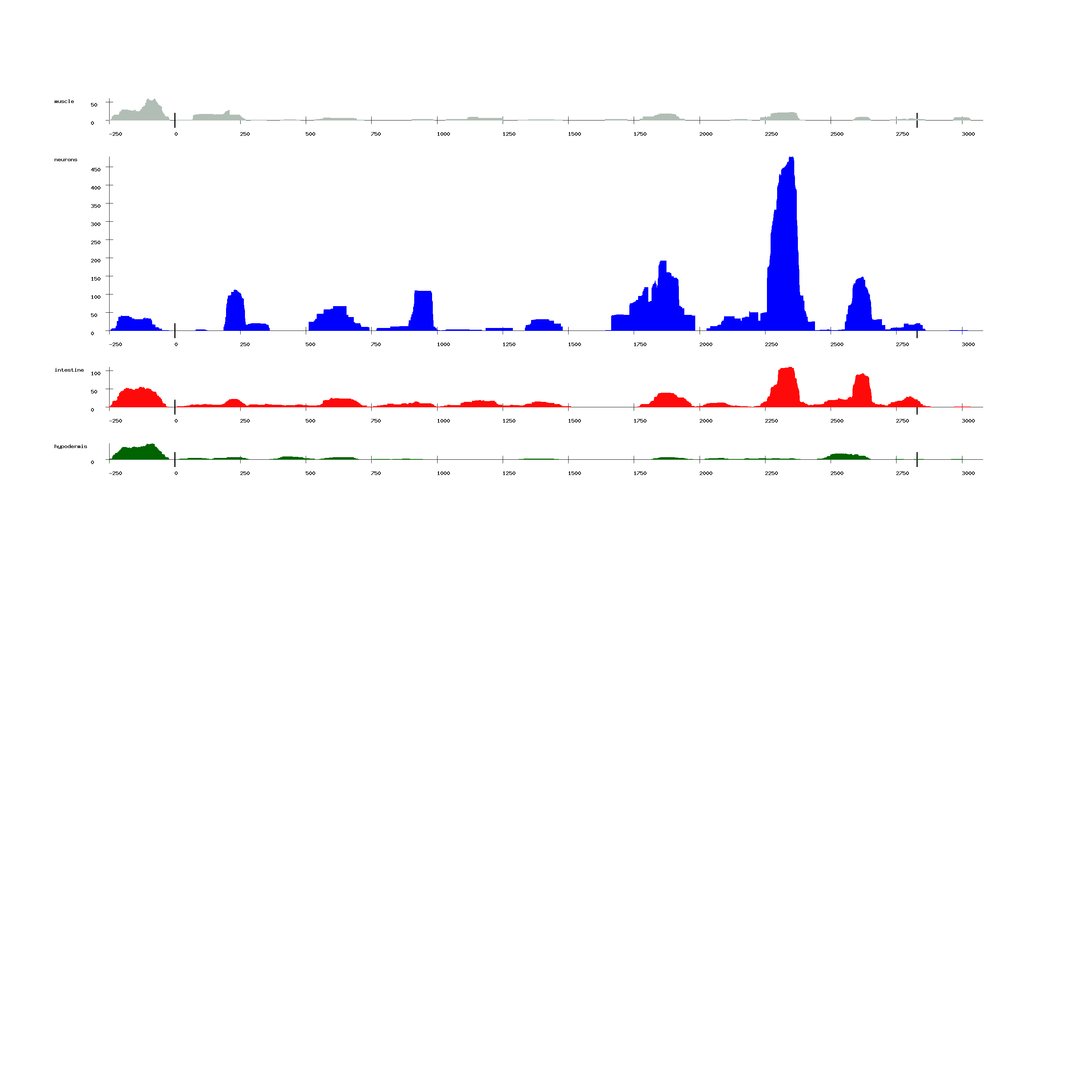

Supplement: Supplementary file 1 [file ijms-24-02970-s001.zip › Supplementary Data S2/1.4544868-4547696.png]

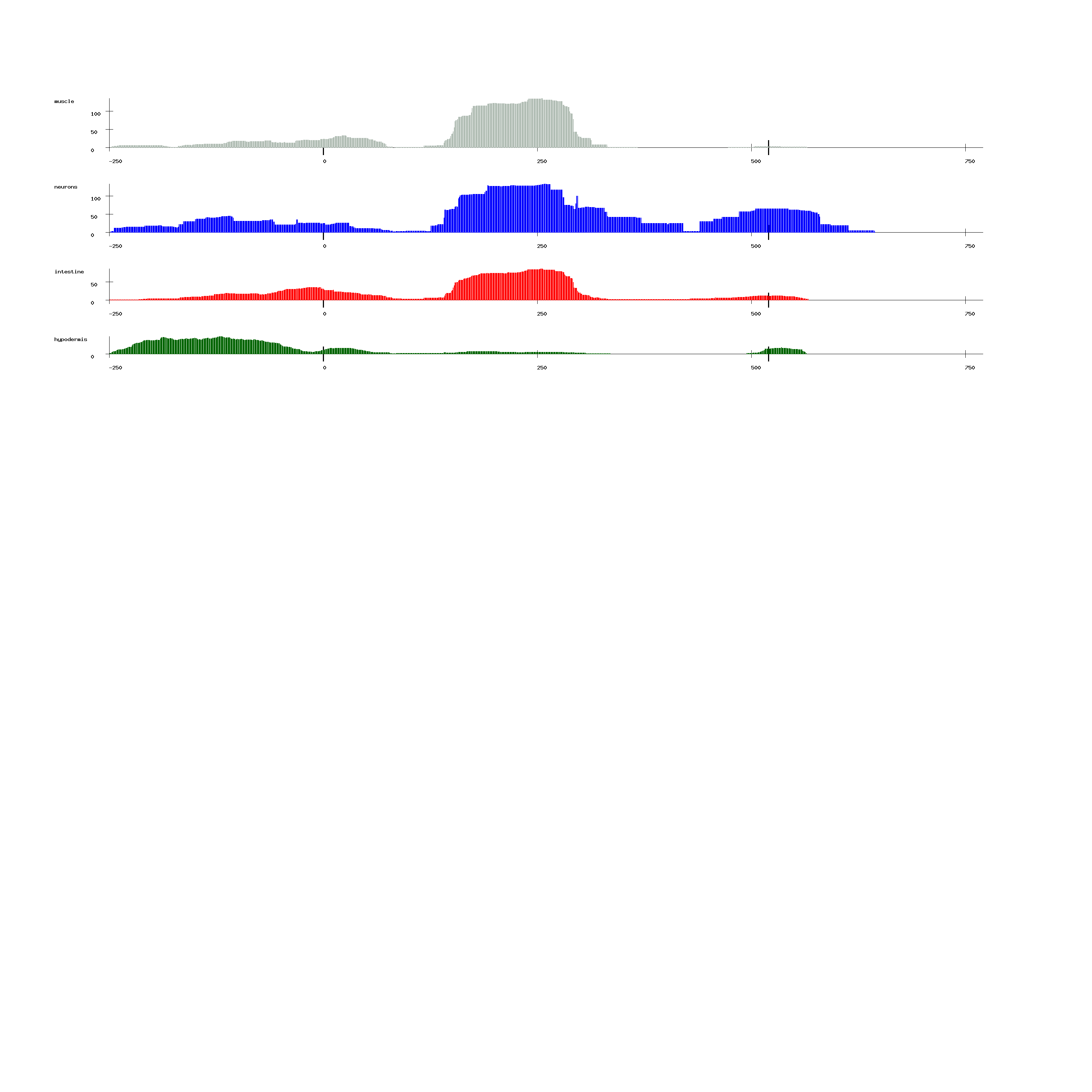

Supplement: Supplementary file 1 [file ijms-24-02970-s001.zip › Supplementary Data S2/1.4573284-4573803.png]

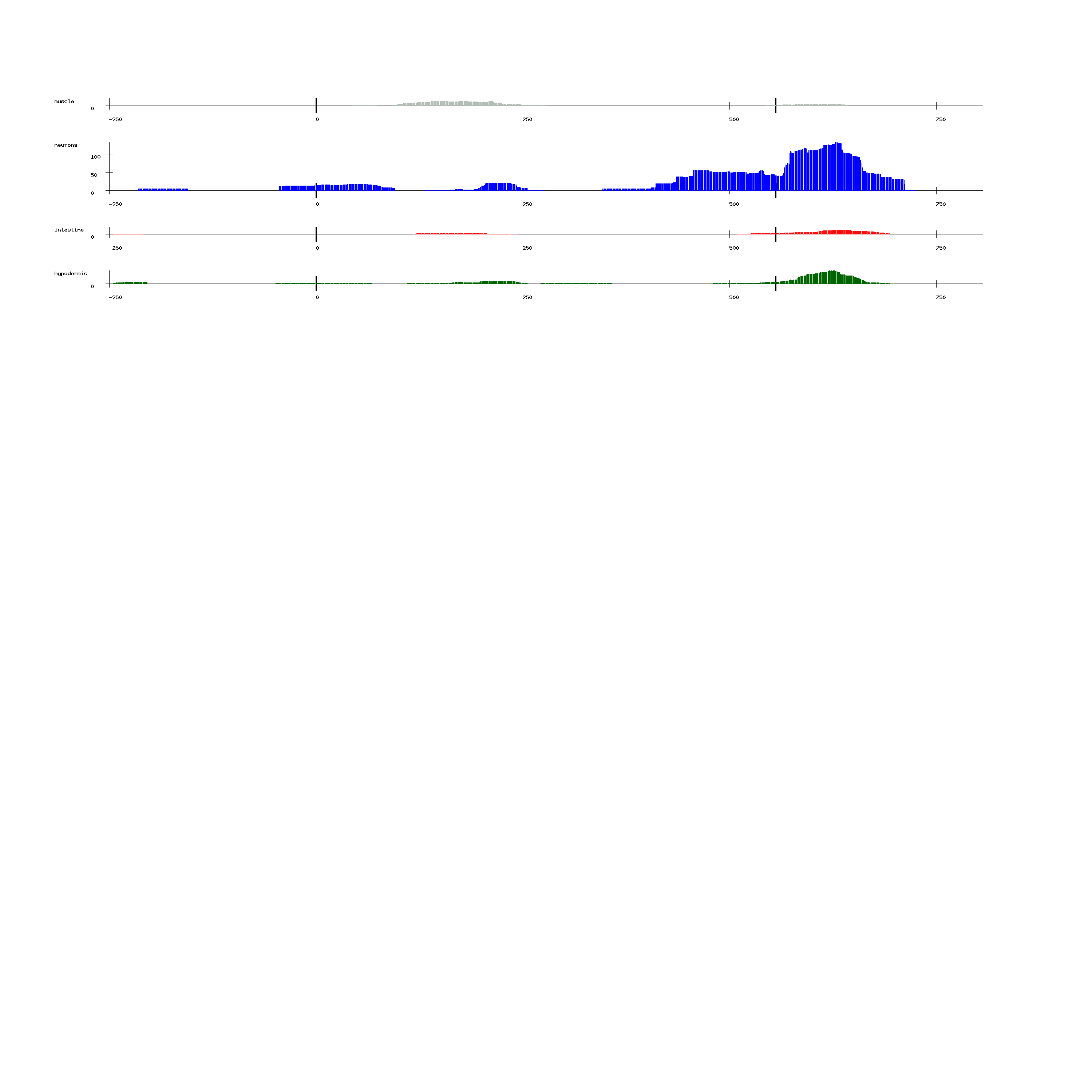

Supplement: Supplementary file 1 [file ijms-24-02970-s001.zip › Supplementary Data S2/1.4574052-4574607.png]

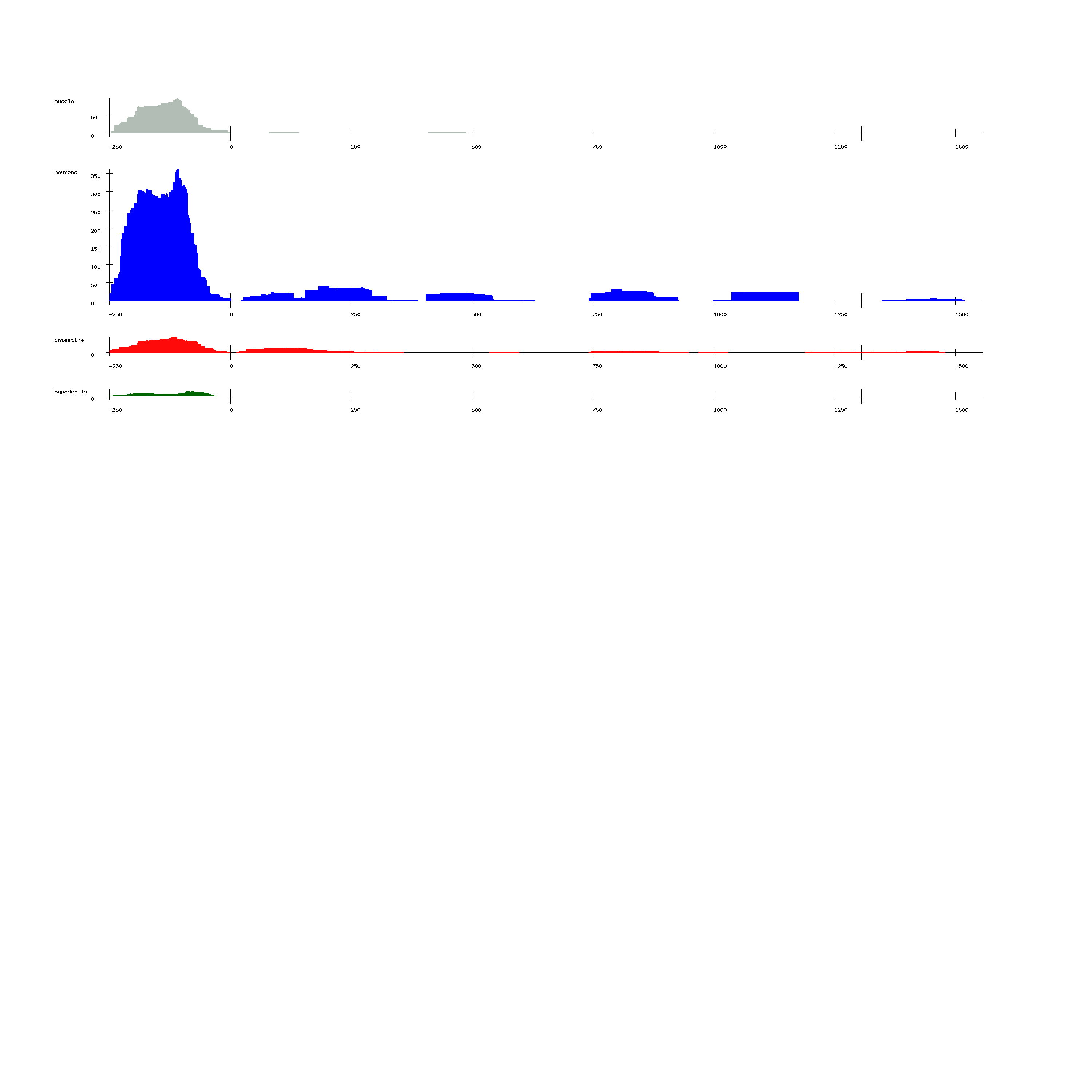

Supplement: Supplementary file 1 [file ijms-24-02970-s001.zip › Supplementary Data S2/1.5152583-5153888.png]

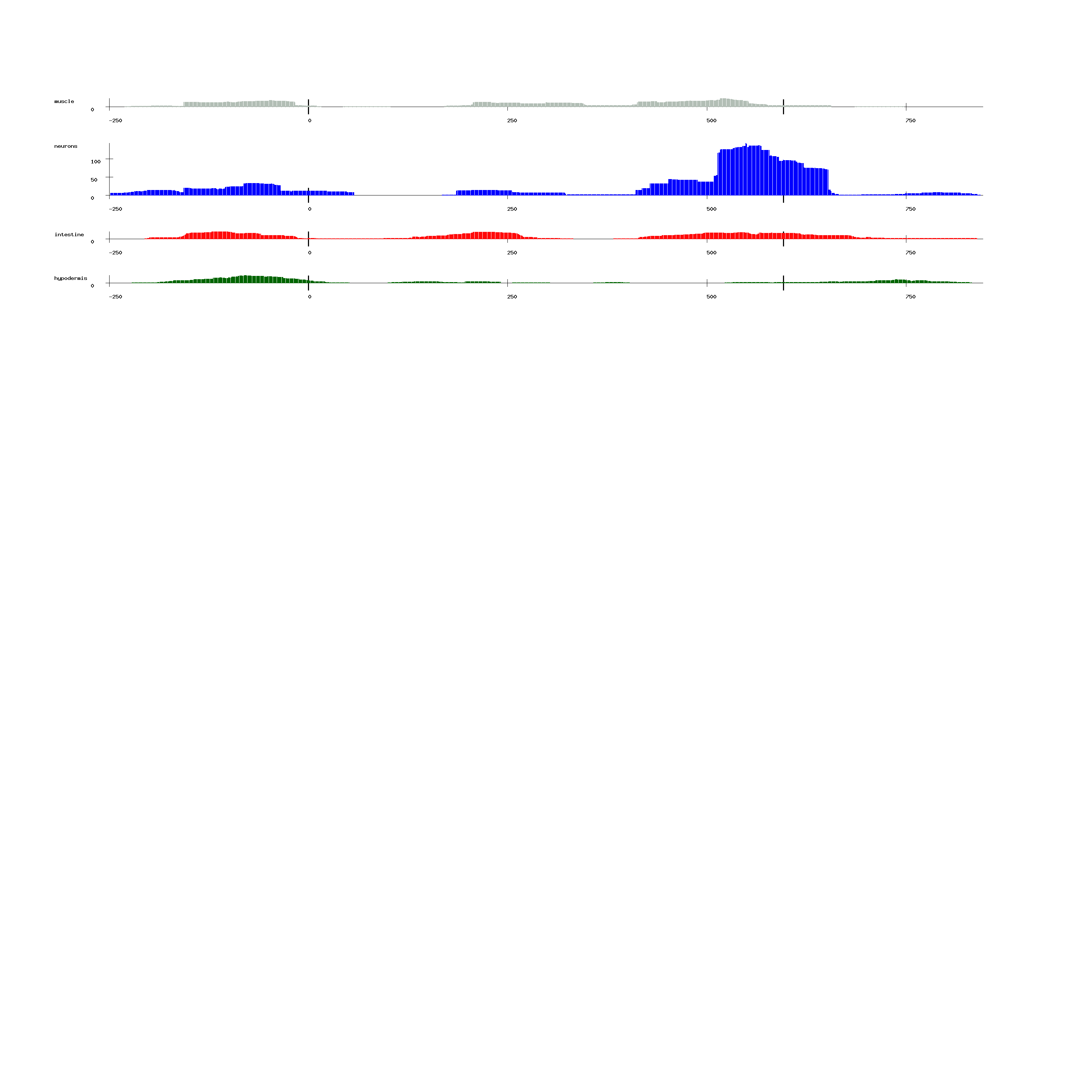

Supplement: Supplementary file 1 [file ijms-24-02970-s001.zip › Supplementary Data S2/1.5640355-5640950.png]

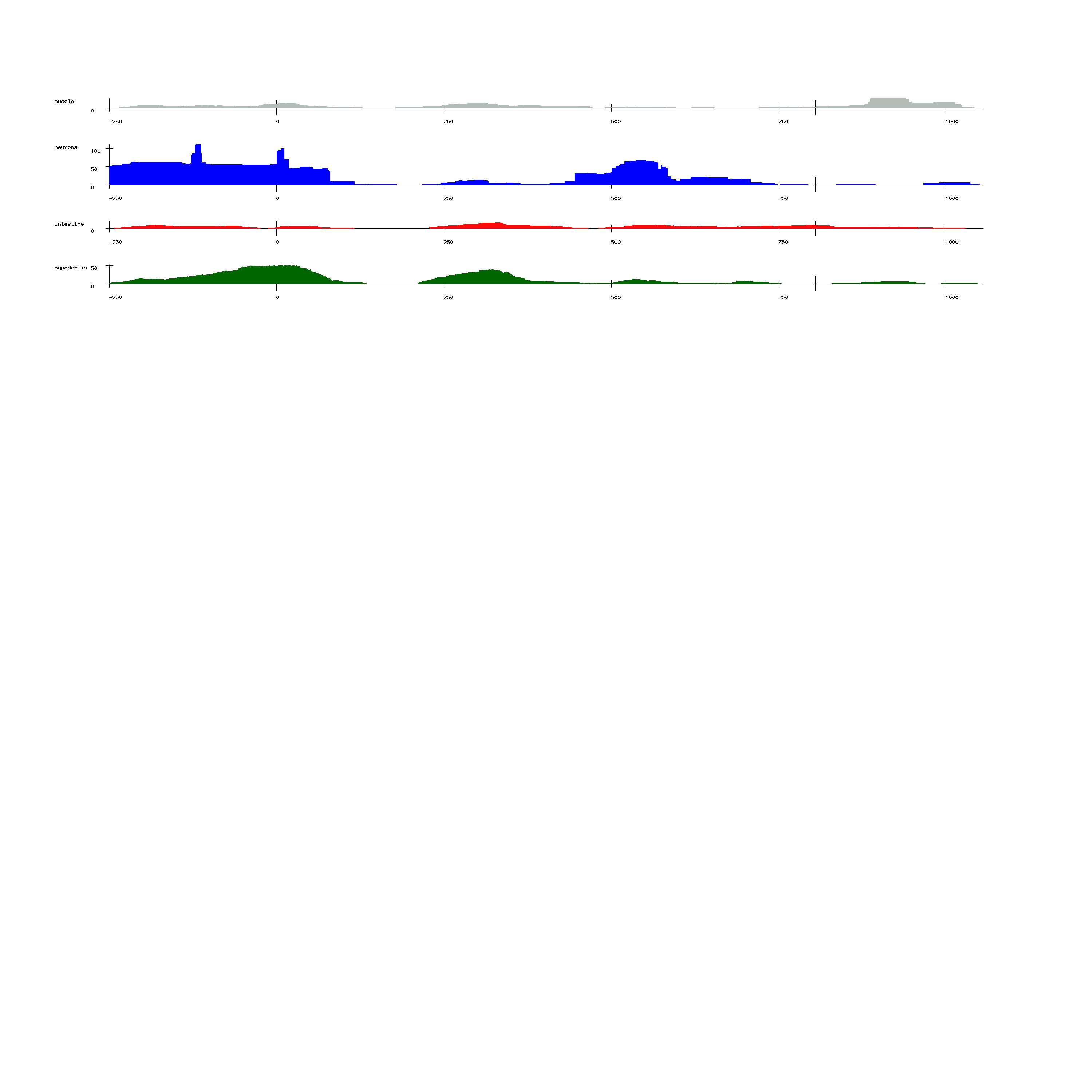

Supplement: Supplementary file 1 [file ijms-24-02970-s001.zip › Supplementary Data S2/1.5975024-5975828.png]

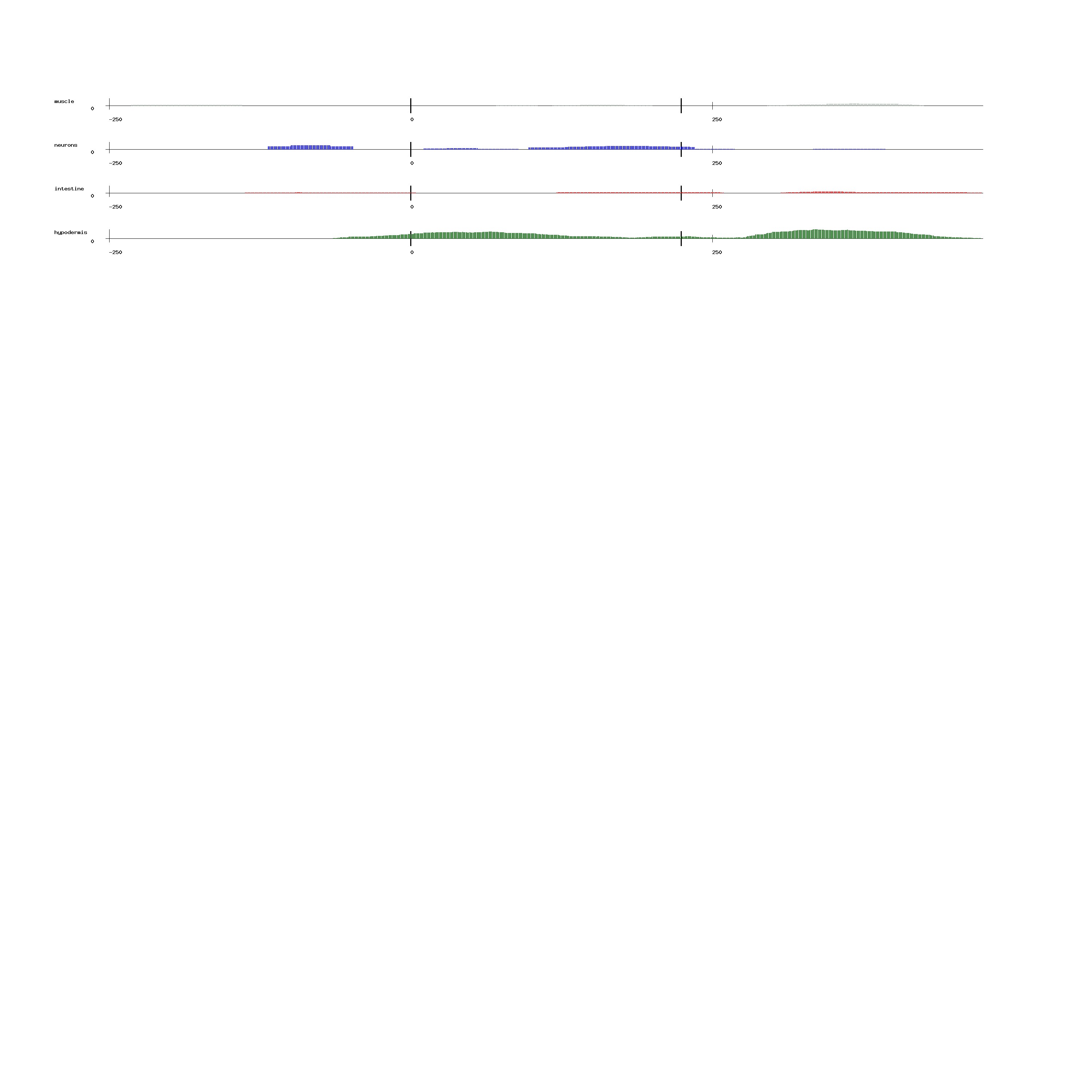

Supplement: Supplementary file 1 [file ijms-24-02970-s001.zip › Supplementary Data S2/1.5977228-5977451.png]

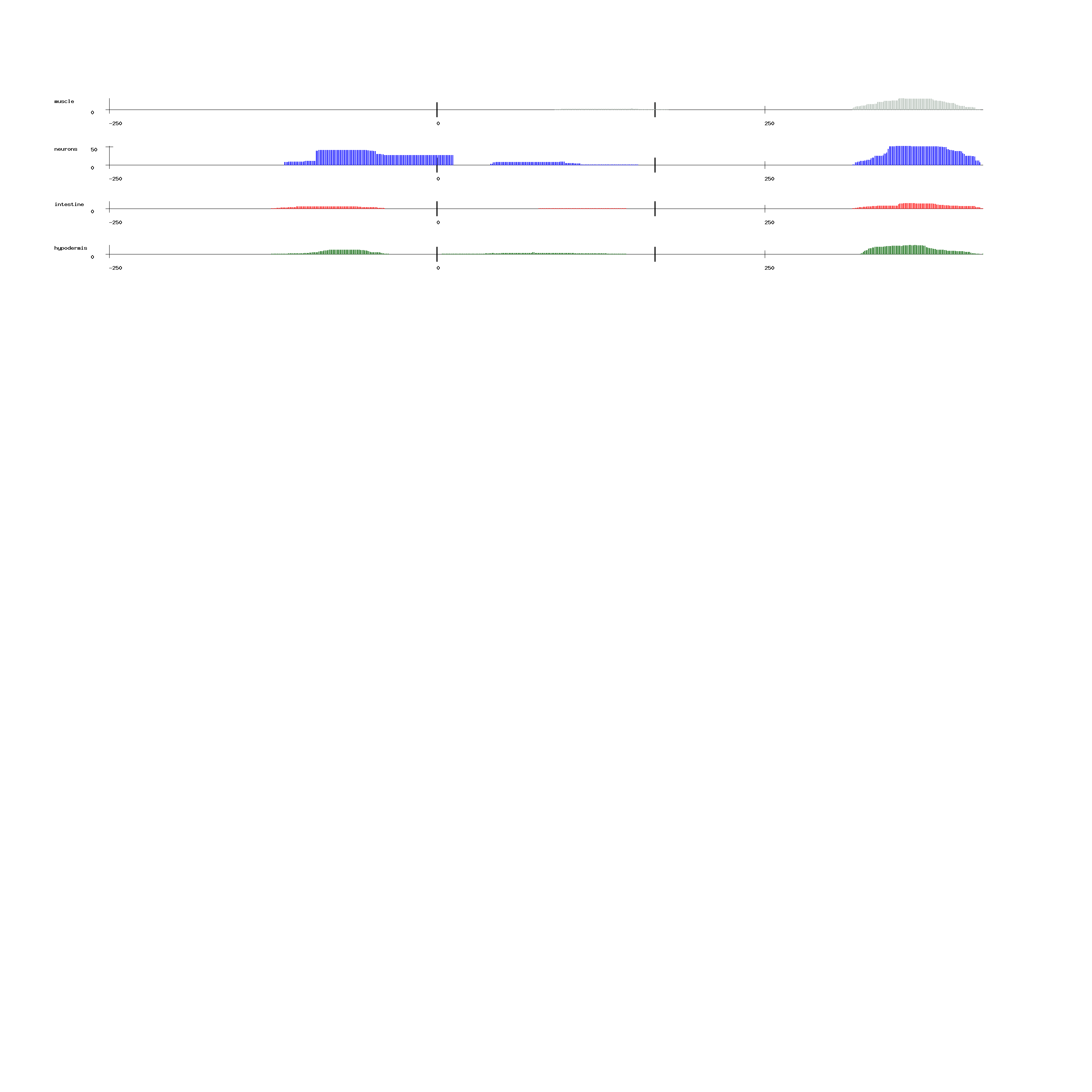

Supplement: Supplementary file 1 [file ijms-24-02970-s001.zip › Supplementary Data S2/1.6647571-6647736.png]

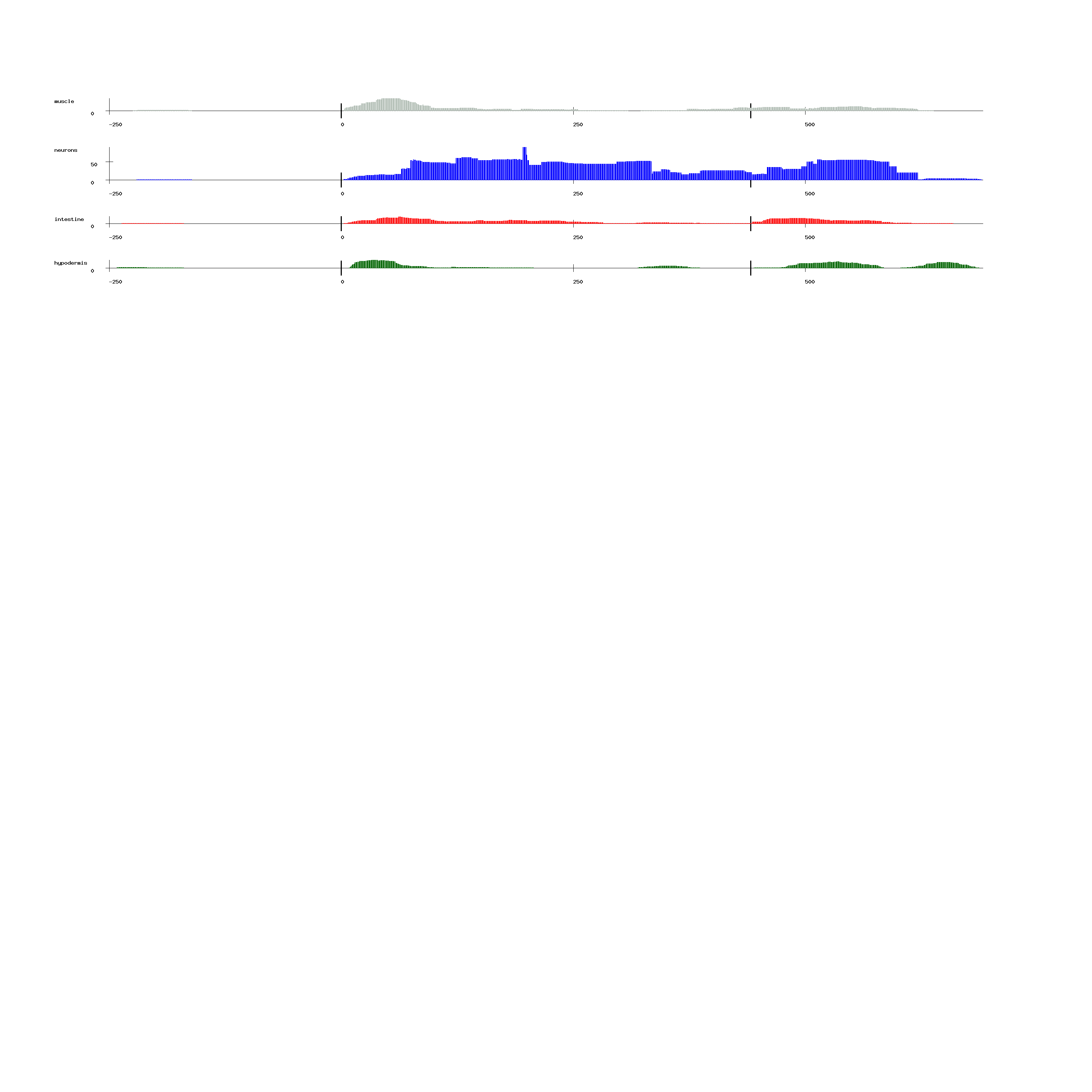

Supplement: Supplementary file 1 [file ijms-24-02970-s001.zip › Supplementary Data S2/1.6647885-6648325.png]

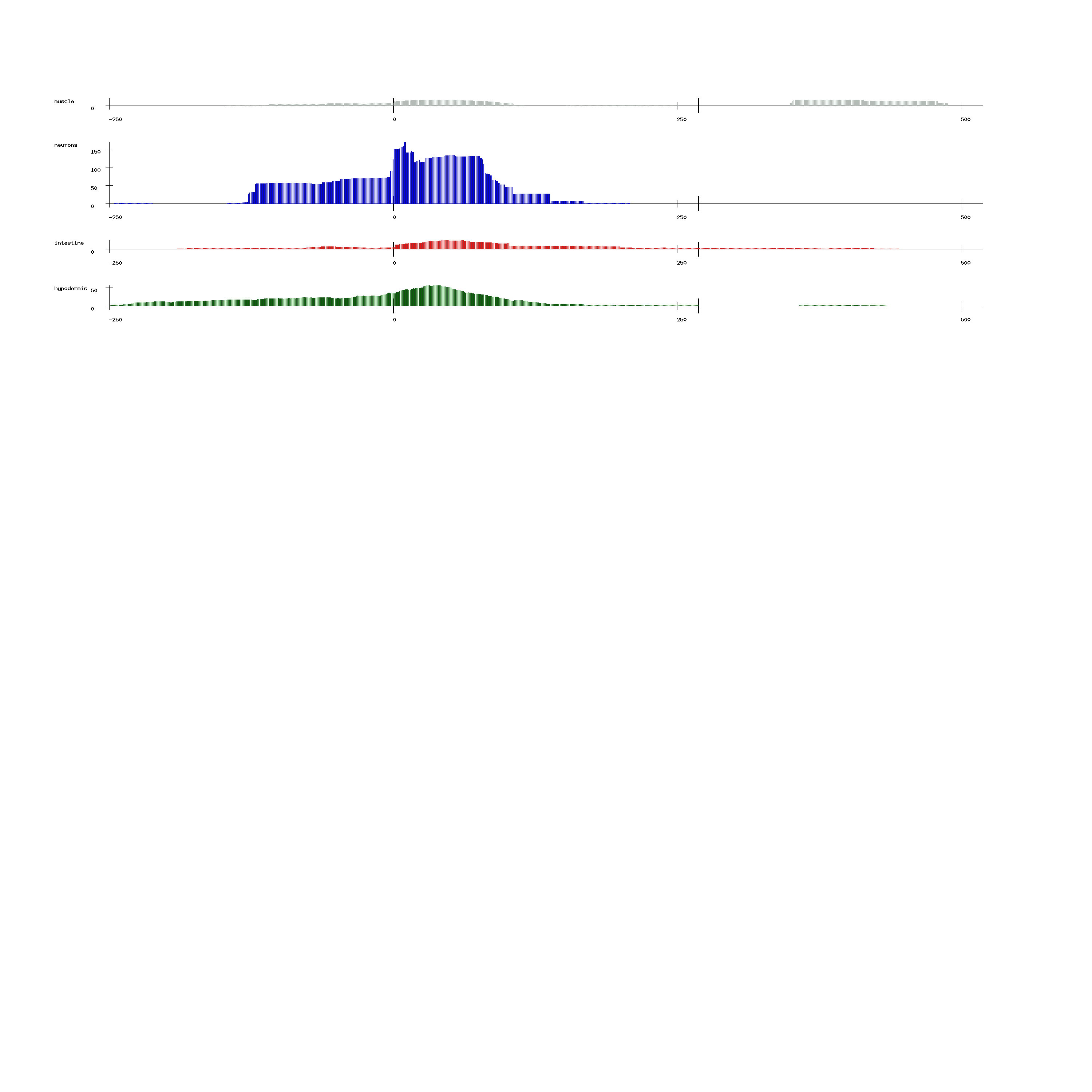

Supplement: Supplementary file 1 [file ijms-24-02970-s001.zip › Supplementary Data S2/1.676497-676765.png]

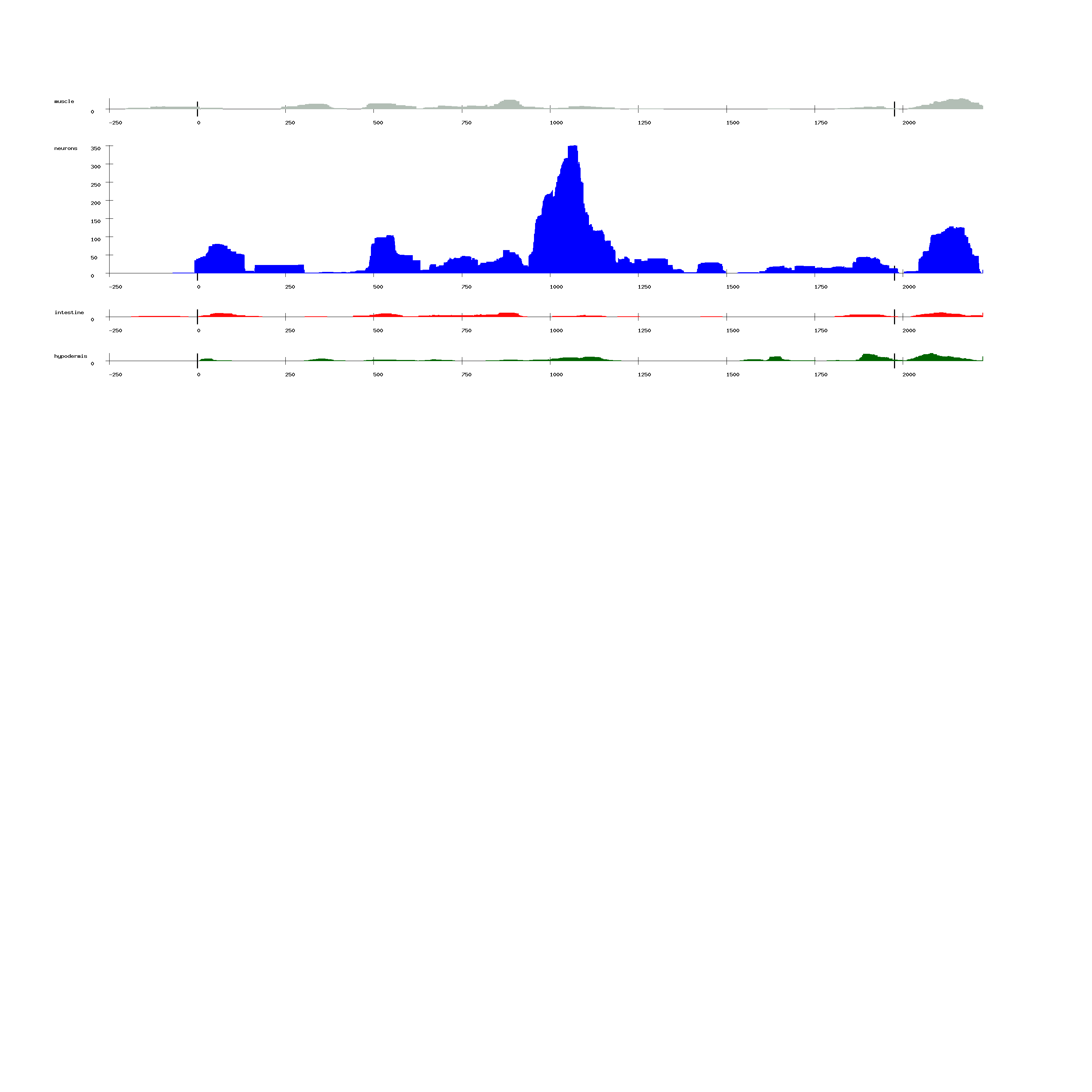

Supplement: Supplementary file 1 [file ijms-24-02970-s001.zip › Supplementary Data S2/1.6771968-6773943.png]

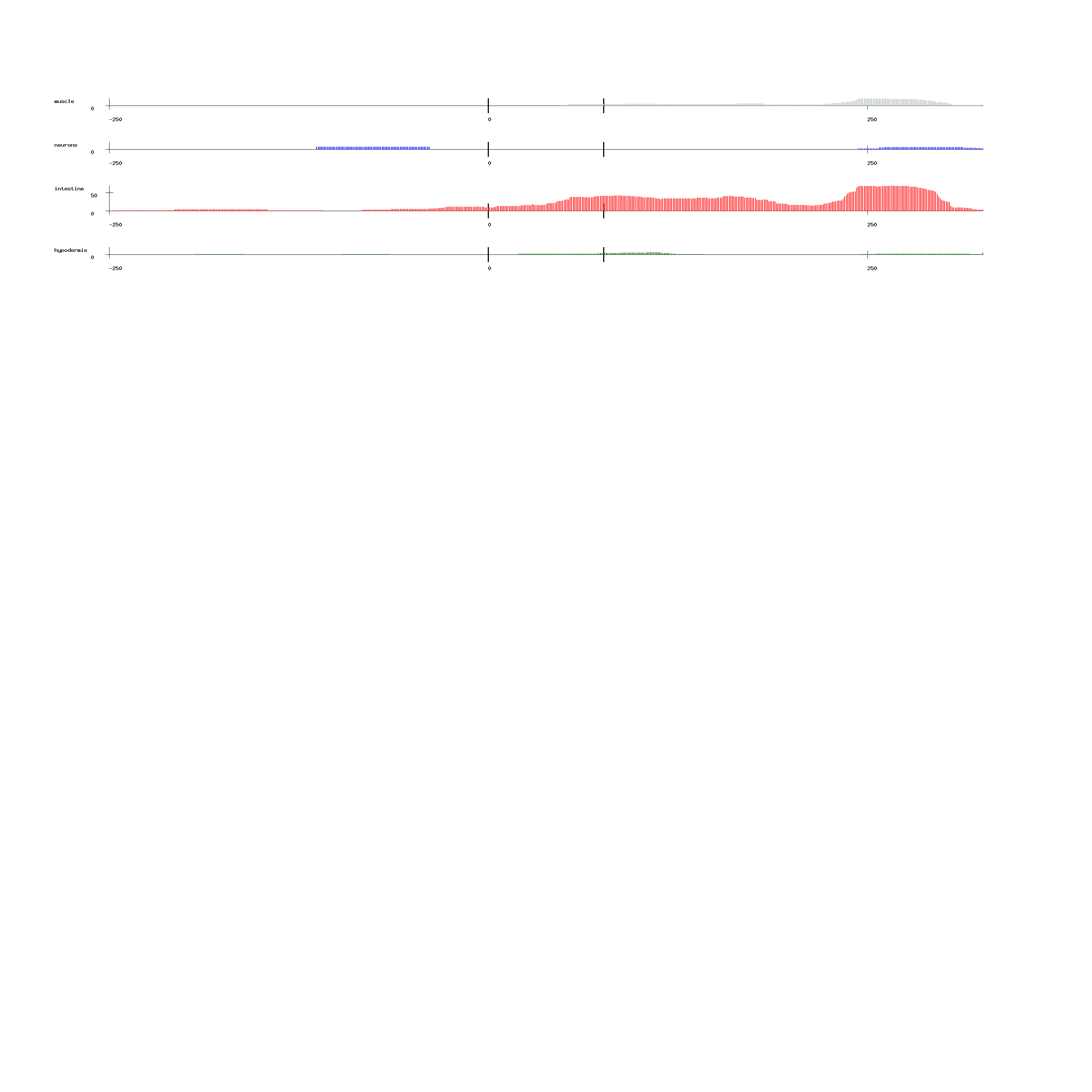

Supplement: Supplementary file 1 [file ijms-24-02970-s001.zip › Supplementary Data S2/1.6854524-6854599.png]

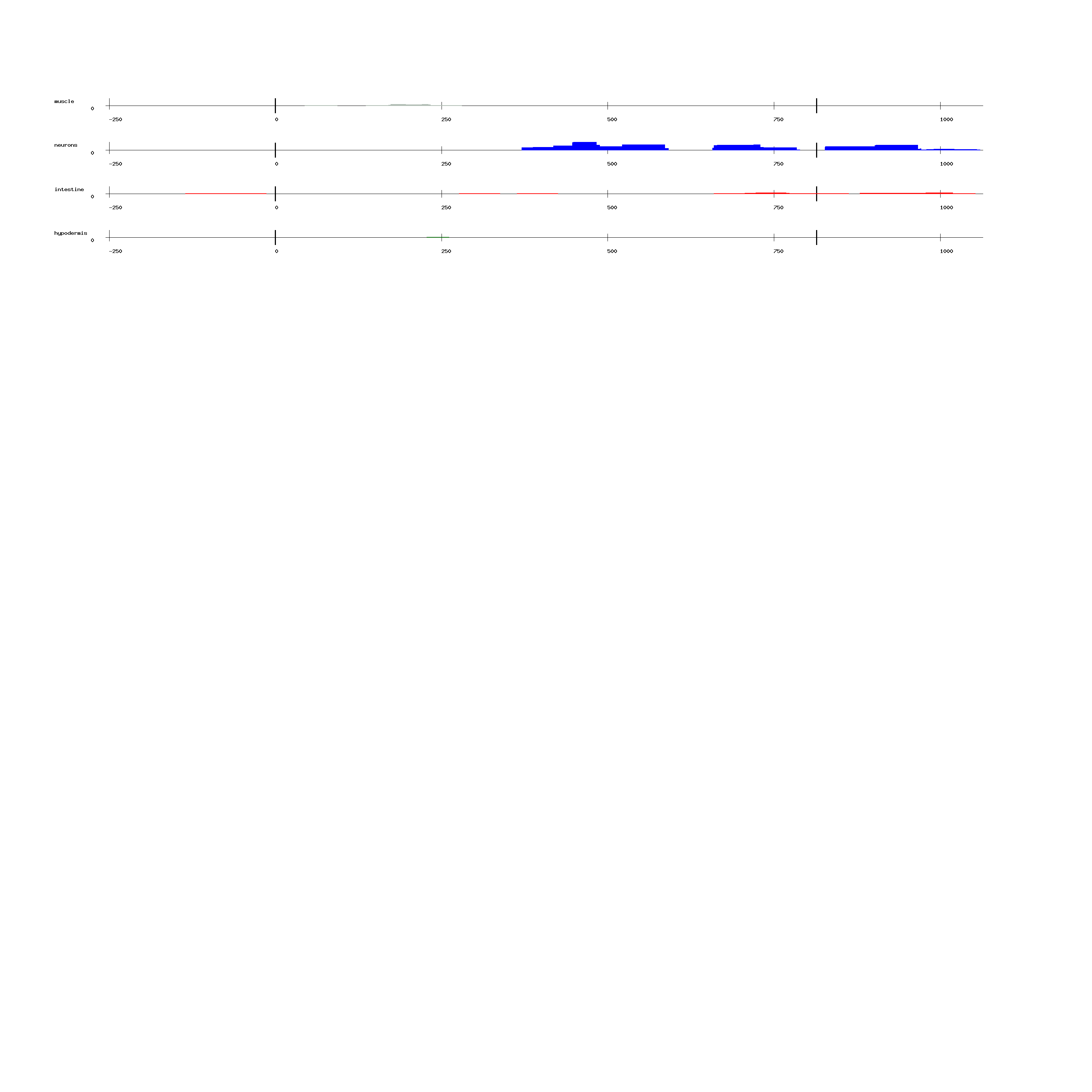

Supplement: Supplementary file 1 [file ijms-24-02970-s001.zip › Supplementary Data S2/1.687047-687860.png]

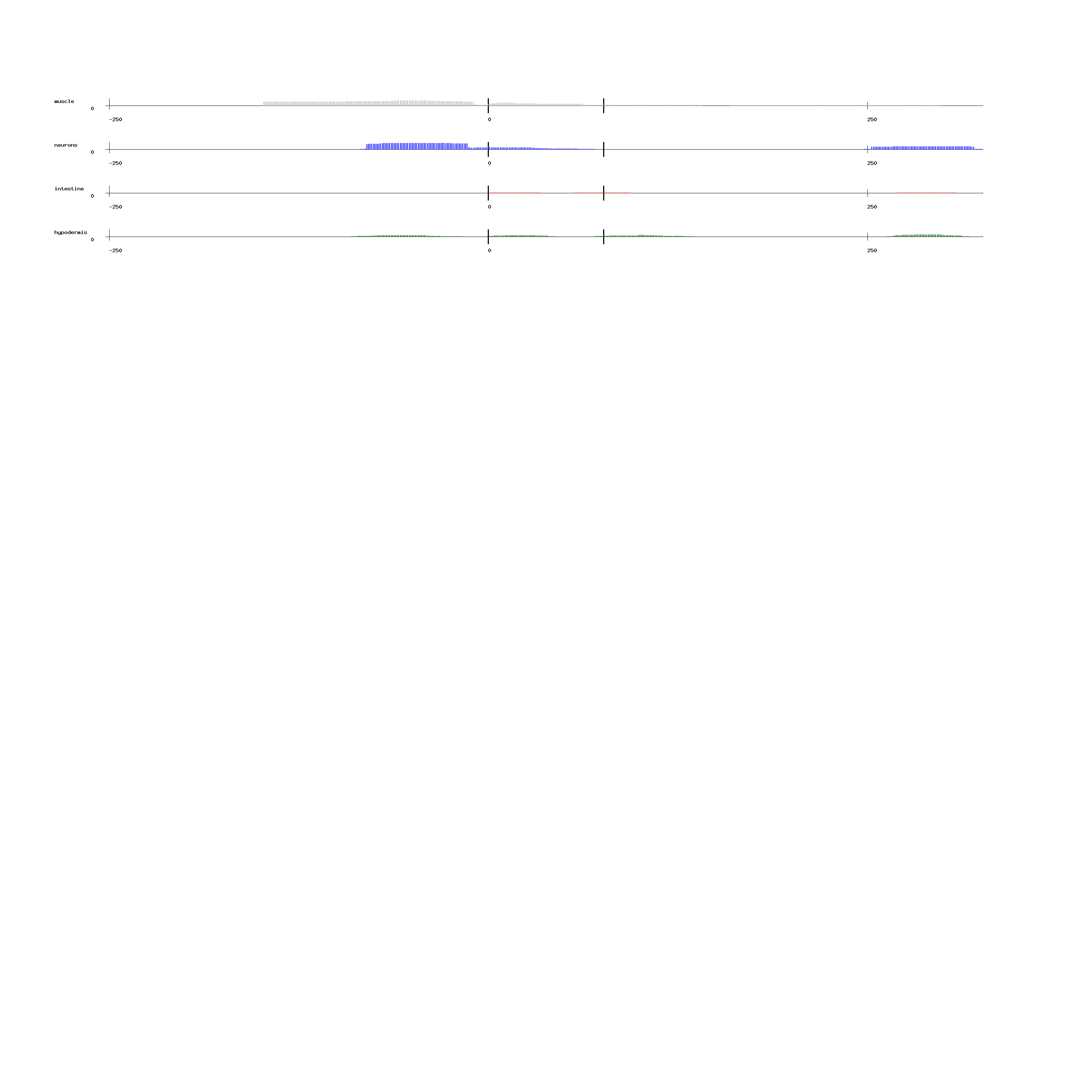

Supplement: Supplementary file 1 [file ijms-24-02970-s001.zip › Supplementary Data S2/1.7740676-7740751.png]

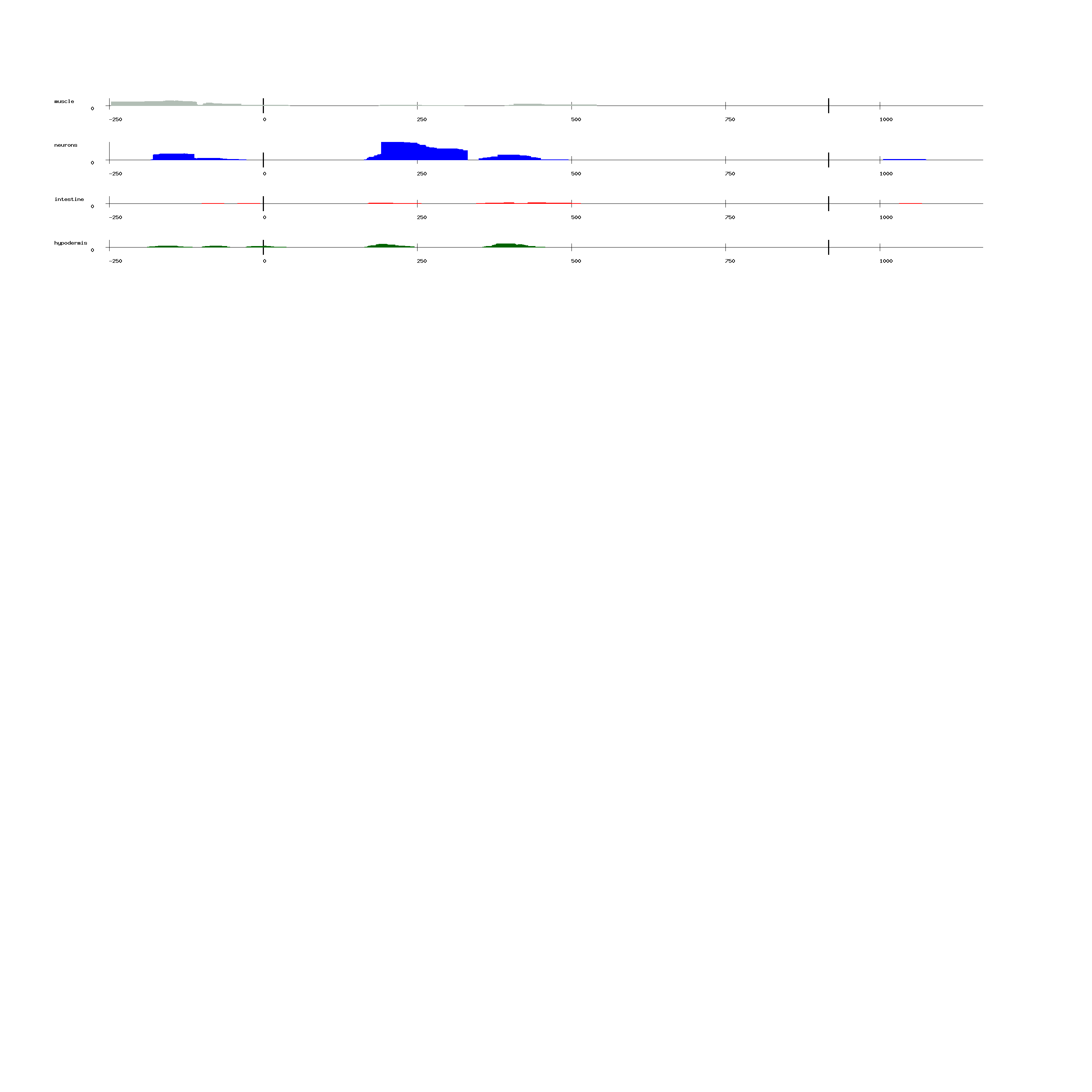

Supplement: Supplementary file 1 [file ijms-24-02970-s001.zip › Supplementary Data S2/1.7740774-7741690.png]

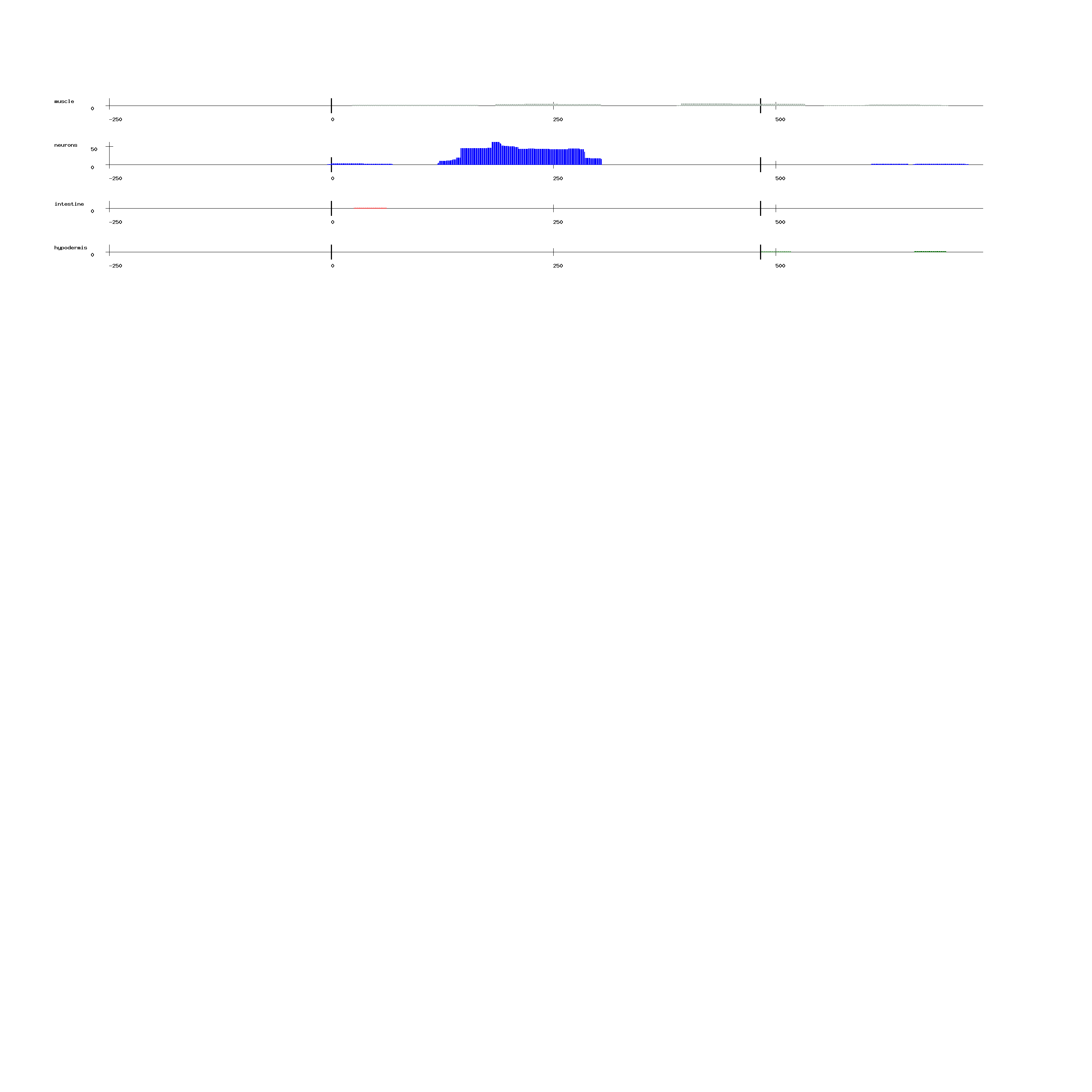

Supplement: Supplementary file 1 [file ijms-24-02970-s001.zip › Supplementary Data S2/1.7741780-7742262.png]

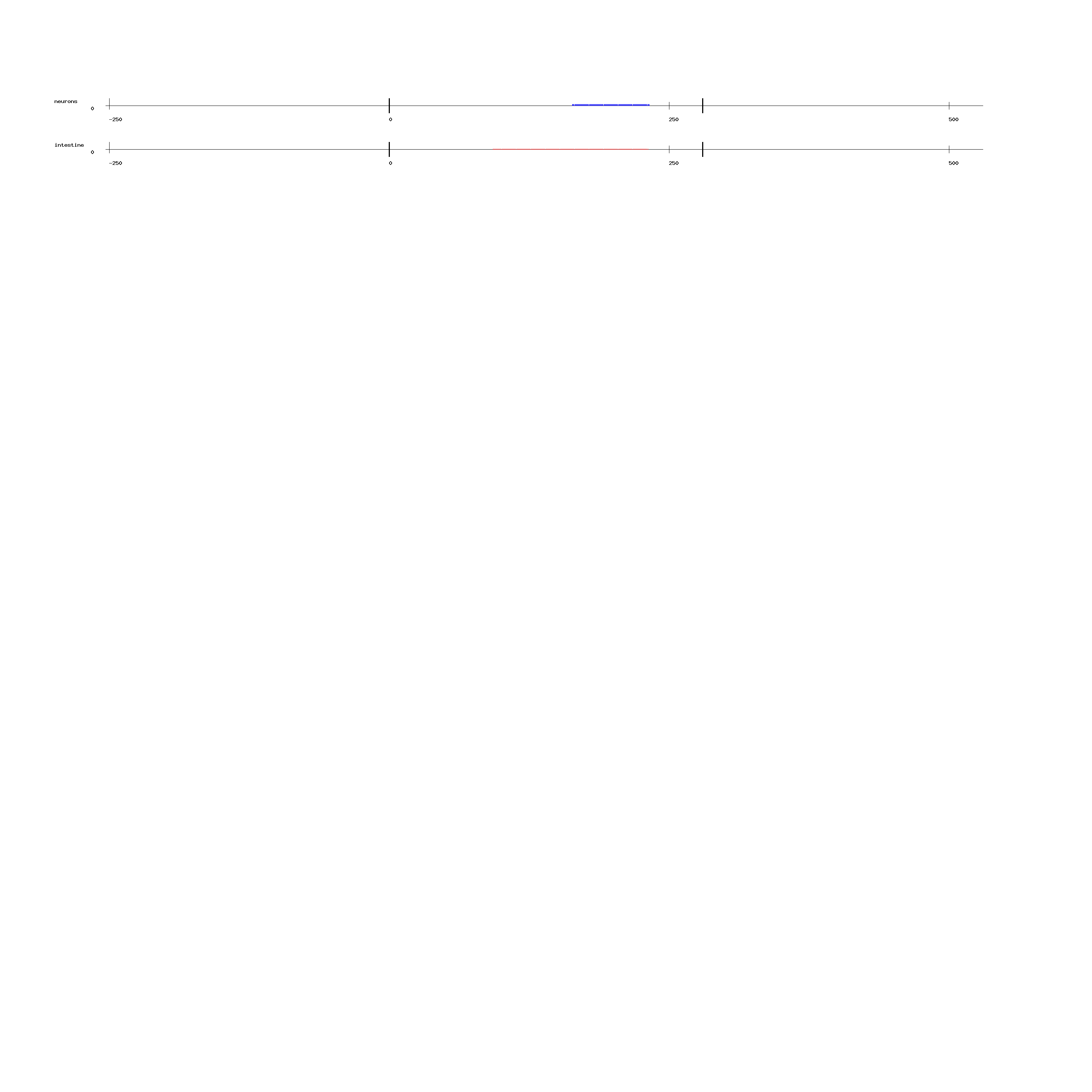

Supplement: Supplementary file 1 [file ijms-24-02970-s001.zip › Supplementary Data S2/1.778056-778335.png]

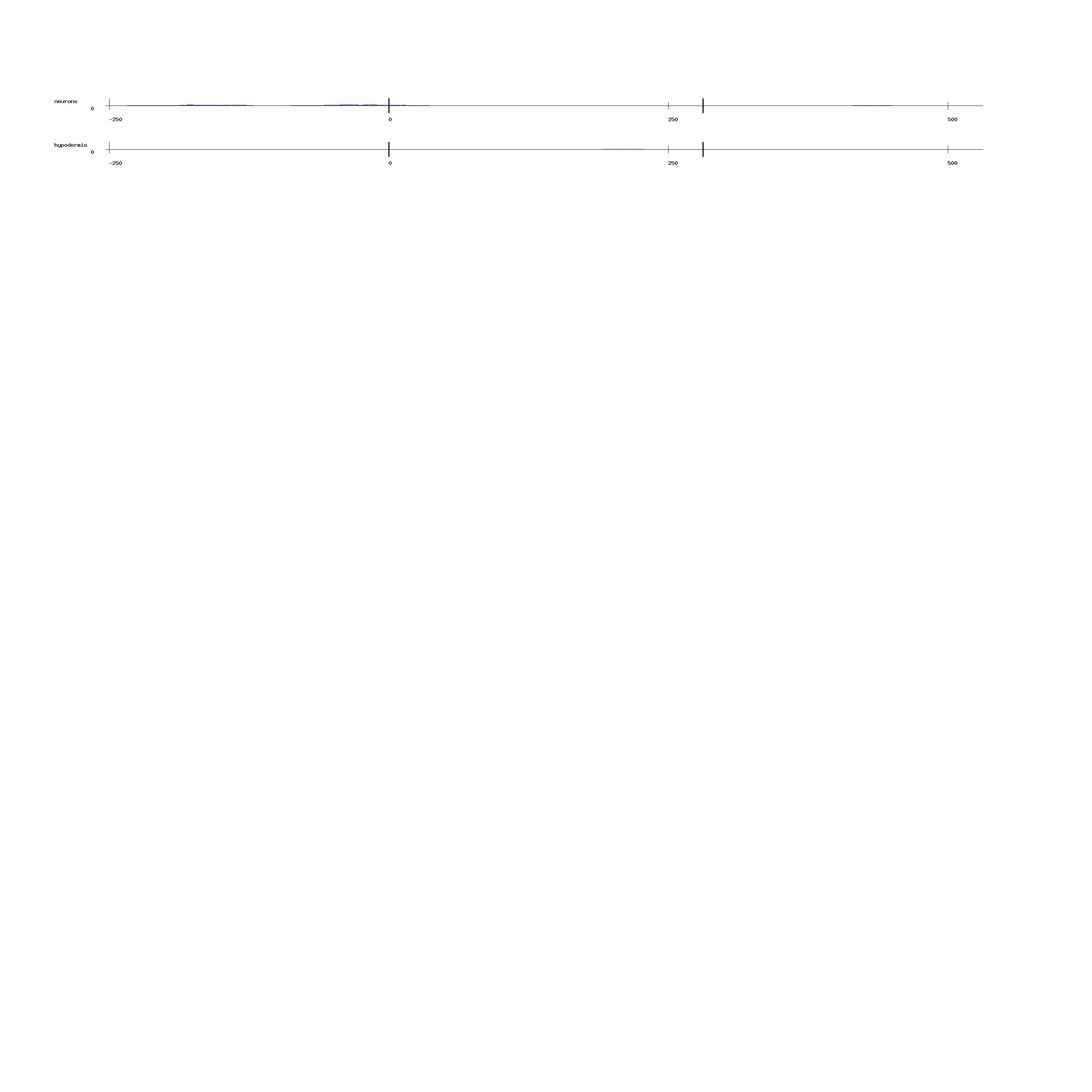

Supplement: Supplementary file 1 [file ijms-24-02970-s001.zip › Supplementary Data S2/1.780732-781012.png]

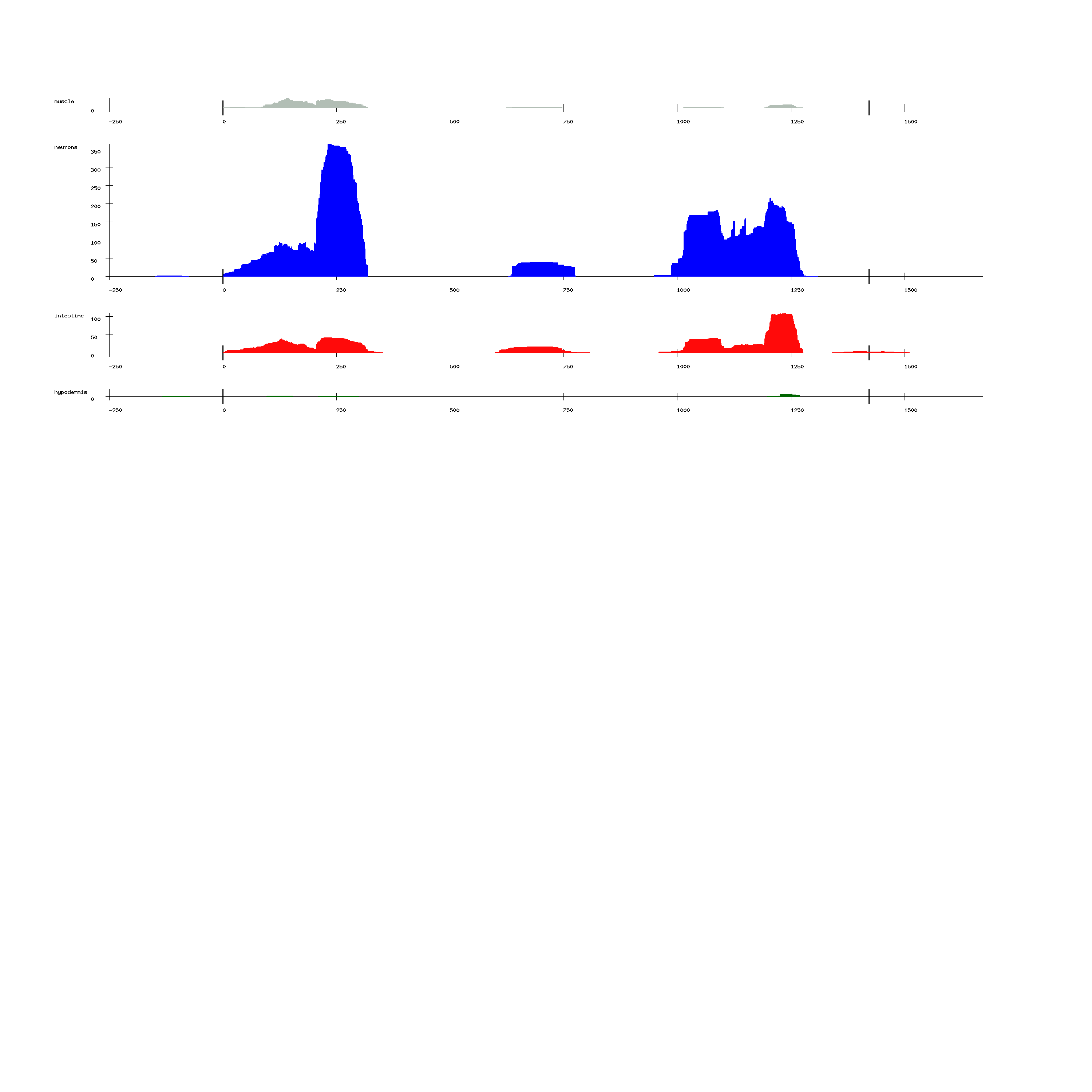

Supplement: Supplementary file 1 [file ijms-24-02970-s001.zip › Supplementary Data S2/1.933478-934899.png]

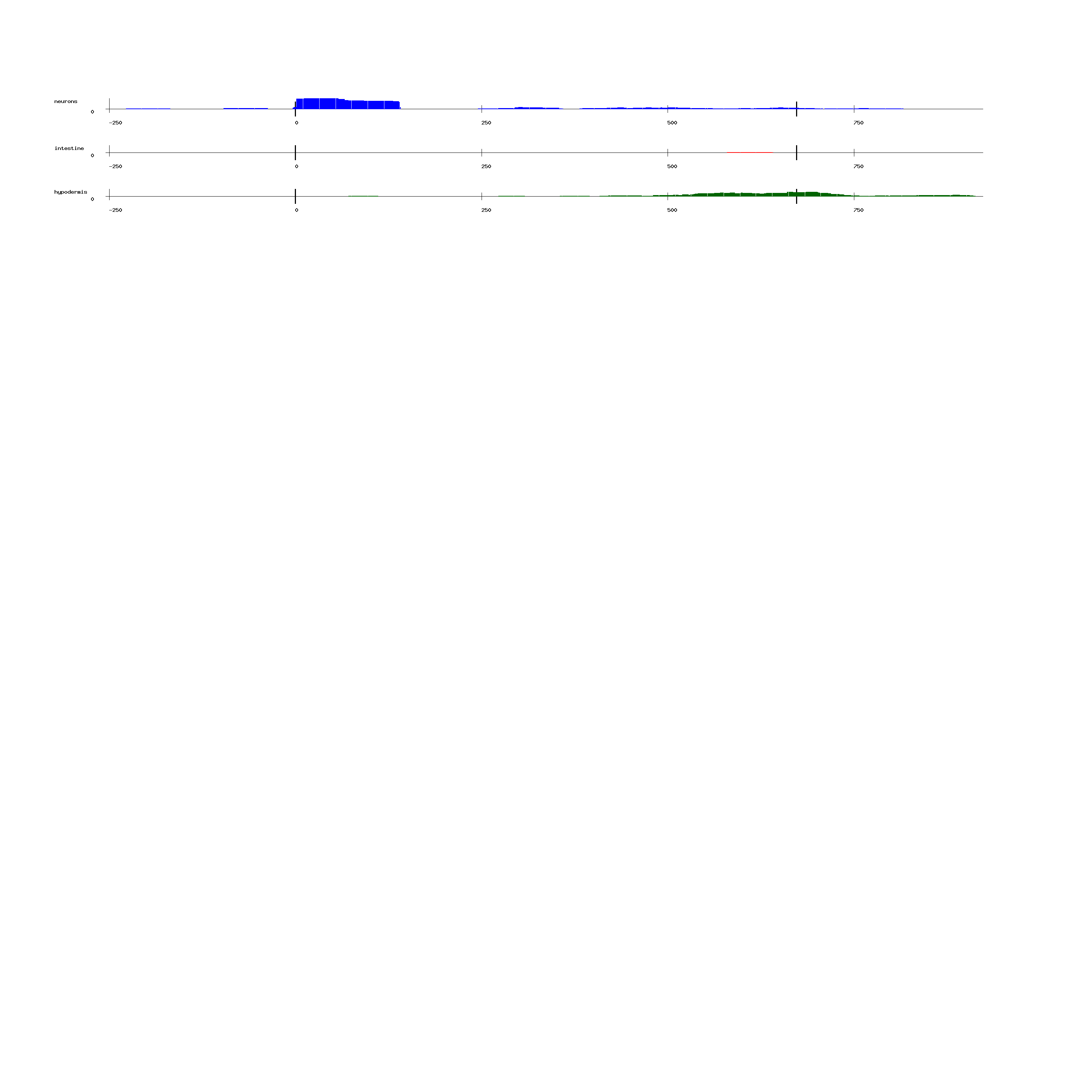

Supplement: Supplementary file 1 [file ijms-24-02970-s001.zip › Supplementary Data S2/1.961892-962564.png]

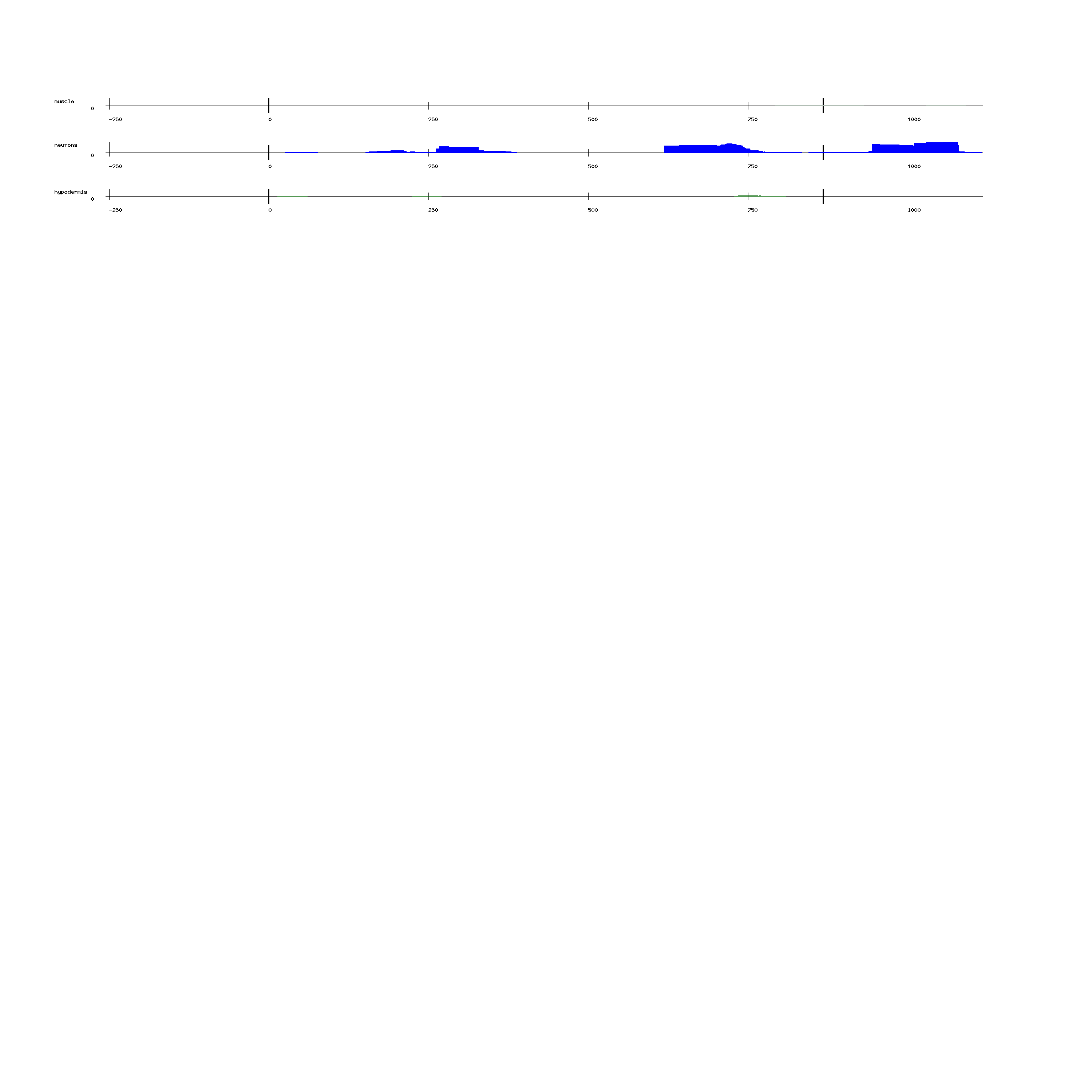

Supplement: Supplementary file 1 [file ijms-24-02970-s001.zip › Supplementary Data S2/1.9636862-9637728.png]

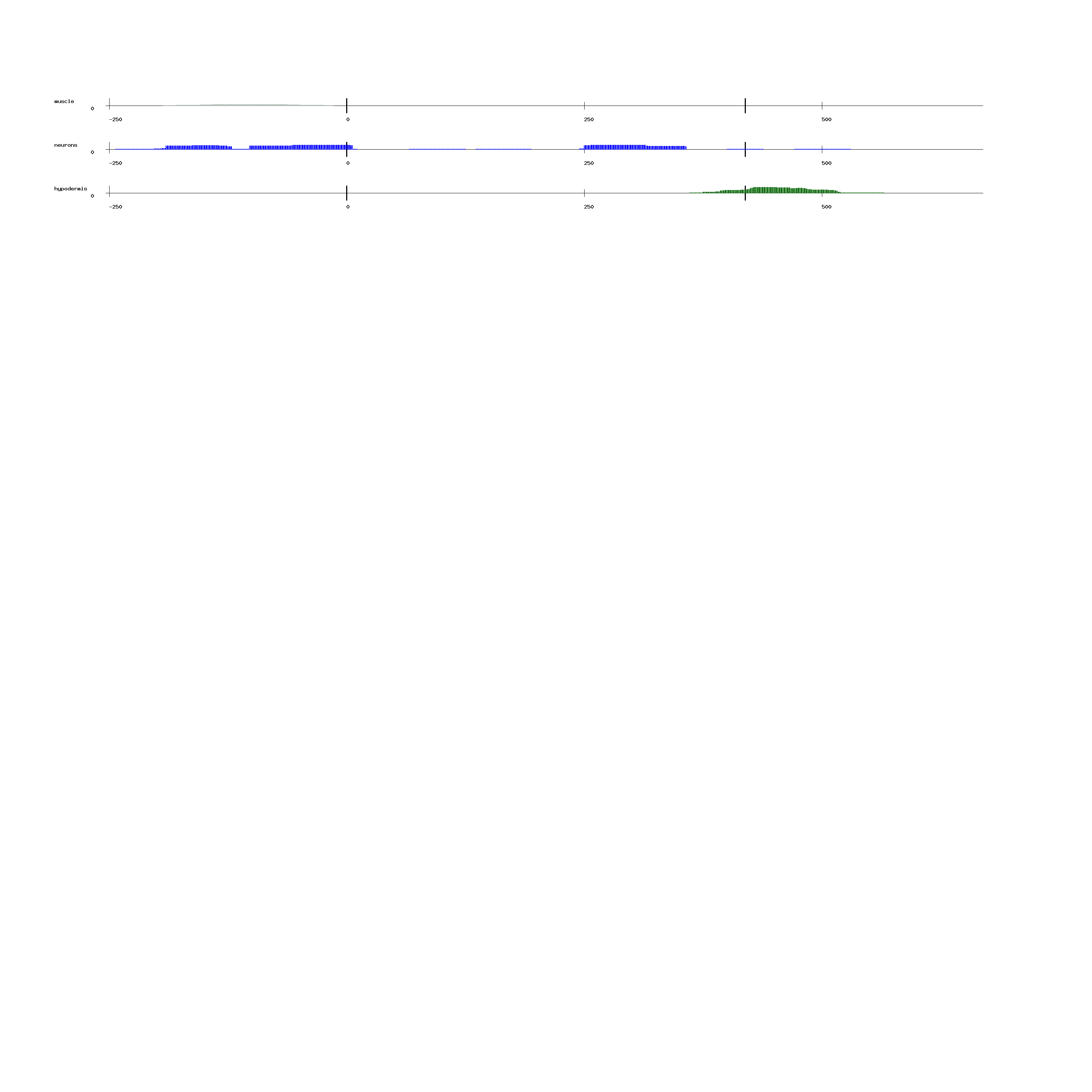

Supplement: Supplementary file 1 [file ijms-24-02970-s001.zip › Supplementary Data S2/1.994259-994677.png]

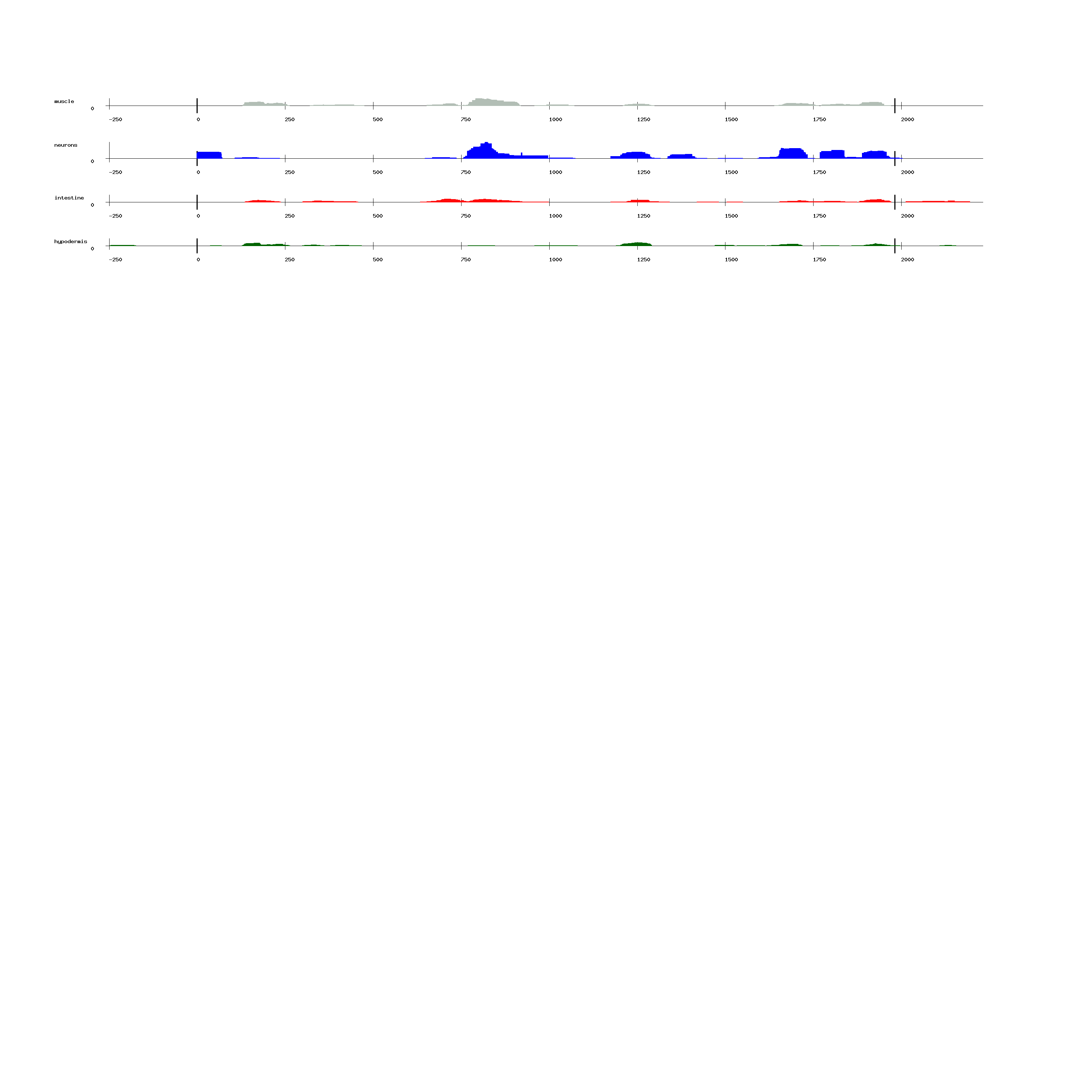

Supplement: Supplementary file 1 [file ijms-24-02970-s001.zip › Supplementary Data S2/2.1034465-1036445.png]

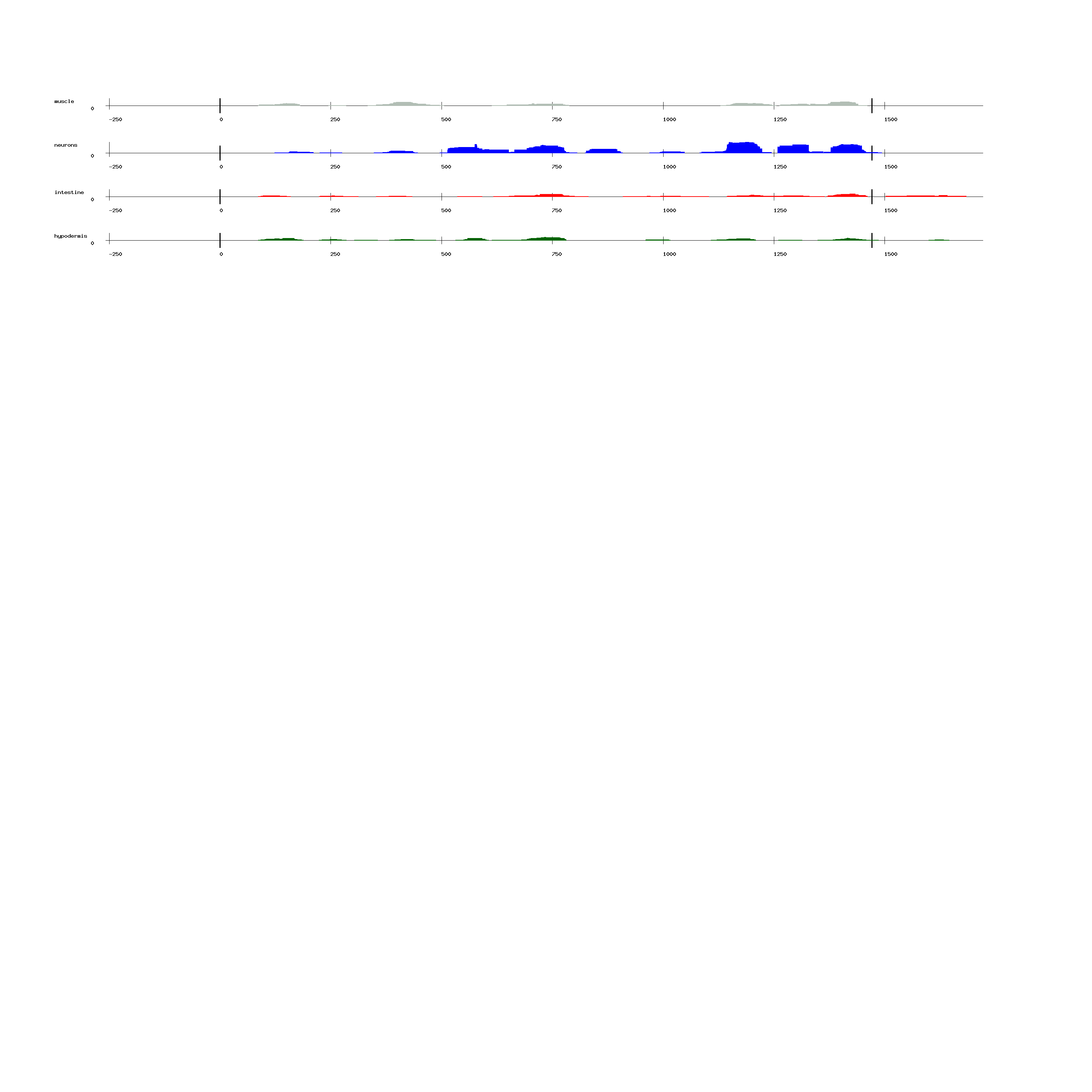

Supplement: Supplementary file 1 [file ijms-24-02970-s001.zip › Supplementary Data S2/2.1037817-1039287.png]

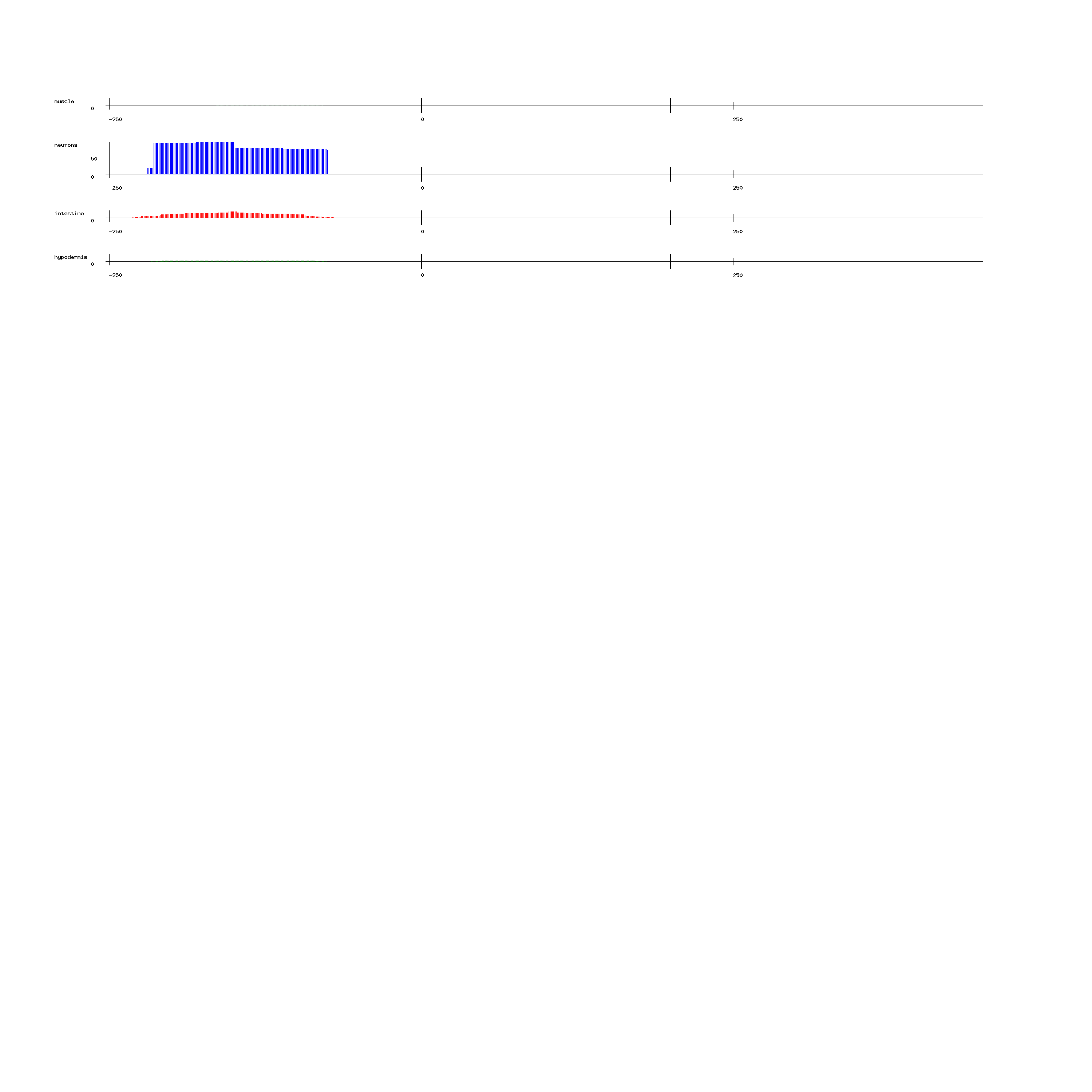

Supplement: Supplementary file 1 [file ijms-24-02970-s001.zip › Supplementary Data S2/2.11423462-11423661.png]

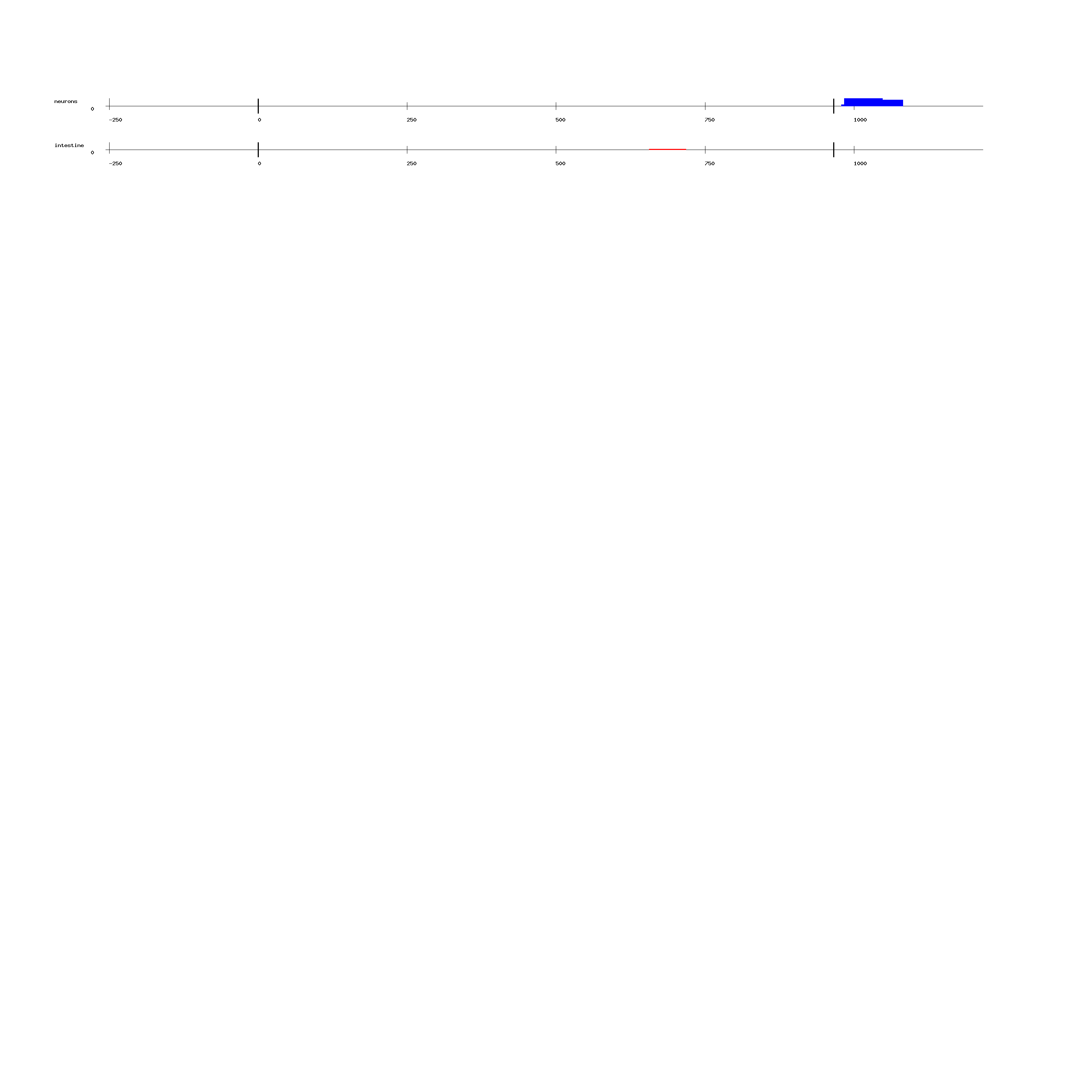

Supplement: Supplementary file 1 [file ijms-24-02970-s001.zip › Supplementary Data S2/2.12171287-12172252.png]

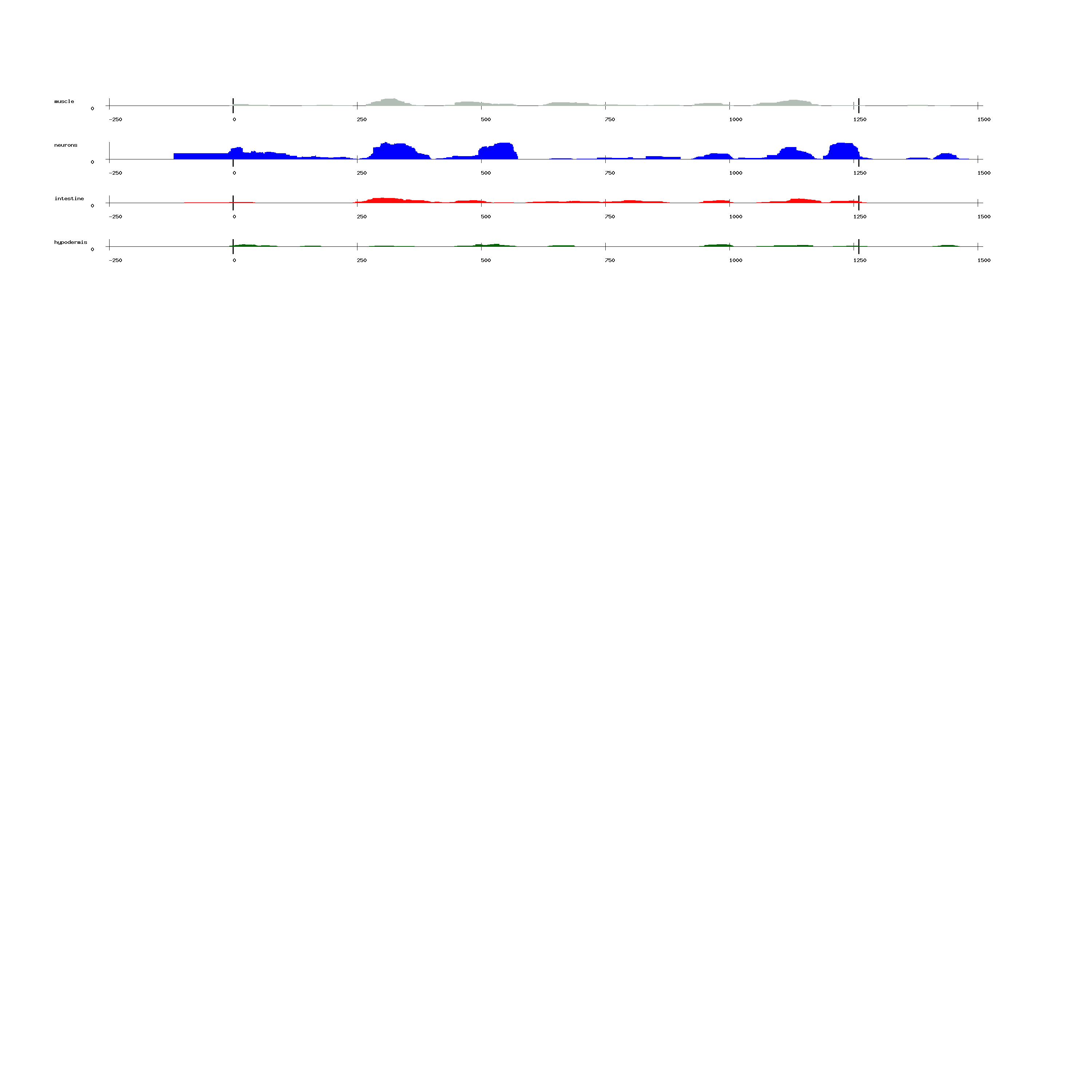

Supplement: Supplementary file 1 [file ijms-24-02970-s001.zip › Supplementary Data S2/2.12649559-12650818.png]

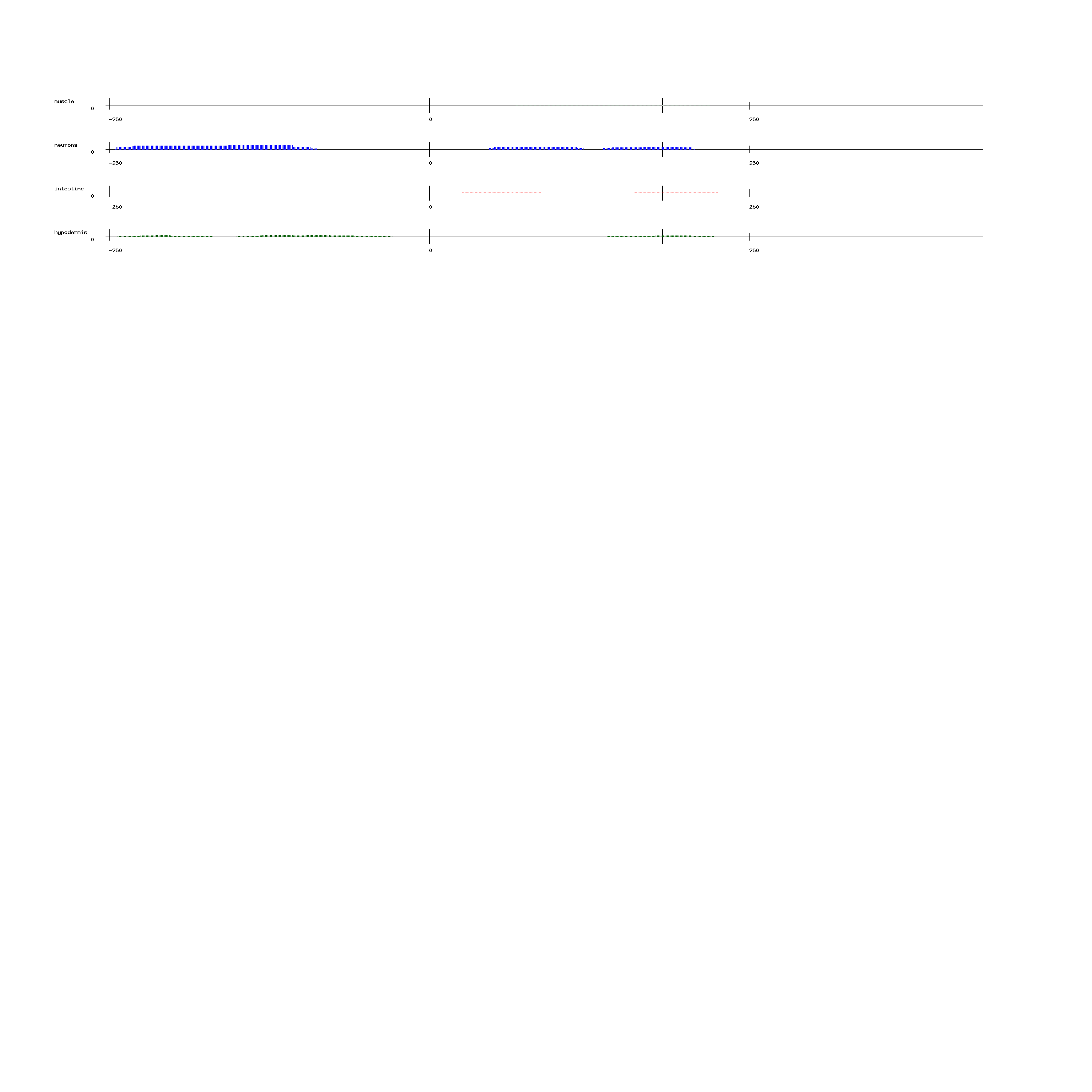

Supplement: Supplementary file 1 [file ijms-24-02970-s001.zip › Supplementary Data S2/2.12749754-12749935.png]

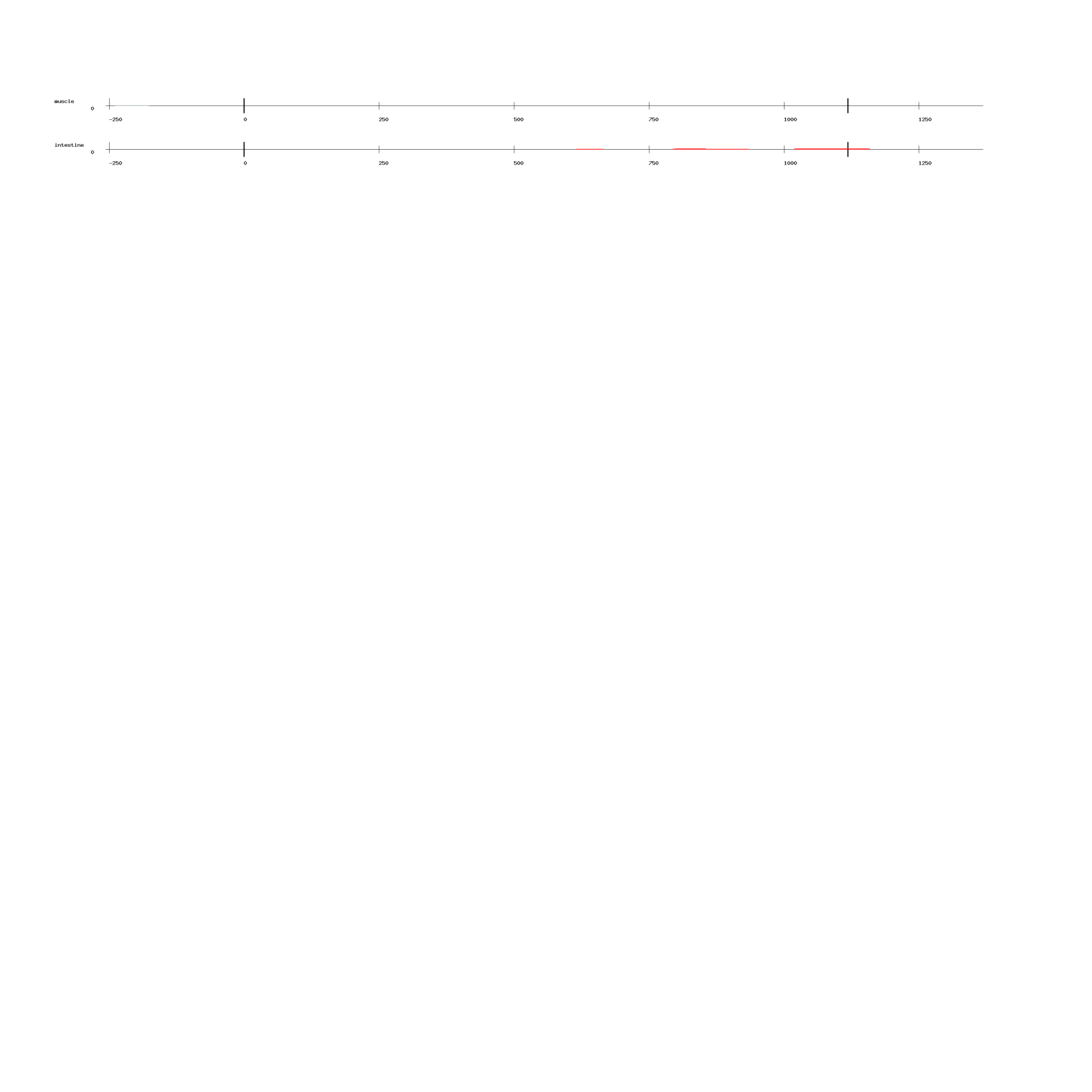

Supplement: Supplementary file 1 [file ijms-24-02970-s001.zip › Supplementary Data S2/2.12757828-12758945.png]

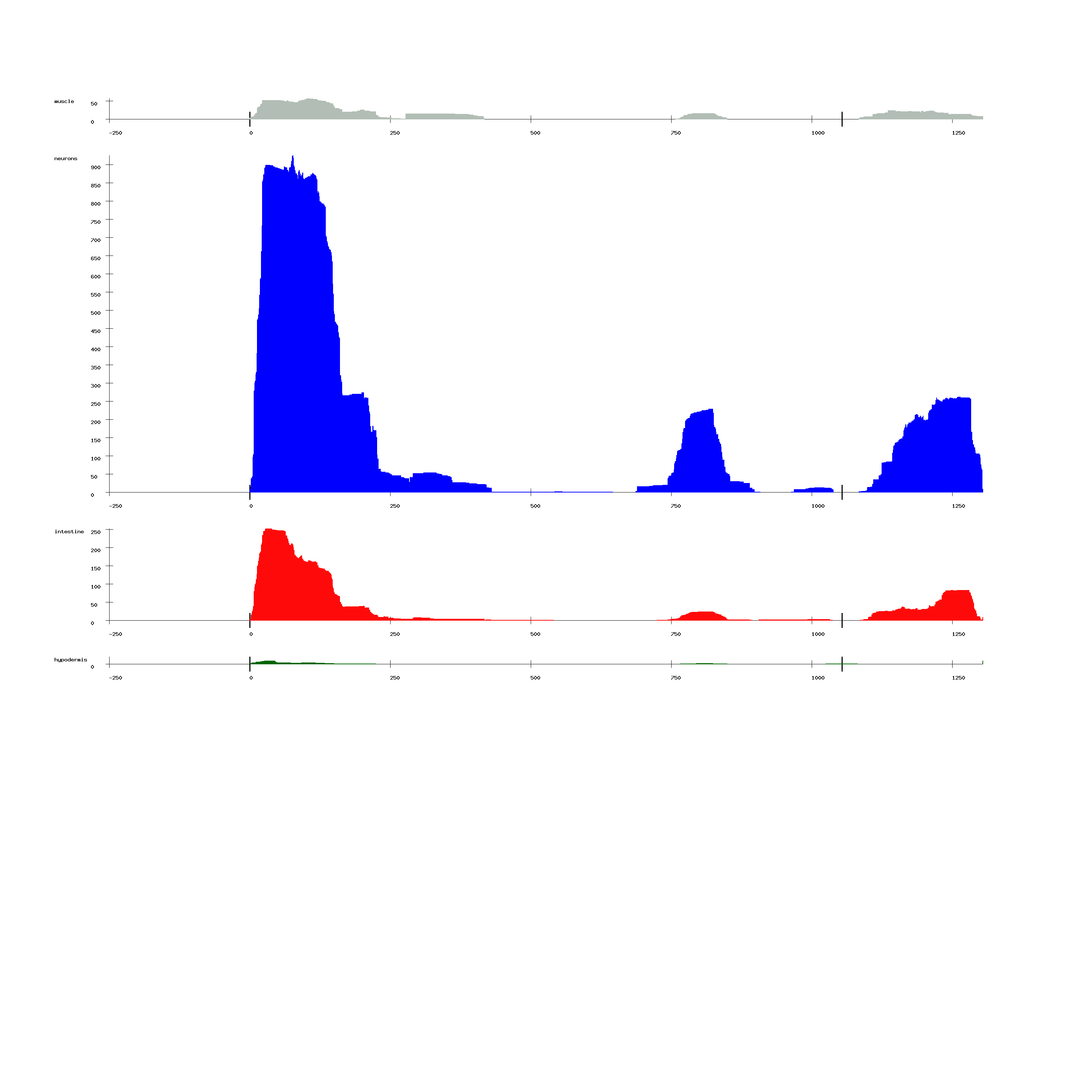

Supplement: Supplementary file 1 [file ijms-24-02970-s001.zip › Supplementary Data S2/2.12843822-12844875.png]

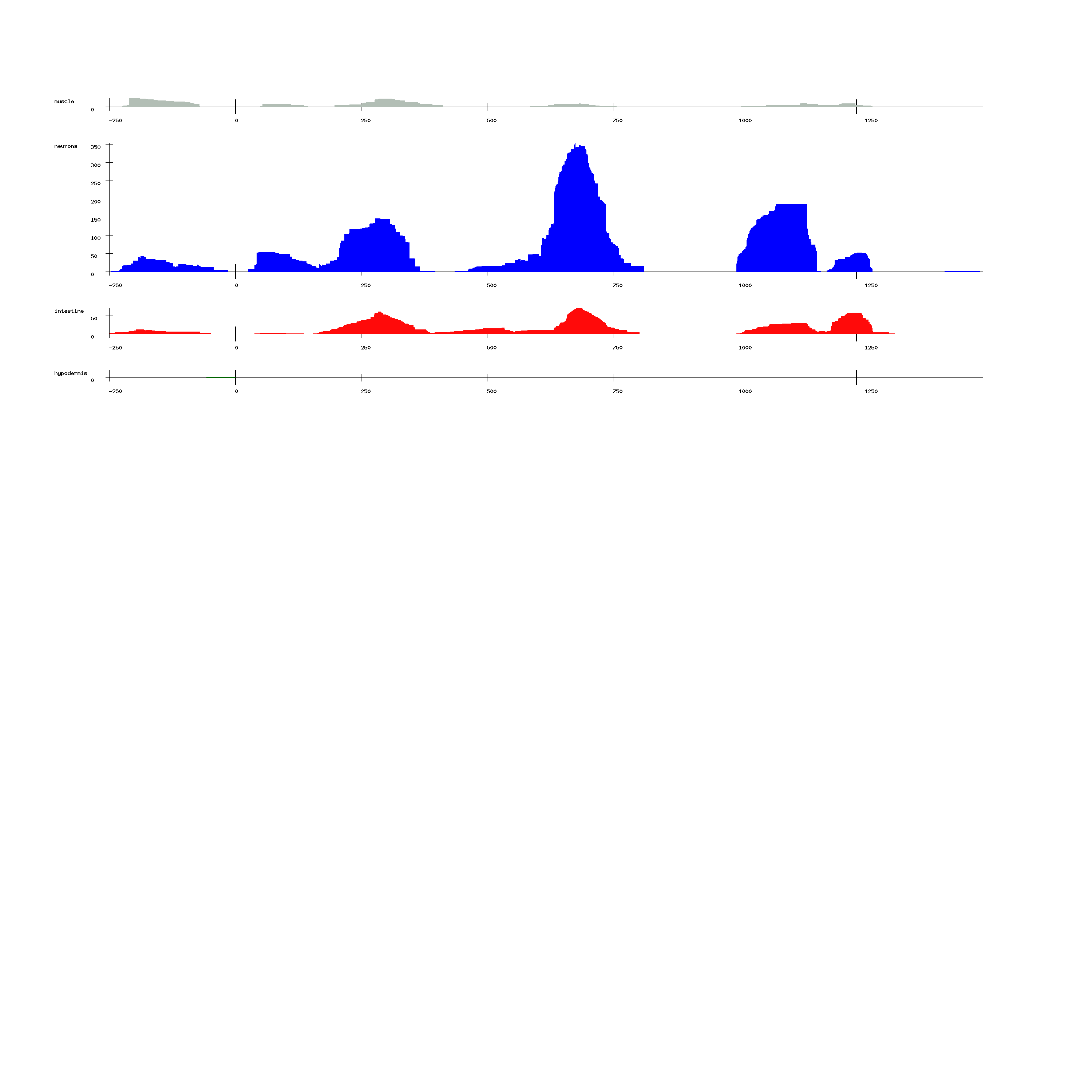

Supplement: Supplementary file 1 [file ijms-24-02970-s001.zip › Supplementary Data S2/2.12844903-12846136.png]

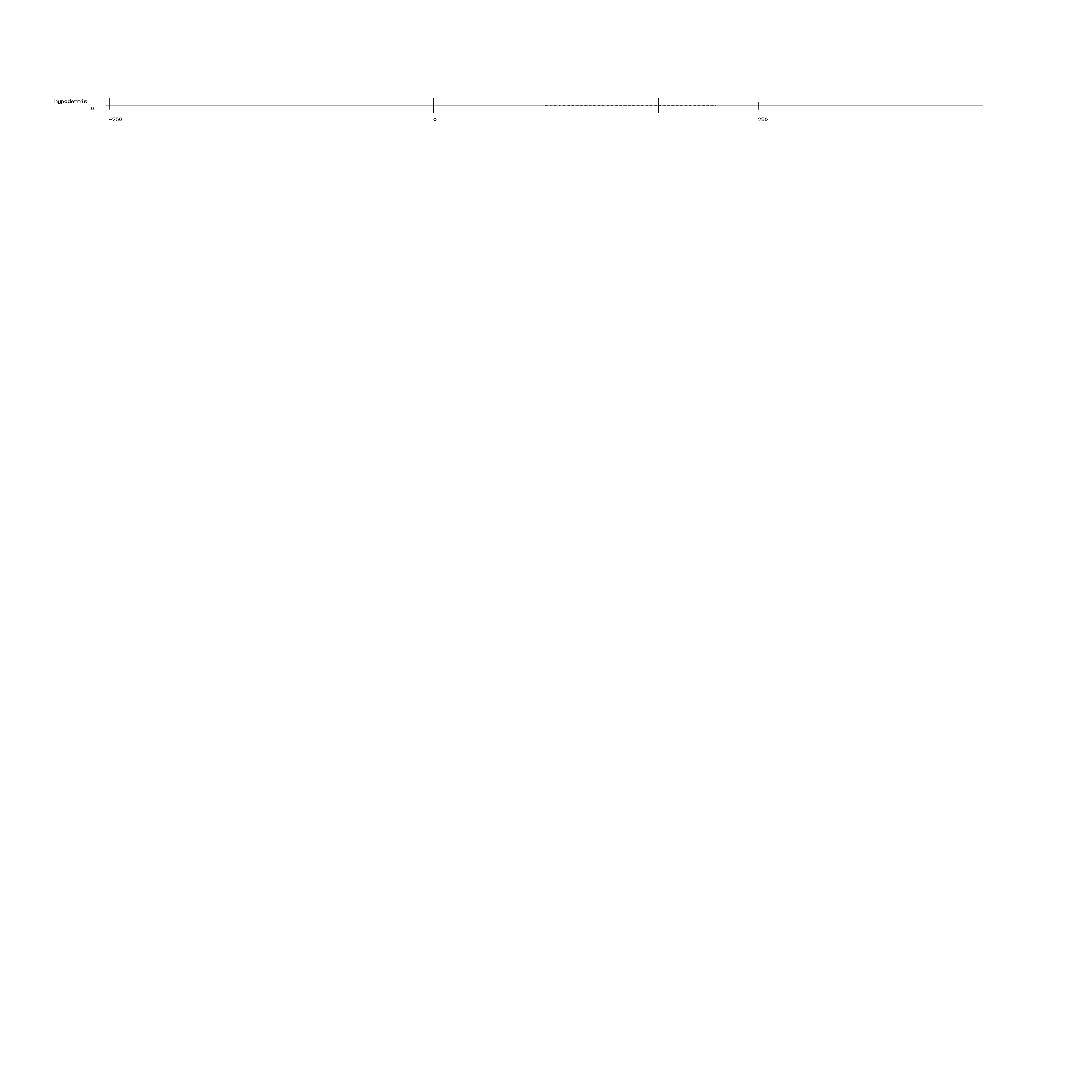

Supplement: Supplementary file 1 [file ijms-24-02970-s001.zip › Supplementary Data S2/2.12951422-12951594.png]

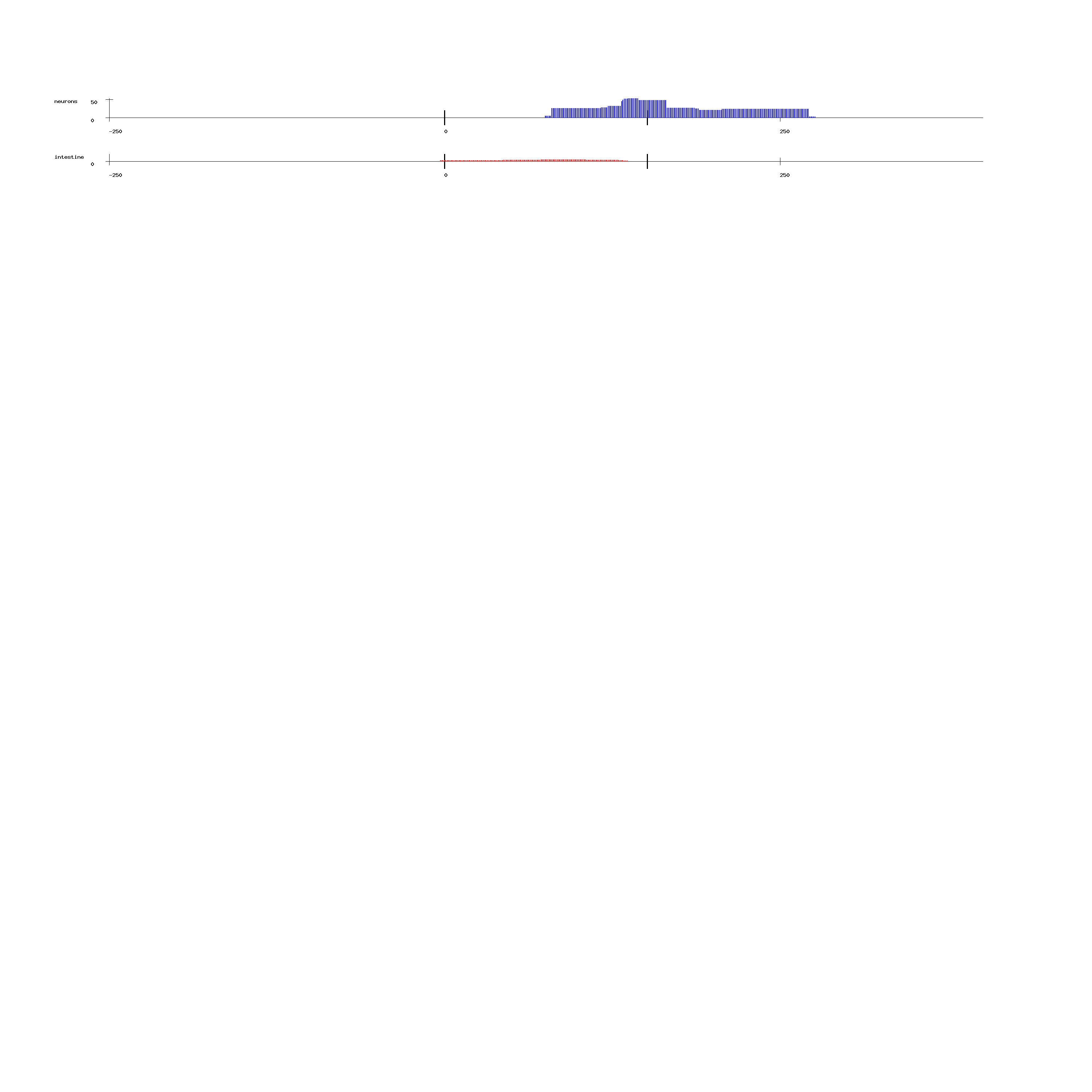

Supplement: Supplementary file 1 [file ijms-24-02970-s001.zip › Supplementary Data S2/2.12979640-12979790.png]

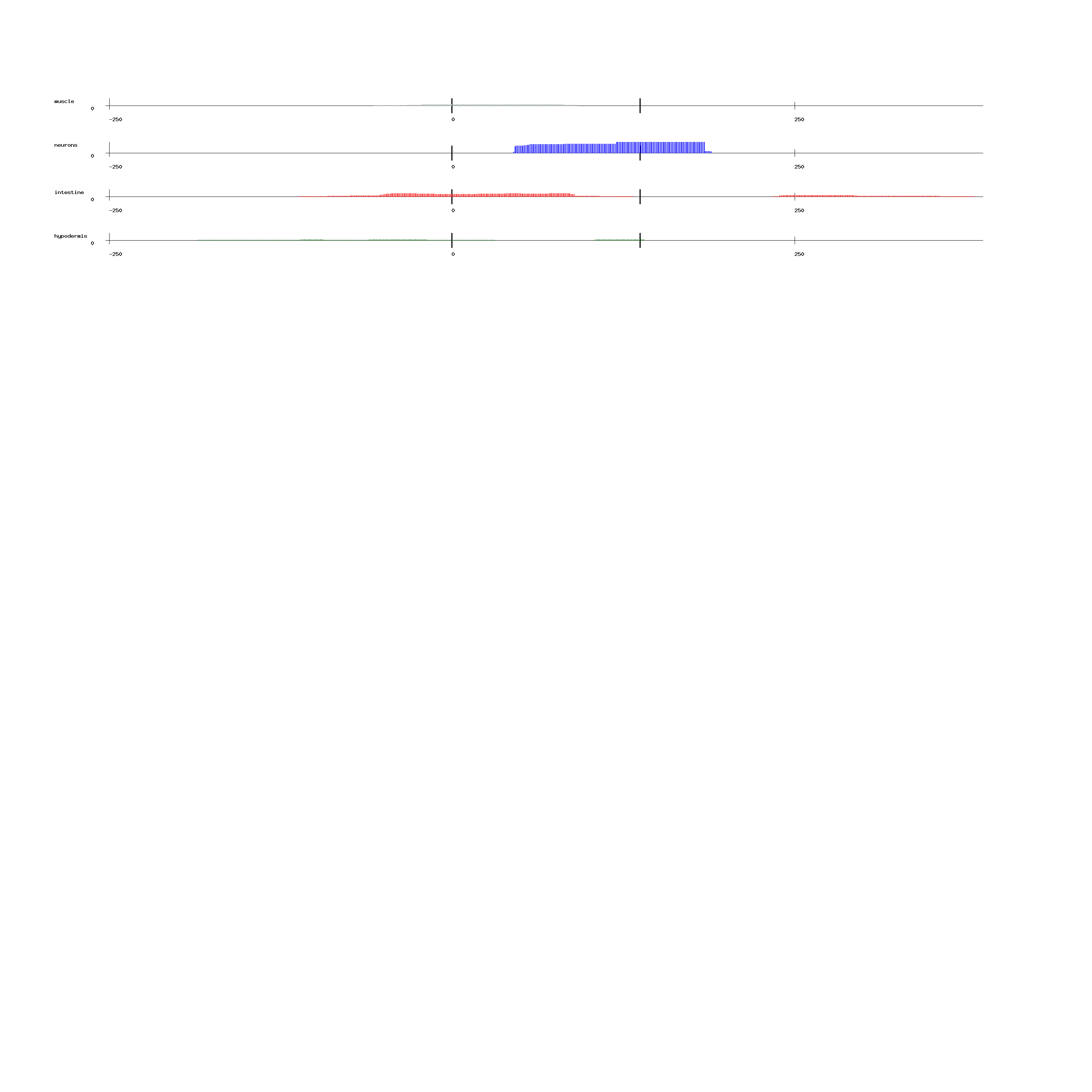

Supplement: Supplementary file 1 [file ijms-24-02970-s001.zip › Supplementary Data S2/2.13037761-13037897.png]

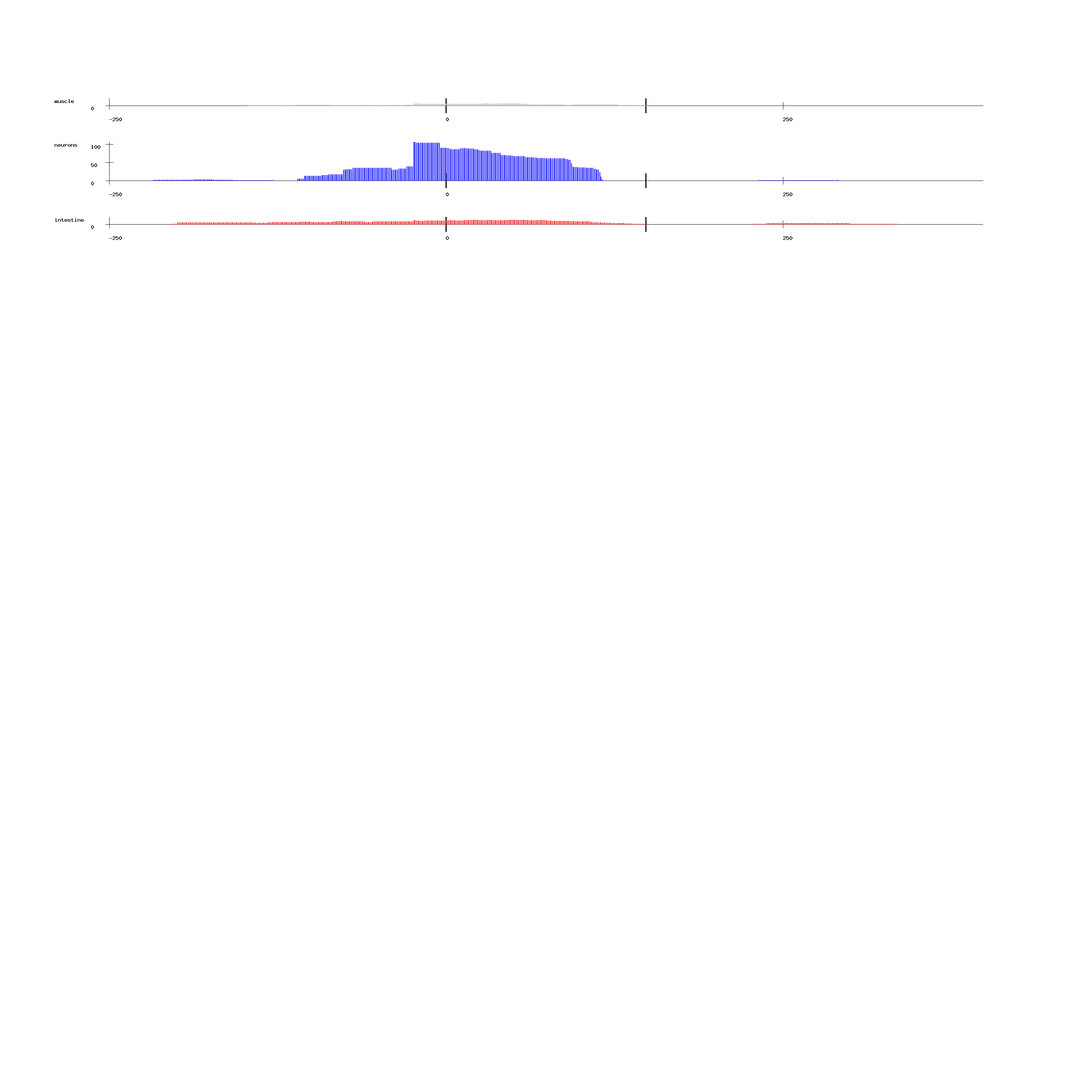

Supplement: Supplementary file 1 [file ijms-24-02970-s001.zip › Supplementary Data S2/2.13184130-13184277.png]

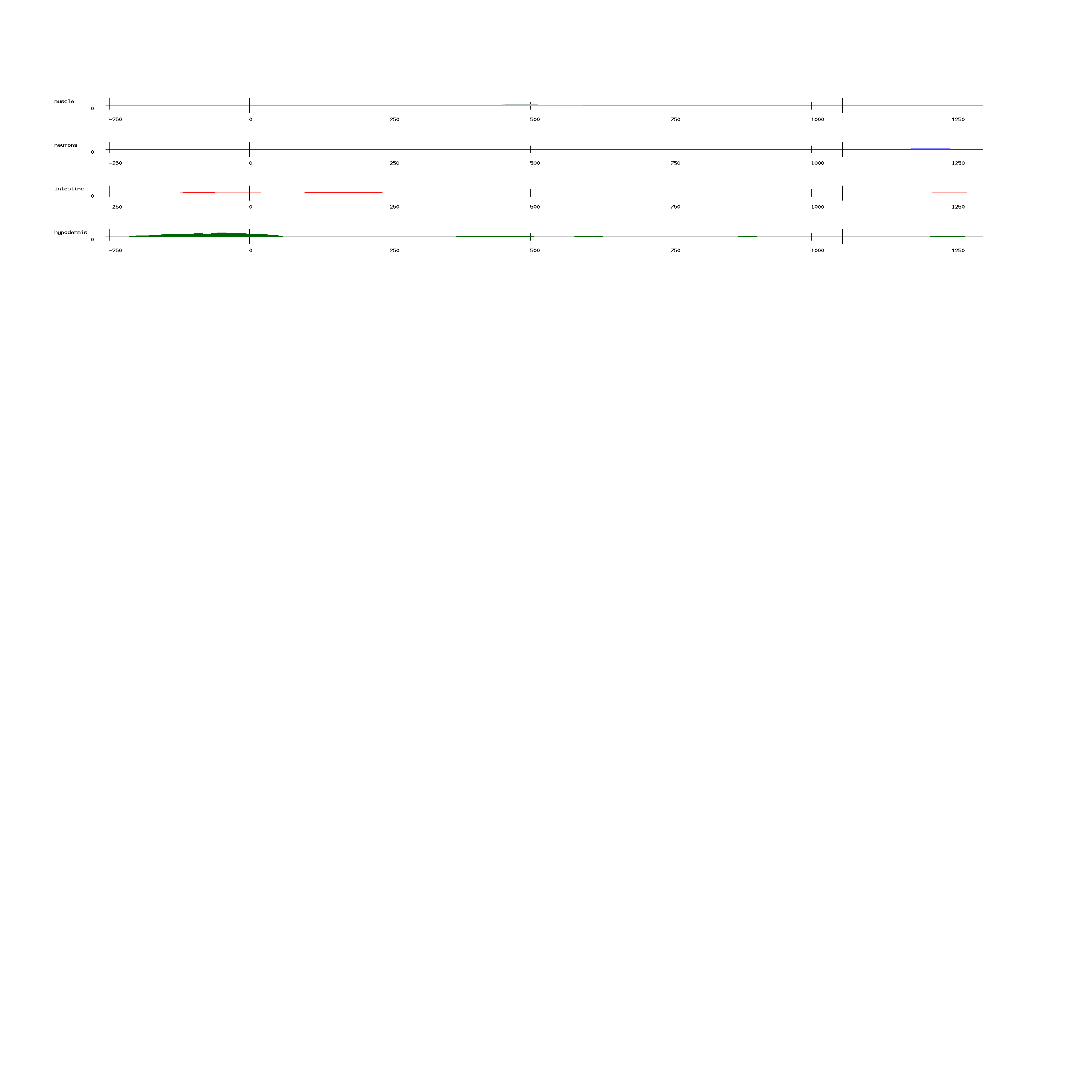

Supplement: Supplementary file 1 [file ijms-24-02970-s001.zip › Supplementary Data S2/2.13243398-13244452.png]

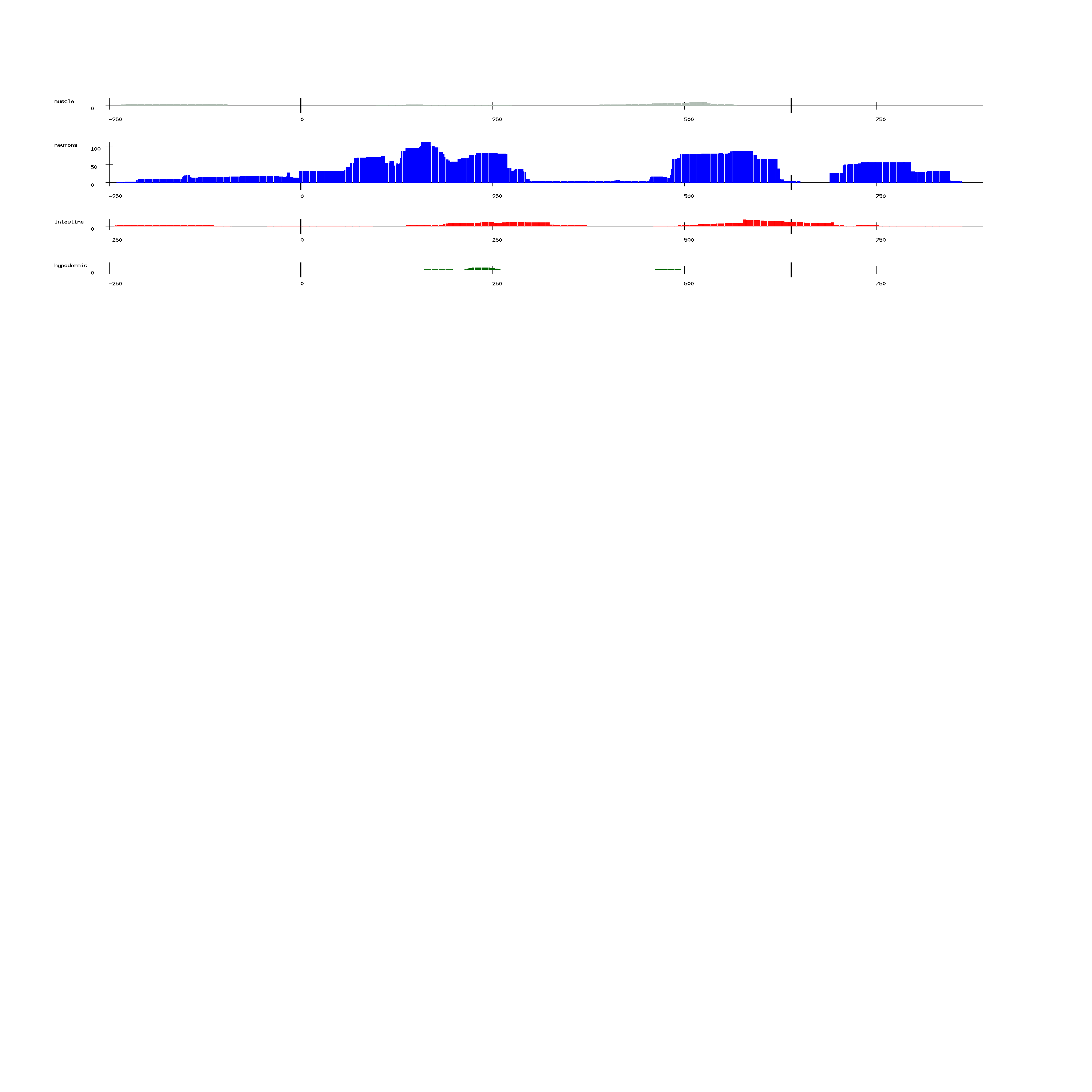

Supplement: Supplementary file 1 [file ijms-24-02970-s001.zip › Supplementary Data S2/2.13334193-13334831.png]

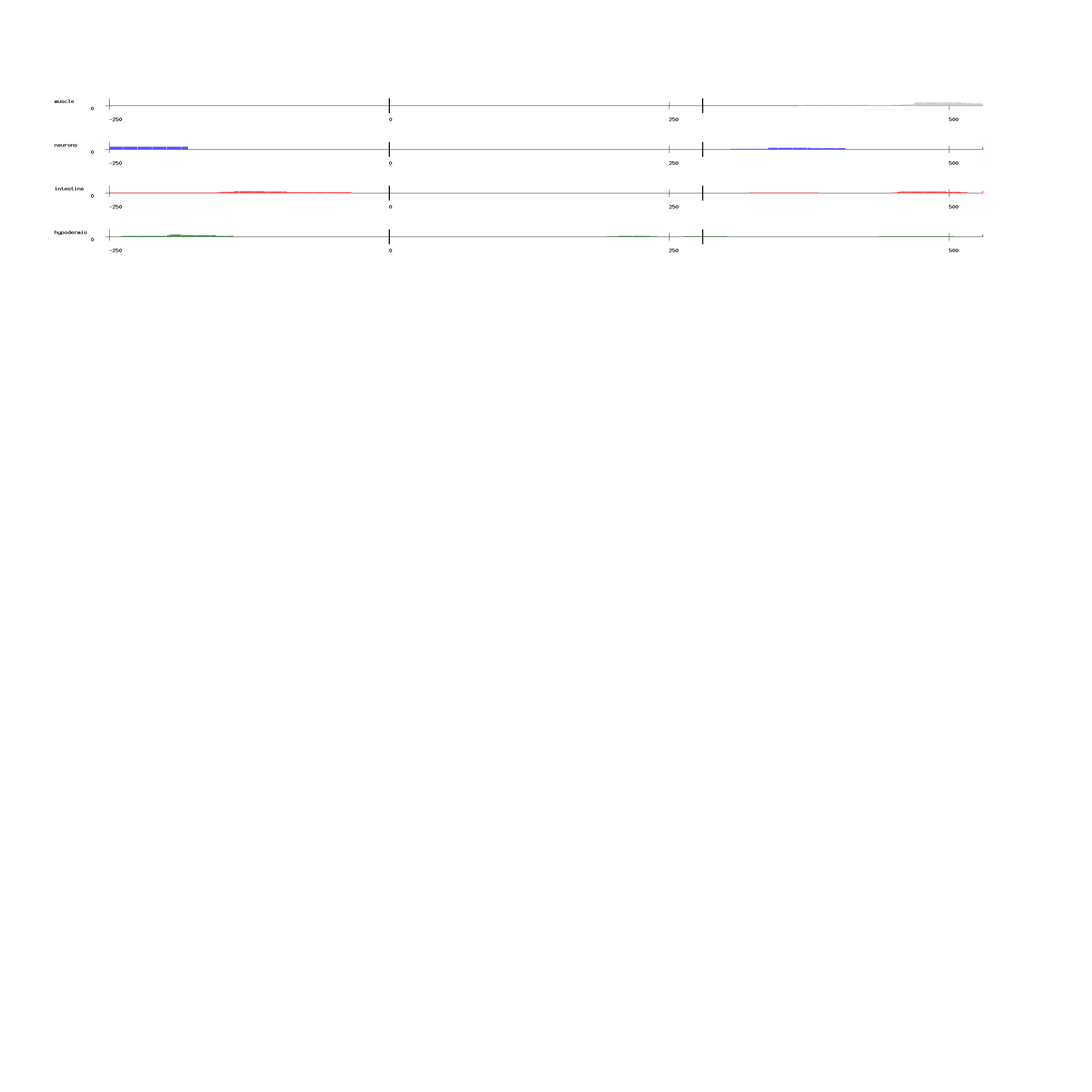

Supplement: Supplementary file 1 [file ijms-24-02970-s001.zip › Supplementary Data S2/2.13598247-13598526.png]

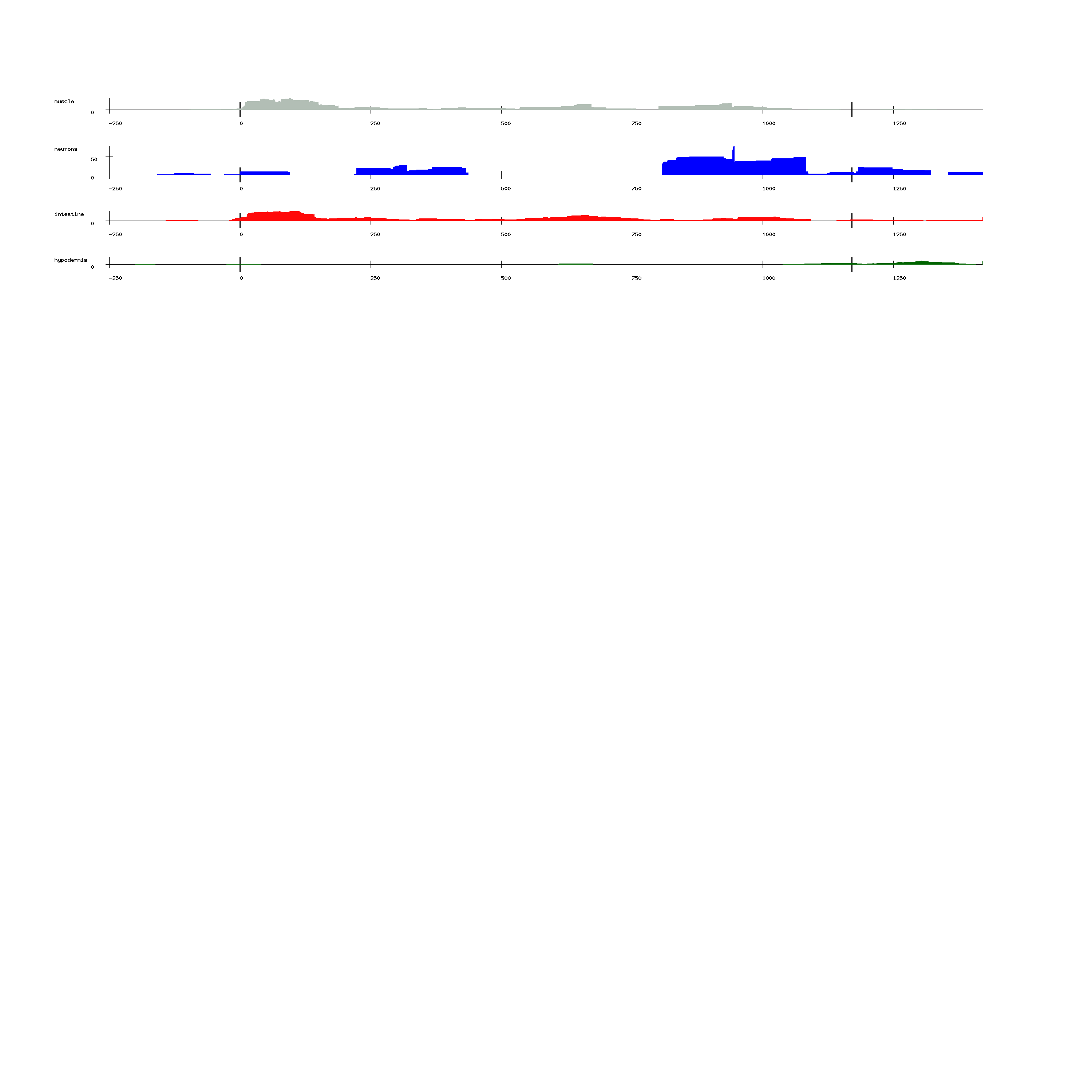

Supplement: Supplementary file 1 [file ijms-24-02970-s001.zip › Supplementary Data S2/2.13598710-13599880.png]

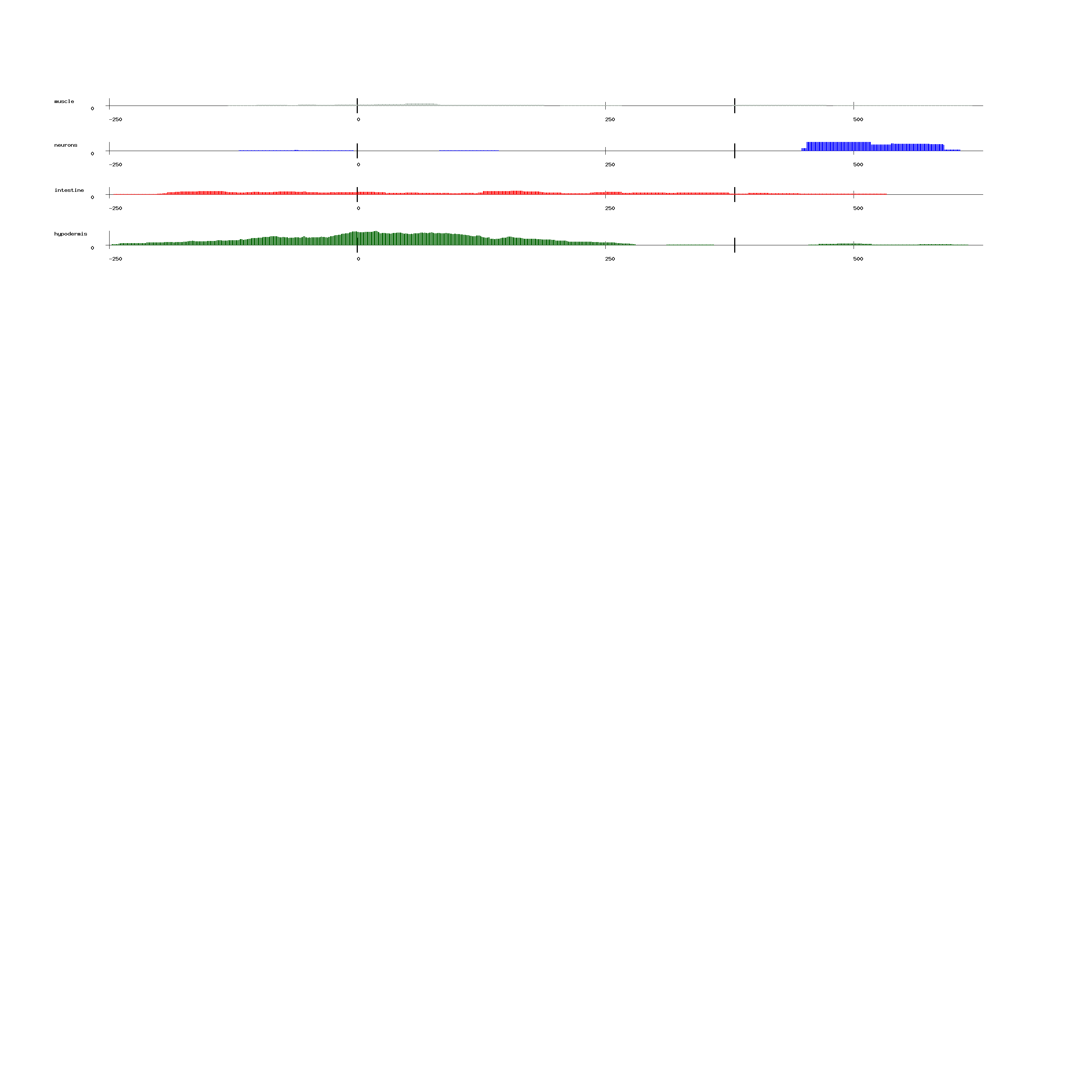

Supplement: Supplementary file 1 [file ijms-24-02970-s001.zip › Supplementary Data S2/2.13737396-13737775.png]

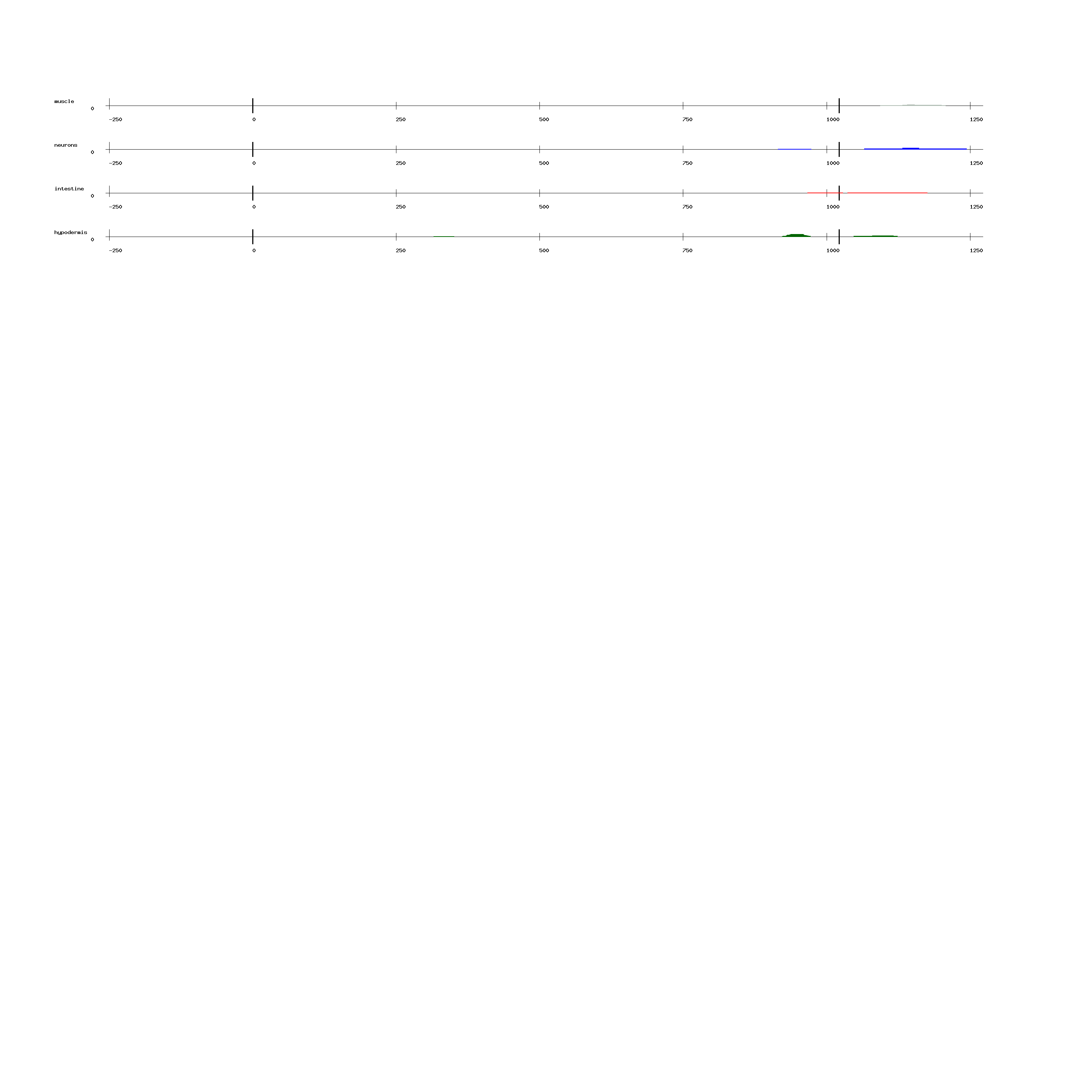

Supplement: Supplementary file 1 [file ijms-24-02970-s001.zip › Supplementary Data S2/2.13740296-13741317.png]

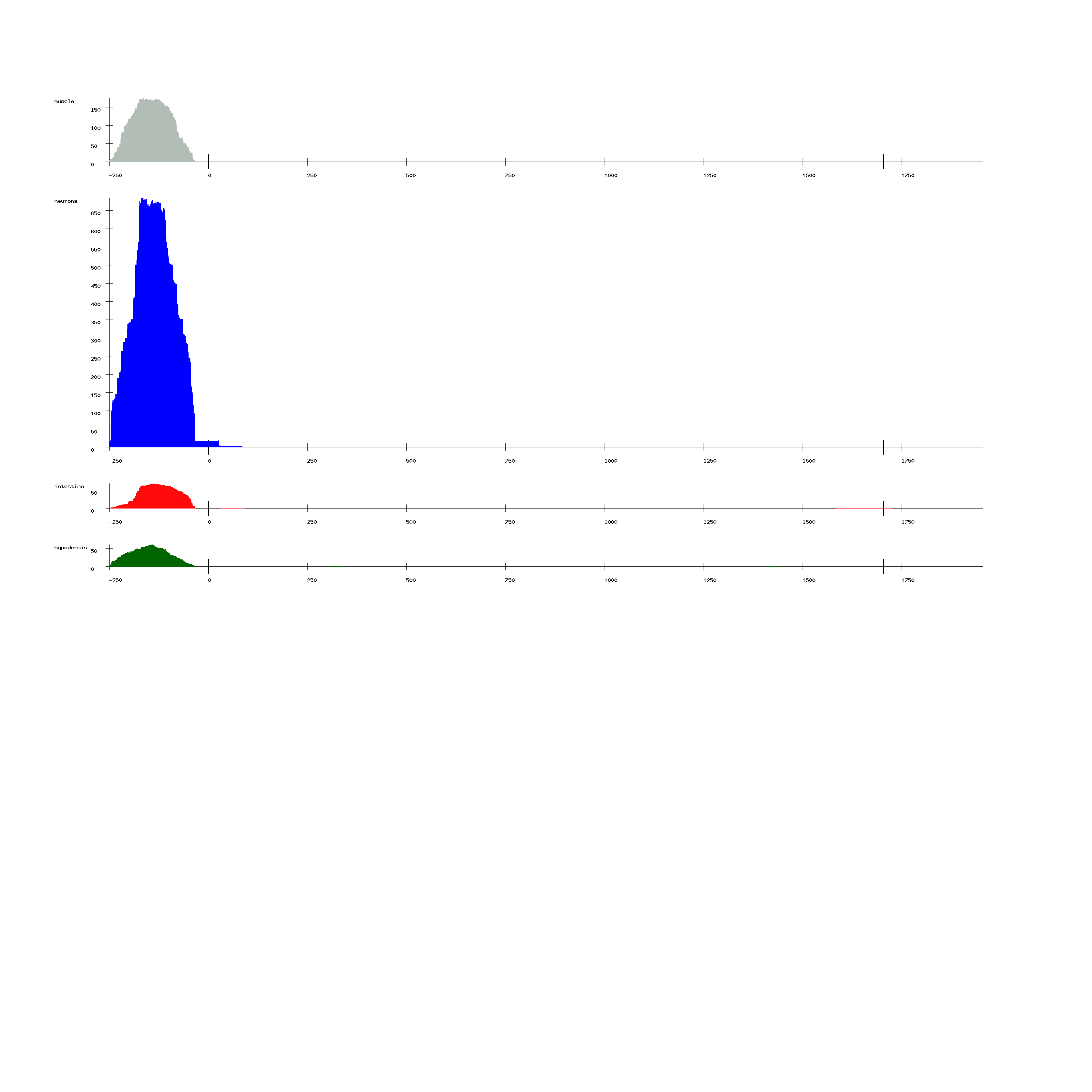

Supplement: Supplementary file 1 [file ijms-24-02970-s001.zip › Supplementary Data S2/2.13761268-13762971.png]

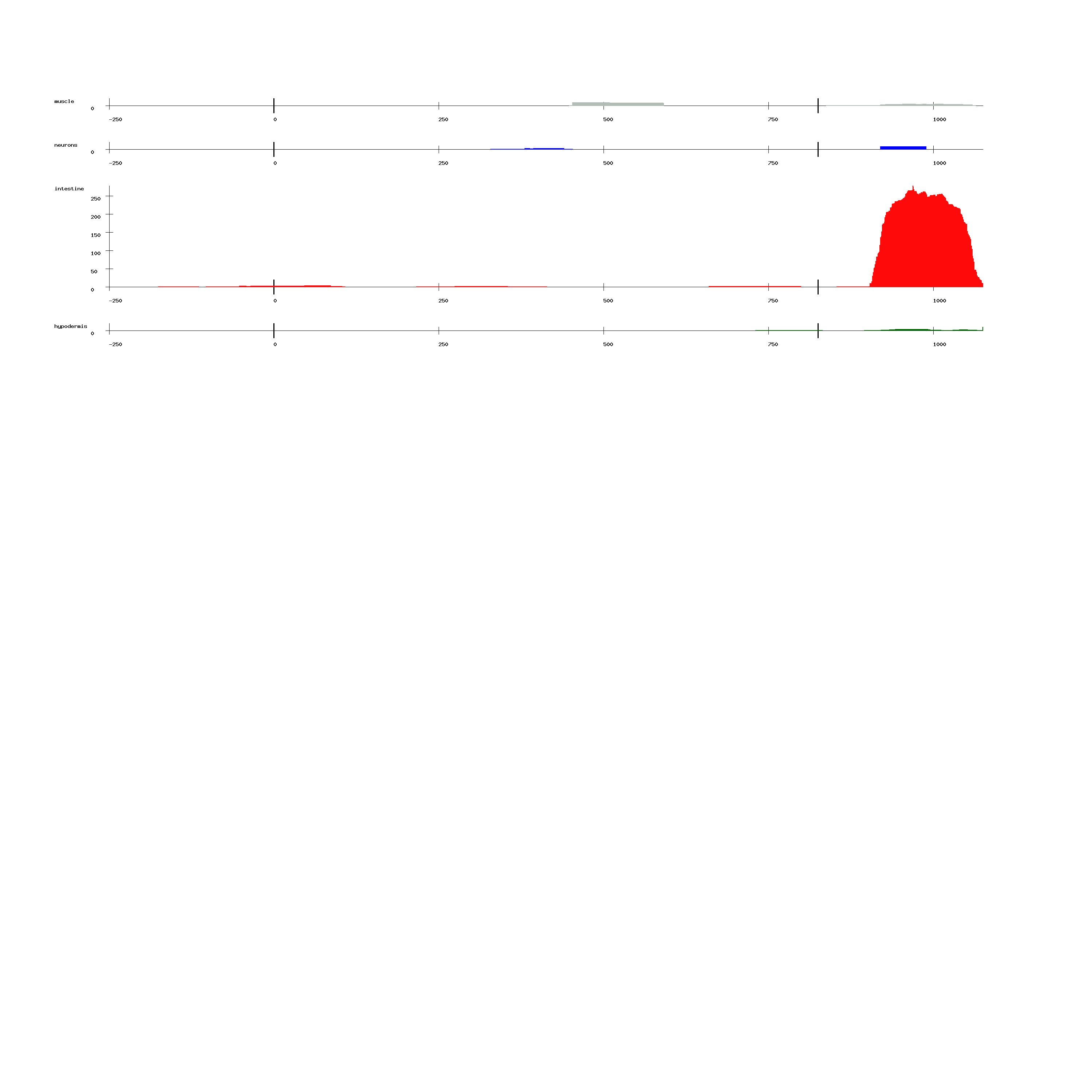

Supplement: Supplementary file 1 [file ijms-24-02970-s001.zip › Supplementary Data S2/2.13831950-13832774.png]
