# Supplementary material for: Unique Features of Satellite DNA Transcription in Different Tissues of Caenorhabditis elegans
Source: Int J Mol Sci. 2023 Feb 3;24(3):2970. doi: 10.3390/ijms24032970 (PMC9918286; doi:10.3390/ijms24032970)
Supplement: Supplementary file 1 [file ijms-24-02970-s001.zip › Table S1.List of all satellites.pdf]

Table S1. Properties of all satellites

In the second line are given the seed motif and the sequence of a representative repeat

Genome	Satellite name	Chromosome	Start	End	Sat.length	Nr Repts	Rpt.length
Cele.WB235	Cele.WB235_1:1	1	1	835	835	55	12
CTAAGCCTAA	CTAAGCCTAAGC						
Cele.WB235	Cele.WB235_1:222749	1	222749	222854	106	5	15
AGACCCATCG	AGACCCATCGTGGTG						
Cele.WB235	Cele.WB235_1:280525	1	280525	281393	869	23	38
AAATTTTAAT	AAATTTTAATTTTTTGAAAATATTTTGGCGGGAATTTA						
Cele.WB235	Cele.WB235_1:281468	1	281468	282278	811	52	15
TCGTGGTGAG	TCGTGGTGAGACCCA						
Cele.WB235	Cele.WB235_1:308028	1	308028	308148	121	7	15
GAGACCCATC	GAGACCCATCGTGGC						
Cele.WB235	Cele.WB235_1:334679	1	334679	335159	481	12	40
AATTTTCTCG	AATTTTCTCGAAATTTTCAGAAGGTTCTAGAATATTTCAG						
Cele.WB235	Cele.WB235_1:338561	1	338561	341137	2577	14	184
GAATACAGAA	GAATACAGAACACCAATTATGCCCGAGAAAAAGATCCTACTCAGAATATAAACATAGTCAAATTTATCGGATGTATAAAGATTCCCGAAGACACTTTCCAATTACCCAAATTGTTCATATTCTAAATCAAATTCTCTTACTAGAACGCTCTTGGCCAATGTACGCAGCCGAACGTATCATAAGT						
Cele.WB235	Cele.WB235_1:354705	1	354705	355291	587	29	19
TATCGATTTT	TATCGATTTTCCTGAAATT						
Cele.WB235	Cele.WB235_1:359078	1	359078	359230	153	8	19
GTACTACCTT	GTACTACCTTTTAATGTAT						
Cele.WB235	Cele.WB235_1:477877	1	477877	478046	170	13	12
TAGGCTTAGG	TAGGCCTAGGCT						
Cele.WB235	Cele.WB235_1:556995	1	556995	557143	149	8	12
CTAAGCCTAA	CTAAGCCTAAGC						
Cele.WB235	Cele.WB235_1:562402	1	562402	562457	56	5	11
GTACTCCTAC	GTACTCCTAAA						
Cele.WB235	Cele.WB235_1:565592	1	565592	565664	73	6	12
GCGTTGCGTG	GCGTTGCGTGCC						
Cele.WB235	Cele.WB235_1:566943	1	566943	567081	139	8	12
AGGCTTAGGC	AGGCCTAGGCTT						
Cele.WB235	Cele.WB235_1:623235	1	623235	624175	941	22	23
AAAAAAAATT	AAAAAAAATTAAATTTTTCAACG						
Cele.WB235	Cele.WB235_1:640909	1	640909	641017	109	6	12
TAAGCCTAAG	TGAGCCTAAGCG						
Cele.WB235	Cele.WB235_1:669658	1	669658	671715	2058	63	32
GAATTCAAAT	GAATTCAAATTTTCTGAGAAAAATTTAGGCGA						
Cele.WB235	Cele.WB235_1:676497	1	676497	676765	269	6	45
TCTGATCCTT	TCTGATCCTTCAGCTCCGAAGGATCACATGGGTACCTTCTGATGT						
Cele.WB235	Cele.WB235_1:687047	1	687047	687860	814	14	44
TTTGCCGGTT	TTTGCCGGTTTGCCGATTTGCCCGACATTTTTCAATTTCGGCAA						
Cele.WB235	Cele.WB235_1:689715	1	689715	689895	181	12	15
GGTGAGACCC	GGTGAGACCCATCGT						
Cele.WB235	Cele.WB235_1:724780	1	724780	724948	169	4	42
TTTGATCTAC	TTTGATCTACAAAAAATGCGGGAGTTTTCCCAGCATTGCTCA						
Cele.WB235	Cele.WB235_1:727038	1	727038	727274	237	5	12
GCTTAGGCTT	GCTTAGGCTTAA						

Cele.WB235	Cele.WB235_1:733645	1	733645	734074	430	11	12	GCAGGTAGGT	GCAGGTAGGTAG
Cele.WB235	Cele.WB235_1:749263	1	749263	749839	577	36	16	TTTCAGGCTT	TTTCAGGCTTTCAGGC
Cele.WB235	Cele.WB235_1:759087	1	759087	759538	452	13	34	TTTCTCAGAA	TTTCTCAGAAAATTTGAATTTCCCGCCAAAATTT
Cele.WB235	Cele.WB235_1:765668	1	765668	765992	325	27	12	TCTAGGTCTA	TCTAGGTCTAAG
Cele.WB235	Cele.WB235_1:778056	1	778056	778335	280	6	40	GAACCTTCTG	GAACCTTCTGGAATATTTGAGAAAACTCTAGAATGTTCTA
Cele.WB235	Cele.WB235_1:779157	1	779157	779331	175	12	12	GCCTAAGCCT	GCCTAAGCCTAA
Cele.WB235	Cele.WB235_1:780732	1	780732	781012	281	7	40	TCTAGAACAT	TCTAGAACATTCCAGAATTTTCTCGAATTTTCCAAAAAGT
Cele.WB235	Cele.WB235_1:806422	1	806422	806644	223	16	12	GCCTAAGCCT	GCCAAAGCCTGA
Cele.WB235	Cele.WB235_1:808432	1	808432	808756	325	8	22	TTAAAATTTT	TTAAAATTTTGTCAGTTTGACA
Cele.WB235	Cele.WB235_1:814462	1	814462	814642	181	13	12	GCTTAGGCTT	GCTTAGGCTTAG
Cele.WB235	Cele.WB235_1:823801	1	823801	824454	654	29	21	TGGCCTAGAT	TGGCCTCGATTCTGTACAGGG
Cele.WB235	Cele.WB235_1:833515	1	833515	834452	938	48	12	CTTAGGCTTA	CTCAGGCTTAGG
Cele.WB235	Cele.WB235_1:846174	1	846174	846372	199	12	12	GCTTAGGCTT	GCTTAGACTTAG
Cele.WB235	Cele.WB235_1:898453	1	898453	898911	459	21	22	TTTTAGCCAA	TTTTAACCAAAAAATCCGTTGA
Cele.WB235	Cele.WB235_1:902260	1	902260	902411	152	8	15	ATCTTCTTTT	ATCTTCTTTTGGTAA
Cele.WB235	Cele.WB235_1:923168	1	923168	925176	2009	89	21	AATTTCTAGG	AATTTCTAGGCCACGATCTGA
Cele.WB235	Cele.WB235_1:933478	1	933478	934899	1422	52	27	TATTTTACTC	TATTTTACTCTCTGTGGCTTCACCAAT
Cele.WB235	Cele.WB235_1:942559	1	942559	942637	79	6	12	AGCCTAAGCC	AGCCTAAGCCTA
Cele.WB235	Cele.WB235_1:942750	1	942750	943044	295	24	12	CTAAGTCTAA	CTAAGTCTAAGT
Cele.WB235	Cele.WB235_1:942557	1	942557	943381	825	46	12	TAAGCCTAAG	TAAGCCTAAGCC
Cele.WB235	Cele.WB235_1:961892	1	961892	962564	673	16	42	CCGGAAATTT	CCGGAAATTTCGAAAACCGGCAATTGCCGAAATTGCCCTTTG
Cele.WB235	Cele.WB235_1:976735	1	976735	977538	804	5	162	ATCATATGAA	ATCATATGAAGCGATTATTTTTTCTTTTATAAACCTGTTCAGGATTTGCATTATTATGAGAAAATCCTAGGCCAATTAGGCTAGCATGTACGGAAGTATTTTTTAAAAATTTATAAAAGTAATGAAAAGCTGATATTTTCAAAATTCCAGAATGACGGAAAA
Cele.WB235	Cele.WB235_1:991239	1	991239	991341	103	8	12	GGTCTAGGTC	GGTCTAGGTCTA
Cele.WB235	Cele.WB235_1:994259	1	994259	994677	419	4	32	CAAAATTTTT	CAAAATTTTTCTAAGAAAATTTGAATTCCCGC
Cele.WB235	Cele.WB235_1:1000379	1	1000379	1000469	91	5	12	AGCCTAAGCC	AGCATAAGCCTA

Cele.WB235	Cele.WB235_1:1000380	1	1000380	1000606	227	14	12	GCCTAAGCCT	GCATAAGCCTAA
Cele.WB235	Cele.WB235_1:1012604	1	1012604	1013479	876	25	35	ATTTTCAATT	ATTTTCAATTCCGGCAATTTGCCGATTTGCCGGAA
Cele.WB235	Cele.WB235_1:1083222	1	1083222	1083538	317	8	35	CGGCAAATCG	CGGCAAATCGGTATTTTGCCGGAATTGAAAATTTT
Cele.WB235	Cele.WB235_1:1097742	1	1097742	1098271	530	23	23	GGCCTAGAAA	GGCCTAGAAATACTGAAAGTGGT
Cele.WB235	Cele.WB235_1:1108035	1	1108035	1108695	661	21	20	ATTTCTAGGC	ATTTCTAGGCTATCGATGAG
Cele.WB235	Cele.WB235_1:1126721	1	1126721	1126876	156	10	12	CCTAAGCCTA	CCTAAGCCTAAG
Cele.WB235	Cele.WB235_1:1136040	1	1136040	1136228	189	5	31	ATTCCCGCCA	ATTCCCGCCAATTTTTTCTCAGAAATTTTGA
Cele.WB235	Cele.WB235_1:1209321	1	1209321	1210686	1366	32	43	CCAGAAATTA	CCAGAAATTAGAATTTTCGGCAAATCGGCAAGCCGGCAAATTG
Cele.WB235	Cele.WB235_1:1211856	1	1211856	1212413	558	15	35	AAATCGGCAA	AAATCGGCAATATGCCTAAAATGAAAATTTCCGGC
Cele.WB235	Cele.WB235_1:1214097	1	1214097	1214254	158	7	11	GTACTGTAGG	GTACTGTAGGG
Cele.WB235	Cele.WB235_1:1278237	1	1278237	1279259	1023	33	19	TTCTAGGCCA	TTCTAGGCCATCTGATAAT
Cele.WB235	Cele.WB235_1:1317490	1	1317490	1319274	1785	47	38	GGGAATTCAA	GGGAATTCAAACTTTAATTTTTTTGAAAACATTTTGGC
Cele.WB235	Cele.WB235_1:1322604	1	1322604	1322925	322	5	42	TTTGCCGGAA	TTTCCCGGAAGTTTTCATTTTCGACAAATTACCGATTTGTCG
Cele.WB235	Cele.WB235_1:1362690	1	1362690	1362850	161	5	32	TCAGAAAATT	TCAGAAAATTTAAATTCCCGCCAAAATTTTTC
Cele.WB235	Cele.WB235_1:1408704	1	1408704	1409384	681	8	71	ATTTATGTGA	ATTTATGTGAAAATTCAGGTTTTTAGGGCATTTTTTGATGGAAATCTGAATATTTCCGCTTTTTCTGATGG
Cele.WB235	Cele.WB235_1:1429375	1	1429375	1430301	927	24	35	AAATTTGAAT	AAATCTGAATTTCCCGCAAAATTTTTTTCTCAGA
Cele.WB235	Cele.WB235_1:1430568	1	1430568	1430936	369	14	11	CTACAGTACT	CTACAGTAATT
Cele.WB235	Cele.WB235_1:1503613	1	1503613	1504297	685	18	31	GGTTACTGTA	GGTTACTGTAACTCAATTATAGGATTCTTCG
Cele.WB235	Cele.WB235_1:1564845	1	1564845	1565136	292	7	35	CGGCAAATCG	CGGCAAATCGGCAAATTGCCGGAATTGAAAATTTC
Cele.WB235	Cele.WB235_1:1593120	1	1593120	1594195	1076	5	189	TTTTTTTTGA	TTTTTTTTGACTATGGAATTATAAAGAAAATCGAATTTCAAAAAAGCATGTATTAGCCAATGTAAACGCGCTCCAATGATGATGTAGCCCACGTGGTATCAGAGTGTCTCTTTACGGCTTGATTTAAATACGTAGATCTACAAATTCACTTATCTGTTTTCTCACGGGTTTAGGATATTTTTTCTGTTA
Cele.WB235	Cele.WB235_1:1605584	1	1605584	1606132	549	6	35	AAATTTTCAA	AAATTTTCAATTCCGGCATTTTGCCGATTTGCCGG
Cele.WB235	Cele.WB235_1:1620047	1	1620047	1620586	540	9	49	CAATAATTTT	CAATAATTTTAAATGTAATTATCGATTTTTCTAAATTTTGCCGAAAAAT
Cele.WB235	Cele.WB235_1:1652306	1	1652306	1652642	337	16	21	TCTCCTGGTA	TCTCCTGGTAAATTTAAAAAT
Cele.WB235	Cele.WB235_1:1653240	1	1653240	1653360	121	7	12	AGCCTAAACC	AGCCTAAACCTG
Cele.WB235	Cele.WB235_1:1658366	1	1658366	1660394	2029	78	26	CAAAACCGGC	CAAAACCGGCAATTGCCGAAAACTTC

Cele.WB235	Cele.WB235_1:1664772	1	1664772	1664951	180	4	45
TCTGATCCTT	TCTGATCCTTCAGCTCCGAAGGATCACATGGGTACCTTCTGATGT						
Cele.WB235	Cele.WB235_1:1704545	1	1704545	1705424	880	24	30
AATTTGAATT	AATTTGAATTTTCCTGGATTTTTAAACGGA						
Cele.WB235	Cele.WB235_1:1755610	1	1755610	1758756	3147	154	20
CCACCAAAAA	CCACCAAAAAGGTTTCTAGG						
Cele.WB235	Cele.WB235_1:1760846	1	1760846	1762239	1394	64	20
TTCTAGGCCA	TTCTAGGCCACCAAAAAGGT						
Cele.WB235	Cele.WB235_1:1796469	1	1796469	1796919	451	9	43
AAATCGGCAA	AAATCGGCAAACCGGCAATTTGCCGAAATTGGAAATTTCTGGC						
Cele.WB235	Cele.WB235_1:1797802	1	1797802	1798215	414	9	43
GCCGGAATTG	GCCGGAATTGAAAATTTCGGGCAAATCGGTAAGCCGGCCAATT						
Cele.WB235	Cele.WB235_1:1807418	1	1807418	1807780	363	11	26
TTGCCGGTTT	TTGCTGGTTTTGGGAATTATCGGCAC						
Cele.WB235	Cele.WB235_1:1814405	1	1814405	1814778	374	9	35
TTTGCCGGAA	TTTGCCGGAAATTTTCAAATCCGGCAATTTGCCGA						
Cele.WB235	Cele.WB235_1:1815369	1	1815369	1815789	421	18	19
AAAATCAATA	AAAATCAATAAATCCTAAG						
Cele.WB235	Cele.WB235_1:1815369	1	1815369	1817144	1776	44	19
AAAATCAATA	AAAATCAATAAATCCTAAG						
Cele.WB235	Cele.WB235_1:1852477	1	1852477	1852561	85	4	21
CCTACTCTAG	CCTACTCTAGGCAGCCCCTAG						
Cele.WB235	Cele.WB235_1:1852567	1	1852567	1852667	101	5	20
CTAGGCAGAC	CTAGGCAGACTTAGCCTACC						
Cele.WB235	Cele.WB235_1:1884484	1	1884484	1884939	456	11	35
AAATCGGCAA	AAATCGGCAAATTGGCGGAATTGAAAATCTCCGGC						
Cele.WB235	Cele.WB235_1:1919828	1	1919828	1922029	2202	58	35
TTGCCGATTT	TTGCCGATTTGCCGGAAATTTTCATTTTTCGCAAA						
Cele.WB235	Cele.WB235_1:1935250	1	1935250	1936853	1604	40	35
GAAATTTTCA	GAAATTTTCAGTTTCGGCAAATTGCCGATTTGCCC						
Cele.WB235	Cele.WB235_1:1935250	1	1935250	1938219	2970	70	35
GAAATTTTCA	GAAATTTTCAGTTTCGGCAAATTGCCGATTTGCCC						
Cele.WB235	Cele.WB235_1:1946493	1	1946493	1947006	514	5	35
TTTGCCGGAA	TTTGCCGGAAATTTTCATTTTTGGCAAAGTGCCGA						
Cele.WB235	Cele.WB235_1:1949999	1	1949999	1950277	279	8	35
TTGCCGGAAA	TTGCCGGAAAGTATCAATTTCGGCTGATTGCCGAT						
Cele.WB235	Cele.WB235_1:1987299	1	1987299	1987635	337	5	38
AAAATCGATA	AAAATCGATAAATTTTCAGTATGCAGTGAAATTCTTGGA						
Cele.WB235	Cele.WB235_1:2014890	1	2014890	2015108	219	13	11
TTTTGCCGAA	TTTCGCCGAAC						
Cele.WB235	Cele.WB235_1:2111857	1	2111857	2112268	412	7	35
CAAACCGGCA	CAAACCGGCATTTTGCCGGAAATTAACATTTAGAG						
Cele.WB235	Cele.WB235_1:2139349	1	2139349	2139629	281	8	35
CGGCAAATTG	CGGCAAATTGCCGATTTGCCGGAAACTTTCAATTC						
Cele.WB235	Cele.WB235_1:2144021	1	2144021	2144187	167	10	15
AGATCTTGTT	AGATCATGTTTTCGG						
Cele.WB235	Cele.WB235_1:2165416	1	2165416	2165854	439	10	35
TTTGCCGGAA	TTTGCCGGAATTTCGGAATTCTGACAGTTTGCCGG						
Cele.WB235	Cele.WB235_1:2233376	1	2233376	2233627	252	6	43
CAAATCGGCA	CAAATCGGCAACTCGACAAATTTCCGAAATTAAAAATTTCCAG						
Cele.WB235	Cele.WB235_1:2251015	1	2251015	2251148	134	4	33
TAAGAAAAGT	TAAGAAAAGTTAGGCCACCAAACATTGGTGGAC						

Cele.WB235	Cele.WB235_1:2254186	1	2254186	2255835	1650	87	12	CACGCAACGC	CACGCAACGCTG
Cele.WB235	Cele.WB235_1:2263541	1	2263541	2264092	552	13	43	ATTTCCGGCA	ATTTCCGGCAAATGGGCAAGTCGGCAAATTGCCGAAATTAAAA
Cele.WB235	Cele.WB235_1:2269401	1	2269401	2269479	79	6	12	TAGGCTTAGG	TAGGCTTAGGCT
Cele.WB235	Cele.WB235_1:2276437	1	2276437	2276626	190	5	12	AGGCTTAGGC	AGGCTCAGGCTC
Cele.WB235	Cele.WB235_1:2333731	1	2333731	2334442	712	8	20	GGACTAGTTT	GGACTAGTTTTTGAGTAGAG
Cele.WB235	Cele.WB235_1:2333731	1	2333731	2334603	873	11	20	GGACTAGTTT	GGACTAGTTTTTGAGTAGAG
Cele.WB235	Cele.WB235_1:2364706	1	2364706	2365240	535	20	20	TTATTGATTT	TTATTAATTTTTCTAGGACA
Cele.WB235	Cele.WB235_1:2370746	1	2370746	2370842	97	6	12	AAGCCTAAGC	AAGCCTAAGCCT
Cele.WB235	Cele.WB235_1:2430196	1	2430196	2431177	982	5	175	GATGCACCAT	GATGCACCATGTCTTTGAAAGCCTATACTTCGGTTCATTTCGTAGCTGTCAAAAACTTTTTAACAACACATCTATAGAAATCAACATGCTTAATATTTCGTCAGTTGACAGTTTTTTGATAACTGCAATGAAATCCGAGATATGAGCTTTCAAAGTTGAGAGACCTCACTCGGTT
Cele.WB235	Cele.WB235_1:2524693	1	2524693	2524982	290	10	18	GCCTAGGAAT	GACTAGGAATCCAATATG
Cele.WB235	Cele.WB235_1:2542918	1	2542918	2543982	1065	56	19	TGTTACACGA	TGTTACACGAAAAAATAGAT
Cele.WB235	Cele.WB235_1:2557777	1	2557777	2557986	210	6	35	TTGCCGGAAT	TTGCCGGAATTAAAAATTTCTGGAAAATTGGCAAT
Cele.WB235	Cele.WB235_1:2650538	1	2650538	2650582	45	4	11	AAACTTGGGC	AAACTTGGGCC
Cele.WB235	Cele.WB235_1:2653859	1	2653859	2654031	173	4	43	TTGCCGATTT	TTGCCGATTTTCCTGAATTGTTCATTTCCGACAATATGACGAT
Cele.WB235	Cele.WB235_1:2738743	1	2738743	2738953	211	6	35	AATTTCAATT	AATTTCAATTCCGGCAATTTGCCGATTTGCCGGAA
Cele.WB235	Cele.WB235_1:2809445	1	2809445	2809654	210	5	35	GGAATTGAAA	GGAATTGAAAATTCCCGGCAAATCGGCAAATTGCC
Cele.WB235	Cele.WB235_1:2817881	1	2817881	2818157	277	5	43	TTGCCGATTT	TTGCCGGTTTCTCGATTTGCCGGATATTTACAATTTCACTATT
Cele.WB235	Cele.WB235_1:2840215	1	2840215	2840304	90	5	12	TAGGCTTAGG	TAGGCTGAGGCG
Cele.WB235	Cele.WB235_1:2844921	1	2844921	2845096	176	4	35	TTTGCCGATT	TTTGCCGATTTGCCAAAAAATTTTAATTCCGGCAA
Cele.WB235	Cele.WB235_1:2858185	1	2858185	2858357	173	4	43	AAATCGGCAA	AAATCGGCAATCCGGCAAATTGCGAAAAATGAAAATTTCCGGC
Cele.WB235	Cele.WB235_1:3028822	1	3028822	3029013	192	5	35	TTGCCGGAAA	TTGCCGGAAATTTTCAATTCCGGCAATTTGCCATT
Cele.WB235	Cele.WB235_1:3114471	1	3114471	3114865	395	8	35	CGATTTGCCG	CGATTTGCCGGAAATATTCATTTTCTGCAAATTGC
Cele.WB235	Cele.WB235_1:3115944	1	3115944	3116232	289	21	12	CTAAGCCTAA	CTATGCCTAAAC
Cele.WB235	Cele.WB235_1:3117070	1	3117070	3117200	131	4	26	GGCAGGCGTC	GGCAGGCGTCAAGCAGGCAGGTTTTA
Cele.WB235	Cele.WB235_1:3171655	1	3171655	3172409	755	29	26	CAGAGTCACT	CAGAGTCACTAAATTTGGTGAACGGC

Cele.WB235	Cele.WB235_1:3258597	1	3258597	3259299	703	58	12	CTAAGTCTAA	CTAAGTCTAAGC
Cele.WB235	Cele.WB235_1:3280164	1	3280164	3280242	79	6	12	AGGCTTAGGC	AGGCTTAGGCTT
Cele.WB235	Cele.WB235_1:3384983	1	3384983	3385061	79	6	12	GGCTTAGGCT	GGCTTAGGCTTC
Cele.WB235	Cele.WB235_1:3389969	1	3389969	3393329	3361	224	15	CTCACCACGA	CTCGCCACGATGGGT
Cele.WB235	Cele.WB235_1:3406451	1	3406451	3406664	214	5	32	AATTCAAATT	AATTCAAATTGTCAAGAATAATTTAGGTGGAA
Cele.WB235	Cele.WB235_1:3424298	1	3424298	3424548	251	6	43	TTGAAAATTT	TTGAAAATTTTCTAAAAATCGGCAAACCGGCAACTTGCCGGAA
Cele.WB235	Cele.WB235_1:3427447	1	3427447	3429127	1681	81	20	GGCAGACCTA	GGCAGACCTAGCCTACTCTA
Cele.WB235	Cele.WB235_1:3442604	1	3442604	3443969	1366	56	35	TTTCGGCAAT	TTTCGGCAATTTCGGCAACTGCCGGTATCGTGAAT
Cele.WB235	Cele.WB235_1:3459203	1	3459203	3459616	414	8	35	TCCGGCAAAT	TCCGGCAAATCGGAAATCTGCCGGAAGTCAAAAAT
Cele.WB235	Cele.WB235_1:3485156	1	3485156	3487576	2421	121	20	AAAAAATAGA	AAAAAATAGATTGATATACC
Cele.WB235	Cele.WB235_1:3553785	1	3553785	3554055	271	6	45	CTGAATCTTC	CTGAATCTTCATCTCCAAAGGATCACAAGGATCCTCCTTTATGAT
Cele.WB235	Cele.WB235_1:3602994	1	3602994	3603167	174	5	35	TTTGCCGGAA	TTTGCCGGAAATGTTCAATTCCGACAATTTGCCGG
Cele.WB235	Cele.WB235_1:3664200	1	3664200	3666098	1899	73	26	ATGGTTAGAG	ATGGTTAGAGTCATAATTTTAGGTGA
Cele.WB235	Cele.WB235_1:3712691	1	3712691	3712852	162	5	14	TTTTTGTAGA	TTTTGTAGAACGTC
Cele.WB235	Cele.WB235_1:3746094	1	3746094	3746497	404	9	45	TCTGATCCTT	TCTGATCCTTCAGCTCCGAAGGATCACATGGGTACCTTCTGATGT
Cele.WB235	Cele.WB235_1:3746917	1	3746917	3747230	314	7	45	CTGAAGGATC	CTGAAGGATCAGAACATCAGAAGGTGCCCATGTGATCCTTCGGAG
Cele.WB235	Cele.WB235_1:3763157	1	3763157	3765957	2801	112	25	TTTACTCTCT	TTTACTCTCTGGCTTCACAGAATAT
Cele.WB235	Cele.WB235_1:3835938	1	3835938	3837024	1087	26	34	TGCCGGAATT	TGTCGGAATTGAAATTTCCAGCAAATCGGCAAAT
Cele.WB235	Cele.WB235_1:3980324	1	3980324	3980429	106	7	15	TCGTGGTGAG	TCGTGGTGAGACCCT
Cele.WB235	Cele.WB235_1:3980821	1	3980821	3981021	201	6	40	TTTCTAGAAA	TTTCTAGAAAATTCGAGAAAATTCCGGAATGGTCCAGAAT
Cele.WB235	Cele.WB235_1:3990050	1	3990050	3990699	650	11	59	AAAACCAGTG	AAAACCAGTGCACATTTGAAATTCCCAATTCTCCACAGTTTTCAGTTTAAAAAAATTTG
Cele.WB235	Cele.WB235_1:4118875	1	4118875	4120034	1160	5	223	TTTCAGAATT	TTTCAGAATTTAAAAACGGCATTCCAAAAACTTTCACAACCGCCAAGCTCAAGCAAAAAAAGTAAACTTCAGTCATACCTTTCAGAGCAAACTTTGAAATTGAATGAAATAGACCATTTATTTGCAAAATTAGTAGATTTTCCAATTTTTAAAAATTAATATCTGGCTTCTAAATAACTTTTTTGAAAATCCCCGTAGCCATTTGTTGCAAATTTTGTGCTC
Cele.WB235	Cele.WB235_1:4167811	1	4167811	4168323	513	13	39	TATTTTTAAT	TATTTTTAATTTGGCCTACTTAAAAAGTAGGTCATGACT
Cele.WB235	Cele.WB235_1:4185588	1	4185588	4185648	61	4	15	TTTCCGGATC	TTTCCGGATCTACGT
Cele.WB235	Cele.WB235_1:4185829	1	4185829	4185964	136	9	15	AGATCCGGAA	AGATCCGGAAAACGT

Cele.WB235 Cele.WB235_1:4280022 1 4280022 4281181 1160 6 226
ACAAGAAGCT
ACAAGAAGCTTCACGTAGTTACAGAAATAGTAGATTTTTAGCCCTACCTTTTAGAGCGTATTTTATTATTTAATGAAAACTACCACTTATAGGCAAAATAGAGGGATTTTCGTAATTTTGAAAATTCATAAAT
CTCTTCAAAGTAATTTTTTTGCGAAATGTCTTTCAGAAACTTTGTAGTAAATTTTAAGCTCTTTTAGAATATATTAACAATATTCCAGTAGGT
Cele.WB235 Cele.WB235_1:4281487 1 4281487 4294537 13051 107 94
TCAAAATCTT TCAAAATCTTCTGTTCTCTAAATATTGGGTGCTTTTCAATGCTCTTTGTAGACAAATCAATGGGAAAATGATCAATTTCTGGAGGCAGTAAATC
Cele.WB235 Cele.WB235_1:4338841 1 4338841 4339537 697 4 174
CTTCAAGAGC
CTTCAAGAGCTCAGTGTTATTTCTGACATGCCAATCTCGACTCTCGACTTGAACATGTTACAAGGAATCGAAGACAACTTAAACAGCCTCCCAGCTGAAGAATCTGATGAGATCCGAGAGAAGTTAAATGAAC
TGAGAAGAAGAAAGCAAGAAAGTGACCAAGCTGAAGCTCTA
Cele.WB235 Cele.WB235_1:4465416 1 4465416 4465569 154 8 19
TTTTTCGTGT TTTTTCGTGTAACAATCTA
Cele.WB235 Cele.WB235_1:4540371 1 4540371 4544820 4450 169 26
AAATCCGCCG AAATCCGCCGCAGTGCCATTTTTCTG
Cele.WB235 Cele.WB235_1:4544868 1 4544868 4547696 2829 104 27
ACTCTCTGTG ACTCTCTGTGGCTTCACCGATTATTTT
Cele.WB235 Cele.WB235_1:4548525 1 4548525 4548705 181 14 12
TTTTTATTTT TTTTTATTTTTA
Cele.WB235 Cele.WB235_1:4573284 1 4573284 4573803 520 13 40
CATTCCAGAA CATTCCAGAATTTTCTCGATTTTTCTAGAAAGTTCTGGAA
Cele.WB235 Cele.WB235_1:4574052 1 4574052 4574607 556 34 15
GGGTCTCACC GGGTCTCACCACGAC
Cele.WB235 Cele.WB235_1:4958855 1 4958855 4958932 78 5 15
CGAAAAGGGG CGAAAAGGGGATCTG
Cele.WB235 Cele.WB235_1:4959104 1 4959104 4959196 93 5 15
CTCCCCTTTT CTCCCCTTTTCGTAT
Cele.WB235 Cele.WB235_1:4961695 1 4961695 4961802 108 6 15
CTCCCCTTTT CTCCCCTTTTCGTAT
Cele.WB235 Cele.WB235_1:5144853 1 5144853 5145677 825 51 15
CGTGGTGAGA CGTGGTGAGACCTTT
Cele.WB235 Cele.WB235_1:5152583 1 5152583 5153888 1306 45 29
AAGGATTTCC AAGGATTTCCCATTATGTCAATACATTGT
Cele.WB235 Cele.WB235_1:5176308 1 5176308 5176383 76 5 15
TCCCCTTTTC TCCCCTTTTCGTTGC
Cele.WB235 Cele.WB235_1:5563061 1 5563061 5563217 157 13 12
GAAATTTTTT GAAATTTTTTCC
Cele.WB235 Cele.WB235_1:5621701 1 5621701 5621793 93 6 15
TCCCCTTTTC TCCCCTTTTCGTTGC
Cele.WB235 Cele.WB235_1:5640355 1 5640355 5640950 596 5 119
GCCACCTTTA
GCCACCTTTAACCCTAATCCAAGCAGAGCAGGCCGTAGGCCTGCTAGGCGGTAGCGACACGCTCAAGCCCAACAGTCCGGGATGAGGGTCGCAGGCCCTCTTACTCTTAGAAACTTTAG
Cele.WB235 Cele.WB235_1:5975024 1 5975024 5975828 805 18 45
TCTGATCCTT TCTGATCCTTCAGCTCCGAAGGATCACATGGGTACCTTCTGATGT
Cele.WB235 Cele.WB235_1:5977228 1 5977228 5977451 224 5 45
AAGGATCAGA AAGGATCAGACCATCAGGAGGTACCCTTACGATCCTTCTTATCTG
Cele.WB235 Cele.WB235_1:6089071 1 6089071 6089158 88 5 17
AGGTAGTCAA AGGTAGTCAATGAGCAA
Cele.WB235 Cele.WB235_1:6514283 1 6514283 6515118 836 13 30
TGGTGGAACT TGGTGGAACTGGATTCGGCAGTGGAAATGC
Cele.WB235 Cele.WB235_1:6647571 1 6647571 6647736 166 11 15
GAGACCCATC GAGACCCACCGTGGT

Cele.WB235	Cele.WB235_1:6647885	1	6647885	6648325	441	11	40
TTCTGGAATG	TTCTGGAATGTTCCAGAACTTTCTAGAAAAATCGAGAAAA						
Cele.WB235	Cele.WB235_1:6771968	1	6771968	6773943	1976	79	25
GTGAAGCCAG	GTGAAGCCAGAGAGTAAAATATACT						
Cele.WB235	Cele.WB235_1:6854524	1	6854524	6854599	76	5	15
AGATCCGGAA	AGATCCGGAAAAACGT						
Cele.WB235	Cele.WB235_1:7003581	1	7003581	7003641	61	4	15
CGAAAAGGGG	CGAAAAGGGGATCAG						
Cele.WB235	Cele.WB235_1:7740676	1	7740676	7740751	76	5	15
TCGTGGTGAG	TCGTGGTGAGACCCA						
Cele.WB235	Cele.WB235_1:7740774	1	7740774	7741690	917	26	35
GCGGGAAATT	GCGGGAAATTCAAATTTTAAGGGAAAAAATTTTTG						
Cele.WB235	Cele.WB235_1:7741780	1	7741780	7742262	483	13	40
TTTCTAGAAA	TTTCTAGAAAATTCGGGAAAACTCTGGAATATTCCAGAAC						
Cele.WB235	Cele.WB235_1:7780058	1	7780058	7780728	671	39	16
AATTTTTCCG	AATTTTTCAGCAGCAA						
Cele.WB235	Cele.WB235_1:7868293	1	7868293	7868548	256	17	15
ACCCATCGTG	ACCCATCGTGGCGAA						
Cele.WB235	Cele.WB235_1:7868653	1	7868653	7868828	176	5	35
GGGAAATTTA	GGGAAATTTAAATTTTCTGTGAAAAATATTTTGGC						
Cele.WB235	Cele.WB235_1:7868937	1	7868937	7869457	521	13	40
GAATGTTCCA	GAATGTTCCAGAACTTTCTAGAAAAATCGAGAAAATTCTG						
Cele.WB235	Cele.WB235_1:8484982	1	8484982	8485111	130	4	32
AAAATCGAAA	AAAATCGAAAAATTCTCGTTTTTCAATTTTTA						
Cele.WB235	Cele.WB235_1:9636862	1	9636862	9637728	867	20	44
CTGTTTTCTG	CTGTTTTCTGAGCATTGTAGATTTTCGGATTGATGGCCGAAAGG						
Cele.WB235	Cele.WB235_1:9819851	1	9819851	9820331	481	31	15
CACGATGGGT	CACGATGGGTCTCAC						
Cele.WB235	Cele.WB235_1:9821430	1	9821430	9822959	1530	23	66
AATCAACCTA	AATCAACCTATTCAGTTGACAATGGTTTTCTTTTATCAAATTTTTTAAAATTTTAAAGAAAACACA						
Cele.WB235	Cele.WB235_1:9925221	1	9925221	9925359	139	5	37
ATAATTTTTC	ATAATTTTTCTTGAGTAATGAGAGACTTTTTCATAAT						
Cele.WB235	Cele.WB235_1:10130602	1	10130602	10133102	2501	100	25
TATTTACTCT	TATTTACTCTAATTTTCTGCCAATT						
Cele.WB235	Cele.WB235_1:10204129	1	10204129	10219199	15071	148	68
ACTGAATAGG	ACTGAATAGGTTGATTTGTGTTTTCTTTCTGAAATTCTAAGAATTTTGGTAAAAGAAAACCATTGTCA						
Cele.WB235	Cele.WB235_1:10266294	1	10266294	10276924	10631	44	231
GCTGAATTCA							
GCTGAATTCAGTTGGGTAGGATGTGACTTCATGGCTGAGGGGAACATCTTGAACATCATCAGCACGATGTGTAGTGTCGAGTGGTCCATCGTATTGATCAGAAATCGGGTAATCAGAATGAGCAGAAGACTTCTTGAACAATCCAGTAATCTTCAATTTCAATGATGGCTCGTCCTTCTTCTCCGATTCTTCAATCACAACTGGGATGACATACTCTGGCAGCTTCTCATA							
Cele.WB235	Cele.WB235_1:10291633	1	10291633	10291724	92	6	14
GCTATACGCT	GCTATACGCTATAC						
Cele.WB235	Cele.WB235_1:10404549	1	10404549	10404632	84	6	12
TAGGCTTAGG	TAGGCTTAGTCT						
Cele.WB235	Cele.WB235_1:10468958	1	10468958	10469050	93	6	15
CGAAAAGGGG	CGAAAAGGGGATCTG						
Cele.WB235	Cele.WB235_1:10469225	1	10469225	10469317	93	6	15
TCCCCTTTTC	TCCCCTTTTCGTATC						
Cele.WB235	Cele.WB235_1:10650908	1	10650908	10650980	73	4	18
AAAAGTTACA	AAAAGTTACAGTGTCCTT						
Cele.WB235	Cele.WB235_1:10851626	1	10851626	10855375	3750	23	163
GCATAGTCAA							

GCATAGTCAAAAATACAAGAATATGCCTATCAAATTATAATAGTTTGTACGGAAGTATTTTTTAAAAACTTGATAAAAATATATAAAAGCTGATTTTTTAAAAAATTCAAAAGTATGGGAAAATCATATGGAA
TCATTCTTTTTTATCTCAAAAAACTGTTCA

Cele.WB235	Cele.WB235_1:10913365	1	10913365	10914502	1138	5	94
TGTAGACAAA	TGTAGACAAATCAATGGAGAATCATCAATTTCTGAAGGCAGTAATTCTCAAAATCTTCTGTTCTCTAAATATTGGGTGCTTTTCGATGTCCTT						
Cele.WB235	Cele.WB235_1:10945938	1	10945938	10952999	7062	238	32
GCACCAAATA	GCACCAAATATCACCAATTTAGCACCGATAGA						
Cele.WB235	Cele.WB235_1:10953026	1	10953026	10953491	466	4	26
TTTTTGAAAA	TTTTTGAAAAATGGCATTGCGGCGGA						
Cele.WB235	Cele.WB235_1:10977031	1	10977031	10977503	473	10	35
TTGCCGATTT	TTACCGATTTGCCGGAATTTTTCAATTCCGGCAAT						
Cele.WB235	Cele.WB235_1:10993314	1	10993314	10993636	323	8	35
GCCGGAATTG	GCCGGAATCGAAATTTTCCGGCAAATCTACAAACT						
Cele.WB235	Cele.WB235_1:11032650	1	11032650	11032782	133	12	11
TATGCCTAGC	TATGCCTAGCC						
Cele.WB235	Cele.WB235_1:11060982	1	11060982	11061207	226	15	15
CGATGCTCCA	CGATGCTCCACCCGG						
Cele.WB235	Cele.WB235_1:11061949	1	11061949	11062380	432	11	34
TTCCGGCAAA	TTCCGGCAAATCGGAAAATTGCCGGATATTAAAT						
Cele.WB235	Cele.WB235_1:11069544	1	11069544	11069616	73	6	12
CGATCTCCAC	CGATCTCCACCT						
Cele.WB235	Cele.WB235_1:11084941	1	11084941	11085053	113	7	14
AGCTTGAAGC	AGCTTGAAGCTTGG						
Cele.WB235	Cele.WB235_1:11113480	1	11113480	11113648	169	4	42
TGCCGGAAAT	TGCCGGAAATTTCAAACCCGGTAGTTTGCCCATTTGCCGAGC						
Cele.WB235	Cele.WB235_1:11144860	1	11144860	11145055	196	13	15
TCGTGGTGAG	TCGTGGTGAGACCCA						
Cele.WB235	Cele.WB235_1:11145522	1	11145522	11145582	61	5	12
TATACATATA	TATACATATACA						
Cele.WB235	Cele.WB235_1:11155001	1	11155001	11155099	99	7	14
AACTACTAAC	AACTACTAACTACT						
Cele.WB235	Cele.WB235_1:11186372	1	11186372	11187503	1132	15	35
TTCCGGCAAA	TTCCGGCAAATCGGCAAATTGCCGGGACTGAAAAT						
Cele.WB235	Cele.WB235_1:11190690	1	11190690	11191449	760	4	35
TCGGCAAATT	TCGGCAAATTACGGAAAATGAAAATTTCCGGCAAA						
Cele.WB235	Cele.WB235_1:11201002	1	11201002	11201638	637	9	35
AATTGCCGAA	AATTGCCAAATTTCTCGTTTTTGGCAATTTTCAAA						
Cele.WB235	Cele.WB235_1:11253589	1	11253589	11254333	745	60	12
CGTTGCGTGT	CGTTGCATGTCG						
Cele.WB235	Cele.WB235_1:11273722	1	11273722	11273834	113	7	16
CTACATGCCT	CTACATGCCTATATGC						
Cele.WB235	Cele.WB235_1:11273722	1	11273722	11274842	1121	68	16
CTACATGCCT	CTACATGCCTATATGC						
Cele.WB235	Cele.WB235_1:11312599	1	11312599	11313143	545	18	26
ACCTACAGTA	ATCTACAGTACCCCTATTCTAGAGTG						
Cele.WB235	Cele.WB235_1:11313617	1	11313617	11315164	1548	48	32
GCAATTTTTA	GCAATTTTTAGGCTAAAAATCACTAAAAAACT						
Cele.WB235	Cele.WB235_1:11342672	1	11342672	11342750	79	6	12
TATATGTATA	TATATGTATATG						
Cele.WB235	Cele.WB235_1:11349013	1	11349013	11350796	1784	47	35
GGCAAATCAG	GGCAAATCAGCAAATTGCGGGAATTGAAAATTTCC						
Cele.WB235	Cele.WB235_1:11357272	1	11357272	11357373	102	5	20
AATAGATTGT	AATAGATTGTTACACGAAAG						

Cele.WB235	Cele.WB235_1:11383715	1	11383715	11384049	335	14	20
AAAAATCAAT	AAAAATCAATAATTTCATCG						
Cele.WB235	Cele.WB235_1:11384374	1	11384374	11386353	1980	95	20
ATTGATTTTT	ATTGATTTTTGAGAAAATT						
Cele.WB235	Cele.WB235_1:11404108	1	11404108	11404462	355	10	30
CCTTGGGCTC	CCTTGGGCTCCCTGAGCTCCAAATTGTGTT						
Cele.WB235	Cele.WB235_1:11420316	1	11420316	11421039	724	15	11
GTACTGTAGG	GTACTGTAGTG						
Cele.WB235	Cele.WB235_1:11425096	1	11425096	11425445	350	9	35
ATTCCGGCAA	ATTCCGGCAATTTGCCGATTTTCCAGAAATTTTCA						
Cele.WB235	Cele.WB235_1:11432554	1	11432554	11432811	258	6	34
ATGCACCATG	ATGCACCATGCCAAAAAATATATCCAAAACCCCG						
Cele.WB235	Cele.WB235_1:11450427	1	11450427	11450505	79	6	12
AAGCCTAAGC	AAGCCTAAGCCT						
Cele.WB235	Cele.WB235_1:11451202	1	11451202	11451316	115	9	12
GCTTAGGCTT	GCTTGGGCTTAG						
Cele.WB235	Cele.WB235_1:11455940	1	11455940	11456197	258	6	34
ATGCACCATG	ATGCACCATGCCAAAAAATATATCCAAAACCCCG						
Cele.WB235	Cele.WB235_1:11470915	1	11470915	11470999	85	7	12
GGCTTAGGCT	GGCTTAGGCTTA						
Cele.WB235	Cele.WB235_1:11474410	1	11474410	11474625	216	5	43
CGGCAAATCG	CGGCAAATCGGCAAATCGGCAACTTGCCGGAATTGAAAATTTC						
Cele.WB235	Cele.WB235_1:11480157	1	11480157	11480277	121	8	15
TCGTGGTGAG	TCGTGGTGAGACCCA						
Cele.WB235	Cele.WB235_1:11480313	1	11480313	11481528	1216	24	35
TGGCGGGAAA	TGGCGGGAAATTCAAATTTTCAGTGAAAAAAATTT						
Cele.WB235	Cele.WB235_1:11484951	1	11484951	11485029	79	6	12
TAGGTCTAGG	TAGGTCTAGGTC						
Cele.WB235	Cele.WB235_1:11485772	1	11485772	11485879	108	5	19
TCAAAAATCA	TCAAAAATCAATAATTTCA						
Cele.WB235	Cele.WB235_1:11486089	1	11486089	11486222	134	7	19
TTATCGATTT	TTATCGATTTTTCCGGAAA						
Cele.WB235	Cele.WB235_1:11485735	1	11485735	11486997	1263	20	19
AAAAATCGAT	AAAAATCAATAATTTCATC						
Cele.WB235	Cele.WB235_1:11532642	1	11532642	11533117	476	17	19
TTTCACAAAA	TTTCACAAAAATCGATAAT						
Cele.WB235	Cele.WB235_1:11531919	1	11531919	11534041	2123	45	19
AAAATCGATA	AAAATCGATAATTTCTCAT						
Cele.WB235	Cele.WB235_1:11540263	1	11540263	11540521	259	16	12
AAGCCAAAGC	AAGCCAAAGAAT						
Cele.WB235	Cele.WB235_1:11655695	1	11655695	11656421	727	60	12
AGGCTCAGGC	AGGGTCAGGCTC						
Cele.WB235	Cele.WB235_1:11656920	1	11656920	11657791	872	54	16
GCATGTAGGC	GCATGTAGGCATGTAG						
Cele.WB235	Cele.WB235_1:11694858	1	11694858	11694997	140	13	11
TTTTGCACAT	TTTTCCACATT						
Cele.WB235	Cele.WB235_1:11763187	1	11763187	11763333	147	6	11
AGTACTGTAG	AGTACTGTAGG						
Cele.WB235	Cele.WB235_1:11829562	1	11829562	11829989	428	5	42
TCGGCAAATC	TCGGCAAATCGGCTATTCCTGGTTTTTCAAAATTTGCCGAGC						
Cele.WB235	Cele.WB235_1:11840154	1	11840154	11840231	78	7	11
CAAAAAATGT	CAAAAAATGTC						

Cele.WB235	Cele.WB235_1:11933362	1	11933362	11933554	193	15	12
TTAGGCTTAG	TTACGCTTAGGC						
Cele.WB235	Cele.WB235_1:11984232	1	11984232	11984346	115	7	12
CTAAGCCTAA	CGAAGCCTAAGC						
Cele.WB235	Cele.WB235_1:12093567	1	12093567	12094297	731	59	11
CACATTTTTT	CACATTTTTTG						
Cele.WB235	Cele.WB235_1:12109993	1	12109993	12110203	211	7	30
GTCATTATCA	GTCATTATCAGCTTCTAGTGTCTTAGAAGA						
Cele.WB235	Cele.WB235_1:12119896	1	12119896	12120034	139	10	12
TAAGCCTAAG	TAAGCCTAAGCG						
Cele.WB235	Cele.WB235_1:12129305	1	12129305	12129768	464	41	11
TTGCACATTT	TTGCACATTTT						
Cele.WB235	Cele.WB235_1:12175889	1	12175889	12176021	133	9	12
GCCTAAGCCT	GCCTAAGCCTAA						
Cele.WB235	Cele.WB235_1:12192426	1	12192426	12192752	327	4	73
AATAAAATAT	AATAAAATATAGAAAATCCTTATGACACACTTAAGCCTAAAGGCCCGAAAAACATACTAGGATGCCCAACTGG						
Cele.WB235	Cele.WB235_1:12223508	1	12223508	12223556	49	4	12
GCCTAAGCCT	GCCTAAGCCTAG						
Cele.WB235	Cele.WB235_1:12258128	1	12258128	12258286	159	5	34
ATGCACCATG	ATGCACCATGCCAAAAAATATATCCAAAACTCCG						
Cele.WB235	Cele.WB235_1:12277734	1	12277734	12277806	73	5	12
AAGCCTAAGC	AAGCCTAAACCG						
Cele.WB235	Cele.WB235_1:12479088	1	12479088	12479300	213	16	12
GAAATTTTTT	GAAATTTTTTCC						
Cele.WB235	Cele.WB235_1:12479365	1	12479365	12479989	625	49	12
AAAAAATTTC	AAAAAATTTCGG						
Cele.WB235	Cele.WB235_1:12480947	1	12480947	12481197	251	5	50
ACACATGCTA	ACACATGCTATAACATTGTCTCCTTCCCCTAGTGCACGAGAGCGGCCGTT						
Cele.WB235	Cele.WB235_1:12562161	1	12562161	12562497	337	20	16
TGATCTGATG	TGATCTGATGATCTGA						
Cele.WB235	Cele.WB235_1:12585721	1	12585721	12585826	106	5	21
ACCACCCCTA	ACCACCCCTACAACTACCACC						
Cele.WB235	Cele.WB235_1:12612870	1	12612870	12613263	394	5	32
GCTGATTGGT	GCTGATTGGTTCAAAAGTGGGCGTGGCCAATC						
Cele.WB235	Cele.WB235_1:12620508	1	12620508	12620574	67	5	12
TACCTATACC	TACCTATACCTA						
Cele.WB235	Cele.WB235_1:12684455	1	12684455	12684521	67	5	11
TTTGGAGCGG	TTTGGAGCGGA						
Cele.WB235	Cele.WB235_1:12892561	1	12892561	12892756	196	5	39
TATTTTTAAT	TATTTTTAATTTAACCTACTTAAAAAGTAGGTCATGACC						
Cele.WB235	Cele.WB235_1:12893074	1	12893074	12893850	777	11	23
ACTACAAACT	ACTACAAACTACAAACTATGAAA						
Cele.WB235	Cele.WB235_1:12899142	1	12899142	12899606	465	31	15
AAAAGGGGAT	AAAAGGGGATCTGCA						
Cele.WB235	Cele.WB235_1:12910137	1	12910137	12910261	125	6	21
AATCCAATAT	AATCAAATATGGTTGAAATCT						
Cele.WB235	Cele.WB235_1:12955954	1	12955954	12956201	248	13	19
CAAGGCACTA	CAAGGTACTAGAAATGCGC						
Cele.WB235	Cele.WB235_1:12977769	1	12977769	12978231	463	10	35
GCCGGAAATT	GCCGGAAATTTTCAATTCTGGCAGTTTACCAATTT						
Cele.WB235	Cele.WB235_1:13014176	1	13014176	13014829	654	16	35
ATTGCCGGAA	ATTGCCGGAATTGAAAAATGCAGGCAAATCGGCAT						

Cele.WB235	Cele.WB235_1:13019817	1	13019817	13020208	392	8	14
AGTTTGTAGT	AGTCTGTAGTTTGT						
Cele.WB235	Cele.WB235_1:13085679	1	13085679	13085769	91	4	22
TCGAGAAAAA	TCGAGAAAAAATCTGAAAAAAT						
Cele.WB235	Cele.WB235_1:13093190	1	13093190	13093270	81	5	16
AATACGGGAA	AATACGGGAATACGGG						
Cele.WB235	Cele.WB235_1:13102088	1	13102088	13102201	114	5	22
TTTTTTCTCG	TTTTTTCTCGAATTTTTTCAGA						
Cele.WB235	Cele.WB235_1:13154184	1	13154184	13155089	906	19	39
TTAAAAAGTA	TTAAAAAGTAGGTCATGACCTAATTTTAATATGACCTAC						
Cele.WB235	Cele.WB235_1:13230567	1	13230567	13230940	374	12	11
GTACTGTAGG	GTACTGTAGGA						
Cele.WB235	Cele.WB235_1:13231021	1	13231021	13231669	649	15	40
GAAAATTCTG	GAAAATTCTGAAATGTTCCAGAACTTTCTAGAAAAATCGA						
Cele.WB235	Cele.WB235_1:13276960	1	13276960	13277319	360	18	20
AAAATAGATT	AAAATAGATTGTTACACGAA						
Cele.WB235	Cele.WB235_1:13277345	1	13277345	13277527	183	7	26
CTCTGGCAGT	CTCTGGCAGTTCACTAAAATAAGTGG						
Cele.WB235	Cele.WB235_1:13404330	1	13404330	13408464	4135	159	26
TTTTAGGTGA	TTTTAGGTGAATGGTTAGAGTCATAA						
Cele.WB235	Cele.WB235_1:13453071	1	13453071	13453246	176	5	35
TGAAAATTTC	TGAAAATTTCCGGCAAATCGGCAAATTGTCGGAAT						
Cele.WB235	Cele.WB235_1:13499215	1	13499215	13500226	1012	11	14
TAGTTTGTAG	TAGTTTGTAGTTTG						
Cele.WB235	Cele.WB235_1:13500544	1	13500544	13500919	376	11	14
TACAAACTAC	TATAAACTACAAAC						
Cele.WB235	Cele.WB235_1:13600712	1	13600712	13601694	983	52	14
TGTAGTATGT	TGTAGTATGTAGTT						
Cele.WB235	Cele.WB235_1:13610087	1	13610087	13613799	3713	116	32
TGAATTCCCG	TGAATTCCCGCCAAAATTTTTCACAGAAATTT						
Cele.WB235	Cele.WB235_1:13613895	1	13613895	13615295	1401	35	40
GAATTTTCTC	GAATTTTCTCGAATTTTCCAGAAGGTTCTGGAACATTCCA						
Cele.WB235	Cele.WB235_1:13689387	1	13689387	13689549	163	13	12
TATACCTATA	TATACCTATACC						
Cele.WB235	Cele.WB235_1:13689822	1	13689822	13689960	139	11	12
ATAGGTATAG	ATAGGTATAGGT						
Cele.WB235	Cele.WB235_1:13768184	1	13768184	13768352	169	7	21
CAAAATTCGT	CAAAATTCGTCCGAAAATGCT						
Cele.WB235	Cele.WB235_1:13800189	1	13800189	13800502	314	9	35
AATTCCGGCA	AATTCCGGCAATTTGCCAATTTACCGGACAATTTA						
Cele.WB235	Cele.WB235_1:13883897	1	13883897	13885190	1294	19	68
TTGGTTGAGG	TTGGTTGAGGCTAGAAAATGTTGTAGTTTGTAGTGTGTTAGCCTCAACCAAAATTAATTTTTTTTTAA						
Cele.WB235	Cele.WB235_1:13925940	1	13925940	13926100	161	8	20
AAAAAATAGA	AAAAAATAGATTGACATACG						
Cele.WB235	Cele.WB235_1:13927845	1	13927845	13927905	61	5	12
GCCTAAGCCT	GCCTAAGCCTGA						
Cele.WB235	Cele.WB235_1:13929375	1	13929375	13929595	221	5	63
TTTTTGACTA	TTTTTGACTACGAGTGTCTTTTTCGCTACTGAGCGAAAAAATAGCTACATTCGCACTTTTGTC						
Cele.WB235	Cele.WB235_1:13935628	1	13935628	13936488	861	15	64
GTAGTCAAAA	GTAGTCAAAAAGTCAAAAGTGCGAATGTAGCTATTTTTTCAGTCAGTAGCGAAAAAGACATTCA						
Cele.WB235	Cele.WB235_1:13960468	1	13960468	13960552	85	4	21
ATATTTTGTG	ATATTTTGTGACATTTTTATT						

Cele.WB235	Cele.WB235_1:13968489	1	13968489	13968559	71	6	10	GATTTTCATA	GATTTTCATA
Cele.WB235	Cele.WB235_1:14002210	1	14002210	14002386	177	11	16	ATCTAAGGAT	ATCTAAGGATCTAAGT
Cele.WB235	Cele.WB235_1:14005076	1	14005076	14005524	449	13	16	AGATCCTTAG	AGATCCTTAGATCCTT
Cele.WB235	Cele.WB235_1:14006294	1	14006294	14006390	97	6	16	AGGATCTAAG	AGGATCTAAGGATCTA
Cele.WB235	Cele.WB235_1:14024312	1	14024312	14025229	918	5	179	CTACCCAAAT	CTACCCAAATTTTTGAAATTTGGAAATTTTTCAAAAATTCTCAAATTTTAAAAATCCCATAATTCAGCCAATTTTTGAGATTTTCAGATATTTTTTAATTGAAAATTCTGTTTGTCTTATCCATTAAACTATATCGCTGCATCTACATTTTTCACAGTACCCCATGACTTTTCCCGATT
Cele.WB235	Cele.WB235_1:14033367	1	14033367	14034207	841	24	40	TTCTGGAAAA	TTCTGGAAAAGTCGGTAAAATTTTGGAGTGTTCCCGAACT
Cele.WB235	Cele.WB235_1:14101896	1	14101896	14102785	890	4	62	GTAGTTTGTA	GTAGTTTGTAGTGTGAGCCTCAACCAAACTAGCCAAAGCTTGAGTGAGGCTATGGTGATTTT
Cele.WB235	Cele.WB235_1:14128159	1	14128159	14128469	311	16	19	AACAATCTAT	AACAATCTATTTTTCGTGT
Cele.WB235	Cele.WB235_1:14137893	1	14137893	14138026	134	7	19	CCCTGATACC	CCCTGATACCCCCGGTCCC
Cele.WB235	Cele.WB235_1:14148103	1	14148103	14148989	887	5	177	CTAACAAAAT	CTAACAAAATAATTAGCGTAAAATTTCCTACATTTTACTAGTTAACAATATTTTGATAGCATCAGTGGTTTTTGAGATACAGAAGTTATAAGATGTACGTCCAAAAATGATTGTGAGCTGCTCTTATCAAAAATTACCATAACTTTTTACTCACTAACGGTATCAAAAATGTTTTAA
Cele.WB235	Cele.WB235_1:14257569	1	14257569	14258742	1174	31	34	AAATTTGAAT	AAATTTGAATTTCCGACCAAAAAGTTTTCTCAAA
Cele.WB235	Cele.WB235_1:14267336	1	14267336	14267775	440	17	22	GCACGGCCTG	GCATGGCCTGACGGTCCTGAGG
Cele.WB235	Cele.WB235_1:14290714	1	14290714	14290861	148	8	15	GGGGGCAACA	GGGGGCAACAAATTA
Cele.WB235	Cele.WB235_1:14292866	1	14292866	14293013	148	8	15	TGTTGCCCCC	TGTTGACCCCTATTA
Cele.WB235	Cele.WB235_1:14321889	1	14321889	14321969	81	4	20	ATATCAAAAA	ATACCAAAAAATAGATTGGC
Cele.WB235	Cele.WB235_1:14324192	1	14324192	14324420	229	4	14	TGTAGTTTGT	TGTAGTTTGTAGTG
Cele.WB235	Cele.WB235_1:14329713	1	14329713	14330118	406	4	101	GCATTTGCAT	GCATTTGCATATAGAGATCAGATAAAAGTTAGAATTTTCCAGCCGTTGCAAGTGCGCTCTATTGAGAACATTTTGAAAATTGAATGTTTTCAAATTAGAGC
Cele.WB235	Cele.WB235_1:14332472	1	14332472	14332641	170	5	23	GCACCATAAG	GCATCATAAGGACTCTAGTTGAT
Cele.WB235	Cele.WB235_1:14338207	1	14338207	14338743	537	26	18	TTGTAGATCA	TTGTAGATCAACGGGGTC
Cele.WB235	Cele.WB235_1:14402777	1	14402777	14402900	124	4	11	GTACTGTAGG	GTACTGTAGGA
Cele.WB235	Cele.WB235_1:14403308	1	14403308	14403517	210	6	35	AATTTAAATT	AATTTAAATTTTCTGCGAAAAATGTTTTAGCGGGA
Cele.WB235	Cele.WB235_1:14430493	1	14430493	14430609	117	10	12	ATATGTATAT	ATATGTATTTGT
Cele.WB235	Cele.WB235_1:14443057	1	14443057	14443637	581	8	73	TGTCGGCCGC	TGTCGGCCGCTACCAAATAAGCACCTTTTAGCACTACGTTGCGCACACACACCAAACTGCGGAACCCCTAACA
Cele.WB235	Cele.WB235_1:14449256	1	14449256	14449536	281	8	35	AAAATTTCCG	AAAATTTCCGGCAAATCGGCAATTTACCGAAAGTG

Cele.WB235	Cele.WB235_1:14452267	1	14452267	14454642	2376	68	35
GAAATTTTCA	GAAATTTTCATTTTCGGCGAAGTGCCGATTTGCCG						
Cele.WB235	Cele.WB235_1:14477083	1	14477083	14477203	121	6	20
GTGAAATTTT	GTGAAATTTTGAAGTTTTAG						
Cele.WB235	Cele.WB235_1:14481352	1	14481352	14481947	596	17	35
CGGCAAATTG	CGGCAAATTGCCGGAATCGAAAATTTCCGGCAAAC						
Cele.WB235	Cele.WB235_1:14554105	1	14554105	14554484	380	9	43
TTTGCCGGTT	TTTGCCGGTTTGCCGATTTGCCGGAAATTTTCATTTTCGGCAA						
Cele.WB235	Cele.WB235_1:14619236	1	14619236	14619481	246	6	35
CAAATCGGCA	CAAATCGGCAAATTTTCGGTGTTGAAAAATTCCGG						
Cele.WB235	Cele.WB235_1:14668547	1	14668547	14668607	61	4	15
CCACGATGGG	CCACGATGGGTCTCG						
Cele.WB235	Cele.WB235_1:14668659	1	14668659	14668849	191	5	40
CCAGAAGGTT	CCAGAAGGTTCTAGAACAATCCAGAATTTTTTCGAATTTT						
Cele.WB235	Cele.WB235_1:14686490	1	14686490	14687331	842	22	40
TCTAGAAAGT	TCTAGAAAGTTCTGGAACATTCCAGAACTTTTTCGAAATT						
Cele.WB235	Cele.WB235_1:14764447	1	14764447	14764676	230	5	34
GAAATTTCAA	GAAACTTCAACTCCTGCAAATTGCCGATTTGCCG						
Cele.WB235	Cele.WB235_1:14772871	1	14772871	14773518	648	19	34
CGGAAATTTC	CGGTAATTTCAATTCCGGCAAGTTGCCGATTTCC						
Cele.WB235	Cele.WB235_1:14811659	1	14811659	14812009	351	10	35
AATTCCGGCA	AATTACGGCACTTTGTCGATTTTCCTGAAATTTTC						
Cele.WB235	Cele.WB235_1:14822628	1	14822628	14822877	250	7	35
CGGTTTTGGA	CGGTTTGGGAAATTTTCGGTAATTTTGACAATTGC						
Cele.WB235	Cele.WB235_1:14843491	1	14843491	14843700	210	6	35
TTTCAATTCC	TTTCAATTCCGGCAATTCGCCGATTTACCAAAAAC						
Cele.WB235	Cele.WB235_1:15025277	1	15025277	15025549	273	10	21
AACTTTTTGG	ACCTTTTTGGCAGATTTTCAA						
Cele.WB235	Cele.WB235_1:15072265	1	15072265	15072421	157	13	12
TTAGGCTTAG	TTAGGCTTAGGC						
Cele.WB235	Cele.WB235_2:0	2	0	161	162	13	12
CCTAAGCCTA	CCTAAGCCTAAG						
Cele.WB235	Cele.WB235_2:144001	2	144001	144084	84	6	12
ATTTCTTGCA	ATTTCTTGCAAC						
Cele.WB235	Cele.WB235_2:233654	2	233654	235183	1530	40	35
TTTCCGGCAA	TTTCCGGCAAATCGGCCACTTGCCGGATTTGAAAA						
Cele.WB235	Cele.WB235_2:368393	2	368393	368601	209	10	18
TAAGCCTAAG	TAAGCCTATGTCTAAGAA						
Cele.WB235	Cele.WB235_2:451705	2	451705	452550	846	32	15
TCTCACCACG	TCTCGCCACGAAGGG						
Cele.WB235	Cele.WB235_2:463793	2	463793	464368	576	9	20
CAATTTTTGA	CAATTTTTGAGTTAAAAAAT						
Cele.WB235	Cele.WB235_2:573638	2	573638	573888	251	20	12
TAGGCTTAGG	TAGGCTTAGGCT						
Cele.WB235	Cele.WB235_2:586310	2	586310	587078	769	12	64
AAATTTTGTA	AAATTTTGTAGTTTGTAGTCTAGCAGACCCATATTGAATACCAGCAAGTTGGGTCTGCCAAATC						
Cele.WB235	Cele.WB235_2:632493	2	632493	632813	321	8	40
TTTCTAGAAA	TTTCTAGAAAGTTCTGGAACATTCGAGAATTTTCTCGATT						
Cele.WB235	Cele.WB235_2:632106	2	632106	632999	894	4	35
GAAAATTTAA	GAAAATTTAAATTTCCCTCCAAAATTTTTTTCTCA						
Cele.WB235	Cele.WB235_2:633057	2	633057	633477	421	27	15
GGGTCTCACC	GGGTCTCACCACGAT						

Cele.WB235	Cele.WB235_2:633567	2	633567	633717	151	4	40
CAGAAGGTTC	CAGAAGGTTCTAGAACAATCCAGAATTTTTTCGAATTTTC						
Cele.WB235	Cele.WB235_2:633813	2	633813	633947	135	4	11
GTACTCCTAC	GTACTCCTACA						
Cele.WB235	Cele.WB235_2:642340	2	642340	642984	645	25	23
GGCCACCAAT	GGCCACCAATTTCTTAAAAATTC						
Cele.WB235	Cele.WB235_2:645177	2	645177	645231	55	4	12
GGCTCAGGCT	GGCTCAGGCTCA						
Cele.WB235	Cele.WB235_2:674091	2	674091	674259	169	9	16
CCTACGTGCC	CCTACGTGCCTTCGTG						
Cele.WB235	Cele.WB235_2:690749	2	690749	690809	61	4	15
GTAGATCTTG	GTAGATCTTGTTTTG						
Cele.WB235	Cele.WB235_2:694364	2	694364	699056	4693	287	15
GAGACCCATC	GAGACCCATCGTGGT						
Cele.WB235	Cele.WB235_2:694391	2	694391	699449	5059	247	15
GGTGAGACCC	GGTGAGACCCATCGT						
Cele.WB235	Cele.WB235_2:851256	2	851256	851616	361	5	31
TGTCTAGCGA	TGTCGAGCGAGACCTCAGAAGAGAGCTGAGT						
Cele.WB235	Cele.WB235_2:857683	2	857683	857758	76	5	15
ATGGGTCTCG	ATGGGTCTCGCCACG						
Cele.WB235	Cele.WB235_2:863584	2	863584	866193	2610	35	72
TCCATCCAGC	TCCATCCAGCTGTGGTTGGTCTCTGGGTGAATCCTCGAGAAGTATCACGAGTAGTACCATTGGCTCCGTTTG						
Cele.WB235	Cele.WB235_2:875486	2	875486	875668	183	12	14
TAGGATCTAG	TAGGATCTAGAGTA						
Cele.WB235	Cele.WB235_2:878150	2	878150	878348	199	15	12
ACCTAGACCT	ACCTAGACCTAG						
Cele.WB235	Cele.WB235_2:882370	2	882370	882522	153	5	11
TGTAGGGGTA	TGTAGGGGTAC						
Cele.WB235	Cele.WB235_2:884525	2	884525	885363	839	4	184
GTTCAAAAGA	GTTCAAAAGATACAAAATTTATCTACACATCTACAAGTTAAACTTGCAACTTAGTTAACCAAATTCAAAAGTTATGAGCCGGGGAACTATAATAACCAACGTTTCACACTAAAAAAGCCCAAAAAAATTCGAAAATCATTTTTATCTCATGACGCGGACAAGCTACAAATGTCTAGTTAGGCTT						
Cele.WB235	Cele.WB235_2:887413	2	887413	887570	158	8	20
CAATCTATTT	CAATCTATTTTCTGGTATGC						
Cele.WB235	Cele.WB235_2:891126	2	891126	892012	887	12	59
CAAATTTTTT	CAAATTTTTTTAAACTGAGAATTATGGAAAATATGGATTTTTAAATGTGCACTGGTTTT						
Cele.WB235	Cele.WB235_2:1033777	2	1033777	1034280	504	17	11
GTACTGTAGG	GTACTGTAGTA						
Cele.WB235	Cele.WB235_2:1034465	2	1034465	1036445	1981	125	15
TCGTGGTGAG	TCGTGGTGAGATCCA						
Cele.WB235	Cele.WB235_2:1037817	2	1037817	1039287	1471	95	15
TCGTGGTGAG	TCGTGGTGAGTCCCA						
Cele.WB235	Cele.WB235_2:1089504	2	1089504	1089883	380	5	26
GTCGGCTGCT	GTCGGCTGCTCACAAACCCTCGAGGT						
Cele.WB235	Cele.WB235_2:1134516	2	1134516	1134593	78	5	15
CGAAAAGGGG	CGAAAAGGGGATCTG						
Cele.WB235	Cele.WB235_2:1134783	2	1134783	1134875	93	6	15
TCCCCTTTTC	TCCCCTTTTCGTATC						
Cele.WB235	Cele.WB235_2:1178522	2	1178522	1178806	285	8	26
TACTGTAGTT	TACTGTAGTTTGACCTTCTAAACGAT						
Cele.WB235	Cele.WB235_2:1204743	2	1204743	1204963	221	4	55
GGGATTATTG	GGGATTATTGGAGTTTCTTTTTCTAGAATTTTTTGAAAATTTTGGACAGTTTTTC						

Cele.WB235 Cele.WB235_2:1224948 2 1224948 1225028 81 5 12
TTAGGCTTAG TTAGGCTTAGGC
Cele.WB235 Cele.WB235_2:1250454 2 1250454 1251360 907 73 12
CTATGCCTAT CTATGCCTATAC
Cele.WB235 Cele.WB235_2:1252963 2 1252963 1253498 536 44 12
CTATGCCTAT CTATGCCTATGC
Cele.WB235 Cele.WB235_2:1308143 2 1308143 1308573 431 7 63
GGTCTGCTAA GGTCTGCTAAATCGAAACTTGTAGTTTGTAGCCTAGCAGACCCAATTTCTTCAAAACTACTTG
Cele.WB235 Cele.WB235_2:1319234 2 1319234 1319605 372 5 73
AGTTTGTAGT ACTTTGTAGTTTGTAGTCTAGCAGACCAAAATTTATTAAAAACTGCAGAAAATTGATGGGTCTGCTAATTAAG
Cele.WB235 Cele.WB235_2:1404044 2 1404044 1405522 1479 12 63
TAGTTTGTAG TAGTTTGTAGTCTAGCAGACCATATTTCTTCAAAACCCCATAGGTCTGCTAAATCGAAACTTT
Cele.WB235 Cele.WB235_2:1408410 2 1408410 1408750 341 5 59
CTACAAACTA CTACAAACTACAAAAGCCTATTTAGCAGACCCACGCCAACTTCTGATGGGTCTGCTAGA
Cele.WB235 Cele.WB235_2:1414614 2 1414614 1415742 1129 9 63
TTGGTCTGCT TTGGTCTGCTAGACTACAAACTACAACTTTTGATTTAGCAGACCCATGGGGTTTTGAAGAAAA
Cele.WB235 Cele.WB235_2:1421457 2 1421457 1422023 567 41 12
CCTATGCCTA CCTATGCCTATG
Cele.WB235 Cele.WB235_2:1455578 2 1455578 1455634 57 4 14
TACGCTATAC TACGCTGTACGCTA
Cele.WB235 Cele.WB235_2:1467853 2 1467853 1467943 91 5 18
GGAGGTCCAG GGAGGTCCAGGAACCCCT
Cele.WB235 Cele.WB235_2:1487465 2 1487465 1487813 349 19 15
GGGGGCAACA GGGGACAACAAAAAA
Cele.WB235 Cele.WB235_2:1496871 2 1496871 1497776 906 6 150
TTGCAACATG
TTGCAACATGCGGTCGGCGGGAAATTCAAATTTTTAGATCAAAAACAGGTTTTTCGACCAATTTTTCATATTTTCTAGTGAAATTATGCTAGAAAAATCAATTTTTCACTCAAAATCTTCAGTTTTCGACATT
TTCTCATTGGAGCGCAC
Cele.WB235 Cele.WB235_2:1564655 2 1564655 1565350 696 7 96
GGCCTGCGGC GGCCTGCGGCCCTCAAACCTGGTTATTAGGCTTGAGTGAATAACATATCTGCTTAGAATAGGATCAAAGCCAGCCCATAGTTACCAAGAGTGTTCG
Cele.WB235 Cele.WB235_2:1578528 2 1578528 1579311 784 24 23
CGCTGTAAGA CGCTGTAAGACCATTTGCTGTAT
Cele.WB235 Cele.WB235_2:1596937 2 1596937 1597005 69 4 17
TCATTCCGTG TCATTCCGTGATTATTT
Cele.WB235 Cele.WB235_2:1605126 2 1605126 1605357 232 13 14
GATCCTAGAT GATCCTAGATCCTA
Cele.WB235 Cele.WB235_2:1630503 2 1630503 1630898 396 5 42
TTGCCGGTTG TTGCCGGTTGCCGAAATTTTTTGGAATCAGCAACTTCGGCAA
Cele.WB235 Cele.WB235_2:1635206 2 1635206 1635601 396 5 42
CAACCGGCAA CAACCGGCAATTGCCGAAGTTGCCGAACCCAAAACATTTCGG
Cele.WB235 Cele.WB235_2:1659657 2 1659657 1659775 119 7 17
GATGCCCCCT GATGCCCCCTCTGAACG
Cele.WB235 Cele.WB235_2:1793298 2 1793298 1793504 207 6 34
ATTTGAATTT ATTTGAATTTCCCGCCAAAATTTTTTCACTGAAA
Cele.WB235 Cele.WB235_2:1796656 2 1796656 1797032 377 11 34
CGGGAAATTC CGGGAAATTCAAATTTTCAGTGAAAAAATTTTGC
Cele.WB235 Cele.WB235_2:1858665 2 1858665 1858980 316 5 65
TATCGAGAGA TATCGAGAGAATTTCCAATGTTTCCAACAAAATCGATTTCCAGTTCCAATAAGACTCTCAGTAAG
Cele.WB235 Cele.WB235_2:1892810 2 1892810 1893486 677 26 26
ATTTACGGAG ATTTACGGAGGTTCGATCCTGTGTCG
Cele.WB235 Cele.WB235_2:1962052 2 1962052 1963013 962 31 31
AGAGCTGAGT AGAGCTGAGTTGTCTAGCGAGATAGCAGAGA

Cele.WB235 Cele.WB235_2:1968841 2 1968841 1969602 762 23 32
AATTCCCGCC AATTCCCGCCAAAAAATTGTAAAGAAAATTTA
Cele.WB235 Cele.WB235_2:1970474 2 1970474 1970784 311 10 31
AGAGCTGAGT AGAGATGAGTTGTCGAGTGAGACCTCAGAAG
Cele.WB235 Cele.WB235_2:1976612 2 1976612 1976897 286 8 32
AATTCCCGCC AATTCCCGCCAAAAAATTTTAAAGAAAATTTA
Cele.WB235 Cele.WB235_2:1977769 2 1977769 1979195 1427 46 31
AGAGCTGAGT AGAGATGAGTTGTCGAGCGAGACCTCAGAAG
Cele.WB235 Cele.WB235_2:1985022 2 1985022 1985847 826 25 32
AATTCCCGCC AATTCCCGCCAAAAAATTGTAAAGAAAATTTA
Cele.WB235 Cele.WB235_2:2000885 2 2000885 2001197 313 26 12
ATGTATATGT ATGTATATGTAT
Cele.WB235 Cele.WB235_2:2002012 2 2002012 2002087 76 5 15
TCGTGGCGAG TCGTTGCGAGACCCT
Cele.WB235 Cele.WB235_2:2002110 2 2002110 2002460 351 10 32
GGCGGGAATT GGCGGGAATTTAAATTTTCTTTGAAATTTTTC
Cele.WB235 Cele.WB235_2:2008283 2 2008283 2009461 1179 38 31
GACAACTCAG GACAACTCAGCTCTCTTCTGAGGTCTCACTC
Cele.WB235 Cele.WB235_2:2050346 2 2050346 2051160 815 5 162
AGTTTTATGA
AGTTTTATGAAATAAAAAAGAATGACTCTATATGATTTTCCCATACTTTTGAATTTTTGAAAAAATCAGCTTTTATATATTTTTATCCATTTTTAAAAAATACTTCCGTACAAACTATTATACTTTGTTTAGC
ATTTTCTGGTATTTTTGACTATGCTGAAG
Cele.WB235 Cele.WB235_2:2110299 2 2110299 2110412 114 5 22
TGGTCAGTGC TGGTCAGTGCAAACAATTTTAT
Cele.WB235 Cele.WB235_2:2151844 2 2151844 2151904 61 4 11
ACCGCAACGC ACCGCAACGCA
Cele.WB235 Cele.WB235_2:2157814 2 2157814 2157944 131 5 26
TCAAGGCGAA TCAAGGCGAAACGGGAGGTGAATCGC
Cele.WB235 Cele.WB235_2:2171107 2 2171107 2172182 1076 24 43
CGAGTTTGGC CGAGTTTGGCCTACAGCAAATTTGGCAAAATCGGCAAATTTGC
Cele.WB235 Cele.WB235_2:2273842 2 2273842 2274055 214 8 21
TGTGCCCTGG TGTGCCCTGGAAAGCTGGTGG
Cele.WB235 Cele.WB235_2:2279378 2 2279378 2279438 61 4 10
CAGCAGAGTG CAGCAGAGTG
Cele.WB235 Cele.WB235_2:2348715 2 2348715 2349106 392 13 11
CTACAGTACC CTACAGTACTC
Cele.WB235 Cele.WB235_2:2361743 2 2361743 2363537 1795 9 163
TGAATTTTTG
TGAATTTTTGAATATATCAGCTTTTGGTTAAATTCTGCATTTTTTAAAAATAAACTTCCGTACGAGCTAGCACCTTTTTTTAAGTATTTTCTGGTACATTTGACTATGCTGAACAGTTTTATAAAATAAAAAA
GAATGACTTCATATAATTTTGAGGTACTTT
Cele.WB235 Cele.WB235_2:2386288 2 2386288 2386363 76 5 15
TCGTGGCGAG TCGTTGCGAGACCCT
Cele.WB235 Cele.WB235_2:2529567 2 2529567 2530441 875 24 35
CCGGAAATTT CCGGAAATTTTCATTTTCGGCAATTTGCCGATTTG
Cele.WB235 Cele.WB235_2:2550207 2 2550207 2550279 73 6 12
TTAGGCTTAG TTAGGCTTAGGA
Cele.WB235 Cele.WB235_2:2577037 2 2577037 2578087 1051 24 31
CTGAAATTCC CTGAAATTCCAATTTTCCAGTGTAAAAATGT
Cele.WB235 Cele.WB235_2:2593754 2 2593754 2594002 249 8 31
AGAGCTGAGT AGAGCTGAGTTGTCTAGCGAGATAGCAGAGA
Cele.WB235 Cele.WB235_2:2599830 2 2599830 2600053 224 7 32
AATTCCCGCC AATTCCCGCCAAAATTTTTCACAGAAAATTTG

Cele.WB235	Cele.WB235_2:2599867	2	2599867	2600422	556	7	32	CCGCCAAAAA	CCGCCAAAAAATTTTAAAGAAAATTTAAATTC
Cele.WB235	Cele.WB235_2:2623068	2	2623068	2623182	115	6	19	AAAATCGATA	AAAATCGATAATTTTGAGA
Cele.WB235	Cele.WB235_2:2629919	2	2629919	2630440	522	10	14	CTACAAACTA	CTACAACCTATAAA
Cele.WB235	Cele.WB235_2:2632149	2	2632149	2632578	430	33	13	AGCCTAAGCT	AGCCTAAGCTCTA
Cele.WB235	Cele.WB235_2:2633519	2	2633519	2633585	67	5	12	CCTAAGCCTA	CTTAAGCCTATG
Cele.WB235	Cele.WB235_2:2712830	2	2712830	2713528	699	7	95	TTCGGGCCTG	TTCGGGCCTGCGGCCCTCAAACCTGGTTTGTAGGCTTGAGCGAATAACAATCTGCTTGGAATAGGATTAAAGTTGGCCCACAGTTTCCAAGAGTG
Cele.WB235	Cele.WB235_2:2731022	2	2731022	2731082	61	4	12	GCCTAAGCCT	GCCTAAGCCTAA
Cele.WB235	Cele.WB235_2:2733211	2	2733211	2733873	663	24	22	ACTACAGTAA	ACTACAGTAATTCTACGTCGCT
Cele.WB235	Cele.WB235_2:2761987	2	2761987	2762171	185	11	16	CACGTAGGCA	CACGTAGGCAAGTAGG
Cele.WB235	Cele.WB235_2:2811262	2	2811262	2812302	1041	6	144	TGGCCTAGAA	TGGCCTAGAAATCCAAGTTTGCAAAAGTTAGTCCACCAACTTCAAATGCCTATAACTTTGCGGAAAATCAAGATTTTTCAGTGATTTTAACGTAACTATGATGCTACTGGCTTTGCGCATCTTTTTGCCCTACTTAACGTTTGG
Cele.WB235	Cele.WB235_2:2817169	2	2817169	2817253	85	6	12	CCTAAGCCTA	CCTAAGCCTAGT
Cele.WB235	Cele.WB235_2:2897150	2	2897150	2899742	2593	100	21	CCAAAAATTG	CCAAAAATTGTGATTTTTAGT
Cele.WB235	Cele.WB235_2:2949876	2	2949876	2951271	1396	9	155	TCCCGCTGTG	TCCCGCTGTGGTCAAAAGTTTTTTGTGTGCTCAGTTCTGTACGACAAATGTGTTAAAATCTTCTGAAAACTCAAAATTCCAAGTTTTCCCAAAAATGGGTCAAATGGTACAGTGGGATTTTCCACGAATTCTAAGTAGATGGTCGTGAGTTCAAC
Cele.WB235	Cele.WB235_2:2957411	2	2957411	2957495	85	7	12	ATAGGCATAG	ATAGGCATAGGC
Cele.WB235	Cele.WB235_2:2966318	2	2966318	2968108	1791	54	14	CTACCTGCCT	CTACCTGCCTATGC
Cele.WB235	Cele.WB235_2:2980111	2	2980111	2980251	141	5	28	AATGTCGGCT	AATGTCGGCTGCTTCTAGAAGCCCGGCA
Cele.WB235	Cele.WB235_2:3033725	2	3033725	3034005	281	20	14	TTAGGGCCTT	TTAGGGCCTTGGGT
Cele.WB235	Cele.WB235_2:3036430	2	3036430	3037140	711	18	19	GCCAAGGCAC	GCCAAGGCACTTGATAAGG
Cele.WB235	Cele.WB235_2:3048248	2	3048248	3048472	225	15	14	CTTATACCTT	CTTATACCTTATAC
Cele.WB235	Cele.WB235_2:3070377	2	3070377	3070511	135	4	31	ATCGATTTTT	ATCGATTTTTTCGGTAAAAAATCAATAAAAT
Cele.WB235	Cele.WB235_2:3102561	2	3102561	3102723	163	6	27	GCTGCTGGAG	GCTGCTGGAGGATCCACTGCATCAACT
Cele.WB235	Cele.WB235_2:3106228	2	3106228	3107119	892	33	27	GCTGCTGGAG	GCTGCTGGAGGATCCACTGCATCAACT
Cele.WB235	Cele.WB235_2:3111494	2	3111494	3112267	774	16	43	TTGCCGGAAT	TTGCCGGAGTTGAAAATTTCCTGTAAATCGGCAAACCGGCAAC
Cele.WB235	Cele.WB235_2:3124197	2	3124197	3124357	161	9	16	TAGGCACGTA	TAGGCACGTAAGCATG

Cele.WB235	Cele.WB235_2:3125607	2	3125607	3125959	353	18	16
TACGTGCCTA	TACGTGCCTACGTGCC						
Cele.WB235	Cele.WB235_2:3136277	2	3136277	3136467	191	8	12
GGCTTAGGCT	GGCTTAGGCTTA						
Cele.WB235	Cele.WB235_2:3145088	2	3145088	3145138	51	4	10
AACTTTCGAA	AACTTTCGAA						
Cele.WB235	Cele.WB235_2:3152725	2	3152725	3152861	137	8	16
AGGCACGTAG	AGGCACGTAGGCATAT						
Cele.WB235	Cele.WB235_2:3153871	2	3153871	3153936	66	4	14
CCAAGGCCCA	CCAAAGCCCAAGGC						
Cele.WB235	Cele.WB235_2:3179723	2	3179723	3180290	568	21	16
GTAGGCACGT	GTAGGCACGTAGGCAC						
Cele.WB235	Cele.WB235_2:3246632	2	3246632	3247175	544	19	20
GTAAATCTAC	GTAAATCTACACTTGCTTCC						
Cele.WB235	Cele.WB235_2:3271556	2	3271556	3272244	689	42	16
CGTAGGCACG	CGTAGGCACGTGGGCG						
Cele.WB235	Cele.WB235_2:3290648	2	3290648	3290783	136	5	27
TTTACTCTCT	TTTACTCTCTGTGGCTTCCCACTATAT						
Cele.WB235	Cele.WB235_2:3301138	2	3301138	3301263	126	4	22
ATTCTGCGCA	ATTCTGCGCACCCCCTGCGCCG						
Cele.WB235	Cele.WB235_2:3365918	2	3365918	3365978	61	4	15
GGTGAGACCC	GGTGAGACCCATCGT						
Cele.WB235	Cele.WB235_2:3415307	2	3415307	3415447	141	4	28
TGCCTCGCCT	TGCCTCGCCTACGTGCCTATTTCAGGCA						
Cele.WB235	Cele.WB235_2:3420081	2	3420081	3420246	166	5	16
GCACGTAGGC	GCACGTAAGCACGTAG						
Cele.WB235	Cele.WB235_2:3459359	2	3459359	3459467	109	5	18
GGCTTAGGCT	GGCTTAGGCTTAGGCTCA						
Cele.WB235	Cele.WB235_2:3471040	2	3471040	3471110	71	4	12
ATACATATAC	ATACATATACAT						
Cele.WB235	Cele.WB235_2:3490327	2	3490327	3491001	675	54	12
TAGGTATAGG	TAGGTAAAGGCA						
Cele.WB235	Cele.WB235_2:3494517	2	3494517	3494748	232	5	39
AATTTCCCGC	AATTTCCCGCCAAAAATTTTACGTTTTTCAGAAAATTCA						
Cele.WB235	Cele.WB235_2:3496033	2	3496033	3496764	732	18	31
GGTTACTGTA	GGTTACTGTAACTCAAGCCTGAGATTCTTCG						
Cele.WB235	Cele.WB235_2:3512519	2	3512519	3512671	153	9	16
GTAGGCACGT	GTAGGTACGTAGGCAC						
Cele.WB235	Cele.WB235_2:3572146	2	3572146	3572433	288	6	42
TTTGAAAACC	TTTGAAAACCTGCAATATCCGGAATTGCCGATTGCCGGAAAT						
Cele.WB235	Cele.WB235_2:3641762	2	3641762	3641852	91	5	20
CTATCGCCTG	CTATCGCCTGCTAGCGCCTA						
Cele.WB235	Cele.WB235_2:3691112	2	3691112	3691360	249	8	31
GCTGATTGGT	GCTGATTGGTCGGCATGGTCTGCTCATCATC						
Cele.WB235	Cele.WB235_2:3753993	2	3753993	3754089	97	4	24
TGCATCTGCC	TGCATCTGCCACAGGAGCTGGCGT						
Cele.WB235	Cele.WB235_2:3937888	2	3937888	3938177	290	6	45
AAGGATCAGA	AAGGATCAGAAGGGTACCTCCTGATGATCTGATCCTTCAGCTTCA						
Cele.WB235	Cele.WB235_2:3941820	2	3941820	3946547	4728	58	79
GTGAATCTCC	GTGAATCTCCTCTCTACCTAATGAGTACTGACAGAATACCGGCAGTATTCTGGGGTCTCATTAGGTTTTAGTTTCTTCT						
Cele.WB235	Cele.WB235_2:3994649	2	3994649	3994788	140	7	20
AAATAGATTG	AAATAGATTGTTACACGAAA						

Cele.WB235	Cele.WB235_2:4030891	2	4030891	4031711	821	4	16
TGCCTACGTG	TGCCTACGTGCCTACG						
Cele.WB235	Cele.WB235_2:4054458	2	4054458	4054819	362	4	21
CTACGTAAGA	CTACGTAAGAGTAGCGCAGGA						
Cele.WB235	Cele.WB235_2:4081925	2	4081925	4082141	217	14	12
CTTAGGCTTA	CTTAGGTTTAGA						
Cele.WB235	Cele.WB235_2:4094407	2	4094407	4094663	257	13	11
GTACTGTAGG	GTACTGTAGGA						
Cele.WB235	Cele.WB235_2:4138713	2	4138713	4139388	676	4	11
TGCCTGCCTA	TGCCTGCCTAC						
Cele.WB235	Cele.WB235_2:4140823	2	4140823	4140867	45	4	11
GGCAGGCAGT	GGCAGGCAGTA						
Cele.WB235	Cele.WB235_2:4205778	2	4205778	4205946	169	5	24
GTGAGGTACG	GTGAGGTACGAGATGAGGTACAAG						
Cele.WB235	Cele.WB235_2:4207582	2	4207582	4207842	261	25	10
GCGTTAGCAG	GCGTTAGCAG						
Cele.WB235	Cele.WB235_2:4210804	2	4210804	4210907	104	5	18
TGCAAGACTA	TGCAAGACTAATAGAGAC						
Cele.WB235	Cele.WB235_2:4259952	2	4259952	4260552	601	24	25
GAGAGTAAAA	GAGAGTAAAATATACGGTGAAGCCA						
Cele.WB235	Cele.WB235_2:4260577	2	4260577	4261267	691	40	15
GTCTCACCAC	GTCTCACCACGAAGG						
Cele.WB235	Cele.WB235_2:4296664	2	4296664	4296988	325	8	35
TTCCGGCAAA	TTCCGGCAAAACGGCAAATTGTCAAAATTGAAAAT						
Cele.WB235	Cele.WB235_2:4309186	2	4309186	4309439	254	23	11
CAGTTGTCAC	CAGTTATCACC						
Cele.WB235	Cele.WB235_2:4312357	2	4312357	4312429	73	4	12
AGCCTAAGCC	AGCCTTAGCCTA						
Cele.WB235	Cele.WB235_2:4365202	2	4365202	4365284	83	4	12
TAAGCCTAAG	TAAGCCTAAGAC						
Cele.WB235	Cele.WB235_2:4442528	2	4442528	4442746	219	20	11
TGCACATTTT	TGCACATTTTT						
Cele.WB235	Cele.WB235_2:4554394	2	4554394	4554914	521	13	40
TCCAGAACCT	TCCAGAACCTTCTGGAAAATTCGAGAAAATTCTGGAATGT						
Cele.WB235	Cele.WB235_2:4555047	2	4555047	4555687	641	20	32
CGGGAATTCA	CGGGAATTCAAAATTTCTGTGAAAAATTTTGG						
Cele.WB235	Cele.WB235_2:4569011	2	4569011	4569221	211	5	35
TTTGCCGGAA	TTTGCCGGAATTTTCAATTTTCGGAAAATTGCCGG						
Cele.WB235	Cele.WB235_2:4684299	2	4684299	4684389	91	6	15
GAGACCCATC	GAGACCCATCGTGGC						
Cele.WB235	Cele.WB235_2:4684295	2	4684295	4684838	544	21	15
TGGTGAGACC	TGGTGAGACCCATCG						
Cele.WB235	Cele.WB235_2:5073308	2	5073308	5077334	4027	52	76
TGTATTTTGA	TGTATTTTGAAAGGTCATCTCAATAGAAAAGATGGAAGACTACAGAAAAACTATTGTGAGACCCCGAAATACTGGC						
Cele.WB235	Cele.WB235_2:5097197	2	5097197	5097899	703	27	26
TTAGACCATT	TTAGACCATTAGATGAATTCCCAAAG						
Cele.WB235	Cele.WB235_2:5144313	2	5144313	5144565	253	9	25
AGAGTAAAAT	AGAGTAAAATAATTGGTGAAGCCAG						
Cele.WB235	Cele.WB235_2:5144410	2	5144410	5146766	2357	80	25
AGCCAGAGAG	AGCCACAGAGTAAAATAATTGGGAA						
Cele.WB235	Cele.WB235_2:5627848	2	5627848	5628154	307	9	34
GGAATTGAAA	GGAATTGAAATTTCCAGCAAATCGGCAAATTGCC						

Cele.WB235	Cele.WB235_2:6073626	2	6073626	6077427	3802	21	181
TTTTTAACTT	TTTTTAACTTTTAAATCCACTAAAACTTGGAATGGTACAGAACATGCAAATTGTAATTTTAAGAACTGAATGTGGTTTCGAATATTCATGGATTGCTTTATTCGAACACTGACTCTGAAATATTTTGCATCCATTTGACTATTAATTGTCATATTATACCGAACACTACTAACTTTACTGG						
Cele.WB235	Cele.WB235_2:6082023	2	6082023	6082291	269	6	45
CTGAAGGATC	CTGAAGGATCAGACCATCAGGAGGTACCCTTACGATCCTTCTTAT						
Cele.WB235	Cele.WB235_2:6185280	2	6185280	6186470	1191	85	14
ACGCTATACG	ACGCTATACGCTAT						
Cele.WB235	Cele.WB235_2:6296590	2	6296590	6296638	49	4	12
CCACTGACCA	CCACTGACCATC						
Cele.WB235	Cele.WB235_2:6296691	2	6296691	6297095	405	6	68
AATAGAGACG	AATAGAGACGAATAAAATTTTAAAAATTTTATTGGTGTCTCTATTTTATCAAGCAAATATATTTTCAA						
Cele.WB235	Cele.WB235_2:6434384	2	6434384	6434544	161	5	32
AAAAATTTTC	AAAAATTTTCACAAAAAATTTAAATTCCTGCA						
Cele.WB235	Cele.WB235_2:6434384	2	6434384	6435137	754	6	32
AAAAATTTTC	AAAAATTTTCACAAAAAATTTAAATTCCTGCA						
Cele.WB235	Cele.WB235_2:7379726	2	7379726	7379898	173	4	41
AAAACGGGAA	AAAACGGGAAAACGGGAATTCCCGTTCCCGTGAAAATTTTA						
Cele.WB235	Cele.WB235_2:7379725	2	7379725	7380258	534	10	41
AAAAACGGGA	AAAAACGGGAAAACGGGAATTCCCGTTCCCGTGAAAATTTT						
Cele.WB235	Cele.WB235_2:7446830	2	7446830	7448192	1363	34	14
GAAGGATCGG	GAAGGATCGGGTCG						
Cele.WB235	Cele.WB235_2:7449335	2	7449335	7450152	818	42	16
CGGAAAAATT	CGGAAAAATTTTGCTG						
Cele.WB235	Cele.WB235_2:7450879	2	7450879	7451382	504	27	10
TCCATCCCGA	TCCATCCCAC						
Cele.WB235	Cele.WB235_2:7475407	2	7475407	7475467	61	4	15
GTAGATCTTG	GTAGATCTTGAAAAT						
Cele.WB235	Cele.WB235_2:7894727	2	7894727	7894819	93	6	15
CGAAAAGGGG	CGAAAAGGGGATCTG						
Cele.WB235	Cele.WB235_2:7894995	2	7894995	7895087	93	6	15
CCCCTTTTCG	CCCCTTTTCGTATCT						
Cele.WB235	Cele.WB235_2:8287652	2	8287652	8292933	5282	61	79
TGAGTCCTGA	TGAGTCCTGACAGAATACCGGCAGTATTCTGGGGTCTCATTAGGTTGTCGTTTCTTTTCTGAATCTCCACTCTTCCGAA						
Cele.WB235	Cele.WB235_2:8460034	2	8460034	8460434	401	10	40
AAAGTTCTGG	AAAGTTCTGGAACATTACAGAAATTTCTCGATTTTTCTAG						
Cele.WB235	Cele.WB235_2:8460726	2	8460726	8460801	76	4	15
GGGTCTCACC	GGGTCTCACCACGAC						
Cele.WB235	Cele.WB235_2:8975477	2	8975477	8977487	2011	24	86
AGCCAGTATT	AGCCAGTATTTCGGGGTCTGGCAATAGCTTCTCTTTAACTTCGAACTCTTCCATCTTTTCCCTATTGCCACGACCTTTCAAAATAC						
Cele.WB235	Cele.WB235_2:9202102	2	9202102	9202507	406	27	15
TGGTGAGACC	TGGTGAGACCCATCG						
Cele.WB235	Cele.WB235_2:9202534	2	9202534	9203060	527	15	35
ATTCAAATTT	ATTCAAATTTTTAGTGAAAAAAATTTTGGAGGGAA						
Cele.WB235	Cele.WB235_2:9202525	2	9202525	9203501	977	16	35
TGGCGGGAAA	TGGCGGGAAATTCAAATTTTTAGTGAAAAAAATTT						
Cele.WB235	Cele.WB235_2:9404845	2	9404845	9405392	548	22	25
GGCTTCACAG	GGCTTCACAGAATAATTTACTCTCC						
Cele.WB235	Cele.WB235_2:9631891	2	9631891	9633131	1241	31	40
TTCTGGAACA	TTCTGGAACATTCCAGAATTTTCTCGATTTTTCTAGAAAG						
Cele.WB235	Cele.WB235_2:9633206	2	9633206	9633906	701	20	35
CCAAAATATT	CCAAAATATTTTTCTCAGAAAATTAAAATTTCCCT						

Cele.WB235	Cele.WB235_2:9633989	2	9633989	9634259	271	17	15
CCACGATGGG	CCACGATGGGTCTCA						
Cele.WB235	Cele.WB235_2:9634505	2	9634505	9634639	135	5	11
GTACTCCTAC	GTACTCCTACA						
Cele.WB235	Cele.WB235_2:9693772	2	9693772	9694139	368	4	42
CAGCCGACAG	CAGCCGACAGCCGAAAAATTGCGATTTTTCGGCTGGTAGCAC						
Cele.WB235	Cele.WB235_2:9719802	2	9719802	9720144	343	16	19
ATAAATATTA	ATAAATATTAATTAAATAA						
Cele.WB235	Cele.WB235_2:10248425	2	10248425	10248517	93	6	15
TCCCCTTTTC	TCCCCTTTTCGTTGC						
Cele.WB235	Cele.WB235_2:10336035	2	10336035	10336399	365	14	26
TATGACTCTA	TATGACTCTAACCATTCACCTTAAAA						
Cele.WB235	Cele.WB235_2:10466292	2	10466292	10466385	94	5	18
TAAATAAGTG	TAAATAAGTGGCTATAAG						
Cele.WB235	Cele.WB235_2:10713445	2	10713445	10714142	698	8	35
TTTCTGGCAA	TTTCTGGCAAATCGGCAGTTTGCCGAAAATTAAAT						
Cele.WB235	Cele.WB235_2:10832628	2	10832628	10833651	1024	24	11
GTACTGTAGG	GTACTGTAGGA						
Cele.WB235	Cele.WB235_2:11122559	2	11122559	11122763	205	16	12
AATAAAAATA	AATAAAAATAAA						
Cele.WB235	Cele.WB235_2:11230254	2	11230254	11230477	224	5	44
AAGGATCAGA	AAGGATCAGATCATCAGGCAGAGCCTCATGCTCTTTTGGAGCTG						
Cele.WB235	Cele.WB235_2:11306986	2	11306986	11307037	52	4	12
ACTATAACTA	ACTACAACTACA						
Cele.WB235	Cele.WB235_2:11365554	2	11365554	11365650	97	5	24
GCTTAGGCTT	GCTTAGGCTTAGGCTTGGGCTCAA						
Cele.WB235	Cele.WB235_2:11423462	2	11423462	11423661	200	9	20
AAAAAATAGA	AAAAAATAGATTGGCATACG						
Cele.WB235	Cele.WB235_2:11527871	2	11527871	11528452	582	13	45
TCTGATCCTT	TCTGATCCTTCAGCTCCGAAGGATCACATGGGTACCTTCTGATGT						
Cele.WB235	Cele.WB235_2:11530067	2	11530067	11530424	358	8	45
GAAGGATCAG	GAAGGATCAGATCATCAGAAGGTACCCATGTGATCCTTCGGATCG						
Cele.WB235	Cele.WB235_2:11534628	2	11534628	11534720	93	6	15
CGAAAAGGGG	CGAAAAGGGGATCTG						
Cele.WB235	Cele.WB235_2:11534895	2	11534895	11534970	76	5	15
TCCCCTTTTC	TCCCCTTTTCGTATC						
Cele.WB235	Cele.WB235_2:11660023	2	11660023	11660287	265	8	33
AATTGAAAAT	AATTGAAAATCCAGCAAATGGGCAAATTGCCGT						
Cele.WB235	Cele.WB235_2:11733044	2	11733044	11733122	79	6	12
CTTAGGCTTA	CTTAGGCTTAGG						
Cele.WB235	Cele.WB235_2:11905368	2	11905368	11905475	108	8	12
AGGCTTAGGC	AGGCTTAAGCTT						
Cele.WB235	Cele.WB235_2:11906637	2	11906637	11907671	1035	44	20
TAAATCTACA	TAAATCTACACAGGGTGGTG						
Cele.WB235	Cele.WB235_2:11930277	2	11930277	11930409	133	10	12
AAGCCTAAGC	AAGCCTAACCAT						
Cele.WB235	Cele.WB235_2:11932324	2	11932324	11932498	175	10	12
AGGCTTAGGC	AGGCTTAGACTT						
Cele.WB235	Cele.WB235_2:12002438	2	12002438	12002513	76	4	15
AAAACAAGAT	AAAAGAAGATCTACA						
Cele.WB235	Cele.WB235_2:12171287	2	12171287	12172252	966	32	30
CAAACTACAG	CAAACTACAGTAGCACTGTAGTACCATAAC						

Cele.WB235	Cele.WB235_2:12239992	2	12239992	12240106	115	7	12
TTAGTCTTAG	TTAGTCTTAGGC						
Cele.WB235	Cele.WB235_2:12247672	2	12247672	12247768	97	7	12
CCTAAGCCTA	CCTAACCCTAAG						
Cele.WB235	Cele.WB235_2:12322418	2	12322418	12322496	79	5	12
TTAGGCTTAG	TTAGGCTCAGGC						
Cele.WB235	Cele.WB235_2:12336337	2	12336337	12336616	280	4	69
AGGTAGGCCT	AGGTAGGCCTGTAGGCATTGACTGGCAGAAAAGTGTAGGCAGGCAGGCAGGTAAAAAGATACATGCGGC						
Cele.WB235	Cele.WB235_2:12350820	2	12350820	12350876	57	5	11
CTACAGTACC	CTACAGTACCT						
Cele.WB235	Cele.WB235_2:12353539	2	12353539	12353707	169	14	12
CCTATACCTA	CCTATACCTATG						
Cele.WB235	Cele.WB235_2:12381010	2	12381010	12381194	185	13	12
AGCCTAAGCC	AGCCTAAGCCTA						
Cele.WB235	Cele.WB235_2:12402768	2	12402768	12404058	1291	30	43
TTTCCGGCAA	TTTCCGGCAAACCGGCAAACCGGTGATTTGCCGAAAATGCGAA						
Cele.WB235	Cele.WB235_2:12455807	2	12455807	12455995	189	6	31
GACATCTCGC	GACATCTCGCAGGACAATATAAAAAGCGACC						
Cele.WB235	Cele.WB235_2:12467214	2	12467214	12467281	68	5	12
GGCTTAGGCT	GGCTTAGGCTTA						
Cele.WB235	Cele.WB235_2:12575393	2	12575393	12575478	86	5	17
ACTTGGTACA	ACTTGGTACATAGACCA						
Cele.WB235	Cele.WB235_2:12577021	2	12577021	12577399	379	8	42
TTGCCGAACC	TTGCCGAACCGTAAAAATTTCGGCAAACGGGAATTGCCAAAG						
Cele.WB235	Cele.WB235_2:12649041	2	12649041	12649452	412	11	37
ATTTTGGCGG	ATTTTGGCGGGAATTCAAATTTTAATTTTTTTAAAAT						
Cele.WB235	Cele.WB235_2:12649559	2	12649559	12650818	1260	83	15
TCGTGGTGAG	TCGTGGTGAGACCCA						
Cele.WB235	Cele.WB235_2:12649559	2	12649559	12651613	2055	136	15
TCGTGGTGAG	TCGTGGTGAGACCCA						
Cele.WB235	Cele.WB235_2:12701130	2	12701130	12701355	226	7	11
CTACAGTACC	CTACAGTACTC						
Cele.WB235	Cele.WB235_2:12719695	2	12719695	12719880	186	5	43
TATTTTCATT	TATTTTCATTTTCGGCAAATTGCCGGTTTGCCGATTTGCCGGG						
Cele.WB235	Cele.WB235_2:12749754	2	12749754	12749935	182	4	45
ATCTGATCCT	ATCTGATCCTTCAGCTCCGAAGGATCAGAAGGGTACCTCCTGGTG						
Cele.WB235	Cele.WB235_2:12757809	2	12757809	12758089	281	8	35
ATTTCAGGCA	ATTTCAGGCAATTTACCGATTTGCCAGAAATGTTC						
Cele.WB235	Cele.WB235_2:12757828	2	12757828	12758945	1118	16	35
TTTGCCGGAA	TTTGCCAGAAATGTTCATTTCCGGCAATTTGTCGA						
Cele.WB235	Cele.WB235_2:12761888	2	12761888	12762610	723	9	14
AACTACAAAC	AACTATAAACTACA						
Cele.WB235	Cele.WB235_2:12763248	2	12763248	12763368	121	7	15
ATTTCCGGAT	ATTTCCGGATCTATG						
Cele.WB235	Cele.WB235_2:12770724	2	12770724	12771186	463	10	35
TCAATTCCGG	TCAATTCCGGCAATTTTTGGATTTGCCGGAAATTT						
Cele.WB235	Cele.WB235_2:12795532	2	12795532	12795808	277	8	35
TGTGTAATAA	TGTGTAATAATAAAATATCACTTTTTTTTCAGGAT						
Cele.WB235	Cele.WB235_2:12843822	2	12843822	12844875	1054	39	27
GTGGGAAGCC	GTGGGAAGCCACAGAGAGTAAAATATA						
Cele.WB235	Cele.WB235_2:12844903	2	12844903	12846136	1234	49	25
GGCTTCCCAC	GGCTTCCCACTATATTTTACTCTCT						

Cele.WB235 Cele.WB235_2:12889260 2 12889260 12889656 397 21 18
ACTACTGTAT ACTACTGTATCTCAGGGG
Cele.WB235 Cele.WB235_2:12905369 2 12905369 12905758 390 5 43
GATTTGCCGG GATTTGCCGGAAATTTTCAATCCCGGCAAATTGCAGGTTCGCC
Cele.WB235 Cele.WB235_2:12942997 2 12942997 12943139 143 5 18
CCTAAGCCTA CCTGAGCCTAAGCTCAAC
Cele.WB235 Cele.WB235_2:12951422 2 12951422 12951594 173 4 43
CGGCAAATTA CGGCAAATTACCAATTTGCCGATTTGCCGGAAATTTTCATTTT
Cele.WB235 Cele.WB235_2:12978404 2 12978404 12978548 145 9 16
GCCTACATGC GCCTACATGCCTACAT
Cele.WB235 Cele.WB235_2:12979640 2 12979640 12979790 151 4 30
TTCAGGCAGG TTCAGGCAGGCATATGTCACATCGAATGTA
Cele.WB235 Cele.WB235_2:13007141 2 13007141 13007285 145 6 19
TATGGTGCAT TATGGTGCATCTGACAAGT
Cele.WB235 Cele.WB235_2:13037761 2 13037761 13037897 137 4 34
TTCAATTCCG TTCAATTCCGGCAATTTCCCGATTTGCCGGAAAT
Cele.WB235 Cele.WB235_2:13061749 2 13061749 13061893 145 8 16
TAGGCACGTA TAGGTACGTAGGCACA
Cele.WB235 Cele.WB235_2:13184130 2 13184130 13184277 148 7 21
AATCTAGCCA AATCTAGCCAGACGCACACCT
Cele.WB235 Cele.WB235_2:13243398 2 13243398 13244452 1055 6 165
AAAATTCAAT
AAAATTCGATTTTGGACTTATCTATTCTCTCTTTTTTATAACATGTATTTCAGTTGATAGAGGACTCAAAATTATAATGCTCGTAATTATTTATAAGTGTGTGTTGTCTCAGAATCAATTCTAAAAAACAGAT
TTTTTTTGAAAGTTTAAGTAATATCCGCGGAG
Cele.WB235 Cele.WB235_2:13281689 2 13281689 13282092 404 10 31
CACGCCTATT CACGCCTATTTGTGGGAGGAGTCTACAAGC
Cele.WB235 Cele.WB235_2:13284530 2 13284530 13285104 575 12 48
TGCCTACGTG TGCCTACGTGCCTACATAAGAAGCGTAGGTAGCTTCAAAGGTGTTGCG
Cele.WB235 Cele.WB235_2:13303475 2 13303475 13303853 379 13 14
TAGTTTGTAG TAGTTTGTAGTTTG
Cele.WB235 Cele.WB235_2:13304324 2 13304324 13304580 257 15 16
GGCATGTAGG GGCATGTAGGCAGGTA
Cele.WB235 Cele.WB235_2:13310035 2 13310035 13310356 322 20 16
GGCATGTAGG GGCATGTAGGTAGGTA
Cele.WB235 Cele.WB235_2:13318641 2 13318641 13320189 1549 49 20
TTTGAATTTT TTTGAATTTTTAAGCCAAAA
Cele.WB235 Cele.WB235_2:13334193 2 13334193 13334831 639 5 122
GATCTACAAA
GATCTACAAAAACGCGGGAAAAGTGACGCAGAGTTTTGAACTGATTTTGCATAAAAATTCCCGCATTTTTTGTAGATCAAACCGTTATGGGACAGCCTGGTGCCACGTGCTTGATCTACGCA
Cele.WB235 Cele.WB235_2:13342774 2 13342774 13342879 106 5 21
CTAGAAAAAC CTAGAAAAACTCGGCCATCAA
Cele.WB235 Cele.WB235_2:13343764 2 13343764 13344901 1138 46 21
TTTTCTAGTT TTTCCTAGTTGATAGCCGAGT
Cele.WB235 Cele.WB235_2:13353644 2 13353644 13353730 87 6 12
CTTAGGCTTA CTTAGGCTTAGT
Cele.WB235 Cele.WB235_2:13372106 2 13372106 13372150 45 4 11
TGGAAAATTG TGGAAAATTGA
Cele.WB235 Cele.WB235_2:13385641 2 13385641 13386400 760 8 95
GGCCTGCGGC GGCCTGCGGCCCTCAAACCTGGTTGGTAGGCTTGAGCGAATAACAATCTGCTTGGAATAGGATTAAAGCTGGCCCTTATTTTTCATGAGTATTCA
Cele.WB235 Cele.WB235_2:13394857 2 13394857 13395660 804 20 35
GATTTGCCGG GATTTGCCGGAAATGTTCAATTCCGGCAATTTACT

Cele.WB235	Cele.WB235_2:13415355	2	13415355	13415629	275	20	11
CCTACAGTAC	CCTATAGTACC						
Cele.WB235	Cele.WB235_2:13422904	2	13422904	13423919	1016	29	35
GAATTTTTGA	GAGTTTTTGATTTTCGGCAATTTGCCAATTTGCCA						
Cele.WB235	Cele.WB235_2:13502158	2	13502158	13503434	1277	26	27
AGCAGCCGAC	AGCAGCCGACGCCTTGCGGGTCGCTCA						
Cele.WB235	Cele.WB235_2:13507307	2	13507307	13507403	97	6	16
ACATGCCTAC	ACATGCCTACATGCCT						
Cele.WB235	Cele.WB235_2:13522160	2	13522160	13522220	61	5	12
AATACCAACC	AATACCAACCAC						
Cele.WB235	Cele.WB235_2:13528439	2	13528439	13528985	547	24	20
GTAAATCGAC	GTAAATCGACAAAAGACATC						
Cele.WB235	Cele.WB235_2:13598247	2	13598247	13598526	280	7	35
TTGCCGGAAT	TTGCCGGAATTGAATATTTGCGGCAAATCTGTAAT						
Cele.WB235	Cele.WB235_2:13598710	2	13598710	13599880	1171	26	45
ACCAGGAGGT	ACCAGGAGGTACCCTTCTTATCCTTCGGAGCTGAATGATCAGATT						
Cele.WB235	Cele.WB235_2:13631132	2	13631132	13631348	217	13	16
ACATGCCTAC	ACATGCCTACTTACCT						
Cele.WB235	Cele.WB235_2:13642102	2	13642102	13642382	281	7	35
CTTGCCGATT	CTTGCCGATTTTTCAGAAATTTTCAATTCCGGCAA						
Cele.WB235	Cele.WB235_2:13650128	2	13650128	13650200	73	6	12
TAGGCTTAGG	TAGGCTTAGGCT						
Cele.WB235	Cele.WB235_2:13668387	2	13668387	13668731	345	12	27
TTCCGGCAAA	TTCCGGCAAATCGGCAATTTTGAAAAT						
Cele.WB235	Cele.WB235_2:13681580	2	13681580	13681759	180	5	36
TTGCCTAAAA	TTGCCTAAAATTAAAATTTCCGGCAAATCAGCAAAT						
Cele.WB235	Cele.WB235_2:13715389	2	13715389	13715533	145	10	12
GCCTAAGCCT	GCCGAAGCCTAA						
Cele.WB235	Cele.WB235_2:13737396	2	13737396	13737775	380	15	21
CTCGTAAATC	CTCGTAGATCGACAAGAACTC						
Cele.WB235	Cele.WB235_2:13740296	2	13740296	13741317	1022	28	35
TTTGCCGGAA	TTTGCCGGAAATTTTGAATATTGGCATATTGCCGA						
Cele.WB235	Cele.WB235_2:13761268	2	13761268	13762971	1704	50	35
CCGGAATTGA	CCGGAATTGAAAATTTCTGGAAAATCGGCAAATTG						
Cele.WB235	Cele.WB235_2:13790809	2	13790809	13791073	265	22	10
CCGCTACCGC	CCGCTACCGC						
Cele.WB235	Cele.WB235_2:13795316	2	13795316	13795382	67	5	11
CCGCTACCGC	CCGCTACCGCA						
Cele.WB235	Cele.WB235_2:13796378	2	13796378	13796708	331	23	12
GCCTAAGCCT	GCCTAAGCCTAA						
Cele.WB235	Cele.WB235_2:13799836	2	13799836	13800686	851	47	18
AATTTACCAA	AATTTACCAATTTACCAA						
Cele.WB235	Cele.WB235_2:13806123	2	13806123	13806285	163	10	12
GCCTAAGCCT	GCCTAAGCCTTA						
Cele.WB235	Cele.WB235_2:13809662	2	13809662	13809758	97	7	12
TTAGGCTTAG	TTCGGCTTAGGA						
Cele.WB235	Cele.WB235_2:13831950	2	13831950	13832774	825	18	34
AATTCCGGCA	AATTCCGGCAATTTGCTGATTCATCAGAAATTTC						
Cele.WB235	Cele.WB235_2:13906504	2	13906504	13906669	166	5	33
AAAATTTGAA	AAAATTTGAATTTCCCGTCAAAATTTTTCTCAG						
Cele.WB235	Cele.WB235_2:13934639	2	13934639	13934753	115	7	12
AGCCTAAGCC	ACCTAAGCCTAC						

Cele.WB235	Cele.WB235_2:13962702	2	13962702	13964354	1653	47	14
TACAAACTAC	TACAAACTACAAAC						
Cele.WB235	Cele.WB235_2:13967824	2	13967824	13968381	558	7	62
TGCTAACTAG	TGCTAACTAGCGCGGGCCTGCTTAGGTGCCGGCTGCGAAAGCAGTCCTGGTTTAATGTCGGC						
Cele.WB235	Cele.WB235_2:13974189	2	13974189	13974267	79	6	12
GGCTTTGGCT	GGCTTTGGCTTT						
Cele.WB235	Cele.WB235_2:13974202	2	13974202	13974430	229	14	12
GCTTAGGCTT	GCTTCGGCTTTG						
Cele.WB235	Cele.WB235_2:13989510	2	13989510	13989877	368	11	11
GTACTGTAGG	GTACTGTAGGA						
Cele.WB235	Cele.WB235_2:13990069	2	13990069	13990966	898	30	30
TTTCTAGAAA	TTTCTAGAAAAATCAGAAAACTTTTGGAAC						
Cele.WB235	Cele.WB235_2:14006143	2	14006143	14006320	178	5	14
GTAGTTTGTA	GTAGTTTGTAGTTT						
Cele.WB235	Cele.WB235_2:14006879	2	14006879	14006969	91	5	12
TAGGCTTAGG	TAGGCTTAGGCT						
Cele.WB235	Cele.WB235_2:14023987	2	14023987	14024052	66	4	12
TAGGCTTAGG	TAGGCTTAGGCT						
Cele.WB235	Cele.WB235_2:14100395	2	14100395	14100701	307	8	34
CCGTGCCTAC	CCGTGCCTACTAACCTACCGACCTTGCCTGCCTC						
Cele.WB235	Cele.WB235_2:14105236	2	14105236	14105489	254	11	18
CTAGGTGCCT	CTAGGTACCTATCCATTC						
Cele.WB235	Cele.WB235_2:14108714	2	14108714	14108890	177	10	16
TAGGCATATA	TAGGCATATAGGCTTA						
Cele.WB235	Cele.WB235_2:14114648	2	14114648	14115030	383	12	22
CTACAGTAAC	CTACAGTAACCCTAGTTTTGAA						
Cele.WB235	Cele.WB235_2:14130915	2	14130915	14131087	173	4	43
GAAAACTAGG	GAAAACTAGGCCACAGAGTTCGAGTTGACGGCCTAGAAATCTA						
Cele.WB235	Cele.WB235_2:14137014	2	14137014	14137524	511	14	34
CGGAATTGAA	CGGAATTGAAATTTCCGGCAAATTGGTAAAATGC						
Cele.WB235	Cele.WB235_2:14166594	2	14166594	14166830	237	18	12
ATAGGCATAG	ATAGGCATAGAC						
Cele.WB235	Cele.WB235_2:14190380	2	14190380	14191034	655	13	13
TATACAAAAA	TATACAAAAAATA						
Cele.WB235	Cele.WB235_2:14192423	2	14192423	14193096	674	16	42
AAAACCGGCA	AAAACCGGCAATTGCTGGAATTGCCGATTGCCGAATATTACC						
Cele.WB235	Cele.WB235_2:14212202	2	14212202	14212432	231	10	21
CAATTTTCTG	CAATTTTCTGTCAGACTTTGA						
Cele.WB235	Cele.WB235_2:14239112	2	14239112	14239812	701	18	35
GCAAATCGGC	GCAAATCGGCAAGTTGCCGGAATTGAAAATTTCAG						
Cele.WB235	Cele.WB235_2:14264215	2	14264215	14264530	316	8	35
CGGCAAATCG	CGGCAAATCGGCAAATTGCCGGAATTGTTAATTTC						
Cele.WB235	Cele.WB235_2:14269513	2	14269513	14269895	383	11	35
CCGGCAAATC	CCGGCAAATGGGCAAATTGCCGAAAATGAAAGCAT						
Cele.WB235	Cele.WB235_2:14273310	2	14273310	14273525	216	5	35
CGGCAAATTG	CGGCAAATCGGCTAATTGCCGAAATTGAAAATTTC						
Cele.WB235	Cele.WB235_2:14274983	2	14274983	14275913	931	26	32
AACCAATCAG	AACCAATCAGCGATGGGCTCCACCCTTTTTGA						
Cele.WB235	Cele.WB235_2:14278881	2	14278881	14279293	413	12	35
GCCGGAAATT	GCCGGAAATTTCCAATTCCGCCAATTTGCCGATTT						
Cele.WB235	Cele.WB235_2:14323963	2	14323963	14331913	7951	43	185
CTCGGACATA							

CTCGGACATAATTGGTGTTCTGTATTCACTTATGATACGTTCGGCTGCGTACATTGGCCAAGAGTGTTCTAGTAAGAGAATTTCATTTAGAATATGAACAATTTGGGTAATTGGAAAGTGTCTTCGGGAATCT
TTATACATCCGACAAATTCGAGTATGTTAGTATTCTGAATTGGATTCTTTTT
Cele.WB235 Cele.WB235_2:14333431 2 14333431 14337579 4149 25 166
TCGTAATTAT
TCGTAATTATTCATAAGTTTGTGTTGTCTCCGAGCCATGTCTGAAAAAACAGATTTTTTTTCGAAAATTTTAAAAAGTTCGTAAAAATTGGATTTTTGACAAATCTATGCTCTCTTTTTGTGTTTAAAATTTA
GTTCAGTGACATACAATTTCAAAAATATAATGT
Cele.WB235 Cele.WB235_2:14337687 2 14337687 14339240 1554 57 27
GGCTTCCCAC GGCTTCCCACCATATTTTACTCTCTGT
Cele.WB235 Cele.WB235_2:14346267 2 14346267 14346344 78 5 15
CGAAAAGGGG CGAAAAGGGGATCTG
Cele.WB235 Cele.WB235_2:14346519 2 14346519 14346611 93 6 15
TCCCCTTTTC TCCCCTTTTCGTATC
Cele.WB235 Cele.WB235_2:14355352 2 14355352 14355470 119 5 18
TAGTCTTGCA TAGTCTTGCAGCCTCTAT
Cele.WB235 Cele.WB235_2:14738853 2 14738853 14738949 97 6 16
GCAGTTTGCC GCCGTTTGCCGAATTG
Cele.WB235 Cele.WB235_2:14780596 2 14780596 14780942 347 4 35
TCCGGCAAAT TCCGGCAAATCGGCAAATTGGTGAAATTTAAAATT
Cele.WB235 Cele.WB235_2:14802929 2 14802929 14803074 146 7 21
ATTGGATTAG ATTGGATTAGATTTGTTCCAT
Cele.WB235 Cele.WB235_2:14836506 2 14836506 14836646 141 5 30
TTCTGGAAAA TTCTGGAAAATTCTAGAATGTTCCAGAACC
Cele.WB235 Cele.WB235_2:14836705 2 14836705 14836840 136 9 15
AGACCCATCG AGACCCATCGTGGCG
Cele.WB235 Cele.WB235_2:14836894 2 14836894 14837772 879 25 35
TTAAATTTTC TTAAATTTTCTGTGAAAAATATTTTGGCGGGAAAA
Cele.WB235 Cele.WB235_2:14870141 2 14870141 14870668 528 13 35
TTTCCGGCAA TTTCCGGCAAATCGGCAAATTGTTGGACTTGAAAA
Cele.WB235 Cele.WB235_2:14878167 2 14878167 14878554 388 11 35
CAGAAAATTT CAGAAAATTTAAATTTCCCGCCAAAATATTTTTCA
Cele.WB235 Cele.WB235_2:14878607 2 14878607 14878727 121 7 15
CCACGATGGG CCACGATGGGTCTCA
Cele.WB235 Cele.WB235_2:14878787 2 14878787 14878927 141 5 30
AATTTTCCAG AATTTTCCAGAAAGTTCTGGAACATTCCAG
Cele.WB235 Cele.WB235_2:14893703 2 14893703 14893787 85 6 12
TTAGGCTTAG TTAGGCTAAGGT
Cele.WB235 Cele.WB235_2:14955292 2 14955292 14955352 61 5 12
AAGCCTAAGC AAGCCCAAGCCT
Cele.WB235 Cele.WB235_2:14984666 2 14984666 14984990 325 14 20
TTCAGTCAAA TTCAGTCAAAATTTAGATTT
Cele.WB235 Cele.WB235_2:14991646 2 14991646 14991736 91 6 15
TCGTGGCGAG TCGTTGCGAGACCCT
Cele.WB235 Cele.WB235_2:14991755 2 14991755 14991977 223 7 32
TTTTGGCGGG TTTTGGCGGGAATTTAAATTTTCTTTGAAATT
Cele.WB235 Cele.WB235_2:14993591 2 14993591 14993681 91 6 15
GGTGAGACCC GGTGAGACCCTTCGT
Cele.WB235 Cele.WB235_2:14993697 2 14993697 14994076 380 11 34
TTGGCGGGAA TTGGCGGGAAATTCAAATTTTCAGTGAAAAAATT
Cele.WB235 Cele.WB235_2:15000612 2 15000612 15000786 175 9 15
AGACCCATCG AGACCCATCGTGGCG
Cele.WB235 Cele.WB235_2:15000726 2 15000726 15000995 270 5 35
ATTTTGGCGG ATTTTGGCGGGTAATTTAAATATTCGGAGAAAAAT

Cele.WB235 Cele.WB235_2:15044151 2 15044151 15044326 176 5 35
TTTGCCGGAA TTTGCCGGAAATTTTAAATTCTGGCAATTTGCCGA
Cele.WB235 Cele.WB235_2:15047976 2 15047976 15048537 562 16 35
TTTGCCGGAA TTTGCCGGAAAGTTTCAATTCAGGCAACTTGTCGT
Cele.WB235 Cele.WB235_2:15111116 2 15111116 15111396 281 8 35
TTTGCCGGAA TTTGCCGGAAATTTTCAATTCCGGCAATATACTGA
Cele.WB235 Cele.WB235_2:15138506 2 15138506 15140272 1767 38 15
GGGTCTCACC GGGTCTCACCACAAC
Cele.WB235 Cele.WB235_2:15186190 2 15186190 15187654 1465 9 163
AAATAAAAAA
AAATAAAAAAGAATGACTCCATATGATTTTCCCATACTTTTGAATTTTCGAAAAAATCAGCTTTTATATATTTTTATCAACTTTTAAAAAAATACTTCCGTACAAGCTAATATACTCTGTTTGGCATTTTCTG
GTAACTTTGACTATGCTGAACAGTTTTATG
Cele.WB235 Cele.WB235_2:15188581 2 15188581 15189224 644 18 34
TTTCAATTCC TTTCAATTCCGGCAACTTTCCGATTTGGCGGAAA
Cele.WB235 Cele.WB235_2:15194481 2 15194481 15194561 81 4 20
TGTAGATTTA TGTAGATTTACGAAGGACAA
Cele.WB235 Cele.WB235_2:15210567 2 15210567 15211143 577 12 35
CCGATTTGCC CCGATTTTCCGGAAATTTCGAATTCCGGCAACTTC
Cele.WB235 Cele.WB235_2:15211519 2 15211519 15211997 479 9 35
TTGCCGGAAT TTGCCAGAATTGAAAAATTCTGGCAAACCGCCAAA
Cele.WB235 Cele.WB235_2:15278603 2 15278603 15278843 241 12 23
GCTACAGTAC GCTACAGTACCCCAGCTTGACTT
Cele.WB235 Cele.WB235_2:15279229 2 15279229 15279409 181 15 12
TTAGGCTTAG TTAGGCTTAGGC
Cele.WB235 Cele.WB235_3:0 3 0 119 120 10 12
CCTAAGCCTA CCTAAGCCTAAG
Cele.WB235 Cele.WB235_3:78610 3 78610 79849 1240 39 31
TAAATAATTA TAAATAATTAGCAGACCAAAGTTGGGTCTGC
Cele.WB235 Cele.WB235_3:80880 3 80880 81257 378 14 21
AAATTTGGCT AAATTTGGCTGGAAATCGTGA
Cele.WB235 Cele.WB235_3:83578 3 83578 83851 274 13 21
CCATTTTAAG CCATTTTAAGCCAATTTTGGA
Cele.WB235 Cele.WB235_3:83578 3 83578 84096 519 25 21
CCATTTTAAG CCATTTTAAGCCAATTTTGGA
Cele.WB235 Cele.WB235_3:85304 3 85304 85614 311 9 31
TGCTAAATAA TGCTAAATAATTAGCAGACCATTTTTGGGTC
Cele.WB235 Cele.WB235_3:118094 3 118094 118166 73 5 12
GCCTAAGCCT GCCTAAGCCTAA
Cele.WB235 Cele.WB235_3:133200 3 133200 133804 605 7 34
AATTCCGGCA AATTCCGGCAATTTACCAATTTGCCAGAAATTTC
Cele.WB235 Cele.WB235_3:187630 3 187630 188214 585 4 31
TTAGCAGACC TTAGCAGACCCAAGTATGGTCTGCTAATTAT
Cele.WB235 Cele.WB235_3:189539 3 189539 190145 607 5 31
TTAGCAGACC TTAGCAGACCCAAGTATGGTCTGCTAAATAA
Cele.WB235 Cele.WB235_3:191259 3 191259 191627 369 7 31
GGTCTGCTAA GGTCTGCTAAATATTTAGCATACCCAACTTT
Cele.WB235 Cele.WB235_3:193180 3 193180 193459 280 9 31
ACCCAAGTAT ACCCAAGTATGGTCTGCTAATTATTTAGCAA
Cele.WB235 Cele.WB235_3:206747 3 206747 206867 121 7 16
TAGGCATCTA TAGGCATCTAGGCAGG
Cele.WB235 Cele.WB235_3:209292 3 209292 209524 233 8 12
AAGCCTAAGC AAGACTAAGCCT

Cele.WB235	Cele.WB235_3:244458	3	244458	244613	156	4	31	AGCTCAAAAT	AGCTCAAAATCTGTAAAAATGCCCATTTTTG
Cele.WB235	Cele.WB235_3:255029	3	255029	255455	427	12	30	TTTTTTGAGC	TGTTTTGAGCCAAAAATTCAAAAAATCTCG
Cele.WB235	Cele.WB235_3:259921	3	259921	260355	435	11	11	CCTACAGTAC	CCTACAGTACA
Cele.WB235	Cele.WB235_3:267063	3	267063	268369	1307	13	101	GGTTACTGTA	GGTTACTGTAGTTCCAAAAGTACGCAAACACTGACTACGGTTCCCTAATACATCAGATATTACGACAAAGTGCAGAAAATCGCTAAAAATGACTTTACTGG
Cele.WB235	Cele.WB235_3:334126	3	334126	334653	528	17	31	TGGTCTGCTA	TGGTCTGCTAAATAATTAGCATACCCAACTT
Cele.WB235	Cele.WB235_3:337086	3	337086	337644	559	17	31	TATGCTAAAT	TATGCTAAATATTTAGCAGACCAAAAATAGG
Cele.WB235	Cele.WB235_3:340719	3	340719	342118	1400	23	31	TTAGCAGACC	TTAGCAGACCCAAGGTTGGTCTGCTAAATAT
Cele.WB235	Cele.WB235_3:345158	3	345158	345375	218	7	31	CTGCTAAATA	CTGCTAAATATTTAGCATACCAACTTTGGGT
Cele.WB235	Cele.WB235_3:346580	3	346580	346716	137	5	31	AATATTTAGC	AATATTTAGCAGACCAAAGATGGGTATGCTA
Cele.WB235	Cele.WB235_3:404417	3	404417	405141	725	7	40	TCCACGTGGA	TCCACGTGGAATTTTTAGAAATTTCTCAAAAAAATTGAAA
Cele.WB235	Cele.WB235_3:410715	3	410715	410901	187	6	31	TTGGGTCTGC	TTGGGTCTGCTAAATATTTAGCAGACCATAA
Cele.WB235	Cele.WB235_3:423133	3	423133	423737	605	6	31	TATTTAGCAG	TATTTAGCAGACCCAATTATGGTCTGCTAAT
Cele.WB235	Cele.WB235_3:424806	3	424806	426326	1521	38	40	TAGAAAGTTC	TAGAAAGTTCTGGAACATTCCAGAATTTTCTCGATTTTTC
Cele.WB235	Cele.WB235_3:424780	3	424780	427345	2566	43	40	ACATTCCAGA	ACATTCCAGAATTTTCTCGATTTTTCTAGAAAGTTCTGGA
Cele.WB235	Cele.WB235_3:427473	3	427473	427977	505	18	11	CTACAGTACT	CTACAGTACTC
Cele.WB235	Cele.WB235_3:449837	3	449837	453285	3449	21	164	ATATAATGCT	ATATAATGCTCGTAATTATTAATAAATGTGTGTTGTCTCAGAGCCACGTCTGAAAATACAGATTTTTTCTCGAAAATTTCAAAAACTTTGTAGAAATTCGATTTTTGACATAAGTGATCTCTCTTTTTTTCTGACATGTATTTCAGTGACAACTGTACTCGAAA
Cele.WB235	Cele.WB235_3:470472	3	470472	471505	1034	16	31	TTTAGCAGAC	TTTAGCAGACCAAAGTTGGGTATGCTAAATA
Cele.WB235	Cele.WB235_3:492131	3	492131	493485	1355	34	31	TTAGCAGACC	TTAGCAGACCAAAGATAGGTATGCTAAATAC
Cele.WB235	Cele.WB235_3:498249	3	498249	499054	806	26	31	GGTCTGCTAA	GGTCTGCTAATTATTCAGCATACCCAAATTT
Cele.WB235	Cele.WB235_3:520005	3	520005	520140	136	6	11	CTACAGTACC	CTACAGTAACC
Cele.WB235	Cele.WB235_3:584894	3	584894	585235	342	11	31	GGTCTGCTAA	GGTCTGCTAATTATTTAGCATACCGAACTTC
Cele.WB235	Cele.WB235_3:587989	3	587989	588144	156	5	31	TGGGTATGCT	TGGGTATGCTAAATAATTAGCAGACCAAAGT
Cele.WB235	Cele.WB235_3:633852	3	633852	634044	193	6	31	TTAGCAGACC	TTAGCAGACTCATGGTAGGTATGCTAAATAT
Cele.WB235	Cele.WB235_3:635824	3	635824	635934	111	6	18	TAGGCTTAGG	TAGTCTTAGGCTAACCTT
Cele.WB235	Cele.WB235_3:690245	3	690245	690736	492	19	15	TGAGACCCAT	TGAGACCAATCGTGA

Cele.WB235	Cele.WB235_3:700450	3	700450	700543	94	5	15
GGGGGCAACA	GGGGGCAACAAATTA						
Cele.WB235	Cele.WB235_3:705083	3	705083	705230	148	8	15
TGTTGCCCCC	TGTTGACCCCTATTA						
Cele.WB235	Cele.WB235_3:855590	3	855590	855838	249	7	31
TAAATATTTA	TAAATATTTAGTAGACCAAAGTTGGGTATGC						
Cele.WB235	Cele.WB235_3:922387	3	922387	922734	348	8	18
TGCAAGACTA	TGCAAGACTATTAGAAGC						
Cele.WB235	Cele.WB235_3:922819	3	922819	923128	310	5	18
TAGTGCTGCA	TAGTGCTGCAGTCTCTAT						
Cele.WB235	Cele.WB235_3:931109	3	931109	931213	105	4	26
CGGCCAGAGT	CGGCCAGAGTCACTATATTTGGTGAA						
Cele.WB235	Cele.WB235_3:931250	3	931250	931684	435	16	27
AGAGAGTAAA	AGAGAGTAAAATATAGTGGGAAGCCAC						
Cele.WB235	Cele.WB235_3:999373	3	999373	999632	260	12	22
TAAATCTACA	TAAATCTACACACTTGGCCTCG						
Cele.WB235	Cele.WB235_3:1011046	3	1011046	1011124	79	5	16
TTATAGTGTT	TTATAGTGTTATAGTG						
Cele.WB235	Cele.WB235_3:1017334	3	1017334	1020579	3246	55	59
TGTGCACTGG	TGTGCACTGGTTTTCAAAAATTTTTAACTGAGAATTATGGAAAAAATATAAATTTAAA						
Cele.WB235	Cele.WB235_3:1052407	3	1052407	1052587	181	10	12
AAGCCTAAGC	AAGCCTAAGCCT						
Cele.WB235	Cele.WB235_3:1056939	3	1056939	1057667	729	14	40
AAAATCCACG	AAAATCCACGTAGATTATCGATTTTTCATAGAATTTTGCA						
Cele.WB235	Cele.WB235_3:1056932	3	1056932	1058332	1401	16	40
TTTTGCAAAA	TTTTGCAAAAATCCACGTAGATTATCGATTTTTCATAGAA						
Cele.WB235	Cele.WB235_3:1119657	3	1119657	1120064	408	4	96
GAGGGCCGCA	GAGGGCCGCAGGCCCGAACACTCTTGGAAACTTTGGGCTGGCTTTGATCCTATTCTAAGCAGATATGTTATTTACTCAAACCTAACAACCAGGTTC						
Cele.WB235	Cele.WB235_3:1148439	3	1148439	1149466	1028	13	35
TTTAAAATTT	TTTAAAATTTCCGGCAAATCGCCAAATTGCCGGAA						
Cele.WB235	Cele.WB235_3:1154872	3	1154872	1155214	343	4	21
ACTACAGTAC	ACTACAGTACCCCGTACGACT						
Cele.WB235	Cele.WB235_3:1245524	3	1245524	1246875	1352	116	11
TTTTTGCACA	TTTTTGCACAT						
Cele.WB235	Cele.WB235_3:1256230	3	1256230	1256326	97	5	12
AGGCTTAGGC	AGGCTTAGGCTC						
Cele.WB235	Cele.WB235_3:1260242	3	1260242	1261618	1377	70	20
AAAAATAGAT	AAAAATAGATTGACAGACGA						
Cele.WB235	Cele.WB235_3:1264671	3	1264671	1264767	97	5	12
AGGCTTAGGC	AGGCTTAGGCTC						
Cele.WB235	Cele.WB235_3:1299759	3	1299759	1303117	3359	134	25
CAGAGAGTAA	CAGAGAGTAAAATAATTGGTGAAGC						
Cele.WB235	Cele.WB235_3:1312388	3	1312388	1312777	390	16	21
TTTTTGGTAA	TTTTTGGTAAAATTTGATAAT						
Cele.WB235	Cele.WB235_3:1314701	3	1314701	1315015	315	12	21
CAAAAAGTTA	CAAAAAGTTATCAAATTTTAC						
Cele.WB235	Cele.WB235_3:1315590	3	1315590	1315824	235	5	35
CAAATCGGCA	CAAATCGACAAATTGCCGGAATTGAAAATTTCCGG						
Cele.WB235	Cele.WB235_3:1320466	3	1320466	1320612	147	5	11
GTACTGTAGG	GTACTGTAGGA						
Cele.WB235	Cele.WB235_3:1320777	3	1320777	1320837	61	4	15
TCGTGGTGAG	TCGTGGTGAGACCCA						

Cele.WB235	Cele.WB235_3:1320896	3	1320896	1321023	128	4	31
AATTCAAATT	AATTCAAATTTATTTAAAAAAATTTGGCGGG						
Cele.WB235	Cele.WB235_3:1326908	3	1326908	1327395	488	33	11
GGTACTGTAG	GGTACTGTAGG						
Cele.WB235	Cele.WB235_3:1334409	3	1334409	1335297	889	40	14
ATACCTAATA	ATACCTAATAACTA						
Cele.WB235	Cele.WB235_3:1385873	3	1385873	1386532	660	5	43
GGCTCTCGGC	GGCTCTCGGCTCGCGCCGAGGGCCGAGTTCCACTAAAAGTTTC						
Cele.WB235	Cele.WB235_3:1534930	3	1534930	1535260	331	16	20
CATAAAAATT	CATAAAAATTCTGAAAAATG						
Cele.WB235	Cele.WB235_3:1536002	3	1536002	1536441	440	21	20
TTTCACTAAA	TTTCACTAAAAATCCTAATT						
Cele.WB235	Cele.WB235_3:1560064	3	1560064	1560620	557	7	21
CCTAAAAATT	CCTAAAAATTTCAATTTTTGT						
Cele.WB235	Cele.WB235_3:1608442	3	1608442	1609120	679	5	72
ATGAATTTTA	ATGAATTTTAATACAATTTTCCTGACGATGAAGTCATAAAAATTCATTTTTTGACCTTTCAACTTTAGTTTT						
Cele.WB235	Cele.WB235_3:1633390	3	1633390	1634249	860	41	11
TACTGTAGGG	TACTGTAGGGG						
Cele.WB235	Cele.WB235_3:1643024	3	1643024	1643390	367	4	92
CTGCGGAACC	CTGCGGAACCCCGAACGTGTCGGCCGCTTCCAAAAACAACCACCTCTTCACACTTCATTGCGCACACACCAAACTACTAGTTTCACGTCAAA						
Cele.WB235	Cele.WB235_3:1656487	3	1656487	1657087	601	50	12
TGAGCCTGAG	TGAGCCTGAGCC						
Cele.WB235	Cele.WB235_3:1671460	3	1671460	1671581	122	11	11
GCAAAAAATG	GCAAAAAATGT						
Cele.WB235	Cele.WB235_3:1714052	3	1714052	1714520	469	35	12
CTAAGCCTAA	CTAAGCCTAACC						
Cele.WB235	Cele.WB235_3:1761961	3	1761961	1762051	91	6	12
AGGCTTAGGC	AGGCTTAGGCTC						
Cele.WB235	Cele.WB235_3:1805381	3	1805381	1807736	2356	56	19
AAAATCGATA	AAAATCGATATTTTATCGG						
Cele.WB235	Cele.WB235_3:1809979	3	1809979	1810419	441	36	11
ATTTTTTGCA	ATTTTTTGCAC						
Cele.WB235	Cele.WB235_3:1892229	3	1892229	1893089	861	44	19
AAAATCGATA	AAAATCGATAATTTCTCGG						
Cele.WB235	Cele.WB235_3:1934627	3	1934627	1936118	1492	61	21
TGGAAATTTT	TGGAAATTTTAGCCCAAAAAC						
Cele.WB235	Cele.WB235_3:1964229	3	1964229	1964835	607	30	12
TAGGCTTAGG	TAGGCTTAGGCT						
Cele.WB235	Cele.WB235_3:1976102	3	1976102	1976245	144	11	11
GTCGCGTTGC	GTCGCATTGCG						
Cele.WB235	Cele.WB235_3:1985833	3	1985833	1986090	258	24	11
TGTGCAAAAA	TGTGCAAAAAA						
Cele.WB235	Cele.WB235_3:2009666	3	2009666	2010596	931	27	31
TGCGTCGGAA	TGCGTCGGAAATTTATTCCGATCATTGCCGG						
Cele.WB235	Cele.WB235_3:2011573	3	2011573	2012317	745	17	31
TCCGATGCAT	TCCGATGCATTGCCGATAATTGCCACCTGGT						
Cele.WB235	Cele.WB235_3:2012861	3	2012861	2014349	1489	36	19
TATCGATTTT	TATTGATTTTTTGCTAAAA						
Cele.WB235	Cele.WB235_3:2035722	3	2035722	2035981	260	20	11
CTACAGTACC	CTACAGTACTT						
Cele.WB235	Cele.WB235_3:2036337	3	2036337	2036525	189	9	20
TGTAGATTTA	TGTGGATTTACAGGAATCTA						

Cele.WB235	Cele.WB235_3:2066729	3	2066729	2067447	719	12	35	TTTGCCGGAA	TTTGCCGGAAATTTTCAATTCCGACAATTTGCCGA
Cele.WB235	Cele.WB235_3:2077634	3	2077634	2082512	4879	29	168	TTTATTTTTG	TTTATTTTTGTTTTTTTGTGCCTACATATACCTGTTGAAATTGGAATTCAAGACAAAAACCCCTGTAGCGCGTCTGAAAATGGATATCTAGGTACAAAAACTTACCAATAATATTTTAAAACAATCTAGAATCCAAATCTATCAAAACATTTCCGGAATTCCATAATG
Cele.WB235	Cele.WB235_3:2103059	3	2103059	2103647	589	25	21	GCCTAAAAAC	GCATAAAAACGTAAATTTTAG
Cele.WB235	Cele.WB235_3:2110682	3	2110682	2111451	770	22	31	ATTTCCCGCC	ATTTCCCGCCAAAAAATATTTGAAAACTTGA
Cele.WB235	Cele.WB235_3:2113231	3	2113231	2114730	1500	39	39	TTTTTGGCGG	TTTTTGGCGGGAAATTTCAAAATTCAAATTTTTGAATAT
Cele.WB235	Cele.WB235_3:2117069	3	2117069	2117228	160	4	40	TCCCGCCAAA	TCCCGCCAAAAAATATTCAAAAAATTTGAATTTTGAAATT
Cele.WB235	Cele.WB235_3:2218366	3	2218366	2219198	833	23	32	CTCAGCCAAT	CTCAGCCAATCAGCAATTAGCTCTGCCTATTT
Cele.WB235	Cele.WB235_3:2235593	3	2235593	2235697	105	5	21	CTGACCAAAT	CTGACCAAATTTGTTTTAGCA
Cele.WB235	Cele.WB235_3:2245221	3	2245221	2245452	232	9	21	GTCAGTGCTG	GTCAGTGCTGTAACACATTTT
Cele.WB235	Cele.WB235_3:2455329	3	2455329	2456409	1081	40	10	AGGCAGGCTT	AGGCAGGCAT
Cele.WB235	Cele.WB235_3:2456635	3	2456635	2456707	73	5	12	CCAAGCCCAA	CCAAGCCCAATC
Cele.WB235	Cele.WB235_3:2470723	3	2470723	2471262	540	27	20	GACCCAGCCT	GACCCAGCCTACCCCAGGCA
Cele.WB235	Cele.WB235_3:2470728	3	2470728	2471526	799	40	20	AGCCTACCCC	AGCCTACCCCAGGCAGACCC
Cele.WB235	Cele.WB235_3:2496715	3	2496715	2496983	269	6	45	TCTGATCCTT	TCTGATCCTTCAGCTCCGAAGGATCACATGGGTACCTTCTGATGT
Cele.WB235	Cele.WB235_3:2497037	3	2497037	2501716	4680	187	25	GTGAAGCCAG	GTGAAGCCAGAGAGTAAAATATACG
Cele.WB235	Cele.WB235_3:2501737	3	2501737	2501917	181	5	40	TTCCAGAATT	TTCCAGAATTTTCCCGATTTTTCTAGAAAGTTCTGGAACG
Cele.WB235	Cele.WB235_3:2502140	3	2502140	2502620	481	29	15	GGTCTCACCA	GGTCTCACCACGACG
Cele.WB235	Cele.WB235_3:2545690	3	2545690	2546082	393	11	35	TTTGCCGGAA	TTTGCCGGAAATTTTCAATTCCGGCAATTTGCCGA
Cele.WB235	Cele.WB235_3:2791812	3	2791812	2792160	349	10	35	TTTGCCGGAA	TTTGCCGGAAAATTCAAATTCCGGCAATTTGCCGC
Cele.WB235	Cele.WB235_3:2836362	3	2836362	2837893	1532	45	19	AAAATCGATA	AAAATTGATAATTTCCGGA
Cele.WB235	Cele.WB235_3:2977664	3	2977664	2978383	720	4	180	TGTCGGCCGC	TGTCGGCCGCTGTATCTTTTGCCTCAATTTTTTTTAAATTTTCAAACATACATATTCCATTAGGTAACATTATGAGCTACAGTTTGTTAGTTGGAATTTTATTTCTAAAATTACTAGTTTCTGAGTTATACTCATTTCAATATCCCCCAAGTGAAATCGCCTAAAAATGAACTCGCGAGG
Cele.WB235	Cele.WB235_3:3018177	3	3018177	3018907	731	5	16	TGTATATATA	TGTATATATATATATA
Cele.WB235	Cele.WB235_3:3023864	3	3023864	3024701	838	18	37	ATTTTGGCGG	ATTTTGGCGGGATTTCAAATTTGAATTTTTTGAAAAT
Cele.WB235	Cele.WB235_3:3030347	3	3030347	3030891	545	14	35	AATTGCCGGA	AATTGCCGGAATTGAAACTTTCCGGCGAATTGGAA

Cele.WB235	Cele.WB235_3:3037879	3	3037879	3038500	622	13	35
AAATCGGCAA	AAATCGGAAAATTGCCGGAATTGAAAATTTCCAGA						
Cele.WB235	Cele.WB235_3:3042951	3	3042951	3043689	739	38	18
GTCGATTTAC	GTCGATTTACGAGATTTT						
Cele.WB235	Cele.WB235_3:3078601	3	3078601	3079369	769	4	151
TTTAAAGGCG	TTTAAAGGCGCATAGGTAGATCAAAACTCAAAGTTGGCGGATTCTAGTCAATATGGGGCTTATTTTAAAGCTCTCGGTAAGCTGAATGTAGTTTAGCTACTGAAAATTGAAAATTTAAACAATTTTTGGGTCTAGCAGCGAAAATTTCGAA						
Cele.WB235	Cele.WB235_3:3085487	3	3085487	3086399	913	5	151
TTTAAAGGCG	TTTAAAGGCGCATAGGTAGATCAAAACTCAAAGTTGGCGGATTCTAGTCAATATGGGGCTTATTTTAAAGCTCTCGGTAAGCTGAATGTAGTTTAGCTACTGACAATTGAAAATTTAAACAATTTTTGGGTCTAGCAGCGAAAATTTCGAA						
Cele.WB235	Cele.WB235_3:3229003	3	3229003	3229241	239	7	30
TAGAACTTTC	TAGAACTTTTCAGTTTTTTCTAGAAAACTC						
Cele.WB235	Cele.WB235_3:3230816	3	3230816	3231055	240	8	30
TAGAACTTTC	TAGAACTTTCCAAATTTCTCTAGAAAATTC						
Cele.WB235	Cele.WB235_3:3233988	3	3233988	3234755	768	13	19
CTTGGCGCAA	CTTGGCGCAATTCTCGTGC						
Cele.WB235	Cele.WB235_3:3272813	3	3272813	3272897	85	4	21
TCCAGCACTG	TCCAGCACTGACAAATTTTGT						
Cele.WB235	Cele.WB235_3:3334092	3	3334092	3334302	211	17	12
AAAATAAAAA	AAAATAAAAATA						
Cele.WB235	Cele.WB235_3:3334358	3	3334358	3337083	2726	109	25
ACTCTCTGGC	ACTCTCTGGCTTCCCACTATATTTT						
Cele.WB235	Cele.WB235_3:3346751	3	3346751	3346936	186	12	12
TAGGCTTAGG	TAGGCTTAGGCT						
Cele.WB235	Cele.WB235_3:3385070	3	3385070	3386871	1802	19	95
TAATCCTATT	TAATCCTATTCCAAGCAGATTGTTATTCGCTCAAGCCTACAAACCAGGTTTGAGGGCCGCAGGCCCGAATACTCATGGAAACTGTGGGCCAGCTT						
Cele.WB235	Cele.WB235_3:3446010	3	3446010	3446749	740	47	15
GTGGTGAGAC	GTGGTGAGACCCGTC						
Cele.WB235	Cele.WB235_3:3542667	3	3542667	3542838	172	5	34
AATTGAAATT	AATTGAAATTTCCGGCAAATCGACAAATTGCGGG						
Cele.WB235	Cele.WB235_3:3647083	3	3647083	3647155	73	4	16
CCTACATACC	CCTACATACCTACATA						
Cele.WB235	Cele.WB235_3:3661285	3	3661285	3661688	404	8	35
TTCCGGCAAA	TTCCGGCAAATCGACAATTTTCCGAAAATGAAAAT						
Cele.WB235	Cele.WB235_3:3684600	3	3684600	3684680	81	5	12
TAAGCCTAAG	TAAGCCTAAGCC						
Cele.WB235	Cele.WB235_3:3740014	3	3740014	3740583	570	9	30
CCTTGTAGGC	CCTTGTAGGTAAGGAGGTAGGTGTAGGCCG						
Cele.WB235	Cele.WB235_3:3746895	3	3746895	3747055	161	10	16
TAGGCATGTA	TAGACATGTAGGCATG						
Cele.WB235	Cele.WB235_3:3794723	3	3794723	3795033	311	5	18
TGCAGCACTA	TGCAGCACTATTAGAGAC						
Cele.WB235	Cele.WB235_3:3895928	3	3895928	3896228	301	8	40
AGAACCTTCT	AGAACCTTCTGGGAATTTCTGGAAAATTCTAGAATGTTCC						
Cele.WB235	Cele.WB235_3:3896301	3	3896301	3896751	451	30	15
CGTGGTGAGA	CGTGGTGAGACCCAT						
Cele.WB235	Cele.WB235_3:3896794	3	3896794	3897038	245	7	35
AATTTAAATT	AATTTAAATTTTCTGTGAAAAATATTTTGGCGGGA						
Cele.WB235	Cele.WB235_3:3897112	3	3897112	3897672	561	14	40
GTTCCAGAAC	GTTCCAGAACTTTCTAGAAAAATCGGGAAAATTCTGGAAT						

Cele.WB235	Cele.WB235_3:3987076	3	3987076	3987676	601	31	19	AAAAATAGAT	AAAAATAGATTGTTACACG
Cele.WB235	Cele.WB235_3:4254156	3	4254156	4254274	119	7	17	AGGGGGCATC	AGGGGGCATTAGGTCAA
Cele.WB235	Cele.WB235_3:4257044	3	4257044	4257162	119	7	17	GATGCCCCCT	GATGCCCCCTCTGAATG
Cele.WB235	Cele.WB235_3:4637893	3	4637893	4638151	259	5	17	ATTATTTTCA	ATTATTTTCATTCCGTG
Cele.WB235	Cele.WB235_3:5285020	3	5285020	5285610	591	28	20	CTAGGTCTGC	CTAGGTCTGCCTAGGGTAGG
Cele.WB235	Cele.WB235_3:5285044	3	5285044	5285922	879	40	20	GGTAGGCTAG	GGTAGGCTAGGTCTGCCCAG
Cele.WB235	Cele.WB235_3:5353177	3	5353177	5358366	5190	198	26	GTTAGAGTCA	GTTAGAGTCATTATGTTAGGTGAATG
Cele.WB235	Cele.WB235_3:5358375	3	5358375	5358505	131	5	26	ACCATTCACC	ACCATTCACCCAAAATAATGACTCTA
Cele.WB235	Cele.WB235_3:5941097	3	5941097	5943578	2482	10	228	CCACCAGATC	CCACCAGATCAGCAGCGTCTCATCTTCGCCGGAAAGCAGCTCGAGGACGGCCGCACCCTGTCTGACTACAACATCCAGAAGGAATCTACTCTTCACTTGGTTCTTCGTCTTAGAGGAGGAATGCAGATCTTTGTCAAGACTTTGACTGGAAAGACCATCACACTTGAAGTTGAAGCTTCCGACACGATCGAGAACGTCAAGGCCAAGATTCAAGACAAGGAGGGAATC
Cele.WB235	Cele.WB235_3:6013025	3	6013025	6013087	63	4	15	CGAAAAGGGG	CGAAAAGGGGATCTG
Cele.WB235	Cele.WB235_3:6013262	3	6013262	6013339	78	5	15	TCCCCTTTTC	TCCCCTTTTCGTATC
Cele.WB235	Cele.WB235_3:6185212	3	6185212	6186090	879	42	19	AAAAATAGAT	AAAAATAGATTGTTACACG
Cele.WB235	Cele.WB235_3:6551383	3	6551383	6551520	138	9	15	CGAAAAGGGG	CGAAAAGGGGATCTG
Cele.WB235	Cele.WB235_3:6551695	3	6551695	6551771	77	5	15	TCCCCTTTTC	TCCCCTTTTCGTATC
Cele.WB235	Cele.WB235_3:6626010	3	6626010	6626226	217	6	21	AAATCTAATC	AAATCTAATCCAATATGGCCG
Cele.WB235	Cele.WB235_3:7405401	3	7405401	7443206	37806	109	94	CAATATTTAG	CAATATTTAGAGAACAGAAGATTTTGAGAAATACTGCCTCCAGAAATTGATGATTTTCCCATTGATTTGTCTACATAAGGCATCGAAAAGCACC
Cele.WB235	Cele.WB235_3:7444445	3	7444445	7448204	3760	143	27	ACAGAGAGTA	ACAGAGAGTAAAAAATAGTGGGAAGCC
Cele.WB235	Cele.WB235_3:7448277	3	7448277	7452238	3962	40	94	ACCCAATATT	ACCCAATATTTAGAGAAGAGAAGATTTTGAGAAATACTGCCTCCAGAAATTGATGATTTTCCCATTGATTTGTCTACATAGGGCATCGAAAAGT
Cele.WB235	Cele.WB235_3:7452275	3	7452275	7453348	1074	91	11	TGGAGCGGTT	TGGAGCGGTTT
Cele.WB235	Cele.WB235_3:7593704	3	7593704	7593825	122	11	11	CGGATAATTA	CGGATAATTAA
Cele.WB235	Cele.WB235_3:7594698	3	7594698	7597267	2570	98	26	ATTAGATGAA	ATTAGATGAAATTCCAAAATTAGACC
Cele.WB235	Cele.WB235_3:7602309	3	7602309	7602577	269	6	45	TCTGATCCTT	TCTGATCCTTCAGCTCCGAAGGATCACATGGGTACCTTCTGATGT
Cele.WB235	Cele.WB235_3:7602873	3	7602873	7603364	492	11	45	GAAGGATCAG	GAAGGATCAGAACATCAGGAGGTACCCATGTGATCATTCGGCTCT
Cele.WB235	Cele.WB235_3:7668665	3	7668665	7669197	533	28	19	AATCTATTTT	AATCTATTTTTCGTGTAAC
Cele.WB235	Cele.WB235_3:7678163	3	7678163	7678429	267	14	19	CAATCTATTT	CAATCTATTTTTCGTGTAA

Cele.WB235	Cele.WB235_3:7708654	3	7708654	7708716	63	4	15
CGAAAAGGGG	CGAAAAGGGGATCTG						
Cele.WB235	Cele.WB235_3:7708901	3	7708901	7708993	93	6	15
TCCCCTTTTC	TCCCCTTTTCGTATC						
Cele.WB235	Cele.WB235_3:8254497	3	8254497	8254602	106	5	21
AAGTTTTACT	AAGTTTTACTTTGATTTTTTT						
Cele.WB235	Cele.WB235_3:8334779	3	8334779	8334979	201	12	16
AGGCACGTAG	AGGAACGTAGGCACGT						
Cele.WB235	Cele.WB235_3:8335590	3	8335590	8336085	496	32	15
ACCCATCGTG	ACCCATCGTGGCGAG						
Cele.WB235	Cele.WB235_3:8336113	3	8336113	8336287	175	5	35
GGGAAATTTA	GGGAAATTTAAATTTTCTGTGAAAAATATTTTGGA						
Cele.WB235	Cele.WB235_3:8344363	3	8344363	8344643	281	7	40
TCTGGAATGT	TCTGGAATGTTCCAGAACTTTCTAGAAAAATTGGGAAAAG						
Cele.WB235	Cele.WB235_3:8897677	3	8897677	8897798	122	11	11
ATTTGGAATA	ATTTGGAATAT						
Cele.WB235	Cele.WB235_3:8958876	3	8958876	8960427	1552	21	59
TTCAAAACTT	TTCAAAACTTTTTAAACTTAGATTTATGGAGAATATGGAATTTCAAATGTGCACTGGTT						
Cele.WB235	Cele.WB235_3:9533702	3	9533702	9534094	393	13	11
GTACTGTAGG	GTACTGTAGGG						
Cele.WB235	Cele.WB235_3:9534180	3	9534180	9534500	321	8	40
TCCAGAACCT	TCCAGAACCTTCTGGAAAATCCGAGAAAATTCTGGAATGT						
Cele.WB235	Cele.WB235_3:9534215	3	9534215	9534836	622	9	40
AATGTTCCAG	AATGTTCCAGAACCTTCTGGAAAATTTGAGAAAATGCTGG						
Cele.WB235	Cele.WB235_3:9853874	3	9853874	9853997	124	4	31
TCTGCTAAAT	TCTGCTAAATAATTAGCAGACCATACTTGAT						
Cele.WB235	Cele.WB235_3:9863897	3	9863897	9863967	71	4	19
TGGTTGAGGC	TGGTTGAGGCTCTAGATTT						
Cele.WB235	Cele.WB235_3:9869882	3	9869882	9869977	96	5	19
ATTTTGGTTG	ATTTTGGTTGAGGTTGAGG						
Cele.WB235	Cele.WB235_3:9940544	3	9940544	9940665	122	6	20
TGACATACCA	TGACATACCAAAAAATAGAT						
Cele.WB235	Cele.WB235_3:9958568	3	9958568	9958766	199	4	31
TATGCTAAAT	TATGCTAAATATTTAGCAGACCCAACGTTGG						
Cele.WB235	Cele.WB235_3:10123441	3	10123441	10123519	79	5	15
CGAAAAGGGG	CGAAAAGGGGATCTC						
Cele.WB235	Cele.WB235_3:10123694	3	10123694	10123786	93	5	15
TCCCCTTTTC	TCCCCTTTTCGCAGA						
Cele.WB235	Cele.WB235_3:10224644	3	10224644	10226007	1364	23	59
AAATGTGCAC	AAATGTGCACTGGTTTTCAAAATTTTTTTAACTAAGAATTATGGAAAAAATGGAAATTT						
Cele.WB235	Cele.WB235_3:10226211	3	10226211	10227863	1653	11	164
TATGGGAAAA	TATGGGAAAATTATATGGAGTCATTCTTTTTTATTGCATAAAACTGTTCAGCATAGTCAAAAATACCAGAATATGCCAATCACAGTATAATAGCTTATACGGAAGTATTTTTTTAAAAATTGATAAAAAATAT ATAAAAGCTGATTTTTTCAAAAATTCAAAAG						
Cele.WB235	Cele.WB235_3:10316800	3	10316800	10317870	1071	27	40
AAAAGTAGGT	AAAAGTAGGTCATGACCTAGTTTTTCATGTGACCTACTTC						
Cele.WB235	Cele.WB235_3:10436267	3	10436267	10436329	63	4	15
CGAAAAGGGG	CGAAAAGGGGATCTG						
Cele.WB235	Cele.WB235_3:10436489	3	10436489	10436581	93	6	15
TCCCCTTTTC	TCCCCTTTTCGTATC						
Cele.WB235	Cele.WB235_3:10480990	3	10480990	10481350	361	14	16
TAGGCACGTA	TAGGCACGTAGGCACA						

Cele.WB235	Cele.WB235_3:10489911	3	10489911	10490082	172	5	34
TTTCCGGCAA	TTTCCGGCAAATCGGCGAATTGCCGGAATTGAAT						
Cele.WB235	Cele.WB235_3:10636973	3	10636973	10637342	370	11	29
GGCCTAGAAA	GGCCTAGAAAACCCATTTTTAAAGTTGGT						
Cele.WB235	Cele.WB235_3:10661890	3	10661890	10662218	329	9	35
CAATTTGCCG	CAATTTGCCGATTTGCCTGAAATTATCATATCCGG						
Cele.WB235	Cele.WB235_3:10678429	3	10678429	10678847	419	11	35
CCGCCAAAAT	CCGCCAAAATATTTTTCACTAAAAATTTGAATTTC						
Cele.WB235	Cele.WB235_3:10683224	3	10683224	10684272	1049	30	35
AAATTCAAAT	AAATTCAAATTTTTAGTGAAAAATATTTTGGCGGA						
Cele.WB235	Cele.WB235_3:10792337	3	10792337	10792694	358	8	45
TCTGATCCTT	TCTGATCCTTCAGCTCCGAAGGATCACATGGGTACCTTCTGATGT						
Cele.WB235	Cele.WB235_3:10796467	3	10796467	10796566	100	5	20
GTTACACGAA	GTTACACGAAAAAAATAGATT						
Cele.WB235	Cele.WB235_3:10811336	3	10811336	10811411	76	5	15
TCCCCTTTTC	TCCCCTTTTCATATC						
Cele.WB235	Cele.WB235_3:10924768	3	10924768	10924858	91	6	12
GGCTTAGGCT	GGCTTAGGCTTA						
Cele.WB235	Cele.WB235_3:10943916	3	10943916	10944702	787	15	37
TTGGCGGGAA	TTGGCAGGAATTCAAATTTTAATTTTTTTAAAATATT						
Cele.WB235	Cele.WB235_3:10975223	3	10975223	10975307	85	5	12
GGCTTAGGCT	GGCTTAAGCTCA						
Cele.WB235	Cele.WB235_3:11002899	3	11002899	11003053	155	5	31
GCAGCCGACG	GCAGCCGACGATATACGCGTTCGCTGGAGTA						
Cele.WB235	Cele.WB235_3:11004687	3	11004687	11006367	1681	84	20
TCTATTTTTT	TCTATTTTTTGGTATGCCAA						
Cele.WB235	Cele.WB235_3:11027127	3	11027127	11027171	45	4	11
AAACTTGGGC	AAACTTGGGCA						
Cele.WB235	Cele.WB235_3:11152069	3	11152069	11152305	237	6	35
AATTGCCGGA	AATTGCCGGAATTAAAGATTTCCGGCAAATTGGCC						
Cele.WB235	Cele.WB235_3:11160584	3	11160584	11160784	201	5	40
TCCAGAACCT	TCCAGAACCTTCTGGAAAATTTGAGAAAATGCTGGAATGT						
Cele.WB235	Cele.WB235_3:11160967	3	11160967	11161287	321	10	32
TTTCTGTGAA	TTTCTGTGAAAAAATTATAACGGGAATTCAAAA						
Cele.WB235	Cele.WB235_3:11189285	3	11189285	11189691	407	12	31
TCTGAGCTAC	TCTGAGCTACAGTACTCCTGTCAAGAACAGT						
Cele.WB235	Cele.WB235_3:11207917	3	11207917	11208073	157	10	12
GCTTAGGCTT	GCCTAGGCTTAG						
Cele.WB235	Cele.WB235_3:11221697	3	11221697	11221865	169	11	14
GATAATTGAA	GATAATTGAAAATC						
Cele.WB235	Cele.WB235_3:11242813	3	11242813	11243253	441	21	21
GGCAATTTTC	GGCAATTTTCCAGTGCAATTC						
Cele.WB235	Cele.WB235_3:11311767	3	11311767	11311976	210	6	35
GATTTGCCGG	GATTTGCCGGAAATATTCAAATCCGGCAAATTGCA						
Cele.WB235	Cele.WB235_3:11314293	3	11314293	11314573	281	6	35
TTGCCGGATT	TTGCCGGATTGTAATATTTTCGGCAAATCGGTAAA						
Cele.WB235	Cele.WB235_3:11331372	3	11331372	11331497	126	4	11
CTACAGTACT	CTACAGTACTT						
Cele.WB235	Cele.WB235_3:11399826	3	11399826	11400260	435	31	14
AATTATAAAT	AATTATAAATTATA						
Cele.WB235	Cele.WB235_3:11422288	3	11422288	11422568	281	6	35
TTTGCCGGAA	TTTGCCGGAAATTTAGATTTTTGGCAAATTGCCGA						

Cele.WB235 Cele.WB235_3:11424870 3 11424870 11425146 277 12 12
TTAGGCTTAG TTAGGCTTAGGC
Cele.WB235 Cele.WB235_3:11475036 3 11475036 11475156 121 10 12
CTTAGGCTTA CTTAGACTTATG
Cele.WB235 Cele.WB235_3:11475344 3 11475344 11475488 145 7 16
TACAAGCCTA TACACGCCTACAAGCC
Cele.WB235 Cele.WB235_3:11495534 3 11495534 11496085 552 15 34
AAATTTCAAT AAATTTCAATTCCGCTAATTTGCCGATTTGCCGG
Cele.WB235 Cele.WB235_3:11546762 3 11546762 11547752 991 24 35
CGGCAAATCG CGGCAAATCGGTAAATTGCCGAAACTGAATATTTC
Cele.WB235 Cele.WB235_3:11593439 3 11593439 11602256 8818 48 184
TACGCAGCCG
TACGCAGCCGAACGTATCATAAGTGAATACAGAACACCAATTATGTCCGAGAAAAAGATCCTACTCAGAATATGAACATATTCAAAAATATCGGAGGTATATAGATTCCCGAAGAGCCTTTCCAATTAACCAA
ACGGTTCAAATTCTAGCAAACATCTCCTTACTTGAACACTCTTGGCCAATG
Cele.WB235 Cele.WB235_3:11687193 3 11687193 11687714 522 14 35
CCGATTTGCC CCGATTTGCCGGAAATTTTCAAATCCGGCAATTTT
Cele.WB235 Cele.WB235_3:11700371 3 11700371 11700706 336 7 11
GTACTGTAGA GTACTGTAGAG
Cele.WB235 Cele.WB235_3:11707265 3 11707265 11707854 590 30 12
GGCTTAGGCT GGCTTAGGTATA
Cele.WB235 Cele.WB235_3:11722253 3 11722253 11722390 138 8 12
TAAGCCTAAG TAAGCCTAAACC
Cele.WB235 Cele.WB235_3:11737191 3 11737191 11737281 91 6 12
CCTAAGCCTA CCTAACCCTAAC
Cele.WB235 Cele.WB235_3:11783258 3 11783258 11783348 91 4 12
AGCCTGAGCC AGCCTGAGCCTC
Cele.WB235 Cele.WB235_3:11784840 3 11784840 11785090 251 9 12
TAAGCCTAAG TAAGCCTAAGCC
Cele.WB235 Cele.WB235_3:11798467 3 11798467 11798684 218 6 34
TTGCCGAATT TTGCCGAATTTTCCGTTTGCCGAGCTCGTCAAAC
Cele.WB235 Cele.WB235_3:11817185 3 11817185 11817612 428 12 35
AATTGCCGGA AATTGCCGGAAACAAAAACTTCCGGCAAATCGGCA
Cele.WB235 Cele.WB235_3:11862696 3 11862696 11862861 166 15 11
ACATTTTTTG ACATTTTTTGC
Cele.WB235 Cele.WB235_3:11864487 3 11864487 11864917 431 13 34
AAGTATTTTT AAGTATTTTTGCCGGTTTTTTTGCCGAAATTTCC
Cele.WB235 Cele.WB235_3:11924167 3 11924167 11924393 227 6 35
TTCCGGCAAA TTCCGGCAAATTGGCAAATTGCTGGAAATTAAAAT
Cele.WB235 Cele.WB235_3:11925619 3 11925619 11926677 1059 29 35
TTCAATTCCG TTCAATTCCGGCAATTTGCCGATTTGCCAGAAATT
Cele.WB235 Cele.WB235_3:12001471 3 12001471 12001873 403 10 40
ATTCCAGAAT ATTCCAGAATTTTTTCGAATTTTCCAGAAAGTTCTAGAAC
Cele.WB235 Cele.WB235_3:12004285 3 12004285 12004750 466 12 35
AAAATTTCCG AAAATTTCCGGCAAATCAGCTAATTGCCAGAATTG
Cele.WB235 Cele.WB235_3:12005666 3 12005666 12006008 343 8 35
GGAATTGAAA GGAATTGAAAACTTCCGGGAAATCGGCAAATTGGC
Cele.WB235 Cele.WB235_3:12009165 3 12009165 12009381 217 11 20
AAATAGATTG AAATAGATTGACAGACGGAA
Cele.WB235 Cele.WB235_3:12017198 3 12017198 12017750 553 10 43
AATTGCCGGT AATTGCCGGTTTGCCGATTTGCCAGAAATTTCGGTTTTCGGCA
Cele.WB235 Cele.WB235_3:12072321 3 12072321 12073804 1484 45 32
AAATTTGTGA AAATTTATGAAAATTTAGGCCCATTTTGCCCA

Cele.WB235	Cele.WB235_3:12130507	3	12130507	12130633	127	9	12
TAAGCCTAAG	TAAGTCTAAGCC						
Cele.WB235	Cele.WB235_3:12141791	3	12141791	12142267	477	10	32
ATTCAAATTT	ATTCAATTTTTACAGATACATTTTGGCGAAA						
Cele.WB235	Cele.WB235_3:12143931	3	12143931	12144015	85	7	12
GGCTTAGGCT	GGCTTAGGCTTA						
Cele.WB235	Cele.WB235_3:12172469	3	12172469	12173356	888	63	11
TTTTTGCACA	TTTTTGCACAT						
Cele.WB235	Cele.WB235_3:12188657	3	12188657	12188828	172	7	19
CAATTTACCA	CAATTTACCAAAAATTTGT						
Cele.WB235	Cele.WB235_3:12190449	3	12190449	12190847	399	13	16
ACGTAGGCAC	ACGCAGGCACGTAGGC						
Cele.WB235	Cele.WB235_3:12196315	3	12196315	12196495	181	14	12
CTTAGACTTA	CTTAGGCTTAGG						
Cele.WB235	Cele.WB235_3:12239290	3	12239290	12239399	110	5	15
CGACCTGCGA	CGACCTGCGACCTAG						
Cele.WB235	Cele.WB235_3:12244502	3	12244502	12244747	246	7	35
AATTGCCGGA	AATTGCCGGAATTCAAAATTTCCGGCTAATCGGCA						
Cele.WB235	Cele.WB235_3:12289600	3	12289600	12291140	1541	44	35
TTGCCAGAAT	TTGCCAGAATTAAAAATTTCCGGAAAATCGGCAAG						
Cele.WB235	Cele.WB235_3:12302154	3	12302154	12302292	139	9	12
TAGGCATAGG	TAGGCATAGACT						
Cele.WB235	Cele.WB235_3:12305325	3	12305325	12305929	605	14	43
AGCAGCCGAC	AGCAGCTGACAGTCACCGGTTGCCGAAAAATTGCAATAATTTC						
Cele.WB235	Cele.WB235_3:12323417	3	12323417	12323485	69	4	17
TGAAAATAAT	TGAAAATAATCACGGAA						
Cele.WB235	Cele.WB235_3:12341199	3	12341199	12341722	524	7	35
TTGCCGGAAT	TTGGCGGAATTGGAAATTTCCGCCAAACCGGCAAA						
Cele.WB235	Cele.WB235_3:12345927	3	12345927	12348234	2308	63	35
CGGGAAAATT	CGGGAAAATTTAAATTTTTTGGAAACAAATTTTGG						
Cele.WB235	Cele.WB235_3:12389240	3	12389240	12389685	446	15	17
ATTTTTTCCG	ATTTTTCCGCCGGTTCG						
Cele.WB235	Cele.WB235_3:12397168	3	12397168	12397264	97	4	24
AAGCACCACC	AAGCACCACCTCCTCCAACTACAA						
Cele.WB235	Cele.WB235_3:12408999	3	12408999	12409471	473	10	35
GATTTGCCGG	GATTTGCCGGAAATTTTCGATTCCGGCAATTTGCC						
Cele.WB235	Cele.WB235_3:12480633	3	12480633	12480804	172	5	35
AATTTGAATT	AATTTGAATTTTCCGCCAAAATCCTTTTCTGAGAT						
Cele.WB235	Cele.WB235_3:12485043	3	12485043	12485356	314	8	35
TTCAAATTTT	TTCAAATTTTATGTGAAAGACATTTTGGCGGTAAA						
Cele.WB235	Cele.WB235_3:12513645	3	12513645	12515229	1585	44	36
GATATTTTCA	GATATTTTCATGTTCGGCAAAAATGCCGATTTGCCG						
Cele.WB235	Cele.WB235_3:12531237	3	12531237	12531912	676	18	35
TTTCCGGCAA	TTTCCGGCAAACCGGCAAATTGCTGGAACTGAGCA						
Cele.WB235	Cele.WB235_3:12532752	3	12532752	12533511	760	21	34
TTTCAATTCC	TTTCAATTCCGGCATTTTACCAATTTGCCGGAAT						
Cele.WB235	Cele.WB235_3:12538530	3	12538530	12538668	139	11	12
TATACATATA	TATACATATACA						
Cele.WB235	Cele.WB235_3:12581020	3	12581020	12581289	270	5	43
GGCAAATCGG	GGCAAATCGGCAAACCGGCAATTTGTCAAATATTCAAATTTCC						
Cele.WB235	Cele.WB235_3:12584489	3	12584489	12584865	377	11	34
TTTCAATTCC	TTTCAATTCCGGCAAATTGGCGATTTACCGGAAA						

Cele.WB235	Cele.WB235_3:12629696	3	12629696	12629957	262	7	35
TAATTCCGGC	TAATTCCGGCAATTTACCGGTTTGCCGGAAAATTT						
Cele.WB235	Cele.WB235_3:12636128	3	12636128	12637282	1155	29	35
GGCAAACCGG	GGCAAACCGGCATGTTTCCGGAATTAACAATTCCC						
Cele.WB235	Cele.WB235_3:12692942	3	12692942	12693363	422	8	35
GGAAATTTTA	GGAAATTTTAAATTCCGGCAATTTGCCGGTTAGCC						
Cele.WB235	Cele.WB235_3:12696947	3	12696947	12698148	1202	5	32
GACCAATCAG	GACCAATCAGCGATTCGCCCCGCCCACTCTCT						
Cele.WB235	Cele.WB235_3:12698391	3	12698391	12698580	190	5	38
TTTTGGCGGG	TTTTGGCGGGAATTCAAATTTTAATTTTTTTGAAAATA						
Cele.WB235	Cele.WB235_3:12722182	3	12722182	12722605	424	13	35
AAATTTTGAT	AAATTTTGATTTTTGGCAATTATCCGATTTGCCGG						
Cele.WB235	Cele.WB235_3:12731873	3	12731873	12732374	502	16	35
ATTTGCCGGA	ATTTGCCGGAACATTTGATTTTCTGGAATATACCG						
Cele.WB235	Cele.WB235_3:12740278	3	12740278	12740831	554	5	32
ACCAATCAGC	ACCAATCAGCGATTTGCTCCGCCCACTTTTGA						
Cele.WB235	Cele.WB235_3:12762055	3	12762055	12762649	595	17	35
AAATTCAAAT	AAATTCAAATTTTCTGATAAATATTTTATGGCGGT						
Cele.WB235	Cele.WB235_3:12817385	3	12817385	12817569	185	5	31
GATTTTTTCA	GATTTTTTCAGGGAAAAACCAAAAAAATTC						
Cele.WB235	Cele.WB235_3:12823844	3	12823844	12823984	141	4	35
CGGCAATTTG	CGGCAATTTGCCAAAAATGAAAATTTCCGGCAAAT						
Cele.WB235	Cele.WB235_3:12835769	3	12835769	12836969	1201	30	20
TGTAGATTTA	TGTAGATTTACGAAATTCTA						
Cele.WB235	Cele.WB235_3:12839625	3	12839625	12840128	504	16	19
TTGGCAATTT	TTGGCATTTGACCAAAAAT						
Cele.WB235	Cele.WB235_3:12878018	3	12878018	12878110	93	5	15
CGAAAAGGGG	CGAAAAGGGGAGATA						
Cele.WB235	Cele.WB235_3:12878285	3	12878285	12878362	78	5	15
TCCCCTTTTC	TCCCCTTTTCGTATC						
Cele.WB235	Cele.WB235_3:12884539	3	12884539	12885107	569	13	35
TGCCGGAATT	TGCCGGAATTTAGAATTTTCGACAAAGTGGCAAAT						
Cele.WB235	Cele.WB235_3:12944421	3	12944421	12944571	151	9	12
TAAGCCTAAG	TGAGCCTAAGCA						
Cele.WB235	Cele.WB235_3:12985357	3	12985357	12986137	781	10	78
CGAAGTTGCT	CGAAGTTGCTGGATCAACGATCACAACAGGCGGATTTGTCTGCACAACCGGATCAGAAGTCGTCGTACTTGGATCTTT						
Cele.WB235	Cele.WB235_3:12987113	3	12987113	12987245	133	7	12
CGCGACACGC	CGCGACACGCAA						
Cele.WB235	Cele.WB235_3:13032223	3	13032223	13032864	642	39	10
TACCGCCCGC	TACCGCCCGC						
Cele.WB235	Cele.WB235_3:13074772	3	13074772	13086747	11976	170	59
TCTCCATAAT	TCTCCATAATTCTCAGTTTAAAAAAATTTGAACATCAATACACATTTGAAATTCCATAT						
Cele.WB235	Cele.WB235_3:13094103	3	13094103	13095343	1241	25	43
CGGAAATTTT	CGGAAATTTTGATTTTCGGTAATTTGCCGGTTTACTGGTTTGC						
Cele.WB235	Cele.WB235_3:13095846	3	13095846	13096595	750	22	34
AAATCGGCAA	AAATCGGCAAATTGCCGGAATTAAAATTTTCGGC						
Cele.WB235	Cele.WB235_3:13099929	3	13099929	13100574	646	18	34
CGGAATTGAA	CGGAATTGAAATTTCCGGCAAATCGACAACTTGC						
Cele.WB235	Cele.WB235_3:13116308	3	13116308	13116374	67	5	11
AAAAATGTGC	AAAAATGTGCA						
Cele.WB235	Cele.WB235_3:13129352	3	13129352	13129936	585	45	12
AGTTATATAG	AGTTATATAGTA						

Cele.WB235	Cele.WB235_3:13167617	3	13167617	13167762	146	5	31
CCAAAAATGA	CCAAAAATGACTGAAATTAGTCAAAAATTGG						
Cele.WB235	Cele.WB235_3:13243432	3	13243432	13245239	1808	95	19
AAAAATAGAT	AAAAATAGATTGGCATACG						
Cele.WB235	Cele.WB235_3:13268025	3	13268025	13268308	284	8	30
TTTCAGGCCA	TTTCAGGCCAATTTCGGTCAATTTTGGACA						
Cele.WB235	Cele.WB235_3:13447046	3	13447046	13447886	841	21	40
ATTTTTCTAG	ATTTTTCTAGAAAGTTCTGGAACATTCCAGAATTTTCCCA						
Cele.WB235	Cele.WB235_3:13447979	3	13447979	13448227	249	7	35
AATTTCCCGC	AATTTCCCGCCAAAATATTTTTCTCAGAAAATTTC						
Cele.WB235	Cele.WB235_3:13448299	3	13448299	13448524	226	15	15
CCACGATGGG	CCACGATGGGTCTCA						
Cele.WB235	Cele.WB235_3:13583331	3	13583331	13583502	172	7	24
AAGAAGGATG	AAGAAAGATGCCGAAGAGAAGAAG						
Cele.WB235	Cele.WB235_3:13750231	3	13750231	13751043	813	52	15
GTGGTGAGAC	GTGGTGAGACCCTTC						
Cele.WB235	Cele.WB235_3:13753764	3	13753764	13754664	901	60	15
TCGTGGTGAG	TCGTGGTGAGACCCT						
Cele.WB235	Cele.WB235_3:13783590	3	13783590	13783788	199	16	12
CTTAGGCTTA	CTTAGGCTTAGG						
Cele.WB235	Cele.WB235_4:0	4	0	137	138	11	12
CCTAAGCCTA	CCTAAGCCTAAG						
Cele.WB235	Cele.WB235_4:119859	4	119859	120107	249	13	19
TGGTTGAGGC	TGGTTGAGGCTCACAATTT						
Cele.WB235	Cele.WB235_4:122695	4	122695	123566	872	28	19
TTGGTTGAGG	TTGGTTGAGGCTCACAATT						
Cele.WB235	Cele.WB235_4:124390	4	124390	125403	1014	21	22
TTGGTTGAGG	TTGGTTGAGGCTCTCTGGATTT						
Cele.WB235	Cele.WB235_4:154583	4	154583	154709	127	8	14
CCTAAGCCTA	CCTAAGCCTAAGGT						
Cele.WB235	Cele.WB235_4:180606	4	180606	180739	134	7	19
CCTCAACCAA	CCTCAACCAAAATTCTGAG						
Cele.WB235	Cele.WB235_4:195511	4	195511	196615	1105	53	20
CTCAACCAAA	CTCAACCAAAAATCCTTAGC						
Cele.WB235	Cele.WB235_4:215697	4	215697	216357	661	32	20
CTCAGCCTCA	CTCAGCCTCAATCAAAAATT						
Cele.WB235	Cele.WB235_4:229906	4	229906	231132	1227	63	18
CTCAACCAAA	CTCAACCAAACTCTTAGC						
Cele.WB235	Cele.WB235_4:236164	4	236164	236448	285	12	22
TGAGGCTCTC	TGAGGCTCTCTAGATTTTTGGT						
Cele.WB235	Cele.WB235_4:238538	4	238538	239383	846	8	18
AGCCTCAACC	AGCCTCAACCAAATTCTT						
Cele.WB235	Cele.WB235_4:280698	4	280698	281177	480	13	37
TTGGCGGGAA	TTGGCGGGAATTCAAAATTTAATTTTTTGAACATATT						
Cele.WB235	Cele.WB235_4:281265	4	281265	281745	481	32	15
TCGTGGTGAG	TCGTGGAGAGACCCT						
Cele.WB235	Cele.WB235_4:349715	4	349715	350206	492	11	45
GAAGGATCAG	GAAGGATCAGAACATCAGGAGGTACCCATGTGATCATTCGGCTCT						
Cele.WB235	Cele.WB235_4:382116	4	382116	382388	273	8	34
TTGCAGGAAA	TTGCAGGAAATTTCAATTACCGCAATTTGCCGGT						
Cele.WB235	Cele.WB235_4:385463	4	385463	385525	63	4	15
TCCCCTTTTC	TCCCCTTTTCGTATC						

Cele.WB235	Cele.WB235_4:545946	4	545946	546072	127	9	12	TAGGCTTAGG	TAGGCTTAGGTT
Cele.WB235	Cele.WB235_4:594389	4	594389	594607	219	10	16	AGGCATGTAG	AGGCATGTAGGCATGT
Cele.WB235	Cele.WB235_4:628375	4	628375	628467	93	6	15	TCCCCTTTTC	TCCCCTTTTCGTTGC
Cele.WB235	Cele.WB235_4:640232	4	640232	640313	82	4	15	TAAGCCTAAG	TAAGCCTAAGCCCAC
Cele.WB235	Cele.WB235_4:688812	4	688812	698192	9381	92	102	TTGCTGCAGC	TTGCTGCAGCATATCTTTCTCCAGTACCACCTTGATATGTCGAACGATTGATAGTTTGAGAATCAAGAGAATCAGTTGGACGATCAATTCCATCTTTGGGTC
Cele.WB235	Cele.WB235_4:775288	4	775288	775412	125	4	31	CAAAAATCGG	CAAAAATCGGGAAATTTTCACAAAAAAATGT
Cele.WB235	Cele.WB235_4:977829	4	977829	978532	704	35	19	CAATAATTTC	CAATAATTTCCCAAAAAAT
Cele.WB235	Cele.WB235_4:1069432	4	1069432	1069486	55	4	12	AGCCTAAGCC	AGCCTAAGCCTA
Cele.WB235	Cele.WB235_4:1071381	4	1071381	1071429	49	4	12	AGGCTTAGGC	AGGCTTAGGCTT
Cele.WB235	Cele.WB235_4:1153760	4	1153760	1154039	280	7	40	ATTTTTCTAG	ATTTTTCTAGAAAGTTCTGGAACATTCCAGACTTTTCCCA
Cele.WB235	Cele.WB235_4:1153661	4	1153661	1154193	533	4	35	AAAATATTTT	AAAATATTTTTCACAGAAAATTTAAATTTCCCTCC
Cele.WB235	Cele.WB235_4:1154252	4	1154252	1154492	241	16	15	GGTCTCACCA	GGTCTCACCACGATG
Cele.WB235	Cele.WB235_4:1153773	4	1153773	1154667	895	10	40	GTTCTGGAAC	GTTCTGGAACATTCCAGACTTTTCCCAATTTTTCTAGAAA
Cele.WB235	Cele.WB235_4:1154757	4	1154757	1155014	258	8	11	GTACTCCTAC	GTAATCCTACC
Cele.WB235	Cele.WB235_4:1295080	4	1295080	1295440	361	9	36	TTATTTTCTG	TTATTTTCTGACAAATTGCCGGTTTGCCGGAAATTT
Cele.WB235	Cele.WB235_4:1299931	4	1299931	1300254	324	8	36	TTTGCCGGAA	TTTGTCGGAAATTTTTATTTTCTGACAAATTGCCGG
Cele.WB235	Cele.WB235_4:1344791	4	1344791	1345475	685	20	34	ATTTCCCGCC	ATTTCCCGCCAAAATTTTTTCACTGAAAATTTGA
Cele.WB235	Cele.WB235_4:1345560	4	1345560	1345725	166	11	15	CCACGATGGG	CCACGATGGGTCTCG
Cele.WB235	Cele.WB235_4:1348897	4	1348897	1349230	334	9	34	ATTGCCGGAA	ATTGCCGGAATTCAAATTCCTAGCAAATCGGCAA
Cele.WB235	Cele.WB235_4:1355992	4	1355992	1356408	417	8	12	TAGGCTTAGG	TAGGCTTAGGCG
Cele.WB235	Cele.WB235_4:1415278	4	1415278	1415575	298	15	21	AAAATTGACT	AAAATCGACTAAAAATCGTCA
Cele.WB235	Cele.WB235_4:1429990	4	1429990	1430044	55	4	12	CATAGGCATA	CATAGGCATAGA
Cele.WB235	Cele.WB235_4:1438688	4	1438688	1438742	55	4	12	TATATGTATA	TATATGTATATG
Cele.WB235	Cele.WB235_4:1440391	4	1440391	1440556	166	11	15	AGACCCATCG	AGACCCATCGTGGTG
Cele.WB235	Cele.WB235_4:1447227	4	1447227	1447319	93	6	15	TCCCCTTTTC	TCCCCTTTTCGTATC
Cele.WB235	Cele.WB235_4:1476030	4	1476030	1477123	1094	29	35	AAAAAATTTT	AAAAAATTTTGGCGGGAAATTCAAATTTTCAGTGA

Cele.WB235	Cele.WB235_4:1477245	4	1477245	1477418	174	5	34
GAAAATTTGA	GAAAATTTGAATTTCCCGCCAAAATTTTTTCACT						
Cele.WB235	Cele.WB235_4:1477450	4	1477450	1477555	106	7	15
CTCACCACGA	CTCACCACGATGGGT						
Cele.WB235	Cele.WB235_4:1494116	4	1494116	1494472	357	5	12
AGGCTTAGGC	AGGCTTAGGCCT						
Cele.WB235	Cele.WB235_4:1515989	4	1515989	1516221	233	8	29
CCACCCGGGA	CCACCCGGGAGGCGTCGGCTGCTCTAAGC						
Cele.WB235	Cele.WB235_4:1543828	4	1543828	1544203	376	4	94
TCCCATTGAT	TCCCATTGATTTGTCTACAAAGGGCATCGAAAAGCACCCAATATTTGGAGAACAGAAGATTTTGAGAATTACTGCCTTCAGAAATGGACAATTT						
Cele.WB235	Cele.WB235_4:1565274	4	1565274	1565421	148	7	21
CCATAGGTGG	CCATAGGTGGAAGATCTCATG						
Cele.WB235	Cele.WB235_4:1569894	4	1569894	1570719	826	7	101
TTGATTTTTC	TTGATTTTTCGTCTTTTGAAGGGATATTGGTTTCGGGGTAGTGGGCGGATATGGTCGGGGTACTGTAGGTATACGGTAGGGTTACTGTAGTTTTGGAAATT						
Cele.WB235	Cele.WB235_4:1624502	4	1624502	1624593	92	6	14
ACTACACACT	ACTACACACTACAC						
Cele.WB235	Cele.WB235_4:1628395	4	1628395	1628484	90	6	12
TTAGGCTTAG	TTAGGCTTCGGC						
Cele.WB235	Cele.WB235_4:1632727	4	1632727	1632835	109	9	12
CTTAAGCTTA	CTTAAGCTTAGG						
Cele.WB235	Cele.WB235_4:1639384	4	1639384	1639487	104	5	23
ATTTAATTAA	ATTTATTTAATTAATTAATATTT						
Cele.WB235	Cele.WB235_4:1654913	4	1654913	1655123	211	6	35
ATTTCCGGCA	ATTTCCGGCAAATCGGCAATATGCCAAAAATGGAT						
Cele.WB235	Cele.WB235_4:1681168	4	1681168	1681390	223	6	37
CGCCAAAATG	CGCCAAAATGATTCCAAAAAATTAAAATTTGAATTCC						
Cele.WB235	Cele.WB235_4:1689083	4	1689083	1689293	211	11	12
CTAAGCCTAA	CTAAGCCTAATA						
Cele.WB235	Cele.WB235_4:1715069	4	1715069	1715131	63	4	15
CGAAAAGGGG	CGAAAAGGGGATCTG						
Cele.WB235	Cele.WB235_4:1740517	4	1740517	1740917	401	20	20
CAGAAAAATC	CAGAAAAATCAATAAATTCT						
Cele.WB235	Cele.WB235_4:1752199	4	1752199	1752289	91	5	12
TAAGCCTAAG	TAAGCCTAAGAC						
Cele.WB235	Cele.WB235_4:1762607	4	1762607	1762703	97	5	16
CACGTAGGCA	CACGTAAGCACGTAGG						
Cele.WB235	Cele.WB235_4:1785293	4	1785293	1785383	91	7	12
TTAGGATTAG	TTAGGATTAGGA						
Cele.WB235	Cele.WB235_4:1787801	4	1787801	1788241	441	26	16
TAGGCACGTA	TAGGTACGTAGGCACG						
Cele.WB235	Cele.WB235_4:1848441	4	1848441	1848937	497	18	21
TGTCGATTTA	TGTCGATTTACGGGGGTTCAG						
Cele.WB235	Cele.WB235_4:1890247	4	1890247	1890331	85	5	12
TAAGCCTAAG	TAAGTCTAAGCT						
Cele.WB235	Cele.WB235_4:1928447	4	1928447	1928762	316	8	35
GAAATTTTCA	GAAATTTTCAATTCCGGCAATTCGCCGATTTGACG						
Cele.WB235	Cele.WB235_4:1931275	4	1931275	1932180	906	26	35
CATTTTCGCC	CATTTTCGCCGAATTGCCGGTTTGCCGGAAATTTC						
Cele.WB235	Cele.WB235_4:1931275	4	1931275	1933389	2115	50	35
CATTTTCGCC	CATTTTCGCCGAATTGCCGGTTTGCCGGAAATTTC						
Cele.WB235	Cele.WB235_4:1949738	4	1949738	1952318	2581	113	21
GTAAATCTAC	GTAAATCTACAGTAATCCCTC						

Cele.WB235	Cele.WB235_4:1965986	4	1965986	1967712	1727	15	80
AATTTCTCAA	AATTTCTCAATAGCGCGCGCTTGCAACATCCGAACGGCGCGAAATTTGAATTTTTGGCCATAAAATAACGAAAATCGGCA						
Cele.WB235	Cele.WB235_4:2096897	4	2096897	2096972	76	5	15
GGGTCTCACC	GGGTCTCGCCACGAT						
Cele.WB235	Cele.WB235_4:2135958	4	2135958	2136208	251	6	40
TTCCAGAATT	TTCCAGAATTTTCTCGAATATTCCAGAAGCTTCTAGAACA						
Cele.WB235	Cele.WB235_4:2136323	4	2136323	2136466	144	5	11
CTACAGTACC	CTACAGTACTA						
Cele.WB235	Cele.WB235_4:2140998	4	2140998	2141533	536	8	43
TTGCCGGAAA	TTGCCGGAAATTTTCAATCGCGGCATTTTGCCGGTTTGCTCAT						
Cele.WB235	Cele.WB235_4:2156341	4	2156341	2157203	863	38	21
GATTTACGGG	GATTTACGGGATGAAAATGTG						
Cele.WB235	Cele.WB235_4:2182563	4	2182563	2182888	326	13	21
AAAATAGCTT	AAAATAGCTGAAAATTCACTA						
Cele.WB235	Cele.WB235_4:2244962	4	2244962	2245187	226	15	15
CGATGGGTCT	CGATGGGTCTCACCA						
Cele.WB235	Cele.WB235_4:2245441	4	2245441	2245572	132	4	11
GTACTCCTAC	GTAATCCTACC						
Cele.WB235	Cele.WB235_4:2393736	4	2393736	2396081	2346	23	70
AAAAATCGAT	AAAAATCGATGTTTTGAAGTTTTTCTCAATAGAGCGCACTTTCAACAGCGAGAAATTTGAATTTTCCACC						
Cele.WB235	Cele.WB235_4:2397301	4	2397301	2398030	730	7	104
CCTGCGAAAG	CCTGCGAAAGCAAATCTCTGGCCTGACGGAGGGCCAGCTAACTCTCTCCCTTAAGCCTTCGGGTGCTTCCTGGGTTATCGGTGAATTTTTGGGTCTGTCAGGGT						
Cele.WB235	Cele.WB235_4:2431055	4	2431055	2431771	717	5	144
TTCTAGGCCA	TTCTAGGCCACCAATTGGAAATCATCATAACTTGGCTTAAAATCCAGATTTTTGGAGTCTGGTACCTGCAACATGTTGCCCTGTAGTTTTTCTACAAGTTAGGATATATTAATTTTTGGTGGCCTAAAAGTCCAAATTATGGAT						
Cele.WB235	Cele.WB235_4:2573920	4	2573920	2576033	2114	13	164
CTTTTTTCTT	CTTTTTTCTTTTATAAAACTGTTTAGCATAGTCGAATATACCAGAAAATACCAAACAAAGTATGCTATCTTGTACGAAAGATTATTTTAAAAAATTCAGAAAATGCATCTAAAGCGGTTTTTTTTCAAAAATTCAAAAGTACCGGAAAATCATAAAAAGTGCTT						
Cele.WB235	Cele.WB235_4:2650676	4	2650676	2650942	267	11	19
CGCCAAGTAT	CGCCAAGTGTCCGAAAATA						
Cele.WB235	Cele.WB235_4:2675283	4	2675283	2676104	822	4	175
AACTTGTGAC	AACTTGTGACGGTGGGTATACATTTTATGTTTTTAAATTGTTCGAAAACCTTCATTTCGTCAATTGGTGAAGTTTCAAAAACTTTGTGAATCTAGTTTTTGAAAAAATTTAAAATTTGTGGCAATTTCACCGATTTTTCGTGAATTTCCGAGCTAAAATTGAAAAATTCATTGTG						
Cele.WB235	Cele.WB235_4:2694935	4	2694935	2695125	191	5	40
GAATTTTCCA	GAATTTTCCAGAAAGTTCTAGAAAATTCCAGAATTTTTTC						
Cele.WB235	Cele.WB235_4:2702010	4	2702010	2702785	776	31	21
TTTGGATTTT	TTTGGATTTTTAGGCTTTAAA						
Cele.WB235	Cele.WB235_4:2754343	4	2754343	2754722	380	12	11
TACTGTAGGA	TACTGTAGGAG						
Cele.WB235	Cele.WB235_4:2754821	4	2754821	2756571	1751	49	40
TTCTGGAAAA	TTCTGGAAAATTCGAGAAAAATCTGGCATATTCCAGAACC						
Cele.WB235	Cele.WB235_4:2815904	4	2815904	2816246	343	4	87
TGAAGTCGTA	TGAAGTCGTAGGATTCTGTGTAGCGACTGGTGTCGTTGTACTCTGTGAAGGGGACGTGGCTTCTTCTGTTGAGTCAGGCTTCTGAGG						
Cele.WB235	Cele.WB235_4:2828548	4	2828548	2830536	1989	74	27
CAGAGAGTAA	CAGAGAGTAAAATAATCGGTGAAGCCA						
Cele.WB235	Cele.WB235_4:2851958	4	2851958	2852560	603	7	35
TTTTCAATTC	TTTTCAATTCAAGCAATTTGCAGTTTTGCCTGAAT						
Cele.WB235	Cele.WB235_4:2855122	4	2855122	2855338	217	5	24
GCTTCTGGAG	GCTTCTGGAGCCACAGGTTATCCA						

Cele.WB235	Cele.WB235_4:2861147	4	2861147	2861589	443	12	19
ACCTAGGCAT	ACCTATGCATAAGGCAGGC						
Cele.WB235	Cele.WB235_4:2872402	4	2872402	2872909	508	14	35
ATTCCGGCAA	ATTCCGGCAATTTGCCGATTTGCCGAAAATTTTCA						
Cele.WB235	Cele.WB235_4:2896505	4	2896505	2896560	56	5	11
TTGTTTACAA	TTGTTTACAAG						
Cele.WB235	Cele.WB235_4:2953071	4	2953071	2953413	343	19	16
TAGGCATGTA	TAGGCATGTAGGAATG						
Cele.WB235	Cele.WB235_4:2964807	4	2964807	2965022	216	4	43
TTTGCCGGAA	TTTGCCGGAATTGAAAACTACCGGCAAATCTGCAAACTGGAAA						
Cele.WB235	Cele.WB235_4:2967003	4	2967003	2967147	145	6	24
GCGGCCGACA	GCGGCCGACACCTTACGGGTTGTA						
Cele.WB235	Cele.WB235_4:2981693	4	2981693	2981773	81	4	16
AGGTAGGAAG	AGATAGGAAGGTAGGA						
Cele.WB235	Cele.WB235_4:3004801	4	3004801	3005510	710	20	34
CCGATTTGCC	CCGATTTGCCGAAATTTTTATTTCTGGCAAATTG						
Cele.WB235	Cele.WB235_4:3050503	4	3050503	3050595	93	6	15
CGAAAAGGGG	CGAAAAGGGGATCTG						
Cele.WB235	Cele.WB235_4:3050770	4	3050770	3050862	93	6	15
TCCCCTTTTC	TCCCCTTTTCGTATC						
Cele.WB235	Cele.WB235_4:3052982	4	3052982	3053132	151	8	15
CACGATGGGT	CACGATGGGTCTCAT						
Cele.WB235	Cele.WB235_4:3098171	4	3098171	3098319	149	4	37
TGGCGGGAAT	TGGCGGGAATTCAAATTTTAATTTTCCGAAAACATTT						
Cele.WB235	Cele.WB235_4:3149092	4	3149092	3151000	1909	53	35
GGCAAATTGC	GGCAAATTGCCGGAATTGAACATTTCCGGCAAATA						
Cele.WB235	Cele.WB235_4:3166479	4	3166479	3166602	124	7	12
CCTAAGCCTA	CCTAAGCCTAAA						
Cele.WB235	Cele.WB235_4:3184703	4	3184703	3185093	391	5	16
TACATGCCTA	TACATGCCTACATGCC						
Cele.WB235	Cele.WB235_4:3189159	4	3189159	3189343	185	11	16
CATGCCTACA	CATGCCTACACGCCTA						
Cele.WB235	Cele.WB235_4:3193096	4	3193096	3194818	1723	67	16
ATGCCTACAT	ATGCCTACATGCCTAC						
Cele.WB235	Cele.WB235_4:3195411	4	3195411	3195573	163	6	18
CCTTCGCCTA	CCTTCGCCTACCTGCATG						
Cele.WB235	Cele.WB235_4:3206393	4	3206393	3209585	3193	168	19
GCCTCACTCA	GCCTCACTCAAAATCCTCA						
Cele.WB235	Cele.WB235_4:3211749	4	3211749	3211845	97	6	16
AGGCATGTAG	AGGCATGTAGGCATGC						
Cele.WB235	Cele.WB235_4:3291067	4	3291067	3291207	141	5	33
CGGCATATGC	CGGCATATGCCGTGAATTTTAAAATCGGCATAA						
Cele.WB235	Cele.WB235_4:3309618	4	3309618	3310597	980	27	35
AATCGGCAAG	AATCGGCAAGTTACCGAAATTGAAAATTTCCGGCA						
Cele.WB235	Cele.WB235_4:3366659	4	3366659	3367988	1330	24	45
TCTGATCCTT	TCTGATCCTTCAGCTCCAAAGGATCAGAAGGGTACCTCCTGGTGA						
Cele.WB235	Cele.WB235_4:3455422	4	3455422	3455812	391	27	12
TAAGCCTAAG	TAAGCCTAAGCT						
Cele.WB235	Cele.WB235_4:3568013	4	3568013	3568493	481	28	16
TGCCTACATG	TGCCTACATGCCCACA						
Cele.WB235	Cele.WB235_4:3608664	4	3608664	3608778	115	7	12
TTAGGCTTAG	TTAGGATTAGGA						

Cele.WB235	Cele.WB235_4:3652033	4	3652033	3652933	901	15	21
TGTAGATTTA	TGTAGATATACGGGAATCTTG						
Cele.WB235	Cele.WB235_4:3670737	4	3670737	3670797	61	4	15
TCGTGGTGAG	TCGTGGTGAGACCCT						
Cele.WB235	Cele.WB235_4:3671203	4	3671203	3671723	521	15	40
TTTCTAGAAA	TTTCTAGAAAATTCGAGAAAATTCCGGATTGGTCCAGAAT						
Cele.WB235	Cele.WB235_4:3696437	4	3696437	3696683	247	6	41
AAATTTTTAA	AAATTTTTAACGAGAAGTGGGCGAAAATCATTCAAAAATTC						
Cele.WB235	Cele.WB235_4:3754942	4	3754942	3755101	160	5	21
TAGTTTGTAG	TAGTTTGTAGTGTGCAGTGTG						
Cele.WB235	Cele.WB235_4:3825814	4	3825814	3826008	195	12	16
TAGGCATGTA	TAGGCATGTAGGCATG						
Cele.WB235	Cele.WB235_4:3834124	4	3834124	3834445	322	5	18
ATTAGTATTG	ATTAGTATTGCCCTACCT						
Cele.WB235	Cele.WB235_4:3991189	4	3991189	3991890	702	6	12
TAAGCCTAAG	TAAGCCTATGCC						
Cele.WB235	Cele.WB235_4:4166741	4	4166741	4166830	90	7	12
GTTGCGTGTC	GTTGCGTGTCGC						
Cele.WB235	Cele.WB235_4:4166744	4	4166744	4167306	563	46	12
GCGTGTCGCG	GCGTGTCGCGTT						
Cele.WB235	Cele.WB235_4:4198521	4	4198521	4198622	102	6	17
GATGCCCCCT	GATGCCCCCTCTGAACG						
Cele.WB235	Cele.WB235_4:4305710	4	4305710	4305967	258	5	11
GTACTGTAGG	GTACTGTAGGA						
Cele.WB235	Cele.WB235_4:4325075	4	4325075	4325582	508	13	11
CTACAGTACT	CTACAGTACTA						
Cele.WB235	Cele.WB235_4:4335057	4	4335057	4335129	73	4	18
TTTTTTGATA	TTTTTTGATAATTCTTGA						
Cele.WB235	Cele.WB235_4:4355433	4	4355433	4355651	219	5	35
TTCCGGCAAA	TTCCGGCAAATCGACAATTTGCCGAAAAACAGAGT						
Cele.WB235	Cele.WB235_4:4401664	4	4401664	4401741	78	5	15
CGAAAAGGGG	CGAAAAGGGGATCTG						
Cele.WB235	Cele.WB235_4:4416113	4	4416113	4421995	5883	213	26
GTCTAACTTT	GTCTAACTTTGGAAATTCATCTAATG						
Cele.WB235	Cele.WB235_4:4459427	4	4459427	4459589	163	12	12
GCCTAAGCCT	GCCTAAGACTAC						
Cele.WB235	Cele.WB235_4:4532759	4	4532759	4533458	700	6	11
GCAGTAGGCA	GCAGTAGGAAG						
Cele.WB235	Cele.WB235_4:4554554	4	4554554	4555060	507	13	11
CTACAGTACT	CTACAGTACTA						
Cele.WB235	Cele.WB235_4:4599072	4	4599072	4599116	45	4	11
TGACAACTTT	TGACAACTTTG						
Cele.WB235	Cele.WB235_4:4702090	4	4702090	4702167	78	5	15
CGAAAAGGGG	CGAAAAGGGGATCTG						
Cele.WB235	Cele.WB235_4:4702342	4	4702342	4702434	93	6	15
TCCCCTTTTC	TCCCCTTTTCGTATC						
Cele.WB235	Cele.WB235_4:4792114	4	4792114	4792760	647	17	35
TTGCCGGAAA	TTGCCGGAAATTTTGGTTTCCAGCAAGTTGCCGAA						
Cele.WB235	Cele.WB235_4:4898047	4	4898047	4898612	566	18	15
GAGACCCATC	GAGACCCATCGTGGT						
Cele.WB235	Cele.WB235_4:4920724	4	4920724	4920924	201	5	40
TTCTAGAAAG	TTCTAGAAAGTTCTGGAACATTCTAGAATTTTCTCGATTT						

Cele.WB235	Cele.WB235_4:4920980	4	4920980	4921503	524	15	35
TTTTCAGAAA	TTTTCAGAAAATTTAAATTTCCCTCCAAAATATTT						
Cele.WB235	Cele.WB235_4:4921570	4	4921570	4922365	796	47	15
GGGTCTCACC	GGGTCTCACCACGAC						
Cele.WB235	Cele.WB235_4:4923556	4	4923556	4923766	211	6	35
AATATTTTTT	AATATTTTTTCAGAAAATTAAAATTTCCCTCCAA						
Cele.WB235	Cele.WB235_4:4923839	4	4923839	4924634	796	47	15
GGGTCTCACC	GGGTCTCACCACGAC						
Cele.WB235	Cele.WB235_4:4925825	4	4925825	4926035	211	6	35
AATATTTTTT	AATATTTTTTCAGAAAATTAAAATTTCCCTCCAA						
Cele.WB235	Cele.WB235_4:4926108	4	4926108	4926918	811	48	15
GGGTCTCACC	GGGTCTCACCACGAC						
Cele.WB235	Cele.WB235_4:4996655	4	4996655	4997045	391	8	39
CCAACTGGAG	CCAACTGGAGCCAGCACTATGACTGCTGTCGGAGGAGCA						
Cele.WB235	Cele.WB235_4:5060074	4	5060074	5060274	201	12	16
CTACGTGCCT	CTACGTGCCTACGTGC						
Cele.WB235	Cele.WB235_4:5273292	4	5273292	5273664	373	18	12
AGAAGGAGGA	AGAAGGAGGAGA						
Cele.WB235	Cele.WB235_4:5299099	4	5299099	5299474	376	15	25
TTTACTCTCT	TTTACTCTCTGTCTTCACAGTATAT						
Cele.WB235	Cele.WB235_4:5302167	4	5302167	5302359	193	10	12
TCCTCTTTCT	TCCTCTTTCTTC						
Cele.WB235	Cele.WB235_4:5456641	4	5456641	5456776	136	9	15
CCACGATGGG	CCACGATGGGTCTCG						
Cele.WB235	Cele.WB235_4:5465022	4	5465022	5467133	2112	44	48
AATTCAATTT	AATTCAATTTTTGATTTGCCTGTAATTTATATTTTTCTGAAGATGAGC						
Cele.WB235	Cele.WB235_4:5690583	4	5690583	5690638	56	5	11
TCCACTGACC	TCCACTGACCG						
Cele.WB235	Cele.WB235_4:5878242	4	5878242	5878392	151	6	25
CCAGAGAGTA	CCAGAGAGTAAAGTATACTGGGAAG						
Cele.WB235	Cele.WB235_4:6009084	4	6009084	6009409	326	6	55
AAGAGTCCCC	AAGAGTCCCCACACACTGAAAGGTTTTAAGGAATTTTTTGAGGATTCCTAGGGTT						
Cele.WB235	Cele.WB235_4:6090427	4	6090427	6090577	151	6	25
TTTACTCTCT	TTTACTCTCTGGCTTCACAGTATAT						
Cele.WB235	Cele.WB235_4:6210092	4	6210092	6210227	136	7	19
ATTGACAGAC	ATTGACAGACGAAAAATAG						
Cele.WB235	Cele.WB235_4:6291557	4	6291557	6291611	55	4	13
TTTCATTTGT	TTTCATTTGTAAT						
Cele.WB235	Cele.WB235_4:6357804	4	6357804	6361081	3278	132	25
TAGAGTAAAT	TAGAGTAAATAAATTAGCAGAACAT						
Cele.WB235	Cele.WB235_4:6469806	4	6469806	6469955	150	10	12
CAGAATATAT	CAGAATATATTC						
Cele.WB235	Cele.WB235_4:6599056	4	6599056	6599139	84	4	22
AAGCGGTAAC	AAGCGGTAACGTCGTCTATATA						
Cele.WB235	Cele.WB235_4:6664978	4	6664978	6665286	309	9	31
CCCGCCAAAA	CCCGCCAAAATTTTTTGAAAAAAATTTAATT						
Cele.WB235	Cele.WB235_4:6921936	4	6921936	6922772	837	9	93
TGCTTCTCGT	TGCTTCTCGTCGTCCACCACCACCACCGAAGGGAACTGGAACTCCACCACCACCACCAACTGGAGAACCACAGGATCTTTCTGGAGAAGGTAA						
Cele.WB235	Cele.WB235_4:7128107	4	7128107	7129879	1773	156	11
TGACAACTTC	TGACAACTTCG						
Cele.WB235	Cele.WB235_4:7441099	4	7441099	7441297	199	5	40
TCCAGAATTT	TCCAGAATTTTCTCGATTTTTCTAGAAAGTTCTGGAACAT						

Cele.WB235	Cele.WB235_4:7441316	4	7441316	7441526	211	6	35
CAGAAAATTT	CAGAAAATTTAAATTTCCCGCCAAAATATTTTTCT						
Cele.WB235	Cele.WB235_4:7441099	4	7441099	7441714	616	6	40
TCCAGAATTT	TCCAGAATTTTCTCGATTTTTCTAGAAAGTTCTGGAACAT						
Cele.WB235	Cele.WB235_4:7683397	4	7683397	7683518	122	6	17
CTCATTGCCT	CTCATTGCCTACCCTTG						
Cele.WB235	Cele.WB235_4:7727050	4	7727050	7727263	214	5	51
CCATCCGAAG	CCATCCGAAGATCCTCAACCATCTGGCCCACCATCTCCGGGACCAGTGGAC						
Cele.WB235	Cele.WB235_4:7734990	4	7734990	7736070	1081	24	45
ACCATCAGGC	ACCATCAGGCGGACCACCAGGACCATTTGATCCATCAGGAGCACC						
Cele.WB235	Cele.WB235_4:8015849	4	8015849	8015941	93	5	15
CGAAAAGGGG	CGAAAAGGGGAGATA						
Cele.WB235	Cele.WB235_4:8016116	4	8016116	8016208	93	6	15
TCCCCTTTTC	TCCCCTTTTCGTATC						
Cele.WB235	Cele.WB235_4:8073709	4	8073709	8073979	271	18	15
CTCACCACGA	CTCACCACGATGGGT						
Cele.WB235	Cele.WB235_4:8255934	4	8255934	8256202	269	6	45
TCTGATCCTT	TCTGATCCTTCAGCTCCGAAGGATCGTAAGGGTACCTCCTGATGG						
Cele.WB235	Cele.WB235_4:8467114	4	8467114	8467274	161	5	32
TTGAATTCCC	TTGAATTCCCGCCAAAATTTTTCAAAGAAATT						
Cele.WB235	Cele.WB235_4:8467000	4	8467000	8468026	1027	15	40
TTCTGGAACA	TTCTGGAACATTCGAGAATTTTCCCGATTTTTCTAGAAAG						
Cele.WB235	Cele.WB235_4:8467091	4	8467091	8469338	2248	33	40
TCCAGAATTT	TCCAGCATTTTCTCAAATTTTCCAGAAGGTTCTGGAACAT						
Cele.WB235	Cele.WB235_4:8469359	4	8469359	8469569	211	6	35
GAAAATTTAA	GAAAATTTAAATTTCCCACCAAAATATTTTTTTCA						
Cele.WB235	Cele.WB235_4:8469615	4	8469615	8470279	665	41	15
TCTCACCACG	TCTCACCACGATGGG						
Cele.WB235	Cele.WB235_4:8565636	4	8565636	8566140	505	13	11
CTACAGTACT	CTACAGTACTA						
Cele.WB235	Cele.WB235_4:8572647	4	8572647	8581868	9222	443	20
CGATATAGCT	CGATATAGCTATAAACAATA						
Cele.WB235	Cele.WB235_4:8789534	4	8789534	8789636	103	6	17
TTCATTCCGT	TTCATTCCGTGATTATT						
Cele.WB235	Cele.WB235_4:8999493	4	8999493	8999648	156	8	17
AGGCAATGAG	AGGCAATGAGAAAGAGT						
Cele.WB235	Cele.WB235_4:9066182	4	9066182	9067563	1382	81	15
CTCACCACGA	CTCACCACGAAGGGT						
Cele.WB235	Cele.WB235_4:9410150	4	9410150	9411415	1266	46	15
ACGATGGGTC	ACGACGGGTCTCACC						
Cele.WB235	Cele.WB235_4:9411472	4	9411472	9411612	141	5	30
AATTTTCCAG	AATTTTCCAGAAGGTTCTGGAACATTCCAG						
Cele.WB235	Cele.WB235_4:9411725	4	9411725	9412004	280	10	11
GTACTCCTAC	GTACTCCTACA						
Cele.WB235	Cele.WB235_4:9433776	4	9433776	9433910	135	4	11
CCCTACAGTA	CCCTACAGTAA						
Cele.WB235	Cele.WB235_4:10025402	4	10025402	10025894	493	10	11
TACTGTAGGG	TACTGTAGGGG						
Cele.WB235	Cele.WB235_4:10262913	4	10262913	10263301	389	26	15
GACCCATCGT	GACCCATCGTGGTGA						
Cele.WB235	Cele.WB235_4:10263336	4	10263336	10264889	1554	42	35
AATTCAAATT	AATTCAAATTTTCAGTGAAAAAAATTTTGGCGGGA						

Cele.WB235	Cele.WB235_4:10540321	4	10540321	10540705	385	5	77
GGAAGCTTCA	GGAAGCTTCATCTTTTGAATTATAAAAAAATAAATTTCATTTCAAAATCAGTGTGTGCACTATCAAAAGTGTCCACT						
Cele.WB235	Cele.WB235_4:10681773	4	10681773	10682153	381	20	19
ATTGTTACAC	ATTGTTACACGAAAAATAG						
Cele.WB235	Cele.WB235_4:10943462	4	10943462	10944138	677	14	45
AGCTCCGAAG	AGCTCCGAAGAATCAGAAGGGTACCTCCTGGTGATGTGATCCTTA						
Cele.WB235	Cele.WB235_4:10945693	4	10945693	10946234	542	12	45
CTTCGGAGCT	CTTCGGAGCTTAAGGTTCAGATCACCAGGAGGTACCCTTGTGATC						
Cele.WB235	Cele.WB235_4:10984356	4	10984356	10985109	754	4	11
TTTGAAATAT	TTTGAAATATA						
Cele.WB235	Cele.WB235_4:10985445	4	10985445	10985549	105	4	26
CCAGAGTCAC	CCAGAGTCACAATTTTTGGTGAACAA						
Cele.WB235	Cele.WB235_4:11033628	4	11033628	11036077	2450	31	79
GAAACTAAAA	GAAACTAAAACCTAATGAGACCCCAGAATACTGCCGGTATTCTGTCAGGACTCATTAGGTAGAGAGGAGATTCACAGAA						
Cele.WB235	Cele.WB235_4:11072392	4	11072392	11075919	3528	90	39
TTATGGAAAA	TTATGGAAAAGTGGCTCTATAGCCACTAGAAATACCAGT						
Cele.WB235	Cele.WB235_4:11178974	4	11178974	11179067	94	6	15
CGAAAAGGGG	CGAAAAGGGGATCTG						
Cele.WB235	Cele.WB235_4:11179242	4	11179242	11179304	63	4	15
TCCCCTTTTC	TCCCCTTTTCGCAGA						
Cele.WB235	Cele.WB235_4:11610541	4	11610541	11615517	4977	199	25
CATTACGGCA	CATTACGGCAGATTCTATAAATTGG						
Cele.WB235	Cele.WB235_4:11615692	4	11615692	11615955	264	13	17
AAAATCAAAC	AAAATCAAACCGGCGGA						
Cele.WB235	Cele.WB235_4:12022220	4	12022220	12022489	270	10	11
GTACTGTAGG	GTACTGTAGGG						
Cele.WB235	Cele.WB235_4:12022575	4	12022575	12022894	320	8	40
TCCAGAACCT	TCCAGAACCTTCTGGAAAATTCGAGAAAATTCTGGAATGT						
Cele.WB235	Cele.WB235_4:12023094	4	12023094	12025460	2367	74	32
CGGGAATTCA	CGGGAATTCAAATTTTCTGTGAAAAATTTTGG						
Cele.WB235	Cele.WB235_4:12155676	4	12155676	12156286	611	8	35
AAATTTGAAT	AAATTTGAATTTCCCGTCAAAATCTTTTTCTCAGA						
Cele.WB235	Cele.WB235_4:12169299	4	12169299	12171242	1944	24	59
TTTTAAAAAT	TTTTAAAAATTTTGAAAACCAGTGAAGTCTTGATAATCCATATTTTCCATAATTCTCAG						
Cele.WB235	Cele.WB235_4:12314464	4	12314464	12315037	574	6	96
GGTTGATTTG	GGTTGATTTGCGGCACTCTACAGATAGTGGATCTGCTTCTTACCAATCTTCCCGTTACCGCCACGCACTAAAGCTCCAAACTATTTTATTTTTTTG						
Cele.WB235	Cele.WB235_4:12326778	4	12326778	12327098	321	18	16
GTGCCTACGT	GTGCCTACGTGCCTAC						
Cele.WB235	Cele.WB235_4:12500507	4	12500507	12500642	136	5	27
ACAATAAAGT	ACAATAAAGTACTATGTATTACCGTAT						
Cele.WB235	Cele.WB235_4:12584972	4	12584972	12585067	96	5	19
AAAAATAGAT	AAAAATAGATTGGCAGACG						
Cele.WB235	Cele.WB235_4:12621478	4	12621478	12621570	93	6	15
CGAAAAGGGG	CGAAAAGGGGATCTG						
Cele.WB235	Cele.WB235_4:12621744	4	12621744	12621836	93	5	15
TCCCCTTTTC	TCCCCTTTTCGTATC						
Cele.WB235	Cele.WB235_4:12638029	4	12638029	12638605	577	6	96
CTTCTGGTAA	CTTCTGGTAACGTTCCCATAATTGGGTTAAATACCACTCATAAGTAACTAGTATGGGACTGAAAAGATACTAAATGAGCTTATTCTAAGGGTGAAG						
Cele.WB235	Cele.WB235_4:12678404	4	12678404	12678576	173	5	35
AAACCGGCAA	AAACCGGCAATTGCCGAAATTGCCGAAAATTCTCG						
Cele.WB235	Cele.WB235_4:12730503	4	12730503	12732683	2181	84	26
CATTGCGGCG	CATTGCGGCGGATTTTCGAAAAATGA						

Cele.WB235 Cele.WB235_4:12891406 4 12891406 12891917 512 9 48
TCATCTTCAG TCATCTTCAGAAAATTATAAATTACGGGCAAATCAAAAATTAAATTGC
Cele.WB235 Cele.WB235_4:12931758 4 12931758 12932786 1029 5 168
TTTTTAAAAA
TTTTTAAAAAATCTAGAATCCAAATCTATCAAAACATTTCCGGAATTCGATAATTTTTATTTTTGTTTTTTTGAGCCTACTAGTACCTGTGAAAATTGGAACTCGACACAAAAACCTCTCTAGCGCGGCTTAA
ATTTACTTTCCTGGCCCACAAACTTACCAATAATA
Cele.WB235 Cele.WB235_4:13007463 4 13007463 13008066 604 12 12
GCTTGGGTTT GCTTGGGGTTAG
Cele.WB235 Cele.WB235_4:13007463 4 13007463 13008198 736 17 12
GCTTGGGTTT GCTTGGGGTTAG
Cele.WB235 Cele.WB235_4:13115382 4 13115382 13116006 625 24 26
ATTAGATGAA ATTAGATGAATTTCCAAAGTTAGACC
Cele.WB235 Cele.WB235_4:13222198 4 13222198 13222342 145 12 12
TATACCTATA TATACCCATACC
Cele.WB235 Cele.WB235_4:13360500 4 13360500 13361979 1480 59 25
TTTACTCTCT TTTACTCTCTGGTTTCACAGAATAA
Cele.WB235 Cele.WB235_4:13431343 4 13431343 13431418 76 5 15
CACAGGAGGC CACAGGAGGCTGGGA
Cele.WB235 Cele.WB235_4:13462457 4 13462457 13462800 344 22 15
ACCCATCGTG ACCCATCGTGGCGAG
Cele.WB235 Cele.WB235_4:13462892 4 13462892 13463563 672 18 35
GAAATTTAAA GAAATTTAAATTTTCTGTGAAAAATATTTTGGCGG
Cele.WB235 Cele.WB235_4:13463617 4 13463617 13463705 89 4 23
AATATTTATT AATATTTATTTATTTATTTAATA
Cele.WB235 Cele.WB235_4:13462893 4 13462893 13464435 1543 25 35
AAATTTAAAT AAATTTAAATTTTCTGTGAAAAATATTTTGGCGGG
Cele.WB235 Cele.WB235_4:13464499 4 13464499 13464709 211 14 15
CCACGATGGG CCACGATGGGTCTCG
Cele.WB235 Cele.WB235_4:13463828 4 13463828 13464877 1050 12 40
GTTCTGGAAC GTTCTGGAACACTCCAGAATTTTCCCGATTTTTCTAGAAA
Cele.WB235 Cele.WB235_4:13464747 4 13464747 13465805 1059 13 27
TTTTTAAAAT TTTTTAAAATTCATATACCGTTCTAAC
Cele.WB235 Cele.WB235_4:13478657 4 13478657 13478875 219 6 31
AATTCAAATT AATTCAAATTTTCTCAAAAAATTTTGGCGGG
Cele.WB235 Cele.WB235_4:13547778 4 13547778 13547822 45 4 11
CACATTTTTT CACATTTTTTC
Cele.WB235 Cele.WB235_4:13575701 4 13575701 13576041 341 30 11
GCACATTTTT GCACATTTTCT
Cele.WB235 Cele.WB235_4:13668023 4 13668023 13668625 603 12 40
AAAATTCTGG AAAATTCTGGAAAGCTCTAGAACCTTTTGAAAAATTCGAG
Cele.WB235 Cele.WB235_4:13734177 4 13734177 13736953 2777 30 93
AGGGCCGCAG AGGGCCGCAGGCCCGAACACTCTTGGAAACTGGGCTGGCTTTAATCCTATTCCAAGCAGATTGTTATTCACTCAAGCCTATCAACCAAGTTTG
Cele.WB235 Cele.WB235_4:13845937 4 13845937 13846071 135 6 11
AGTACTGTAG AGTACTGTAGG
Cele.WB235 Cele.WB235_4:13846283 4 13846283 13847033 751 45 15
CCCATCGTGG CCCATCGTGGCGAGA
Cele.WB235 Cele.WB235_4:13847379 4 13847379 13847579 201 5 40
AATGTTCCAG AATGTTCCAGAACTTTCTAGAAAAATCGAGAAAATTCTGG
Cele.WB235 Cele.WB235_4:13861901 4 13861901 13862381 481 12 40
CAGAACCTTC CAGAACCTTCTAGAAAATCCGAGAAAATTCTGGAATGTTC
Cele.WB235 Cele.WB235_4:13861930 4 13861930 13862600 671 11 40
CTGGAATGTT CTGGAATGTTCCAAAACCTTCTGGAAAATTTGAGAAAATG

Cele.WB235 Cele.WB235_4:13884400 4 13884400 13884820 421 24 15
ACGATGGGTC ACGATGGGTCTCACC
Cele.WB235 Cele.WB235_4:13909776 4 13909776 13910308 533 19 32
CGACCTGCGA CGACCTGCGACCTAGCGACCTGGGTCGCTTTG
Cele.WB235 Cele.WB235_4:13922018 4 13922018 13922152 135 6 11
AGTACTGTAG AGTACTGTAGG
Cele.WB235 Cele.WB235_4:13922430 4 13922430 13922519 90 6 15
ACCCATCGTG ACCCATCGTGGTGAG
Cele.WB235 Cele.WB235_4:13922549 4 13922549 13923902 1354 39 35
GGGAAATTTA GGGAAATTTAAATTTTCTGAAAAAAATATTTTGGC
Cele.WB235 Cele.WB235_4:13964027 4 13964027 13964143 117 4 29
TACATTGCAA TACATTGCAAGATTTTCTTACTCTGTAGG
Cele.WB235 Cele.WB235_4:14205760 4 14205760 14206748 989 13 76
AATGCTAGAG AATGCTAGAGGACACTCATTTGCACGAAGGGAAGTGGGAGAGACTGTGGAGTTGTCGGTGTCTTTGTGCATGCTCG
Cele.WB235 Cele.WB235_4:14208040 4 14208040 14209239 1200 70 14
GCTATGCGCT GCTATGCGCTGTTC
Cele.WB235 Cele.WB235_4:14209284 4 14209284 14209609 326 13 25
TAATGCCAAT TAATGCCAATTTATAGAATCTGCCA
Cele.WB235 Cele.WB235_4:14209637 4 14209637 14209721 85 6 14
AGCGTATAGC AGCGTATAGCGTAT
Cele.WB235 Cele.WB235_4:14210874 4 14210874 14215456 4583 61 76
TCGTGCAAAG TCGTGCAAAGGAGTGTCCTCTAGCATTCGAGCATGCACAAAGACACCGACAACTCCACAGTCTCTCCCACTTCCCT
Cele.WB235 Cele.WB235_4:14218012 4 14218012 14218147 136 9 15
GGGTCTCACC GGGTCTCACCACGAT
Cele.WB235 Cele.WB235_4:14417231 4 14417231 14419021 1791 7 200
GAAAAAGTGA
GAAAAAGTGAATTTTCAGTCATACCTTGTAGACCACAATTTATATGCAAAATTAATAGATTTTCCAAACTTTGAAAATTAATTTCTGGCTTCAAACTAACTTTTTTAAAAATTCCCGTAGCCATTTTGTAGCA
AATTTTATGTTCTATTAGAATTTCAAAAGAGAGTTTCAAAAAATTTTGAAACCATTAAGTTCAAGCA
Cele.WB235 Cele.WB235_4:14426797 4 14426797 14427553 757 36 21
CAACTACCAC CAACTACCACAACTACCGTAC
Cele.WB235 Cele.WB235_4:14447004 4 14447004 14447298 295 13 21
ACTACCGTAC ACTACCGTACCAACTACCACA
Cele.WB235 Cele.WB235_4:14476813 4 14476813 14476978 166 11 15
GACCCATCGT GACCTATCGTGGCGA
Cele.WB235 Cele.WB235_4:14476805 4 14476805 14480237 3433 67 15
CGTGGTGAGA CGTGGCGAGACCTAT
Cele.WB235 Cele.WB235_4:14740244 4 14740244 14740404 161 5 40
TTTTCTAGAA TTTTCTAGAAAATTCTGGACCATTCCAGAACTTTCTCGGA
Cele.WB235 Cele.WB235_4:14740489 4 14740489 14741012 524 15 35
AAATTTGAAT AAATTTGAATTTCCCGCCAAAATTGTTTGCACTGC
Cele.WB235 Cele.WB235_4:14781306 4 14781306 14781436 131 5 26
TGGTTAGAGT TGGTTAGAGTCATTATTTTAGGTGAA
Cele.WB235 Cele.WB235_4:15034367 4 15034367 15034488 122 9 11
TGTGCAAAAA TGTGCAAAAAT
Cele.WB235 Cele.WB235_4:15059144 4 15059144 15059784 641 16 40
TTTTCTAGAA TTTTCTAGAAAGTTCTGGAACATTCCAGAAATTTCCCGAC
Cele.WB235 Cele.WB235_4:15060108 4 15060108 15060798 691 39 15
GGTCTCACCA GGTCTCACCACGATG
Cele.WB235 Cele.WB235_4:15166280 4 15166280 15166460 181 11 15
GAGACCCATC GAGACCCATCGTGGC
Cele.WB235 Cele.WB235_4:15166547 4 15166547 15166687 141 4 35
AAATTTTCTG AAATTTTCTGTGAAAAATATTTTGGCGGGAAATTT

Cele.WB235	Cele.WB235_4:15166752	4	15166752	15167072	321	8	40	GAATGTTCCA	GAATGTTCCAGAACTTTCTAGAACAATCGAGAAAATTCTG
Cele.WB235	Cele.WB235_4:15188409	4	15188409	15188529	121	8	15	GGTGAGACCC	GGTGAGACCCTTCGT
Cele.WB235	Cele.WB235_4:15188545	4	15188545	15190957	2413	70	34	TTGGCGGGAA	TTGGCGGGAAATTCAAATTTTCAGTGAAAAAATT
Cele.WB235	Cele.WB235_4:15199553	4	15199553	15199645	93	6	15	CGAAAAGGGG	CGAAAAGGGGATCTG
Cele.WB235	Cele.WB235_4:15199820	4	15199820	15199897	78	5	15	TCCCCTTTTC	TCCCCTTTTCGTATC
Cele.WB235	Cele.WB235_4:15201340	4	15201340	15201723	384	11	35	GGGAAATTCA	GGGAAATTCAAATTTTTAGTAAAAAAATATTTTGGC
Cele.WB235	Cele.WB235_4:15201392	4	15201392	15202170	779	9	35	GTAAAAAATA	GTAAAAAATATTTTGGGAGGAAATTCAAATTTTTA
Cele.WB235	Cele.WB235_4:15225095	4	15225095	15227378	2284	12	168	AAAAACAAAA	AAAAACAAAAATAAAAATTTTGGAATGTTGGGAGAATTTTTATAGATTTGGATTCTAGATTTTTAACAAATATTTTTGGTAAGTTTCTGGACCTAGATATCAATTTTCAGATGCGCTAGAGGGGTTTTTGTCTTGAATTCCAATTTCAACAGATATATATACAGGCACAA
Cele.WB235	Cele.WB235_4:15225869	4	15225869	15227795	1927	11	168	AACAAAAATA	AACAAAAATAAAAATTTTGGAATGTTGGGAGAATTTTTATAGATTTGGATTCTAGATTTTTAACAAATATTTTTGGTAAGTTTCTGGACCTAGATATCAATTTTCAGATGCGCTAGAGGGGTTTTTGTGTTGAAATTTATTTTTCACAGGTATATATACAGGCACAAAAA
Cele.WB235	Cele.WB235_4:15294619	4	15294619	15295382	764	4	14	TTGTAGTTTG	TTGTAATTTGTAGT
Cele.WB235	Cele.WB235_4:15319979	4	15319979	15320069	91	6	15	TCGTGGTGAG	TCGTGGTGGGACCCT
Cele.WB235	Cele.WB235_4:15320096	4	15320096	15321040	945	27	35	GAAATTCAAA	GAAATTCAAATTTTAAGTGAAAAAAATTTTGGCGG
Cele.WB235	Cele.WB235_4:15374013	4	15374013	15374693	681	17	40	GAACATTCCA	GAACATTCCAGAATTTTCTCGATTTTTCTAGAAAGTTCTG
Cele.WB235	Cele.WB235_4:15375074	4	15375074	15376143	1070	51	15	CCACGATGGG	CCACGATGGGTCTCA
Cele.WB235	Cele.WB235_4:15409425	4	15409425	15409737	313	12	26	CAGAGTCACT	CAGAGTCACTAAATTTGGTGAACGGC
Cele.WB235	Cele.WB235_4:15410980	4	15410980	15411070	91	8	10	TGTTATGATA	GTTATGATAG
Cele.WB235	Cele.WB235_4:15511605	4	15511605	15513283	1679	10	168	GAATTCCGGA	GAATTCCGGAAATGTTTTGATAGATTTGGATTCTGGTTTTTTTTTAAATATTATTGGAAAGTTTGTGGACCAGGAAAGTAAATTTAAGCCGCGCTAAAGAGGTTTTTGTGTCGAGTTCCAATTTTTACAGGTACTAGTAGGCGCAAAAAAACAAAAATAAAAATTATG
Cele.WB235	Cele.WB235_4:15517213	4	15517213	15517493	281	8	35	TTGCCGGATT	TTGCCGGATTTGAAAATTTATGGTAATTTGCCGTT
Cele.WB235	Cele.WB235_4:15636148	4	15636148	15636960	813	51	14	GGACTAGGGA	GGACTAAGGACTAG
Cele.WB235	Cele.WB235_4:15648768	4	15648768	15648845	78	6	11	TGCACATTTT	TGCACATTTTT
Cele.WB235	Cele.WB235_4:15708521	4	15708521	15708971	451	14	32	TTGAATTCCC	TTGAATTCCCGCCAAAATTTTTCAAAGAAATT
Cele.WB235	Cele.WB235_4:15709349	4	15709349	15709549	201	5	40	ATTTTCCAGA	ATTTTCCAGAAGGTTCTGGAACATTCCAGAATTTTCTCAA
Cele.WB235	Cele.WB235_4:15765625	4	15765625	15765904	280	6	40	GATTTTTCTA	GATTTTTCTAGAAAGTTCTGGAACATTCCAGAATTTTCTC

Cele.WB235	Cele.WB235_4:15765291	4	15765291	15766235	945	8	35
AAAATTTAAA	AAAATTTAAATTTCCCGCCAAAATATTTTTCTCAG						
Cele.WB235	Cele.WB235_4:15766277	4	15766277	15766397	121	8	15
GGGTCTCACC	GGGTCTCACCACGAT						
Cele.WB235	Cele.WB235_4:15766300	4	15766300	15766555	256	17	15
CCACGATGGG	CCACGATGGGTCTCA						
Cele.WB235	Cele.WB235_4:15766783	4	15766783	15767403	621	19	11
GTACTCCTAC	GTACTCCTACA						
Cele.WB235	Cele.WB235_4:15776137	4	15776137	15776526	390	5	42
GCTCGGCAAA	GCTCGGCAAATATTGAAAAAAGAGATTTGCCGAATTTGCCGT						
Cele.WB235	Cele.WB235_4:15784879	4	15784879	15785128	250	6	31
TCGACAGAAG	TCGACAGAAGCTGTCGATCATCAGACTTTCC						
Cele.WB235	Cele.WB235_4:15826645	4	15826645	15826960	316	21	15
CCCATCGTGG	CCCATCGTGGCGAGA						
Cele.WB235	Cele.WB235_4:15826993	4	15826993	15827272	280	8	35
AATTTAAATT	AATTTAAATTTCTGTGAAAAATATTTGGCGGGA						
Cele.WB235	Cele.WB235_4:15826528	4	15826528	15827586	1059	8	40
GTTCCAGAAC	GTTCCAGAACCTTCTGGGAATTTCTGGAAAATTCTAGAAT						
Cele.WB235	Cele.WB235_4:15842446	4	15842446	15843519	1074	16	37
CGGGAATTCA	CGGGAATTCAAATTTTAATTTTTCTAAAACATTTTGG						
Cele.WB235	Cele.WB235_4:15859222	4	15859222	15859379	158	8	20
TGTTACACGA	TGTTACACGAAAAAAATAGAT						
Cele.WB235	Cele.WB235_4:15895865	4	15895865	15896183	319	8	40
GAACATTCCA	GAACATTCCAGACTTTTCCCAATTTTTCTAGAAAGTTCTG						
Cele.WB235	Cele.WB235_4:15971109	4	15971109	15972271	1163	29	35
TTCCCGCCAA	TTCCCGCCAAAAATTTTTTCTCAGTTAATTTGAAT						
Cele.WB235	Cele.WB235_4:15971205	4	15971205	15972491	1287	15	15
CACGATGGGT	CACGATGGGTCTCGT						
Cele.WB235	Cele.WB235_4:16094815	4	16094815	16095563	749	18	11
GTACTGTAGG	GTACTGTAGTA						
Cele.WB235	Cele.WB235_4:16128108	4	16128108	16129641	1534	16	15
GGGTCTCACC	GGGTCTCACCGCGAA						
Cele.WB235	Cele.WB235_4:16153344	4	16153344	16153449	106	7	15
GTGGTGAGAC	GTGGTGAGACCCTTC						
Cele.WB235	Cele.WB235_4:16223761	4	16223761	16224157	397	25	15
TCTCACCACG	TCACACCACGAAAGG						
Cele.WB235	Cele.WB235_4:16244112	4	16244112	16245385	1274	65	19
AAATAGATTG	AAATAGATTGTTACACGGA						
Cele.WB235	Cele.WB235_4:16403739	4	16403739	16404089	351	10	35
CAAATCGGCA	CAAATCGGCAAATTGCCGAAATTGTAAAGTTCCGG						
Cele.WB235	Cele.WB235_4:16408366	4	16408366	16409098	733	21	35
TTTGGCATAT	TTTGGCATATTGCTGATTTGCCGGAAAATTTCAAC						
Cele.WB235	Cele.WB235_4:16409693	4	16409693	16409938	246	7	35
GCAAATCGGC	GCAAATCGGCAAAATGTCCGAATTGAAATTTTCCG						
Cele.WB235	Cele.WB235_4:16425000	4	16425000	16425220	221	8	20
TTTTAGTGAA	TTTTAGTGAAAATTTGAGAT						
Cele.WB235	Cele.WB235_4:16427870	4	16427870	16428521	652	13	50
GGGCGTGTTT	GGGCGTGTTTTCACATGCCCCGCCCACAAAATTGATGTTTCCACCAAAAT						
Cele.WB235	Cele.WB235_4:16458486	4	16458486	16458684	199	5	40
TTCCAGAATT	TTCCAGAATTTTCTCGATTTTTCTAGAAAGTTCTGGAACG						
Cele.WB235	Cele.WB235_4:16458853	4	16458853	16459033	181	10	15
TGGGTCTCAC	TGGGTCTCACCACGA						

Cele.WB235	Cele.WB235_4:16471145	4	16471145	16471947	803	4	37
ATTCAAATTT	ATTCAAATTTTAATTTTTTGAAAATATTTTGACGGGA						
Cele.WB235	Cele.WB235_4:16491101	4	16491101	16491225	125	5	21
TTCAAGCCTT	TTCAAGCCTTTTCAGACCAAT						
Cele.WB235	Cele.WB235_4:16511822	4	16511822	16512828	1007	39	11
CTACAGTACT	CTACAGTACCT						
Cele.WB235	Cele.WB235_4:16522968	4	16522968	16523369	402	28	11
CCCCTACAGT	CCCCTACAGTA						
Cele.WB235	Cele.WB235_4:16649049	4	16649049	16649496	448	15	30
AAAATCAAAA	AAAATCAAAAATTAGATGAATTTTCGATTA						
Cele.WB235	Cele.WB235_4:16675606	4	16675606	16675766	161	4	40
TTCCAGAATT	TTCCAGAATTTTCCCGATTTTTCTAGAAAGTTCTGGAACG						
Cele.WB235	Cele.WB235_4:16675832	4	16675832	16676039	208	6	35
TTGAATTTCC	TTGAATTTCCCGCCAAAATATTTTTCTCAGAAAAT						
Cele.WB235	Cele.WB235_4:16681092	4	16681092	16681615	524	4	150
TTGAGGGCCG	TTGAGGGCCGCAGTCCCGAATACACGAAGAAGTGTTGGAGATCCGTCAAGCCTATTCTAAGCATAATAATTCAGTTCGAGTGTCGCAAGCCCAAATAGACGCAGAAGCGTTGGACAACTTTTAGGCCTATTCTGAGCCCAATAATTCATG						
Cele.WB235	Cele.WB235_4:16880641	4	16880641	16880761	121	8	15
TCGTGGTGAG	TCGTGGTGGGACCCT						
Cele.WB235	Cele.WB235_4:16910220	4	16910220	16910354	135	6	11
AGTACTGTAG	AGTACTGTAGG						
Cele.WB235	Cele.WB235_4:16910542	4	16910542	16910677	136	9	15
TCGTGGTGAG	TCGTGGTGAGACCCA						
Cele.WB235	Cele.WB235_4:16910719	4	16910719	16910928	210	6	35
GAAATTTAAA	GAAATTTAAATTTTCTGTGAAAAATATTTTGGCGG						
Cele.WB235	Cele.WB235_4:16910953	4	16910953	16911111	159	4	40
AAAATTCTGG	AACATTCTGGAATGTTCCAGAACTTTCTAGAAAAATCGAG						
Cele.WB235	Cele.WB235_4:16963408	4	16963408	16964890	1483	77	19
AAAAATAGAT	AAAAATAGATTGTTACACG						
Cele.WB235	Cele.WB235_4:17059789	4	17059789	17062957	3169	48	66
CAAATCAACC	CAAATCAACCTATTCAGTTGACAATGGTTTTCTTTTATCAAATTTTTAAAAATTTTAAAGAAAACA						
Cele.WB235	Cele.WB235_4:17076321	4	17076321	17076588	268	10	11
CCTACAGTAC	CCTACAGTACT						
Cele.WB235	Cele.WB235_4:17128248	4	17128248	17129128	881	20	44
GATTGATGGC	GATTGATGGCCGAAAGGCTGTTTTCTGAGCATTGTTGTATTTCG						
Cele.WB235	Cele.WB235_4:17134995	4	17134995	17135342	348	5	18
GCACTCCCTA	GCACTCCCTATTAGTCTT						
Cele.WB235	Cele.WB235_4:17140454	4	17140454	17141354	901	60	15
ACCCATCGTG	ACCCATCGTGGTGAG						
Cele.WB235	Cele.WB235_4:17141438	4	17141438	17141648	211	6	35
AAATTTTCTG	AAATTTTCTGAGAAAAATATTTTGGCGGGAAATTT						
Cele.WB235	Cele.WB235_4:17147999	4	17147999	17148449	451	28	15
TCTCACCACG	TCTCACCACGAAGTG						
Cele.WB235	Cele.WB235_4:17147932	4	17147932	17148762	831	9	37
ATTCCCGCCA	ATTCCCGCCAAAATATTTCCAAAAAATTAAAATTTTA						
Cele.WB235	Cele.WB235_4:17262649	4	17262649	17262776	128	4	32
GAAGAAATTT	GAAGAAATTTGAATTTGCACCAAAATTTTTCT						
Cele.WB235	Cele.WB235_4:17285019	4	17285019	17285079	61	4	15
GTAGATCTTG	GTAGATCTTGTTTTG						
Cele.WB235	Cele.WB235_4:17323162	4	17323162	17323330	169	7	24
TCAACAACAA	TCAACAACAACAGGAGAAGCTTCT						

Cele.WB235	Cele.WB235_4:17345179	4	17345179	17345256	78	5	15
TTCCCCTTTT	TTCCCCTTTTCGTAT						
Cele.WB235	Cele.WB235_4:17492409	4	17492409	17493609	1201	48	25
ACTCTCTGGC	ACTCTCTGGCTTCACCATATATTTT						
Cele.WB235	Cele.WB235_4:17493616	4	17493616	17493814	199	16	12
GGCTTAGGCT	GGCTTAGGCTTA						
Cele.WB235	Cele.WB235_5:6	5	6	311	306	25	12
TAAGCCTAAG	TAAGCCTAAGCC						
Cele.WB235	Cele.WB235_5:100578	5	100578	100668	91	6	15
AAAAGGAGAA	AAAAGGAGAATTTCG						
Cele.WB235	Cele.WB235_5:100901	5	100901	100991	91	6	15
TTCTCCTTTT	TTCTCCTTTTCGTCG						
Cele.WB235	Cele.WB235_5:317369	5	317369	318431	1063	6	175
AAATTTTGGG	AAATTTTGGGTTCACTCTTTTTACTAGACAGTGATTGTTGCATTGTAGGATTCGGTGGACTTGTCCAACAACTTACTTTGGCATGAGTTTAATAAAAAGGTTTTACCATGAACTGAATCAATTTAAAATAAAAACCTCGAGAACGAAAACATCCTCAGTAATTTCAATCCTGTTC						
Cele.WB235	Cele.WB235_5:335210	5	335210	335330	121	7	16
CATGCCTACA	CATGCCTACATGCCTA						
Cele.WB235	Cele.WB235_5:342974	5	342974	343198	225	14	16
GGCATGTAGG	GGCATGTAGGCATGTA						
Cele.WB235	Cele.WB235_5:365726	5	365726	365780	55	4	12
AGGCTTAGGC	AGGCTTAGGCTA						
Cele.WB235	Cele.WB235_5:775516	5	775516	775721	206	6	34
CAATTTGCCG	CAATTTGCCGGTTTGTCGGAAATTTCGAATCCGG						
Cele.WB235	Cele.WB235_5:775870	5	775870	776051	182	5	35
TTTCCGGCAA	TTTCCGGCAAACCGGCGAATTGCCGGAATTGAAAA						
Cele.WB235	Cele.WB235_5:777315	5	777315	777395	81	5	16
CTACATGCCT	CTACATGCCTACATGC						
Cele.WB235	Cele.WB235_5:777834	5	777834	778359	526	12	16
AGGCATGTAG	AGGCATGTAGGCATGT						
Cele.WB235	Cele.WB235_5:779287	5	779287	779455	169	10	16
AGGCATGTAG	AGGCATGTAGGCATGT						
Cele.WB235	Cele.WB235_5:842648	5	842648	842948	301	24	10
GGCGTCAGCG	GGCGTCAGCG						
Cele.WB235	Cele.WB235_5:885274	5	885274	885474	201	12	16
ATGCCTACAT	ATGCCTAGATGCCTAG						
Cele.WB235	Cele.WB235_5:904306	5	904306	904460	155	7	20
CCTACCCCAG	CCTACCCCAGGCTGACCTAG						
Cele.WB235	Cele.WB235_5:904415	5	904415	904569	155	8	20
GGCAGACCTA	GGCAGGCCTAACCTACCCCA						
Cele.WB235	Cele.WB235_5:967850	5	967850	968138	289	7	35
TGCCGATTTG	TGCCGATTTGCCGGAAATGTTCATTTTCGAGAATT						
Cele.WB235	Cele.WB235_5:1015554	5	1015554	1016070	517	9	35
GCATTTGCCG	GCATTTGCCGGTTTCGGAAATTTTCGGCAATTTCG						
Cele.WB235	Cele.WB235_5:1025021	5	1025021	1025505	485	20	22
ACAGTACTCC	ACAGTACTCCTACAGTACCACT						
Cele.WB235	Cele.WB235_5:1027504	5	1027504	1027725	222	11	13
GCCTATGGCC	GCCTATGGCAAAC						
Cele.WB235	Cele.WB235_5:1028226	5	1028226	1028460	235	18	13
CGTAGGCCAT	CGTAGGCCATATA						
Cele.WB235	Cele.WB235_5:1047118	5	1047118	1047214	97	6	18
AAGCCTAAGC	AAGCCTAAGTCTAAATCT						

Cele.WB235 Cele.WB235_5:1104557 5 1104557 1105016 460 17 27
TTACTCTCTG TTACTCTCTGTGGCTTCACAGATTAAG
Cele.WB235 Cele.WB235_5:1129473 5 1129473 1129617 145 10 12
GGCTTAGGCT GGCTTCGGCTTA
Cele.WB235 Cele.WB235_5:1142352 5 1142352 1142454 103 5 12
CTAAGCCTAA CTGAGCCTAAGC
Cele.WB235 Cele.WB235_5:1197262 5 1197262 1197339 78 5 15
CGAAAAGGGG CGAAAAGGGGATCTG
Cele.WB235 Cele.WB235_5:1197514 5 1197514 1197589 76 5 15
TCCCCTTTTC TCCCCTTTTCGTATC
Cele.WB235 Cele.WB235_5:1201995 5 1201995 1204402 2408 13 185
AATATAAACA
AATATAAACATACACGAATTTGTCTGATGTATAAAGATTCCCGAAGACACTTTCCAATTACCCAAATTGTTCATATTCTAAATGAGATTCTCTTACTAGAACACTCTTGGCCAATGTACGCAGCCGAACGTAT
CATAAGTGAATACAGAACACCAGTTATGTCCGAGCAAAAAGATCCGATTCAG
Cele.WB235 Cele.WB235_5:1227227 5 1227227 1227275 49 4 12
AAGCCTAAGC AAGCCTAAGCTT
Cele.WB235 Cele.WB235_5:1230606 5 1230606 1230858 253 19 12
AAGCCTAAGC AAGCCTAAGCCT
Cele.WB235 Cele.WB235_5:1236678 5 1236678 1236956 279 15 12
CTAAGCCTAA CTAGGCCTAAAC
Cele.WB235 Cele.WB235_5:1239922 5 1239922 1240281 360 10 35
GAAATTTTCA GAAATTTTCAATTCCGGCAAATTGCCTAATTGCCA
Cele.WB235 Cele.WB235_5:1243605 5 1243605 1243701 97 7 12
CTTAGGCTTA CTTAGGCTTAGG
Cele.WB235 Cele.WB235_5:1249610 5 1249610 1249931 322 10 11
TACCTACTAG TACCTACTAGA
Cele.WB235 Cele.WB235_5:1254384 5 1254384 1254459 76 5 15
TCGTGGTGAG TCGTGGTGAGACCCA
Cele.WB235 Cele.WB235_5:1254384 5 1254384 1256245 1862 31 15
TCGTGGTGAG TCGTGGTGAGACCCA
Cele.WB235 Cele.WB235_5:1254387 5 1254387 1257024 2638 44 15
TGGTGAGACC TGGTGAGACCCATCG
Cele.WB235 Cele.WB235_5:1254505 5 1254505 1257773 3269 35 35
GCGGGAAATT GCGGGAAATTCAAATTTTCAGTGAAAAAAATTTTG
Cele.WB235 Cele.WB235_5:1273379 5 1273379 1274003 625 14 35
AATTCTGGCA AATTCTGGCAAATTACCGATTTGCCGGAAATTTTC
Cele.WB235 Cele.WB235_5:1273379 5 1273379 1274654 1276 15 35
AATTCTGGCA AATTCTGGCAAATTACCGATTTGCCGGAAATTTTC
Cele.WB235 Cele.WB235_5:1305908 5 1305908 1306004 97 7 12
AGGCTTAGGC AGGCTTAGGCTC
Cele.WB235 Cele.WB235_5:1341430 5 1341430 1341604 175 13 12
CTAAGCCTAA CTAAGCTTAAGC
Cele.WB235 Cele.WB235_5:1352314 5 1352314 1352754 441 9 40
AATGTTCTAG AATGTTCTAGAACCTCCTGGAAATTTCGAGAAAATTCTGA
Cele.WB235 Cele.WB235_5:1355392 5 1355392 1355652 261 6 40
CTAGAACATT CTAGAACTTTCCAGAATTTTCTCAAACTTTCTAGAAGGTT
Cele.WB235 Cele.WB235_5:1368467 5 1368467 1368669 203 6 34
AAATTCAAAT AAATTCAAATTTTCTGAGAAATAAATTTGACGGA
Cele.WB235 Cele.WB235_5:1377073 5 1377073 1377773 701 21 35
ATTTTCAATT ATTTTCAATTCCAGCAATTTGCCGATTTTCTTGAA
Cele.WB235 Cele.WB235_5:1385851 5 1385851 1386349 499 9 35
TTTGCCGGAA TTTGCCGGAAAATTTCAATTCCGGCAAAATGTGGA

Cele.WB235	Cele.WB235_5:1388994	5	1388994	1389344	351	8	35
TGCCGGAAAT	TGCCGGAAATTTTGATTTTTGGTAATTTGCCGATT						
Cele.WB235	Cele.WB235_5:1403527	5	1403527	1403806	280	7	40
ATTTTTCTAG	ATTTTTCTAGAAAGTTCTGGAACATTCCAGACTTTTCCCA						
Cele.WB235	Cele.WB235_5:1403881	5	1403881	1404196	316	21	15
CGATGGGTCT	CGATGGGTCTCACCA						
Cele.WB235	Cele.WB235_5:1404428	5	1404428	1404696	269	8	11
CTACAGTACC	CTACAGTACTA						
Cele.WB235	Cele.WB235_5:1423375	5	1423375	1423735	361	24	12
CCTAAGCCTA	CCTAAGCCTAAG						
Cele.WB235	Cele.WB235_5:1481682	5	1481682	1481898	217	10	12
AGCCTAAGCC	AGCCTAAGCCTA						
Cele.WB235	Cele.WB235_5:1530916	5	1530916	1531076	161	4	40
GTTCTAGAAC	GTTCTAGAACATTCCAGAATTTTTTCGAATTTTCCAGAAG						
Cele.WB235	Cele.WB235_5:1539714	5	1539714	1540122	409	5	89
GCACTACGTT	GCACTACGTTGCGCACACACTAAGCTACTCGTTTCACGCCAGGCTGTGGAACCCCGAACGTGTCGCCCGCTCCAAATAACTATCGTTCA						
Cele.WB235	Cele.WB235_5:1637931	5	1637931	1639035	1105	23	48
CCCGTAATTT	CCCGTAATTTATAAATTTCTGAAGATAAGCAATTTAAATTTTGAGTTT						
Cele.WB235	Cele.WB235_5:1661122	5	1661122	1661200	79	5	18
GCCTAAGCCT	GCCTAAGCCTAAGCATAC						
Cele.WB235	Cele.WB235_5:1718733	5	1718733	1718863	131	9	13
GCTCAAGCTA	GCTCAAGCTAGGT						
Cele.WB235	Cele.WB235_5:1813009	5	1813009	1813219	211	7	35
TCCGGCAATT	TCCGGCAATTTGTCGATTTGCCGGAAATTTTAAAA						
Cele.WB235	Cele.WB235_5:1825303	5	1825303	1825405	103	7	12
AGCCTGAGCC	AGCCTGAGCCTA						
Cele.WB235	Cele.WB235_5:1848647	5	1848647	1848932	286	14	17
TAAGCCTAAG	TGAGCCTAAGCTTAGCT						
Cele.WB235	Cele.WB235_5:1850026	5	1850026	1850346	321	23	10
GGGCGTCAGC	GGGCGTCAGC						
Cele.WB235	Cele.WB235_5:1907825	5	1907825	1908395	571	7	93
CAGTACCCTC	CAGTACCCTCAAGGATCATCTGCTCCACGGTATCCTGGTAACTACAATGCCTTTGCCAGCACTTCCGATGCTCCACAGTATCCAGGATCCTCC						
Cele.WB235	Cele.WB235_5:1911033	5	1911033	1911178	146	7	11
CTACAGTACT	CTACAGTACTA						
Cele.WB235	Cele.WB235_5:1940104	5	1940104	1940384	281	8	35
GGCAAATCGG	GGCAAATCGGCAATTTTCCGAAAATGAAAATTTCT						
Cele.WB235	Cele.WB235_5:2102635	5	2102635	2102940	306	21	13
GCCTAAGCCT	GCTTAAGCCTCGG						
Cele.WB235	Cele.WB235_5:2141423	5	2141423	2141937	515	18	11
CTACAGTACC	CTACAGTACTA						
Cele.WB235	Cele.WB235_5:2164618	5	2164618	2164730	113	6	16
ATGCCTACAT	ATGCCTACATGCCTAC						
Cele.WB235	Cele.WB235_5:2389657	5	2389657	2390366	710	4	177
GAGTCTCACG	GAGTCTCACGATGTGTCAACGTGTCTCAGTCGGCTTGATCTACGTAGATCTACAAAAAATGCGGGAGAAGAGACGCAGACTTATTCTCTGATTTTGCATGAGTAAGAGCGTGCTGGCGTCACATTTTTGTCGGCAAAAAATTCCCGCATTTTTTGTAGATCAAACCGTAATGGGACG						
Cele.WB235	Cele.WB235_5:2391673	5	2391673	2391869	197	4	35
AAATTTTCAA	AAATTTTCAATTCCTCCAGTTTGCCAATTTGCCGG						
Cele.WB235	Cele.WB235_5:2394729	5	2394729	2394777	49	4	12
AGGCTTAGGC	AGGCTTAAGCTC						
Cele.WB235	Cele.WB235_5:2497936	5	2497936	2498645	710	4	177
GAGTCTCACG							

GAGTCTCACGATGTGTCAACGTGTCTCAGTCGGCTTGATCTACGTAGATCTACAAAAAATGCGGGAGAAGAGACGCAGACTTATTCTCTGATTTTGCATGAGTAAGAGCGTGCTGGCGTCACATTTTTGTCGG
CAAAAAAATTCCCGCATTTTTTGTAGATCAAACCGTAATGGGACG

Cele.WB235	Cele.WB235_5:2499952	5	2499952	2500148	197	4	35
AAATTTTCAA	AAATTTTCAATTCCTCCAGTTTGCCAATTTGCCGG						
Cele.WB235	Cele.WB235_5:2503008	5	2503008	2503056	49	4	12
AGGCTTAGGC	AGGCTTAAGCTC						
Cele.WB235	Cele.WB235_5:2586211	5	2586211	2586358	148	6	12
CCTAAGCCTA	CCTAGGCCTAAA						
Cele.WB235	Cele.WB235_5:2634833	5	2634833	2635037	205	7	20
CCCCGACGCT	CCCCGACGCTCGAGCTCAAA						
Cele.WB235	Cele.WB235_5:2634964	5	2634964	2635164	201	10	20
GCTCGAGCTC	GCTCGAGCTCAAACCCCGAC						
Cele.WB235	Cele.WB235_5:2636817	5	2636817	2636991	175	6	21
GATTAGATTT	GATTAGATTTCGACCATATTG						
Cele.WB235	Cele.WB235_5:2640455	5	2640455	2641508	1054	15	35
TCAATTCCGG	TCAATTCCGGCAATTTTCCGATTTGCCGGAAATTG						
Cele.WB235	Cele.WB235_5:2654959	5	2654959	2655536	578	16	35
TTTGCCGGAA	TTTGCCGGAAATTTTCAATTCCGACAATTTGCCGG						
Cele.WB235	Cele.WB235_5:2660817	5	2660817	2661267	451	10	35
TTTCCGGCAA	TTTCCGGCAAATCGGAAAAGTGTCCAAGTTTAAAA						
Cele.WB235	Cele.WB235_5:2670563	5	2670563	2670782	220	6	35
GCCGGAAATT	GCCGGAAATTTTCAATTCCGGCAACTCCCCGATTT						
Cele.WB235	Cele.WB235_5:2676568	5	2676568	2676778	211	14	15
CTCACCACGA	CTCACCACGAAGGGT						
Cele.WB235	Cele.WB235_5:2712581	5	2712581	2712737	157	13	12
CTAAGCCTAA	CTAAGCCTAAGC						
Cele.WB235	Cele.WB235_5:2716232	5	2716232	2716355	124	4	35
TTTCTCAGAA	TTTCTCAGAAAATTTGAATTTCCCGCCAAAATGTT						
Cele.WB235	Cele.WB235_5:2777566	5	2777566	2778653	1088	65	10
TACCGCCCGC	TACCGCCCGC						
Cele.WB235	Cele.WB235_5:2886582	5	2886582	2886789	208	19	11
GCACATTTTT	GCACATTTTTT						
Cele.WB235	Cele.WB235_5:3056063	5	3056063	3056653	591	10	59
AATTTCAAAT	AATTTCAAATGTGCACTGGTTTTCAAAATTTTTTAAACTGAGAATTATGGAAAATATGG						
Cele.WB235	Cele.WB235_5:3085214	5	3085214	3085707	494	4	16
GCATGTAGGC	GCATGTAGGCATGTAG						
Cele.WB235	Cele.WB235_5:3092621	5	3092621	3092687	67	5	11
TTTGGCAAGA	TTTGGCAAGAA						
Cele.WB235	Cele.WB235_5:3098854	5	3098854	3099539	686	35	19
AATCTATTTT	AATCTATTTTTCGTAAGTC						
Cele.WB235	Cele.WB235_5:3145392	5	3145392	3145472	81	6	10
GCGGGCGGTA	GCGGGCGATA						
Cele.WB235	Cele.WB235_5:3332437	5	3332437	3332702	266	4	18
TGCAAGACTA	TGCAAGACTATTAGAGGC						
Cele.WB235	Cele.WB235_5:3373171	5	3373171	3373906	736	20	21
CTAACATTTT	CTAACATTTTTCAATTTTCGT						
Cele.WB235	Cele.WB235_5:3520159	5	3520159	3520417	259	7	11
TACTGTAGGA	TACTGTAGAGG						
Cele.WB235	Cele.WB235_5:3520502	5	3520502	3520742	241	6	40
TCCAGAACCT	TCCAGAACCTTCTGGAAAATCCGAGAAAATTCTGGAATGT						
Cele.WB235	Cele.WB235_5:3520923	5	3520923	3521917	995	31	32
CGGGAATTCA	CGGGAATTCAACATTTCTGTGAAAAATTTTGG						

Cele.WB235	Cele.WB235_5:3621266	5	3621266	3621341	76	5	15	CGTGGTGAGA	CGTGGCGAGACCCAC
Cele.WB235	Cele.WB235_5:3629745	5	3629745	3629789	45	4	11	CAAAAAATGT	CAAAAAATGTG
Cele.WB235	Cele.WB235_5:3632093	5	3632093	3632198	106	5	21	TTTCAGTTTT	TTTCAGTTTTCGTCTATCATT
Cele.WB235	Cele.WB235_5:3651539	5	3651539	3651616	78	4	21	AGTTTGTAGT	AGTCTGTAGTTTGTCGTTTGT
Cele.WB235	Cele.WB235_5:3679665	5	3679665	3679834	170	4	42	TTTCCAGCAA	TTTCCAGCAAATTGGCAAATCGGCAAATTGTCGGAATTAAAA
Cele.WB235	Cele.WB235_5:3694442	5	3694442	3694754	313	8	39	GATTTACGGG	GATTTACGGGCATTGATCAGTTTCAAAAAATCTTGTGTC
Cele.WB235	Cele.WB235_5:3702753	5	3702753	3703068	316	9	35	AAAATTTCCG	AAAATTTCCGGCAAATTGACAAATTTATGGAATTG
Cele.WB235	Cele.WB235_5:3731028	5	3731028	3731508	481	23	11	AGGATTACTG	AGGATTACTGT
Cele.WB235	Cele.WB235_5:3763703	5	3763703	3764133	431	4	35	GCCGATTTGC	GCCGATTTGCCGGAAATGTTCAGTTCTGGCAAATT
Cele.WB235	Cele.WB235_5:3765128	5	3765128	3765284	157	4	35	TTGCCGATTT	TTGCAGATTTGCCGGAAAAATTCAATTCCGGCAAT
Cele.WB235	Cele.WB235_5:3767901	5	3767901	3768001	101	6	10	TAGCCATGCC	TAGCCATGCC
Cele.WB235	Cele.WB235_5:3816982	5	3816982	3817234	253	6	42	ACCGGCAATT	ACCGGCAATTGCCAGAATTGCCGATCGCTGAAAATTTCCAAA
Cele.WB235	Cele.WB235_5:4029541	5	4029541	4030122	582	5	42	CCAGCCGACA	CCAGCCGACAGCCGAAATTTTCACGATTTTCGGCTGGTAGCG
Cele.WB235	Cele.WB235_5:4050682	5	4050682	4051242	561	14	40	GAACCTTCTG	GAACCTTCTGAAAAATTCGAGAAAATTTTGGAATGTTTTT
Cele.WB235	Cele.WB235_5:4052517	5	4052517	4053360	844	21	40	CTAGAAAGTT	CTAGAAAGTTCTGGAATATTCCAGAATTTTATTGAACTTC
Cele.WB235	Cele.WB235_5:4053377	5	4053377	4053861	485	15	32	TTTCACAGAA	TTTCACAGAAAATTTGAATTCCCGCCAAAATT
Cele.WB235	Cele.WB235_5:4146424	5	4146424	4146572	149	4	35	TTTGCCGGAA	TTTGCCGGAAAATTTAATTTTTGCCAAATTGCCGT
Cele.WB235	Cele.WB235_5:4190322	5	4190322	4190733	412	11	35	TTCCGGCAAA	TTCCGGCAAACCGGCAATTTCCCGAAAACGAAAAA
Cele.WB235	Cele.WB235_5:4193678	5	4193678	4193984	307	5	35	AATTTTCAAT	AATTTTAAATTCCTGCAATTTGCCAGTTTGCCAGA
Cele.WB235	Cele.WB235_5:4197817	5	4197817	4198139	323	8	35	TTTCAATTCC	TTTCAATTCCGGAAGTTTGCCGATTTGCCGGAAAT
Cele.WB235	Cele.WB235_5:4244061	5	4244061	4247526	3466	18	200	TGTGGTCCAG	TGTGGTCCAGAAGGTATGACTGAAAATTCACTTTTTCTGCTTGAACTTGGCAGATTTAAAATTTTTCAAAATGCTGTTTTCAAATTCTGAAAGAACTTAAAATTTGCTACAAAATGGCTACGGGAATTTCTAAAAAAGTTAGTTTCAAGCCAGAAATTAATTTTCAAAAATTGGAAAATCTATTAATTTTGTATATAAAT
Cele.WB235	Cele.WB235_5:4268549	5	4268549	4268631	83	4	18	AAATATTCTT	AAATATTCTTATTTTCAG
Cele.WB235	Cele.WB235_5:4333269	5	4333269	4336644	3376	135	25	AGAGTAAAAT	AGAGTAAAATATTCTGTGAAGCCAG
Cele.WB235	Cele.WB235_5:4443918	5	4443918	4444112	195	5	35	ATTCCGGCAA	ATTCCGGCAAGTTGCCGGTTTGCCGGAAGTATTCA
Cele.WB235	Cele.WB235_5:4553148	5	4553148	4553225	78	5	15	CCCCTTTTCG	CCCCTTTTCGTTGCT

Cele.WB235	Cele.WB235_5:4653023	5	4653023	4653224	202	8	18
CCTGGCTGTC	CCTGGCTGTCCATCGTTT						
Cele.WB235	Cele.WB235_5:4685532	5	4685532	4685994	463	10	35
GCAAATCGGC	GCAAATCGGCAAATTGCCGGATTCGAAAATGTCCG						
Cele.WB235	Cele.WB235_5:4718387	5	4718387	4718578	192	5	35
CGGCAAATCG	CGGCAAATCGGCAACTTGCCGGAATTTTAAATTTC						
Cele.WB235	Cele.WB235_5:4773346	5	4773346	4773621	276	22	11
CACGAAGTTG	CACAAAGTTGT						
Cele.WB235	Cele.WB235_5:4784597	5	4784597	4784789	193	11	16
TAGGCATGTA	TAGGCATGTAGGAAAG						
Cele.WB235	Cele.WB235_5:4866206	5	4866206	4866377	172	5	17
AAATGCCAAA	AAATGCCAAAACTGCCA						
Cele.WB235	Cele.WB235_5:5004204	5	5004204	5005690	1487	35	11
GTACTGTAGG	GTACTGTAGGA						
Cele.WB235	Cele.WB235_5:5005844	5	5005844	5006744	901	59	15
TCGTGGTGAG	TCGTGGTGAGACTCT						
Cele.WB235	Cele.WB235_5:5009264	5	5009264	5010522	1259	36	35
AAATTTGAAT	AAATTTGAATTTCCCGCCAAAATTTTTTTCACTGA						
Cele.WB235	Cele.WB235_5:5010548	5	5010548	5010653	106	7	15
GGGTCTCACC	GGGTCTCACCACGAT						
Cele.WB235	Cele.WB235_5:5073280	5	5073280	5076759	3480	131	26
AAAGTTAGAC	AAAGTTAGACCATTAGATGAATTTCC						
Cele.WB235	Cele.WB235_5:5117986	5	5117986	5118371	386	10	35
TGCCGGAAAT	TGCCGGAAATTTCGATTTTCGGCAAAATGCCGATT						
Cele.WB235	Cele.WB235_5:5125946	5	5125946	5126346	401	8	35
TTGCCGATTT	TTGCCGATTTGCCGGAACTTTCCAATTTCGACAAG						
Cele.WB235	Cele.WB235_5:5215493	5	5215493	5215916	424	9	35
CGATTTGCCG	CGATTTGCCGGAAATTTTTATTTTTGGCAAATTGC						
Cele.WB235	Cele.WB235_5:5283302	5	5283302	5285834	2533	98	26
CAGAGTCACT	CAGAGTCACTAATTTTGGTGAACGGT						
Cele.WB235	Cele.WB235_5:5415371	5	5415371	5416162	792	8	96
TCTGCTTAGA	TCTGCTTAGAATAGGATTAAAGCCAGCCCACAGTTTTCAAGAGTATTCGGGCCTGCGGCCCTCAAACCTGGTTATTAGGCTTGAGTGAATAACATA						
Cele.WB235	Cele.WB235_5:5640056	5	5640056	5640116	61	4	15
CGTGGTGAGA	CGTGGTGAGACCCTT						
Cele.WB235	Cele.WB235_5:5646992	5	5646992	5647147	156	8	19
AAAAATAGAT	AAAAATAGATTGGCAGACG						
Cele.WB235	Cele.WB235_5:5754995	5	5754995	5755070	76	5	15
CGAAAAGGGG	CAAAAAGGGGATCTG						
Cele.WB235	Cele.WB235_5:5755255	5	5755255	5755347	93	6	15
TCCCCTTTTC	TCCCCTTTTCGTATC						
Cele.WB235	Cele.WB235_5:6071658	5	6071658	6071958	301	20	15
ACCCATCGTG	ACCCATCGTGGTGAG						
Cele.WB235	Cele.WB235_5:6071995	5	6071995	6072240	246	7	35
ATTTTGGCGG	ATTTTGGCGGGAAATTTAAATTTTCTGTGAAAAAT						
Cele.WB235	Cele.WB235_5:6072277	5	6072277	6072797	521	13	40
TTCCAGAACT	TTCCAGAACTTTCTAGAAAAATTGGGAAAAGTCTGGAATG						
Cele.WB235	Cele.WB235_5:6088840	5	6088840	6089240	401	10	40
TGGAATGTTC	TGGAATGTTCCAGAACTTTCTAGAAAAATTGGGAAAAGTC						
Cele.WB235	Cele.WB235_5:6072983	5	6072983	6091328	18346	45	226
CTTTGAAGAG	CTTTGAAGAGATTTATGAATTTTCAAAGTTTGAAAAATCCACCTATTTTTGCCTATTGATGGTAGTTTTCATTAAATAATAAAATACGCACTAAAAGGTAGGGCTGAAAATGTACTATTTCTGTAACTACGTGAAGCTTCGTGTACCTACTGAAATATTGTTAATATATTCTGAAAGAGCTTAAAATTTACTACAAAGTTTCTGAAAGACATTTCAAAAAAAATTA						

Cele.WB235	Cele.WB235_5:6091617	5	6091617	6091992	376	25	15
CCCATCGTGG	CCCATCGTGGTGAGA						
Cele.WB235	Cele.WB235_5:6171700	5	6171700	6176164	4465	182	24
TTTCAGTTTA	TTTGAGTTTATCATCCTTATCTTT						
Cele.WB235	Cele.WB235_5:6176242	5	6176242	6183220	6979	54	117
TGTTGGTTCG	TGTTGGTTCGACAGTAGGTGCATTAGCCTCTGGAACTGGAACATCCTTTTGCTCGACGATTGCAGCTTGTTGTACTTCAGATGTTTCCTTTGATTCCACAGGTGCAAGTTTCTCAAC						
Cele.WB235	Cele.WB235_5:6188375	5	6188375	6189068	694	15	42
AGGTGCTGCT	AGGTGCTGCTTCCTTCGGAACATTTTCTTGAGTTGGCTCCGA						
Cele.WB235	Cele.WB235_5:6219658	5	6219658	6220602	945	16	59
ATGTGCACTG	ATGTGCACTGGTTTTCAAAAATTTTTAAACTGAGAATAATGGAGAATAGGGAATTTCAA						
Cele.WB235	Cele.WB235_5:6296625	5	6296625	6296906	282	8	35
AATTTCCCGC	AATTTCCCGCCAAAATTTTTTTCAGAGAAAATTTG						
Cele.WB235	Cele.WB235_5:6297848	5	6297848	6298284	437	5	35
TTTCCCGCCA	TTTCCGCCAAAATTGTTTTTCACTGAAAATTTGAA						
Cele.WB235	Cele.WB235_5:6299893	5	6299893	6299998	106	7	15
GGGTCTCACC	GGGTCTCACCACGAT						
Cele.WB235	Cele.WB235_5:6392866	5	6392866	6392950	85	4	21
TTGGTGACAA	TTGGTGACAATGTGGAAACAA						
Cele.WB235	Cele.WB235_5:6587809	5	6587809	6587899	91	6	15
GGTGAGACCC	GGTGAGACCCTTCGT						
Cele.WB235	Cele.WB235_5:6910515	5	6910515	6910653	139	7	17
CTCATTGCCT	CTCATTGACTACCCTTG						
Cele.WB235	Cele.WB235_5:6937435	5	6937435	6937626	192	10	19
AACAATCTAT	AACAATCTATTTTTCGTGT						
Cele.WB235	Cele.WB235_5:6942140	5	6942140	6942965	826	14	59
AATTCAAGAC	AATTCAAGACTTCACTGGTTTTCAAAAAACTTAAAACCGAGAATTATGGGGAATATGGA						
Cele.WB235	Cele.WB235_5:6944029	5	6944029	6944530	502	36	12
TTTAATTTTA	TTTAATTTTAAT						
Cele.WB235	Cele.WB235_5:6984815	5	6984815	6985177	363	11	32
AATTCAAATT	AATTCAAATTTTTTGTGAAAATATTTGGCGGG						
Cele.WB235	Cele.WB235_5:7115024	5	7115024	7115184	161	4	40
TTCTAGAACC	TTCTAGAACCTTCTGGAAAGTTCAAGAGAATTCTGGAATG						
Cele.WB235	Cele.WB235_5:7259575	5	7259575	7259935	361	6	60
CTAGCTAGAT	CTAGCTAGATTGAACGCTCTCAAGAGAAAGCATCTTGTTGCTGCCCACAAGAAGAAGGCT						
Cele.WB235	Cele.WB235_5:7337779	5	7337779	7337924	146	8	18
TTTCATAAGA	TTTCATAAGAAGTAAATC						
Cele.WB235	Cele.WB235_5:7442929	5	7442929	7443089	161	4	40
GAAGGTTCTA	GAAGGTTCTAGAATATTCCAGAATATTCCAAAACTTTCCA						
Cele.WB235	Cele.WB235_5:7443218	5	7443218	7444716	1499	50	11
CTACAGTACC	CTACAGTACCT						
Cele.WB235	Cele.WB235_5:7703008	5	7703008	7703129	122	6	17
CTCATTGCCT	CTCATTGCCTACCCTTG						
Cele.WB235	Cele.WB235_5:7720637	5	7720637	7720787	151	10	15
GGGTCTCACC	GGGTCTCACCACGAA						
Cele.WB235	Cele.WB235_5:7912986	5	7912986	7913626	641	16	40
TCTGGAACAT	TCTGGAACATTCCAGAATTTCCTCGATTTTTCTAGAAAGT						
Cele.WB235	Cele.WB235_5:7913711	5	7913711	7913991	281	8	35
CAGAAAATTT	CAGAAAATTTAAATTTCCCGCAAAAATATTTTTCA						
Cele.WB235	Cele.WB235_5:7914059	5	7914059	7914224	166	11	15
CCACGATGGG	CCACGATGGGTCTCA						
Cele.WB235	Cele.WB235_5:7915927	5	7915927	7916107	181	11	15
ACCCATCGTG	ACCCATCGTGGCGAG						

Cele.WB235	Cele.WB235_5:7916137	5	7916137	7916840	704	20	35	GGGAAATTTA	GGGAAATTTAAATTTTCTGTGAAAAATATTTTGGC
Cele.WB235	Cele.WB235_5:7916966	5	7916966	7925007	8042	201	40	TTCTAGAAAA	TTCTAGAAAAATTGGGAAAAGTCTGGAATGTTCCAGAACT
Cele.WB235	Cele.WB235_5:7988900	5	7988900	7991510	2611	90	29	CATAGTGGGA	CATAGTGGGAAATCCTTACAATGTATCGA
Cele.WB235	Cele.WB235_5:7993172	5	7993172	7993472	301	12	25	CCAGAGAGTA	CCAGAGAGTAAATTATACTGTGAAG
Cele.WB235	Cele.WB235_5:8314150	5	8314150	8315036	887	51	17	TAGTCAATGA	TAGTCAATGATCTGAGG
Cele.WB235	Cele.WB235_5:8316057	5	8316057	8316570	514	17	15	GGGTCTCACC	GGGTCTCACCGCGAA
Cele.WB235	Cele.WB235_5:8699367	5	8699367	8702913	3547	59	60	ATTCAGTTGA	ATTCAGTTGAGCTCGTTATGTCGGTTGAAGACACAATTGGAGTGGTGCTTGAGGAGCTGG
Cele.WB235	Cele.WB235_5:8705331	5	8705331	8709788	4458	38	114	GTGTTTCAGA	GTGTTTCAGAATTACATGGAGTCTCACTACTTGCAGAATTTGTGGCTGTAGACGATTGACCTGCTGTAGTTGAATCGCTTGAAACTGTCATAGTGGATGTTCCATCAGACGTTT
Cele.WB235	Cele.WB235_5:8714029	5	8714029	8714965	937	12	78	TGCAAGGCTC	TGCAAGGCTCCGATGATGATACCACTTCCAAAGTCGAGGTGTTACAATTTTTGGATGTAATAGAACTTGAATCAGTGA
Cele.WB235	Cele.WB235_5:8836966	5	8836966	8837173	208	8	26	TTCGCCCTGA	TTCGCCCTGAGCGATTTACCTCCCGT
Cele.WB235	Cele.WB235_5:8879060	5	8879060	8879267	208	8	26	TTCGCCCTGA	TTCGCCCTGAGCGATTTACCTCCCGT
Cele.WB235	Cele.WB235_5:9428459	5	9428459	9435308	6850	32	214	GTAACTGCTC	GTAACTGCTCAAAGCTTCTGGCACCTATTGGGATGTTGTTGGTAAATTCTGAAAAAACTTGGAATTTGCAACAAAGTTTCTGAAAGAATTTTCGAAAAAAGTTGATTTGAAGTGATTTGGGAATTTTCAAAGTTTCATAAATTTACCTATTTTGCCTATAAATGGTCGTTTTCATTTAATAATAAAATACGCACAAAGGTACTGTAGGTTTTCT
Cele.WB235	Cele.WB235_5:9439011	5	9439011	9439221	211	6	35	AGAAAATTTA	AGAAAATTTAAATTTCCCTCCAAAATTTTTTTCTC
Cele.WB235	Cele.WB235_5:9439276	5	9439276	9439441	166	11	15	CGATGGGTCT	CGATGGGTCTCACCA
Cele.WB235	Cele.WB235_5:9439666	5	9439666	9439923	258	8	11	GTACTCCTAC	GTACTCCTACA
Cele.WB235	Cele.WB235_5:9601934	5	9601934	9602249	316	21	15	GGTGAGACCC	GGTGAGACCCTTCGT
Cele.WB235	Cele.WB235_5:9602338	5	9602338	9603453	1116	32	35	GCGGGAAATT	GCGGGAAATTCAAATTTTCAGTGAAAAAAATTTTG
Cele.WB235	Cele.WB235_5:9603508	5	9603508	9604068	561	15	40	TTTCTAGAAA	TTTCTAGAAAATTCGGGAAAACTCTGGAATATTCCAGAAC
Cele.WB235	Cele.WB235_5:9933015	5	9933015	9933120	106	5	21	TTCTAATTCG	TTCTAATTCGACGAGCATCCC
Cele.WB235	Cele.WB235_5:10268112	5	10268112	10269248	1137	33	34	TTGGCGGGAA	TTGGCGGGAAATTCAAATTTTCAGTGAAAAAATT
Cele.WB235	Cele.WB235_5:10446631	5	10446631	10446995	365	14	26	CCAGAGTCAC	CCAGAGTCACTATAATTGGTGAACGG
Cele.WB235	Cele.WB235_5:10449424	5	10449424	10450674	1251	50	25	TTTACTCTCT	TTTACTCTCTGGCTTCACAGTATAT
Cele.WB235	Cele.WB235_5:10450709	5	10450709	10450922	214	19	11	AGATACACTT	AGATACACTTG
Cele.WB235	Cele.WB235_5:10605694	5	10605694	10612481	6788	174	39	GGCCACCAGA	GGCCACCAGAAATACCAGTTTATGAAAAAGTGGCTCTAG
Cele.WB235	Cele.WB235_5:11057749	5	11057749	11057963	215	10	11	CACATTTTTT	CACATTTTTTG

Cele.WB235	Cele.WB235_5:11108155	5	11108155	11108378	224	5	45
TCTGATCCTT	TCTGATCCTTCAGCTCCGAAGGATCACATGGGTACCTTCTGATGT						
Cele.WB235	Cele.WB235_5:11108491	5	11108491	11108983	493	11	45
CTGAAGGATC	CTGAAGGATCAGACCATCAGGAGGTACCCTTACGATCCTTCTTAT						
Cele.WB235	Cele.WB235_5:11273771	5	11273771	11274491	721	16	45
ACAACTGAAT	ACAACTGAATCAACTGCAGCACCAAGCACATCTACAGAGACACCT						
Cele.WB235	Cele.WB235_5:12077837	5	12077837	12077917	81	5	16
AAAAATTATT	AAAAATTATTGAATAC						
Cele.WB235	Cele.WB235_5:12370870	5	12370870	12371218	349	5	18
GCACTCCCTA	GCACTCCCTATTAGTCTT						
Cele.WB235	Cele.WB235_5:12510458	5	12510458	12510577	120	8	14
CTATACGCTA	CTATACGCTATACG						
Cele.WB235	Cele.WB235_5:12633007	5	12633007	12633394	388	10	39
TGGTACGGTA	TGGTACGGTAGTTGTAGTGGTAGTGGAGGTTGTTGTAGG						
Cele.WB235	Cele.WB235_5:13365913	5	13365913	13366445	533	28	19
GTTACACGAA	GTTACACGAAAAAATAGATT						
Cele.WB235	Cele.WB235_5:13440191	5	13440191	13440658	468	12	40
TCTGGAATGT	TCTGGAATGTTCCAGAACTTTCTAAAAAAAATCGGGAAAAT						
Cele.WB235	Cele.WB235_5:13640261	5	13640261	13645859	5599	335	16
GAAAAATTTT	GAAAAATTTTGCTGTT						
Cele.WB235	Cele.WB235_5:13640261	5	13640261	13646660	6400	357	16
GAAAAATTTT	GAAAAATTTTGCTGTT						
Cele.WB235	Cele.WB235_5:13882296	5	13882296	13882386	91	6	15
TCGTGGTGAG	TCGTGGTGAGACCCT						
Cele.WB235	Cele.WB235_5:13882409	5	13882409	13882690	282	8	35
GCGGGAAATT	GCGGGAAATTCAAATTTTCAGTGAAAAAAATTTTA						
Cele.WB235	Cele.WB235_5:13882781	5	13882781	13883141	361	10	40
TTTCTAGAAA	TTTCTAGAAAATTCGAGAAAATTCCGGAATGGTCCAGAAT						
Cele.WB235	Cele.WB235_5:13956630	5	13956630	13956708	79	6	12
TCTTTCTCCT	TCTTTCTCCTTT						
Cele.WB235	Cele.WB235_5:14053790	5	14053790	14054393	604	4	151
CATTTCAGAT	CATTTCAGATATTCCATCACCACCAACACCTTCATCCACTTCTTCGCCTGGAACGACGGGATTTTCTACAAAAATCGGATTTTTTCTCTATGTCAGTTTAATTGATGTATAATTCCGAAAAGTCAGTCTTTCCTAGGTTTTTAAATTGAAA						
Cele.WB235	Cele.WB235_5:14057570	5	14057570	14057714	145	6	11
CTACAGTACC	CTACAGTACCC						
Cele.WB235	Cele.WB235_5:14104614	5	14104614	14106168	1555	20	57
GAACTCAACA	GAACTCAACAAGCTGATCAAGAGCAACACCGTGGATCCCGATCTGCCGGACAGAAGG						
Cele.WB235	Cele.WB235_5:14107921	5	14107921	14109961	2041	34	60
CTGCTGATTC	CTGCTGATTCCGAGAAGAAGGCCCCAACCAAGAGAGAATCTGATAAGCCAAAGACTGAAT						
Cele.WB235	Cele.WB235_5:14124319	5	14124319	14124409	91	6	15
CGTGGTGAGA	CGTGTTGAGACCCTA						
Cele.WB235	Cele.WB235_5:14124469	5	14124469	14125286	818	21	35
GGAAATTCAA	GGAAATTCAAATTTTAAGTGAAAAAAATTTTGGCG						
Cele.WB235	Cele.WB235_5:14156207	5	14156207	14156297	91	6	15
GGGTCTCACC	GGGTCTCACCACGAA						
Cele.WB235	Cele.WB235_5:14204941	5	14204941	14205737	797	14	64
GTAGTCAAAA	GTAGTCAAAAAGACAAAAGTGCGAATGTAGCTATTTTTTCAGTCAGTAGCGAAAAAGACATTCA						
Cele.WB235	Cele.WB235_5:14320937	5	14320937	14321192	256	5	24
GCCGCTCCAG	GCCGCTCCAGCACCAGCACCAGAA						
Cele.WB235	Cele.WB235_5:14348178	5	14348178	14348446	269	6	45
TCTGATCCTT	TCTGATCCTTCAGATAAGAAGGATCGTAAGGGTACCTCCTGATGG						

Cele.WB235 Cele.WB235_5:14348461 5 14348461 14348640 180 4 45
CTGAAGGATC CTGAAGGATCAGACCATCAGGAGGTACCCTTACGATCCTTCTTAT
Cele.WB235 Cele.WB235_5:14444028 5 14444028 14444120 93 6 15
TCCCCTTTTC TCCCCTTTTCGTATC
Cele.WB235 Cele.WB235_5:14586076 5 14586076 14591096 5021 31 162
TTCCGTACAA
TTCCGTACAAGCTATCATACTTTGTTTGGCATTTTCTGGTATATTTGGTCGTGCTGAACAATTTTTTGAAATAAAACAGAATCGCTTCATATGATTTTCCCATACTTTTGAATTTTTGAAAATAATAGCTTCT
GGTTAATTTCTGAATTTTTAAAAATTAAC
Cele.WB235 Cele.WB235_5:14750559 5 14750559 14751884 1326 53 25
TGTGAAGCCA TGTGAAGCCAGAGGGTAAATTATTC
Cele.WB235 Cele.WB235_5:14754513 5 14754513 14757536 3024 84 27
CTGTGGCTTC CTGTGGCTTCCAACCATATTTTACTCT
Cele.WB235 Cele.WB235_5:14766633 5 14766633 14767947 1315 28 49
GCTACGATAT GCTACGATATCGTAGCACTTTTTTATTTTTCCATATTCCACCAGAAAAT
Cele.WB235 Cele.WB235_5:14768011 5 14768011 14769734 1724 59 27
GGAAGCCACA GGAAGCCACAGAGAGTAAAATATGGTG
Cele.WB235 Cele.WB235_5:15009961 5 15009961 15010009 49 4 12
TCTAAGTCTA TCTAAGTCTAAA
Cele.WB235 Cele.WB235_5:15168733 5 15168733 15168927 195 6 35
GGAATTGAAA GGAATTGAAAATTTCCGGCAAATTGGCAAATTGGC
Cele.WB235 Cele.WB235_5:15170122 5 15170122 15170278 157 6 26
TTCACCTAAA TTCACCTAAAAATATGACTCTAACCA
Cele.WB235 Cele.WB235_5:15295547 5 15295547 15296333 787 8 15
TCTCACCACG TCTCACCACGATGGG
Cele.WB235 Cele.WB235_5:15320778 5 15320778 15321338 561 14 40
CGATTTTTCT CGATTTTTCTAGAAAATTCTGGAACACTCCAGAATTTTCC
Cele.WB235 Cele.WB235_5:15321484 5 15321484 15322280 797 53 15
CCACGATGGG CCACGATGGGTCTCA
Cele.WB235 Cele.WB235_5:15322538 5 15322538 15322672 135 5 11
GTACTCCTAC GTACTCCTACA
Cele.WB235 Cele.WB235_5:15525755 5 15525755 15526135 381 5 73
TTAGGCTTAA TTAGGCTTAAGTGTGTCATAAGGATTTCCAATATTTTCTTCCAGTTGGGCATCCTAGTATGTTTTTCGGGCCT
Cele.WB235 Cele.WB235_5:15582778 5 15582778 15582853 76 5 15
AGACCCATCG AGACCCATCGTAGTG
Cele.WB235 Cele.WB235_5:15582889 5 15582889 15584265 1377 36 35
TTCAAATTTT TTCAAATTTTCTGTGAGAAAAATTTTGGGGGGAAA
Cele.WB235 Cele.WB235_5:15615355 5 15615355 15615426 72 5 12
CTTAGGCTTA CTTAGACTTAGG
Cele.WB235 Cele.WB235_5:15770931 5 15770931 15771023 93 6 15
CGAAAAGGGG CGAAAAGGGGATCTG
Cele.WB235 Cele.WB235_5:15823540 5 15823540 15823605 66 4 11
GTACTGTAGG GTACTGTAGGG
Cele.WB235 Cele.WB235_5:15941558 5 15941558 15942466 909 7 37
GCGGGAATTC GCGGGAATTCAAATTTTAATTATTTGAAATCATTTTG
Cele.WB235 Cele.WB235_5:16191704 5 16191704 16191915 212 6 42
TTTCGGCAAC TTTCGGCAACCGGCAATTGCCGATGTTGCCGAACCCGAAAAA
Cele.WB235 Cele.WB235_5:16273671 5 16273671 16273852 182 5 36
GGGAATTCAA GGGAATTCAAATTTCAATTTTTGAAAACATTTTGGC
Cele.WB235 Cele.WB235_5:16273937 5 16273937 16274402 466 31 15
TGGTGAGACC TGGTGAGACCTTTCG
Cele.WB235 Cele.WB235_5:16330598 5 16330598 16333224 2627 16 164
AAAAAATCAG

AAAAAATCAGCTTTTATATATTTTCATCAATTTTTAAAAAAATACTTCCGTACAAGCTATTATACTTTGATTGGCATATTTTGGTGTTTTTGACTATGCTGAACATTTTTATGAAATAAAAAAGAATGACTCC
ATATGATTTTCTAGGACTTTTGAATTTTTGA

Cele.WB235 Cele.WB235_5:16449792 5 16449792 16451728 1937 16 121
AAAAGATAGT
AAAAGATAGTTGCTCTTTGAGCAGAAGGTTGCAAACCGCAAGAGCAAACTTGCCAAATGACAAGCCACCCAAGTACTCGTTGCACCTACCTATCATAAGCTCGTAGACTATAATTTCTCTG

Cele.WB235 Cele.WB235_5:16530645 5 16530645 16530908 264 16 12
CATTGACTTG CATTGACTTGGG

Cele.WB235 Cele.WB235_5:16536032 5 16536032 16536834 803 14 22
GGGTACTGTA GGGTACTGTAGTAGTACTGTAT

Cele.WB235 Cele.WB235_5:16547005 5 16547005 16547587 583 10 11
GTACTGTAGG GTACTGTAGGG

Cele.WB235 Cele.WB235_5:16547704 5 16547704 16548504 801 18 40
CTGGAATGTT CTGGAATGTTCCAAAACCTTCTGGAAAATTTGAGAAAATG

Cele.WB235 Cele.WB235_5:16547704 5 16547704 16548822 1119 19 40
CTGGAATGTT CTGGAATGTTCCAAAACCTTCTGGAAAATTTGAGAAAATG

Cele.WB235 Cele.WB235_5:16552748 5 16552748 16552808 61 4 15
CGAAAAGGGG CAAAAAGGGGATCTG

Cele.WB235 Cele.WB235_5:16553018 5 16553018 16553153 136 7 15
GCAGATCCCC GCAGATCCCCTTTTT

Cele.WB235 Cele.WB235_5:16583907 5 16583907 16584035 129 4 32
GCTGATTGGT GCTGATTGGTGAGATACTGAGCATTTCTGAAA

Cele.WB235 Cele.WB235_5:16625438 5 16625438 16625585 148 7 21
ATTTTTCAAT ATTTTTCAATCTTCGTCTAAA

Cele.WB235 Cele.WB235_5:16672548 5 16672548 16672735 188 11 17
TAACCTGACT TAACCTGACTGCAACAG

Cele.WB235 Cele.WB235_5:16699106 5 16699106 16699538 433 26 16
ACATACCTAC ACATACCTACATACCT

Cele.WB235 Cele.WB235_5:16878345 5 16878345 16878591 247 7 11
CTACAGTACC CTACAGTACCT

Cele.WB235 Cele.WB235_5:16941584 5 16941584 16941761 178 5 21
CTAATTAGCG CTAAGTAGCGTCGCTTCCTTT

Cele.WB235 Cele.WB235_5:16945467 5 16945467 16945572 106 5 21
TGCTAATTAG TGCTAATTAGCGCGGCTGCGT

Cele.WB235 Cele.WB235_5:17043954 5 17043954 17044434 481 12 40
AAAATTCTGG AAAATTCTGGAATGTTCTAGAGCCTTTTGGAAAATTTGAA

Cele.WB235 Cele.WB235_5:17086843 5 17086843 17087795 953 8 119
AACTGTGGGC
AACTGTGGGCTGGCTTTAATCCTAACCCAAGCAGAGCAGGATGTAGGCCTGAAACGTGGTTGTTATTCAATCAAGCCTAACAACCAGGTTTGAGGGCCGCAGGCCCGAAAACTCTTGGA

Cele.WB235 Cele.WB235_5:17308169 5 17308169 17308466 298 8 37
AAAACATTTT AAAACATTTTGGCGGGAATTCAAAATTTAATTTTTTG

Cele.WB235 Cele.WB235_5:17364710 5 17364710 17364818 109 9 12
CGCAACGCGA CGCAACGCGATA

Cele.WB235 Cele.WB235_5:17384240 5 17384240 17394505 10266 169 59
ATGGTGAATA ATGGTGAATATGGAAATTCAAGACTTCACTGGTTTTCAAAATTTTTAAAACCGAAAATT

Cele.WB235 Cele.WB235_5:17412072 5 17412072 17412375 304 16 19
TTTTCGTGTA TTTTCGTGTAACAATCTAT

Cele.WB235 Cele.WB235_5:17495404 5 17495404 17495538 135 5 11
CCTACAGTAC CCTACAGTACT

Cele.WB235 Cele.WB235_5:17522891 5 17522891 17524718 1828 7 255
AACAAGTCGG
AACAAGTCGGGAACGTTCGGAAAGCTCTGAGGAAAACTCCGACAGTATTATGCTTAAGTACGCAATTGAGCTATCTTTATTAGATTCGAAAGAGGACGCTTCAAAAATTGAAGCAATAACGAAGAAGCTTAAG
TTGGAACTGGCGAAGCTTAAATTGAGAATTACGCAGTCTTTATCAGATTCAAAAGACGACTCTTTGTCCAATGAAGCAAATCTGCAGAACGATCTACCTTCGACTTCTGATAGCAAAACTCC

Cele.WB235	Cele.WB235_5:17587204	5	17587204	17587343	140	10	12
ACGCGACACG	ACGCGACACGCA						
Cele.WB235	Cele.WB235_5:17619161	5	17619161	17619418	258	8	11
CCTACAGTAC	CCTACAGTACT						
Cele.WB235	Cele.WB235_5:17629258	5	17629258	17629682	425	26	16
CCAGGGTTCC	CCAGGGTTCCAGGGTT						
Cele.WB235	Cele.WB235_5:17629258	5	17629258	17630037	780	29	16
CCAGGGTTCC	CCAGGGTTCCAGGGTT						
Cele.WB235	Cele.WB235_5:17653226	5	17653226	17653322	97	6	16
GAATAATATT	GAATAATATTGGAGAA						
Cele.WB235	Cele.WB235_5:17747299	5	17747299	17748649	1351	6	143
CAGTGGGATT	CAGTGGGATTCTGAATTTATTCCAATCAAACGTCCGGGGTTCGAGTCCGCACAGGGGTATTTATTTTTTGCAAGCTGTGATTTACAAGTTTATAGCTGCAAAAGTGATTTTTATCGAATTTTTAGGAAAATGTCCGAATGGCA						
Cele.WB235	Cele.WB235_5:17780829	5	17780829	17781140	312	5	20
ATTTGACCTA	ATTTGACCTACTTTTTTAAA						
Cele.WB235	Cele.WB235_5:17817728	5	17817728	17818258	531	13	34
TCAAATTTTC	TCAGATTTTCTGAAAAAAAACTTTATCGGGAAAT						
Cele.WB235	Cele.WB235_5:17880208	5	17880208	17882252	2045	55	35
TCCGGCAAAT	TCCGGCAAATCAAAAATTTGCCGAAAATGAAAATT						
Cele.WB235	Cele.WB235_5:17885285	5	17885285	17887191	1907	37	44
TTGCCGGAAA	TTGCCGGAAATAGTAATTTTTGGCAAATTGCCGGTTTGCTGAAT						
Cele.WB235	Cele.WB235_5:17991128	5	17991128	17991485	358	5	35
AATTCCGGCA	ACTTCCGGCAAGTTGCCGATTTGCCGGAAATCTTC						
Cele.WB235	Cele.WB235_5:18042826	5	18042826	18043263	438	10	35
TTTCCGGCAA	TTTCCGGCAAATCGGCGATTTGCCTGAATTGAAAG						
Cele.WB235	Cele.WB235_5:18110272	5	18110272	18110517	246	7	35
AAATTTTCGG	AAATTTTCGGCAAATTCGATAATTGCCGGTTTTGT						
Cele.WB235	Cele.WB235_5:18169395	5	18169395	18171299	1905	61	31
GTCAATTTTT	GTCAATTTTTGGCGGGAAATTCAAATTTTCA						
Cele.WB235	Cele.WB235_5:18173031	5	18173031	18173742	712	10	78
GAGCTTTGGG	GAGCTTTGGGGCTTTTGGCTTGATCTCAGTCGACTTGATGGATTCTGGCTTGGGTTCTGCAGGCTTTTCGGGCTCCTT						
Cele.WB235	Cele.WB235_5:18201732	5	18201732	18201816	85	4	21
GCACTGACCA	GCACTGACCAAATGCAATGTT						
Cele.WB235	Cele.WB235_5:18314435	5	18314435	18314705	271	6	45
TTTGGAGCTG	TTTGGAGCTGAAGATTCAAGTCATAAGGGGGGACCCTTATGATCC						
Cele.WB235	Cele.WB235_5:18354225	5	18354225	18354524	300	14	14
ACAAACTACA	ACAAACTACAAACT						
Cele.WB235	Cele.WB235_5:18388249	5	18388249	18388686	438	22	19
CAAGGCACTA	CAAGGCACTAGAAATGCGT						
Cele.WB235	Cele.WB235_5:18389794	5	18389794	18390302	509	18	19
TGACGCAATT	TGACGCAATTCACGTGCCT						
Cele.WB235	Cele.WB235_5:18400360	5	18400360	18401785	1426	72	20
AAAAATAGAT	AAAAATAGATTGATACACGA						
Cele.WB235	Cele.WB235_5:18410594	5	18410594	18410720	127	9	14
GTCTGGGGTC	GTCTGGGGTCTGGG						
Cele.WB235	Cele.WB235_5:18415750	5	18415750	18416287	538	16	31
TTGGCCCAGA	TTGGCCCAGAGCTCTCTTGACCTAAGCCCCC						
Cele.WB235	Cele.WB235_5:18447331	5	18447331	18447583	253	9	28
CCAAGAGAAT	CCAAGAGAATTTCAACCGCCTAGAAATT						
Cele.WB235	Cele.WB235_5:18473949	5	18473949	18474108	160	4	32
GAATTCAAAT	GAATTCAAATTTTCTGAAAAAATTTTTGGCGT						

Cele.WB235	Cele.WB235_5:18475570	5	18475570	18475679	110	6	12
GTATATGTAT	GTATATGTATAT						
Cele.WB235	Cele.WB235_5:18536998	5	18536998	18537693	696	5	31
AAAATTTTGA	AAAATTTTGACCCAGAAATCGAAAAATTCGC						
Cele.WB235	Cele.WB235_5:18566411	5	18566411	18566663	253	16	12
AGGCTTAGGC	AGGCCTAGGCTT						
Cele.WB235	Cele.WB235_5:18573477	5	18573477	18573762	286	11	19
TTGACGCAAT	TTGACACAATTTTAGTACC						
Cele.WB235	Cele.WB235_5:18575312	5	18575312	18575616	305	15	19
TAGTGCCTTG	TAGTGCCTTGATGCAATTT						
Cele.WB235	Cele.WB235_5:18577906	5	18577906	18578272	367	11	33
TGTCGGCTGC	TGTCGGCTGCTAAAACTGTAGCCACCCGGCAGG						
Cele.WB235	Cele.WB235_5:18581399	5	18581399	18581759	361	22	12
AGCCTAAGCC	AGCCTAAGCCTA						
Cele.WB235	Cele.WB235_5:18584208	5	18584208	18585555	1348	50	20
GTAAATCTAC	GTAAATCTACATTGACATCC						
Cele.WB235	Cele.WB235_5:18588013	5	18588013	18588271	259	18	12
TAAGCCTAAG	TAAGCCTAAGCC						
Cele.WB235	Cele.WB235_5:18597908	5	18597908	18598040	133	9	12
AGCCTAAGCC	AGCCTAAGCCTA						
Cele.WB235	Cele.WB235_5:18616302	5	18616302	18616398	97	6	12
CTAAGCCTAA	CTAATCCTAAGC						
Cele.WB235	Cele.WB235_5:18619123	5	18619123	18619731	609	29	19
GTGCCTTGAC	GTGCTTTGACGCAATTCTA						
Cele.WB235	Cele.WB235_5:18632123	5	18632123	18632283	161	8	20
GATTTTTCTG	GATTTTTTTGAAAATTTATT						
Cele.WB235	Cele.WB235_5:18632668	5	18632668	18632722	55	4	12
TAAGCCTAAG	TAAGCCTAGGCC						
Cele.WB235	Cele.WB235_5:18636764	5	18636764	18637321	558	15	39
GTAATTTATA	GTAATTTATAATTTTTTAAAGATGAATAATAGATTGCCC						
Cele.WB235	Cele.WB235_5:18639700	5	18639700	18639844	145	9	12
GCTTAGGCTT	GCTTCGGCTTAG						
Cele.WB235	Cele.WB235_5:18641364	5	18641364	18641663	300	5	11
TTTCAGGCCT	TTTAGGCCTAC						
Cele.WB235	Cele.WB235_5:18644347	5	18644347	18644921	575	24	21
AAAAATCGAT	AAAAATCGATAAAATATGGAG						
Cele.WB235	Cele.WB235_5:18666434	5	18666434	18666663	230	6	35
GGAAATTCAA	GAAAATTCAAATTTTCTGAGAAAATTTTTTTTGGC						
Cele.WB235	Cele.WB235_5:18675402	5	18675402	18675828	427	12	20
AGAAAAATCA	AGAAAAATCAATAAATTTTT						
Cele.WB235	Cele.WB235_5:18676328	5	18676328	18676917	590	23	19
TGACGCAATT	TGACGCATTTTTAGTGCCT						
Cele.WB235	Cele.WB235_5:18677260	5	18677260	18677830	571	26	19
ACTAGAATTG	ACTAGAAGTGCGCCAATAC						
Cele.WB235	Cele.WB235_5:18678760	5	18678760	18678838	79	6	12
GGCTCAGGCT	GGCTCCGGCTCA						
Cele.WB235	Cele.WB235_5:18684513	5	18684513	18684741	229	8	19
TTCTAGTGCC	TTCTAGTGCCTTGGCGCTT						
Cele.WB235	Cele.WB235_5:18722989	5	18722989	18723101	113	7	12
AGCCTGAGCC	AGCCTGAGCCTG						
Cele.WB235	Cele.WB235_5:18727688	5	18727688	18728599	912	46	20
AAAAATAGAT	AAAAATAGATTGTTACACGA						

Cele.WB235	Cele.WB235_5:18737977	5	18737977	18738195	219	6	31
TGTAAAAATG	TGAAAAAATGTCTGAAACTTCCAATTTACAA						
Cele.WB235	Cele.WB235_5:18777092	5	18777092	18777231	140	4	35
TCAGAAAATT	TCAGAAAATTTAAATTTCCCTCCAAAATTTTTTTC						
Cele.WB235	Cele.WB235_5:18797535	5	18797535	18797724	190	5	24
GCCTAAGCCT	GCCTAAACCTAACCCCGAGTCTAG						
Cele.WB235	Cele.WB235_5:18855835	5	18855835	18856039	205	6	31
TCGTTTTTTC	TCGTTTTTTCATGAAAAAACTCAATTTTCCG						
Cele.WB235	Cele.WB235_5:18865200	5	18865200	18865555	356	7	40
GAATTTTTTG	GAATTTTTTGCAATTTTATTGAATTTTCCAAATTTTCAGC						
Cele.WB235	Cele.WB235_5:18879684	5	18879684	18879863	180	5	40
TTTTGCCGAA	TTTTACCGAAAATTGCAAATTTTGTGGAATGTATCACATT						
Cele.WB235	Cele.WB235_5:18881944	5	18881944	18883813	1870	74	17
ATTAGCAGCG	TTAGCAGCGACCAAAAA						
Cele.WB235	Cele.WB235_5:18886295	5	18886295	18887294	1000	12	18
AATTAGCAGC	AATTAGCAGCGACCAAAA						
Cele.WB235	Cele.WB235_5:18888872	5	18888872	18889346	475	14	32
TTGTAGTTTG	TTGTAGTTTGTAGTCTAAGCAGCGCCCAAAAC						
Cele.WB235	Cele.WB235_5:18940941	5	18940941	18941109	169	8	21
ATTATTGATT	ATTATTGATTTTTCAAGAAAA						
Cele.WB235	Cele.WB235_5:18964514	5	18964514	18964623	110	6	18
AGCAGCGCCC	AGCAGCGCCCAAAAAAAA						
Cele.WB235	Cele.WB235_5:18966918	5	18966918	18967045	128	5	18
GCAGCGCCCA	GCAGCGCCCAAAAGATTA						
Cele.WB235	Cele.WB235_5:18970132	5	18970132	18970454	323	18	18
TGCTAATTTT	TGCTAATTTTTTTGGCGC						
Cele.WB235	Cele.WB235_5:18979816	5	18979816	18980485	670	37	18
GCGCTGCTAA	GCGCTGCTAATTTTTTGG						
Cele.WB235	Cele.WB235_5:18984459	5	18984459	18985309	851	31	20
ATTTATTGAT	ATTTATCGATTTTTCTGTAA						
Cele.WB235	Cele.WB235_5:18986276	5	18986276	18986397	122	6	20
ATAAATTTTC	ATAAATTTTCAGAAAAATCG						
Cele.WB235	Cele.WB235_5:18987130	5	18987130	18987201	72	4	18
TAGCAGCGCC	TAGCAGCGCCCAAGAAAT						
Cele.WB235	Cele.WB235_5:18989479	5	18989479	18989970	492	14	32
TGTAGTTTGT	TGTAGTTTGTAGTTTAGCAGCGCCCAAAATTG						
Cele.WB235	Cele.WB235_5:18993608	5	18993608	18993851	244	8	20
TATCGATTTT	TATCGATTTTTCTGCAAATT						
Cele.WB235	Cele.WB235_5:19022623	5	19022623	19023690	1068	10	19
TGGCGCATTT	TGGCGCGTTTTCGGTGTGT						
Cele.WB235	Cele.WB235_5:19026746	5	19026746	19027590	845	33	20
AAAATCGATA	ATAATCGATAAATTCCCAGA						
Cele.WB235	Cele.WB235_5:19038887	5	19038887	19039001	115	9	12
ATACATATAC	ATACATATACAT						
Cele.WB235	Cele.WB235_5:19043595	5	19043595	19043791	197	8	12
ATATGTATAT	ATATGTATATGT						
Cele.WB235	Cele.WB235_5:19047920	5	19047920	19048249	330	23	14
TATATGTTAT	TATATGTTATATGT						
Cele.WB235	Cele.WB235_5:19052161	5	19052161	19052388	228	7	33
GGAATTCAAA	GGAATTCAAATTTCTTCAAAAAAAATTTTGGCG						
Cele.WB235	Cele.WB235_5:19065856	5	19065856	19066708	853	32	26
TGAAATTTCA	TGAAATTTCACAAAATATCGATTTTC						

Cele.WB235	Cele.WB235_5:19099300	5	19099300	19100374	1075	31	20
AAAAATCAAT	AAAAATCAATCAATTTCCGA						
Cele.WB235	Cele.WB235_5:19100422	5	19100422	19100868	447	10	20
ATTGATTTTT	ATTGATTTTTCTGAAAATTT						
Cele.WB235	Cele.WB235_5:19101640	5	19101640	19102041	402	17	20
CAGAAAAATC	CAGAAAAATCGATAAATTTT						
Cele.WB235	Cele.WB235_5:19106243	5	19106243	19106368	126	6	18
ATTAGCAGCG	ATTAGCAGCGACCAAAAA						
Cele.WB235	Cele.WB235_5:19112578	5	19112578	19114304	1727	66	20
TATCGATTTT	TATTGATTTTTCTGAAAATT						
Cele.WB235	Cele.WB235_5:19117969	5	19117969	19118122	154	5	33
TTAGCAGCGC	TTAGCAGCGCCCAAAAACTTGTAGTTTGTAGTC						
Cele.WB235	Cele.WB235_5:19121195	5	19121195	19121753	559	25	20
AATTTATTGA	AATTTATTGATTTTTCCGGA						
Cele.WB235	Cele.WB235_5:19122509	5	19122509	19124474	1966	65	33
AGCAGCGCCC	AGCAGCGCCCAAAATCTTGTAGTTTGTAGTCTT						
Cele.WB235	Cele.WB235_5:19129872	5	19129872	19130673	802	34	20
GATTTTTCTG	GATTTTTCTGAAAATATATC						
Cele.WB235	Cele.WB235_5:19148365	5	19148365	19148726	362	16	19
TGGCGCATTT	TGGCGCATTTCTTGAGCCT						
Cele.WB235	Cele.WB235_5:19193880	5	19193880	19195207	1328	74	18
GCTGCTAATT	GCTGCTAATTTTTTGGGC						
Cele.WB235	Cele.WB235_5:19229130	5	19229130	19229634	505	37	12
AGGCTCAGGC	AGGCTCAGGCTC						
Cele.WB235	Cele.WB235_5:19232598	5	19232598	19232808	211	15	12
GCCTAAGCCT	GCCTGAGCCTGA						
Cele.WB235	Cele.WB235_5:19261027	5	19261027	19261174	148	7	21
CACACGAGCT	CACACGATCTTCAGATTCCAC						
Cele.WB235	Cele.WB235_5:19278568	5	19278568	19278735	168	5	29
CTCGTCAGTG	CTCGTCAGTGGGTGGACGAGGAATGAGCG						
Cele.WB235	Cele.WB235_5:19315501	5	19315501	19315626	126	4	22
GTGCGCAGAA	GTGCGCAGAATCTGCTCAGGGG						
Cele.WB235	Cele.WB235_5:19354949	5	19354949	19355211	263	11	21
TCTAATTAGA	TCAAATTAGATTACCTTTTTT						
Cele.WB235	Cele.WB235_5:19356787	5	19356787	19357065	279	13	21
TTCTAATTAG	TTCTAATTAGATGCGCATTCC						
Cele.WB235	Cele.WB235_5:19372325	5	19372325	19372591	267	11	19
GATCCTTGGC	GATCCTTAGCGCTTTTTTT						
Cele.WB235	Cele.WB235_5:19373212	5	19373212	19373421	210	9	19
TTGCGCCAAG	TTGCGTCAAGACACTAAAA						
Cele.WB235	Cele.WB235_5:19376610	5	19376610	19376889	280	9	31
CGGGAAATTC	CGGGAAATTCAAATTTTCAGTTAATTTTTGT						
Cele.WB235	Cele.WB235_5:19379891	5	19379891	19380043	153	5	19
AGAATTGCGC	AGAATTGCGCCAAGGCACG						
Cele.WB235	Cele.WB235_5:19380090	5	19380090	19380527	438	16	19
GCCTTGACGC	GCCTTGACGCAATTCTAGT						
Cele.WB235	Cele.WB235_5:19402253	5	19402253	19402490	238	14	17
GAATTAGAAA	GAATTAGAAAACTTTGA						
Cele.WB235	Cele.WB235_5:19420419	5	19420419	19420546	128	5	25
AAAAATCTGT	AAAAATCTGTTCTTCGAGGTCCCTA						
Cele.WB235	Cele.WB235_5:19427400	5	19427400	19427634	235	7	22
ACGTATACAG	ACGTATACAGGCATATACACAT						

Cele.WB235 Cele.WB235_5:19427290 5 19427290 19427806 517 5 21
TATTTTCAAA TATTTTCAAAAAAATTTAAAA
Cele.WB235 Cele.WB235_5:19466947 5 19466947 19467275 329 4 82
CCCCGAACGT CCCCGAACGTGTCGGCCGCTTCCAAATAACCACCTCCTTGCACTACGTTGCGCACACACTCATTTCACGCCAAGCTGCGGAA
Cele.WB235 Cele.WB235_5:19487887 5 19487887 19488024 138 6 23
TTTTCAGAAA TTTTCAGAAATAGACATTTCAGA
Cele.WB235 Cele.WB235_5:19488506 5 19488506 19489530 1025 58 17
TTGCAAGAAA TTGCAAGAAAATCTGCA
Cele.WB235 Cele.WB235_5:19495245 5 19495245 19495315 71 5 10
TGGCGAAAGC TGGCGAAAGC
Cele.WB235 Cele.WB235_5:19495335 5 19495335 19495400 66 5 13
TTGGGGTGGG TTGGGGTGGGCCT
Cele.WB235 Cele.WB235_5:19498459 5 19498459 19499190 732 4 183
TTTCTCTGCG
TTTCTCTGCGTCTCGCCTTGGCTCACAGTATTTTTCACACCAAACTTCGAGGCTATGTATCTTTGCAACGGGTTGAGATATTGAAAAATTTCCAACTAACAAAATGCTCTAAAATTTATTCTGTATCTTGTGA
AATAAACTTTAAAACCCTAGGCAAATCGCAAACAGCGAAATACACCCAAG
Cele.WB235 Cele.WB235_5:19517965 5 19517965 19518323 359 22 12
CTTAGGCTTA CTTGGGCTTAGG
Cele.WB235 Cele.WB235_5:19528638 5 19528638 19528776 139 11 12
CTTAGGCTTA CTTAGGCTTAGG
Cele.WB235 Cele.WB235_5:19535226 5 19535226 19535475 250 6 31
AATTTCCCGC AATTTCCCGCTAAAATTTAACAGAAAATTTA
Cele.WB235 Cele.WB235_5:19550939 5 19550939 19551875 937 7 14
TTTGTAGTTT TTTGTAGTTTGTGT
Cele.WB235 Cele.WB235_5:19551915 5 19551915 19552281 367 11 14
ACAAACTACA ACAAACTACATACT
Cele.WB235 Cele.WB235_5:19555359 5 19555359 19555663 305 8 27
GCAGCCGACA GCAGCCGACACCTCACAGGTCCCTGTA
Cele.WB235 Cele.WB235_5:19615691 5 19615691 19616051 361 8 31
TTCAAATTTT TTCAAATTTTCAGTCAATTTTTGGCGGGAGT
Cele.WB235 Cele.WB235_5:19630875 5 19630875 19631274 400 12 31
GGCGGGAAAT GGCGGGAAATTCAAATTTTCAGTCAATTTTT
Cele.WB235 Cele.WB235_5:19645096 5 19645096 19645264 169 6 21
ACCCCGATTT ACTCCGATTTTCATCCAAAAA
Cele.WB235 Cele.WB235_5:19679307 5 19679307 19679396 90 4 22
ATTGGATTTT ATTGGATTTTTCACCCCAAAAA
Cele.WB235 Cele.WB235_5:19689942 5 19689942 19690431 490 10 31
AAAATTTGAA AAAATTTGAATTTCCCGCCAAAAATTGACTG
Cele.WB235 Cele.WB235_5:19753320 5 19753320 19753624 305 19 16
CTTAGATCTC CTTAGATCTCAATTCA
Cele.WB235 Cele.WB235_5:19755742 5 19755742 19756252 511 14 34
TTTCCCGCCA TTTCCCGCCAAAATAATTTCTCCGAAAATTTGTA
Cele.WB235 Cele.WB235_5:19762622 5 19762622 19764008 1387 40 35
CCGGCAAATC CCGGCAAATCGACAAATTGCCGGATTTGTAAATTT
Cele.WB235 Cele.WB235_5:19795837 5 19795837 19797364 1528 40 35
GAAATTTTCA GAAATTTTCATTTCCGGCAAATTACTGATCTGCCG
Cele.WB235 Cele.WB235_5:19799341 5 19799341 19799769 429 12 35
AATTTCCGGC AATTTCCGGCAATACGGCAAATTACCAGAATTAAA
Cele.WB235 Cele.WB235_5:19801650 5 19801650 19801767 118 5 12
AAGCCTAAGC AAGCCTAATCTT
Cele.WB235 Cele.WB235_5:19821185 5 19821185 19822021 837 37 19
TAGTGCCTTG TAGTGCATTGGCGCAATTC

Cele.WB235	Cele.WB235_5:19863556	5	19863556	19864754	1199	28	31
TTCCCGCCTA	TTCCCGCCTAAAACTACTCGAAAATTCAAAT						
Cele.WB235	Cele.WB235_5:19866564	5	19866564	19867301	738	21	33
TTTGAATTTC	TTTGAATTTCCCACCAAATATTTTCTCAAAAAT						
Cele.WB235	Cele.WB235_5:19901870	5	19901870	19902110	241	10	20
TTATTGATTT	TTATTGATTTTTCTGGGAAT						
Cele.WB235	Cele.WB235_5:19907460	5	19907460	19907660	201	9	20
CAGAAAAATC	CAGAAAAATCAATAAATTTT						
Cele.WB235	Cele.WB235_5:19909526	5	19909526	19910097	572	12	20
TTATTGATTT	TTATCGATTTTTATGGAAAT						
Cele.WB235	Cele.WB235_5:19933050	5	19933050	19934036	987	29	32
ACTACAAACT	ACTACAAACTACAAGTTTTTTGGCGCTGCTAA						
Cele.WB235	Cele.WB235_5:19935725	5	19935725	19935960	236	11	20
TTTGAATTTT	TTTGAATTTTTGAGTGAAAA						
Cele.WB235	Cele.WB235_5:19957566	5	19957566	19957879	314	8	12
TATGTATATG	TATGTATATGTA						
Cele.WB235	Cele.WB235_5:19963720	5	19963720	19963804	85	4	21
CTCAATTCAA	CTCAATTCAATCAAGGAAATT						
Cele.WB235	Cele.WB235_5:19966309	5	19966309	19966447	139	11	12
ATATACATAT	ATATACATATAC						
Cele.WB235	Cele.WB235_5:19993167	5	19993167	19993682	516	14	32
AAATTTGAAT	AAATTTGAATGTTCGCCACAATTTTTCAAAAC						
Cele.WB235	Cele.WB235_5:20001413	5	20001413	20002864	1452	51	12
GCTTGGGCTT	GCTTGGGCTTAG						
Cele.WB235	Cele.WB235_5:20101712	5	20101712	20102976	1265	44	32
TTTTGGGCGC	TTTTGGGCGCTGCTTAGACTATAAACTACAAT						
Cele.WB235	Cele.WB235_5:20153934	5	20153934	20154193	260	7	37
AATTTGAATT	AATTTGAATTCCCGCCAAAACATTTTTGTAAAATTAA						
Cele.WB235	Cele.WB235_5:20210955	5	20210955	20211513	559	29	18
CATTGTCACT	CATTGTCACTGGCTTCTC						
Cele.WB235	Cele.WB235_5:20236849	5	20236849	20237152	304	5	41
GCATTTTTCG	GCATTTTTCGCCGATTTTTGATTAATTTTCAGTTAAAATTC						
Cele.WB235	Cele.WB235_5:20246749	5	20246749	20247078	330	11	30
CTCAAATTTG	CTCAAATTTGCACTGAAAATTCCAATTATC						
Cele.WB235	Cele.WB235_5:20260289	5	20260289	20260916	628	16	11
CTACAGTACT	CTACAGTACTT						
Cele.WB235	Cele.WB235_5:20263899	5	20263899	20264125	227	7	32
TTAGCAGCGC	TTAGCAGCGCCCAAAATCTTGTAGTTTGTAGT						
Cele.WB235	Cele.WB235_5:20268029	5	20268029	20268566	538	15	31
CTACAAACTA	CTACAAACTACTAGTTTTGGGCGCTGCTAAG						
Cele.WB235	Cele.WB235_5:20273044	5	20273044	20275266	2223	68	31
GGCGCTGCTA	GGCGCTGCTAAACTACAAACTACAAGAATTG						
Cele.WB235	Cele.WB235_5:20315897	5	20315897	20316651	755	12	42
ATTGCCGGTT	ATTGCCGGTTTTGAACATTTTCGGCAATCGGCAATTTCGGCA						
Cele.WB235	Cele.WB235_5:20406247	5	20406247	20406428	182	9	20
AAAATTCAAA	AAAATTCAAATTTTTGACTG						
Cele.WB235	Cele.WB235_5:20407384	5	20407384	20407602	219	4	31
GAAAAAGTGC	GAAAAAGTGCTGAAAATCACATTTTTGAACT						
Cele.WB235	Cele.WB235_5:20471611	5	20471611	20471894	284	9	32
CCGCCAAAAT	CCGCCAAAATTTTTTAAAGAAAATATGAATTC						
Cele.WB235	Cele.WB235_5:20494724	5	20494724	20495104	381	15	20
TTTCACTAAA	TTTCACTAAAAACTTCAAAT						

Cele.WB235	Cele.WB235_5:20524563	5	20524563	20524679	117	5	16
GTACTAGGGT	GTACTAGGGTACTAGG						
Cele.WB235	Cele.WB235_5:20588792	5	20588792	20588997	206	5	11
CCTACAGTAC	CCTACAGTACT						
Cele.WB235	Cele.WB235_5:20605796	5	20605796	20606242	447	40	11
GTTGCGGTTG	GTTGCGGTTGC						
Cele.WB235	Cele.WB235_5:20705245	5	20705245	20705371	127	4	32
GTGCGTCCGG	GTGCGTCCGGCGTCCGGAAAAAGCGGACGTGT						
Cele.WB235	Cele.WB235_5:20839492	5	20839492	20839569	78	5	15
CGAAAAGGGG	CGAAAAGGGGATCTG						
Cele.WB235	Cele.WB235_5:20839745	5	20839745	20839822	78	5	15
CCCCTTTTCG	CCCCTTTTCGTATCT						
Cele.WB235	Cele.WB235_5:20923994	5	20923994	20924168	175	14	12
GCTTAGGCTT	GCTTAGGCTTAG						
Cele.WB235	Cele.WB235_6:0	6	0	251	252	21	12
CTAAGCCTAA	CTAAGCCTAAGC						
Cele.WB235	Cele.WB235_6:28398	6	28398	28532	135	5	11
GTACTGTAGT	GTACTGTAGTA						
Cele.WB235	Cele.WB235_6:109717	6	109717	114226	4510	167	27
CACAGAGAGT	CACAGAGAGTAAAATATAGTGGGAAGC						
Cele.WB235	Cele.WB235_6:142149	6	142149	142814	666	19	35
GCGGGAAATT	GCGGGAAATTCAAATTTTTAGTGAAAAATATTTTG						
Cele.WB235	Cele.WB235_6:142874	6	142874	143634	761	19	40
GAAAATTCTG	GAAAATTCTGGAATGTTCCAGAACTTTCTAGAAAAATCGG						
Cele.WB235	Cele.WB235_6:161009	6	161009	161190	182	5	32
TGTAGTTTGT	TGTAGTTTGTAGTGTTAGCAGCGCCCAAAACT						
Cele.WB235	Cele.WB235_6:161586	6	161586	161691	106	7	15
GTCTCACCAC	GTCTCACCACGAAGG						
Cele.WB235	Cele.WB235_6:168652	6	168652	168890	239	6	40
TTCCAGAATT	TTCCAGAATTTTCTCGATTTTTCTAGAAAGTTCTGGAACA						
Cele.WB235	Cele.WB235_6:169222	6	169222	169627	406	27	15
ACGATGGGTC	ACGATGGGTCTCACC						
Cele.WB235	Cele.WB235_6:188398	6	188398	190104	1707	53	32
CGCTGCTAAG	CGCTGCTAAGACTACAAACTACAAGTTTTGGG						
Cele.WB235	Cele.WB235_6:190303	6	190303	190811	509	10	20
AAAAATCAAT	AAAAATCAATAAATTTCCAA						
Cele.WB235	Cele.WB235_6:209349	6	209349	209653	305	8	36
ATAAAAATTT	ATAAAAATTTCCGGCAAACCGGCAATTTGTCAGAAA						
Cele.WB235	Cele.WB235_6:256843	6	256843	256905	63	4	15
CGAAAAGGGG	CGAAAAGGGGATCTG						
Cele.WB235	Cele.WB235_6:257079	6	257079	257156	78	5	15
TCCCCTTTTC	TCCCCTTTTCGTAGA						
Cele.WB235	Cele.WB235_6:311553	6	311553	312211	659	7	94
GCAGTAATTC	GCAGTAATTCTCAAAATCTTCTGTTCTCTAAATATTGGGTGCTTTTCGATGCCCTTTGTAGACAAATCAATGAGAAAATTGTCAATTTCTGAAG						
Cele.WB235	Cele.WB235_6:316974	6	316974	317093	120	8	14
ATAGCGTATA	ATAGCGTATAGCGT						
Cele.WB235	Cele.WB235_6:404178	6	404178	404266	89	7	11
CATAACTGAG	CATAACTGAGA						
Cele.WB235	Cele.WB235_6:443181	6	443181	444059	879	19	40
AAAATTCTGG	AAAATTCTGGAAAGCTCTAGAACCTTCTGAAAAATTCGAG						
Cele.WB235	Cele.WB235_6:516173	6	516173	516819	647	15	15
CCACGATGGG	CCACGGTGGGTCTCA						

Cele.WB235	Cele.WB235_6:516878	6	516878	517017	140	5	30
AATTTTCCAG	AATTTTCCAGAAGGTTCTGGAACATTCCAG						
Cele.WB235	Cele.WB235_6:703442	6	703442	703568	127	5	21
GGTCAGTGCA	GGTCAGTGCATTACCAATGTT						
Cele.WB235	Cele.WB235_6:723810	6	723810	724054	245	5	22
TGGTCAGTGC	TGGTCAGAGCTGGCGGCCAATC						
Cele.WB235	Cele.WB235_6:863878	6	863878	863962	85	4	21
ACTGACCAAA	ACTGACCAAAAATAGACATGC						
Cele.WB235	Cele.WB235_6:920806	6	920806	920909	104	5	20
ATGCACTGAC	ATGCACTGACCGAAAATATG						
Cele.WB235	Cele.WB235_6:943488	6	943488	943966	479	11	15
CACGATGGGT	CACGATGGGTCTCGT						
Cele.WB235	Cele.WB235_6:944053	6	944053	944332	280	13	11
CTACAGTACT	CTACAGTACCT						
Cele.WB235	Cele.WB235_6:954503	6	954503	954587	85	4	21
CAGTGCATTC	CAGTGCATTCAGAACTTTGAT						
Cele.WB235	Cele.WB235_6:954523	6	954523	954714	192	6	21
TCAGTGCATT	TCAGTGCATTCCGAACTTTCG						
Cele.WB235	Cele.WB235_6:988793	6	988793	988877	85	4	21
TGGTCAGTGC	TGGTCAGTGCAAGAAACAATT						
Cele.WB235	Cele.WB235_6:1008023	6	1008023	1008695	673	4	21
TGACAAAACA	TGACAAAACATAACTTTGCAC						
Cele.WB235	Cele.WB235_6:1016701	6	1016701	1016827	127	6	21
GTCAGTGCAT	GTCAGTGCATTGATAGTTTCT						
Cele.WB235	Cele.WB235_6:1022677	6	1022677	1022779	103	5	20
TTGGTCAGTG	TTGGTCAGTGCATTTTAGTC						
Cele.WB235	Cele.WB235_6:1058514	6	1058514	1058619	106	4	21
GTCAGTGCAA	GTCAGTGTAATTAGAATAATG						
Cele.WB235	Cele.WB235_6:1062534	6	1062534	1062618	85	4	21
GTCAGTGCAA	GTCAGTGCAAATTCAAATATT						
Cele.WB235	Cele.WB235_6:1094209	6	1094209	1094314	106	5	21
TTGGTCAGTG	TTGGTCAGTGCGATTTTTTAA						
Cele.WB235	Cele.WB235_6:1136184	6	1136184	1140765	4582	45	102
TGTACACTTC	TGTACACTTCTCCCAAAATGACTGTAAGAATGGACTCCTTATATGATTTTAAACCCGACTCTGGACATACAAACTTCTCTCAAAAAGAAAATAGACTTCACA						
Cele.WB235	Cele.WB235_6:1214446	6	1214446	1215096	651	6	21
GTCAGTGCAA	GTCAGTGCAAAAGTATTTTTG						
Cele.WB235	Cele.WB235_6:1278787	6	1278787	1279105	319	7	22
GTCAGTGCAA	GTCAGTGCAAATTGTGCACATG						
Cele.WB235	Cele.WB235_6:1292997	6	1292997	1293291	295	14	21
TTTGCACTGA	TTTGCACTGACCAACACGAAT						
Cele.WB235	Cele.WB235_6:1296886	6	1296886	1296996	111	5	22
TCGCACTGAC	TCGCACTGACCAAACTTAGAAA						
Cele.WB235	Cele.WB235_6:1320585	6	1320585	1320992	408	5	21
TTGCACTGAC	TTGCACTGACCAAAATTAAGC						
Cele.WB235	Cele.WB235_6:1325710	6	1325710	1325901	192	7	21
TTGCACTGAC	TTGAACTGACAAAGTGCAAAT						
Cele.WB235	Cele.WB235_6:1334194	6	1334194	1334262	69	4	17
TGGTCAGTGC	TGGTCAGTGCAAAAAGT						
Cele.WB235	Cele.WB235_6:1375049	6	1375049	1375406	358	9	40
ATTTTTCTAG	ATTTTTCTAGAAAGTTCTGGAACACTCCAGAATTTTCTCG						
Cele.WB235	Cele.WB235_6:1375505	6	1375505	1376170	666	19	35
CCCGCCAAAA	CCCGCCAAAATATTTTTCACAGAAAAATTAAATTT						

Cele.WB235	Cele.WB235_6:1376205	6	1376205	1376549	345	23	15	CGATGGGTCT	CGATGGGTCTCACCT
Cele.WB235	Cele.WB235_6:1376606	6	1376606	1376746	141	5	30	AATTTTCCAG	AATTTTCCAGAAAGTTCTGGAACATTCCAG
Cele.WB235	Cele.WB235_6:1376859	6	1376859	1377239	381	13	11	GTACTCCTAC	GTACTCCTACA
Cele.WB235	Cele.WB235_6:1508523	6	1508523	1508943	421	18	20	AGGCAGACCT	AGGCAGACCTAGCCTACCCT
Cele.WB235	Cele.WB235_6:1509783	6	1509783	1509855	73	5	12	CTAAGCCTAA	CTAAGCCAAAGA
Cele.WB235	Cele.WB235_6:1524262	6	1524262	1524376	115	5	23	AAAAAATTTG	AAAAAATTTGTTTCGCACTGAAC
Cele.WB235	Cele.WB235_6:1544700	6	1544700	1544784	85	4	21	TCTGAGCACT	TCTGAGCACTGACCAAAATTA
Cele.WB235	Cele.WB235_6:1548656	6	1548656	1549346	691	19	30	ACTACCCAAC	ACTACCCAACCGGCTCCGGCAACTAGTCCG
Cele.WB235	Cele.WB235_6:1549825	6	1549825	1550305	481	5	108	ATCCTCAACA	ATCCTCAACACCATCTGCATCATCTAGCTCTTCCGGAACGATGTCGACTATTTCAGGATCAACTGGTTCCACAGTTACAGTCGTGCCTGGATCCTCTAGCACATTTGC
Cele.WB235	Cele.WB235_6:1610992	6	1610992	1611280	289	10	11	GTACTGTAGG	GTACTGTAGTA
Cele.WB235	Cele.WB235_6:1636150	6	1636150	1638568	2419	93	26	GGTGAACGGT	GGTGAACGGTCAGAGTCACTATTTTT
Cele.WB235	Cele.WB235_6:1642568	6	1642568	1643478	911	35	26	GACTCTAACC	GACTCTAACCATTCACCTAAAAATAT
Cele.WB235	Cele.WB235_6:1643531	6	1643531	1644396	866	34	11	GGAGCGGTTT	GGAGCGGTTTT
Cele.WB235	Cele.WB235_6:1644418	6	1644418	1644678	261	10	26	GCCGCAATGC	GCCGCAATGCCATTTTTCTGAAATCC
Cele.WB235	Cele.WB235_6:1718471	6	1718471	1719647	1177	4	165	CTTTTCCAAA	CTTTTCCAAAATGGCAATTTTTGATGAAAAAAATCCGAATTTTCACAAATTTCAAAAAAATTTTTAAACTTTATAATTTTAGCTTTGAATATTCCAAATGGAACTGTCATAACCTATCAATTTGATATATAAACTCGTGCCTACCTTCAGGCATTCAGTTTTCAC
Cele.WB235	Cele.WB235_6:1752278	6	1752278	1755168	2891	107	27	ACTCTCTGTG	ACTCTCTGTGGCTTCCCACCATATTTT
Cele.WB235	Cele.WB235_6:1836329	6	1836329	1837109	781	39	18	AAGCCTATGC	AAGCCTATGCCGAAGCCG
Cele.WB235	Cele.WB235_6:1850672	6	1850672	1850839	168	11	14	TAGGCCCTCA	TAGGCCCTCAAGCG
Cele.WB235	Cele.WB235_6:1916196	6	1916196	1916614	419	14	22	CCGGCGTGGT	CCGGCGTGGTTTGAAGCCGACA
Cele.WB235	Cele.WB235_6:1945521	6	1945521	1946157	637	32	20	GTTACACGAA	GTTACACGAAAAAAATAGATT
Cele.WB235	Cele.WB235_6:1954050	6	1954050	1954308	259	13	20	GTTACACGAA	GTTACACGAAAAAAATAGATT
Cele.WB235	Cele.WB235_6:1980629	6	1980629	1980811	183	4	46	TGGTAAATTG	TGGTAAATTGCTTCCCGGATCACGCACTTTTCTTATTTCCTAACTA
Cele.WB235	Cele.WB235_6:2002759	6	2002759	2003439	681	17	40	GAATTTTCTC	GAATTTTCTCGATTTTTCTAGAAAGTTCTGGAACATTCCA
Cele.WB235	Cele.WB235_6:2004050	6	2004050	2004599	550	27	19	ATATTTATTT	ATATTTATTTATTTAATTA
Cele.WB235	Cele.WB235_6:2010907	6	2010907	2011305	399	28	12	AATAAATAAC	AATAAATAAATA

Cele.WB235	Cele.WB235_6:2055472	6	2055472	2055647	176	7	25
TATATTTTAC	TATATTTTACTCTCTGGCATCACAC						
Cele.WB235	Cele.WB235_6:2056950	6	2056950	2057275	326	12	25
TCTGGCTTCA	TCTGGCTTCACAGTAAAATTTACTA						
Cele.WB235	Cele.WB235_6:2057847	6	2057847	2058368	522	13	40
TTTTCTCGAT	TTTTCTCGATATTTCTAGAAAGTTCTGGAACATTCCAGAA						
Cele.WB235	Cele.WB235_6:2057868	6	2057868	2059356	1489	20	40
GTTCTGGAAC	GTTCTGGAACATTCCAGAATTTTCTCGATATTTCTAGAAA						
Cele.WB235	Cele.WB235_6:2263547	6	2263547	2263756	210	16	11
CCATACCTAT	CCATACCTATA						
Cele.WB235	Cele.WB235_6:2307188	6	2307188	2307299	112	4	32
CGACCTGCGA	CGACCTGCGACCTAGAGGGTTAGGTCGCATCG						
Cele.WB235	Cele.WB235_6:2379254	6	2379254	2380684	1431	55	26
TTTTAGTGAA	TTTTAGTGAACTGCCAGAGTCACTAA						
Cele.WB235	Cele.WB235_6:2563170	6	2563170	2563230	61	5	12
AGGCTTAGGC	AGGCTTAGGCTA						
Cele.WB235	Cele.WB235_6:2989994	6	2989994	2990074	81	5	16
AGAGAGTATA	AGAGAGTATAGATTAG						
Cele.WB235	Cele.WB235_6:2991583	6	2991583	2991919	337	7	48
AAGATGAGGA	AAGATGAGGAATTTAATTTTTGATTTGCCCGTAATTTATATTTTTCTG						
Cele.WB235	Cele.WB235_6:3007216	6	3007216	3008016	801	42	19
GTGTAACAAT	GTGTAACAATCTATTTTTC						
Cele.WB235	Cele.WB235_6:3075483	6	3075483	3075588	106	7	15
TCACCACGAT	TCACCACGATGGGTC						
Cele.WB235	Cele.WB235_6:3261930	6	3261930	3262051	122	11	11
AAAATGTGCT	AAAATGTGCTG						
Cele.WB235	Cele.WB235_6:3338986	6	3338986	3339541	556	27	18
TTTTGTCAGT	TTTTGTCAGTGCTATAGT						
Cele.WB235	Cele.WB235_6:3347373	6	3347373	3347577	205	10	17
TTTTAACGTG	TTTTAACGTGACCTAGT						
Cele.WB235	Cele.WB235_6:3365934	6	3365934	3366110	177	12	11
TAGCTCAGCT	TAGCTCAGCTT						
Cele.WB235	Cele.WB235_6:3392053	6	3392053	3392273	221	10	18
TTGTCAGTGC	TTGTCAGTGCTATGGTTT						
Cele.WB235	Cele.WB235_6:3631392	6	3631392	3631893	502	43	11
CAGTTGTCAC	CAGTTGTCACC						
Cele.WB235	Cele.WB235_6:3693989	6	3693989	3694090	102	5	20
TTTGGTCAGT	TTTGGTCAGTGCTTTTCGAC						
Cele.WB235	Cele.WB235_6:3713912	6	3713912	3714032	121	5	20
CCATGTAGCT	CAATGTAGCTACATAGTTTC						
Cele.WB235	Cele.WB235_6:3924546	6	3924546	3924685	140	7	20
AAAATAGATT	AAAATAGATTGACATACCAG						
Cele.WB235	Cele.WB235_6:4026010	6	4026010	4030548	4539	181	25
GTTCTGCCAA	GTTCTGCCAATTTATTTACTCCAAT						
Cele.WB235	Cele.WB235_6:4030571	6	4030571	4031446	876	34	25
ATTGGCAGAA	ATTGGCAGAACATTAGAGTAAATAT						
Cele.WB235	Cele.WB235_6:4031472	6	4031472	4056497	25026	496	20
GCTATATCGT	GCTATATCGTATCGTAAATA						
Cele.WB235	Cele.WB235_6:4285394	6	4285394	4285533	140	7	20
ACAGACGAAA	ACAGACGAAAAAATAGGTTG						
Cele.WB235	Cele.WB235_6:5045129	6	5045129	5051107	5979	23	226
GAAAATTCAT							

GAAAATTCATAAATCTCTTCAAAGTAACTTTTTTATAAATGTCTTTCAGAAACTTTGTAGTAAATTTTAAGCTCTTTCTGAATATATTAACAATATTCCAGTAGGTACAAGAAGCTTCACGTAGTTACAGAAA
TAGTACATTTTCAGCCCTACCTTTTAGTGCGTATTTTATTATTAAATGAAAACTACCATATATAGACAAAAATAGATGAATTTTCGTAATTGT
Cele.WB235 Cele.WB235_6:5045783 6 5045783 5053744 7962 32 226
ATGAATTTTC
ATGAATTTTCGTAATTGTGAAAATTCATAAATCTCTTCAAAGTAACTTTTTTATAAATGTCTTTCAGAAACTTTATAGTAAATTTTAAGCTCTTTCTGAATATATTAACAATATTCCAGTAGGTACAAGAAGC
TTCACGTAGTTACAGAAATAGTACATTTTCAGCCCTACCTTTTAGTGCGTATTTTGTTATTAAATGAAAACTACCATTTATAGGCAAAAATAG
Cele.WB235 Cele.WB235_6:5056273 6 5056273 5057278 1006 38 27
TATTTTACTC TATTTTACTCTCTGTGGCTTCCCACTA
Cele.WB235 Cele.WB235_6:5125291 6 5125291 5125369 79 6 13
GCCATAGGCG GCCATAGGCGTAA
Cele.WB235 Cele.WB235_6:5227815 6 5227815 5227935 121 8 15
TGGTGAGACC TGGTGAGACCCATCC
Cele.WB235 Cele.WB235_6:5229333 6 5229333 5229440 108 5 23
AATAAATATT AATAAATATTAATTAATTAAATG
Cele.WB235 Cele.WB235_6:5229858 6 5229858 5230323 466 31 15
CCACGATGGG CCACGATGGGTCTCA
Cele.WB235 Cele.WB235_6:5230547 6 5230547 5230804 258 10 11
CTACAGTACT CTACAGTACTA
Cele.WB235 Cele.WB235_6:5542238 6 5542238 5542420 183 7 26
TAGTGACTCT TAGTGACTCTGGCCGTTCACTATAAT
Cele.WB235 Cele.WB235_6:5913335 6 5913335 5915857 2523 97 26
TTCTAAATTT TTCTAAATTTGAAAATTCATCTACCG
Cele.WB235 Cele.WB235_6:6114472 6 6114472 6114558 87 5 17
TAATTACTGG TAATTACTGGAATAAAA
Cele.WB235 Cele.WB235_6:6212839 6 6212839 6212968 130 8 16
GCACTGACAA GCACTGACAATTTTTA
Cele.WB235 Cele.WB235_6:6826365 6 6826365 6830125 3761 40 94
TGCCTTCAGA TGCCTTCAGAAATTGACAATTTTCCCATTGATTTGTCTACATAGGGCATCGAAAAGCACCCAATATTTAGAGAACAGAAGATTTTGAGAATTAC
Cele.WB235 Cele.WB235_6:7077538 6 7077538 7079254 1717 55 20
TCGTTTATAG TCGTTTATAGCAGTGTCGTG
Cele.WB235 Cele.WB235_6:7081038 6 7081038 7081216 179 9 20
TTTTTCGTCT TTTTTCGTCTGTCAATCTAT
Cele.WB235 Cele.WB235_6:7351473 6 7351473 7361391 9919 248 40
AATGGGCGCC AATGGGCGCCGGTTTCGCTTTGAGCGATTCCTTACCCTTA
Cele.WB235 Cele.WB235_6:7574716 6 7574716 7575007 292 5 18
TAGTCTTGCA TAGTCTTGCAGTCTCTAA
Cele.WB235 Cele.WB235_6:7686957 6 7686957 7687172 216 6 12
TCTTCCTCCT TCTTCTTCCTCC
Cele.WB235 Cele.WB235_6:7879877 6 7879877 7880045 169 7 24
GGTGGCTGGT GGTGGCTGGTCACAAAAGTAAGTT
Cele.WB235 Cele.WB235_6:7895537 6 7895537 7895652 116 6 19
CAGACGAAAA CAGACGAAAAATATATTGG
Cele.WB235 Cele.WB235_6:7918015 6 7918015 7918071 57 4 14
TTACCAAAAT TTACCAAAATCTAG
Cele.WB235 Cele.WB235_6:8181963 6 8181963 8182276 314 28 11
GAGCGGTTTT GAGCGGTCTTG
Cele.WB235 Cele.WB235_6:8602386 6 8602386 8602451 66 5 11
GCAAAAAATG GCAAAAAATGT
Cele.WB235 Cele.WB235_6:9007305 6 9007305 9007574 270 10 11
GTACTGTAGG GTACTGTAGTA
Cele.WB235 Cele.WB235_6:9007668 6 9007668 9008148 481 12 40
CTTCTGGAAA CTTCTGGAAAATCCGAGAAAATTCTGGAATGTTCCAGAAC

Cele.WB235	Cele.WB235_6:9008356	6	9008356	9008996	641	20	32
CGGGAATTCA	CGGGAATTCAAAATTTCTGTGAAAATTTTTAG						
Cele.WB235	Cele.WB235_6:9184992	6	9184992	9188750	3759	150	25
CAGAGAGTAA	CAGAGAGTAAACTATACTGTGAAGC						
Cele.WB235	Cele.WB235_6:9188817	6	9188817	9189060	244	7	35
AAAATTTGAA	AAAATTTGAAATTCCCGCCAAAATATTTTTCACTG						
Cele.WB235	Cele.WB235_6:9605306	6	9605306	9607639	2334	65	32
TCCCGCCAAA	TCACGCCAAAAATTTTCAAAGAAAATTTGAAT						
Cele.WB235	Cele.WB235_6:9607653	6	9607653	9607728	76	5	15
ATGGGTCTCG	ATGGGTCTCGCCACG						
Cele.WB235	Cele.WB235_6:10360970	6	10360970	10361186	217	4	25
ATTGGCAGAA	ATTGGCAGAACATTAGAGTAAATAT						
Cele.WB235	Cele.WB235_6:10361494	6	10361494	10369166	7673	299	25
GTGAAGCCAG	GTGAAGCCAGAGAGTAAAAAATATG						
Cele.WB235	Cele.WB235_6:10369202	6	10369202	10370313	1112	99	11
TGGAGCGGTT	TGGAGCGGTAT						
Cele.WB235	Cele.WB235_6:10973510	6	10973510	10974510	1001	30	27
CACTGACCAA	CACTGACCAAAAAAAAATATTATACTTG						
Cele.WB235	Cele.WB235_6:11260441	6	11260441	11260545	105	4	26
AAATTAATCT	AAATTAATCTGTCACTCTAACTTTTA						
Cele.WB235	Cele.WB235_6:11440976	6	11440976	11441039	64	4	14
CTATACGCTA	CTATACGCTATACG						
Cele.WB235	Cele.WB235_6:11441051	6	11441051	11442751	1701	68	25
CCGTAATGCC	CCGTAATGCCTAACTTTAGAATCTG						
Cele.WB235	Cele.WB235_6:11442777	6	11442777	11443790	1014	39	26
CGGCGGATTT	CGGCGGATTTTAGAAAAATGACATTG						
Cele.WB235	Cele.WB235_6:11489369	6	11489369	11489615	247	7	11
TCCTACAGTA	TCCTACCGTAA						
Cele.WB235	Cele.WB235_6:11688529	6	11688529	11688958	430	4	108
TTTCACGTTT	TTTCACGTTTTCAGGTGATTATATAGGTCACGAACTAATTTATTTTGAAAATTGACCTGTGGACCTATAACTTTGGAGATATGGTAAATTGAGTATGGGCTTTTAGAT						
Cele.WB235	Cele.WB235_6:11745151	6	11745151	11745371	221	10	22
TCAGTGCAAA	TCAGTGCAAAGTGACAATTATG						
Cele.WB235	Cele.WB235_6:11785744	6	11785744	11787512	1769	68	26
ACCATTCACC	ACCATTCACCTAAAAATATGACTCTA						
Cele.WB235	Cele.WB235_6:11787527	6	11787527	11789550	2024	75	26
AAAGTTAGAC	AAAGTTAGACCATTAGATGAATTTAT						
Cele.WB235	Cele.WB235_6:11789572	6	11789572	11789748	177	16	11
CGAAGTTGTC	CAAAGTTGTCA						
Cele.WB235	Cele.WB235_6:11859496	6	11859496	11859817	322	5	18
TGCAATACTA	TGCAATACTAATAGGGAG						
Cele.WB235	Cele.WB235_6:11886530	6	11886530	11888254	1725	68	25
TTCACCATAT	TTCACCATATATTTTACTCTCTGGC						
Cele.WB235	Cele.WB235_6:11888285	6	11888285	11889641	1357	6	226
TGTACTATTT	TGTACTATTTCTGTAACTACGTGAAGCTTCTTGTACCTACTAAAATATTGTTGGTATATTCAAAAAGAACTTAAAATTTACTACAAAGTTTCTGAAAGACATTTCAAAAAAAGTTACTTTGAAGAGATTTATGAATTTTCAAAGTTACGAAAATCCATCTATTTTTGCCTATAAATGGTAGTTTTCATTAAATAATAAAATACGCACTAAAAGGTAGGGCTGAAAA						
Cele.WB235	Cele.WB235_6:12276123	6	12276123	12276239	117	6	19
ATAGATTGTT	ATAGATTGTTACACGAAAA						
Cele.WB235	Cele.WB235_6:12276852	6	12276852	12278772	1921	55	32
GGTGCTATTT	GGTGCTATTTGGTGCTCCATCGGTGCTACATC						
Cele.WB235	Cele.WB235_6:12502706	6	12502706	12502822	117	6	19
AAAAATAGAT	AAAAATAGATTGTTACACG						

Cele.WB235	Cele.WB235_6:12521171	6	12521171	12522034	864	10	15
GGGTCTCACC	GGGTCTCACCGCGAA						
Cele.WB235	Cele.WB235_6:12578040	6	12578040	12578842	803	12	32
CGACCTAGGT	CGACCTAGGTCGCATTGCGACCTGCGACCTAG						
Cele.WB235	Cele.WB235_6:12606642	6	12606642	12606920	279	9	31
TCGACAGAAG	TCGACAGAAGCTGTCGGATCTATTATCTTCC						
Cele.WB235	Cele.WB235_6:12607668	6	12607668	12607975	308	5	26
AAAAATGGCA	AAAAATGGCATTGCGGCGGATTTCAG						
Cele.WB235	Cele.WB235_6:13242451	6	13242451	13244463	2013	18	108
TTTCAGATTT	TTTCAGATTTTCAGATTTTCACTTGATTATATAGGTCACGACCCAAGTTATTTTGAAAATTGAGCTACACGGCTGTAACTTTGGAGCTATGGGTAATCAAGTATGAGC						
Cele.WB235	Cele.WB235_6:13423594	6	13423594	13423926	333	8	18
TGCAAGACTA	TGCAAGACTATTAAAGGC						
Cele.WB235	Cele.WB235_6:13560726	6	13560726	13560906	181	11	15
TCGTGGTGAG	TCGTGGTGAGACCCT						
Cele.WB235	Cele.WB235_6:13684248	6	13684248	13684998	751	7	108
TGGGTAATCA	TGGGTAATCAGGTACGGGCTTTCAGATTTTCAAGTTTTCAGGTGACTATCTAGGTCACGAGCCAATTTCAGCCAGGAGTGGGACTAGATATATGAATTTCTTGGGTTA						
Cele.WB235	Cele.WB235_6:13775030	6	13775030	13775085	56	5	11
GTGCTTGGTC	GTGCTTGGTCA						
Cele.WB235	Cele.WB235_6:13795118	6	13795118	13795677	560	14	40
GAACATTCCA	GAACATTCCAGAATTTTATCGATTTTTCTAGAAAGTTCTG						
Cele.WB235	Cele.WB235_6:13795780	6	13795780	13795990	211	6	35
CAGAAAATTT	CAGAAAATTTCAATTTCCCGCAAAAATATTTTTCA						
Cele.WB235	Cele.WB235_6:13795118	6	13795118	13796907	1790	19	40
GAACATTCCA	GAACATTCCAGAATTTTATCGATTTTTCTAGAAAGTTCTG						
Cele.WB235	Cele.WB235_6:13797037	6	13797037	13797540	504	19	11
CTACAGTACT	CTACAGTACTC						
Cele.WB235	Cele.WB235_6:13923839	6	13923839	13924439	601	4	153
TGAGGTGCAG	TGAGGTGCAGGACGTGATGAGCTTTGTTGAACTGGGAACTGTTGATTCCCGGAGCCGTGATTCATAGATGAGTTGGAGGGACCTGCTGTGGAACTTGTTTCAACTTGAGCAATTCCATGAGCCATCAAGAAGCTTTCCTGCAGACTGGGCAAC						
Cele.WB235	Cele.WB235_6:13981254	6	13981254	13981504	251	10	25
TCTCTGGCTT	TCTCTGGCTTCACAGAATATTTTAA						
Cele.WB235	Cele.WB235_6:14119472	6	14119472	14121076	1605	43	37
TTTAATTTTT	TTTAATTTTTTGAAAATATTTTGGCGCGCATTCAAAT						
Cele.WB235	Cele.WB235_6:14121147	6	14121147	14122257	1111	73	15
TCGTGGTGAG	TCGTGGTGAGAACCA						
Cele.WB235	Cele.WB235_6:14126276	6	14126276	14126426	151	12	12
ATAGGCATAG	ATAGGCATAGGC						
Cele.WB235	Cele.WB235_6:14154417	6	14154417	14154672	256	13	20
TAACAATCTA	TAACAATCTATTTTTTTCGTG						
Cele.WB235	Cele.WB235_6:14262861	6	14262861	14263101	241	5	48
GGAGGTGGAG	GGAGGTGGAGAAAGTGTAGTTCCCGGTGGTTGTGGTTCTTCCGAGGGT						
Cele.WB235	Cele.WB235_6:14323895	6	14323895	14325001	1107	10	18
TGCAAGACTA	TGCAAGACTAATAGAGAC						
Cele.WB235	Cele.WB235_6:14362236	6	14362236	14362311	76	5	15
TCGTGGCGAG	TCGTGGCGAGACCCA						
Cele.WB235	Cele.WB235_6:14384735	6	14384735	14389919	5185	108	48
AAATTACGGG	AAATTACGGGCAAATCAAAAATTAATTTCATCATCTTCAGAAAAATAT						
Cele.WB235	Cele.WB235_6:14638909	6	14638909	14639063	155	5	22
TTTGTGGGAG	TTTGTGGGAGTGAACTTACACT						
Cele.WB235	Cele.WB235_6:14723633	6	14723633	14724049	417	13	32
TTGAATTCCC	TTGAATTCCCGCCAAAATTTTTCACAGAAAAT						

Cele.WB235 Cele.WB235_6:14723638 6 14723638 14724190 553 16 32
TTCCCGCCAA TTCCCGCCAAAATTTTTCACAGAAAATTTGAA
Cele.WB235 Cele.WB235_6:14723559 6 14723559 14724409 851 5 40
TTCTGGAACA TTCTGGAACATTCCAGCATTTTCTCAAGTTTTCCAGAAGG
Cele.WB235 Cele.WB235_6:14724532 6 14724532 14724801 270 10 11
CCTACAGTAC CTACAGTACAA
Cele.WB235 Cele.WB235_6:14908166 6 14908166 14909416 1251 49 25
TGGCTTCACA TGGCTTCACAGTCTAATTTACTCTC
Cele.WB235 Cele.WB235_6:14937379 6 14937379 14937623 245 7 35
CAGAAAATTT CAGAAAATTTAAATTTCCCGCCAAAATATTTTTCT
Cele.WB235 Cele.WB235_6:15051665 6 15051665 15051975 311 5 72
TGGTTGTGTT TGGTTGTGTTCCCAAAAGTGAGAAAAGAGGGTACAGACCTCTGTACGAATATGTCTTATCAGCTTCAAAAGA
Cele.WB235 Cele.WB235_6:15226880 6 15226880 15229061 2182 20 108
AAGTCTTCTT AAGTCTTCTTGGTTCTGTGTCTTTGCTTCGAAATGTTGACCATCTCCTTGCAGGATCTTATTGTCAGATGGAACCTTCTTGGCATACCTCTCTCCACTGATGTGAGTG
Cele.WB235 Cele.WB235_6:15295722 6 15295722 15295953 232 11 21
TGAAAGATTG TGAAAGATTGGTCAGTGTCTA
Cele.WB235 Cele.WB235_6:15470071 6 15470071 15470810 740 22 10
TATCTAGATC TATCTATATC
Cele.WB235 Cele.WB235_6:15482252 6 15482252 15482427 176 5 35
ATTTAAATTT ATTTAAATTTCCCGCCAAAATATTTTTCACAGAAC
Cele.WB235 Cele.WB235_6:15482468 6 15482468 15483998 1531 97 15
GTCTCACCAC GTCTCACCGCGAAGG
Cele.WB235 Cele.WB235_6:15507328 6 15507328 15507477 150 4 21
TGTCCGTGAT TGTCCGTGATGATCGTGAGCA
Cele.WB235 Cele.WB235_6:15597064 6 15597064 15597272 209 8 26
GGTCTAACTT GGTCTAACTTTATAAATGGATCTGAT
Cele.WB235 Cele.WB235_6:15635133 6 15635133 15635373 241 6 40
AAAGTTCTGG AAAGTTCTGGAACATTCCAGACTTTTCCCAATTTTTCTAG
Cele.WB235 Cele.WB235_6:15635446 6 15635446 15635796 351 10 35
CAGAAAATTT CAGAAAATTTAAATTTCCCTCCAAACTTTTTTTCT
Cele.WB235 Cele.WB235_6:15635841 6 15635841 15636081 241 15 15
GGGTCTCACC GGGTCTCACCACGAT
Cele.WB235 Cele.WB235_6:15687867 6 15687867 15688727 861 33 26
AATTCATCTA AATTCATCTAGTGGTCTAACTTTGGA
Cele.WB235 Cele.WB235_6:15738820 6 15738820 15738988 169 8 21
GGCTGCCGAG GGCTGCCGAGGGTAAGCTAGG
Cele.WB235 Cele.WB235_6:15806999 6 15806999 15807439 441 22 20
CATACCAAAA CATACCAAAAAACAGATTGG
Cele.WB235 Cele.WB235_6:15807460 6 15807460 15811152 3693 142 26
GTGAACGGCC GTGAACGGCCAGAGTCACTAAATTTG
Cele.WB235 Cele.WB235_6:15811201 6 15811201 15811280 80 4 20
TAACAATCTA TAACAATCTATTTTTTCGTG
Cele.WB235 Cele.WB235_6:15929033 6 15929033 15929676 644 4 158
TATCAATTTT
TATCAATTTTCAGACGCGCTACGGGGGTTTTTGTCTTGAATTCCAATTTCAACAGGCGCAAAAAAACAAAAATAAAATTTTTGGATTTTTGGAAAAGTTTTTATAGATTTGTATTCTAGATTGTTTAAAAATA
TTATTGGTACGTTTTTGGACCTATA
Cele.WB235 Cele.WB235_6:15929110 6 15929110 15929778 669 4 158
CAAAAATAAA
CAAAAATAAAATTTTTGGATTTTTGGAAAAGTTTTTATAGATTTGTATTCTAGATTGTTTAAAAATATTATTGGTACGTTTTTGGACCTATATATCAATTTTCAGACGCGCTACGGGGGTTTTTGTCTTGAAT
TCCAATTTCAACAGGCGCAAAAAAA
Cele.WB235 Cele.WB235_6:16050430 6 16050430 16050549 120 6 20
CCACCCTGTA CCACCCTGTAATATCATGGA

Cele.WB235	Cele.WB235_6:16100349	6	16100349	16100596	248	8	11
GTACTGTAGG	GTACTGTAGTA						
Cele.WB235	Cele.WB235_6:16100682	6	16100682	16101122	441	11	40
TCCAGAACCT	TCCAGAACCTTCTGGAAAATCCGAGAAAATTCTGGAATGT						
Cele.WB235	Cele.WB235_6:16100718	6	16100718	16101792	1075	13	40
ATGTTCCAGA	ATGTTCCAGAACCTTCTGGAAAATTTGAGAAAATGCTGGA						
Cele.WB235	Cele.WB235_6:16135006	6	16135006	16135334	329	5	18
ATTAGTTTTG	ATTAGTTTTGCACTCTCT						
Cele.WB235	Cele.WB235_6:16158526	6	16158526	16158670	145	9	14
TACCATATAC	TACCAAATACCATA						
Cele.WB235	Cele.WB235_6:16199460	6	16199460	16199628	169	12	14
CTTAGGGCTT	CTTAGGGCTTCAGG						
Cele.WB235	Cele.WB235_6:16286380	6	16286380	16286520	141	7	20
TACAGGGTGG	TACAGGGTGGCCCATAATTA						
Cele.WB235	Cele.WB235_6:16372783	6	16372783	16373033	251	11	16
TTCCGGCAAT	TTCCGGCAATCGGCTA						
Cele.WB235	Cele.WB235_6:16505118	6	16505118	16505433	316	18	15
TCACCACGAA	TCACCACGAAGGGTC						
Cele.WB235	Cele.WB235_6:16758347	6	16758347	16761606	3260	43	76
AGCATTAGAG	AGCATTAGAGCATGTACACAGACACCGACAACACCACAGTCTCTCCCACTTACCTTCGTGCAAAGGAGTGTCTTCT						
Cele.WB235	Cele.WB235_6:16837630	6	16837630	16838124	495	4	52
GCATGTCTTC	GCATGTCTTCCAGGCAGAGAATCGCTCTCCTAGGTTTCCAAAAACACGTCTA						
Cele.WB235	Cele.WB235_6:16851316	6	16851316	16851756	441	13	40
TTTCTAGAAA	TTTCTAGAAAGTTCTGGAATATTCCAGAATTTTCCCGAAT						
Cele.WB235	Cele.WB235_6:16852072	6	16852072	16852162	91	6	15
GGGTCTCACC	GGGTCTCACCGCGAA						
Cele.WB235	Cele.WB235_6:16884509	6	16884509	16885875	1367	37	10
TGGATCGGGA	TGGATCGGGA						
Cele.WB235	Cele.WB235_6:16886473	6	16886473	16886932	460	20	14
ATAGCGCATA	ATAGCGCATAGCGC						
Cele.WB235	Cele.WB235_6:17087637	6	17087637	17087742	106	5	21
GGTGGACCCA	GGTGGACCCACGGGTTCCTAT						
Cele.WB235	Cele.WB235_6:17125270	6	17125270	17125459	190	5	38
CCGTTTCGCC	CCGTTTCGCCTTGAGCGATTCACCCTTAAATGGGCCTC						
Cele.WB235	Cele.WB235_6:17179152	6	17179152	17179971	820	21	39
ATTGATACTT	ATTGATACTTTGTCGGGTTTAGATCATCGGCCGAGCTTC						
Cele.WB235	Cele.WB235_6:17288074	6	17288074	17288675	602	6	40
TTTCTCGAAT	TTTCTCGAATTTTCCAGAAGGTTCTAGAACATTCCAGAAA						
Cele.WB235	Cele.WB235_6:17311002	6	17311002	17311107	106	7	15
GAGACCCATC	GAGACCCATCATGGC						
Cele.WB235	Cele.WB235_6:17311164	6	17311164	17311479	316	9	35
AAATTTTCTG	AAATTTTCTGTGAAAAATATTTTGGCGGGAAATTT						
Cele.WB235	Cele.WB235_6:17310825	6	17310825	17311809	985	10	40
AATGTTCCAG	AATGTTCCAGAACCTTCTGGGAATTTCTGGAAAATTCTAG						
Cele.WB235	Cele.WB235_6:17344107	6	17344107	17344254	148	8	15
GGGGGCAACA	GGGGGCAACAAATTA						
Cele.WB235	Cele.WB235_6:17441768	6	17441768	17447783	6016	134	45
CCACCAGCAC	CCACCAGCACTGCTTCGAGTACGACAGAGAGCACTTCGACTCCAT						
Cele.WB235	Cele.WB235_6:17674798	6	17674798	17675174	377	7	36
CCACCAACTC	CCACCAACACCAAAATCAGCTGCTCCATCAGAGGTT						
Cele.WB235	Cele.WB235_6:17718013	6	17718013	17718930	918	12	12
TTAGGCTTAG	TCAGGCTTAGGC						
