# Supplementary material for: Unique Features of Satellite DNA Transcription in Different Tissues of Caenorhabditis elegans
Source: Int J Mol Sci. 2023 Feb 3;24(3):2970. doi: 10.3390/ijms24032970 (PMC9918286; doi:10.3390/ijms24032970)
Supplement: Supplementary file 1 [file ijms-24-02970-s001.zip › Table S2. WB235 satellite families and their members.pdf]

```
 ************************************************************
The program is running under the following parameters:
   Output:
      Prefix of the family name WB235
      Columns for the name: 150
      Cluster with more that 0 sequences are printed
      File with the table:
/home/peypoch/disk1/bioinformatica/sra/nemapodes/WB235/WB235.fa.1-0-0-4-
800-10-1-60-2-0-0-2-0-1-2.sat.fa.0-1-1-1-1-1-0.6-0-1-150-1.fam.t
      File with families in format fasta:
/home/peypoch/disk1/bioinformatica/sra/nemapodes/WB235/WB235.fa.1-0-0-4-
800-10-1-60-2-0-0-2-0-1-2.sat.fa.0-1-1-1-1-1-0.6-0-1-150-1.fam.fa
   Alphabet 0 (size 5)  (0:dna bases, 1 aminoacids, 2 codons)
   Length of the mer 1
   Circular permutations are allowed
   Reverse sequencess are considerd.
   Sequences are aligned while similarity : 0.600000
   Alignment score: Normalized respect to the length and number of
sequences.
   Alignment parameters:
      opening gap: -3.000000
      extensio gap: -1.000000
      extreme gaph: -0.500000
   Concensus type:  without gap columns
   Similarity matrix file:
/home/peypoch/disk1/bioinformatica/malig/darreraversio/score_matrix.3.-1
   Multifasta file:
/home/peypoch/disk1/bioinformatica/sra/nemapodes/WB235/WB235.fa.1-0-0-4-
800-10-1-60-2-0-0-2-0-1-2.sat.fa

 ************************************************************
Number of sequences=3978
There are 402 alignments:

>WB235_Fam_1_35_172  Nr. of seq. 172 Alignment length(with gaps) = 44
Alignment score = 0.653800
WB235_2:11660023-11660287 Satlength=265 Nr of Repeats=8 RepeatLength=33
seed=AATTGAAAAT  Num.seqs=8 Similarity=0.834055
0    AATT--G--AA-AA---TCCGGCAAATGGGC-AAATTGCCG-G-
WB235_1:3835938-3837024 Satlength=1087 Nr of Repeats=26 RepeatLength=34
seed=TGCCGGAATT  Num.seqs=16 Similarity=0.878105
28    AATT--G--AA--A-TTTCCAGCAAATCGGC-AAATTGCCG-G-
WB235_2:5627848-5628154 Satlength=307 Nr of Repeats=9 RepeatLength=34
seed=GGAATTGAAA  Num.seqs=9 Similarity=0.831481
32    AATT--G--AA--A-TTTCCGGCAAATCGGC-AAATTGCCG-G-
Rev.of_WB235_2:15188581-15189224 Satlength=644 Nr of Repeats=18
RepeatLength=34 seed=TTTCAATTCC  Num.seqs=12 Similarity=0.906120
42    AATT--G--AA--A-TTTCCGGCAAATCGGC-AAATTGCCG-G-
Rev.of_WB235_3:11495534-11496085 Satlength=552 Nr of Repeats=15
RepeatLength=34 seed=AAATTTCAAT  Num.seqs=10 Similarity=0.841394
45    AATT--G--AA--A-TTTCCGGCAAATCGGC-AAATTGCCG-G-
```

WB235_3:13095846-13096595 Satlength=750 Nr of Repeats=22 RepeatLength=34
seed=AAATCGGCAA Num.seqs=21 Similarity=0.849300
50 AATT--G--AA--A-TTTCCGGCAAATCGGC-AAATTGCCG-G-
WB235_3:13099929-13100574 Satlength=646 Nr of Repeats=18 RepeatLength=34
seed=CGGAATTGAA Num.seqs=15 Similarity=0.854715
65 AATT--G--AA--A-TTTCCGGCAAATCGGC-AAATTGCCG-G-
Rev.of_WB235_3:12584489-12584865 Satlength=377 Nr of Repeats=11
RepeatLength=34 seed=TTTCAATTCC Num.seqs=7 Similarity=0.788982
42 AATT--G--AA--A-TTTCCGGTAAATCGGC-AAATTGCCG-G-
Rev.of_WB235_1:14772871-14773518 Satlength=648 Nr of Repeats=19
RepeatLength=34 seed=CGGAAATTTC Num.seqs=18 Similarity=0.965142
48 AATT--G--AA--A-TTTCCGGCAAATCGGC-AAATTGCTG-G-
WB235_2:14870141-14870668 Satlength=528 Nr of Repeats=13 RepeatLength=35
seed=TTTCCGGCAA Num.seqs=8 Similarity=0.791837
77 AATT--GA-AA--A-TTTCCGGCAAATCGGC-AAATTGCTG-G-
Rev.of_WB235_4:382116-382388 Satlength=273 Nr of Repeats=8
RepeatLength=34 seed=TTGCAGGAAA Num.seqs=8 Similarity=0.823529
52 AATT--G--AA--A-TTTCCTGCAAACCGGC-AAATTGCCG-G-
Rev.of_WB235_5:775516-775721 Satlength=206 Nr of Repeats=6
RepeatLength=34 seed=CAATTTGCCG Num.seqs=5 Similarity=0.788235
64 AATT--G--AA--A-TTTCCGGCAAACCGGC-AAATTGCCG-A-
Rev.of_WB235_2:13037761-13037897 Satlength=137 Nr of Repeats=4
RepeatLength=34 seed=TTCAATTCCG Num.seqs=4 Similarity=0.803922
41 AATT--G--AA--A-TTTCCGGCAAATCGAC-GAATTGCCG-G-
WB235_1:11061949-11062380 Satlength=432 Nr of Repeats=11 RepeatLength=34
seed=TTCCGGCAAA Num.seqs=9 Similarity=0.844227
43 AATT--T--AA--A-TTTCCGGCAAATCGGA-AAATTGCCG-G-
Rev.of_WB235_4:3004801-3005510 Satlength=710 Nr of Repeats=20
RepeatLength=34 seed=CCGATTTGCC Num.seqs=12 Similarity=0.843731
57 AGTT--G--AA--A-TTTCCGGCAAATCGGT-AAATTGCCG-G-
WB235_3:12531237-12531912 Satlength=676 Nr of Repeats=18 RepeatLength=35
seed=TTTCCGGCAA Num.seqs=13 Similarity=0.878877
77 AATT--G--AAC-A-TTTCCGGCAAATCGGT-AAATTGCCG-G-
Rev.of_WB235_1:1012604-1013479 Satlength=876 Nr of Repeats=25
RepeatLength=35 seed=ATTTTCAATT Num.seqs=25 Similarity=0.921397
44 AATT--G--AA-AA-TTTCCGGCAAATCGGC-AAACTGCCG-G-
WB235_1:1564845-1565136 Satlength=292 Nr of Repeats=7 RepeatLength=35
seed=CGGCAAATCG Num.seqs=6 Similarity=0.895873
47 AATT--G--AA-AA-TTTCCGGCAAATCGGC-AAATTGCCG-G-
Rev.of_WB235_1:1605584-1606132 Satlength=549 Nr of Repeats=6
RepeatLength=35 seed=AAATTTTCAA Num.seqs=5 Similarity=0.878095
81 AATT--G--AA-AA-TTTCCGGCAAATCGGC-AAATTGCCG-G-
Rev.of_WB235_1:1946493-1947006 Satlength=514 Nr of Repeats=5
RepeatLength=35 seed=TTTGCCGGAA Num.seqs=3 Similarity=0.759259
54 AATT--G--AA-AA-TTTCCGGCAAATCGGC-AAATTGCCG-G-
WB235_1:2809445-2809654 Satlength=210 Nr of Repeats=5 RepeatLength=35
seed=GGAATTGAAA Num.seqs=3 Similarity=0.898413
67 AATT--G--AA-AA-TTTCCGGCAAATCGGC-AAATTGCCG-G-
Rev.of_WB235_1:10977031-10977503 Satlength=473 Nr of Repeats=10
RepeatLength=35 seed=TTGCCGATTT Num.seqs=6 Similarity=0.702469
61 AATT--G--AA-AA-TTTCCGGCAAATCGGC-AAATTGCCG-G-
Rev.of_WB235_2:12770724-12771186 Satlength=463 Nr of Repeats=10
RepeatLength=35 seed=TCAATTCCGG Num.seqs=9 Similarity=0.876190
75 AATT--G--AA-AA-TTTCCGGCAAATCGGC-AAATTGCCG-G-

Rev.of_WB235_2:13394857-13395660 Satlength=804 Nr of Repeats=20
RepeatLength=35 seed=GATTTGCCGG Num.seqs=15 Similarity=0.816490
56   AATT--G--AA-AA-TTTCCGGCAAATCGGC-AAATTGCCG-G-
WB235_2:14239112-14239812 Satlength=701 Nr of Repeats=18 RepeatLength=35
seed=GCAAATCGGC Num.seqs=14 Similarity=0.835060
49   AATT--G--AA-AA-TTTCCGGCAAATCGGC-AAATTGCCG-G-
Rev.of_WB235_2:15111116-15111396 Satlength=281 Nr of Repeats=8
RepeatLength=35 seed=TTTGCCGGAA Num.seqs=8 Similarity=0.858503
54   AATT--G--AA-AA-TTTCCGGCAAATCGGC-AAATTGCCG-G-
WB235_3:1148439-1149466 Satlength=1028 Nr of Repeats=13 RepeatLength=35
seed=TTTAAAATTT Num.seqs=8 Similarity=0.863946
71   AATT--G--AA-AA-TTTCCGGCAAATCGGC-AAATTGCCG-G-
WB235_3:1315590-1315824 Satlength=235 Nr of Repeats=5 RepeatLength=35
seed=CAAATCGGCA Num.seqs=3 Similarity=0.695238
50   AATT--G--AA-AA-TTTCCGGCAAATCGGC-AAATTGCCG-G-
Rev.of_WB235_3:2545690-2546082 Satlength=393 Nr of Repeats=11
RepeatLength=35 seed=TTTGCCGGAA Num.seqs=7 Similarity=0.807710
54   AATT--G--AA-AA-TTTCCGGCAAATCGGC-AAATTGCCG-G-
WB235_3:3661285-3661688 Satlength=404 Nr of Repeats=8 RepeatLength=35
seed=TTCCGGCAAA Num.seqs=5 Similarity=0.760000
79   AATT--G--AA-AA-TTTCCGGCAAATCGGC-AAATTGCCG-G-
WB235_3:11817185-11817612 Satlength=428 Nr of Repeats=12 RepeatLength=35
seed=AATTGCCGGA Num.seqs=10 Similarity=0.840847
60   AATT--G--AA-AA-TTTCCGGCAAATCGGC-AAATTGCCG-G-
Rev.of_WB235_3:12408999-12409471 Satlength=473 Nr of Repeats=10
RepeatLength=35 seed=GATTTGCCGG Num.seqs=7 Similarity=0.791446
56   AATT--G--AA-AA-TTTCCGGCAAATCGGC-AAATTGCCG-G-
Rev.of_WB235_5:4197817-4198139 Satlength=323 Nr of Repeats=8
RepeatLength=35 seed=TTTCAATTCC Num.seqs=7 Similarity=0.885714
77   AATT--G--AA-AA-TTTCCGGCAAATCGGC-AAATTGCCG-G-
WB235_5:4718387-4718578 Satlength=192 Nr of Repeats=5 RepeatLength=35
seed=CGGCAAATCG Num.seqs=3 Similarity=0.479798
47   AATT--G--AA-AA-TTTCCGGCAAATCGGC-AAATTGCCG-G-
WB235_1:13014176-13014829 Satlength=654 Nr of Repeats=16 RepeatLength=35
seed=ATTGCCGGAA Num.seqs=10 Similarity=0.780741
61   AATT--G--AA-AA-TTTCCGGCAAATCGGN-AAATTGCCG-G-
WB235_2:233654-235183 Satlength=1530 Nr of Repeats=40 RepeatLength=35
seed=TTTCCGGCAA Num.seqs=27 Similarity=0.708272
78   AATT--G--AA-AA-TTTCCGGCAAATCGGC-ANATTGCCG-G-
Rev.of_WB235_5:2391673-2391869 Satlength=197 Nr of Repeats=4
RepeatLength=35 seed=AAATTTTCAA Num.seqs=3 Similarity=0.780864
81   AATT--G--AA-AA-TTTCCGGCNAATCGGC-AAATTGCCG-G-
Rev.of_WB235_5:2499952-2500148 Satlength=197 Nr of Repeats=4
RepeatLength=35 seed=AAATTTTCAA Num.seqs=3 Similarity=0.780864
81   AATT--G--AA-AA-TTTCCGGCNAATCGGC-AAATTGCCG-G-
Rev.of_WB235_3:11687193-11687714 Satlength=522 Nr of Repeats=14
RepeatLength=35 seed=CCGATTTGCC Num.seqs=10 Similarity=0.809053
58   AATT--G--AA-AA-TTTCNGGCAAATCGGC-AAATTGCCG-G-
Rev.of_WB235_3:11925619-11926677 Satlength=1059 Nr of Repeats=29
RepeatLength=35 seed=TTCAATTCCG Num.seqs=21 Similarity=0.856834
76   AATT--G--AA-AA-TTTCTGGCAAATCGGC-AAATTGCCG-G-
Rev.of_WB235_5:1377073-1377773 Satlength=701 Nr of Repeats=21
RepeatLength=35 seed=ATTTTCAATT Num.seqs=15 Similarity=0.700680
79   AATT--G--AA-AA-TTTCNGGCAAATCGGC-AAATTGCCG-G-

WB235_2:14264215-14264530 Satlength=316 Nr of Repeats=8 RepeatLength=35
seed=CGGCAAATCG Num.seqs=7 Similarity=0.780864
47   AATT--G--NA-AA-TTTCCGGCAAATCGGC-AAATTGCCG-G-
Rev.of_WB235_2:14278881-14279293 Satlength=413 Nr of Repeats=12
RepeatLength=35 seed=GCCGGAAATT Num.seqs=11 Similarity=0.952900
51   AATT--G--GA-AA-TTTCCGGCAAATCGGC-AAATTGCCG-G-
Rev.of_WB235_1:1814405-1814778 Satlength=374 Nr of Repeats=9
RepeatLength=35 seed=TTTGCCGGAA Num.seqs=8 Similarity=0.766700
54   AATT--G--AA-AA-TTTCCGGCAAATCGGC-AACTTGCCG-G-
Rev.of_WB235_2:15047976-15048537 Satlength=562 Nr of Repeats=16
RepeatLength=35 seed=TTTGCCGGAA Num.seqs=11 Similarity=0.731650
54   AATT--G--AA-AA-TTTCCGGCAAATCGGC-AANTTGCCG-G-
Rev.of_WB235_1:2139349-2139629 Satlength=281 Nr of Repeats=8
RepeatLength=35 seed=CGGCAAATTG Num.seqs=5 Similarity=0.885714
68   AATT--G--AA-AA-TTTCCGGCAAATCGGC-AATTTGCCG-G-
Rev.of_WB235_5:2670563-2670782 Satlength=220 Nr of Repeats=6
RepeatLength=35 seed=GCCGGAAATT Num.seqs=4 Similarity=0.752381
51   AATT--G--AA-AA-TTTCCGGCAAATCGGG-AAATTGCCG-G-
Rev.of_WB235_2:15210567-15211143 Satlength=577 Nr of Repeats=12
RepeatLength=35 seed=CCGATTTGCC Num.seqs=11 Similarity=0.807446
58   AATT--C--AA-AA-TTTCCGGCAAATCGGC-AAATTGCCG-G-
WB235_3:12244502-12244747 Satlength=246 Nr of Repeats=7 RepeatLength=35
seed=AATTGCCGGA Num.seqs=7 Similarity=0.912925
60   AATT--C--AA-AA-TTTCCGGCAAATCGGC-AAATTGCCG-G-
Rev.of_WB235_3:2791812-2792160 Satlength=349 Nr of Repeats=10
RepeatLength=35 seed=TTTGCCGGAA Num.seqs=8 Similarity=0.859864
89   AATT--A--AA-AA-TTTCCGGCAAATCGGC-AAATTGCCG-G-
WB235_3:12884539-12885107 Satlength=569 Nr of Repeats=13 RepeatLength=35
seed=TGCCGGAATT Num.seqs=8 Similarity=0.817687
63   AATT--T--AA-AA-TTTCCGGCAAATCGGC-AAATTGCCG-G-
WB235_5:2660817-2661267 Satlength=451 Nr of Repeats=10 RepeatLength=35
seed=TTTCCGGCAA Num.seqs=7 Similarity=0.807710
78   AATT--T--AA-AA-TTTCCGGCAAATCGGC-AAATTGCCG-G-
WB235_1:11349013-11350796 Satlength=1784 Nr of Repeats=47 RepeatLength=35
seed=GGCAAATCAG Num.seqs=42 Similarity=0.911023
48   AATT--G--AA-AA-TTTCCGGCAAATCAGC-AAATTGCCG-G-
WB235_2:13598247-13598526 Satlength=280 Nr of Repeats=7 RepeatLength=35
seed=TTGCCGGAAT Num.seqs=6 Similarity=0.784127
62   AATT--G--AA-TA-TTTCCGGCAAATCGGC-AAATTGCCG-G-
Rev.of_WB235_5:3765128-3765284 Satlength=157 Nr of Repeats=4
RepeatLength=35 seed=TTGCCGATTT Num.seqs=3 Similarity=0.784127
96   AATT--G--AA-TN-TTTCCGGCAAATCGGC-AAATTGCCG-G-
Rev.of_WB235_1:12977769-12978231 Satlength=463 Nr of Repeats=10
RepeatLength=35 seed=GCCGGAAATT Num.seqs=7 Similarity=0.776871
51   AATT--N--AN-AA-TTTCCGGCAAATCGGC-AAATTGCCG-G-
Rev.of_WB235_5:2640455-2641508 Satlength=1054 Nr of Repeats=15
RepeatLength=35 seed=TCAATTCCGG Num.seqs=10 Similarity=0.832381
75   AATT--G--AG-AA-TTTCCGGCAAATCGGC-AAATTGCCG-G-
WB235_2:14273310-14273525 Satlength=216 Nr of Repeats=5 RepeatLength=35
seed=CGGCAAATTG Num.seqs=3 Similarity=0.586563
55   AATT--G--AA-AA-TTTCCGNNAAATCGGC-AAATTGCCG-G-
Rev.of_WB235_4:1928447-1928762 Satlength=316 Nr of Repeats=8
RepeatLength=35 seed=GAAATTTTCA Num.seqs=7 Similarity=0.927438
82   AATT--G--AA-AA-TTTCCGTCAAATCGGC-AAATTGCCG-G-

Rev.of_WB235_2:15044151-15044326 Satlength=176 Nr of Repeats=5 RepeatLength=35 seed=TTTGCCGGAA Num.seqs=3 Similarity=0.771429
54 AATT--N--AA-AA-TTTCCGGCAAATCGGC-AAATTGCCN-G-
Rev.of_WB235_1:2165416-2165854 Satlength=439 Nr of Repeats=10 RepeatLength=35 seed=TTTGCCGGAA Num.seqs=6 Similarity=0.735873
54 AATT--C--AA-AA-TTTCCGGCAAATCGGC-AAATTGTCG-G-
WB235_1:13453071-13453246 Satlength=176 Nr of Repeats=5 RepeatLength=35 seed=TGAAAATTTC Num.seqs=5 Similarity=0.878095
72 AATT--G--AA-AA-TTTCCGGCAAATCGGC-AAATTGTCG-G-
WB235_1:14619236-14619481 Satlength=246 Nr of Repeats=6 RepeatLength=35 seed=CAAATCGGCA Num.seqs=4 Similarity=0.733333
85 AATT--G--AA-AA-TTTCCGGCAAATCGGC-AAATTGTCG-G-
Rev.of_WB235_3:2066729-2067447 Satlength=719 Nr of Repeats=12 RepeatLength=35 seed=TTTGCCGGAA Num.seqs=10 Similarity=0.926349
89 AATT--G--AA-AA-TTTCCGGCAAATCGGC-AAATTGTCG-G-
Rev.of_WB235_5:5125946-5126346 Satlength=401 Nr of Repeats=8 RepeatLength=35 seed=TTGCCGATTT Num.seqs=5 Similarity=0.767619
61 AATT--G--AA-AA-TTTCCGGCAAATCGGC-AANTTGNCG-G-
WB235_2:14269513-14269895 Satlength=383 Nr of Repeats=11 RepeatLength=35 seed=CCGGCAAATC Num.seqs=8 Similarity=0.757275
81 AATT--G--AA-AC-TTTCCGGCAAATCGGC-AAATTGGCG-G-
WB235_3:12005666-12006008 Satlength=343 Nr of Repeats=8 RepeatLength=35 seed=GGAATTGAAA Num.seqs=5 Similarity=0.843810
102 AATT--G--AA-AA-TTTCCGGCAAATCGGC-AAATTGGCG-G-
WB235_5:15168733-15168927 Satlength=195 Nr of Repeats=6 RepeatLength=35 seed=GGAATTGAAA Num.seqs=4 Similarity=0.904762
102 AATT--G--AA-AA-TTTCCGGCAAATCGGC-AAATTGGCG-G-
Rev.of_WB235_1:14843491-14843700 Satlength=210 Nr of Repeats=6 RepeatLength=35 seed=TTTCAATTCC Num.seqs=5 Similarity=0.878095
77 AATT--G--AA-AG-TTTTCGGCAAATCGGC-AAATTGCCG-G-
Rev.of_WB235_4:2872402-2872909 Satlength=508 Nr of Repeats=14 RepeatLength=35 seed=ATTCCGGCAA Num.seqs=11 Similarity=0.820606
107 AATT--G--AA-AA-TTTTCGGCAAATCGGC-AAATTGCCG-G-
Rev.of_WB235_1:2738743-2738953 Satlength=211 Nr of Repeats=6 RepeatLength=35 seed=AATTTCAATT Num.seqs=6 Similarity=0.860317
44 AATT--G--AA-AT-TTTCCGGCAAATCGGC-AAGTTGCCG-G-
WB235_1:10993314-10993636 Satlength=323 Nr of Repeats=8 RepeatLength=35 seed=GCCGGAATTG Num.seqs=7 Similarity=0.829478
64 AATT--G--AA-AT-TTTCCGGCAAATCGGC-AAATTGCCG-G-
Rev.of_WB235_2:13642102-13642382 Satlength=281 Nr of Repeats=7 RepeatLength=35 seed=CTTGCCGATT Num.seqs=6 Similarity=0.862857
62 AATT--G--AA-AA-TTTCTGGCAAATCGGC-AAGTTGCCG-G-
WB235_4:3309618-3310597 Satlength=980 Nr of Repeats=27 RepeatLength=35 seed=AATCGGCAAG Num.seqs=25 Similarity=0.850540
87 AATT--G--AA-AA-TTTCCGGCAAATCGGC-AAGTTACCG-G-
Rev.of_WB235_3:10661890-10662218 Satlength=329 Nr of Repeats=9 RepeatLength=35 seed=CAATTTGCCG Num.seqs=6 Similarity=0.885714
65 ATAT--G--AA-AA-TTTCCGGCAAATCGGC-AAATTGCCG-G-
Rev.of_WB235_5:1813009-1813219 Satlength=211 Nr of Repeats=7 RepeatLength=35 seed=TCCGGCAATT Num.seqs=5 Similarity=0.833333
70 ATTT--N--AA-AA-TTTCCGGCAAATCGGC-AAATTGCCG-G-
WB235_1:1884484-1884939 Satlength=456 Nr of Repeats=11 RepeatLength=35 seed=AAATCGGCAA Num.seqs=8 Similarity=0.753742
51 AATT--G--AA-AA-TTTCCGGCAAATCGGC-AAATTGCCG-A-

Rev.of_WB235_2:13740296-13741317 Satlength=1022 Nr of Repeats=28 RepeatLength=35 seed=TTTGCCGGAA Num.seqs=18 Similarity=0.750016
54   AATT--C--AA-AA-TTTCCGGCAAATCGGC-AAATTGCCG-A-
Rev.of_WB235_1:1949999-1950277 Satlength=279 Nr of Repeats=8 RepeatLength=35 seed=TTGCCGGAAA Num.seqs=6 Similarity=0.654655
53   AATT--G--AA-CA-TTTCCGGCAAATCGGC-AATTTGCCG-A-
WB235_5:17880208-17882252 Satlength=2045 Nr of Repeats=55 RepeatLength=35 seed=TCCGGCAAAT Num.seqs=37 Similarity=0.726298
80   AATT--G--AA-AA-TTTCCGGCAAATCGGC-AATTTGCCG-A-
WB235_4:3149092-3151000 Satlength=1909 Nr of Repeats=53 RepeatLength=35 seed=GGCAAATTGC Num.seqs=42 Similarity=0.867264
56   AATT--G--AA-CA-TTTCCGGCAAATAGGC-AAATTGCCG-A-
WB235_1:1083222-1083538 Satlength=317 Nr of Repeats=8 RepeatLength=35 seed=CGGCAAATCG Num.seqs=7 Similarity=0.873016
47   AATT--G--AA-AA-TTTCCGGCAAATCGGC-ATTTTGCCG-G-
Rev.of_WB235_5:1385851-1386349 Satlength=499 Nr of Repeats=9 RepeatLength=35 seed=TTTGCCGGAA Num.seqs=6 Similarity=0.779048
54   AATT--G--AA-AA-TTTCCGGCAAATCCGC-AATTTGCCG-G-
Rev.of_WB235_5:17991128-17991485 Satlength=358 Nr of Repeats=5 RepeatLength=35 seed=AATTCCGGCA Num.seqs=3 Similarity=0.822222
73   AATT--G--AA-AA-TTTCCGGCAAAACGGC-ATATTGCCG-G-
WB235_1:14481352-14481947 Satlength=596 Nr of Repeats=17 RepeatLength=35 seed=CGGCAAATTG Num.seqs=11 Similarity=0.941818
55   AATC--G--AA-AA-TTTCCGGCAAACCGGC-AAATTGCCG-G-
Rev.of_WB235_5:4193678-4193984 Satlength=307 Nr of Repeats=5 RepeatLength=35 seed=AATTTTCAAT Num.seqs=4 Similarity=0.847619
80   AATT--G--AA-AA-TTTCCGGCAAACCGGC-AAATTGCAG-G-
Rev.of_WB235_3:12629696-12629957 Satlength=262 Nr of Repeats=7 RepeatLength=35 seed=TAATTCCGGC Num.seqs=6 Similarity=0.916191
74   AATT--A--AA-AA-TTTCCGGCAAACCGGC-AAATTGCCG-G-
WB235_3:12636128-12637282 Satlength=1155 Nr of Repeats=29 RepeatLength=35 seed=GGCAAACCGG Num.seqs=25 Similarity=0.815309
83   AATT--A--AN-AA-TTTCCGGCAAACCGGC-AAATTGCCG-G-
Rev.of_WB235_3:12692942-12693363 Satlength=422 Nr of Repeats=8 RepeatLength=35 seed=GGAAATTTTA Num.seqs=5 Similarity=0.820952
83   AATT--T--AA-AA-TTTCCGGCAAACCGGC-AAATTGCCG-G-
WB235_5:775870-776051 Satlength=182 Nr of Repeats=5 RepeatLength=35 seed=TTTCCGGCAA Num.seqs=3 Similarity=0.834921
78   AATT--G--AA-GA-TTTCCGGCAAACCGGC-NAATTGCCG-G-
Rev.of_WB235_1:13800189-13800502 Satlength=314 Nr of Repeats=9 RepeatLength=35 seed=AATTCCGGCA Num.seqs=7 Similarity=0.756173
73   AATT--T--AA-AT-TTTCCGGCAAATNGGC-AAATTGCCG-G-
WB235_3:3030347-3030891 Satlength=545 Nr of Repeats=14 RepeatLength=35 seed=AATTGCCGGA Num.seqs=11 Similarity=0.836537
95   AATT--T--AA-AA-TTTCCGGCAAATTGGC-AAATTGCCG-G-
WB235_3:11152069-11152305 Satlength=237 Nr of Repeats=6 RepeatLength=35 seed=AATTGCCGGA Num.seqs=4 Similarity=0.720635
95   AATT--A--AA-AA-TTTCCGGCAAATTGGC-AAATTGCCG-G-
WB235_3:11924167-11924393 Satlength=227 Nr of Repeats=6 RepeatLength=35 seed=TTCCGGCAAA Num.seqs=4 Similarity=0.676190
114   AATT--G--AA-AA-TTTCCGGCAAATTGGC-AAATTGCCG-G-
Rev.of_WB235_5:2654959-2655536 Satlength=578 Nr of Repeats=16 RepeatLength=35 seed=TTTGCCGGAA Num.seqs=10 Similarity=0.790053
124   AATT--G--AA-AA-TTTCCGGCAAATTGGC-AAATTGNCG-G-

WB235_3:3542667-3542838 Satlength=172 Nr of Repeats=5 RepeatLength=34
seed=AATTGAAATT Num.seqs=4 Similarity=0.800794
34   AATT--G--AA--A-TTTCCGGCAAAGCGGC-AAATTGTCG-G-
Rev.of_WB235_1:3602994-3603167 Satlength=174 Nr of Repeats=5
RepeatLength=35 seed=TTTGCCGGAA Num.seqs=3 Similarity=0.822222
54   AATT--G--AA-CA-TTTCCGGCAAACCGGC-AAATTGTCG-G-
WB235_1:11186372-11187503 Satlength=1132 Nr of Repeats=15 RepeatLength=35
seed=TTCCGGCAAA Num.seqs=9 Similarity=0.743915
44   AACT--G--AA-AA-TTTCCGGCAAATCGGC-AAATTGCCN-G-
WB235_3:11546762-11547752 Satlength=991 Nr of Repeats=24 RepeatLength=35
seed=CGGCAAATCG Num.seqs=16 Similarity=0.809524
47   AACT--G--AA-AA-TTTCCGGCAAATCGGC-AAATTGCCA-A-
Rev.of_WB235_2:12757809-12758089 Satlength=281 Nr of Repeats=8
RepeatLength=35 seed=ATTTCAGGCA Num.seqs=8 Similarity=0.791832
73   AAAT--G--AA-AA-TTTCTGGCAAATCGGC-AAATTGCCT-G-
Rev.of_WB235_2:12757828-12758945 Satlength=1118 Nr of Repeats=16
RepeatLength=35 seed=TTTGCCGGAA Num.seqs=10 Similarity=0.752804
89   AAAT--G--AA-AA-TTTCCGGCAAATCGGC-AANTTGCCN-G-
WB235_2:4296664-4296988 Satlength=325 Nr of Repeats=8 RepeatLength=35
seed=TTCCGGCAAA Num.seqs=5 Similarity=0.790476
44   AATT--G--AA-AA-TTTCCGGCAAAACGGC-AAATTGCCA-A-
WB235_3:12004285-12004750 Satlength=466 Nr of Repeats=12 RepeatLength=35
seed=AAAATTTCCG Num.seqs=9 Similarity=0.877249
74   AATT--G--AA-AA-TTTCCGGCAAATCAGC-TAATTGCCA-G-
WB235_3:12289600-12291140 Satlength=1541 Nr of Repeats=44 RepeatLength=35
seed=TTGCCAGAAT Num.seqs=44 Similarity=0.791362
97   AATT--G--AA-AA-ATTCCGGNAAATCGGC-AAATTGCCA-G-
Rev.of_WB235_5:1273379-1274003 Satlength=625 Nr of Repeats=14
RepeatLength=35 seed=AATTCTGGCA Num.seqs=12 Similarity=0.883983
108   AATT--G--AA-AA-TTTCCGGCAAATCGGT-AATTTGCCA-G-
Rev.of_WB235_5:1273379-1274654 Satlength=1276 Nr of Repeats=15
RepeatLength=35 seed=AATTCTGGCA Num.seqs=12 Similarity=0.883983
108   AATT--G--AA-AA-TTTCCGGCAAATCGGT-AATTTGCCA-G-
WB235_2:14780596-14780942 Satlength=347 Nr of Repeats=4 RepeatLength=35
seed=TCCGGCAAAT Num.seqs=3 Similarity=0.586420
45   AATT--T--AN-AA-TNTCCGGCAAATCGGC-AAATTGCCG-A-
WB235_5:19799341-19799769 Satlength=429 Nr of Repeats=12 RepeatLength=35
seed=AATTTCCGGC Num.seqs=11 Similarity=0.926580
76   AATT--T--AA-AA-TTTCCGGCAAATCGGC-AAATAACCG-G-
Rev.of_WB235_1:14764447-14764676 Satlength=230 Nr of Repeats=5
RepeatLength=34 seed=GAAATTTCAA Num.seqs=4 Similarity=0.901961
46   AGTT--G--AA--A-TTTCCGGCAAATTGGC-TAATTGCCG-G-
WB235_2:14137014-14137524 Satlength=511 Nr of Repeats=14 RepeatLength=34
seed=CGGAATTGAA Num.seqs=13 Similarity=0.833082
65   AATT--G--AA--A-TTTCCGGCAAATTGGT-AAAATGCCG-G-
Rev.of_WB235_3:12532752-12533511 Satlength=760 Nr of Repeats=21
RepeatLength=34 seed=TTTCAATTCC Num.seqs=15 Similarity=0.747664
76   AATT--G--AA--A-NTTCCGGCAAATTGGT-AAATTGCCG-G-
Rev.of_WB235_3:133200-133804 Satlength=605 Nr of Repeats=7
RepeatLength=34 seed=AATTCCGGCA Num.seqs=5 Similarity=0.976471
72   AATT--G--AA--A-TTTTTGGCAAATTGGT-AAATTGCCG-G-
WB235_4:1348897-1349230 Satlength=334 Nr of Repeats=9 RepeatLength=34
seed=ATTGCCGGAA Num.seqs=7 Similarity=0.886088
94   AATT--G--AA--A-TTTCTGGCAAATTGGC-AAATTGCCG-G-

WB235_5:4685532-4685994 Satlength=463 Nr of Repeats=10 RepeatLength=35
seed=GCAAATCGGC Num.seqs=6 Similarity=0.837460
15   -ATTC-G--AA-AA-TGTCCGGCAAATCGGC-AAATTGCCG-G-
Rev.of_WB235_2:13831950-13832774 Satlength=825 Nr of Repeats=18
RepeatLength=34 seed=AATTCCGGCA Num.seqs=12 Similarity=0.803922
4   AATT--G--AA--A-TTTCCGATAAATCAGC-AAATTGCCG-G-
WB235_3:10489911-10490082 Satlength=172 Nr of Repeats=5 RepeatLength=34
seed=TTTCCGGCAA Num.seqs=4 Similarity=0.819841
8   AATC-----AA-AA-TTTCCGGCAAATTGAC-AAATTGCCG-G-
WB235_5:3702753-3703068 Satlength=316 Nr of Repeats=9 RepeatLength=35
seed=AAAATTTCCG Num.seqs=9 Similarity=0.834921
5   AATT--G--AA-AA-TTTCCGGCAAACAGGC-AAATTTCTG-G-
WB235_1:2557777-2557986 Satlength=210 Nr of Repeats=6 RepeatLength=35
seed=TTGCCGGAAT Num.seqs=5 Similarity=0.794286
28   AATT--G--AA-AA-TTTTTGGNAAATCGGC-AATTTGCCG-G-
Rev.of_WB235_1:2844921-2845096 Satlength=176 Nr of Repeats=4
RepeatLength=35 seed=TTTGCCGATT Num.seqs=3 Similarity=0.733333
28   AATT--A--AA-AA-TTTTTGNCAAATCGGC-AAATTGCCG-G-
Rev.of_WB235_1:11425096-11425445 Satlength=350 Nr of Repeats=9
RepeatLength=35 seed=ATTCCGGCAA Num.seqs=6 Similarity=0.888254
38   AATT--G--AA-AA-TTTCTGGAAAATCGGC-AAATTGCCG-G-
Rev.of_WB235_1:14811659-14812009 Satlength=351 Nr of Repeats=10
RepeatLength=35 seed=AATTCCGGCA Num.seqs=10 Similarity=0.804115
39   AATT--G--AA-AA-TTTCAGGAAAATCGGC-AAATTGCCG-G-
WB235_3:3037879-3038500 Satlength=622 Nr of Repeats=13 RepeatLength=35
seed=AAATCGGCAA Num.seqs=8 Similarity=0.885714
52   AATT--G--AA-AA-TTTCCAGAAAATCGGC-AAATTGCCG-G-
WB235_2:13761268-13762971 Satlength=1704 Nr of Repeats=50 RepeatLength=35
seed=CCGGAATTGA Num.seqs=37 Similarity=0.869813
66   AATT--G--AA-AA-TTTCTGGAAAATCAAC-AAATTGCCG-G-
Rev.of_WB235_5:1239922-1240281 Satlength=360 Nr of Repeats=10
RepeatLength=35 seed=GAAATTTTCA Num.seqs=8 Similarity=0.842177
48   AATT--G--AA-AA-TTTCTGGCAATTTGGC-AATTTGCCG-G-
WB235_2:10713445-10714142 Satlength=698 Nr of Repeats=8 RepeatLength=35
seed=TTTCTGGCAA Num.seqs=5 Similarity=0.900952
44   AAAT--T--AA-AA-TTTCTGGCAAACCGGC-AATTTGCCG-A-
WB235_3:12341199-12341722 Satlength=524 Nr of Repeats=7 RepeatLength=35
seed=TTGCCGGAAT Num.seqs=5 Similarity=0.729630
63   AATT--G--NA-AA-TTTCTGGCAAACCGGC-AATTTGCCG-G-
WB235_2:15211519-15211997 Satlength=479 Nr of Repeats=9 RepeatLength=35
seed=TTGCCGGAAT Num.seqs=6 Similarity=0.720635
63   AATT--T--AA-AA-TTTCTGGCAAACCGCC-AAATTGCCG-G-
Rev.of_WB235_3:11311767-11311976 Satlength=210 Nr of Repeats=6
RepeatLength=35 seed=GATTTGCCGG Num.seqs=5 Similarity=0.850926
22   ATTT----TAA-TA-TTTCCGGCAAATCGAT-AAATTGCCG-G-
Rev.of_WB235_5:4443918-4444112 Satlength=195 Nr of Repeats=5
RepeatLength=35 seed=ATTCCGGCAA Num.seqs=3 Similarity=0.822222
38   AATT----TAA-TA-GTTCCGGCAAATCGGC-AAATTGCCG-G-
WB235_5:19762622-19764008 Satlength=1387 Nr of Repeats=40 RepeatLength=35
seed=CCGGCAAATC Num.seqs=26 Similarity=0.887707
47   ATTT--G-TAA--A-TTTCCGGCAAATCGAN-AAATTACCG-G-
Rev.of_WB235_4:2851958-2852560 Satlength=603 Nr of Repeats=7
RepeatLength=35 seed=TTTTCAATTC Num.seqs=5 Similarity=0.801905
9   AATT--G--AA-AA-ATTCAGGCAAATCTGC-AAATTGCTT-G-

Rev.of_WB235_5:3763703-3764133 Satlength=431 Nr of Repeats=4 RepeatLength=35 seed=GCCGATTTGC Num.seqs=3 Similarity=0.743827
25   ANTT--G--AA-CA-TTTCAGGCAAATCGGC-AAATTGNCA-G-
WB235_1:1211856-1212413 Satlength=558 Nr of Repeats=15 RepeatLength=35 seed=AAATCGGCAA Num.seqs=12 Similarity=0.802020
17   AAAT--G--AA-AG-ATTCCGGCAAATCGGC-AATTTGCCN-A-
Rev.of_WB235_1:1919828-1922029 Satlength=2202 Nr of Repeats=58 RepeatLength=35 seed=TTGCCGATTT Num.seqs=42 Similarity=0.889475
27   AAAT--G--AA-AA-TTTCCGGCAAATCGGC-AATTTGCNA-A-
WB235_5:4190322-4190733 Satlength=412 Nr of Repeats=11 RepeatLength=35 seed=TTCCGGCAAA Num.seqs=7 Similarity=0.865760
45   AAAT--G--AA-AA-TTTCCGGCAAACCGGC-AATTTGCCA-A-
Rev.of_WB235_3:11422288-11422568 Satlength=281 Nr of Repeats=6 RepeatLength=35 seed=TTTGCCGGAA Num.seqs=4 Similarity=0.790476
55   AAAT--C--AA-AA-TTTCCGGCAAATCGGC-AATTTGCCA-G-
WB235_3:12823844-12823984 Satlength=141 Nr of Repeats=4 RepeatLength=35 seed=CGGCAATTTG Num.seqs=4 Similarity=0.936508
56   AAAT--C--AA-AA-TTTCCGGCAAATCGGC-AATTTGCCA-A-
Rev.of_WB235_5:1388994-1389344 Satlength=351 Nr of Repeats=8 RepeatLength=35 seed=TGCCGGAAAT Num.seqs=5 Similarity=0.790476
88   AAAT--C--AA-AA-TTTCCGGCAAATCGGC-AATTTGCCA-A-
Rev.of_WB235_3:12722182-12722605 Satlength=424 Nr of Repeats=13 RepeatLength=35 seed=AAATTTTGAT Num.seqs=9 Similarity=0.900529
82   AAAT--C--AA-AA-TTTCCGGCAAATCGGC-AAATTGCCA-A-
Rev.of_WB235_5:4146424-4146572 Satlength=149 Nr of Repeats=4 RepeatLength=35 seed=TTTGCCGGAA Num.seqs=3 Similarity=0.682540
55   AAAT--N--AA-AA-TTTCCGGCAAAACGGC-AATTTACCA-A-
Rev.of_WB235_5:5215493-5215916 Satlength=424 Nr of Repeats=9 RepeatLength=35 seed=CGATTTGCCG Num.seqs=6 Similarity=0.576768
58   AAAT--A--AA-AA-TTTCCGGCAAATCGGC-AATTTGCC-GA-
WB235_6:209349-209653 Satlength=305 Nr of Repeats=8 RepeatLength=36 seed=ATAAAAATTT Num.seqs=7 Similarity=0.865961
73   AAAT--A--AA-AA-TTTCCGGCAAATCGGC-AATTTGCCAGA-
Rev.of_WB235_4:1295080-1295440 Satlength=361 Nr of Repeats=9 RepeatLength=36 seed=TTATTTTCTG Num.seqs=8 Similarity=0.838624
77   AAAT--A--AA-AA-TTTCCGGCAAATGGGC-AATTTGTCAGA-
Rev.of_WB235_4:1299931-1300254 Satlength=324 Nr of Repeats=8 RepeatLength=36 seed=TTTGCCGGAA Num.seqs=5 Similarity=0.859259
91   AAAT--A--AA-AA-TTTCCGGCAAATCGGC-AATTTGTCAGA-
Rev.of_WB235_1:1935250-1936853 Satlength=1604 Nr of Repeats=40 RepeatLength=35 seed=GAAATTTTCA Num.seqs=33 Similarity=0.890620
48   AACT--G--AA-AA-TTTCGGGCAAATCGGC-AATTTGCCG-A-
Rev.of_WB235_1:1935250-1938219 Satlength=2970 Nr of Repeats=70 RepeatLength=35 seed=GAAATTTTCA Num.seqs=48 Similarity=0.836375
48   AACT--G--AA-AA-TTTCCGGCAAATCGGC-AATTTGCCG-A-
WB235_1:14449256-14449536 Satlength=281 Nr of Repeats=8 RepeatLength=35 seed=AAAATTTCCG Num.seqs=8 Similarity=0.952381
75   AAGT--G--AA-AA-TTTCCGGCAAATCGGC-AATTTACCG-A-
Rev.of_WB235_1:14452267-14454642 Satlength=2376 Nr of Repeats=68 RepeatLength=35 seed=GAAATTTTCA Num.seqs=67 Similarity=0.938593
83   AAGT--G--AA-AA-TTTCCGGCAAATCGGC-AATTTACCG-A-
WB235_5:1940104-1940384 Satlength=281 Nr of Repeats=8 RepeatLength=35 seed=GGCAAATCGG Num.seqs=8 Similarity=0.914286
84   AAAT--G--AA-AA-TTTCCGGCAAATCGGC-AATTTTCCG-A-

WB235_4:1654913-1655123 Satlength=211 Nr of Repeats=6 RepeatLength=35
seed=ATTTCCGGCA Num.seqs=6 Similarity=0.819683
78   AAAT--G--AA-AA-TTTCCGGCAAATCGGC-AATATGCCG-A-
WB235_4:16403739-16404089 Satlength=351 Nr of Repeats=10 RepeatLength=35
seed=CAAATCGGCA Num.seqs=8 Similarity=0.824490
86   AATT--G--AA-AA-TTTCCGGCAAATCGGC-AATATGCCA-A-
WB235_4:16409693-16409938 Satlength=246 Nr of Repeats=7 RepeatLength=35
seed=GCAAATCGGC Num.seqs=7 Similarity=0.842177
120   AATT--G--AA-AA-TTTCCGGCAAATCGGC-AATATGCCA-A-
Rev.of_WB235_4:16408366-16409098 Satlength=733 Nr of Repeats=21
RepeatLength=35 seed=TTTGGCATAT Num.seqs=16 Similarity=0.860000
106   AATT--G--AA-AT-TTTCCGGCAAATCGGC-AATATGCCA-A-
Rev.of_WB235_2:4569011-4569221 Satlength=211 Nr of Repeats=5
RepeatLength=35 seed=TTTGCCGGAA Num.seqs=4 Similarity=0.757716
55   AAAT--G--AA-AT-TTTCCGGCAAATCGGT-AATTTGCCG-A-
Rev.of_WB235_5:19795837-19797364 Satlength=1528 Nr of Repeats=40
RepeatLength=35 seed=GAAATTTTCA Num.seqs=29 Similarity=0.756979
83   AAAT--G--AA-AA-TTTCCGGCAAATCNGT-AATTTGCCG-A-
WB235_1:11190690-11191449 Satlength=760 Nr of Repeats=4 RepeatLength=35
seed=TCGGCAAATT Num.seqs=3 Similarity=1.000000
20   AAAT--G--AA-AA-TTTCCGGCAAATCGGC-AAATTACGG-A-
Rev.of_WB235_2:2529567-2530441 Satlength=875 Nr of Repeats=24
RepeatLength=35 seed=CCGGAAATTT Num.seqs=16 Similarity=0.798413
51   AAAT--G--AA-AA-TTTCCGGCAAATCGGC-AAATCGCCG-A-
Rev.of_WB235_5:967850-968138 Satlength=289 Nr of Repeats=7
RepeatLength=35 seed=TGCCGATTTG Num.seqs=5 Similarity=0.809524
26   AAAT--G--AA-CA-TTTCCGGCAAATCGGC-AAATTCTCG-A-
Rev.of_WB235_2:13422904-13423919 Satlength=1016 Nr of Repeats=29
RepeatLength=35 seed=GAATTTTTGA Num.seqs=29 Similarity=0.850622
48   AAAT--C--AA-AA-ATTCCGGCAAATCGGC-AAATTGCCG-A-
Rev.of_WB235_3:12731873-12732374 Satlength=502 Nr of Repeats=16
RepeatLength=35 seed=ATTTGCCGGA Num.seqs=10 Similarity=0.867936
56   AAAT--C--AA-AA-TTTCCGGCAAATCGGC-ATATTGCCG-G-
Rev.of_WB235_5:5117986-5118371 Satlength=386 Nr of Repeats=10
RepeatLength=35 seed=TGCCGGAAAT Num.seqs=6 Similarity=0.730794
88   AAAT--C--AA-AA-TTTCCGGCAAATCGGC-ATTTTGCCG-G-
WB235_4:4355433-4355651 Satlength=219 Nr of Repeats=5 RepeatLength=35
seed=TTCCGGCAAA Num.seqs=3 Similarity=0.675676
45   AAAT--C--AG-AG-TTTCCGGCAAATCGAC-AATTTGCCG-A-
WB235_2:13681580-13681759 Satlength=180 Nr of Repeats=5 RepeatLength=36
seed=TTGCCTAAAA Num.seqs=4 Similarity=0.938272
29   AAAT--T--AA-AA-TTTCCGGCAAATCAGCAAATTTGCCT-A-
Rev.of_WB235_4:1931275-1932180 Satlength=906 Nr of Repeats=26
RepeatLength=35 seed=CATTTTCGCC Num.seqs=20 Similarity=0.946867
40   AAAT--G--GA-AA-TTTCCGGCAAACCGGC-AATTCGGCG-A-
Rev.of_WB235_4:1931275-1933389 Satlength=2115 Nr of Repeats=50
RepeatLength=35 seed=CATTTTCGCC Num.seqs=38 Similarity=0.954101
40   AAAT--G--GA-AA-TTTCCGGCAAACCGGC-AATTCGGCG-A-
Rev.of_WB235_1:3028822-3029013 Satlength=192 Nr of Repeats=5
RepeatLength=35 seed=TTGCCGGAAA Num.seqs=3 Similarity=0.808642
19   AATT-AC--AA--A-TTTCCGGCAAATCGGC-AATTTGTCG-G-
WB235_5:18042826-18043263 Satlength=438 Nr of Repeats=10 RepeatLength=35
seed=TTTCCGGCAA Num.seqs=6 Similarity=0.790432
45   AATT-TG--AA--AGTTTCCGGCAAATCGGC-ATTTTGCCG-G-

Rev.of_WB235_1:3114471-3114865 Satlength=395 Nr of Repeats=8 RepeatLength=35 seed=CGATTTGCCG Num.seqs=6 Similarity=0.862857
23   AAAT--G--AA-TA-TTTCCGGCAAATCGGC-AGTTTGCCG-G-
WB235_3:11314293-11314573 Satlength=281 Nr of Repeats=6 RepeatLength=35 seed=TTGCCGGATT Num.seqs=4 Similarity=0.815873
28   ATTG--G--AA-TA-TTTTCGGCAAATCGGC-AATTTGCCG-G-
WB235_1:3459203-3459616 Satlength=414 Nr of Repeats=8 RepeatLength=35 seed=TCCGGCAAAT Num.seqs=5 Similarity=0.939048
46   AAGT--C--AA-AA-ATTCCGGCAAATCGGA-AATCTGCCG-G-
Rev.of_WB235_4:4792114-4792760 Satlength=647 Nr of Repeats=17 RepeatLength=35 seed=TTGCCGGAAA Num.seqs=13 Similarity=0.909646
54   AAAC--C--AA-AA-TTTCCGGCAAATCGGA-AACTTGCCG-G-
WB235_1:2111857-2112268 Satlength=412 Nr of Repeats=7 RepeatLength=35 seed=CAAACCGGCA Num.seqs=5 Similarity=0.675926
15   AATT-----AA-CA-TTTNGAGCAAACCGGC-AANTTGCCG-AA

*   *
Consensus:

AAtTgAAaATTTCCGGCAAATCGGCAAaTTGCCGg

>WB235_Fam_2_12_169 Nr. of seq. 169 Alignment length(with gaps) = 14 Alignment score = 0.769942
WB235_3:2455329-2456409 Satlength=1081 Nr of Repeats=40 RepeatLength=10 seed=AGGCAGGCTT Num.seqs=27 Similarity=0.883001
0   AG-GC---AGGCTT
Rev.of_WB235_1:1-835 Satlength=835 Nr of Repeats=55 RepeatLength=12 seed=CTAAGCCTAA Num.seqs=50 Similarity=0.958639
2   AG-GCTT-AGGCTT
Rev.of_WB235_1:556995-557143 Satlength=149 Nr of Repeats=8 RepeatLength=12 seed=CTAAGCCTAA Num.seqs=5 Similarity=0.811111
2   AG-GCTT-AGGCTT
WB235_1:566943-567081 Satlength=139 Nr of Repeats=8 RepeatLength=12 seed=AGGCTTAGGC Num.seqs=5 Similarity=0.844444
12   AG-GCTT-AGGCTT
Rev.of_WB235_1:640909-641017 Satlength=109 Nr of Repeats=6 RepeatLength=12 seed=TAAGCCTAAG Num.seqs=4 Similarity=0.759259
13   AG-GCTT-AGGCTT
WB235_1:727038-727274 Satlength=237 Nr of Repeats=5 RepeatLength=12 seed=GCTTAGGCTT Num.seqs=3 Similarity=0.777778
2   AG-GCTT-AGGCTT
Rev.of_WB235_1:779157-779331 Satlength=175 Nr of Repeats=12 RepeatLength=12 seed=GCCTAAGCCT Num.seqs=10 Similarity=0.933333
10   AG-GCTT-AGGCTT
Rev.of_WB235_1:806422-806644 Satlength=223 Nr of Repeats=16 RepeatLength=12 seed=GCCTAAGCCT Num.seqs=10 Similarity=0.782716
10   AG-GCTT-AGGCTT
WB235_1:814462-814642 Satlength=181 Nr of Repeats=13 RepeatLength=12 seed=GCTTAGGCTT Num.seqs=9 Similarity=0.895062
2   AG-GCTT-AGGCTT
WB235_1:833515-834452 Satlength=938 Nr of Repeats=48 RepeatLength=12 seed=CTTAGGCTTA Num.seqs=32 Similarity=0.819668
9   AG-GCTT-AGGCTT

WB235_1:846174-846372 Satlength=199 Nr of Repeats=12 RepeatLength=12 seed=GCTTAGGCTT Num.seqs=8 Similarity=0.888889
2   AG-GCTT-AGGCTT
Rev.of_WB235_1:942559-942637 Satlength=79 Nr of Repeats=6 RepeatLength=12 seed=AGCCTAAGCC Num.seqs=5 Similarity=0.866667
11   AG-GCTT-AGGCTT
Rev.of_WB235_1:1000380-1000606 Satlength=227 Nr of Repeats=14 RepeatLength=12 seed=GCCTAAGCCT Num.seqs=9 Similarity=0.854938
10   AG-GCTT-AGGCTT
Rev.of_WB235_1:1126721-1126876 Satlength=156 Nr of Repeats=10 RepeatLength=12 seed=CCTAAGCCTA Num.seqs=6 Similarity=0.866667
9   AG-GCTT-AGGCTT
WB235_1:2269401-2269479 Satlength=79 Nr of Repeats=6 RepeatLength=12 seed=TAGGCTTAGG Num.seqs=5 Similarity=0.844444
11   AG-GCTT-AGGCTT
WB235_1:2276437-2276626 Satlength=190 Nr of Repeats=5 RepeatLength=12 seed=AGGCTTAGGC Num.seqs=3 Similarity=0.703704
12   AG-GCTT-AGGCTT
Rev.of_WB235_1:2370746-2370842 Satlength=97 Nr of Repeats=6 RepeatLength=12 seed=AAGCCTAAGC Num.seqs=4 Similarity=0.839744
12   AG-GCTT-AGGCTT
WB235_1:2840215-2840304 Satlength=90 Nr of Repeats=5 RepeatLength=12 seed=TAGGCTTAGG Num.seqs=4 Similarity=0.833333
11   AG-GCTT-AGGCTT
Rev.of_WB235_1:3115944-3116232 Satlength=289 Nr of Repeats=21 RepeatLength=12 seed=CTAAGCCTAA Num.seqs=15 Similarity=0.766138
2   AG-GCTT-AGGCTT
WB235_1:3280164-3280242 Satlength=79 Nr of Repeats=6 RepeatLength=12 seed=AGGCTTAGGC Num.seqs=5 Similarity=0.888889
12   AG-GCTT-AGGCTT
WB235_1:3384983-3385061 Satlength=79 Nr of Repeats=6 RepeatLength=12 seed=GGCTTAGGCT Num.seqs=5 Similarity=0.788889
13   AG-GCTT-AGGCTT
WB235_1:10404549-10404632 Satlength=84 Nr of Repeats=6 RepeatLength=12 seed=TAGGCTTAGG Num.seqs=4 Similarity=0.777778
11   AG-GCTT-AGGCTT
Rev.of_WB235_1:11450427-11450505 Satlength=79 Nr of Repeats=6 RepeatLength=12 seed=AAGCCTAAGC Num.seqs=5 Similarity=1.000000
12   AG-GCTT-AGGCTT
WB235_1:11451202-11451316 Satlength=115 Nr of Repeats=9 RepeatLength=12 seed=GCTTAGGCTT Num.seqs=8 Similarity=0.813492
2   AG-GCTT-AGGCTT
WB235_1:11470915-11470999 Satlength=85 Nr of Repeats=7 RepeatLength=12 seed=GGCTTAGGCT Num.seqs=7 Similarity=0.968254
13   AG-GCTT-AGGCTT
WB235_1:11933362-11933554 Satlength=193 Nr of Repeats=15 RepeatLength=12 seed=TTAGGCTTAG Num.seqs=11 Similarity=0.838695
10   AG-GCTT-AGGCTT
Rev.of_WB235_1:12119896-12120034 Satlength=139 Nr of Repeats=10 RepeatLength=12 seed=TAAGCCTAAG Num.seqs=6 Similarity=0.859259
13   AG-GCTT-AGGCTT
Rev.of_WB235_1:12223508-12223556 Satlength=49 Nr of Repeats=4 RepeatLength=12 seed=GCCTAAGCCT Num.seqs=4 Similarity=0.796296
10   AG-GCTT-AGGCTT

Rev.of_WB235_1:13927845-13927905 Satlength=61 Nr of Repeats=5 RepeatLength=12 seed=GCCTAAGCCT Num.seqs=5 Similarity=0.866667
10   AG-GCTT-AGGCTT
WB235_1:15072265-15072421 Satlength=157 Nr of Repeats=13 RepeatLength=12 seed=TTAGGCTTAG Num.seqs=13 Similarity=0.982906
10   AG-GCTT-AGGCTT
Rev.of_WB235_2:0-161 Satlength=162 Nr of Repeats=13 RepeatLength=12 seed=CCTAAGCCTA Num.seqs=12 Similarity=1.000000
9   AG-GCTT-AGGCTT
WB235_2:573638-573888 Satlength=251 Nr of Repeats=20 RepeatLength=12 seed=TAGGCTTAGG Num.seqs=18 Similarity=0.816993
11   AG-GCTT-AGGCTT
WB235_2:2550207-2550279 Satlength=73 Nr of Repeats=6 RepeatLength=12 seed=TTAGGCTTAG Num.seqs=6 Similarity=0.785185
10   AG-GCTT-AGGCTT
Rev.of_WB235_2:2633519-2633585 Satlength=67 Nr of Repeats=5 RepeatLength=12 seed=CCTAAGCCTA Num.seqs=4 Similarity=0.833333
9   AG-GCTT-AGGCTT
Rev.of_WB235_2:2731022-2731082 Satlength=61 Nr of Repeats=4 RepeatLength=12 seed=GCCTAAGCCT Num.seqs=3 Similarity=0.777778
10   AG-GCTT-AGGCTT
WB235_2:3136277-3136467 Satlength=191 Nr of Repeats=8 RepeatLength=12 seed=GGCTTAGGCT Num.seqs=6 Similarity=0.785185
13   AG-GCTT-AGGCTT
WB235_2:4081925-4082141 Satlength=217 Nr of Repeats=14 RepeatLength=12 seed=CTTAGGCTTA Num.seqs=10 Similarity=0.748148
9   AG-GCTT-AGGCTT
Rev.of_WB235_2:4312357-4312429 Satlength=73 Nr of Repeats=4 RepeatLength=12 seed=AGCCTAAGCC Num.seqs=3 Similarity=0.851852
11   AG-GCTT-AGGCTT
WB235_2:11733044-11733122 Satlength=79 Nr of Repeats=6 RepeatLength=12 seed=CTTAGGCTTA Num.seqs=5 Similarity=0.844444
9   AG-GCTT-AGGCTT
WB235_2:11905368-11905475 Satlength=108 Nr of Repeats=8 RepeatLength=12 seed=AGGCTTAGGC Num.seqs=6 Similarity=0.748148
12   AG-GCTT-AGGCTT
Rev.of_WB235_2:11930277-11930409 Satlength=133 Nr of Repeats=10 RepeatLength=12 seed=AAGCCTAAGC Num.seqs=6 Similarity=0.792593
12   AG-GCTT-AGGCTT
WB235_2:11932324-11932498 Satlength=175 Nr of Repeats=10 RepeatLength=12 seed=AGGCTTAGGC Num.seqs=6 Similarity=0.822222
12   AG-GCTT-AGGCTT
Rev.of_WB235_2:12247672-12247768 Satlength=97 Nr of Repeats=7 RepeatLength=12 seed=CCTAAGCCTA Num.seqs=5 Similarity=0.741026
9   AG-GCTT-AGGCTT
WB235_2:12322418-12322496 Satlength=79 Nr of Repeats=5 RepeatLength=12 seed=TTAGGCTTAG Num.seqs=4 Similarity=0.777778
10   AG-GCTT-AGGCTT
Rev.of_WB235_2:12381010-12381194 Satlength=185 Nr of Repeats=13 RepeatLength=12 seed=AGCCTAAGCC Num.seqs=8 Similarity=0.884921
11   AG-GCTT-AGGCTT
WB235_2:12467214-12467281 Satlength=68 Nr of Repeats=5 RepeatLength=12 seed=GGCTTAGGCT Num.seqs=4 Similarity=0.944444
13   AG-GCTT-AGGCTT

WB235_2:13650128-13650200 Satlength=73 Nr of Repeats=6 RepeatLength=12
seed=TAGGCTTAGG Num.seqs=6 Similarity=0.962963
11   AG-GCTT-AGGCTT
Rev.of_WB235_2:13715389-13715533 Satlength=145 Nr of Repeats=10
RepeatLength=12 seed=GCCTAAGCCT Num.seqs=6 Similarity=0.725926
10   AG-GCTT-AGGCTT
Rev.of_WB235_2:13796378-13796708 Satlength=331 Nr of Repeats=23
RepeatLength=12 seed=GCCTAAGCCT Num.seqs=15 Similarity=0.803175
10   AG-GCTT-AGGCTT
Rev.of_WB235_2:13806123-13806285 Satlength=163 Nr of Repeats=10
RepeatLength=12 seed=GCCTAAGCCT Num.seqs=7 Similarity=0.798942
10   AG-GCTT-AGGCTT
WB235_2:13809662-13809758 Satlength=97 Nr of Repeats=7 RepeatLength=12
seed=TTAGGCTTAG Num.seqs=6 Similarity=0.755556
10   AG-GCTT-AGGCTT
Rev.of_WB235_2:13934639-13934753 Satlength=115 Nr of Repeats=7
RepeatLength=12 seed=AGCCTAAGCC Num.seqs=5 Similarity=0.733333
11   AG-GCTT-AGGCTT
WB235_2:13974202-13974430 Satlength=229 Nr of Repeats=14 RepeatLength=12
seed=GCTTAGGCTT Num.seqs=11 Similarity=0.711888
2   AG-GCTT-AGGCTT
WB235_2:14006879-14006969 Satlength=91 Nr of Repeats=5 RepeatLength=12
seed=TAGGCTTAGG Num.seqs=3 Similarity=0.851852
11   AG-GCTT-AGGCTT
WB235_2:14023987-14024052 Satlength=66 Nr of Repeats=4 RepeatLength=12
seed=TAGGCTTAGG Num.seqs=3 Similarity=0.925926
11   AG-GCTT-AGGCTT
WB235_2:14893703-14893787 Satlength=85 Nr of Repeats=6 RepeatLength=12
seed=TTAGGCTTAG Num.seqs=4 Similarity=0.814815
10   AG-GCTT-AGGCTT
Rev.of_WB235_2:14955292-14955352 Satlength=61 Nr of Repeats=5
RepeatLength=12 seed=AAGCCTAAGC Num.seqs=5 Similarity=0.888889
12   AG-GCTT-AGGCTT
WB235_2:15279229-15279409 Satlength=181 Nr of Repeats=15 RepeatLength=12
seed=TTAGGCTTAG Num.seqs=15 Similarity=0.970370
10   AG-GCTT-AGGCTT
Rev.of_WB235_3:0-119 Satlength=120 Nr of Repeats=10 RepeatLength=12
seed=CCTAAGCCTA Num.seqs=10 Similarity=1.000000
9   AG-GCTT-AGGCTT
Rev.of_WB235_3:118094-118166 Satlength=73 Nr of Repeats=5 RepeatLength=12
seed=GCCTAAGCCT Num.seqs=3 Similarity=0.851852
10   AG-GCTT-AGGCTT
Rev.of_WB235_3:209292-209524 Satlength=233 Nr of Repeats=8
RepeatLength=12 seed=AAGCCTAAGC Num.seqs=5 Similarity=0.822222
12   AG-GCTT-AGGCTT
Rev.of_WB235_3:1052407-1052587 Satlength=181 Nr of Repeats=10
RepeatLength=12 seed=AAGCCTAAGC Num.seqs=7 Similarity=0.841270
12   AG-GCTT-AGGCTT
Rev.of_WB235_3:1714052-1714520 Satlength=469 Nr of Repeats=35
RepeatLength=12 seed=CTAAGCCTAA Num.seqs=27 Similarity=0.758492
2   AG-GCTT-AGGCTT
WB235_3:1964229-1964835 Satlength=607 Nr of Repeats=30 RepeatLength=12
seed=TAGGCTTAGG Num.seqs=18 Similarity=0.824256
11   AG-GCTT-AGGCTT

WB235_3:3346751-3346936 Satlength=186 Nr of Repeats=12 RepeatLength=12 seed=TAGGCTTAGG Num.seqs=9 Similarity=0.901235
11   AG-GCTT-AGGCTT
Rev.of_WB235_3:3684600-3684680 Satlength=81 Nr of Repeats=5 RepeatLength=12 seed=TAAGCCTAAG Num.seqs=3 Similarity=0.777778
13   AG-GCTT-AGGCTT
WB235_3:10924768-10924858 Satlength=91 Nr of Repeats=6 RepeatLength=12 seed=GGCTTAGGCT Num.seqs=5 Similarity=0.822222
13   AG-GCTT-AGGCTT
WB235_3:10975223-10975307 Satlength=85 Nr of Repeats=5 RepeatLength=12 seed=GGCTTAGGCT Num.seqs=3 Similarity=0.777778
13   AG-GCTT-AGGCTT
WB235_3:11207917-11208073 Satlength=157 Nr of Repeats=10 RepeatLength=12 seed=GCTTAGGCTT Num.seqs=6 Similarity=0.755556
2   AG-GCTT-AGGCTT
WB235_3:11424870-11425146 Satlength=277 Nr of Repeats=12 RepeatLength=12 seed=TTAGGCTTAG Num.seqs=8 Similarity=0.916667
10   AG-GCTT-AGGCTT
WB235_3:11475036-11475156 Satlength=121 Nr of Repeats=10 RepeatLength=12 seed=CTTAGGCTTA Num.seqs=8 Similarity=0.797619
9   AG-GCTT-AGGCTT
WB235_3:11707265-11707854 Satlength=590 Nr of Repeats=30 RepeatLength=12 seed=GGCTTAGGCT Num.seqs=20 Similarity=0.774854
13   AG-GCTT-AGGCTT
Rev.of_WB235_3:11722253-11722390 Satlength=138 Nr of Repeats=8 RepeatLength=12 seed=TAAGCCTAAG Num.seqs=5 Similarity=0.822222
13   AG-GCTT-AGGCTT
Rev.of_WB235_3:11737191-11737281 Satlength=91 Nr of Repeats=6 RepeatLength=12 seed=CCTAAGCCTA Num.seqs=5 Similarity=0.833333
9   AG-GCTT-AGGCTT
Rev.of_WB235_3:11784840-11785090 Satlength=251 Nr of Repeats=9 RepeatLength=12 seed=TAAGCCTAAG Num.seqs=7 Similarity=0.936508
13   AG-GCTT-AGGCTT
Rev.of_WB235_3:12130507-12130633 Satlength=127 Nr of Repeats=9 RepeatLength=12 seed=TAAGCCTAAG Num.seqs=6 Similarity=0.814815
13   AG-GCTT-AGGCTT
WB235_3:12143931-12144015 Satlength=85 Nr of Repeats=7 RepeatLength=12 seed=GGCTTAGGCT Num.seqs=7 Similarity=0.968254
13   AG-GCTT-AGGCTT
Rev.of_WB235_3:12944421-12944571 Satlength=151 Nr of Repeats=9 RepeatLength=12 seed=TAAGCCTAAG Num.seqs=7 Similarity=0.830688
13   AG-GCTT-AGGCTT
WB235_3:13783590-13783788 Satlength=199 Nr of Repeats=16 RepeatLength=12 seed=CTTAGGCTTA Num.seqs=15 Similarity=1.000000
9   AG-GCTT-AGGCTT
Rev.of_WB235_4:0-137 Satlength=138 Nr of Repeats=11 RepeatLength=12 seed=CCTAAGCCTA Num.seqs=10 Similarity=1.000000
9   AG-GCTT-AGGCTT
WB235_4:545946-546072 Satlength=127 Nr of Repeats=9 RepeatLength=12 seed=TAGGCTTAGG Num.seqs=7 Similarity=0.788360
11   AG-GCTT-AGGCTT
Rev.of_WB235_4:1069432-1069486 Satlength=55 Nr of Repeats=4 RepeatLength=12 seed=AGCCTAAGCC Num.seqs=3 Similarity=0.851852
11   AG-GCTT-AGGCTT

WB235_4:1071381-1071429 Satlength=49 Nr of Repeats=4 RepeatLength=12 seed=AGGCTTAGGC Num.seqs=4 Similarity=0.888889
12   AG-GCTT-AGGCTT
WB235_4:1355992-1356408 Satlength=417 Nr of Repeats=8 RepeatLength=12 seed=TAGGCTTAGG Num.seqs=5 Similarity=0.822222
11   AG-GCTT-AGGCTT
WB235_4:1494116-1494472 Satlength=357 Nr of Repeats=5 RepeatLength=12 seed=AGGCTTAGGC Num.seqs=4 Similarity=0.740741
12   AG-GCTT-AGGCTT
WB235_4:1628395-1628484 Satlength=90 Nr of Repeats=6 RepeatLength=12 seed=TTAGGCTTAG Num.seqs=4 Similarity=0.888889
10   AG-GCTT-AGGCTT
Rev.of_WB235_4:1689083-1689293 Satlength=211 Nr of Repeats=11 RepeatLength=12 seed=CTAAGCCTAA Num.seqs=7 Similarity=0.777778
2   AG-GCTT-AGGCTT
Rev.of_WB235_4:1752199-1752289 Satlength=91 Nr of Repeats=5 RepeatLength=12 seed=TAAGCCTAAG Num.seqs=4 Similarity=0.796296
13   AG-GCTT-AGGCTT
Rev.of_WB235_4:3166479-3166602 Satlength=124 Nr of Repeats=7 RepeatLength=12 seed=CCTAAGCCTA Num.seqs=5 Similarity=0.800000
9   AG-GCTT-AGGCTT
Rev.of_WB235_4:3991189-3991890 Satlength=702 Nr of Repeats=6 RepeatLength=12 seed=TAAGCCTAAG Num.seqs=4 Similarity=0.759259
13   AG-GCTT-AGGCTT
WB235_4:17493616-17493814 Satlength=199 Nr of Repeats=16 RepeatLength=12 seed=GGCTTAGGCT Num.seqs=15 Similarity=1.000000
13   AG-GCTT-AGGCTT
Rev.of_WB235_5:6-311 Satlength=306 Nr of Repeats=25 RepeatLength=12 seed=TAAGCCTAAG Num.seqs=24 Similarity=0.964573
13   AG-GCTT-AGGCTT
WB235_5:1129473-1129617 Satlength=145 Nr of Repeats=10 RepeatLength=12 seed=GGCTTAGGCT Num.seqs=7 Similarity=0.814815
13   AG-GCTT-AGGCTT
Rev.of_WB235_5:1142352-1142454 Satlength=103 Nr of Repeats=5 RepeatLength=12 seed=CTAAGCCTAA Num.seqs=3 Similarity=0.851852
2   AG-GCTT-AGGCTT
Rev.of_WB235_5:1227227-1227275 Satlength=49 Nr of Repeats=4 RepeatLength=12 seed=AAGCCTAAGC Num.seqs=4 Similarity=0.944444
12   AG-GCTT-AGGCTT
Rev.of_WB235_5:1230606-1230858 Satlength=253 Nr of Repeats=19 RepeatLength=12 seed=AAGCCTAAGC Num.seqs=14 Similarity=0.804640
12   AG-GCTT-AGGCTT
Rev.of_WB235_5:1236678-1236956 Satlength=279 Nr of Repeats=15 RepeatLength=12 seed=CTAAGCCTAA Num.seqs=9 Similarity=0.836420
2   AG-GCTT-AGGCTT
WB235_5:1243605-1243701 Satlength=97 Nr of Repeats=7 RepeatLength=12 seed=CTTAGGCTTA Num.seqs=6 Similarity=0.822222
9   AG-GCTT-AGGCTT
Rev.of_WB235_5:1341430-1341604 Satlength=175 Nr of Repeats=13 RepeatLength=12 seed=CTAAGCCTAA Num.seqs=11 Similarity=0.826263
2   AG-GCTT-AGGCTT
Rev.of_WB235_5:1423375-1423735 Satlength=361 Nr of Repeats=24 RepeatLength=12 seed=CCTAAGCCTA Num.seqs=17 Similarity=0.861928
9   AG-GCTT-AGGCTT

Rev.of_WB235_5:1481682-1481898 Satlength=217 Nr of Repeats=10 RepeatLength=12 seed=AGCCTAAGCC Num.seqs=7 Similarity=0.788360
11 AG-GCTT-AGGCTT
Rev.of_WB235_5:2586211-2586358 Satlength=148 Nr of Repeats=6 RepeatLength=12 seed=CCTAAGCCTA Num.seqs=4 Similarity=0.833333
9 AG-GCTT-AGGCTT
Rev.of_WB235_5:2712581-2712737 Satlength=157 Nr of Repeats=13 RepeatLength=12 seed=CTAAGCCTAA Num.seqs=13 Similarity=0.839031
2 AG-GCTT-AGGCTT
WB235_5:15615355-15615426 Satlength=72 Nr of Repeats=5 RepeatLength=12 seed=CTTAGGCTTA Num.seqs=3 Similarity=0.925926
9 AG-GCTT-AGGCTT
WB235_5:18566411-18566663 Satlength=253 Nr of Repeats=16 RepeatLength=12 seed=AGGCTTAGGC Num.seqs=10 Similarity=0.935802
12 AG-GCTT-AGGCTT
Rev.of_WB235_5:18581399-18581759 Satlength=361 Nr of Repeats=22 RepeatLength=12 seed=AGCCTAAGCC Num.seqs=15 Similarity=0.860317
11 AG-GCTT-AGGCTT
Rev.of_WB235_5:18588013-18588271 Satlength=259 Nr of Repeats=18 RepeatLength=12 seed=TAAGCCTAAG Num.seqs=13 Similarity=0.874644
13 AG-GCTT-AGGCTT
Rev.of_WB235_5:18616302-18616398 Satlength=97 Nr of Repeats=6 RepeatLength=12 seed=CTAAGCCTAA Num.seqs=4 Similarity=0.888889
2 AG-GCTT-AGGCTT
Rev.of_WB235_5:18632668-18632722 Satlength=55 Nr of Repeats=4 RepeatLength=12 seed=TAAGCCTAAG Num.seqs=3 Similarity=0.851852
13 AG-GCTT-AGGCTT
WB235_5:18639700-18639844 Satlength=145 Nr of Repeats=9 RepeatLength=12 seed=GCTTAGGCTT Num.seqs=6 Similarity=0.925926
2 AG-GCTT-AGGCTT
Rev.of_WB235_5:19232598-19232808 Satlength=211 Nr of Repeats=15 RepeatLength=12 seed=GCCTAAGCCT Num.seqs=11 Similarity=0.818182
10 AG-GCTT-AGGCTT
WB235_5:19517965-19518323 Satlength=359 Nr of Repeats=22 RepeatLength=12 seed=CTTAGGCTTA Num.seqs=15 Similarity=0.813757
9 AG-GCTT-AGGCTT
WB235_5:19528638-19528776 Satlength=139 Nr of Repeats=11 RepeatLength=12 seed=CTTAGGCTTA Num.seqs=10 Similarity=0.938272
9 AG-GCTT-AGGCTT
Rev.of_WB235_5:19801650-19801767 Satlength=118 Nr of Repeats=5 RepeatLength=12 seed=AAGCCTAAGC Num.seqs=3 Similarity=0.851852
12 AG-GCTT-AGGCTT
WB235_5:20923994-20924168 Satlength=175 Nr of Repeats=14 RepeatLength=12 seed=GCTTAGGCTT Num.seqs=13 Similarity=1.000000
2 AG-GCTT-AGGCTT
Rev.of_WB235_6:0-251 Satlength=252 Nr of Repeats=21 RepeatLength=12 seed=CTAAGCCTAA Num.seqs=21 Similarity=1.000000
2 AG-GCTT-AGGCTT
WB235_6:2563170-2563230 Satlength=61 Nr of Repeats=5 RepeatLength=12 seed=AGGCTTAGGC Num.seqs=5 Similarity=0.888889
12 AG-GCTT-AGGCTT
WB235_6:17718013-17718930 Satlength=918 Nr of Repeats=12 RepeatLength=12 seed=TTAGGCTTAG Num.seqs=11 Similarity=0.979798
10 AG-GCTT-AGGCTT

WB235_1:477877-478046 Satlength=170 Nr of Repeats=13 RepeatLength=12 seed=TAGGCTTAGG Num.seqs=8 Similarity=0.813492
11   AG-GCNT-AGGCTT
Rev.of_WB235_1:1000379-1000469 Satlength=91 Nr of Repeats=5 RepeatLength=12 seed=AGCCTAAGCC Num.seqs=3 Similarity=0.740741
11   AG-GCNT-AGGCTT
Rev.of_WB235_1:12175889-12176021 Satlength=133 Nr of Repeats=9 RepeatLength=12 seed=GCCTAAGCCT Num.seqs=7 Similarity=0.767196
10   AG-GCTT-AGGCNT
Rev.of_WB235_2:2817169-2817253 Satlength=85 Nr of Repeats=6 RepeatLength=12 seed=CCTAAGCCTA Num.seqs=4 Similarity=0.754274
21   AG-GCTT-AGGCGT
Rev.of_WB235_4:4459427-4459589 Satlength=163 Nr of Repeats=12 RepeatLength=12 seed=GCCTAAGCCT Num.seqs=11 Similarity=0.751515
22   AG-GCTT-AGGCGT
Rev.of_WB235_1:12277734-12277806 Satlength=73 Nr of Repeats=5 RepeatLength=12 seed=AAGCCTAAGC Num.seqs=3 Similarity=0.777778
12   AG-GCTT-CGGCTT
WB235_2:1224948-1225028 Satlength=81 Nr of Repeats=5 RepeatLength=12 seed=TTAGGCTTAG Num.seqs=3 Similarity=0.925926
10   AG-GCTT-AGGTTT
WB235_4:3608664-3608778 Satlength=115 Nr of Repeats=7 RepeatLength=12 seed=TTAGGCTTAG Num.seqs=5 Similarity=0.866667
10   AG-GCTT-AGGATT
WB235_4:1632727-1632835 Satlength=109 Nr of Repeats=9 RepeatLength=12 seed=CTTAAGCTTA Num.seqs=9 Similarity=0.975309
9   AA-GCTT-AGGCTT
Rev.of_WB235_4:1890247-1890331 Satlength=85 Nr of Repeats=5 RepeatLength=12 seed=TAAGCCTAAG Num.seqs=3 Similarity=0.851852
13   AA-GCTT-AGGCTT
Rev.of_WB235_4:3455422-3455812 Satlength=391 Nr of Repeats=27 RepeatLength=12 seed=TAAGCCTAAG Num.seqs=21 Similarity=0.808059
13   AA-GCTT-AGGCTT
WB235_5:365726-365780 Satlength=55 Nr of Repeats=4 RepeatLength=12 seed=AGGCTTAGGC Num.seqs=3 Similarity=1.000000
12   AG-GCTT-AGGCTA
Rev.of_WB235_2:4365202-4365284 Satlength=83 Nr of Repeats=4 RepeatLength=12 seed=TAAGCCTAAG Num.seqs=3 Similarity=0.925926
13   AG-TCTT-AGGCTT
WB235_2:12239992-12240106 Satlength=115 Nr of Repeats=7 RepeatLength=12 seed=TTAGTCTTAG Num.seqs=5 Similarity=0.833333
22   AG-TCTT-AGGCTT
Rev.of_WB235_6:1509783-1509855 Satlength=73 Nr of Repeats=5 RepeatLength=12 seed=CTAAGCCTAA Num.seqs=4 Similarity=0.851852
14   AG-TCTT-TGGCTT
Rev.of_WB235_2:2632149-2632578 Satlength=430 Nr of Repeats=33 RepeatLength=13 seed=AGCCTAAGCT Num.seqs=33 Similarity=0.987568
12   AGAGCTT-AGGCTT
Rev.of_WB235_1:11984232-11984346 Satlength=115 Nr of Repeats=7 RepeatLength=12 seed=CTAAGCCTAA Num.seqs=5 Similarity=0.766667
2   AG-GTTT-AGGCTT
WB235_4:13007463-13008066 Satlength=604 Nr of Repeats=12 RepeatLength=12 seed=GCTTGGGTTT Num.seqs=9 Similarity=0.907407
8   GG-GTTT-AGGCTT

WB235_4:13007463-13008198 Satlength=736 Nr of Repeats=17 RepeatLength=12 seed=GCTTGGGTTT Num.seqs=11 Similarity=0.903030
8   GG-GTTT-AGGCTT
Rev.of_WB235_1:1653240-1653360 Satlength=121 Nr of Repeats=7 RepeatLength=12 seed=AGCCTAAACC Num.seqs=5 Similarity=0.702564
11   AG-GTTT-AGGCTC
WB235_3:1256230-1256326 Satlength=97 Nr of Repeats=5 RepeatLength=12 seed=AGGCTTAGGC Num.seqs=3 Similarity=0.925926
12   AG-GCTT-AGGCTC
WB235_3:1264671-1264767 Satlength=97 Nr of Repeats=5 RepeatLength=12 seed=AGGCTTAGGC Num.seqs=3 Similarity=0.925926
12   AG-GCTT-AGGCTC
WB235_3:1761961-1762051 Satlength=91 Nr of Repeats=6 RepeatLength=12 seed=AGGCTTAGGC Num.seqs=4 Similarity=0.668803
12   AG-GCTT-AGGCTC
WB235_5:1305908-1306004 Satlength=97 Nr of Repeats=7 RepeatLength=12 seed=AGGCTTAGGC Num.seqs=5 Similarity=1.000000
12   AG-GCTT-AGGCTC
Rev.of_WB235_5:1825303-1825405 Satlength=103 Nr of Repeats=7 RepeatLength=12 seed=AGCCTGAGCC Num.seqs=5 Similarity=0.955556
17   AG-GCTT-AGGCTC
WB235_5:2394729-2394777 Satlength=49 Nr of Repeats=4 RepeatLength=12 seed=AGGCTTAGGC Num.seqs=4 Similarity=0.870370
12   AG-GCTT-AGGCTC
WB235_5:2503008-2503056 Satlength=49 Nr of Repeats=4 RepeatLength=12 seed=AGGCTTAGGC Num.seqs=4 Similarity=0.870370
12   AG-GCTT-AGGCTC
Rev.of_WB235_5:18597908-18598040 Satlength=133 Nr of Repeats=9 RepeatLength=12 seed=AGCCTAAGCC Num.seqs=7 Similarity=0.841270
23   AG-GCTT-AGGCTC
WB235_1:11655695-11656421 Satlength=727 Nr of Repeats=60 RepeatLength=12 seed=AGGCTCAGGC Num.seqs=59 Similarity=0.880322
18   AG-GCTC-AGGCTC
WB235_2:645177-645231 Satlength=55 Nr of Repeats=4 RepeatLength=12 seed=GGCTCAGGCT Num.seqs=3 Similarity=1.000000
19   AG-GCTC-AGGCTC
Rev.of_WB235_3:1656487-1657087 Satlength=601 Nr of Repeats=50 RepeatLength=12 seed=TGAGCCTGAG Num.seqs=50 Similarity=0.966440
19   AG-GCTC-AGGCTC
Rev.of_WB235_3:11783258-11783348 Satlength=91 Nr of Repeats=4 RepeatLength=12 seed=AGCCTGAGCC Num.seqs=3 Similarity=0.851852
23   AG-GCTC-AGGCTC
WB235_5:18678760-18678838 Satlength=79 Nr of Repeats=6 RepeatLength=12 seed=GGCTCAGGCT Num.seqs=5 Similarity=0.955556
19   AG-GCTC-AGGCTC
Rev.of_WB235_5:18722989-18723101 Satlength=113 Nr of Repeats=7 RepeatLength=12 seed=AGCCTGAGCC Num.seqs=6 Similarity=1.000000
23   AG-GCTC-AGGCTC
WB235_5:19229130-19229634 Satlength=505 Nr of Repeats=37 RepeatLength=12 seed=AGGCTCAGGC Num.seqs=32 Similarity=0.903226
18   AG-GCTC-AGGCTC
WB235_2:13353644-13353730 Satlength=87 Nr of Repeats=6 RepeatLength=12 seed=CTTAGGCTTA Num.seqs=4 Similarity=0.743590
10   AG-GCTTAAGNCTT

WB235_4:1785293-1785383 Satlength=91 Nr of Repeats=7 RepeatLength=12 seed=TTAGGATTAG Num.seqs=6 Similarity=0.962963
10   AG-GATT-AGGATT
Rev.of_WB235_1:11540263-11540521 Satlength=259 Nr of Repeats=16 RepeatLength=12 seed=AAGCCAAAGC Num.seqs=13 Similarity=0.854701
12   TG-GCTT-TGGCTT
WB235_2:13974189-13974267 Satlength=79 Nr of Repeats=6 RepeatLength=12 seed=GGCTTTGGCT Num.seqs=5 Similarity=1.000000
19   TG-GCTT-TGGCTT
Rev.of_WB235_3:2456635-2456707 Satlength=73 Nr of Repeats=5 RepeatLength=12 seed=CCAAGCCCAA Num.seqs=3 Similarity=0.851852
20   GG-GCTT-GGGCTT
WB235_5:20001413-20002864 Satlength=1452 Nr of Repeats=51 RepeatLength=12 seed=GCTTGGGCTT Num.seqs=38 Similarity=0.904694
20   GG-GCTT-GGGCTT
Rev.of_WB235_5:3767901-3768001 Satlength=101 Nr of Repeats=6 RepeatLength=10 seed=TAGCCATGCC Num.seqs=4 Similarity=0.933333
1   AG-GCAT--GGC-T
Rev.of_WB235_1:11032650-11032782 Satlength=133 Nr of Repeats=12 RepeatLength=11 seed=TATGCCTAGC Num.seqs=12 Similarity=1.000000
7   AG-GCAT-AGGC-T
Rev.of_WB235_2:1250454-1251360 Satlength=907 Nr of Repeats=73 RepeatLength=12 seed=CTATGCCTAT Num.seqs=70 Similarity=0.936784
14   AG-GCAT-AGGCAT
Rev.of_WB235_2:1252963-1253498 Satlength=536 Nr of Repeats=44 RepeatLength=12 seed=CTATGCCTAT Num.seqs=42 Similarity=0.937798
14   AG-GCAT-AGGCAT
Rev.of_WB235_2:1421457-1422023 Satlength=567 Nr of Repeats=41 RepeatLength=12 seed=CCTATGCCTA Num.seqs=30 Similarity=0.874585
21   AG-GCAT-AGGCAT
WB235_2:2957411-2957495 Satlength=85 Nr of Repeats=7 RepeatLength=12 seed=ATAGGCATAG Num.seqs=7 Similarity=1.000000
22   AG-GCAT-AGGCAT
WB235_2:14166594-14166830 Satlength=237 Nr of Repeats=18 RepeatLength=12 seed=ATAGGCATAG Num.seqs=11 Similarity=0.834343
22   AG-GCAT-AGGCAT
WB235_3:12302154-12302292 Satlength=139 Nr of Repeats=9 RepeatLength=12 seed=TAGGCATAGG Num.seqs=6 Similarity=0.829630
23   AG-GCAT-AGGCAT
WB235_4:1429990-1430044 Satlength=55 Nr of Repeats=4 RepeatLength=12 seed=CATAGGCATA Num.seqs=3 Similarity=0.925926
21   AG-GCAT-AGGCAT
WB235_6:14126276-14126426 Satlength=151 Nr of Repeats=12 RepeatLength=12 seed=ATAGGCATAG Num.seqs=11 Similarity=0.979798
22   AG-GCAT-AGGCAT

*
Consensus:

AGGCTTAGGCTT

>WB235_Fam_3_15_136 Nr. of seq. 136 Alignment length(with gaps) = 15 Alignment score = 0.942162

WB235_1:222749-222854 Satlength=106 Nr of Repeats=5 RepeatLength=15
seed=AGACCCATCG Num.seqs=4 Similarity=0.911111
0   AGACCCATCGTGGTG
WB235_1:281468-282278 Satlength=811 Nr of Repeats=52 RepeatLength=15
seed=TCGTGGTGAG Num.seqs=48 Similarity=0.862569
7   AGACCCATCGTGGTG
WB235_1:689715-689895 Satlength=181 Nr of Repeats=12 RepeatLength=15
seed=GGTGAGACCC Num.seqs=12 Similarity=0.900337
11   AGACCCATCGTGGTG
Rev.of_WB235_1:4574052-4574607 Satlength=556 Nr of Repeats=34
RepeatLength=15 seed=GGGTCTCACC Num.seqs=31 Similarity=0.896583
6   AGACCCATCGTGGTG
WB235_1:7868293-7868548 Satlength=256 Nr of Repeats=17 RepeatLength=15
seed=ACCCATCGTG Num.seqs=17 Similarity=0.847712
2   AGACCCATCGTGGTG
Rev.of_WB235_1:9819851-9820331 Satlength=481 Nr of Repeats=31
RepeatLength=15 seed=CACGATGGGT Num.seqs=30 Similarity=0.857982
12   AGACCCATCGTGGTG
WB235_1:11144860-11145055 Satlength=196 Nr of Repeats=13 RepeatLength=15
seed=TCGTGGTGAG Num.seqs=13 Similarity=0.842735
7   AGACCCATCGTGGTG
WB235_1:11480157-11480277 Satlength=121 Nr of Repeats=8 RepeatLength=15
seed=TCGTGGTGAG Num.seqs=8 Similarity=0.930159
7   AGACCCATCGTGGTG
Rev.of_WB235_2:451705-452550 Satlength=846 Nr of Repeats=32
RepeatLength=15 seed=TCTCACCACG Num.seqs=30 Similarity=0.884138
3   AGACCCATCGTGGTG
Rev.of_WB235_2:633057-633477 Satlength=421 Nr of Repeats=27
RepeatLength=15 seed=GGGTCTCACC Num.seqs=26 Similarity=0.898256
6   AGACCCATCGTGGTG
WB235_2:694364-699056 Satlength=4693 Nr of Repeats=287 RepeatLength=15
seed=GAGACCCATC Num.seqs=267 Similarity=0.890323
14   AGACCCATCGTGGTG
WB235_2:694391-699449 Satlength=5059 Nr of Repeats=247 RepeatLength=15
seed=GGTGAGACCC Num.seqs=190 Similarity=0.923290
11   AGACCCATCGTGGTG
WB235_2:1037817-1039287 Satlength=1471 Nr of Repeats=95 RepeatLength=15
seed=TCGTGGTGAG Num.seqs=92 Similarity=0.911069
7   AGACCCATCGTGGTG
WB235_2:3365918-3365978 Satlength=61 Nr of Repeats=4 RepeatLength=15
seed=GGTGAGACCC Num.seqs=4 Similarity=0.911111
11   AGACCCATCGTGGTG
WB235_2:4684295-4684838 Satlength=544 Nr of Repeats=21 RepeatLength=15
seed=TGGTGAGACC Num.seqs=13 Similarity=0.879202
10   AGACCCATCGTGGTG
Rev.of_WB235_2:8460726-8460801 Satlength=76 Nr of Repeats=4
RepeatLength=15 seed=GGGTCTCACC Num.seqs=3 Similarity=0.822222
6   AGACCCATCGTGGTG
Rev.of_WB235_2:9633989-9634259 Satlength=271 Nr of Repeats=17
RepeatLength=15 seed=CCACGATGGG Num.seqs=16 Similarity=0.891852
13   AGACCCATCGTGGTG
WB235_2:12649559-12650818 Satlength=1260 Nr of Repeats=83 RepeatLength=15
seed=TCGTGGTGAG Num.seqs=82 Similarity=0.874683
7   AGACCCATCGTGGTG

WB235_2:12649559-12651613 Satlength=2055 Nr of Repeats=136 RepeatLength=15 seed=TCGTGGTGAG Num.seqs=133 Similarity=0.866505
7 AGACCCATCGTGGTG
WB235_2:14836705-14836840 Satlength=136 Nr of Repeats=9 RepeatLength=15 seed=AGACCCATCG Num.seqs=9 Similarity=0.881481
0 AGACCCATCGTGGTG
Rev.of_WB235_2:14878607-14878727 Satlength=121 Nr of Repeats=7 RepeatLength=15 seed=CCACGATGGG Num.seqs=6 Similarity=0.881481
13 AGACCCATCGTGGTG
Rev.of_WB235_3:2502140-2502620 Satlength=481 Nr of Repeats=29 RepeatLength=15 seed=GGTCTCACCA Num.seqs=26 Similarity=0.920957
5 AGACCCATCGTGGTG
WB235_3:3896301-3896751 Satlength=451 Nr of Repeats=30 RepeatLength=15 seed=CGTGGTGAGA Num.seqs=30 Similarity=0.932158
8 AGACCCATCGTGGTG
Rev.of_WB235_3:13448299-13448524 Satlength=226 Nr of Repeats=15 RepeatLength=15 seed=CCACGATGGG Num.seqs=15 Similarity=0.930582
13 AGACCCATCGTGGTG
WB235_3:13750231-13751043 Satlength=813 Nr of Repeats=52 RepeatLength=15 seed=GTGGTGAGAC Num.seqs=46 Similarity=0.862158
9 AGACCCATCGTGGTG
WB235_3:13753764-13754664 Satlength=901 Nr of Repeats=60 RepeatLength=15 seed=TCGTGGTGAG Num.seqs=60 Similarity=0.880778
7 AGACCCATCGTGGTG
WB235_4:281265-281745 Satlength=481 Nr of Repeats=32 RepeatLength=15 seed=TCGTGGTGAG Num.seqs=32 Similarity=0.882258
7 AGACCCATCGTGGTG
Rev.of_WB235_4:1154252-1154492 Satlength=241 Nr of Repeats=16 RepeatLength=15 seed=GGTCTCACCA Num.seqs=16 Similarity=0.880741
5 AGACCCATCGTGGTG
WB235_4:1440391-1440556 Satlength=166 Nr of Repeats=11 RepeatLength=15 seed=AGACCCATCG Num.seqs=11 Similarity=0.880404
0 AGACCCATCGTGGTG
Rev.of_WB235_4:2096897-2096972 Satlength=76 Nr of Repeats=5 RepeatLength=15 seed=GGGTCTCACC Num.seqs=5 Similarity=0.840000
6 AGACCCATCGTGGTG
Rev.of_WB235_4:2244962-2245187 Satlength=226 Nr of Repeats=15 RepeatLength=15 seed=CGATGGGTCT Num.seqs=15 Similarity=0.914497
10 AGACCCATCGTGGTG
Rev.of_WB235_4:3052982-3053132 Satlength=151 Nr of Repeats=8 RepeatLength=15 seed=CACGATGGGT Num.seqs=7 Similarity=0.843386
12 AGACCCATCGTGGTG
WB235_4:3670737-3670797 Satlength=61 Nr of Repeats=4 RepeatLength=15 seed=TCGTGGTGAG Num.seqs=4 Similarity=0.940741
7 AGACCCATCGTGGTG
WB235_4:4898047-4898612 Satlength=566 Nr of Repeats=18 RepeatLength=15 seed=GAGACCCATC Num.seqs=15 Similarity=0.903492
14 AGACCCATCGTGGTG
Rev.of_WB235_4:4921570-4922365 Satlength=796 Nr of Repeats=47 RepeatLength=15 seed=GGGTCTCACC Num.seqs=41 Similarity=0.943631
6 AGACCCATCGTGGTG
Rev.of_WB235_4:4923839-4924634 Satlength=796 Nr of Repeats=47 RepeatLength=15 seed=GGGTCTCACC Num.seqs=41 Similarity=0.943631
6 AGACCCATCGTGGTG

Rev.of_WB235_4:4926108-4926918 Satlength=811 Nr of Repeats=48 RepeatLength=15 seed=GGGTCTCACC Num.seqs=42 Similarity=0.944767
6 AGACCCATCGTGGTG
Rev.of_WB235_4:5456641-5456776 Satlength=136 Nr of Repeats=9 RepeatLength=15 seed=CCACGATGGG Num.seqs=7 Similarity=0.898413
13 AGACCCATCGTGGTG
Rev.of_WB235_4:8073709-8073979 Satlength=271 Nr of Repeats=18 RepeatLength=15 seed=CTCACCACGA Num.seqs=18 Similarity=0.902977
2 AGACCCATCGTGGTG
Rev.of_WB235_4:8469615-8470279 Satlength=665 Nr of Repeats=41 RepeatLength=15 seed=TCTCACCACG Num.seqs=39 Similarity=0.951297
3 AGACCCATCGTGGTG
Rev.of_WB235_4:9066182-9067563 Satlength=1382 Nr of Repeats=81 RepeatLength=15 seed=CTCACCACGA Num.seqs=60 Similarity=0.961833
2 AGACCCATCGTGGTG
Rev.of_WB235_4:9410150-9411415 Satlength=1266 Nr of Repeats=46 RepeatLength=15 seed=ACGATGGGTC Num.seqs=43 Similarity=0.911013
11 AGACCCATCGTGGTG
WB235_4:10262913-10263301 Satlength=389 Nr of Repeats=26 RepeatLength=15 seed=GACCCATCGT Num.seqs=25 Similarity=0.898074
1 AGACCCATCGTGGTG
WB235_4:13462457-13462800 Satlength=344 Nr of Repeats=22 RepeatLength=15 seed=ACCCATCGTG Num.seqs=19 Similarity=0.894997
2 AGACCCATCGTGGTG
Rev.of_WB235_4:13884400-13884820 Satlength=421 Nr of Repeats=24 RepeatLength=15 seed=ACGATGGGTC Num.seqs=21 Similarity=0.916191
11 AGACCCATCGTGGTG
WB235_4:13922430-13922519 Satlength=90 Nr of Repeats=6 RepeatLength=15 seed=ACCCATCGTG Num.seqs=5 Similarity=0.946667
2 AGACCCATCGTGGTG
WB235_4:14476805-14480237 Satlength=3433 Nr of Repeats=67 RepeatLength=15 seed=CGTGGTGAGA Num.seqs=53 Similarity=0.889437
8 AGACCCATCGTGGTG
WB235_4:15188409-15188529 Satlength=121 Nr of Repeats=8 RepeatLength=15 seed=GGTGAGACCC Num.seqs=8 Similarity=0.926984
11 AGACCCATCGTGGTG
WB235_4:15319979-15320069 Satlength=91 Nr of Repeats=6 RepeatLength=15 seed=TCGTGGTGAG Num.seqs=6 Similarity=0.887407
7 AGACCCATCGTGGTG
Rev.of_WB235_4:15375074-15376143 Satlength=1070 Nr of Repeats=51 RepeatLength=15 seed=CCACGATGGG Num.seqs=44 Similarity=0.911299
13 AGACCCATCGTGGTG
Rev.of_WB235_4:15766277-15766397 Satlength=121 Nr of Repeats=8 RepeatLength=15 seed=GGGTCTCACC Num.seqs=8 Similarity=0.939683
6 AGACCCATCGTGGTG
Rev.of_WB235_4:15766300-15766555 Satlength=256 Nr of Repeats=17 RepeatLength=15 seed=CCACGATGGG Num.seqs=17 Similarity=0.875163
13 AGACCCATCGTGGTG
WB235_4:15826645-15826960 Satlength=316 Nr of Repeats=21 RepeatLength=15 seed=CCCATCGTGG Num.seqs=21 Similarity=0.916191
3 AGACCCATCGTGGTG
Rev.of_WB235_4:15971205-15972491 Satlength=1287 Nr of Repeats=15 RepeatLength=15 seed=CACGATGGGT Num.seqs=13 Similarity=0.863248
12 AGACCCATCGTGGTG

Rev.of_WB235_4:16458853-16459033 Satlength=181 Nr of Repeats=10 RepeatLength=15 seed=TGGGTCTCAC Num.seqs=8 Similarity=0.895238
7 AGACCCATCGTGGTG
WB235_4:16880641-16880761 Satlength=121 Nr of Repeats=8 RepeatLength=15 seed=TCGTGGTGAG Num.seqs=8 Similarity=0.866667
7 AGACCCATCGTGGTG
WB235_4:16910542-16910677 Satlength=136 Nr of Repeats=9 RepeatLength=15 seed=TCGTGGTGAG Num.seqs=9 Similarity=0.980247
7 AGACCCATCGTGGTG
WB235_4:17140454-17141354 Satlength=901 Nr of Repeats=60 RepeatLength=15 seed=ACCCATCGTG Num.seqs=60 Similarity=0.889768
2 AGACCCATCGTGGTG
Rev.of_WB235_4:17147999-17148449 Satlength=451 Nr of Repeats=28 RepeatLength=15 seed=TCTCACCACG Num.seqs=25 Similarity=0.847111
3 AGACCCATCGTGGTG
Rev.of_WB235_5:1403881-1404196 Satlength=316 Nr of Repeats=21 RepeatLength=15 seed=CGATGGGTCT Num.seqs=21 Similarity=0.927196
10 AGACCCATCGTGGTG
Rev.of_WB235_5:2676568-2676778 Satlength=211 Nr of Repeats=14 RepeatLength=15 seed=CTCACCACGA Num.seqs=14 Similarity=0.840316
2 AGACCCATCGTGGTG
WB235_5:3621266-3621341 Satlength=76 Nr of Repeats=5 RepeatLength=15 seed=CGTGGTGAGA Num.seqs=5 Similarity=0.840000
8 AGACCCATCGTGGTG
Rev.of_WB235_5:5010548-5010653 Satlength=106 Nr of Repeats=7 RepeatLength=15 seed=GGGTCTCACC Num.seqs=7 Similarity=0.898413
6 AGACCCATCGTGGTG
WB235_5:6071658-6071958 Satlength=301 Nr of Repeats=20 RepeatLength=15 seed=ACCCATCGTG Num.seqs=20 Similarity=0.901287
2 AGACCCATCGTGGTG
WB235_5:6091617-6091992 Satlength=376 Nr of Repeats=25 RepeatLength=15 seed=CCCATCGTGG Num.seqs=25 Similarity=0.889185
3 AGACCCATCGTGGTG
Rev.of_WB235_5:6299893-6299998 Satlength=106 Nr of Repeats=7 RepeatLength=15 seed=GGGTCTCACC Num.seqs=7 Similarity=0.881481
6 AGACCCATCGTGGTG
WB235_5:6587809-6587899 Satlength=91 Nr of Repeats=6 RepeatLength=15 seed=GGTGAGACCC Num.seqs=6 Similarity=0.917037
11 AGACCCATCGTGGTG
Rev.of_WB235_5:7914059-7914224 Satlength=166 Nr of Repeats=11 RepeatLength=15 seed=CCACGATGGG Num.seqs=11 Similarity=0.877172
13 AGACCCATCGTGGTG
WB235_5:7915927-7916107 Satlength=181 Nr of Repeats=11 RepeatLength=15 seed=ACCCATCGTG Num.seqs=10 Similarity=0.907161
2 AGACCCATCGTGGTG
Rev.of_WB235_5:8316057-8316570 Satlength=514 Nr of Repeats=17 RepeatLength=15 seed=GGGTCTCACC Num.seqs=16 Similarity=0.932593
6 AGACCCATCGTGGTG
Rev.of_WB235_5:9439276-9439441 Satlength=166 Nr of Repeats=11 RepeatLength=15 seed=CGATGGGTCT Num.seqs=11 Similarity=0.899798
10 AGACCCATCGTGGTG
Rev.of_WB235_5:15295547-15296333 Satlength=787 Nr of Repeats=8 RepeatLength=15 seed=TCTCACCACG Num.seqs=7 Similarity=0.923810
3 AGACCCATCGTGGTG

Rev.of_WB235_5:15321484-15322280 Satlength=797 Nr of Repeats=53 RepeatLength=15 seed=CCACGATGGG Num.seqs=50 Similarity=0.875935
13   AGACCCATCGTGGTG
WB235_5:15582778-15582853 Satlength=76 Nr of Repeats=5 RepeatLength=15 seed=AGACCCATCG Num.seqs=5 Similarity=0.928889
0   AGACCCATCGTGGTG
Rev.of_WB235_6:161586-161691 Satlength=106 Nr of Repeats=7 RepeatLength=15 seed=GTCTCACCAC Num.seqs=7 Similarity=0.932275
4   AGACCCATCGTGGTG
Rev.of_WB235_6:169222-169627 Satlength=406 Nr of Repeats=27 RepeatLength=15 seed=ACGATGGGTC Num.seqs=27 Similarity=0.913897
11   AGACCCATCGTGGTG
Rev.of_WB235_6:943488-943966 Satlength=479 Nr of Repeats=11 RepeatLength=15 seed=CACGATGGGT Num.seqs=10 Similarity=0.887407
12   AGACCCATCGTGGTG
Rev.of_WB235_6:1376205-1376549 Satlength=345 Nr of Repeats=23 RepeatLength=15 seed=CGATGGGTCT Num.seqs=22 Similarity=0.845695
10   AGACCCATCGTGGTG
Rev.of_WB235_6:3075483-3075588 Satlength=106 Nr of Repeats=7 RepeatLength=15 seed=TCACCACGAT Num.seqs=7 Similarity=0.923810
1   AGACCCATCGTGGTG
WB235_6:5227815-5227935 Satlength=121 Nr of Repeats=8 RepeatLength=15 seed=TGGTGAGACC Num.seqs=8 Similarity=0.860317
10   AGACCCATCGTGGTG
Rev.of_WB235_6:15635841-15636081 Satlength=241 Nr of Repeats=15 RepeatLength=15 seed=GGGTCTCACC Num.seqs=14 Similarity=0.906227
6   AGACCCATCGTGGTG
Rev.of_WB235_6:16852072-16852162 Satlength=91 Nr of Repeats=6 RepeatLength=15 seed=GGGTCTCACC Num.seqs=6 Similarity=0.922963
6   AGACCCATCGTGGTG
Rev.of_WB235_2:15138506-15140272 Satlength=1767 Nr of Repeats=38 RepeatLength=15 seed=GGGTCTCACC Num.seqs=35 Similarity=0.915593
6   AGACCCNTCGTGGTG
WB235_5:16273937-16274402 Satlength=466 Nr of Repeats=31 RepeatLength=15 seed=TGGTGAGACC Num.seqs=31 Similarity=0.865806
10   AGACCCNTCGTGGTG
Rev.of_WB235_6:12521171-12522034 Satlength=864 Nr of Repeats=10 RepeatLength=15 seed=GGGTCTCACC Num.seqs=9 Similarity=0.920988
6   AGACCCNTCGTGGTG
WB235_1:6647571-6647736 Satlength=166 Nr of Repeats=11 RepeatLength=15 seed=GAGACCCATC Num.seqs=11 Similarity=0.883636
14   AGACCCATCGTGGCG
Rev.of_WB235_2:857683-857758 Satlength=76 Nr of Repeats=5 RepeatLength=15 seed=ATGGGTCTCG Num.seqs=3 Similarity=1.000000
23   AGACCCATCGTGGCG
WB235_2:2002012-2002087 Satlength=76 Nr of Repeats=5 RepeatLength=15 seed=TCGTGGCGAG Num.seqs=5 Similarity=0.928889
22   AGACCCATCGTGGCG
WB235_2:2386288-2386363 Satlength=76 Nr of Repeats=5 RepeatLength=15 seed=TCGTGGCGAG Num.seqs=5 Similarity=0.928889
22   AGACCCATCGTGGCG
WB235_2:4684299-4684389 Satlength=91 Nr of Repeats=6 RepeatLength=15 seed=GAGACCCATC Num.seqs=6 Similarity=0.899259
14   AGACCCATCGTGGCG

WB235_2:14991646-14991736 Satlength=91 Nr of Repeats=6 RepeatLength=15
seed=TCGTGGCGAG Num.seqs=6 Similarity=0.940741
22 AGACCCATCGTGGCG
WB235_2:15000612-15000786 Satlength=175 Nr of Repeats=9 RepeatLength=15
seed=AGACCCATCG Num.seqs=8 Similarity=0.895238
15 AGACCCATCGTGGCG
WB235_3:8335590-8336085 Satlength=496 Nr of Repeats=32 RepeatLength=15
seed=ACCCATCGTG Num.seqs=31 Similarity=0.914170
17 AGACCCATCGTGGCG
WB235_4:13846283-13847033 Satlength=751 Nr of Repeats=45 RepeatLength=15
seed=CCCATCGTGG Num.seqs=40 Similarity=0.901766
18 AGACCCATCGTGGCG
WB235_4:15166280-15166460 Satlength=181 Nr of Repeats=11 RepeatLength=15
seed=GAGACCCATC Num.seqs=10 Similarity=0.917037
14 AGACCCATCGTGGCG
Rev.of_WB235_6:9607653-9607728 Satlength=76 Nr of Repeats=5
RepeatLength=15 seed=ATGGGTCTCG Num.seqs=3 Similarity=1.000000
23 AGACCCATCGTGGCG
WB235_6:14362236-14362311 Satlength=76 Nr of Repeats=5 RepeatLength=15
seed=TCGTGGCGAG Num.seqs=5 Similarity=0.928889
22 AGACCCATCGTGGCG
WB235_6:17311002-17311107 Satlength=106 Nr of Repeats=7 RepeatLength=15
seed=GAGACCCATC Num.seqs=7 Similarity=0.847619
14 AGACCCATCGTGGCG
Rev.of_WB235_4:1345560-1345725 Satlength=166 Nr of Repeats=11
RepeatLength=15 seed=CCACGATGGG Num.seqs=11 Similarity=0.880404
28 AGACCCATCGTGGNG
Rev.of_WB235_4:13464499-13464709 Satlength=211 Nr of Repeats=14
RepeatLength=15 seed=CCACGATGGG Num.seqs=14 Similarity=0.902320
28 AGACCCATCGTGGNG
WB235_4:14476813-14476978 Satlength=166 Nr of Repeats=11 RepeatLength=15
seed=GACCCATCGT Num.seqs=11 Similarity=0.878788
16 AGACCCATCGTGGNG
Rev.of_WB235_6:516173-516819 Satlength=647 Nr of Repeats=15
RepeatLength=15 seed=CCACGATGGG Num.seqs=14 Similarity=0.873016
28 AGACCCATCGTGGNG
Rev.of_WB235_6:5229858-5230323 Satlength=466 Nr of Repeats=31
RepeatLength=15 seed=CCACGATGGG Num.seqs=27 Similarity=0.802469
28 AGACCCATCGTGGNG
Rev.of_WB235_1:14668547-14668607 Satlength=61 Nr of Repeats=4
RepeatLength=15 seed=CCACGATGGG Num.seqs=4 Similarity=0.881481
28 AGACCCATCGTGGAG
Rev.of_WB235_1:3389969-3393329 Satlength=3361 Nr of Repeats=224
RepeatLength=15 seed=CTCACCACGA Num.seqs=206 Similarity=0.875278
2 AGACCCTTCGTGGTG
WB235_1:3980324-3980429 Satlength=106 Nr of Repeats=7 RepeatLength=15
seed=TCGTGGTGAG Num.seqs=7 Similarity=0.949206
7 AGACCCTTCGTGGTG
WB235_1:5144853-5145677 Satlength=825 Nr of Repeats=51 RepeatLength=15
seed=CGTGGTGAGA Num.seqs=47 Similarity=0.827485
8 AGACCCTTCGTGGTG
WB235_1:7740676-7740751 Satlength=76 Nr of Repeats=5 RepeatLength=15
seed=TCGTGGTGAG Num.seqs=5 Similarity=0.911111
7 AGACCCTTCGTGGTG

WB235_2:1034465-1036445 Satlength=1981 Nr of Repeats=125 RepeatLength=15 seed=TCGTGGTGAG Num.seqs=119 Similarity=0.915200
7   AGACCCTTCGTGGTG
Rev.of_WB235_2:4260577-4261267 Satlength=691 Nr of Repeats=40 RepeatLength=15 seed=GTCTCACCAC Num.seqs=34 Similarity=0.828821
4   AGACCCTTCGTGGTG
WB235_2:9202102-9202507 Satlength=406 Nr of Repeats=27 RepeatLength=15 seed=TGGTGAGACC Num.seqs=25 Similarity=0.902519
10   AGACCCTTCGTGGTG
WB235_2:14993591-14993681 Satlength=91 Nr of Repeats=6 RepeatLength=15 seed=GGTGAGACCC Num.seqs=6 Similarity=0.905185
11   AGACCCTTCGTGGTG
WB235_3:1320777-1320837 Satlength=61 Nr of Repeats=4 RepeatLength=15 seed=TCGTGGTGAG Num.seqs=4 Similarity=0.955556
7   AGACCCTTCGTGGTG
WB235_3:3446010-3446749 Satlength=740 Nr of Repeats=47 RepeatLength=15 seed=GTGGTGAGAC Num.seqs=44 Similarity=0.837350
9   AGACCCTTCGTGGTG
Rev.of_WB235_4:1477450-1477555 Satlength=106 Nr of Repeats=7 RepeatLength=15 seed=CTCACCACGA Num.seqs=7 Similarity=0.873016
2   AGACCCTTCGTGGTG
Rev.of_WB235_4:14218012-14218147 Satlength=136 Nr of Repeats=9 RepeatLength=15 seed=GGGTCTCACC Num.seqs=9 Similarity=0.930864
6   AGACCCTTCGTGGTG
Rev.of_WB235_4:15060108-15060798 Satlength=691 Nr of Repeats=39 RepeatLength=15 seed=GGTCTCACCA Num.seqs=32 Similarity=0.917921
5   AGACCCTTCGTGGTG
Rev.of_WB235_4:16128108-16129641 Satlength=1534 Nr of Repeats=16 RepeatLength=15 seed=GGGTCTCACC Num.seqs=11 Similarity=0.935354
6   AGACCCTTCGTGGTG
WB235_4:16153344-16153449 Satlength=106 Nr of Repeats=7 RepeatLength=15 seed=GTGGTGAGAC Num.seqs=7 Similarity=0.898413
9   AGACCCTTCGTGGTG
Rev.of_WB235_4:16223761-16224157 Satlength=397 Nr of Repeats=25 RepeatLength=15 seed=TCTCACCACG Num.seqs=21 Similarity=0.820106
3   AGACCCTTCGTGGTG
WB235_5:1254384-1254459 Satlength=76 Nr of Repeats=5 RepeatLength=15 seed=TCGTGGTGAG Num.seqs=5 Similarity=0.911111
7   AGACCCTTCGTGGTG
WB235_5:1254384-1256245 Satlength=1862 Nr of Repeats=31 RepeatLength=15 seed=TCGTGGTGAG Num.seqs=29 Similarity=0.903667
7   AGACCCTTCGTGGTG
WB235_5:1254387-1257024 Satlength=2638 Nr of Repeats=44 RepeatLength=15 seed=TGGTGAGACC Num.seqs=41 Similarity=0.901138
10   AGACCCTTCGTGGTG
WB235_5:5005844-5006744 Satlength=901 Nr of Repeats=59 RepeatLength=15 seed=TCGTGGTGAG Num.seqs=50 Similarity=0.805823
7   AGACCCTTCGTGGTG
WB235_5:5640056-5640116 Satlength=61 Nr of Repeats=4 RepeatLength=15 seed=CGTGGTGAGA Num.seqs=4 Similarity=0.911111
8   AGACCCTTCGTGGTG
Rev.of_WB235_5:7720637-7720787 Satlength=151 Nr of Repeats=10 RepeatLength=15 seed=GGGTCTCACC Num.seqs=10 Similarity=0.934815
6   AGACCCTTCGTGGTG

WB235_5:9601934-9602249 Satlength=316 Nr of Repeats=21 RepeatLength=15
seed=GGTGAGACCC Num.seqs=21 Similarity=0.928889
11   AGACCCTTCGTGGTG
WB235_5:13882296-13882386 Satlength=91 Nr of Repeats=6 RepeatLength=15
seed=TCGTGGTGAG Num.seqs=6 Similarity=0.857778
7   AGACCCTTCGTGGTG
WB235_5:14124319-14124409 Satlength=91 Nr of Repeats=6 RepeatLength=15
seed=CGTGGTGAGA Num.seqs=6 Similarity=0.893333
8   AGACCCTTCGTGGTG
Rev.of_WB235_5:14156207-14156297 Satlength=91 Nr of Repeats=6
RepeatLength=15 seed=GGGTCTCACC Num.seqs=6 Similarity=0.917037
6   AGACCCTTCGTGGTG
WB235_6:13560726-13560906 Satlength=181 Nr of Repeats=11 RepeatLength=15
seed=TCGTGGTGAG Num.seqs=10 Similarity=0.857778
7   AGACCCTTCGTGGTG
Rev.of_WB235_6:15482468-15483998 Satlength=1531 Nr of Repeats=97
RepeatLength=15 seed=GTCTCACCAC Num.seqs=92 Similarity=0.888135
4   AGACCCTTCGTGGTG
Rev.of_WB235_6:16505118-16505433 Satlength=316 Nr of Repeats=18
RepeatLength=15 seed=TCACCACGAA Num.seqs=15 Similarity=0.947513
16   AGACCCTTCGTGGTG
WB235_6:14121147-14122257 Satlength=1111 Nr of Repeats=73 RepeatLength=15
seed=TCGTGGTGAG Num.seqs=70 Similarity=0.770484
7   AGACCTATCGTGGTG
WB235_1:308028-308148 Satlength=121 Nr of Repeats=7 RepeatLength=15
seed=GAGACCCATC Num.seqs=6 Similarity=0.764583
14   AGACCCATCGTGACG
WB235_3:690245-690736 Satlength=492 Nr of Repeats=19 RepeatLength=15
seed=TGAGACCCAT Num.seqs=13 Similarity=0.851852
28   AGACCCATCGTGATG

***** ***** *
Consensus:

AGACCCaTCGTGGTG

>WB235_Fam_4_35_115 Nr. of seq. 115 Alignment length(with gaps) = 45
Alignment score = 0.601883
WB235_1:1136040-1136228 Satlength=189 Nr of Repeats=5 RepeatLength=31
seed=ATTCCCGCCA Num.seqs=3 Similarity=0.628472
0   ATT--CCCGCC--AATT-----TT-TT-CT-CAGGAAATTTGA--
Rev.of_WB235_3:1320896-1321023 Satlength=128 Nr of Repeats=4
RepeatLength=31 seed=AATTCAAATT Num.seqs=3 Similarity=0.885305
3   ATT--CCCGCC--AAAA-----TT-TT-TT-GAAGAAATTTGA--
WB235_4:6664978-6665286 Satlength=309 Nr of Repeats=9 RepeatLength=31
seed=CCCGCCAAAA Num.seqs=7 Similarity=0.897593
3   ATT--CCCGCC--AAAA-----TT-TT-TT-GAAAAAATTTGA--
Rev.of_WB235_4:13478657-13478875 Satlength=219 Nr of Repeats=6
RepeatLength=31 seed=AATTCAAATT Num.seqs=4 Similarity=0.942652
3   ATT--CCCGCC--AAAA-----TT-TT-TT-GAGGAAATTTGA--
Rev.of_WB235_5:19052161-19052388 Satlength=228 Nr of Repeats=7
RepeatLength=33 seed=GGAATTCAAA Num.seqs=5 Similarity=0.967677
6   AT-T-CCCGCC--AAAATT---TT-TT-TT-GAATAAATTTGA--

Rev.of_WB235_3:12141791-12142267 Satlength=477 Nr of Repeats=10
RepeatLength=32 seed=ATTCAAATTT Num.seqs=6 Similarity=0.602694
34 ATT--TTCGCC--AAAA-N---TT-TT-CT-GAAGAAATTTGA--
WB235_4:17262649-17262776 Satlength=128 Nr of Repeats=4 RepeatLength=32
seed=GAAGAAATTT Num.seqs=3 Similarity=0.861111
52 ATT--TGCACC--AAAA-T---TT-TT-CT-GAAGAAATTTGA--
Rev.of_WB235_1:669658-671715 Satlength=2058 Nr of Repeats=63
RepeatLength=32 seed=GAATTCAAAT Num.seqs=41 Similarity=0.870732
4 ATT-C-CCGCC--AAAA-T---TT-TT-CT-CAGAAAATTTGA--
WB235_1:1362690-1362850 Satlength=161 Nr of Repeats=5 RepeatLength=32
seed=TCAGAAAATT Num.seqs=5 Similarity=0.983333
19 ATT-C-CCGCC--AAAA-T---TT-TT-CT-CAGAAAATTTGA--
WB235_4:15708521-15708971 Satlength=451 Nr of Repeats=14 RepeatLength=32
seed=TTGAATTCCC Num.seqs=13 Similarity=0.905983
28 ATT-C-CCGCC--AAAA-T---TT-TT-CA-CAGAAAATTTGA--
WB235_5:4053377-4053861 Satlength=485 Nr of Repeats=15 RepeatLength=32
seed=TTTCACAGAA Num.seqs=11 Similarity=0.921212
47 ATT-C-CCGCC--AAAA-T---TT-TT-CA-CAGAAAATTTGA--
WB235_6:14723633-14724049 Satlength=417 Nr of Repeats=13 RepeatLength=32
seed=TTGAATTCCC Num.seqs=13 Similarity=0.927885
28 ATT-C-CCGCC--AAAA-T---TT-TT-CA-CAGAAAATTTGA--
WB235_5:19993167-19993682 Satlength=516 Nr of Repeats=14 RepeatLength=32
seed=AAATTTGAAT Num.seqs=9 Similarity=0.806958
24 ATT-T-CCGCC--AAAA-T---TT-TT-CT-CAGAAAATTTGA--
WB235_1:13610087-13613799 Satlength=3713 Nr of Repeats=116
RepeatLength=32 seed=TGAATTCCCG Num.seqs=116 Similarity=0.922301
29 ATT-C-CCGCC--AAAA-T---TT-TT-CA-CAGAAATTTTGA--
Rev.of_WB235_2:4555047-4555687 Satlength=641 Nr of Repeats=20
RepeatLength=32 seed=CGGGAATTCA Num.seqs=20 Similarity=0.921491
39 ATT-C-CCGCC--AAAA-T---TT-TT-CA-CAGAAATTTTGA--
Rev.of_WB235_3:11160967-11161287 Satlength=321 Nr of Repeats=10
RepeatLength=32 seed=TTTCTGTGAA Num.seqs=10 Similarity=0.896296
58 ATT-C-CCGCC--AAAA-T---TT-TT-CA-CAGAAATTTTGA--
WB235_4:8467114-8467274 Satlength=161 Nr of Repeats=5 RepeatLength=32
seed=TTGAATTCCC Num.seqs=5 Similarity=0.891667
60 ATT-C-CCGCC--AAAA-T---TT-TT-CA-CAGAAATTTTGA--
Rev.of_WB235_4:12023094-12025460 Satlength=2367 Nr of Repeats=74
RepeatLength=32 seed=CGGGAATTCA Num.seqs=72 Similarity=0.926774
39 ATT-C-CCGCC--AAAA-T---TT-TT-CA-CAGAAATTTTGA--
Rev.of_WB235_5:3520923-3521917 Satlength=995 Nr of Repeats=31
RepeatLength=32 seed=CGGGAATTCA Num.seqs=29 Similarity=0.920874
39 ATT-C-CCGCC--AAAA-T---TT-TT-CA-CAGAAATTTTGA--
Rev.of_WB235_6:9008356-9008996 Satlength=641 Nr of Repeats=20
RepeatLength=32 seed=CGGGAATTCA Num.seqs=20 Similarity=0.894298
39 ATT-C-CCGCC--AAAA-T---TT-TT-CA-CAGAAATTTTGA--
WB235_6:14723638-14724190 Satlength=553 Nr of Repeats=16 RepeatLength=32
seed=TTCCCGCCAA Num.seqs=11 Similarity=0.947727
33 ATT-C-CCGCC--AAAA-T---TT-TT-CA-CAGAAATTTTGA--
Rev.of_WB235_5:6984815-6985177 Satlength=363 Nr of Repeats=11
RepeatLength=32 seed=AATTCAAATT Num.seqs=7 Similarity=0.908730
35 ATT-C-CCGCC--AAAA-T---TT-TT-CA-CAAAAAATTTGA--
WB235_2:1968841-1969602 Satlength=762 Nr of Repeats=23 RepeatLength=32
seed=AATTCCCGCC Num.seqs=16 Similarity=0.855208
31 ATT-C-CCGCC--AAAA-A---TT-TT-CA-NAGAAAATTTGA--

WB235_2:1976612-1976897 Satlength=286 Nr of Repeats=8 RepeatLength=32
seed=AATTCCCGCC Num.seqs=5 Similarity=0.858333
31   ATT-C-CCGCC--AAAA-A---TT-TT-CA-NAGAAAATTTGA--
WB235_2:1985022-1985847 Satlength=826 Nr of Repeats=25 RepeatLength=32
seed=AATTCCCGCC Num.seqs=18 Similarity=0.860566
31   ATT-C-CCGCC--AAAA-A---TT-TT-CA-NAGAAAATTTGA--
Rev.of_WB235_2:2002110-2002460 Satlength=351 Nr of Repeats=10
RepeatLength=32 seed=GGCGGGAATT Num.seqs=7 Similarity=0.843675
41   ATT-C-CCGCC--AAAA-A---TT-TT-CA-AAGAAAATTTGA--
WB235_6:9605306-9607639 Satlength=2334 Nr of Repeats=65 RepeatLength=32
seed=TCCCGCCAAA Num.seqs=47 Similarity=0.846978
66   ATT-C-CCGCC--AAAA-A---TT-TT-CA-AAGAAAATTTGA--
Rev.of_WB235_2:14991755-14991977 Satlength=223 Nr of Repeats=7
RepeatLength=32 seed=TTTTGGCGGG Num.seqs=5 Similarity=0.841667
45   ATT-C-CCGCC--AAAA-A---TT-TT-CA-NAGAAAATTTGA--
WB235_2:6434384-6434544 Satlength=161 Nr of Repeats=5 RepeatLength=32
seed=AAAAATTTTC Num.seqs=5 Similarity=0.916667
41   ATT-C-CCGCC--AAAA-A---TT-TT-CA-CAGAAAATTTGA--
WB235_2:6434384-6435137 Satlength=754 Nr of Repeats=6 RepeatLength=32
seed=AAAAATTTTC Num.seqs=5 Similarity=0.916667
41   ATT-C-CCGCC--AAAA-A---TT-TT-CA-CAGAAAATTTGA--
WB235_5:20471611-20471894 Satlength=284 Nr of Repeats=9 RepeatLength=32
seed=CCGCCAAAAT Num.seqs=7 Similarity=0.853175
4   ATT-C-CCGCC--AAAA-T---TT-TT-CT-TTGAAAATTTGA--
WB235_1:994259-994677 Satlength=419 Nr of Repeats=4 RepeatLength=32
seed=CAAAATTTTT Num.seqs=3 Similarity=0.888889
9   A-T-CGCCGCC--AAAA-T---TT-TT-CT-CAGAAAATTTGA--
Rev.of_WB235_5:18473949-18474108 Satlength=160 Nr of Repeats=4
RepeatLength=32 seed=GAATTCAAAT Num.seqs=3 Similarity=0.888889
4   ATT-C-ACGCC--AAAA-A---TT-TT-TT-CAGAAAATTTGA--
WB235_2:2599830-2600053 Satlength=224 Nr of Repeats=7 RepeatLength=32
seed=AATTCCCGCC Num.seqs=6 Similarity=0.855556
31   ATT-C-CCGCC--AAAA-A---AT-TT-CA-AAGAAAATTTAA--
WB235_2:2599867-2600422 Satlength=556 Nr of Repeats=7 RepeatLength=32
seed=CCGCCAAAAA Num.seqs=5 Similarity=0.866667
36   ATT-C-CCGCC--AAAA-A---AT-TT-CA-AAGAAAATTTAA--
WB235_2:13906504-13906669 Satlength=166 Nr of Repeats=5 RepeatLength=33
seed=AAAATTTGAA Num.seqs=5 Similarity=0.749495
24   ATT-TCCCGCC--AAAA-T--T-T-TT-CT-CAGAAAATTTGA--
Rev.of_WB235_5:1368467-1368669 Satlength=203 Nr of Repeats=6
RepeatLength=34 seed=AAATTCAAAT Num.seqs=5 Similarity=0.823529
37   ATT-TCCCGCC--AAAA-T--TAT-TT-CT-CAGAAAATTTGA--
WB235_1:759087-759538 Satlength=452 Nr of Repeats=13 RepeatLength=34
seed=TTTCTCAGAA Num.seqs=8 Similarity=0.787245
50   ATT-TCCCACC--AAAA-T--TTT-TT-CT-CAGAAAATTTGA--
WB235_5:2716232-2716355 Satlength=124 Nr of Repeats=4 RepeatLength=35
seed=TTTCTCAGAA Num.seqs=3 Similarity=0.923810
51   ATT-TCCCGCC--AAAA-T-GTTT-TT-CT-CAGAAAATTTGA--
WB235_1:14257569-14258742 Satlength=1174 Nr of Repeats=31 RepeatLength=34
seed=AAATTTGAAT Num.seqs=19 Similarity=0.788556
25   ATT-TCCCGCC--AAAA-A-GT-T-TT-CT-CAGAAAATTTGA--
WB235_3:12480633-12480804 Satlength=172 Nr of Repeats=5 RepeatLength=35
seed=AATTTGAATT Num.seqs=3 Similarity=0.923810
26   ATT-TCCCGCC--AAAA-TCAT-T-TT-CT-GAGAAAATTTGA--

WB235_2:1793298-1793504 Satlength=207 Nr of Repeats=6 RepeatLength=34
seed=ATTTGAATTT Num.seqs=4 Similarity=0.980392
27   ATT-TCCCGCC--AAAA---TTTT-TT-CA-CTGAAAATTTGA--
Rev.of_WB235_2:1796656-1797032 Satlength=377 Nr of Repeats=11
RepeatLength=34 seed=CGGGAAATTC Num.seqs=9 Similarity=0.956427
41   ATT-TCCCGCC--AAAA---TTTT-TT-CA-CTGAAAATTTGA--
Rev.of_WB235_2:14993697-14994076 Satlength=380 Nr of Repeats=11
RepeatLength=34 seed=TTGGCGGGAA Num.seqs=7 Similarity=0.880511
45   ATT-TCCCGCC--AAAA---TTTT-TT-CA-CTGAAAATTTGA--
WB235_4:1344791-1345475 Satlength=685 Nr of Repeats=20 RepeatLength=34
seed=ATTTCCCGCC Num.seqs=16 Similarity=0.995098
33   ATT-TCCCGCC--AAAA---TTTT-TT-CA-CTGAAAATTTGA--
WB235_4:1477245-1477418 Satlength=174 Nr of Repeats=5 RepeatLength=34
seed=GAAAATTTGA Num.seqs=3 Similarity=0.828571
57   ATT-TCCCGCC--AAAA---TTTT-TT-CA-CTGAAAATTTGA--
Rev.of_WB235_4:15188545-15190957 Satlength=2413 Nr of Repeats=70
RepeatLength=34 seed=TTGGCGGGAA Num.seqs=46 Similarity=0.965786
45   ATT-TCCCGCC--AAAA---TTTT-TT-CA-CTGAAAATTTGA--
Rev.of_WB235_5:10268112-10269248 Satlength=1137 Nr of Repeats=33
RepeatLength=34 seed=TTGGCGGGAA Num.seqs=20 Similarity=0.915765
45   ATT-TCCCGCC--AAAA---TTTT-TT-CA-CTGAAAATTTGA--
WB235_1:1429375-1430301 Satlength=927 Nr of Repeats=24 RepeatLength=35
seed=AAATTTGAAT Num.seqs=15 Similarity=0.827628
60   ATT-TCCCGCC--AAAA-T-TTTT-TT-CA-CTGAAAATTTGA--
Rev.of_WB235_1:7740774-7741690 Satlength=917 Nr of Repeats=26
RepeatLength=35 seed=GCGGGAAATT Num.seqs=17 Similarity=0.870370
77   ATT-TCCCGCC--AAAA-T-TTTT-TT-CA-CTGAAAATTTGA--
Rev.of_WB235_1:11480313-11481528 Satlength=1216 Nr of Repeats=24
RepeatLength=35 seed=TGGCGGGAAA Num.seqs=17 Similarity=0.860771
79   ATT-TCCCGCC--AAAA-T-TTTT-TT-CA-CTGAAAATTTGA--
Rev.of_WB235_2:9202534-9203060 Satlength=527 Nr of Repeats=15
RepeatLength=35 seed=ATTCAAATTT Num.seqs=10 Similarity=0.900952
70   ATT-TCCCGCC--AAAA-T-TTTT-TT-CA-CTGAAAATTTGA--
Rev.of_WB235_2:9202525-9203501 Satlength=977 Nr of Repeats=16
RepeatLength=35 seed=TGGCGGGAAA Num.seqs=10 Similarity=0.909418
79   ATT-TCCCGCC--AAAA-T-TTTT-TT-CA-CTGAAAATTTGA--
Rev.of_WB235_4:1476030-1477123 Satlength=1094 Nr of Repeats=29
RepeatLength=35 seed=AAAAAATTTT Num.seqs=18 Similarity=0.995767
88   ATT-TCCCGCC--AAAA-T-TTTT-TT-CA-CTGAAAATTTGA--
Rev.of_WB235_4:10263336-10264889 Satlength=1554 Nr of Repeats=42
RepeatLength=35 seed=AATTCAAATT Num.seqs=30 Similarity=0.878991
71   ATT-TCCCGCC--AAAA-T-TTTT-TT-CA-CTGAAAATTTGA--
WB235_4:12155676-12156286 Satlength=611 Nr of Repeats=8 RepeatLength=35
seed=AAATTTGAAT Num.seqs=5 Similarity=0.847619
60   ATT-TCCCGCC--AAAA-T-TTTT-TT-CA-CTGAAAATTTGA--
WB235_4:14740489-14741012 Satlength=524 Nr of Repeats=15 RepeatLength=35
seed=AAATTTGAAT Num.seqs=9 Similarity=0.935450
60   ATT-TCCCGCC--AAAA-T-TTTT-TT-CA-CTGAAAATTTGA--
Rev.of_WB235_4:15320096-15321040 Satlength=945 Nr of Repeats=27
RepeatLength=35 seed=GAAATTCAAA Num.seqs=18 Similarity=0.936508
73   ATT-TCCCGCC--AAAA-T-TTTT-TT-CA-CTGAAAATTTGA--
WB235_4:15971109-15972271 Satlength=1163 Nr of Repeats=29 RepeatLength=35
seed=TTCCCGCCAA Num.seqs=20 Similarity=0.886717
70   ATT-TCCCGCC--AAAA-T-TTTT-TT-CA-CTGAAAATTTGA--

Rev.of_WB235_5:1254505-1257773 Satlength=3269 Nr of Repeats=35 RepeatLength=35 seed=GCGGGAAATT Num.seqs=21 Similarity=0.865809
77 ATT-TCCCGCC--AAAA-T-TTTT-TT-CA-CTGAAAATTTGA--
WB235_5:5009264-5010522 Satlength=1259 Nr of Repeats=36 RepeatLength=35 seed=AAATTTGAAT Num.seqs=26 Similarity=0.926974
60 ATT-TCCCGCC--AAAA-T-TTTT-TT-CA-CTGAAAATTTGA--
WB235_5:6296625-6296906 Satlength=282 Nr of Repeats=8 RepeatLength=35 seed=AATTTCCCGC Num.seqs=7 Similarity=0.909297
67 ATT-TCCCGCC--AAAA-T-TTTT-TT-CA-CTGAAAATTTGA--
WB235_5:6297848-6298284 Satlength=437 Nr of Repeats=5 RepeatLength=35 seed=TTTCCCGCCA Num.seqs=3 Similarity=0.916667
69 ATT-TCCCGCC--AAAA-T-TTTT-TT-CA-CTGAAAATTTGA--
Rev.of_WB235_5:9602338-9603453 Satlength=1116 Nr of Repeats=32 RepeatLength=35 seed=GCGGGAAATT Num.seqs=21 Similarity=0.936327
77 ATT-TCCCGCC--AAAA-T-TTTT-TT-CA-CTGAAAATTTGA--
Rev.of_WB235_5:13882409-13882690 Satlength=282 Nr of Repeats=8 RepeatLength=35 seed=GCGGGAAATT Num.seqs=5 Similarity=0.946667
77 ATT-TCCCGCC--AAAA-T-TTTT-TT-CA-CTGAAAATTTGA--
Rev.of_WB235_5:14124469-14125286 Satlength=818 Nr of Repeats=21 RepeatLength=35 seed=GGAAATTCAA Num.seqs=14 Similarity=0.914181
74 ATT-TCCCGCC--AAAA-T-TTTT-TT-CA-CTGAAAATTTGA--
Rev.of_WB235_5:15582889-15584265 Satlength=1377 Nr of Repeats=36 RepeatLength=35 seed=TTCAAATTTT Num.seqs=30 Similarity=0.828501
69 ATT-TCCCGCC--AAAA-T-TTTT-TT-CA-CTGAAAATTTGA--
WB235_6:9188817-9189060 Satlength=244 Nr of Repeats=7 RepeatLength=35 seed=AAAATTTGAA Num.seqs=5 Similarity=0.908571
94 ATT-TCCCGCC--AAAA-T-TTTT-TT-CA-CTGAAAATTTGA--
WB235_3:10678429-10678847 Satlength=419 Nr of Repeats=11 RepeatLength=35 seed=CCGCCAAAAT Num.seqs=10 Similarity=0.943280
38 ATT-TCCCGCC--AAAA-T-ATTT-TT-CA-CTAAAAATTTGA--
Rev.of_WB235_3:10683224-10684272 Satlength=1049 Nr of Repeats=30 RepeatLength=35 seed=AAATTCAAAT Num.seqs=28 Similarity=0.892702
72 ATT-TCCCGCC--AAAA-T-ATTT-TT-CA-CTAAAAATTTGA--
Rev.of_WB235_4:15201340-15201723 Satlength=384 Nr of Repeats=11 RepeatLength=35 seed=GGGAAATTCA Num.seqs=7 Similarity=0.941950
40 ATT-TCCCGCC--AAAA-T-ATTT-TT-TA-CTAAAAATTTGA--
Rev.of_WB235_4:15201392-15202170 Satlength=779 Nr of Repeats=9 RepeatLength=35 seed=GTAAAAAATA Num.seqs=7 Similarity=0.929252
57 ATT-TCCCGCC--AAAA-T-ATTT-TT-TA-CTAAAAATTTGA--
Rev.of_WB235_1:7868653-7868828 Satlength=176 Nr of Repeats=5 RepeatLength=35 seed=GGGAAATTTA Num.seqs=5 Similarity=0.946667
40 ATT-TCCCGCC--AAAA-T-ATTT-TT-CA-CAGAAAATTTAA--
WB235_2:14878167-14878554 Satlength=388 Nr of Repeats=11 RepeatLength=35 seed=CAGAAAATTT Num.seqs=9 Similarity=0.949206
56 ATT-TCCCGCC--AAAA-T-ATTT-TT-CA-CAGAAAATTTAA--
Rev.of_WB235_4:13462892-13463563 Satlength=672 Nr of Repeats=18 RepeatLength=35 seed=GAAATTTAAA Num.seqs=15 Similarity=0.948481
73 ATT-TCCCGCC--AAAA-T-ATTT-TT-CA-CAGAAAATTTAA--
Rev.of_WB235_4:13462893-13464435 Satlength=1543 Nr of Repeats=25 RepeatLength=35 seed=AAATTTAAAT Num.seqs=19 Similarity=0.791694
72 ATT-TCCCGCC--AAAA-T-ATTT-TT-CA-CAGAAAATTTAA--
Rev.of_WB235_4:13922549-13923902 Satlength=1354 Nr of Repeats=39 RepeatLength=35 seed=GGGAAATTTA Num.seqs=27 Similarity=0.939655
40 ATT-TCCCGCC--AAAA-T-ATTT-TT-CA-CAGAAAATTTAA--

Rev.of_WB235_4:15166547-15166687 Satlength=141 Nr of Repeats=4 RepeatLength=35 seed=AAATTTTCTG Num.seqs=4 Similarity=0.904762
66 ATT-TCCCGCC--AAAA-T-ATTT-TT-CA-CAGAAAATTTAA--
Rev.of_WB235_4:16910719-16910928 Satlength=210 Nr of Repeats=6 RepeatLength=35 seed=GAAATTTAAA Num.seqs=5 Similarity=0.900952
73 ATT-TCCCGCC--AAAA-T-ATTT-TT-CA-CAGAAAATTTAA--
Rev.of_WB235_4:17141438-17141648 Satlength=211 Nr of Repeats=6 RepeatLength=35 seed=AAATTTTCTG Num.seqs=6 Similarity=0.928889
66 ATT-TCCCGCC--AAAA-T-ATTT-TT-CA-CAGAAAATTTAA--
Rev.of_WB235_5:6071995-6072240 Satlength=246 Nr of Repeats=7 RepeatLength=35 seed=ATTTTGGCGG Num.seqs=7 Similarity=0.952834
48 ATT-TCCCGCC--AAAA-T-ATTT-TT-CA-CAGAAAATTTAA--
WB235_5:7913711-7913991 Satlength=281 Nr of Repeats=8 RepeatLength=35 seed=CAGAAAATTT Num.seqs=8 Similarity=0.964626
56 ATT-TCCCGCC--AAAA-T-ATTT-TT-CA-CAGAAAATTTAA--
Rev.of_WB235_5:7916137-7916840 Satlength=704 Nr of Repeats=20 RepeatLength=35 seed=GGGAAATTTA Num.seqs=17 Similarity=0.971429
40 ATT-TCCCGCC--AAAA-T-ATTT-TT-CA-CAGAAAATTTAA--
WB235_6:1375505-1376170 Satlength=666 Nr of Repeats=19 RepeatLength=35 seed=CCCGCCAAAA Num.seqs=19 Similarity=0.932275
72 ATT-TCCCGCC--AAAA-T-ATTT-TT-CA-CAGAAAATTTAA--
WB235_6:13795780-13795990 Satlength=211 Nr of Repeats=6 RepeatLength=35 seed=CAGAAAATTT Num.seqs=6 Similarity=0.949206
56 ATT-TCCCGCC--AAAA-T-ATTT-TT-CA-CAGAAAATTTAA--
WB235_6:15482252-15482427 Satlength=176 Nr of Repeats=5 RepeatLength=35 seed=ATTTAAATTT Num.seqs=5 Similarity=0.939048
62 ATT-TCCCGCC--AAAA-T-ATTT-TT-CA-CAGAAAATTTAA--
Rev.of_WB235_6:17311164-17311479 Satlength=316 Nr of Repeats=9 RepeatLength=35 seed=AAATTTTCTG Num.seqs=7 Similarity=0.909297
66 ATT-TCCCGCC--AAAA-T-ATTT-TT-CA-CAGAAAATTTAA--
Rev.of_WB235_4:15826993-15827272 Satlength=280 Nr of Repeats=8 RepeatLength=35 seed=AATTTAAATT Num.seqs=7 Similarity=0.896599
71 ATT-TCCCGCC--AAAA-T-ATTT-TT-CN-CAGAAAATTTAA--
WB235_2:632106-632999 Satlength=894 Nr of Repeats=4 RepeatLength=35 seed=GAAAATTTAA Num.seqs=3 Similarity=0.923810
58 ATT-TCCCTCC--AAAA-T-ATTT-TT-CA-CAGAAAATTTAA--
Rev.of_WB235_2:14836894-14837772 Satlength=879 Nr of Repeats=25 RepeatLength=35 seed=TTAAATTTTC Num.seqs=22 Similarity=0.939312
68 ATT-TCCCTCC--AAAA-T-ATTT-TT-CA-CAGAAAATTTAA--
Rev.of_WB235_3:8336113-8336287 Satlength=175 Nr of Repeats=5 RepeatLength=35 seed=GGGAAATTTA Num.seqs=4 Similarity=0.974603
75 ATT-TCCCTCC--AAAA-T-ATTT-TT-CA-CAGAAAATTTAA--
Rev.of_WB235_1:14403308-14403517 Satlength=210 Nr of Repeats=6 RepeatLength=35 seed=AATTTAAATT Num.seqs=5 Similarity=0.859048
71 ATT-TCCCGCC--AAAA-T-ATTT-TT-CT-CAGAAAATTTAA--
WB235_3:13447979-13448227 Satlength=249 Nr of Repeats=7 RepeatLength=35 seed=AATTTCCCGC Num.seqs=5 Similarity=0.885714
102 ATT-TCCCGCC--AAAA-T-ATTT-TT-CT-CAGAAAATTTAA--
WB235_4:7441316-7441526 Satlength=211 Nr of Repeats=6 RepeatLength=35 seed=CAGAAAATTT Num.seqs=6 Similarity=0.926349
91 ATT-TCCCGCC--AAAA-T-ATTT-TT-CT-CAGAAAATTTAA--
WB235_5:9439011-9439221 Satlength=211 Nr of Repeats=6 RepeatLength=35 seed=AGAAAATTTA Num.seqs=6 Similarity=0.888254
92 ATT-TCCCGCC--AAAA-T-ATTT-TT-CT-CAGAAAATTTAA--

WB235_5:18777092-18777231 Satlength=140 Nr of Repeats=4 RepeatLength=35
seed=TCAGAAAATT Num.seqs=3 Similarity=0.923810
90   ATT-TCCCGCC--AAAA-T-ATTT-TT-CT-CAGAAAATTTAA--
WB235_6:14937379-14937623 Satlength=245 Nr of Repeats=7 RepeatLength=35
seed=CAGAAAATTT Num.seqs=6 Similarity=0.893333
91   ATT-TCCCGCC--AAAA-T-ATTT-TT-CT-CAGAAAATTTAA--
WB235_4:8469359-8469569 Satlength=211 Nr of Repeats=6 RepeatLength=35
seed=GAAAATTTAA Num.seqs=6 Similarity=0.898413
93   ATT-TCCCTCC--AAAA-T-ATTT-TT-CT-CAGAAAATTTAA--
WB235_6:15635446-15635796 Satlength=351 Nr of Repeats=10 RepeatLength=35
seed=CAGAAAATTT Num.seqs=10 Similarity=0.875556
126   ATT-TCCCTCC--AAAA-T-ATTT-TT-CT-CAGAAAATTTAA--
WB235_4:15765291-15766235 Satlength=945 Nr of Repeats=8 RepeatLength=35
seed=AAAATTTAAA Num.seqs=7 Similarity=0.883900
94   ATT-TCCCNCC--AAAA-T-ATTT-TT-CT-CAGAAAATTTAA--
Rev.of_WB235_2:15000726-15000995 Satlength=270 Nr of Repeats=5
RepeatLength=35 seed=ATTTTGGCGG Num.seqs=4 Similarity=0.904762
83   ATT-TCCCGCC--AAAA-T-ATTT-TT-CT-CCGAAAATTTAA--
WB235_4:16675832-16676039 Satlength=208 Nr of Repeats=6 RepeatLength=35
seed=TTGAATTTCC Num.seqs=4 Similarity=0.917460
64   ATT-TCCCGCC--AAAA-T-ATTT-TT-CA-CAGAAAATTTGA--
Rev.of_WB235_6:142149-142814 Satlength=666 Nr of Repeats=19
RepeatLength=35 seed=GCGGGAAATT Num.seqs=15 Similarity=0.805405
77   ATT-TCCCGCC--AAAA-T-ATTT-TT-CA-CAAAAAATTTGA--
WB235_2:9633206-9633906 Satlength=701 Nr of Repeats=20 RepeatLength=35
seed=CCAAAATATT Num.seqs=20 Similarity=0.928822
41   ATT-TCCCTCC--AAAA-T-ATTT-TT-TT-CAGAAAATTTAA--
WB235_4:1153661-1154193 Satlength=533 Nr of Repeats=4 RepeatLength=35
seed=AAAATATTTT Num.seqs=3 Similarity=0.898413
43   ATT-TCCCTCC--AAAA-T-ATTT-TT-TT-CAGAAAATTTAA--
Rev.of_WB235_3:3896794-3897038 Satlength=245 Nr of Repeats=7
RepeatLength=35 seed=AATTTAAATT Num.seqs=6 Similarity=0.890794
71   ATT-TCCCGCC--AAAA-T-ATTT-TT-TT-CAGAAAATTTAA--
WB235_4:4920980-4921503 Satlength=524 Nr of Repeats=15 RepeatLength=35
seed=TTTTCAGAAA Num.seqs=13 Similarity=0.973626
52   ATT-TCCCTCC--AAAA-T-ATTT-TT-TT-CAGAAAATTAAA--
WB235_4:4923556-4923766 Satlength=211 Nr of Repeats=6 RepeatLength=35
seed=AATATTTTTT Num.seqs=6 Similarity=0.964444
80   ATT-TCCCTCC--AAAA-T-ATTT-TT-TT-CAGAAAATTAAA--
WB235_4:4925825-4926035 Satlength=211 Nr of Repeats=6 RepeatLength=35
seed=AATATTTTTT Num.seqs=6 Similarity=0.939048
80   ATT-TCCCTCC--AAAA-T-ATTT-TT-TT-CAGAAAATTAAA--
Rev.of_WB235_3:12485043-12485356 Satlength=314 Nr of Repeats=8
RepeatLength=35 seed=TTCAAATTTT Num.seqs=5 Similarity=0.658559
34   ATT-TNCCGCC--AAAA-T-GTCT-TT-CA-CAGAAAATTTGA--
WB235_5:19866564-19867301 Satlength=738 Nr of Repeats=21 RepeatLength=33
seed=TTTGAATTTC Num.seqs=17 Similarity=0.722086
26   -TT-TCCCACC--AAAA-T---TT-TC--T-CAAAAAATTTGAA-
WB235_5:19755742-19756252 Satlength=511 Nr of Repeats=14 RepeatLength=34
seed=TTTCCCGCCA Num.seqs=12 Similarity=0.859774
32   -TT-TCCCGCC--AAAA-T---AT-TCACT-CNAAAAATTTGAA-
Rev.of_WB235_3:12762055-12762649 Satlength=595 Nr of Repeats=17
RepeatLength=35 seed=AAATTCAAAT Num.seqs=16 Similarity=0.851746
35   -TT-TCCCGCCATAAAA-T---AT-TT-CT-CAAATAATTTGAA-

Rev.of_WB235_3:12345927-12348234 Satlength=2308 Nr of Repeats=63 RepeatLength=35 seed=CGGGAAAATT Num.seqs=39 Similarity=0.947253
40 -TT-TCCCGCC--AAAA-T---TTGTT--TCCAAAAAATTTGAAT
Rev.of_WB235_5:17817728-17818258 Satlength=531 Nr of Repeats=13 RepeatLength=34 seed=TCAAATTTTC Num.seqs=8 Similarity=0.860204
0 ATT-T-CCGCC--AAAG-T-TTTT-TT-AA-AAGAAAATTTGA--

* ** ** * * * ** *
Consensus:

ATTtcCCGCCAAAAtntTTTTCaCaGAAAATTTgA

>WB235_Fam_5_40_94 Nr. of seq. 94 Alignment length(with gaps) = 41 Alignment score = 0.668164
WB235_1:334679-335159 Satlength=481 Nr of Repeats=12 RepeatLength=40 seed=AATTTTCTCG Num.seqs=12 Similarity=0.914646
0 AATTTTCTCGAAATTTCCAGAAGGTTCTAGAACATTTCA-G
Rev.of_WB235_5:1352314-1352754 Satlength=441 Nr of Repeats=9 RepeatLength=40 seed=AATGTTCTAG Num.seqs=6 Similarity=0.738753
36 AATTTTCTCGAATTTTCCAGAAGGTTCTAGAACATTTCA-G
WB235_1:14668659-14668849 Satlength=191 Nr of Repeats=5 RepeatLength=40 seed=CCAGAAGGTT Num.seqs=4 Similarity=0.800000
16 AATTTTCTCGAATTTTCCAGAAGGTTCTAGAACAATCCA-G
WB235_1:13613895-13615295 Satlength=1401 Nr of Repeats=35 RepeatLength=40 seed=GAATTTTCTC Num.seqs=35 Similarity=0.957535
39 AATTTTCTCGAATTTTCCAGAAGGTTCTGGAGCATTCCA-G
Rev.of_WB235_6:16100718-16101792 Satlength=1075 Nr of Repeats=13 RepeatLength=40 seed=ATGTTCCAGA Num.seqs=12 Similarity=0.873676
75 AATTTTCTCGAATTTTCCAGAAGGTTCTGGAACATTCCA-G
WB235_4:2135958-2136208 Satlength=251 Nr of Repeats=6 RepeatLength=40 seed=TTCCAGAATT Num.seqs=5 Similarity=0.866667
74 AATTTTCTCGAATTTTCCAGAAGGTTCTAGAACATTCCA-G
Rev.of_WB235_4:13668023-13668625 Satlength=603 Nr of Repeats=12 RepeatLength=40 seed=AAAATTCTGG Num.seqs=9 Similarity=0.870370
86 AATTTTCTCGAATTTTCCAGAAGGTTCTAGAACATTCCA-G
Rev.of_WB235_6:443181-444059 Satlength=879 Nr of Repeats=19 RepeatLength=40 seed=AAAATTCTGG Num.seqs=12 Similarity=0.943434
86 AATTTTCTCGAATTTTCCAGAAGGTTCTAGAACATTCCA-G
Rev.of_WB235_4:13861930-13862600 Satlength=671 Nr of Repeats=11 RepeatLength=40 seed=CTGGAATGTT Num.seqs=9 Similarity=0.940741
40 AATTTTCTCAAATTTTCCAGAAGGTTCTGGAACATTCCA-G
Rev.of_WB235_5:16547704-16548504 Satlength=801 Nr of Repeats=18 RepeatLength=40 seed=CTGGAATGTT Num.seqs=16 Similarity=0.952222
40 AATTTTCTCAAATTTTCCAGAAGGTTCTGGAACATTCCA-G
Rev.of_WB235_5:16547704-16548822 Satlength=1119 Nr of Repeats=19 RepeatLength=40 seed=CTGGAATGTT Num.seqs=16 Similarity=0.952222
40 AATTTTCTCAAATTTTCCAGAAGGTTCTGGAACATTCCA-G
Rev.of_WB235_5:17043954-17044434 Satlength=481 Nr of Repeats=12 RepeatLength=40 seed=AAAATTCTGG Num.seqs=12 Similarity=0.900000
46 AATTTTCTCAAATTTTCCAAAAGGCTCTAGAACATTCCA-G
WB235_6:17288074-17288675 Satlength=602 Nr of Repeats=6 RepeatLength=40 seed=TTTCTCGAAT Num.seqs=4 Similarity=0.866667
43 AAATTTCTCGAATCTTCCAGAAGGTTCTAGAACATTCCA-G

WB235_1:4573284-4573803 Satlength=520 Nr of Repeats=13 RepeatLength=40
seed=CATTCCAGAA Num.seqs=12 Similarity=0.966667
22 ATTTTTCTAGAAAGTTCTGGAACATTCCAGAATTTTCCC-G
WB235_3:2501737-2501917 Satlength=181 Nr of Repeats=5 RepeatLength=40
seed=TTCCAGAATT Num.seqs=4 Similarity=0.966667
24 ATTTTTCTAGAAAGTTCTGGAACATTCCAGAATTTTCCC-G
WB235_4:15765625-15765904 Satlength=280 Nr of Repeats=6 RepeatLength=40
seed=GATTTTTCTA Num.seqs=4 Similarity=0.911111
39 ATTTTTCTAGAAAGTTCTGGAACATTCCAGAATTTTCCC-G
Rev.of_WB235_5:6088840-6089240 Satlength=401 Nr of Repeats=10
RepeatLength=40 seed=TGGAATGTTC Num.seqs=10 Similarity=0.916622
29 ATTTTTCTAGAAAGTTCTGGAACATTCCAGAATTTTCCC-G
Rev.of_WB235_5:13440191-13440658 Satlength=468 Nr of Repeats=12
RepeatLength=40 seed=TCTGGAATGT Num.seqs=11 Similarity=0.922424
31 ATTTTTCTAGAAAGTTCTGGAACATTCCAGAATTTTCCC-G
Rev.of_WB235_6:142874-143634 Satlength=761 Nr of Repeats=19
RepeatLength=40 seed=GAAAATTCTG Num.seqs=19 Similarity=0.974269
37 ATTTTTCTAGAAAGTTCTGGAACATTCCAGAATTTTCCC-G
Rev.of_WB235_4:16910953-16911111 Satlength=159 Nr of Repeats=4
RepeatLength=40 seed=AAAATTCTGG Num.seqs=3 Similarity=0.977778
36 ATTTTTCTAGAAAGTTCTGGAACATTCCAGAATTTTCTC-G
WB235_5:15320778-15321338 Satlength=561 Nr of Repeats=14 RepeatLength=40
seed=CGATTTTTCT Num.seqs=14 Similarity=0.947985
38 ATTTTTCTAGAAAGTTCTGGAACATTCCAGAATTTTCTC-G
WB235_6:1375049-1375406 Satlength=358 Nr of Repeats=9 RepeatLength=40
seed=ATTTTTCTAG Num.seqs=6 Similarity=0.937778
40 ATTTTTCTAGAAAGTTCTGGAACATTCCAGAATTTTCTC-G
Rev.of_WB235_1:6647885-6648325 Satlength=441 Nr of Repeats=11
RepeatLength=40 seed=TTCTGGAATG Num.seqs=11 Similarity=0.960000
32 ATTTTTCTAGAAAGTTCTGGAACATTCCAGAATTTTCTC-G
Rev.of_WB235_1:7868937-7869457 Satlength=521 Nr of Repeats=13
RepeatLength=40 seed=GAATGTTCCA Num.seqs=11 Similarity=0.935758
67 ATTTTTCTAGAAAGTTCTGGAACATTCCAGAATTTTCTC-G
WB235_2:9631891-9633131 Satlength=1241 Nr of Repeats=31 RepeatLength=40
seed=TTCTGGAACA Num.seqs=31 Similarity=0.949462
54 ATTTTTCTAGAAAGTTCTGGAACATTCCAGAATTTTCTC-G
WB235_3:424780-427345 Satlength=2566 Nr of Repeats=43 RepeatLength=40
seed=ACATTCCAGA Num.seqs=41 Similarity=0.892358
61 ATTTTTCTAGAAAGTTCTGGAACATTCCAGAATTTTCTC-G
Rev.of_WB235_3:3897112-3897672 Satlength=561 Nr of Repeats=14
RepeatLength=40 seed=GTTCCAGAAC Num.seqs=14 Similarity=0.942857
63 ATTTTTCTAGAAAGTTCTGGAACATTCCAGAATTTTCTC-G
WB235_4:7441099-7441297 Satlength=199 Nr of Repeats=5 RepeatLength=40
seed=TCCAGAATTT Num.seqs=4 Similarity=0.966667
65 ATTTTTCTAGAAAGTTCTGGAACATTCCAGAATTTTCTC-G
WB235_4:7441099-7441714 Satlength=616 Nr of Repeats=6 RepeatLength=40
seed=TCCAGAATTT Num.seqs=4 Similarity=0.966667
65 ATTTTTCTAGAAAGTTCTGGAACATTCCAGAATTTTCTC-G
WB235_4:8467091-8469338 Satlength=2248 Nr of Repeats=33 RepeatLength=40
seed=TCCAGAATTT Num.seqs=27 Similarity=0.869136
65 ATTTTTCTAGAAAGTTCTGGAACATTCCAGAATTTTCTC-G
Rev.of_WB235_4:13847379-13847579 Satlength=201 Nr of Repeats=5
RepeatLength=40 seed=AATGTTCCAG Num.seqs=5 Similarity=0.973333
66 ATTTTTCTAGAAAGTTCTGGAACATTCCAGAATTTTCTC-G

Rev.of_WB235_4:15166752-15167072 Satlength=321 Nr of Repeats=8 RepeatLength=40 seed=GAATGTTCCA Num.seqs=8 Similarity=0.897619
67 ATTTTTCTAGAAAGTTCTGGAACATTCCAGAATTTTCTC-G
WB235_4:16458486-16458684 Satlength=199 Nr of Repeats=5 RepeatLength=40 seed=TTCCAGAATT Num.seqs=4 Similarity=0.966667
64 ATTTTTCTAGAAAGTTCTGGAACATTCCAGAATTTTCTC-G
WB235_6:168652-168890 Satlength=239 Nr of Repeats=6 RepeatLength=40 seed=TTCCAGAATT Num.seqs=5 Similarity=0.986667
64 ATTTTTCTAGAAAGTTCTGGAACATTCCAGAATTTTCTC-G
WB235_6:2002759-2003439 Satlength=681 Nr of Repeats=17 RepeatLength=40 seed=GAATTTTCTC Num.seqs=17 Similarity=0.955392
69 ATTTTTCTAGAAAGTTCTGGAACATTCCAGAATTTTCTC-G
WB235_6:2057847-2058368 Satlength=522 Nr of Repeats=13 RepeatLength=40 seed=TTTTCTCGAT Num.seqs=12 Similarity=0.960606
32 ATTTTTCTAGAAAGTTCTGGAACATTCCAGAATTTTCTC-G
Rev.of_WB235_1:13231021-13231669 Satlength=649 Nr of Repeats=15 RepeatLength=40 seed=GAAAATTCTG Num.seqs=13 Similarity=0.967949
37 ATTTTTCTAGAAAGTTCTGGAACATTTCAGAATTTTCTC-G
Rev.of_WB235_6:17310825-17311809 Satlength=985 Nr of Repeats=10 RepeatLength=40 seed=AATGTTCCAG Num.seqs=9 Similarity=0.879630
66 ATTTTTCTAGAAAGTTCTGGAACATTTCAGAATTTTCTC-G
WB235_4:16675606-16675766 Satlength=161 Nr of Repeats=4 RepeatLength=40 seed=TTCCAGAATT Num.seqs=4 Similarity=0.950000
64 ATTTTTCTAGAAAGTTCTGGAACGTTCCAGAATTTTCCC-G
WB235_5:4052517-4053360 Satlength=844 Nr of Repeats=21 RepeatLength=40 seed=CTAGAAAGTT Num.seqs=18 Similarity=0.796514
46 AATTTCCTAGAAAGTTCTGGAACATTCCAGAATTTTCTT-G
Rev.of_WB235_3:8344363-8344643 Satlength=281 Nr of Repeats=7 RepeatLength=40 seed=TCTGGAATGT Num.seqs=7 Similarity=0.971429
31 ATTTTTCTAGAAAGTTCTGGAACATTCCAGACTTTTCCC-A
WB235_4:1153760-1154039 Satlength=280 Nr of Repeats=7 RepeatLength=40 seed=ATTTTTCTAG Num.seqs=6 Similarity=0.911111
40 ATTTTTCTAGAAAGTTCTGGAACATTCCAGACTTTTCCC-A
WB235_4:1153773-1154667 Satlength=895 Nr of Repeats=10 RepeatLength=40 seed=GTTCTGGAAC Num.seqs=7 Similarity=0.844444
53 ATTTTTCTAGAAAGTTCTGGAACATTCCAGACTTTTCCC-A
WB235_5:1403527-1403806 Satlength=280 Nr of Repeats=7 RepeatLength=40 seed=ATTTTTCTAG Num.seqs=6 Similarity=0.880000
40 ATTTTTCTAGAAAGTTCTGGAACATTCCAGACTTTTCCC-A
Rev.of_WB235_5:7916966-7925007 Satlength=8042 Nr of Repeats=201 RepeatLength=40 seed=TTCTAGAAAA Num.seqs=194 Similarity=0.914186
52 ATTTTTCTAGAAAGTTCTGGAACATTCCAGACTTTTCCC-A
Rev.of_WB235_1:778056-778335 Satlength=280 Nr of Repeats=6 RepeatLength=40 seed=GAACCTTCTG Num.seqs=4 Similarity=0.850000
7 AAGGTTCTAGAACATTCCAGAATTTTCTCAAATTTTCCA-G
Rev.of_WB235_2:4554394-4554914 Satlength=521 Nr of Repeats=13 RepeatLength=40 seed=TCCAGAACCT Num.seqs=13 Similarity=0.958120
11 AAGGTTCTGGAACATTCCAGAATTTTCTCAAATTTTCCA-G
WB235_4:8467000-8468026 Satlength=1027 Nr of Repeats=15 RepeatLength=40 seed=TTCTGGAACA Num.seqs=14 Similarity=0.912088
44 AAGGTTCTGGAACATTCCAGAATTTTCTCAAATTTTCCA-G
WB235_6:14723559-14724409 Satlength=851 Nr of Repeats=5 RepeatLength=40 seed=TTCTGGAACA Num.seqs=4 Similarity=0.866667
44 AAGGTTCTGGAACATTCCAGAATTTTCTCAAATTTTCCA-G

Rev.of_WB235_6:16100682-16101122 Satlength=441 Nr of Repeats=11 RepeatLength=40 seed=TCCAGAACCT Num.seqs=11 Similarity=0.950303
11 AAGGTTCTGGAACATTCCAGAATTTTCTCAAATTTTCCA-G
Rev.of_WB235_3:11160584-11160784 Satlength=201 Nr of Repeats=5 RepeatLength=40 seed=TCCAGAACCT Num.seqs=5 Similarity=0.960000
11 AAGGTTCTGGAACATTCCAGCATTTTCTCAAATTTTCCA-G
WB235_4:15709349-15709549 Satlength=201 Nr of Repeats=5 RepeatLength=40 seed=ATTTTCCAGA Num.seqs=5 Similarity=0.920000
31 AAGGTTCTGGAACATTCCAGAATTTTCTCAAATTTTCCA-G
WB235_2:633567-633717 Satlength=151 Nr of Repeats=4 RepeatLength=40 seed=CAGAAGGTTC Num.seqs=3 Similarity=0.800000
37 AAGGTTCTGGAACATTCCAGAATTTTCTAGAATTTTCCA-G
Rev.of_WB235_3:3895928-3896228 Satlength=301 Nr of Repeats=8 RepeatLength=40 seed=AGAACCTTCT Num.seqs=6 Similarity=0.824444
48 AAGGTTCTGGAACATTCCAGAATTTTCTAGAATTTTCCA-G
Rev.of_WB235_3:9534180-9534500 Satlength=321 Nr of Repeats=8 RepeatLength=40 seed=TCCAGAACCT Num.seqs=8 Similarity=0.945238
51 AAGGTTCTGGAACATTCCAGAATTTTCTCGAATTTTCCA-G
Rev.of_WB235_3:9534215-9534836 Satlength=622 Nr of Repeats=9 RepeatLength=40 seed=AATGTTCCAG Num.seqs=8 Similarity=0.903571
56 AAGGTTCTGGAACATTCCAGAATTTTCTCGAATTTTCCA-G
Rev.of_WB235_4:12022575-12022894 Satlength=320 Nr of Repeats=8 RepeatLength=40 seed=TCCAGAACCT Num.seqs=7 Similarity=0.946032
51 AAGGTTCTGGAACATTCCAGAATTTTCTCGAATTTTCCA-G
Rev.of_WB235_4:13861901-13862381 Satlength=481 Nr of Repeats=12 RepeatLength=40 seed=CAGAACCTTC Num.seqs=12 Similarity=0.914646
89 AAGGTTCTGGAACATTCCAGAATTTTCTCGAATTTTCCA-G
Rev.of_WB235_5:3520502-3520742 Satlength=241 Nr of Repeats=6 RepeatLength=40 seed=TCCAGAACCT Num.seqs=6 Similarity=0.931111
51 AAGGTTCTGGAACATTCCAGAATTTTCTCGAATTTTCCA-G
Rev.of_WB235_6:9007668-9008148 Satlength=481 Nr of Repeats=12 RepeatLength=40 seed=CTTCTGGAAA Num.seqs=12 Similarity=0.953030
83 AAGGTTCTGGAACATTCCAGAATTTTCTCGAATTTTCCA-G
WB235_1:780732-781012 Satlength=281 Nr of Repeats=7 RepeatLength=40 seed=TCTAGAACAT Num.seqs=7 Similarity=0.838095
45 AAGGTTCTAGAACATTCCAGAATTTTCTCGAATTTTCCA-A
WB235_5:1355392-1355652 Satlength=261 Nr of Repeats=6 RepeatLength=40 seed=CTAGAACATT Num.seqs=5 Similarity=0.712195
46 AANGTTCTAGAACATTCCAGAATTTTCTCGAATTTTCCA-G
Rev.of_WB235_1:14033367-14034207 Satlength=841 Nr of Repeats=24 RepeatLength=40 seed=TTCTGGAAAA Num.seqs=18 Similarity=0.755338
82 AAGGTTCTAGAACATTCCAGAATTTTATCGAATTTTCCA-G
Rev.of_WB235_4:2754821-2756571 Satlength=1751 Nr of Repeats=49 RepeatLength=40 seed=TTCTGGAAAA Num.seqs=36 Similarity=0.839524
82 AAGGTTCTAGAACATTCCAGAATTTTTTTCGAATTTTCCA-G
Rev.of_WB235_5:4050682-4051242 Satlength=561 Nr of Repeats=14 RepeatLength=40 seed=GAACCTTCTG Num.seqs=12 Similarity=0.857071
47 AAGGTTCAAAAACATTCCAGAATTTTCTCGAATTTTCCA-G
WB235_5:1530916-1531076 Satlength=161 Nr of Repeats=4 RepeatLength=40 seed=GTTCTAGAAC Num.seqs=4 Similarity=0.875339
84 AAGGTTCTAGAACATTCCAGAATTTTCTCGAATTTACCAGG
WB235_3:12001471-12001873 Satlength=403 Nr of Repeats=10 RepeatLength=40 seed=ATTCCAGAAT Num.seqs=8 Similarity=0.860714
13 AAGGTTCTAGAACATTCCAGAATTTTTTAAAATTTTCCA-G

Rev.of_WB235_5:7115024-7115184 Satlength=161 Nr of Repeats=4 RepeatLength=40 seed=TTCTAGAACC Num.seqs=4 Similarity=0.794444
52 AAGGTTCTAGAACATTCCAGAATTTTCTNAAACTTTCCA-G
WB235_5:7442929-7443089 Satlength=161 Nr of Repeats=4 RepeatLength=40 seed=GAAGGTTCTA Num.seqs=4 Similarity=0.794444
79 AAGGTTCTAGAACATTCCAGAATTTTCTNAAAATTTCCA-G
WB235_4:2694935-2695125 Satlength=191 Nr of Repeats=5 RepeatLength=40 seed=GAATTTTCCA Num.seqs=4 Similarity=0.822222
29 AAAGTTCTAGAAAATTCCAGAATTTTTTCGAATTTTCCA-G
Rev.of_WB235_1:3980821-3981021 Satlength=201 Nr of Repeats=6 RepeatLength=40 seed=TTTCTAGAAA Num.seqs=4 Similarity=0.816667
43 AAAATTCTGGACCATTCCGGAATTTTCTCGAATTTTCTA-G
Rev.of_WB235_4:3671203-3671723 Satlength=521 Nr of Repeats=15 RepeatLength=40 seed=TTTCTAGAAA Num.seqs=11 Similarity=0.820606
43 AAAATTCTGGACCATTCCNGAATTTTCTCGAATTTTCTA-G
Rev.of_WB235_1:7741780-7742262 Satlength=483 Nr of Repeats=13 RepeatLength=40 seed=TTTCTAGAAA Num.seqs=9 Similarity=0.834259
43 AAAGTTCTGGAATATTCCAGANTTTTCCCGAATTTTCTA-G
WB235_4:14740244-14740404 Satlength=161 Nr of Repeats=5 RepeatLength=40 seed=TTTTCTAGAA Num.seqs=3 Similarity=0.755556
72 AAAGTTCTGGAACATTCCAGAATTTTCCCGAATTTTCTA-G
Rev.of_WB235_5:9603508-9604068 Satlength=561 Nr of Repeats=15 RepeatLength=40 seed=TTTCTAGAAA Num.seqs=13 Similarity=0.855128
83 AAAATTCTGGAACATTCCAGAATTTTCTCGAATTTTCTA-G
WB235_6:16851316-16851756 Satlength=441 Nr of Repeats=13 RepeatLength=40 seed=TTTCTAGAAA Num.seqs=9 Similarity=0.814815
113 AAANTTCTGGAACATTCCAGAATTTTCTCGAATTTTCTA-G
Rev.of_WB235_5:13882781-13883141 Satlength=361 Nr of Repeats=10 RepeatLength=40 seed=TTTCTAGAAA Num.seqs=8 Similarity=0.826190
83 AAAGTTCTGGAACATTCCAGAATTTTCTCGAATTTTCTA-G
WB235_1:14686490-14687331 Satlength=842 Nr of Repeats=22 RepeatLength=40 seed=TCTAGAAAGT Num.seqs=19 Similarity=0.888304
75 AAAGTTCTGGAACATTCCAGAATTTTCTCGATTTTTCTA-G
WB235_4:13463828-13464877 Satlength=1050 Nr of Repeats=12 RepeatLength=40 seed=GTTCTGGAAC Num.seqs=10 Similarity=0.891111
83 AAAGTTCTGGAACATTCCAGAATTTTCTCGATTTTTCTA-G
WB235_6:2057868-2059356 Satlength=1489 Nr of Repeats=20 RepeatLength=40 seed=GTTCTGGAAC Num.seqs=15 Similarity=0.890159
83 AAAGTTCTGGAACATTCCAGAATTTTCTCGATTTTTCTA-G
WB235_6:13795118-13795677 Satlength=560 Nr of Repeats=14 RepeatLength=40 seed=GAACATTCCA Num.seqs=13 Similarity=0.911111
89 AAAGTTCTGGAACATTCCAGAATTTTCTCGATTTTTCTA-G
WB235_6:13795118-13796907 Satlength=1790 Nr of Repeats=19 RepeatLength=40 seed=GAACATTCCA Num.seqs=16 Similarity=0.884722
89 AAAGTTCTGGAACATTCCAGAATTTTCTCGATTTTTCTA-G
WB235_4:15895865-15896183 Satlength=319 Nr of Repeats=8 RepeatLength=40 seed=GAACATTCCA Num.seqs=6 Similarity=0.922222
89 AAAGTTCTGGAACATTCCAGAATTTTCTCAATTTTTCTA-G
WB235_2:632493-632813 Satlength=321 Nr of Repeats=8 RepeatLength=40 seed=TTTCTAGAAA Num.seqs=8 Similarity=0.917857
113 AAAATTCTGGAACATTCCAGAATTTTCCCGATTTTTCTA-G
WB235_3:424806-426326 Satlength=1521 Nr of Repeats=38 RepeatLength=40 seed=TAGAAAGTTC Num.seqs=38 Similarity=0.903556
117 AAAGTTCTGGAACATTCCAGAATTTTCCCGATTTTTCTA-G

Rev.of_WB235_4:15826528-15827586 Satlength=1059 Nr of Repeats=8
RepeatLength=40 seed=GTTCCAGAAC Num.seqs=7 Similarity=0.814286
133   AAAGTTCTGGAACATTCCAGAATTTTCCCGATTTTTCTA-G
Rev.of_WB235_5:6072277-6072797 Satlength=521 Nr of Repeats=13
RepeatLength=40 seed=TTCCAGAACT Num.seqs=13 Similarity=0.959829
132   AAAGTTCTGGAACATTCCAGAATTTTCCCGATTTTTCTA-G
WB235_4:4920724-4920924 Satlength=201 Nr of Repeats=5 RepeatLength=40
seed=TTCTAGAAAG Num.seqs=5 Similarity=0.913333
154   AAAGTTCTGGAACATTCNAGAATTTTCCCGATTTTTCTA-G
WB235_2:8460034-8460434 Satlength=401 Nr of Repeats=10 RepeatLength=40
seed=AAAGTTCTGG Num.seqs=10 Similarity=0.901482
80   AAAGTTCTGGAACATTCCAGAATTTTCCCAATTTTTCTA-G
WB235_3:13447046-13447886 Satlength=841 Nr of Repeats=21 RepeatLength=40
seed=ATTTTTCTAG Num.seqs=21 Similarity=0.903175
110   AAAGTTCTGGAACATTCCAGAATTTTCCCAATTTTTCTA-G
WB235_4:15374013-15374693 Satlength=681 Nr of Repeats=17 RepeatLength=40
seed=GAACATTCCA Num.seqs=17 Similarity=0.919608
89   AAAGTTCTGGAACATTCCAGAATTTTCCCAATTTTTCTA-G
WB235_5:7912986-7913626 Satlength=641 Nr of Repeats=16 RepeatLength=40
seed=TCTGGAACAT Num.seqs=16 Similarity=0.859790
85   AAAGTTCTGGAACATTCCAGAATTTTCCCAATTTTTCTA-G
WB235_6:15635133-15635373 Satlength=241 Nr of Repeats=6 RepeatLength=40
seed=AAAGTTCTGG Num.seqs=6 Similarity=0.904444
80   AAAGTTCTGGAACATTCCAGAATTTTCCCAATTTTTCTA-G
WB235_4:15059144-15059784 Satlength=641 Nr of Repeats=16 RepeatLength=40
seed=TTTTCTAGAA Num.seqs=16 Similarity=0.900000
112   AAAGTTCTGGAACATTCCAGAAATTTCCCGATTTTTCTA-G

*   * *   *   ***   *   *
Consensus:

AannTTCTnGAAcaTTCcaGAAttTTCtnGAaTTTTCcaG

>WB235_Fam_6_11_75 Nr. of seq. 75 Alignment length(with gaps) = 12
Alignment score = 0.809099
WB235_1:562402-562457 Satlength=56 Nr of Repeats=5 RepeatLength=11
seed=GTACTCCTAC Num.seqs=5 Similarity=0.830303
0   GTACTC-CTACC
Rev.of_WB235_1:1214097-1214254 Satlength=158 Nr of Repeats=7
RepeatLength=11 seed=GTACTGTAGG Num.seqs=6 Similarity=0.870707
4   GTACTC-CTACA
Rev.of_WB235_1:13230567-13230940 Satlength=374 Nr of Repeats=12
RepeatLength=11 seed=GTACTGTAGG Num.seqs=9 Similarity=0.952862
4   GTACTC-CTACA
Rev.of_WB235_1:14402777-14402900 Satlength=124 Nr of Repeats=4
RepeatLength=11 seed=GTACTGTAGG Num.seqs=3 Similarity=0.919192
4   GTACTC-CTACA
WB235_2:633813-633947 Satlength=135 Nr of Repeats=4 RepeatLength=11
seed=GTACTCCTAC Num.seqs=3 Similarity=0.838384
11   GTACTC-CTACA
WB235_2:9634505-9634639 Satlength=135 Nr of Repeats=5 RepeatLength=11
seed=GTACTCCTAC Num.seqs=4 Similarity=0.878788
11   GTACTC-CTACA

Rev.of_WB235_2:13989510-13989877 Satlength=368 Nr of Repeats=11 RepeatLength=11 seed=GTACTGTAGG Num.seqs=8 Similarity=0.878788
4 GTACTC-CTACA
WB235_3:427473-427977 Satlength=505 Nr of Repeats=18 RepeatLength=11 seed=CTACAGTACT Num.seqs=12 Similarity=0.773779
6 GTACTC-CTACA
Rev.of_WB235_3:1320466-1320612 Satlength=147 Nr of Repeats=5 RepeatLength=11 seed=GTACTGTAGG Num.seqs=3 Similarity=0.919192
4 GTACTC-CTACA
Rev.of_WB235_3:9533702-9534094 Satlength=393 Nr of Repeats=13 RepeatLength=11 seed=GTACTGTAGG Num.seqs=8 Similarity=0.796537
4 GTACTC-CTACA
WB235_4:1154757-1155014 Satlength=258 Nr of Repeats=8 RepeatLength=11 seed=GTACTCCTAC Num.seqs=5 Similarity=0.854545
11 GTACTC-CTACA
WB235_4:2245441-2245572 Satlength=132 Nr of Repeats=4 RepeatLength=11 seed=GTACTCCTAC Num.seqs=3 Similarity=0.838384
11 GTACTC-CTACA
Rev.of_WB235_4:2754343-2754722 Satlength=380 Nr of Repeats=12 RepeatLength=11 seed=TACTGTAGGA Num.seqs=8 Similarity=0.787698
14 GTACTC-CTACA
Rev.of_WB235_4:4305710-4305967 Satlength=258 Nr of Repeats=5 RepeatLength=11 seed=GTACTGTAGG Num.seqs=3 Similarity=1.000000
4 GTACTC-CTACA
WB235_4:4325075-4325582 Satlength=508 Nr of Repeats=13 RepeatLength=11 seed=CTACAGTACT Num.seqs=9 Similarity=0.878788
6 GTACTC-CTACA
WB235_4:4554554-4555060 Satlength=507 Nr of Repeats=13 RepeatLength=11 seed=CTACAGTACT Num.seqs=9 Similarity=0.878788
6 GTACTC-CTACA
WB235_4:9411725-9412004 Satlength=280 Nr of Repeats=10 RepeatLength=11 seed=GTACTCCTAC Num.seqs=8 Similarity=0.883117
11 GTACTC-CTACA
Rev.of_WB235_4:13845937-13846071 Satlength=135 Nr of Repeats=6 RepeatLength=11 seed=AGTACTGTAG Num.seqs=4 Similarity=0.939394
5 GTACTC-CTACA
Rev.of_WB235_4:13922018-13922152 Satlength=135 Nr of Repeats=6 RepeatLength=11 seed=AGTACTGTAG Num.seqs=4 Similarity=0.939394
5 GTACTC-CTACA
WB235_4:15766783-15767403 Satlength=621 Nr of Repeats=19 RepeatLength=11 seed=GTACTCCTAC Num.seqs=13 Similarity=0.888112
11 GTACTC-CTACA
Rev.of_WB235_4:16094815-16095563 Satlength=749 Nr of Repeats=18 RepeatLength=11 seed=GTACTGTAGG Num.seqs=12 Similarity=0.878788
4 GTACTC-CTACA
Rev.of_WB235_4:16910220-16910354 Satlength=135 Nr of Repeats=6 RepeatLength=11 seed=AGTACTGTAG Num.seqs=4 Similarity=0.939394
5 GTACTC-CTACA
WB235_4:17076321-17076588 Satlength=268 Nr of Repeats=10 RepeatLength=11 seed=CCTACAGTAC Num.seqs=6 Similarity=0.716667
5 GTACTC-CTACA
Rev.of_WB235_5:3520159-3520417 Satlength=259 Nr of Repeats=7 RepeatLength=11 seed=TACTGTAGGA Num.seqs=5 Similarity=0.763889
14 GTACTC-CTACA

Rev.of_WB235_5:5004204-5005690 Satlength=1487 Nr of Repeats=35 RepeatLength=11 seed=GTACTGTAGG Num.seqs=22 Similarity=0.888233
4 GTACTC-CTACA
WB235_5:9439666-9439923 Satlength=258 Nr of Repeats=8 RepeatLength=11 seed=GTACTCCTAC Num.seqs=6 Similarity=0.870707
11 GTACTC-CTACA
WB235_5:15322538-15322672 Satlength=135 Nr of Repeats=5 RepeatLength=11 seed=GTACTCCTAC Num.seqs=4 Similarity=0.878788
11 GTACTC-CTACA
Rev.of_WB235_5:16547005-16547587 Satlength=583 Nr of Repeats=10 RepeatLength=11 seed=GTACTGTAGG Num.seqs=6 Similarity=0.846465
4 GTACTC-CTACA
WB235_5:17495404-17495538 Satlength=135 Nr of Repeats=5 RepeatLength=11 seed=CCTACAGTAC Num.seqs=3 Similarity=0.919192
5 GTACTC-CTACA
WB235_5:20588792-20588997 Satlength=206 Nr of Repeats=5 RepeatLength=11 seed=CCTACAGTAC Num.seqs=3 Similarity=1.000000
5 GTACTC-CTACA
WB235_6:1376859-1377239 Satlength=381 Nr of Repeats=13 RepeatLength=11 seed=GTACTCCTAC Num.seqs=10 Similarity=0.886869
11 GTACTC-CTACA
WB235_6:5230547-5230804 Satlength=258 Nr of Repeats=10 RepeatLength=11 seed=CTACAGTACT Num.seqs=7 Similarity=0.849928
6 GTACTC-CTACA
WB235_6:11489369-11489615 Satlength=247 Nr of Repeats=7 RepeatLength=11 seed=TCCTACAGTA Num.seqs=5 Similarity=0.854545
4 GTACTC-CTACA
WB235_6:13797037-13797540 Satlength=504 Nr of Repeats=19 RepeatLength=11 seed=CTACAGTACT Num.seqs=14 Similarity=0.771368
6 GTACTC-CTACA
WB235_6:14724532-14724801 Satlength=270 Nr of Repeats=10 RepeatLength=11 seed=CCTACAGTAC Num.seqs=6 Similarity=0.738889
5 GTACTC-CTACA
WB235_1:1430568-1430936 Satlength=369 Nr of Repeats=14 RepeatLength=11 seed=CTACAGTACT Num.seqs=10 Similarity=0.753704
6 GTACTN-CTACA
Rev.of_WB235_2:1033777-1034280 Satlength=504 Nr of Repeats=17 RepeatLength=11 seed=GTACTGTAGG Num.seqs=11 Similarity=0.819284
4 GTACTN-CTACA
WB235_2:2348715-2349106 Satlength=392 Nr of Repeats=13 RepeatLength=11 seed=CTACAGTACC Num.seqs=9 Similarity=0.763889
6 GTACTN-CTACA
WB235_2:12701130-12701355 Satlength=226 Nr of Repeats=7 RepeatLength=11 seed=CTACAGTACC Num.seqs=5 Similarity=0.775000
6 GTACTN-CTACA
WB235_4:8565636-8566140 Satlength=505 Nr of Repeats=13 RepeatLength=11 seed=CTACAGTACT Num.seqs=9 Similarity=0.865320
6 GTACTN-CTACA
Rev.of_WB235_4:12022220-12022489 Satlength=270 Nr of Repeats=10 RepeatLength=11 seed=GTACTGTAGG Num.seqs=7 Similarity=0.832612
4 GTACTN-CTACA
WB235_4:16511822-16512828 Satlength=1007 Nr of Repeats=39 RepeatLength=11 seed=CTACAGTACT Num.seqs=30 Similarity=0.778225
6 GTACTN-CTACA

WB235_5:1911033-1911178 Satlength=146 Nr of Repeats=7 RepeatLength=11 seed=CTACAGTACT Num.seqs=6 Similarity=0.723148
6   GTACTN-CTACA
WB235_5:16878345-16878591 Satlength=247 Nr of Repeats=7 RepeatLength=11 seed=CTACAGTACC Num.seqs=5 Similarity=0.786111
6   GTACTN-CTACA
WB235_5:20260289-20260916 Satlength=628 Nr of Repeats=16 RepeatLength=11 seed=CTACAGTACT Num.seqs=11 Similarity=0.854545
6   GTACTN-CTACA
WB235_6:944053-944332 Satlength=280 Nr of Repeats=13 RepeatLength=11 seed=CTACAGTACT Num.seqs=11 Similarity=0.823691
6   GTACTN-CTACA
Rev.of_WB235_6:1610992-1611280 Satlength=289 Nr of Repeats=10 RepeatLength=11 seed=GTACTGTAGG Num.seqs=6 Similarity=0.790741
4   GTACTN-CTACA
Rev.of_WB235_6:9007305-9007574 Satlength=270 Nr of Repeats=10 RepeatLength=11 seed=GTACTGTAGG Num.seqs=7 Similarity=0.832612
4   GTACTN-CTACA
Rev.of_WB235_6:16100349-16100596 Satlength=248 Nr of Repeats=8 RepeatLength=11 seed=GTACTGTAGG Num.seqs=5 Similarity=0.755556
4   GTACTN-CTACA
Rev.of_WB235_2:10832628-10833651 Satlength=1024 Nr of Repeats=24 RepeatLength=11 seed=GTACTGTAGG Num.seqs=16 Similarity=0.896970
4   GTACTA-CTACA
WB235_5:1404428-1404696 Satlength=269 Nr of Repeats=8 RepeatLength=11 seed=CTACAGTACC Num.seqs=6 Similarity=0.798148
6   GTACTA-CTACA
Rev.of_WB235_6:28398-28532 Satlength=135 Nr of Repeats=5 RepeatLength=11 seed=GTACTGTAGT Num.seqs=3 Similarity=1.000000
4   GTACTA-CTACA
Rev.of_WB235_1:11420316-11421039 Satlength=724 Nr of Repeats=15 RepeatLength=11 seed=GTACTGTAGG Num.seqs=9 Similarity=0.841751
4   GTACCC-CTACA
Rev.of_WB235_2:882370-882522 Satlength=153 Nr of Repeats=5 RepeatLength=11 seed=TGTAGGGGTA Num.seqs=3 Similarity=1.000000
11   GTACCC-CTACA
WB235_2:12350820-12350876 Satlength=57 Nr of Repeats=5 RepeatLength=11 seed=CTACAGTACC Num.seqs=4 Similarity=0.919192
6   GTACCC-CTACA
WB235_2:13415355-13415629 Satlength=275 Nr of Repeats=20 RepeatLength=11 seed=CCTACAGTAC Num.seqs=12 Similarity=0.853076
5   GTACCC-CTACA
WB235_3:259921-260355 Satlength=435 Nr of Repeats=11 RepeatLength=11 seed=CCTACAGTAC Num.seqs=7 Similarity=0.965368
5   GTACCC-CTACA
Rev.of_WB235_3:1326908-1327395 Satlength=488 Nr of Repeats=33 RepeatLength=11 seed=GGTACTGTAG Num.seqs=21 Similarity=0.844156
5   GTACCC-CTACA
Rev.of_WB235_3:1633390-1634249 Satlength=860 Nr of Repeats=41 RepeatLength=11 seed=TACTGTAGGG Num.seqs=30 Similarity=0.811076
14   GTACCC-CTACA
WB235_4:9433776-9433910 Satlength=135 Nr of Repeats=4 RepeatLength=11 seed=CCCTACAGTA Num.seqs=3 Similarity=0.919192
4   GTACCC-CTACA

WB235_4:16522968-16523369 Satlength=402 Nr of Repeats=28 RepeatLength=11
seed=CCCCTACAGT Num.seqs=17 Similarity=0.887701
14   GTACCC-CTACA
WB235_5:14057570-14057714 Satlength=145 Nr of Repeats=6 RepeatLength=11
seed=CTACAGTACC Num.seqs=4 Similarity=0.818182
6   GTACCC-CTACA
Rev.of_WB235_5:15823540-15823605 Satlength=66 Nr of Repeats=4
RepeatLength=11 seed=GTACTGTAGG Num.seqs=3 Similarity=0.919192
4   GTACCC-CTACA
WB235_5:17619161-17619418 Satlength=258 Nr of Repeats=8 RepeatLength=11
seed=CCTACAGTAC Num.seqs=6 Similarity=0.886869
5   GTACCC-CTACA
Rev.of_WB235_2:4094407-4094663 Satlength=257 Nr of Repeats=13
RepeatLength=11 seed=GTACTGTAGG Num.seqs=9 Similarity=0.735043
4   GTACNC-CTACA
WB235_3:520005-520140 Satlength=136 Nr of Repeats=6 RepeatLength=11
seed=CTACAGTACC Num.seqs=4 Similarity=0.858586
6   GTACCA-CTACA
WB235_3:2035722-2035981 Satlength=260 Nr of Repeats=20 RepeatLength=11
seed=CTACAGTACC Num.seqs=14 Similarity=0.795788
6   GTACCN-CTACA
WB235_5:2141423-2141937 Satlength=515 Nr of Repeats=18 RepeatLength=11
seed=CTACAGTACC Num.seqs=14 Similarity=0.742063
6   GTACCN-CTACA
Rev.of_WB235_4:10025402-10025894 Satlength=493 Nr of Repeats=10
RepeatLength=11 seed=TACTGTAGGG Num.seqs=6 Similarity=0.935354
14   GTAACC-CTACA
Rev.of_WB235_5:3731028-3731508 Satlength=481 Nr of Repeats=23
RepeatLength=11 seed=AGGATTACTG Num.seqs=15 Similarity=0.843001
19   GTAATC-CTACA
WB235_3:11331372-11331497 Satlength=126 Nr of Repeats=4 RepeatLength=11
seed=CTACAGTACT Num.seqs=3 Similarity=0.838384
6   GTACTT-CTACA
Rev.of_WB235_3:11700371-11700706 Satlength=336 Nr of Repeats=7
RepeatLength=11 seed=GTACTGTAGA Num.seqs=5 Similarity=0.854545
15   GTACNT-CTACA
WB235_5:7443218-7444716 Satlength=1499 Nr of Repeats=50 RepeatLength=11
seed=CTACAGTACC Num.seqs=38 Similarity=0.685961
6   GTACCT-CTACA
WB235_4:2136323-2136466 Satlength=144 Nr of Repeats=5 RepeatLength=11
seed=CTACAGTACC Num.seqs=4 Similarity=0.796296
6   GTACCTACTACA
Rev.of_WB235_1:11763187-11763333 Satlength=147 Nr of Repeats=6
RepeatLength=11 seed=AGTACTGTAG Num.seqs=4 Similarity=0.731481
16   GTACTNACTACA

***   ****
Consensus:

GTACtcCTACA

>WB235_Fam_7_15_70 Nr. of seq. 70 Alignment length(with gaps) = 15
Alignment score = 0.826961

WB235_1:902260-902411 Satlength=152 Nr of Repeats=8 RepeatLength=15
seed=ATCTTCTTTT Num.seqs=5 Similarity=0.911111
0 ATCTTCTTTTCGTAG
WB235_1:2144021-2144187 Satlength=167 Nr of Repeats=10 RepeatLength=15
seed=AGATCTTGTT Num.seqs=8 Similarity=0.831746
13 ATCTTGTTTTCGTAG
Rev.of_WB235_2:12002438-12002513 Satlength=76 Nr of Repeats=4
RepeatLength=15 seed=AAAACAAGAT Num.seqs=3 Similarity=0.792593
25 ATCTTGTTTTNGTAG
WB235_2:690749-690809 Satlength=61 Nr of Repeats=4 RepeatLength=15
seed=GTAGATCTTG Num.seqs=4 Similarity=0.675347
26 ATCTTGTTTTGGTAG
WB235_2:7475407-7475467 Satlength=61 Nr of Repeats=4 RepeatLength=15
seed=GTAGATCTTG Num.seqs=4 Similarity=0.675347
26 ATCTTGTTTTGGTAG
WB235_4:17285019-17285079 Satlength=61 Nr of Repeats=4 RepeatLength=15
seed=GTAGATCTTG Num.seqs=4 Similarity=0.675347
26 ATCTTGTTTTGGTAG
Rev.of_WB235_5:100578-100668 Satlength=91 Nr of Repeats=6 RepeatLength=15
seed=AAAAGGAGAA Num.seqs=6 Similarity=0.822222
10 TTCTCCTTTTCGTNG
WB235_5:100901-100991 Satlength=91 Nr of Repeats=6 RepeatLength=15
seed=TTCTCCTTTT Num.seqs=6 Similarity=0.792593
15 TTCTCCTTTTCGTNG
Rev.of_WB235_1:4958855-4958932 Satlength=78 Nr of Repeats=5
RepeatLength=15 seed=CGAAAAGGGG Num.seqs=4 Similarity=0.807407
12 CTCCCCTTTTCGTAT
WB235_1:4959104-4959196 Satlength=93 Nr of Repeats=5 RepeatLength=15
seed=CTCCCCTTTT Num.seqs=3 Similarity=0.847222
15 CTCCCCTTTTCGTAT
WB235_1:4961695-4961802 Satlength=108 Nr of Repeats=6 RepeatLength=15
seed=CTCCCCTTTT Num.seqs=4 Similarity=0.869792
15 CTCCCCTTTTCGTAT
Rev.of_WB235_2:1134516-1134593 Satlength=78 Nr of Repeats=5
RepeatLength=15 seed=CGAAAAGGGG Num.seqs=4 Similarity=0.777778
12 CTCCCCTTTTCGTAT
Rev.of_WB235_3:6551383-6551520 Satlength=138 Nr of Repeats=9
RepeatLength=15 seed=CGAAAAGGGG Num.seqs=8 Similarity=0.847619
12 CTCCCCTTTTCGTAT
Rev.of_WB235_3:12878018-12878110 Satlength=93 Nr of Repeats=5
RepeatLength=15 seed=CGAAAAGGGG Num.seqs=3 Similarity=0.881481
12 CTCCCCTTTTCGTAT
WB235_4:1447227-1447319 Satlength=93 Nr of Repeats=6 RepeatLength=15
seed=TCCCCTTTTC Num.seqs=5 Similarity=0.840000
16 CTCCCCTTTTCGTAT
Rev.of_WB235_4:4702090-4702167 Satlength=78 Nr of Repeats=5
RepeatLength=15 seed=CGAAAAGGGG Num.seqs=4 Similarity=0.777778
12 CTCCCCTTTTCGTAT
Rev.of_WB235_4:8015849-8015941 Satlength=93 Nr of Repeats=5
RepeatLength=15 seed=CGAAAAGGGG Num.seqs=3 Similarity=0.881481
12 CTCCCCTTTTCGTAT
WB235_4:12621744-12621836 Satlength=93 Nr of Repeats=5 RepeatLength=15
seed=TCCCCTTTTC Num.seqs=3 Similarity=0.881481
16 CTCCCCTTTTCGTAT

WB235_1:5176308-5176383 Satlength=76 Nr of Repeats=5 RepeatLength=15 seed=TCCCCTTTTC Num.seqs=5 Similarity=0.768889
16   CTCCCCTTTTCGTAG
Rev.of_WB235_1:10468958-10469050 Satlength=93 Nr of Repeats=6 RepeatLength=15 seed=CGAAAAGGGG Num.seqs=5 Similarity=0.768889
27   CTCCCCTTTTCGTAG
WB235_1:10469225-10469317 Satlength=93 Nr of Repeats=6 RepeatLength=15 seed=TCCCCTTTTC Num.seqs=5 Similarity=0.768889
16   CTCCCCTTTTCGTAG
WB235_2:1134783-1134875 Satlength=93 Nr of Repeats=6 RepeatLength=15 seed=TCCCCTTTTC Num.seqs=5 Similarity=0.768889
16   CTCCCCTTTTCGTAG
Rev.of_WB235_2:7894727-7894819 Satlength=93 Nr of Repeats=6 RepeatLength=15 seed=CGAAAAGGGG Num.seqs=5 Similarity=0.768889
27   CTCCCCTTTTCGTAG
WB235_2:7894995-7895087 Satlength=93 Nr of Repeats=6 RepeatLength=15 seed=CCCCTTTTCG Num.seqs=5 Similarity=0.733333
17   CTCCCCTTTTCGTAG
WB235_2:10248425-10248517 Satlength=93 Nr of Repeats=6 RepeatLength=15 seed=TCCCCTTTTC Num.seqs=5 Similarity=0.733333
16   CTCCCCTTTTCGTAG
Rev.of_WB235_2:11534628-11534720 Satlength=93 Nr of Repeats=6 RepeatLength=15 seed=CGAAAAGGGG Num.seqs=5 Similarity=0.768889
27   CTCCCCTTTTCGTAG
WB235_2:11534895-11534970 Satlength=76 Nr of Repeats=5 RepeatLength=15 seed=TCCCCTTTTC Num.seqs=5 Similarity=0.768889
16   CTCCCCTTTTCGTAG
Rev.of_WB235_2:14346267-14346344 Satlength=78 Nr of Repeats=5 RepeatLength=15 seed=CGAAAAGGGG Num.seqs=4 Similarity=0.748148
27   CTCCCCTTTTCGTAG
WB235_3:7708901-7708993 Satlength=93 Nr of Repeats=6 RepeatLength=15 seed=TCCCCTTTTC Num.seqs=5 Similarity=0.768889
16   CTCCCCTTTTCGTAG
WB235_3:10436489-10436581 Satlength=93 Nr of Repeats=6 RepeatLength=15 seed=TCCCCTTTTC Num.seqs=5 Similarity=0.768889
16   CTCCCCTTTTCGTAG
WB235_4:628375-628467 Satlength=93 Nr of Repeats=6 RepeatLength=15 seed=TCCCCTTTTC Num.seqs=5 Similarity=0.768889
16   CTCCCCTTTTCGTAG
Rev.of_WB235_4:3050503-3050595 Satlength=93 Nr of Repeats=6 RepeatLength=15 seed=CGAAAAGGGG Num.seqs=5 Similarity=0.760000
27   CTCCCCTTTTCGTAG
WB235_4:3050770-3050862 Satlength=93 Nr of Repeats=6 RepeatLength=15 seed=TCCCCTTTTC Num.seqs=5 Similarity=0.768889
16   CTCCCCTTTTCGTAG
Rev.of_WB235_4:4401664-4401741 Satlength=78 Nr of Repeats=5 RepeatLength=15 seed=CGAAAAGGGG Num.seqs=4 Similarity=0.748148
27   CTCCCCTTTTCGTAG
WB235_4:4702342-4702434 Satlength=93 Nr of Repeats=6 RepeatLength=15 seed=TCCCCTTTTC Num.seqs=5 Similarity=0.768889
16   CTCCCCTTTTCGTAG
WB235_4:8016116-8016208 Satlength=93 Nr of Repeats=6 RepeatLength=15 seed=TCCCCTTTTC Num.seqs=5 Similarity=0.768889
16   CTCCCCTTTTCGTAG

Rev.of_WB235_4:11178974-11179067 Satlength=94 Nr of Repeats=6
RepeatLength=15 seed=CGAAAAGGGG Num.seqs=4 Similarity=0.748148
27   CTCCCCTTTTCGTAG
Rev.of_WB235_4:12621478-12621570 Satlength=93 Nr of Repeats=6
RepeatLength=15 seed=CGAAAAGGGG Num.seqs=5 Similarity=0.768889
27   CTCCCCTTTTCGTAG
Rev.of_WB235_4:15199553-15199645 Satlength=93 Nr of Repeats=6
RepeatLength=15 seed=CGAAAAGGGG Num.seqs=5 Similarity=0.768889
27   CTCCCCTTTTCGTAG
Rev.of_WB235_5:1197262-1197339 Satlength=78 Nr of Repeats=5
RepeatLength=15 seed=CGAAAAGGGG Num.seqs=4 Similarity=0.748148
27   CTCCCCTTTTCGTAG
WB235_5:1197514-1197589 Satlength=76 Nr of Repeats=5 RepeatLength=15
seed=TCCCCTTTTC Num.seqs=5 Similarity=0.768889
16   CTCCCCTTTTCGTAG
Rev.of_WB235_5:5754995-5755070 Satlength=76 Nr of Repeats=5
RepeatLength=15 seed=CGAAAAGGGG Num.seqs=5 Similarity=0.768889
27   CTCCCCTTTTCGTAG
WB235_5:5755255-5755347 Satlength=93 Nr of Repeats=6 RepeatLength=15
seed=TCCCCTTTTC Num.seqs=5 Similarity=0.733333
16   CTCCCCTTTTCGTAG
WB235_5:14444028-14444120 Satlength=93 Nr of Repeats=6 RepeatLength=15
seed=TCCCCTTTTC Num.seqs=5 Similarity=0.768889
16   CTCCCCTTTTCGTAG
Rev.of_WB235_5:15770931-15771023 Satlength=93 Nr of Repeats=6
RepeatLength=15 seed=CGAAAAGGGG Num.seqs=5 Similarity=0.768889
27   CTCCCCTTTTCGTAG
Rev.of_WB235_5:16552748-16552808 Satlength=61 Nr of Repeats=4
RepeatLength=15 seed=CGAAAAGGGG Num.seqs=4 Similarity=0.748148
27   CTCCCCTTTTCGTAG
WB235_4:17345179-17345256 Satlength=78 Nr of Repeats=5 RepeatLength=15
seed=TTCCCCTTTT Num.seqs=4 Similarity=0.866667
15   TTCCCCTTTTCGTAT
WB235_1:5621701-5621793 Satlength=93 Nr of Repeats=6 RepeatLength=15
seed=TCCCCTTTTC Num.seqs=5 Similarity=0.768889
16   ATCCCCTTTTCGCAG
WB235_2:14346519-14346611 Satlength=93 Nr of Repeats=6 RepeatLength=15
seed=TCCCCTTTTC Num.seqs=5 Similarity=0.768889
16   ATCCCCTTTTCGCAG
Rev.of_WB235_3:6013025-6013087 Satlength=63 Nr of Repeats=4
RepeatLength=15 seed=CGAAAAGGGG Num.seqs=3 Similarity=0.762963
27   ATCCCCTTTTCGCAG
WB235_3:6013262-6013339 Satlength=78 Nr of Repeats=5 RepeatLength=15
seed=TCCCCTTTTC Num.seqs=4 Similarity=0.748148
16   ATCCCCTTTTCGCAG
WB235_3:6551695-6551771 Satlength=77 Nr of Repeats=5 RepeatLength=15
seed=TCCCCTTTTC Num.seqs=4 Similarity=0.748148
16   ATCCCCTTTTCGCAG
Rev.of_WB235_3:7708654-7708716 Satlength=63 Nr of Repeats=4
RepeatLength=15 seed=CGAAAAGGGG Num.seqs=3 Similarity=0.762963
27   ATCCCCTTTTCGCAG
WB235_3:10123694-10123786 Satlength=93 Nr of Repeats=5 RepeatLength=15
seed=TCCCCTTTTC Num.seqs=3 Similarity=0.762963
16   ATCCCCTTTTCGCAG

Rev.of_WB235_3:10436267-10436329 Satlength=63 Nr of Repeats=4 RepeatLength=15 seed=CGAAAAGGGG Num.seqs=3 Similarity=0.762963
27   ATCCCCTTTTCGCAG
WB235_3:10811336-10811411 Satlength=76 Nr of Repeats=5 RepeatLength=15 seed=TCCCCTTTTC Num.seqs=5 Similarity=0.751111
16   ATCCCCTTTTCGCAG
WB235_3:12878285-12878362 Satlength=78 Nr of Repeats=5 RepeatLength=15 seed=TCCCCTTTTC Num.seqs=4 Similarity=0.777778
16   ATCCCCTTTTCGCAG
WB235_4:385463-385525 Satlength=63 Nr of Repeats=4 RepeatLength=15 seed=TCCCCTTTTC Num.seqs=3 Similarity=0.762963
16   ATCCCCTTTTCGCAG
Rev.of_WB235_4:1715069-1715131 Satlength=63 Nr of Repeats=4 RepeatLength=15 seed=CGAAAAGGGG Num.seqs=3 Similarity=0.703704
27   ATCCCCTTTTCGCAG
WB235_4:11179242-11179304 Satlength=63 Nr of Repeats=4 RepeatLength=15 seed=TCCCCTTTTC Num.seqs=3 Similarity=0.762963
16   ATCCCCTTTTCGCAG
WB235_4:15199820-15199897 Satlength=78 Nr of Repeats=5 RepeatLength=15 seed=TCCCCTTTTC Num.seqs=4 Similarity=0.748148
16   ATCCCCTTTTCGCAG
WB235_5:4553148-4553225 Satlength=78 Nr of Repeats=5 RepeatLength=15 seed=CCCCTTTTCG Num.seqs=4 Similarity=0.748148
17   ATCCCCTTTTCGCAG
WB235_5:16553018-16553153 Satlength=136 Nr of Repeats=7 RepeatLength=15 seed=GCAGATCCCC Num.seqs=6 Similarity=0.946667
26   ATCCCCTTTTCGCAG
Rev.of_WB235_5:20839492-20839569 Satlength=78 Nr of Repeats=5 RepeatLength=15 seed=CGAAAAGGGG Num.seqs=4 Similarity=0.822222
27   ATCCCCTTTTCGCAG
WB235_5:20839745-20839822 Satlength=78 Nr of Repeats=5 RepeatLength=15 seed=CCCCTTTTCG Num.seqs=4 Similarity=0.748148
17   ATCCCCTTTTCGCAG
Rev.of_WB235_6:256843-256905 Satlength=63 Nr of Repeats=4 RepeatLength=15 seed=CGAAAAGGGG Num.seqs=3 Similarity=0.762963
27   ATCCCCTTTTCGCAG
Rev.of_WB235_3:10123441-10123519 Satlength=79 Nr of Repeats=5 RepeatLength=15 seed=CGAAAAGGGG Num.seqs=3 Similarity=0.733333
27   ATCCCCTTTTCGNAG
WB235_6:257079-257156 Satlength=78 Nr of Repeats=5 RepeatLength=15 seed=TCCCCTTTTC Num.seqs=4 Similarity=0.762963
16   ATCCCCTTTTCGTAG
Rev.of_WB235_1:12899142-12899606 Satlength=465 Nr of Repeats=31 RepeatLength=15 seed=AAAAGGGGAT Num.seqs=28 Similarity=0.945444
25   ATCCCCTTTTTGCAG
Rev.of_WB235_1:7003581-7003641 Satlength=61 Nr of Repeats=4 RepeatLength=15 seed=CGAAAAGGGG Num.seqs=4 Similarity=0.807407
27   ATCCCCTTTTCGCTG

**   **** *
Consensus:

cTCCCCTTTTCGtAG

>WB235_Fam_8_16_50 Nr. of seq. 50 Alignment length(with gaps) = 16
Alignment score = 0.867364
WB235_1:11273722-11273834 Satlength=113 Nr of Repeats=7 RepeatLength=16
seed=CTACATGCCT Num.seqs=7 Similarity=0.805556
0 CTACATGCCTACATGC
WB235_1:11273722-11274842 Satlength=1121 Nr of Repeats=68 RepeatLength=16
seed=CTACATGCCT Num.seqs=64 Similarity=0.868304
0 CTACATGCCTACATGC
Rev.of_WB235_1:11656920-11657791 Satlength=872 Nr of Repeats=54
RepeatLength=16 seed=GCATGTAGGC Num.seqs=52 Similarity=0.946833
0 CTACATGCCTACATGC
WB235_2:12978404-12978548 Satlength=145 Nr of Repeats=9 RepeatLength=16
seed=GCCTACATGC Num.seqs=7 Similarity=0.861111
6 CTACATGCCTACATGC
Rev.of_WB235_2:13304324-13304580 Satlength=257 Nr of Repeats=15
RepeatLength=16 seed=GGCATGTAGG Num.seqs=13 Similarity=0.840812
1 CTACATGCCTACATGC
Rev.of_WB235_2:13310035-13310356 Satlength=322 Nr of Repeats=20
RepeatLength=16 seed=GGCATGTAGG Num.seqs=19 Similarity=0.773879
1 CTACATGCCTACATGC
WB235_2:13507307-13507403 Satlength=97 Nr of Repeats=6 RepeatLength=16
seed=ACATGCCTAC Num.seqs=6 Similarity=0.888889
2 CTACATGCCTACATGC
WB235_2:13631132-13631348 Satlength=217 Nr of Repeats=13 RepeatLength=16
seed=ACATGCCTAC Num.seqs=12 Similarity=0.958333
2 CTACATGCCTACATGC
Rev.of_WB235_3:3746895-3747055 Satlength=161 Nr of Repeats=10
RepeatLength=16 seed=TAGGCATGTA Num.seqs=10 Similarity=0.920370
3 CTACATGCCTACATGC
Rev.of_WB235_4:594389-594607 Satlength=219 Nr of Repeats=10
RepeatLength=16 seed=AGGCATGTAG Num.seqs=9 Similarity=0.796296
2 CTACATGCCTACATGC
Rev.of_WB235_4:2953071-2953413 Satlength=343 Nr of Repeats=19
RepeatLength=16 seed=TAGGCATGTA Num.seqs=16 Similarity=0.859028
3 CTACATGCCTACATGC
WB235_4:3184703-3185093 Satlength=391 Nr of Repeats=5 RepeatLength=16
seed=TACATGCCTA Num.seqs=4 Similarity=0.958333
1 CTACATGCCTACATGC
WB235_4:3189159-3189343 Satlength=185 Nr of Repeats=11 RepeatLength=16
seed=CATGCCTACA Num.seqs=10 Similarity=0.816667
3 CTACATGCCTACATGC
WB235_4:3193096-3194818 Satlength=1723 Nr of Repeats=67 RepeatLength=16
seed=ATGCCTACAT Num.seqs=46 Similarity=0.819565
4 CTACATGCCTACATGC
Rev.of_WB235_4:3211749-3211845 Satlength=97 Nr of Repeats=6
RepeatLength=16 seed=AGGCATGTAG Num.seqs=6 Similarity=0.872222
2 CTACATGCCTACATGC
WB235_4:3568013-3568493 Satlength=481 Nr of Repeats=28 RepeatLength=16
seed=TGCCTACATG Num.seqs=23 Similarity=0.914032
5 CTACATGCCTACATGC
Rev.of_WB235_4:3825814-3826008 Satlength=195 Nr of Repeats=12
RepeatLength=16 seed=TAGGCATGTA Num.seqs=10 Similarity=0.983333
3 CTACATGCCTACATGC

WB235_5:335210-335330 Satlength=121 Nr of Repeats=7 RepeatLength=16 seed=CATGCCTACA Num.seqs=6 Similarity=0.972222
3   CTACATGCCTACATGC
Rev.of_WB235_5:342974-343198 Satlength=225 Nr of Repeats=14 RepeatLength=16 seed=GGCATGTAGG Num.seqs=14 Similarity=0.928571
1   CTACATGCCTACATGC
WB235_5:777315-777395 Satlength=81 Nr of Repeats=5 RepeatLength=16 seed=CTACATGCCT Num.seqs=5 Similarity=0.933333
0   CTACATGCCTACATGC
Rev.of_WB235_5:777834-778359 Satlength=526 Nr of Repeats=12 RepeatLength=16 seed=AGGCATGTAG Num.seqs=9 Similarity=0.907407
2   CTACATGCCTACATGC
Rev.of_WB235_5:779287-779455 Satlength=169 Nr of Repeats=10 RepeatLength=16 seed=AGGCATGTAG Num.seqs=9 Similarity=0.944444
2   CTACATGCCTACATGC
WB235_5:885274-885474 Satlength=201 Nr of Repeats=12 RepeatLength=16 seed=ATGCCTACAT Num.seqs=11 Similarity=0.956061
4   CTACATGCCTACATGC
WB235_5:2164618-2164730 Satlength=113 Nr of Repeats=6 RepeatLength=16 seed=ATGCCTACAT Num.seqs=4 Similarity=0.875000
4   CTACATGCCTACATGC
Rev.of_WB235_5:4784597-4784789 Satlength=193 Nr of Repeats=11 RepeatLength=16 seed=TAGGCATGTA Num.seqs=9 Similarity=0.944444
3   CTACATGCCTACATGC
Rev.of_WB235_5:3085214-3085707 Satlength=494 Nr of Repeats=4 RepeatLength=16 seed=GCATGTAGGC Num.seqs=3 Similarity=0.944444
0   CTACAAGCCTACATGC
WB235_3:11475344-11475488 Satlength=145 Nr of Repeats=7 RepeatLength=16 seed=TACAAGCCTA Num.seqs=5 Similarity=0.866667
1   CTACAAGCCTACAAGC
WB235_2:674091-674259 Satlength=169 Nr of Repeats=9 RepeatLength=16 seed=CCTACGTGCC Num.seqs=6 Similarity=0.844444
7   CTACGTGCCTACGTGC
Rev.of_WB235_2:2761987-2762171 Satlength=185 Nr of Repeats=11 RepeatLength=16 seed=CACGTAGGCA Num.seqs=10 Similarity=0.966667
7   CTACGTGCCTACGTGC
Rev.of_WB235_2:3124197-3124357 Satlength=161 Nr of Repeats=9 RepeatLength=16 seed=TAGGCACGTA Num.seqs=7 Similarity=0.849206
11   CTACGTGCCTACGTGC
WB235_2:3125607-3125959 Satlength=353 Nr of Repeats=18 RepeatLength=16 seed=TACGTGCCTA Num.seqs=12 Similarity=0.845960
9   CTACGTGCCTACGTGC
Rev.of_WB235_2:3179723-3180290 Satlength=568 Nr of Repeats=21 RepeatLength=16 seed=GTAGGCACGT Num.seqs=18 Similarity=0.936819
12   CTACGTGCCTACGTGC
Rev.of_WB235_2:3271556-3272244 Satlength=689 Nr of Repeats=42 RepeatLength=16 seed=CGTAGGCACG Num.seqs=38 Similarity=0.966809
13   CTACGTGCCTACGTGC
Rev.of_WB235_2:3420081-3420246 Satlength=166 Nr of Repeats=5 RepeatLength=16 seed=GCACGTAGGC Num.seqs=3 Similarity=0.944444
8   CTACGTGCCTACGTGC
Rev.of_WB235_2:3512519-3512671 Satlength=153 Nr of Repeats=9 RepeatLength=16 seed=GTAGGCACGT Num.seqs=8 Similarity=0.958333
12   CTACGTGCCTACGTGC

```
WB235_2:4030891-4031711 Satlength=821 Nr of Repeats=4 RepeatLength=16
seed=TGCCTACGTG  Num.seqs=3 Similarity=1.000000
13    CTACGTGCCTACGTGC
Rev.of_WB235_2:13061749-13061893 Satlength=145 Nr of Repeats=8
RepeatLength=16 seed=TAGGCACGTA  Num.seqs=6 Similarity=0.916667
11    CTACGTGCCTACGTGC
Rev.of_WB235_3:8334779-8334979 Satlength=201 Nr of Repeats=12
RepeatLength=16 seed=AGGCACGTAG  Num.seqs=11 Similarity=0.887879
10    CTACGTGCCTACGTGC
Rev.of_WB235_3:10480990-10481350 Satlength=361 Nr of Repeats=14
RepeatLength=16 seed=TAGGCACGTA  Num.seqs=13 Similarity=0.961538
11    CTACGTGCCTACGTGC
Rev.of_WB235_3:12190449-12190847 Satlength=399 Nr of Repeats=13
RepeatLength=16 seed=ACGTAGGCAC  Num.seqs=10 Similarity=0.916667
14    CTACGTGCCTACGTGC
Rev.of_WB235_4:1762607-1762703 Satlength=97 Nr of Repeats=5
RepeatLength=16 seed=CACGTAGGCA  Num.seqs=4 Similarity=0.916667
7    CTACGTGCCTACGTGC
Rev.of_WB235_4:1787801-1788241 Satlength=441 Nr of Repeats=26
RepeatLength=16 seed=TAGGCACGTA  Num.seqs=23 Similarity=0.944005
11    CTACGTGCCTACGTGC
WB235_4:5060074-5060274 Satlength=201 Nr of Repeats=12 RepeatLength=16
seed=CTACGTGCCT  Num.seqs=11 Similarity=0.969697
8    CTACGTGCCTACGTGC
WB235_4:12326778-12327098 Satlength=321 Nr of Repeats=18 RepeatLength=16
seed=GTGCCTACGT  Num.seqs=14 Similarity=0.887363
12    CTACGTGCCTACGTGC
Rev.of_WB235_2:3152725-3152861 Satlength=137 Nr of Repeats=8
RepeatLength=16 seed=AGGCACGTAG  Num.seqs=7 Similarity=0.853175
10    CTACGTGCCTACNTGC
Rev.of_WB235_2:14108714-14108890 Satlength=177 Nr of Repeats=10
RepeatLength=16 seed=TAGGCATATA  Num.seqs=9 Similarity=0.842593
3    CTATATGCCTATATGC
WB235_3:3647083-3647155 Satlength=73 Nr of Repeats=4 RepeatLength=16
seed=CCTACATACC  Num.seqs=3 Similarity=0.888889
7    CTACATACCTACATAC
WB235_5:16699106-16699538 Satlength=433 Nr of Repeats=26 RepeatLength=16
seed=ACATACCTAC  Num.seqs=24 Similarity=0.960145
10    CTACATACCTACATAC
Rev.of_WB235_3:206747-206867 Satlength=121 Nr of Repeats=7
RepeatLength=16 seed=TAGGCATCTA  Num.seqs=6 Similarity=0.827778
3    CTAGGTGCATAGATGC
Rev.of_WB235_4:2981693-2981773 Satlength=81 Nr of Repeats=4
RepeatLength=16 seed=AGGTAGGAAG  Num.seqs=3 Similarity=0.722222
2    CTACCTACCTTCCTAC

***    * *     *
Consensus:

CTACaTGCCTACaTGC

>WB235_Fam_9_20_46  Nr. of seq. 46 Alignment length(with gaps) = 20
Alignment score = 0.797488
```

WB235_1:2542918-2543982 Satlength=1065 Nr of Repeats=56 RepeatLength=19
seed=TGTTACACGA Num.seqs=56 Similarity=0.965915
0   TGTTACACG-AAAAATAGAT
Rev.of_WB235_1:4465416-4465569 Satlength=154 Nr of Repeats=8
RepeatLength=19 seed=TTTTTCGTGT Num.seqs=7 Similarity=0.900000
14   TGTTACACG-AAAAATAGAT
Rev.of_WB235_1:14128159-14128469 Satlength=311 Nr of Repeats=16
RepeatLength=19 seed=AACAATCTAT Num.seqs=10 Similarity=1.000000
4   TGTTACACG-AAAAATAGAT
WB235_3:3987076-3987676 Satlength=601 Nr of Repeats=31 RepeatLength=19
seed=AAAAATAGAT Num.seqs=20 Similarity=1.000000
9   TGTTACACG-AAAAATAGAT
WB235_3:6185212-6186090 Satlength=879 Nr of Repeats=42 RepeatLength=19
seed=AAAAATAGAT Num.seqs=38 Similarity=0.969155
9   TGTTACACG-AAAAATAGAT
Rev.of_WB235_3:7668665-7669197 Satlength=533 Nr of Repeats=28
RepeatLength=19 seed=AATCTATTTT Num.seqs=28 Similarity=0.920040
1   TGTTACACG-AAAAATAGAT
Rev.of_WB235_3:7678163-7678429 Satlength=267 Nr of Repeats=14
RepeatLength=19 seed=CAATCTATTT Num.seqs=14 Similarity=0.974552
2   TGTTACACG-AAAAATAGAT
WB235_3:13243432-13245239 Satlength=1808 Nr of Repeats=95 RepeatLength=19
seed=AAAAATAGAT Num.seqs=93 Similarity=0.938765
9   TGTTACACG-AAAAATAGAT
WB235_4:10681773-10682153 Satlength=381 Nr of Repeats=20 RepeatLength=19
seed=ATTGTTACAC Num.seqs=20 Similarity=0.974146
17   TGTTACACG-AAAAATAGAT
WB235_4:16963408-16964890 Satlength=1483 Nr of Repeats=77 RepeatLength=19
seed=AAAAATAGAT Num.seqs=58 Similarity=0.957844
9   TGTTACACG-AAAAATAGAT
Rev.of_WB235_5:6937435-6937626 Satlength=192 Nr of Repeats=10
RepeatLength=19 seed=AACAATCTAT Num.seqs=9 Similarity=1.000000
4   TGTTACACG-AAAAATAGAT
WB235_5:13365913-13366445 Satlength=533 Nr of Repeats=28 RepeatLength=19
seed=GTTACACGAA Num.seqs=28 Similarity=0.981064
1   TGTTACACG-AAAAATAGAT
Rev.of_WB235_5:17412072-17412375 Satlength=304 Nr of Repeats=16
RepeatLength=19 seed=TTTTCGTGTA Num.seqs=15 Similarity=0.934503
13   TGTTACACG-AAAAATAGAT
Rev.of_WB235_6:3007216-3008016 Satlength=801 Nr of Repeats=42
RepeatLength=19 seed=GTGTAACAAT Num.seqs=31 Similarity=0.982192
8   TGTTACACG-AAAAATAGAT
WB235_6:12276123-12276239 Satlength=117 Nr of Repeats=6 RepeatLength=19
seed=ATAGATTGTT Num.seqs=4 Similarity=1.000000
13   TGTTACACG-AAAAATAGAT
WB235_6:12502706-12502822 Satlength=117 Nr of Repeats=6 RepeatLength=19
seed=AAAAATAGAT Num.seqs=4 Similarity=0.894737
9   TGTTACACG-AAAAATAGAT
WB235_4:16244112-16245385 Satlength=1274 Nr of Repeats=65 RepeatLength=19
seed=AAATAGATTG Num.seqs=45 Similarity=0.932093
11   TGTTACACG-GAAAATAGAT
WB235_1:11357272-11357373 Satlength=102 Nr of Repeats=5 RepeatLength=20
seed=AATAGATTGT Num.seqs=4 Similarity=0.933333
13   TGTTACACGAAAAAATAGAT

```
WB235_1:13276960-13277319 Satlength=360 Nr of Repeats=18 RepeatLength=20
seed=AAAATAGATT  Num.seqs=17 Similarity=0.992157
31    TGTTACACGAAAAAAATAGAT
WB235_3:10796467-10796566 Satlength=100 Nr of Repeats=5 RepeatLength=20
seed=GTTACACGAA  Num.seqs=4 Similarity=1.000000
21    TGTTACACGAAAAAAATAGAT
WB235_4:15859222-15859379 Satlength=158 Nr of Repeats=8 RepeatLength=20
seed=TGTTACACGA  Num.seqs=5 Similarity=1.000000
20    TGTTACACGAAAAAAATAGAT
WB235_5:18400360-18401785 Satlength=1426 Nr of Repeats=72 RepeatLength=20
seed=AAAAATAGAT  Num.seqs=57 Similarity=0.937761
30    TGTTACACGAAAAAAATAGAT
WB235_5:18727688-18728599 Satlength=912 Nr of Repeats=46 RepeatLength=20
seed=AAAAATAGAT  Num.seqs=37 Similarity=1.000000
30    TGTTACACGAAAAAAATAGAT
WB235_6:1945521-1946157 Satlength=637 Nr of Repeats=32 RepeatLength=20
seed=GTTACACGAA  Num.seqs=28 Similarity=1.000000
21    TGTTACACGAAAAAAATAGAT
WB235_6:1954050-1954308 Satlength=259 Nr of Repeats=13 RepeatLength=20
seed=GTTACACGAA  Num.seqs=11 Similarity=1.000000
21    TGTTACACGAAAAAAATAGAT
Rev.of_WB235_6:14154417-14154672 Satlength=256 Nr of Repeats=13
RepeatLength=20 seed=TAACAATCTA  Num.seqs=8 Similarity=0.966667
25    TGTTACACGAAAAAAATAGAT
Rev.of_WB235_6:15811201-15811280 Satlength=80 Nr of Repeats=4
RepeatLength=20 seed=TAACAATCTA  Num.seqs=3 Similarity=0.955556
25    TGTTACACGAAAAAAATAGAT
WB235_2:3994649-3994788 Satlength=140 Nr of Repeats=7 RepeatLength=20
seed=AAATAGATTG  Num.seqs=6 Similarity=0.964444
32    TGTTACACTAAAAAAATAGAT
WB235_4:6210092-6210227 Satlength=136 Nr of Repeats=7 RepeatLength=19
seed=ATTGACAGAC  Num.seqs=5 Similarity=0.971930
17    TGACAGACG-AAAAATAGAT
WB235_4:12584972-12585067 Satlength=96 Nr of Repeats=5 RepeatLength=19
seed=AAAAATAGAT  Num.seqs=5 Similarity=0.971930
28    TGACAGACG-AAAAATAGAT
Rev.of_WB235_5:3098854-3099539 Satlength=686 Nr of Repeats=35
RepeatLength=19 seed=AATCTATTTT  Num.seqs=24 Similarity=0.944826
20    TGACAGACG-AAAAATAGAT
WB235_5:5646992-5647147 Satlength=156 Nr of Repeats=8 RepeatLength=19
seed=AAAAATAGAT  Num.seqs=5 Similarity=1.000000
28    TGGCAGACG-AAAAATAGAT
WB235_3:1260242-1261618 Satlength=1377 Nr of Repeats=70 RepeatLength=20
seed=AAAAATAGAT  Num.seqs=42 Similarity=0.907822
29    TGACAGACGAAAAAAATAGAT
WB235_3:12009165-12009381 Satlength=217 Nr of Repeats=11 RepeatLength=20
seed=AAATAGATTG  Num.seqs=7 Similarity=0.904762
31    TGACAGACGAAAAAAATAGAT
WB235_6:4285394-4285533 Satlength=140 Nr of Repeats=7 RepeatLength=20
seed=ACAGACGAAA  Num.seqs=6 Similarity=0.937778
41    TGACAGACGAAAAAAATAGAT
Rev.of_WB235_6:7081038-7081216 Satlength=179 Nr of Repeats=9
RepeatLength=20 seed=TTTTTCGTCT  Num.seqs=7 Similarity=0.961905
33    TGACAGACGAAAAAAATAGAT
```

WB235_6:7895537-7895652 Satlength=116 Nr of Repeats=6 RepeatLength=19
seed=CAGACGAAAA Num.seqs=5 Similarity=0.887719
22   TGGCAGACG-AAAAATATAT
WB235_1:3485156-3487576 Satlength=2421 Nr of Repeats=121 RepeatLength=20
seed=AAAAAATAGA Num.seqs=121 Similarity=0.970083
28   TGACATACCAAAAAATAGAT
WB235_1:13925940-13926100 Satlength=161 Nr of Repeats=8 RepeatLength=20
seed=AAAAAATAGA Num.seqs=8 Similarity=0.900000
28   TGACATACCAAAAAATAGAT
WB235_3:9940544-9940665 Satlength=122 Nr of Repeats=6 RepeatLength=20
seed=TGACATACCA Num.seqs=5 Similarity=0.973333
39   TGACATACCAAAAAATAGAT
Rev.of_WB235_3:11004687-11006367 Satlength=1681 Nr of Repeats=84
RepeatLength=20 seed=TCTATTTTTT Num.seqs=84 Similarity=0.960031
38   TGACATACCAAAAAATAGAT
WB235_6:3924546-3924685 Satlength=140 Nr of Repeats=7 RepeatLength=20
seed=AAAATAGATT Num.seqs=6 Similarity=0.955556
30   TGACATACCAAAAAATAGAT
Rev.of_WB235_2:887413-887570 Satlength=158 Nr of Repeats=8
RepeatLength=20 seed=CAATCTATTT Num.seqs=6 Similarity=0.902222
41   TGNCATACCAAAAAATAGAT
WB235_6:15806999-15807439 Satlength=441 Nr of Repeats=22 RepeatLength=20
seed=CATACCAAAA Num.seqs=22 Similarity=0.934488
42   TGGCATACCAAAAAATAGAT
WB235_2:11423462-11423661 Satlength=200 Nr of Repeats=9 RepeatLength=20
seed=AAAAAATAGA Num.seqs=8 Similarity=0.892857
28   TGGCATACGAAAAAATAGAT
WB235_1:14321889-14321969 Satlength=81 Nr of Repeats=4 RepeatLength=20
seed=ATATCAAAAA Num.seqs=4 Similarity=0.855556
43   TGGCATATCAAAAAATAAAT

** * *   ****** **
Consensus:

TGttAcACGaAAAAATAGAT

>WB235_Fam_10_25_40 Nr. of seq. 40 Alignment length(with gaps) = 28
Alignment score = 0.748245
WB235_1:3763157-3765957 Satlength=2801 Nr of Repeats=112 RepeatLength=25
seed=TTTACTCTCT Num.seqs=112 Similarity=0.895307
0   TTTACTCTCT--GGC-TTCACCAAATAT
Rev.of_WB235_6:10361494-10369166 Satlength=7673 Nr of Repeats=299
RepeatLength=25 seed=GTGAAGCCAG Num.seqs=291 Similarity=0.913572
18   TTTACTCTCT--GGC-TTCACCAAATAT
Rev.of_WB235_2:5144410-5146766 Satlength=2357 Nr of Repeats=80
RepeatLength=25 seed=AGCCAGAGAG Num.seqs=74 Similarity=0.877056
14   TTTACTCTCT--GGC-TTCACCAATTAT
Rev.of_WB235_3:1299759-1303117 Satlength=3359 Nr of Repeats=134
RepeatLength=25 seed=CAGAGAGTAA Num.seqs=126 Similarity=0.920857
36   TTTACTCTCT--GGC-TTCACCAATTAT
Rev.of_WB235_2:4259952-4260552 Satlength=601 Nr of Repeats=24
RepeatLength=25 seed=GAGAGTAAAA Num.seqs=24 Similarity=0.932560
9   TTTACTCTCT--GGC-TTCACCGTATAT

WB235_4:17492409-17493609 Satlength=1201 Nr of Repeats=48 RepeatLength=25 seed=ACTCTCTGGC Num.seqs=48 Similarity=0.888227
28 TTTACTCTCT--GGC-TTCACCGTATAT
Rev.of_WB235_3:2497037-2501716 Satlength=4680 Nr of Repeats=187 RepeatLength=25 seed=GTGAAGCCAG Num.seqs=184 Similarity=0.887060
18 TTTACTCTCT--GGC-TTCACCATATAT
WB235_2:9404845-9405392 Satlength=548 Nr of Repeats=22 RepeatLength=25 seed=GGCTTCACAG Num.seqs=20 Similarity=0.925053
10 TTTACTCTCT--GGC-TTCACAGTATAT
WB235_4:5299099-5299474 Satlength=376 Nr of Repeats=15 RepeatLength=25 seed=TTTACTCTCT Num.seqs=15 Similarity=0.890286
25 TTTACTCTCT--GGC-TTCACAGTATAT
Rev.of_WB235_5:7993172-7993472 Satlength=301 Nr of Repeats=12 RepeatLength=25 seed=CCAGAGAGTA Num.seqs=12 Similarity=0.873131
12 TTTACTCTCT--GGC-TTCACAGTATAT
WB235_5:10449424-10450674 Satlength=1251 Nr of Repeats=50 RepeatLength=25 seed=TTTACTCTCT Num.seqs=50 Similarity=0.898601
25 TTTACTCTCT--GGC-TTCACAGTATAT
Rev.of_WB235_6:9184992-9188750 Satlength=3759 Nr of Repeats=150 RepeatLength=25 seed=CAGAGAGTAA Num.seqs=149 Similarity=0.922331
11 TTTACTCTCT--GGC-TTCACAGTATAT
WB235_6:2055472-2055647 Satlength=176 Nr of Repeats=7 RepeatLength=25 seed=TATATTTTAC Num.seqs=7 Similarity=0.852698
20 TTTACTCTCT--GGC-TTCACANTATAT
WB235_6:2056950-2057275 Satlength=326 Nr of Repeats=12 RepeatLength=25 seed=TCTGGCTTCA Num.seqs=11 Similarity=0.885576
32 TTTACTCTCT--GGC-TTCACACTATAT
WB235_6:14908166-14909416 Satlength=1251 Nr of Repeats=49 RepeatLength=25 seed=TGGCTTCACA Num.seqs=48 Similarity=0.906856
34 TTTACTCTCT--GGC-TTCACAATATAT
WB235_6:11886530-11888254 Satlength=1725 Nr of Repeats=68 RepeatLength=25 seed=TTCACCATAT Num.seqs=64 Similarity=0.956852
13 TTCACTCTCT--GGC-TTCACCATATAT
Rev.of_WB235_1:6771968-6773943 Satlength=1976 Nr of Repeats=79 RepeatLength=25 seed=GTGAAGCCAG Num.seqs=69 Similarity=0.904109
18 TTTACTCTCT--GGC-TTCACAGAATAA
WB235_4:6090427-6090577 Satlength=151 Nr of Repeats=6 RepeatLength=25 seed=TTTACTCTCT Num.seqs=6 Similarity=0.882667
25 TTTACTCTCT--GGC-TTCACAGAATAA
Rev.of_WB235_5:14750559-14751884 Satlength=1326 Nr of Repeats=53 RepeatLength=25 seed=TGTGAAGCCA Num.seqs=53 Similarity=0.918336
19 TTTACTCTCT--GGC-TTCACAGAATAA
WB235_4:13360500-13361979 Satlength=1480 Nr of Repeats=59 RepeatLength=25 seed=TTTACTCTCT Num.seqs=56 Similarity=0.877229
25 TTTACTCTCT--GGC-TTCACAGAATAT
Rev.of_WB235_5:4333269-4336644 Satlength=3376 Nr of Repeats=135 RepeatLength=25 seed=AGAGTAAAAT Num.seqs=135 Similarity=0.922491
33 TTTACTCTCT--GGC-TTCACAGAATAT
WB235_6:13981254-13981504 Satlength=251 Nr of Repeats=10 RepeatLength=25 seed=TCTCTGGCTT Num.seqs=10 Similarity=0.913481
30 TTTACTCTCT--GGC-TTCACAGAATAT
WB235_2:12844903-12846136 Satlength=1234 Nr of Repeats=49 RepeatLength=25 seed=GGCTTCCCAC Num.seqs=45 Similarity=0.990626
10 TTTACTCTCT--GGC-TTCCCACTATAT

```
WB235_1:933478-934899 Satlength=1422 Nr of Repeats=52 RepeatLength=27
seed=TATTTTACTC  Num.seqs=46 Similarity=0.859742
22    TTTACTCTCTGTGGC-TTCCCACTATAT
WB235_2:3290648-3290783 Satlength=136 Nr of Repeats=5 RepeatLength=27
seed=TTTACTCTCT  Num.seqs=5 Similarity=1.000000
25    TTTACTCTCTGTGGC-TTCCCACTATAT
WB235_2:14337687-14339240 Satlength=1554 Nr of Repeats=57 RepeatLength=27
seed=GGCTTCCCAC  Num.seqs=48 Similarity=0.918571
37    TTTACTCTCTGTGGC-TTCCCACTATAT
Rev.of_WB235_3:931250-931684 Satlength=435 Nr of Repeats=16
RepeatLength=27 seed=AGAGAGTAAA  Num.seqs=15 Similarity=0.926631
35    TTTACTCTCTGTGGC-TTCCCACTATAT
Rev.of_WB235_3:7444445-7448204 Satlength=3760 Nr of Repeats=143
RepeatLength=27 seed=ACAGAGAGTA  Num.seqs=126 Similarity=0.934432
37    TTTACTCTCTGTGGC-TTCCCACTATAT
Rev.of_WB235_6:109717-114226 Satlength=4510 Nr of Repeats=167
RepeatLength=27 seed=CACAGAGAGT  Num.seqs=167 Similarity=0.846944
38    TTTACTCTCTGTGGC-TTCCCACTATAT
WB235_6:5056273-5057278 Satlength=1006 Nr of Repeats=38 RepeatLength=27
seed=TATTTTACTC  Num.seqs=31 Similarity=0.933897
22    TTTACTCTCTGTGGC-TTCCCACTATAT
WB235_5:14754513-14757536 Satlength=3024 Nr of Repeats=84 RepeatLength=27
seed=CTGTGGCTTC  Num.seqs=77 Similarity=0.911293
33    TTTACTCTCTGTGGC-TTCCCACCATAT
WB235_6:1752278-1755168 Satlength=2891 Nr of Repeats=107 RepeatLength=27
seed=ACTCTCTGTG  Num.seqs=106 Similarity=0.943527
55    TTTACTCTCTGTGGC-TTCCCACCATAT
Rev.of_WB235_2:12843822-12844875 Satlength=1054 Nr of Repeats=39
RepeatLength=27 seed=GTGGGAAGCC  Num.seqs=39 Similarity=0.975075
47    TTTACTCTCTATGGC-TTCCCACTATAT
Rev.of_WB235_5:14768011-14769734 Satlength=1724 Nr of Repeats=59
RepeatLength=27 seed=GGAAGCCACA  Num.seqs=57 Similarity=0.946781
17    TTTACTCTCTGTGGC-TTCCAACCATAT
WB235_5:1104557-1105016 Satlength=460 Nr of Repeats=17 RepeatLength=27
seed=TTACTCTCTG  Num.seqs=17 Similarity=0.900145
26    TTTACTCTCTGTGGC-TTCACAGTATAT
WB235_3:3334358-3337083 Satlength=2726 Nr of Repeats=109 RepeatLength=25
seed=ACTCTCTGGC  Num.seqs=109 Similarity=0.899274
28    TTTACTCTCT--GGC-TTCCCATCATAT
Rev.of_WB235_4:5878242-5878392 Satlength=151 Nr of Repeats=6
RepeatLength=25 seed=CCAGAGAGTA  Num.seqs=6 Similarity=0.875556
37    TTTACTCTCT--GGT-TTCCCAGTATAT
Rev.of_WB235_2:5144313-5144565 Satlength=253 Nr of Repeats=9
RepeatLength=25 seed=AGAGTAAAAT  Num.seqs=6 Similarity=0.823932
8    TTTACTCTCT--GGCTTTCCCAAT-TAT
WB235_1:4544868-4547696 Satlength=2829 Nr of Repeats=104 RepeatLength=27
seed=ACTCTCTGTG  Num.seqs=100 Similarity=0.845700
3    TTTACTCTCTGTGGC-TTCACCGATNAT
Rev.of_WB235_4:2828548-2830536 Satlength=1989 Nr of Repeats=74
RepeatLength=27 seed=CAGAGAGTAA  Num.seqs=69 Similarity=0.869860
11    TTTACTCTCTGTGGC-TTCACCAATTAT

** *******  **  ***       *
Consensus:
```

TTTACTCTCTGGCTTCaCantATAT

>WB235_Fam_11_20_29 Nr. of seq. 29 Alignment length(with gaps) = 25
Alignment score = 0.605057
WB235_1:354705-355291 Satlength=587 Nr of Repeats=29 RepeatLength=19
seed=TATCGATTTT Num.seqs=25 Similarity=0.914854
0   TATCGATTTTT-C---TG-G-AATT
Rev.of_WB235_5:19026746-19027590 Satlength=845 Nr of Repeats=33
RepeatLength=20 seed=AAAATCGATA Num.seqs=22 Similarity=0.814643
10   TATCGATTTTT-C---TG-GAAATT
WB235_5:18984459-18985309 Satlength=851 Nr of Repeats=31 RepeatLength=20
seed=ATTTATTGAT Num.seqs=20 Similarity=0.886667
17   TATCGATTTTT-C---TG-GAAATT
WB235_5:19112578-19114304 Satlength=1727 Nr of Repeats=66 RepeatLength=20
seed=TATCGATTTT Num.seqs=59 Similarity=0.867212
20   TATCGATTTTT-C---TG-GAAATT
WB235_5:18993608-18993851 Satlength=244 Nr of Repeats=8 RepeatLength=20
seed=TATCGATTTT Num.seqs=5 Similarity=0.900000
20   TATCGATTTTT-C---TG-CAAATT
WB235_5:19909526-19910097 Satlength=572 Nr of Repeats=12 RepeatLength=20
seed=TTATTGATTT Num.seqs=8 Similarity=0.876190
19   TATTGATTTTT-A---TG-GAAATT
Rev.of_WB235_1:1815369-1815789 Satlength=421 Nr of Repeats=18
RepeatLength=19 seed=AAAATCAATA Num.seqs=14 Similarity=0.796414
10   TATTGATTTT--C---TT-AGAATT
Rev.of_WB235_1:1815369-1817144 Satlength=1776 Nr of Repeats=44
RepeatLength=19 seed=AAAATCAATA Num.seqs=32 Similarity=0.769949
10   TATTGATTTT--C---TN-NGAATT
Rev.of_WB235_4:1740517-1740917 Satlength=401 Nr of Repeats=20
RepeatLength=20 seed=CAGAAAAATC Num.seqs=20 Similarity=0.835088
14   TATTGATTTTT-C---TG-AGAATT
WB235_5:18632123-18632283 Satlength=161 Nr of Repeats=8 RepeatLength=20
seed=GATTTTTCTG Num.seqs=8 Similarity=0.871429
24   TATTGATTTTT-C---TG-AAAATT
Rev.of_WB235_5:18675402-18675828 Satlength=427 Nr of Repeats=12
RepeatLength=20 seed=AGAAAAATCA Num.seqs=8 Similarity=0.902381
33   TATTGATTTTT-C---TG-AAAATT
Rev.of_WB235_5:18986276-18986397 Satlength=122 Nr of Repeats=6
RepeatLength=20 seed=ATAAATTTTC Num.seqs=5 Similarity=0.960000
43   TATTGATTTTT-C---TG-AAAATT
Rev.of_WB235_5:19099300-19100374 Satlength=1075 Nr of Repeats=31
RepeatLength=20 seed=AAAAATCAAT Num.seqs=22 Similarity=0.750066
31   TATTGATTTTT-C---TG-AAAATT
WB235_5:19100422-19100868 Satlength=447 Nr of Repeats=10 RepeatLength=20
seed=ATTGATTTTT Num.seqs=8 Similarity=0.861905
41   TATTGATTTTT-C---TG-AAAATT
WB235_5:19129872-19130673 Satlength=802 Nr of Repeats=34 RepeatLength=20
seed=GATTTTTCTG Num.seqs=28 Similarity=0.875661
24   TATTGATTTTT-C---TG-AAAATT
Rev.of_WB235_5:19101640-19102041 Satlength=402 Nr of Repeats=17
RepeatLength=20 seed=CAGAAAAATC Num.seqs=12 Similarity=0.905051
14   TATTGATTTTT-C---TG-GAAATT

```
WB235_5:19901870-19902110 Satlength=241 Nr of Repeats=10 RepeatLength=20
seed=TTATTGATTT  Num.seqs=8 Similarity=0.866667
19    TATTGATTTTT-C---TG-GAAATT
Rev.of_WB235_5:19907460-19907660 Satlength=201 Nr of Repeats=9
RepeatLength=20 seed=CAGAAAAATC  Num.seqs=8 Similarity=0.873810
14    TATTGATTTTT-C---TG-GAAATT
WB235_5:19121195-19121753 Satlength=559 Nr of Repeats=25 RepeatLength=20
seed=AATTTATTGA  Num.seqs=16 Similarity=0.823333
16    TATTGATTTTT-T---TG-GAAATT
Rev.of_WB235_6:190303-190811 Satlength=509 Nr of Repeats=10
RepeatLength=20 seed=AAAAATCAAT  Num.seqs=9 Similarity=0.907407
31    TATTGATTTTT-N---TG-GAAATT
Rev.of_WB235_1:11485772-11485879 Satlength=108 Nr of Repeats=5
RepeatLength=19 seed=TCAAAAATCA  Num.seqs=4 Similarity=0.873611
12    -ATTGATTTTT-GA--TG--AAATT
Rev.of_WB235_1:11383715-11384049 Satlength=335 Nr of Repeats=14
RepeatLength=20 seed=AAAAATCAAT  Num.seqs=11 Similarity=0.883636
29    -ATTGATTTTTCGA--TG--AAATT
Rev.of_WB235_1:11485735-11486997 Satlength=1263 Nr of Repeats=20
RepeatLength=19 seed=AAAAATCGAT  Num.seqs=12 Similarity=0.725830
29    -ATCGATTTTT-GA--NG--AAATT
Rev.of_WB235_2:2623068-2623182 Satlength=115 Nr of Repeats=6
RepeatLength=19 seed=AAAATCGATA  Num.seqs=6 Similarity=0.601058
47    -ATCGATTTTC-GA--TG--AAATT
Rev.of_WB235_1:11532642-11533117 Satlength=476 Nr of Repeats=17
RepeatLength=19 seed=TTTCACAAAA  Num.seqs=12 Similarity=0.943647
17    -ATTGATTTTT-G---TGA-AAATT
Rev.of_WB235_1:11531919-11534041 Satlength=2123 Nr of Repeats=45
RepeatLength=19 seed=AAAATCGATA  Num.seqs=29 Similarity=0.730706
29    -ATCGATTTTT-N---TGA-AAATT
Rev.of_WB235_4:977829-978532 Satlength=704 Nr of Repeats=35
RepeatLength=19 seed=CAATAATTTC  Num.seqs=27 Similarity=0.905233
24    -ATTGATTTTT-T-G-GG--AAATT
WB235_1:11384374-11386353 Satlength=1980 Nr of Repeats=95 RepeatLength=20
seed=ATTGATTTTT  Num.seqs=91 Similarity=0.879707
40    -ATTGATTTTT-T-GAGG--AAATT
WB235_1:2364706-2365240 Satlength=535 Nr of Repeats=20 RepeatLength=20
seed=TTATTGATTT  Num.seqs=12 Similarity=0.816162
38    -ATTGATTTTT-C-TAGG--ACATT

** ******            ***
Consensus:

tATtGATTTTTcTGnAAATT

>WB235_Fam_12_45_28  Nr. of seq. 28 Alignment length(with gaps) = 46
Alignment score = 0.807319
WB235_2:11230254-11230477 Satlength=224 Nr of Repeats=5 RepeatLength=44
seed=AAGGATCAGA  Num.seqs=3 Similarity=0.696296
0    AAGGATCAGAACATCA-GGAAG-AGCCTCGTGATCCTTTGGAGCTG
Rev.of_WB235_1:3553785-3554055 Satlength=271 Nr of Repeats=6
RepeatLength=45 seed=CTGAATCTTC  Num.seqs=6 Similarity=0.990123
9    AAGATTCAGATCATAAAGGAGG-ATCCTTGTGATCCTTTGGAGCTG
```

WB235_5:18314435-18314705 Satlength=271 Nr of Repeats=6 RepeatLength=45 seed=TTTGGAGCTG Num.seqs=6 Similarity=0.928889
35   AAGATTCAGTTCATAAGGGGGG-ACCCTTATGATCCTTTGGAGCTG
Rev.of_WB235_1:676497-676765 Satlength=269 Nr of Repeats=6 RepeatLength=45 seed=TCTGATCCTT Num.seqs=4 Similarity=0.792593
10   AAGGATCAGACCATCA-GGAGGTACCCTTGTGATCCTTCTTATCTG
Rev.of_WB235_3:7602309-7602577 Satlength=269 Nr of Repeats=6 RepeatLength=45 seed=TCTGATCCTT Num.seqs=4 Similarity=0.841975
10   AAGGATCAGACCATCA-GGAGGTACCCTTGTGATCCTTCTTATCTG
Rev.of_WB235_5:14348178-14348446 Satlength=269 Nr of Repeats=6 RepeatLength=45 seed=TCTGATCCTT Num.seqs=4 Similarity=0.812346
10   AAGGATCAGACCATCA-GGAGGTACCCTTGTGATCCTTCTTATCTG
WB235_1:5977228-5977451 Satlength=224 Nr of Repeats=5 RepeatLength=45 seed=AAGGATCAGA Num.seqs=3 Similarity=0.841975
45   AAGGATCAGACCATCA-GAAGGTACCCTTACGATCCTTCTTATCTG
Rev.of_WB235_5:11108155-11108378 Satlength=224 Nr of Repeats=5 RepeatLength=45 seed=TCTGATCCTT Num.seqs=3 Similarity=0.822222
55   AAGGATCAGACCATCA-GGAGGTACCCTTACGATTCTTCTTATCTG
Rev.of_WB235_1:1664772-1664951 Satlength=180 Nr of Repeats=4 RepeatLength=45 seed=TCTGATCCTT Num.seqs=3 Similarity=0.743210
10   AAGGATCAGAACATCA-GAAGGTACCCATGTGATCCTTCGGAGCTG
WB235_5:14348461-14348640 Satlength=180 Nr of Repeats=4 RepeatLength=45 seed=CTGAAGGATC Num.seqs=3 Similarity=0.841975
42   AAGGATCAGAACATCA-GAAGGTACCCATGTGATCCTTCGGAGCTG
Rev.of_WB235_1:3746094-3746497 Satlength=404 Nr of Repeats=9 RepeatLength=45 seed=TCTGATCCTT Num.seqs=7 Similarity=0.825044
10   AAGGATCAGANCATCA-GAAGGTACCCATGTGATCCTTCGGAGCTG
WB235_1:3746917-3747230 Satlength=314 Nr of Repeats=7 RepeatLength=45 seed=CTGAAGGATC Num.seqs=5 Similarity=0.792593
42   AAGGATCAGANCATCA-GAAGGTACCCATGTGATCCTTCGGAGCTG
Rev.of_WB235_1:5975024-5975828 Satlength=805 Nr of Repeats=18 RepeatLength=45 seed=TCTGATCCTT Num.seqs=12 Similarity=0.835690
10   AAGGATCAGAACATCA-GAAGGTACCCTTGTGATCCTTCGGATCTG
Rev.of_WB235_2:11527871-11528452 Satlength=582 Nr of Repeats=13 RepeatLength=45 seed=TCTGATCCTT Num.seqs=9 Similarity=0.846914
10   AAGGATCAGANCATCA-GAAGGTACCCATGTGATCCTTCGGATCTG
WB235_2:11530067-11530424 Satlength=358 Nr of Repeats=8 RepeatLength=45 seed=GAAGGATCAG Num.seqs=5 Similarity=0.828148
44   AAGGATCAGANCATCA-GAAGGTACCCATGTGATCCTTCGGATCTG
Rev.of_WB235_3:2496715-2496983 Satlength=269 Nr of Repeats=6 RepeatLength=45 seed=TCTGATCCTT Num.seqs=4 Similarity=0.802469
10   AAGGATCAGACCATCA-GGAGGTACCCATGTGATCCTTCGGATCTG
WB235_3:7602873-7603364 Satlength=492 Nr of Repeats=11 RepeatLength=45 seed=GAAGGATCAG Num.seqs=7 Similarity=0.820811
44   AAGGATCAGANCATCA-GGAGGTACCCATGTGATCCTTCGGATCTG
Rev.of_WB235_3:10792337-10792694 Satlength=358 Nr of Repeats=8 RepeatLength=45 seed=TCTGATCCTT Num.seqs=5 Similarity=0.801481
10   AAGGATCAGANCATCA-GGAGGTACCCATGTGATCCTTCGGATCTG
WB235_4:349715-350206 Satlength=492 Nr of Repeats=11 RepeatLength=45 seed=GAAGGATCAG Num.seqs=7 Similarity=0.826455
44   AAGGATCAGANCATCA-GGAGGTACCCATGTGATCCTTCGGATCTG
WB235_2:6082023-6082291 Satlength=269 Nr of Repeats=6 RepeatLength=45 seed=CTGAAGGATC Num.seqs=4 Similarity=0.797531
42   AAGGATCAGAACATCA-GAAGGTACCCATACGATCCTTCGGAGCTG

WB235_5:11108491-11108983 Satlength=493 Nr of Repeats=11 RepeatLength=45 seed=CTGAAGGATC Num.seqs=8 Similarity=0.864550
42   AAGGATCAGAACATCA-GAAGGTACCCATACGATCCTTCGGAGCTG
Rev.of_WB235_4:8255934-8256202 Satlength=269 Nr of Repeats=6 RepeatLength=45 seed=TCTGATCCTT Num.seqs=4 Similarity=0.851852
55   AAGGATCAGACCATCA-GGAGGTACCCTTACGATCCTTCGGAGCTG
WB235_2:3937888-3938177 Satlength=290 Nr of Repeats=6 RepeatLength=45 seed=AAGGATCAGA Num.seqs=5 Similarity=0.643210
45   AAGGATCAGATCANCA-GGAGGTACCCTTCTGATCCTTCGGAGCTG
Rev.of_WB235_2:12749754-12749935 Satlength=182 Nr of Repeats=4 RepeatLength=45 seed=ATCTGATCCT Num.seqs=3 Similarity=0.950617
56   AAGGATCAGATCACCA-GGAGGTACCCTTNTGATCCTTCGGAGCTG
WB235_4:3366659-3367988 Satlength=1330 Nr of Repeats=24 RepeatLength=45 seed=TCTGATCCTT Num.seqs=17 Similarity=0.598811
72   AAGGATCAGATCANCA-GGAGGTACCCTTCTGATCCTTCGNAGCTG
Rev.of_WB235_4:10943462-10944138 Satlength=677 Nr of Repeats=14 RepeatLength=45 seed=AGCTCCGAAG Num.seqs=11 Similarity=0.932121
89   AAGGATCAGATCACCA-GGAGGTACCCTTCTGATCCTTCGGAGCTT
WB235_4:10945693-10946234 Satlength=542 Nr of Repeats=12 RepeatLength=45 seed=CTTCGGAGCT Num.seqs=11 Similarity=0.945050
124   AAGGATCAGATCACCA-GGAGGTACCCTTCTGATCCTTCGGAGCTT
WB235_2:13598710-13599880 Satlength=1171 Nr of Repeats=26 RepeatLength=45 seed=ACCAGGAGGT Num.seqs=26 Similarity=0.929709
57   AATGATCAGATCACCA-GGAGGTACCCTTCTTATCCTTCGGAGCTG

```
**   ****  **  * *   * * **     ** ***   * **
```

Consensus:

AAGGATCAGAnCAtCAGgAGGTACCCtTgTGATCCTTCGgAgCTG

>WB235_Fam_13_43_26 Nr. of seq. 26 Alignment length(with gaps) = 47 Alignment score = 0.705990
WB235_1:1322604-1322925 Satlength=322 Nr of Repeats=5 RepeatLength=42 seed=TTTGCCGGAA Num.seqs=3 Similarity=0.705426
0   TTTGCCGGAAAT--TTTCATTTCCGGCAA-ATTGCCGATTTGNCG--
WB235_2:12951422-12951594 Satlength=173 Nr of Repeats=4 RepeatLength=43 seed=CGGCAAATTA Num.seqs=4 Similarity=0.803618
21   TTTGCCGGAAAT--TTTCATTTCCGGCAA-ATTACCAATTTGCCGA-
Rev.of_WB235_1:1796469-1796919 Satlength=451 Nr of Repeats=9 RepeatLength=43 seed=AAATCGGCAA Num.seqs=7 Similarity=0.793282
3   TTTGCCGGAAAT--TTTCAATTCCGGCAA-ATTGCCGGTTTGCCGA-
WB235_2:12905369-12905758 Satlength=390 Nr of Repeats=5 RepeatLength=43 seed=GATTTGCCGG Num.seqs=3 Similarity=0.875969
41   TTTGCCGGAAAT--TTTCAATCCCGGCAA-ATTGCAGGTTTGCCGA-
WB235_4:2140998-2141533 Satlength=536 Nr of Repeats=8 RepeatLength=43 seed=TTGCCGGAAA Num.seqs=5 Similarity=0.868148
44   TTTGCCGGAAAT--TTTCAATCCCGGCAT-TTTGCCGGTTTGCCGA-
Rev.of_WB235_1:3424298-3424548 Satlength=251 Nr of Repeats=6 RepeatLength=43 seed=TTGAAAATTT Num.seqs=5 Similarity=0.810853
18   TTTGCCGAAAAT--TTTCAATTCCGCCAA-TTTGCCGGTTTGCCGA-
Rev.of_WB235_2:3111494-3112267 Satlength=774 Nr of Repeats=16 RepeatLength=43 seed=TTGCCGGAAT Num.seqs=13 Similarity=0.825084
27   TTTACAGGAAAT--TTTCAATTCCGGCAA-GTTGCCGGTTTGCCGA-

Rev.of_WB235_4:2964807-2965022 Satlength=216 Nr of Repeats=4
RepeatLength=43 seed=TTTGCCGGAA Num.seqs=3 Similarity=0.739899
28   TTTNCAGGAAAT--TTTCAATTCCGGCAA-ACTGCCGGTTTGCAGA-
Rev.of_WB235_1:2263541-2264092 Satlength=552 Nr of Repeats=13
RepeatLength=43 seed=ATTTCCGGCA Num.seqs=12 Similarity=0.752353
12   TTTGCCGGAAAT--TTTTATTTTCGGCAA-NTTGCCGGTTTGCCGA-
Rev.of_WB235_1:11474410-11474625 Satlength=216 Nr of Repeats=5
RepeatLength=43 seed=CGGCAAATCG Num.seqs=5 Similarity=0.801550
50   TTTGCCGGAAAT--TTTCATTTTCGGCAA-NTTGCCGGTTTGCCGA-
Rev.of_WB235_2:12402768-12404058 Satlength=1291 Nr of Repeats=30
RepeatLength=43 seed=TTTCCGGCAA Num.seqs=30 Similarity=0.867914
54   TTTGCCGGAAAT--TTTCATTTTCGGCAA-ATTGCCGGTTTGCCGA-
WB235_3:12017198-12017750 Satlength=553 Nr of Repeats=10 RepeatLength=43
seed=AATTGCCGGT Num.seqs=7 Similarity=0.748854
69   TTTGCCGGAAAA--TTTCATTTTCGGCAA-ATTGCCGGTTTGCCGA-
WB235_3:13094103-13095343 Satlength=1241 Nr of Repeats=25 RepeatLength=43
seed=CGGAAATTTT Num.seqs=17 Similarity=0.776106
48   TTTGCCGGAAAT--TTTNATTTTCGGCAA-TTTGCCNGTTTGCCGA-
WB235_2:12719695-12719880 Satlength=186 Nr of Repeats=5 RepeatLength=43
seed=TATTTTCATT Num.seqs=3 Similarity=0.979328
52   TTTGCCGGGTAT--TTTCATTTTCGGCAA-ATTGCCGGTTTGCCGA-
WB235_1:687047-687860 Satlength=814 Nr of Repeats=14 RepeatLength=44
seed=TTTGCCGGTT Num.seqs=9 Similarity=0.927609
28   TTTGCCCGAAATT-TTTCAATTTCGGCAA-TTTGCCGGTTTGCCGA-
Rev.of_WB235_1:1797802-1798215 Satlength=414 Nr of Repeats=9
RepeatLength=43 seed=GCCGGAATTG Num.seqs=7 Similarity=0.864157
25   TTTGCCAGAAAT--TTTCAATTCCGGCTA-TGTGCCGGTTTGCCGA-
WB235_1:2817881-2818157 Satlength=277 Nr of Repeats=5 RepeatLength=43
seed=TTGCCGATTT Num.seqs=3 Similarity=0.834625
29   TTTGCCGGAAAT--TTACAATTCC-ACTATTTTGCCGGTTTGCCGA-
Rev.of_WB235_1:1209321-1210686 Satlength=1366 Nr of Repeats=32
RepeatLength=43 seed=CCAGAAATTA Num.seqs=22 Similarity=0.892882
24   TTTGCCGAAAAT--TCTAATTTCTGGCAA-TTTGCCGGTTTGCTGA-
Rev.of_WB235_1:2858185-2858357 Satlength=173 Nr of Repeats=4
RepeatLength=43 seed=AAATCGGCAA Num.seqs=4 Similarity=0.906977
46   TTTGCCGGGAAT--TTTCATTTTTTGCAA-TTTGCCGGATTGCCGA-
Rev.of_WB235_3:12581020-12581289 Satlength=270 Nr of Repeats=5
RepeatLength=43 seed=GGCAAATCGG Num.seqs=3 Similarity=0.689394
49   TTTGCCGGAAAT--TTTNATATTTNACAA-ATTGCCGGTTTGCCGA-
WB235_1:2653859-2654031 Satlength=173 Nr of Repeats=4 RepeatLength=43
seed=TTGCCGATTT Num.seqs=4 Similarity=0.798450
36   TTTGCCGGAAAT-GTTT-ATTTCCGACAA-TTGGCAGATTTGCCGA-
WB235_1:14554105-14554484 Satlength=380 Nr of Repeats=9 RepeatLength=43
seed=TTTGCCGGTT Num.seqs=8 Similarity=0.931340
27   TTCGACGGAAAT--TTTTATTTTCGGCAC-TTTGCCGGTTTGCCGG-
Rev.of_WB235_5:3679665-3679834 Satlength=170 Nr of Repeats=4
RepeatLength=42 seed=TTTCCAGCAA Num.seqs=3 Similarity=0.767196
11   TTTGCTGGAAA---TCTCAATTCCGGCAA-TTCGCTGATTTGCCGA-
Rev.of_WB235_1:2233376-2233627 Satlength=252 Nr of Repeats=6
RepeatLength=43 seed=CAAATCGGCA Num.seqs=5 Similarity=0.795349
47   TTTGCTGGAAAT--TTTTAATTTCGGAAA-TTTGCCGAGTTGCCGA-
WB235_5:17885285-17887191 Satlength=1907 Nr of Repeats=37 RepeatLength=44
seed=TTGCCGGAAA Num.seqs=31 Similarity=0.808472
1   TTTGCCGGAAAT--ATTCAATTTTGGCAA-ATTGNCGGTTTGCTGAA

WB235_1:11113480-11113648 Satlength=169 Nr of Repeats=4 RepeatLength=42 seed=TGCCGGAAAT Num.seqs=4 Similarity=0.788360
2   GCTGCCGGAAAT---TTCAAACCCGGCAA-TTTGCCCATTTGCCGA-

*   *   ***   *
Consensus:

TTTGCCGGAAATTTTcAaTTcCGGCAAtTTGCCGgTTTGCCGA

>WB235_Fam_14_11_23 Nr. of seq. 23 Alignment length(with gaps) = 12
Alignment score = 0.800725
WB235_1:11694858-11694997 Satlength=140 Nr of Repeats=13 RepeatLength=11 seed=TTTTGCACAT Num.seqs=9 Similarity=0.838384
0   TTTTGCACAT-T
WB235_1:12093567-12094297 Satlength=731 Nr of Repeats=59 RepeatLength=11 seed=CACATTTTTT Num.seqs=45 Similarity=0.854790
5   TTTTGCACAT-T
WB235_1:12129305-12129768 Satlength=464 Nr of Repeats=41 RepeatLength=11 seed=TTGCACATTT Num.seqs=32 Similarity=0.962854
2   TTTTGCACAT-T
WB235_2:4442528-4442746 Satlength=219 Nr of Repeats=20 RepeatLength=11 seed=TGCACATTTT Num.seqs=18 Similarity=0.986532
3   TTTTGCACAT-T
WB235_3:1245524-1246875 Satlength=1352 Nr of Repeats=116 RepeatLength=11 seed=TTTTTGCACA Num.seqs=73 Similarity=0.910060
10   TTTTGCACAT-T
Rev.of_WB235_3:1671460-1671581 Satlength=122 Nr of Repeats=11 RepeatLength=11 seed=GCAAAAAATG Num.seqs=11 Similarity=0.960331
6   TTTTGCACAT-T
WB235_3:1809979-1810419 Satlength=441 Nr of Repeats=36 RepeatLength=11 seed=ATTTTTTGCA Num.seqs=32 Similarity=0.920088
8   TTTTGCACAT-T
Rev.of_WB235_3:1985833-1986090 Satlength=258 Nr of Repeats=24 RepeatLength=11 seed=TGTGCAAAAA Num.seqs=17 Similarity=0.959002
9   TTTTGCACAT-T
WB235_3:12172469-12173356 Satlength=888 Nr of Repeats=63 RepeatLength=11 seed=TTTTTGCACA Num.seqs=46 Similarity=1.000000
10   TTTTGCACAT-T
Rev.of_WB235_3:13116308-13116374 Satlength=67 Nr of Repeats=5 RepeatLength=11 seed=AAAAATGTGC Num.seqs=4 Similarity=1.000000
3   TTTTGCACAT-T
WB235_4:15648768-15648845 Satlength=78 Nr of Repeats=6 RepeatLength=11 seed=TGCACATTTT Num.seqs=5 Similarity=0.903030
3   TTTTGCACAT-T
WB235_5:2886582-2886789 Satlength=208 Nr of Repeats=19 RepeatLength=11 seed=GCACATTTTT Num.seqs=17 Similarity=0.908200
4   TTTTGCACAT-T
Rev.of_WB235_5:3629745-3629789 Satlength=45 Nr of Repeats=4 RepeatLength=11 seed=CAAAAAATGT Num.seqs=4 Similarity=0.878788
5   TTTTGCACAT-T
WB235_5:11057749-11057963 Satlength=215 Nr of Repeats=10 RepeatLength=11 seed=CACATTTTTT Num.seqs=9 Similarity=0.946128
5   TTTTGCACAT-T

Rev.of_WB235_6:8602386-8602451 Satlength=66 Nr of Repeats=5 RepeatLength=11 seed=GCAAAAAATG Num.seqs=4 Similarity=0.878788
6 TTTTGCACAT-T
WB235_3:11862696-11862861 Satlength=166 Nr of Repeats=15 RepeatLength=11 seed=ACATTTTTTG Num.seqs=13 Similarity=0.919192
6 TTTTGNACAT-T
Rev.of_WB235_1:11840154-11840231 Satlength=78 Nr of Repeats=7 RepeatLength=11 seed=CAAAAAATGT Num.seqs=7 Similarity=0.942280
5 TTTTGGACAT-T
WB235_4:13547778-13547822 Satlength=45 Nr of Repeats=4 RepeatLength=11 seed=CACATTTTTT Num.seqs=4 Similarity=0.919192
5 TTTTCCACAT-T
WB235_4:13575701-13576041 Satlength=341 Nr of Repeats=30 RepeatLength=11 seed=GCACATTTTT Num.seqs=22 Similarity=0.772787
4 TTTAGCACAT-T
Rev.of_WB235_6:3261930-3262051 Satlength=122 Nr of Repeats=11 RepeatLength=11 seed=AAAATGTGCT Num.seqs=7 Similarity=0.826720
13 TTTAGCACAT-T
Rev.of_WB235_4:15034367-15034488 Satlength=122 Nr of Repeats=9 RepeatLength=11 seed=TGTGCAAAAA Num.seqs=7 Similarity=0.838384
9 TTTTGCACAA-T
Rev.of_WB235_5:3092621-3092687 Satlength=67 Nr of Repeats=5 RepeatLength=11 seed=TTTGGCAAGA Num.seqs=4 Similarity=0.818182
10 TCTTGCCCAA-T
WB235_2:144001-144084 Satlength=84 Nr of Repeats=6 RepeatLength=12 seed=ATTTCTTGCA Num.seqs=5 Similarity=0.844444
21 TCTTGCAAAATT

* *   * *
Consensus:

TTTTGCACATT

>WB235_Fam_15_31_22 Nr. of seq. 22 Alignment length(with gaps) = 33 Alignment score = 0.785911
WB235_3:78610-79849 Satlength=1240 Nr of Repeats=39 RepeatLength=31 seed=TAAATAATTA Num.seqs=37 Similarity=0.866835
0 TAAATAATTAGCAGA-CCAAAGTTGGG-TCTGC
WB235_3:85304-85614 Satlength=311 Nr of Repeats=9 RepeatLength=31 seed=TGCTAAATAA Num.seqs=8 Similarity=0.907834
28 TAAATAATTAGCAGA-CCAAAGTTGGG-TATGC
Rev.of_WB235_3:498249-499054 Satlength=806 Nr of Repeats=26 RepeatLength=31 seed=GGTCTGCTAA Num.seqs=19 Similarity=0.861661
48 TAAATAATTAGCAGA-CCAAAGTTGGG-TATGC
WB235_3:587989-588144 Satlength=156 Nr of Repeats=5 RepeatLength=31 seed=TGGGTATGCT Num.seqs=5 Similarity=1.000000
53 TAAATAATTAGCAGA-CCAAAGTTGGG-TATGC
Rev.of_WB235_3:584894-585235 Satlength=342 Nr of Repeats=11 RepeatLength=31 seed=GGTCTGCTAA Num.seqs=11 Similarity=0.968719
17 TAAATAATTAGCAGA-CCGAAGTTGGG-TATGC
Rev.of_WB235_3:334126-334653 Satlength=528 Nr of Repeats=17 RepeatLength=31 seed=TGGTCTGCTA Num.seqs=15 Similarity=0.868920
18 TAAATATTTAGCAGA-CCAAAGTTGGG-TATGC

```
WB235_3:346580-346716 Satlength=137 Nr of Repeats=5 RepeatLength=31
seed=AATATTTAGC  Num.seqs=3 Similarity=0.885305
33    TAAATATTTAGCAGA-CCAAAGTTGGG-TATGC
WB235_3:470472-471505 Satlength=1034 Nr of Repeats=16 RepeatLength=31
seed=TTTAGCAGAC  Num.seqs=10 Similarity=0.931183
37    TAAATATTTAGCAGA-CCAAAGTTGGG-TATGC
WB235_3:492131-493485 Satlength=1355 Nr of Repeats=34 RepeatLength=31
seed=TTAGCAGACC  Num.seqs=25 Similarity=0.924301
38    TAAATATTTAGCAGA-CCAAAGATGGG-TATGC
WB235_3:855590-855838 Satlength=249 Nr of Repeats=7 RepeatLength=31
seed=TAAATATTTA  Num.seqs=6 Similarity=0.862366
31    TAAATATTTAGCAGA-CCAAAGTTAGG-TATGC
Rev.of_WB235_3:187630-188214 Satlength=585 Nr of Repeats=4
RepeatLength=31 seed=TTAGCAGACC  Num.seqs=3 Similarity=0.942652
3    TAAATAATTAGCAGA-CCATAGTTGGG-TCTGC
WB235_3:410715-410901 Satlength=187 Nr of Repeats=6 RepeatLength=31
seed=TTGGGTCTGC  Num.seqs=6 Similarity=0.868100
21    TAAATAATTAGCAGA-CCATAATTGGG-TCTGC
Rev.of_WB235_3:423133-423737 Satlength=605 Nr of Repeats=6
RepeatLength=31 seed=TATTTAGCAG  Num.seqs=5 Similarity=0.901075
37    TAAATAATTAGCAGA-CCATAATTGGG-TCTGC
Rev.of_WB235_3:189539-190145 Satlength=607 Nr of Repeats=5
RepeatLength=31 seed=TTAGCAGACC  Num.seqs=3 Similarity=0.971326
3    TAATTATTTAGCAGA-CCATACTTGGG-TCTGC
Rev.of_WB235_3:191259-191627 Satlength=369 Nr of Repeats=7
RepeatLength=31 seed=GGTCTGCTAA  Num.seqs=6 Similarity=0.868100
17    TAAATATTTAGCAGA-CCATACTTGGG-TCTGC
Rev.of_WB235_3:193180-193459 Satlength=280 Nr of Repeats=9
RepeatLength=31 seed=ACCCAAGTAT  Num.seqs=9 Similarity=0.906810
27    TAAACATTTAGCAGA-CCATACTTGGG-TCTGC
WB235_3:9853874-9853997 Satlength=124 Nr of Repeats=4 RepeatLength=31
seed=TCTGCTAAAT  Num.seqs=3 Similarity=0.971326
26    TAAATAATTAGCAGA-CCATACTTGAT-TCTGC
WB235_3:337086-337644 Satlength=559 Nr of Repeats=17 RepeatLength=31
seed=TATGCTAAAT  Num.seqs=16 Similarity=0.883154
26    TAAATATTTAGCAGA-CCAAAAGTAGG-TATGC
WB235_3:340719-342118 Satlength=1400 Nr of Repeats=23 RepeatLength=31
seed=TTAGCAGACC  Num.seqs=22 Similarity=0.867430
38    TAAATATTTAGCAGA-CCAAAANTAGG-TATGC
Rev.of_WB235_3:345158-345375 Satlength=218 Nr of Repeats=7
RepeatLength=31 seed=CTGCTAAATA  Num.seqs=7 Similarity=0.938556
45    TAAATATTTAGCAGA-CCCAAAGTTGG-TATGC
WB235_3:9958568-9958766 Satlength=199 Nr of Repeats=4 RepeatLength=31
seed=TATGCTAAAT  Num.seqs=3 Similarity=0.812500
26    TAAATATTTAGCAGA-CCCAANGT-GGNTATGC
WB235_3:633852-634044 Satlength=193 Nr of Repeats=6 RepeatLength=31
seed=TTAGCAGACC  Num.seqs=4 Similarity=0.781250
7    TAAATAATTAGCAGACCCATAGGTAGG-TATGC

***  * ******** **  *  *    * ***
Consensus:

TAAATAtTTAGCAGACCAaAgtTgGGTaTGC
```

>WB235_Fam_16_26_20 Nr. of seq. 20 Alignment length(with gaps) = 26
Alignment score = 0.760324
WB235_1:3171655-3172409 Satlength=755 Nr of Repeats=29 RepeatLength=26
seed=CAGAGTCACT Num.seqs=29 Similarity=0.926235
0   CAGAGTCACTATTTTTGGGGAACGGC
WB235_4:15409425-15409737 Satlength=313 Nr of Repeats=12 RepeatLength=26
seed=CAGAGTCACT Num.seqs=12 Similarity=0.943279
0   CAGAGTCACTATTTTTGGTGAACGGC
WB235_5:5283302-5285834 Satlength=2533 Nr of Repeats=98 RepeatLength=26
seed=CAGAGTCACT Num.seqs=94 Similarity=0.876599
0   CAGAGTCACTATTTTTGGTGAACGGC
WB235_6:15807460-15811152 Satlength=3693 Nr of Repeats=142
RepeatLength=26 seed=GTGAACGGCC Num.seqs=142 Similarity=0.927510
17   CAGAGTCACTATTTTTGGTGAACGGC
WB235_6:1636150-1638568 Satlength=2419 Nr of Repeats=93 RepeatLength=26
seed=GGTGAACGGT Num.seqs=93 Similarity=0.946896
16   CAGAGTCACTATTTTTGGTGAACGGT
Rev.of_WB235_6:5542238-5542420 Satlength=183 Nr of Repeats=7
RepeatLength=26 seed=TAGTGACTCT Num.seqs=7 Similarity=0.836567
11   CAGAGTCACTAATTTTGGTGAACGGC
WB235_3:931109-931213 Satlength=105 Nr of Repeats=4 RepeatLength=26
seed=CGGCCAGAGT Num.seqs=4 Similarity=0.863248
22   CAGAGTCACTACTTATGGTGAACGGC
WB235_5:10446631-10446995 Satlength=365 Nr of Repeats=14 RepeatLength=26
seed=CCAGAGTCAC Num.seqs=14 Similarity=0.974641
25   CAGAGTCACTATAATTGGTGAACGGC
Rev.of_WB235_1:13277345-13277527 Satlength=183 Nr of Repeats=7
RepeatLength=26 seed=CTCTGGCAGT Num.seqs=7 Similarity=0.941392
5   CAGAGCCACTTATTTTAGTGAACTGC
WB235_4:10985445-10985549 Satlength=105 Nr of Repeats=4 RepeatLength=26
seed=CCAGAGTCAC Num.seqs=4 Similarity=0.863248
25   CAGAGTCACTATTTTTAGTGAACTGC
WB235_6:2379254-2380684 Satlength=1431 Nr of Repeats=55 RepeatLength=26
seed=TTTTAGTGAA Num.seqs=55 Similarity=0.934801
38   CAGAGTCACTAATTTTAGTGAACTGC
WB235_1:3664200-3666098 Satlength=1899 Nr of Repeats=73 RepeatLength=26
seed=ATGGTTAGAG Num.seqs=73 Similarity=0.974476
21   TAGAGTCATATTTTTAGGTGAATGGT
Rev.of_WB235_2:10336035-10336399 Satlength=365 Nr of Repeats=14
RepeatLength=26 seed=TATGACTCTA Num.seqs=14 Similarity=0.950409
36   TAGAGTCATATTTTTAGGTGAATGGT
Rev.of_WB235_6:1642568-1643478 Satlength=911 Nr of Repeats=35
RepeatLength=26 seed=GACTCTAACC Num.seqs=35 Similarity=0.961474
33   TAGAGTCATATTTTTAGGTGAATGGT
WB235_1:13404330-13408464 Satlength=4135 Nr of Repeats=159
RepeatLength=26 seed=TTTTAGGTGA Num.seqs=159 Similarity=0.972965
37   TAGAGTCATAATTTTAGGTGAATGGT
WB235_3:5353177-5358366 Satlength=5190 Nr of Repeats=198 RepeatLength=26
seed=GTTAGAGTCA Num.seqs=196 Similarity=0.933400
24   TAGAGTCATTATTTTAGGTGAATGGT
Rev.of_WB235_3:5358375-5358505 Satlength=131 Nr of Repeats=5
RepeatLength=26 seed=ACCATTCACC Num.seqs=5 Similarity=0.917949
26   TAGAGTCATTATTTTAGGTGAATGGT

WB235_4:14781306-14781436 Satlength=131 Nr of Repeats=5 RepeatLength=26
seed=TGGTTAGAGT Num.seqs=5 Similarity=0.958974
48   TAGAGTCATTATTTTAGGTGAATGGT
Rev.of_WB235_5:15170122-15170278 Satlength=157 Nr of Repeats=6
RepeatLength=26 seed=TTCACCTAAA Num.seqs=6 Similarity=0.846154
48   TAGAGTCATTATTTTAGGTGAATGGT
Rev.of_WB235_6:11785744-11787512 Satlength=1769 Nr of Repeats=68
RepeatLength=26 seed=ACCATTCACC Num.seqs=68 Similarity=0.956012
26   TAGAGTCATTATTTTAGGTGAATGGT

**** **   * *** *
Consensus:

cAGAGTCActatTTTtGGTGAAcGGc

>WB235_Fam_17_37_19 Nr. of seq. 19 Alignment length(with gaps) = 43
Alignment score = 0.743370
WB235_5:16273671-16273852 Satlength=182 Nr of Repeats=5 RepeatLength=36
seed=GGGAATTCAA Num.seqs=4 Similarity=0.962963
25   TTTTAATTTTT--GAAAAC-ATTTTGGC-GGGAA-TTC--AAA
WB235_3:3023864-3024701 Satlength=838 Nr of Repeats=18 RepeatLength=37
seed=ATTTTGGCGG Num.seqs=16 Similarity=0.920120
54   ATTTAATTTTTC-GAAAAC-ATTTTGGC-GGGAA-TTC--AAA
WB235_5:17308169-17308466 Satlength=298 Nr of Repeats=8 RepeatLength=37
seed=AAAACATTTT Num.seqs=5 Similarity=0.894737
86   ATTTAATTTTTT-GAAAAC-ATTTTGGC-GGGAA-TTC--AAA
WB235_4:3098171-3098319 Satlength=149 Nr of Repeats=4 RepeatLength=37
seed=TGGCGGGAAT Num.seqs=4 Similarity=0.867868
58   TTTTAATTTTCC-GAAAAC-ATTTTGGC-GGGAA-TTC--AAA
WB235_2:12649041-12649452 Satlength=412 Nr of Repeats=11 RepeatLength=37
seed=ATTTTGGCGG Num.seqs=7 Similarity=0.951952
54   TTTTAATTTTTT-GAAAAT-ATTTTGGC-GGGAA-TTC--AAA
WB235_3:10943916-10944702 Satlength=787 Nr of Repeats=15 RepeatLength=37
seed=TTGGCGGGAA Num.seqs=10 Similarity=0.935135
57   TTTTAATTTTTT-GAAAAT-ATTTTGGC-GGGAA-TTC--AAA
WB235_4:280698-281177 Satlength=480 Nr of Repeats=13 RepeatLength=37
seed=TTGGCGGGAA Num.seqs=9 Similarity=0.933934
57   TTTTAATTTTTT-GAAAAT-ATTTTGGC-GGGAA-TTC--AAA
Rev.of_WB235_4:17147932-17148762 Satlength=831 Nr of Repeats=9
RepeatLength=37 seed=ATTCCCGCCA Num.seqs=6 Similarity=0.896697
68   TTTTAATTTTTT-GAAAAT-ATTTTGGC-GGGAA-TTC--AAA
WB235_6:14119472-14121076 Satlength=1605 Nr of Repeats=43 RepeatLength=37
seed=TTTAATTTTT Num.seqs=26 Similarity=0.924934
74   TTTTAATTTTTT-GAAAAT-ATTTTGGC-GGGAA-TTC--AAA
Rev.of_WB235_4:1681168-1681390 Satlength=223 Nr of Repeats=6
RepeatLength=37 seed=CGCCAAAATG Num.seqs=6 Similarity=0.894294
63   TTTTAATTTTTT-GAAAAC-ATTTTGGC-GGGAA-TTC--AAA
WB235_4:16471145-16471947 Satlength=803 Nr of Repeats=4 RepeatLength=37
seed=ATTCAAATTT Num.seqs=3 Similarity=0.951952
66   TTTTAATTTTTT-GAAAAT-ACTTTGGC-GGGAA-TTC--AAA
Rev.of_WB235_5:20153934-20154193 Satlength=260 Nr of Repeats=7
RepeatLength=37 seed=AATTTGAATT Num.seqs=7 Similarity=0.941656
75   TTTTAATTTTTC-AAAAAT-ATTTTGGC-GGGAA-TTC--AAA

WB235_4:15842446-15843519 Satlength=1074 Nr of Repeats=16 RepeatLength=37
seed=CGGGAATTCA Num.seqs=13 Similarity=0.831370
61   TTTTAATTTTTT-G-AAATCATTTTGGC-GGGAA-TTC--AAA
WB235_5:15941558-15942466 Satlength=909 Nr of Repeats=7 RepeatLength=37
seed=GCGGGAATTC Num.seqs=5 Similarity=0.877477
97   TTTTAATTATTT-G-AAATCATTTTGGC-GGGAA-TTC--AAA
WB235_1:1317490-1319274 Satlength=1785 Nr of Repeats=47 RepeatLength=38
seed=GGGAATTCAA Num.seqs=45 Similarity=0.939890
25   TTTTAATTTTTTTGAAAAC-ATTTTGGC-GGGAA-TTC--AAA
WB235_3:12698391-12698580 Satlength=190 Nr of Repeats=5 RepeatLength=38
seed=TTTTGGCGGG Num.seqs=4 Similarity=0.844017
56   ATTTAATTTTTTTGAAAAT-ATTTTGGC-GGGAA-TTC--AAA
WB235_1:280525-281393 Satlength=869 Nr of Repeats=23 RepeatLength=38
seed=AAATTTTAAT Num.seqs=17 Similarity=0.995872
34   TTTTAATTTTTT-GAAAAT-ATTTTGGC-GGGAATTTA--AAA
WB235_3:2113231-2114730 Satlength=1500 Nr of Repeats=39 RepeatLength=39
seed=TTTTTGGCGG Num.seqs=29 Similarity=0.914530
55   TTCAAA-TTTTC-AAATAT-TTTTTGGCGGGAAA-TTCAAAAA
Rev.of_WB235_3:2117069-2117228 Satlength=160 Nr of Repeats=4
RepeatLength=40 seed=TCCCGCCAAA Num.seqs=3 Similarity=0.866667
67   TTTAAATTTTTT-GAATAT-TTTTTGGCGGGAAA-TTCAAAAA

** ** **   *** * ** * *   * *   ******
Consensus:

GGGAATTCAAATTTTAATTTTTtGAAAAtATTTTGGC

>WB235_Fam_18_19_17 Nr. of seq. 17 Alignment length(with gaps) = 19
Alignment score = 0.859649
WB235_1:12955954-12956201 Satlength=248 Nr of Repeats=13 RepeatLength=19
seed=CAAGGCACTA Num.seqs=13 Similarity=0.816464
0   CAAGGCACTAGAAATGCGC
Rev.of_WB235_5:18575312-18575616 Satlength=305 Nr of Repeats=15
RepeatLength=19 seed=TAGTGCCTTG Num.seqs=12 Similarity=0.808612
10   CAAGGCACTAGAATTGCGC
Rev.of_WB235_5:18684513-18684741 Satlength=229 Nr of Repeats=8
RepeatLength=19 seed=TTCTAGTGCC Num.seqs=5 Similarity=0.817544
13   CAAGGCACTAGAATTGCGC
WB235_5:19373212-19373421 Satlength=210 Nr of Repeats=9 RepeatLength=19
seed=TTGCGCCAAG Num.seqs=7 Similarity=0.744444
13   CAAGGCACTAGAATTGCGC
Rev.of_WB235_3:3233988-3234755 Satlength=768 Nr of Repeats=13
RepeatLength=19 seed=CTTGGCGCAA Num.seqs=8 Similarity=0.764411
4   CAAGGCACGAGAATTGCGC
WB235_5:19379891-19380043 Satlength=153 Nr of Repeats=5 RepeatLength=19
seed=AGAATTGCGC Num.seqs=3 Similarity=0.859649
9   CAAGGCACGAGAATTGCGC
Rev.of_WB235_5:19148365-19148726 Satlength=362 Nr of Repeats=16
RepeatLength=19 seed=TGGCGCATTT Num.seqs=14 Similarity=0.787160
21   CAAGGCACGAGAAATGCGC
WB235_5:18388249-18388686 Satlength=438 Nr of Repeats=22 RepeatLength=19
seed=CAAGGCACTA Num.seqs=21 Similarity=0.750042
0   CAAGGCACTANAATTGCGT

Rev.of_WB235_5:18389794-18390302 Satlength=509 Nr of Repeats=18
RepeatLength=19 seed=TGACGCAATT Num.seqs=14 Similarity=0.787931
2   CAAGGCACTAGAATTGCGT
Rev.of_WB235_5:18676328-18676917 Satlength=590 Nr of Repeats=23
RepeatLength=19 seed=TGACGCAATT Num.seqs=15 Similarity=0.830476
2   CAAGGCACTAGAATTGCGT
WB235_5:18677260-18677830 Satlength=571 Nr of Repeats=26 RepeatLength=19
seed=ACTAGAATTG Num.seqs=23 Similarity=0.820539
6   CAAGGCACTAGAATTGCGT
Rev.of_WB235_5:19380090-19380527 Satlength=438 Nr of Repeats=16
RepeatLength=19 seed=GCCTTGACGC Num.seqs=11 Similarity=0.888995
6   CAAGGCACTAGAATTGCGT
Rev.of_WB235_5:19821185-19822021 Satlength=837 Nr of Repeats=37
RepeatLength=19 seed=TAGTGCCTTG Num.seqs=30 Similarity=0.844807
10   CAAGGCACTAGAATTGCGT
Rev.of_WB235_5:18573477-18573762 Satlength=286 Nr of Repeats=11
RepeatLength=19 seed=TTGACGCAAT Num.seqs=9 Similarity=0.666667
3   CAANGCACTAGAATTGCGT
Rev.of_WB235_5:18619123-18619731 Satlength=609 Nr of Repeats=29
RepeatLength=19 seed=GTGCCTTGAC Num.seqs=26 Similarity=0.876923
8   CAAGGCACTAGAANTGCGT
WB235_2:3036430-3037140 Satlength=711 Nr of Repeats=18 RepeatLength=19
seed=GCCAAGGCAC Num.seqs=11 Similarity=0.680606
17   CAAGGCACTNGAAANGCGC
Rev.of_WB235_5:19022623-19023690 Satlength=1068 Nr of Repeats=10
RepeatLength=19 seed=TGGCGCATTT Num.seqs=6 Similarity=0.714620
2   CAACTCACCAAAATTGCGC

*** ***   ** ***
Consensus:

CAAGGCACtAGAAtTGCGc

>WB235_Fam_19_14_17 Nr. of seq. 17 Alignment length(with gaps) = 14
Alignment score = 0.892682
WB235_2:11306986-11307037 Satlength=52 Nr of Repeats=4 RepeatLength=12
seed=ACTATAACTA Num.seqs=3 Similarity=0.851852
0   ACTATA-ACTACA-
WB235_1:11155001-11155099 Satlength=99 Nr of Repeats=7 RepeatLength=14
seed=AACTACTAAC Num.seqs=7 Similarity=0.803175
6   ACTACTAACTACTA
Rev.of_WB235_1:13019817-13020208 Satlength=392 Nr of Repeats=8
RepeatLength=14 seed=AGTTTGTAGT Num.seqs=5 Similarity=0.885714
10   ACTACAAACTACAA
Rev.of_WB235_1:13499215-13500226 Satlength=1012 Nr of Repeats=11
RepeatLength=14 seed=TAGTTTGTAG Num.seqs=7 Similarity=0.845805
18   ACTACAAACTACAA
WB235_1:13500544-13500919 Satlength=376 Nr of Repeats=11 RepeatLength=14
seed=TACAAACTAC Num.seqs=7 Similarity=0.918367
23   ACTACAAACTACAA
Rev.of_WB235_1:14324192-14324420 Satlength=229 Nr of Repeats=4
RepeatLength=14 seed=TGTAGTTTGT Num.seqs=3 Similarity=0.936508
20   ACTACAAACTACAA

WB235_2:2629919-2630440 Satlength=522 Nr of Repeats=10 RepeatLength=14 seed=CTACAAACTA Num.seqs=6 Similarity=0.796825
22 ACTACAAACTACAA
WB235_2:12761888-12762610 Satlength=723 Nr of Repeats=9 RepeatLength=14 seed=AACTACAAAC Num.seqs=6 Similarity=0.841270
20 ACTACAAACTACAA
Rev.of_WB235_2:13303475-13303853 Satlength=379 Nr of Repeats=13 RepeatLength=14 seed=TAGTTTGTAG Num.seqs=8 Similarity=0.829932
18 ACTACAAACTACAA
WB235_2:13962702-13964354 Satlength=1653 Nr of Repeats=47 RepeatLength=14 seed=TACAAACTAC Num.seqs=31 Similarity=0.870968
23 ACTACAAACTACAA
Rev.of_WB235_2:14006143-14006320 Satlength=178 Nr of Repeats=5 RepeatLength=14 seed=GTAGTTTGTA Num.seqs=3 Similarity=0.809524
19 ACTACAAACTACAA
Rev.of_WB235_4:15294619-15295382 Satlength=764 Nr of Repeats=4 RepeatLength=14 seed=TTGTAGTTTG Num.seqs=3 Similarity=0.936508
21 ACTACAAACTACAA
WB235_5:18354225-18354524 Satlength=300 Nr of Repeats=14 RepeatLength=14 seed=ACAAACTACA Num.seqs=10 Similarity=0.942857
10 ACTACAAACTACAA
Rev.of_WB235_5:19550939-19551875 Satlength=937 Nr of Repeats=7 RepeatLength=14 seed=TTTGTAGTTT Num.seqs=5 Similarity=0.804444
22 ACTACAAACTACAA
WB235_5:19551915-19552281 Satlength=367 Nr of Repeats=11 RepeatLength=14 seed=ACAAACTACA Num.seqs=7 Similarity=0.931973
10 ACTACANACTACAA
Rev.of_WB235_1:13600712-13601694 Satlength=983 Nr of Repeats=52 RepeatLength=14 seed=TGTAGTATGT Num.seqs=42 Similarity=0.960511
20 ACTACATACTACAT
WB235_4:1624502-1624593 Satlength=92 Nr of Repeats=6 RepeatLength=14 seed=ACTACACACT Num.seqs=5 Similarity=1.000000
21 ACTACACACTACAC

**** *****
Consensus:

ACTACAaACTACAA

>WB235_Fam_20_12_15 Nr. of seq. 15 Alignment length(with gaps) = 14 Alignment score = 0.745805
WB235_4:15410980-15411070 Satlength=91 Nr of Repeats=8 RepeatLength=10 seed=TGTTATGATA Num.seqs=5 Similarity=0.763636
0 TG-T-TATG--ATA
Rev.of_WB235_1:11145522-11145582 Satlength=61 Nr of Repeats=5 RepeatLength=12 seed=TATACATATA Num.seqs=5 Similarity=1.000000
0 TG-TATATG-TATA
WB235_1:11342672-11342750 Satlength=79 Nr of Repeats=6 RepeatLength=12 seed=TATATGTATA Num.seqs=5 Similarity=1.000000
8 TG-TATATG-TATA
WB235_1:14430493-14430609 Satlength=117 Nr of Repeats=10 RepeatLength=12 seed=ATATGTATAT Num.seqs=6 Similarity=0.888889
9 TG-TATATG-TATA

WB235_2:2000885-2001197 Satlength=313 Nr of Repeats=26 RepeatLength=12
seed=ATGTATATGT Num.seqs=26 Similarity=0.950769
11   TG-TATATG-TATA
Rev.of_WB235_2:3471040-3471110 Satlength=71 Nr of Repeats=4
RepeatLength=12 seed=ATACATATAC Num.seqs=3 Similarity=1.000000
11   TG-TATATG-TATA
Rev.of_WB235_3:12538530-12538668 Satlength=139 Nr of Repeats=11
RepeatLength=12 seed=TATACATATA Num.seqs=10 Similarity=1.000000
0   TG-TATATG-TATA
WB235_4:1438688-1438742 Satlength=55 Nr of Repeats=4 RepeatLength=12
seed=TATATGTATA Num.seqs=3 Similarity=1.000000
8   TG-TATATG-TATA
WB235_5:18475570-18475679 Satlength=110 Nr of Repeats=6 RepeatLength=12
seed=GTATATGTAT Num.seqs=5 Similarity=0.955556
7   TG-TATATG-TATA
Rev.of_WB235_5:19038887-19039001 Satlength=115 Nr of Repeats=9
RepeatLength=12 seed=ATACATATAC Num.seqs=8 Similarity=1.000000
11   TG-TATATG-TATA
WB235_5:19957566-19957879 Satlength=314 Nr of Repeats=8 RepeatLength=12
seed=TATGTATATG Num.seqs=6 Similarity=1.000000
10   TG-TATATG-TATA
Rev.of_WB235_5:19966309-19966447 Satlength=139 Nr of Repeats=11
RepeatLength=12 seed=ATATACATAT Num.seqs=10 Similarity=1.000000
7   TG-TATATG-TATA
WB235_5:19043595-19043791 Satlength=197 Nr of Repeats=8 RepeatLength=12
seed=ATATGTATAT Num.seqs=5 Similarity=0.777778
11   TA-TNTATA-TNTA
WB235_5:19047920-19048249 Satlength=330 Nr of Repeats=23 RepeatLength=14
seed=TATATGTTAT Num.seqs=22 Similarity=0.960421
10   TGTTATATGTTATA
Rev.of_WB235_6:16158526-16158670 Satlength=145 Nr of Repeats=9
RepeatLength=14 seed=TACCATATAC Num.seqs=7 Similarity=0.841270
12   TGGTATATGGTATA

* * ***   **
Consensus:

TGTATATGTATA

>WB235_Fam_21_18_14 Nr. of seq. 14 Alignment length(with gaps) = 18
Alignment score = 0.786121
WB235_2:4210804-4210907 Satlength=104 Nr of Repeats=5 RepeatLength=18
seed=TGCAAGACTA Num.seqs=3 Similarity=0.950617
0   TGCAAGACTAATAGAGAC
WB235_3:922387-922734 Satlength=348 Nr of Repeats=8 RepeatLength=18
seed=TGCAAGACTA Num.seqs=5 Similarity=0.822222
0   TGCAAGACTAATAGAGAC
Rev.of_WB235_2:14355352-14355470 Satlength=119 Nr of Repeats=5
RepeatLength=18 seed=TAGTCTTGCA Num.seqs=3 Similarity=0.901235
10   TGCAAGACTAATAGAGGC
WB235_6:13423594-13423926 Satlength=333 Nr of Repeats=8 RepeatLength=18
seed=TGCAAGACTA Num.seqs=5 Similarity=0.851852
18   TGCAAGACTAATAGAGGC

WB235_5:3332437-3332702 Satlength=266 Nr of Repeats=4 RepeatLength=18
seed=TGCAAGACTA Num.seqs=3 Similarity=0.876543
18   TGCAAGACTANTAGAGGC
Rev.of_WB235_6:7574716-7575007 Satlength=292 Nr of Repeats=5
RepeatLength=18 seed=TAGTCTTGCA Num.seqs=3 Similarity=0.901235
10   TGCAAGACTATTAGAGGC
WB235_6:14323895-14325001 Satlength=1107 Nr of Repeats=10 RepeatLength=18
seed=TGCAAGACTA Num.seqs=6 Similarity=0.841975
0   TGCAAGACTAATAAAGGC
Rev.of_WB235_4:3834124-3834445 Satlength=322 Nr of Repeats=5
RepeatLength=18 seed=ATTAGTATTG Num.seqs=3 Similarity=0.802469
12   TGCAATACTAATAGGGAG
WB235_6:11859496-11859817 Satlength=322 Nr of Repeats=5 RepeatLength=18
seed=TGCAATACTA Num.seqs=3 Similarity=0.901235
18   TGCAAGACTAATAGGGAG
Rev.of_WB235_4:17134995-17135342 Satlength=348 Nr of Repeats=5
RepeatLength=18 seed=GCACTCCCTA Num.seqs=3 Similarity=0.950617
21   TGCAAGACTATTAGGGAG
Rev.of_WB235_5:12370870-12371218 Satlength=349 Nr of Repeats=5
RepeatLength=18 seed=GCACTCCCTA Num.seqs=3 Similarity=0.950617
21   TGCAAGACTATTAGGGAG
Rev.of_WB235_6:16135006-16135334 Satlength=329 Nr of Repeats=5
RepeatLength=18 seed=ATTAGTTTTG Num.seqs=3 Similarity=0.950617
12   TGCAAAACTAATAGAGAG
Rev.of_WB235_3:922819-923128 Satlength=310 Nr of Repeats=5
RepeatLength=18 seed=TAGTGCTGCA Num.seqs=3 Similarity=0.777778
10   TGCAGCACTANTAGAGAC
WB235_3:3794723-3795033 Satlength=311 Nr of Repeats=5 RepeatLength=18
seed=TGCAGCACTA Num.seqs=3 Similarity=0.851852
18   TGCAGCACTATTAGAGAC

**** **** ** *
Consensus:

TGCAAgACTAaTAGaGac

>WB235_Fam_22_59_11 Nr. of seq. 11 Alignment length(with gaps) = 62
Alignment score = 0.794330
WB235_1:3990050-3990699 Satlength=650 Nr of Repeats=11 RepeatLength=59
seed=AAAACCAGTG Num.seqs=11 Similarity=0.799486
0   AAAACCAGTGCACATTTGAAATT-CCATA--TTCTCCATAATTTTCAGTTTAAAATATTTTG
Rev.of_WB235_3:8958876-8960427 Satlength=1552 Nr of Repeats=21
RepeatLength=59 seed=TTCAAAACTT Num.seqs=20 Similarity=0.972643
2   AAAACCAGTGCACATTTGAAATT-CCATA--TTCTCCATAAATCTCAGTTTAAAAAGTTTTG
Rev.of_WB235_5:6219658-6220602 Satlength=945 Nr of Repeats=16
RepeatLength=59 seed=ATGTGCACTG Num.seqs=16 Similarity=0.951601
15   AAAACCAGTGCACATTTGAAATT-CCATA--TTCTCCATTATTCTCAGTTTAAAAATTTTTG
WB235_3:13074772-13086747 Satlength=11976 Nr of Repeats=206
RepeatLength=59 seed=TCTCCATAAT Num.seqs=197 Similarity=0.927617
29   AAAAACAGTGCACATTTGAAATT-CCATA--TTCTCCATAATTCTCAGTTTAAAAAAATTTG
Rev.of_WB235_3:10224644-10226007 Satlength=1364 Nr of Repeats=23
RepeatLength=59 seed=AAATGTGCAC Num.seqs=20 Similarity=0.928397
17   AAAACCAGTGCACATTT-AAATTTCCATA--TTCTCCATAATTCTCAGTTTAAAAAATTTTG

Rev.of_WB235_5:3056063-3056653 Satlength=591 Nr of Repeats=10 RepeatLength=59 seed=AATTTCAAAT Num.seqs=10 Similarity=0.895123
23 AAAACCAGTGCACATTTGAAATTNCCATA--TTTTCCATAATTCTCAGTTTAAAAATTTTTG
Rev.of_WB235_5:17384240-17394505 Satlength=10266 Nr of Repeats=173 RepeatLength=59 seed=ATGGTGAATA Num.seqs=165 Similarity=0.936880
36 AAAACCAGTGCACATTTGAATTT-CCATA--TTCACCATAATTCTCGGTTTTAAAAATTTTG
Rev.of_WB235_2:891126-892012 Satlength=887 Nr of Repeats=12 RepeatLength=59 seed=CAAATTTTTT Num.seqs=11 Similarity=0.987673
0 AAAACCAGTTCACATTTAAAAAT-CCATA--TTTTCCATAATTCTCAGTTTAAAAAAATTTG
Rev.of_WB235_3:1017334-1020579 Satlength=3246 Nr of Repeats=55 RepeatLength=59 seed=TGTGCACTGG Num.seqs=55 Similarity=0.955928
14 AAAACCAGTGCACATTT-AAATT--TATATTTTTTCCATAATTCTCAGTTTAAAAAGTTTTG
WB235_4:12169299-12171242 Satlength=1944 Nr of Repeats=24 RepeatLength=59 seed=TTTTAAAAAT Num.seqs=23 Similarity=0.964628
45 AAAACCAGTGAAGTCTTGATATT-CCATA--TTCTCCATAATTCTCGGTTTTAAAAATTTTG
Rev.of_WB235_5:6942140-6942965 Satlength=826 Nr of Repeats=14 RepeatLength=59 seed=AATTCAAGAC Num.seqs=13 Similarity=0.956541
81 AAAACCAGTGAAGTCTTGAATTT-CCATA--TTCCCCATAATTCTCGGTTTTAAATTTTTTG

**** **** * ** * * *** ** **** * * ** **** *** ****
Consensus:

AAAACCAGTGCACATTTgAAATTCCATATTcTCCATAATTCTCaGTTTaAAAAaTTTTG

>WB235_Fam_23_32_11 Nr. of seq. 11 Alignment length(with gaps) = 35 Alignment score = 0.789437
WB235_5:20268029-20268566 Satlength=538 Nr of Repeats=15 RepeatLength=31 seed=CTACAAACTA Num.seqs=9 Similarity=0.899642
0 CTACAAACTACG-A-G-TTTTGGGCGCTGCTAAG-
Rev.of_WB235_5:18888872-18889346 Satlength=475 Nr of Repeats=14 RepeatLength=32 seed=TTGTAGTTTG Num.seqs=9 Similarity=0.939815
13 CTACAAACTACA-A-A-TTTTGGGCGCTGCTTAGA
WB235_5:19933050-19934036 Satlength=987 Nr of Repeats=29 RepeatLength=32 seed=ACTACAAACT Num.seqs=22 Similarity=0.934365
31 CTACAAACTACA-A-G-TTTTGGGCGCTGCTTAGA
WB235_5:20101712-20102976 Satlength=1265 Nr of Repeats=44 RepeatLength=32 seed=TTTTGGGCGC Num.seqs=27 Similarity=0.907170
14 CTACAAACTACA-A-T-TTTTGGGCGCTGCTTAGA
WB235_6:188398-190104 Satlength=1707 Nr of Repeats=53 RepeatLength=32 seed=CGCTGCTAAG Num.seqs=43 Similarity=0.970561
21 CTACAAACTACA-AG--TTTTGGGCGCTGCTAAGA
Rev.of_WB235_5:19117969-19118122 Satlength=154 Nr of Repeats=5 RepeatLength=33 seed=TTAGCAGCGC Num.seqs=4 Similarity=0.979798
30 CTACAAACTACA-AGA-TTTTGGGCGCTGCTAAGA
Rev.of_WB235_5:19122509-19124474 Satlength=1966 Nr of Repeats=65 RepeatLength=33 seed=AGCAGCGCCC Num.seqs=49 Similarity=0.900143
61 CTACAAACTACA-AGA-TTTTGGGCGCTGCTAAGA
WB235_5:20273044-20275266 Satlength=2223 Nr of Repeats=68 RepeatLength=31 seed=GGCGCTGCTA Num.seqs=43 Similarity=0.966992
19 CTACAAACTACA-A-G-ATTTGGGCGCTGCTAA-A
Rev.of_WB235_6:161009-161190 Satlength=182 Nr of Repeats=5 RepeatLength=32 seed=TGTAGTTTGT Num.seqs=4 Similarity=0.881944
44 CTACAAACTACA-A-G-ATTTGGGCGCTGCTAACA

```
Rev.of_WB235_5:20263899-20264125 Satlength=227 Nr of Repeats=7
RepeatLength=32 seed=TTAGCAGCGC  Num.seqs=5 Similarity=0.931313
30    CTACAAACTACA-A-GTTTTTGGGCGCTGCTAA-A
Rev.of_WB235_5:18989479-18989970 Satlength=492 Nr of Repeats=14
RepeatLength=32 seed=TGTAGTTTGT  Num.seqs=9 Similarity=0.905443
12    CTACAAACTACACA-A-TTTTGGGCGCTGCTAA-A

***********  *    ************* *
Consensus:

CTACAAACTACAAnTTTTGGGCGCTGCTaAgA

>WB235_Fam_24_20_11  Nr. of seq. 11 Alignment length(with gaps) = 21
Alignment score = 0.822222
WB235_1:1852567-1852667 Satlength=101 Nr of Repeats=5 RepeatLength=20
seed=CTAGGCAGAC  Num.seqs=5 Similarity=1.000000
0    CTAGGCAG-ACTTAGCCTACC
WB235_1:3427447-3429127 Satlength=1681 Nr of Repeats=81 RepeatLength=20
seed=GGCAGACCTA  Num.seqs=78 Similarity=0.844356
3    CTAGGCAG-ACCTAGCCTACC
Rev.of_WB235_3:5285020-5285610 Satlength=591 Nr of Repeats=28
RepeatLength=20 seed=CTAGGTCTGC  Num.seqs=25 Similarity=0.872444
14    CTAGGCAG-ACCTAGCCTACC
Rev.of_WB235_3:5285044-5285922 Satlength=879 Nr of Repeats=40
RepeatLength=20 seed=GGTAGGCTAG  Num.seqs=28 Similarity=0.844293
20    CTAGGCAG-ACCTAGCCTACC
WB235_6:1508523-1508943 Satlength=421 Nr of Repeats=18 RepeatLength=20
seed=AGGCAGACCT  Num.seqs=16 Similarity=0.885556
22    CTAGGCAG-ACCTAGCCTACC
WB235_5:904415-904569 Satlength=155 Nr of Repeats=8 RepeatLength=20
seed=GGCAGACCTA  Num.seqs=7 Similarity=0.809524
3    CTAGGCAG-ACCTAGTCTACC
WB235_3:2470723-2471262 Satlength=540 Nr of Repeats=27 RepeatLength=20
seed=GACCCAGCCT  Num.seqs=26 Similarity=0.980718
7    CCAGGCAG-ACCCAGCCTACC
WB235_3:2470728-2471526 Satlength=799 Nr of Repeats=40 RepeatLength=20
seed=AGCCTACCCC  Num.seqs=38 Similarity=0.909815
12    CCAGGCAG-ACCCAGCCTACC
WB235_5:904306-904460 Satlength=155 Nr of Repeats=7 RepeatLength=20
seed=CCTACCCCAG  Num.seqs=6 Similarity=0.871111
14    CCAGGCAG-ACCTAGCCTACC
WB235_1:1852477-1852561 Satlength=85 Nr of Repeats=4 RepeatLength=21
seed=CCTACTCTAG  Num.seqs=4 Similarity=1.000000
15    CTAGGCAGCCCCTAGCCTACT
Rev.of_WB235_6:15738820-15738988 Satlength=169 Nr of Repeats=8
RepeatLength=21 seed=GGCTGCCGAG  Num.seqs=8 Similarity=0.916100
31    CTCGGCAGCCCCTAGCCTACC

*  *****  *  ** ****
Consensus:

CtAGGCAGACCTAGCCTACC
```

>WB235_Fam_25_12_11 Nr. of seq. 11 Alignment length(with gaps) = 12
Alignment score = 0.746970
WB235_2:2151844-2151904 Satlength=61 Nr of Repeats=4 RepeatLength=11
seed=ACCGCAACGC Num.seqs=3 Similarity=1.000000
0   AC-CGCAACGCA
Rev.of_WB235_5:20605796-20606242 Satlength=447 Nr of Repeats=40
RepeatLength=11 seed=GTTGCGGTTG Num.seqs=39 Similarity=0.965976
8   AC-CGCAACGCA
Rev.of_WB235_3:1976102-1976245 Satlength=144 Nr of Repeats=11
RepeatLength=11 seed=GTCGCGTTGC Num.seqs=9 Similarity=0.885522
2   AC-CGCAACGCG
Rev.of_WB235_1:565592-565664 Satlength=73 Nr of Repeats=6 RepeatLength=12
seed=GCGTTGCGTG Num.seqs=6 Similarity=0.896296
4   ACGCGGCACGCA
WB235_1:2254186-2255835 Satlength=1650 Nr of Repeats=87 RepeatLength=12
seed=CACGCAACGC Num.seqs=80 Similarity=0.934001
6   ACGCAGCACGCA
Rev.of_WB235_1:11253589-11254333 Satlength=745 Nr of Repeats=60
RepeatLength=12 seed=CGTTGCGTGT Num.seqs=58 Similarity=0.901727
15   ACGCGACACGCA
WB235_3:12987113-12987245 Satlength=133 Nr of Repeats=7 RepeatLength=12
seed=CGCGACACGC Num.seqs=5 Similarity=0.822222
25   ACGCGACACGCA
Rev.of_WB235_4:4166741-4166830 Satlength=90 Nr of Repeats=7
RepeatLength=12 seed=GTTGCGTGTC Num.seqs=6 Similarity=0.962963
26   ACGCGACACGCA
Rev.of_WB235_4:4166744-4167306 Satlength=563 Nr of Repeats=46
RepeatLength=12 seed=GCGTGTCGCG Num.seqs=44 Similarity=0.893587
23   ACGCGACACGCA
WB235_5:17587204-17587343 Satlength=140 Nr of Repeats=10 RepeatLength=12
seed=ACGCGACACG Num.seqs=8 Similarity=0.972222
24   ACGCGACACGCA
WB235_5:17364710-17364818 Satlength=109 Nr of Repeats=9 RepeatLength=12
seed=CGCAACGCGA Num.seqs=9 Similarity=0.907407
8   ACGCGATACGCA

** *   ****
Consensus:

ACgCGacACGCA

>WB235_Fam_26_26_10 Nr. of seq. 10 Alignment length(with gaps) = 27
Alignment score = 0.768999
WB235_2:5097197-5097899 Satlength=703 Nr of Repeats=27 RepeatLength=26
seed=TTAGACCATT Num.seqs=27 Similarity=0.928117
0   TTAGACCATTAGATGAATTTC-CAAAG
Rev.of_WB235_4:4416113-4421995 Satlength=5883 Nr of Repeats=213
RepeatLength=26 seed=GTCTAACTTT Num.seqs=207 Similarity=0.933392
6   TTAGACCATTAGATGAATTTC-CAAAG
WB235_4:13115382-13116006 Satlength=625 Nr of Repeats=24 RepeatLength=26
seed=ATTAGATGAA Num.seqs=24 Similarity=0.974173
7   TTAGACCATTAGATGAATTTC-CAAAG

WB235_5:5073280-5076759 Satlength=3480 Nr of Repeats=131 RepeatLength=26
seed=AAAGTTAGAC Num.seqs=113 Similarity=0.928636
22   TTAGACCATTAGATGAATTTC-CAAAG
WB235_3:7594698-7597267 Satlength=2570 Nr of Repeats=98 RepeatLength=26
seed=ATTAGATGAA Num.seqs=93 Similarity=0.955479
7   TTAGACCATTAGATGAATTCC-CAAAG
Rev.of_WB235_6:15687867-15688727 Satlength=861 Nr of Repeats=33
RepeatLength=26 seed=AATTCATCTA Num.seqs=32 Similarity=0.950269
19   TTAGACCAGTAGATGAATTCC-CAAAG
WB235_6:11787527-11789550 Satlength=2024 Nr of Repeats=75 RepeatLength=26
seed=AAAGTTAGAC Num.seqs=72 Similarity=0.907026
22   TTAGACCATTAGATGAATTTA-TAAAG
Rev.of_WB235_6:15597064-15597272 Satlength=209 Nr of Repeats=8
RepeatLength=26 seed=GGTCTAACTT Num.seqs=8 Similarity=0.899267
7   TTAGACCGTCAGATCCATTTA-TAAAG
Rev.of_WB235_6:5913335-5915857 Satlength=2523 Nr of Repeats=97
RepeatLength=26 seed=TTCTAAATTT Num.seqs=97 Similarity=0.940589
6   TTAGAACGGTAGATGAATTTT-CAAAT
Rev.of_WB235_4:13464747-13465805 Satlength=1059 Nr of Repeats=13
RepeatLength=27 seed=TTTTTAAAAT Num.seqs=11 Similarity=0.892256
26   TTAGAACGGTATATGAATTTTAAAAAG

```
***** *   * **  ***    ***
```

Consensus:

TTAGAcCatTAGATGAATTtccAAAG

>WB235_Fam_27_21_10 Nr. of seq. 10 Alignment length(with gaps) = 23
Alignment score = 0.611433
WB235_6:920806-920909 Satlength=104 Nr of Repeats=5 RepeatLength=20
seed=ATGCACTGAC Num.seqs=3 Similarity=0.866667
0   AT-GCACTGACCAAAAATATG--
Rev.of_WB235_6:954503-954587 Satlength=85 Nr of Repeats=4 RepeatLength=21
seed=CAGTGCATTC Num.seqs=4 Similarity=0.830688
8   AT-GCACTGACCAAAGTTCTGA-
Rev.of_WB235_6:954523-954714 Satlength=192 Nr of Repeats=6
RepeatLength=21 seed=TCAGTGCATT Num.seqs=4 Similarity=0.735450
9   AT-GCACTGACCAAACTTCTGA-
Rev.of_WB235_6:1016701-1016827 Satlength=127 Nr of Repeats=6
RepeatLength=21 seed=GTCAGTGCAT Num.seqs=6 Similarity=0.944974
10   AT-GCACTGACCGAAACTATCA-
Rev.of_WB235_6:1022677-1022779 Satlength=103 Nr of Repeats=5
RepeatLength=20 seed=TTGGTCAGTG Num.seqs=3 Similarity=0.955556
13   AT-GCACTGACCAAGACTA-AA-
Rev.of_WB235_6:3693989-3694090 Satlength=102 Nr of Repeats=5
RepeatLength=20 seed=TTTGGTCAGT Num.seqs=4 Similarity=0.933333
14   AA-GCACTGACCAAA-ATA-GAA
WB235_6:863878-863962 Satlength=85 Nr of Repeats=4 RepeatLength=21
seed=ACTGACCAAA Num.seqs=4 Similarity=0.936508
25   AT-GCACTGACCAAAAATA-GAT
WB235_6:1296886-1296996 Satlength=111 Nr of Repeats=5 RepeatLength=22
seed=TCGCACTGAC Num.seqs=5 Similarity=0.915152
22   ATCGCACTGACCAAACTTA-GAA

Rev.of_WB235_6:703442-703568 Satlength=127 Nr of Repeats=5
RepeatLength=21 seed=GGTCAGTGCA Num.seqs=4 Similarity=0.904762
32   AT-GCACTGACCAAAATTG-GTA
Rev.of_WB235_6:1058514-1058619 Satlength=106 Nr of Repeats=4
RepeatLength=21 seed=GTCAGTGCAA Num.seqs=3 Similarity=0.661376
10   TT-GCACTGACCANNACTCTAA-

*********   *
Consensus:

ATGCACTGACCAAaanTatga

>WB235_Fam_28_163_9 Nr. of seq. 9 Alignment length(with gaps) = 170
Alignment score = 0.739924
WB235_1:976735-977538 Satlength=804 Nr of Repeats=5 RepeatLength=162
seed=ATCATATGAA Num.seqs=4 Similarity=0.781893
0   ATCATATGAAGCGATTCTTTTTTCTTTTATAAACCTGTTCAGAATTGGCAAAACTACCAGAAAATAC-
TAAACAAATTAGGCTAGCATGAACGGAAGTA-TTTTTT-A-AAAATTGAT-AAAAATA-ATGAAAAGCTG-A-
TATTTTCAAAATTCCAGAATGACGGAAAA
Rev.of_WB235_2:2050346-2051160 Satlength=815 Nr of Repeats=5
RepeatLength=162 seed=AGTTTTATGA Num.seqs=3 Similarity=0.983539
36
ATCATATAGAGTCATTCTTTTTTATTTCATAAAACTCTTCAGCATAGTCAAAAATACCAGAAAATGC-
TAAACAAAGTATAATAGTTTGTACGGAAGTA-TTTTTT-A-AAAATGGAT-AAAAATATAT-AAAAGCTG-A-
TTTTTTCAAAAATTCAAAAGTATGGGAAA
WB235_1:10851626-10855375 Satlength=3750 Nr of Repeats=23
RepeatLength=163 seed=GCATAGTCAA Num.seqs=23 Similarity=0.935660
41
ATCATATGGAGTCATTCTTTTTTATCTCAAAAAACTGTTCAGCATAGTCAAAAATACCAGCAAATAC-
CAAACAGAGTATATTAGCTTGTACGGAAGTA-TTTTTT-AGAAAATTGAT-AAAAATATAT-AAAAGCTG-A-
TTTTTTCAAAAATTCAAAAGTATGGGAAA
Rev.of_WB235_2:15186190-15187654 Satlength=1465 Nr of Repeats=9
RepeatLength=163 seed=AAATAAAAAA Num.seqs=8 Similarity=0.966696
190
ATCATATGGAGTCATTCTTTTTTATTTCATAAAACTGTTCAGCATAGTTAAAATTACCAGAATATGC-
CAATCAAAGTATATTAGCTTGTACGGAAGTATTTTTTT-A-AAAATTGAT-AAAAATATAT-AAAAGCTG-A-
TTTTTTCAAAAATTCAAAAGTATGGGAAA
WB235_3:10226211-10227863 Satlength=1653 Nr of Repeats=11
RepeatLength=164 seed=TATGGGAAAA Num.seqs=9 Similarity=0.973351
318
ATCATATGGAGTCATTCTTTTTTATTGCATAAAACTGTTCAGTATAGTCAAAAATACCAGAATATGC-
CAATCAAAGTATAATAGCTTGTACGGAAGTATTTTTTT-A-AAAATTGATAAAAAATATAT-AAAAGCTG-A-
TTTTTTCAAAAATTCAAAAGTATGGGAAA
Rev.of_WB235_5:16330598-16333224 Satlength=2627 Nr of Repeats=16
RepeatLength=164 seed=AAAAAATCAG Num.seqs=12 Similarity=0.959226
303
ATCATATGGAGTCATTCTTTTTTATTTCATAAAAATGTTCAGCATAGTCAAAAACACCAGAATATGC-
CAATCAAAGTATAATAGCTTGTACGGAAGTATTTTTTT-A-AAAATTGAT-AAAAATATAT-AAAATCTG-
ATTTTTTTCAAAAATTCAAAAGTCCTAGAAA
Rev.of_WB235_5:14586076-14591096 Satlength=5021 Nr of Repeats=31
RepeatLength=162 seed=TTCCGTACAA Num.seqs=29 Similarity=0.941779
95
ATCATATGAAGTCATTCTTTTTTATTTTATAGAACTGTTCAGCACAACCAAATATACCAGAAAATGC-

CAAACAAAGTATGCTAGCTTGTACGGAAGTTAATTTTT-A-AAAATTCAG---AAATTAACCAGAAGCTA-T-
TATTTTCAAAAATTCAAAAGTAAGGGAAA
Rev.of_WB235_2:2361743-2363537 Satlength=1795 Nr of Repeats=9
RepeatLength=163 seed=TGAATTTTTG Num.seqs=7 Similarity=0.906387
150
ATCATATGAAGTCATTCTTTTTTATTTTATAAAACTGTTCAGCATAGTCAAATGTACCAGAAAATACTTAAAA
AAAG-GTGCTAGCTCGTACGGAAGTTTATTTTTAA-AAAATGCAG---AATTTAACCAAAAGCTG-T-
TATTTTCAAAAATTCAAAAGTACCTCAAA
WB235_4:2573920-2576033 Satlength=2114 Nr of Repeats=13 RepeatLength=164
seed=CTTTTTTCTT Num.seqs=12 Similarity=0.979921
180
ATCATAAAAAGTGCTTCTTTTTTCTTTTATAACACTGTTTAGCATAGTCAAATATACCAGAAAATAC-
CAAACAAAGTATGCTATCTTGTACGAAAGATTATTTTA-A-AAAATTCAG--AAAATGCATCTAAAGCGGTT-
TTTTTTCAAAAATTCAAAAGTACCGGAAA

```
******   **   ********* *   * *    * ** ** *     ***   ***** * ** *  **
* *      **    * *** ***    ****  * *****  *    ** *  *    ** *     *
********* * ** **       ***
```

Consensus:

ATCATATggAGTcATTCTTTTTTaTTTcATAAAACTGTTCAGcATAGtCAAAaaTACCAGAAaATgCcAAaCA
AAGTATanTAGCTTGTACGGAAGTattTTTTTAAAAATtgAtaaAAATatAtAAAAGCTGaTtTTTTCAAAAA
TTCAAAAGTAngggAAA

>WB235_Fam_29_42_9 Nr. of seq. 9 Alignment length(with gaps) = 42
Alignment score = 0.733686
WB235_1:961892-962564 Satlength=673 Nr of Repeats=16 RepeatLength=42
seed=CCGGAAATTT Num.seqs=16 Similarity=0.888889
0   CCGGAAATTTCGAAAACCGGCAATTGCCGAAATTGCCGATTG
WB235_2:3572146-3572433 Satlength=288 Nr of Repeats=6 RepeatLength=42
seed=TTTGAAAACC Num.seqs=4 Similarity=0.851852
8   CCGGAAATTTTGAAAACCGGCAATAGCCGAAATTGCCGAGTG
Rev.of_WB235_5:20315897-20316651 Satlength=755 Nr of Repeats=12
RepeatLength=42 seed=ATTGCCGGTT Num.seqs=9 Similarity=0.803351
24   CCGAAAATTTTAAAAACCGGCAATTGCCGAAATTGCCGATTG
WB235_5:3816982-3817234 Satlength=253 Nr of Repeats=6 RepeatLength=42
seed=ACCGGCAATT Num.seqs=6 Similarity=0.881481
15   CTGAAAATTTCCAAAACCGGCAATTGCCAAAATTGCCGATTG
WB235_2:14192423-14193096 Satlength=674 Nr of Repeats=16 RepeatLength=42
seed=AAAACCGGCA Num.seqs=10 Similarity=0.749832
12   CCGGNAATATTAAAAACCGGCAATTGCCGGAATTGCCGATTG
Rev.of_WB235_2:1630503-1630898 Satlength=396 Nr of Repeats=5
RepeatLength=42 seed=TTGCCGGTTG Num.seqs=3 Similarity=0.809524
23   CAAAAAATTTCGGCAACCGGCAATTGCCGAAGTTGCCGAACC
WB235_2:1635206-1635601 Satlength=396 Nr of Repeats=5 RepeatLength=42
seed=CAACCGGCAA Num.seqs=3 Similarity=0.809524
55   CAAAAAATTTCGGCAACCGGCAATTGCCGAAGTTGCCGAACC
WB235_5:16191704-16191915 Satlength=212 Nr of Repeats=6 RepeatLength=42
seed=TTTCGGCAAC Num.seqs=4 Similarity=0.899471
49   CGAAAAATTTCGGCAACCGGCAATTGCCGATGTTGCCGAACC
WB235_2:12577021-12577399 Satlength=379 Nr of Repeats=8 RepeatLength=42
seed=TTGCCGAACC Num.seqs=7 Similarity=0.797619
32   GTAAAAATTTCGGCAACCNGGAATTGCCAAAGTTGCCGAACC

*** *   **** * *** ***   *******
Consensus:

CngaAAATTTcgaaAACCGGCAATTGCCgAAaTTGCCGAntg

>WB235_Fam_30_31_9 Nr. of seq. 9 Alignment length(with gaps) = 37
Alignment score = 0.708709
WB235_3:2110682-2111451 Satlength=770 Nr of Repeats=22 RepeatLength=31
seed=ATTTCCCGCC Num.seqs=14 Similarity=0.922486
0   ATTTCCCGCCAAAAAATA---TTT---GAAAATTTGA
Rev.of_WB235_5:18169395-18171299 Satlength=1905 Nr of Repeats=61
RepeatLength=31 seed=GTCAATTTTT Num.seqs=48 Similarity=0.882004
20   ATTTCCCGCCAAAAATTG---ACT---GAAAATTTGA
WB235_5:19535226-19535475 Satlength=250 Nr of Repeats=6 RepeatLength=31
seed=AATTTCCCGC Num.seqs=5 Similarity=0.756250
30   ATTTCCCGCCAAAAATTG---ACT---GAAAATTTGA
Rev.of_WB235_5:19630875-19631274 Satlength=400 Nr of Repeats=12
RepeatLength=31 seed=GGCGGGAAAT Num.seqs=8 Similarity=0.930876
41   ATTTCCCGCCAAAAATTG---ACT---GAAAATTTGA
WB235_5:19689942-19690431 Satlength=490 Nr of Repeats=10 RepeatLength=31
seed=AAAATTTGAA Num.seqs=9 Similarity=0.942652
22   ATTTCCCGCCAAAAATTG---ACT---GAAAATTTGA
Rev.of_WB235_5:19376610-19376889 Satlength=280 Nr of Repeats=9
RepeatLength=31 seed=CGGGAAATTC Num.seqs=7 Similarity=0.774706
39   ATTTCCCGCCAAAAATTA---ACT---GAAAATTTGA
Rev.of_WB235_5:19615691-19616051 Satlength=361 Nr of Repeats=8
RepeatLength=31 seed=TTCAAATTTT Num.seqs=7 Similarity=0.819764
32   AATTCCCGCCAAAAATTG---ACT---GAAAATTTGA
WB235_5:19863556-19864754 Satlength=1199 Nr of Repeats=28 RepeatLength=31
seed=TTCCCGCCTA Num.seqs=17 Similarity=0.854243
2   ATTTCCCGCCTAAAACT----ACTC--GAAAATTCAA
Rev.of_WB235_5:18666434-18666663 Satlength=230 Nr of Repeats=6
RepeatLength=35 seed=GGAAATTCAA Num.seqs=4 Similarity=0.776235
6   ATTTTCCGCCAAAAAATATTTTCT-CCGAAAATTTGA

* ** ***** **** *   *   ******* *
Consensus:

ATTTCCCGCCAAAAAtTgaCTGAAAATTTGA

>WB235_Fam_31_26_9 Nr. of seq. 9 Alignment length(with gaps) = 26
Alignment score = 0.798789
WB235_4:11610541-11615517 Satlength=4977 Nr of Repeats=199
RepeatLength=25 seed=CATTACGGCA Num.seqs=198 Similarity=0.948255
0   CATTACGGCAGATTCTA-TAAATTGG
Rev.of_WB235_4:14209284-14209609 Satlength=326 Nr of Repeats=13
RepeatLength=25 seed=TAATGCCAAT Num.seqs=13 Similarity=0.948034
5   CATTACGGCAGATTCTA-TAAATTGG
Rev.of_WB235_6:11441051-11442751 Satlength=1701 Nr of Repeats=68
RepeatLength=25 seed=CCGTAATGCC Num.seqs=68 Similarity=0.907381
8   CATTACGGCAGATTCTA-TAAATTGG

Rev.of_WB235_1:4540371-4544820 Satlength=4450 Nr of Repeats=169 RepeatLength=26 seed=AAATCCGCCG Num.seqs=166 Similarity=0.908932
15 CATTGCGGCGGATTTTAGAAAAATGG
Rev.of_WB235_6:1644418-1644678 Satlength=261 Nr of Repeats=10 RepeatLength=26 seed=GCCGCAATGC Num.seqs=10 Similarity=0.971510
35 CATTGCGGCGGATTTTAGAAAAATGG
WB235_6:11442777-11443790 Satlength=1014 Nr of Repeats=39 RepeatLength=26 seed=CGGCGGATTT Num.seqs=38 Similarity=0.921509
31 CATTGCGGCGGATTTTAGAAAAATGG
WB235_1:10953026-10953491 Satlength=466 Nr of Repeats=4 RepeatLength=26 seed=TTTTTGAAAA Num.seqs=3 Similarity=0.931624
38 CATTGCGGCGGATTTTTGAAAAATGG
WB235_4:12730503-12732683 Satlength=2181 Nr of Repeats=84 RepeatLength=26 seed=CATTGCGGCG Num.seqs=80 Similarity=0.917965
26 CATTGCGGCGGATTTCAGAAAAATGG
WB235_6:12607668-12607975 Satlength=308 Nr of Repeats=5 RepeatLength=26 seed=AAAAATGGCA Num.seqs=4 Similarity=0.897436
44 CATTGCGGCGGATTTCAGAAAAATGG

**** **** ****   *** ***
Consensus:

CATTgCGGCgGATTttAgaAAAaTGG

>WB235_Fam_32_18_9 Nr. of seq. 9 Alignment length(with gaps) = 23 Alignment score = 0.628019
WB235_1:11485772-11485879 Satlength=108 Nr of Repeats=5 RepeatLength=19 seed=TCAAAAATCA Num.seqs=4 Similarity=0.873611
21 CA--TC-AAAAATCAATAA-TTT
WB235_1:11383715-11384049 Satlength=335 Nr of Repeats=14 RepeatLength=20 seed=AAAAATCAAT Num.seqs=11 Similarity=0.883636
24 CA--TCGAAAAATCAATAA-TTT
WB235_1:11485735-11486997 Satlength=1263 Nr of Repeats=20 RepeatLength=19 seed=AAAAATCGAT Num.seqs=12 Similarity=0.725830
23 CN--TC-AAAAATCGATAA-TTT
WB235_2:2623068-2623182 Satlength=115 Nr of Repeats=6 RepeatLength=19 seed=AAAATCGATA Num.seqs=6 Similarity=0.601058
24 CA--TC-GAAAATCGATAA-TTT
WB235_1:11532642-11533117 Satlength=476 Nr of Repeats=17 RepeatLength=19 seed=TTTCACAAAA Num.seqs=12 Similarity=0.943647
35 CA---C-AAAAATCAATAATTTT
WB235_1:11531919-11534041 Satlength=2123 Nr of Repeats=45 RepeatLength=19 seed=AAAATCGATA Num.seqs=29 Similarity=0.730706
42 CA---N-AAAAATCGATAATTTT
WB235_4:977829-978532 Satlength=704 Nr of Repeats=35 RepeatLength=19 seed=CAATAATTTC Num.seqs=27 Similarity=0.905233
28 CC-C-A-AAAAATCAATAA-TTT
Rev.of_WB235_1:11384374-11386353 Satlength=1980 Nr of Repeats=95 RepeatLength=20 seed=ATTGATTTTT Num.seqs=91 Similarity=0.879707
32 CCTC-A-AAAAATCAATAA-TTT
Rev.of_WB235_1:2364706-2365240 Satlength=535 Nr of Repeats=20 RepeatLength=20 seed=TTATTGATTT Num.seqs=12 Similarity=0.816162
34 CCTA-G-AAAAATCAATAA-TGT

```
****** **** * **
Consensus:

cAAAAATCaATAATTTCa

>WB235_Fam_33_18_9  Nr. of seq. 9 Alignment length(with gaps) = 19
Alignment score = 0.838207
WB235_5:18881944-18883813 Satlength=1870 Nr of Repeats=74 RepeatLength=17
seed=ATTAGCAGCG  Num.seqs=48 Similarity=0.873325
0    -ATTAGCAGCGCCCAAAA-
WB235_5:18966918-18967045 Satlength=128 Nr of Repeats=5 RepeatLength=18
seed=GCAGCGCCCA  Num.seqs=3 Similarity=0.950617
4    -ATTAGCAGCGCCCAAAAA
Rev.of_WB235_5:18970132-18970454 Satlength=323 Nr of Repeats=18
RepeatLength=18 seed=TGCTAATTTT  Num.seqs=16 Similarity=0.939506
7    -ATTAGCAGCGCCCAAAAA
Rev.of_WB235_5:18979816-18980485 Satlength=670 Nr of Repeats=37
RepeatLength=18 seed=GCGCTGCTAA  Num.seqs=25 Similarity=0.926420
11    -ATTAGCAGCGCCCAAAAA
WB235_5:18987130-18987201 Satlength=72 Nr of Repeats=4 RepeatLength=18
seed=TAGCAGCGCC  Num.seqs=3 Similarity=0.901235
2    -ATTAGCAGCGCCCAAGAA
WB235_5:18886295-18887294 Satlength=1000 Nr of Repeats=12 RepeatLength=18
seed=AATTAGCAGC  Num.seqs=10 Similarity=0.960494
0    AATTAGCAGCGACCAAAA-
WB235_5:19106243-19106368 Satlength=126 Nr of Repeats=6 RepeatLength=18
seed=ATTAGCAGCG  Num.seqs=4 Similarity=1.000000
1    AATTAGCAGCGACCAAAA-
Rev.of_WB235_5:19193880-19195207 Satlength=1328 Nr of Repeats=74
RepeatLength=18 seed=GCTGCTAATT  Num.seqs=61 Similarity=0.900627
10    AATTAGCAGCGACCAAAA-
WB235_5:18964514-18964623 Satlength=110 Nr of Repeats=6 RepeatLength=18
seed=AGCAGCGCCC  Num.seqs=5 Similarity=0.896296
3    -AATAGCAGCGCCCAAAAA

* ******** **** *
Consensus:

ATTAGCAGCGcCCAAAAa

>WB235_Fam_34_12_9  Nr. of seq. 9 Alignment length(with gaps) = 15
Alignment score = 0.680247
WB235_6:2263547-2263756 Satlength=210 Nr of Repeats=16 RepeatLength=11
seed=CCATACCTAT  Num.seqs=13 Similarity=1.000000
0    -CC-ATACCT-ATA-
WB235_1:12620508-12620574 Satlength=67 Nr of Repeats=5 RepeatLength=12
seed=TACCTATACC  Num.seqs=4 Similarity=0.944444
3    -CT-ATACCT-ATAC
WB235_1:13689387-13689549 Satlength=163 Nr of Repeats=13 RepeatLength=12
seed=TATACCTATA  Num.seqs=10 Similarity=0.955556
7    -CT-ATACCT-ATAC
```

Rev.of_WB235_1:13689822-13689960 Satlength=139 Nr of Repeats=11 RepeatLength=12 seed=ATAGGTATAG Num.seqs=10 Similarity=0.955556
4   -CT-ATACCT-ATAC
Rev.of_WB235_2:3490327-3491001 Satlength=675 Nr of Repeats=54 RepeatLength=12 seed=TAGGTATAGG Num.seqs=45 Similarity=0.881481
3   -CT-ATACCT-ATAC
WB235_2:12353539-12353707 Satlength=169 Nr of Repeats=14 RepeatLength=12 seed=CCTATACCTA Num.seqs=14 Similarity=0.968254
5   -CT-ATACCT-ATAC
WB235_4:13222198-13222342 Satlength=145 Nr of Repeats=12 RepeatLength=12 seed=TATACCTATA Num.seqs=12 Similarity=0.944444
7   -CT-ATACCT-ATAC
WB235_2:3048248-3048472 Satlength=225 Nr of Repeats=15 RepeatLength=14 seed=CTTATACCTT Num.seqs=14 Similarity=0.947671
1   CCTTATACCTTATA-
WB235_3:1334409-1335297 Satlength=889 Nr of Repeats=40 RepeatLength=14 seed=ATACCTAATA Num.seqs=28 Similarity=0.877047
4   CCTAATACCTAATA-

* ****** ***
Consensus:

CTATACCTATAc

>WB235_Fam_35_14_9 Nr. of seq. 9 Alignment length(with gaps) = 14 Alignment score = 0.904762
WB235_1:10291633-10291724 Satlength=92 Nr of Repeats=6 RepeatLength=14 seed=GCTATACGCT Num.seqs=5 Similarity=1.000000
0   GCTATACGCTATAC
WB235_2:1455578-1455634 Satlength=57 Nr of Repeats=4 RepeatLength=14 seed=TACGCTATAC Num.seqs=4 Similarity=0.952381
4   GCTATACGCTATAC
WB235_2:6185280-6186470 Satlength=1191 Nr of Repeats=85 RepeatLength=14 seed=ACGCTATACG Num.seqs=85 Similarity=1.000000
5   GCTATACGCTATAC
Rev.of_WB235_4:14209637-14209721 Satlength=85 Nr of Repeats=6 RepeatLength=14 seed=AGCGTATAGC Num.seqs=6 Similarity=1.000000
3   GCTATACGCTATAC
Rev.of_WB235_6:316974-317093 Satlength=120 Nr of Repeats=8 RepeatLength=14 seed=ATAGCGTATA Num.seqs=7 Similarity=0.891156
5   GCTATACGCTATAC
WB235_6:11440976-11441039 Satlength=64 Nr of Repeats=4 RepeatLength=14 seed=CTATACGCTA Num.seqs=3 Similarity=0.936508
1   GCTATACGCTATAC
WB235_5:12510458-12510577 Satlength=120 Nr of Repeats=8 RepeatLength=14 seed=CTATACGCTA Num.seqs=7 Similarity=0.891156
1   GCTATACGCTATGC
Rev.of_WB235_6:16886473-16886932 Satlength=460 Nr of Repeats=20 RepeatLength=14 seed=ATAGCGCATA Num.seqs=18 Similarity=0.808279
5   GCTATGCGCTATGC
WB235_4:14208040-14209239 Satlength=1200 Nr of Repeats=70 RepeatLength=14 seed=GCTATGCGCT Num.seqs=52 Similarity=0.786869
0   GCTATGCGCTGTAC

```
***** **** * *
Consensus:

GCTATaCGCTATaC

>WB235_Fam_36_12_9  Nr. of seq. 9 Alignment length(with gaps) = 12
Alignment score = 0.876543
WB235_1:765668-765992 Satlength=325 Nr of Repeats=27 RepeatLength=12
seed=TCTAGGTCTA  Num.seqs=27 Similarity=0.944286
0    TCTAGGTCTAGG
WB235_1:991239-991341 Satlength=103 Nr of Repeats=8 RepeatLength=12
seed=GGTCTAGGTC  Num.seqs=7 Similarity=0.936508
4    TCTAGGTCTAGG
WB235_1:11484951-11485029 Satlength=79 Nr of Repeats=6 RepeatLength=12
seed=TAGGTCTAGG  Num.seqs=5 Similarity=1.000000
2    TCTAGGTCTAGG
Rev.of_WB235_2:878150-878348 Satlength=199 Nr of Repeats=15
RepeatLength=12 seed=ACCTAGACCT  Num.seqs=12 Similarity=0.858586
1    TCTAGGTCTAGG
WB235_1:942750-943044 Satlength=295 Nr of Repeats=24 RepeatLength=12
seed=CTAAGTCTAA  Num.seqs=23 Similarity=1.000000
1    TCTAAGTCTAAG
WB235_1:942557-943381 Satlength=825 Nr of Repeats=46 RepeatLength=12
seed=TAAGCCTAAG  Num.seqs=40 Similarity=0.768310
2    TCTAAGTCTAAG
WB235_1:3258597-3259299 Satlength=703 Nr of Repeats=58 RepeatLength=12
seed=CTAAGTCTAA  Num.seqs=57 Similarity=0.888053
1    TCTAAGTCTAAG
Rev.of_WB235_3:12196315-12196495 Satlength=181 Nr of Repeats=14
RepeatLength=12 seed=CTTAGACTTA  Num.seqs=12 Similarity=0.929293
6    TCTAAGTCTAAG
WB235_5:15009961-15010009 Satlength=49 Nr of Repeats=4 RepeatLength=12
seed=TCTAAGTCTA  Num.seqs=4 Similarity=0.944444
6    TCTAAGTCTAAG

**** ***** *
Consensus:

TCTAaGTCTAaG

>WB235_Fam_37_21_8  Nr. of seq. 8 Alignment length(with gaps) = 23
Alignment score = 0.616977
WB235_2:463793-464368 Satlength=576 Nr of Repeats=9 RepeatLength=20
seed=CAATTTTTGA  Num.seqs=7 Similarity=0.942857
17    TT-TTTGAGTTAAAAAT-T-CAA
Rev.of_WB235_2:13318641-13320189 Satlength=1549 Nr of Repeats=49
RepeatLength=20 seed=TTTGAATTTT  Num.seqs=35 Similarity=0.825770
21    AT-TTTGACTTAAAAAT-T-CAA
Rev.of_WB235_3:1934627-1936118 Satlength=1492 Nr of Repeats=61
RepeatLength=21 seed=TGGAAATTTT  Num.seqs=49 Similarity=0.853795
19    TT-TTTGGGTTAAAATT-TCCAA
```

WB235_3:1560064-1560620 Satlength=557 Nr of Repeats=7 RepeatLength=21
seed=CCTAAAAATT Num.seqs=5 Similarity=0.695238
27   TT-TTTNACCTAAAAAT-TNCAA
WB235_3:1536002-1536441 Satlength=440 Nr of Repeats=21 RepeatLength=20
seed=TTTCACTAAA Num.seqs=20 Similarity=0.889265
22   AA-TTTCAC-TAAAAATCT-CAA
Rev.of_WB235_4:16425000-16425220 Satlength=221 Nr of Repeats=8
RepeatLength=20 seed=TTTTAGTGAA Num.seqs=5 Similarity=1.000000
33   AT-TTTCAC-TAAAAATCT-CAA
WB235_5:20494724-20495104 Satlength=381 Nr of Repeats=15 RepeatLength=20
seed=TTTCACTAAA Num.seqs=11 Similarity=0.741991
22   AT-TTTCAC-TAAAAATTT-CAA
Rev.of_WB235_2:2897150-2899742 Satlength=2593 Nr of Repeats=100
RepeatLength=21 seed=CCAAAAATTG Num.seqs=87 Similarity=0.735669
26   TTTTTGGAC-TAAAAATCT-CAA

***   **   ***** * *
Consensus:

CAAaTTTTgAcnTAAAAATnT

>WB235_Fam_38_18_8 Nr. of seq. 8 Alignment length(with gaps) = 19
Alignment score = 0.681391
WB235_4:640232-640313 Satlength=82 Nr of Repeats=4 RepeatLength=15
seed=TAAGCCTAAG Num.seqs=3 Similarity=1.000000
0   TAAGCCTA-AGCC-CA-C-
WB235_5:1848647-1848932 Satlength=286 Nr of Repeats=14 RepeatLength=17
seed=TAAGCCTAAG Num.seqs=9 Similarity=0.930283
0   TAAGCCTA-AG-CTTAGCT
Rev.of_WB235_3:635824-635934 Satlength=111 Nr of Repeats=6
RepeatLength=18 seed=TAGGCTTAGG Num.seqs=4 Similarity=0.527778
8   TAAGCCTACAG-CTAAGCC
WB235_2:368393-368601 Satlength=209 Nr of Repeats=10 RepeatLength=18
seed=TAAGCCTAAG Num.seqs=6 Similarity=0.619753
12   TAAGCCTA-AGTCTAAGCC
WB235_2:12942997-12943139 Satlength=143 Nr of Repeats=5 RepeatLength=18
seed=CCTAAGCCTA Num.seqs=3 Similarity=0.660819
16   TAAGCCTA-AGNCTAAACC
WB235_5:1047118-1047214 Satlength=97 Nr of Repeats=6 RepeatLength=18
seed=AAGCCTAAGC Num.seqs=4 Similarity=0.691358
19   TAAGCCTA-AGCCTAAACC
WB235_5:1661122-1661200 Satlength=79 Nr of Repeats=5 RepeatLength=18
seed=GCCTAAGCCT Num.seqs=3 Similarity=0.614035
21   TANGCCTA-AGCCTAAGCA
Rev.of_WB235_2:3459359-3459467 Satlength=109 Nr of Repeats=5
RepeatLength=18 seed=GGCTTAGGCT Num.seqs=3 Similarity=0.901235
12   TAAGCCTA-AGCCTGAGCT

** ***** ** * * *
Consensus:

TAAGCCTAAGcCTaAgCc

>WB235_Fam_39_10_8 Nr. of seq. 8 Alignment length(with gaps) = 11
Alignment score = 0.758117
WB235_2:4207582-4207842 Satlength=261 Nr of Repeats=25 RepeatLength=10
seed=GCGTTAGCAG Num.seqs=24 Similarity=0.977778
0   GCGTTAGCAG-
WB235_5:842648-842948 Satlength=301 Nr of Repeats=24 RepeatLength=10
seed=GGCGTCAGCG Num.seqs=18 Similarity=0.893682
9   GCGTCAGCGG-
WB235_5:1850026-1850346 Satlength=321 Nr of Repeats=23 RepeatLength=10
seed=GGGCGTCAGC Num.seqs=15 Similarity=0.949206
18   GCGTCAGCGG-
Rev.of_WB235_2:13790809-13791073 Satlength=265 Nr of Repeats=22
RepeatLength=10 seed=CCGCTACCGC Num.seqs=18 Similarity=1.000000
0   GCGGTAGCGG-
Rev.of_WB235_3:13032223-13032864 Satlength=642 Nr of Repeats=39
RepeatLength=10 seed=TACCGCCCGC Num.seqs=24 Similarity=0.944444
6   GCGGTAGCGG-
Rev.of_WB235_5:2777566-2778653 Satlength=1088 Nr of Repeats=65
RepeatLength=10 seed=TACCGCCCGC Num.seqs=59 Similarity=0.973972
6   GCGGTAGCGG-
WB235_5:3145392-3145472 Satlength=81 Nr of Repeats=6 RepeatLength=10
seed=GCGGGCGGTA Num.seqs=4 Similarity=0.933333
6   GCGGTAGCGG-
Rev.of_WB235_2:13795316-13795382 Satlength=67 Nr of Repeats=5
RepeatLength=11 seed=CCGCTACCGC Num.seqs=4 Similarity=0.919192
0   GCGGTAGCGGT

*** *** *
Consensus:

GCGgtAGCGG

>WB235_Fam_40_166_7 Nr. of seq. 7 Alignment length(with gaps) = 173
Alignment score = 0.741077
WB235_6:15929033-15929676 Satlength=644 Nr of Repeats=4 RepeatLength=158
seed=TATCAATTTT Num.seqs=3 Similarity=0.924051
0   TATCAATTTTCAGACGCGCTACGGGGGTTTTTGTCTTGAATTCCAATTTCAAC-----------
AGGCGCAAAAAAACAAAAATAAAATTTTTGGATT-TTTGGAAAAGT-TTTT-
ATAGATTTCTATTCTAGATTGTTTAA-AAATATTATTGGTACGTTTTTGTACCAANA
WB235_6:15929110-15929778 Satlength=669 Nr of Repeats=4 RepeatLength=158
seed=CAAAAATAAA Num.seqs=3 Similarity=0.935302
66   TATCAATTTTCAGACGCGCTACGGGGGTTTTTGTCTTGAATTCCAATTTCAAC-----------
AGGCGCAAAAAAACAAAAATAAAATTTTTGGATT-TTTGGAAAAGT-TTTT-
ATAGATTTGTATTCTAGATTGTTTAA-AAATATTATTGGTACGTTTTTGTACCAANA
Rev.of_WB235_3:2077634-2082512 Satlength=4879 Nr of Repeats=29
RepeatLength=168 seed=TTTATTTTTG Num.seqs=28 Similarity=0.979634
86   TATCCATTTTCAGACGCGCTACAGGGGTTTTTGTCTTGAATTCCAATTTCAACAGGTA-
TATGTAGGCACAAAAAAACAAAAATAAACATTTTGGAAT-TCTGGA-AA-
TGTTTTGATAGATTTGGATTCTAGATTGTTTTA-AAATATTATTGGTAAGTTTTTGTACCTAGA
WB235_4:15225095-15227378 Satlength=2284 Nr of Repeats=12
RepeatLength=168 seed=AAAAACAAAA Num.seqs=9 Similarity=0.953704
239   TATCAATTTTCAGATGCGCTAGAGGGGTTTTTGTCTTGAATTCCAATTTCAACAGATA-
TATACAGGCACAAAAAAACAAAAATAAAAATTTTGGAATGTTGGGAGAA-T-TTTT-
ATAGATTTGGATTCTAGATT-TTTAACAAATATTTTTGGTAAGTTTCTGGACCTAGA

WB235_4:15225869-15227795 Satlength=1927 Nr of Repeats=11
RepeatLength=168 seed=AACAAAAATA Num.seqs=9 Similarity=0.953704
242   TATCAATTTTCAGATGCGCTAGAGGGGTTTTTGTCTTGAATTCCAATTTCAACAGATA-
TATACAGGCACAAAAAAACAAAAATAAAAATTTTGGAATGTTGGGAGAA-T-TTTT-
ATAGATTTGGATTCTAGATT-TTTAACAAATATTTTTGGTAAGTTTCTGGACCTAGA
Rev.of_WB235_4:12931758-12932786 Satlength=1029 Nr of Repeats=5
RepeatLength=168 seed=TTTTTAAAAA Num.seqs=3 Similarity=0.984127
141   AAGTAAATTTAAGCCGCGCTAGAGAGGTTTTTGTGTCGAGTTCCAATTTTCACAGGTACTA-
GTAGGCTCAAAAAAACAAAAATAAAAATTATCGAAT-TCCGGA-AA-
TGTTTTGATAGATTTGGATTCTAGATTTTTTAA-AAATATTATTGGTAAGTTTGTGGGCCAGGA
WB235_4:15511605-15513283 Satlength=1679 Nr of Repeats=10
RepeatLength=168 seed=GAATTCCGGA Num.seqs=8 Similarity=0.944161
261   AAGTAACTTAAAGCCGCGCTAGAAAGGTTTTTGTGTCGAGTTACAATTTTCACAGGTACTA-
GTAGGCGCAAAAAAACAAAAATAAAAATTATGGAAT-TCCGGA-AA-
TGTTTTGATAGATTTGGATTCTAGATTTTTTAA-AAATATTATAGGTGAGTTTTTGGGACAGGA

*   * ** ** ******   ********* * ** ** ****** **   ****
***************** ** * ** * * *** ** * **** ******** ********** *** *
******* * *** **** **   *   *
Consensus:

tAtcAAtTTTcAGacGCGCTAgaGgGGTTTTTGTcTtGAaTTCCAATTTcaACagntatannAGGCnCAAAAA
AACAAAAATAAAAaTTtTGGAaTTtnGGAnAATTTTTATAGATTTGgATTCTAGATTnTTTAAAAATATTaTT
GGTAaGTTTtTGgaCCaagA

>WB235_Fam_41_95_7 Nr. of seq. 7 Alignment length(with gaps) = 96
Alignment score = 0.868386
WB235_4:13734177-13736953 Satlength=2777 Nr of Repeats=30 RepeatLength=93
seed=AGGGCCGCAG Num.seqs=27 Similarity=0.958174
0   AGGGCCGCAGGCCCGAATAGTCTTGGAAACT--GGGCTGGCTATAATCCTATTCCAAGCAGGT-
TGTTATTCACTCAAGCCTATCAACCAAGTTTG
Rev.of_WB235_2:2712830-2713528 Satlength=699 Nr of Repeats=7
RepeatLength=95 seed=TTCGGGCCTG Num.seqs=6 Similarity=0.981287
17   AGGGCCGCAGGCCCGAATACTCATGGAAACTGTGGGCCAGCTTTAATCCTATTCCAAGCAGAT-
TGTTATTCGCTCAAGCCTACAAACCAGGTTTG
WB235_3:3385070-3386871 Satlength=1802 Nr of Repeats=19 RepeatLength=95
seed=TAATCCTATT Num.seqs=13 Similarity=0.948178
43   AGGGCCGCAGGCCCGAATACTCATGGAAACTGTGGGCCAGCTTTAATCCTATTCCAAGCAGAT-
TGTTATTCGCTCAAGCCTACAAACCAGGTTTG
Rev.of_WB235_2:13385641-13386400 Satlength=760 Nr of Repeats=8
RepeatLength=95 seed=GGCCTGCGGC Num.seqs=5 Similarity=0.859649
108   AGGGCCGCAGGCCCGAATACTCATGGAAACTAAGGGCCAGCTTTAATCCTATTCCAAGCAGAT-
TGTTATTCGCTCAAGCCTACCAACCAGGTTTG
Rev.of_WB235_2:1564655-1565350 Satlength=696 Nr of Repeats=7
RepeatLength=96 seed=GGCCTGCGGC Num.seqs=6 Similarity=0.968518
109
AGGGCCGCAGGCCCGAACACTCTTGGAAACTATGGGCTGGCTTTGATCCTATTCTAAGCAGATATGTTATTCA
CTCAAGCCTAACAACCAGGTTCG
WB235_3:1119657-1120064 Satlength=408 Nr of Repeats=4 RepeatLength=96
seed=GAGGGCCGCA Num.seqs=3 Similarity=0.949074
191
AGGGCCGCAGGCCCGAACACTCTTGGAAACTATGGGCTGGCTTTGATCCTATTCTAAGCAGATATGTTATTCA
CTCAAGCCTAATAACCAGGTTNG

Rev.of_WB235_5:5415371-5416162 Satlength=792 Nr of Repeats=8
RepeatLength=96 seed=TCTGCTTAGA Num.seqs=7 Similarity=0.949735
254
AGGGCCGCAGGCCCGAACACTCTTGGAAACTATGGGCTGGCTTTGATCCTATTCTAAGCAGATATGTTATTCA
CTCAAGCCTAATAACCAGGTTTG

***************** * ** ******** **** *** * ********* ****** * ********
********** ***** *** *
Consensus:

AGGGCCGCAGGCCCGAAtACTCtTGGAAACTatGGGCtgGCTTTaATCCTATTCcAAGCAGATTGTTATTCaC
TCAAGCCTAnnAACCAGGTTtG

>WB235_Fam_42_94_7 Nr. of seq. 7 Alignment length(with gaps) = 94
Alignment score = 0.948666
WB235_1:4281487-4294537 Satlength=13051 Nr of Repeats=139 RepeatLength=94
seed=TCAAAATCTT Num.seqs=123 Similarity=0.961982
0
TCAAAATCTTCTGTTCTCTAAATATTGGGTGCTTTTCGATGCCCTATGTAGACAAATCAATGGGAAAATCATC
AATTTCTGAAGGCAGTAATTC
Rev.of_WB235_3:7405401-7443206 Satlength=37806 Nr of Repeats=414
RepeatLength=94 seed=CAATATTTAG Num.seqs=380 Similarity=0.958602
27
TCAAAATCTTCTGTTCTCTAAATATTGGGTGCTTTTCGATGCCCTATGTAGACAAATCAATGGGAAAATCATC
AATTTCTGGAGGCAGTAATTC
Rev.of_WB235_3:7448277-7452238 Satlength=3962 Nr of Repeats=40
RepeatLength=94 seed=ACCCAATATT Num.seqs=39 Similarity=0.958308
30
TCAAAATCTTCTGTTCTCTAAATATTGGGTGCTTTTCGATGCCCTATGTAGACAAATCAATGGGAAAATCATC
AATTTCTGGAGGCAGTAATTC
WB235_1:10913365-10914502 Satlength=1138 Nr of Repeats=5 RepeatLength=94
seed=TGTAGACAAA Num.seqs=4 Similarity=0.971631
46
TCAAAATCTTCTGTTCTCTAAATATTGGGTGCTTTTCGATGTCCTTTGTAGACAAATCAATGGGAGAATCATC
AATTTCTGAAGGCAGTAATTC
Rev.of_WB235_4:1543828-1544203 Satlength=376 Nr of Repeats=4
RepeatLength=94 seed=TCCCATTGAT Num.seqs=3 Similarity=0.924350
65
TCAAAATCTTCTGTTCTCTAAATATTGGGTGCTTTTCGATGCCCTTTGTAGACAAATCAATGGGAAAATTGTC
CATTTCTGAAGGCAGTAATTC
WB235_6:311553-312211 Satlength=659 Nr of Repeats=7 RepeatLength=94
seed=GCAGTAATTC Num.seqs=7 Similarity=0.944613
84
TCAAAATCTTCTGTTCTCTAAATATTGGGTGCTTTTCGATGCCCTTTGTAGACAAATCAATGAGAAAATTGTC
AATTTCTGAAGGCAGTAATTC
Rev.of_WB235_6:6826365-6830125 Satlength=3761 Nr of Repeats=40
RepeatLength=94 seed=TGCCTTCAGA Num.seqs=40 Similarity=0.978523
87
TCAAAATCTTCTGTTCTCTAAATATTGAGTGCTTTTCGATGCCCTATGTAGACAAATCAATGGGAAAATTGTC
AATTTCTGAAGGCAGTAATTC

*************************** ************* *** **************** ** *** **
******* ************
Consensus:

TCAAAATCTTCTGTTCTCTAAATATTGGGTGCTTTTCGATGCCCTaTGTAGACAAATCAATGGGAAAATcaTC
AATTTCTGaAGGCAGTAATTC

>WB235_Fam_43_19_7 Nr. of seq. 7 Alignment length(with gaps) = 23
Alignment score = 0.690821
WB235_3:9863897-9863967 Satlength=71 Nr of Repeats=4 RepeatLength=19
seed=TGGTTGAGGC Num.seqs=3 Similarity=0.906433
0   -TGGTTGAGG--CTCTAG-ATTT
Rev.of_WB235_4:180606-180739 Satlength=134 Nr of Repeats=7
RepeatLength=19 seed=CCTCAACCAA Num.seqs=7 Similarity=0.826232
9   -TGGTTGAGG--CTCTNG-ATTT
WB235_4:119859-120107 Satlength=249 Nr of Repeats=13 RepeatLength=19
seed=TGGTTGAGGC Num.seqs=12 Similarity=0.868155
0   -TGGTTGAGG--CTCTCT-ATTT
WB235_3:9869882-9869977 Satlength=96 Nr of Repeats=5 RepeatLength=19
seed=ATTTTGGTTG Num.seqs=5 Similarity=0.818333
15   -TGGTTGAGG--CTCAGG-ATTT
Rev.of_WB235_4:195511-196615 Satlength=1105 Nr of Repeats=53
RepeatLength=20 seed=CTCAACCAAA Num.seqs=32 Similarity=0.882930
27   -TGGTTGAGG--CTCAGGATTTT
WB235_4:124390-125403 Satlength=1014 Nr of Repeats=21 RepeatLength=22
seed=TTGGTTGAGG Num.seqs=16 Similarity=0.915152
0   TTGGTTGAGGCTCTCTGG-ATTT
WB235_4:236164-236448 Satlength=285 Nr of Repeats=12 RepeatLength=22
seed=TGAGGCTCTC Num.seqs=11 Similarity=0.886501
5   TTGGTTGAGGCTCTCTGG-ATTT

********* ***   ***
Consensus:

TGGTTGAGGCTCtgGATTT

>WB235_Fam_44_226_6 Nr. of seq. 6 Alignment length(with gaps) = 227
Alignment score = 0.831816
WB235_5:9428459-9435308 Satlength=6850 Nr of Repeats=32 RepeatLength=214
seed=GTAACTGCTC Num.seqs=31 Similarity=0.958289
0
GTAACTGCTCAAAGCTTCTGGCACCTATTGGGATGTTGTTGGTAAATTCTGAAAAAGCTTGGAATTTGCAACA
AAGTTTCTGAAAGAAATTTC-
AAAAAAAGTTGATTTGAAGTGATTTGGGAATTTTCAAAGTTTCATAAATTTACCTA-
TTTTGCCTATAAATGGTCGTTTTCATTTAATAATAAAATACGCAC--AAAGGTA---CTG--TAGGT----
TTTCT
Rev.of_WB235_1:4280022-4281181 Satlength=1160 Nr of Repeats=6
RepeatLength=226 seed=ACAAGAAGCT Num.seqs=5 Similarity=0.915789
22
GTAACTACGTGAAGCTTCTTGTACCTACTGGAATATTGTTAATATATTCTAAAAGAGCTTAAAATTTACTACA
AAGTTTCTGAAAGACATTTCGCAAAAAAATTACTTTGAAGAGATTTATGAATTTTCAAAATTACGAAAATCCC
TCTA-
TTTTGCCTATAAGTGGTAGTTTTCATTAAATAATAAAATACGCACTAAAAGGTAGGGCTGAAAATGTACTATT
TCT
WB235_5:6072983-6091328 Satlength=18346 Nr of Repeats=79 RepeatLength=226
seed=CTTTGAAGAG Num.seqs=52 Similarity=0.955813
104

GTAACTACGTGAAGCTTCTTGTACCTACTGAAATATTGTTAATATATTCTGAAAGAGCTTAAAATTTACTACA
AAGTTTCTGAAAGACATTTC-
AAAAAAAATTACTTTGAAGAGATTTATGAATTTTCAAAGTTTGAAAAATCCACCTATTTTTGCCTATTAATGG
TAGTTTTCATTAACTAATAAAATACGCACTAAAAGGTAGGGCTGAAAATGTACTATTTCT
WB235_6:11888285-11889641 Satlength=1357 Nr of Repeats=6 RepeatLength=226
seed=TGTACTATTT Num.seqs=6 Similarity=0.964602
214
GTAACTACGTGAAGCTTCTTGTACCTACTGAAATATTGTTGGTATATTCTAAAAGAGCTTAAAATTTACTACA
AAGTTTCTAAAAGACATTTC-
AAAAAAAGTTACTTTGAAGAGATTTATGAATTTTCAAAGTTACGAAAATTCGTCTATTTTTGCCTATAAATGG
TAGTTTTCATTAAATAATAAAATACGCATTATAAGGTAGGGCTGAAAATGTACTATTTCT
WB235_6:5045129-5051107 Satlength=5979 Nr of Repeats=23 RepeatLength=226
seed=GAAAATTCAT Num.seqs=17 Similarity=0.593389
136
GTAACTACGTGAAGCTTCTTGTACCTACTGAAATATTGTTAATATATTCAGAAAGANCTTAAAANTTACTACG
AAGTTTCTGAAAGACATTTT-
TAAAAAAGTTACTTTGAAGAGATTTATGAATTTTCAAANTTTNGAAAATTCATCTATTTTTGTCTATAAATGG
TAGTTTTCATTTAATAATAAAATACGCACTAAAAGGTAGGGCTGAAAATGAACTATTTCT
Rev.of_WB235_6:5045783-5053744 Satlength=7962 Nr of Repeats=32
RepeatLength=226 seed=ATGAATTTTC Num.seqs=26 Similarity=0.592515
146
GTAACTACGTGAAGCTTCTTGTACCTACTGAAATATTGTTAATATATTCAGAAAGANCTTAAAATTTACTACG
AAGTTTCTGAAAGACATTTT-
TAAAAAAGTTACTTTGAAGAGATTTATGAATTTTCAAANTTTNGAAAATTCATCTATTTTTGTTTATAAATGG
TAGTTTTCATTTAATAATAAAATACNCACTAAAAGGTAGGGCTGAAAATGTACTATTTCT

****** *   ******** * ***** ** ** ***** ** **** *** * *** ** ** * **
******** ***** ****   ****** ** ******* ***** *********** **   ****
*** ***** *** * **** ********* * *********** **   ******   ***   * *
*****
Consensus:

GTAACTACGTGAAGCTTCTTGTACCTACTGaAATATTGTTaaTATATTCtgAAAGAgCTTAAAATTTACTACa
AAGTTTCTGAAAGACATTTcaAAAAAAgTTACTTTGAAGAGATTTATGAATTTTCAAAgTTtcgAAAATtCat
CTAtTTTTGcCTATAAATGGTAGTTTTCATTaAATAATAAAATACGCACTAAAAGGTAGGGCTGAAAATGTAC
TATTTCT

>WB235_Fam_45_33_6 Nr. of seq. 6 Alignment length(with gaps) = 33
Alignment score = 0.649832
WB235_1:12612870-12613263 Satlength=394 Nr of Repeats=5 RepeatLength=32
seed=GCTGATTGGT Num.seqs=3 Similarity=0.861111
0   GCTGATTGGTTCA-AAAGTGGGCGTGGCTAATC
Rev.of_WB235_3:12740278-12740831 Satlength=554 Nr of Repeats=5
RepeatLength=32 seed=ACCAATCAGC Num.seqs=3 Similarity=0.916667
10   GCTGATTGGTTCA-AAAGTGGGCGGAGCAAATC
Rev.of_WB235_3:12696947-12698148 Satlength=1202 Nr of Repeats=5
RepeatLength=32 seed=GACCAATCAG Num.seqs=3 Similarity=0.861111
11   GCTGATTGG-TCAGAGAGTGGGCGGGGCGAATC
Rev.of_WB235_2:14274983-14275913 Satlength=931 Nr of Repeats=26
RepeatLength=32 seed=AACCAATCAG Num.seqs=18 Similarity=0.796296
11   GCTGATTGGTTCC-AAAATGGGTGGAGCTGAGC
WB235_5:16583907-16584035 Satlength=129 Nr of Repeats=4 RepeatLength=32
seed=GCTGATTGGT Num.seqs=4 Similarity=0.508488
0   GCTGATTGG-TAAGAAACTGGGCGTGGCTGAAA

Rev.of_WB235_3:2218366-2219198 Satlength=833 Nr of Repeats=23 RepeatLength=32 seed=CTCAGCCAAT Num.seqs=17 Similarity=0.871936
14   GTTGATTGGCTGAGAAA-TAGGCGGAGCTAAAC

* ******* *   * * * ** * ** *
Consensus:

GCTGATTGGtTcAgAAAgTGGGCGgaGCtaAtC

>WB235_Fam_46_32_6 Nr. of seq. 6 Alignment length(with gaps) = 32
Alignment score = 0.838889
WB235_2:851256-851616 Satlength=361 Nr of Repeats=5 RepeatLength=31 seed=TGTCTAGCGA Num.seqs=3 Similarity=0.799283
0   TGTCGAGCGAGACCTCAGAAG-AGAGCTGAGT
WB235_2:1970474-1970784 Satlength=311 Nr of Repeats=10 RepeatLength=31 seed=AGAGCTGAGT Num.seqs=10 Similarity=0.801505
22   TGTCGAGCGAGACAGCAGAAGAAGAGCTGAGT
WB235_2:2593754-2594002 Satlength=249 Nr of Repeats=8 RepeatLength=31 seed=AGAGCTGAGT Num.seqs=8 Similarity=0.806548
22   TGTCGAGCGAGACAGCAGAAGAAGAGCTGAGT
WB235_2:1962052-1963013 Satlength=962 Nr of Repeats=31 RepeatLength=31 seed=AGAGCTGAGT Num.seqs=31 Similarity=0.809341
21   TGTCTAGCGAGATAGCAGAN-AAGAGCTGAGT
WB235_2:1977769-1979195 Satlength=1427 Nr of Repeats=46 RepeatLength=31 seed=AGAGCTGAGT Num.seqs=46 Similarity=0.811262
21   TGTCTAGCGAGATAGCAGAN-AAGAGCTGAGT
Rev.of_WB235_2:2008283-2009461 Satlength=1179 Nr of Repeats=38 RepeatLength=31 seed=GACAACTCAG Num.seqs=38 Similarity=0.826733
35   TGTCTAGCGAGATAGCAGAA-AAGAGCTGAGT

**** *******   ****   **********
Consensus:

TGTCgAGCGAGAcAGCAGAagAAGAGCTGAGT

>WB235_Fam_47_21_6 Nr. of seq. 6 Alignment length(with gaps) = 25
Alignment score = 0.624889
WB235_3:2235593-2235697 Satlength=105 Nr of Repeats=5 RepeatLength=21 seed=CTGACCAAAT Num.seqs=4 Similarity=0.841270
0   CTGACC---AAATTTGT-TTCAGCA
Rev.of_WB235_3:2245221-2245452 Satlength=232 Nr of Repeats=9 RepeatLength=21 seed=GTCAGTGCTG Num.seqs=6 Similarity=0.856085
5   CTGACA---AAATTTGT-TCCAGCA
WB235_3:3272813-3272897 Satlength=85 Nr of Repeats=4 RepeatLength=21 seed=TCCAGCACTG Num.seqs=4 Similarity=0.936508
14   CTGACA---AAATTTGT-TCCAGCA
Rev.of_WB235_6:723810-724054 Satlength=245 Nr of Repeats=5 RepeatLength=22 seed=TGGTCAGTGC Num.seqs=4 Similarity=0.777778
7   CTGACC---AAATTGGTCGCCAGCA
Rev.of_WB235_6:988793-988877 Satlength=85 Nr of Repeats=4 RepeatLength=21 seed=TGGTCAGTGC Num.seqs=4 Similarity=0.968254
7   CTGACC---AAATTGTT-TCTTGCA

WB235_6:1524262-1524376 Satlength=115 Nr of Repeats=5 RepeatLength=23
seed=AAAAAATTTG Num.seqs=4 Similarity=0.971014
6   CTGACCAAAAAATTTGT-TTC-GCA

*****   ***** *   ***
Consensus:

CTGACcAAATTtGTTcCaGCA

>WB235_Fam_48_21_6 Nr. of seq. 6 Alignment length(with gaps) = 22
Alignment score = 0.621212
WB235_6:1008023-1008695 Satlength=673 Nr of Repeats=4 RepeatLength=21
seed=TGACAAAACA Num.seqs=3 Similarity=1.000000
0   TGACAAAA-CATAACTTTGCAC
Rev.of_WB235_6:11745151-11745371 Satlength=221 Nr of Repeats=10
RepeatLength=22 seed=TCAGTGCAAA Num.seqs=8 Similarity=0.818182
3   TGACAAAAGTGTCACTTTGCAC
Rev.of_WB235_6:1062534-1062618 Satlength=85 Nr of Repeats=4
RepeatLength=21 seed=GTCAGTGCAA Num.seqs=4 Similarity=0.841270
4   TGACAATA-TTTGAATTTGCAC
WB235_6:1320585-1320992 Satlength=408 Nr of Repeats=5 RepeatLength=21
seed=TTGCACTGAC Num.seqs=3 Similarity=0.747475
15   TGACAAAA-TTTCGTNTTGCAC
Rev.of_WB235_6:1214446-1215096 Satlength=651 Nr of Repeats=6
RepeatLength=21 seed=GTCAGTGCAA Num.seqs=4 Similarity=0.809524
4   TGACCAAA-AATGCATTTGCAC
WB235_6:1325710-1325901 Satlength=192 Nr of Repeats=7 RepeatLength=21
seed=TTGCACTGAC Num.seqs=6 Similarity=0.630303
15   TGACCAAC-TCTTCATTTGCAC

**** *   *   ******
Consensus:

TGACaAAAtnTnaaTTTGCAC

>WB235_Fam_49_19_6 Nr. of seq. 6 Alignment length(with gaps) = 21
Alignment score = 0.706878
WB235_1:11486089-11486222 Satlength=134 Nr of Repeats=7 RepeatLength=19
seed=TTATCGATTT Num.seqs=5 Similarity=0.873684
46   TCNA--GAAATTATCGATTTT
Rev.of_WB235_3:2836362-2837893 Satlength=1532 Nr of Repeats=45
RepeatLength=19 seed=AAAATCGATA Num.seqs=33 Similarity=0.907895
57   TCCG--GAAATTATCGATTTT
Rev.of_WB235_3:1805381-1807736 Satlength=2356 Nr of Repeats=56
RepeatLength=19 seed=AAAATCGATA Num.seqs=36 Similarity=0.647665
57   CCGA--TAAATTATCGATTTT
Rev.of_WB235_3:1892229-1893089 Satlength=861 Nr of Repeats=44
RepeatLength=19 seed=AAAATCGATA Num.seqs=39 Similarity=0.805668
57   CCGA--GNAATTATCGATTTT
WB235_3:2012861-2014349 Satlength=1489 Nr of Repeats=36 RepeatLength=19
seed=TATCGATTTT Num.seqs=22 Similarity=0.729931
47   TCGT--AAAATTATCGATTTT

WB235_5:18940941-18941109 Satlength=169 Nr of Repeats=8 RepeatLength=21
seed=ATTATTGATT Num.seqs=8 Similarity=0.778409
23   GAAAAATTATTGATTTTTCAA

* * **   *
Consensus:

gaAATTATCGATTTTtCga

>WB235_Fam_50_18_6 Nr. of seq. 6 Alignment length(with gaps) = 19
Alignment score = 0.644444
WB235_1:6089071-6089158 Satlength=88 Nr of Repeats=5 RepeatLength=17
seed=AGGTAGTCAA Num.seqs=3 Similarity=1.000000
0   AGGTAGTCAATGAGCA-A-
Rev.of_WB235_4:7683397-7683518 Satlength=122 Nr of Repeats=6
RepeatLength=17 seed=CTCATTGCCT Num.seqs=4 Similarity=0.764706
14   GGGTAGTCAATGAGCA-C-
WB235_5:8314150-8315036 Satlength=887 Nr of Repeats=51 RepeatLength=17
seed=TAGTCAATGA Num.seqs=33 Similarity=0.909685
3   AGGTAGTCAATGATCT-A-
WB235_4:8999493-8999648 Satlength=156 Nr of Repeats=8 RepeatLength=17
seed=AGGCAATGAG Num.seqs=5 Similarity=0.781482
3   -AGTAGGCAATGAGAA-AG
Rev.of_WB235_5:6910515-6910653 Satlength=139 Nr of Repeats=7
RepeatLength=17 seed=CTCATTGCCT Num.seqs=5 Similarity=0.703704
13   -NGTAGGCAATGAGCA-AG
Rev.of_WB235_5:7703008-7703129 Satlength=122 Nr of Repeats=6
RepeatLength=17 seed=CTCATTGCCT Num.seqs=4 Similarity=0.697531
13   -NGTAGGCAATGAGCACAG

**** ******
Consensus:

ngGTAGgCAATGAGCAAg

>WB235_Fam_51_16_6 Nr. of seq. 6 Alignment length(with gaps) = 16
Alignment score = 0.697917
WB235_2:875486-875668 Satlength=183 Nr of Repeats=12 RepeatLength=14
seed=TAGGATCTAG Num.seqs=10 Similarity=0.807407
2   TC-TAGGATCT-AGGA
Rev.of_WB235_2:1605126-1605357 Satlength=232 Nr of Repeats=13
RepeatLength=14 seed=GATCCTAGAT Num.seqs=10 Similarity=0.832804
2   TC-TAGGATCT-AGGA
WB235_1:14002210-14002386 Satlength=177 Nr of Repeats=11 RepeatLength=16
seed=ATCTAAGGAT Num.seqs=11 Similarity=0.872727
6   TCTAAGGATCTAAGGA
Rev.of_WB235_1:14005076-14005524 Satlength=449 Nr of Repeats=13
RepeatLength=16 seed=AGATCCTTAG Num.seqs=10 Similarity=0.811111
10   TCTAAGGATCTAAGGA
WB235_1:14006294-14006390 Satlength=97 Nr of Repeats=6 RepeatLength=16
seed=AGGATCTAAG Num.seqs=6 Similarity=0.972222
11   TCTAAGGATCTAAGGA

WB235_4:15636148-15636960 Satlength=813 Nr of Repeats=51 RepeatLength=14 seed=GGACTAGGGA Num.seqs=48 Similarity=0.885234
3   TAGGGACTAGGGA-C-

*   *
Consensus:

TCtaAGGATCTaAGGA

>WB235_Fam_52_15_6 Nr. of seq. 6 Alignment length(with gaps) = 15
Alignment score = 0.952593
WB235_1:14290714-14290861 Satlength=148 Nr of Repeats=8 RepeatLength=15 seed=GGGGGCAACA Num.seqs=5 Similarity=0.822222
0   GGGGGCAACAAAATA
Rev.of_WB235_1:14292866-14293013 Satlength=148 Nr of Repeats=8 RepeatLength=15 seed=TGTTGCCCCC Num.seqs=5 Similarity=0.822222
10   GGGGGCAACAAAATA
Rev.of_WB235_3:705083-705230 Satlength=148 Nr of Repeats=8 RepeatLength=15 seed=TGTTGCCCCC Num.seqs=5 Similarity=0.795556
10   GGGGGCAACAAAATA
WB235_6:17344107-17344254 Satlength=148 Nr of Repeats=8 RepeatLength=15 seed=GGGGGCAACA Num.seqs=5 Similarity=0.822222
0   GGGGGCAACAAAATA
WB235_2:1487465-1487813 Satlength=349 Nr of Repeats=19 RepeatLength=15 seed=GGGGGCAACA Num.seqs=12 Similarity=0.807407
0   GGGGGCAACAAAAAA
WB235_3:700450-700543 Satlength=94 Nr of Repeats=5 RepeatLength=15 seed=GGGGGCAACA Num.seqs=3 Similarity=0.822222
0   GGGGGCAACAAAAAA

************* *
Consensus:

GGGGGCAACAAAAtA

>WB235_Fam_53_12_6 Nr. of seq. 6 Alignment length(with gaps) = 14
Alignment score = 0.649206
WB235_1:4548525-4548705 Satlength=181 Nr of Repeats=14 RepeatLength=12 seed=TTTTTATTTT Num.seqs=12 Similarity=0.947811
0   TTTTTATTT-TTA-
Rev.of_WB235_2:11122559-11122763 Satlength=205 Nr of Repeats=16 RepeatLength=12 seed=AATAAAAATA Num.seqs=14 Similarity=1.000000
2   TTTTTATTT-TTA-
Rev.of_WB235_3:3334092-3334302 Satlength=211 Nr of Repeats=17 RepeatLength=12 seed=AAAATAAAAA Num.seqs=16 Similarity=0.986111
4   TTTTTATTT-TTA-
WB235_5:6944029-6944530 Satlength=502 Nr of Repeats=36 RepeatLength=12 seed=TTTAATTTTA Num.seqs=31 Similarity=1.000000
1   TTTTAATTT-TAA-
WB235_4:6291557-6291611 Satlength=55 Nr of Repeats=4 RepeatLength=13 seed=TTTCATTTGT Num.seqs=3 Similarity=0.931624
1   TTTTCATTT-GTAA

Rev.of_WB235_6:2010907-2011305 Satlength=399 Nr of Repeats=28 RepeatLength=12 seed=AATAAATAAC Num.seqs=17 Similarity=1.000000
0   TATTTATTTATT--

* ** ****
Consensus:

TTTTtATTTTTA

>WB235_Fam_54_11_6 Nr. of seq. 6 Alignment length(with gaps) = 11 Alignment score = 0.838384
WB235_2:4309186-4309439 Satlength=254 Nr of Repeats=23 RepeatLength=11 seed=CAGTTGTCAC Num.seqs=19 Similarity=0.897927
0   CAGTTGTCACT
WB235_6:3631392-3631893 Satlength=502 Nr of Repeats=43 RepeatLength=11 seed=CAGTTGTCAC Num.seqs=40 Similarity=0.871173
0   CAGTTGTCACC
Rev.of_WB235_4:4599072-4599116 Satlength=45 Nr of Repeats=4 RepeatLength=11 seed=TGACAACTTT Num.seqs=4 Similarity=1.000000
9   AAGTTGTCACA
Rev.of_WB235_4:7128107-7129879 Satlength=1773 Nr of Repeats=156 RepeatLength=11 seed=TGACAACTTC Num.seqs=113 Similarity=0.796811
9   AAGTTGTCACG
WB235_5:4773346-4773621 Satlength=276 Nr of Repeats=22 RepeatLength=11 seed=CACGAAGTTG Num.seqs=19 Similarity=0.914939
18   AAGTTGTCACG
WB235_6:11789572-11789748 Satlength=177 Nr of Repeats=16 RepeatLength=11 seed=CGAAGTTGTC Num.seqs=16 Similarity=0.870707
9   AAGTTGTCACG

****
Consensus:

aAGTTGTCACg

>WB235_Fam_55_48_5 Nr. of seq. 5 Alignment length(with gaps) = 48 Alignment score = 0.900000
WB235_4:5465022-5467133 Satlength=2112 Nr of Repeats=44 RepeatLength=48 seed=AATTCAATTT Num.seqs=43 Similarity=0.962532
0   AATTCAATTTTTGATTTGCCCGTAATTTATATTTTTCTGAAGATGAGC
Rev.of_WB235_4:12891406-12891917 Satlength=512 Nr of Repeats=9 RepeatLength=48 seed=TCATCTTCAG Num.seqs=8 Similarity=0.978175
46   AATTTAATTTTTGATTTGCCCGTAATTTATAATTTTCTGAAGATGAGC
WB235_6:2991583-2991919 Satlength=337 Nr of Repeats=7 RepeatLength=48 seed=AAGATGAGGA Num.seqs=7 Similarity=0.933862
39   AATTTATTTTTTGATTTGCCCGTAATTTATATTTTTCTGAAGATGAGG
Rev.of_WB235_6:14384735-14389919 Satlength=5185 Nr of Repeats=108 RepeatLength=48 seed=AAATTACGGG Num.seqs=108 Similarity=0.957391
76   AATTTAATTGTTGATTTGCCCGTAATTTATATTTTTCTGAAGATGAGG
WB235_5:1637931-1639035 Satlength=1105 Nr of Repeats=23 RepeatLength=48 seed=CCCGTAATTT Num.seqs=23 Similarity=0.933685
18   AATTTAAATTTTGAGTTGCCCGTAATTTATAAATTTCTGAAGATGAGC

**** * * **** **************** **************

Consensus:

AATTtAatTtTTGAtTTGCCCGTAATTTATAttTTTCTGAAGATGAGc

>WB235_Fam_56_21_5 Nr. of seq. 5 Alignment length(with gaps) = 25
Alignment score = 0.616000
WB235_2:9719802-9720144 Satlength=343 Nr of Repeats=16 RepeatLength=19
seed=ATAAATATTA Num.seqs=10 Similarity=0.961014
0   --ATAAATATTAATTAAATAA----
Rev.of_WB235_4:1639384-1639487 Satlength=104 Nr of Repeats=5
RepeatLength=23 seed=ATTTAATTAA Num.seqs=3 Similarity=0.729469
0   AAATAAATATTAATTAAATAAAT--
WB235_6:5229333-5229440 Satlength=108 Nr of Repeats=5 RepeatLength=23
seed=AATAAATATT Num.seqs=3 Similarity=0.922705
1   AAATAAATATTAATTAATTAAAT--
Rev.of_WB235_6:2004050-2004599 Satlength=550 Nr of Repeats=27
RepeatLength=19 seed=ATATTTATTT Num.seqs=18 Similarity=0.931201
4   --ATAT----TAATTAAATAAATAA
Rev.of_WB235_4:13463617-13463705 Satlength=89 Nr of Repeats=4
RepeatLength=23 seed=AATATTTATT Num.seqs=3 Similarity=0.961353
4   --ATATTTATTAAATAAATAAATAA

***   *** *** ***
Consensus:

ATAaatatTAAtTAAaTAAat

>WB235_Fam_57_25_5 Nr. of seq. 5 Alignment length(with gaps) = 25
Alignment score = 0.946667
WB235_1:10130602-10133102 Satlength=2501 Nr of Repeats=100
RepeatLength=25 seed=TATTTACTCT Num.seqs=100 Similarity=0.949274
0   TATTTACTCTAATGTTCTGCCAATT
WB235_6:4026010-4030548 Satlength=4539 Nr of Repeats=181 RepeatLength=25
seed=GTTCTGCCAA Num.seqs=178 Similarity=0.933006
13   TATTTACTCTAATGTTCTGCCAATT
Rev.of_WB235_4:6357804-6361081 Satlength=3278 Nr of Repeats=132
RepeatLength=25 seed=TAGAGTAAAT Num.seqs=128 Similarity=0.931929
11   TATTTACTCTAATGTTCTGCTAATT
Rev.of_WB235_6:4030571-4031446 Satlength=876 Nr of Repeats=34
RepeatLength=25 seed=ATTGGCAGAA Num.seqs=33 Similarity=0.891706
24   TATTTACTCTAATGTTCTGCCAATA
Rev.of_WB235_6:10360970-10361186 Satlength=217 Nr of Repeats=4
RepeatLength=25 seed=ATTGGCAGAA Num.seqs=3 Similarity=0.837607
24   TATTTACTCTAATGTTCTGCCAATA

******************** ***
Consensus:

TATTTACTCTAATGTTCTGCcAATt

>WB235_Fam_58_21_5 Nr. of seq. 5 Alignment length(with gaps) = 23
Alignment score = 0.667391

WB235_3:83578-83851 Satlength=274 Nr of Repeats=13 RepeatLength=21 seed=CCATTTTAAG Num.seqs=13 Similarity=0.947904
0 CCATTTTAAGCC-AATTTTGGA-
WB235_3:83578-84096 Satlength=519 Nr of Repeats=25 RepeatLength=21 seed=CCATTTTAAG Num.seqs=16 Similarity=0.877588
0 CCATTTTAAGCC-AATTTTGGA-
Rev.of_WB235_3:80880-81257 Satlength=378 Nr of Repeats=14 RepeatLength=21 seed=AAATTTGGCT Num.seqs=11 Similarity=0.774105
18 CCATTTTAAGCCAAATTTTCAA-
Rev.of_WB235_4:1415278-1415575 Satlength=298 Nr of Repeats=15 RepeatLength=21 seed=AAAATTGACT Num.seqs=10 Similarity=0.908289
18 CGATTTTTAGTC-AATTTTTGA-
Rev.of_WB235_4:2182563-2182888 Satlength=326 Nr of Repeats=13 RepeatLength=21 seed=AAAATAGCTT Num.seqs=9 Similarity=0.765993
29 CTATTTTTAGTC-AATTT-TAAG

```
* ***** ** * *****   *
```

Consensus:

CcATTTTaAGcCAATTTtngA

>WB235_Fam_59_22_5 Nr. of seq. 5 Alignment length(with gaps) = 22 Alignment score = 0.630303
WB235_5:18201732-18201816 Satlength=85 Nr of Repeats=4 RepeatLength=21 seed=GCACTGACCA Num.seqs=4 Similarity=1.000000
0 GCACTGACCAAATGCAATGTT-
Rev.of_WB235_6:1094209-1094314 Satlength=106 Nr of Repeats=5 RepeatLength=21 seed=TTGGTCAGTG Num.seqs=5 Similarity=0.746032
11 GCACTGACCAATTNNAAAATT-
WB235_6:1292997-1293291 Satlength=295 Nr of Repeats=14 RepeatLength=21 seed=TTTGCACTGA Num.seqs=14 Similarity=0.853480
18 GCACTGACCAACACGAAT-TTT
Rev.of_WB235_2:2110299-2110412 Satlength=114 Nr of Repeats=5 RepeatLength=22 seed=TGGTCAGTGC Num.seqs=3 Similarity=0.959596
31 GCACTGACCAATAAAAATGTTT
Rev.of_WB235_6:1278787-1279105 Satlength=319 Nr of Repeats=7 RepeatLength=22 seed=GTCAGTGCAA Num.seqs=5 Similarity=0.692754
8 GCACTGACCATGTGCACAATTT

```
**********     *   **
```

Consensus:

GCACTGACCAantnnAatnTTt

>WB235_Fam_60_19_5 Nr. of seq. 5 Alignment length(with gaps) = 21 Alignment score = 0.695238
WB235_2:13799836-13800686 Satlength=851 Nr of Repeats=47 RepeatLength=18 seed=AATTTACCAA Num.seqs=36 Similarity=0.737116
0 AATTTACC-AA-TTTACCAA-
WB235_3:12188657-12188828 Satlength=172 Nr of Repeats=7 RepeatLength=19 seed=CAATTTACCA Num.seqs=5 Similarity=0.791667
7 AATTTGNC-AA-TTTACCAAA

WB235_3:12839625-12840128 Satlength=504 Nr of Repeats=16 RepeatLength=19
seed=TTGGCAATTT Num.seqs=10 Similarity=0.809747
22   AATTTGGC-AA-TTTACCAAA
Rev.of_WB235_3:1312388-1312777 Satlength=390 Nr of Repeats=16
RepeatLength=21 seed=TTTTTGGTAA Num.seqs=13 Similarity=0.873016
2   AAGTTACCAAATTTTACCAAA
WB235_3:1314701-1315015 Satlength=315 Nr of Repeats=12 RepeatLength=21
seed=CAAAAAGTTA Num.seqs=10 Similarity=0.923810
17   AAGTTACCAAATTTTAACAAA

** ** * ** **** ***
Consensus:

AAtTTacCAATTTAcCAAa

>WB235_Fam_61_20_5 Nr. of seq. 5 Alignment length(with gaps) = 21
Alignment score = 0.652381
WB235_2:3246632-3247175 Satlength=544 Nr of Repeats=19 RepeatLength=20
seed=GTAAATCTAC Num.seqs=12 Similarity=0.565197
0   GTAAATCTACACT-AGCTTCC
WB235_2:11906637-11907671 Satlength=1035 Nr of Repeats=44 RepeatLength=20
seed=TAAATCTACA Num.seqs=34 Similarity=0.664092
1   GTAAATCTACACN-GGCTTGC
Rev.of_WB235_4:3652033-3652933 Satlength=901 Nr of Repeats=15
RepeatLength=21 seed=TGTAGATTTA Num.seqs=10 Similarity=0.670875
11   GTAAATCTACANTAGGCTCCC
Rev.of_WB235_2:15194481-15194561 Satlength=81 Nr of Repeats=4
RepeatLength=20 seed=TGTAGATTTA Num.seqs=4 Similarity=0.688889
11   GTAAATCTACATT-GTCCTTT
WB235_5:18584208-18585555 Satlength=1348 Nr of Repeats=50 RepeatLength=20
seed=GTAAATCTAC Num.seqs=35 Similarity=0.635294
20   GTAAATCTACATN-GACCTCC

***********   *
Consensus:

GTAAATCTACAntggCttcc

>WB235_Fam_62_17_5 Nr. of seq. 5 Alignment length(with gaps) = 18
Alignment score = 0.848148
WB235_2:1596937-1597005 Satlength=69 Nr of Repeats=4 RepeatLength=17
seed=TCATTCCGTG Num.seqs=4 Similarity=1.000000
0   TCATTCC-GTGATTATTT
WB235_3:4637893-4638151 Satlength=259 Nr of Repeats=5 RepeatLength=17
seed=ATTATTTTCA Num.seqs=4 Similarity=1.000000
10   TCATTCC-GTGATTATTT
Rev.of_WB235_3:12323417-12323485 Satlength=69 Nr of Repeats=4
RepeatLength=17 seed=TGAAAATAAT Num.seqs=4 Similarity=1.000000
3   TCATTCC-GTGATTATTT
WB235_4:8789534-8789636 Satlength=103 Nr of Repeats=6 RepeatLength=17
seed=TTCATTCCGT Num.seqs=6 Similarity=1.000000
16   TCATTCC-GTGATTATTT

Rev.of_WB235_6:6114472-6114558 Satlength=87 Nr of Repeats=5 RepeatLength=17 seed=TAATTACTGG Num.seqs=4 Similarity=0.921569
14   T-ATTCCAGTAATTATTT

* ***** ** *******
Consensus:

TcATTCCGTgATTATTT

>WB235_Fam_63_11_5 Nr. of seq. 5 Alignment length(with gaps) = 14
Alignment score = 0.697619
WB235_2:4138713-4139388 Satlength=676 Nr of Repeats=4 RepeatLength=11 seed=TGCCTGCCTA Num.seqs=3 Similarity=0.919192
0   TGCCT--GCCTAC-
Rev.of_WB235_2:4140823-4140867 Satlength=45 Nr of Repeats=4 RepeatLength=11 seed=GGCAGGCAGT Num.seqs=4 Similarity=0.898990
8   TGCCT--GCCTAC-
Rev.of_WB235_4:4532759-4533458 Satlength=700 Nr of Repeats=6 RepeatLength=11 seed=GCAGTAGGCA Num.seqs=5 Similarity=0.878788
3   TGCCT--GCCTAC-
Rev.of_WB235_1:733645-734074 Satlength=430 Nr of Repeats=11 RepeatLength=12 seed=GCAGGTAGGT Num.seqs=7 Similarity=0.841270
3   TGCCTA--CCTACC
WB235_2:2966318-2968108 Satlength=1791 Nr of Repeats=54 RepeatLength=14 seed=CTACCTGCCT Num.seqs=35 Similarity=0.800720
9   TGCCTATGCCTACC

*****   *****
Consensus:

TGCCTgCCTAC

>WB235_Fam_64_11_5 Nr. of seq. 5 Alignment length(with gaps) = 11
Alignment score = 0.951515
WB235_1:12684455-12684521 Satlength=67 Nr of Repeats=5 RepeatLength=11 seed=TTTGGAGCGG Num.seqs=4 Similarity=0.777778
0   TTTGGAGCGGA
WB235_3:7452275-7453348 Satlength=1074 Nr of Repeats=91 RepeatLength=11 seed=TGGAGCGGTT Num.seqs=84 Similarity=0.892314
2   TTTGGAGCGGT
WB235_6:1643531-1644396 Satlength=866 Nr of Repeats=34 RepeatLength=11 seed=GGAGCGGTTT Num.seqs=25 Similarity=0.945050
3   TTTGGAGCGGT
WB235_6:8181963-8182276 Satlength=314 Nr of Repeats=28 RepeatLength=11 seed=GAGCGGTTTT Num.seqs=21 Similarity=0.864358
4   TTTGGAGCGGT
WB235_6:10369202-10370313 Satlength=1112 Nr of Repeats=99 RepeatLength=11 seed=TGGAGCGGTT Num.seqs=97 Similarity=0.865250
2   TTTGGAGCGGT

****
Consensus:

TTTGGAGCGGt

>WB235_Fam_65_184_4 Nr. of seq. 4 Alignment length(with gaps) = 189
Alignment score = 0.862140
WB235_1:338561-341137 Satlength=2577 Nr of Repeats=14 RepeatLength=184
seed=GAATACAGAA Num.seqs=14 Similarity=0.974916
0 GAATACAGAACACCAATTATGCCCGAG-AAAAAG-
ATCCTACTCAGAATATAAACATAGTCGAATTTATCGGATGTATAAAGATTCCCGAAGACACTTTCCAATTACC
CAAATTGTTCATATTCTA--AATGAAAT-
TCTCTTACTAGAACACTCTTGGCCAATGTACGCAGCCGAACGTATCATAAGT
Rev.of_WB235_2:14323963-14331913 Satlength=7951 Nr of Repeats=43
RepeatLength=185 seed=CTCGGACATA Num.seqs=38 Similarity=0.981228
27 GAATACAGAACACCAATTATGTCCGAG-
AAAAAGAATCCAATTCAGAATATTAACATACTCGAATTTGTCGGATGTATAAAGATTCCCGAAGACACTTTCC
AATTACCCAAATTGTTCATATTCTA--AATGAAAT-
TCTCTTACTAGAACACTCTTGGCCAATGTACGCAGCCGAACGTATCATAAGT
WB235_5:1201995-1204402 Satlength=2408 Nr of Repeats=13 RepeatLength=185
seed=AATATAAACA Num.seqs=11 Similarity=0.972875
45 GAATACAGAACACCAGTTATGTCCGAGCAAAAAG-
ATCCGATTCAGAATATAAACATATTTGAATTTGACGGATGTATAAAGATTCCCGAAGACACTTTCCAATTACC
CAAATTGTTCATATTCTA--AATGAGAT-
TCTCTTACTAGAACACTCTTGGCCAATGTACGCAGCCGAACGTATCATAAGT
WB235_3:11593439-11602256 Satlength=8818 Nr of Repeats=48
RepeatLength=184 seed=TACGCAGCCG Num.seqs=45 Similarity=0.968570
160 GAATACAGAACACCAATTATGTCCGAG-AAAAAG-
ATCCTACTCAGAATATGAACATATTCGAATACATCGGAGGTATATAGATTCCCGAAGAGCCTTTCCAATTAAC
CAAACGGTTCAAATTCTAGCAA--ACATCTC-
CTTACTTGAACACTCTTGGCCAATGTACGCAGCCGAACGTATCATAAGT

*************** ***** ***** ****** **** * ********* ****** * **** ****
***** ************* *********** ***** ***** ****** ** * ** ** ******
****
Consensus:

GAATACAGAACACCAaTTATGtCCGAGAAAAAGATCCtAcTCAGAATATaAACATAtTcGAATttatCGGAtG
TATAaAGATTCCCGAAGAcaCTTTCCAATTAcCCAAAttGTTCAtATTCTAAAtgAaATTCtCTTACTaGAAC
ACTCTTGGCCAATGTACGCAGCCGAACGTATCATAAGT

>WB235_Fam_66_64_4 Nr. of seq. 4 Alignment length(with gaps) = 67
Alignment score = 0.721393
WB235_2:1308143-1308573 Satlength=431 Nr of Repeats=7 RepeatLength=63
seed=GGTCTGCTAA Num.seqs=6 Similarity=0.855035
0 GGTCTGCTAAATCGAAACTTGTAGTTTGTAGCCTAGCAGACCAAATTTCTTCAAAACT-AC---TTG
WB235_2:1404044-1405522 Satlength=1479 Nr of Repeats=12 RepeatLength=63
seed=TAGTTTGTAG Num.seqs=8 Similarity=0.873016
20 -GTCTGCTAAATCGAAACTTGTAGTTTGTAGTCTAGCAGACCAAATTTCTTCAAAACC-CC--ATTG
Rev.of_WB235_2:1414614-1415742 Satlength=1129 Nr of Repeats=9
RepeatLength=63 seed=TTGGTCTGCT Num.seqs=6 Similarity=0.976014
43 -GTCTGCTAAATCAAAAGTTGTAGTTTGTAGTCTAGCAGACCAATTTTCTTCAAAACC-CC--ATGG
WB235_2:586310-587078 Satlength=769 Nr of Repeats=12 RepeatLength=64
seed=AAATTTTGTA Num.seqs=12 Similarity=0.921402
13 GGTCTGCCAGATCAAATTTTGTAGTTTGTAGTCTAGCAGACCCA--TT-TAGAATACCAACAAATTG

****** * *** ** ************* ********* * ** * ** ** * * *
Consensus:

gGTCTGCtAaATCaAAacTTGTAGTTTGTAGtCTAGCAGACCaAatTTcTtcAAaACcaCaTtG

>WB235_Fam_67_39_4 Nr. of seq. 4 Alignment length(with gaps) = 40
Alignment score = 0.856944
WB235_1:4167811-4168323 Satlength=513 Nr of Repeats=13 RepeatLength=39
seed=TATTTTTAAT Num.seqs=8 Similarity=0.954823
0   TA-TTTTTAATTTGGCCTACTTAAAAAGTAGGTCATGACC
WB235_1:13154184-13155089 Satlength=906 Nr of Repeats=19 RepeatLength=39
seed=TTAAAAAGTA Num.seqs=13 Similarity=0.817735
19   TA-ATTTTAATTTGGCCTACTTAAAAAGTAGGTCATGACC
WB235_1:12892561-12892756 Satlength=196 Nr of Repeats=5 RepeatLength=39
seed=TATTTTTAAT Num.seqs=5 Similarity=0.965812
0   TA-TTTTTAATTTAACCTACTTAAAAAGTAGGTCATGACC
WB235_3:10316800-10317870 Satlength=1071 Nr of Repeats=27 RepeatLength=40
seed=AAAAGTAGGT Num.seqs=25 Similarity=0.956889
23   TAGTTTTTAACGTGACCTCCTTAAAAAGTAGGTCATGACC

** ****** * *** *********************
Consensus:

TAtTTTTAAttTgaCCTaCTTAAAAAGTAGGTCATGACC

>WB235_Fam_68_36_4 Nr. of seq. 4 Alignment length(with gaps) = 36
Alignment score = 0.754630
WB235_1:3442604-3443969 Satlength=1366 Nr of Repeats=56 RepeatLength=35
seed=TTTCGGCAAT Num.seqs=35 Similarity=0.808371
0   TTTCGGCAATTTCGGCAATTGCCGGTTTCNTGAAT-
Rev.of_WB235_4:12678404-12678576 Satlength=173 Nr of Repeats=5
RepeatLength=35 seed=AAACCGGCAA Num.seqs=3 Similarity=0.759259
28   TTTCGGCAATTTCGGCAATTGCCGGTTTTGAGAAT-
WB235_1:14822628-14822877 Satlength=250 Nr of Repeats=7 RepeatLength=35
seed=CGGTTTTGGA Num.seqs=5 Similarity=0.885714
21   -TTCGGCAATTTCGATAATTGCCGGTTTTGGAAAAT
WB235_5:18110272-18110517 Satlength=246 Nr of Repeats=7 RepeatLength=35
seed=AAATTTTCGG Num.seqs=7 Similarity=0.945578
30   -TTCGGCAAATTTGATAATTGCCGGTTTTGGAAATT

******** ** * ************   **
Consensus:

tTTCGGCAAtTTcGacAATTGCCGGTTTtggaAAtt

>WB235_Fam_69_30_4 Nr. of seq. 4 Alignment length(with gaps) = 30
Alignment score = 0.777778
WB235_2:13990069-13990966 Satlength=898 Nr of Repeats=30 RepeatLength=30
seed=TTTCTAGAAA Num.seqs=24 Similarity=0.857649
0   TTTCTAGAAAAATCTGGAAACTTCTAGAAC
Rev.of_WB235_3:3229003-3229241 Satlength=239 Nr of Repeats=7
RepeatLength=30 seed=TAGAACTTTC Num.seqs=5 Similarity=0.786667
26   TTTCTAGAAAAATTTGGAAAGTTCTAGAAT
Rev.of_WB235_3:3230816-3231055 Satlength=240 Nr of Repeats=8
RepeatLength=30 seed=TAGAACTTTC Num.seqs=6 Similarity=0.925926
26   TTTCTAGAGAAATTTGGAAAGTTCTAGAAT

Rev.of_WB235_2:14836506-14836646 Satlength=141 Nr of Repeats=5 RepeatLength=30 seed=TTCTGGAAAA Num.seqs=3 Similarity=0.762963
9   TTTCCAGAAGGTTCTGGAACATTCTAGAAT

```
**** ***    * *****  ********
```
Consensus:

TTTCtAGAaaaaTcTGGAAagTTCTAGAAt

>WB235_Fam_70_30_4 Nr. of seq. 4 Alignment length(with gaps) = 30
Alignment score = 0.983333
WB235_2:14878787-14878927 Satlength=141 Nr of Repeats=5 RepeatLength=30 seed=AATTTTCCAG Num.seqs=3 Similarity=0.792593
0   AATTTTCCAGAAGGTTCTGGAACATTCCAG
WB235_4:9411472-9411612 Satlength=141 Nr of Repeats=5 RepeatLength=30 seed=AATTTTCCAG Num.seqs=3 Similarity=0.822222
0   AATTTTCCAGAAGGTTCTGGAACATTCCAG
WB235_6:1376606-1376746 Satlength=141 Nr of Repeats=5 RepeatLength=30 seed=AATTTTCCAG Num.seqs=3 Similarity=0.792593
0   AATTTTCCAGAAGGTTCTGGAACATTCCAG
WB235_6:516878-517017 Satlength=140 Nr of Repeats=5 RepeatLength=30 seed=AATTTTCCAG Num.seqs=3 Similarity=0.807407
0   AATTTTCCAGAAGGTTCTGGAACNTTCCAG

```
*********************** ******
```
Consensus:

AATTTTCCAGAAGGTTCTGGAACaTTCCAG

>WB235_Fam_71_21_4 Nr. of seq. 4 Alignment length(with gaps) = 21
Alignment score = 0.907407
WB235_1:12910137-12910261 Satlength=125 Nr of Repeats=6 RepeatLength=21 seed=AATCCAATAT Num.seqs=5 Similarity=0.790476
0   AATCCAATATGGANGAAATCT
Rev.of_WB235_2:14802929-14803074 Satlength=146 Nr of Repeats=7 RepeatLength=21 seed=ATTGGATTAG Num.seqs=6 Similarity=0.792593
8   AATCCAATATGGAAGAAATCT
WB235_3:6626010-6626226 Satlength=217 Nr of Repeats=6 RepeatLength=21 seed=AAATCTAATC Num.seqs=5 Similarity=0.822727
15   AATCCAATATGGNCGAAATCT
Rev.of_WB235_5:2636817-2636991 Satlength=175 Nr of Repeats=6 RepeatLength=21 seed=GATTAGATTT Num.seqs=5 Similarity=0.841270
25   AATCCAATATGGACNAAATCT

```
************   ******
```
Consensus:

AATCCAATATGGacgAAATCT

>WB235_Fam_72_19_4 Nr. of seq. 4 Alignment length(with gaps) = 20
Alignment score = 0.761111
WB235_4:229906-231132 Satlength=1227 Nr of Repeats=63 RepeatLength=18 seed=CTCAACCAAA Num.seqs=43 Similarity=0.898938
0   CTCAACCAA--ACTCTTAGC

WB235_4:238538-239383 Satlength=846 Nr of Repeats=8 RepeatLength=18
seed=AGCCTCAACC Num.seqs=5 Similarity=0.925926
15   CTCAACCAA--ACTCTTAGC
Rev.of_WB235_4:122695-123566 Satlength=872 Nr of Repeats=28
RepeatLength=19 seed=TTGGTTGAGG Num.seqs=19 Similarity=0.809582
9   CTCAACCAA-AATTGTTAGC
WB235_4:215697-216357 Satlength=661 Nr of Repeats=32 RepeatLength=20
seed=CTCAGCCTCA Num.seqs=30 Similarity=0.934713
14   CTCAACCAAAAATTCTCAGC

********* * * * ***
Consensus:

CTCAACCAAaAcTcTtAGC

>WB235_Fam_73_20_4 Nr. of seq. 4 Alignment length(with gaps) = 20
Alignment score = 0.744444
WB235_1:14477083-14477203 Satlength=121 Nr of Repeats=6 RepeatLength=20
seed=GTGAAATTTT Num.seqs=6 Similarity=0.705820
0   GTGAAATTTTAAATTTTTAA
WB235_2:14984666-14984990 Satlength=325 Nr of Repeats=14 RepeatLength=20
seed=TTCAGTCAAA Num.seqs=10 Similarity=0.802963
16   GTCAAAATTTAAATTTTTCA
WB235_5:19935725-19935960 Satlength=236 Nr of Repeats=11 RepeatLength=20
seed=TTTGAATTTT Num.seqs=7 Similarity=0.936508
7   GTGAAAATTTGAATTTTTGA
WB235_5:20406247-20406428 Satlength=182 Nr of Repeats=9 RepeatLength=20
seed=AAAATTCAAA Num.seqs=6 Similarity=0.778836
3   CTGAAAATTCAAATTTCTGA

* *** ** ***** * *
Consensus:

gTgAAAaTTtaAATTTtTgA

>WB235_Fam_74_17_4 Nr. of seq. 4 Alignment length(with gaps) = 18
Alignment score = 0.902778
WB235_2:1659657-1659775 Satlength=119 Nr of Repeats=7 RepeatLength=17
seed=GATGCCCCCT Num.seqs=5 Similarity=0.729630
0   GATGCCCCCTTTGAACG-
Rev.of_WB235_3:4254156-4254274 Satlength=119 Nr of Repeats=7
RepeatLength=17 seed=AGGGGGCATC Num.seqs=5 Similarity=0.749020
10   GATGCCCCCTCTGAACG-
WB235_3:4257044-4257162 Satlength=119 Nr of Repeats=7 RepeatLength=17
seed=GATGCCCCCT Num.seqs=5 Similarity=0.729630
17   GATGCCCCCTCTGAACG-
WB235_4:4198521-4198622 Satlength=102 Nr of Repeats=6 RepeatLength=17
seed=GATGCCCCCT Num.seqs=4 Similarity=0.722222
0   GATGCCCCCTCTGAACGN

********** ******
Consensus:

GATGCCCCCTcTGAACG

>WB235_Fam_75_16_4 Nr. of seq. 4 Alignment length(with gaps) = 16
Alignment score = 0.847222
WB235_1:7780058-7780728 Satlength=671 Nr of Repeats=39 RepeatLength=16
seed=AATTTTTCCG Num.seqs=31 Similarity=0.814158
0   AATTTTTCCGCNGCGA
Rev.of_WB235_2:7449335-7450152 Satlength=818 Nr of Repeats=42
RepeatLength=16 seed=CGGAAAAATT Num.seqs=27 Similarity=0.719401
10   AATTTTTCCGCNGCNA
Rev.of_WB235_5:13640261-13645859 Satlength=5599 Nr of Repeats=335
RepeatLength=16 seed=GAAAAATTTT Num.seqs=281 Similarity=0.744871
8   AATTTTTCAGCAGCAA
Rev.of_WB235_5:13640261-13646660 Satlength=6400 Nr of Repeats=357
RepeatLength=16 seed=GAAAAATTTT Num.seqs=293 Similarity=0.745623
8   AATTTTTCCGCAGCAA

******** ** ** *
Consensus:

AATTTTTCcGCaGCaA

>WB235_Fam_76_14_4 Nr. of seq. 4 Alignment length(with gaps) = 15
Alignment score = 0.727778
WB235_2:3033725-3034005 Satlength=281 Nr of Repeats=20 RepeatLength=14
seed=TTAGGGCCTT Num.seqs=20 Similarity=0.947368
0   TTAGGGCCTTGGGC-
Rev.of_WB235_4:154583-154709 Satlength=127 Nr of Repeats=8
RepeatLength=14 seed=CCTAAGCCTA Num.seqs=7 Similarity=0.918367
5   TTAGGGCCTTAGGC-
WB235_6:16199460-16199628 Satlength=169 Nr of Repeats=12 RepeatLength=14
seed=CTTAGGGCTT Num.seqs=12 Similarity=0.894661
6   TTAGGGCTTAGGGC-
Rev.of_WB235_2:3153871-3153936 Satlength=66 Nr of Repeats=4
RepeatLength=14 seed=CCAAGGCCCA Num.seqs=3 Similarity=0.746032
4   TT-GGGCCTTGGGCC

** **** * ***
Consensus:

TTaGGGCcTtgGGC

>WB235_Fam_77_15_4 Nr. of seq. 4 Alignment length(with gaps) = 15
Alignment score = 0.955556
WB235_1:4185588-4185648 Satlength=61 Nr of Repeats=4 RepeatLength=15
seed=TTTCCGGATC Num.seqs=4 Similarity=0.911111
0   TTTCCGGATCTACGT
Rev.of_WB235_1:4185829-4185964 Satlength=136 Nr of Repeats=9
RepeatLength=15 seed=AGATCCGGAA Num.seqs=9 Similarity=1.000000
11   TTTCCGGATCTACGT
Rev.of_WB235_1:6854524-6854599 Satlength=76 Nr of Repeats=5
RepeatLength=15 seed=AGATCCGGAA Num.seqs=5 Similarity=0.928889
11   TTTCCGGATCTACGT

WB235_2:12763248-12763368 Satlength=121 Nr of Repeats=7 RepeatLength=15
seed=ATTTCCGGAT Num.seqs=6 Similarity=0.922963
14   TTTCCGGATCTACGA

****
Consensus:

TTTCCGGATCTACGt

>WB235_Fam_78_13_4 Nr. of seq. 4 Alignment length(with gaps) = 13
Alignment score = 0.897436
WB235_5:1027504-1027725 Satlength=222 Nr of Repeats=11 RepeatLength=13
seed=GCCTATGGCC Num.seqs=7 Similarity=0.912088
0   GCCTATGGCCTAC
Rev.of_WB235_5:1028226-1028460 Satlength=235 Nr of Repeats=18
RepeatLength=13 seed=CGTAGGCCAT Num.seqs=18 Similarity=0.894754
1   GCCTATGGCCTAC
WB235_5:2102635-2102940 Satlength=306 Nr of Repeats=21 RepeatLength=13
seed=GCCTAAGCCT Num.seqs=17 Similarity=0.930618
7   GCCTATGGCCTAA
Rev.of_WB235_6:5125291-5125369 Satlength=79 Nr of Repeats=6
RepeatLength=13 seed=GCCATAGGCG Num.seqs=6 Similarity=0.931624
9   GCCTATGGCTTAC

********* **
Consensus:

GCCTATGGCcTAc

>WB235_Fam_79_12_4 Nr. of seq. 4 Alignment length(with gaps) = 12
Alignment score = 0.840278
WB235_1:2014890-2015108 Satlength=219 Nr of Repeats=13 RepeatLength=11
seed=TTTTGCCGAA Num.seqs=10 Similarity=0.811448
0   TTTTGCCGAAC-
WB235_1:5563061-5563217 Satlength=157 Nr of Repeats=13 RepeatLength=12
seed=GAAATTTTTT Num.seqs=13 Similarity=0.948718
7   TTTTTCCGAAAT
WB235_1:12479088-12479300 Satlength=213 Nr of Repeats=16 RepeatLength=12
seed=GAAATTTTTT Num.seqs=12 Similarity=0.915825
7   TTTTTCCGAAAT
Rev.of_WB235_1:12479365-12479989 Satlength=625 Nr of Repeats=49
RepeatLength=12 seed=AAAAAATTTC Num.seqs=34 Similarity=0.902159
17   TTTTTCCGAAAT

**** *****
Consensus:

TTTTtCCGAAat

>WB235_Fam_80_12_4 Nr. of seq. 4 Alignment length(with gaps) = 12
Alignment score = 0.662037
WB235_4:5273292-5273664 Satlength=373 Nr of Repeats=18 RepeatLength=12
seed=AGAAGGAGGA Num.seqs=11 Similarity=0.864646
0   AGAAGGAGGAAA

Rev.of_WB235_4:5302167-5302359 Satlength=193 Nr of Repeats=10
RepeatLength=12 seed=TCCTCTTTCT Num.seqs=8 Similarity=0.896825
10   AGAAAGAGGAGA
Rev.of_WB235_6:7686957-7687172 Satlength=216 Nr of Repeats=6
RepeatLength=12 seed=TCTTCCTCCT Num.seqs=4 Similarity=0.870370
1   AGGAGGAGGAAG
Rev.of_WB235_5:13956630-13956708 Satlength=79 Nr of Repeats=6
RepeatLength=12 seed=TCTTTCTCCT Num.seqs=5 Similarity=0.866667
1   ANAAGGAGAAAG

* * *** *
Consensus:

AgaAgGAGgAaa

>WB235_Fam_81_200_3 Nr. of seq. 3 Alignment length(with gaps) = 223
Alignment score = 0.702043
WB235_4:14417231-14419021 Satlength=1791 Nr of Repeats=7 RepeatLength=200
seed=GAAAAAGTGA Num.seqs=6 Similarity=0.937778
0   GAAA---AAGTGA-A-TTTTCAG-TCATAC------CTTG--TAGACCA--
CAATTTATATGCAAAATTAATAGATTTTCCAAACTTTGAAAATTAATTTCTGGCTTCAAACTAACTTTTTTAA
AAATTCCCGTAGCCATTTTGTAGCAAATTTTATGTTCTATCAGAATTTAAAAACAGAATTTCAAAAAATTTTG
AAACCACCAAGTTCAAGCA-------
Rev.of_WB235_5:4244061-4247526 Satlength=3466 Nr of Repeats=18
RepeatLength=200 seed=TGTGGTCCAG Num.seqs=13 Similarity=0.916410
37   GAAA---AAGTGA-A-TTTTCAG-TCATAC------CTTC--TGGACCA--
CAATTTATATGCAAAATTAATAGATTTTCCAAACTTTGAAAATTAATTTCTGGCTTGAAACTAACTTTTTTAG
AAATTCCCGTAGCCATTTTGTAGCAAATTTTAAGTTCTTTCAGAATTTGAAAACGGCATTTCAAAAAATTTAA
AATCTGCCAAGTTCAAGCA-------
WB235_1:4118875-4120034 Satlength=1160 Nr of Repeats=5 RepeatLength=223
seed=TTTCAGAATT Num.seqs=4 Similarity=0.971101
161
TAAACTTCAGTCATACCTTTCAGAGCAAACTTTGAAATTGAATGAAATAGACCATTTATTTGCAAAATTAGTA
GATTTTCCAATTTTTAAAAATTAATATCTGGCTTCAAAATAACTTTTTTGAAAATCCCCGTAGCCATTTTGTT
GCAAATTTTGTGCTCTTTCAGAATTTAAAAACGGCATTCCAAAAACTTTCACAACCGCCAAGTTCAAGCAAAA
AAAG

***   *** * * ****** ** **   **   * * * * ****** **********
************ *** ********* ******** *** ********** ****
**************** ********* * *** ********* ***** * *** ****** ***   * *
****
Consensus:

gAAAaAGTgAAtTTTCAGtCAtACcTTgTggAccACaATTTATaTGCAAAATTAaTAGATTTTCCAAacTTTg
AAAATTAATtTCTGGCTTcAAAcTAACTTTTTTaaAAATtCCCGTAGCCATTTTGTaGCAAATTTTatGtTCT
tTCAGAATTTaAAAACgGcATTtCAAAAAaTTTnaaAaCcgCCAAGTTCAAGCA

>WB235_Fam_82_167_3 Nr. of seq. 3 Alignment length(with gaps) = 172
Alignment score = 0.656977
WB235_3:449837-453285 Satlength=3449 Nr of Repeats=21 RepeatLength=164
seed=ATATAATGCT Num.seqs=17 Similarity=0.956420
0
ATATAATGCTCGTAATTATTTATAAGTGTGTGTTGTCTCAGAGCCACGTCTGAAAATACAGATTTTTTCTCGA

AAATTT---CAAAAACTTTGTA-GAAATTCGATTTTTGAC--
ATAAATGATCTCTCTTTTTTTCTGACATGTATTTCAGTGACAAA-TGGANT-AGAAA
WB235_2:14333431-14337579 Satlength=4149 Nr of Repeats=25
RepeatLength=166 seed=TCGTAATTAT Num.seqs=23 Similarity=0.971618
8   -
TATAATGTTCGTAATTATTCATAAGTTTGTGTTGTCTCAGAGCCATGTCTGAAAAAACAGATTTTTTTTCGAA
AATTT---TAAAAAGTTCGTA-
AAAATTGGATTTTTGACAAATCTATGCTCTCTTTTTTTGTTTAAAATTTAGTTCAGTGACATA-
CAATTTCAAAAA
WB235_2:13243398-13244452 Satlength=1055 Nr of Repeats=6 RepeatLength=165
seed=AAAATTCAAT Num.seqs=5 Similarity=0.933737
94   TTATAATGCTCGTAATTATTTATAAGTGTGTGTTGTCTCAGAATCAATTCT-
AAAAAACAGATTTTTTTT-GAAAGTTTAAGTAATATCCGCGGAGAAAATTCAATTTTGGACATTTCTAT--
TATCT-TTTTTTTAAAACATGTATTTCAGTGA-ATAGAAGACTCAAAA-

******* *********** ****** ************** ** *** **** *********** *
**** ***   ** *   * * ***** ***** ***   * ** * *** ***** *   * **
** ******** * *   * * **
Consensus:

nTATAATGcTCGTAATTATTtATAAGTgTGTGTTGTCTCAGAgcCAngTCTgAAAAaACAGATTTTTTtTcGA
AAaTTTtAAaAacttcGtAaAAATTcgATTTTtGACanaTctATgnTcTCTnTTTTTtTntaAcATgTAtTTC
AGTGAcAtAnaganTcAaAAa

>WB235_Fam_83_90_3 Nr. of seq. 3 Alignment length(with gaps) = 95
Alignment score = 0.673684
WB235_5:19466947-19467275 Satlength=329 Nr of Repeats=4 RepeatLength=82
seed=CCCCGAACGT Num.seqs=4 Similarity=0.948510
0   CCCCGAACGTGTCGGCCGCTTCC--AAATAACCACCTCCTTGCACTACGTTGCGCACACA--------
CTCATTTCACGCCAAGCTGCGGAA---
WB235_5:1539714-1540122 Satlength=409 Nr of Repeats=5 RepeatLength=89
seed=GCACTACGTT Num.seqs=3 Similarity=0.740741
38   CCCTGAACGTGTCGGCCGC-TCC--
AAATAACTACCTTCTNGCACTACGTTGCGCACACACTAAGCTACTCGTTTCACGCCAAGCTACGGAA---
WB235_3:1643024-1643390 Satlength=367 Nr of Repeats=4 RepeatLength=92
seed=CTGCGGAACC Num.seqs=3 Similarity=1.000000
81   ---
CGAACGTGTCGGCCGCTTCCAAAAACAACCACCTCTTCACACTTCATTGCGCACACACCAAACTACTAGTTTC
ACGTCAAACTGCGGAACCC

************** *** *** *** **** * **** * ***********   **
******* *** ** *****
Consensus:

ccccGAACGTGTCGGCCGCtTCCAAAtAACcACCTccTngCACTaCgTTGCGCACACAcnaanctaCTcgTTT
CACGcCAAgCTgCGGAA

>WB235_Fam_84_79_3 Nr. of seq. 3 Alignment length(with gaps) = 79
Alignment score = 0.912799
WB235_2:3941820-3946547 Satlength=4728 Nr of Repeats=58 RepeatLength=79
seed=GTGAATCTCC Num.seqs=53 Similarity=0.979228
0
GTGAATCTCCTCTCCACCTAATGATTCCTGACAGAATACCGGCAGTATTCTGGGGTCTCATTAGGTTTTAGTT
TCTTCT

Rev.of_WB235_4:11033628-11036077 Satlength=2450 Nr of Repeats=31
RepeatLength=79 seed=GAAACTAAAA Num.seqs=31 Similarity=0.920108
75
GTGAATCTCCTCTCTACCTAATGAGTCCTGACAGAATACCGGCAGTATTCTGGGGTCTCATTAGGTTTTAGTT
TCTTCT
WB235_2:8287652-8292933 Satlength=5282 Nr of Repeats=61 RepeatLength=79
seed=TGAGTCCTGA Num.seqs=58 Similarity=0.902451
21
CTGAATCTCCACTCTTCCTAATGAGTCCTGACAGAATACCGGCAGTATTTTGGGGTCTCATTAGGTTTTNGTT
TCTTTT

********* *** ******** *********************** ******************
******* *
Consensus:

gTGAATCTCCtCTCtaCCTAATGAgTCCTGACAGAATACCGGCAGTATTcTGGGGTCTCATTAGGTTTTaGTT
TCTTcT

>WB235_Fam_85_76_3 Nr. of seq. 3 Alignment length(with gaps) = 76
Alignment score = 0.906433
WB235_4:14205760-14206748 Satlength=989 Nr of Repeats=13 RepeatLength=76
seed=AATGCTAGAG Num.seqs=13 Similarity=0.967611
0
AATGCTAGAGGACACTCCTTTGCACGAAAGGAAGTGGGAGAGACTGTGGAGTTGTCGGTGTCTTTGTGCATGC
TCG
Rev.of_WB235_4:14210874-14215456 Satlength=4583 Nr of Repeats=61
RepeatLength=76 seed=TCGTGCAAAG Num.seqs=60 Similarity=0.948617
27
AATGCTAGAGGACATTCCTTTGCACGAAAGGAAGTGGGAGAGACTGTGGAGTTGTCGGTGTCTTTGTGCATGC
TCG
Rev.of_WB235_6:16758347-16761606 Satlength=3260 Nr of Repeats=43
RepeatLength=76 seed=AGCATTAGAG Num.seqs=34 Similarity=0.949620
6
AATGCTAGAAGACACTCCTTTGCACGAAGGAAAGTGGGAGAGACTGTGGTGTTGTCGGTGTCTGTGTACATGC
TCT

********* **** ************* * ***************** ************ ***
****
Consensus:

AATGCTAGAgGACAcTCCTTTGCACGAAaGgAAGTGGGAGAGACTGTGGaGTTGTCGGTGTCTtTGTgCATGC
TCg

>WB235_Fam_86_66_3 Nr. of seq. 3 Alignment length(with gaps) = 68
Alignment score = 0.892157
WB235_1:9821430-9822959 Satlength=1530 Nr of Repeats=23 RepeatLength=66
seed=AATCAACCTA Num.seqs=16 Similarity=0.986869
0   AATCAACCTATTCAGTTGACAATGGTTTTCTTTTATCAAATTTTTTAAAATTT--TAAAGAAAACACA
WB235_4:17059789-17062957 Satlength=3169 Nr of Repeats=48 RepeatLength=66
seed=CAAATCAACC Num.seqs=48 Similarity=0.956050
64   AATCAACCTATTCAGTTGACAATGGTTTTCTTTTATCAAATTTTTTAAAATTT--
TAAAGAAAACACA
Rev.of_WB235_1:10204129-10219199 Satlength=15071 Nr of Repeats=223
RepeatLength=68 seed=ACTGAATAGG Num.seqs=140 Similarity=0.978159

16
AATCAACCTATTCAGTTGACAATGGTTTTCTTTTACCAAAATTCTTAGAATTTCAGAAAGAAAACACA

*********************************** **** ** *** *****   ************
Consensus:

AATCAACCTATTCAGTTGACAATGGTTTTCTTTTAtCAAAtTTtTTAaAATTTtAAAGAAAACACA

>WB235_Fam_87_64_3 Nr. of seq. 3 Alignment length(with gaps) = 64
Alignment score = 0.954861
WB235_1:13929375-13929595 Satlength=221 Nr of Repeats=5 RepeatLength=63
seed=TTTTTGACTA Num.seqs=3 Similarity=0.876736
0   TTTTTGACTACGGAATGTCTTTTTCGCTACTGACTG-AAAAATAGCTACATTCGCACTTTTGNC
Rev.of_WB235_1:13935628-13936488 Satlength=861 Nr of Repeats=15
RepeatLength=64 seed=GTAGTCAAAA Num.seqs=10 Similarity=0.982870
11   TTTTTGACTACTGAATGTCTTTTTCGCTACTGACTGAAAAAATAGCTACATTCGCACTTTTGTC
Rev.of_WB235_5:14204941-14205737 Satlength=797 Nr of Repeats=14
RepeatLength=64 seed=GTAGTCAAAA Num.seqs=9 Similarity=0.983796
11   TTTTTGACTACTGAATGTCTTTTTCGCTACTGACTGAAAAAATAGCTACATTCGCACTTTTGTC

*********** ************************ ************************* *
Consensus:

TTTTTGACTACtGAATGTCTTTTTCGCTACTGACTGaAAAAATAGCTACATTCGCACTTTTGtC

>WB235_Fam_88_34_3 Nr. of seq. 3 Alignment length(with gaps) = 34
Alignment score = 0.980392
WB235_1:11432554-11432811 Satlength=258 Nr of Repeats=6 RepeatLength=34
seed=ATGCACCATG Num.seqs=4 Similarity=0.679739
0   ATGCACCATGTCAAAAAATATATCCAAAAGTACG
WB235_1:11455940-11456197 Satlength=258 Nr of Repeats=6 RepeatLength=34
seed=ATGCACCATG Num.seqs=4 Similarity=0.679739
0   ATGCACCATGTCAAAAAATATATCCAAAAGTACG
WB235_1:12258128-12258286 Satlength=159 Nr of Repeats=5 RepeatLength=34
seed=ATGCACCATG Num.seqs=4 Similarity=0.712418
0   ATGCACCATGTCAAAAAATATATCCAAAANTACG

***************************** ****
Consensus:

ATGCACCATGTCAAAAAATATATCCAAAAgTACG

>WB235_Fam_89_32_3 Nr. of seq. 3 Alignment length(with gaps) = 32
Alignment score = 0.972222
WB235_4:13909776-13910308 Satlength=533 Nr of Repeats=19 RepeatLength=32
seed=CGACCTGCGA Num.seqs=14 Similarity=0.994048
0   CGACCTGCGACCTAGCGACCTGGGTCGCTTTG
WB235_6:2307188-2307299 Satlength=112 Nr of Repeats=4 RepeatLength=32
seed=CGACCTGCGA Num.seqs=3 Similarity=0.805556
0   CGACCTGCGACCTAGCGACCTAGGTCGCTTTG
WB235_6:12578040-12578842 Satlength=803 Nr of Repeats=12 RepeatLength=32
seed=CGACCTAGGT Num.seqs=8 Similarity=0.956845
15   CGACCTGCGACCTAGCGACCTAGGTCGCTTTG

```
********************* **********
Consensus:

CGACCTGCGACCTAGCGACCTaGGTCGCTTTG

>WB235_Fam_90_27_3  Nr. of seq. 3 Alignment length(with gaps) = 27
Alignment score = 0.658436
WB235_4:2967003-2967147 Satlength=145 Nr of Repeats=6 RepeatLength=24
seed=GCGGCCGACA  Num.seqs=6 Similarity=0.855556
0    -GCGGCCGACACCTTACGGGTTG--TA
WB235_2:13502158-13503434 Satlength=1277 Nr of Repeats=26 RepeatLength=27
seed=AGCAGCCGAC  Num.seqs=20 Similarity=0.791813
0    AGCAGCCGACACCTTGCNGGTTGCTTA
WB235_5:19555359-19555663 Satlength=305 Nr of Repeats=8 RepeatLength=27
seed=GCAGCCGACA  Num.seqs=6 Similarity=0.584291
1    AGCAGCCGACACCTCACTGGTCCCTNA

** **********  * ***     *
Consensus:

aGCaGCCGACACCTtaCnGGTtgcttA

>WB235_Fam_91_26_3  Nr. of seq. 3 Alignment length(with gaps) = 26
Alignment score = 0.965812
WB235_2:2157814-2157944 Satlength=131 Nr of Repeats=5 RepeatLength=26
seed=TCAAGGCGAA  Num.seqs=5 Similarity=0.979487
0    TCAAGGCGAAACGGGAGGTGAATCGC
Rev.of_WB235_5:8836966-8837173 Satlength=208 Nr of Repeats=8
RepeatLength=26 seed=TTCGCCCTGA  Num.seqs=7 Similarity=0.907204
10    TCAGGGCGAAACGGGAGGTGAATCGC
Rev.of_WB235_5:8879060-8879267 Satlength=208 Nr of Repeats=8
RepeatLength=26 seed=TTCGCCCTGA  Num.seqs=7 Similarity=0.907204
10    TCAGGGCGAAACGGGAGGTGAATCGC

*** **********************
Consensus:

TCAgGGCGAAACGGGAGGTGAATCGC

>WB235_Fam_92_21_3  Nr. of seq. 3 Alignment length(with gaps) = 23
Alignment score = 0.801932
WB235_2:13342774-13342879 Satlength=106 Nr of Repeats=5 RepeatLength=21
seed=CTAGAAAAAC  Num.seqs=5 Similarity=0.898413
0    CTA-GAAAAACTCGGCCATCAA-
Rev.of_WB235_2:13343764-13344901 Satlength=1138 Nr of Repeats=46
RepeatLength=21 seed=TTTTCTAGTT  Num.seqs=34 Similarity=0.794245
8    CTA-GAAAAACTCGGCCATCAA-
WB235_2:642340-642984 Satlength=645 Nr of Repeats=25 RepeatLength=23
seed=GGCCACCAAT  Num.seqs=20 Similarity=0.753776
13    CTATAAAAAACTCGGCCACCAAT

***  ************* ***
Consensus:
```

CTAgAAAAACTCGGCCAtCAA

>WB235_Fam_93_21_3 Nr. of seq. 3 Alignment length(with gaps) = 23
Alignment score = 0.608696
WB235_3:2103059-2103647 Satlength=589 Nr of Repeats=25 RepeatLength=21
seed=GCCTAAAAAC Num.seqs=16 Similarity=0.805291
0   GCCTAAAAA--CGTAAATTTTAA
Rev.of_WB235_4:2702010-2702785 Satlength=776 Nr of Repeats=31
RepeatLength=21 seed=TTTGGATTTT Num.seqs=25 Similarity=0.861799
15   GCCTAAAAA--TCCAAATTTTAA
WB235_1:898453-898911 Satlength=459 Nr of Repeats=21 RepeatLength=22
seed=TTTTAGCCAA Num.seqs=17 Similarity=0.862299
16   -CCAAAAAATCCGTTAATTTTAG

** *****   *******
Consensus:

gCCtAAAAAcgtaAATTTTAa

>WB235_Fam_94_21_3 Nr. of seq. 3 Alignment length(with gaps) = 23
Alignment score = 0.748792
WB235_4:3754942-3755101 Satlength=160 Nr of Repeats=5 RepeatLength=21
seed=TAGTTTGTAG Num.seqs=3 Similarity=0.661376
0   TAG-TTT-ATAGTTTGCAGTTTG
WB235_5:3651539-3651616 Satlength=78 Nr of Repeats=4 RepeatLength=21
seed=AGTTTGTAGT Num.seqs=3 Similarity=0.640212
1   TAG-TTT-GTAGTTTGTNGTTTG
Rev.of_WB235_1:12893074-12893850 Satlength=777 Nr of Repeats=11
RepeatLength=23 seed=ACTACAAACT Num.seqs=10 Similarity=1.000000
4   TAGTTTTCATAGTTTGTAGTTTG

*** *** ******* *****
Consensus:

TAGTTTaTAGTTTGtaGTTTG

>WB235_Fam_95_20_3 Nr. of seq. 3 Alignment length(with gaps) = 22
Alignment score = 0.656566
WB235_3:3042951-3043689 Satlength=739 Nr of Repeats=38 RepeatLength=18
seed=GTCGATTTAC Num.seqs=33 Similarity=0.778900
0   GTCGATTTACG-AGA-TTN--T
Rev.of_WB235_2:13737396-13737775 Satlength=380 Nr of Repeats=15
RepeatLength=21 seed=CTCGTAAATC Num.seqs=9 Similarity=0.754630
13   GTCGATTTACGAGGAGTTC-TT
WB235_4:1848441-1848937 Satlength=497 Nr of Repeats=18 RepeatLength=21
seed=TGTCGATTTA Num.seqs=15 Similarity=0.738095
21   GTCGATTTACG-GGAGTTCAGT

*********** ** **   *
Consensus:

GTCGATTTACGgGAgTTcnT

>WB235_Fam_96_22_3 Nr. of seq. 3 Alignment length(with gaps) = 22
Alignment score = 0.777778
WB235_2:2733211-2733873 Satlength=663 Nr of Repeats=24 RepeatLength=22
seed=ACTACAGTAA Num.seqs=18 Similarity=0.729847
0   ACTACAGTAACCCTACANTACC
WB235_5:1025021-1025505 Satlength=485 Nr of Repeats=20 RepeatLength=22
seed=ACAGTACTCC Num.seqs=16 Similarity=0.776630
3   ANTACAGTACTCCTACAGTACC
Rev.of_WB235_5:16536032-16536834 Satlength=803 Nr of Repeats=14
RepeatLength=22 seed=GGGTACTGTA Num.seqs=9 Similarity=0.840067
12   ACTACAGTACCCATACAGTACT

* ******* * **** ***
Consensus:

AcTACAGTAccCcTACAgTACc

>WB235_Fam_97_19_3 Nr. of seq. 3 Alignment length(with gaps) = 21
Alignment score = 0.608466
WB235_1:2524693-2524982 Satlength=290 Nr of Repeats=10 RepeatLength=18
seed=GCCTAGGAAT Num.seqs=8 Similarity=0.772487
0   GCCTAGGAAT-CCAAT--ATG
Rev.of_WB235_1:1278237-1279259 Satlength=1023 Nr of Repeats=33
RepeatLength=19 seed=TTCTAGGCCA Num.seqs=21 Similarity=0.753383
8   GCCTAGAAATCCTCA--GATG
Rev.of_WB235_1:1108035-1108695 Satlength=661 Nr of Repeats=21
RepeatLength=20 seed=ATTTCTAGGC Num.seqs=13 Similarity=0.625153
10   GCCTAGAAAT-CTNATNGATG

****** *** * *   ***
Consensus:

GCCTAGaAATCtnAtgATG

>WB235_Fam_98_18_3 Nr. of seq. 3 Alignment length(with gaps) = 21
Alignment score = 0.761905
WB235_6:3338986-3339541 Satlength=556 Nr of Repeats=27 RepeatLength=18
seed=TTTTGTCAGT Num.seqs=20 Similarity=0.766579
0   TTTTGTCAGTGCT---ATAGT
WB235_6:3392053-3392273 Satlength=221 Nr of Repeats=10 RepeatLength=18
seed=TTGTCAGTGC Num.seqs=7 Similarity=0.848325
2   TTTTGTCAGTGCT---ATAGT
Rev.of_WB235_6:1544700-1544784 Satlength=85 Nr of Repeats=4
RepeatLength=21 seed=TCTGAGCACT Num.seqs=4 Similarity=0.925926
17   TTTTGTCAGTGCTCAGATAAT

*************   *** *
Consensus:

TTTTGTCAGTGCTATAgT

>WB235_Fam_99_21_3 Nr. of seq. 3 Alignment length(with gaps) = 21
Alignment score = 0.767196

WB235_3:1154872-1155214 Satlength=343 Nr of Repeats=4 RepeatLength=21
seed=ACTACAGTAC Num.seqs=3 Similarity=0.788360
0   ACTACAGTACCCCCNACNACT
WB235_4:14426797-14427553 Satlength=757 Nr of Repeats=36 RepeatLength=21
seed=CAACTACCAC Num.seqs=36 Similarity=0.943361
10   ACTACCGTACCAACTACCACA
WB235_4:14447004-14447298 Satlength=295 Nr of Repeats=13 RepeatLength=21
seed=ACTACCGTAC Num.seqs=12 Similarity=0.895142
21   ACTACCGTACCAACTACCACA

***** ***** * ** **
Consensus:

ACTACcGTACCaaCtACcACa

>WB235_Fam_100_21_3 Nr. of seq. 3 Alignment length(with gaps) = 21
Alignment score = 0.957672
WB235_5:3373171-3373906 Satlength=736 Nr of Repeats=20 RepeatLength=21
seed=CTAACATTTT Num.seqs=12 Similarity=0.927850
0   CTAACATTTTTCAATTTTCGT
WB235_5:16625438-16625585 Satlength=148 Nr of Repeats=7 RepeatLength=21
seed=ATTTTTCAAT Num.seqs=7 Similarity=0.848828
5   CTAACATTTTTCAATTTTCGT
WB235_5:3632093-3632198 Satlength=106 Nr of Repeats=5 RepeatLength=21
seed=TTTCAGTTTT Num.seqs=5 Similarity=0.911111
8   CTAACATTTTTCAGTTTTCGT

************* *******
Consensus:

CTAACATTTTTCAaTTTTCGT

>WB235_Fam_101_21_3 Nr. of seq. 3 Alignment length(with gaps) = 21
Alignment score = 0.682540
WB235_6:1214446-1215096 Satlength=651 Nr of Repeats=6 RepeatLength=21
seed=GTCAGTGCAA Num.seqs=4 Similarity=0.809524
34   AAATGCATTTTTGGTCAGTGC
Rev.of_WB235_6:1325710-1325901 Satlength=192 Nr of Repeats=7
RepeatLength=21 seed=TTGCACTGAC Num.seqs=6 Similarity=0.630303
44   AAATGAAGAGTTGGTCAGTGC
WB235_6:15295722-15295953 Satlength=232 Nr of Repeats=11 RepeatLength=21
seed=TGAAAGATTG Num.seqs=9 Similarity=0.957672
28   GTGCCTATGAAAGATTGGTCA

**   *   * *
Consensus:

GTgcaaatgnAtnnttGgTca

>WB235_Fam_102_20_3 Nr. of seq. 3 Alignment length(with gaps) = 20
Alignment score = 0.688889
WB235_2:13528439-13528985 Satlength=547 Nr of Repeats=24 RepeatLength=20
seed=GTAAATCGAC Num.seqs=16 Similarity=0.655886
10   ACACNACGTCGTAAATCGAC

Rev.of_WB235_3:2036337-2036525 Satlength=189 Nr of Repeats=9 RepeatLength=20 seed=TGTAGATTTA Num.seqs=6 Similarity=0.697778
21   ATAGAACCCCGTAAATCTAC
Rev.of_WB235_3:12835769-12836969 Satlength=1201 Nr of Repeats=30 RepeatLength=20 seed=TGTAGATTTA Num.seqs=22 Similarity=0.674981
21   ATAGANCTTCGTAAATCTAC

******* *** *   * *
Consensus:

GTAAATCtACAtAgaaCntC

>WB235_Fam_103_20_3 Nr. of seq. 3 Alignment length(with gaps) = 20 Alignment score = 0.777778
WB235_4:8572647-8581868 Satlength=9222 Nr of Repeats=443 RepeatLength=20 seed=CGATATAGCT Num.seqs=408 Similarity=0.824886
0   CGATATAGCTATTAACGATA
Rev.of_WB235_6:4031472-4056497 Satlength=25026 Nr of Repeats=1238 RepeatLength=20 seed=GCTATATCGT Num.seqs=1039 Similarity=0.874603
9   CGATATAGCTATTTACGATA
Rev.of_WB235_6:7077538-7079254 Satlength=1717 Nr of Repeats=55 RepeatLength=20 seed=TCGTTTATAG Num.seqs=33 Similarity=0.899747
18   CGACACAGCTATAAACGACA

*** * ****** **** *
Consensus:

CGAtAtAGCTATtaACGAtA

>WB235_Fam_104_16_3 Nr. of seq. 3 Alignment length(with gaps) = 16 Alignment score = 0.777778
WB235_1:749263-749839 Satlength=577 Nr of Repeats=36 RepeatLength=16 seed=TTTCAGGCTT Num.seqs=36 Similarity=0.942460
0   TTTCAGGCTTTCAGGC
WB235_5:17629258-17629682 Satlength=425 Nr of Repeats=26 RepeatLength=16 seed=CCAGGGTTCC Num.seqs=25 Similarity=1.000000
2   TTCCAGGGTTCCAGGG
WB235_5:17629258-17630037 Satlength=780 Nr of Repeats=29 RepeatLength=16 seed=CCAGGGTTCC Num.seqs=27 Similarity=0.987654
2   TTCCAGGGTTCCAGGG

** **** ** ****
Consensus:

TTcCAGGgTTcCAGGg

>WB235_Fam_105_11_3 Nr. of seq. 3 Alignment length(with gaps) = 12 Alignment score = 0.787037
WB235_3:8897677-8897798 Satlength=122 Nr of Repeats=11 RepeatLength=11 seed=ATTTGGAATA Num.seqs=11 Similarity=0.903030
0   ATT-TGGAATAT
WB235_4:10984356-10985109 Satlength=754 Nr of Repeats=4 RepeatLength=11 seed=TTTGAAATAT Num.seqs=3 Similarity=1.000000
1   ATT-TGAAATAT

Rev.of_WB235_4:6469806-6469955 Satlength=150 Nr of Repeats=10 RepeatLength=12 seed=CAGAATATAT Num.seqs=7 Similarity=0.915344
6   ATTCTGGAATAT

*** ** *****
Consensus:

ATTTGgAATAT

>WB235_Fam_106_11_3 Nr. of seq. 3 Alignment length(with gaps) = 12
Alignment score = 0.722222
WB235_4:5690583-5690638 Satlength=56 Nr of Repeats=5 RepeatLength=11 seed=TCCACTGACC Num.seqs=5 Similarity=0.951515
0   TCCACTGACCA-
WB235_2:6296590-6296638 Satlength=49 Nr of Repeats=4 RepeatLength=12 seed=CCACTGACCA Num.seqs=4 Similarity=0.944444
1   CCCACTGACCAT
Rev.of_WB235_6:13775030-13775085 Satlength=56 Nr of Repeats=5 RepeatLength=11 seed=GTGCTTGGTC Num.seqs=5 Similarity=0.951515
5   AGCACTGACCA-

****
Consensus:

ncCACTGACCA

>WB235_Fam_107_10_3 Nr. of seq. 3 Alignment length(with gaps) = 10
Alignment score = 0.733333
WB235_2:7450879-7451382 Satlength=504 Nr of Repeats=27 RepeatLength=10 seed=TCCATCCCGA Num.seqs=19 Similarity=0.831650
0   TCCATCCCGA
Rev.of_WB235_6:16884509-16885875 Satlength=1367 Nr of Repeats=37 RepeatLength=10 seed=TGGATCGGGA Num.seqs=23 Similarity=0.908300
4   TCCATCCCGA
WB235_6:15470071-15470810 Satlength=740 Nr of Repeats=22 RepeatLength=10 seed=TATCTAGATC Num.seqs=15 Similarity=0.964444
2   TCTATCTAGA

** *** **
Consensus:

TCcATCccGA

>WB235_Fam_108_177_2 Nr. of seq. 2 Alignment length(with gaps) = 177
Alignment score = 1.000000
WB235_5:2389657-2390366 Satlength=710 Nr of Repeats=4 RepeatLength=177 seed=GAGTCTCACG Num.seqs=3 Similarity=0.943196
0
GAGTCTCACGATGTGTCAACGTGTCTCAGTCGGCTTGATCTACGTAGATCTACAAAAAATGCGGGAGAAGAGA
CGCAGACTTATTCTCTGATTTTGCATGAGTAAGAGCGTGCTGGCGTCACATTTTTGTCGGCAAAAAATTCCCG
CATTTTTTTGTAGATCAAACCGTAATGGGACG
WB235_5:2497936-2498645 Satlength=710 Nr of Repeats=4 RepeatLength=177 seed=GAGTCTCACG Num.seqs=3 Similarity=0.943196
0

GAGTCTCACGATGTGTCAACGTGTCTCAGTCGGCTTGATCTACGTAGATCTACAAAAAATGCGGGAGAAGAGA
CGCAGACTTATTCTCTGATTTTGCATGAGTAAGAGCGTGCTGGCGTCACATTTTTGTCGGCAAAAAATTCCCG
CATTTTTTGTAGATCAAACCGTAATGGGACG

****
****
****
Consensus:

GAGTCTCACGATGTGTCAACGTGTCTCAGTCGGCTTGATCTACGTAGATCTACAAAAAATGCGGGAGAAGAGA
CGCAGACTTATTCTCTGATTTTGCATGAGTAAGAGCGTGCTGGCGTCACATTTTTGTCGGCAAAAAATTCCCG
CATTTTTTGTAGATCAAACCGTAATGGGACG

>WB235_Fam_109_151_2 Nr. of seq. 2 Alignment length(with gaps) = 151
Alignment score = 0.924945
WB235_3:3078601-3079369 Satlength=769 Nr of Repeats=4 RepeatLength=151
seed=TTTAAAGGCG Num.seqs=3 Similarity=0.500000
0
TTTAAAGGCGCATAGGTAGNTCAAANTTCAAANTTGACGGATTCTAGTCAATATGGGGCTTATTTTAAAGCTC
TCAGTAAGCTNAATNTANTTNAGCTACTNAAAATTGAANATTTTNAAATTTTTTGGGTCTCGCAGCGAAAATT
TCGAA
WB235_3:3085487-3086399 Satlength=913 Nr of Repeats=5 RepeatLength=151
seed=TTTAAAGGCG Num.seqs=3 Similarity=0.500000
0
TTTAAAGGCGCATAGGTAGTTCAAANTTCAAANTTGACGGATTCTAGTCAATATGGGGCTTATTTTAAAGCTC
TCAGTAAGCTNAATNTANTTNAGCTACTAACAATTGAANATTTTNAAATTTTTTGGGTCTCGCAGCGAAAATT
TCGAA

****
****
******** * ***
Consensus:

TTTAAAGGCGCATAGGTAGtTCAAANTTCAAANTTGACGGATTCTAGTCAATATGGGGCTTATTTTAAAGCTC
TCAGTAAGCTNAATNTANTTNAGCTACTaAaAATTGAANATTTTNAAATTTTTTGGGTCTCGCAGCGAAAATT
TCGAA

>WB235_Fam_110_119_2 Nr. of seq. 2 Alignment length(with gaps) = 119
Alignment score = 0.619048
WB235_1:5640355-5640950 Satlength=596 Nr of Repeats=5 RepeatLength=119
seed=GCCACCTTTA Num.seqs=5 Similarity=0.966387
0
GCCACCTTTAACCCTAATCCAAGCAGAGCAGGCTGTAGGCCTGCTATGCGGTAGCGACACGCTCAGGCCCAAT
AGTCCGGGATGAGGGCCGCAGGCCCTCTTACTCTTAGAAACTTTAG
WB235_5:17086843-17087795 Satlength=953 Nr of Repeats=8 RepeatLength=119
seed=AACTGTGGGC Num.seqs=8 Similarity=0.947979
111
GCTGGCCTTAATCCTAACCCACGCAGAGCAGGATGTAGGCCTGAAACGTGGTTGTTATTCAATCAAGCCTAAC
AACCAGGTTTGAGGGCCGCAGGCCCGAAAACTCTTGGAAACTGTGG

**   * **** ***** *** ********** ********* * * *** * * * *** *** **
* * ** ***************   ****** ****** * *
Consensus:

GCcacCcTTAAcCCTAAcCCAaGCAGAGCAGGaTGTAGGCCTGaaAcGcGGTaGcgAcaCaaTCAaGCCcAAc
AacCaGGgaTGAGGGCCGCAGGCCCgaaaACTCTTaGAAACTgTaG

>WB235_Fam_111_110_2 Nr. of seq. 2 Alignment length(with gaps) = 110
Alignment score = 0.745455
WB235_6:11688529-11688958 Satlength=430 Nr of Repeats=4 RepeatLength=108
seed=TTTCACGTTT Num.seqs=3 Similarity=0.860082
0   TTTCACGTTTTCAGGTGATTATCTAGGTCACGAACTAATTTATTTTGAAAATTGACCT--
GTGGACCTATAACTTTGGAGATATGGGAAATTGAGTATGGGCTTTCAGAT
WB235_6:13242451-13244463 Satlength=2013 Nr of Repeats=18
RepeatLength=108 seed=TTTCAGATTT Num.seqs=17 Similarity=0.875000
100   TTTCACATTTTCACTTGATTATATAGGTCACGACCCAAATTATTTTGAAAATTGAGCTACACGG--
CTGTAACTTTGGAGCTATGGGTAATCAAGTATGAGCTTTCAGAT

****** ****** ******* ********** * ** *************** **   ** **
*********** ****** *** ****** **********
Consensus:

TTTCACaTTTTCAcgTGATTATaTAGGTCACGAaCcAAaTTATTTTGAAAATTGAcCTacacGGacCTaTAAC
TTTGGAGaTATGGGaAATcaAGTATGaGCTTTCAGAT

>WB235_Fam_112_86_2 Nr. of seq. 2 Alignment length(with gaps) = 86
Alignment score = 0.689922
WB235_2:5073308-5077334 Satlength=4027 Nr of Repeats=52 RepeatLength=76
seed=TGTATTTTGA Num.seqs=36 Similarity=0.973545
0   TGTATTTTGAAAGGTCATCTCAATA--GAAAAGATGGAAGACTAC--AG--AA--A-AA-
CTATTGAGAGACCCCGAAATACTGGC
Rev.of_WB235_2:8975477-8977487 Satlength=2011 Nr of Repeats=24
RepeatLength=86 seed=AGCCAGTATT Num.seqs=23 Similarity=0.984619
1
TGTATTTTGAAAGGTCGTGGCAATAGGGAAAAGATGGAAGAGTTCGAAGTTAACGAGAAGCTATTGCCAGACC
CCGAAATACTGGC

**************** * ***** ************* * * ** ** * ** ******
****
Consensus:

TGTATTTTGAAAGGTCaTcgCAATAggGAAAAGATGGAAGAcTaCgaAGttAAcgAgAAgCTATTGacAGACC
CCGAAATACTGGC

>WB235_Fam_113_73_2 Nr. of seq. 2 Alignment length(with gaps) = 73
Alignment score = 0.945205
WB235_1:12192426-12192752 Satlength=327 Nr of Repeats=4 RepeatLength=73
seed=AATAAAATAT Num.seqs=3 Similarity=0.963470
0
AATAAAATATTGGAAATCCCTATGACACACTTAAGCCTAAAGGCCCGAAAAACATACTAGGATGCCCAACTGG
Rev.of_WB235_5:15525755-15526135 Satlength=381 Nr of Repeats=5
RepeatLength=73 seed=TTAGGCTTAA Num.seqs=3 Similarity=0.939117
40
AATAAAATATTGGAAATCCTTATGACACACTTAAGCCTAAAGGCCCGAAAAAAAAACTAGGATGCCCAACTGG

****************** ******************************* * *****************
Consensus:

AATAAAATATTGGAAATCCcTATGACACACTTAAGCCTAAAGGCCCGAAAAAaAaACTAGGATGCCCAACTGG

>WB235_Fam_114_44_2 Nr. of seq. 2 Alignment length(with gaps) = 44
Alignment score = 0.681818
WB235_2:9693772-9694139 Satlength=368 Nr of Repeats=4 RepeatLength=42
seed=CAGCCGACAG Num.seqs=3 Similarity=0.533769
0   -CAGCCGACAGCCGAAAAATT--GGAATTTTCGGCTGGTAGCAC
WB235_5:4029541-4030122 Satlength=582 Nr of Repeats=5 RepeatLength=42
seed=CCAGCCGACA Num.seqs=3 Similarity=0.684755
0   CCAGCCGACAGCCGAAATTTTCACG-ATTTTCGGCTGGTNGCG-

**************** **   * ************* **
Consensus:

cCAGCCGACAGCCGAAAaaTTcacGaATTTTCGGCTGGTaGCac

>WB235_Fam_115_44_2 Nr. of seq. 2 Alignment length(with gaps) = 44
Alignment score = 0.704545
WB235_1:11829562-11829989 Satlength=428 Nr of Repeats=5 RepeatLength=42
seed=TCGGCAAATC Num.seqs=3 Similarity=0.723514
0   TCGGCAAA-TCGGCAATTNCTGGTTTTTTNAA-ATTTGCCGAGC
Rev.of_WB235_4:15776137-15776526 Satlength=390 Nr of Repeats=5
RepeatLength=42 seed=GCTCGGCAAA Num.seqs=3 Similarity=0.957672
0   ACGGCAAATTCGGCAAAT-CT-CTTTTTTCAATATTTGCCGAGC

******* ******* * ** ****** ** ***********
Consensus:

aCGGCAAAtTCGGCAAaTnCTgcTTTTTTcAAtATTTGCCGAGC

>WB235_Fam_116_44_2 Nr. of seq. 2 Alignment length(with gaps) = 44
Alignment score = 0.969697
WB235_1:9636862-9637728 Satlength=867 Nr of Repeats=20 RepeatLength=44
seed=CTGTTTTCTG Num.seqs=18 Similarity=0.984749
0   CTGTTTTCTGAGCATTTTAGATTTTCGGATTGATGGCCGAAAGG
WB235_4:17128248-17129128 Satlength=881 Nr of Repeats=20 RepeatLength=44
seed=GATTGATGGC Num.seqs=20 Similarity=0.962360
27   CTGTTTTCTGAGCATTGTAGATTTTCGGATTGATGGCCGAAAGG

**************** ***************************
Consensus:

CTGTTTTCTGAGCATTgTAGATTTTCGGATTGATGGCCGAAAGG

>WB235_Fam_117_41_2 Nr. of seq. 2 Alignment length(with gaps) = 41
Alignment score = 0.711382
WB235_6:17125270-17125459 Satlength=190 Nr of Repeats=5 RepeatLength=38
seed=CCGTTTCGCC Num.seqs=4 Similarity=0.982456
0   CCGTTTCGCCTTGAGCGATT-C--ACCCTTAAATGGGCCTC
WB235_6:7351473-7361391 Satlength=9919 Nr of Repeats=248 RepeatLength=40
seed=AATGGGCGCC Num.seqs=247 Similarity=0.954892
30   -GGTTTCGCTTTGAGCGATTCCTTACCCTTAAATGGGCGCC

******* *********** * ************** *
Consensus:

ccGTTTCGCcTTGAGCGATTcCttACCCTTAAATGGGCccC

>WB235_Fam_118_41_2 Nr. of seq. 2 Alignment length(with gaps) = 41
Alignment score = 1.000000
WB235_2:7379726-7379898 Satlength=173 Nr of Repeats=4 RepeatLength=41
seed=AAAACGGGAA Num.seqs=3 Similarity=0.978320
0   AAAACGGGAAAACGGGAATTCCCGTTCCCGTGAAAATTTTA
WB235_2:7379725-7380258 Satlength=534 Nr of Repeats=10 RepeatLength=41
seed=AAAAACGGGA Num.seqs=6 Similarity=0.956640
40   AAAACGGGAAAACGGGAATTCCCGTTCCCGTGAAAATTTTA

****
Consensus:

AAAACGGGAAAACGGGAATTCCCGTTCCCGTGAAAATTTTA

>WB235_Fam_119_40_2 Nr. of seq. 2 Alignment length(with gaps) = 40
Alignment score = 0.975000
WB235_3:1056939-1057667 Satlength=729 Nr of Repeats=14 RepeatLength=40
seed=AAAATCCACG Num.seqs=12 Similarity=0.883838
0   AAAATCCACGTAGATTATTGATTTTTCATNGAATTTTGCA
WB235_3:1056932-1058332 Satlength=1401 Nr of Repeats=16 RepeatLength=40
seed=TTTTGCAAAA Num.seqs=11 Similarity=0.759596
33   AAAATCCACGTAGATTATTGATTTTTCATGGAATTTTGCA

***************************** **********
Consensus:

AAAATCCACGTAGATTATTGATTTTTCATgGAATTTTGCA

>WB235_Fam_120_39_2 Nr. of seq. 2 Alignment length(with gaps) = 39
Alignment score = 0.897436
WB235_4:11072392-11075919 Satlength=3528 Nr of Repeats=90 RepeatLength=39
seed=TTATGGAAAA Num.seqs=86 Similarity=0.949069
0   TTATGGAAAAGTGGCTCTAGAGCCACAAGAAATACCAGT
WB235_5:10605694-10612481 Satlength=6788 Nr of Repeats=174
RepeatLength=39 seed=GGCCACCAGA Num.seqs=173 Similarity=0.944141
20   TTATGAAAAAGTGGCTCTAGGGCCACCAGAAATACCAGT

***** ************** ***** ************
Consensus:

TTATGaAAAAGTGGCTCTAGaGCCACaAGAAATACCAGT

>WB235_Fam_121_36_2 Nr. of seq. 2 Alignment length(with gaps) = 36
Alignment score = 0.601852
WB235_4:3291067-3291207 Satlength=141 Nr of Repeats=5 RepeatLength=33
seed=CGGCATATGC Num.seqs=3 Similarity=0.946128
0   CGGCATA-TGCCGTGAATTTTAAAAT-CGGCATAA-

Rev.of_WB235_4:15517213-15517493 Satlength=281 Nr of Repeats=8 RepeatLength=35 seed=TTGCCGGATT Num.seqs=8 Similarity=0.983673
32 -GGCAAATTACCATAAATTTTCAAATCCGGCAAAAC

**** * * ** * ****** **** ***** **
Consensus:

cGGCAaAtTaCCaTaAATTTTaAAATcCGGCAaAAc

>WB235_Fam_122_34_2 Nr. of seq. 2 Alignment length(with gaps) = 34
Alignment score = 0.612745
WB235_4:1515989-1516221 Satlength=233 Nr of Repeats=8 RepeatLength=29 seed=CCACCCGGGA Num.seqs=8 Similarity=0.850575
0 CCACCCGGGAGGTGTCGGCTGCT----CTA-AAC
WB235_5:18577906-18578272 Satlength=367 Nr of Repeats=11 RepeatLength=33 seed=TGTCGGCTGC Num.seqs=10 Similarity=0.876094
11 -CTCCCGGCAGGTGTCGGCTGCTAAAACTATAGC

* ***** ************** *** * *
Consensus:

cCaCCCGGcAGGTGTCGGCTGCTaaaaCTAtAaC

>WB235_Fam_123_32_2 Nr. of seq. 2 Alignment length(with gaps) = 32
Alignment score = 0.671875
WB235_3:255029-255455 Satlength=427 Nr of Repeats=12 RepeatLength=30 seed=TTTTTTGAGC Num.seqs=9 Similarity=0.761574
0 TTTTTTGAGCCAAAAATTC-AAAAAAT-CTCG
WB235_3:244458-244613 Satlength=156 Nr of Repeats=4 RepeatLength=31 seed=AGCTCAAAAT Num.seqs=3 Similarity=0.711806
6 -TTTTTGAGCTCAAAATTCTGAAAAATGCTCA

********* ******* ****** ***
Consensus:

tTTTTTGAGCcaAAAATTCtaAAAAATgCTCa

>WB235_Fam_124_32_2 Nr. of seq. 2 Alignment length(with gaps) = 32
Alignment score = 1.000000
WB235_1:10945938-10952999 Satlength=7062 Nr of Repeats=238 RepeatLength=32 seed=GCACCAAATA Num.seqs=152 Similarity=0.889392
0 GCACCAAATAGCACCAATTTAGCACCGATGGA
Rev.of_WB235_6:12276852-12278772 Satlength=1921 Nr of Repeats=55 RepeatLength=32 seed=GGTGCTATTT Num.seqs=46 Similarity=0.867432
15 GCACCAAATAGCACCAATTTAGCACCGATGGA

****
Consensus:

GCACCAAATAGCACCAATTTAGCACCGATGGA

>WB235_Fam_125_31_2 Nr. of seq. 2 Alignment length(with gaps) = 31
Alignment score = 0.752688

WB235_1:1503613-1504297 Satlength=685 Nr of Repeats=18 RepeatLength=31
seed=GGTTACTGTA Num.seqs=11 Similarity=0.738025
0   GGTTACTGTAACTCAATTATAGGATACTTNG
WB235_2:3496033-3496764 Satlength=732 Nr of Repeats=18 RepeatLength=31
seed=GGTTACTGTA Num.seqs=11 Similarity=0.669318
0   GGTTACTGTAGATCGATCCTAGGATACTTTG

********** ** ** ********** *
Consensus:

GGTTACTGTAaaTCaATcaTAGGATACTTtG

>WB235_Fam_126_31_2 Nr. of seq. 2 Alignment length(with gaps) = 31
Alignment score = 0.784946
WB235_2:2577037-2578087 Satlength=1051 Nr of Repeats=24 RepeatLength=31
seed=CTGAAATTCC Num.seqs=16 Similarity=0.855556
0   CTGAAATTCCAATTTTCCAGTGTAAAAATGT
WB235_5:18737977-18738195 Satlength=219 Nr of Repeats=6 RepeatLength=31
seed=TGTAAAAATG Num.seqs=5 Similarity=0.651613
20   CTGAAATTATGAATTTCCAATGTAAAAATGT

********   * ****** ***********
Consensus:

CTGAAATTacaAaTTTCCAaTGTAAAAATGT

>WB235_Fam_127_31_2 Nr. of seq. 2 Alignment length(with gaps) = 31
Alignment score = 0.956989
WB235_4:15784879-15785128 Satlength=250 Nr of Repeats=6 RepeatLength=31
seed=TCGACAGAAG Num.seqs=4 Similarity=0.665825
0   TCGACAGAAGCTGTCGGAAATCACACTTTCC
WB235_6:12606642-12606920 Satlength=279 Nr of Repeats=9 RepeatLength=31
seed=TCGACAGAAG Num.seqs=8 Similarity=0.729437
0   TCGACAGAAGCTGTCGGACATCACACTTTCC

****************** ************
Consensus:

TCGACAGAAGCTGTCGGAaATCACACTTTCC

>WB235_Fam_128_29_2 Nr. of seq. 2 Alignment length(with gaps) = 29
Alignment score = 1.000000
WB235_1:5152583-5153888 Satlength=1306 Nr of Repeats=45 RepeatLength=29
seed=AAGGATTTCC Num.seqs=45 Similarity=0.955138
0   AAGGATTTCCCACTATGTCAATACATTGT
Rev.of_WB235_5:7988900-7991510 Satlength=2611 Nr of Repeats=90
RepeatLength=29 seed=CATAGTGGGA Num.seqs=90 Similarity=0.932705
17   AAGGATTTCCCACTATGTCAATACATTGT

****
Consensus:

AAGGATTTCCCACTATGTCAATACATTGT

```
>WB235_Fam_129_27_2  Nr. of seq. 2 Alignment length(with gaps) = 27
Alignment score = 0.901235
WB235_2:3102561-3102723 Satlength=163 Nr of Repeats=6 RepeatLength=27
seed=GCTGCTGGAG  Num.seqs=6 Similarity=0.881481
0    GCTGCTGGAGGATCCACTGCATCAACT
WB235_2:3106228-3107119 Satlength=892 Nr of Repeats=33 RepeatLength=27
seed=GCTGCTGGAG  Num.seqs=33 Similarity=0.919285
0    GCTGCTGGAGGATCAACTGCATCAACC

************** ***********
Consensus:

GCTGCTGGAGGATCaACTGCATCAACc

>WB235_Fam_130_26_2  Nr. of seq. 2 Alignment length(with gaps) = 26
Alignment score = 1.000000
WB235_1:1658366-1660394 Satlength=2029 Nr of Repeats=78 RepeatLength=26
seed=CAAAACCGGC  Num.seqs=78 Similarity=0.926569
0    CAAAACCGGCAATTGCCGAAAATTCC
Rev.of_WB235_1:1807418-1807780 Satlength=363 Nr of Repeats=11
RepeatLength=26 seed=TTGCCGGTTT  Num.seqs=8 Similarity=0.798535
12    CAAAACCGGCAATTGCCGAAAATTCC

**************************
Consensus:

CAAAACCGGCAATTGCCGAAAATTCC

>WB235_Fam_131_24_2  Nr. of seq. 2 Alignment length(with gaps) = 24
Alignment score = 0.722222
WB235_2:3753993-3754089 Satlength=97 Nr of Repeats=4 RepeatLength=24
seed=TGCATCTGCC  Num.seqs=4 Similarity=0.750000
0    TGCATCTGCCGCTGGAGCTGGCGC
Rev.of_WB235_5:14320937-14321192 Satlength=256 Nr of Repeats=5
RepeatLength=24 seed=GCCGCTCCAG  Num.seqs=3 Similarity=0.814815
3    GGCATCTGGTGCCGGAGCTGGAGC

*******  ** ******** **
Consensus:

gGCATCTGccGCcGGAGCTGGaGC

>WB235_Fam_132_24_2  Nr. of seq. 2 Alignment length(with gaps) = 24
Alignment score = 0.680556
WB235_2:11365554-11365650 Satlength=97 Nr of Repeats=5 RepeatLength=24
seed=GCTTAGGCTT  Num.seqs=3 Similarity=0.648148
0    GCTTAGGCTTGGGCTTAGGCTNAA
Rev.of_WB235_5:18797535-18797724 Satlength=190 Nr of Repeats=5
RepeatLength=24 seed=GCCTAAGCCT  Num.seqs=3 Similarity=0.666667
2    GCCTAGACTCAGGCTTAGGCTTAG

** *** **  ********** *
Consensus:
```

GCcTAGaCTcaGGCTTAGGCTtAa

>WB235_Fam_133_23_2 Nr. of seq. 2 Alignment length(with gaps) = 23
Alignment score = 0.630435
WB235_1:923168-925176 Satlength=2009 Nr of Repeats=89 RepeatLength=21
seed=AATTTCTAGG Num.seqs=60 Similarity=0.911757
0 AATTTCTAGGCCACGA-TTTGA-
Rev.of_WB235_1:1097742-1098271 Satlength=530 Nr of Repeats=23
RepeatLength=23 seed=GGCCTAGAAA Num.seqs=23 Similarity=0.818525
12 TATTTCTAGGCCACCACGTTCAG

************* * ** *
Consensus:

aATTTCTAGGCCACcAcgTTcAg

>WB235_Fam_134_23_2 Nr. of seq. 2 Alignment length(with gaps) = 23
Alignment score = 0.673913
WB235_5:19354949-19355211 Satlength=263 Nr of Repeats=11 RepeatLength=21
seed=TCTAATTAGA Num.seqs=10 Similarity=0.939330
0 TCTAATTAGAT-CACCTT-TTTT
WB235_5:19356787-19357065 Satlength=279 Nr of Repeats=13 RepeatLength=21
seed=TTCTAATTAG Num.seqs=10 Similarity=0.650000
20 -CTAATTAGATNCGCCNTCTTTT

********** * ** * ****
Consensus:

tCTAATTAGATnCaCCtTcTTTT

>WB235_Fam_135_23_2 Nr. of seq. 2 Alignment length(with gaps) = 23
Alignment score = 0.659420
WB235_5:19679307-19679396 Satlength=90 Nr of Repeats=4 RepeatLength=22
seed=ATTGGATTTT Num.seqs=3 Similarity=0.959596
0 ATTGGATTTTTCACCCCAAAAA-
WB235_1:623235-624175 Satlength=941 Nr of Repeats=22 RepeatLength=23
seed=AAAAAAAATT Num.seqs=14 Similarity=0.950948
16 ATTAAATTTTTCAACAAAAAAAA

*** ******** * *****
Consensus:

ATTaaATTTTTCAaCaaAAAAAa

>WB235_Fam_136_22_2 Nr. of seq. 2 Alignment length(with gaps) = 22
Alignment score = 0.643939
WB235_3:1534930-1535260 Satlength=331 Nr of Repeats=16 RepeatLength=20
seed=CATAAAAATT Num.seqs=13 Similarity=0.947009
0 CATAAAAAT-TCAGAAAA-ATG
Rev.of_WB235_1:13960468-13960552 Satlength=85 Nr of Repeats=4
RepeatLength=21 seed=ATATTTTGTG Num.seqs=4 Similarity=0.936508
0 AATAAAAATGTCACAAAATAT-

******** *** **** **
Consensus:

aATAAAAATgTCAcAAAAtATg

>WB235_Fam_137_22_2 Nr. of seq. 2 Alignment length(with gaps) = 22
Alignment score = 0.681818
WB235_4:1949738-1952318 Satlength=2581 Nr of Repeats=113 RepeatLength=21
seed=GTAAATCTAC Num.seqs=78 Similarity=0.823929
0   GTAAATCTACAGTAAT-NCCCC
WB235_3:999373-999632 Satlength=260 Nr of Repeats=12 RepeatLength=22
seed=TAAATCTACA Num.seqs=10 Similarity=0.692929
1   GTAAATCTACACACATAGCCCC

***********   ** ****
Consensus:

GTAAATCTACAcaaATagCCCC

>WB235_Fam_138_22_2 Nr. of seq. 2 Alignment length(with gaps) = 22
Alignment score = 0.621212
WB235_4:16491101-16491225 Satlength=125 Nr of Repeats=5 RepeatLength=21
seed=TTCAAGCCTT Num.seqs=3 Similarity=0.915344
0   TTCAAGCC-TTTTCAGACCAAT
Rev.of_WB235_1:13768184-13768352 Satlength=169 Nr of Repeats=7
RepeatLength=21 seed=CAAAATTCGT Num.seqs=6 Similarity=0.814646
4   TTTGAGCNATTTTCGGACGAAT

** *** ***** *** ***
Consensus:

TTcaAGCcaTTTTCaGACcAAT

>WB235_Fam_139_22_2 Nr. of seq. 2 Alignment length(with gaps) = 22
Alignment score = 1.000000
WB235_1:13085679-13085769 Satlength=91 Nr of Repeats=4 RepeatLength=22
seed=TCGAGAAAAA Num.seqs=3 Similarity=1.000000
0   TCGAGAAAAAATCTGAAAAAAT
Rev.of_WB235_1:13102088-13102201 Satlength=114 Nr of Repeats=5
RepeatLength=22 seed=TTTTTTCTCG Num.seqs=4 Similarity=1.000000
11   TCGAGAAAAAATCTGAAAAAAT

****
Consensus:

TCGAGAAAAAATCTGAAAAAAT

>WB235_Fam_140_22_2 Nr. of seq. 2 Alignment length(with gaps) = 22
Alignment score = 0.954545
WB235_2:3301138-3301263 Satlength=126 Nr of Repeats=4 RepeatLength=22
seed=ATTCTGCGCA Num.seqs=3 Similarity=0.656566
0   ATTCTGCGCACCCCCTGCGCNG

Rev.of_WB235_5:19315501-19315626 Satlength=126 Nr of Repeats=4 RepeatLength=22 seed=GTGCGCAGAA Num.seqs=3 Similarity=0.676768
11 ATTCTGCGCACCCCCTGCGCAG

******************** *
Consensus:

ATTCTGCGCACCCCCTGCGCaG

>WB235_Fam_141_20_2 Nr. of seq. 2 Alignment length(with gaps) = 20
Alignment score = 0.933333
WB235_1:1755610-1758756 Satlength=3147 Nr of Repeats=154 RepeatLength=20
seed=CCACCAAAAA Num.seqs=145 Similarity=0.874815
0 CCACCAAAAAGGTTTCTAGG
WB235_1:1760846-1762239 Satlength=1394 Nr of Repeats=64 RepeatLength=20
seed=TTCTAGGCCA Num.seqs=51 Similarity=0.812235
13 CCACCAAAAATGTTTCTAGG

********** *********
Consensus:

CCACCAAAAAgGTTTCTAGG

>WB235_Fam_142_20_2 Nr. of seq. 2 Alignment length(with gaps) = 20
Alignment score = 0.950000
WB235_1:2333731-2334442 Satlength=712 Nr of Repeats=8 RepeatLength=20
seed=GGACTAGTTT Num.seqs=6 Similarity=0.880000
0 GGACTAGTTTTCGAGTAGAG
WB235_1:2333731-2334603 Satlength=873 Nr of Repeats=11 RepeatLength=20
seed=GGACTAGTTT Num.seqs=7 Similarity=0.745276
0 GGACTAGTTTTNGAGTAGAG

*********** ********
Consensus:

GGACTAGTTTTcGAGTAGAG

>WB235_Fam_143_20_2 Nr. of seq. 2 Alignment length(with gaps) = 20
Alignment score = 0.866667
WB235_5:2634833-2635037 Satlength=205 Nr of Repeats=7 RepeatLength=20
seed=CCCCGACGCT Num.seqs=5 Similarity=0.920000
0 CCCCGACGCTCGAGCTCAAA
WB235_5:2634964-2635164 Satlength=201 Nr of Repeats=10 RepeatLength=20
seed=GCTCGAGCTC Num.seqs=10 Similarity=0.928889
7 CCCCGAAGCTCGAGCTCAAG

****** ************
Consensus:

CCCCGAaGCTCGAGCTCAAa

>WB235_Fam_144_20_2 Nr. of seq. 2 Alignment length(with gaps) = 20
Alignment score = 0.800000

WB235_6:16050430-16050549 Satlength=120 Nr of Repeats=6 RepeatLength=20
seed=CCACCCTGTA Num.seqs=5 Similarity=0.706349
0   CCACCCTGTAATATCATGGA
Rev.of_WB235_6:16286380-16286520 Satlength=141 Nr of Repeats=7
RepeatLength=20 seed=TACAGGGTGG Num.seqs=5 Similarity=0.920000
10   CCACCCTGTATAATTATGGA

********** ** *****
Consensus:

CCACCCTGTAaaATcATGGA

>WB235_Fam_145_19_2 Nr. of seq. 2 Alignment length(with gaps) = 19
Alignment score = 0.684211
WB235_2:14105236-14105489 Satlength=254 Nr of Repeats=11 RepeatLength=18
seed=CTAGGTGCCT Num.seqs=8 Similarity=0.804233
0   CTAGGTGCCTA-TCCATTC
Rev.of_WB235_4:2861147-2861589 Satlength=443 Nr of Repeats=12
RepeatLength=19 seed=ACCTAGGCAT Num.seqs=8 Similarity=0.751880
6   CTAGGTGCCTACTTTATGC

*********** * ** *
Consensus:

CTAGGTGCCTAcTccATgC

>WB235_Fam_146_19_2 Nr. of seq. 2 Alignment length(with gaps) = 19
Alignment score = 0.929825
WB235_4:2650676-2650942 Satlength=267 Nr of Repeats=11 RepeatLength=19
seed=CGCCAAGTAT Num.seqs=8 Similarity=0.694345
0   CGCCAAGTATCGAGAAAAG
Rev.of_WB235_5:19372325-19372591 Satlength=267 Nr of Repeats=11
RepeatLength=19 seed=GATCCTTGGC Num.seqs=9 Similarity=0.898636
11   CGCCAAGGATCGAGAAAAG

******* ***********
Consensus:

CGCCAAGgATCGAGAAAAG

>WB235_Fam_147_17_2 Nr. of seq. 2 Alignment length(with gaps) = 17
Alignment score = 0.656863
WB235_2:14738853-14738949 Satlength=97 Nr of Repeats=6 RepeatLength=16
seed=GCAGTTTGCC Num.seqs=6 Similarity=0.794444
0   GCAGTTTGCC-GAATTG
Rev.of_WB235_6:16372783-16373033 Satlength=251 Nr of Repeats=11
RepeatLength=16 seed=TTCCGGCAAT Num.seqs=7 Similarity=0.976190
13   -CCGATTGCCGGAATTG

* * ***** ******
Consensus:

gCaGaTTGCCgGAATTG

>WB235_Fam_148_17_2 Nr. of seq. 2 Alignment length(with gaps) = 17
Alignment score = 0.803922
WB235_6:6212839-6212968 Satlength=130 Nr of Repeats=8 RepeatLength=16
seed=GCACTGACAA Num.seqs=7 Similarity=0.876984
0   GCACTGA-CAATTTTTT
Rev.of_WB235_6:1334194-1334262 Satlength=69 Nr of Repeats=4
RepeatLength=17 seed=TGGTCAGTGC Num.seqs=4 Similarity=0.882353
10   GCACTGACCAACTTTTT

******* *** *****
Consensus:

GCACTGAcCAAcTTTTT

>WB235_Fam_149_16_2 Nr. of seq. 2 Alignment length(with gaps) = 16
Alignment score = 0.750000
WB235_5:18410594-18410720 Satlength=127 Nr of Repeats=9 RepeatLength=14
seed=GTCTGGGGTC Num.seqs=9 Similarity=0.978836
1   GGT-CTGGGGT-CTGG
WB235_5:20524563-20524679 Satlength=117 Nr of Repeats=5 RepeatLength=16
seed=GTACTAGGGT Num.seqs=3 Similarity=0.888889
1   GGTACTGGGGTACTGG

** ******* *****
Consensus:

GTaCTGGGGTaCTGGG

>WB235_Fam_150_15_2 Nr. of seq. 2 Alignment length(with gaps) = 15
Alignment score = 0.622222
WB235_1:11069544-11069616 Satlength=73 Nr of Repeats=6 RepeatLength=12
seed=CGATCTCCAC Num.seqs=6 Similarity=0.933333
0   CGAT-CTCCACCA--
WB235_1:11060982-11061207 Satlength=226 Nr of Repeats=15 RepeatLength=15
seed=CGATGCTCCA Num.seqs=15 Similarity=0.900106
0   CGATGCTCCACCCGA

**** *******
Consensus:

CGATgCTCCACCaga

>WB235_Fam_151_14_2 Nr. of seq. 2 Alignment length(with gaps) = 14
Alignment score = 0.761905
WB235_2:14190380-14191034 Satlength=655 Nr of Repeats=13 RepeatLength=13
seed=TATACAAAAA Num.seqs=9 Similarity=0.837607
0   TATACAAAAA-GTA
Rev.of_WB235_1:3712691-3712852 Satlength=162 Nr of Repeats=5
RepeatLength=14 seed=TTTTTGTAGA Num.seqs=3 Similarity=0.588235
10   TCTACAAAAANGTA

* ******** ***
Consensus:

TaTACAAAAAnGTA

>WB235_Fam_152_14_2 Nr. of seq. 2 Alignment length(with gaps) = 14
Alignment score = 0.666667
WB235_5:1718733-1718863 Satlength=131 Nr of Repeats=9 RepeatLength=13
seed=GCTCAAGCTA Num.seqs=6 Similarity=0.815385
0   GCTCAAGC-TAGGA
WB235_6:1850672-1850839 Satlength=168 Nr of Repeats=11 RepeatLength=14
seed=TAGGCCCTCA Num.seqs=8 Similarity=0.823129
9   CCTCAAGCGTAGGC

******* ****
Consensus:

cCTCAAGCgTAGGa

>WB235_Fam_153_14_2 Nr. of seq. 2 Alignment length(with gaps) = 14
Alignment score = 0.619048
WB235_3:11221697-11221865 Satlength=169 Nr of Repeats=11 RepeatLength=14
seed=GATAATTGAA Num.seqs=10 Similarity=0.980952
0   GATAATTGAAAATC
Rev.of_WB235_3:11399826-11400260 Satlength=435 Nr of Repeats=31
RepeatLength=14 seed=AATTATAAAT Num.seqs=31 Similarity=0.976651
0   TATAATTTATAATT

****** * ***
Consensus:

gATAATTgAaAATc

>WB235_Fam_154_11_2 Nr. of seq. 2 Alignment length(with gaps) = 11
Alignment score = 0.878788
WB235_1:2650538-2650582 Satlength=45 Nr of Repeats=4 RepeatLength=11
seed=AAACTTGGGC Num.seqs=4 Similarity=0.770833
0   AAACTTGGGCT
WB235_3:11027127-11027171 Satlength=45 Nr of Repeats=4 RepeatLength=11
seed=AAACTTGGGC Num.seqs=4 Similarity=0.858586
0   AAACTTGGGCA

****
Consensus:

AAACTTGGGCa

>WB235_Fam_155_11_2 Nr. of seq. 2 Alignment length(with gaps) = 11
Alignment score = 0.636364
WB235_2:13372106-13372150 Satlength=45 Nr of Repeats=4 RepeatLength=11
seed=TGGAAAATTG Num.seqs=4 Similarity=1.000000
0   TGGAAAATTGA
WB235_3:7593704-7593825 Satlength=122 Nr of Repeats=11 RepeatLength=11
seed=CGGATAATTA Num.seqs=11 Similarity=1.000000
0   CGGATAATTAA

*** **** *
Consensus:

cGGAaAATTaA

>WB235_Fam_156_11_2 Nr. of seq. 2 Alignment length(with gaps) = 11
Alignment score = 0.636364
WB235_6:404178-404266 Satlength=89 Nr of Repeats=7 RepeatLength=11
seed=CATAACTGAG Num.seqs=6 Similarity=1.000000
0   CATAACTGAGA
Rev.of_WB235_6:3365934-3366110 Satlength=177 Nr of Repeats=12
RepeatLength=11 seed=TAGCTCAGCT Num.seqs=8 Similarity=0.883117
2   TATAGCTGAGC

*** *****
Consensus:

cATAaCTGAGa

>WB235_Fam_157_255_1 Nr. of seq. 1 Alignment length(with gaps) = 255
Alignment score = 0.000000
WB235_5:17522891-17524718 Satlength=1828 Nr of Repeats=7 RepeatLength=255
seed=AACAAGTCGG Num.seqs=5 Similarity=0.720930
0
AACAAGTCGGGAACGTTCGGAAAGCTCTGAGGAAAACTCCGACAATATCATGCTTAAGTACGCAATTGAGCTA
TCTTTATTAGATTCGAAAAAGGACTCTTCAAAAATTGAAGCAATAACGAAGAAGCTTAAGTTGGAACTGGCGA
AGCTTAAATTGGGANTTATGCAGTCTTTATCAGATTCAAAAGACGATTCTTCGACCAATGAAGCAAGTCCGGA
GNACGATCTACCTTCGACTTCTGATGGCAGAACTCC

****
****
****
****
Consensus:

AACAAGTCGGGAACGTTCGGAAAGCTCTGAGGAAAACTCCGACAATATCATGCTTAAGTACGCAATTGAGCTA
TCTTTATTAGATTCGAAAAAGGACTCTTCAAAAATTGAAGCAATAACGAAGAAGCTTAAGTTGGAACTGGCGA
AGCTTAAATTGGGANTTATGCAGTCTTTATCAGATTCAAAAGACGATTCTTCGACCAATGAAGCAAGTCCGGA
GNACGATCTACCTTCGACTTCTGATGGCAGAACTCC

>WB235_Fam_158_230_1 Nr. of seq. 1 Alignment length(with gaps) = 230
Alignment score = 0.000000
WB235_1:10266294-10276924 Satlength=10631 Nr of Repeats=46
RepeatLength=231 seed=GCTGAATTCA Num.seqs=45 Similarity=0.806912
0
GCTGAATTCAGTTGGGTANGATGTGACTTCATGGCTGAGTGGAATATCTTGAAGTTATCAGCACGATGTGTAG
AGTCGAGTGGTCCATCGTATTGTTCAGAAATTGGATAATCAAGATGAGATGGAGATTTCTTGAACAATCCAGT
GATCTTCGATTTCAATGATGGCTCGTCCTTCTTCTCTGTCTCTTCAATCACAACTGGGATGACATGCTCTGGC
AGCTTCTCATA

****
****

****
****
Consensus:

GCTGAATTCAGTTGGGTANGATGTGACTTCATGGCTGAGTGGAATATCTTGAAGTTATCAGCACGATGTGTAG
AGTCGAGTGGTCCATCGTATTGTTCAGAAATTGGATAATCAAGATGAGATGGAGATTTCTTGAACAATCCAGT
GATCTTCGATTTCAATGATGGCTCGTCCTTCTTCTCTGTCTCTTCAATCACAACTGGGATGACATGCTCTGGC
AGCTTCTCATA

>WB235_Fam_159_228_1 Nr. of seq. 1 Alignment length(with gaps) = 228
Alignment score = 0.000000
WB235_3:5941097-5943578 Satlength=2482 Nr of Repeats=10 RepeatLength=228
seed=CCACCAGATC Num.seqs=6 Similarity=0.843765
0
CCACCAGATCAGCAACGTCTCATCTTCGCCGGAAAGCAACTCGAGGACGGCCGCACCCTTTCGGACTACAACA
TCCAGAAGGAATCAACTCTTCATTTGGTTCTCCGTCTGAGAGGAGGTATGCAGATCTTCGTCAAGACATTGAC
CGGAAAGACCATCACCCTCGAAGTCGAAGCCTCCGACACCATCGAAAATGTCAAGGCCAAGATCCAAGACAAG
GAAGGAATC

****
****
****
****
Consensus:

CCACCAGATCAGCAACGTCTCATCTTCGCCGGAAAGCAACTCGAGGACGGCCGCACCCTTTCGGACTACAACA
TCCAGAAGGAATCAACTCTTCATTTGGTTCTCCGTCTGAGAGGAGGTATGCAGATCTTCGTCAAGACATTGAC
CGGAAAGACCATCACCCTCGAAGTCGAAGCCTCCGACACCATCGAAAATGTCAAGGCCAAGATCCAAGACAAG
GAAGGAATC

>WB235_Fam_160_189_1 Nr. of seq. 1 Alignment length(with gaps) = 189
Alignment score = 0.000000
WB235_1:1593120-1594195 Satlength=1076 Nr of Repeats=5 RepeatLength=189
seed=TTTTTTTTGA Num.seqs=3 Similarity=1.000000
0
TTTTTTTTGACTATGGAATTATAAAGAAAATCGAATTTCAAAAAAGCATGTATTAGCCAATGTAAACGCGCTC
CAATGATGATGTAGCCCACGTGGTATCAGAGTGTCTCTTTACGGCTTGATTTAAATACGTAGATCTACAAATT
CACTTATCTGTTTTCTCACGGGTTTAGGATATTTTTTCTGTTA

****
****
****
Consensus:

TTTTTTTTGACTATGGAATTATAAAGAAAATCGAATTTCAAAAAAGCATGTATTAGCCAATGTAAACGCGCTC
CAATGATGATGTAGCCCACGTGGTATCAGAGTGTCTCTTTACGGCTTGATTTAAATACGTAGATCTACAAATT
CACTTATCTGTTTTCTCACGGGTTTAGGATATTTTTTCTGTTA

>WB235_Fam_161_184_1 Nr. of seq. 1 Alignment length(with gaps) = 184
Alignment score = 0.000000
WB235_2:884525-885363 Satlength=839 Nr of Repeats=4 RepeatLength=184
seed=GTTCAAAAGA Num.seqs=3 Similarity=1.000000
0
GTTCAAAAGATACAAAATTTATCTACACATCTACAAGTTAAACTTGCAACTTAGTTAACCAAATTCAAAAGTT

ATGAGCCGGGGAACTATAATAACCAACGTTTCACACTAAAAAAGCCCAAAAAAATTCGAAAATCATTTTTATC
TCATGACGCGGACAAGCTACAAATGTCTAGTTAGGCTT

****
****
****
Consensus:

GTTCAAAAGATACAAAATTTATCTACACATCTACAAGTTAAACTTGCAACTTAGTTAACCAAATTCAAAAGTT
ATGAGCCGGGGAACTATAATAACCAACGTTTCACACTAAAAAAGCCCAAAAAAATTCGAAAATCATTTTTATC
TCATGACGCGGACAAGCTACAAATGTCTAGTTAGGCTT

>WB235_Fam_162_183_1 Nr. of seq. 1 Alignment length(with gaps) = 183
Alignment score = 0.000000
WB235_5:19498459-19499190 Satlength=732 Nr of Repeats=4 RepeatLength=183
seed=TTTCTCTGCG Num.seqs=3 Similarity=0.615789
0
TTTCTCTGCGTCTCGCCTCGCCTCACACTATTTTNCACACCAAACTTTGAGNCTATGTATCTCTGTAACGGGT
TGCGCTTTCTGAAAAATTCCAACTGACGGGATATATAAAATTTNGAATTCTTTCTCACCGTTAAAAAACTAAA
CACCTAGGCAAGACGCAGACAGCGAGANACCCCCAAT

****
****
****
Consensus:

TTTCTCTGCGTCTCGCCTCGCCTCACACTATTTTNCACACCAAACTTTGAGNCTATGTATCTCTGTAACGGGT
TGCGCTTTCTGAAAAATTCCAACTGACGGGATATATAAAATTTNGAATTCTTTCTCACCGTTAAAAAACTAAA
CACCTAGGCAAGACGCAGACAGCGAGANACCCCCAAT

>WB235_Fam_163_181_1 Nr. of seq. 1 Alignment length(with gaps) = 181
Alignment score = 0.000000
WB235_2:6073626-6077427 Satlength=3802 Nr of Repeats=21 RepeatLength=181
seed=TTTTTAACTT Num.seqs=21 Similarity=0.964606
0
TTTTTAACTTTTAAATCCACTAAAACTCGGAATGGTACAGAACATGCAAATTGTAATTTCAAGAACTGAATGT
GGTTTCGAATATTTATGGATTGCTTTATTCTAAAACTGACTCTGAAATATTTTGCATCCATTTGACTACTAAT
TGTCATATTATACCGAACACTACTAACTTTAGTGG

****
****
****
Consensus:

TTTTTAACTTTTAAATCCACTAAAACTCGGAATGGTACAGAACATGCAAATTGTAATTTCAAGAACTGAATGT
GGTTTCGAATATTTATGGATTGCTTTATTCTAAAACTGACTCTGAAATATTTTGCATCCATTTGACTACTAAT
TGTCATATTATACCGAACACTACTAACTTTAGTGG

>WB235_Fam_164_180_1 Nr. of seq. 1 Alignment length(with gaps) = 180
Alignment score = 0.000000
WB235_3:2977664-2978383 Satlength=720 Nr of Repeats=4 RepeatLength=180
seed=TGTCGGCCGC Num.seqs=3 Similarity=0.750153
0
TGTCGGCCGCTGTATCTTTTGCCTCAATTTTTTTTNAATTTTNAAACATNCATATTCCAGTAGATAACATTAT

GAGCTACAGTTTGTTAGTTAAAATTTTATTTCTAAAATTACTAGTTTCTGAGTTATACTCATTCCAACCCACC
CCAAGAGAAATTTCCTAAAAATGAACTCGCGAGG

****
****
****
Consensus:

TGTCGGCCGCTGTATCTTTTGCCTCAATTTTTTTTNAATTTTNAAACATNCATATTCCAGTAGATAACATTAT
GAGCTACAGTTTGTTAGTTAAAATTTTATTTCTAAAATTACTAGTTTCTGAGTTATACTCATTCCAACCCACC
CCAAGAGAAATTTCCTAAAAATGAACTCGCGAGG

>WB235_Fam_165_179_1 Nr. of seq. 1 Alignment length(with gaps) = 179
Alignment score = 0.000000
WB235_1:14024312-14025229 Satlength=918 Nr of Repeats=5 RepeatLength=179
seed=CTACCCAAAT Num.seqs=3 Similarity=0.995034
0
CTACCCAAATTTTTGAAATTTGGAAATTTTTCAAAAATTCTCAAATTTTCAAAATCCCATAATTCAGCCAATT
TTTGAGATTTTCAGATATTTTTTAATTGAAAATTCTGTTTGTCTTATCCATTAAACTATATCGCTGCATCTAC
ATTTTTCACAGTACCCCATGACTTTTCCCGATT

****
****
****
Consensus:

CTACCCAAATTTTTGAAATTTGGAAATTTTTCAAAAATTCTCAAATTTTCAAAATCCCATAATTCAGCCAATT
TTTGAGATTTTCAGATATTTTTTAATTGAAAATTCTGTTTGTCTTATCCATTAAACTATATCGCTGCATCTAC
ATTTTTCACAGTACCCCATGACTTTTCCCGATT

>WB235_Fam_166_177_1 Nr. of seq. 1 Alignment length(with gaps) = 177
Alignment score = 0.000000
WB235_1:14148103-14148989 Satlength=887 Nr of Repeats=5 RepeatLength=177
seed=CTAACAAAAT Num.seqs=4 Similarity=0.721286
0
CTAACAAAATAATCAGCGTAAAATTTCCTACATTTTACTAGTTAACAATATTTTGATAACATCAGTGGTTTTT
GAGATACGCAAGTTTTAAGGTGGACGCCCAAAAATAGTTGTGAGCAGCTTTAATCAAAAACCGACATAACTTT
TTACTCACTAACGGTATCAAAAATGTTTTAA

****
****
****
Consensus:

CTAACAAAATAATCAGCGTAAAATTTCCTACATTTTACTAGTTAACAATATTTTGATAACATCAGTGGTTTTT
GAGATACGCAAGTTTTAAGGTGGACGCCCAAAAATAGTTGTGAGCAGCTTTAATCAAAAACCGACATAACTTT
TTACTCACTAACGGTATCAAAAATGTTTTAA

>WB235_Fam_167_175_1 Nr. of seq. 1 Alignment length(with gaps) = 175
Alignment score = 0.000000
WB235_1:2430196-2431177 Satlength=982 Nr of Repeats=5 RepeatLength=175
seed=GATGCACCAT Num.seqs=3 Similarity=0.840552
0
GATGCACCATGTCTTTGACAGTCAATATCTCGGTTTATTTTGTAGCTATCAAAAAATTTTCAACTACAAATCT

ATAGAAAATTAAATGTTCAACATTTTCTCAGTTGACAGTTTTTTGATAACTGCAATGACATCCGAGATATGAG
CTTTCAAAGTTGAGAGACCTCACTCGGTT

****
****
****
Consensus:

GATGCACCATGTCTTTGACAGTCAATATCTCGGTTTATTTTGTAGCTATCAAAAAATTTTCAACTACAAATCT
ATAGAAAATTAAATGTTCAACATTTTCTCAGTTGACAGTTTTTTGATAACTGCAATGACATCCGAGATATGAG
CTTTCAAAGTTGAGAGACCTCACTCGGTT

>WB235_Fam_168_175_1 Nr. of seq. 1 Alignment length(with gaps) = 175
Alignment score = 0.000000
WB235_4:2675283-2676104 Satlength=822 Nr of Repeats=4 RepeatLength=175
seed=AACTTGTGAC Num.seqs=3 Similarity=0.715980
0
AACTTGTGACGGTGGGTATGGATTTTATGCACATAATTTGTTCGAAATAGTCTACCNAGTAAACTGGTGAAGT
TTCAAAAACTTTGTGGATCTAGTTTTTGAAAAAAGTTCAAAATTGTGCCAATTTTGACCAATTTTGGTGGTTT
TCTGAGCTAAAATTGAAAAGTTGGGAATG

****
****
****
Consensus:

AACTTGTGACGGTGGGTATGGATTTTATGCACATAATTTGTTCGAAATAGTCTACCNAGTAAACTGGTGAAGT
TTCAAAAACTTTGTGGATCTAGTTTTTGAAAAAAGTTCAAAATTGTGCCAATTTTGACCAATTTTGGTGGTTT
TCTGAGCTAAAATTGAAAAGTTGGGAATG

>WB235_Fam_169_175_1 Nr. of seq. 1 Alignment length(with gaps) = 175
Alignment score = 0.000000
WB235_5:317369-318431 Satlength=1063 Nr of Repeats=6 RepeatLength=175
seed=AAATTTTGGG Num.seqs=4 Similarity=0.911111
0
AAATTTTGGGTTCACTCTTTTTACTAGACAGTGATTGTTGCATTGTAGGATTCGGTGGACTTGTCCAACAACT
TACTTTGGCATGAGTTTAATAAAAAGGTTTTACCATGAACTGAATCAATTCAAAATAAAAACCTCGAGAATGA
AAACATCCTCAGTAATTTCAATCCTGTTC

****
****
****
Consensus:

AAATTTTGGGTTCACTCTTTTTACTAGACAGTGATTGTTGCATTGTAGGATTCGGTGGACTTGTCCAACAACT
TACTTTGGCATGAGTTTAATAAAAAGGTTTTACCATGAACTGAATCAATTCAAAATAAAAACCTCGAGAATGA
AAACATCCTCAGTAATTTCAATCCTGTTC

>WB235_Fam_170_174_1 Nr. of seq. 1 Alignment length(with gaps) = 174
Alignment score = 0.000000
WB235_1:4338841-4339537 Satlength=697 Nr of Repeats=4 RepeatLength=174
seed=CTTCAAGAGC Num.seqs=4 Similarity=0.868455
0
CTTCAAGAGCTCAGTGTTATTTATGACATGCCAACCTCGACAATTGACCTGAACATGCTCCAAGGAATCGAAG

ACAACTTGAACAGCCTTCCAGCTGAAGAATCTGATAAGATCCGAGAGAAGATTAACGATCTGAGAAGAAGGAA
ACAAGAAAGCGATCAAGCTGAAGCTCTT

****
****
****
Consensus:

CTTCAAGAGCTCAGTGTTATTTATGACATGCCAACCTCGACAATTGACCTGAACATGCTCCAAGGAATCGAAG
ACAACTTGAACAGCCTTCCAGCTGAAGAATCTGATAAGATCCGAGAGAAGATTAACGATCTGAGAAGAAGGAA
ACAAGAAAGCGATCAAGCTGAAGCTCTT

>WB235_Fam_171_165_1 Nr. of seq. 1 Alignment length(with gaps) = 165
Alignment score = 0.000000
WB235_6:1718471-1719647 Satlength=1177 Nr of Repeats=4 RepeatLength=165
seed=CTTTTCCAAA Num.seqs=3 Similarity=1.000000
0
CTTTTCCAAAATGGCAATTTTTGATGAAAAAAAATCCGAATTTTCACAAATTTCAAAAAAATTTTTAAACTTTA
TAATTTTAGCTTTGAATATTCCAAATGGAACTGTCATAACCTATCAATTTGATATATAAACTCGTGCCTACCT
TCAGGCATTCAGTTTTCAC

****
****
****
Consensus:

CTTTTCCAAAATGGCAATTTTTGATGAAAAAAAATCCGAATTTTCACAAATTTCAAAAAAATTTTTAAACTTTA
TAATTTTAGCTTTGAATATTCCAAATGGAACTGTCATAACCTATCAATTTGATATATAAACTCGTGCCTACCT
TCAGGCATTCAGTTTTCAC

>WB235_Fam_172_155_1 Nr. of seq. 1 Alignment length(with gaps) = 155
Alignment score = 0.000000
WB235_2:2949876-2951271 Satlength=1396 Nr of Repeats=9 RepeatLength=155
seed=TCCCGCTGTG Num.seqs=6 Similarity=0.781691
0
TCCCGCTGTGGTTAAAAGTTTTTTGCGTGCTCAGTTCTGTACGGTAAATGTGTTAAAAACTTCTGGAAACTTG
AAATTCCAAGTTATTCAGAAAATTGTTCAAATGGCGCAGTGGGAAATTCGATTTCTCAGACCCAAGAGGCCAC
GGGTTCAAT

****
****
****
Consensus:

TCCCGCTGTGGTTAAAAGTTTTTTGCGTGCTCAGTTCTGTACGGTAAATGTGTTAAAAACTTCTGGAAACTTG
AAATTCCAAGTTATTCAGAAAATTGTTCAAATGGCGCAGTGGGAAATTCGATTTCTCAGACCCAAGAGGCCAC
GGGTTCAAT

>WB235_Fam_173_153_1 Nr. of seq. 1 Alignment length(with gaps) = 153
Alignment score = 0.000000
WB235_6:13923839-13924439 Satlength=601 Nr of Repeats=4 RepeatLength=153
seed=TGAGGTGCAG Num.seqs=3 Similarity=0.872186
0
TGAGGTGCAGGGCGTGATGAGCTTTGTTGAACTGGGAACTGTTGATTCCCGGAGCCGTGATTCATAGATGAGT

TGGAGGGACCTGCTGTGGAACTTGTTTCAACTTGAGCAATTCCATGAGCCATCAAGAAGCTTTCCGGCAGATT
GGGCAAC

****
****
****
Consensus:

TGAGGTGCAGGGCGTGATGAGCTTTGTTGAACTGGGAACTGTTGATTCCCGGAGCCGTGATTCATAGATGAGT
TGGAGGGACCTGCTGTGGAACTTGTTTCAACTTGAGCAATTCCATGAGCCATCAAGAAGCTTTCCGGCAGATT
GGGCAAC

>WB235_Fam_174_151_1 Nr. of seq. 1 Alignment length(with gaps) = 151
Alignment score = 0.000000
WB235_5:14053790-14054393 Satlength=604 Nr of Repeats=4 RepeatLength=151
seed=CATTTCAGAT Num.seqs=3 Similarity=0.988227
0
CATTTCAGATATTCCATCACCACCAACACCTTCATCCACTTCTTCGCCTGGAACGACGGGATTTTCTACAAAA
ATCGGATTTTTTCTCTATGTCAGTTTAATTGATGTATAATTCCGAAAAGTCAGTCTTTCCTAGGTTTTTAAAT
TGAAA

****
****
*****
Consensus:

CATTTCAGATATTCCATCACCACCAACACCTTCATCCACTTCTTCGCCTGGAACGACGGGATTTTCTACAAAA
ATCGGATTTTTTCTCTATGTCAGTTTAATTGATGTATAATTCCGAAAAGTCAGTCTTTCCTAGGTTTTTAAAT
TGAAA

>WB235_Fam_175_150_1 Nr. of seq. 1 Alignment length(with gaps) = 150
Alignment score = 0.000000
WB235_2:1496871-1497776 Satlength=906 Nr of Repeats=6 RepeatLength=150
seed=TTGCAACATG Num.seqs=4 Similarity=0.817145
0
TTGCAACATGCGGTCGGCGGAAAATTCGAATTTTTCAGTCCAAAACAGGTTTTTCGACCAATTTTTCATATTT
TCAAGCGAAATTATGATAGAAAATTCAAATTTTCACTAAAAATCGCCAATTTTCCACATTTTCTCATTGGAGC
GCAC

****
****
****
Consensus:

TTGCAACATGCGGTCGGCGGAAAATTCGAATTTTTCAGTCCAAAACAGGTTTTTCGACCAATTTTTCATATTT
TCAAGCGAAATTATGATAGAAAATTCAAATTTTCACTAAAAATCGCCAATTTTCCACATTTTCTCATTGGAGC
GCAC

>WB235_Fam_176_150_1 Nr. of seq. 1 Alignment length(with gaps) = 150
Alignment score = 0.000000
WB235_4:16681092-16681615 Satlength=524 Nr of Repeats=4 RepeatLength=150
seed=TTGAGGGCCG Num.seqs=3 Similarity=0.940741
0
TTGAGGGCCGCAGTCCCGAATACACGAAGAAGTGTTGGAGATCCGTCAAGCCTATTCTAAGCATAATAATTCA

GTTCGAGTGTCGCAAGCCCAAATAGACGCAGAAGCGTTGGACAACTTTTAGGCCTATTCTGAGCCCAATAATT
CATG

****
****
****
Consensus:

TTGAGGGCCGCAGTCCCGAATACACGAAGAAGTGTTGGAGATCCGTCAAGCCTATTCTAAGCATAATAATTCA
GTTCGAGTGTCGCAAGCCCAAATAGACGCAGAAGCGTTGGACAACTTTTAGGCCTATTCTGAGCCCAATAATT
CATG

>WB235_Fam_177_146_1 Nr. of seq. 1 Alignment length(with gaps) = 146
Alignment score = 0.000000
WB235_2:2811262-2812302 Satlength=1041 Nr of Repeats=6 RepeatLength=144
seed=TGGCCTAGAA Num.seqs=4 Similarity=0.634815
0
TGGCCTAGAAATCNCAAGTTTGCAAAAGTTAGGCCACCAACTTCAAATGCCTATAACTCAGCGGAAAATCAAT
ATTTTCCATTGATTTTAACTGCTAAGATGTAGCCAATTACGTAGCGCAACTTTTTGGCCTACTTAACAATTGG

****
****
Consensus:

TGGCCTAGAAATCNCAAGTTTGCAAAAGTTAGGCCACCAACTTCAAATGCCTATAACTCAGCGGAAAATCAAT
ATTTTCCATTGATTTTAACTGCTAAGATGTAGCCAATTACGTAGCGCAACTTTTTGGCCTACTTAACAATTGG

>WB235_Fam_178_144_1 Nr. of seq. 1 Alignment length(with gaps) = 144
Alignment score = 0.000000
WB235_4:2431055-2431771 Satlength=717 Nr of Repeats=5 RepeatLength=144
seed=TTCTAGGCCA Num.seqs=4 Similarity=0.915326
0
TTCTAGGCCACCAATTGGAAATCATCATAACTTGGCTTAAAATCCAGATTTTTGGAGTCTGGTAACTGCAACA
TGTTGCCCTGTAGTTTTTCTACAAGTTAGGATATATTAATTTTTGGTGGCCTAAAAGTCCAAATTATGGAT

****
****
Consensus:

TTCTAGGCCACCAATTGGAAATCATCATAACTTGGCTTAAAATCCAGATTTTTGGAGTCTGGTAACTGCAACA
TGTTGCCCTGTAGTTTTTCTACAAGTTAGGATATATTAATTTTTGGTGGCCTAAAAGTCCAAATTATGGAT

>WB235_Fam_179_143_1 Nr. of seq. 1 Alignment length(with gaps) = 143
Alignment score = 0.000000
WB235_5:17747299-17748649 Satlength=1351 Nr of Repeats=6 RepeatLength=143
seed=CAGTGGGATT Num.seqs=4 Similarity=0.759132
0
CAGTGGGATTTTCAATGTCTTCCAATCAAACGTCCGGGGTTCGAGTCCGCACAGGGGTATTTATTTTTTGCAA
GCTGTGATTAACAAGTTTATAGCTGCAAAAGTCATTTTAATCGAACTTTCAGGAAAATGTCCGAATGGCG

****
****
Consensus:

CAGTGGGATTTTCAATGTCTTCCAATCAAACGTCCGGGGTTCGAGTCCGCACAGGGGTATTTATTTTTTGCAA
GCTGTGATTAACAAGTTTATAGCTGCAAAAGTCATTTTAATCGAACTTTCAGGAAAATGTCCGAATGGCG

>WB235_Fam_180_122_1 Nr. of seq. 1 Alignment length(with gaps) = 122
Alignment score = 0.000000
WB235_2:13334193-13334831 Satlength=639 Nr of Repeats=5 RepeatLength=122
seed=GATCTACAAA Num.seqs=3 Similarity=0.927140
0
GATCTACAAAAATGCGGGAAAAGTGACACAGAGTTCTCAACTGATTTTGCATAAAAATTCCCGCATTTTTTGG
AGATCAAACCGTGATGGGACAGCCTGGCACCACTTGCTTGATCTACGTA

****
****
Consensus:

GATCTACAAAAATGCGGGAAAAGTGACACAGAGTTCTCAACTGATTTTGCATAAAAATTCCCGCATTTTTTGG
AGATCAAACCGTGATGGGACAGCCTGGCACCACTTGCTTGATCTACGTA

>WB235_Fam_181_121_1 Nr. of seq. 1 Alignment length(with gaps) = 121
Alignment score = 0.000000
WB235_5:16449792-16451728 Satlength=1937 Nr of Repeats=16
RepeatLength=121 seed=AAAAGATAGT Num.seqs=16 Similarity=0.955556
0
AAAAGATAGTTGCCCTAAGAGCAGAAGGTTGCAAACCGCAAGAGCAAACTTGCCAAATGACAAGCCACCCAAG
TACTCGTTGCACCTACCTATCATAAGCTCGTAGACTATAATTTCTCTG

****
****
Consensus:

AAAAGATAGTTGCCCTAAGAGCAGAAGGTTGCAAACCGCAAGAGCAAACTTGCCAAATGACAAGCCACCCAAG
TACTCGTTGCACCTACCTATCATAAGCTCGTAGACTATAATTTCTCTG

>WB235_Fam_182_117_1 Nr. of seq. 1 Alignment length(with gaps) = 117
Alignment score = 0.000000
WB235_5:6176242-6183220 Satlength=6979 Nr of Repeats=54 RepeatLength=117
seed=TGTTGGTTCG Num.seqs=49 Similarity=0.881703
0
TGTTGGTTCGACAGTAGGTGCACTAGTCTCTGGCACTGGAACATCCTTTTGCTCAACAATTTCAGCTGGTTGT
ACTTCAGATGTTTCTTTTGATTCCACAGGTGCAAGTTTCTCAAC

****
****
Consensus:

TGTTGGTTCGACAGTAGGTGCACTAGTCTCTGGCACTGGAACATCCTTTTGCTCAACAATTTCAGCTGGTTGT
ACTTCAGATGTTTCTTTTGATTCCACAGGTGCAAGTTTCTCAAC

>WB235_Fam_183_114_1 Nr. of seq. 1 Alignment length(with gaps) = 114
Alignment score = 0.000000
WB235_5:8705331-8709788 Satlength=4458 Nr of Repeats=38 RepeatLength=114
seed=GTGTTTCAGA Num.seqs=36 Similarity=0.968737
0

GTGTTTCAGAATTACATGGAGTCTCACTACTTGCAGAATTTGTGGCTGTAGACGATGGGCCTGCTGTAGTTGA
ATCGTTTGGAACTGTCATAGTGGATGTTCCATCAGACGTTT

****
****
Consensus:

GTGTTTCAGAATTACATGGAGTCTCACTACTTGCAGAATTTGTGGCTGTAGACGATGGGCCTGCTGTAGTTGA
ATCGTTTGGAACTGTCATAGTGGATGTTCCATCAGACGTTT

>WB235_Fam_184_108_1 Nr. of seq. 1 Alignment length(with gaps) = 108
Alignment score = 0.000000
WB235_6:1549825-1550305 Satlength=481 Nr of Repeats=5 RepeatLength=108
seed=ATCCTCAACA Num.seqs=3 Similarity=0.732510
0
ATCCTCAACACCATCNGCGTCATCTAGTTCCTCCGGAACTATGTCGACAATTTCAGGATCAACTGGTTCNACA
GTTACAGTTGTACCAGGATCNTCTAGCACATTTGG

****
****
Consensus:

ATCCTCAACACCATCNGCGTCATCTAGTTCCTCCGGAACTATGTCGACAATTTCAGGATCAACTGGTTCNACA
GTTACAGTTGTACCAGGATCNTCTAGCACATTTGG

>WB235_Fam_185_108_1 Nr. of seq. 1 Alignment length(with gaps) = 108
Alignment score = 0.000000
WB235_6:13684248-13684998 Satlength=751 Nr of Repeats=7 RepeatLength=108
seed=TGGGTAATCA Num.seqs=6 Similarity=0.845267
0
TGGGTAATCAGGTACGGGCTTTCAGATTTTCAAGTTTTCACGTGAATATCTAGGTCACGAGCCAATTTCAGCC
AGGAGTGGGACTAGAGATATGAAACTCTTGGGTTA

****
****
Consensus:

TGGGTAATCAGGTACGGGCTTTCAGATTTTCAAGTTTTCACGTGAATATCTAGGTCACGAGCCAATTTCAGCC
AGGAGTGGGACTAGAGATATGAAACTCTTGGGTTA

>WB235_Fam_186_108_1 Nr. of seq. 1 Alignment length(with gaps) = 108
Alignment score = 0.000000
WB235_6:15226880-15229061 Satlength=2182 Nr of Repeats=20
RepeatLength=108 seed=AAGTCTTCTT Num.seqs=18 Similarity=0.959655
0
AAGTCTTCTTGATTCTGAGTTCTTGCTTCGAACTGTTGACCATCTCCTTGCAGGATCTTATTGTCAGATGGAA
CCTTCTTGGCATACCGTTCTCCACTGATGTGAGTG

****
****
Consensus:

AAGTCTTCTTGATTCTGAGTTCTTGCTTCGAACTGTTGACCATCTCCTTGCAGGATCTTATTGTCAGATGGAA
CCTTCTTGGCATACCGTTCTCCACTGATGTGAGTG

>WB235_Fam_187_104_1 Nr. of seq. 1 Alignment length(with gaps) = 104
Alignment score = 0.000000
WB235_4:2397301-2398030 Satlength=730 Nr of Repeats=7 RepeatLength=104
seed=CCTGCGAAAG Num.seqs=6 Similarity=0.982906
0
CCTGCGAAAGCGAATCTCTGGCCTGGCGGAGGGCCAACTAACTCTCTCCCTCAAGCCTTCGGGTGCTTCCTGG
GTTATCGGTGAATTTTTGGGTCTGTCAGGGT

****
****
Consensus:

CCTGCGAAAGCGAATCTCTGGCCTGGCGGAGGGCCAACTAACTCTCTCCCTCAAGCCTTCGGGTGCTTCCTGG
GTTATCGGTGAATTTTTGGGTCTGTCAGGGT

>WB235_Fam_188_102_1 Nr. of seq. 1 Alignment length(with gaps) = 102
Alignment score = 0.000000
WB235_4:688812-698192 Satlength=9381 Nr of Repeats=92 RepeatLength=102
seed=TTGCTGCAGC Num.seqs=90 Similarity=0.947271
0
TTGCTGCAGCATATCTTTCTCCAGTACCACCTTGATATGTCGAACGATTGATAGTTTGAGAATCAAGAGAATC
TGTTGGACGATCAATTCCATCTTTGGGTC

****
****
Consensus:

TTGCTGCAGCATATCTTTCTCCAGTACCACCTTGATATGTCGAACGATTGATAGTTTGAGAATCAAGAGAATC
TGTTGGACGATCAATTCCATCTTTGGGTC

>WB235_Fam_189_102_1 Nr. of seq. 1 Alignment length(with gaps) = 102
Alignment score = 0.000000
WB235_6:1136184-1140765 Satlength=4582 Nr of Repeats=45 RepeatLength=102
seed=TGTACACTTC Num.seqs=30 Similarity=0.981008
0
TGTACACTTCTCCCAAAATGACTGTAAGAATGGACTCCTTATATGATTTTAAACCCGACTCTGGACATACAAA
CTTCTCTCAAAAAGAAAATAGACTTCACA

****
****
Consensus:

TGTACACTTCTCCCAAAATGACTGTAAGAATGGACTCCTTATATGATTTTAAACCCGACTCTGGACATACAAA
CTTCTCTCAAAAAGAAAATAGACTTCACA

>WB235_Fam_190_101_1 Nr. of seq. 1 Alignment length(with gaps) = 101
Alignment score = 0.000000
WB235_1:14329713-14330118 Satlength=406 Nr of Repeats=4 RepeatLength=101
seed=GCATTTGCAT Num.seqs=3 Similarity=0.826797
0
GCATTTGCATATNGAGATCAGATAAAAGTTAGAATTTTCCAGCCGTTGCAAGTGCGCTCTATTGAGAACATTT
TGAAAATTGAATGTTTTCACATTAGAGC

****
****
Consensus:

GCATTTGCATATNGAGATCAGATAAAAGTTAGAATTTTCCAGCCGTTGCAAGTGCGCTCTATTGAGAACATTT
TGAAAATTGAATGTTTTCACATTAGAGC

>WB235_Fam_191_101_1 Nr. of seq. 1 Alignment length(with gaps) = 101
Alignment score = 0.000000
WB235_3:267063-268369 Satlength=1307 Nr of Repeats=13 RepeatLength=101
seed=GGTTACTGTA Num.seqs=11 Similarity=0.942394
0
GGTTACTGTAGCTCCAGAAATACGCAAACACCGACTACGGTTCCCTAATACATCAGATATTACGACAAAGTGC
AGAAAATCGCTAAAAATGACTTTACTGG

****
****
Consensus:

GGTTACTGTAGCTCCAGAAATACGCAAACACCGACTACGGTTCCCTAATACATCAGATATTACGACAAAGTGC
AGAAAATCGCTAAAAATGACTTTACTGG

>WB235_Fam_192_101_1 Nr. of seq. 1 Alignment length(with gaps) = 101
Alignment score = 0.000000
WB235_4:1569894-1570719 Satlength=826 Nr of Repeats=7 RepeatLength=101
seed=TTGATTTTTC Num.seqs=5 Similarity=0.952475
0
TTGATTTTTCGTCTTATGAAGGAATATTGGTTTCGGGGTAGTGGGCGGATATGGTCGGGGTACTGTAGGTATA
CGGTAGGGTTACTGTAGTTTTGGAAATT

****
****
Consensus:

TTGATTTTTCGTCTTATGAAGGAATATTGGTTTCGGGGTAGTGGGCGGATATGGTCGGGGTACTGTAGGTATA
CGGTAGGGTTACTGTAGTTTTGGAAATT

>WB235_Fam_193_96_1 Nr. of seq. 1 Alignment length(with gaps) = 96
Alignment score = 0.000000
WB235_4:12314464-12315037 Satlength=574 Nr of Repeats=6 RepeatLength=96
seed=GGTTGATTTG Num.seqs=5 Similarity=0.991667
0
GGTTGATTTGCGGCACTCTACAGATAGTGGATCTGCTTCTTACCAATCTTCCCGTTACCGCCACGCACTTAAG
CTCCAAACTATTTTATTTTTTTG

****
****
Consensus:

GGTTGATTTGCGGCACTCTACAGATAGTGGATCTGCTTCTTACCAATCTTCCCGTTACCGCCACGCACTTAAG
CTCCAAACTATTTTATTTTTTTG

>WB235_Fam_194_96_1 Nr. of seq. 1 Alignment length(with gaps) = 96
Alignment score = 0.000000
WB235_4:12638029-12638605 Satlength=577 Nr of Repeats=6 RepeatLength=96
seed=CTTCTGGTAA Num.seqs=6 Similarity=0.952778
0
CTTCTGGTAATGTTCCCATAATTGGGTTAAATACCACTCATAAGTAACTAGTATGGGACTGAAAAGATACTAA
ATGAGCTTATTCTAAGGGTGAAG

****
****
Consensus:

CTTCTGGTAATGTTCCCATAATTGGGTTAAATACCACTCATAAGTAACTAGTATGGGACTGAAAAGATACTAA
ATGAGCTTATTCTAAGGGTGAAG

>WB235_Fam_195_93_1 Nr. of seq. 1 Alignment length(with gaps) = 93
Alignment score = 0.000000
WB235_4:6921936-6922772 Satlength=837 Nr of Repeats=9 RepeatLength=93
seed=TGCTTCTCGT Num.seqs=8 Similarity=0.966206
0
TGCTTCTCGTCGTCCACCACCACCACCGAAGGGAACTGGAACCCCACCACCACCACCAACTGGAGAACCACAG
GATCTTTCTGGAGAAGGTAA

****
****
Consensus:

TGCTTCTCGTCGTCCACCACCACCACCGAAGGGAACTGGAACCCCACCACCACCACCAACTGGAGAACCACAG
GATCTTTCTGGAGAAGGTAA

>WB235_Fam_196_93_1 Nr. of seq. 1 Alignment length(with gaps) = 93
Alignment score = 0.000000
WB235_5:1907825-1908395 Satlength=571 Nr of Repeats=7 RepeatLength=93
seed=CAGTACCCTC Num.seqs=5 Similarity=0.954122
0
CAGTACCCTCAAGGATCATCTGCTCCACGGTATCCCGGTAACTACAATGCCTTTGCCAGCACTTCCGATGCTC
CACAGTATCCAGGATCCTCC

****
****
Consensus:

CAGTACCCTCAAGGATCATCTGCTCCACGGTATCCCGGTAACTACAATGCCTTTGCCAGCACTTCCGATGCTC
CACAGTATCCAGGATCCTCC

>WB235_Fam_197_87_1 Nr. of seq. 1 Alignment length(with gaps) = 87
Alignment score = 0.000000
WB235_4:2815904-2816246 Satlength=343 Nr of Repeats=4 RepeatLength=87
seed=TGAAGTCGTA Num.seqs=3 Similarity=0.685393
0
TGAAGTCGTAGGATTCTGTGTGGTGACTGGTGTCGTTGTANTCTGTGAAGGGGACGTGGCTTCTTCCGTTGTG
TCATGCTTCTNNGG

****
****
Consensus:

TGAAGTCGTAGGATTCTGTGTGGTGACTGGTGTCGTTGTANTCTGTGAAGGGGACGTGGCTTCTTCCGTTGTG
TCATGCTTCTNNGG

>WB235_Fam_198_81_1 Nr. of seq. 1 Alignment length(with gaps) = 81
Alignment score = 0.000000
WB235_1:14443057-14443637 Satlength=581 Nr of Repeats=8 RepeatLength=73
seed=TGTCGGCCGC Num.seqs=6 Similarity=0.624829
0
TGTCGGCCGCTACCAAACTATTTAGCANACACACCAAGCACTACGTTGCGCACACACACCAAACTGCGGAACC
CCGAACAG

****
****
Consensus:

TGTCGGCCGCTACCAAACTATTTAGCANACACACCAAGCACTACGTTGCGCACACACACCAAACTGCGGAACC
CCGAACAG

>WB235_Fam_199_80_1 Nr. of seq. 1 Alignment length(with gaps) = 80
Alignment score = 0.000000
WB235_4:1965986-1967712 Satlength=1727 Nr of Repeats=15 RepeatLength=80
seed=AATTTCTCAA Num.seqs=14 Similarity=0.856227
0
AATTTCTCAATAGGGCGCGCTTGCAACATCTGAACGGCGCGAAATTTGAATTTTTGGCCATAAAATAACGAAA
ACTAACT

****
****
Consensus:

AATTTCTCAATAGGGCGCGCTTGCAACATCTGAACGGCGCGAAATTTGAATTTTTGGCCATAAAATAACGAAA
ACTAACT

>WB235_Fam_200_78_1 Nr. of seq. 1 Alignment length(with gaps) = 78
Alignment score = 0.000000
WB235_3:12985357-12986137 Satlength=781 Nr of Repeats=10 RepeatLength=78
seed=CGAAGTTGCT Num.seqs=10 Similarity=0.831974
0
CGAAGTTGCTGGACCAAAGATCACAACAGGCGGATTTGGCGGCATAACCGGAGCAACAGTAGTGTCATTTGGT
TCTTC

****
*****
Consensus:

CGAAGTTGCTGGACCAAAGATCACAACAGGCGGATTTGGCGGCATAACCGGAGCAACAGTAGTGTCATTTGGT
TCTTC

>WB235_Fam_201_78_1 Nr. of seq. 1 Alignment length(with gaps) = 78
Alignment score = 0.000000
WB235_5:8714029-8714965 Satlength=937 Nr of Repeats=12 RepeatLength=78
seed=TGCAAGGCTC Num.seqs=12 Similarity=0.929325
0
TGCAAGGCTCCGATGATGATACCACTTCTAAAGTCGAGGTGTCACAATTTTCGGGTGTCGTAGAACTTGAATC
AGTAC

****
*****
Consensus:

TGCAAGGCTCCGATGATGATACCACTTCTAAAGTCGAGGTGTCACAATTTTCGGGTGTCGTAGAACTTGAATC
AGTAC

>WB235_Fam_202_78_1 Nr. of seq. 1 Alignment length(with gaps) = 78
Alignment score = 0.000000
WB235_5:18173031-18173742 Satlength=712 Nr of Repeats=10 RepeatLength=78
seed=GAGCTTTGGG Num.seqs=7 Similarity=0.947904
0
GAGCTTTGGGGCTTTTGGCTTGATCTCAGTCGACTTGATGGATTCTGGCTTGGGTTCTGCAGGCTTTTCGGGC
TCCTT

****
*****
Consensus:

GAGCTTTGGGGCTTTTGGCTTGATCTCAGTCGACTTGATGGATTCTGGCTTGGGTTCTGCAGGCTTTTCGGGC
TCCTT

>WB235_Fam_203_77_1 Nr. of seq. 1 Alignment length(with gaps) = 77
Alignment score = 0.000000
WB235_4:10540321-10540705 Satlength=385 Nr of Repeats=5 RepeatLength=77
seed=GGAAGCTTCA Num.seqs=4 Similarity=0.982684
0
GGAAGCTTCATCTTTTGAATTATAAAAAAATAAATTTCATTTCAAAATCAGTGTGTGCACTATCAAAAGTGTC
CACT

****
****
Consensus:

GGAAGCTTCATCTTTTGAATTATAAAAAAATAAATTTCATTTCAAAATCAGTGTGTGCACTATCAAAAGTGTC
CACT

>WB235_Fam_204_73_1 Nr. of seq. 1 Alignment length(with gaps) = 73
Alignment score = 0.000000
WB235_2:1319234-1319605 Satlength=372 Nr of Repeats=5 RepeatLength=73
seed=AGTTTGTAGT Num.seqs=3 Similarity=0.920852
0
ANTTTGTAGTTTGTAGTCTAGCAGACCAAAATTTATTAAAAACTGCAGAAAATTGATGGGTCTGCTAATTAAG

****
Consensus:

ANTTTGTAGTTTGTAGTCTAGCAGACCAAAATTTATTAAAAACTGCAGAAAATTGATGGGTCTGCTAATTAAG

>WB235_Fam_205_72_1 Nr. of seq. 1 Alignment length(with gaps) = 72
Alignment score = 0.000000
WB235_2:863584-866193 Satlength=2610 Nr of Repeats=35 RepeatLength=72
seed=TCCATCCAGC Num.seqs=29 Similarity=0.928845
0
TCCATCCAGCTGTGGTTGGTCTCTGGGTGAATCCTCGAGAAGTATCACTAGTAGTACCATTGGCTCCGTTCG

****
Consensus:

TCCATCCAGCTGTGGTTGGTCTCTGGGTGAATCCTCGAGAAGTATCACTAGTAGTACCATTGGCTCCGTTCG

>WB235_Fam_206_72_1 Nr. of seq. 1 Alignment length(with gaps) = 72
Alignment score = 0.000000
WB235_3:1608442-1609120 Satlength=679 Nr of Repeats=5 RepeatLength=72
seed=ATGAATTTTA Num.seqs=3 Similarity=0.975309
0
ATGAATTTTAATACAATTTTCCTGACGATGAAGTCATAAAAATTCATTTTTTGACCTTTCAACTTTAGTTTT

****
Consensus:

ATGAATTTTAATACAATTTTCCTGACGATGAAGTCATAAAAATTCATTTTTTGACCTTTCAACTTTAGTTTT

>WB235_Fam_207_72_1 Nr. of seq. 1 Alignment length(with gaps) = 72
Alignment score = 0.000000
WB235_6:15051665-15051975 Satlength=311 Nr of Repeats=5 RepeatLength=72
seed=TGGTTGTGTT Num.seqs=4 Similarity=0.990741
0
TGGTTGTGTTCCCAAAAGTGAGAAAAGAGGGTACAGACCTCTGTACGAATATGTCTTATCAGCTTCAAAAGA

****
Consensus:

TGGTTGTGTTCCCAAAAGTGAGAAAAGAGGGTACAGACCTCTGTACGAATATGTCTTATCAGCTTCAAAAGA

>WB235_Fam_208_71_1 Nr. of seq. 1 Alignment length(with gaps) = 71
Alignment score = 0.000000
WB235_1:1408704-1409384 Satlength=681 Nr of Repeats=8 RepeatLength=71
seed=ATTTATGTGA Num.seqs=6 Similarity=0.847840
0
ATTTATGTGAAAATTCAGGTTTTTAGGGCATTTTTTGAGGGAAATCTGAATTTTTCCGATTTTTCTGACGA

****
Consensus:

ATTTATGTGAAAATTCAGGTTTTTAGGGCATTTTTTGAGGGAAATCTGAATTTTTCCGATTTTTCTGACGA

>WB235_Fam_209_70_1 Nr. of seq. 1 Alignment length(with gaps) = 70
Alignment score = 0.000000

WB235_4:2393736-2396081 Satlength=2346 Nr of Repeats=23 RepeatLength=70
seed=AAAAATCGAT Num.seqs=14 Similarity=0.884668
0
AAAAATCGATTTTTTGAAGTTTTTCTCAATAGAGCGCATTTTCAACAGCGAAAAAATTGAATTTTCCGCC

****
Consensus:

AAAAATCGATTTTTTGAAGTTTTTCTCAATAGAGCGCATTTTCAACAGCGAAAAAATTGAATTTTCCGCC

>WB235_Fam_210_69_1 Nr. of seq. 1 Alignment length(with gaps) = 69
Alignment score = 0.000000
WB235_2:12336337-12336616 Satlength=280 Nr of Repeats=4 RepeatLength=69
seed=AGGTAGGCCT Num.seqs=3 Similarity=0.747619
0
AGGTAGGCCTGTAGGCATTGACTGNCAGAAAAGCGTAGACAGGCAAGCAGGTAAAAAGATACATGAGGC

****
Consensus:

AGGTAGGCCTGTAGGCATTGACTGNCAGAAAAGCGTAGACAGGCAAGCAGGTAAAAAGATACATGAGGC

>WB235_Fam_211_68_1 Nr. of seq. 1 Alignment length(with gaps) = 68
Alignment score = 0.000000
WB235_1:13883897-13885190 Satlength=1294 Nr of Repeats=19 RepeatLength=68
seed=TTGGTTGAGG Num.seqs=14 Similarity=0.987503
0   TTGGTTGAGGCTAGAAAATGTTGTAGTTTGTAGTGTGTTAGCCTCAACCAAAATTAATTTTTTTTTAA

****
Consensus:

TTGGTTGAGGCTAGAAAATGTTGTAGTTTGTAGTGTGTTAGCCTCAACCAAAATTAATTTTTTTTTAA

>WB235_Fam_212_68_1 Nr. of seq. 1 Alignment length(with gaps) = 68
Alignment score = 0.000000
WB235_2:6296691-6297095 Satlength=405 Nr of Repeats=6 RepeatLength=68
seed=AATAGAGACG Num.seqs=4 Similarity=0.901961
0   AATAGAGACGAATAAAAATTTAAAATTTTTATTAGTGTCTCTATTTTATAAAGAAAATACATTTTCAA

****
Consensus:

AATAGAGACGAATAAAAATTTAAAATTTTTATTAGTGTCTCTATTTTATAAAGAAAATACATTTTCAA

>WB235_Fam_213_65_1 Nr. of seq. 1 Alignment length(with gaps) = 65
Alignment score = 0.000000
WB235_2:1858665-1858980 Satlength=316 Nr of Repeats=5 RepeatLength=65
seed=TATCGAGAGA Num.seqs=3 Similarity=0.658497
0   TATCGAGAGAATNTTCCACATTCACTTTGAATTCCNTTTCCAATTTCAATAAGACTCTCAGTAAG

****
Consensus:

TATCGAGAGAATNTTCCACATTCACTTTGAATTCCNTTTCCAATTTCAATAAGACTCTCAGTAAG

>WB235_Fam_214_62_1 Nr. of seq. 1 Alignment length(with gaps) = 62
Alignment score = 0.000000
WB235_1:14101896-14102785 Satlength=890 Nr of Repeats=4 RepeatLength=62
seed=GTAGTTTGTA Num.seqs=3 Similarity=0.928315
0   GTAGTTTGTAGCTTTAGCCTCAACCAAATTAGCTAAAGCTTGAGTGAGGCTATGGTGATTTT

****
Consensus:

GTAGTTTGTAGCTTTAGCCTCAACCAAATTAGCTAAAGCTTGAGTGAGGCTATGGTGATTTT

>WB235_Fam_215_62_1 Nr. of seq. 1 Alignment length(with gaps) = 62
Alignment score = 0.000000
WB235_2:13967824-13968381 Satlength=558 Nr of Repeats=7 RepeatLength=62
seed=TGCTAACTAG Num.seqs=6 Similarity=0.822222
0   TGCTAACTAGCTACGGCCAGCTTAGGTGCCGGCTGCACGAGCAGTCCTGGTTTAATGTCGGC

****
Consensus:

TGCTAACTAGCTACGGCCAGCTTAGGTGCCGGCTGCACGAGCAGTCCTGGTTTAATGTCGGC

>WB235_Fam_216_60_1 Nr. of seq. 1 Alignment length(with gaps) = 60
Alignment score = 0.000000
WB235_5:7259575-7259935 Satlength=361 Nr of Repeats=6 RepeatLength=60
seed=CTAGCTAGAT Num.seqs=6 Similarity=0.943704
0   CTAGCTAGATTAAACGCTCTCAAGAGACAGCATGTTGTTGTTGCCCACAAGAAGAAGGCT

****
Consensus:

CTAGCTAGATTAAACGCTCTCAAGAGACAGCATGTTGTTGTTGCCCACAAGAAGAAGGCT

>WB235_Fam_217_60_1 Nr. of seq. 1 Alignment length(with gaps) = 60
Alignment score = 0.000000
WB235_5:8699367-8702913 Satlength=3547 Nr of Repeats=59 RepeatLength=60
seed=ATTCAGTTGA Num.seqs=58 Similarity=0.926281
0   ATTCAGTTGAGCTCGTTATGTCGGTTGAAGACACAATTGGAGTGGTGCTTGAGGAGCTGG

****
Consensus:

ATTCAGTTGAGCTCGTTATGTCGGTTGAAGACACAATTGGAGTGGTGCTTGAGGAGCTGG

>WB235_Fam_218_60_1 Nr. of seq. 1 Alignment length(with gaps) = 60
Alignment score = 0.000000
WB235_5:14107921-14109961 Satlength=2041 Nr of Repeats=34 RepeatLength=60
seed=CTGCTGATTC Num.seqs=34 Similarity=0.924896
0   CTGCTGATTCCGAGAAGAAGGCCCCAGCCAAGAGAGAAGCTGATAAGCCAAAGACTGAAT

****
Consensus:

CTGCTGATTCCGAGAAGAAGGCCCCAGCCAAGAGAGAAGCTGATAAGCCAAAGACTGAAT

>WB235_Fam_219_59_1 Nr. of seq. 1 Alignment length(with gaps) = 59
Alignment score = 0.000000
WB235_2:1408410-1408750 Satlength=341 Nr of Repeats=5 RepeatLength=59
seed=CTACAAACTA Num.seqs=3 Similarity=0.969868
0   CTACAAACTACAAAAGCCTATTTAGCAGACCCACGCCAACTTCTGATGGGTCTGCTAGA

****
Consensus:

CTACAAACTACAAAAGCCTATTTAGCAGACCCACGCCAACTTCTGATGGGTCTGCTAGA

>WB235_Fam_220_57_1 Nr. of seq. 1 Alignment length(with gaps) = 57
Alignment score = 0.000000
WB235_5:14104614-14106168 Satlength=1555 Nr of Repeats=20 RepeatLength=57
seed=GAACTCAACA Num.seqs=13 Similarity=0.959214
0   GAACTCAACAAGCTGATCAAGAGCAACACCGTGGATCCCGAGCTGCCGGACAGAAGG

****
Consensus:

GAACTCAACAAGCTGATCAAGAGCAACACCGTGGATCCCGAGCTGCCGGACAGAAGG

>WB235_Fam_221_55_1 Nr. of seq. 1 Alignment length(with gaps) = 55
Alignment score = 0.000000
WB235_2:1204743-1204963 Satlength=221 Nr of Repeats=4 RepeatLength=55
seed=GGGATTATTG Num.seqs=4 Similarity=0.903030
0   GGGATTATTGGAGTTTCTTTTTCTAGAATTTTTTGAAAATTTTGGACAGTTTTTT

****
Consensus:

GGGATTATTGGAGTTTCTTTTTCTAGAATTTTTTGAAAATTTTGGACAGTTTTTT

>WB235_Fam_222_55_1 Nr. of seq. 1 Alignment length(with gaps) = 55
Alignment score = 0.000000
WB235_4:6009084-6009409 Satlength=326 Nr of Repeats=6 RepeatLength=55
seed=AAGAGTCCCC Num.seqs=4 Similarity=1.000000
0   AAGAGTCCCCACACACTGAAAGGTTTTAAGGAATTTTTTGAGGATTCCTAGGGTT

****
Consensus:

AAGAGTCCCCACACACTGAAAGGTTTTAAGGAATTTTTTGAGGATTCCTAGGGTT

>WB235_Fam_223_52_1 Nr. of seq. 1 Alignment length(with gaps) = 52
Alignment score = 0.000000
WB235_6:16837630-16838124 Satlength=495 Nr of Repeats=4 RepeatLength=52
seed=GCATGTCTTC Num.seqs=3 Similarity=0.626263
0   GCATGTCTTCCAAGCGTTTTGAGCCACGCNTGGGATTTCCTATACACGGCTG

****

Consensus:

GCATGTCTTCCAAGCGTTTTGAGCCACGCNTGGGATTTCCTATACACGGCTG

>WB235_Fam_224_51_1 Nr. of seq. 1 Alignment length(with gaps) = 51
Alignment score = 0.000000
WB235_4:7727050-7727263 Satlength=214 Nr of Repeats=5 RepeatLength=51
seed=CCATCCGAAG Num.seqs=3 Similarity=0.912854
0   CCATCCGAAGATCCTCAACCATCTGGCCCACCATCTCCGGGACCAGTGGAC

****
Consensus:

CCATCCGAAGATCCTCAACCATCTGGCCCACCATCTCCGGGACCAGTGGAC

>WB235_Fam_225_50_1 Nr. of seq. 1 Alignment length(with gaps) = 50
Alignment score = 0.000000
WB235_1:12480947-12481197 Satlength=251 Nr of Repeats=5 RepeatLength=50
seed=ACACATGCTA Num.seqs=5 Similarity=0.952000
0   ACACATGCTATAACATTGTCTCCTTCCCCTAGTGCACGAGAGCGGCCGTT

****
Consensus:

ACACATGCTATAACATTGTCTCCTTCCCCTAGTGCACGAGAGCGGCCGTT

>WB235_Fam_226_50_1 Nr. of seq. 1 Alignment length(with gaps) = 50
Alignment score = 0.000000
WB235_4:16427870-16428521 Satlength=652 Nr of Repeats=13 RepeatLength=50
seed=GGGCGTGTTT Num.seqs=10 Similarity=0.897482
0   GGGCGTGTTTTCACATGCCCCGCCCACAAAATTGATGTTTCCACCAAAAT

****
Consensus:

GGGCGTGTTTTCACATGCCCCGCCCACAAAATTGATGTTTCCACCAAAAT

>WB235_Fam_227_49_1 Nr. of seq. 1 Alignment length(with gaps) = 49
Alignment score = 0.000000
WB235_1:1620047-1620586 Satlength=540 Nr of Repeats=9 RepeatLength=49
seed=CAATAATTTT Num.seqs=7 Similarity=0.909297
0   CAATAATTTTAAATTAAATTATCGATTTTTCTAAATTTTTCCGAAAAAT

****
Consensus:

CAATAATTTTAAATTAAATTATCGATTTTTCTAAATTTTTCCGAAAAAT

>WB235_Fam_228_49_1 Nr. of seq. 1 Alignment length(with gaps) = 49
Alignment score = 0.000000
WB235_5:14766633-14767947 Satlength=1315 Nr of Repeats=28 RepeatLength=49
seed=GCTACGATAT Num.seqs=20 Similarity=0.947870
0   GCTACGATATCGTAGCACTTTTTTATTTTTCCATATTCCACCAAAAAAC

****
Consensus:

GCTACGATATCGTAGCACTTTTTTATTTTTCCATATTCCACCAAAAAAC

>WB235_Fam_229_48_1 Nr. of seq. 1 Alignment length(with gaps) = 48
Alignment score = 0.000000
WB235_2:13284530-13285104 Satlength=575 Nr of Repeats=12 RepeatLength=48
seed=TGCCTACGTG Num.seqs=10 Similarity=0.812346
0   TGCCTACGTGCCTACAAAACAGGCGTAGGCAGCTTTAAAGGTGATGCA

****
Consensus:

TGCCTACGTGCCTACAAAACAGGCGTAGGCAGCTTTAAAGGTGATGCA

>WB235_Fam_230_48_1 Nr. of seq. 1 Alignment length(with gaps) = 48
Alignment score = 0.000000
WB235_6:14262861-14263101 Satlength=241 Nr of Repeats=5 RepeatLength=48
seed=GGAGGTGGAG Num.seqs=5 Similarity=0.841667
0   GGAGGTGGAGAAAGTGTAGTTCCCGGTGGTCGNGGCTCTTCNGAGGGT

****
Consensus:

GGAGGTGGAGAAAGTGTAGTTCCCGGTGGTCGNGGCTCTTCNGAGGGT

>WB235_Fam_231_46_1 Nr. of seq. 1 Alignment length(with gaps) = 46
Alignment score = 0.000000
WB235_6:1980629-1980811 Satlength=183 Nr of Repeats=4 RepeatLength=46
seed=TGGTAAATTG Num.seqs=3 Similarity=0.835749
0   TGGTAAATTGCCTCCCGGANCACCCACTTTTCTTATTTCCCAACTA

****
Consensus:

TGGTAAATTGCCTCCCGGANCACCCACTTTTCTTATTTCCCAACTA

>WB235_Fam_232_45_1 Nr. of seq. 1 Alignment length(with gaps) = 45
Alignment score = 0.000000
WB235_4:7734990-7736070 Satlength=1081 Nr of Repeats=24 RepeatLength=45
seed=ACCATCAGGC Num.seqs=24 Similarity=0.963714
0   ACCATCAGGCGGACCACCAGGACCATTTGATCCATCAGGAGCACC

****
Consensus:

ACCATCAGGCGGACCACCAGGACCATTTGATCCATCAGGAGCACC

>WB235_Fam_233_45_1 Nr. of seq. 1 Alignment length(with gaps) = 45
Alignment score = 0.000000

WB235_5:11273771-11274491 Satlength=721 Nr of Repeats=16 RepeatLength=45
seed=ACAACTGAAT Num.seqs=16 Similarity=0.992593
0   ACAACTGAATCAACTGCAGCACCAAGCACATCTACAGAGACACCT

****
Consensus:

ACAACTGAATCAACTGCAGCACCAAGCACATCTACAGAGACACCT

>WB235_Fam_234_45_1 Nr. of seq. 1 Alignment length(with gaps) = 45
Alignment score = 0.000000
WB235_6:17441768-17447783 Satlength=6016 Nr of Repeats=134
RepeatLength=45 seed=CCACCAGCAC Num.seqs=122 Similarity=0.923840
0   CCACCAGCACTGCTTCGAGTACGACAGAAAGCACTTCGACTCCAT

****
Consensus:

CCACCAGCACTGCTTCGAGTACGACAGAAAGCACTTCGACTCCAT

>WB235_Fam_235_43_1 Nr. of seq. 1 Alignment length(with gaps) = 43
Alignment score = 0.000000
WB235_2:2171107-2172182 Satlength=1076 Nr of Repeats=24 RepeatLength=43
seed=CGAGTTTGGC Num.seqs=23 Similarity=0.818978
0   CGAGTTTGGCCTACANCAAATTTGGCAAATTCGGCAAATTTGC

****
Consensus:

CGAGTTTGGCCTACANCAAATTTGGCAAATTCGGCAAATTTGC

>WB235_Fam_236_43_1 Nr. of seq. 1 Alignment length(with gaps) = 43
Alignment score = 0.000000
WB235_2:14130915-14131087 Satlength=173 Nr of Repeats=4 RepeatLength=43
seed=GAAAACTAGG Num.seqs=4 Similarity=0.715762
0   GAAAACTAGGCCACAGAGTTCAAATTGATGGCCTAGAAATCTA

****
Consensus:

GAAAACTAGGCCACAGAGTTCAAATTGATGGCCTAGAAATCTA

>WB235_Fam_237_43_1 Nr. of seq. 1 Alignment length(with gaps) = 43
Alignment score = 0.000000
WB235_3:1385873-1386532 Satlength=660 Nr of Repeats=5 RepeatLength=43
seed=GGCTCTCGGC Num.seqs=3 Similarity=0.627907
0   GGCTCTCGGCTCGCGCCGAGAGCCGAGNTCTACTNAAAGTTTC

****
Consensus:

GGCTCTCGGCTCGCGCCGAGAGCCGAGNTCTACTNAAAGTTTC

>WB235_Fam_238_43_1 Nr. of seq. 1 Alignment length(with gaps) = 43
Alignment score = 0.000000
WB235_3:12305325-12305929 Satlength=605 Nr of Repeats=14 RepeatLength=43
seed=AGCAGCCGAC Num.seqs=11 Similarity=0.800423
0   AGCAGCCGACAGTTGCCGGTTGCCGAAAAATTGCAGTAATTTC

****
Consensus:

AGCAGCCGACAGTTGCCGGTTGCCGAAAAATTGCAGTAATTTC

>WB235_Fam_239_42_1 Nr. of seq. 1 Alignment length(with gaps) = 42
Alignment score = 0.000000
WB235_1:724780-724948 Satlength=169 Nr of Repeats=4 RepeatLength=42
seed=TTTGATCTAC Num.seqs=4 Similarity=0.968254
0   TTTGATCTACAAAAAATGCGGGAATTTTCCCAGCATTGCTCA

****
Consensus:

TTTGATCTACAAAAAATGCGGGAATTTTCCCAGCATTGCTCA

>WB235_Fam_240_42_1 Nr. of seq. 1 Alignment length(with gaps) = 42
Alignment score = 0.000000
WB235_5:6188375-6189068 Satlength=694 Nr of Repeats=15 RepeatLength=42
seed=AGGTGCTGCT Num.seqs=9 Similarity=0.859788
0   AGGTGCTGCTTCCTTTGGAACATCTGCTTGGGTTGGCTCCGA

****
Consensus:

AGGTGCTGCTTCCTTTGGAACATCTGCTTGGGTTGGCTCCGA

>WB235_Fam_241_41_1 Nr. of seq. 1 Alignment length(with gaps) = 41
Alignment score = 0.000000
WB235_4:3696437-3696683 Satlength=247 Nr of Repeats=6 RepeatLength=41
seed=AAATTTTTAA Num.seqs=6 Similarity=0.947967
0   AAATTTTTAACGAGAAGTGGGCGAAAATCATTCAAAAATTC

****
Consensus:

AAATTTTTAACGAGAAGTGGGCGAAAATCATTCAAAAATTC

>WB235_Fam_242_41_1 Nr. of seq. 1 Alignment length(with gaps) = 41
Alignment score = 0.000000
WB235_5:20236849-20237152 Satlength=304 Nr of Repeats=5 RepeatLength=41
seed=GCATTTTTCG Num.seqs=3 Similarity=0.772358
0   GCATTTTTCGCCGATTTTTNATTAATTTTCAGTGAAAATTC

****
Consensus:

GCATTTTTCGCCGATTTTTNATTAATTTTCAGTGAAAATTC

>WB235_Fam_243_40_1 Nr. of seq. 1 Alignment length(with gaps) = 40
Alignment score = 0.000000
WB235_3:404417-405141 Satlength=725 Nr of Repeats=7 RepeatLength=40
seed=TCCACGTGGA Num.seqs=5 Similarity=0.650794
0   TCCACGTGGAATTTTCAGAATTTTCNCAGAAAATTTGAAA

****
Consensus:

TCCACGTGGAATTTTCAGAATTTTCNCAGAAAATTTGAAA

>WB235_Fam_244_40_1 Nr. of seq. 1 Alignment length(with gaps) = 40
Alignment score = 0.000000
WB235_5:18865200-18865555 Satlength=356 Nr of Repeats=7 RepeatLength=40
seed=GAATTTTTTG Num.seqs=6 Similarity=0.926667
0   GAATTTTTTGCAATTCTACTGAATTTTCCAAATTTTCAGC

****
Consensus:

GAATTTTTTGCAATTCTACTGAATTTTCCAAATTTTCAGC

>WB235_Fam_245_40_1 Nr. of seq. 1 Alignment length(with gaps) = 40
Alignment score = 0.000000
WB235_5:18879684-18879863 Satlength=180 Nr of Repeats=5 RepeatLength=40
seed=TTTTGCCGAA Num.seqs=3 Similarity=0.629630
0   TTTTGCCAAAAATNTGTCAATTTTGTGGAATTTCTNTCAT

****
Consensus:

TTTTGCCAAAAATNTGTCAATTTTGTGGAATTTCTNTCAT

>WB235_Fam_246_39_1 Nr. of seq. 1 Alignment length(with gaps) = 39
Alignment score = 0.000000
WB235_2:3494517-3494748 Satlength=232 Nr of Repeats=5 RepeatLength=39
seed=AATTTCCCGC Num.seqs=3 Similarity=0.769444
0   AATTTCCCGCCAAAATTGAATTTTTTTAAAGAAAATTTA

****
Consensus:

AATTTCCCGCCAAAATTGAATTTTTTTAAAGAAAATTTA

>WB235_Fam_247_39_1 Nr. of seq. 1 Alignment length(with gaps) = 39
Alignment score = 0.000000
WB235_4:4996655-4997045 Satlength=391 Nr of Repeats=8 RepeatLength=39
seed=CCAACTGGAG Num.seqs=7 Similarity=0.784921
0   CCAACTGGAGCCAGCACNATGACCGCTGTTGGAGGAGCA

****
Consensus:

CCAACTGGAGCCAGCACNATGACCGCTGTTGGAGGAGCA

>WB235_Fam_248_39_1 Nr. of seq. 1 Alignment length(with gaps) = 39
Alignment score = 0.000000
WB235_5:3694442-3694754 Satlength=313 Nr of Repeats=8 RepeatLength=39
seed=GATTTACGGG Num.seqs=6 Similarity=0.849573
0   GATTTACGGGCTTTAATCAGTTTCGAAAAAGCTTGTGTC

****
Consensus:

GATTTACGGGCTTTAATCAGTTTCGAAAAAGCTTGTGTC

>WB235_Fam_249_39_1 Nr. of seq. 1 Alignment length(with gaps) = 39
Alignment score = 0.000000
WB235_5:12633007-12633394 Satlength=388 Nr of Repeats=10 RepeatLength=39
seed=TGGTACGGTA Num.seqs=9 Similarity=0.880342
0   TGGTACGGTAGTTGTAGTGGTAGTGGAGGTTGTTGTTGG

****
Consensus:

TGGTACGGTAGTTGTAGTGGTAGTGGAGGTTGTTGTTGG

>WB235_Fam_250_39_1 Nr. of seq. 1 Alignment length(with gaps) = 39
Alignment score = 0.000000
WB235_5:18636764-18637321 Satlength=558 Nr of Repeats=15 RepeatLength=39
seed=GTAATTTATA Num.seqs=13 Similarity=0.917598
0   GTAATTTATAATTTTTTAAAGATGAGTAATATATTGCCC

****
Consensus:

GTAATTTATAATTTTTTAAAGATGAGTAATATATTGCCC

>WB235_Fam_251_39_1 Nr. of seq. 1 Alignment length(with gaps) = 39
Alignment score = 0.000000
WB235_6:17179152-17179971 Satlength=820 Nr of Repeats=21 RepeatLength=39
seed=ATTGATACTT Num.seqs=21 Similarity=0.932601
0   ATTGATACTTTGTCGGGTTTAGACCATCGGCCGAGCTTC

****
Consensus:

ATTGATACTTTGTCGGGTTTAGACCATCGGCCGAGCTTC

>WB235_Fam_252_38_1 Nr. of seq. 1 Alignment length(with gaps) = 38
Alignment score = 0.000000
WB235_1:1987299-1987635 Satlength=337 Nr of Repeats=5 RepeatLength=38
seed=AAAATCGATA Num.seqs=3 Similarity=1.000000
0   AAAATCGATAATTTTCAGTATGCAGTGAAATTCTTGGA

****

Consensus:

AAAATCGATAATTTTCAGTATGCAGTGAAATTCTTGGA

>WB235_Fam_253_37_1 Nr. of seq. 1 Alignment length(with gaps) = 37
Alignment score = 0.000000
WB235_1:9925221-9925359 Satlength=139 Nr of Repeats=5 RepeatLength=37
seed=ATAATTTTTC Num.seqs=3 Similarity=1.000000
0   ATAATTTTTCTTGAGTAATGAGAGACTTTTTCATAAT

****
Consensus:

ATAATTTTTCTTGAGTAATGAGAGACTTTTTCATAAT

>WB235_Fam_254_36_1 Nr. of seq. 1 Alignment length(with gaps) = 36
Alignment score = 0.000000
WB235_3:12513645-12515229 Satlength=1585 Nr of Repeats=44 RepeatLength=36
seed=GATATTTTCA Num.seqs=44 Similarity=0.951257
0   GATATTTTCATGTTCGGCAAAAGTACCGATTTGCCA

****
Consensus:

GATATTTTCATGTTCGGCAAAAGTACCGATTTGCCA

>WB235_Fam_255_36_1 Nr. of seq. 1 Alignment length(with gaps) = 36
Alignment score = 0.000000
WB235_6:17674798-17675174 Satlength=377 Nr of Repeats=7 RepeatLength=36
seed=CCACCAACTC Num.seqs=6 Similarity=0.728395
0   CCACCAACTCCAAAATCAGCTGCTCCATCAGAAGTC

****
Consensus:

CCACCAACTCCAAAATCAGCTGCTCCATCAGAAGTC

>WB235_Fam_256_35_1 Nr. of seq. 1 Alignment length(with gaps) = 35
Alignment score = 0.000000
WB235_1:11201002-11201638 Satlength=637 Nr of Repeats=9 RepeatLength=35
seed=AATTGCCGAA Num.seqs=7 Similarity=0.793210
0   AATTGCCGAATTTCTCGTTTTTGGCAATTTACCAA

****
Consensus:

AATTGCCGAATTTCTCGTTTTTGGCAATTTACCAA

>WB235_Fam_257_35_1 Nr. of seq. 1 Alignment length(with gaps) = 35
Alignment score = 0.000000
WB235_2:12795532-12795808 Satlength=277 Nr of Repeats=8 RepeatLength=35
seed=TGTGTAATAA Num.seqs=5 Similarity=0.794286
0   TGTGTAATAAGAAAATTTCAAATTTTTTTCACGAA

****
Consensus:

TGTGTAATAAGAAAATTTCAAATTTTTTTCACGAA

>WB235_Fam_258_35_1 Nr. of seq. 1 Alignment length(with gaps) = 35
Alignment score = 0.000000
WB235_5:1015554-1016070 Satlength=517 Nr of Repeats=9 RepeatLength=35
seed=GCATTTGCCG Num.seqs=6 Similarity=0.817143
0   GCATTTGCCGATTTTTAAAATTTCTGGCAATTCCA

****
Consensus:

GCATTTGCCGATTTTTAAAATTTCTGGCAATTCCA

>WB235_Fam_259_34_1 Nr. of seq. 1 Alignment length(with gaps) = 34
Alignment score = 0.000000
WB235_2:14100395-14100701 Satlength=307 Nr of Repeats=8 RepeatLength=34
seed=CCGTGCCTAC Num.seqs=7 Similarity=0.828198
0   CCGTGCCTACTAACCTACCGACCTTGCCTGCCTT

****
Consensus:

CCGTGCCTACTAACCTACCGACCTTGCCTGCCTT

>WB235_Fam_260_34_1 Nr. of seq. 1 Alignment length(with gaps) = 34
Alignment score = 0.000000
WB235_3:11798467-11798684 Satlength=218 Nr of Repeats=6 RepeatLength=34
seed=TTGCCGAATT Num.seqs=5 Similarity=0.811765
0   TTGCCGAATTTGCCGTTTGCCGAGCTCGTCAAAT

****
Consensus:

TTGCCGAATTTGCCGTTTGCCGAGCTCGTCAAAT

>WB235_Fam_261_34_1 Nr. of seq. 1 Alignment length(with gaps) = 34
Alignment score = 0.000000
WB235_3:11864487-11864917 Satlength=431 Nr of Repeats=13 RepeatLength=34
seed=AAGTATTTTT Num.seqs=12 Similarity=0.954843
0   AAGTATTTTTGCCGGTTTTTTTGCCGAAATTTCC

****
Consensus:

AAGTATTTTTGCCGGTTTTTTTGCCGAAATTTCC

>WB235_Fam_262_33_1 Nr. of seq. 1 Alignment length(with gaps) = 33
Alignment score = 0.000000

WB235_1:2251015-2251148 Satlength=134 Nr of Repeats=4 RepeatLength=33
seed=TAAGAAAAGT Num.seqs=3 Similarity=0.892256
0 TAAGAAAAGTTAGGCCACCAAACTTTGGTGAAC

****
Consensus:

TAAGAAAAGTTAGGCCACCAAACTTTGGTGAAC

>WB235_Fam_263_32_1 Nr. of seq. 1 Alignment length(with gaps) = 32
Alignment score = 0.000000
WB235_1:8484982-8485111 Satlength=130 Nr of Repeats=4 RepeatLength=32
seed=AAAATCGAAA Num.seqs=3 Similarity=1.000000
0 AAAATCGAAAAATTCTCGTTTTTCAATTTTTA

****
Consensus:

AAAATCGAAAAATTCTCGTTTTTCAATTTTTA

>WB235_Fam_264_32_1 Nr. of seq. 1 Alignment length(with gaps) = 32
Alignment score = 0.000000
WB235_1:11313617-11315164 Satlength=1548 Nr of Repeats=48 RepeatLength=32
seed=GCAATTTTTA Num.seqs=37 Similarity=0.890140
0 GCAATTTTTAGGCTAAAAATCACTAAAAAACT

****
Consensus:

GCAATTTTTAGGCTAAAAATCACTAAAAAACT

>WB235_Fam_265_32_1 Nr. of seq. 1 Alignment length(with gaps) = 32
Alignment score = 0.000000
WB235_3:12072321-12073804 Satlength=1484 Nr of Repeats=45 RepeatLength=32
seed=AAATTTGTGA Num.seqs=34 Similarity=0.906566
0 AAATTTGTGAAAATTTAGGCCCATTTTGCTCA

****
Consensus:

AAATTTGTGAAAATTTAGGCCCATTTTGCTCA

>WB235_Fam_266_32_1 Nr. of seq. 1 Alignment length(with gaps) = 32
Alignment score = 0.000000
WB235_5:20705245-20705371 Satlength=127 Nr of Repeats=4 RepeatLength=32
seed=GTGCGTCCGG Num.seqs=3 Similarity=0.888889
0 GTGCGTCCGGCGTCCGGAAAAAGCGGACGCGT

****
Consensus:

GTGCGTCCGGCGTCCGGAAAAAGCGGACGCGT

```
>WB235_Fam_267_31_1  Nr. of seq. 1 Alignment length(with gaps) = 31
Alignment score = 0.000000
WB235_2:3070377-3070511 Satlength=135 Nr of Repeats=4 RepeatLength=31
seed=ATCGATTTTT  Num.seqs=3 Similarity=0.700337
0    ATCGATTTTTTCGTTAAAAAATCAATAAAAT

*******************************
Consensus:

ATCGATTTTTTCGTTAAAAAATCAATAAAAT

>WB235_Fam_268_31_1  Nr. of seq. 1 Alignment length(with gaps) = 31
Alignment score = 0.000000
WB235_2:3691112-3691360 Satlength=249 Nr of Repeats=8 RepeatLength=31
seed=GCTGATTGGT  Num.seqs=8 Similarity=0.553571
0    GCTGATTGGTCGGCGNGGTTCGCCCATCNTT

*******************************
Consensus:

GCTGATTGGTCGGCGNGGTTCGCCCATCNTT

>WB235_Fam_269_31_1  Nr. of seq. 1 Alignment length(with gaps) = 31
Alignment score = 0.000000
WB235_2:12455807-12455995 Satlength=189 Nr of Repeats=6 RepeatLength=31
seed=GACATCTCGC  Num.seqs=4 Similarity=0.584229
0    GACATCTCGCAGGACATTATAAGAAGCGACC

*******************************
Consensus:

GACATCTCGCAGGACATTATAAGAAGCGACC

>WB235_Fam_270_31_1  Nr. of seq. 1 Alignment length(with gaps) = 31
Alignment score = 0.000000
WB235_2:13281689-13282092 Satlength=404 Nr of Repeats=10 RepeatLength=31
seed=CACGCCTATT  Num.seqs=7 Similarity=0.811572
0    CACGCCTATTTTATGGGCGGAGTCTACAAGC

*******************************
Consensus:

CACGCCTATTTTATGGGCGGAGTCTACAAGC

>WB235_Fam_271_31_1  Nr. of seq. 1 Alignment length(with gaps) = 31
Alignment score = 0.000000
WB235_3:2009666-2010596 Satlength=931 Nr of Repeats=27 RepeatLength=31
seed=TGCGTCGGAA  Num.seqs=23 Similarity=0.733775
0    TGCGTCGGAAATTNATTCCGATCATCGCCGG

*******************************
Consensus:

TGCGTCGGAAATTNATTCCGATCATCGCCGG
```

>WB235_Fam_272_31_1 Nr. of seq. 1 Alignment length(with gaps) = 31
Alignment score = 0.000000
WB235_3:2011573-2012317 Satlength=745 Nr of Repeats=17 RepeatLength=31
seed=TCCGATGCAT Num.seqs=13 Similarity=0.625486
0   TCCGATGCATTGCCGATAATCGGAACCTGGT

****
Consensus:

TCCGATGCATTGCCGATAATCGGAACCTGGT

>WB235_Fam_273_31_1 Nr. of seq. 1 Alignment length(with gaps) = 31
Alignment score = 0.000000
WB235_3:11002899-11003053 Satlength=155 Nr of Repeats=5 RepeatLength=31
seed=GCAGCCGACG Num.seqs=4 Similarity=0.524411
0   GCAGCCGACGCTATACGGGTCTGATGGAGTA

****
Consensus:

GCAGCCGACGCTATACGGGTCTGATGGAGTA

>WB235_Fam_274_31_1 Nr. of seq. 1 Alignment length(with gaps) = 31
Alignment score = 0.000000
WB235_3:11189285-11189691 Satlength=407 Nr of Repeats=12 RepeatLength=31
seed=TCTGAGCTAC Num.seqs=11 Similarity=0.755258
0   TCTGAGCTACAGTACTCCTGTCAAGAACAAT

****
Consensus:

TCTGAGCTACAGTACTCCTGTCAAGAACAAT

>WB235_Fam_275_31_1 Nr. of seq. 1 Alignment length(with gaps) = 31
Alignment score = 0.000000
WB235_3:12817385-12817569 Satlength=185 Nr of Repeats=5 RepeatLength=31
seed=GATTTTTTCA Num.seqs=3 Similarity=0.659722
0   GATTTTTTCAGNGAAAANCNAAAAATTTTCG

****
Consensus:

GATTTTTTCAGNGAAAANCNAAAAATTTTCG

>WB235_Fam_276_31_1 Nr. of seq. 1 Alignment length(with gaps) = 31
Alignment score = 0.000000
WB235_3:13167617-13167762 Satlength=146 Nr of Repeats=5 RepeatLength=31
seed=CCAAAAATGA Num.seqs=4 Similarity=0.863799
0   CCAAAAATGACTGAAAATAGTCAAAAATTAG

****
Consensus:

CCAAAAATGACTGAAAATAGTCAAAAATTAG

>WB235_Fam_277_31_1 Nr. of seq. 1 Alignment length(with gaps) = 31
Alignment score = 0.000000
WB235_4:775288-775412 Satlength=125 Nr of Repeats=4 RepeatLength=31
seed=CAAAAATCGG Num.seqs=4 Similarity=0.949821
0   CAAAAATCGGGAAATTTTCACAAAAAAAATGC

****
Consensus:

CAAAAATCGGGAAATTTTCACAAAAAAAATGC

>WB235_Fam_278_31_1 Nr. of seq. 1 Alignment length(with gaps) = 31
Alignment score = 0.000000
WB235_5:18415750-18416287 Satlength=538 Nr of Repeats=16 RepeatLength=31
seed=TTGGCCCAGA Num.seqs=11 Similarity=0.885826
0   TTGGCCCAGAGCTCTCTTGACCCAAGCCCCC

****
Consensus:

TTGGCCCAGAGCTCTCTTGACCCAAGCCCCC

>WB235_Fam_279_31_1 Nr. of seq. 1 Alignment length(with gaps) = 31
Alignment score = 0.000000
WB235_5:18536998-18537693 Satlength=696 Nr of Repeats=5 RepeatLength=31
seed=AAAATTTTGA Num.seqs=3 Similarity=0.971326
0   AAAATTTTGACCCAGAAATCGAAAAAATTCGC

****
Consensus:

AAAATTTTGACCCAGAAATCGAAAAAATTCGC

>WB235_Fam_280_31_1 Nr. of seq. 1 Alignment length(with gaps) = 31
Alignment score = 0.000000
WB235_5:18855835-18856039 Satlength=205 Nr of Repeats=6 RepeatLength=31
seed=TCGTTTTTTC Num.seqs=5 Similarity=0.819355
0   TCGTTTTTTCATGAAAAAACTCAATTTTCGG

****
Consensus:

TCGTTTTTTCATGAAAAAACTCAATTTTCGG

>WB235_Fam_281_31_1 Nr. of seq. 1 Alignment length(with gaps) = 31
Alignment score = 0.000000
WB235_5:20407384-20407602 Satlength=219 Nr of Repeats=4 RepeatLength=31
seed=GAAAAAGTGC Num.seqs=3 Similarity=0.784722
0   GAAAAAGTGCTGAAAATCACATTTTTGAACT

****

Consensus:

GAAAAAGTGCTGAAAATCACATTTTTGAACT

>WB235_Fam_282_30_1 Nr. of seq. 1 Alignment length(with gaps) = 30
Alignment score = 0.000000
WB235_1:1704545-1705424 Satlength=880 Nr of Repeats=24 RepeatLength=30
seed=AATTTGAATT Num.seqs=15 Similarity=0.732487
0   AATTTGAATTTTCCCGAATTTTTNAACGAA

****
Consensus:

AATTTGAATTTTCCCGAATTTTTNAACGAA

>WB235_Fam_283_30_1 Nr. of seq. 1 Alignment length(with gaps) = 30
Alignment score = 0.000000
WB235_1:6514283-6515118 Satlength=836 Nr of Repeats=13 RepeatLength=30
seed=TGGTGGAACT Num.seqs=11 Similarity=0.953131
0   TGGTGGAACTGGATTCGGCAGTGGAAATGC

****
Consensus:

TGGTGGAACTGGATTCGGCAGTGGAAATGC

>WB235_Fam_284_30_1 Nr. of seq. 1 Alignment length(with gaps) = 30
Alignment score = 0.000000
WB235_1:11404108-11404462 Satlength=355 Nr of Repeats=10 RepeatLength=30
seed=CCTTGGGCTC Num.seqs=7 Similarity=0.875132
0   CCTTGGGCTCCCTGAGCTCCAAATTGTGCT

****
Consensus:

CCTTGGGCTCCCTGAGCTCCAAATTGTGCT

>WB235_Fam_285_30_1 Nr. of seq. 1 Alignment length(with gaps) = 30
Alignment score = 0.000000
WB235_1:12109993-12110203 Satlength=211 Nr of Repeats=7 RepeatLength=30
seed=GTCATTATCA Num.seqs=7 Similarity=0.974603
0   GTCATTATCAGCTTCTGGTGTCTTAGAAGA

****
Consensus:

GTCATTATCAGCTTCTGGTGTCTTAGAAGA

>WB235_Fam_286_30_1 Nr. of seq. 1 Alignment length(with gaps) = 30
Alignment score = 0.000000
WB235_2:12171287-12172252 Satlength=966 Nr of Repeats=32 RepeatLength=30
seed=CAAACTACAG Num.seqs=27 Similarity=0.878569
0   CAAACTACAGTAGTACTGTAGTACCATAAC

****
Consensus:

CAAACTACAGTAGTACTGTAGTACCATAAC

>WB235_Fam_287_30_1 Nr. of seq. 1 Alignment length(with gaps) = 30
Alignment score = 0.000000
WB235_2:12979640-12979790 Satlength=151 Nr of Repeats=4 RepeatLength=30
seed=TTCAGGCAGG Num.seqs=3 Similarity=0.644444
0   TTCAGGCAGGCATAGGTTACTTTGAAAATT

****
Consensus:

TTCAGGCAGGCATAGGTTACTTTGAAAATT

>WB235_Fam_288_30_1 Nr. of seq. 1 Alignment length(with gaps) = 30
Alignment score = 0.000000
WB235_3:3740014-3740583 Satlength=570 Nr of Repeats=9 RepeatLength=30
seed=CCTTGTAGGC Num.seqs=6 Similarity=0.842963
0   CCTTGTAGGCAAGGAGGTAGGCGTAGGTCA

****
Consensus:

CCTTGTAGGCAAGGAGGTAGGCGTAGGTCA

>WB235_Fam_289_30_1 Nr. of seq. 1 Alignment length(with gaps) = 30
Alignment score = 0.000000
WB235_3:13268025-13268308 Satlength=284 Nr of Repeats=8 RepeatLength=30
seed=TTTCAGGCCA Num.seqs=5 Similarity=0.822222
0   TTTCAGGCCAATTTCNGTCATTTTTGGACA

****
Consensus:

TTTCAGGCCAATTTCNGTCATTTTTGGACA

>WB235_Fam_290_30_1 Nr. of seq. 1 Alignment length(with gaps) = 30
Alignment score = 0.000000
WB235_4:16649049-16649496 Satlength=448 Nr of Repeats=15 RepeatLength=30
seed=AAAATCAAAA Num.seqs=12 Similarity=0.938721
0   AAAATCAAAAATTAGATGAATTTTCGATTA

****
Consensus:

AAAATCAAAAATTAGATGAATTTTCGATTA

>WB235_Fam_291_30_1 Nr. of seq. 1 Alignment length(with gaps) = 30
Alignment score = 0.000000

WB235_5:20246749-20247078 Satlength=330 Nr of Repeats=11 RepeatLength=30
seed=CTCAAATTTG Num.seqs=10 Similarity=0.911111
0   CTCAAATTTGCACTAAAAATTCCAATTTTT

****
Consensus:

CTCAAATTTGCACTAAAAATTCCAATTTTT

>WB235_Fam_292_30_1 Nr. of seq. 1 Alignment length(with gaps) = 30
Alignment score = 0.000000
WB235_6:1548656-1549346 Satlength=691 Nr of Repeats=19 RepeatLength=30
seed=ACTACCCAAC Num.seqs=18 Similarity=0.923021
0   ACTACCCAACCGGCTCCGGCAACCAGTCCA

****
Consensus:

ACTACCCAACCGGCTCCGGCAACCAGTCCA

>WB235_Fam_293_29_1 Nr. of seq. 1 Alignment length(with gaps) = 29
Alignment score = 0.000000
WB235_3:10636973-10637342 Satlength=370 Nr of Repeats=11 RepeatLength=29
seed=GGCCTAGAAA Num.seqs=7 Similarity=0.700055
0   GGCCTAGAAANTTCTATTTGAAAGTTGAT

****
Consensus:

GGCCTAGAAANTTCTATTTGAAAGTTGAT

>WB235_Fam_294_29_1 Nr. of seq. 1 Alignment length(with gaps) = 29
Alignment score = 0.000000
WB235_4:13964027-13964143 Satlength=117 Nr of Repeats=4 RepeatLength=29
seed=TACATTGCAA Num.seqs=4 Similarity=0.946360
0   TACATTGCAAGATCTTCTTACTATGTAGG

****
Consensus:

TACATTGCAAGATCTTCTTACTATGTAGG

>WB235_Fam_295_29_1 Nr. of seq. 1 Alignment length(with gaps) = 29
Alignment score = 0.000000
WB235_5:19278568-19278735 Satlength=168 Nr of Repeats=5 RepeatLength=29
seed=CTCGTCAGTG Num.seqs=4 Similarity=0.954023
0   CTCGTCAGTGGGTGGACGAGGAATGAGCG

****
Consensus:

CTCGTCAGTGGGTGGACGAGGAATGAGCG

>WB235_Fam_296_28_1 Nr. of seq. 1 Alignment length(with gaps) = 28
Alignment score = 0.000000
WB235_2:2980111-2980251 Satlength=141 Nr of Repeats=5 RepeatLength=28
seed=AATGTCGGCT Num.seqs=5 Similarity=0.847619
0   AATGTCGGCTGCTTCTAGAAACCAGGCA

****
Consensus:

AATGTCGGCTGCTTCTAGAAACCAGGCA

>WB235_Fam_297_28_1 Nr. of seq. 1 Alignment length(with gaps) = 28
Alignment score = 0.000000
WB235_2:3415307-3415447 Satlength=141 Nr of Repeats=4 RepeatLength=28
seed=TGCCTCGCCT Num.seqs=3 Similarity=0.968254
0   TGCCTCGCCTACGTGCTTATTTCAGGCA

****
Consensus:

TGCCTCGCCTACGTGCTTATTTCAGGCA

>WB235_Fam_298_28_1 Nr. of seq. 1 Alignment length(with gaps) = 28
Alignment score = 0.000000
WB235_5:18447331-18447583 Satlength=253 Nr of Repeats=9 RepeatLength=28
seed=CCAAGAGAAT Num.seqs=9 Similarity=0.915344
0   CCAAGAGAATTTCAACCACCTGGAAATT

****
Consensus:

CCAAGAGAATTTCAACCACCTGGAAATT

>WB235_Fam_299_27_1 Nr. of seq. 1 Alignment length(with gaps) = 27
Alignment score = 0.000000
WB235_2:13668387-13668731 Satlength=345 Nr of Repeats=12 RepeatLength=27
seed=TTCCGGCAAA Num.seqs=8 Similarity=0.904762
0   TTCCGGCAAATCGGCAAATTGCAAAAT

****
Consensus:

TTCCGGCAAATCGGCAAATTGCAAAAT

>WB235_Fam_300_27_1 Nr. of seq. 1 Alignment length(with gaps) = 27
Alignment score = 0.000000
WB235_4:12500507-12500642 Satlength=136 Nr of Repeats=5 RepeatLength=27
seed=ACAATAAAGT Num.seqs=5 Similarity=1.000000
0   ACAATAAAGTACTATGTATTACCGTAT

****
Consensus:

ACAATAAAGTACTATGTATTACCGTAT

>WB235_Fam_301_27_1 Nr. of seq. 1 Alignment length(with gaps) = 27
Alignment score = 0.000000
WB235_6:10973510-10974510 Satlength=1001 Nr of Repeats=30 RepeatLength=27
seed=CACTGACCAA Num.seqs=21 Similarity=0.803410
0   CACTGACCAAAGAAAATATTANACGTG

****
Consensus:

CACTGACCAAAGAAAATATTANACGTG

>WB235_Fam_302_26_1 Nr. of seq. 1 Alignment length(with gaps) = 26
Alignment score = 0.000000
WB235_1:3117070-3117200 Satlength=131 Nr of Repeats=4 RepeatLength=26
seed=GGCAGGCGTC Num.seqs=3 Similarity=0.931624
0   GGCAGGCGTCAAGCAGGCAGGTTTCA

****
Consensus:

GGCAGGCGTCAAGCAGGCAGGTTTCA

>WB235_Fam_303_26_1 Nr. of seq. 1 Alignment length(with gaps) = 26
Alignment score = 0.000000
WB235_1:11312599-11313143 Satlength=545 Nr of Repeats=18 RepeatLength=26
seed=ACCTACAGTA Num.seqs=12 Similarity=0.881896
0   ACCTACAGTACCCCTGTCCTAGAATG

****
Consensus:

ACCTACAGTACCCCTGTCCTAGAATG

>WB235_Fam_304_26_1 Nr. of seq. 1 Alignment length(with gaps) = 26
Alignment score = 0.000000
WB235_2:1178522-1178806 Satlength=285 Nr of Repeats=8 RepeatLength=26
seed=TACTGTAGTT Num.seqs=5 Similarity=0.621429
0   TACTGTAGTTTGACCTACTTANGGAT

****
Consensus:

TACTGTAGTTTGACCTACTTANGGAT

>WB235_Fam_305_26_1 Nr. of seq. 1 Alignment length(with gaps) = 26
Alignment score = 0.000000
WB235_2:1892810-1893486 Satlength=677 Nr of Repeats=26 RepeatLength=26
seed=ATTTACGGAG Num.seqs=22 Similarity=0.829282
0   ATTTACGGAGCTTCAAGCTTGTGTCG

****
Consensus:

ATTTACGGAGCTTCAAGCTTGTGTCG

>WB235_Fam_306_26_1 Nr. of seq. 1 Alignment length(with gaps) = 26
Alignment score = 0.000000
WB235_5:19065856-19066708 Satlength=853 Nr of Repeats=32 RepeatLength=26
seed=TGAAATTTCA Num.seqs=23 Similarity=0.906760
0   TGAAATTTCACAAAATATCGATTTTC

****
Consensus:

TGAAATTTCACAAAATATCGATTTTC

>WB235_Fam_307_26_1 Nr. of seq. 1 Alignment length(with gaps) = 26
Alignment score = 0.000000
WB235_6:11260441-11260545 Satlength=105 Nr of Repeats=4 RepeatLength=26
seed=AAATTAATCT Num.seqs=4 Similarity=0.803419
0   AAATTAATCTGTCACTCTAACTTTTT

****
Consensus:

AAATTAATCTGTCACTCTAACTTTTT

>WB235_Fam_308_25_1 Nr. of seq. 1 Alignment length(with gaps) = 25
Alignment score = 0.000000
WB235_2:1089504-1089883 Satlength=380 Nr of Repeats=5 RepeatLength=26
seed=GTCGGCTGCT Num.seqs=3 Similarity=0.339869
0   GTCGGCTGCTTNCAAACCCTGAGTT

****
Consensus:

GTCGGCTGCTTNCAAACCCTGAGTT

>WB235_Fam_309_25_1 Nr. of seq. 1 Alignment length(with gaps) = 25
Alignment score = 0.000000
WB235_5:19420419-19420546 Satlength=128 Nr of Repeats=5 RepeatLength=25
seed=AAAAATCTGT Num.seqs=3 Similarity=0.822222
0   AAAAATCTGTTCTNNGAGGTCCCTG

****
Consensus:

AAAAATCTGTTCTNNGAGGTCCCTG

>WB235_Fam_310_24_1 Nr. of seq. 1 Alignment length(with gaps) = 24
Alignment score = 0.000000
WB235_2:4205778-4205946 Satlength=169 Nr of Repeats=5 RepeatLength=24
seed=GTGAGGTACG Num.seqs=3 Similarity=0.611111
0   GTGAGGTACGAGGTGAGGTANGAG

****

Consensus:

GTGAGGTACGAGGTGAGGTANGAG

>WB235_Fam_311_24_1 Nr. of seq. 1 Alignment length(with gaps) = 24
Alignment score = 0.000000
WB235_3:12397168-12397264 Satlength=97 Nr of Repeats=4 RepeatLength=24
seed=AAGCACCACC Num.seqs=4 Similarity=0.972222
0   AAGCACCACCTCCTCCAACTACAA

****
Consensus:

AAGCACCACCTCCTCCAACTACAA

>WB235_Fam_312_24_1 Nr. of seq. 1 Alignment length(with gaps) = 24
Alignment score = 0.000000
WB235_3:13583331-13583502 Satlength=172 Nr of Repeats=7 RepeatLength=24
seed=AAGAAGGATG Num.seqs=6 Similarity=0.825926
0   AAGAAGGATGCTGAAGAGAAGAAG

****
Consensus:

AAGAAGGATGCTGAAGAGAAGAAG

>WB235_Fam_313_24_1 Nr. of seq. 1 Alignment length(with gaps) = 24
Alignment score = 0.000000
WB235_4:2855122-2855338 Satlength=217 Nr of Repeats=5 RepeatLength=24
seed=GCTTCTGGAG Num.seqs=3 Similarity=0.777778
0   GCTTCTGGAGCTACAGGTTATCCA

****
Consensus:

GCTTCTGGAGCTACAGGTTATCCA

>WB235_Fam_314_24_1 Nr. of seq. 1 Alignment length(with gaps) = 24
Alignment score = 0.000000
WB235_4:17323162-17323330 Satlength=169 Nr of Repeats=7 RepeatLength=24
seed=TCAACAACAA Num.seqs=7 Similarity=1.000000
0   TCAACAACAACAGGAGAAGCTTCT

****
Consensus:

TCAACAACAACAGGAGAAGCTTCT

>WB235_Fam_315_24_1 Nr. of seq. 1 Alignment length(with gaps) = 24
Alignment score = 0.000000
WB235_5:6171700-6176164 Satlength=4465 Nr of Repeats=182 RepeatLength=24
seed=TTTCAGTTTA Num.seqs=178 Similarity=0.769398
0   TTTCAGTTTATCATCATTCTCCTG

****
Consensus:

TTTCAGTTTATCATCATTCTCCTG

>WB235_Fam_316_24_1 Nr. of seq. 1 Alignment length(with gaps) = 24
Alignment score = 0.000000
WB235_6:7879877-7880045 Satlength=169 Nr of Repeats=7 RepeatLength=24
seed=GGTGGCTGGT Num.seqs=7 Similarity=1.000000
0   GGTGGCTGGTCACAAAAGTAAGTT

****
Consensus:

GGTGGCTGGTCACAAAAGTAAGTT

>WB235_Fam_317_23_1 Nr. of seq. 1 Alignment length(with gaps) = 23
Alignment score = 0.000000
WB235_1:14332472-14332641 Satlength=170 Nr of Repeats=5 RepeatLength=23
seed=GCACCATAAG Num.seqs=3 Similarity=0.726852
0   GCATCATAAGGATCATAGTTGAT

****
Consensus:

GCATCATAAGGATCATAGTTGAT

>WB235_Fam_318_23_1 Nr. of seq. 1 Alignment length(with gaps) = 23
Alignment score = 0.000000
WB235_2:1578528-1579311 Satlength=784 Nr of Repeats=24 RepeatLength=23
seed=CGCTGTAAGA Num.seqs=16 Similarity=0.806763
0   CGCTGTAAGACCCTTCGCTGTAC

****
Consensus:

CGCTGTAAGACCCTTCGCTGTAC

>WB235_Fam_319_23_1 Nr. of seq. 1 Alignment length(with gaps) = 23
Alignment score = 0.000000
WB235_2:15278603-15278843 Satlength=241 Nr of Repeats=12 RepeatLength=23
seed=GCTACAGTAC Num.seqs=8 Similarity=0.821946
0   GCTACAGTACCCCAGCTTGACTT

****
Consensus:

GCTACAGTACCCCAGCTTGACTT

>WB235_Fam_320_23_1 Nr. of seq. 1 Alignment length(with gaps) = 23
Alignment score = 0.000000

WB235_5:19487887-19488024 Satlength=138 Nr of Repeats=6 RepeatLength=23
seed=TTTTCAGAAA Num.seqs=5 Similarity=0.631944
0   TTTTCAGAAATAGGTATTTCAGA

****
Consensus:

TTTTCAGAAATAGGTATTTCAGA

>WB235_Fam_321_22_1 Nr. of seq. 1 Alignment length(with gaps) = 22
Alignment score = 0.000000
WB235_1:808432-808756 Satlength=325 Nr of Repeats=8 RepeatLength=22
seed=TTAAAATTTT Num.seqs=6 Similarity=0.886869
0   TTAAAATTTTGTCAGTTTGACC

****
Consensus:

TTAAAATTTTGTCAGTTTGACC

>WB235_Fam_322_22_1 Nr. of seq. 1 Alignment length(with gaps) = 22
Alignment score = 0.000000
WB235_1:14267336-14267775 Satlength=440 Nr of Repeats=17 RepeatLength=22
seed=GCACGGCCTG Num.seqs=13 Similarity=0.701226
0   GCACGGCCTGGCGGTCCTGAGG

****
Consensus:

GCACGGCCTGGCGGTCCTGAGG

>WB235_Fam_323_22_1 Nr. of seq. 1 Alignment length(with gaps) = 22
Alignment score = 0.000000
WB235_2:14114648-14115030 Satlength=383 Nr of Repeats=12 RepeatLength=22
seed=CTACAGTAAC Num.seqs=8 Similarity=0.798395
0   CTACAGTAACCCTAGTATTGAA

****
Consensus:

CTACAGTAACCCTAGTATTGAA

>WB235_Fam_324_22_1 Nr. of seq. 1 Alignment length(with gaps) = 22
Alignment score = 0.000000
WB235_4:6599056-6599139 Satlength=84 Nr of Repeats=4 RepeatLength=22
seed=AAGCGGTAAC Num.seqs=3 Similarity=1.000000
0   AAGCGGTAACGTCGTCTATATA

****
Consensus:

AAGCGGTAACGTCGTCTATATA

```
>WB235_Fam_325_22_1  Nr. of seq. 1 Alignment length(with gaps) = 22
Alignment score = 0.000000
WB235_5:16945467-16945572 Satlength=106 Nr of Repeats=5 RepeatLength=21
seed=TGCTAATTAG  Num.seqs=3 Similarity=0.545455
0    TGCTAATTAGCGCGGNTGCGTN

**********************
Consensus:

TGCTAATTAGCGCGGNTGCGTN

>WB235_Fam_326_22_1  Nr. of seq. 1 Alignment length(with gaps) = 22
Alignment score = 0.000000
WB235_5:19427400-19427634 Satlength=235 Nr of Repeats=7 RepeatLength=22
seed=ACGTATACAG  Num.seqs=5 Similarity=0.915152
0    ACGTATACAGGCATATACACAT

**********************
Consensus:

ACGTATACAGGCATATACACAT

>WB235_Fam_327_22_1  Nr. of seq. 1 Alignment length(with gaps) = 22
Alignment score = 0.000000
WB235_6:1916196-1916614 Satlength=419 Nr of Repeats=14 RepeatLength=22
seed=CCGGCGTGGT  Num.seqs=9 Similarity=0.677134
0    CCGGCGTGGTNTTGAAGCGGTC

**********************
Consensus:

CCGGCGTGGTNTTGAAGCGGTC

>WB235_Fam_328_22_1  Nr. of seq. 1 Alignment length(with gaps) = 22
Alignment score = 0.000000
WB235_6:14638909-14639063 Satlength=155 Nr of Repeats=5 RepeatLength=22
seed=TTTGTGGGAG  Num.seqs=3 Similarity=0.757576
0    TTTGTGGGAGGCGACATATACC

**********************
Consensus:

TTTGTGGGAGGCGACATATACC

>WB235_Fam_329_21_1  Nr. of seq. 1 Alignment length(with gaps) = 21
Alignment score = 0.000000
WB235_1:823801-824454 Satlength=654 Nr of Repeats=29 RepeatLength=21
seed=TGGCCTAGAT  Num.seqs=21 Similarity=0.658586
0    TGGCCTAGATTNTTTACAGGG

*********************
Consensus:

TGGCCTAGATTNTTTACAGGG
```

>WB235_Fam_330_21_1 Nr. of seq. 1 Alignment length(with gaps) = 21
Alignment score = 0.000000
WB235_1:1652306-1652642 Satlength=337 Nr of Repeats=16 RepeatLength=21
seed=TCTCCTGGTA Num.seqs=16 Similarity=0.963492
0   TCTCCTGGTAAATTTAAAAAT

****
Consensus:

TCTCCTGGTAAATTTAAAAAT

>WB235_Fam_331_21_1 Nr. of seq. 1 Alignment length(with gaps) = 21
Alignment score = 0.000000
WB235_1:12585721-12585826 Satlength=106 Nr of Repeats=5 RepeatLength=21
seed=ACCACCCCTA Num.seqs=5 Similarity=0.815873
0   ACCACCCCTACAACNACCACC

****
Consensus:

ACCACCCCTACAACNACCACC

>WB235_Fam_332_21_1 Nr. of seq. 1 Alignment length(with gaps) = 21
Alignment score = 0.000000
WB235_1:15025277-15025549 Satlength=273 Nr of Repeats=10 RepeatLength=21
seed=AACTTTTTGG Num.seqs=6 Similarity=0.791919
0   AACTTTTTGGCAATTTTCCAA

****
Consensus:

AACTTTTTGGCAATTTTCCAA

>WB235_Fam_333_21_1 Nr. of seq. 1 Alignment length(with gaps) = 21
Alignment score = 0.000000
WB235_2:2273842-2274055 Satlength=214 Nr of Repeats=8 RepeatLength=21
seed=TGTGCCCTGG Num.seqs=6 Similarity=0.936508
0   TGTGCCCTGGAAAGCTGGCAG

****
Consensus:

TGTGCCCTGGAAAGCTGGCAG

>WB235_Fam_334_21_1 Nr. of seq. 1 Alignment length(with gaps) = 21
Alignment score = 0.000000
WB235_2:4054458-4054819 Satlength=362 Nr of Repeats=4 RepeatLength=21
seed=CTACGTAAGA Num.seqs=3 Similarity=0.830688
0   CTACGTAAGAGTAGCGCAGGA

****
Consensus:

CTACGTAAGAGTAGCGCAGGA

>WB235_Fam_335_21_1 Nr. of seq. 1 Alignment length(with gaps) = 21
Alignment score = 0.000000
WB235_2:13184130-13184277 Satlength=148 Nr of Repeats=7 RepeatLength=21
seed=AATCTAGCCA Num.seqs=7 Similarity=0.930461
0   AATCTAGCCAGACGCACACCT

****
Consensus:

AATCTAGCCAGACGCACACCT

>WB235_Fam_336_21_1 Nr. of seq. 1 Alignment length(with gaps) = 21
Alignment score = 0.000000
WB235_2:14212202-14212432 Satlength=231 Nr of Repeats=10 RepeatLength=21
seed=CAATTTTCTG Num.seqs=8 Similarity=0.841270
0   CAATTTTCTGTTNAACTTTGA

****
Consensus:

CAATTTTCTGTTNAACTTTGA

>WB235_Fam_337_21_1 Nr. of seq. 1 Alignment length(with gaps) = 21
Alignment score = 0.000000
WB235_3:8254497-8254602 Satlength=106 Nr of Repeats=5 RepeatLength=21
seed=AAGTTTTACT Num.seqs=5 Similarity=0.974603
0   AAGTTTTACTTTGATTTTTTT

****
Consensus:

AAGTTTTACTTTGATTTTTTT

>WB235_Fam_338_21_1 Nr. of seq. 1 Alignment length(with gaps) = 21
Alignment score = 0.000000
WB235_3:11242813-11243253 Satlength=441 Nr of Repeats=21 RepeatLength=21
seed=GGCAATTTTC Num.seqs=20 Similarity=0.903425
0   GGCAATTTTCCAGTGCAATTC

****
Consensus:

GGCAATTTTCCAGTGCAATTC

>WB235_Fam_339_21_1 Nr. of seq. 1 Alignment length(with gaps) = 21
Alignment score = 0.000000
WB235_4:1565274-1565421 Satlength=148 Nr of Repeats=7 RepeatLength=21
seed=CCATAGGTGG Num.seqs=7 Similarity=0.794407
0   CCATAGGTGGAAGATCTNATA

****

Consensus:

CCATAGGTGGAAGATCTNATA

>WB235_Fam_340_21_1 Nr. of seq. 1 Alignment length(with gaps) = 21
Alignment score = 0.000000
WB235_4:2156341-2157203 Satlength=863 Nr of Repeats=38 RepeatLength=21
seed=GATTTACGGG Num.seqs=31 Similarity=0.686520
0   GATTTACGGGGTGAACATGTG

****
Consensus:

GATTTACGGGGTGAACATGTG

>WB235_Fam_341_21_1 Nr. of seq. 1 Alignment length(with gaps) = 21
Alignment score = 0.000000
WB235_5:6392866-6392950 Satlength=85 Nr of Repeats=4 RepeatLength=21
seed=TTGGTGACAA Num.seqs=4 Similarity=1.000000
0   TTGGTGACAATGTGGAAACAA

****
Consensus:

TTGGTGACAATGTGGAAACAA

>WB235_Fam_342_21_1 Nr. of seq. 1 Alignment length(with gaps) = 21
Alignment score = 0.000000
WB235_5:9933015-9933120 Satlength=106 Nr of Repeats=5 RepeatLength=21
seed=TTCTAATTCG Num.seqs=5 Similarity=0.726984
0   TTCTAATTCGACGCGCATNGT

****
Consensus:

TTCTAATTCGACGCGCATNGT

>WB235_Fam_343_21_1 Nr. of seq. 1 Alignment length(with gaps) = 21
Alignment score = 0.000000
WB235_5:16941584-16941761 Satlength=178 Nr of Repeats=5 RepeatLength=21
seed=CTAATTAGCG Num.seqs=3 Similarity=0.640212
0   CTAATTAGCGTCGCTTCCNTT

****
Consensus:

CTAATTAGCGTCGCTTCCNTT

>WB235_Fam_344_21_1 Nr. of seq. 1 Alignment length(with gaps) = 21
Alignment score = 0.000000
WB235_5:18644347-18644921 Satlength=575 Nr of Repeats=24 RepeatLength=21
seed=AAAAATCGAT Num.seqs=18 Similarity=0.739602
0   AAAAATCGATAAAATTTGGAN

****
Consensus:

AAAAATCGATAAAATTTGGAN

>WB235_Fam_345_21_1 Nr. of seq. 1 Alignment length(with gaps) = 21
Alignment score = 0.000000
WB235_5:19261027-19261174 Satlength=148 Nr of Repeats=7 RepeatLength=21
seed=CACACGAGCT Num.seqs=7 Similarity=0.727891
0   CACACGAGCTTGAGAGCCCGG

****
Consensus:

CACACGAGCTTGAGAGCCCGG

>WB235_Fam_346_21_1 Nr. of seq. 1 Alignment length(with gaps) = 21
Alignment score = 0.000000
WB235_5:19427290-19427806 Satlength=517 Nr of Repeats=5 RepeatLength=21
seed=TATTTTCAAA Num.seqs=3 Similarity=0.957672
0   TATTTTCAAAAAAATTTAAAA

****
Consensus:

TATTTTCAAAAAAATTTAAAA

>WB235_Fam_347_21_1 Nr. of seq. 1 Alignment length(with gaps) = 21
Alignment score = 0.000000
WB235_5:19645096-19645264 Satlength=169 Nr of Repeats=6 RepeatLength=21
seed=ACCCCGATTT Num.seqs=5 Similarity=0.911111
0   ACCCCGATTTTCACCCAAAAA

****
Consensus:

ACCCCGATTTTCACCCAAAAA

>WB235_Fam_348_21_1 Nr. of seq. 1 Alignment length(with gaps) = 21
Alignment score = 0.000000
WB235_5:19963720-19963804 Satlength=85 Nr of Repeats=4 RepeatLength=21
seed=CTCAATTCAA Num.seqs=4 Similarity=0.883598
0   CTCAATTCAATCAAGGAAATC

****
Consensus:

CTCAATTCAATCAAGGAAATC

>WB235_Fam_349_21_1 Nr. of seq. 1 Alignment length(with gaps) = 21
Alignment score = 0.000000

WB235_6:3713912-3714032 Satlength=121 Nr of Repeats=5 RepeatLength=20
seed=CCATGTAGCT Num.seqs=4 Similarity=0.561869
0   CCATGTAGCTTCAAAATTNCT

****
Consensus:

CCATGTAGCTTCAAAATTNCT

>WB235_Fam_350_21_1 Nr. of seq. 1 Alignment length(with gaps) = 21
Alignment score = 0.000000
WB235_6:15507328-15507477 Satlength=150 Nr of Repeats=4 RepeatLength=21
seed=TGTCCGTGAT Num.seqs=3 Similarity=0.666667
0   TGTCCGTGATGATCATGNGCA

****
Consensus:

TGTCCGTGATGATCATGNGCA

>WB235_Fam_351_21_1 Nr. of seq. 1 Alignment length(with gaps) = 21
Alignment score = 0.000000
WB235_6:17087637-17087742 Satlength=106 Nr of Repeats=5 RepeatLength=21
seed=GGTGGACCCA Num.seqs=5 Similarity=0.822222
0   GGTGGACCCANGGGTTCCTAG

****
Consensus:

GGTGGACCCANGGGTTCCTAG

>WB235_Fam_352_20_1 Nr. of seq. 1 Alignment length(with gaps) = 20
Alignment score = 0.000000
WB235_2:3641762-3641852 Satlength=91 Nr of Repeats=5 RepeatLength=20
seed=CTATCGCCTG Num.seqs=4 Similarity=0.833333
0   CTATCGCCTGCTATCGCCTA

****
Consensus:

CTATCGCCTGCTATCGCCTA

>WB235_Fam_353_20_1 Nr. of seq. 1 Alignment length(with gaps) = 20
Alignment score = 0.000000
WB235_5:17780829-17781140 Satlength=312 Nr of Repeats=5 RepeatLength=20
seed=ATTTGACCTA Num.seqs=4 Similarity=0.900000
0   ATTTGACCTACTTTTTAAAA

****
Consensus:

ATTTGACCTACTTTTTAAAA

>WB235_Fam_354_19_1 Nr. of seq. 1 Alignment length(with gaps) = 19
Alignment score = 0.000000
WB235_1:359078-359230 Satlength=153 Nr of Repeats=8 RepeatLength=19
seed=GTACTACCTT Num.seqs=8 Similarity=0.982456
0   GTACTACCTTTTAATGTAT

****
Consensus:

GTACTACCTTTTAATGTAT

>WB235_Fam_355_19_1 Nr. of seq. 1 Alignment length(with gaps) = 19
Alignment score = 0.000000
WB235_1:14137893-14138026 Satlength=134 Nr of Repeats=7 RepeatLength=19
seed=CCCTGATACC Num.seqs=7 Similarity=0.788889
0   CCCTGATACCCCCTAGTCC

****
Consensus:

CCCTGATACCCCCTAGTCC

>WB235_Fam_356_19_1 Nr. of seq. 1 Alignment length(with gaps) = 19
Alignment score = 0.000000
WB235_2:13007141-13007285 Satlength=145 Nr of Repeats=6 RepeatLength=19
seed=TATGGTGCAT Num.seqs=4 Similarity=0.707602
0   TATGGTGCATCTAACTAGT

****
Consensus:

TATGGTGCATCTAACTAGT

>WB235_Fam_357_19_1 Nr. of seq. 1 Alignment length(with gaps) = 19
Alignment score = 0.000000
WB235_4:3206393-3209585 Satlength=3193 Nr of Repeats=168 RepeatLength=19
seed=GCCTCACTCA Num.seqs=132 Similarity=0.897464
0   GCCTCACTCAAAATCCTCA

****
Consensus:

GCCTCACTCAAAATCCTCA

>WB235_Fam_358_18_1 Nr. of seq. 1 Alignment length(with gaps) = 18
Alignment score = 0.000000
WB235_1:10650908-10650980 Satlength=73 Nr of Repeats=4 RepeatLength=18
seed=AAAAGTTACA Num.seqs=4 Similarity=1.000000
0   AAAAGTTACAGTGTCCTT

****
Consensus:

AAAAGTTACAGTGTCCTT

```
>WB235_Fam_359_18_1  Nr. of seq. 1 Alignment length(with gaps) = 18
Alignment score = 0.000000
WB235_1:14338207-14338743 Satlength=537 Nr of Repeats=26 RepeatLength=18
seed=TTGTAGATCA  Num.seqs=22 Similarity=0.869489
0    TTGTAGATCAACGGGGTC

******************
Consensus:

TTGTAGATCAACGGGGTC

>WB235_Fam_360_18_1  Nr. of seq. 1 Alignment length(with gaps) = 18
Alignment score = 0.000000
WB235_2:1467853-1467943 Satlength=91 Nr of Repeats=5 RepeatLength=18
seed=GGAGGTCCAG  Num.seqs=5 Similarity=0.859259
0    GGAGGTCCAGGTACCCCG

******************
Consensus:

GGAGGTCCAGGTACCCCG

>WB235_Fam_361_18_1  Nr. of seq. 1 Alignment length(with gaps) = 18
Alignment score = 0.000000
WB235_2:10466292-10466385 Satlength=94 Nr of Repeats=5 RepeatLength=18
seed=TAAATAAGTG  Num.seqs=3 Similarity=0.950617
0    TAAATAAGTGGCTATAAT

******************
Consensus:

TAAATAAGTGGCTATAAT

>WB235_Fam_362_18_1  Nr. of seq. 1 Alignment length(with gaps) = 18
Alignment score = 0.000000
WB235_2:12889260-12889656 Satlength=397 Nr of Repeats=21 RepeatLength=18
seed=ACTACTGTAT  Num.seqs=18 Similarity=0.797628
0    ACTACTGTATCNCAGGAG

******************
Consensus:

ACTACTGTATCNCAGGAG

>WB235_Fam_363_18_1  Nr. of seq. 1 Alignment length(with gaps) = 18
Alignment score = 0.000000
WB235_4:3195411-3195573 Satlength=163 Nr of Repeats=6 RepeatLength=18
seed=CCTTCGCCTA  Num.seqs=4 Similarity=0.765432
0    CCTTCGCCTACCTACGTG

******************
Consensus:
```

CCTTCGCCTACCTACGTG

>WB235_Fam_364_18_1 Nr. of seq. 1 Alignment length(with gaps) = 18
Alignment score = 0.000000
WB235_4:4335057-4335129 Satlength=73 Nr of Repeats=4 RepeatLength=18
seed=TTTTTTGATA Num.seqs=4 Similarity=0.962963
0   TTTTTTGATAATTCTTGA

****
Consensus:

TTTTTTGATAATTCTTGA

>WB235_Fam_365_18_1 Nr. of seq. 1 Alignment length(with gaps) = 18
Alignment score = 0.000000
WB235_5:4268549-4268631 Satlength=83 Nr of Repeats=4 RepeatLength=18
seed=AAATATTCTT Num.seqs=3 Similarity=0.851852
0   AAATATTCTTATTTTCAG

****
Consensus:

AAATATTCTTATTTTCAG

>WB235_Fam_366_18_1 Nr. of seq. 1 Alignment length(with gaps) = 18
Alignment score = 0.000000
WB235_5:4653023-4653224 Satlength=202 Nr of Repeats=8 RepeatLength=18
seed=CCTGGCTGTC Num.seqs=5 Similarity=0.607407
0   CCTGGTTGTCCNTCTNNT

****
Consensus:

CCTGGTTGTCCNTCTNNT

>WB235_Fam_367_18_1 Nr. of seq. 1 Alignment length(with gaps) = 18
Alignment score = 0.000000
WB235_5:7337779-7337924 Satlength=146 Nr of Repeats=8 RepeatLength=18
seed=TTTCATAAGA Num.seqs=7 Similarity=0.943563
0   TTTCATAAGAAGTAAATN

****
Consensus:

TTTCATAAGAAGTAAATN

>WB235_Fam_368_18_1 Nr. of seq. 1 Alignment length(with gaps) = 18
Alignment score = 0.000000
WB235_5:20210955-20211513 Satlength=559 Nr of Repeats=29 RepeatLength=18
seed=CATTGTCACT Num.seqs=27 Similarity=0.884774
0   CATTGTCACTTTCTTCTC

****

Consensus:

CATTGTCACTTTCTTCTC

>WB235_Fam_369_18_1 Nr. of seq. 1 Alignment length(with gaps) = 18
Alignment score = 0.000000
WB235_6:1836329-1837109 Satlength=781 Nr of Repeats=39 RepeatLength=18
seed=AAGCCTATGC Num.seqs=28 Similarity=0.765628
0   AAGCCTATGCCTTTCCCA

****
Consensus:

AAGCCTATGCCTTTCCCA

>WB235_Fam_370_17_1 Nr. of seq. 1 Alignment length(with gaps) = 17
Alignment score = 0.000000
WB235_2:12575393-12575478 Satlength=86 Nr of Repeats=5 RepeatLength=17
seed=ACTTGGTACA Num.seqs=5 Similarity=0.890196
0   ACTTGGTACATAGGCCA

****
Consensus:

ACTTGGTACATAGGCCA

>WB235_Fam_371_17_1 Nr. of seq. 1 Alignment length(with gaps) = 17
Alignment score = 0.000000
WB235_3:12389240-12389685 Satlength=446 Nr of Repeats=15 RepeatLength=17
seed=ATTTTTTCCG Num.seqs=10 Similarity=0.687449
0   ATTTTTTCCGCAAGTAG

****
Consensus:

ATTTTTTCCGCAAGTAG

>WB235_Fam_372_17_1 Nr. of seq. 1 Alignment length(with gaps) = 17
Alignment score = 0.000000
WB235_4:11615692-11615955 Satlength=264 Nr of Repeats=13 RepeatLength=17
seed=AAAATCAAAC Num.seqs=10 Similarity=0.809259
0   AAAATCAAACCGGCGGA

****
Consensus:

AAAATCAAACCGGCGGA

>WB235_Fam_373_17_1 Nr. of seq. 1 Alignment length(with gaps) = 17
Alignment score = 0.000000
WB235_5:4866206-4866377 Satlength=172 Nr of Repeats=5 RepeatLength=17
seed=AAATGCCAAA Num.seqs=3 Similarity=0.641975
0   AAATGCCAAAANTGCCA

****
Consensus:

AAATGCCAAAANTGCCA

>WB235_Fam_374_17_1 Nr. of seq. 1 Alignment length(with gaps) = 17
Alignment score = 0.000000
WB235_5:16672548-16672735 Satlength=188 Nr of Repeats=11 RepeatLength=17
seed=TAACCTGACT Num.seqs=11 Similarity=0.957219
0   TAACCTGACTGCTACAG

****
Consensus:

TAACCTGACTGCTACAG

>WB235_Fam_375_17_1 Nr. of seq. 1 Alignment length(with gaps) = 17
Alignment score = 0.000000
WB235_5:19402253-19402490 Satlength=238 Nr of Repeats=14 RepeatLength=17
seed=GAATTAGAAA Num.seqs=13 Similarity=0.554584
0   GAATTAGAAAAATTTGA

****
Consensus:

GAATTAGAAAAATTTGA

>WB235_Fam_376_17_1 Nr. of seq. 1 Alignment length(with gaps) = 17
Alignment score = 0.000000
WB235_5:19488506-19489530 Satlength=1025 Nr of Repeats=58 RepeatLength=17
seed=TTGCAAGAAA Num.seqs=55 Similarity=0.923312
0   TTGCAAGAAAATCTGCA

****
Consensus:

TTGCAAGAAAATCTGCA

>WB235_Fam_377_17_1 Nr. of seq. 1 Alignment length(with gaps) = 17
Alignment score = 0.000000
WB235_6:3347373-3347577 Satlength=205 Nr of Repeats=10 RepeatLength=17
seed=TTTTAACGTG Num.seqs=8 Similarity=0.901961
0   TTTTAACGTGACCTAGT

****
Consensus:

TTTTAACGTGACCTAGT

>WB235_Fam_378_16_1 Nr. of seq. 1 Alignment length(with gaps) = 16
Alignment score = 0.000000

WB235_1:12562161-12562497 Satlength=337 Nr of Repeats=20 RepeatLength=16
seed=TGATCTGATG Num.seqs=18 Similarity=0.831699
0   TGATCTGATGATCTGA

****
Consensus:

TGATCTGATGATCTGA

>WB235_Fam_379_16_1 Nr. of seq. 1 Alignment length(with gaps) = 16
Alignment score = 0.000000
WB235_1:13093190-13093270 Satlength=81 Nr of Repeats=5 RepeatLength=16
seed=AATACGGGAA Num.seqs=5 Similarity=1.000000
0   AATACGGGAATACGGG

****
Consensus:

AATACGGGAATACGGG

>WB235_Fam_380_16_1 Nr. of seq. 1 Alignment length(with gaps) = 16
Alignment score = 0.000000
WB235_3:1011046-1011124 Satlength=79 Nr of Repeats=5 RepeatLength=16
seed=TTATAGTGTT Num.seqs=3 Similarity=0.888889
0   TTATAGTGTTATAGTG

****
Consensus:

TTATAGTGTTATAGTG

>WB235_Fam_381_16_1 Nr. of seq. 1 Alignment length(with gaps) = 16
Alignment score = 0.000000
WB235_3:3018177-3018907 Satlength=731 Nr of Repeats=5 RepeatLength=16
seed=TGTATATATA Num.seqs=3 Similarity=1.000000
0   TGTATATATATATATA

****
Consensus:

TGTATATATATATATA

>WB235_Fam_382_16_1 Nr. of seq. 1 Alignment length(with gaps) = 16
Alignment score = 0.000000
WB235_5:12077837-12077917 Satlength=81 Nr of Repeats=5 RepeatLength=16
seed=AAAAATTATT Num.seqs=5 Similarity=1.000000
0   AAAAATTATTGAATAC

****
Consensus:

AAAAATTATTGAATAC

>WB235_Fam_383_16_1 Nr. of seq. 1 Alignment length(with gaps) = 16
Alignment score = 0.000000
WB235_5:17653226-17653322 Satlength=97 Nr of Repeats=6 RepeatLength=16
seed=GAATAATATT Num.seqs=6 Similarity=0.972222
0   GAATAATATTGGAGAA

****
Consensus:

GAATAATATTGGAGAA

>WB235_Fam_384_16_1 Nr. of seq. 1 Alignment length(with gaps) = 16
Alignment score = 0.000000
WB235_5:19753320-19753624 Satlength=305 Nr of Repeats=19 RepeatLength=16
seed=CTTAGATCTC Num.seqs=19 Similarity=0.931287
0   CTTAGATCTCAATTCC

****
Consensus:

CTTAGATCTCAATTCC

>WB235_Fam_385_16_1 Nr. of seq. 1 Alignment length(with gaps) = 16
Alignment score = 0.000000
WB235_6:2989994-2990074 Satlength=81 Nr of Repeats=5 RepeatLength=16
seed=AGAGAGTATA Num.seqs=5 Similarity=1.000000
0   AGAGAGTATAGATTAG

****
Consensus:

AGAGAGTATAGATTAG

>WB235_Fam_386_15_1 Nr. of seq. 1 Alignment length(with gaps) = 15
Alignment score = 0.000000
WB235_3:12239290-12239399 Satlength=110 Nr of Repeats=5 RepeatLength=15
seed=CGACCTGCGA Num.seqs=3 Similarity=0.940741
0   CGACCTGCGACCTAG

****
Consensus:

CGACCTGCGACCTAG

>WB235_Fam_387_15_1 Nr. of seq. 1 Alignment length(with gaps) = 15
Alignment score = 0.000000
WB235_4:13431343-13431418 Satlength=76 Nr of Repeats=5 RepeatLength=15
seed=CACAGGAGGC Num.seqs=5 Similarity=1.000000
0   CACAGGAGGCTGGGA

****
Consensus:

CACAGGAGGCTGGGA

>WB235_Fam_388_14_1 Nr. of seq. 1 Alignment length(with gaps) = 14
Alignment score = 0.000000
WB235_1:11084941-11085053 Satlength=113 Nr of Repeats=7 RepeatLength=14
seed=AGCTTGAAGC Num.seqs=5 Similarity=0.800000
0   AGCTTGAAGCTTGG

****
Consensus:

AGCTTGAAGCTTGG

>WB235_Fam_389_14_1 Nr. of seq. 1 Alignment length(with gaps) = 14
Alignment score = 0.000000
WB235_2:7446830-7448192 Satlength=1363 Nr of Repeats=34 RepeatLength=14
seed=GAAGGATCGG Num.seqs=26 Similarity=0.910916
0   GAAGGATCGGGCCG

****
Consensus:

GAAGGATCGGGCCG

>WB235_Fam_390_14_1 Nr. of seq. 1 Alignment length(with gaps) = 14
Alignment score = 0.000000
WB235_6:7918015-7918071 Satlength=57 Nr of Repeats=4 RepeatLength=14
seed=TTACCAAAAT Num.seqs=4 Similarity=0.888889
0   TTACCAAAATCTAA

****
Consensus:

TTACCAAAATCTAA

>WB235_Fam_391_13_1 Nr. of seq. 1 Alignment length(with gaps) = 13
Alignment score = 0.000000
WB235_5:19495335-19495400 Satlength=66 Nr of Repeats=5 RepeatLength=13
seed=TTGGGGTGGG Num.seqs=5 Similarity=1.000000
0   TTGGGGTGGGCCT

****
Consensus:

TTGGGGTGGGCCT

>WB235_Fam_392_12_1 Nr. of seq. 1 Alignment length(with gaps) = 12
Alignment score = 0.000000
WB235_2:13522160-13522220 Satlength=61 Nr of Repeats=5 RepeatLength=12
seed=AATACCAACC Num.seqs=5 Similarity=0.933333
0   AATACCAACCAC

****
Consensus:

AATACCAACCAC

>WB235_Fam_393_12_1 Nr. of seq. 1 Alignment length(with gaps) = 12
Alignment score = 0.000000
WB235_3:13129352-13129936 Satlength=585 Nr of Repeats=45 RepeatLength=12
seed=AGTTATATAG Num.seqs=41 Similarity=0.931707
0   AGTTATATAGTA

****
Consensus:

AGTTATATAGTA

>WB235_Fam_394_12_1 Nr. of seq. 1 Alignment length(with gaps) = 12
Alignment score = 0.000000
WB235_5:16530645-16530908 Satlength=264 Nr of Repeats=16 RepeatLength=12
seed=CATTGACTTG Num.seqs=13 Similarity=0.842209
0   CATTGACTTGAG

****
Consensus:

CATTGACTTGAG

>WB235_Fam_395_11_1 Nr. of seq. 1 Alignment length(with gaps) = 11
Alignment score = 0.000000
WB235_4:2896505-2896560 Satlength=56 Nr of Repeats=5 RepeatLength=11
seed=TTGTTTACAA Num.seqs=5 Similarity=1.000000
0   TTGTTTACAAG

****
Consensus:

TTGTTTACAAG

>WB235_Fam_396_11_1 Nr. of seq. 1 Alignment length(with gaps) = 11
Alignment score = 0.000000
WB235_5:1249610-1249931 Satlength=322 Nr of Repeats=10 RepeatLength=11
seed=TACCTACTAG Num.seqs=6 Similarity=0.789899
0   TACCTACTAGA

****
Consensus:

TACCTACTAGA

>WB235_Fam_397_11_1 Nr. of seq. 1 Alignment length(with gaps) = 11
Alignment score = 0.000000
WB235_5:10450709-10450922 Satlength=214 Nr of Repeats=19 RepeatLength=11
seed=AGATACACTT Num.seqs=15 Similarity=0.983838
0   AGATACACTTC

****

Consensus:

AGATACACTTC

>WB235_Fam_398_11_1 Nr. of seq. 1 Alignment length(with gaps) = 11
Alignment score = 0.000000
WB235_5:18641364-18641663 Satlength=300 Nr of Repeats=5 RepeatLength=11
seed=TTTCAGGCCT Num.seqs=3 Similarity=0.796296
0   TTTCAGGCCTA

****
Consensus:

TTTCAGGCCTA

>WB235_Fam_399_10_1 Nr. of seq. 1 Alignment length(with gaps) = 10
Alignment score = 0.000000
WB235_1:13968489-13968559 Satlength=71 Nr of Repeats=6 RepeatLength=10
seed=GATTTTCATA Num.seqs=5 Similarity=1.000000
0   GATTTTCATA

****
Consensus:

GATTTTCATA

>WB235_Fam_400_10_1 Nr. of seq. 1 Alignment length(with gaps) = 10
Alignment score = 0.000000
WB235_2:2279378-2279438 Satlength=61 Nr of Repeats=4 RepeatLength=10
seed=CAGCAGAGTG Num.seqs=3 Similarity=1.000000
0   CAGCAGAGTG

****
Consensus:

CAGCAGAGTG

>WB235_Fam_401_10_1 Nr. of seq. 1 Alignment length(with gaps) = 10
Alignment score = 0.000000
WB235_2:3145088-3145138 Satlength=51 Nr of Repeats=4 RepeatLength=10
seed=AACTTTCGAA Num.seqs=3 Similarity=1.000000
0   AACTTTCGAA

****
Consensus:

AACTTTCGAA

>WB235_Fam_402_10_1 Nr. of seq. 1 Alignment length(with gaps) = 10
Alignment score = 0.000000
WB235_5:19495245-19495315 Satlength=71 Nr of Repeats=5 RepeatLength=10
seed=TGGCGAAAGC Num.seqs=3 Similarity=1.000000
12   GCTGGCGAAA

****
Consensus:

TGGCGAAAGC
