# Supplementary material for: Unique Features of Satellite DNA Transcription in Different Tissues of Caenorhabditis elegans
Source: Int J Mol Sci. 2023 Feb 3;24(3):2970. doi: 10.3390/ijms24032970 (PMC9918286; doi:10.3390/ijms24032970)
Supplement: Supplementary file 1 [file ijms-24-02970-s001.zip › Table S3. List of WB235 satellite families.pdf]

Genome	Family name	Number of sequences	Alignment length	Score	Consensus without gaps
WB235	WB235_Fam_1_35_172	172	44	0.653800	AAtTgAAaATTTCCGGCAAATCGGCAAaTTGCCGg
WB235	WB235_Fam_2_12_169	169	14	0.769942	AGGCTTAGGCTT
WB235	WB235_Fam_3_15_136	136	15	0.942162	AGACCCaTCGTGGTG
WB235	WB235_Fam_4_35_115	115	45	0.601883	ATTtcCCGCCAAAAtntTTTTCaCaGAAAATTTgA
WB235	WB235_Fam_5_40_94	94	41	0.668164	AannTTCTnGAAcaTTCcaGAAttTTCtnGAaTTTTCcaG
WB235	WB235_Fam_6_11_75	75	12	0.809099	GTACtcCTACA
WB235	WB235_Fam_7_15_70	70	15	0.826961	cTCCCCTTTTCGtAG
WB235	WB235_Fam_8_16_50	50	16	0.867364	CTACaTGCCTACaTGC
WB235	WB235_Fam_9_20_46	46	20	0.797488	TGttAcACGaAAAAATAGAT
WB235	WB235_Fam_10_25_40	40	28	0.748245	TTTACTCTCTGGCTTCaCantATAT
WB235	WB235_Fam_11_20_29	29	25	0.605057	tATtGATTTTTcTGnAAATT
WB235	WB235_Fam_12_45_28	28	46	0.807319	AAGGATCAGAnCAtCAGgAGGTACCCtTgTGATCCTTCGgAgCTG
WB235	WB235_Fam_13_43_26	26	47	0.705990	TTTGCCGGAAATTTTcAaTTcCGGCAAtTTGCCGgTTTGCCGA
WB235	WB235_Fam_14_11_23	23	12	0.800725	TTTTGCACATT
WB235	WB235_Fam_15_31_22	22	33	0.785911	TAAATAtTTAGCAGACCAaAgtTgGGTaTGC
WB235	WB235_Fam_16_26_20	20	26	0.760324	cAGAGTCActatTTTtGGTGAAcGGc
WB235	WB235_Fam_17_37_19	19	43	0.743370	GGGAATTCAAATTTTAATTTTTtGAAAAtATTTTGGC
WB235	WB235_Fam_18_19_17	17	19	0.859649	CAAGGCACtAGAAtTGCGc
WB235	WB235_Fam_19_14_17	17	14	0.892682	ACTACAaACTACAA
WB235	WB235_Fam_20_12_15	15	14	0.745805	TGTATATGTATA
WB235	WB235_Fam_21_18_14	14	18	0.786121	TGCAAgACTAaTAGaGac
WB235	WB235_Fam_22_59_11	11	62	0.794330	AAAACCAGTGCACATTTgAAATTCCATATTcTCCATAATTCTCaGTTTaAAAAaTTTTG
WB235	WB235_Fam_23_32_11	11	35	0.789437	CTACAAACTACAAnTTTTGGGCGCTGCTaAgA
WB235	WB235_Fam_24_20_11	11	21	0.822222	CtAGGCAGACCTAGCCTACC
WB235	WB235_Fam_25_12_11	11	12	0.746970	ACgCGacACGCA
WB235	WB235_Fam_26_26_10	10	27	0.768999	TTAGAcCatTAGATGAATTtccAAAG
WB235	WB235_Fam_27_21_10	10	23	0.611433	ATGCACTGACCAAaanTatga
WB235	WB235_Fam_28_163_9	9	170	0.739924	ATCATATggAGTcATTCTTTTTTaTTTcATAAAACTGTTCAGcATAGtCAAAaaTACCAGAAaATgCcAAaCAAAGTATanTAGCTTGTACGGAAGTattTTTTTAAAAATtgAtaaAAATatAtAAAAGCTGaTtTTTTCAAAAATTCAAAAGTAngggAAA
WB235	WB235_Fam_29_42_9	9	42	0.733686	CngaAAATTTcgaaAACCGGCAATTGCCgAAaTTGCCGAntg
WB235	WB235_Fam_30_31_9	9	37	0.708709	ATTTCCCGCCAAAAAtTgaCTGAAAATTTGA
WB235	WB235_Fam_31_26_9	9	26	0.798789	CATTgCGGCgGATTttAgaAAAaTGG
WB235	WB235_Fam_32_18_9	9	23	0.628019	cAAAAATCaATAATTTCa
WB235	WB235_Fam_33_18_9	9	19	0.838207	ATTAGCAGCGcCCAAAAa
WB235	WB235_Fam_34_12_9	9	15	0.680247	CTATACCTATAc
WB235	WB235_Fam_35_14_9	9	14	0.904762	GCTATaCGCTATaC
WB235	WB235_Fam_36_12_9	9	12	0.876543	TCTAaGTCTAaG
WB235	WB235_Fam_37_21_8	8	23	0.616977	CAAaTTTTgAcnTAAAAATnT
WB235	WB235_Fam_38_18_8	8	19	0.681391	TAAGCCTAAGcCTaAgCc
WB235	WB235_Fam_39_10_8	8	11	0.758117	GCGgtAGCGG
WB235	WB235_Fam_40_166_7	7	173	0.741077	tAtcAAtTTTcAGacGCGCTAgaGgGGTTTTTGTcTtGAaTTCCAATTTcaACagntatannAGGCnCAAAAAAACAAAAATAAAAaTTtTGGAaTTtnGGAnAATTTTTATAGATTTGgATTCTAGATTnTTTAAAAATATTaTTGGTAaGTTTtTGgaCCaagA
WB235	WB235_Fam_41_95_7	7	96	0.868386	AGGGCCGCAGGCCCGAAtACTCtTGGAAACTatGGGCtgGCTTTaATCCTATTCcAAGCAGATTGTTATTCaCTCAAGCCTAnnAACCAGGTTtG

WB235	WB235_Fam_42_94_7	7	94	0.948666	
TCAAAATCTTCTGTTCTCTAAATATTGGGTGCTTTTCGATGCCCTaTGTAGACAAATCAATGGGAAAATcaTCAATTTCTGaAGGCAGTAATTC					
WB235	WB235_Fam_43_19_7	7	23	0.690821	TGGTTGAGGCTCtgGATTT
WB235	WB235_Fam_44_226_6	6	227	0.831816	
GTAACTACGTGAAGCTTCTTGTACCTACTGaAATATTGTTaaTATATTCtgAAAGAgCTTAAAATTTACTACaAAGTTTCTGAAAGACATTTcaAAAAAAgTTACTTTGAAGAGATTTATGAATTTTCAAAgTTt					
cgAAAATtCatCTAtTTTTGcCTATAAATGGTAGTTTTCATTaAATAATAAAATACGCACTAAAAGGTAGGGCTGAAAATGTACTATTTCT					
WB235	WB235_Fam_45_33_6	6	33	0.649832	GCTGATTGGtTcAgAAAgTGGGCGgaGCtaAtC
WB235	WB235_Fam_46_32_6	6	32	0.838889	TGTCgAGCGAGAcAGCAGAagAAGAGCTGAGT
WB235	WB235_Fam_47_21_6	6	25	0.624889	CTGACcAAATTtGTTcCaGCA
WB235	WB235_Fam_48_21_6	6	22	0.621212	TGACaAAAtnTnaaTTTGCAC
WB235	WB235_Fam_49_19_6	6	21	0.706878	gaAATTATCGATTTTtCga
WB235	WB235_Fam_50_18_6	6	19	0.644444	ngGTAGgCAATGAGCAAg
WB235	WB235_Fam_51_16_6	6	16	0.697917	TCtaAGGATCTaAGGA
WB235	WB235_Fam_52_15_6	6	15	0.952593	GGGGGCAACAAAAtA
WB235	WB235_Fam_53_12_6	6	14	0.649206	TTTTtATTTTTA
WB235	WB235_Fam_54_11_6	6	11	0.838384	aAGTTGTCACg
WB235	WB235_Fam_55_48_5	5	48	0.900000	AATTtAatTtTTGAtTTGCCCGTAATTTATAttTTTCTGAAGATGAGc
WB235	WB235_Fam_56_21_5	5	25	0.616000	ATAaatatTAAtTAAaTAAat
WB235	WB235_Fam_57_25_5	5	25	0.946667	TATTTACTCTAATGTTCTGCcAATt
WB235	WB235_Fam_58_21_5	5	23	0.667391	CcATTTTaAGcCAATTTTtngA
WB235	WB235_Fam_59_22_5	5	22	0.630303	GCACTGACCAantnnAatnTTt
WB235	WB235_Fam_60_19_5	5	21	0.695238	AAtTTacCAATTTAcCAAa
WB235	WB235_Fam_61_20_5	5	21	0.652381	GTAAATCTACAntggCttcc
WB235	WB235_Fam_62_17_5	5	18	0.848148	TcATTCCGTgATTATTT
WB235	WB235_Fam_63_11_5	5	14	0.697619	TGCCTgCCTAC
WB235	WB235_Fam_64_11_5	5	11	0.951515	TTTGGAGCGGt
WB235	WB235_Fam_65_184_4	4	189	0.862140	
GAATACAGAACACCAaTTATGtCCGAGAAAAAGATCCtAcTCAGAATATaAACATAtTcGAATttatCGGAtGTATAaAGATTCCCGAAGAcaCTTTCCAATTAcCCAAAttGTTCAtATTCTAAAtgAaATTCt					
CTTACTaGAACACTCTTGGCCAATGTACGCAGCCGAACGTATCATAAGT					
WB235	WB235_Fam_66_64_4	4	67	0.721393	
gGTCTGCtAaATCaAAacTTGTAGTTTGTAGtCTAGCAGACCaAatTTcTtcAAaACcaCaTtG					
WB235	WB235_Fam_67_39_4	4	40	0.856944	TAtTTTTAAttTgaCCTaCTTAAAAAGTAGGTCATGACC
WB235	WB235_Fam_68_36_4	4	36	0.754630	tTTCGGCAAtTTcGacAATTGCCGGTTTtggaAAtt
WB235	WB235_Fam_69_30_4	4	30	0.777778	TTTCtAGAaaaaTcTGGAAagTTCTAGAAt
WB235	WB235_Fam_70_30_4	4	30	0.983333	AATTTTCCAGAAGGTTCTGGAACaTTCCAG
WB235	WB235_Fam_71_21_4	4	21	0.907407	AATCCAATATGGacgAAATCT
WB235	WB235_Fam_72_19_4	4	20	0.761111	CTCAACCAAaAcTcTtAGC
WB235	WB235_Fam_73_20_4	4	20	0.744444	gTgAAAaTTtaAATTTtTgA
WB235	WB235_Fam_74_17_4	4	18	0.902778	GATGCCCCCTcTGAACG
WB235	WB235_Fam_75_16_4	4	16	0.847222	AATTTTTCcGCaGCaA
WB235	WB235_Fam_76_14_4	4	15	0.727778	TTaGGGCcTtgGGC
WB235	WB235_Fam_77_15_4	4	15	0.955556	TTTCCGGATCTACGt
WB235	WB235_Fam_78_13_4	4	13	0.897436	GCCTATGGCcTAc
WB235	WB235_Fam_79_12_4	4	12	0.840278	TTTTtCCGAAat
WB235	WB235_Fam_80_12_4	4	12	0.662037	AgaAgGAGgAaa
WB235	WB235_Fam_81_200_3	3	223	0.702043	
gAAAaAGTgAAtTTTCAGtCAtACcTTgTggAccACaATTTATaTGCAAAATTAaTAGATTTTCCAAacTTTgAAAATTAATtTCTGGCTTcAAAcTAACTTTTTTaaAAATtCCCGTAGCCATTTTGTaGCAAA					
TTTTatGtTCTtTCAGAATTTaAAAACgGcATTtCAAAAAaTTTnaaAaCcgCCAAGTTCAAGCA					

WB235 WB235_Fam_82_167_3 3 172 0.656977
nTATAATGcTCGTAATTATTtATAAGTgTGTGTTGTCTCAGAgcCAngTCTgAAAAaACAGATTTTTTtTcGAAAaTTTtAAaAacttcGtAaAAATTcgATTTTtGACanaTctATgnTcTCTnTTTTTtTnta
AcATgTAtTTCAGTGAcAtAnaganTcAaAAa
WB235 WB235_Fam_83_90_3 3 95 0.673684
ccccGAACGTGTCGGCCGCtTCCAAAtAACcACCTccTngCACTaCgTTGCGCACACAcnaanctaCTcgTTTCACGcCAAgCTgCGGAA
WB235 WB235_Fam_84_79_3 3 79 0.912799
gTGAATCTCCtCTCtaCCTAATGAgTCCTGACAGAATACCGGCAGTATTcTGGGGTCTCATTAGGTTTTaGTTTCTTcT
WB235 WB235_Fam_85_76_3 3 76 0.906433
AATGCTAGAgGACAcTCCTTTGCACGAAaGgAAGTGGGAGAGACTGTGGaGTTGTCGGTGTCTtTGTgCATGCTCg
WB235 WB235_Fam_86_66_3 3 68 0.892157
AATCAACCTATTCAGTTGACAATGGTTTTCTTTTAtCAAAtTTtTTAaAATTTtAAAGAAAACACA
WB235 WB235_Fam_87_64_3 3 64 0.954861
TTTTTGACTACtGAATGTCTTTTTCGCTACTGACTGaAAAAATAGCTACATTCGCACTTTTGtC
WB235 WB235_Fam_88_34_3 3 34 0.980392 ATGCACCATGTCAAAAAATATATCCAAAAgTACG
WB235 WB235_Fam_89_32_3 3 32 0.972222 CGACCTGCGACCTAGCGACCTaGGTCGCTTTG
WB235 WB235_Fam_90_27_3 3 27 0.658436 aGCaGCCGACACCTTaCnGGTtgcttA
WB235 WB235_Fam_91_26_3 3 26 0.965812 TCAgGGCGAAACGGGAGGTGAATCGC
WB235 WB235_Fam_92_21_3 3 23 0.801932 CTAgAAAAACTCGGCCAtCAA
WB235 WB235_Fam_93_21_3 3 23 0.608696 gCCtAAAAAcgtaAATTTTAa
WB235 WB235_Fam_94_21_3 3 23 0.748792 TAGTTTaTAGTTTGtaGTTTG
WB235 WB235_Fam_95_20_3 3 22 0.656566 GTCGATTTACGgGAgTTcnT
WB235 WB235_Fam_96_22_3 3 22 0.777778 AcTACAGTAccCcTACAgTACc
WB235 WB235_Fam_97_19_3 3 21 0.608466 GCCTAGaAATCtnAtgATG
WB235 WB235_Fam_98_18_3 3 21 0.761905 TTTTGTCAGTGCTATAgT
WB235 WB235_Fam_99_21_3 3 21 0.767196 ACTACcGTACCaaCtACcACa
WB235 WB235_Fam_100_21_3 3 21 0.957672 CTAACATTTTTCAaTTTTCGT
WB235 WB235_Fam_101_21_3 3 21 0.682540 GTgcaaatgnAtnnttGgTca
WB235 WB235_Fam_102_20_3 3 20 0.688889 GTAAATCtACAtAgaaCntC
WB235 WB235_Fam_103_20_3 3 20 0.777778 CGAtAtAGCTATtaACGAtA
WB235 WB235_Fam_104_16_3 3 16 0.777778 TTcCAGGgTTcCAGGg
WB235 WB235_Fam_105_11_3 3 12 0.787037 ATTTGgAATAT
WB235 WB235_Fam_106_11_3 3 12 0.722222 ncCACTGACCA
WB235 WB235_Fam_107_10_3 3 10 0.733333 TCcATCccGA
WB235 WB235_Fam_108_177_2 2 177 1.000000
GAGTCTCACGATGTGTCAACGTGTCTCAGTCGGCTTGATCTACGTAGATCTACAAAAAATGCGGGAGAAGAGACGCAGACTTATTCTCTGATTTTGCATGAGTAAGAGCGTGCTGGCGTCACATTTTTGTCGGCA
AAAAATTCCCGCATTTTTTGTAGATCAAACCGTAATGGGACG
WB235 WB235_Fam_109_151_2 2 151 0.924945
TTTAAAGGCGCATAGGTAGtTCAAANTTCAAANTTGACGGATTCTAGTCAATATGGGGCTTATTTTAAAGCTCTCAGTAAGCTNAATNTANTTNAGCTACTaAaAATTGAANATTTTNAAATTTTTTGGGTCTCG
CAGCGAAAATTTCGAA
WB235 WB235_Fam_110_119_2 2 119 0.619048
GCcacCcTTAAcCCTAAcCCAaGCAGAGCAGGaTGTAGGCCTGaaAcGcGGTaGcgAcaCaaTCAaGCCcAAcAacCaGGgaTGAGGGCCGCAGGCCCgaaaACTCTTaGAAACTgTaG
WB235 WB235_Fam_111_110_2 2 110 0.745455
TTTCACaTTTTCAcgTGATTATaTAGGTCACGAaCcAAaTTATTTTGAAAATTGAcCTacacGGacCTaTAACTTTGGAGaTATGGGaAATcaAGTATGaGCTTTCAGAT
WB235 WB235_Fam_112_86_2 2 86 0.689922
TGTATTTTGAAAGGTCaTcgCAATAggGAAAAGATGGAAGAcTaCgaAGttAAcgAgAAgCTATTGacAGACCCCGAAATACTGGC
WB235 WB235_Fam_113_73_2 2 73 0.945205
AATAAAATATTGGAAATCCcTATGACACACTTAAGCCTAAAGGCCCGAAAAAaAaACTAGGATGCCCAACTGG
WB235 WB235_Fam_114_44_2 2 44 0.681818 cCAGCCGACAGCCGAAAaaTTcacGaATTTTCGGCTGGTaGCac
WB235 WB235_Fam_115_44_2 2 44 0.704545 aCGGCAAAtTCGGCAAaTnCTgcTTTTTTcAAtATTTGCCGAGC
WB235 WB235_Fam_116_44_2 2 44 0.969697 CTGTTTTCTGAGCATTgTAGATTTTCGGATTGATGGCCGAAAGG

WB235	WB235_Fam_117_41_2	2	41	0.711382	ccGTTTCGCcTTGAGCGATTcCttACCCTTAAATGGGCccC
WB235	WB235_Fam_118_41_2	2	41	1.000000	AAAACGGGAAAACGGGAATTCCCGTTCCCGTGAAAATTTTA
WB235	WB235_Fam_119_40_2	2	40	0.975000	AAAATCCACGTAGATTATTGATTTTTCATgGAATTTTGCA
WB235	WB235_Fam_120_39_2	2	39	0.897436	TTATGaAAAAGTGGCTCTAGaGCCACaAGAAATACCAGT
WB235	WB235_Fam_121_36_2	2	36	0.601852	cGGCAaAtTaCCaTaAATTTTaAAATcCGGCAaAAc
WB235	WB235_Fam_122_34_2	2	34	0.612745	cCaCCCGGcAGGTGTCGGCTGCTaaaaCTAtAaC
WB235	WB235_Fam_123_32_2	2	32	0.671875	tTTTTTGAGCcaAAAATTCtaAAAAATgCTCa
WB235	WB235_Fam_124_32_2	2	32	1.000000	GCACCAAATAGCACCAATTTAGCACCGATGGA
WB235	WB235_Fam_125_31_2	2	31	0.752688	GGTTACTGTAaaTCaATcaTAGGATACTTtG
WB235	WB235_Fam_126_31_2	2	31	0.784946	CTGAAATTacaAaTTTCCAaTGTAAAAATGT
WB235	WB235_Fam_127_31_2	2	31	0.956989	TCGACAGAAGCTGTCGGAaATCACACTTTCC
WB235	WB235_Fam_128_29_2	2	29	1.000000	AAGGATTTCCCACTATGTCAATACATTGT
WB235	WB235_Fam_129_27_2	2	27	0.901235	GCTGCTGGAGGATCaACTGCATCAACc
WB235	WB235_Fam_130_26_2	2	26	1.000000	CAAAACCGGCAATTGCCGAAAATTCC
WB235	WB235_Fam_131_24_2	2	24	0.722222	gGCATCTGccGCcGGAGCTGGaGC
WB235	WB235_Fam_132_24_2	2	24	0.680556	GCcTAGaCTcaGGCTTAGGCTtAa
WB235	WB235_Fam_133_23_2	2	23	0.630435	aATTTCTAGGCCACcAcgTTcAg
WB235	WB235_Fam_134_23_2	2	23	0.673913	tCTAATTAGATnCaCCtTcTTTT
WB235	WB235_Fam_135_23_2	2	23	0.659420	ATTaaATTTTTCAaCaaAAAAAa
WB235	WB235_Fam_136_22_2	2	22	0.643939	aATAAAAATgTCAcAAAAtATg
WB235	WB235_Fam_137_22_2	2	22	0.681818	GTAAATCTACAcaaATagCCCC
WB235	WB235_Fam_138_22_2	2	22	0.621212	TTcaAGCcaTTTTCaGACcAAT
WB235	WB235_Fam_139_22_2	2	22	1.000000	TCGAGAAAAAATCTGAAAAAAT
WB235	WB235_Fam_140_22_2	2	22	0.954545	ATTCTGCGCACCCCCTGCGCaG
WB235	WB235_Fam_141_20_2	2	20	0.933333	CCACCAAAAAgGTTTCTAGG
WB235	WB235_Fam_142_20_2	2	20	0.950000	GGACTAGTTTTcGAGTAGAG
WB235	WB235_Fam_143_20_2	2	20	0.866667	CCCCGAaGCTCGAGCTCAAa
WB235	WB235_Fam_144_20_2	2	20	0.800000	CCACCCTGTAaaATcATGGA
WB235	WB235_Fam_145_19_2	2	19	0.684211	CTAGGTGCCTAcTccATgC
WB235	WB235_Fam_146_19_2	2	19	0.929825	CGCCAAGgATCGAGAAAAG
WB235	WB235_Fam_147_17_2	2	17	0.656863	gCaGaTTGCCgGAATTG
WB235	WB235_Fam_148_17_2	2	17	0.803922	GCACTGAcCAAcTTTTT
WB235	WB235_Fam_149_16_2	2	16	0.750000	GTaCTGGGGTaCTGGG
WB235	WB235_Fam_150_15_2	2	15	0.622222	CGATgCTCCACCaga
WB235	WB235_Fam_151_14_2	2	14	0.761905	TaTACAAAAAnGTA
WB235	WB235_Fam_152_14_2	2	14	0.666667	cCTCAAGCgTAGGa
WB235	WB235_Fam_153_14_2	2	14	0.619048	gATAATTgAaAATc
WB235	WB235_Fam_154_11_2	2	11	0.878788	AAACTTGGGCa
WB235	WB235_Fam_155_11_2	2	11	0.636364	cGGAaAATTaA
WB235	WB235_Fam_156_11_2	2	11	0.636364	cATAaCTGAGa
WB235	WB235_Fam_157_255_1	1	255	0.000000	AACAAGTCGGGAACGTTCGGAAAGCTCTGAGGAAAACTCCGACAATATCATGCTTAAGTACGCAATTGAGCTATCTTTATTAGATTCGAAAAAGGACTCTTCAAAAATTGAAGCAATAACGAAGAAGCTTAAGTTGGAACTGGCGAAGCTTAAATTGGGANTTATGCAGTCTTTATCAGATTCAAAAGACGATTCTTCGACCAATGAAGCAAGTCCGGAGNACGATCTACCTTCGACTTCTGATGGCAGAACTCC
WB235	WB235_Fam_158_230_1	1	230	0.000000	GCTGAATTCAGTTGGGTANGATGTGACTTCATGGCTGAGTGGAATATCTTGAAGTTATCAGCACGATGTGTAGAGTCGAGTGGTCCATCGTATTGTTCAGAAATTGGATAATCAAGATGAGATGGAGATTTCTTGAACAATCCAGTGATCTTCGATTTCAATGATGGCTCGTCCTTCTTCTCTGTCTCTTCAATCACAACTGGGATGACATGCTCTGGCAGCTTCTCATA
WB235	WB235_Fam_159_228_1	1	228	0.000000	CCACCAGATCAGCAACGTCTCATCTTCGCCGGAAAGCAACTCGAGGACGGCCGCACCCTTTCGGACTACAACATCCAGAAGGAATCAACTCTTCATTTGGTTCTCCGTCTGAGAGGAGGTATGCAGATCTTCGTCAAGACATTGACCGGAAAGACCATCACCCTCGAAGTCGAAGCCTCCGACACCATCGAAAATGTCAAGGCCAAGATCCAAGACAAGGAAGGAATC

WB235 WB235_Fam_160_189_1 1 189 0.000000
TTTTTTTTGACTATGGAATTATAAAGAAAATCGAATTTCAAAAAAGCATGTATTAGCCAATGTAAACGCGCTCCAATGATGATGTAGCCCACGTGGTATCAGAGTGTCTCTTTACGGCTTGATTTAAATACGTAG
ATCTACAAATTCACTTATCTGTTTTCTCACGGGTTTAGGATATTTTTTCTGTTA
WB235 WB235_Fam_161_184_1 1 184 0.000000
GTTCAAAAGATACAAAATTTATCTACACATCTACAAGTTAAACTTGCAACTTAGTTAACCAAATTCAAAAGTTATGAGCCGGGGAACTATAATAACCAACGTTTCACACTAAAAAAGCCCAAAAAAAATTCGAAAA
TCATTTTTATCTCATGACGCGGACAAGCTACAAATGTCTAGTTAGGCTT
WB235 WB235_Fam_162_183_1 1 183 0.000000
TTTCTCTGCGTCTCGCCTCGCCTCACACTATTTTNCACACCAAACTTTGAGNCTATGTATCTCTGTAACGGGTTGCGCTTTCTGAAAAATTCCAACTGACGGGATATATAAAATTTNGAATTCTTTCTCACCGTT
AAAAAACTAAACACCTAGGCAAGACGCAGACAGCGAGANACCCCCAAT
WB235 WB235_Fam_163_181_1 1 181 0.000000
TTTTTAACTTTTAAATCCACTAAAACTCGGAATGGTACAGAACATGCAAATTGTAATTTCAAGAACTGAATGTGGTTTCGAATATTTATGGATTGCTTTATTCTAAAACTGACTCTGAAATATTTTGCATCCATT
TGACTACTAATTGTCATATTATACCGAACACTACTAACTTTAGTGG
WB235 WB235_Fam_164_180_1 1 180 0.000000
TGTCGGCCGCTGTATCTTTTGCCTCAATTTTTTTTNAATTTTNAAACATNCATATTCCAGTAGATAACATTATGAGCTACAGTTTGTTAGTTAAAATTTTATTTCTAAAATTACTAGTTTCTGAGTTATACTCAT
TCCAACCCACCCCAAGAGAAATTTCCTAAAAATGAACTCGCGAGG
WB235 WB235_Fam_165_179_1 1 179 0.000000
CTACCCAAATTTTTGAAATTTGGAAATTTTTCAAAAATTCTCAAATTTTCAAAATCCCATAATTCAGCCAATTTTTGAGATTTTCAGATATTTTTTAATTGAAAATTCTGTTTGTCTTATCCATTAAACTATATC
GCTGCATCTACATTTTTCACAGTACCCCATGACTTTTCCCGATT
WB235 WB235_Fam_166_177_1 1 177 0.000000
CTAACAAAATAATCAGCGTAAAATTTCCTACATTTTACTAGTTAACAATATTTTGATAACATCAGTGGTTTTTGAGATACGCAAGTTTTAAGGTGGACGCCCAAAAATAGTTGTGAGCAGCTTTAATCAAAAACC
GACATAACTTTTTACTCACTAACGGTATCAAAAATGTTTTAA
WB235 WB235_Fam_167_175_1 1 175 0.000000
GATGCACCATGTCTTTGACAGTCAATATCTCGGTTTATTTTGTAGCTATCAAAAAATTTTCAACTACAAATCTATAGAAAATTAAATGTTCAACATTTTCTCAGTTGACAGTTTTTTGATAACTGCAATGACATC
CGAGATATGAGCTTTCAAAGTTGAGAGACCTCACTCGGTT
WB235 WB235_Fam_168_175_1 1 175 0.000000
AACTTGTGACGGTGGGTATGGATTTTATGCACATAATTTGTTCGAAATAGTCTACCNAGTAAACTGGTGAAGTTTCAAAAACTTTGTGGATCTAGTTTTTGAAAAAAGTTCAAAATTGTGCCAATTTTGACCAAT
TTTGGTGGTTTTCTGAGCTAAAATTGAAAAGTTGGGAATG
WB235 WB235_Fam_169_175_1 1 175 0.000000
AAATTTTGGGTTCACTCTTTTTACTAGACAGTGATTGTTGCATTGTAGGATTCGGTGGACTTGTCCAACAACTTACTTTGGCATGAGTTTAATAAAAAGGTTTTACCATGAACTGAATCAATTCAAAATAAAAAC
CTCGAGAATGAAAACATCCTCAGTAATTTCAATCCTGTTC
WB235 WB235_Fam_170_174_1 1 174 0.000000
CTTCAAGAGCTCAGTGTTATTTATGACATGCCAACCTCGACAATTGACCTGAACATGCTCCAAGGAATCGAAGACAACTTGAACAGCCTTCCAGCTGAAGAATCTGATAAGATCCGAGAGAAGATTAACGATCTG
AGAAGAAGGAAACAAGAAAGCGATCAAGCTGAAGCTCTT
WB235 WB235_Fam_171_165_1 1 165 0.000000
CTTTTCCAAAATGGCAATTTTTGATGAAAAAAATCCGAATTTTCACAAATTTCAAAAAAATTTTTAAACTTTATAATTTTAGCTTTGAATATTCCAAATGGAACTGTCATAACCTATCAATTTGATATATAAACT
CGTGCCTACCTTCAGGCATTCAGTTTTCAC
WB235 WB235_Fam_172_155_1 1 155 0.000000
TCCCGCTGTGGTTAAAAGTTTTTTGCGTGCTCAGTTCTGTACGGTAAATGTGTTAAAAACTTCTGGAAACTTGAAATTCCAAGTTATTCAGAAAATTGTTCAAATGGCGCAGTGGGAAATTCGATTTCTCAGACC
CAAGAGGCCACGGGTTCAAT
WB235 WB235_Fam_173_153_1 1 153 0.000000
TGAGGTGCAGGGCGTGATGAGCTTTGTTGAACTGGGAACTGTTGATTCCCGGAGCCGTGATTCATAGATGAGTTGGAGGGACCTGCTGTGGAACTTGTTTCAACTTGAGCAATTCCATGAGCCATCAAGAAGCTT
TCCGGCAGATTGGGCAAC
WB235 WB235_Fam_174_151_1 1 151 0.000000
CATTTCAGATATTCCATCACCACCAACACCTTCATCCACTTCTTCGCCTGGAACGACGGGATTTTCTACAAAAATCGGATTTTTTCTCTATGTCAGTTTAATTGATGTATAATTCCGAAAAGTCAGTCTTTCCTA
GGTTTTTAAATTGAAA
WB235 WB235_Fam_175_150_1 1 150 0.000000
TTGCAACATGCGGTCGGCGGAAAATTCGAATTTTTCAGTCCAAAACAGGTTTTTCGACCAATTTTTCATATTTTCAAGCGAAATTATGATAGAAAATTCAAATTTTCACTAAAAATCGCCAATTTTCCACATTTT
CTCATTGGAGCGCAC

WB235 WB235_Fam_176_150_1 1 150 0.000000
TTGAGGGCCGCAGTCCCGAATACACGAAGAAGTGTTGGAGATCCGTCAAGCCTATTCTAAGCATAATAATTCAGTTCGAGTGTCGCAAGCCCAAATAGACGCAGAAGCGTTGGACAACTTTTAGGCCTATTCTGA
GCCCAATAATTCATG
WB235 WB235_Fam_177_146_1 1 146 0.000000
TGGCCTAGAAATCNCAAGTTTGCAAAAGTTAGGCCACCAACTTCAAATGCCTATAACTCAGCGGAAAATCAATATTTTCCATTGATTTTAACTGCTAAGATGTAGCCAATTACGTAGCGCAACTTTTTGGCCTAC
TTAACAATTGG
WB235 WB235_Fam_178_144_1 1 144 0.000000
TTCTAGGCCACCAATTGGAAATCATCATAACTTGGCTTAAAATCCAGATTTTTGGAGTCTGGTAACTGCAACATGTTGCCCTGTAGTTTTTCTACAAGTTAGGATATATTAATTTTTGGTGGCCTAAAAGTCCAA
ATTATGGAT
WB235 WB235_Fam_179_143_1 1 143 0.000000
CAGTGGGATTTTCAATGTCTTCCAATCAAACGTCCGGGGTTCGAGTCCGCACAGGGGTATTTATTTTTTGCAAGCTGTGATTAACAAGTTTATAGCTGCAAAAGTCATTTTAATCGAACTTTCAGGAAAATGTCC
GAATGGCG
WB235 WB235_Fam_180_122_1 1 122 0.000000
GATCTACAAAAATGCGGGAAAAGTGACACAGAGTTCTCAACTGATTTTGCATAAAAATTCCCGCATTTTTGGAGATCAAACCGTGATGGGACAGCCTGGCACCACTTGCTTGATCTACGTA
WB235 WB235_Fam_181_121_1 1 121 0.000000
AAAAGATAGTTGCCCTAAGAGCAGAAGGTTGCAAACCGCAAGAGCAAACTTGCCAAATGACAAGCCACCCAAGTACTCGTTGCACCTACCTATCATAAGCTCGTAGACTATAATTTCTCTG
WB235 WB235_Fam_182_117_1 1 117 0.000000
TGTTGGTTCGACAGTAGGTGCACTAGTCTCTGGCACTGGAACATCCTTTTGCTCAACAATTTCAGCTGGTTGTACTTCAGATGTTTCTTTTGATTCCACAGGTGCAAGTTTCTCAAC
WB235 WB235_Fam_183_114_1 1 114 0.000000
GTGTTTCAGAATTACATGGAGTCTCACTACTTGCAGAATTTGTGGCTGTAGACGATGGGCCTGCTGTAGTTGAATCGTTTGGAACTGTCATAGTGGATGTTCCATCAGACGTTT
WB235 WB235_Fam_184_108_1 1 108 0.000000
ATCCTCAACACCATCNGCGTCATCTAGTTCCTCCGGAACTATGTCGACAATTTCAGGATCAACTGGTTCNACAGTTACAGTTGTACCAGGATCNTCTAGCACATTTGG
WB235 WB235_Fam_185_108_1 1 108 0.000000
TGGGTAATCAGGTACGGGCTTTCAGATTTTCAAGTTTTCACGTGAATATCTAGGTCACGAGCCAATTTCAGCCAGGAGTGGGACTAGAGATATGAAACTCTTGGGTTA
WB235 WB235_Fam_186_108_1 1 108 0.000000
AAGTCTTCTTGATTCTGAGTTCTTGCTTCGAACTGTTGACCATCTCCTTGCAGGATCTTATTGTCAGATGGAACCTTCTTGGCATACCGTTCTCCACTGATGTGAGTG
WB235 WB235_Fam_187_104_1 1 104 0.000000
CCTGCGAAAGCGAATCTCTGGCCTGGCGGAGGGCCAACTAACTCTCTCCCTCAAGCCTTCGGGTGCTTCCTGGGTTATCGGTGAATTTTTGGGTCTGTCAGGGT
WB235 WB235_Fam_188_102_1 1 102 0.000000
TTGCTGCAGCATATCTTTCTCCAGTACCACCTTGATATGTCGAACGATTGATAGTTTGAGAATCAAGAGAATCTGTTGGACGATCAATTCCATCTTTGGGTC
WB235 WB235_Fam_189_102_1 1 102 0.000000
TGTACACTTCTCCCAAAATGACTGTAAGAATGGACTCCTTATATGATTTTAAACCCGACTCTGGACATACAAACTTCTCTCAAAAAGAAAATAGACTTCACA
WB235 WB235_Fam_190_101_1 1 101 0.000000
GCATTTGCATATNGAGATCAGATAAAAGTTAGAATTTTCCAGCCGTTGCAAGTGCGCTCTATTGAGAACATTTTGAAAATTGAATGTTTTCACATTAGAGC
WB235 WB235_Fam_191_101_1 1 101 0.000000
GGTTACTGTAGCTCCAGAAATACGCAAACACCGACTACGGTTCCCTAATACATCAGATATTACGACAAAGTGCAGAAAATCGCTAAAAATGACTTTACTGG
WB235 WB235_Fam_192_101_1 1 101 0.000000
TTGATTTTTCGTCTTATGAAGGAATATTGGTTTCGGGGTAGTGGGCGGATATGGTCGGGGTACTGTAGGTATACGGTAGGGTTACTGTAGTTTTGGAAATT
WB235 WB235_Fam_193_96_1 1 96 0.000000
GGTTGATTTGCGGCACTCTACAGATAGTGGATCTGCTTCTTACCAATCTTCCCGTTACCGCCACGCACTTAAGCTCCAAACTATTTTATTTTTTTG
WB235 WB235_Fam_194_96_1 1 96 0.000000
CTTCTGGTAATGTTCCCATAATTGGGTTAAATACCACTCATAAGTAACTAGTATGGGACTGAAAAGATACTAAATGAGCTTATTCTAAGGGTGAAG
WB235 WB235_Fam_195_93_1 1 93 0.000000
TGCTTCTCGTCGTCCACCACCACCACCGAAGGGAACTGGAACCCCACCACCACCACCAACTGGAGAACCACAGGATCTTTCTGGAGAAGGTAA
WB235 WB235_Fam_196_93_1 1 93 0.000000
CAGTACCCTCAAGGATCATCTGCTCCACGGTATCCCGGTAACTACAATGCCTTTGCCAGCACTTCCGATGCTCCACAGTATCCAGGATCCTCC
WB235 WB235_Fam_197_87_1 1 87 0.000000
TGAAGTCGTAGGATTCTGTGTGGTGACTGGTGTCGTTGTANTCTGTGAAGGGGACGTGGCTTCTTCCGTTGTGTCATGCTTCTNNGG
WB235 WB235_Fam_198_81_1 1 81 0.000000
TGTCGGCCGCTACCAAACTATTTAGCANACACACCAAGCACTACGTTGCGCACACACACCAAACTGCGGAACCCCGAACAG

WB235 WB235_Fam_199_80_1 1 80 0.000000
AATTTCTCAATAGGGCGCGCTTGCAACATCTGAACGGCGCGAAATTTGAATTTTTGGCCATAAAATAACGAAAACTAACT
WB235 WB235_Fam_200_78_1 1 78 0.000000
CGAAGTTGCTGGACCAAAGATCACAACAGGCGGATTTGGCGGCATAACCGGAGCAACAGTAGTGTCATTTGGTTCTTC
WB235 WB235_Fam_201_78_1 1 78 0.000000
TGCAAGGCTCCGATGATGATACCACTTCTAAAGTCGAGGTGTCACAATTTTCGGGTGTCGTAGAACTTGAATCAGTAC
WB235 WB235_Fam_202_78_1 1 78 0.000000
GAGCTTTGGGGCTTTTGGCTTGATCTCAGTCGACTTGATGGATTCTGGCTTGGGTTCTGCAGGCTTTTCGGGCTCCTT
WB235 WB235_Fam_203_77_1 1 77 0.000000
GGAAGCTTCATCTTTTGAATTATAAAAAAATAAATTTCATTTCAAAATCAGTGTGTGCACTATCAAAAGTGTCCACT
WB235 WB235_Fam_204_73_1 1 73 0.000000
ANTTTGTAGTTTGTAGTCTAGCAGACCAAAATTTATTAAAAACTGCAGAAAATTGATGGGTCTGCTAATTAAG
WB235 WB235_Fam_205_72_1 1 72 0.000000
TCCATCCAGCTGTGGTTGGTCTCTGGGTGAATCCTCGAGAAGTATCACTAGTAGTACCATTGGCTCCGTTCG
WB235 WB235_Fam_206_72_1 1 72 0.000000
ATGAATTTTAATACAATTTTCCTGACGATGAAGTCATAAAAATTCATTTTTTGACCTTTCAACTTTAGTTTT
WB235 WB235_Fam_207_72_1 1 72 0.000000
TGGTTGTGTTCCCAAAAGTGAGAAAAGAGGGTACAGACCTCTGTACGAATATGTCTTATCAGCTTCAAAAGA
WB235 WB235_Fam_208_71_1 1 71 0.000000
ATTTATGTGAAAATTCAGGTTTTTAGGGCATTTTTTGAGGGAAATCTGAATTTTTCCGATTTTTCTGACGA
WB235 WB235_Fam_209_70_1 1 70 0.000000
AAAAATCGATTTTTTGAAGTTTTTCTCAATAGAGCGCATTTTCAACAGCGAAAAAATTGAATTTTCCGCC
WB235 WB235_Fam_210_69_1 1 69 0.000000
AGGTAGGCCTGTAGGCATTGACTGNCAGAAAAGCGTAGACAGGCAAGCAGGTAAAAAGATACATGAGGC
WB235 WB235_Fam_211_68_1 1 68 0.000000
TTGGTTGAGGCTAGAAAATGTTGTAGTTTGTAGTGTGTTAGCCTCAACCAAAATTAATTTTTTTTTAA
WB235 WB235_Fam_212_68_1 1 68 0.000000
AATAGAGACGAATAAAAATTTAAAATTTTTATTAGTGTCTCTATTTTATAAAGAAAATACATTTTCAA
WB235 WB235_Fam_213_65_1 1 65 0.000000
TATCGAGAGAATNTTCCACATTCACTTTGAATTCCNTTTCCAATTTCAATAAGACTCTCAGTAAG
WB235 WB235_Fam_214_62_1 1 62 0.000000
GTAGTTTGTAGCTTTAGCCTCAACCAAATTAGCTAAAGCTTGAGTGAGGCTATGGTGATTTT
WB235 WB235_Fam_215_62_1 1 62 0.000000
TGCTAACTAGCTACGGCCAGCTTAGGTGCCGGCTGCACGAGCAGTCCTGGTTTAATGTCGGC
WB235 WB235_Fam_216_60_1 1 60 0.000000
CTAGCTAGATTAAACGCTCTCAAGAGACAGCATGTTGTTGTTGCCCACAAGAAGAAGGCT
WB235 WB235_Fam_217_60_1 1 60 0.000000
ATTCAGTTGAGCTCGTTATGTCGGTTGAAGACACAATTGGAGTGGTGCTTGAGGAGCTGG
WB235 WB235_Fam_218_60_1 1 60 0.000000
CTGCTGATTCCGAGAAGAAGGCCCCAGCCAAGAGAGAAGCTGATAAGCCAAAGACTGAAT
WB235 WB235_Fam_219_59_1 1 59 0.000000
CTACAAACTACAAAAGCCTATTTAGCAGACCCACGCCAACTTCTGATGGGTCTGCTAGA
WB235 WB235_Fam_220_57_1 1 57 0.000000
GAACTCAACAAGCTGATCAAGAGCAACACCGTGGATCCCGAGCTGCCGGACAGAAGG
WB235 WB235_Fam_221_55_1 1 55 0.000000
GGGATTATTGGAGTTTCTTTTTCTAGAATTTTTTGAAAATTTTGGACAGTTTTTT
WB235 WB235_Fam_222_55_1 1 55 0.000000
AAGAGTCCCCACACACTGAAAGGTTTTAAGGAATTTTTTGAGGATTCCTAGGGTT
WB235 WB235_Fam_223_52_1 1 52 0.000000
GCATGTCTTCCAAGCGTTTTGAGCCACGCNTGGGATTTCCTATACACGGCTG

WB235	WB235_Fam_224_51_1	1	51	0.000000	CCATCCGAAGATCCTCAACCATCTGGCCCACCATCTCCGGGACCAGTGGAC
WB235	WB235_Fam_225_50_1	1	50	0.000000	ACACATGCTATAACATTGTCTCCTTCCCCTAGTGCACGAGAGCGGCCGTT
WB235	WB235_Fam_226_50_1	1	50	0.000000	GGGCGTGTTTTCACATGCCCCGCCCACAAAATTGATGTTTCCACCAAAAT
WB235	WB235_Fam_227_49_1	1	49	0.000000	CAATAATTTTAAATTAAATTATCGATTTTTCTAAATTTTTCCGAAAAAT
WB235	WB235_Fam_228_49_1	1	49	0.000000	GCTACGATATCGTAGCACTTTTTTATTTTTCCATATTCCACCAAAAAAC
WB235	WB235_Fam_229_48_1	1	48	0.000000	TGCCTACGTGCCTACAAAACAGGCGTAGGCAGCTTTAAAGGTGATGCA
WB235	WB235_Fam_230_48_1	1	48	0.000000	GGAGGTGGAGAAAGTGTAGTTCCCGGTGGTCGNGGCTCTTCNGAGGGT
WB235	WB235_Fam_231_46_1	1	46	0.000000	TGGTAAATTGCCTCCCGGANCACCCACTTTTCTTATTTCCCAACTA
WB235	WB235_Fam_232_45_1	1	45	0.000000	ACCATCAGGCGGACCACCAGGACCATTTGATCCATCAGGAGCACC
WB235	WB235_Fam_233_45_1	1	45	0.000000	ACAACTGAATCAACTGCAGCACCAAGCACATCTACAGAGACACCT
WB235	WB235_Fam_234_45_1	1	45	0.000000	CCACCAGCACTGCTTCGAGTACGACAGAAAGCACTTCGACTCCAT
WB235	WB235_Fam_235_43_1	1	43	0.000000	CGAGTTTGGCCTACANCAAATTTGGCAAATTCGGCAAATTTGC
WB235	WB235_Fam_236_43_1	1	43	0.000000	GAAAACTAGGCCACAGAGTTCAAATTGATGGCCTAGAAATCTA
WB235	WB235_Fam_237_43_1	1	43	0.000000	GGCTCTCGGCTCGCGCCGAGAGCCGAGNTCTACTNAAAGTTTC
WB235	WB235_Fam_238_43_1	1	43	0.000000	AGCAGCCGACAGTTGCCGGTTGCCGAAAAATTGCAGTAATTTC
WB235	WB235_Fam_239_42_1	1	42	0.000000	TTTGATCTACAAAAAATGCGGGAATTTTCCCAGCATTGCTCA
WB235	WB235_Fam_240_42_1	1	42	0.000000	AGGTGCTGCTTCCTTTGGAACATCTGCTTGGGTTGGCTCCGA
WB235	WB235_Fam_241_41_1	1	41	0.000000	AAATTTTTAACGAGAAGTGGGCGAAAATCATTCAAAAATTC
WB235	WB235_Fam_242_41_1	1	41	0.000000	GCATTTTTCGCCGATTTTTNATTAATTTTCAGTGAAAATTC
WB235	WB235_Fam_243_40_1	1	40	0.000000	TCCACGTGGAATTTTCAGAATTTTCNCAGAAAATTTGAAA
WB235	WB235_Fam_244_40_1	1	40	0.000000	GAATTTTTTGCAATTCTACTGAATTTTCCAAATTTTCAGC
WB235	WB235_Fam_245_40_1	1	40	0.000000	TTTTGCCAAAAATNTGTCAATTTTGTGGAATTTCTNTCAT
WB235	WB235_Fam_246_39_1	1	39	0.000000	AATTTCCCGCCAAAATTGAATTTTTTTAAAGAAAATTTA
WB235	WB235_Fam_247_39_1	1	39	0.000000	CCAACTGGAGCCAGCACNATGACCGCTGTTGGAGGAGCA
WB235	WB235_Fam_248_39_1	1	39	0.000000	GATTTACGGGCTTTAATCAGTTTCGAAAAAGCTTGTGTC
WB235	WB235_Fam_249_39_1	1	39	0.000000	TGGTACGGTAGTTGTAGTGGTAGTGGAGGTTGTTGTTGG
WB235	WB235_Fam_250_39_1	1	39	0.000000	GTAATTTATAATTTTTTAAAGATGAGTAATATATTGCCC
WB235	WB235_Fam_251_39_1	1	39	0.000000	ATTGATACTTTGTCGGGTTTAGACCATCGGCCGAGCTTC
WB235	WB235_Fam_252_38_1	1	38	0.000000	AAAATCGATAATTTTCAGTATGCAGTGAAATTCTTGGA
WB235	WB235_Fam_253_37_1	1	37	0.000000	ATAATTTTTCTTGAGTAATGAGAGACTTTTTCATAAT
WB235	WB235_Fam_254_36_1	1	36	0.000000	GATATTTTCATGTTCGGCAAAAGTACCGATTTGCCA
WB235	WB235_Fam_255_36_1	1	36	0.000000	CCACCAACTCCAAAATCAGCTGCTCCATCAGAAGTC
WB235	WB235_Fam_256_35_1	1	35	0.000000	AATTGCCGAATTTCTCGTTTTTGGCAATTTACCAA
WB235	WB235_Fam_257_35_1	1	35	0.000000	TGTGTAATAAGAAAATTTCAAATTTTTTTCACGAA
WB235	WB235_Fam_258_35_1	1	35	0.000000	GCATTTGCCGATTTTTAAAATTTCTGGCAATTCCA
WB235	WB235_Fam_259_34_1	1	34	0.000000	CCGTGCCTACTAACCTACCGACCTTGCCTGCCTT
WB235	WB235_Fam_260_34_1	1	34	0.000000	TTGCCGAATTTGCCGTTTGCCGAGCTCGTCAAAT
WB235	WB235_Fam_261_34_1	1	34	0.000000	AAGTATTTTTGCCGGTTTTTTTGCCGAAATTTCC
WB235	WB235_Fam_262_33_1	1	33	0.000000	TAAGAAAAGTTAGGCCACCAAACTTTGGTGAAC
WB235	WB235_Fam_263_32_1	1	32	0.000000	AAAATCGAAAAATTCTCGTTTTTCAATTTTTA
WB235	WB235_Fam_264_32_1	1	32	0.000000	GCAATTTTTAGGCTAAAAATCACTAAAAAACT
WB235	WB235_Fam_265_32_1	1	32	0.000000	AAATTTGTGAAAATTTAGGCCCATTTTGCTCA
WB235	WB235_Fam_266_32_1	1	32	0.000000	GTGCGTCCGGCGTCCGGAAAAAGCGGACGCGT
WB235	WB235_Fam_267_31_1	1	31	0.000000	ATCGATTTTTTCGTTAAAAAATCAATAAAAT
WB235	WB235_Fam_268_31_1	1	31	0.000000	GCTGATTGGTCGGCGNGGTTCGCCCATCNTT

WB235	WB235_Fam_269_31_1	1	31	0.000000	GACATCTCGCAGGACATTATAAGAAGCGACC
WB235	WB235_Fam_270_31_1	1	31	0.000000	CACGCCTATTTTATGGGCGGAGTCTACAAGC
WB235	WB235_Fam_271_31_1	1	31	0.000000	TGCGTCGGAAATTNATTCCGATCATCGCCGG
WB235	WB235_Fam_272_31_1	1	31	0.000000	TCCGATGCATTGCCGATAATCGGAACCTGGT
WB235	WB235_Fam_273_31_1	1	31	0.000000	GCAGCCGACGCTATACGGGTCTGATGGAGTA
WB235	WB235_Fam_274_31_1	1	31	0.000000	TCTGAGCTACAGTACTCCTGTCAAGAACAAT
WB235	WB235_Fam_275_31_1	1	31	0.000000	GATTTTTTCAGNGAAAANCNAAAAATTTTCG
WB235	WB235_Fam_276_31_1	1	31	0.000000	CCAAAAATGACTGAAAATAGTCAAAAATTAG
WB235	WB235_Fam_277_31_1	1	31	0.000000	CAAAAATCGGGAAATTTTCACAAAAAAATGC
WB235	WB235_Fam_278_31_1	1	31	0.000000	TTGGCCCAGAGCTCTCTTGACCCAAGCCCCC
WB235	WB235_Fam_279_31_1	1	31	0.000000	AAAATTTTGACCCAGAAATCGAAAAATTCGC
WB235	WB235_Fam_280_31_1	1	31	0.000000	TCGTTTTTTCATGAAAAAACTCAATTTTCGG
WB235	WB235_Fam_281_31_1	1	31	0.000000	GAAAAAGTGCTGAAAATCACATTTTTGAACT
WB235	WB235_Fam_282_30_1	1	30	0.000000	AATTTGAATTTTCCCGAATTTTTNAACGAA
WB235	WB235_Fam_283_30_1	1	30	0.000000	TGGTGGAACTGGATTCGGCAGTGGAAATGC
WB235	WB235_Fam_284_30_1	1	30	0.000000	CCTTGGGCTCCCTGAGCTCCAAATTGTGCT
WB235	WB235_Fam_285_30_1	1	30	0.000000	GTCATTATCAGCTTCTGGTGTCTTAGAAGA
WB235	WB235_Fam_286_30_1	1	30	0.000000	CAAACTACAGTAGTACTGTAGTACCATAAC
WB235	WB235_Fam_287_30_1	1	30	0.000000	TTCAGGCAGGCATAGGTTACTTTGAAAATT
WB235	WB235_Fam_288_30_1	1	30	0.000000	CCTTGTAGGCAAGGAGGTAGGCGTAGGTCA
WB235	WB235_Fam_289_30_1	1	30	0.000000	TTTCAGGCCAATTTCNGTCATTTTTGGACA
WB235	WB235_Fam_290_30_1	1	30	0.000000	AAAATCAAAAATTAGATGAATTTTCGATTA
WB235	WB235_Fam_291_30_1	1	30	0.000000	CTCAAATTTGCACTAAAAATTCCAATTTTT
WB235	WB235_Fam_292_30_1	1	30	0.000000	ACTACCCAACCGGCTCCGGCAACCAGTCCA
WB235	WB235_Fam_293_29_1	1	29	0.000000	GGCCTAGAAANTTCTATTTGAAAGTTGAT
WB235	WB235_Fam_294_29_1	1	29	0.000000	TACATTGCAAGATCTTCTTACTATGTAGG
WB235	WB235_Fam_295_29_1	1	29	0.000000	CTCGTCAGTGGGTGGACGAGGAATGAGCG
WB235	WB235_Fam_296_28_1	1	28	0.000000	AATGTCGGCTGCTTCTAGAAACCAGGCA
WB235	WB235_Fam_297_28_1	1	28	0.000000	TGCCTCGCCTACGTGCTTATTTCAGGCA
WB235	WB235_Fam_298_28_1	1	28	0.000000	CCAAGAGAATTTCAACCACCTGGAAATT
WB235	WB235_Fam_299_27_1	1	27	0.000000	TTCCGGCAAATCGGCAAATTGCAAAAT
WB235	WB235_Fam_300_27_1	1	27	0.000000	ACAATAAAGTACTATGTATTACCGTAT
WB235	WB235_Fam_301_27_1	1	27	0.000000	CACTGACCAAAGAAAATATTANACGTG
WB235	WB235_Fam_302_26_1	1	26	0.000000	GGCAGGCGTCAAGCAGGCAGGTTTCA
WB235	WB235_Fam_303_26_1	1	26	0.000000	ACCTACAGTACCCCTGTCCTAGAATG
WB235	WB235_Fam_304_26_1	1	26	0.000000	TACTGTAGTTTGACCTACTTANGGAT
WB235	WB235_Fam_305_26_1	1	26	0.000000	ATTTACGGAGCTTCAAGCTTGTGTCG
WB235	WB235_Fam_306_26_1	1	26	0.000000	TGAAATTTCACAAAATATCGATTTTC
WB235	WB235_Fam_307_26_1	1	26	0.000000	AAATTAATCTGTCACTCTAACTTTTT
WB235	WB235_Fam_308_25_1	1	25	0.000000	GTCGGCTGCTTNCAAACCCTGAGTT
WB235	WB235_Fam_309_25_1	1	25	0.000000	AAAAATCTGTTCTNNGAGGTCCCTG
WB235	WB235_Fam_310_24_1	1	24	0.000000	GTGAGGTACGAGGTGAGGTANGAG
WB235	WB235_Fam_311_24_1	1	24	0.000000	AAGCACCACCTCCTCCAACTACAA
WB235	WB235_Fam_312_24_1	1	24	0.000000	AAGAAGGATGCTGAAGAGAAGAAG
WB235	WB235_Fam_313_24_1	1	24	0.000000	GCTTCTGGAGCTACAGGTTATCCA
WB235	WB235_Fam_314_24_1	1	24	0.000000	TCAACAACAACAGGAGAAGCTTCT
WB235	WB235_Fam_315_24_1	1	24	0.000000	TTTCAGTTTATCATCATTCTCCTG
WB235	WB235_Fam_316_24_1	1	24	0.000000	GGTGGCTGGTCACAAAAGTAAGTT
WB235	WB235_Fam_317_23_1	1	23	0.000000	GCATCATAAGGATCATAGTTGAT
WB235	WB235_Fam_318_23_1	1	23	0.000000	CGCTGTAAGACCCTTCGCTGTAC

WB235	WB235_Fam_319_23_1	1	23	0.000000	GCTACAGTACCCCAGCTTGACTT
WB235	WB235_Fam_320_23_1	1	23	0.000000	TTTTCAGAAATAGGTATTTCAGA
WB235	WB235_Fam_321_22_1	1	22	0.000000	TTAAAATTTTGTCAGTTTGACC
WB235	WB235_Fam_322_22_1	1	22	0.000000	GCACGGCCTGGCGGTCCTGAGG
WB235	WB235_Fam_323_22_1	1	22	0.000000	CTACAGTAACCCTAGTATTGAA
WB235	WB235_Fam_324_22_1	1	22	0.000000	AAGCGGTAACGTCGTCTATATA
WB235	WB235_Fam_325_22_1	1	22	0.000000	TGCTAATTAGCGCGGNTGCGTN
WB235	WB235_Fam_326_22_1	1	22	0.000000	ACGTATACAGGCATATACACAT
WB235	WB235_Fam_327_22_1	1	22	0.000000	CCGGCGTGGTNTTGAAGCGGTC
WB235	WB235_Fam_328_22_1	1	22	0.000000	TTTGTGGGAGGCGACATATACC
WB235	WB235_Fam_329_21_1	1	21	0.000000	TGGCCTAGATTNTTTACAGGG
WB235	WB235_Fam_330_21_1	1	21	0.000000	TCTCCTGGTAAATTTAAAAAT
WB235	WB235_Fam_331_21_1	1	21	0.000000	ACCACCCCTACAACNACCACC
WB235	WB235_Fam_332_21_1	1	21	0.000000	AACTTTTTGGCAATTTTCCAA
WB235	WB235_Fam_333_21_1	1	21	0.000000	TGTGCCCTGGAAAGCTGGCAG
WB235	WB235_Fam_334_21_1	1	21	0.000000	CTACGTAAGAGTAGCGCAGGA
WB235	WB235_Fam_335_21_1	1	21	0.000000	AATCTAGCCAGACGCACACCT
WB235	WB235_Fam_336_21_1	1	21	0.000000	CAATTTTCTGTTNAACTTTGA
WB235	WB235_Fam_337_21_1	1	21	0.000000	AAGTTTTACTTTGATTTTTTT
WB235	WB235_Fam_338_21_1	1	21	0.000000	GGCAATTTTCCAGTGCAATTC
WB235	WB235_Fam_339_21_1	1	21	0.000000	CCATAGGTGGAAGATCTNATA
WB235	WB235_Fam_340_21_1	1	21	0.000000	GATTTACGGGGTGAACATGTG
WB235	WB235_Fam_341_21_1	1	21	0.000000	TTGGTGACAATGTGGAAACAA
WB235	WB235_Fam_342_21_1	1	21	0.000000	TTCTAATTCGACGCGCATNGT
WB235	WB235_Fam_343_21_1	1	21	0.000000	CTAATTAGCGTCGCTTCCNTT
WB235	WB235_Fam_344_21_1	1	21	0.000000	AAAAATCGATAAAATTTGGAN
WB235	WB235_Fam_345_21_1	1	21	0.000000	CACACGAGCTTGAGAGCCCGG
WB235	WB235_Fam_346_21_1	1	21	0.000000	TATTTTCAAAAAAATTTAAAA
WB235	WB235_Fam_347_21_1	1	21	0.000000	ACCCCGATTTTCACCCAAAAA
WB235	WB235_Fam_348_21_1	1	21	0.000000	CTCAATTCAATCAAGGAAATC
WB235	WB235_Fam_349_21_1	1	21	0.000000	CCATGTAGCTTCAAAATTNCT
WB235	WB235_Fam_350_21_1	1	21	0.000000	TGTCCGTGATGATCATGNGCA
WB235	WB235_Fam_351_21_1	1	21	0.000000	GGTGGACCCANGGGTTCCTAG
WB235	WB235_Fam_352_20_1	1	20	0.000000	CTATCGCCTGCTATCGCCTA
WB235	WB235_Fam_353_20_1	1	20	0.000000	ATTTGACCTACTTTTTAAAA
WB235	WB235_Fam_354_19_1	1	19	0.000000	GTACTACCTTTTAATGTAT
WB235	WB235_Fam_355_19_1	1	19	0.000000	CCCTGATACCCCCTAGTCC
WB235	WB235_Fam_356_19_1	1	19	0.000000	TATGGTGCATCTAACTAGT
WB235	WB235_Fam_357_19_1	1	19	0.000000	GCCTCACTCAAAATCCTCA
WB235	WB235_Fam_358_18_1	1	18	0.000000	AAAAGTTACAGTGTCCTT
WB235	WB235_Fam_359_18_1	1	18	0.000000	TTGTAGATCAACGGGGTC
WB235	WB235_Fam_360_18_1	1	18	0.000000	GGAGGTCCAGGTACCCCG
WB235	WB235_Fam_361_18_1	1	18	0.000000	TAAATAAGTGGCTATAAT
WB235	WB235_Fam_362_18_1	1	18	0.000000	ACTACTGTATCNCAGGAG
WB235	WB235_Fam_363_18_1	1	18	0.000000	CCTTCGCCTACCTACGTG
WB235	WB235_Fam_364_18_1	1	18	0.000000	TTTTTTGATAATTCTTGA
WB235	WB235_Fam_365_18_1	1	18	0.000000	AAATATTCTTATTTTCAG
WB235	WB235_Fam_366_18_1	1	18	0.000000	CCTGGTTGTCCNTCTNNT
WB235	WB235_Fam_367_18_1	1	18	0.000000	TTTCATAAGAAGTAAATN
WB235	WB235_Fam_368_18_1	1	18	0.000000	CATTGTCACTTTCTTCTC

WB235	WB235_Fam_369_18_1	1	18	0.000000	AAGCCTATGCCTTTCCCA
WB235	WB235_Fam_370_17_1	1	17	0.000000	ACTTGGTACATAGGCCA
WB235	WB235_Fam_371_17_1	1	17	0.000000	ATTTTTTCCGCAAGTAG
WB235	WB235_Fam_372_17_1	1	17	0.000000	AAAATCAAACCGGCGGA
WB235	WB235_Fam_373_17_1	1	17	0.000000	AAATGCCAAAANTGCCA
WB235	WB235_Fam_374_17_1	1	17	0.000000	TAACCTGACTGCTACAG
WB235	WB235_Fam_375_17_1	1	17	0.000000	GAATTAGAAAAATTTGA
WB235	WB235_Fam_376_17_1	1	17	0.000000	TTGCAAGAAAATCTGCA
WB235	WB235_Fam_377_17_1	1	17	0.000000	TTTTAACGTGACCTAGT
WB235	WB235_Fam_378_16_1	1	16	0.000000	TGATCTGATGATCTGA
WB235	WB235_Fam_379_16_1	1	16	0.000000	AATACGGGAATACGGG
WB235	WB235_Fam_380_16_1	1	16	0.000000	TTATAGTGTTATAGTG
WB235	WB235_Fam_381_16_1	1	16	0.000000	TGTATATATATATATA
WB235	WB235_Fam_382_16_1	1	16	0.000000	AAAAATTATTGAATAC
WB235	WB235_Fam_383_16_1	1	16	0.000000	GAATAATATTGGAGAA
WB235	WB235_Fam_384_16_1	1	16	0.000000	CTTAGATCTCAATTCC
WB235	WB235_Fam_385_16_1	1	16	0.000000	AGAGAGTATAGATTAG
WB235	WB235_Fam_386_15_1	1	15	0.000000	CGACCTGCGACCTAG
WB235	WB235_Fam_387_15_1	1	15	0.000000	CACAGGAGGCTGGGA
WB235	WB235_Fam_388_14_1	1	14	0.000000	AGCTTGAAGCTTGG
WB235	WB235_Fam_389_14_1	1	14	0.000000	GAAGGATCGGGCCG
WB235	WB235_Fam_390_14_1	1	14	0.000000	TTACCAAAATCTAA
WB235	WB235_Fam_391_13_1	1	13	0.000000	TTGGGGTGGGCCT
WB235	WB235_Fam_392_12_1	1	12	0.000000	AATACCAACCAC
WB235	WB235_Fam_393_12_1	1	12	0.000000	AGTTATATAGTA
WB235	WB235_Fam_394_12_1	1	12	0.000000	CATTGACTTGAG
WB235	WB235_Fam_395_11_1	1	11	0.000000	TTGTTTACAAG
WB235	WB235_Fam_396_11_1	1	11	0.000000	TACCTACTAGA
WB235	WB235_Fam_397_11_1	1	11	0.000000	AGATACACTTC
WB235	WB235_Fam_398_11_1	1	11	0.000000	TTTCAGGCCTA
WB235	WB235_Fam_399_10_1	1	10	0.000000	GATTTTCATA
WB235	WB235_Fam_400_10_1	1	10	0.000000	CAGCAGAGTG
WB235	WB235_Fam_401_10_1	1	10	0.000000	AACTTTCGAA
WB235	WB235_Fam_402_10_1	1	10	0.000000	TGGCGAAAGC
